# Comparative efficacy and tolerability of medications for attention-deficit hyperactivity disorder in children, adolescents, and adults: a systematic review and network meta-analysis

**DOI:** 10.1016/S2215-0366(18)30269-4

**Published:** 2018-09

**Authors:** Samuele Cortese, Nicoletta Adamo, Cinzia Del Giovane, Christina Mohr-Jensen, Adrian J Hayes, Sara Carucci, Lauren Z Atkinson, Luca Tessari, Tobias Banaschewski, David Coghill, Chris Hollis, Emily Simonoff, Alessandro Zuddas, Corrado Barbui, Marianna Purgato, Hans-Christoph Steinhausen, Farhad Shokraneh, Jun Xia, Andrea Cipriani

**Affiliations:** a Center for Innovation in Mental Health, Academic Unit of Psychology, and Clinical and Experimental Sciences (CNS and Psychiatry), Faculty of Medicine, University of Southampton, Southampton, UK; b Solent NHS Trust, Southampton, UK; c New York University Child Study Center, New York, NY, USA; d Division of Psychiatry and Applied Psychology, School of Medicine, University of Nottingham, Nottingham, UK; e Department of Child and Adolescent Psychiatry, King's College London, and Institute of Psychiatry, Psychology and Neuroscience, and National Institute for Health Research (NIHR) Maudsley Biomedical Research Centre, London, UK; f Institute of Primary Health Care, University of Bern, Switzerland; g Department of Child and Adolescent Psychiatry, Aalborg Psychiatric Hospital, Aalborg University Hospital, Aalborg, Denmark; h Department of Psychiatry, University of Oxford, and Oxford Health NHS Foundation Trust, Warneford Hospital, Oxford, UK; i Child and Adolescent Neuropsychiatry Unit, Department of Biomedical Sciences, University of Cagliari and “A Cao” Paediatric Hospital, “G Brotzu” Hospital Trust, Cagliari, Italy; j Department of Child and Adolescent Psychiatry and Psychotherapy, Bolzano, Italy; k Department of Child and Adolescent Psychiatry and Psychotherapy, Central Institute of Mental Health, Medical Faculty Mannheim and University of Heidelberg, Mannheim, Germany; l Departments of Paediatrics and Psychiatry, Faculty of Medicine, Dentistry and Health Sciences, University of Melbourne, Melbourne, Vic, Australia; m Division of Neuroscience, Ninewells Hospital and Medical School, University of Dundee, Dundee, UK; n Murdoch Childrens' Research Institute, Melbourne, Vic, Australia; o NIHR Nottingham Biomedical Research Centre, NIHR MindTech MedTech and In-vitro Diagnostic Cooperative, and Centre for ADHD and Neurodevelopmental Disorders Across the Lifespan (CANDAL), Institute of Mental Health, University of Nottingham, Nottingham, UK; p WHO Collaborating Centre for Research and Training in Mental Health and Service Evaluation, Department of Neuroscience, Biomedicine, and Movement Sciences, Section of Psychiatry, University of Verona, Verona, Italy; q Department of Child and Adolescent Psychiatry, Psychiatric University Clinic Zurich, Zurich, Switzerland; r Clinical Psychology and Epidemiology, Department of Psychology, University of Basel, Basel, Switzerland; s Child and Adolescent Mental Health Centre, Capital Region Psychiatry, Copenhagen, Denmark; t Department of Child and Adolescent Psychiatry, University of Southern Denmark, Odense, Denmark; u Cochrane Schizophrenia Group, Division of Psychiatry and Clinical Psychology, School of Medicine, University of Nottingham, Nottingham, UK; v Research Center for Modeling in Health, Institute for Future Studies in Health, Kerman University of Medical Sciences, Kerman, Iran; w Systematic Review Solutions, and Nottingham Health China, University of Nottingham, Ningbo, China

## Abstract

**Background:**

The benefits and safety of medications for attention-deficit hyperactivity disorder (ADHD) remain controversial, and guidelines are inconsistent on which medications are preferred across different age groups. We aimed to estimate the comparative efficacy and tolerability of oral medications for ADHD in children, adolescents, and adults.

**Methods:**

We did a literature search for published and unpublished double-blind randomised controlled trials comparing amphetamines (including lisdexamfetamine), atomoxetine, bupropion, clonidine, guanfacine, methylphenidate, and modafinil with each other or placebo. We systematically contacted study authors and drug manufacturers for additional information. Primary outcomes were efficacy (change in severity of ADHD core symptoms based on teachers' and clinicians' ratings) and tolerability (proportion of patients who dropped out of studies because of side-effects) at timepoints closest to 12 weeks, 26 weeks, and 52 weeks. We estimated summary odds ratios (ORs) and standardised mean differences (SMDs) using pairwise and network meta-analysis with random effects. We assessed the risk of bias of individual studies with the Cochrane risk of bias tool and confidence of estimates with the Grading of Recommendations Assessment, Development, and Evaluation approach for network meta-analyses. This study is registered with PROSPERO, number CRD42014008976.

**Findings:**

133 double-blind randomised controlled trials (81 in children and adolescents, 51 in adults, and one in both) were included. The analysis of efficacy closest to 12 weeks was based on 10 068 children and adolescents and 8131 adults; the analysis of tolerability was based on 11 018 children and adolescents and 5362 adults. The confidence of estimates varied from high or moderate (for some comparisons) to low or very low (for most indirect comparisons). For ADHD core symptoms rated by clinicians in children and adolescents closest to 12 weeks, all included drugs were superior to placebo (eg, SMD −1·02, 95% CI −1·19 to −0·85 for amphetamines, −0·78, −0·93 to −0·62 for methylphenidate, −0·56, −0·66 to −0·45 for atomoxetine). By contrast, for available comparisons based on teachers' ratings, only methylphenidate (SMD −0·82, 95% CI −1·16 to −0·48) and modafinil (−0·76, −1·15 to −0·37) were more efficacious than placebo. In adults (clinicians' ratings), amphetamines (SMD −0·79, 95% CI −0·99 to −0·58), methylphenidate (−0·49, −0·64 to −0·35), bupropion (−0·46, −0·85 to −0·07), and atomoxetine (−0·45, −0·58 to −0·32), but not modafinil (0·16, −0·28 to 0·59), were better than placebo. With respect to tolerability, amphetamines were inferior to placebo in both children and adolescents (odds ratio [OR] 2·30, 95% CI 1·36–3·89) and adults (3·26, 1·54–6·92); guanfacine was inferior to placebo in children and adolescents only (2·64, 1·20–5·81); and atomoxetine (2·33, 1·28–4·25), methylphenidate (2·39, 1·40–4·08), and modafinil (4·01, 1·42–11·33) were less well tolerated than placebo in adults only. In head-to-head comparisons, only differences in efficacy (clinicians' ratings) were found, favouring amphetamines over modafinil, atomoxetine, and methylphenidate in both children and adolescents (SMDs −0·46 to −0·24) and adults (−0·94 to −0·29). We did not find sufficient data for the 26-week and 52-week timepoints.

**Interpretation:**

Our findings represent the most comprehensive available evidence base to inform patients, families, clinicians, guideline developers, and policymakers on the choice of ADHD medications across age groups. Taking into account both efficacy and safety, evidence from this meta-analysis supports methylphenidate in children and adolescents, and amphetamines in adults, as preferred first-choice medications for the short-term treatment of ADHD. New research should be funded urgently to assess long-term effects of these drugs.

**Funding:**

Stichting Eunethydis (European Network for Hyperkinetic Disorders), and the UK National Institute for Health Research Oxford Health Biomedical Research Centre.

**Research in context**

**Evidence before this study**

We searched PubMed, BIOSIS Previews, CINAHL, the Cochrane Central Register of Controlled Trials, Embase, ERIC (Education Resources Information Center), MEDLINE, PsycINFO, OpenGrey, Web of Science Core Collection, ProQuest Dissertations and Theses (UK and Ireland), ProQuest Dissertations and Theses (Abstracts and International), and the WHO International Trials Registry Platform (including ClinicalTrials.gov) from database inception up to April 7, 2017, with no restrictions by language, for published and unpublished double-blind randomised controlled trials comparing amphetamines (including lisdexamfetamine), atomoxetine, bupropion, clonidine, guanfacine, methylphenidate, and modafinil with each other or placebo. We used the search terms: “adhd” OR “hkd” OR “addh” OR “hyperkine*” OR “attention deficit*” OR “hyper-activ*” OR “hyperactiv*” OR “overactive” OR “inattentive” OR “impulsiv*”, combined with a list of ADHD medications (appendix pp 3–15). We also hand-searched the websites of the US Food and Drug Administration, the European Medicines Agency, and relevant drug manufacturers, and references of previous systematic reviews and guidelines, for additional information. Further, we contacted study authors and drug manufacturers to gather unpublished information or data. Over the past few decades, a substantial increase has been noted across many countries in prescription of medications for attention-deficit hyperactivity disorder (ADHD). However, the benefits and safety of these medications remain a matter for debate. Published meta-analyses of head-to-head trials and network meta-analyses provide inconsistent findings on the comparative benefits and harms of ADHD medications.

**Added value of this study**

Our study, based on advanced methodology for network meta-analyses, represents the most comprehensive synthesis to date on the comparative efficacy and tolerability of medications for ADHD across age groups. Unlike previous network meta-analyses of ADHD treatments, we have included unpublished data, which were gathered systematically from study authors, the websites of regulatory agencies, and drug manufacturers, using a common set of inclusion criteria for trials in children, adolescents, and adults. We focused on a series of clinically relevant outcomes—namely, efficacy on ADHD core symptoms, global clinical functioning, tolerability, effects on weight and blood pressure, and acceptability. We also investigated important effect-modifiers (eg, dose and comorbidities). We retained only a few studies with outcomes beyond 12 weeks. All medications we included in our study (except modafinil in adults) were more efficacious than placebo for the acute treatment of ADHD. Medications for ADHD were less efficacious and less well tolerated in adults than in children and adolescents. However, included drugs were not equivalent, and their profile in terms of efficacy, tolerability, and acceptability varied across age groups.

**Implications of all the available evidence**

Evidence from our network meta-analysis supports methylphenidate (in children and adolescents) and amphetamines (in adults) as the preferred first pharmacological choice for short-term pharmacological treatment of ADHD. This network meta-analysis should inform future guidelines and daily clinical decision-making on the choice of medications for ADHD across age ranges, along with available evidence on cost-effectiveness and considering patients' preferences. The paucity of trials with randomised outcomes beyond 12 weeks highlights the need to fund studies to assess long-term effects of these drugs. Furthermore, future research should include individual patient data in network meta-analyses of ADHD medications, which will allow a more reliable estimation of predictors of individual response.

## Introduction

Attention-deficit hyperactivity disorder (ADHD) is characterised by age-inappropriate and impairing levels of inattention, hyperactivity, or impulsivity, or a combination.^1^ It is estimated to affect around 5% of school-age children (aged ≤18 years)^2^ and 2·5% of adults worldwide.^3^ Annual incremental costs for ADHD have been estimated at US$143–266 billion in the USA^4^ and are substantial in other countries.^5^, ^6^ Available pharmacological treatments for ADHD include psychostimulants (eg, methylphenidate and amphetamines) and non-psychostimulant medications (eg, atomoxetine and α2-agonists). In the past few decades, prescriptions for ADHD drugs have increased significantly both in the USA^7^ and other countries.^8^ However, even though recommended in clinical guidelines,^9^, ^10^, ^11^, ^12^, ^13^, ^14^ the efficacy and safety of ADHD medications remains controversial.^15^, ^16^, ^17^ Furthermore, current guidelines are inconsistent in their treatment recommendations.^9^, ^10^, ^11^, ^12^, ^13^, ^14^ Although some guidelines rank methylphenidate over amphetamines (eg, in children),^9^ others recommend psychostimulants as first-line treatment without any distinction between methylphenidate and amphetamines being made.^10^, ^11^ Additionally, the non-psychostimulant atomoxetine is variously recommended by available guidelines as third-line,^9^ second-line,^10^, ^11^ and potentially first-line treatment.^12^ The methods used for sequencing these recommendations are not always specified and most commonly—including the 2018 UK National Institute for Health and Care Excellence (NICE) guidelines^9^—incorporate national drug licencing regulatory approval and cost-effectiveness with expert opinion in conjunction with the few head-to-head comparisons that are available.

Network meta-analyses facilitate estimation of the comparative efficacy and tolerability of two or more interventions, even when they have not been investigated head-to-head in randomised controlled trials.^18^ Thus, compared with standard pairwise meta-analyses, network meta-analyses have been found to increase the precision of the estimates.^18^ Previous network meta-analyses in ADHD have focused on either children and adolescents^19^, ^20^, ^21^, ^22^, ^23^, ^24^ or adults only,^25^, ^26^, ^27^, ^28^ have typically compared only a few drugs,^24^, ^25^, ^27^, ^29^ or have addressed exclusively the safety of treatments.^26^

To fill this gap, we did a systematic review and network meta-analysis of double-blind randomised controlled trials in children, adolescents, and adults with ADHD, using data from published reports and unpublished data gathered systematically from drug manufacturers or study authors. We aimed specifically to compare ADHD medications in terms of efficacy on core ADHD symptoms, clinical global functioning, tolerability, acceptability, and other clinically important outcomes—eg, blood pressure and weight changes.

## Methods

### Search strategy and selection criteria

We searched PubMed, BIOSIS Previews, CINAHL, the Cochrane Central Register of Controlled Trials, EMBASE, ERIC, MEDLINE, PsycINFO, OpenGrey, Web of Science Core Collection, ProQuest Dissertations and Theses (UK and Ireland), ProQuest Dissertations and Theses (abstracts and international), and the WHO International Trials Registry Platform, including ClinicalTrials.gov, from the date of database inception to April 7, 2017, with no language restrictions. We used the search terms “adhd” OR “hkd” OR “addh” OR “hyperkine*” OR “attention deficit*” OR “hyper-activ*” OR “hyperactiv*” OR “overactive” OR “inattentive” OR “impulsiv*” combined with a list of ADHD medications (appendix pp 3–15). The US Food and Drug Administration (FDA), European Medicines Agency (EMA), and relevant drug manufacturers' websites, and references of previous systematic reviews and guidelines, were hand-searched for additional information. We also contacted study authors and drug manufacturers to gather unpublished information and data (appendix p 15).

We included double-blind randomised controlled trials (parallel group, crossover, or cluster), of at least 1 week's duration, that enrolled children (aged ≥5 years and <12 years), adolescents (aged ≥12 years and <18 years), or adults (≥18 years) with a primary diagnosis of ADHD according to DSM-III, DSM III-R, DSM-IV(TR), DSM-5, ICD-9, or ICD-10. We did not restrict our search by ADHD subtype or presentation, gender, intelligence quotient (IQ), socioeconomic status, or comorbidities (except for those needing concomitant pharmacotherapy). We included studies if they assessed any of the following medications, as oral monotherapy, compared with each other or with placebo: amphetamines (including lisdexamfetamine), atomoxetine, bupropion, clonidine, guanfacine, methyl-phenidate (including dexmethylphenidate), and modafinil. We excluded studies with enrichment designs (eg, trials selecting drug responders only after a run-in phase), because these types of trial can potentially inflate efficacy and tolerability estimates. Full inclusion and exclusion criteria are in the appendix (pp 16, 17).

Our study protocol was registered with PROSPERO (number CRD42014008976) and published.^30^ We followed the PRISMA extension for network meta-analyses.^31^

### Procedures

Data were extracted by at least two independent investigators. We assessed risk of bias with the Cochrane risk of bias tool.^32^ We estimated the certainty of evidence with the Grading of Recommendations Assessment, Development, and Evaluation (GRADE) approach for network meta-analyses (appendix pp 18, 19).^33^

### Outcomes

For our primary analyses we considered efficacy, which we measured as the change in severity of ADHD core symptoms based on clinicians' ratings for children, adolescents, and adults. The appendix (pp 273, 274) contains a list of rating scales considered for inclusion. For children and adolescents, we also considered teachers' ratings as a primary efficacy outcome because they provide a complementary view to clinicians' ratings, and information from multiple raters increases the validity of ADHD diagnosis.^34^ We also considered tolerability in children, adolescents, and adults—ie, the proportion of participants who left the study because of any side-effect.

Secondary outcomes included the change in severity of ADHD core symptoms based on parents' ratings for children and adolescents and self-reports for adults, clinical global functioning measured by the Clinical Global Impression–Improvement (CGI-I, clinicians' ratings), acceptability (ie, the proportion of participants who left the study for any reason), and change in weight and blood pressure. We assessed those outcomes available at the times closest to 12 weeks (primary endpoint), 26 weeks, and 52 weeks.

### Statistical analysis

We did all analyses separately for studies in children and adolescents and for studies in adults. First, we did pairwise meta-analyses (active drug *vs* placebo, or active drug *vs* another active drug) for all outcomes and comparisons at every available timepoint, using a random-effects model.^35^ We calculated standardised mean differences (SMDs), Hedges's adjusted g, and odds ratios (ORs), with relative 95% CIs, for continuous and dichotomous outcomes. We assessed statistical heterogeneity within each pairwise comparison by calculating the *I*^2^ statistic and its 95% CI.^36^ Second, we did network meta-analyses within a frequentist framework assuming equal heterogeneity parameter τ across all comparisons and accounting for correlations induced by multiarm studies.^37^, ^38^ We based the assessment of statistical heterogeneity in the entire network on the magnitude of the common τ^2^ estimated from the network meta-analysis models.^39^ We compared the magnitude of the heterogeneity variance with the empirical distribution.^40^, ^41^ We used the loop-specific approach^42^ and the design-by-treatment model^43^ to evaluate incoherence locally and globally, respectively. We established a hierarchy of competing interventions using surface under the cumulative ranking curve (SUCRA) and mean ranks.^44^

We planned a set of subgroup and sensitivity analyses to assess the effect of clinical and study design effect-modifiers—eg, duration of study, gender, age (children *vs* adolescents), psychiatric comorbidities, IQ, crossover design, medication status, industry sponsorship, inequalities in doses, risk of bias, and data imputation.^30^ We restricted the primary analysis to studies using medications within the therapeutic range (as per FDA recommendations, where applicable). Additionally, we investigated effects at different dose regimens in two sets of sensitivity analyses. First, we excluded studies that did not use the FDA-licensed dose (appendix pp 277–79). Second, we included studies in which the dose ranges used were recommended in national or international guidelines or formularies but differed from FDA recom-mendations. Finally, to investigate possible differences between lisdexamfetamine and other amphetamines, we did a post-hoc analysis separating this compound, because lisdexamfetamine is metabolised differently from other amphetamines, which could affect its efficacy and tolerability.^45^

We did all analyses with STATA version 14. Additional details are reported in the appendix (pp 20–24, 277–82). Changes to the original protocol are listed in the appendix (p 25).

### Role of the funding source

The funder had no role in study design, data collection, data analysis, data interpretation, or writing of the report. SCo, NA, CDG, and AC had full access to all data in the study, and AC was responsible for the final decision to submit for publication.

## Results

**Figure 1:**
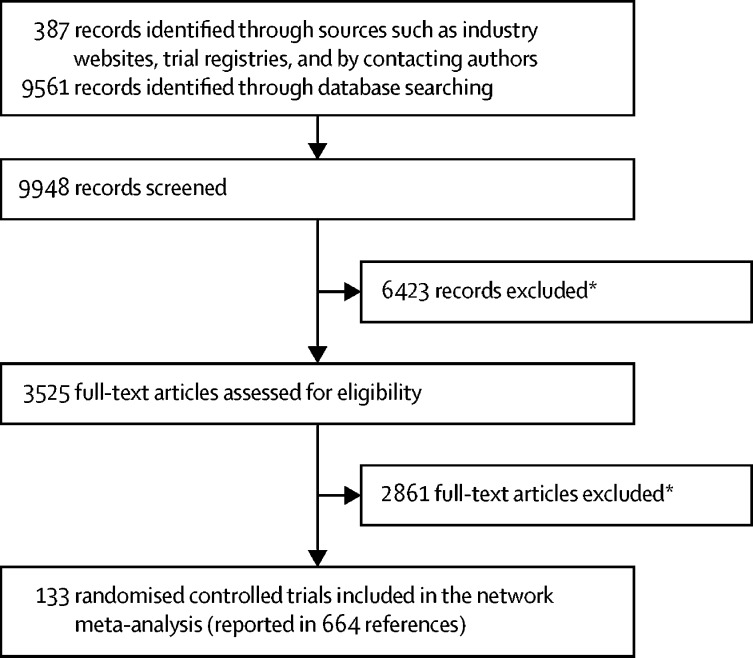
Selection of studies for inclusion *The main reasons for exclusion included open-label or single-blind studies, studies including patients with comorbid disorders, and combination therapy trials. We only searched for completed trials, which removed ongoing studies, particularly from clinicaltrials.gov.

The literature search, study selection, and data extraction were done between Jan 11, 2014, and Sept 9, 2017, and data analysis was done from Sept 10, 2017, to Feb 24, 2018. The study selection process is shown in figure 1; a list of excluded studies, with reasons for exclusion, and a list of retained studies is provided in the appendix (pp 26–272). 133 studies were retained for the network meta-analysis, 81 in children and adolescents, 51 in adults, and one including children, adolescents, and adults. In total, 14 346 children and adolescents and 10 296 adults were included. For 83% of studies, additional data and information not reported in the full-text paper were used. The appendix (pp 283–381) reports the main characteristics of included studies. The risk of bias was rated overall low in 23·5% of studies in children and adolescents, unclear in 65·4%, and high in 11·1%. The risk of bias was overall low in 27·5% of studies in adults, unclear in 56·8%, and high in 15·7% (appendix pp 382–458).

**Figure 2:**
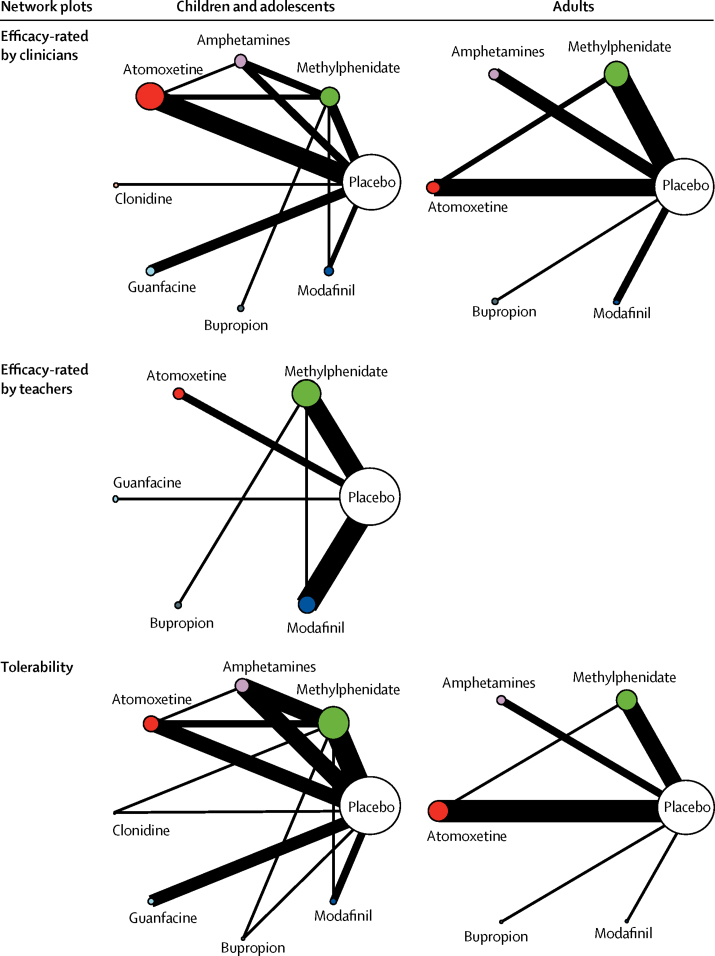
Network of eligible comparisons for efficacy and tolerability The width of the lines is proportional to the number of trials comparing every pair of treatments, and the size of every circle is proportional to the number of randomly assigned participants (sample size). The number of trials for pairs of treatments ranged from 22 (eg, studies of tolerability of methylphenidate *vs* placebo in children and adolescents) to one (several comparisons).

**Figure 3:**
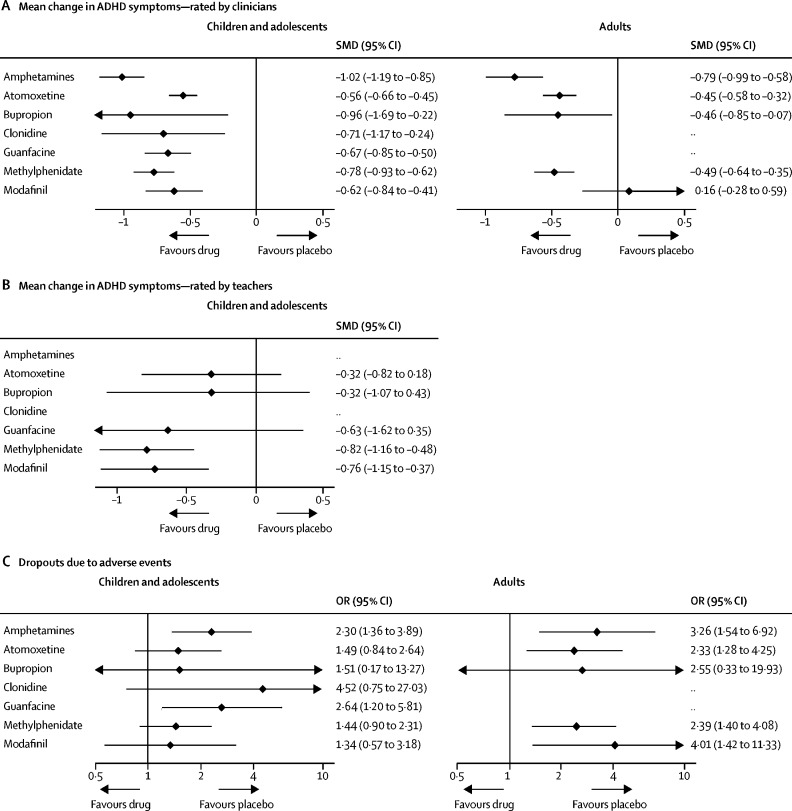
Forest plots of network meta-analysis results Plots include all trials for efficacy and tolerability and are compared with placebo as reference. No data for clonidine and guanfacine in adults are reported because no studies identified by our search tested these two drugs in adults. ADHD=attention-deficit hyperactivity disorder. OR=odds ratio. SMD=standardised mean difference.

**Figure 4:**
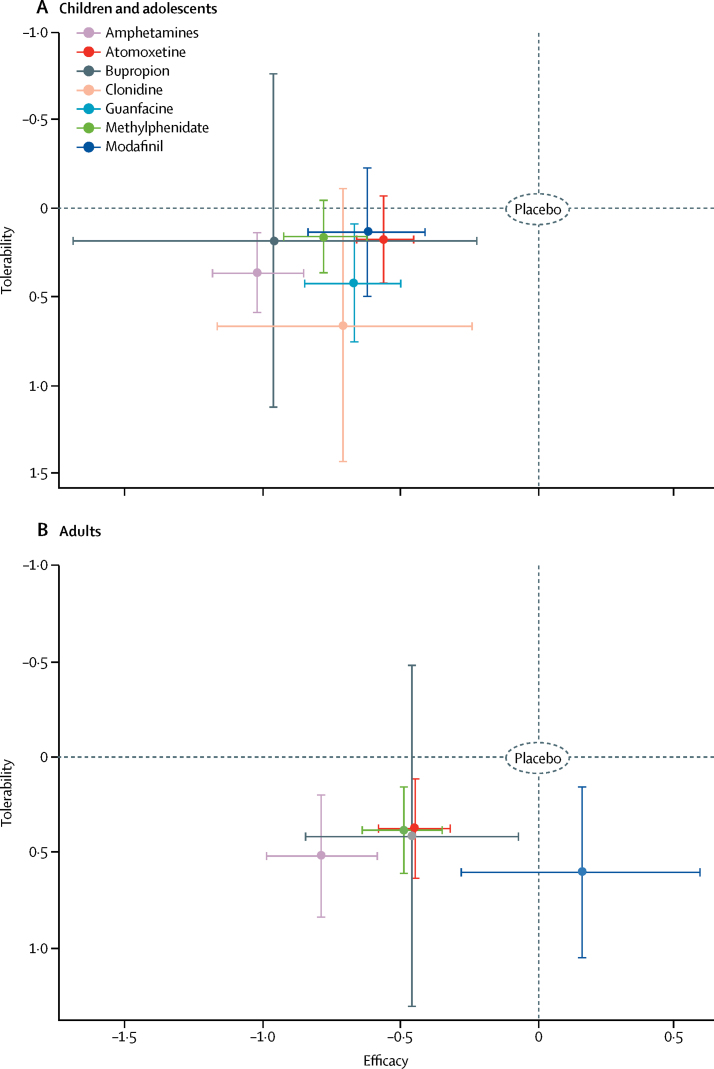
Two-dimensional graphs of efficacy versus tolerability in studies in children and adolescents and adults Effect sizes for individual drugs are represented by coloured nodes, with bars representing corresponding 95% CIs.

Figure 2 shows the network plots for the primary outcomes closest to 12 weeks. Network plots for secondary outcomes are reported in the appendix (pp 624–29). Results of the pairwise meta-analyses and related heterogeneity are reported in the appendix (pp 459–71). Results of the network meta-analyses of primary outcomes at 12 weeks are shown in figure 3, Table 1, Table 2, and the appendix (pp 472, 473). Table 1, Table 2 also show the confidence of estimates for every comparison. Figure 4 summarises data for efficacy (in 10 068 children and adolescents and 8131 adults) and tolerability (in 11 018 children and adolescents and 5362 adults).

**Table 1:** Effect of ADHD drugs in children and adults at timepoints closest to 12 weeks in terms of efficacy, as rated by clinicians and teachers

	**Atomoxetine**		**Bupropion**		**Clonidine**		**Guanfacine**		**Methylphenidate**		**Modafinil**		**Placebo**	
	Children	Adults	Children	Adults	Children	Adults	Children	Adults	Children	Adults	Children	Adults	Children	Adults
**Amphetamines**														
Clinicians	−0·46 (−0·65 to −0·27)*	−0·34 (−0·58 to −0·10)*	−0·06 (−0·81 to 0·68)†	−0·33 (−0·77 to 0·11)*	−0·31 (−0·81 to 0·18)*	..	−0·35 (−0·59 to −0·10)*	..	−0·24 (−0·44 to −0·05)*	−0·29 (−0·54 to −0·05)*	−0·39 (−0·67 to −0·12)*	**−0·94 (−1·43 to −0·46)**‡	**−1·02 (−1·19 to −0·85)**‡	**−0·79 (−0·99 to −0·58)**‡
Teachers	..	..	..	..	..	..	..	..	..	..	..	..	..	..
**Atomoxetine**														
Clinicians	..	..	0·40 (−0·34 to 1·14)*	0·01 (−0·41 to 0·42)*	0·15 (−0·33 to 0·63)*	..	0·11 (−0·09 to 0·32)*	..	0·22 (0·05 to 0·39)*	0·04 (−0·14 to 0·23)‡	0·07 (−0·17 to 0·31)*	−0·61 (−1·06 to −0·15)*	−0·56 (−0·66 to −0·45)*	−0·45 (−0·58 to −0·32)*
Teachers	..	..	0·00 (−0·90 to 0·90)†	..	..	..	0·31 (−0·79 to 1·42)†	..	0·50 (−0·11 to 1·10)*	..	0·44 (− 0·19 to 1·07)*	..	−0·32 (−0·82 to 0·18)†	..
**Bupropion**														
Clinicians	..	..	..	..	−0·25 (−1·12 to 0·62)†	..	−0·28 (−1·04 to 0·47)†	..	−0·18 (−0·90 to 0·54)†	0·04 (−0·38 to 0·45)*	−0·33 (−1·10 to 0·43)†	−0·62 (−1·20 to −0·03)*	**−0·96 (−1·69 to −0·22)**‡	−0·46 (−0·85 to −0·07)*
Teachers	..	..	..	..	..	..	0·31 (−0·92 to 1·55)†	..	0·50 (−0·17 to 1·17)*	..	0·44 (−0·38 to 1·26)*	..	−0·32 (−1·07 to 0·43**)**†	..
**Clonidine**														
Clinicians	..	..	..	..	..	..	−0·03 (−0·53 to 0·46)†	..	0·07 (−0·42 to 0·56)†	..	−0·08 (−0·59 to 0·43)†	..	**−0·71 (−1·17 to −0·24)**‡	..
**Guanfacine**														
Clinicians	..	..	..	..	..	..	..	..	0·11 (−0·13 to 0·34)*	..	−0·05 (−0·32 to 0·23)*	..	**−0·67 (−0·85 to −0·50)**‡	..
Teachers	..	..	..	..	..	..	..	..	0·18 (−0·86 to 1·22)†	..	0·12 (−0·93 to 1·18)†	..	**−0·63 (−1·62 to 0·35)**†	..
**Methylphenidate**														
Clinicians	..	..	..	..	..	..	..	..	..	..	−0·15 (−0·41 to 0·10)*	−0·65 (−1·11 to −0·19)*	**−0·78 (−0·93 to −0·62)**‡	**−0·49 (−0·64 to −0·35)**‡
Teachers	..	..	..	..	..	..	..	..	..	..	−0·06 (−0·53 to 0·42)†	..	−0·82 (−1·16 to −0·48)*	..
**Modafinil**														
Clinicians	..	..	..	..	..	..	..	..	..	..	..	..	−0·62 (−0·84 to −0·41)*	0·16 (−0·28 to 0·59)*
Teachers	..	..	..	..	..	..	..	..	..	..	..	..	**−0·76 (−1·15 to −0·37)**†	..

Data are standardised mean difference (95% CI) between treatments. Results in bold are significant. Negative values favour the treatment in the row and positive values favour the treatment in the column. Drugs are reported in alphabetical order. Results are based on network estimates. No data for clonidine and guanfacine in adults are reported because no studies identified by our search tested these two drugs in adults. No teacher ratings were available for clonidine. ADHD=attention-deficit hyperactivity disorder.

* Low quality of evidence.

† Very low quality of evidence.

‡ Moderate quality of evidence.

**Table 2:** Effect of ADHD drugs in children and adults at timepoints closest to 12 weeks in terms of tolerability

	**Atomoxetine**		**Bupropion**		**Clonidine**		**Guanfacine**		**Methylphenidate**		**Modafinil**		**Placebo**	
	Children	Adults	Children	Adults	Children	Adults	Children	Adults	Children	Adults	Children	Adults	Children	Adults
Amphetamines	1·54 (0·79–3·01)*	1·40 (0·54–3·66)†	1·53 (0·17–13·88)†	1·28 (0·14–11·40)†	0·51 (0·08–3·27)†	..	0·87 (0·35–2·16)†	..	1·60 (0·94–2·73)*	1·36 (0·54–3·43)†	1·72 (0·64–4·59)†	0·81 (0·23–2·93)†	**2·30 (1·36–3·89)**‡	**3·26 (1·54–6·92)**‡
Atomoxetine	..	..	0·99 (0·11–9·15)†	0·91 (0·11–7·77)†	0·33 (0·05–2·14)†	..	0·57 (0·22–1·47)†	..	1·04 (0·55–1·94)†	0·97 (0·47–2·02)*	1·11 (0·40–3·09)†	0·58 (0·18–1·93)†	1·49 (0·84–2·64)*	2·33 (1·28–4·25)*
Bupropion	..	..	..	..	0·33 (0·02–5·51)†	..	0·57 (0·06–5·77)†	..	1·05 (0·12–9·14)†	1·07 (0·13–8·92)†	1·12 (0·11–11·62)†	0·64 (0·06–6·37)†	1·51 (0·17–13·27)†	2·55 (0·33–19·93)†
Clonidine	..	..	..	..	..	..	1·71 (0·24–12·22)†	..	3·14 (0·51–19·33)†	..	3·36 (0·46–24·64)†	..	4·52 (0·75–27·03)†	..
Guanfacine	..	..	..	..	..	..	..	..	1·83 (0·74–4·57)†	..	1·97 (0·63–6·16)†	..	2·64 (1·20–5·81)*	..
Methylphenidate	..	..	..	..	..	..	..	..	..	..	1·07 (0·41–2·83)†	0·60 (0·19–1·92)†	1·44 (0·90–2·31)*	**2·39 (1·40–4·08)**§
Modafinil	..	..	..	..	..	..	..	..	..	..	..	..	1·34 (0·57–3·18)†	**4·01 (1·42–11·33)**‡

Data are odds ratio (95% CI). Values above 1 favour the treatment in the column and values below 1 favour the treatment in the row. Results in bold are significant. Drugs are reported in alphabetical order. Results are based on network estimates. No data for clonidine and guanfacine in adults are reported because no studies identified by our search tested these two drugs in adults. ADHD=attention-deficit hyperactivity disorder.

* Low quality of evidence.

† Very low quality of evidence.

‡ Moderate quality of evidence.

§ High quality of evidence.

With respect to ADHD core symptoms rated by clinicians in children and adolescents, all drugs were superior to placebo (figure 3, table 1). In adults, amphetamines, methylphenidate, bupropion, and atomoxetine were superior to placebo, but modafinil was not superior to placebo; no data were available for guanfacine and clonidine. In children, adolescents, and adults, amphetamines were significantly superior to modafinil, atomoxetine, and methylphenidate (table 1). Additionally, in children and adolescents, amphetamines were superior to guanfacine and methylphenidate was superior to atomoxetine. In adults, methylphenidate, atomoxetine, and bupropion were superior to modafinil. By contrast, according to teachers' ratings of children's ADHD core symptoms, only methylphenidate and modafinil were superior to placebo (no data were available for amphetamines and clonidine; table 1).

With respect to tolerability, in children and adolescents, only guanfacine and amphetamines were less well tolerated than placebo (figure 3, table 2). In adults, modafinil, amphetamines, methylphenidate, and atomoxetine were inferior to placebo (no data were available for guanfacine and clonidine). No differences in tolerability were noted between active drugs, in children, adolescents, and adults.

In children and adolescents, the common heterogeneity SD for efficacy (teachers' and clinicians' ratings) and tolerability was 0·355, 0·188, and 0·268, respectively. In adults, the common heterogeneity SD for efficacy rated by clinicians and tolerability was 0·178 and 0·282, respectively. The test of global inconsistency did not show any significant difference for the primary outcomes. Additional details are reported in the appendix (p 474).

Parents' ratings of their child's ADHD core symptoms and adults' self-ratings of their own ADHD core symptoms, with respect to efficacy of active drugs versus placebo, were similar to clinicians' ratings. Exceptions were guanfacine, which was not superior to placebo according to parents' ratings (SMD −0·23, 95% CI −0·90 to 0·45), and bupropion, which was not superior to placebo with respect to parents' ratings (0·24, −0·44 to 0·92) and adults' self-reports (−0·30, −0·61 to 0·01; appendix pp 475–76).

In children and adolescents, all compounds were superior to placebo on the CGI-I scale, except for clonidine (OR 2·78, 95% CI 0·91–8·53). In adults, amphetamines (4·86, 3·30–7·17), bupropion (3·43, 1·45–8·14), and methylphenidate (3·08, 2·04–4·65) were superior to placebo on the CGI-I scale (appendix p 476).

Weight was decreased significantly by amphetamines (in children and adolescents, SMD −0·71, 95% CI −1·15 to −0·27; in adults, −0·60, −1·03 to −0·18), methylphenidate (in children and adolescents, −0·77, −1·09 to −0·45; in adults, −0·74, −1·20 to −0·28), atomoxetine (in children and adolescents, −0·84, −1·16 to −0·52), and modafinil (in children and adolescents, −0·93, −1·59 to −0·26), compared with placebo (appendix pp 476, 477). Systolic blood pressure was increased with use of amphetamines (SMD 0·09, 95% CI 0·01–0·18) and atomoxetine (0·12, 0·02–0·22) in children and adolescents, and with use of methylphenidate (0·17, 0·05–0·30) in adults, compared with placebo (appendix p 477). Use of amphetamines (0·21, 0·12–0·31), atomoxetine (0·28, 0·18–0·37), and methylphenidate (0·24, 0·14–0·33) in children and adults, and atomoxetine (0·19, 0·08–0·30) and methylphenidate (0·20, 0·08–0·32) in adults, significantly increased diastolic blood pressure compared with placebo (appendix p 478).

For acceptability, compared with placebo, methylphenidate (OR 0·69, 95% CI 0·52–0·91) in children and adolescents and amphetamines (0·68, 0·49–0·95) in adults were significantly better (appendix p 478).

In subgroup and sensitivity analyses, data were sufficient to assess the effect of study length, comorbidities, IQ, crossover design, unfair dose comparisons, and data imputation. Findings of these analyses were generally robust (appendix pp 479–91). Because of a paucity of data, we could not assess the effect of gender, age (children *vs* adolescents), low risk of bias, medication status, and industry sponsorship. Sensitivity analyses investigating the effect of different maximum doses confirmed the results of the primary dose analysis (appendix pp 492–575).

Post-hoc analyses separating lisdexamfetamine from other amphetamines highlighted some differences. In children, lisdexamfetamine was less well tolerated compared with placebo (OR 2·69, 95% CI 1·40–5·16), whereas tolerability of the other amphetamines was slightly better (1·83, 0·84–4·02); in adults, the opposite pattern emerged (*vs* placebo: lisdexamfetamine, 2·74, 0·80–9·30; other amphetamines, 3·66, 1·36–9·87). Network meta-analyses heterogeneity for the dose and post-hoc analyses are reported in the appendix (pp 576–78).

Data for network meta-analyses inconsistency and SUCRA and mean rank are reported in the appendix (pp 579–85). Empirical heterogeneity variance for continuous outcomes for drug versus placebo comparisons was 0·05 (50% percentile) and 0·24 (75% percentile); for binary outcomes it was 0·12 (50% percentile) and 0·34 (75% percentile). Funnel plots are shown in the appendix (pp 630–32). We retained only a few studies—all in adults—with reported outcomes closest to 26 weeks or 52 weeks (appendix pp 586–88); therefore results for outcomes at these timepoints were deemed not informative.

Of 42 mixed comparisons (ie, combining direct and indirect evidence), the confidence in estimate for primary outcome comparisons was rated as very low in 13 comparisons, low in 18, moderate in ten, and high in one. Of 59 indirect comparisons, the confidence in estimate was very low in 37 comparisons, low in 20, and moderate in two (appendix pp 589–623, 633–43).

## Discussion

To the best of our knowledge, our network meta-analysis represents the most comprehensive comparative synthesis to date on the efficacy and tolerability of medications for children, adolescents, and adults with ADHD. We have addressed the limitations of previous network meta-analyses, which focused selectively on children and adolescents^19^, ^20^, ^21^, ^22^, ^23^, ^24^ or adults,^25^, ^26^, ^27^, ^28^ or included only published material,^21^, ^22^, ^23^, ^24^, ^26^ non-blinded trials,^19^, ^21^, ^22^, ^23^, ^24^ or non-core ADHD outcomes.^19^, ^22^, ^25^, ^28^

Overall, all medications, except modafinil in adults, were more efficacious than placebo for the short-term treatment of ADHD, and they were less efficacious and less well tolerated in adults than in children and adolescents. However, the included medications were not equivalent in relation to their mean effect size, which ranged from moderate to high and varied according to the type of rater. Furthermore, even though amphetamines were the most efficacious compounds in children, adolescents, and adults, the effects of medications varied across age groups for several outcomes. With respect to tolerability, in children, only amphetamines and guanfacine were less well tolerated than placebo, whereas in adults, methylphenidate, amphetamines, and atomoxetine were worse than placebo. Additionally, amphetamines significantly increased diastolic blood pressure in children and adolescents, but not in adults. In children and adolescents, methylphenidate was the only drug with better acceptability than placebo; in adults, amphetamines were the only compound with better acceptability than placebo. Atomoxetine had the lowest mean effect size in children and adolescents based on clinicians' ratings, but in adults, its efficacy on ADHD core symptoms was comparable with that of methylphenidate. The large confidence interval in relation to the efficacy and tolerability of bupropion, clonidine, guanfacine, and modafinil suggests that caution should be used when interpreting these data. Another relevant finding, which requires replication in head-to-head trials, is the absence of significant differences between amphetamines and methylphenidate on the CGI-I measure.

Accounting for all included outcomes, our results support methylphenidate in children and adolescents, and amphetamines in adults, as the first pharmacological choice for ADHD. In fact, in adults, amphetamines were not only the most efficacious compounds, as rated by clinicians and by self-report, but also as well tolerated as methylphenidate and the only compounds with better acceptability than placebo. In children and adolescents, even though amphetamines were marginally superior to methylphenidate according to clinicians' ratings, methylphenidate was the only compound with better acceptability than placebo and, unlike amphetamines, was not worse than placebo in terms of tolerability. Additionally, our results on secondary outcomes highlight the importance of monitoring weight and blood pressure changes with atomoxetine as much as with stimulants.

Our conclusions from this analysis concur partly with NICE guidelines,^9^ in which methylphenidate is recommended as the first choice in children and adolescents and methylphenidate or lisdexamfetamine as first choice in adults. Additionally, although NICE recommend atomoxetine or guanfacine as a possible third-line choice in children, our results suggest that, despite comparable efficacy on ADHD core symptoms rated by parents, atomoxetine was equal to placebo in terms of tolerability, whereas guanfacine was worse. However, it is noteworthy that the NICE recommendations were informed not only by empirical evidence but also by considerations on costs and licence and flexibility of formulations.

Although post-hoc analyses did indicate differences between the amphetamine prodrug lisdexamfetamine and other amphetamines, the few studies that we were able to include in this comparison (four studies of lisdexamfetamine in children and adolescents and one of amphetamines; and two studies of lisdexamfetamine in adults and one of amphetamines) prevent us from drawing any firm conclusions from these findings. We would, therefore, not feel confident at this stage to recommend lisdexamfetamine over the other amphetamines for adults, as was suggested by NICE, although based on UK costs.^9^

An important factor to consider in the interpretation of our findings is the medication dose. There is considerable interindividual variation in terms of most effective dose. In general, we found no substantial differences in either efficacy or tolerability across the various medications when the maximum dose allowed was the dose defined by the FDA or by guidelines (suggesting in general higher maximum doses than the FDA). We excluded some studies^9^, ^46^, ^47^ because they included doses higher than those recommended in available guidelines, thus poorly reflecting common clinical practice. It is possible that inclusion of these studies would have changed the efficacy and tolerability results.

In general, results for the primary outcomes were robust in our sensitivity analyses, suggesting that trials of short duration (<3 weeks), presence of psychiatric comorbidities, low IQ as an inclusion criterion, dose comparisons that we judged unfair, crossover design, and missing data imputation did not significantly affect the results.

Our study has some limitations. Although we did our best to include all available trials and retrieve unpublished data, we cannot rule out the possibility of missing information. The latest update of studies included in the network meta-analysis was in April, 2017. We did a PubMed search in May, 2018, and found only three additional studies that met our inclusion critieria.^48^, ^49^, ^50^ Since we already had 133 included studies, we decided that adding these three studies would not have changed the final results materially. Additionally, some nodes in our network included only few studies. To adhere to the assumption of transitivity and reduce the risk of biased estimates (for instance, those that included enrichment designs), we had to discard many studies that were initially selected as potentially relevant (appendix pp 26–235). Most included studies compared an active drug with placebo and the number of actual head-to-head trials was quite small, so comparative efficacy between interventions was frequently based on indirect comparisons.

We found significant statistical heterogeneity in the pairwise meta-analyses, and the study population in our review included participants with different previous exposures and responses to ADHD medications. These characteristics were quite evenly distributed across the included studies and across the different nodes in the network, therefore, even if they contributed to statistical heterogeneity, it is unlikely that they have implications in terms of clinical heterogeneity and affected the validity of our results. On the contrary, heterogeneity can be seen as increasing the external validity of our findings, because the patients seen in real-world clinical practice tend to have similar variations. Although we included studies that used different rating scales to assess the core symptoms of ADHD, we selected carefully only validated scales that measure exclusively the same triad of symptoms—ie, inattention, hyperactivity, and impulsivity.

Our results should also consider the risk of bias of individual studies and GRADE quality ratings. After gathering additional unpublished information, the overall number of unclear items—across all items of the risk of bias—decreased from 63·5% to 35·2%. This reduction points to an urgent need for complete and open reporting in this research area. Additionally, the confidence of estimate for primary outcomes was low or very low in multiple comparisons, reducing the certainty of the findings. Most very low ratings were for indirect comparisons, suggesting the need for additional well designed head-to-head studies. Whereas previous pairwise^16^, ^51^ or network meta-analyses^19^ of ADHD medications rated all comparisons as low or very low quality, attributable in part to unpublished information that we gathered and a more nuanced assessment, we could rate some comparisons as high or moderate quality. Of note, these comparisons included the most commonly used drugs for ADHD (ie, methylphenidate and amphetamines). Additionally, our stringent criteria for the risk of bias (ie, a study was assessed at overall low risk only when all individual items were at low risk) could have contributed to downgrade the final GRADE ratings.

We planned to do analyses for outcomes closest to 12 weeks, 26 weeks, and 52 weeks, but few data were available for 26 weeks and 52 weeks and analyses at these timepoints were, therefore, not possible. This scarcity of data reflects ethical issues associated with doing long-term, placebo-controlled, randomised controlled trials of effective treatments. Thus, our findings can inform only the choice of short-term medication treatment for ADHD. Moreover, because of a paucity of data, we were unable to properly undertake all the planned sensitivity analyses. Finally, we did not include studies of antipsychotic or tricyclic antidepressant compounds because, although commonly prescribed for patients with ADHD, they are not used routinely to treat ADHD core symptoms, and their inclusion would, therefore, violate the assumption of transitivity in the networks.

Notwithstanding these caveats, our findings represent the best currently available evidence base (not constrained by local costs and licencing) to inform future guidelines internationally and shared decision-making between patients, carers, and clinicians, when a balance has to be made between efficacy and tolerability of ADHD medications.

For the **FDA website** see https://www.fda.gov

For the **EMA website** see http://www.ema.europa.eu/ema

## Data sharing

## References

[1] American Psychiatric Association (2013). Diagnostic and statistical manual of mental disorders.

[2] Polanczyk G, de Lima MS, Horta BL, Biederman J, Rohde LA (2007). The worldwide prevalence of ADHD: a systematic review and metaregression analysis. Am J Psychiatry.

[3] Simon V, Czobor P, Balint S, Meszaros A, Bitter I (2009). Prevalence and correlates of adult attention-deficit hyperactivity disorder: meta-analysis. Br J Psychiatry.

[4] Doshi JA, Hodgkins P, Kahle J (2012). Economic impact of childhood and adult attention-deficit/hyperactivity disorder in the United States. J Am Acad Child Adolesc Psychiatry.

[5] Holden SE, Jenkins-Jones S, Poole CD, Morgan CL, Coghill D, Currie CJ (2013). The prevalence and incidence, resource use and financial costs of treating people with attention deficit/hyperactivity disorder (ADHD) in the United Kingdom (1998 to 2010). Child Adolesc Psychiatry Ment Health.

[6] Le HH, Hodgkins P, Postma MJ (2014). Economic impact of childhood/adolescent ADHD in a European setting: the Netherlands as a reference case. Eur Child Adolesc Psychiatry.

[7] Chai G, Governale L, McMahon AW, Trinidad JP, Staffa J, Murphy D (2012). Trends of outpatient prescription drug utilization in US children, 2002–2010. Pediatrics.

[8] Renoux C, Shin JY, Dell'Aniello S, Fergusson E, Suissa S (2016). Prescribing trends of attention-deficit hyperactivity disorder (ADHD) medications in UK primary care, 1995–2015. Br J Clin Pharmacol.

[9] National Institute for Health and Care Excellence (March, 2018). Attention deficit hyperactivity disorder: diagnosis and management. https://www.nice.org.uk/guidance/ng87.

[10] Bolea-Alamanac B, Nutt DJ, Adamou M (2014). Evidence-based guidelines for the pharmacological management of attention deficit hyperactivity disorder: update on recommendations from the British Association for Psychopharmacology. J Psychopharmacol.

[11] Kooij SJ, Bejerot S, Blackwell A (2010). European consensus statement on diagnosis and treatment of adult ADHD: the European Network Adult ADHD. BMC Psychiatry.

[12] Pliszka S (2007). Practice parameter for the assessment and treatment of children and adolescents with attention-deficit/hyperactivity disorder. J Am Acad Child Adolesc Psychiatry.

[13] Wolraich M, Brown L, Brown RT (2011). ADHD: clinical practice guideline for the diagnosis, evaluation, and treatment of attention-deficit/hyperactivity disorder in children and adolescents. Pediatrics.

[14] Canadian ADHD Resource Alliance (April 25, 2018). Canadian ADHD Practice Guidelines. https://www.caddra.ca/canadian-adhd-practice-guidelines.

[15] Banaschewski T, Buitelaar J, Chui CS (2016). Methylphenidate for ADHD in children and adolescents: throwing the baby out with the bathwater. Evid Based Ment Health.

[16] Punja S, Shamseer L, Hartling L (2016). Amphetamines for attention deficit hyperactivity disorder (ADHD) in children and adolescents. Cochrane Database Syst Rev.

[17] Romanos M, Reif A, Banaschewski T (2016). Methylphenidate for attention-deficit/hyperactivity disorder. JAMA.

[18] Cipriani A, Higgins JP, Geddes JR, Salanti G (2013). Conceptual and technical challenges in network meta-analysis. Ann Intern Med.

[19] Catala-Lopez F, Hutton B, Nunez-Beltran A (2017). The pharmacological and non-pharmacological treatment of attention deficit hyperactivity disorder in children and adolescents: a systematic review with network meta-analyses of randomised trials. PLoS One.

[20] Joseph A, Ayyagari R, Bischof M (2014). Systematic literature review and mixed treatment comparison of Gxr versus other treatments in children and adolescents with attention deficit hyperactivity disorder (ADHD). Value Health.

[21] Joseph A, Ayyagari R, Xie M (2017). Comparative efficacy and safety of attention-deficit/hyperactivity disorder pharmacotherapies, including guanfacine extended release: a mixed treatment comparison. Eur Child Adolesc Psychiatry.

[22] Li Y, Gao J, He S, Zhang Y, Wang Q (2017). An evaluation on the efficacy and safety of treatments for attention deficit hyperactivity disorder in children and adolescents: a comparison of multiple treatments. Mol Neurobiol.

[23] Luan R, Mu Z, Yue F, He S (2017). Efficacy and tolerability of different interventions in children and adolescents with attention deficit hyperactivity disorder. Front Psychiatry.

[24] Roskell NS, Setyawan J, Zimovetz EA, Hodgkins P (2014). Systematic evidence synthesis of treatments for ADHD in children and adolescents: indirect treatment comparisons of lisdexamfetamine with methylphenidate and atomoxetine. Curr Med Res Opin.

[25] Bushe C, Day K, Reed V (2016). A network meta-analysis of atomoxetine and osmotic release oral system methylphenidate in the treatment of attention-deficit/hyperactivity disorder in adult patients. J Psychopharmacol.

[26] de Chierrito OD, de Guerrero SP, Dos Borges RC (2017). Safety of treatments for ADHD in adults: pairwise and network meta-analyses. J Atten Disord.

[27] Zimovetz EA, Joseph A, Ayyagari R, Mauskopf JA (2018). A cost-effectiveness analysis of lisdexamfetamine dimesylate in the treatment of adults with attention-deficit/hyperactivity disorder in the UK. Eur J Health Econ.

[28] Peterson K, McDonagh MS, Fu R (2008). Comparative benefits and harms of competing medications for adults with attention-deficit hyperactivity disorder: a systematic review and indirect comparison meta-analysis. Psychopharmacology (Berl).

[29] King S, Griffin S, Hodges Z (2006). A systematic review and economic model of the effectiveness and cost-effectiveness of methylphenidate, dexamfetamine and atomoxetine for the treatment of attention deficit hyperactivity disorder in children and adolescents. Health Technol Assess.

[30] Cortese S, Adamo N, Mohr-Jensen C (2017). Comparative efficacy and tolerability of pharmacological interventions for attention-deficit/hyperactivity disorder in children, adolescents and adults: protocol for a systematic review and network meta-analysis. BMJ Open.

[31] Hutton B, Salanti G, Caldwell DM (2015). The PRISMA extension statement for reporting of systematic reviews incorporating network meta-analyses of health care interventions: checklist and explanations. Ann Intern Med.

[32] Cochrane Collaboration Assessing risk of bias in included studies. http://methods.cochrane.org/bias/assessing-risk-bias-included-studies.

[33] Salanti G, Del GC, Chaimani A, Caldwell DM, Higgins JP (2014). Evaluating the quality of evidence from a network meta-analysis. PLoS One.

[34] Martel MM, Schimmack U, Nikolas M, Nigg JT (2015). Integration of symptom ratings from multiple informants in ADHD diagnosis: a psychometric model with clinical utility. Psychol Assess.

[35] DerSimonian R, Laird N (1986). Meta-analysis in clinical trials. Control Clin Trials.

[36] Higgins JP, Thompson SG, Deeks JJ, Altman DG (2003). Measuring inconsistency in meta-analyses. BMJ.

[37] Miladinovic B, Hozo I, Chaimani A, Djulbegovic B (2014). Indirect treatment comparison. Stata J.

[38] Salanti G (2012). Indirect and mixed-treatment comparison, network, or multiple-treatments meta-analysis: many names, many benefits, many concerns for the next generation evidence synthesis tool. Res Synth Methods.

[39] Jackson D, Barrett JK, Rice S, White IR, Higgins JP (2014). A design-by-treatment interaction model for network meta-analysis with random inconsistency effects. Stat Med.

[40] Turner RM, Davey J, Clarke MJ, Thompson SG, Higgins JP (2012). Predicting the extent of heterogeneity in meta-analysis, using empirical data from the Cochrane Database of Systematic Reviews. Int J Epidemiol.

[41] Rhodes KM, Turner RM, Higgins JP (2015). Predictive distributions were developed for the extent of heterogeneity in meta-analyses of continuous outcome data. J Clin Epidemiol.

[42] Veroniki AA, Vasiliadis HS, Higgins JP, Salanti G (2013). Evaluation of inconsistency in networks of interventions. Int J Epidemiol.

[43] Higgins JP, Jackson D, Barrett JK, Lu G, Ades AE, White IR (2012). Consistency and inconsistency in network meta-analysis: concepts and models for multi-arm studies. Res Synth Methods.

[44] Salanti G, Ades AE, Ioannidis JP (2011). Graphical methods and numerical summaries for presenting results from multiple-treatment meta-analysis: an overview and tutorial. J Clin Epidemiol.

[45] Ermer JC, Pennick M, Frick G (2016). Lisdexamfetamine dimesylate: prodrug delivery, amphetamine exposure and duration of efficacy. Clin Drug Investig.

[46] Retz W, Rosler M, Ose C (2012). Multiscale assessment of treatment efficacy in adults with ADHD: a randomized placebo-controlled, multi-centre study with extended-release methylphenidate. World J Biol Psychiatry.

[47] Biederman J, Mick E, Surman C (2010). A randomized, 3-phase, 34-week, double-blind, long-term efficacy study of osmotic-release oral system-methylphenidate in adults with attention-deficit/hyperactivity disorder. J Clin Psychopharmacol.

[48] Weisler RH, Greenbaum M, Arnold V (2017). Efficacy and safety of SHP465 mixed amphetamine salts in the treatment of attention-deficit/hyperactivity disorder in adults: results of a randomized, double-blind, placebo-controlled, forced-dose clinical study. CNS Drugs.

[49] Brams M, Childress AC, Greenbaum M (2018). SHP465 mixed amphetamine salts in the treatment of attention-deficit/hyperactivity disorder in children and adolescents: results of a randomized, double-blind placebo-controlled study. J Child Adolesc Psychopharmacol.

[50] Griffiths KR, Leikauf JE, Tsang TW (2018). Response inhibition and emotional cognition improved by atomoxetine in children and adolescents with ADHD: the ACTION randomized controlled trial. J Psychiatr Res.

[51] Storebo OJ, Ramstad E, Krogh HB (2015). Methylphenidate for children and adolescents with attention deficit hyperactivity disorder (ADHD). Cochrane Database Syst Rev.

