# Supplementary material for: Comparative efficacy and tolerability of medications for attention-deficit hyperactivity disorder in children, adolescents, and adults: a systematic review and network meta-analysis
Source: Lancet Psychiatry. 2018 Sep;5(9):727–38. doi: 10.1016/S2215-0366(18)30269-4 (PMC6109107; doi:10.1016/S2215-0366(18)30269-4)
Supplement: Supplementary appendix [file mmc1.pdf]

THE LANCET
Psychiatry

Supplementary appendix

This appendix formed part of the original submission and has been peer reviewed. We post it as supplied by the authors.

Supplement to: Cortese S, Adamo N, Del Giovane C, et al. Comparative efficacy and tolerability of medications for attention-deficit hyperactivity disorder in children, adolescents, and adults: a systematic review and network meta-analysis. *Lancet Psychiatry* 2018; published online Aug 7. http://dx.doi.org/10.1016/S2215-0366(18)30269-4.

ONLINE APPENDIX

Index

Appendix 1. Additional details on search strategy………………………………………….. .p.3
Appendix 2. Additional details on selection criteria……………………………………………p.16
Appendix 3. Additional details on study selection, data extraction and risk of bias/quality assessment………………………………………………………………………………p.18
Appendix 4. Additional details on the statistical analysis… p.20
Appendix 5. Criteria for judging the confidence in network estimates.. ....p.22
Appendix 6. Additional *post hoc* analyses and changes to the pre-specified protocol…............p.25
Appendix 7. Studies/citations discarded after assessing their full text, with reasons for exclusion...p.26
Appendix 8. Studies/citations retained for the network meta-analysis ....p. 236
Table S1. Scales/subscales for children/adolescents considered for possible inclusion..............p. 273
Table S2. Scales/subscales for adults considered for possible inclusion ....p. 275
Table S3. Maximum FDA licensed doses or maximum doses recommended in guidelines/formularies for children/adolescents..p. 277
Table S4. Maximum FDA licensed doses or maximum doses recommended in guidelines/formularies for adults..p. 279
Table S5. Washout periods ....p. 280
Table S6. Starting doses in children/adolescents ....p. 281
Table S7. Starting doses in adults ....p. 282
Table S8. Inclusion/exclusion criteria for each study included in the network meta-analysis...p.283
Table S9. Participants medication status at baseline, for each study included in the network meta-analysis ....p. 330
Table S10. Characteristics of the trials included in the network meta-analysis ....p. 356
Tables S11. Rating of individual items of the Risk of Bias tool for each study ....p. 382
Tables S12. Results of the pairwise meta-analyses for each of the primary and secondary outcomes closest to 12 weeks in the Main dose analysis, and related heterogeneity ....p. 459
Tables S13. Results of the network meta-analyses for each of the primary outcomes closest to 12 weeks in the Main dose analysis ....p. 472
Tables S14. NMA heterogeneity, Main dose analysis, primary outcomes ....p. 474
Tables S15. Results of the network meta-analyses for each of the secondary outcomes closest to 12 weeks in the Main dose analysis...p. 475
Tables S16. Sensitivity analyses, primary outcomes for the Main dose analysis, outcomes closest to 12 weeks ....p. 479
Tables S17. Results of the *dose* analyses for each of the primary and secondary outcomes closest to 12 weeks ....p. 492
Tables S18. NMA heterogeneity, *post hoc* analyses, primary outcomes.....................................p. 576
Tables S19. Evaluation of incoherence..p. 579
Tables S20. Ranking according to SUCRA and Mean rank for the primary and secondary outcomes, Main dose analysis, closest to 12 weeks ....p. 582
Tables S21. Results of the pairwise meta-analyses for each of the primary and secondary outcomes in the Main, FDA and Inclusive dose analysis (outcomes closest to 26 and 52 weeks)...p. 586
Tables S22. Summary of the GRADE ratings ....p. 589
Figures S1. Network plots for the secondary outcomes, Main dose analysis,

outcomes closest to 12 weeks ....p. 624
Figures S2. Comparisons adjusted funnel plots....p. 630
Figures S3. Bar plots showing the contribution of the risk of bias from each direct comparison to the network estimates ....p. 633
Figures S4. Plots presenting the confidence and predictive intervals for each network estimate ....p. 637
Figures S5. Bar plots showing the contribution of indirectness from each direct comparison to the network estimates....p. 640
Supplemental References....p. 644
Additional acknowledgments….p. 648

Appendix 1. Additional details on search strategy

Search in electronic sources

- Two experienced medical information specialists (FS and JX) developed the search strategy. The Cochrane Handbook [1] and Cochrane's MECIR [2] for conducting the search, PRISMA guideline for reporting the search [3], and PRESS guideline for peer-reviewing the search strategies were followed. [4] Keywords were collected through experts' opinion, controlled vocabulary (APA Thesaurus, CINAHL Headings, Medical Subject Headings = MeSH, and Excerpta Medica Tree = EMTREE), and reviewing the primary search results. Because of poor reporting of outcomes in medical research, [5-9] the search was not limited adding specific outcomes.

- The following electronic databases and international trial registries were searched PubMed, BIOSIS Previews, CINAHL, Cochrane Library, EMBASE, ERIC, MEDLINE, PsycINFO, OpenGrey, Web of Science Core Collection, ProQuest Dissertations & Theses: UK & Ireland, ProQuest Dissertations & Theses A&I, and WHO International Trials Registry Platform (CTRP) (including *ClinicalTrials.gov*)

- The WHO International Trials Registry Platform (CTRP) includes the following:
  - *Australian New Zealand Clinical Trials Registry (ANZCTR) (including clinical trials from Therapeutic Goods Administration (TGA))*
  - *Brazilian Clinical Trials Registry (ReBec)*
  - *Chinese Clinical Trial Register (ChiCTR)*
  - *Clinical Research Information Service (CRiS), Republic of Korea*
  - *ClinicalTrials.gov (including clinical trials from FDA)*
  - *Clinical Trials Registry - India (CTRI)*
  - *Cuban Public Registry of Clinical Trials (RPCEC)*
  - *EU Clinical Trials Register (EU-CTR) (including clinical trials from the European Medicines Agency (EMA))*
  - *German Clinical Trials Register (DRKS)*
  - *Iranian Registry of Clinical Trials (IRCT)*
  - *ISRCTN.org (including clinical trials from controlled-trials.com, The Wellcome Trust (UK), UK trials (UK), Action Medical Research (UK), the Medicines and Healthcare products Regulatory Agency (MHRA), and National Research Register)*
  - *Japan Primary Registries Network (JPRN) (including clinical trials from UMIN-CTR, JapicCTI, and JMACCT)*
  - *Pan African Clinical Trial Registry (PACTR)*
  - *Sri Lanka Clinical Trials Registry (SLCTR)*
  - *The Netherlands National Trial Register (NTR)*
  - *Thai Clinical Trials Register (TCTR)*

Search syntax for each database (in alphabetical order)

A. BIOSIS Previews

TOPIC: (adhd OR hkd OR addh OR hyperkine* OR "attention deficit*" OR hyper-activ* OR hyperactiv* OR overactive OR inattentive OR impulsiv*) AND **TOPIC:** (Adderall OR Amphetamine OR Desoxyn* OR Phenopromin OR Amfetamine OR Phenamine OR Centramina OR Fenamine OR Levoamphetamine OR Dexamfetamine OR Dexamphetamine OR Dexedrine OR Dextroamphetamine OR DextroStat OR Oxydess OR Methylamphetamine OR Methylenedioxyamphetamine OR Methamphetamine OR Chloroamphetamine OR Metamfetamine OR Deoxyephedrine OR Desoxyephedrine OR Ecstasy OR Atomoxetine OR Biphentin OR Bupropion OR Amfebutamone OR Zyntabac OR Quomen OR Wellbutrin OR Zyban OR Catapres* OR Clonidine OR Klofenil OR Clofenil OR Chlophazolin OR Gemiton OR Hemiton OR Isoglaucon OR Klofelin OR Clopheline OR Clofelin OR Dixarit OR Concerta OR Daytrana OR Methylphenidate OR Equasym OR Methylin OR Tsentedrin OR Centedrin OR Phenidylate OR Ritalin* OR Duraclon OR Elvanse OR Focalin OR Dexmethylphenidate OR Guanfacine OR Estulic OR Tenex OR Kapvay OR Lisdexamfetamine OR Vyvanse OR Medikinet OR Metadate OR Modafinil OR Nexiclon OR Quillivant OR Strattera) AND **TOPIC:** (RCT OR ((clinical OR control*) NEAR/10 trial*) OR crossover OR "cross over" OR cross-over OR randomi* OR (random* NEAR/1 (allocat* OR assign* OR select*)) OR blind* OR placebo OR "control group")
Indexes=BIOSIS Previews Timespan=All years

B. EMBASE

1. exp Attention Deficit Disorder with Hyperactivity/ or (adhd or hkd or addh or hyperkine* or "attention deficit*" or hyper-activ* or hyperactiv* or overactive or inattentive or impulsiv*).ti,ab.
2. exp Amphetamines/ or exp Bupropion/ or exp Clonidine/ or exp Methylphenidate/ or exp Dexmethylphenidate/ or exp Guanfacine/ or (Adderall or Amphetamine or Desoxyn* or Phenopromin or Amfetamine or Phenamine or Centramina or Fenamine or Levoamphetamine or Dexamfetamine or Dexamphetamine or Dexedrine or Dextroamphetamine or DextroStat or Oxydess or Methylamphetamine or Methylenedioxyamphetamine or Methamphetamine or Chloroamphetamine or Metamfetamine or Deoxyephedrine or Desoxyephedrine or Ecstasy or Atomoxetine or Biphentin or Bupropion or Amfebutamone or Zyntabac or Quomen or Wellbutrin or Zyban or Catapres* or Clonidine or Klofenil or Clofenil or Chlophazolin or Gemiton or Hemiton or Isoglaucon or Klofelin or Clopheline or Clofelin or Dixarit or Concerta or Daytrana or Methylphenidate or Equasym or Methylin or Tsentedrin or Centedrin or Phenidylate or Ritalin* or Duraclon or Elvanse or Focalin or Dexmethylphenidate or Guanfacine or Estulic or Tenex or Kapvay or Lisdexamfetamine or Vyvanse or Medikinet or Metadate or Modafinil or Nexiclon or Quillivant or Strattera).ti,ab.
3. (random$ or factorial$ or crossover$ or (cross over$) or cross-over$ or placebo$ or (doubl$ adj blind$) or (singl$ adj blind$) or assign$ or allocat$ or volunteer$).mp. or crossover-procedure/ or double-blind procedure/ or randomized controlled trial/ or single-blind procedure/
4. limit 3 to human
5. 1 and 2 and 4

C. ERIC

((SU.EXACT.EXPLODE("Attention Deficit Disorders") OR ti(adhd OR hkd OR addh OR hyperkine* OR "attention deficit*" OR hyper-activ* OR hyperactiv* OR overactive OR inattentive OR impulsiv*) OR ab(adhd OR hkd OR addh OR hyperkine* OR "attention deficit*" OR hyper-activ* OR hyperactiv* OR overactive OR inattentive OR impulsiv*)) AND (ti(Adderall OR Amphetamine OR Desoxyn* OR Phenopromin OR Amfetamine OR Phenamine OR Centramina OR Fenamine OR Levoamphetamine OR Dexamfetamine OR Dexamphetamine OR Dexedrine OR Dextroamphetamine OR DextroStat OR Oxydess OR Methylamphetamine OR Methylenedioxyamphetamine OR Methamphetamine OR Chloroamphetamine OR Metamfetamine OR Deoxyephedrine OR Desoxyephedrine OR Ecstasy OR Atomoxetine OR Biphentin OR Bupropion OR Amfebutamone OR Zyntabac OR Quomen OR Wellbutrin OR Zyban OR Catapres* OR Clonidine OR Klofenil OR Clofenil OR Chlophazolin OR Gemiton OR Hemiton OR Isoglaucon OR Klofelin OR Clopheline OR Clofelin OR Dixarit OR Concerta OR Daytrana OR Methylphenidate OR Equasym OR Methylin OR Tsentedrin OR Centedrin OR Phenidylate OR Ritalin* OR Duraclon OR Elvanse OR Focalin OR Dexmethylphenidate OR Guanfacine OR Estulic OR Tenex OR Kapvay OR Lisdexamfetamine OR Vyvanse OR Medikinet OR Metadate OR Modafinil OR Nexiclon OR Quillivant OR Strattera) OR ab(Adderall OR Amphetamine OR Desoxyn* OR Phenopromin OR Amfetamine OR Phenamine OR Centramina OR Fenamine OR Levoamphetamine OR Dexamfetamine OR Dexamphetamine OR Dexedrine OR Dextroamphetamine OR DextroStat OR Oxydess OR Methylamphetamine OR Methylenedioxyamphetamine OR Methamphetamine OR Chloroamphetamine OR Metamfetamine OR Deoxyephedrine OR Desoxyephedrine OR Ecstasy OR Atomoxetine OR Biphentin OR Bupropion OR Amfebutamone OR Zyntabac OR Quomen OR Wellbutrin OR Zyban OR Catapres* OR Clonidine OR Klofenil OR Clofenil OR Chlophazolin OR Gemiton OR Hemiton OR Isoglaucon OR Klofelin OR Clopheline OR Clofelin OR Dixarit OR Concerta OR Daytrana OR Methylphenidate OR Equasym OR Methylin OR Tsentedrin OR Centedrin OR Phenidylate OR Ritalin* OR Duraclon OR Elvanse OR Focalin OR Dexmethylphenidate OR Guanfacine OR Estulic OR Tenex OR Kapvay OR Lisdexamfetamine OR Vyvanse OR Medikinet OR Metadate OR Modafinil OR Nexiclon OR Quillivant OR Strattera))) AND (ti(RCT OR ((clinical OR control*) NEAR/10 trial*) OR crossover OR "cross over" OR cross-over OR randomi* OR (random* NEAR/1 (allocat* OR assign* OR select*)) OR blind* OR placebo OR "control group") OR ab(RCT OR ((clinical OR control*) NEAR/10 trial*) OR crossover OR "cross over" OR cross-over OR randomi* OR (random* NEAR/1 (allocat* OR assign* OR select*)) OR blind* OR placebo OR "control group"))

D. International Clinical Trials Registry Platform (WHO ICTRP)

(adhd OR hkd OR addh OR hyperkine* OR "attention deficit*" OR hyper-activ* OR hyperactiv* OR overactive OR inattentive OR impulsiv*) in Condition Field AND (Adderall OR Amphetamine OR Desoxyn* OR Phenopromin OR Amfetamine OR Phenamine OR Centramina OR Fenamine OR Levoamphetamine OR Dexamfetamine OR Dexamphetamine OR Dexedrine OR Dextroamphetamine OR DextroStat OR Oxydess OR Methylamphetamine OR Methylenedioxyamphetamine OR Methamphetamine OR Chloroamphetamine OR Metamfetamine OR Deoxyephedrine OR Desoxyephedrine OR Ecstasy OR Atomoxetine OR Biphentin OR Bupropion OR Amfebutamone OR Zyntabac OR Quomen OR Wellbutrin OR Zyban OR Catapres* OR Clonidine OR Klofenil OR Clofenil OR Chlophazolin OR Gemiton OR Hemiton OR Isoglaucon OR Klofelin OR Clopheline OR Clofelin OR Dixarit OR Concerta OR Daytrana OR Methylphenidate OR Equasym OR Methylin OR Tsentedrin OR Centedrin OR Phenidylate OR Ritalin* OR Duraclon OR Elvanse OR Focalin OR Dexmethylphenidate OR Guanfacine OR Estulic OR Tenex OR Kapvay OR

Lisdexamfetamine OR Vyvanse OR Medikinet OR Metadate OR Modafinil OR Nexiclon OR Quillivant OR Strattera) in Intervention Field

E. MEDLINE
1. exp Attention Deficit Disorder with Hyperactivity/ or (adhd or hkd or addh or hyperkine* or "attention deficit*" or hyper-activ* or hyperactiv* or overactive or inattentive or impulsiv*).ti,ab.
2. exp Amphetamines/ or exp Bupropion/ or exp Clonidine/ or exp Methylphenidate/ or exp Dexmethylphenidate/ or exp Guanfacine/ or (Adderall or Amphetamine or Desoxyn* or Phenopromin or Amfetamine or Phenamine or Centramina or Fenamine or Levoamphetamine or Dexamfetamine or Dexamphetamine or Dexedrine or Dextroamphetamine or DextroStat or Oxydess or Methylamphetamine or Methylenedioxyamphetamine or Methamphetamine or Chloroamphetamine or Metamfetamine or Deoxyephedrine or Desoxyephedrine or Ecstasy or Atomoxetine or Biphentin or Bupropion or Amfebutamone or Zyntabac or Quomen or Wellbutrin or Zyban or Catapres* or Clonidine or Klofenil or Clofenil or Chlophazolin or Gemiton or Hemiton or Isoglaucon or Klofelin or Clopheline or Clofelin or Dixarit or Concerta or Daytrana or Methylphenidate or Equasym or Methylin or Tsentedrin or Centedrin or Phenidylate or Ritalin* or Duraclon or Elvanse or Focalin or Dexmethylphenidate or Guanfacine or Estulic or Tenex or Kapvay or Lisdexamfetamine or Vyvanse or Medikinet or Metadate or Modafinil or Nexiclon or Quillivant or Strattera).ti,ab.
3. (randomized controlled trial or controlled clinical trial).pt. or random$.ab. or placebo.ab. or drug therapy.fs. or trial.ab. or groups.ab.
4. exp animals/ not humans.sh.
5. 3 not 4
6. 1 and 2 and 5

F. ProQuest Dissertations & Theses: UK & Ireland and ProQuest Dissertations & Theses A&I
((ti(adhd OR hkd OR addh OR hyperkine* OR "attention deficit*" OR hyper-activ* OR hyperactiv* OR overactive OR inattentive OR impulsiv*) OR ab(adhd OR hkd OR addh OR hyperkine* OR "attention deficit*" OR hyper-activ* OR hyperactiv* OR overactive OR inattentive OR impulsiv*)) AND (ti(Adderall OR Amphetamine OR Desoxyn* OR Phenopromin OR Amfetamine OR Phenamine OR Centramina OR Fenamine OR Levoamphetamine OR Dexamfetamine OR Dexamphetamine OR Dexedrine OR Dextroamphetamine OR DextroStat OR Oxydess OR Methylamphetamine OR Methylenedioxyamphetamine OR Methamphetamine OR Chloroamphetamine OR Metamfetamine OR Deoxyephedrine OR Desoxyephedrine OR Ecstasy OR Atomoxetine OR Biphentin OR Bupropion OR Amfebutamone OR Zyntabac OR Quomen OR Wellbutrin OR Zyban OR Catapres* OR Clonidine OR Klofenil OR Clofenil OR Chlophazolin OR Gemiton OR Hemiton OR Isoglaucon OR Klofelin OR Clopheline OR Clofelin OR Dixarit OR Concerta OR Daytrana OR Methylphenidate OR Equasym OR Methylin OR Tsentedrin OR Centedrin OR Phenidylate OR Ritalin* OR Duraclon OR Elvanse OR Focalin OR Dexmethylphenidate OR Guanfacine OR Estulic OR Tenex OR Kapvay OR Lisdexamfetamine OR Vyvanse OR Medikinet OR Metadate OR Modafinil OR Nexiclon OR Quillivant OR Strattera) OR ab(Adderall OR Amphetamine OR Desoxyn* OR Phenopromin OR Amfetamine OR Phenamine OR Centramina OR Fenamine OR Levoamphetamine OR Dexamfetamine OR Dexamphetamine OR Dexedrine OR Dextroamphetamine OR DextroStat OR Oxydess OR Methylamphetamine OR Methylenedioxyamphetamine OR Methamphetamine OR Chloroamphetamine OR Metamfetamine OR Deoxyephedrine OR Desoxyephedrine OR Ecstasy OR Atomoxetine OR Biphentin OR Bupropion OR Amfebutamone OR Zyntabac OR Quomen OR Wellbutrin OR Zyban OR Catapres* OR Clonidine OR Klofenil OR Clofenil OR Chlophazolin OR Gemiton OR Hemiton OR Isoglaucon OR Klofelin OR Clopheline OR Clofelin OR Dixarit OR Concerta OR Daytrana OR Methylphenidate OR Equasym OR Methylin OR Tsentedrin OR Centedrin OR Phenidylate OR Ritalin* OR Duraclon OR Elvanse OR Focalin OR Dexmethylphenidate OR Guanfacine OR Estulic OR Tenex OR Kapvay OR Lisdexamfetamine OR Vyvanse OR Medikinet OR Metadate OR Modafinil OR Nexiclon OR Quillivant OR Strattera))) AND (ti(RCT OR ((clinical OR control*) NEAR/10 trial*) OR crossover OR "cross over" OR cross-over OR randomi* OR (random* NEAR/1 (allocat* OR assign* OR select*)) OR blind* OR placebo OR "control group") OR ab(RCT OR ((clinical OR control*) NEAR/10 trial*) OR crossover OR "cross over" OR cross-over OR randomi* OR (random* NEAR/1 (allocat* OR assign* OR select*)) OR blind* OR placebo OR "control group"))

G. PsycINFO
1. exp Attention Deficit Disorder with Hyperactivity/ or (adhd or hkd or addh or hyperkine* or "attention deficit*" or hyper-activ* or hyperactiv* or overactive or inattentive or impulsiv*).ti,ab.
2. exp Bupropion/ or exp Clonidine/ or exp Methylphenidate/ or (Adderall or Amphetamine or Desoxyn* or Phenopromin or Amfetamine or Phenamine or Centramina or Fenamine or Levoamphetamine or Dexamfetamine or Dexamphetamine or Dexedrine or Dextroamphetamine or DextroStat or Oxydess or Methylamphetamine or Methylenedioxyamphetamine or Methamphetamine or Chloroamphetamine or Metamfetamine or Deoxyephedrine or Desoxyephedrine or Ecstasy or Atomoxetine or Biphentin or Bupropion or Amfebutamone or Zyntabac or Quomen or

Wellbutrin or Zyban or Catapres* or Clonidine or Klofenil or Clofenil or Chlophazolin or Gemiton or Hemiton or Isoglaucon or Klofelin or Clopheline or Clofelin or Dixarit or Concerta or Daytrana or Methylphenidate or Equasym or Methylin or Tsentedrin or Centedrin or Phenidylate or Ritalin* or Duraclon or Elvanse or Focalin or Dexmethylphenidate or Guanfacine or Estulic or Tenex or Kapvay or Lisdexamfetamine or Vyvanse or Medikinet or Metadate or Modafinil or Nexiclon or Quillivant or Strattera).ti,ab.
3. (double-blind or random* assigned or control).tw.
4. and/1-3
5. limit 4 to human

H. PubMed
("Attention Deficit Disorder with Hyperactivity"[Mesh] OR adhd[tiab] OR hkd[tiab] OR addh[tiab] OR hyperkine*[tiab] OR "attention deficit*"[tiab] OR hyper-activ*[tiab] OR hyperactiv*[tiab] OR overactive[tiab] OR inattentive[tiab] OR impulsiv*[tiab]) AND ("Amphetamines"[Mesh] OR "Bupropion"[Mesh] OR "Clonidine"[Mesh] OR "Methylphenidate"[Mesh] OR "Dexmethylphenidate"[Mesh] OR "Guanfacine"[Mesh] OR Adderall[tiab] OR Amphetamine[tiab] OR Desoxyn*[tiab] OR Phenopromin[tiab] OR Amfetamine[tiab] OR Phenamine[tiab] OR Centramina[tiab] OR Fenamine[tiab] OR Levoamphetamine[tiab] OR Dexamfetamine[tiab] OR Dexamphetamine[tiab] OR Dexedrine[tiab] OR Dextroamphetamine[tiab] OR DextroStat[tiab] OR Oxydess[tiab] OR Methylamphetamine[tiab] OR Methylenedioxyamphetamine[tiab] OR Methamphetamine[tiab] OR Chloroamphetamine[tiab] OR Metamfetamine[tiab] OR Deoxyephedrine[tiab] OR Desoxyephedrine[tiab] OR Ecstasy[tiab] OR Atomoxetine[tiab] OR Biphentin[tiab] OR Bupropion[tiab] OR Amfebutamone[tiab] OR Zyntabac[tiab] OR Quomen[tiab] OR Wellbutrin[tiab] OR Zyban[tiab] OR Catapres*[tiab] OR Clonidine[tiab] OR Klofenil[tiab] OR Clofenil[tiab] OR Chlophazolin[tiab] OR Gemiton[tiab] OR Hemiton[tiab] OR Isoglaucon[tiab] OR Klofelin[tiab] OR Clopheline[tiab] OR Clofelin[tiab] OR Dixarit[tiab] OR Concerta[tiab] OR Daytrana[tiab] OR Methylphenidate[tiab] OR Equasym[tiab] OR Methylin[tiab] OR Tsentedrin[tiab] OR Centedrin[tiab] OR Phenidylate[tiab] OR Ritalin*[tiab] OR Duraclon[tiab] OR Elvanse[tiab] OR Focalin[tiab] OR Dexmethylphenidate[tiab] OR Guanfacine[tiab] OR Estulic[tiab] OR Tenex[tiab] OR Kapvay[tiab] OR Lisdexamfetamine[tiab] OR Vyvanse[tiab] OR Medikinet[tiab] OR Metadate[tiab] OR Modafinil[tiab] OR Nexiclon[tiab] OR Quillivant[tiab] OR Strattera[tiab]) AND (randomized controlled trial[pt] OR controlled clinical trial[pt] OR randomized[tiab] OR placebo[tiab] OR clinical trials as topic[mesh:noexp] OR randomly[tiab] OR trial[ti]) NOT (animals[mh] NOT humans[mh])

I. SIGLE
(adhd OR hkd OR addh OR hyperkine* OR "attention deficit*" OR hyper-activ* OR hyperactiv* OR overactive OR inattentive OR impulsiv*) AND (Adderall OR Amphetamine OR Desoxyn* OR Phenopromin OR Amfetamine OR Phenamine OR Centramina OR Fenamine OR Levoamphetamine OR Dexamfetamine OR Dexamphetamine OR Dexedrine OR Dextroamphetamine OR DextroStat OR Oxydess OR Methylamphetamine OR Methylenedioxyamphetamine OR Methamphetamine OR Chloroamphetamine OR Metamfetamine OR Deoxyephedrine OR Desoxyephedrine OR Ecstasy OR Atomoxetine OR Biphentin OR Bupropion OR Amfebutamone OR Zyntabac OR Quomen OR Wellbutrin OR Zyban OR Catapres* OR Clonidine OR Klofenil OR Clofenil OR Chlophazolin OR Gemiton OR Hemiton OR Isoglaucon OR Klofelin OR Clopheline OR Clofelin OR Dixarit OR Concerta OR Daytrana OR Methylphenidate OR Equasym OR Methylin OR Tsentedrin OR Centedrin OR Phenidylate OR Ritalin* OR Duraclon OR Elvanse OR Focalin OR Dexmethylphenidate OR Guanfacine OR Estulic OR Tenex OR Kapvay OR Lisdexamfetamine OR Vyvanse OR Medikinet OR Metadate OR Modafinil OR Nexiclon OR Quillivant OR Strattera)

J. The Cochrane Library
#1 MeSH descriptor: [Attention Deficit Disorder with Hyperactivity] explode all trees
#2 (adhd or hkd or addh or hyperkine* or "attention deficit*" or hyper-activ* or hyperactiv* or overactive or inattentive or impulsiv*):ti,ab
#3 MeSH descriptor: [Amphetamines] explode all trees
#4 MeSH descriptor: [Bupropion] explode all trees
#5 MeSH descriptor: [Clonidine] explode all trees
#6 MeSH descriptor: [Methylphenidate] explode all trees
#7 MeSH descriptor: [Dexmethylphenidate] explode all trees
#8 MeSH descriptor: [Guanfacine] explode all trees
#9 (Adderall or Amphetamine or Desoxyn* or Phenopromin or Amfetamine or Phenamine or Centramina or Fenamine or Levoamphetamine or Dexamfetamine or Dexamphetamine or Dexedrine or Dextroamphetamine or DextroStat or Oxydess or Methylamphetamine or Methylenedioxyamphetamine or Methamphetamine or Chloroamphetamine or Metamfetamine or Deoxyephedrine or Desoxyephedrine or Ecstasy or Atomoxetine or Biphentin or Bupropion or Amfebutamone or Zyntabac or Quomen or Wellbutrin or Zyban or Catapres* or Clonidine or Klofenil or Clofenil or Chlophazolin or Gemiton or Hemiton or Isoglaucon or Klofelin or Clopheline or Clofelin or Dixarit or Concerta or

Daytrana or Methylphenidate or Equasym or Methylin or Tsentedrin or Centedrin or Phenidylate or Ritalin* or Duraclon or Elvanse or Focalin or Dexmethylphenidate or Guanfacine or Estulic or Tenex or Kapvay or Lisdexamfetamine or Vyvanse or Medikinet or Metadate or Modafinil or Nexiclon or Quillivant or Strattera):ti,ab
#10 #1 or #2
#11 #3 or #4 or #5 or #6 or #7 or #8 or #9
#12 #10 and #11

K. Web of Science
TOPIC: (adhd OR hkd OR addh OR hyperkine* OR "attention deficit*" OR hyper-activ* OR hyperactiv* OR overactive OR inattentive OR impulsiv*) AND **TOPIC:** (Adderall OR Amphetamine OR Desoxyn* OR Phenopromin OR Amfetamine OR Phenamine OR Centramina OR Fenamine OR Levoamphetamine OR Dexamfetamine OR Dexamphetamine OR Dexedrine OR Dextroamphetamine OR DextroStat OR Oxydess OR Methylamphetamine OR Methylenedioxyamphetamine OR Methamphetamine OR Chloroamphetamine OR Metamfetamine OR Deoxyephedrine OR Desoxyephedrine OR Ecstasy OR Atomoxetine OR Biphentin OR Bupropion OR Amfebutamone OR Zyntabac OR Quomen OR Wellbutrin OR Zyban OR Catapres* OR Clonidine OR Klofenil OR Clofenil OR Chlophazolin OR Gemiton OR Hemiton OR Isoglaucon OR Klofelin OR Clopheline OR Clofelin OR Dixarit OR Concerta OR Daytrana OR Methylphenidate OR Equasym OR Methylin OR Tsentedrin OR Centedrin OR Phenidylate OR Ritalin* OR Duraclon OR Elvanse OR Focalin OR Dexmethylphenidate OR Guanfacine OR Estulic OR Tenex OR Kapvay OR Lisdexamfetamine OR Vyvanse OR Medikinet OR Metadate OR Modafinil OR Nexiclon OR Quillivant OR Strattera) AND **TOPIC:** (RCT OR ((clinical OR control*) NEAR/10 trial*) OR crossover OR "cross over" OR cross-over OR randomi* OR (random* NEAR/1 (allocat* OR assign* OR select*)) OR blind* OR placebo OR "control group")
Indexes=SCI-EXPANDED, SSCI, CPCI-S, CPCI-SSH Timespan=All years

Pertinent reviews screened to find any possible additional pertinent study

Pertinent reviews were retrieved from the search reported above as well from a targeted search in Pubmed using the following search terms/syntax:

(Attention Deficit Disorder with Hyperactivity[Mesh] OR adhd[tiab] OR hkd[tiab] OR addh[tiab] OR hyperkine*[tiab] OR attention deficit*[tiab] OR hyper-activ*[tiab] OR hyperactiv*[tiab] OR overactive[tiab] OR inattentive[tiab] OR impulsiv*[tiab]) AND (Amphetamines[Mesh] OR Bupropion[Mesh] OR Clonidine[Mesh] OR Methylphenidate[Mesh] OR Dexmethylphenidate[Mesh] OR Guanfacine[Mesh] OR Adderall[tiab] OR Amphetamine[tiab] OR Desoxyn*[tiab] OR Phenopromin[tiab] OR Amfetamine[tiab] OR Phenamine[tiab] OR Centramina[tiab] OR Fenamine[tiab] OR Levoamphetamine[tiab] OR Dexamfetamine[tiab] OR Dexamphetamine[tiab] OR Dexedrine[tiab] OR Dextroamphetamine[tiab] OR DextroStat[tiab] OR Oxydess[tiab] OR Methylamphetamine[tiab] OR Methylenedioxyamphetamine[tiab] OR Methamphetamine[tiab] OR Chloroamphetamine[tiab] OR Metamfetamine[tiab] OR Deoxyephedrine[tiab] OR Desoxyephedrine[tiab] OR Ecstasy[tiab] OR Atomoxetine[tiab] OR Biphentin[tiab] OR Bupropion[tiab] OR Amfebutamone[tiab] OR Zyntabac[tiab] OR Quomen[tiab] OR Wellbutrin[tiab] OR Zyban[tiab] OR Catapres*[tiab] OR Clonidine[tiab] OR Klofenil[tiab] OR Clofenil[tiab] OR Chlophazolin[tiab] OR Gemiton[tiab] OR Hemiton[tiab] OR Isoglaucon[tiab] OR Klofelin[tiab] OR Clopheline[tiab] OR Clofelin[tiab] OR Dixarit[tiab] OR Concerta[tiab] OR Daytrana[tiab] OR Methylphenidate[tiab] OR Equasym[tiab] OR Methylin[tiab] OR Tsentedrin[tiab] OR Centedrin[tiab] OR Phenidylate[tiab] OR Ritalin*[tiab] OR Duraclon[tiab] OR Elvanse[tiab] OR Focalin[tiab] OR Dexmethylphenidate[tiab] OR Guanfacine[tiab] OR Estulic[tiab] OR Tenex[tiab] OR Kapvay[tiab] OR Lisdexamfetamine[tiab] OR Vyvanse[tiab] OR Medikinet[tiab] OR Metadate[tiab] OR Modafinil[tiab] OR Nexiclon[tiab] OR Quillivant[tiab] OR Strattera[tiab] OR stimulant* [tiab] OR psychostimulant* [tiab] OR non stimulant* [tiab] OR non-stimulant* [tiab] OR non psychostimulant* OR non-psychostimulant* [tiab] OR alpha-2 agonist* [tiab] OR pharmacolog* [tiab] OR psychopharmacol* [tiab] OR pharmacother* [tiab] OR medication* [tiab] treatment* [tiab] OR management [tiab] OR intervention* [tiab]) AND (systematic review* OR systematic overview OR meta-analy* OR metaanaly* OR metanaly* OR meta-review OR umbrella review OR review of reviews)

The last search was run on April 7th, 2017.

List of the systematic reviews providing references that were hand-searched

1. No authors listed. Attention Deficit Hyperactivity Disorder in Children and Adolescents. Comparative Effectiveness Review Summary Guides for Clinicians. Rockville MD2007.

2. Aagaard L, Hansen EH. The occurrence of adverse drug reactions reported for attention deficit hyperactivity disorder (ADHD) medications in the pediatric population: a qualitative review of empirical studies. *Neuropsychiatr Dis Treat.* 2011;7:729-744
3. Arnold LE, Hodgkins P, Caci H, Kahle J, Young S. Effect of treatment modality on long-term outcomes in attention-deficit/hyperactivity disorder: a systematic review. *PLoS One*. 2015;10(2):e0116407.
4. Asherson P, Stes S, Nilsson Markhed M, et al. The effects of atomoxetine on emotional control in adults with ADHD: An integrated analysis of multicenter studies. *Eur Psychiatry.* 2015;30(4):511-520.
5. Banaschewski T, Coghill D, Santosh P, et al. Long-acting medications for the hyperkinetic disorders. A systematic review and European treatment guideline. *Eur Child Adolesc Psychiatry.* 2006;15(8):476-495.
6. Bangs ME, Tauscher-Wisniewski S, Polzer J, et al. Meta-analysis of suicide-related behavior events in patients treated with atomoxetine. *J Am Acad Child Adolesc Psychiatry*. 2008;47(2):209-218.
7. Bangs ME, Wietecha LA, Wang S, Buchanan AS, Kelsey DK. Meta-analysis of suicide-related behavior or ideation in child, adolescent, and adult patients treated with atomoxetine. *J Child Adolesc Psychopharmacol.* 2014;24(8):426-434.
8. Benkert D, Krause KH, Wasem J, Aidelsburger P. Effectiveness of pharmaceutical therapy of ADHD (Attention-Deficit/Hyperactivity Disorder) in adults - health technology assessment. *GMS health technology assessment. 2*010;6:Doc13.
9. Biederman J, Spencer TJ, Newcorn JH, et al. Effect of comorbid symptoms of oppositional defiant disorder on responses to atomoxetine in children with ADHD: a meta-analysis of controlled clinical trial data. *Psychopharmacology (Berl). 2*007;190(1):31-41.
10. Bloch MH, Panza KE, Landeros-Weisenberger A, Leckman JF. Meta-analysis: treatment of attention-deficit/hyperactivity disorder in children with comorbid tic disorders. *J Am Acad Child Adolesc Psychiatry.* 2009;48(9):884-893.
11. Brams M, Moon E, Pucci M, Lopez FA. Duration of effect of oral long-acting stimulant medications for ADHD throughout the day. *Curr Med Res Opin.* 2010;26(8):1809-182.
12. Bukstein OG, Head J. Guanfacine ER for the treatment of adolescent attention-deficit/hyperactivity disorder. *Expert Opin Pharmacother.* 2012;13(15):2207-2213.
13. Buitelaar JK, Wilens TE, Zhang S, Ning Y, Feldman PD. Comparison of symptomatic versus functional changes in children and adolescents with ADHD during randomized, double-blind treatment with psychostimulants, atomoxetine, or placebo. *J Child Psychol Psychiatry.* 2009;50(3):335-342.
14. Buoli M, Serati M, Cahn W. Alternative pharmacological strategies for adult ADHD treatment: a systematic review. *Expert Rev Neurother*. 2016;16(2):131-144.
15. Bushe CJ, Savill N. Atomoxetine in children and adolescents with attention-deficit/hyperactivity disorder. Systematic review of review papers 2009-2011. An update for clinicians*. J Cent Nerv Syst Dis*. 2011;3:209-217.
16. Bushe CJ, Savill NC. Suicide related events and attention deficit hyperactivity disorder treatments in children and adolescents: a meta-analysis of atomoxetine and methylphenidate comparator clinical trials. *Child Adolesc Psychiatry Ment Health*. 2013;7:19.
17. Bushe CJ, Savill NC. Systematic review of atomoxetine data in childhood and adolescent attention-deficit hyperactivity disorder 2009-2011: focus on clinical efficacy and safety. *J Psychopharmacol*. 2014;28(3):204-211.
18. Bushe C, Day K, Reed V, et al. A network meta-analysis of atomoxetine and osmotic release oral system methylphenidate in the treatment of attention-deficit/hyperactivity disorder in adult patients. *J Psychopharmacol*. 2016;30(5):444-458.
19. Caballero J, Nahata MC. Atomoxetine hydrochloride for the treatment of attention-deficit/hyperactivity disorder. *Clin Ther.* 2003;25(12):3065-3083.
20. Camporeale A, Day KA, Ruff D, Arsenault J, Williams D, Kelsey DK. Profile of sexual and genitourinary treatment-emergent adverse events associated with atomoxetine treatment: a pooled analysis. *Drugs Saf.* 2013;36(8):663-671.
21. Castells X, Ramos-Quiroga JA, Rigau D, et al. Efficacy of methylphenidate for adults with attention-deficit hyperactivity disorder: a meta-regression analysis. *CNS Drugs*. 2011;25(2):157-169.
22. Castells X, Ramos-Quiroga JA, Bosch R, Nogueira M, Casas M. Amphetamines for Attention Deficit Hyperactivity Disorder (ADHD) in adults. *Cochrane Database Syst Rev.* 2011(6):CD007813.
23. Castells X, Cunill R, Capella D. Treatment discontinuation with methylphenidate in adults with attention deficit hyperactivity disorder: a meta-analysis of randomized clinical trials. *Eur J Clin Pharmacol*. 2013;69(3):347-356.
24. Chan E, Fogler JM, Hammerness PG. Treatment of Attention-Deficit/Hyperactivity Disorder in Adolescents: A Systematic Review. *JAMA*. 2016;315(18):1997-2008.
25. Charach A, Dashti B, Carson P, et al. Attention Deficit Hyperactivity Disorder: Effectiveness of Treatment in At-Risk Preschoolers; Long-Term Effectiveness in All Ages; and Variability in Prevalence, Diagnosis, and Treatment. Rockville MD, 2011.

26. Cheng JY, Chen RY, Ko JS, Ng EM. Efficacy and safety of atomoxetine for attention-deficit/hyperactivity disorder in children and adolescents-meta-analysis and meta-regression analysis. *Psychopharmacology (Berl).* 2007;194(2):197-209.
27. Chierrito de OD, Guerrero de SP, Borges Dos RC et al. Safety of Treatments for ADHD in Adults: Pairwise and Network Meta-Analyses. *J Atten Disord*. 2017;1087054717696773.
28. Clavenna A, Bonati M. Safety of medicines used for ADHD in children: a review of published prospective clinical trials. *Arch Dis Child.* 2014;99(9):866-872.
29. Coghill D. The impact of medications on quality of life in attention-deficit hyperactivity disorder: a systematic review. *CNS Drugs*. 2010;24(10):843-866.
30. Coghill D, Banaschewski T, Zuddas A, Pelaz A, Gagliano A, Doepfner M. Long-acting methylphenidate formulations in the treatment of attention-deficit/hyperactivity disorder: a systematic review of head-to-head studies. *BMC Psychiatry*. 2013;13:237.
31. Coghill DR, Caballero B, Sorooshian S, Civil R. A systematic review of the safety of lisdexamfetamine dimesylate. *CNS Drugs*. 2014;28(6):497-511.
32. Coghill DR, Seth S, Pedroso S, Usala T, Currie J, Gagliano A. Effects of methylphenidate on cognitive functions in children and adolescents with attention-deficit/hyperactivity disorder: evidence from a systematic review and a meta-analysis. *Biol Psychiatry*. 2014;76(8):603-615.
33. Cohen SC, Mulqueen JM, Ferracioli-Oda E, et al. Meta-Analysis: Risk of Tics Associated With Psychostimulant Use in Randomized, Placebo-Controlled Trials. *J Am Acad Child Adolesc Psychiatry*. 2015;54(9):728-736.
34. Connor DF, Fletcher KE, Swanson JM. A meta-analysis of clonidine for symptoms of attention-deficit hyperactivity disorder. *J Am Acad Child Adolesc Psychiatry.* 1999;38(12):1551-1559.
35. Connor DF, Glatt SJ, Lopez ID, Jackson D, Melloni RH, Jr. Psychopharmacology and aggression. I: A meta-analysis of stimulant effects on overt/covert aggression-related behaviors in ADHD. *J Am Acad Child Adolesc Psychiatry.* 2002;41(3):253-261.
36. Connor DF, Arnsten AF, Pearson GS, Greco GF. Guanfacine extended release for the treatment of Attention-Deficit/Hyperactivity Disorder in children and adolescents. *Exp Opin Pharmacother*. 2014;15(11):1601-10.
37. Cortese S, D'Acunto G, Konofal E, Masi G, Vitiello B. New Formulations of Methylphenidate for the Treatment of Attention-Deficit/Hyperactivity Disorder: Pharmacokinetics, Efficacy, and Tolerability. *CNS Drugs.* 2017;31(2):149-160.
38. Coughlin CG, Cohen SC, Mulqueen JM, Ferracioli-Oda E, Stuckelman ZD, Bloch MH. Meta-Analysis: Reduced Risk of Anxiety with Psychostimulant Treatment in Children with Attention-Deficit/Hyperactivity Disorder. *J Child Adolesc Psychopharmacol*. 2015;25(8):611-617.
39. Cunill R, Castells X, Tobias A, Capella D. Atomoxetine for attention deficit hyperactivity disorder in the adulthood: a meta-analysis and meta-regression. *Pharmacoepidemiol Drug Saf.* 2013;22(9):961-969.
40. Cunill R, Castells X, Tobias A, Capella D. Pharmacological treatment of attention deficit hyperactivity disorder with co-morbid drug dependence. *J Psychopharmacol*. 2015;29(1):15-23.
41. Cunill R, Castells X, Tobias A, Capella D. Efficacy, safety and variability in pharmacotherapy for adults with attention deficit hyperactivity disorder: a meta-analysis and meta-regression in over 9000 patients. *Psychopharmacology (Berl).* 2016;233(2):187-197.
42. De Crescenzo F, Cortese S, Adamo N, Janiri L. Pharmacological and non-pharmacological treatment of adults with ADHD: a meta-review. *Evid Based Ment Health*. 2017;20(1):4-11.
43. Donnelly M, Haby MM, Carter R, Andrews G, Vos T. Cost-effectiveness of dexamphetamine and methylphenidate for the treatment of childhood attention deficit hyperactivity disorder. *Aust N Z J Psychiatry*. 2004;38(8):592-601.
44. Donnelly C, Bangs M, Trzepacz P, et al. Safety and Tolerability of Atomoxetine over 3 to 4 Years in Children with ADHD. *J Am Acad Child Adolesc Psychiatry.*2009 2012-12-14 2009;48(2):176-185.
45. Eiland LS, Guest AL. Atomoxetine treatment of attention-deficit/hyperactivity disorder. *Annals of Pharmacotherapy.* 2004;38(1):86-90.
46. Epstein T, Patsopoulos NA, Weiser M. Immediate-release methylphenidate for attention deficit hyperactivity disorder (ADHD) in adults. *Cochrane Database Syst Rev*. 2014;18;(9): (Note: This review was retracted)
47. Escobar R, Schacht A, Wehmeier PM, Wagner T. Quality of life and attention-deficit/hyperactivity disorder core symptoms: a pooled analysis of 5 non-US atomoxetine clinical trials. *J Clin Psychopharmacol.* 2010;30(2):145-151.
48. Faraone SV, Biederman J. Efficacy of Adderall for Attention-Deficit/Hyperactivity Disorder: a meta-analysis. Journal *J Atten Disord*. 2002;6(2):69-75.
49. Faraone SV, Biederman J, Roe C. Comparative efficacy of Adderall and methylphenidate in attention-deficit/hyperactivity disorder: a meta-analysis. *J Clin Psychopharmacol*. 2002;22(5):468-473.
50. Faraone SV, Spencer T, Aleardi M, Pagano C, Biederman J. Meta-analysis of the efficacy of methylphenidate for treating adult attention-deficit/hyperactivity disorder. *J Clin Psychopharmacol.* 2004;24(1):24-29.

51. Faraone SV, Biederman J, Spencer TJ, Aleardi M. Comparing the efficacy of medications for ADHD using meta-analysis. *MedGenMed*. 2006;8(4):4.
52. Faraone SV, Biederman J, Morley CP, Spencer TJ. Effect of stimulants on height and weight: a review of the literature. *J Am Acad Child Adolesc Psychiatry.* 2008;47(9):994-1009.
53. Faraone SV. Using Meta-analysis to Compare the Efficacy of Medications for Attention-Deficit/Hyperactivity Disorder in Youths. *P &T*. 2009;34(12):678-694.
54. Faraone SV, Buitelaar J. Comparing the efficacy of stimulants for ADHD in children and adolescents using meta-analysis. *Eur Child Adolesc Psychiatry*. 2010;19(4):353-364.
55. Faraone SV, Glatt SJ. Effects of extended-release guanfacine on ADHD symptoms and sedation-related adverse events in children with ADHD. *J Atten Disord.* 2010;13(5):532-538.
56. Faraone SV, Glatt SJ. A comparison of the efficacy of medications for adult attention-deficit/hyperactivity disorder using meta-analysis of effect sizes. *J Clin Psychiatry*. 2010;71(6):754-763.
57. Faraone SV. Understanding the effect size of lisdexamfetaminedimesylate for treating ADHD in children and adults. *J Atten Disord*. 2012;16(2):128-137.
58. Faraone SV, McBurnett K, Sallee FR, Steeber J, Lopez FA. Guanfacine extended release: A novel treatment for attention-deficit/ hyperactivity disorder in children and adolescents. *Clin Ther.* 2013;35(11):1778-1793.
59. Fredriksen M, Halmoy A, Faraone SV, Haavik J. Long-term efficacy and safety of treatment with stimulants and atomoxetine in adult ADHD: a review of controlled and naturalistic studies. *Eur Neuropsychopharmacol*. 2013;23(6):508-527.
60. Fridman M, Hodgkins PS, Kahle JS, Erder MH. Predicted effect size of lisdexamfetamine treatment of attention deficit/hyperactivity disorder (ADHD) in European adults: Estimates based on indirect analysis using a systematic review and meta-regression analysis. *Eur Psychiatry*. 2015;30(4):521-527.
61. Frolich J, Banaschewski T, Spanagel R, Dopfner M, Lehmkuhl G. [The medical treatment of attention deficit hyperactivity disorder (ADHD) with amphetamines in children and adolescents]. *Z Kinder Jugendpsychiatr Psychother*. 2012;40(5):287-299; quiz 299-300.
62. Garnock-Jones KP, Keating GM. Atomoxetine: a review of its use in attention-de cit hyperactivity disorder in children and adolescents. *Paediatr Drugs*. 2009;11(3):203–6.
63. Gayleard JL, Mychailyszyn MP. Atomoxetine treatment for children and adolescents with Attention-Deficit/Hyperactivity Disorder (ADHD): a comprehensive meta-analysis of outcomes on parent-rated core symptomatology. *Atten Defic Hyperact Disord*. 2017. doi: 10.1007/s12402-017-0216-y.
64. Ghanizadeh A. Atomoxetine for treating ADHD symptoms in autism: a systematic review. *J Atten Disord*. 2013;17(8):635-640.
65. Gibson AP, Bettinger TL, Patel NC, Crismon ML. Atomoxetine versus stimulants for treatment of attention deficit/hyperactivity disorder. *Ann Pharmacother*. 2006;40(6):1134-1142.
66. Gilmore A, Milne R. Methylphenidate in children with hyperactivity: review and cost-utility analysis. *Pharmacoepidemiol Drug Saf.* 2001;10(2):85-94.
67. Gobbo MA, Louza MR. Influence of stimulant and non-stimulant drug treatment on driving performance in patients with attention deficit hyperactivity disorder: a systematic review. *Eur Neuropsychopharmacol*. 2014;24(9):1425-1443.
68. Godfrey J. Safety of therapeutic methylphenidate in adults: a systematic review of the evidence. *J Psychopharmacol* (Oxford, England). 2009;23(2):194-205.
69. Gonzalez de Dios J, Cardo E, Servera M. [Methylphenidate in the treatment of attention-deficit/hyperactivity disorder: are we achieving an adequate clinical practice?]. *Rev Neurol*. 2006;43(12):705-714.
70. Greenhill LL, Newcorn JH, Gao H, Feldman PD. Effect of two different methods of initiating atomoxetine on the adverse event profile of atomoxetine. *J Am Acad Child Adolesc Psychiatry.* 2007;46(5):566-572.
71. Hanwella R, Senanayake M, de Silva V. Comparative efficacy and acceptability of methylphenidate and atomoxetine in treatment of attention deficit hyperactivity disorder in children and adolescents: a meta-analysis. *BMC Psychiatry*. 2011;11:176.
72. Hammerness P, McCarthy K, Mancuso E, Gendron C, Geller D. Atomoxetine for the treatment of attention de cit/hyperactivity disorder in children and adolescents-A review. *Neuropsychiat Dis Treat*. 2009;5:215–26.
73. Hazell PL, Kohn MR, Dickson R, Walton RJ, Granger RE, Wyk GW. Core ADHD symptom improvement with atomoxetine versus methylphenidate: a direct comparison meta-analysis. *J Atten Disord*. 2011;15(8):674-683.
74. Hennissen L, Bakker MJ, Banaschewski T et al. Cardiovascular Effects of Stimulant and Non-Stimulant Medication for Children and Adolescents with ADHD: A Systematic Review and Meta-Analysis of Trials of Methylphenidate, Amphetamines and Atomoxetine. *CNS Drugs*. 2017;31(3):199-215.
75. Higgins ES. A comparative analysis of antidepressants and stimulants for the treatment of adults with attention-deficit hyperactivity disorder. *J Fam Pract.* 1999;48(1):15-20.
76. Hirota T, Schwartz S, Correll CU. Alpha-2 agonists for attention-deficit/hyperactivity disorder in youth: a systematic review and meta-analysis of monotherapy and add-on trials to stimulant therapy. *J Am Acad Child Adolesc Psychiatry*. 2014;53(2):153-173.

77. Hodgkins P, Shaw M, McCarthy S, Sallee FR. The pharmacology and clinical outcomes of amphetamines to treat ADHD: does composition matter? *CNS Drugs*. 2012;26(3):245-268.
78. Hodgkins P, Shaw M, Coghill D, Hechtman L. Amfetamine and methylphenidate medications for attention-deficit/hyperactivity disorder: complementary treatment options. *Eur Child Adolesc Psychiatry.* 2012;21(9):477-492.
79. Hutchison SL, Ghuman JK, Ghuman HS, Karpov I, Schuster JM. Efficacy of atomoxetine in the treatment of attention-deficit hyperactivity disorder in patients with common comorbidities in children, adolescents and adults: a review. *Ther Adv Psychopharmacol.* 2016;6(5):317-334.
80. Jadad AR, Booker L, Gauld M, et al. The treatment of attention-deficit hyperactivity disorder: an annotated bibliography and critical appraisal of published systematic reviews and metaanalyses. *Can J Psychiatry*. 1999;44(10):1025-1035.
81. Jadad AR, Boyle M, Cunningham C, Kim M, Schachar R. Treatment of attention-deficit/hyperactivity disorder. *Evid Rep Technol Assess (Summary*). 1999(11):i-viii, 1-341.
82. Jain R, Katic A. Current and Investigational Medication Delivery Systems for Treating Attention-Deficit/Hyperactivity Disorder. *Prim Care Companion CNS Disord.* 2016;18:18.
83. Joseph A, Ayyagari R, Xie M et al. Comparative efficacy and safety of attention-deficit/hyperactivity disorder pharmacotherapies, including guanfacine extended release: a mixed treatment comparison. *Eur Child Adolesc Psychiatry*. 2017;26(8):875-897.
84. Kidwell KM, Van Dyk TR, Lundahl A, Nelson TD. Stimulant Medications and Sleep for Youth With ADHD: A Meta-analysis. *Pediatrics*. 2015;136(6):1144-1153.
85. King S, Griffin S, Hodges Z, et al. A systematic review and economic model of the effectiveness and cost-effectiveness of methylphenidate, dexamfetamine and atomoxetine for the treatment of attention deficit hyperactivity disorder in children and adolescents. *Health Technol Assess (Winchester, England).* 2006;10(23):iii-iv, xiii-146.
86. Klassen A, Miller A, Raina P, Lee SK, Olsen L. Attention-deficit hyperactivity disorder in children and youth: a quantitative systematic review of the efficacy of different management strategies. *Can J Psychiatry*. 1999;44(10):1007-1016.
87. Koesters M, Becker T, Kilian R, Fegert JM, Weinmann S. Limits of meta-analysis: methylphenidate in the treatment of adult attention-deficit hyperactivity disorder. *J Psychopharmacol*. 2009;23(7):733-744.
88. Kratochvil CJ, Wilens TE, Greenhill LL, et al. Effects of long-term atomoxetine treatment for young children with attention-deficit/hyperactivity disorder. *J Am Acad Child Adolesc Psychiatry*. 2006;45(8):919-927.
89. Kratochvil CJ, Milton DR, Vaughan BS, Greenhill LL. Acute atomoxetine treatment of younger and older children with ADHD: a meta-analysis of tolerability and efficacy. *Child Adolesc Psychiatry Ment Health*. 2008;2(1):25.
90. Lindsay SE, Gudelsky GA, Heaton PC. Use of modafinil for the treatment of attention deficit/hyperactivity disorder. *Ann Pharmacother.* 2006;40(10):1829-1833.
91. Li Y, Gao J, He S, Zhang Y, Wang Q. An Evaluation on the Efficacy and Safety of Treatments for Attention Deficit Hyperactivity Disorder in Children and Adolescents: a Comparison of Multiple Treatments. *Mol Neurobiol*. 2016. DOI: 10.1007/s12035-016-0179-6
92. Linderkamp F, Lauth G. Efficacy of pharmacological versus psychotherapeutic therapies in adults with attention deficit/hyperactivity disorder (ADHD): An empirical meta-analysis. *Verhaltenstherapie*. 2011;21:229–38.
93. Lv XZ, Shu Z, Zhang YW, Wu SS, Zhan SY. [Effectiveness and safety of methylphenidate and atomoxetine for attention deficit hyperactivity disorder: a systematic review]. *Zhongguo Dang Dai Er Ke Za Zhi = Chinese journal of contemporary pediatrics*. 2011;13(5):365-369.
94. Lv XZ, Shu Z, Zhang YW, Wu SS, Zhan SY. Effectiveness and safety of methylphenidate and atomoxetine for attention deficit hyperactivity disorder: a systematic review. *Transl Pediatr*. 2012;1(1):47-53.
95. Maia CR, Cortese S, Caye A, et al. Long-Term Efficacy of Methylphenidate Immediate-Release for the Treatment of Childhood ADHD: A Systematic Review and Meta-Analysis. *J Atten Disord*. 2017;21(1):3-13.
96. Maneeton N, Maneeton B, Srisurapanont M, Martin SD. Bupropion for adults with attention-deficit hyperactivity disorder: meta-analysis of randomized, placebo-controlled trials. *Psychiatry Clin Neurosci*. 2011;65(7):611-617.
97. Maneeton N, Maneeton B, Intaprasert S, Woottiluk P. A systematic review of randomized controlled trials of bupropion versus methylphenidate in the treatment of attention-deficit/hyperactivity disorder. *Neuropsychiatr Dis Treat*. 2014;10:1439-1449.
98. Maneeton N, Maneeton B, Suttajit S, Reungyos J, Srisurapanont M, Martin SD. Exploratory meta-analysis on lisdexamfetamine versus placebo in adult ADHD. *Drug Des Devel Ther*. 2014;8:1685-1693.
99. Maneeton N, Maneeton B, Woottiluk P, et al. Comparative efficacy, acceptability, and tolerability of dexmethylphenidate versus placebo in child and adolescent ADHD: a meta-analysis of randomized controlled trials. *Neuropsychiatr Dis Treat*. 2015;11:2943-2952.

100. Maneeton B, Maneeton N, Likhitsathian S, et al. Comparative efficacy, acceptability, and tolerability of lisdexamfetamine in child and adolescent ADHD: a meta-analysis of randomized, controlled trials. *Drug Des Devel Ther*. 2015;9:1927-1936.
101. McDonagh MS, Peterson K, Thakurta S, Low A. Drug Class Review: Pharmacologic Treatments for Attention Deficit Hyperactivity Disorder: Final Update 4 Report. Portland OR: 2011 by Oregon Health & Science University, 2011.
102. Meszaros A, Czobor P, Balint S, Simon V, Bitter I. [Pharmacotherapy of adult Attention Deficit/Hyperactivity Disorder (ADHD): a systematic review]. Psychiatria Hungarica: *A Magyar Pszichiatriai Tarsasag tudomanyos folyoirata*. 2007;22(4):259-270.
103. Meszaros A, Czobor P, Balint S, Komlosi S, Simon V, Bitter I. Pharmacotherapy of adult attention deficit hyperactivity disorder (ADHD): a meta-analysis. *Int J Neuropsychopharmacol*. 2009;12(8):1137-1147.
104. Michelson D, Read HA, Ruff DD, Witcher J, Zhang S, McCracken J. CYP2D60 and Clinical Response to Atomoxetine in Children and Adolescents with ADHD. *J Am Acad Child Adolesc Psychiatry.* 2007;46(2):242-251.
105. Mick E, McManus DD, Goldberg RJ. Meta-analysis of increased heart rate and blood pressure associated with CNS stimulant treatment of ADHD in adults. *Eur Neuropsychopharmacol*. 2013;23(6):534-541.
106. Ming X, Mulvey M, Mohanty S, Patel V. Safety and efficacy of clonidine and clonidine extended-release in the treatment of children and adolescents with attention deficit and hyperactivity disorders. *Adolesc Health Med Ther.* 2011;2:105-112.
107. Moriyama TS, Polanczyk GV, Terzi FS, Faria KM, Rohde LA. Psychopharmacology and psychotherapy for the treatment of adults with ADHD-a systematic review of available meta-analyses. *CNS Spectr*. 2013;18(6):296-306.
108. Mosholder AD, Gelperin K, Hammad TA, Phelan K, Johann-Liang R. Hallucinations and other psychotic symptoms associated with the use of attention-deficit/ hyperactivity disorder drugs in children. *Pediatrics.* 2009;123(2):611-616.
109. Najib J. The efficacy and safety profile of lisdexamfetamine dimesylate, a prodrug of d-amphetamine, for the treatment of attention-deficit/hyperactivity disorder in children and adults. *Clin Ther.* 2009;31(1):142-176.
110. Najib, J. Lisdexamfetamine in the treatment of adolescents and children with attention-deficit/hyperactivity disorder. *Adolesc Health Med Ther* 2012; 3: 51-66.
111. Ng QX. A Systematic Review of the Use of Bupropion for Attention-Deficit/Hyperactivity Disorder in Children and Adolescents. *J Child Adolesc Psychopharmacol*. 2017;27(2):112-16.
112. Ottenbacher K J, Cooper HM. Drug treatment of hyperactivity in children. *Dev Med Child Neurology*. 1983;25:358-66
113. Parker J, Wales G, Chalhoub N, Harpin V. The long-term outcomes of interventions for the management of attention-deficit hyperactivity disorder in children and adolescents: a systematic review of randomized controlled trials. *Psychol Res Behav Manag*. 2013;6:87-99.
114. Peterson K, McDonagh MS, Fu R. Comparative benefits and harms of competing medications for adults with attention-deficit hyperactivity disorder: a systematic review and indirect comparison meta-analysis. *Psychopharmacology*. 2008;197(1):1-11.
115. Polzer J, Bangs ME, Zhang S, et al. Meta-analysis of aggression or hostility events in randomized, controlled clinical trials of atomoxetine for ADHD. *Biol Psychiatry*. 2007;61(5):713-719.
116. Prasad V, Brogan E, Mulvaney C, Grainge M, Stanton W, Sayal K. How effective are drug treatments for children with ADHD at improving on-task behaviour and academic achievement in the school classroom? A systematic review and meta-analysis. *Eur Child Adolesc Psychiatry*. 2013;22(4):203-216.
117. Pringsheim T, Steeves T. Pharmacological treatment for Attention Deficit Hyperactivity Disorder (ADHD) in children with comorbid tic disorders. *Cochrane Database Syst Rev*. 2011(4):CD007990.
118. Pringsheim T, Hirsch L, Gardner D, Gorman DA. The pharmacological management of oppositional behaviour, conduct problems, and aggression in children and adolescents with attention-deficit hyperactivity disorder, oppositional defiant disorder, and conduct disorder: a systematic review and meta-analysis. Part 1: psychostimulants, alpha-2 agonists, and atomoxetine. *Can J Psychiatry*. 2015;60(2):42-51.
119. Punja S, Zorzela L, Hartling L, Urichuk L, Vohra S. Long-acting versus short-acting methylphenidate for paediatric ADHD: a systematic review and meta-analysis of comparative efficacy. *BMJ Open*. 2013;3(3).
120. Punja S, Shamseer L, Hartling L, et al. Amphetamines for attention deficit hyperactivity disorder (ADHD) in children and adolescents. *Cochrane Database Syst Rev*. 2016;2:CD009996.
121. Rajeh A, Amanullah S, Shivakumar K, Cole J. Interventions in ADHD: A comparative review of stimulant medications and behavioral therapies. *Asian J Psychiatr*. 2017;25:131-135.
122. Ravishankar V, Chowdappa SV, Benegal V, Muralidharan K. The efficacy of atomoxetine in treating adult attention deficit hyperactivity disorder (ADHD): A meta-analysis of controlled trials. *Asian J Psychiatr*. 2016;24:53-58.

123. Reichow B, Volkmar FR, Bloch MH. Systematic review and meta-analysis of pharmacological treatment of the symptoms of attention-deficit/hyperactivity disorder in children with pervasive developmental disorders. *J Autism Dev Disord*. 2013;43(10):2435-2441.
124. Rezaei G, Hosseini SA, Akbari Sari A, et al. Comparative efficacy of methylphenidate and atomoxetine in the treatment of attention deficit hyperactivity disorder in children and adolescents: A systematic review and meta-analysis. *Med J Islam Repub Iran*. 2016;30:325.
125. Roskell NS, Setyawan J, Zimovetz EA, Hodgkins P. Systematic evidence synthesis of treatments for ADHD in children and adolescents: indirect treatment comparisons of lisdexamfetamine with methylphenidate and atomoxetine. *Curr Med Res Opin*. 2014;30(8):1673-1685.
126. Ruggiero S, Clavenna A, Reale L, Capuano A, Rossi F, Bonati M. Guanfacine for attention deficit and hyperactivity disorder in pediatrics: a systematic review and meta-analysis. *Eur Neuropsycholopharmacol*. 2014;24(10):1578-1590.
127. Sallee F, Connor DF, Newcorn JH. A review of the rationale and clinical utilization of alpha2-adrenoceptor agonists for the treatment of attention-deficit/hyperactivity and related disorders. *J Child Adolesc Psychopharmacol*. 2013;23(5):308-319.
128. Schachar R, Jadad AR, Gauld M, et al. Attention-deficit hyperactivity disorder: critical appraisal of extended treatment studies. *Can J Psychiatry*. 2002;47(4):337-348.
129. Schachter HM, Pham B, King J, Langford S, Moher D. How efficacious and safe is short-acting methylphenidate for the treatment of attention-deficit disorder in children and adolescents? A meta-analysis. *CMAJ*. 2001;165(11):1475-1488.
130. Schwartz S, Correll CU. Efficacy and safety of atomoxetine in children and adolescents with attention-deficit/hyperactivity disorder: results from a comprehensive meta-analysis and metaregression. *J Am Acad Child Adolesc Psychiatry*. 2014;53(2):174-187.
131. Segenreich D, Mattos P. Bupropion efficacy in the treatment of ADHD. A systematic review and critical analysis of evidences. [Portuguese]. Eficacia da bupropiona no tratamento do TDAH. Uma revisao sistematica e analise critica de evidencias. *Rev Psiquiatr Clin.* 2004;31(3):117-123.
132. Sevecke K, Dopfner M, Lehmkuhl G. The effectiveness of stimulants of retard forms in children and adolescents with ADHD - a systematic overview. *Z Kinder Jugendpsychiatr Psychother.* 2004;32(4):265-278
133. Sibley MH, Kuriyan AB, Evans SW, Waxmonsky JG, Smith BH. Pharmacological and psychosocial treatments for adolescents with ADHD: an updated systematic review of the literature. *Clin Psychol Rev*. 2014;34(3):218-232.
134. Sopko MA, Jr., Caberwal H, Chavez B. The safety and efficacy of methylphenidate and dexmethylphenidate in adults with attention deficit/hyperactivity disorder. *J Cent Nerv Syst Dis.* 2010;2:15-30.
135. Storebo OJ, Krogh HB, Ramstad E, et al. Methylphenidate for attention-deficit/hyperactivity disorder in children and adolescents: Cochrane systematic review with meta-analyses and trial sequential analyses of randomised clinical trials. *BMJ* 2015;351:h5203.
136. Storebo OJ, Ramstad E, Krogh HB, et al. Methylphenidate for children and adolescents with attention deficit hyperactivity disorder (ADHD). *Cochrane Database Syst Rev*. 2015(11):CD009885.
137. Stuhec M, Munda B, Svab V, Locatelli I. Comparative efficacy and acceptability of atomoxetine, lisdexamfetamine, bupropion and methylphenidate in treatment of attention deficit hyperactivity disorder in children and adolescents: a meta-analysis with focus on bupropion. *J Affect Disord*. 2015;178:149-159. (Related to Locatelli I, --Venisnik K. A network meta-analysis of atomoxetine, methylphenidate, lisdexamfetamine, and bupropion for the treatment of attention deficit hyperactivity disorder in children and adolescents. *Value Health.* 2016;19 (7):A521-A522.)
138. Tamminga HG, Reneman L, Huizenga HM, Geurts HM. Effects of methylphenidate on executive functioning in attention-deficit/hyperactivity disorder across the lifespan: a meta-regression analysis. *Psychol Med*. 2016;46(9):1791-1807.
139. Tanaka Y, Rohde LA, Jin L, Feldman PD, Upadhyaya HP. A meta-analysis of the consistency of atomoxetine treatment effects in pediatric patients with attention-deficit/hyperactivity disorder from 15 clinical trials across four geographic regions. *J Child Adolesc Psychopharmacol*. 2013;23(4):262-270.
140. Thomson A, Maltezos S, Paliokosta E, Xenitidis K. Amfetamine for attention deficit hyperactivity disorder in people with intellectual disabilities. *Cochrane Database Syst Rev*. 2009(1):CD007009.
141. Thurber S, Walker CE. Medication and hyperactivity: a meta-analysis. *J Gen Psychol.* 1983;108{1st Half):79-86.
142. Turner D. A review of the use of modafinil for attention-deficit hyperactivity disorder. *Expert Rev Neurother.* 2006;6(4):455-468.
143. van de Loo-Neus GH, Rommelse N, Buitelaar JK. To stop or not to stop? How long should medication treatment of attention-deficit hyperactivity disorder be extended? *Eur Neuropsychopharmacol*. 2011;21(8):584-599.

144. Van der Oord S, Prins PJ, Oosterlaan J, Emmelkamp PM. Efficacy of methylphenidate, psychosocial treatments and their combination in school-aged children with ADHD: a meta-analysis. *Clin Psychol Rev*. 2008;28(5):783-800.
145. van Wyk GW, Hazell PL, Kohn MR, Granger RE, Walton RJ. How oppositionality, inattention, and hyperactivity affect response to atomoxetine versus methylphenidate: a pooled meta-analysis. *J Atten Disord*. 2012;16(4):314-324.
146. Verbeeck W, Tuinier S, Bekkering GE. Antidepressants in the treatment of adult attention-deficit hyperactivity disorder: a systematic review. *Adv Ther.* 2009; 26(2): 170-184
147. Wang SM, Han C, Lee SJ, et al. Modafinil for the treatment of attention-deficit/hyperactivity disorder: A meta-analysis. *J Psychiatr Res*. 2017;84:292-300.
148. Wehmeier PM, Schacht A, Escobar R, Savill N, Harpin V. Differences between children and adolescents in treatment response to atomoxetine and the correlation between health-related quality of life and Attention Deficit/Hyperactivity Disorder core symptoms: Meta-analysis of five atomoxetine trials. *Child Adolesc Psychiatry Ment Health*. 2010;4:30.
149. Weisler RH, Childress AC. Treating attention-deficit/hyperactivity disorder in adults: focus on once-daily medications. *Prim Care Companion CNS Disord*. 2011;13(6).
150. Wernicke JF, Faries D, Breitung R, Girod D. QT correction methods in children and adolescents. *J Cardiovasc Electrophysiol.* 2005;16(1):76-81
151. Weyandt LL, Oster DR, Marraccini ME, et al. Pharmacological interventions for adolescents and adults with ADHD: stimulant and nonstimulant medications and misuse of prescription stimulants. *Psychol Res Behav Manag*. 2014;7:223–49.
152. Wilens TE, Biederman J, Spencer TJ, Prince J. Pharmacotherapy of adult attention deficit/hyperactivity disorder: a review. *J Clin Psychopharmacol*. 1995;15(4):270-279.
153. Wilens TE, Spencer TJ, Biederman J. A review of the pharmacotherapy of adults with attention-deficit/hyperactivity disorder. *J Atten Disord*. 2002;5(4):189-202.
154. Wilens TE, Kratochvil C, Newcorn JH, Gao H. Do children and adolescents with ADHD respond differently to atomoxetine? *J Am Acad Child Adolesc Psychiatry.* 2006;45(2):149-157.
155. Wilens TE, Newcorn JH, Kratochvil CJ, et al. Long-term atomoxetine treatment in adolescents with attention-deficit/hyperactivity disorder. *J Pediatr*. 2006;149(1):112-119.
156. Wietecha LA, Ruff DD, Allen AJ, Greenhill LL, Newcorn JH. Atomoxetine tolerability in pediatric and adult patients receiving different dosing strategies. *J Clin Psychiatry.* 2013;74(12):1217-1223.

In additon, we scanned the references included in and excluded from the 2008 NICE Guidelienes on ADHD (https://www.nice.org.uk/guidance/cg72/evidence)

Websites of drug manufacturers searched to find additional relevant reports:

Amphetamines
Adderall - Shire
https://www.shire.com/

Atomoxetine
Strattera - Eli Lilly
http://www.lilly.co.uk/en/index.aspx

Bupropion
Bupropion - GSK:
https://www.gsk.com/

Clonidine
Clonicel - Shionogi
http://www.shionogi.com/

Dexmethylphenidate
Focalin/XR - Novartis
https://www.novartis.com

Guanfacine
Intuniv - Promius pharma/Shire
http://promiuspharma.com/

https://www.shire.com/

Lisdexamfetamine
Vyvanse - Shire Development
https://www.shire.com/

Methylphenidate
Aptensio - Rhodes Pharms
http://www.rhodespharma.com
Concerta - Janssen Pharms
http://www.janssen.com
Daytrana - Noven Pharms
http://www.noven.com
Focalin/XR - Novartis
https://www.novartis.com
Metadate CD - UCB Inc
https://www.ucb.com/
Quivillant - Nextwave Pharms (owned by Pfizer)
http://www.pfizer.com/
Ritalin - Novartis: https://www.novartis.com

Modafinil
Provigil - Cephalon (currently owned by Reva Pharmaceuticals)
http://www.tevausa.com/

Drug manufactures contacted to gather additional data/information and query about any additional study not retrieved in our search:

1. Abbott
2. Arbor Pharmaceuticals
3. Benevolent Co
4. Celgene
5. Cephalon
6. Concordia Pharmaceuticals Inc. (including Shionogi Inc./Addrenex Pharmaceuticals)
7. Glaxo
8. Highland/Ironshore
9. Janssen (Cilag), including Ortho-McNeil
10. Lilly and Co.
11. Medice
12. Novartis Pharmaceuticals
13. Noven Therapeutics
14. Orient Pharma Co., Ltd
15. Pfizer
16. Rhodes Pharmaceuticals, L.P and Purdue (affiliated)
17. Shire Pharmaceuticals (including UCB Pharma, Celltech and new Rive, acquired by Shire)
18. Teva

Appendix 2. Additional details on selection criteria

Types of participants

Additional note on inclusion criteria

Studies that included either in-or outpatients were eligible.

Exclusion criteria

The following were excluded: 1) studies using DSM-II criteria for ADHD, since these were not standardized; 2) studies recruiting patients with a diagnosis of Minimal Brain Dysfunction, which is not comparable with DSM definitions of ADHD or ICD definitions of HKD; 3) trials in which ADHD was a comorbid disorder secondary to a genetic syndrome; 4) studies enrolling subjects defined as "hyperkinetic" or "hyperactive" without application of standardised diagnostic criteria; 5) studies recruiting patients who were taking ADHD medications prior to entering the study, unless participants completed an appropriate wash out period before starting the study trial (see Appendix Table 5 in this Supplement for the details about recommended wash out periods for each individual drug); 6) studies where (a) all subjects had previously responded (according to the definition provided in the study) to the same medication tested in the randomized phase (irrespective of washout period) or (b) where all subjects were responders or stabilized/optimized to an ADHD medication (where "stabilized" or "optimized" means "responders") during a run-in/open label phase before of randomization (irrespective of wash out period); if the meaning of "stabilized" was not clear from the text of the study, we contacted study authors to query if "stabilized" or "optimized" meant "responders"; 7) studies in which all included subjects were deemed to be "resistant" to a previous ADHD drug, as all these situations would violate the transitivity assumption of NMA [10]; 8) trials in which all participants had a comoribid disorder pharmacologically treated with a medication other than an ADHD drug. 9) Data from the withdrawal phase of a trial were not included.

Types of interventions

Additional details

Studies where drugs were delivered in the form of tablets, capsules, chewable compounds or liquid formulations were eligible. Both fixed-dose and flexible-dose designs were allowed. Studies assessing the efficacy of multimodal treatments including the combination of ADHD drug(s) plus psychotherapy (for ADHD or other disorders/conditions) were excluded. However, studies in which ADHD drugs of interest for the present meta-analysis were combined with psychoeducation only, rather than psychotherapy, were retained. Study arms with medication only as monotherapy were included from studies testing non-pharmacological interventions if compared to another medication only or placebo arm from the same study. Studies comparing any ADHD drug to treatment as usual or assessing the efficacy of additional drugs in participants resistant to the first ADHD drug were not included. Studies using a single dose of drug were also excluded. As for the minimum duration of the pharmacological treatment, while previous meta-analyses have included studies with treatment duration of 1 day [e.g., [11]] and other meta-analyses have excluded studies lasting less than 3 weeks [e.g.,[12]], we included trials with treatment duration of at least 7 consecutive days, since response to adequate doses of psychostimulants can be appreciated after approximately 1 week of treatment and, to our knowledge, there is no clear evidence that placebo effects change over time in studies of ADHD drugs.

Types of studies

Additional details on inclusion criteria

For cross-over studies, to address concerns around possible "carry over" effects[13], we used data from the pre-crossover phase, whenever this was reported in the paper. When data for the pre-cross over phase were not reported, we contacted study authors to gather them. If pre-crossover data were not reported and not available upon request, we used data at the endpoint (after crossing over), derived from appropriate statistical methods (i.e., paired t-test) [14], only if there was an appropriate washout period (see Appendix Table 5 in this Supplement) between the two phases (pre and post crossover) of the trial. As for cluster trials, according to the Cochrane Handbook, they can be combined with individually randomised RCTs [15]. In this case, we planned to perform approximately correct analyses by dividing the binary data (the number of participants and the number experiencing the event) as presented in a report by a 'design effect'[15]. This is calculated by using the mean number of participants per cluster (m) and the intra-class correlation coefficient (ICC) (Design effect = 1 + (m - 1) * ICC) [15]. We planned to estimate the ICC will be by using the between-cluster variance component and the within-cluster variance component of the study [16]. However, no cluster trials were found to be included in the present meta-analysis.

Exclusion criteria

Quasi-randomized controlled trials, in which treatment assignment is decided through methods such as alternate days of the week, or studies using Latin square approach without adequate randomization were excluded. Open-label or

single blind RCTs, long-term studies using a maintenance design, and N-of-1 trials were also excluded.

Types of outcome measures

Additional details on primary outcomes: Where there were ratings of ADHD symptoms severity based on two or more scales, only one scale was selected among the following ones, in the following order of preference: ADHD Rating Scale (*total score*), SNAP ADHD *(total score*), Conners rating scale (any version, *ADHD total score*), or other ADHD scales. Total scores for ADHD symptoms were selected and evaluated. When total scores were not available and only sub-scales of ADHD measures (i.e., measuring the dimensions of inattention and hyperactivity/impulsivity symptoms of ADHD separately) were reported, the effect size for each of these was calculated separately and aggregated to estimate the overall effect. If only scores from a subscale measuring one ADHD dimension (i.e., inattention or hyperactivity/impulsivity) were available, we used those scores for the analyses. When endpoint scores were not reported but change scores were, we used the latter scores.[17] We conducted separate analyses for measures rated by 1) clinicians, 2) parents, 3) teachers, and 4) patients (self). Teachers and (after an amendment to the original protocol) clinicians' scores were considered for the primary analysis of studies in children/adolescents. Clinicians' scores were considered for the primary analysis of studies in adults.
Scales/subscales considered for inclusion are reported in Appendix Tables1-2 in this Supplement.

Additional note on secondary outcomes

- As for the Clinical Global Impressions-Improvement (CGI-I, investigator's rating), the proportion of participants who improved at endpoint based on the final CGI-I score of 1-2 was considered;
- Acceptability of treatment was defined as the proportion of patients who left the study early for any reason during the first 12 weeks of treatment, consistent with Cipriani et al. [18]

The outcomes were chosen as reflecting the most relevant ones from a clinical standpoint, following a consensus among the European ADHD Guidelines Group (EAGG) members.

Appendix 3. Additional details on study selection, data extraction and risk of bias/quality assessment

Selection of studies

Study selection was conducted independently by three investigators (NA, SCa, SC). Discrepancies were resolved by a third reviewer (AC) and, if needed, by other members of the review team (ES, DC, TB, AZ). Papers in non-English language were translated. Data were extracted independently by three researchers (CM-J, AH, LT), and double-checked by three other investigators independently (AC, NA, SC).

Studies identified through electronic and manual searches were listed with citation, titles and abstracts, in Endnote; duplicates were excluded using the Endnote function “remove duplicates”. The eligibility for inclusion process was conducted in two separate stages:

1. Two investigators (NA and SCa) independently screened title and abstracts of all non-duplicated papers and excluded those clearly not pertinent. A final list was agreed with discrepancies resolved by consensus between the two authors. When consensus was not reached, a third senior author (SC) acted as arbitrator. If any doubt about inclusion existed, the article proceeded to the next stage;
2. The full-text version of the articles passing stage 1 screening was downloaded and assessed for eligibility by two authors (NA and SCa), independently. Discrepancies were resolved by consensus between the two authors with arbitration by a third senior (SC) and, if needed, by a panel of five senior investigators (AC, ES, DC, TB, AZ). Data from multiple reports of the same study were linked together. Where required, we contacted the corresponding author or drug manufacturer to inquire on study eligibility.

For each individual study, sources of information/data were any (one or more) of the following:

- Journal article
- Information/data from ClinicalTrials.gov or other trial registries (see Appendix 2)
- Material retrieved on the Food and Drug Administration (FDA) website
- Information/data from the short Clinical Study Report (CSR) available on the drug manufacturer’s website
- Information/data from the full CSR, retrieved upon request to the drug manufacturer or via https://www.clinicalstudydatarequest.com/
- Unpublished information/data provided by the study author(s)
- Unpublished information/data provided by the drug manufacturer

For each retained study, the following data were collected:

- Study citation, year(s) of study, year of publication, location, setting, number of centres, design (type of RCT), sample size, diagnostic criteria, funding/sponsor (industry or academic);
- Characteristics of study participants, including: gender distribution, mean and range of age, presence and type of co-morbid (neuro)psychiatric conditions, mean (and SD) IQ, number randomized into each group, and number of dropouts, and whether ADHD medications naïve at baseline or previously exposed to other ADHD medications;
- Characteristics of interventions including mean and maximum doses, formulation, add-on interventions (if any), and whether forced dose or optimised treatment;
- Time(s) of outcome measurement;
- Outcome measures reported including whether the data were based on an intention-to-treat (ITT) or completers only sample. For ITT samples, methods of imputation were noted.

Quality assessment-risk of bias

Risk of bias was assessed by three investigators (CM-J, AH, LT) using the Cochrane risk of bias tool, and double checked by two review authors (SC and CH).

Risk of bias was assessed for each included study using the Cochrane Collaboration 'risk of bias' tool, as a reference [15]. As in Cipriani et al. [19], the original Cochrane Collaboration 'risk of bias' tool was slightly modified, to include the following domains:

1. Sequence generation: was the allocation sequence adequately generated?
2. Allocation concealment: was allocation adequately concealed?
3. Blinding of participants/parents, therapist and outcome assessors for each main outcome: was knowledge of the allocated treatment adequately prevented during the study?
4. Incomplete outcome data for the primary outcomes: were incomplete outcome data adequately addressed?

5. Selective outcome reporting: are reports of the study free from suggestion of selective outcome reporting?

As can be noted, the item “blinding of therapist” was added to the original Cochrane risk of bias to provide additional information since in trials of medications, therapist and assessor may not be the same person.

A description of what was reported to have happened in each study was provided, and a judgment on the risk of bias was made for each domain, based on the following three categories: “high risk of bias”, “low risk of bias” and “unclear risk of bias”. The potential bias for “industry sponsorship” was assessed as a separate item. As in Catala-Lopez et al. [12], the overall rating of risk of bias for each study was the lowest rating for any of the criteria (e.g., if any domain was scored *high* risk of bias, the study was considered at *high* risk of bias; if all items were scored *low* risk of bias, the study was considered at overall *low* risk). Where necessary, the authors of the studies or drug manufacturers were contacted for further information.

Appendix 4. Additional details on the statistical analysis

Synthesis of results

The analyses were performed using STATA v.14. (MRC Biostatistics Unit, Cambridge, UK, http://cmimg.cochrane.org/network-meta-analysis-toolkit); the codes and description of the methodology are available at http://www.mtm.uoi.gr/index.php/stata-routines-for-network-meta-analysis [20-22].

Dealing with missing data

Missing dichotomous outcome data were managed according to the ITT principle, and it was assumed that participants in the full analysis set who dropped out after randomization had a negative outcome. Missing continuous outcome data were analyzed using last observation carried forward to the final assessment (LOCF) if LOCF data were reported by the trial authors; if LOCF (or other imputation method) data were not available, missing data were analyzed using a validated method. Published SD, where available, were used. If SD were not available from the publication, SD were calculated from p-values, t-values, confidence intervals or standard errors [23]. If these values were missing, attempts were made to obtain SD or p-values, t-values, confidence intervals or standard errors from trial authors. Where SDs were not available, a validated method for imputation was used [24]. We checked that the original SDs were normally distributed, so that the imputed SD represented the average. Where imputation was employed, data were interpreted with caution, and the degree of heterogeneity observed was taken into account when interpreting findings. A sensitivity analysis was also conducted to examine the effect of imputation of the findings.

Assessment of clinical and methodological heterogeneity within treatment comparisons

The studies synthesized in each pairwise comparison need to be similar enough in terms of patient characteristics, setting, and outcome definitions, among others, in order to obtain interpretable and useful results [15]. To evaluate the degree of clinical and methodological heterogeneity, we generated descriptive statistics for trial and study population characteristics across all eligible trials. We assessed the presence of clinical heterogeneity within each pairwise comparison by comparing these characteristics [15].

Assessment of transitivity across treatment comparisons

The assumption of transitivity underlies NMA and needs careful evaluation. In the case that transitivity is not plausible in a network of trials, the indirect and mixed treatment effect estimates are not valid. To infer about the assumption of transitivity [25], we:

1. assessed whether the included interventions were similar when evaluated in RCTs with different designs by looking at the characteristics of included studies;
2. compared the distribution of the potential effect modifiers across the different pairwise comparisons by performing subgroup and sensitivity analyses (see the section 'Investigation of heterogeneity and incoherence' and 'Sensitivity analyses' below). If the distributions were balanced across comparisons, we concluded against evidence of intransitivity [26].

Assessment of statistical heterogeneity

In standard pairwise meta-analyses, we estimated different heterogeneity variances for each pairwise comparison. In network meta-analysis, we assumed a common estimate for the heterogeneity variance (τ^2) within and across comparisons. The presence of statistical heterogeneity within each pairwise comparison was assessed by visual inspection of the forest plots and by calculating the I-squared statistic [27]. The assessment of statistical heterogeneity in the entire network was based on the magnitude of the common τ^2 estimated from the NMA models [28]. We compared the magnitude of the heterogeneity variance with the empirical distribution as derived by Turner et al. [29] for dichotomous outcomes and as derived by Rhodes et al. [30] for continuous outcomes.

Assessment of statistical incoherence

To evaluate the presence of statistical incoherence locally, we used the loop-specific approach [31] and the Separate Indirect from Direct Evidence (SIDE, or node-splitting [32]) approach. The loop-specific method evaluates the incoherence assumption by calculating the incoherence factor (IF) as the difference between direct and indirect estimates for a specific comparison in each closed loop formed by the network of trials (using the Bucher method) and their relative 95% confidence intervals. For dichotomous outcomes, we reported the ratio of two odds ratios (ROR) from direct and indirect evidence in the loop. Then, we examined whether there were any material discrepancies; if the 95% CI did overlap with 1, the hypothesis of incoherence was not rejected, as described in Salanti.[33] We assumed a common heterogeneity estimate within each loop. The SIDE approach separates the evidence on a particular comparison, called node, into direct and indirect. The difference between direct and indirect is calculated and statistically tested. Both approaches were performed in STATA using the 'ifplot' and 'intervalplot' commands respectively. To check the assumption of incoherence in the entire network, we used the 'design-by-treatment' model [34]. This method accounts for different source of inconsistency that can occur when studies with different designs (two-arm

trials vs. three-arm trials) give different results as well as disagreement between direct and indirect evidence. Using this approach, we inferred the presence of inconsistency from any source in the entire network based on a chi-square test. The design-by-treatment model was performed in STATA using the 'mvmeta' command.

Investigation of heterogeneity and incoherence

We planned subgroup analyses to investigate the possible sources of heterogeneity and incoherence by using the following effect modifiers (for primary outcomes only):

1. studies sponsored *vs.* those not sponsored by pharmaceutical companies;
2. males *vs.* females
3. children *vs.* adolescents, since some medications (e.g., SSRIs) have been reported to have different efficacy in children *vs.* adolescents [35]; [if study data were not available for children (aged < 12 years) and adolescents (aged ≥ 12) separately, we planned to include them only in the main analysis (i.e. combining children and adolescents together).

However, we could only perform a subgroup analysis including industry sponsored studies, since only 21.8% of the studies were non-sponsored. The other subgroup analyses planned *a priori* were not conducted due to insufficient data.

Sensitivity analyses

We planned the following sensitivity analyses by excluding:

1) studies where all participants had IQ < 70;
2) studies where all participants had psychiatric/neurologic comorbidities;
3) studies lasting less than 2 and 3 weeks;
4) studies for which imputation of missing data was required;
5) studies with overall high or unclear risk of bias;
6) cross-over trials;
7) studies including patients resistant to ADHD medication;
8) studies recruiting only non-treatment-naïve patients;
9) studies excluding participants who previously did not respond to the same class of medication tested in the trial.

A final sensitivity analysis addressed whether unbalanced doses affected the results. To exclude trials with non-equivalent comparisons, we applied a previously validated approach used for antidepressant trials [36]. For this, we put together a roster (see Appendix Tables 3, 4 and 6, 7 in the Supplement) in which low and high doses of the drugs included in the present NMA are described. This roster was employed to detect inequalities in dosing that could affect comparative efficacy by excluding trials with low doses of one drug and high doses of the other (or vice-versa). We did not consider starting doses if these were supposed to be increased during the trial.

Appendix 5. Criteria for judging the confidence in network estimates

For each primary outcome, we evaluated the confidence in network estimates considering the following domains: study limitations, imprecision, inconsistency, indirectness and publication bias. We assigned 'no concern', 'some concerns' or 'major concerns' to each domain according to the criteria described below and then we provided an overall judgment across domains. We derived the judgments with the support of the web application CINeMA [37].

Study limitations

We assigned numerical scores to the overall rating of risk of bias for each study (see Appendix 3 for details on the criteria for deriving the overall risk of bias per study): 1 for *low*, 2 for *moderate* and 3 for *high* risk of bias. We considered the 'average' risk of bias to summarize the risk of bias across studies for each direct comparison. We evaluated the network estimates judgments for study limitations by calculating a weighted average of the risk of bias across direct comparisons using the direct contributions for each network estimate as weight. For example, if a network estimate received more than 50% of contribution from comparisons with low risk of bias, we assigned 'no concern' for study limitations to that estimate. We presented the contribution of each piece of direct evidence to the network estimate in a bar graph. In the graph, the bars are coloured according to the bias level of each direct comparison (green for low, yellow for unclear and red for high risk of bias) and their length is proportional to the percentage contribution of each direct comparison to the network estimates. The bar graphs for each outcome are showed in Appendix Figures 4.

Imprecision

We evaluated the imprecision of the estimates depending on whether their confidence intervals included values that could lead into different clinical decisions. Based on the opinion from members of the European ADHD Guidelines Group, we considered an odds ratios lower than 0.75 and larger than 1.25 as clinically important for dichotomous outcomes and a standardize mean difference lower than -0.2 and larger than 0.2 as clinically important for continuous outcomes. We illustrate the general strategy that we applied to judge imprecision of each relative network estimate in the figure below.

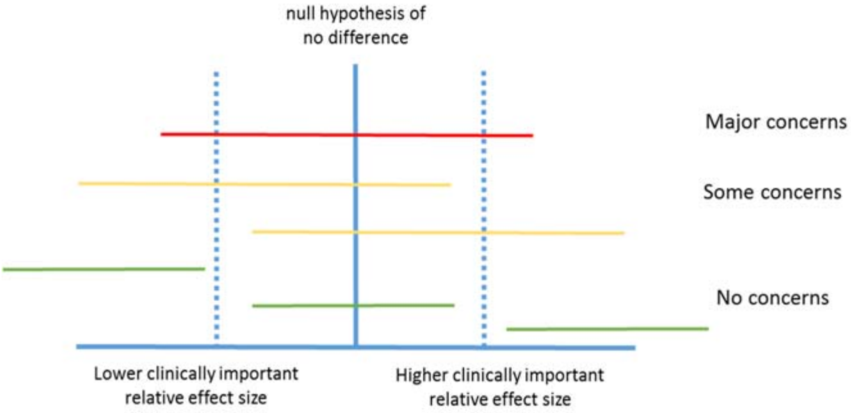

Relative treatment effects derived from the network meta-analysis are reported in Appendix Tables 13 in this Supplement.

Inconsistency

In the context of NMA, we need to consider two sources of inconsistency: the heterogeneity across studies and the incoherence (disagreement between direct and indirect evidence). We evaluated the two sources separately.

<u>Heterogeneity</u>

We evaluated the heterogeneity of the estimates according to the agreement of prediction intervals with the confidence intervals in relation to the clinically important effects, which were already defined in Imprecision. We assigned 'no concern' to the estimate when the confidence (black line) and prediction (coloured lines) intervals agree in relation to clinically important effect. We illustrate the general strategy that we applied to judge heterogeneity of each relative network estimate in the figure below.

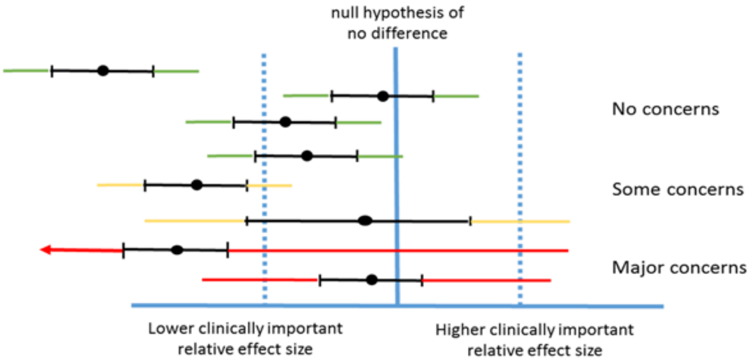

There might be situations in which the confidence interval is narrow and the prediction interval extends into clinically unimportant effects but it does not cross the null hypothesis of no difference which might be considered 'no concern' instead of 'some concerns' (see fifth scenario in the graph above). The plots presenting the confidence and predictive intervals for each network estimates are presented in Appendix Figures 5.

We also compared the heterogeneity variance estimated in each direct comparison with the reference heterogeneity variance derived by Turner at el. (29) and Rhodes et al. (30) to complete our judgments based on the previous criteria. The 50% quantile of the reference heterogeneity variance for a subjective outcome for pharmacological intervention versus placebo is 0.12 and between pharmacological interventions is 0.096; for a semi-subjective outcome is 0.049 and 0.040, respectively.

Incoherence

We assessed incoherence locally by using the SIDE (Separating Direct from Indirect Evidence or node-splitting)[32] and the loop-specific[31] approaches. We used the design-by-treatment interaction model to assess incoherence globally. We assigned 'no concern' to those comparisons for which only direct evidence exists or that receive more than 90% of contribution from direct evidence only. We assigned 'no concerns', 'some concerns' and 'major concerns' to those comparisons for which only indirect evidence exists when the p-value of the design-by-treatment interaction model is more 0.10, between 0.01 and 0.10, and less than 0.01, respectively. To judge incoherence for comparisons receiving contribution from both direct and indirect evidence (less than 90% from direct evidence) we used the criteria reported in the table below that considers the p-values of the design-by-treatment interaction model and the node-splitting approach.

		Design by treatment interaction model		
		p-value>0.1	0.01<p-value<0.1	p-value<0.01
SIDE approach	p-value>0.1	No concerns	No concerns	Some concerns
	0.01<p-value<0.1	Some concerns	Some concerns	Major concerns
	p-value<0.01	Some concerns	Major concerns	Major concerns

We reported the results from the loop-specific approach, SIDE approach and design-by-treatment interaction model in Appendix Tables 19 in this Supplement.

Indirectness

We judged each study for indirectness according to how relevant it is to the research question of the review and we assigned the following levels: completely relevant, partially relevant or not relevant. We allocated a numerical score to the judgments (1 for completely, 2 for partially, 3 for not relevant) and we used the average to summarized the study-level judgements across studies for each direct comparison. We evaluated the network estimates judgments for indirectness by calculating a weighted average of indirectness levels across direct comparisons using the contributions for each network estimate as weight. We integrated the evaluation for indirectness with the assessments for transitivity. We assigned 'no concern', 'some concerns', or 'major concerns' to the network estimates for indirectness using the approach as in study limitations and taking into account the considerations for transitivity. We reported in Appendix Figures 6 the bar graph that combines the contribution of each direct comparison to the network estimates with their relative level of indirectness.

Publication bias

We considered the comprehensiveness of the search strategy and the likelihood that studies may have been conducted but were not published. For each outcome, we plotted a comparison-adjusted funnel plot of all trials comparing at least one treatment versus placebo to see the presence of asymmetry [38]. The comparison-adjusted funnel plots are showed in Appendix Figures 3 in this Supplement.
We considered our search strategy comprehensive. We did not recommend downgrading because of publication bias in any comparison. However, due to the difficulties to judge the presence of publication bias we cannot completely exclude its impact in our estimates.

Overall rating

We assigned an overall rating of the confidence in each network estimates, which goes from high to very low, considering the assessments in all domains jointly (see Appendix Tables 22 in this Supplement).

Appendix 6. Additional *post hoc* analyses and changes to the pre-specified protocol

- **Dose of medications**: The original, pre-specified protocol published in Cortese et al. [39] stated "Only studies where medications were given within the licensed or recommended dose level (see tables 2 and 3) will be included". On reflection, the authors' group (European ADHD Guidelines Group, EAGG) deemed that it would not be appropriate to combine studies using the maximum licensed doses and studies using the maximum recommended (but not licensed) doses as the highest doses in the trial. To provide more clinically informative results, the authors' group decided to carry out three separate sets of analyses for each outcome:
    1. "Main" dose analysis, limited to studies on FDA licensed medications for ADHD, at the maximum dose licensed by the FDA, plus unlicensed medications for ADHD, as per our pre-specified protocol, at any dose. In case of forced titration trials on FDA licensed drugs using doses higher than the maximum licensed FDA dose, data relative to the maximum FDA licensed dose used during the titration, if available, were used.

    In addition to 1 (already planned in our first version of the protocol) two *post hoc* analyses were conducted:

    2. "FDA" dose analysis, restricted to studies including only FDA licensed medications for ADHD, at the maximum dose licensed by the FDA.
    3. "Inclusive" dose analysis, including studies on FDA licensed medications, at the maximum dose recommended in the most commonly used guidelines/formularies on ADHD medications (see below), plus studies on non FDA-licensed medications, at any dose.

  The maximum FDA licensed doses and the maximum doses recommended in the most commonly used guidelines/formularies are reported in Appendix Tables 3 (children/adolescents) and 4 (adults).

- **Lisdexamfetamine *vs.* other amphetamines:** Whereas in the original, pre-specified protocol, lisdexamfetamine was listed separately from amphetamines, on reflection the EAGG deemed it appropriate to conduct the main set of analyses lumping lisdexamfetamine with other amphetamines (since lisdexamfetamine is an amphetamine). However, lisdexamfetamine is metabolised very differently from other amphetamines. While other amphetamines undergo extensive first pass metabolism by CYP2D6 enzymes, lisdexamfetamine is protected from this as the prodrug needs to be metabolised first before the lisdexamfetamine is released. This might have an impact on efficacy and tolerability. As such, the EAGG deemed important to carry put a *post hoc* set of analyses in which lisdexamfetamine was considered separately from the other amphetamines.

- **Teachers' ratings for outcomes of studies in children:** The original protocol stated: "Teachers' and clinicians' scores will be considered for the primary analysis of studies in children/adolescents and adults, respectively". Teachers' rating were selected as primary outcome, in accordance to other meta-analyses [e.g, [11]], because they might provide less biased estimates. However, since the number of retained studies with clinicians' ratings largely exceeded that of studies with teachers' ratings for children (e.g., for the efficacy analysis closest to 12 weeks: studies with clinicians' rating: n=46; studies with teachers' ratings: n= 16), we deemed the analysis based on clinicians' ratings important and informative. Furthermore, clinicians' ratings may provide an alternative and complementary view to teachers' ratings. Indeed, information from multiple raters has been shown to increase the validity of ADHD diagnosis [40]. Therefore, we added clinicians' ratings of ADHD core symptoms as a primary outcome for children/adolescents.

- **Note on selection of scales for efficacy outcomes:** We specify here that if only scores from a subscale measuring one ADHD dimension (i.e., inattention or hyperactivity/impulsivity) were available, we used those scores for the analyses.

- **Additional sensitivity analysis:** Studies lasting ≥ 1 week were eligible for the present meta-analysis, since the authors' group deemed this period appropriate to appreciate a response to psychostimulants, and indeed several trials found a clinically significant response to psychostimulants after 1 week of treatment (e.g.[41-43]; response to non-psychostimulants, such as atomoxetine, takes longer, which is reflected in the longer duration of trials on this class of drugs). As per protocol, a sensitivity analysis was planned removing studies lasting less than 2 weeks, due to concerns that in some cases 1 week may not be appropriate to appreciate the effect of psychostimulants. An additional sensitivity analysis was conducted removing studies lasting less than 3 weeks, to make our results more comparable with those of other meta-analyses [e.g. [12, 44]].

- **Additional specification:** We specify here that trials in which all participants had a comoribid disorder pharmacologically treated with a medication other than an ADHD drug were excluded.

Appendix 7. Studies/citations discarded after assessing their full text, with reasons for exclusions. *This list includes also the international trial registries references that were excluded after assessing study inclusion/exclusion criteria.*

Aarskog1977

- Aarskog D, Fevang FO, Klove H, Stoa KF, Thorsen T. The effect of the stimulant drugs, dextroamphetamine and methylphenidate, on secretion of growth hormone in hyperactive children. *J Pediatr.* 1977;90(1):136-139.

Reason for exclusion: No DSM criteria; Less than seven days treatment

Aarts2015

- Aarts E, van Holstein M, Hoogman M, et al. Reward modulation of cognitive function in adult attention-deficit/hyperactivity disorder: a pilot study on the role of striatal dopamine. *Behav Pharmacol.* 2015;26(1-2):227-240.

Reason for exclusion: No RCT

Abbasi2011 (NCT01099072)

- Abbasi SH, Heidari S, Mohammadi MR, Tabrizi M, Ghaleiha A, Akhondzadeh S. Acetyl-L-carnitine as an adjunctive therapy in the treatment of attention-deficit/hyperactivity disorder in children and adolescents: a placebo-controlled trial. *Child Psychiatry Hum Dev.* 2011;42(3):367-75.
- https://clinicaltrials.gov/ct2/show/NCT01099072

Reason for exclusion: Treatment of interest for the present meta-analysis +placebo vs. treatment of interest + supplementation with Acetyl-L-carnitine

Abikoff1985a

- Abikoff H, Gittelman R. Hyperactive children treated with stimulants. Is cognitive training a useful adjunct? *Arch Gen Psychiatry.* 1985;42(10):953-961.

Reason for exclusion: No RCT

Abikoff1985b

- Abikoff H, Gittelman R. The normalizing effects of methylphenidate on the classroom behavior of ADDH children. *J Abnorm Child Psychol.* 1985;13(1):33-44.

Reason for exclusion: Initial single blind phase including placebo; then medication or cognitive training or attention training; follow up with single blind placebo phase

Abikoff2004

- Abikoff H, Hechtman L, Klein RG, et al. Symptomatic improvement in children with ADHD treated with long-term methylphenidate and multimodal psychosocial treatment. *J Am Acad Child Adolesc Psychiatry.* 2004;43(7):802-811
- Abikoff H, Hechtman L, Klein RG, et al. Social functioning in children with ADHD treated with long-term methylphenidate and multimodal psychosocial treatment. *J Am Acad Child Adolesc Psychiatry.* 2004;43(7):820-829
- Klein RG, Abikoff H, Hechtman L, Weiss G. Design and rationale of controlled study of long-term methylphenidate and multimodal psychosocial treatment in children with ADHD. *J Am Acad Child Adolesc Psychiatry.* 2004;43(7):792-801.

Reason for exclusion: No arms of interest for the present meta-analysis (methylphenidate; methylphenidate+psychosocial treatment; methylphenidate+ attention psychosocial control treatment)

Abikoff2005

- Abikoff H, McGough J, Vitiello B, et al. Sequential pharmacotherapy for children with comorbid attention-deficit/hyperactivity and anxiety disorders. *J Am Acad Child Adolesc Psychiatry.* 2005;44(5):418-427.

Reason for exclusion: Stimulant + fluvoxamine (STIM/FLV) or stimulant + placebo (STIM/PL).

Ackerman1982

- Ackerman PT, Dykman RA, Holcomb PJ, McCray DS. Methylphenidate effects on cognitive style and reaction time in four groups of children. *Psychiatry Res.*1982;7(2):199-213

Reason for exclusion: No clear DSM diagnosis; no pre-cross over data available (not possible to contact author, no e-mail contacts)

Ackerman1983

- Ackerman PT, Dykman RA, Holcomb PJ, McCray DS. Effects of high and low dosages of methylphenidate in children with strong and sensitive nervous systems. *Pavlov J Biol Sci.* 1983;18(1):36-48.

Reason for exclusion: Not all subjects had a diagnosis of ADHD; not possible to contact authors (no e-mail contacts)

ACTRN12610000432011

- http://www.anzctr.org.au/ACTRN12610000432011.aspx

Reasons for exclusion: Study was closed prior to competition and no data are available

ACTRN12617000156381

- http://www.anzctr.org.au/ACTRN12617000156381.aspx

Reasons for exclusion: Compound (Curcumin) of no interest for the present meta-analysis vs palcebo

ACTRN12616000125426

- http://www.anzctr.org.au/ACTRN12616000125426.asp

Reasons for exclusion: No arms of interest for the present meta-analysis; allocation not concealed

ACTRN12616000569404

- http://www.anzctr.org.au/ACTRN12616000569404.aspx

Reasons for exclusion: Open label

ACTRN12616000576426

- http://www.anzctr.org.au/ACTRN12616000576426.aspx

Reasons for exclusion: No participants with ADHD

ACTRN12616001332415

- http://www.anzctr.org.au/ACTRN12616001332415.aspx

Reasons for exclusion: No treatment of interest for the present meta-analysis, uncontrolled

ACTRN12616001448437

- http://www.anzctr.org.au/ACTRN12616001448437.aspx

Reasons for exclusion: No pharmacological treatment

ACTRN12605000507684

- http://www.anzctr.org.au/ACTRN12605000507684.aspx

Reasons for exclusion: Non randomised

ACTRN12607000138482

- http://www.anzctr.org.au/ACTRN12607000138482.aspx

Reasons for exclusion: No participants with ADHD

ACTRN12608000059369

- http://www.anzctr.org.au/ACTRN12608000059369.aspx

Reasons for exclusion: No RCT

ACTRN12609000271202

- http://www.anzctr.org.au/ACTRN12609000271202.aspx

Reasons for exclusion: No RCT

ACTRN12609000625279

- http://www.anzctr.org.au/ACTRN12609000625279.aspx

Reasons for exclusion: No participants with ADHD

ACTRN12610000652077

- http://www.anzctr.org.au/ACTRN12610000652077.aspx

Reasons for exclusion: Single dose

ACTRN12610000978066

- http://www.anzctr.org.au/ACTRN12610000978066.aspx

Reasons for exclusion: Intervention of no interest for the present meta-analysis vs. placebo

ACTRN12612000827831

- http://www.anzctr.org.au/ACTRN12612000827831.aspx

Reasons for exclusion: Intervention of no interest for the present meta-analysis vs. placebo

ACTRN12613000480785

- http://www.anzctr.org.au/ACTRN12613000480785.aspx

Reasons for exclusion: No double blind RCT; no interventions of interest for the present meta-analysis; age range: 3-4 years

ACTRN12610001093077

- http://www.anzctr.org.au/ACTRN12610001093077.aspx

Reasons for exclusion: No participants with ADHD

ACTRN12611000445976

- http://www.anzctr.org.au/ACTRN12611000445976.aspx

Reasons for exclusion: No participants with ADHD

ACTRN12612000718842

- http://www.anzctr.org.au/ACTRN12612000718842.aspx

Reasons for exclusion: No treatment of interest for the present meta-analysis

ACTRN12612000391875

- http://www.anzctr.org.au/ACTRN12612000391875.aspx

Reasons for exclusion: No participants with ADHD

ACTRN12613000896774

- http://www.anzctr.org.au/ACTRN12613000896774.aspx

Reasons for exclusion: No treatment of interest for the present meta-analysis

ACTRN12614000306617

- http://www.anzctr.org.au/ACTRN12614000306617.aspx

Reasons for exclusion: No RCT

ACTRN12615000093583

- http://www.anzctr.org.au/ACTRN12615000093583.aspx

Reasons for exclusion: No participants with ADHD

ACTRN12615000790549

- http://www.anzctr.org.au/ACTRN12615000790549.aspx

Reasons for exclusion: Treatment of no interest for the present meta-analysis vs. placebo

ACTRN12615001246572

- http://www.anzctr.org.au/ACTRN12615001246572.aspx

Reasons for exclusion: No participants with ADHD

Adamou2006

- Adamou M, Plummer W, Maidment I, Mirtsou-Fidani V, Hale A. Atomoxetine and cortical activity in adults with ADHD. *Int J Neuropsychopharmacol.* 2006;9:S258.

Reason for exclusion: No RCT

Adler2005

- Adler LA, Spencer TJ, Milton DR, Moore RJ, Michelson D. Long-term, open-label study of the safety and efficacy of atomoxetine in adults with attention-deficit/hyperactivity disorder: an interim analysis. *J Clin Psychiatry.* 2005;66(3):294-299.

Reason for exclusion: Open label trial

Adler2006

- Laing A, Aristides M. Attention deficit hyperactivity disorder (ADHD) in adults: SF-6D utilities from SF-36 scores in a randomised trial of atomoxetine. *Value Health.* 2005;8(6):A199-A199.
- Adler L, Dietrich A, Reimherr FW, et al. Safety and tolerability of once versus twice daily atomoxetine in adults with ADHD. *Ann Clin Psychiatry.* 2006;18(2):107-113.
- Adler LA, Sutton VK, Moore RJ, et al. Quality of life assessment in adult patients with attention-deficit/hyperactivity disorder treated with atomoxetine. *J Clin Psychopharmacol.* 2006;26(6):648-652.

Reason for exclusion: Comparison of two doses of the same compound (atomoxetine), no placebo arm, no other arms

Adler2008
- Adler LA. Adult ADHD pharmacotherapy. *Prim Care Companion J Clin Psychiatry*. 2008;10:469.

Reason for exclusion: No RCT

Adler2009
- Adler LA. Pharmacotherapy for adult ADHD. *J Clin Psychiatry.* 2009;70:e12.

Reason for exclusion: No RCT

Adler2011(NCT00468143)
- Adler LA, Lynch LR, Shaw DM, et al. Medication adherence and symptom reduction in adults treated with mixed amphetamine salts in a randomized crossover study. *Postgrad Med.* 2011;123(5):71-79.
- https://clinicaltrials.gov/ct2/show/NCT00468143

Reason for exclusion: No double blind and no controlled

Adler2014
- Related conference proceeding: Goto T, Adler L, Upadhyaya H, et al. Executive function in adult patients with attention-deficit/hyperactivity disorder during treatment with atomoxetine in a randomized, placebo-controlled withdrawal study. *Int J Neuropsychopharmacol.* 2012;15:220.
- Adler L, Tanaka Y, Williams D, et al. Executive function in adults with attention-deficit/hyperactivity disorder during treatment with atomoxetine in a randomized, placebo-controlled, withdrawal study. *J Clin Psychopharmacol.* 2014;34(4):461-466.

Reason for exclusion: Subjects responders in open label phase

Agarwal2001
- Agarwal V, Sitholey P, Kumar S, Prasad M. Double-blind, placebo-controlled trial of clonidine in hyperactive children with mental retardation. *Ment Retard.* 2001;39(4):259-267.

Reason for exclusion: Cross-over without wash out. Authors not able to provide pre-cross over data

Agay2010
- Agay N, Yechiam E, Carmel Z, Levkovitz Y. Non-specific effects of methylphenidate (Ritalin) on cognitive ability and decision-making of ADHD and healthy adults. *Psychopharmacology (Berl).* 2010;210(4):511-519.

Reason for exclusion: Single dose, no outcomes of interest

Agay2014(NCT01124032)
- Agay N, Yechiam E, Carmel Z, Levkovitz Y. Methylphenidate enhances cognitive performance in adults with poor baseline capacities regardless of attention-deficit/hyperactivity disorder diagnosis. *J Clin Psychopharmacol.* 2014;34(2):261-265.
- https://clinicaltrials.gov/ct2/show/NCT01124032

Reason for exclusion: Single dose

Aharonovich2006
- Aharonovich E, Garawi F, Bisaga A, et al. Concurrent cannabis use during treatment for comorbid ADHD and cocaine dependence: effects on outcome. *Am J Drug Alcohol Abuse.* 2006;32(4):629-635.

Reason for exclusion: Concurrent CBT

Ahmann1993
- Ahmann P, Theyre F, Waltonen S, Van Erem A. Double blind placebo-controlled crossover of Ritalin®. A useful clinical tool? *Ann Neurol.* 1990; 28;444
- Ahmann PA, Waltonen SJ, Olson KA, Theye FW, Van Erem AJ, LaPlant RJ. Placebo-controlled evaluation of Ritalin side effects. *Pediatrics*. 1993;91(6):1101-1106.

Reason for exclusion: Cross-over, no wash out, not possible to gather pre-cross over data

Ahmann2000

- Ahmann PA, Theye FW, Berg R, et al. Long-term behavioral response to adderall in children and adolescents with ADHD. *Pediatr Res.* 2000:22a.

Reason for exclusion: Participants responders from a previous RCT phase

Ahmann2001

- Preliminary results in: Ahmann P, Theye F, Waltonen S, et al. Efficacy and side effects profile of adderall in newly diagnosed children with attention deficit disorder. Preliminary results. *Ann Neurol.* 1998:541.
- Ahmann P, Theye F, Waltonen S et al. Safety and efficacy of Aderall in children newly diagnosed with Attention Deficit Hyperactivity Disorder. *Neurology.* 1999; S2: A154
- Ahmann PA, Theye FW, Berg R, Linquist AJ, Van Erem AJ, Campbell LR. Placebo-controlled evaluation of amphetamine mixture-dextroamphetamine salts and amphetamine salts (Adderall): efficacy rate and side effects. *Pediatrics.* 2001;107(1): E10.

Reason for exclusion: cross-over, no wash out, not possible to gather pre-cross over data; no diagnostic criteria

Akhondzadeh2003

- Akhondzadeh S, Tavakolian R, Davari-Ashtiani R, Arabgol F, Amini H. Selegiline in the treatment of attention deficit hyperactivity disorder in children: a double blind and randomized trial. *Prog Neuropsychopharmacol Biol Psychiatry.* 2003;27(5):841-845.

Reason for exclusion: Medication of interest vs. medication of non interest for the present meta-analysis; no placebo arm

Akhondzadeh2004(ISRCTN64132371)

- Akhondzadeh S, Mohammadi MR, Khademi M. Zinc sulfate as an adjunct to methylphenidate for the treatment of attention deficit hyperactivity disorder in children: a double blind and randomized trial *BMC Psychiatry.* 2004;4:9.
- http://isrctn.org/ISRCTN64132371

Reason for exclusion: No arms of interest for the present meta-analysis (methylphenidate + zinc vs methylphenidate + placebo)

Akhondzadeh2005

- Akhondzadeh S, Mohammadi MM, Momeni, F. Passiflora incarta in the treatment of attention-deficit hyperactivity disorder in children and adolescents, *Therapy* 2005*;* 2(4): 609-614

Reason for exclusion: Compound of no interest for the present meta-analysis vs. methylphenidate, no placebo arm

Alban2004

- Alban JP, Hopson MM, Ly V, Whyte J. Effect of methylphenidate on vital signs and adverse effects in adults with traumatic brain injury. *Am J Phys Med Rehabil.* 2004;83(2):131-137.

Reason for exclusion: No participants with ADHD

Alexandris1968

- Alexandris A, Lundell FW. Effect of thioridazine, amphetamine and placebo on the hyperkinetic syndrome and cognitive area in mentally deficient children. *Can Med Assoc J.* 13 1968;98(2):92-96.

Reason for exclusion: No DSM/ICD criteria

Allen2002

- Allen AJ, Wernicke JF, Dunn D et al. Safety and efficacy of atomoxetine in pediatric CYP2D6 extensive vs. poor metabolizers. *Biol Psychiatry* 2002; 51:37S
- Related to: Michelson D, Read HA, Ruff DD, Witcher J, Zhang S, McCracken J. CYP2D60 and Clinical Response to Atomoxetine in Children and Adolescents with ADHD. *J Am Acad Child Adolesc Psychiatry.* 2007;46(2):242-51

Reason for exclusion: Meta analysis

Altszuler2017

- Altszuler AR, Morrow AS, Merrill BM, et al. The Effects of Stimulant Medication and Training on Sports Competence Among Children With ADHD. *J Clin Child Adolesc Psychol.* 2017:1-13.

Reason for exclusion: No outcomes of interest; concomitant sport training

Aman1974

- Aman, MG, Sprague, RL. The state-dependent effects of methylphenidate and dextroamphetamine. *J Nerv Ment Dis.* 1974;158 (4) 268-279.

Reason for exclusion: No DSM/ICD criteria

Aman1991a

- Aman MG, Turbott SH. Prediction of clinical response in children taking methylphenidate. *J Autism Dev Disord.* 1991;21(2):211-228.

Reason for exclusion: No RCT

Aman1991b

- Aman MG, Marks RE, Turbott SH, Wilsher CP, Merry SN. Clinical effects of methylphenidate and thioridazine in intellectually subaverage children. *J Am Acad Child Adolesc Psychiatry.* 1991;30(2): 246- 256.

Reason for exclusion: Not all participants with ADHD

Aman1993

- Aman MG, Kern RA, McGhee DE, Arnold LE. Fenfluramine and methylphenidate in children with mental retardation and ADHD: clinical and side effects. *J Am Acad Child Adolesc Psychiatry.* 1993;32(4):851-859.
- Aman MG, Kern RA, McGhee DE, Arnold LE. Fenfluramine and methylphenidate in children with mental retardation and attention deficit hyperactivity disorder: laboratory effects. *J Autism Dev Disord.* 1993;23(3):491-506

Reason for exclusion: No mention of randomization (Latin square)- no additional information from authors

Aman2003

- Aman, MG, Kern, RA, Osborne P, Tumuluru R, Rojahn J, Medico V. Fenfluramine and methylphenidate in children with mental retardation and borderline IQ: Clinical effects *Am J Ment Retard.* 1997;101(5):521-34
- Aman MG, Armstrong S, Buican B, Sillick T. Four-year follow-up of children with low intelligence and ADHD: A replication. Research in Developmental Disabilities, 23, 119-134. *Res Dev Disabil.* 2002;23(2):119-34
- Aman MG, Buican B, Arnold LE. Methylphenidate treatment in children with borderline IQ and mental retardation: analysis of three aggregated studies. *J Child Adolesc Psychopharmacol.* 2003;13(1):29-40.

Reason for exclusion: Not all participants> 5 years, not all subjects had a diagnosis of ADHD; no mention of randomization

Aman2004

- Aman MG, De Smedt G, Derivan A, et al. Double-blind, placebo-controlled study of risperidone for the treatment of disruptive behaviors in children with subaverage intelligence. *Am J Psychiatry.* 2002;159(8):1337-1346.
- Snyder R, Turgay A, Aman M, Binder C, Fisman S, Carroll A & The Risperidone Conduct Study Group. Effects of risperidone on conduct and disruptive behavior disorders in the children with subaverage IQs. *J Am Acad Child Adolesc Psychiatry.* 2002;41(9):1026-36.
- Aman MG, Binder C, Turgay A. Risperidone effects in the presence/absence of psychostimulant medicine in children with ADHD, other disruptive behavior disorders, and subaverage IQ. *J Child Adolesc Psychopharmacol.* 2004;14(2):243-54.

Reason for exclusion: Placebo (with or without stimulants) vs. risperidone (with or without risperidone)

Aman2014(NCT00796302)

- Farmer CA, Arnold LE, Bukstein OG, et al. The treatment of severe child aggression (TOSCA) study: Design challenges. *Child Adolesc Psychiatry Ment Health.* 2011;5(36).
- Aman MG, Bukstein OG, Gadow KD, et al. What does risperidone add to parent training and stimulant for severe aggression in child attention-deficit/hyperactivity disorder? *J Am Acad Child Adolesc Psychiatry.* 2014;53(1):47-60 e41.
- Gadow KD, Arnold LE, Molina BSG, et al. Risperidone added to parent training and stimulant medication: Effects on attention-deficit/hyperactivity disorder, oppositional defiant disorder, conduct disorder, and peer aggression. *J Am Acad Child Adolesc Psychiatry.* 2014;53(9):948-959.e941.
- Farmer CA, Brown NV, Gadow KD, et al. Comorbid Symptomatology Moderates Response to Risperidone, Stimulant, and Parent Training in Children with Severe Aggression, Disruptive Behavior Disorder, and Attention-Deficit/Hyperactivity Disorder. *J Child Adolesc Psychopharmacol.* 2015;25(3):213-224.
- Rundberg-Rivera EV, Townsend LD, Schneider J, et al. Participant Satisfaction in a Study of Stimulant, Parent Training, and Risperidone in Children with Severe Physical Aggression. *J Child Adolesc Psychopharmacol.* 2015;25(3):225-233.
- Arnold LE, Gadow KD, Farmer CA, Findling RL, Bukstein O, Molina BS, Brown NV, Li X, Rundberg-Rivera EV, Bangalore S, Buchan-Page K, Hurt EA, Rice R, McNamara NK, Aman MG. Comorbid anxiety and social avoidance in treatment of severe childhood aggression: response to adding risperidone to stimulant and parent training; mediation of disruptive symptom response. *J Child Adolesc Psychopharmacol.* 2015;25(3):203-12.

- Gadow KD, Brown NV, Arnold LE, et al. Severely Aggressive Children Receiving Stimulant Medication Versus Stimulant and Risperidone: 12-Month Follow-Up of the TOSCA Trial. *J Am Acad Child Adolesc Psychiatry.* 2016;55(6):469-478.
- Farmer CA, Epstein JN, Findling RL, Gadow KD, Arnold LE, Kipp H, Kolko DJ, Butter E, Schneider J, Bukstein OG, McNamara NK, Molina BS, Aman MG. Risperidone Added to Psychostimulant in Children with Severe Aggression and Attention-Deficit/Hyperactivity Disorder: Lack of Effect on Attention and Short-Term Memory. *J Child Adolesc Psychopharmacol.* 2017;27(2):117-124
- https://clinicaltrials.gov/ct2/show/NCT00796302

Reason for exclusion: No arms of interest for the present meta-analysis (parent training+ stimulants + placebo vs. parent training+ stimulants+risperidone)

Amery1984

- Amery B, Minichiello MD, Brown GL. Aggression in hyperactive boys: Response to d-amphetamine. *J Am Acad Child Psychiatry.* 1984(3):291-294.

Reason for exclusion: Cross-over without wash out; no pre cross-over data; not possible to contact author

Amiri2012

- Amiri S, Farhang S, Ghoreishizadeh MA, Malek A, Mohammadzadeh S. Double-blind controlled trial of venlafaxine for treatment of adults with attention deficit/hyperactivity disorder. *Hum Psychopharmacol.* 2012;27(1):76-81.

Reason for exclusion: Medication of no interest for the present meta-analysis vs. placebo, no other arm

Anderson1980

- Anderson J. Methylphenidate and hyperactivity. *S Afr Med J.* 1980;57:181-2.

Reasons for exclusion: No RCT

Anderson2002

- Anderson CM, Polcari A, Lowen SB, Renshaw PF, Teicher MH. Effects of methylphenidate on functional magnetic resonance relaxometry of the cerebellar vermis in boys with ADHD. *Am J Psychiatry.* 2002;159(8):1322-1328.

Reason for exclusion: Contacted authors twice (13.12.15 and 18.1.16) to obtain data on outcomes of interest for the present meta-analysis but we were not able to obtain relevant data

Andriola2000

- Andriola MR. Efficacy and safety of methylphenidate and pemoline in children with attention deficit hyperactivity disorder. *Curr Ther Res Clin.* 2000;61(4):208-15.

Reasons for exclusion: No RCT

Anonymous1996

- No authors listed**.** Clonidine for treatment of attention-deficit/hyperactivity disorder. *Med Lett Drugs Ther.*. 1996; 38, 109-110.

Reason for exclusion: No RCT

Anonymous2002

- Anonymous. Dexmethylphenidate (Focalin) for ADHD. *Med Lett Drugs Ther.* 2002;44(1130):45-6

Reason for exclusion: No RCT

Anton1969

- Anton A, Greer M. Dextroamphetamine, catecholamines, and behavior. *Arch Neurol.* 1969;21:248–252.

Reason for exclusion: No DSM/ICD criteria as per protocol

Arabgol2009

- Arabgol F, Panaghi L, Hebrani P. Reboxetine versus methylphenidate in treatment of children and adolescents with attention deficit-hyperactivity disorder. *Eur Child Adolesc Psychiatry.* 2009;18(1):53-59.

Reason for exclusion: Medication of interest vs. medication of no interest for the present meta-analysis

Arnett1996

- Arnett Peter A. The Effect of Ritalin on Response to Reward and Punishment in Children with ADHD. *Child-Study-Journal.* 1996(1):51-70.

Reason for exclusion: No relevant outcomes; not possible to contact authors

Arnold1972a

- Arnold LE, Strobl D, Weisenberg A. Hyperkinetic adult. Study of the "paradoxical" amphetamine response. *JAMA.* 1972;222(6):693-694.

Reason for exclusion: Case report, No DSM/ICD criteria

Arnold1972b

- Arnold LE, Wender, PH, McCloskey K, Snyder, SH. Levoamphetamine and dextroamphetamine comparative efficacy in the hyperkinetic syndrome. *Arch Gen Psychiatry*1972;27(6):816-22

Reason for exclusion: No ADHD diagnosis According to DSM/ICD criteria as per protocol

Arnold1976

- Arnold LE, Huestis RD, Smeltzer DJ, Scheib J, Wemmer D, Colner G. Levoamphetamine vs dextroamphetamine in minimal brain dysfunction. Replication, time response, and differential effect by diagnostic group and family rating. *Arch Gen Psychiatry.* 1976;33(3):292-301.

Reason for exclusion: Diagnosis of Minimal Brain Dysfunction, no DSM-ICD criteria

Arnold1978a

- Arnold LE, Huestis RD, Wemmer D, Smeltzer DJ. Differential effect of amphetamine optical isomers on Bender Gestalt performance of the minimally brain dysfunctioned. *J Learn Disabil.* 1978;11(3):127-132.

Reason for exclusion: No ADHD diagnosis According to DSM/ICD criteria as per protocol

Arnold1978b

- Arnold LE, Christopher J, Huestis R, Smeltzer DJ. Methylphenidate vs dextroamphetamine vs caffeine in minimal brain dysfunction: controlled comparison by placebo washout design with Bayes' analysis. *Arch Gen Psychiatry.* 1978;35(4):463-473.

Reason for exclusion: no ADHD diagnosis According to DSM/ICD criteria as per protocol

Arnold1989

- Arnold LE, Kleykamp D, Votolato NA, Taylor WA, Kontras SB, Tobin K. Gamma-linolenic acid for attention-deficit hyperactivity disorder: placebo-controlled comparison to D-amphetamine. *Biol Psychiatry.* 1989;25(2):222-228
- Arnold LE, Pinkham SM, Votolato, N. Does zinc moderate essential fatty acid and amphetamine treatment of attention-deficit/hyperactivity disorder? *J Child Adolesc Psychopharmacol.* 2000;10(2): 111-117.

Reason for exclusion: Cross-over without wash out, no pre cross-over data available

Arnold2005

- Arnold LE, Lindsay RL, Conners CK, et al. A double-blind, placebo-controlled withdrawal trial of dexmethylphenidate hydrochloride in children with attention deficit hyperactivity disorder. *J Child Adolesc Psychopharmacol.* 2005;14(4):542-554

Reasons for exclusion: withdrawal design

Arnold2010(NCT00151983)

- Arnold LE, Bozzolo DR, Hodgkins P, et al. Switching from oral extended-release methylphenidate to the methylphenidate transdermal system: continued attention-deficit/hyperactivity disorder symptom control and tolerability after abrupt conversion. *Curr Med Res Opin.* 2010;26(1):129-137.
- https://clinicaltrials.gov/ct2/show/NCT00151983

Reason for exclusion: Open label

Arnold2011

- Arnold LE, Disilvestro RA, Bozzolo D, et al. Zinc for attention-deficit/hyperactivity disorder: placebo-controlled double-blind pilot trial alone and combined with amphetamine. *J Child Adolesc Psychopharmacol.* 2011;21(1):1-19.

Reason for exclusion: Treatment of no interest for the present meta-analysis vs placebo

Arnold2016(NCT02520388)

- Arnold VK, DeSousa NJ, Incledon B, et al. Pivotal phase 3 trial evaluating the efficacy and safety of HLD200, a novel delayed-release and extended-release formulation of methylphenidate, in children with attention-deficit/ hyperactivity disorder. *J Am Acad Child Adolesc Psychiatry.* 2016;55 (10 Supplement 1):S170.
- https://clinicaltrials.gov/ct2/show/NCT02520388

Reason for exclusion: Subjects had current or prior response on MPH

Ashare2010
- Ashare RL, Hawk LW, Jr., Shiels K, Rhodes JD, Pelham WE, Jr., Waxmonsky JG. Methylphenidate enhances prepulse inhibition during processing of task-relevant stimuli in attention-deficit/hyperactivity disorder. *Psychophysiology.* 2010;47(5):838-845.

Reason for exclusion: Less than 7 days treatment (3 days)

Asherson2004
- Asherson P, Libretto SE. Long-acting methylphenidate for the treatment of adults with attention deficit hyperactivity disorder. *Br J Dev Disab* 2004; 50(2): 143-151.

Reason for exclusion: No RCT

Ashkenasi2011(NCT00989950)
- Ashkenasi A. Effect of transdermal methylphenidate wear times on sleep in children with attention deficit hyperactivity disorder. *Pediatr Neurol.* 2011;45(6):381-386.
- https://clinicaltrials.gov/ct2/show/NCT00989950

Reason for exclusion: Open label, formulation of no interest for the present meta-analysis (methylphenidate transdermal)

Auiler2002
- Auiler JF, Liu K, Lynch JM, Gelotte CK. Effect of food on early drug exposure from extended-release stimulants: results from the Concerta, Adderall XR Food Evaluation (CAFE) Study. *Curr Med Res Opin.* 2002;18(5):311-316

Reason for exclusion: Open label

B4Z-MC-LYAU
- https://assets.contentful.com/hadumfdtzsru/4Gzz2vxGr62UKAuaM8qECS/745ee21daafc3898b79e497635d01f89/Atomoxetine-B4Z-MC-LYAU.pdf
- Additional information provided by manufacturer

Reason for exclusion: No usable data

B4Z-MC-LYDO
- https://www.clinicaltrialsregister.eu/ctr-search/search?query=eudract_number:2007-007672-41

Reasons for exclusion: Randomized withdrawal study

B4Z-BP-LYBS
- https://www.clinicaltrialsregister.eu/ctr-search/search?query=eudract_number:2006-005512-27
- https://assets.contentful.com/hadumfdtzsru/3qmYCZsljqOIOYKEQe0wQG/137824269a90fdf714a2b7730cd7f8a6/Atomoxetine-B4Z-BP-LYBS.pdf

Reasons for exclusion: Open label

B4Z-US-HFBC
- https://www.accessdata.fda.gov/drugsatfda_docs/nda/2002/21-411_Strattera_biopharmr_P1.pdf (page 47)
- https://assets.contentful.com/hadumfdtzsru/cVYgRZb4zYmCEawQkses8/8a8fbd4d649ab0b57eadc0d7bc79054c/Atomoxetine-B4Z-US-HFBC.pdf

Reasons for exclusion: No RCT

B4Z-MC-HFBF
- https://www.accessdata.fda.gov/drugsatfda_docs/nda/2002/21-411_Strattera_biopharmr_P1.pdf (page 49)
- https://assets.contentful.com/hadumfdtzsru/4a1gyFxxuUcckU8suo24CW/a56bf14d4a9c25fba401da62fd65705b/Atomoxetine-B4Z-MC-HFBF.pdf

Reasons for exclusion: Open label

B4Z-MC-HFBE
- https://www.accessdata.fda.gov/drugsatfda_docs/nda/2002/21-411_Strattera_biopharmr_P1.pdf (page 49)
- https://assets.contentful.com/hadumfdtzsru/6EQer0lecEW6CqQ8GugA4I/184149952ceaed7d1631349c306bbf4c/Atomoxetine-B4Z-MC-HFBE.pdf

Reasons for exclusion: Open label

B4Z-MC-LYAD

- https://assets.contentful.com/hadumfdtzsru/1WD918BrfGy4002Yc0aOMK/39d9ebcc753f5f9e0b6f6c9b1755c00e/Atomoxetine-B4Z-MC-LYAD.pdf

Reasons for exclusion: No participants with ADHD

B4Z-MC-LYAB

- https://assets.contentful.com/hadumfdtzsru/FSv9FfcL0iYC40sqOa62y/60c46311fb3408ba47aef7c07a87d45e/Atomoxetine-B4Z-MC-LYAB.pdf

Reasons for exclusion: Open label

B4Z-MC-LYAQ

- https://assets.contentful.com/hadumfdtzsru/1nNm0D966wASoWkk6yGIUq/be6e4258cd352aef54203e06b8e28df7/Atomoxetine-B4Z-MC-LYAQ.pdf

Reasons for exclusion: No participants with ADHD

B4Z-MC-LYAR

- https://assets.contentful.com/hadumfdtzsru/1rK8tUlSaEawaUcu4c8gMS/bd234d2225ed56fe1842b8282c512caa/Atomoxetine-B4Z-MC-LYAR.pdf

Reasons for exclusion: Open label

B4Z-MC-LYBB

- *https://assets.contentful.com/hadumfdtzsru/5BnSDR7sg8EAACoK2SSgg8/07da69cadeb6e9b55ac12012f437e8e2/Atomoxetine-B4Z-MC-LYBB.pdf*

Reasons for exclusion: Open label

B4Z-MC-LYBN

- *https://assets.contentful.com/hadumfdtzsru/5vbWm1YQ1OGWewMYOQGAiE/6b3eb283ef2340992a9ade62d031a967/Atomoxetine-B4Z-MC-LYBN.pdf*

Reasons for exclusion: No participants with ADHD

B4Z-MC-LYBO

- https://assets.contentful.com/hadumfdtzsru/QIuyl7GX4sI6iaOuQ6kiw/4e991ac3c8bd7899f2737fac4bad6bda/Atomoxetine-B4Z-MC-LYBO.pdf

Reasons for exclusion: No participants with ADHD

B4Z-MC-LYBU

- https://assets.contentful.com/hadumfdtzsru/1HxhFKJ85i0i28mMcaoe4e/9c31d04ced0fbd94ed81607f16b6999c/Atomoxetine-B4Z-MC-LYBU.pdf

Reasons for exclusion: Participants selected if non responders to stimulants

B4Z-MC-LYCG

- https://assets.contentful.com/hadumfdtzsru/2lzHrNFZ8siSIY8O0kwSQG/bd02db76eb1536d69164d1c6910b94ed/Atomoxetine-B4Z-MC-LYCG.pdf

Reasons for exclusion: No participants with ADHD

B4Z-US-LYCE

- https://assets.contentful.com/hadumfdtzsru/49cPOEoTHaoa4kSOCogWcO/7531b008e0f5d89c1f0a43e5f970870d/Atomoxetine-B4Z-US-LYCE.pdf

Reasons for exclusion: Open label

B4Z-FW-LYCT

- https://assets.contentful.com/hadumfdtzsru/5QO3vt3I4gACqguSWcmmiS/b7b53219472d89d0f0c5b4b83b3fa828/Atomoxetine-B4Z-FW-LYCT.pdf

Reasons for exclusion: No participants with ADHD

B4Z-SB-LYDD

- https://assets.contentful.com/hadumfdtzsru/5gUVE8l7WMCkM6wKISYOom/fe7def589801266b75910682ca6f1896/Atomoxetine-B4Z-SB-LYDD.pdf

Reasons for exclusion: Open label

B4Z-CA-S012

- *https://assets.contentful.com/hadumfdtzsru/2wTyRiu4iQyAUAEEI6kmAA/3b996e020f33e84c33ec1e333a66aa81/Atomoxetine-B4Z-CA-S012.pdf*

Reasons for exclusion: Open label

B4Z-CA-S013

- https://assets.contentful.com/hadumfdtzsru/fa1xjFCkpyOqIIkWwEAWE/6e2e2f1d95b21b8366f0deab2cc532ab/Atomoxetine-B4Z-CA-S013.pdf

Reasons for exclusion: Open label

B4Z-FW-LYDP

- https://assets.contentful.com/hadumfdtzsru/4vs4jAbpEcaIMi4S4Uq0c8/303dc268260548a7277e564030c33925/Atomoxetine-B4Z-FW-LYDP.pdf

Reasons for exclusion: No participants with ADHD, Open label

Bailey2011

- Derefinko KJ, Bailey UL, Milich R, Lorch EP, Riley E. The effects of stimulant medication on the online story narrations of children with ADHD. *School Ment Health.* 2009;1;171-182.
- Bailey UL, Derefinko KJ, Milich R, Lorch EP, Metze A. The effects of stimulant medication on free recall of story events among children with ADHD. *J Psychopathol Behav Assess.* 2011;33(4):409-419.

Reason for exclusion: Less than seven days treatment

Balthazor1991

- Balthazor MJ, Wagner RK, Pelham WE. The specificity of the effects of stimulant medication on classroom learning-related measures of cognitive processing for attention deficit disorder children. *J Abnorm Child Psychol.* 1991;19(1):35-52.

Reason for exclusion: Less than seven days treatment

Barcai1971

- Barcai, A. Predicting the response of children with learning disabilities and behavior problems to dextramphetamine sulfate: the clinical interview and the finger twich test. *Pediatrics*. 1971;47;73-80.

Reason for exclusion: No ADHD

Baren2000

- Baren M, Swanson JM, Wigal SB. Lack of effect of different breakfast conditions on the pharmacokinetics and efficacy of OROS methylphenidate HCI extended-release tablets in children with ADHD. *Pediatr Res.* 2000:23a.

Reason for exclusion: Cross-over *than seven days treatment*

Barkley1977

- Barkley RA. The effects of methylphenidate on various types of activity level and attention in hyperkinetic children. *J Abnorm Child Psychol.* 1977;5(4):351-369.
- Barkley RA, Jackson TL, Jr. Hyperkinesis, autonomic nervous system activity and stimulant drug effects. *J Child Psychol Psychiatry.* 1977;18(4):347-357.

Reason for exclusion: No DSM/ICD criteria as per protocol

Barkley1979a

- Barkley RA, Cunningham CE. The effects of methylphenidate on the mother-child interactions of hyperactive children. *Arch Gen Psychiatry.* 1979;36(2):201-208.

Reason for exclusion: No DSM/ICD criteria

Barkley1979b

- Barkley RA, Cunningham CE. Stimulant drugs and activity level in hyperactive children. *Am J Orthopsychiatry.*1979(3):491-499. http://onlinelibrary.wiley.com/o/cochrane/clcentral/articles/001/CN-00544001/frame.html.

Reason for exclusion: No DSM/ICD criteria

Barkley1983

- Barkley RA, Cunningham CE, Karlsson J. The speech of hyperactive children and their mothers: comparison with normal children and stimulant drug effects. *J Learn Disabil.* 1983;16(2):105-110.

Reason for exclusion: No DSM/ICD criteria

Barkley1984

- Barkley RA, Karlsson J, Strzelecki E, Murphy JV. Effects of age and Ritalin dosage on the mother-child interactions of hyperactive children. *J Consult Clin Psychol.* 1984;52(5):750-758

Reason for exclusion: No outcomes of interest (Dr Barkley confirmed data are not available anymore)

Barkley1985

- Barkley RA, Karlsson J, Pollard S, Murphy JV. Developmental changes in the mother-child interactions of hyperactive boys: effects of two dose levels of Ritalin. *J Child Psychol Psychiatry.* 1985;26(5):705-715.

Reason for exclusion: No outcomes of interest (Dr Barkley confirmed data are not available anymore)

Barkley1988a

- Barkley RA. The effects of methylphenidate on the interactions of preschool ADHD children with their mothers. *J Am Acad Child Adolesc Psychiatry.* 1988(3):336-341.
  Reasons for exclusion: Pre-schoolers (2.5 to 4 years)

Barkley1988b

- Barkley RA, Fischer M, Newby RF, Breen MJ. Development of a multimethod clinical protocol for assessing stimulant drug response in children with attention deficit disorder. *J Clin Child Psychol.* 1988;17(1):14-24

Reason for exclusion: Cross-over without wash out, no pre cross-over data available

Barkley1989a

- Barkley RA, McMurray MB, Edelbrock CS, Robbins K. The response of aggressive and nonaggressive ADHD children to two doses of methylphenidate. [Erratum appears in *J Am Acad Child Adolesc Psychiatry.* 1990;29(4):670]. *J Am Acad Child Adolesc Psychiatry.* 1989;28(6):873-881.
- Overlaps with sample in: Barkley RA, McMurray MB, Edelbrock CS, Robbins K. Side effects of methylphenidate in children with attention deficit hyperactivity disorder: a systemic, placebo-controlled evaluation. *Pediatrics.* 1990;86(2):184-192.

Reasons for exclusion: After email exchange with Dr Barkley, agreed that randomization method is not appropriate ("Children were assigned to conditions by rolling a die. Drug conditions were numbered in sequence. If that child's die produced a number for a drug condition that was already full of needed participants, then the die was thrown again and the subject assigned to that condition. This continued until all drug orders were full")

Barkley1989b

- Barkley RA. Hyperactive girls and boys: stimulant drug effects on mother-child interactions.[Erratum appears in *J Child Psychol Psychiatry* 1993;34(3):437]. *J Child Psychol Psychiatry.* 1989;30(3):379-390.

Reason for exclusion: no relevant outcome available (Dr Barkley confirmed data are not available any more)

Barkley1990

- Barkley RA, McMurray MB, Edelbrock CS, Robbins K. Side effects of methylphenidate in children with attention deficit hyperactivity disorder: a systemic, placebo-controlled evaluation. *Pediatrics.* 1990;86(2):184-192.

Reason for exclusion: Not appropriate randomization

Barkley1991

- Barkley RA, DuPaul GJ, McMurray MB. Attention deficit disorder with and without hyperactivity: clinical response to three dose levels of methylphenidate. *Pediatrics.* 1991;87(4):519-531.
- Same sample as: DuPaul GJ, Barkley RA, McMurray MB. Response of children with ADHD to methylphenidate: interaction with internalizing symptoms. *J Am Acad Child Adolesc Psychiatry.* 1994;33(6):894–903.

Reason for exclusion: Cross-over without wash out, no pre cross-over data available

Barkley1997

- Barkley RA, Koplowitz S, Anderson T, McMurray MB. Sense of time in children with ADHD: effects of duration, distraction, and stimulant medication. *J Int Neuropsychol Soc.* 1997;3(4):359-369.

Reason for exclusion: No outcomes of interest (Dr Barkley confirmed data are not available any more)

Barkley2000

- Barkley RA, Connor DF, Kwasnik D. Challenges to determining adolescent medication response in an outpatient clinical setting: comparing Adderall and methylphenidate for ADHD. *J Atten Disord.* 2000;4(2):102–13

Reason for exclusion: Cross-over without wash out, no pre cross-over data available

Barkley2005

- Barkley RA, Murphy KR, O'Connell T, Connor DF. Effects of two doses of methylphenidate on simulator driving performance in adults with attention deficit hyperactivity disorder. *J Safety Res.* 2005;36(2):121-131.

Reason for exclusion: Acute single dose

Barkley2007

- Barkley RA, Anderson DL, Kruesi M. A pilot study of the effects of atomoxetine on driving performance in adults with ADHD. *J Atten Disord.* 2007;10(3):306-316

Reason for exclusion: Cross-over without wash out, no pre cross-over data available

Barragán2014

- Barragan E, Breuer D, Dopfner M. Efficacy and Safety of Omega-3/6 Fatty Acids, Methylphenidate, and a Combined Treatment in Children With ADHD.*J Atten Disord. 2*017;21(5):433-441

Reason for exclusion: Open label

Barrickman1995

- Barrickman LL, Perry PJ, Allen AJ, et al. Bupropion versus methylphenidate in the treatment of attention-deficit hyperactivity disorder. *J Am Acad Child Adolesc Psychiatry.* 1995;34(5):649-657.

Reason for exclusion: No usable data

Bawden1997

- Bawden HN, MacDonald GW, Shea S. Treatment of children with Williams syndrome with methylphenidate. *J Child Neurol.* 1997;12(4):248-252.

Reason for exclusion: No RCT

Beal1979

- Beal D, Gillis JS. Methylphenidate hydrochloride and judgmental behavior in hyperkinetic children. *Curr Ther Res, Clin Exp.* 1979(6):931-939.

Reason for exclusion: No DSM/ICD criteria as per protocol

Beal1988

- Beal D, Gillis JS. The effect of methylphenidate hydrochloride on interpersonal learning in hyperkinetic children. *Res Commun Psychol, Psychiatr Behav.* 1988;13(4):285-300.

Reason for exclusion: According to NICE, no mention if allocation was randomized; not possible to contact the authors to clarify

Beale1994

- Beale IL, McDowell JP. Effects of methylphenidate on attention in children with moderate mental retardation. *J Dev Phys Disabil.* 1994;6(2):137-148.

Reason for exclusion: Not clear if randomised (not possible to contact authors;) no outcomes of interest for the present meta-analysis

Becker-Mattes1985

- Becker-Mattes A, Mattes JA, Abikoff H, Brandt L. State-dependent learning in hyperactive children receiving methylphenidate. *Am J Psychiatry.* 1985;142(4):455-459.

Reason for exclusion: Less than seven days treatment; No DSM/ICD criteria as per protocol

Bedard2003

- Bedard AC, Ickowicz A, Logan GD, Hogg-Johnson S, Schachar R, Tannock R. Selective inhibition in children with attention-deficit hyperactivity disorder off and on stimulant medication. *J Abnorm Child Psychol.* 2003;31(3):315-327.

Reason for exclusion: Less than seven days treatment

Bedard2004

- Bedard AC, Martinussen R, Ickowicz A, Tannock R. Methylphenidate improves visual-spatial memory in children with attention-deficit/hyperactivity disorder. *J Am Acad Child Adolesc Psychiatry.* 2004; 43(3): 260-268.

Reason for exclusion: Less than seven days treatment

Bedard2007

- Bedard AC, Jain U, Johnson SH, Tannock R. Effects of methylphenidate on working memory components: influence of measurement. *J Child Psychol Psychiatry.* 2007;48(9):872-880.

Reason for exclusion: Less than seven days treatment

Bedard2008

- Bedard AC, Tannock R. Anxiety, methylphenidate response, and working memory in children with ADHD. *J Atten Disord.* 2008;11(5):546-557

Reason for exclusion: Less than seven days treatment

Bedard2015(NCT01709695)

- Bedard AC, Schulz KP, Krone B, et al. Neural mechanisms underlying the therapeutic actions of guanfacine treatment in youth with ADHD: a pilot fMRI study. *Psychiatry Res.* 2015;231(3):353-356
- *https://clinicaltrials.gov/ct2/show/NCT01709695*

Reason for exclusion: No usable data (authors not able to provide additional data)

Beery2013

- Beery SH, Quay HC, Pelham WE, Jr. Differential Response to Methylphenidate in Inattentive and Combined Subtype ADHD. *J Atten Disord.* 2017;21(1):62-7

Reason for exclusion: Concomitant behavioural treatment

Bekker2005

- Bekker EM, Böcker KBE, Van Hunsel F, van der Berg MC, Kenemans, JL. Acute effects of nicotine on attention and response inhibition. *Pharmacol Biochem Behav*. 2005;82(3):539-48.

Reason for exclusion: Not ADHD

Ben-Pazi2006

- Ben-Pazi H, Shalev RS, Gross-Tsur V, Bergman H. Age and medication effects on rhythmic responses in ADHD: possible oscillatory mechanisms? *Neuropsychologia.* 2006;44(3):412-416.

Reason for exclusion: No mention of randomization

Benedetto-Nasho 1999

- Benedetto-Nasho E, Tannock R. Math computation, error patterns and stimulant effects in children with Attention Deficit Hyperactivity Disorder. *J Atten Disord.* 1999;3:121-34.

Reason for exclusion: Less than seven days treatment

Bental2008

- Bental B, Tirosh E. The effects of methylphenidate on word decoding accuracy in boys with attention-deficit/hyperactivity disorder. *J Clin Psychopharmacol.* 2008;28(1):89-92.

Reason for exclusion: Single dose

Berkson1965

- Berkson G. Stereotyped movements of mental defectives: VI. No effect of amphetamine or a barbiturate. *Percept Mot Skills.* 1965;21(3):698.

Reason for exclusion: No DSM/ICD criteria

Berman1999

- Berman T, Douglas VI, Barr RG. Effects of methylphenidate on complex cognitive processing in attention-deficit hyperactivity disorder. *J Abnorm Psychol.* 1999;108(1):90-105.

Reason for exclusion: Experiment 1: Less than seven days treatment; Additionally, no drop pout reported; no other useful outcomes (scale used in the trial not specifc for ADHD symptoms)

Beyer2013

- Beyer Von Morgenstern S, Becker I, Sinzig J. Improvement of facial affect recognition in children and adolescents with attention-deficit/hyperactivity disorder under methylphenidate. *Acta Neuropsychiatrica.* 2013;26(4):202-208.

Reason for exclusion: Less than seven days treatment

Biederman1989

- Biederman J, Baldessarini RJ, Wright V, Knee D, Harmatz JS. A double-blind placebo controlled study of desipramine in the treatment of ADD: I. Efficacy. *J Am Acad Child Adolesc Psychiatry.* 1989;28(5):777-84.
- Biederman J, Baldessarini RJ, Wright V, Knee D, Harmatz JS, Goldblatt A. A double-blind placebo controlled study of desipramine in the treatment ADD: II. Serum drug levels and cardiovascular findings. *J Am Acad Child Adolesc Psychiatry.* 1989;28(6):903-11.
- Biederman J, Baldessarini RJ, Wright V, Keenan K, Faraone S. a double-blind placebo controlled-study of desipramine in the treatment of ADD .3. lack of impact of comorbidity and family history factors on clinical-response. *J Am Acad Child Adolesc Psychiatry.* 1993;32(1):199-204.

Reason for exclusion: Medication of no interest for the present meta-analysis vs. placebo

Biederman2003 (CRIT124D0007)

- Biederman J, Quinn D, Weiss M, et al. Efficacy and safety of Ritalin LA, a new, once daily, extended-release dosage form of methylphenidate, in children with attention deficit hyperactivity disorder. *Paediatr Drugs.* 2003;5(12):833-841.
- Biederman J. Methylphenidate hydrochloride extended release capsules: once-daily therapy for ADHD. 155th Annual Meeting of the American Psychiatric Association 2002.

Reason for exclusion: Participants: responders to previous treatment

Biederman2006a

- Biederman J, Mick E, Faraone S, et al. A double-blind comparison of galantamine hydrogen bromide and placebo in adults with attention-deficit/hyperactivity disorder: a pilot study. *J Clin Psychopharmacol.* 2006;26:163–166.

Reason for exclusion: Medication of no interest for the present meta-analysis vs. placebo, no other arms

Biederman2006b

- Biederman J, Mick E, Spencer T, et al. An open-label trial of OROS methylphenidate in adults with late-onset ADHD. *CNS Spectr.* 2006;11(5):390-396.

Reason for exclusion: No RCT

Biederman2007(NCT00557011)

- Biederman J, Boellner SW, Childress A, Lopez FA, Krishnan S, Mandler H. Improvements in symptoms of attention-deficit/hyperactivity disorder in school-aged children with lisdexamfetamine dimesylate LDX; NRP104 and mixed amphetamine salts extended-release vs. placebo. *J Dev Behav Pediatr.*2006;27(5):442-442
- Biederman J, Boellner SW, Childress A, Lopez FA, Krishnan S, Zhang Y. Lisdexamfetamine dimesylate and mixed amphetamine salts extended-release in children with ADHD: a double-blind, placebo-controlled, crossover analog classroom study. *Biol Psychiatry*. 2007;62(9):970-976.
- Erratum: Biederman. Lisdexamfetamine dimesylate and mixed amphetamine salts extended-release in children with ADHD: A double-blind, placebo-controlled, crossover analog classroom study (vol 62, pg 970, 2007). *Biol Psychiatry.* 2007;62:1334.
- Secondary analysis in: Lopez FA, Scheckner B, Childress AC. Physician perception of clinical improvement in children with attention-deficit/hyperactivity disorder: a post hoc comparison of lisdexamfetamine dimesylate and mixed amphetamine salts extended release in a crossover analog classroom study. *Neuropsychiatr Dis Treat.* 2011;7:267-273.
- https://clinicaltrials.gov/ct2/show/NCT00557011 (additional ID: NRP104-201)
- Additional information from manufacturer

Reason for exclusion: Participants: responders to previous medication; no pre cross-over data

Biederman2010(NCT00181571)

- Biederman J, Mick E, Surman C, et al. A randomized double-blind, placebo-controlled study of OROS methylphenidate in the treatment of adults with attention-deficit/hyperactivity disorder. *Eur Neuropsychopharmacol.* 2005;15(Suppl. 3):S631
- Biederman J, Mick E, Surman C, et al. A randomized, 3-phase, 34-week, double-blind, long-term efficacy study of osmotic-release oral system-methylphenidate in adults with attention-deficit/hyperactivity disorder. *J Clin Psychopharmacol.* 2010;30(5):549-553.
- Biederman J, Mick E. A randomized, three phase 34 week double-blind long-term efficacy study of extended-release methylphenidate in adults with ADHD. *Eur Neuropsychopharmacol.* 2010;20(Suppl. 3):S329-S330.
- Biederman J, Mick E, Fried R, Wilner N, Spencer TJ, Faraone SV. Are stimulants effective in the treatment of executive function deficits? Results from a randomized double-blind study of OROS-methylphenidate in adults with ADHD. *Eur Neuropsychopharmacol*. 2011;21(7):508-515

- Secondary analysis in: Biederman J, Mick E, Spencer T, Surman C, Faraone SV. Is response to OROS-methylphenidate treatment moderated by treatment with antidepressants or psychiatric comorbidity? A secondary analysis from a large randomized double-blind study of adults with ADHD. *CNS Neurosci Ther.* 2012;18(2):126-132.

Reason for exclusion: Dose above maximum dose of the Inclusive analysis
Note: Subsample in Biederman2006a (see list of included papers)

Biederman2014(NCT01533493)
- Biederman J, Fried R, Tarko L, et al. Memantine in the Treatment of Executive Function Deficits in Adults With ADHD: A Pilot-Randomized Double-Blind Controlled Clinical Trial. *J Atten Disord.* 2014;21(4):343-352.
- https://clinicaltrials.gov/ct2/show/NCT01533493

Reason for exclusion: Memantine (of no interest for the present meta-analysis) + methylphenidate (OROS) vs methylphenidate (OROS) +placebo

Bilodeau2014
- Bilodeau M, Simon T, Beauchamp MH, et al. Duloxetine in adults with ADHD: a randomized, placebo-controlled pilot study. *J Atten Disord.* 2014;18(2):169-75.

Reason for exclusion: Medication of no interest for the present meta-analysis vs placebo, no other arms

Blader2009(NCT00228046)
- Blader JC, Schooler NR, Jensen PS, Pliszka SR, Kafantaris V. Adjunctive divalproex versus placebo for children with ADHD and aggression refractory to stimulant monotherapy. *Am J Psychiatry.* 2009;166(12):1392-1401.
- https://clinicaltrials.gov/ct2/show/NCT00228046

Reason for exclusion: Flexibly dosed divalproex or a placebo adjunctive to stimulant

Blix2009
- Blix O, Dalteg A, Nilsson P. Treatment of opioid dependence and ADHD/ADD with opioid maintenance and central stimulants. *Am J Psychiatry.* 2009; 11(1): 5-14

Reason for exclusion: No RCT

Bliznakova2007
- Bliznakova L, Gerstner S, Schmidt MH, Becker K. Methylphenidate double-blind trial: Indication and performing. [German] Der methylphenidat-doppelblindversuch - Indikation und durchfuhrung. *Klin Padiatr.* 2007;219(1):9-16.

Reason for exclusion: Information from Cochrane review (Storebo et al., 2015): N-of-1 trial

Blockmans2006
- Blockmans, D, Persoons, P, Van-Houdenhove, B, Bobbaers, H. Does methylphenidate reduce the symptoms of chronic fatigue syndrome? *Am J Med.* 2006 ;119(2):167.e23-30.

Reason for exclusion: No participants with ADHD

Blum2011(NCT00530257)
- Blum NJ, Jawad AF, Clarke AT, Power TJ. Effect of osmotic-release oral system methylphenidate on different domains of attention and executive functioning in children with attention-deficit-hyperactivity disorder. *Dev Med Child Neurol.* 2011;53(9):843-849.
- https://clinicaltrials.gov/ct2/show/NCT00530257

Reason for exclusion: Enrichment design (participants responders to previous treatment)

Boesen2016
- Boesen K, Gotzsche PC. Quality of Life of Adult Patients With Attention-Deficit/Hyperactivity Disorder Taking Methylphenidate. *JAMA Psychiatry.* 2016;73(5):533-534.

Reason for exclusion: Letter commentary, no empirical data

Boileau1976
- Boileau RA, Ballard JE, Sprague RL, Sleator EK, Massey BH. Effect of methylphenidate on cardiorespiratory responses in hyperactive children. *Research Quarterly.* 1976;47(4):590-596.

Reason for exclusion: No DSM/ICD criteria

Borcherding1989

- Borcherding BG, Keysor CS, Cooper TB, Rapoport JL. Differential effects of methylphenidate and dextroamphetamine on the motor activity level of hyperactive children. *Neuropsychopharmacology.* 1989;2(4):255-263.
- Elia J, Borcherding BG, Potter WZ, Mefford IN, Rapoport JL, Keysor CS. Stimulant drug treatment of hyperactivity: biochemical correlates. *Clin Pharmacol Ther*. 1990;48(1):57-66
- Borcherding BG, Keysor CS, Rapoport JL, Elia J, Amass J. Motor/vocal tics and compulsive behaviors on stimulant drugs: is there a common vulnerability? *Psychiatry Res.*1990;33(1):83-94
- Sharp WS,Walter JM,MarshWL, Ritchie GF,HamburgerSD, Elia J, Borcherding BG, Potter WZ, Mefford IN, Rapoport JL, Keysor CS. Stimulant drug treatment of hyperactivity: biochemical correlates. *Clin Pharmacol Ther.* 1990;48(1):57-66.
- Elia J, Borcherding BG, Rapoport JL, Keysor CS. Methylphenidate and dextroamphetamine treatments of hyperactivity: are there true nonresponders? *Psychiatry Res.* 1991;36(2):141-155.
- Elia J, Welsh PA, Gullotta CS, Rapoport JL. Classroom academic performance: improvement with both methylphenidate and dextroamphetamine in ADHD boys. *J Child Psychol Psychiatry.* 1993;34(5):785-804.
- Schmidt ME, Kruesi MJ, Elia J, et al. Effect of dextroamphetamine and methylphenidate on calcium and magnesium concentration in hyperactive boys. *Psychiatry Res.* 1994;54(2):199-210
- Castellanos FX, Elia J, Kruesi MJP, et al. Cerebrospinal fluid homovanillic acid predicts behavioral response to stimulants in 45 boys with attention deficit hyperactivity disorder. *Neuropsychopharmacology.* 1996;14(2):125-137.
- Castellanos FX, Giedd JN, Elia J, et al. Controlled stimulant treatment of ADHD and comorbid Tourette's syndrome: effects of stimulant and dose. *J Am Acad Child Adolesc Psychiatry.* 1997;36(5):589-596.
- Castellanos FX. ADHD in girls: clinical comparability of a research sample. *J Am Acad Child Adolesc Psychiatry.* 1999;38(1):40–7

Reason for exclusion: Co-treatment (therapeutic art)

Borden1989

- Borden KA, Brown RT. Attributional outcomes: The subtle messages of treatments for attention deficit disorder. *Cognit Ther Res*. 1989(2):147-160.

Reason for exclusion: No arms of interest for the present meta-analysis (cognitive training, methylphenidate+cognitive training, cognitive training + placebo)

Bos2015

- Bos DJ, Oranje B, Veerhoek ES, et al. Reduced Symptoms of Inattention after Dietary Omega-3 Fatty Acid Supplementation in Boys with and without Attention Deficit/Hyperactivity Disorder. *Neuropsychopharmacology.* 2015;40(10):2298-2306.

Reason for exclusion: No arms of interest for the present meta-analysis (margarine daily plus either eicosapentaenoic acid or placebo)

Bostic2000

- Bostic JQ, Biederman J, Spencer TJ, et al. Pemoline treatment of adolescents with attention deficit hyperactivity disorder: A short-term controlled trial. *J Child Adolesc Psychopharmacol.* 2000;10(3):205-216.

Reason for exclusion: Medication of no intersst for the present meta-analysis (pemoline) vs placebo

Bouffard2003

- Bouffard R, Hechtman L, Minde K, Iaboni-Kassab F. The efficacy of 2 different dosages of methylphenidate in treating adults with attention-deficit hyperactivity disorder. *Can J Psychiatry*. 2003;48(8):546-554.
- Additional information/data from study authors

Reason for exclusion: No usable data

Brams2006

- Brams M, Silva R, Childress A, et al. Efficacy and safety of extended-release dexmethylphenidate in children with inattentive subtype ADHD: A 12-hour placebo-controlled laboratory classroom study. *Int J Neuropsychopharmacol.* 2006;9:S229

Reason for exclusion: Participants "stabilized" on methylphenidate before trial

Brams2008(NCT00564954; CRIT124EUS19)

- Brams M, Muniz R, Childress A, et al. A randomized, double-blind, crossover study of once-daily dexmethylphenidate in children with attention-deficit hyperactivity disorder: rapid onset of effect. *CNS Drugs.* 2008;22(8):693-704.
- https://clinicaltrials.gov/ct2/show/NCT00564954

Reason for exclusion: Participants: responders to previous treatment (confirmed by first author) and no wash out between cross-over

Brams2011

- Brams M, Tenorio E, Wang C, Muniz R. Clonidine hydrochloride extended release tablet monotherapy for children and adolescents with Attention Deficit/Hyperactivity Disorder. *Ann Neurol.* 2012;70(15):S143-S144.

Reason for exclusion: After contacting the authors, not possible to retrieve full text; however, excluded since participants: responders to previous treatment

Brams2012a (NCT00776009; CRIT124EUS21)

- Muniz R, Pestreich L, McCague K, Padilla A, Brams M, Childress A. Extended-Release Dexmethylphenidate 30 mg Improves Late-Day Attention Deficit Hyperactivity Disorder (ADHD) Symptom Control in Children with ADHD: A Randomized, Double-Blind Crossover Study. *J Child Adolesc Psychopharmacol.* 2010;20(6):534-535.
- Muniz R, Pestreich L, McCague K, Padilla A, Brams M, Childress A. Extended-release dexmethylphenidate 30 mg improves late-day adhd symptom control in children with adhd: a randomized, double-blind, crossover study. *163rd Annual Meeting of the American Psychiatric Association; 2010 May 22-26; New Orleans, LA.2010.*
- Padilla A, Pestreich L, McCague K, Muniz R. Late-Day Attention Deficit Hyperactivity Disorder (ADHD) Symptom Control Improvement with Extended-Release Dexmethylphenidate in Children with ADHD of All Ethnicities: A Sub-Analysis. *J Child Adolesc Psychopharmacol.* 2010;20(6):534-534.
- Muniz R, Pestreich L, McCague K, Padilla A, Brams M, Childress A. Extended-release dexmethylphenidate 30 mg improves late-day attention deficit hyperactivity disorder (ADHD) symptom control in children with ADHD: a randomized, double-blind crossover study. *163rd Annual Meeting of the American Psychiatric Association; 2010 May 22-26; New Orleans, LA.* 2010.
- Brams M, Turnbow J, Pestreich L, et al. A randomized, double-blind study of 30 versus 20 mg dexmethylphenidate extended-release in children with attention-deficit/hyperactivity disorder: late-day symptom control. *J Clin Psychopharmacol.* 2012;32(5):637-644.
- Erratum: Brams M. A Randomized, Double-Blind Study of 30 Versus 20 mg Dexmethylphenidate Extended-Release in Children With Attention-Deficit/Hyperactivity Disorder: Late-Day Symptom Control. *J Clin Psychopharmacol*. 2012;32(6):766
- No authors. Dexmethylphenidate may be effective later in the day. *The Brown University Child & Adolescent Psychopharmacology Update* 2012;14 (11):1-8.
- Silva RR, Brams M, McCague K, Pestreich L, Muniz R. Extended-release dexmethylphenidate 30 mg/d versus 20 mg/d: duration of attention, behavior, and performance benefits in children with attention-deficit/hyperactivity disorder. *Clin Neuropharmacol.* 2013;36(4):117-121.
- https://clinicaltrials.gov/ct2/show/NCT00776009

Reason for exclusion: Participants: responders to previous treatment

Brams2012b

- Brams M, Weisler R, Findling RL, et al. Maintenance of efficacy of lisdexamfetamine dimesylate in adults with attention-deficit/hyperactivity disorder: randomized withdrawal design. *J Clin Psychiatry.* Jul 2012;73(7):977-983.
- Weisler RH, Babcock T, Adeyi B, Brams M. Relationship of ADHD symptoms and global illness severity in adults treated with lisdexamfetamine dimesylate. *Postgrad Med.* 2014;126(5):31-41.

Reason for exclusion: Participants responders before randomization phase

Breitbart2001

- Breitbart, W, Rosenfeld, B, Kaim, M, Funesti-Esch, J. A randomized, double-blind, placebo-controlled trial of psychostimulantsfor the treatment of fatigue in ambulatory patients with human immunodeficiency virus disease. *Arch Intern Med.* 2001;161(3):411-42.

Reason for exclusion: No participants with ADHD

Broad1982

- Broad J. Assessing Stimulant Treatment of Hyperactivity by Bristol Social Adjustment Guides. *Queen's Univ., Kingston (Ontario).* 1979:13.
- Broad JC. Assessing stimulant treatment of hyperkinesis by Bristol Social Adjustment Guides. *J Psychiatr Treat Eval.* 1982(4):355-358.

Reason for exclusion: DSM-II diagnosis.

Brown1979a

- Brown GL, Hunt RD, Ebert MH, Bunney WE, Jr., Kopin IJ. Plasma levels of d-amphetamine in hyperactive children. Serial behavior and motor responses. *Psychopharmacology (Berl).* 1979;62(2):133-140.
- Brown GL, Ebert MH, Hunt RD, Rapoport JL. Urinary 3-methyoxy-4-hydroxyphenylglycol and homovanillic acid response to d-amphetamine in hyperactive children. *Biol Psychiatry.* 1981;16(8):779-787

Reason for exclusion: No DSM/ICD criteria

Brown1979b
- Brown RT, Sleator EK. Methylphenidate in hyperkinetic children: differences in dose effects on impulsive behavior. *Pediatrics.* 1979;64(4):408-411.

Reason for exclusion: No outcomes of interest; First author replied but no data available anymore

Brown1980
- Brown GL, Ebert MH, Mikkelsen EJ, Hunt RD. Methylphenidate in hyperkinetic children children and plasma amphetamine levels following a sustained release preparation. *J Am Acad Child Psychiatry.* 1980;19(2):225-239.

Reason for exclusion: Less than seven days treatment

Brown1984a
- Brown RT, Slimmer LW, Wynne ME. How much stimulant medication is appropriate for hyperactive school children? *J Sch Health.* 1984;54(3):128-130.

Reason for exclusion: No DSM/ICD criteria as per protocol

Brown1984b
- Brown RT, Wynne ME. Sustained attention in boys with attention deficit disorder and the effect of methylphenidate. *Pediatric Nursing.* 1984;10(1):35-39.

Reason for exclusion: Design pertinent but no outcomes of interest; additional outcomes not available

Brown1984c
- Brown RT, Wynne ME, Slimmer LW. Attention deficit disorder and the effect of methylphenidate on attention, behavioral, and cardiovascular functioning. *J Clin Psychiatry.* 1984;45(11):473-476

Reason for exclusion: Cross-over without wash out; no pre cross-over data available

Brown1985a
- Brown RT, Borden KA, Clingerman SR. Adherence to methylphenidate therapy in a pediatric population: a preliminary investigation. *Psychopharmacol Bull.* 1985;21(1):28-36.

Reason for exclusion: No outcome of interest; First author replied data is no longer available

Brown1985b
- Brown RT, Borden KA, Clingerman SR. Pharmacotherapy in ADD adolescents with special attention to multimodality treatments. *Psychopharmacol Bull.* 1985;21(2):192-211.

Reason for exclusion: No RCT (review)

Brown1985c
- Brown RT, Wynne ME, Medenis R. Methylphenidate and cognitive therapy: A comparison of treatment approaches with hyperactive boys. *J Abnorm Child Psychol.* 1985(1):69-87.

Reason for exclusion: No arms of interest for the present meta-analysis (methylphenidate, cognitive training, cognitive training plus methylphenidate, no treatment)

Brown1986a
- Brown RT, Borden KA, Wynne ME, Schleser R, Clingerman SR. Methylphenidate and cognitive therapy with ADD children: a methodological reconsideration. *J Abnorm Child Psychol.* 1986;14(4):481-497.

Reason for exclusion: No arms of interests for the present meta-analysis: methylphenidate, cognitive training, cognitive training plus methylphenidate, no treatment

Brown1986b
- Brown RT, Wynne ME, Borden KA, Clingerman SR, Geniesse R, Spunt AL. Methylphenidate and cognitive therapy in children with attention deficit disorder: a double-blind trial. *J Dev Behav Pediatr.* 1986;7(3):163-174.

Reason for exclusion: not appropriate design

Brown1987

- Brown RT, Borden KA, Wynne ME. Compliance with pharmacological and cognitive treatments for attention deficit disorder. *J Am Acad Child Adolesc Psychiatry.* 1987;26(4):521-526.

Reason for exclusion: Concurrent additional treatments

Brown1988a

- Brown RT, Borden KA, Wynne ME, Spunt AL, Clingerman SR. Patterns of compliance in a treatment program for children with attention deficit disorder. *J. Compliance Health Care.* 1988(1):23-39.

Reason for exclusion: No pertinent arms for the present meta-analysis ((1) cognitive therapy plus placebo, (2) cognitive therapy plus methylphenidate, (3) methylphenidate plus attention control, or (4) placebo plus attention control)

Brown1988b

- Brown RT, Sexson SB. A controlled trial of methylphenidate in black adolescents. Attentional, behavioral, and physiological effects. *Clin Pediatr (Phila).*1988;27(2):74-81
- Brown RT, Sexson SB. Effects of methylphenidate on cardiovascular responses in attention deficit hyperactivity disordered adolescents. *J Adolesc Health Care*. 1989;10(3):179-183.

Reason for exclusion: Cross-over without wash out; no pre cross-over data

Brown1991

- Brown RT, Jaffe SL, Silverstein J, Magee H. Methylphenidate and hospitalized adolescents with conduct disorder: Dose effects on classroom behavior, academic performance, and impulsivity. *J Youth Adolesc.* 1991;20(5):501-518.

Reason for exclusion: Less than seven days treatment

Broyd2005

- Broyd SJ, Johnstone SJ, Barry RJ, et al. The effect of methylphenidate on response inhibition and the event-related potential of children with attention deficit/hyperactivity disorder. *Int J Psychophysiol.* 2005;58(1):47-58.

Reason for exclusion: No RCT

Bruera2006

- Bruera, E, Valero, V, Driver, L, Shen, L, Willey, J, Zhang, T, Palmer,JL (2006) Patient-controlled methylphenidate for cancer fatigue: adouble-blind, randomized, placebo-controlled trial *J Clin Oncol.* 2006;24(13):2073-8.

Reason for exclusion: No participants with ADHD

Buhrmester1992

- Buhrmester D, Whalen CK, Henker B, MacDonald V, Hinshaw SP. Prosocial behavior in hyperactive boys: effects of stimulant medication and comparison with normal boys. *J Abnorm Child Psychol.* 1992;20(1):103-121.

Reason for exclusion: Less than seven days treatment

Buitelaar1996

- Buitelaar JK, Swaab Barneveld H, Gaag RJ. Prediction of Clinical Response to Methylphenidate in Children with ADHD. *Proceedings of the X World Congress of Psychiatry,* 1996.

Reason for exclusion: Single dose

Buitelaar2006

- Buitelaar JK, Barton J, Danckaerts M, et al. A comparison of North American versus non-North American ADHD study populations. *Eur Child Adolesc Psychiatry.* 2006;15(3):177-181.

Reason for exclusion: No RCT

Bukstein1998

- Bukstein OG, Kolko DJ. Effects of methylphenidate on aggressive urban children with attention deficit hyperactivity disorder. *J Clin Child Psychol.* 1998;27(3):340-351.

Reason for exclusion: Additional behavioral treatment component

Burgio1985

- Burgio L, Page T, Capriotti R. Clinical behavioral pharmacology: methods for evaluating medications and contingency management. *J Appl Behav Anal.* 1985;18(1):45-59

Reason for exclusion: No RCT

Bush2004

- Bush G, Spencer TJ, Surman C, et al. Functional MRI of two classes of ADHD therapy (methylphenidate and galantamine) versus placebo. *Biol Psychiatry.* 2004;55:195S-S.

Reason for exclusion: *No mention of randomization; Author confirmed that no additional publication in full text is available*

Butter1975
- Butter HJ, Lapierre YD. The effect of methylphenidate on cardiovascular sensory differentiation on the hyperkinetic syndrome. *Int J Clin Pharmacol Biopharm.* 1975;11(4):309-314.

Reason for exclusion: No diagnostic criteria as per protocol

Butter1983
- Butter HJ, Lapierre Y, Firestone P, Blank A. A comparative study of the efficacy of ACTH4-9 analog, methylphenidate, and placebo on attention deficit disorder with hyperkinesis. *J Clin Psychopharmacol.* 1983;3(4):226-230
- Butter HJ, Lapierre Y, Firestone P, Blank A. Efficacy of ACTH 4-9 analog, methylphenidate, and placebo on attention deficit disorder with hyperkinesis. *Prog Neuropsychopharmacol Biol Psychiatry.* 1984;8(4-6):661-664.

Reason for exclusion: No diagnostic criteria as per protocol; no scales on ADHD core symptoms

Byrne1998
- Byrne JM, Bawden HN, DeWolfe NA, Beattie TL. Clinical assessment of psychopharmacological treatment of preschoolers with ADHD. *J Clin Experim Child Neuropsychol.* 1998;20(5):613-627.

Reason for exclusion: Preschoolers; No RCT

Caballero2003
- Caballero J, Nahata MC. Atomoxetine hydrochloride for the treatment of attention-deficit/hyperactivity disorder. *Clin Ther.* 2003;25(12):3065-3083.

Reason for exclusion: Review

Campell1971
- Campbell SB, Douglas VI, Morgenstern G. Cognitive styles in hyperactive children and the effect of methylphenidate. *J Child Psychol Psychiatry.* 1971;12(1):55-67

Reason for exclusion: No DSM/ICD diagnosis

Carlson1991
- Carlson CL, Pelham WE, Jr., Swanson JM, Wagner JL. A divided attention analysis of the effects of methylphenidate on the arithmetic performance of children with attention-deficit hyperactivity disorder. *J Child Psychol Psychiatry.* 1991;32(3):463-471.

Reason for exclusion: Single dose (placebo)

Carlson 1992
- Carlson GA, Rapport MD, Kelly KL, Pataki CS. The effects of methylphenidate and lithium on attention and activity level. *J Am Acad Child Adolesc Psychiatry.* 1992;31(2):262-270.

Reason for exclusion: Seven participants children, not all with ADHD

Carlson1992
- Carlson CL, Pelham WE, Milich R, Dixon J. Single and combined effects of methylphenidate and behavior-therapy on the classroom performance of children with attention-deficit hyperactivity disorder. *J Abnorm Child Psychol.* 1992;20(2):213-232.

Reason for exclusion: Less than seven consecutive days of treatment

Carlson1993
- Carlson CL, Pelham WE, Milich R, Hoza B. ADHD boys' performance and attributions following success and failure: drug effects and individual differences. *Cognit Ther Res.* 1993;17(3):269-287.

Reason for exclusion: Less than seven consecutive days of treatment

Carlson1995
- Pataki CS, Carlson GA, Kelly KL, Rapport MD, Biancaniello, TM. Side effects of methylphenidate and desipramine alone and in combination in children. *J Am Acad Child Adolesc Psychiatry*. 32(5), 1065-1072

- Rapport MD, Carlson GA, Kelly KL, Pataki C. methylphenidate and desipramine in hospitalized children .1. separate and combined effects on cognitive function. *J Am Acad Child Adolesc Psychiatry.* 1993;32(2):333-342
- Carlson GA, Rapport MD, Kelly KL, Pataki CS. Methylphenidate and desipramine in hospitalized children with comorbid behavior and mood disorders: Separate and combined effects on behavior and mood. J *Child Adolesc Psychopharmacol.*1995(3):191-204

Reason for exclusion: Cross-over without wash out, no pre cross-over data available

Carlson2007

- Carlson GA, Dunn D, Kelsey D, et al. A pilot study for augmenting atomoxetine with methylphenidate: safety of concomitant therapy in children with attention-deficit/hyperactivity disorder. *Child Adolesc Psychiatry Ment Health.* 2007;1(1):10.

Reason for exclusion: Phase 1: not randomized; phase 2: responders: Assigned to co-treatment Mmethylphenidate-atomoxetine or methylphenidate-placebo

Carpentier2005

- Carpentier PJ, de Jong CA, Dijkstra BA, Verbrugge CA, Krabbe PF. A controlled trial of methylphenidate in adults with attention deficit/hyperactivity disorder and substance use disorders. *Addiction.* 2005;100(12):1868-1874.

Reason for exclusion: Co-treatment during trial

Casat1995

- Casat CD, Pearson DA, Van Davelaar MJ, Cherek DR. Methylphenidate effects on a laboratory aggression measure in children with ADHD. *Psychopharmacol Bull.* 1995;31(2):353-356.

Reason for exclusion: Less than seven days treatment

Casey2014

- Casey BJ, Durston S. The impact of stimulants on cognition and the brain in attention-deficit/hyperactivity disorder: what does age have to do with it? *Biol Psychiatry.* 2014;76(8):596-598.

Reason for exclusion: Commentary- no empirical data

Castaneda2000

- Castaneda R, Levy R, Hardy M, Trujillo M. Long-acting stimulants for the treatment of attention-deficit disorder in cocaine-dependent adults. *Psychiatric Services.* 2000;51(2):169-171.

Reason for exclusion: Review/commentary, no empirical data

Cetin2013

- Cetin FH, Taner YI, Torun YT, Tunca H. Atomoxetine and methylphenidate for the treatment of attention deficit hyperactivity disorder: A six-month follow-up study. *Klinik Psikofarmakol Bulteni.* 2013;23:S80.

Reason for exclusion: Abstract only available; not possible to contact authors (no email address) to ask if full text available and/or query re: inclusion criteria

Chacko2005

- Chacko A, Pelham WE, Gnagy EM, et al. Stimulant medication effects in a summer treatment program among young children with attention-deficit/hyperactivity disorder. *J Am Acad Child Adolesc Psychiatry.* 2005;44(3):249-257.

Reason for exclusion: Less than seven days treatment

Chappel1995

- Chappell PB, Riddle MA, Scahill L, et al. Guanfacine treatment of comorbid attention-deficit hyperactivity disorder and Tourette's syndrome: preliminary clinical experience. *J Am Acad Child Adolesc Psychiatry.* 1995;34(9):1140-1146.

Reason for exclusion: Open label

Chatoor1983

- Chatoor I, Wells KC, Conners CK. The effects of nocturnally administered stimulant medication on EEG sleep and behavior in hyperactive children. *J Am Acad Child Psychiatr.* 1983(4):337-342.

Reason for exclusion: Less than seven days treatment; not appropriate design

Chen2012

- Chen YH, Lin XX, Chen H, et al. Letter to the Editor: The change of the cortisol levels in children with ADHD treated by methylphenidate or atomoxetine. *J Psychiatr Res.* 2012(3):415-416.

Reason for exclusion: Letter to the editor. No additional information form authors on the trial mentioned in the letter

Chen2014

- Chen TH, Wu SW, Welge JA, et al. Reduced short interval cortical inhibition correlates with atomoxetine response in children with attention-deficit hyperactivity disorder (ADHD). *J Child Neurol.* 2014;29(12):1672-1679.

Reason for exclusion: Open label not controlled study

Cherkasova2014

- Cherkasova MV, Faridi N, Casey KF, et al. Amphetamine-induced dopamine release and neurocognitive function in treatment-naive adults with ADHD. *Neuropsychopharmacol.* 2014;39(6):1498-1507.

Reason for exclusion: Single dose.

ChiCTR-INR-17011042

- http://www.chictr.org.cn/showproj.aspx?proj=18724

Reasons for exclusion: Compound of no interest for the present meta-analysis (JingNing granule) vs atomoxetine

ChiCTR-TRC-10001127

- http://www.chictr.org/en/proj/show.aspx?proj=278

Reasons for exclusion: Interventions not pertinent for the present meta-analysis (methylphenidate +behavioural intervention+physical activity vs methylphenidate +behavioural intervention)

Childress2006

- Childress A, Silva R, Brams M, et al. Efficacy and safety of extended-release dexmethylphenidate in children with ADHD: A 12-hour placebo-controlled laboratory classroom study. *Int J Neuropsychopharmacol.* 2006;9:S229-S30.

Reason for exclusion: Participants responders to previous stimulants

Childress2015 (NCT01986062)

- Childress AC, Brams M, Cutler AJ, et al. The Efficacy and Safety of Evekeo, Racemic Amphetamine Sulfate, for Treatment of Attention-Deficit/Hyperactivity Disorder Symptoms: A Multicenter, Dose-Optimized, Double-Blind, Randomized, Placebo-Controlled Crossover Laboratory Classroom Study. *J Child Adolesc Psychopharmacol.* 2015;25(5):402-414.
- https://clinicaltrials.gov/ct2/show/NCT01986062

Reason for exclusion: Authors confirmed that participants had to be responders to be recruited in the study

Childress2016 (NCT01835548)

- Childress AC, Kollins SH, Cutler AJ, Marraffino A, Sikes CR. Efficacy, Safety, and Tolerability of an Extended-Release Orally Disintegrating Methylphenidate Tablet in Children 6-12 Years of Age with Attention-Deficit/Hyperactivity Disorder in the Laboratory Classroom Setting.
  J Child Adolesc Psychopharmacol. 2017;27(1):66-74
- https://clinicaltrials.gov/ct2/show/NCT01835548

Reason for exclusion: Authors confirmed that: "subjects had to have at least a 30% response from baseline on no medication (Visit 2) to be considered optimized. Subjects could be titrated to a higher dose to further improve symptoms at the discretion of the investigator".

Choi2015

- Choi ES, Lee WK. Comparative effects of emotion management training and social skills training in Korean children with ADHD. *J Atten Disord.* 2015;19(2):138-46

Reason for exclusion: No arms of interest for the present meta-analysis (methylphenidate + exercise vs methylphenidate + education)

Chou2012

- Chou WJ, Chen SJ, Chen Y-S, et al. Remission in children and adolescents diagnosed with attention-deficit/hyperactivity disorder via an effective and tolerable titration scheme for osmotic release oral system methylphenidate. *J Child Adolesc Psychopharmacol.* 2012;22(3):215-225.

Reason for exclusion: No RCT

Chou2015

- Chou TL, Chia S, Shang CY, Gau SS. Differential therapeutic effects of 12-week treatment of atomoxetine and methylphenidate on drug-naive children with attention deficit/hyperactivity disorder: A counting Stroop functional MRI study. *Eur Neuropsychopharmacol.* 2015;25(12):2300-2310.

Reason for exclusion: Open label study

Christ2013

- Christ W, Mayer H, Wiemer-Kruel A. Methylphenidate therapy for children with epilepsy. Results of a double blinded observational study. *Monatsschr Kinderheilkd.* 2013;161(8):720-726.

Reason for exclusion: Not randomization

Chronis-Tuscano2008(NCT00318981)

- Chronis-Tuscano A, Seymour KE, Stein MA, et al. Efficacy of osmotic-release oral system (OROS) methylphenidate for mothers with attention-deficit/hyperactivity disorder (ADHD): preliminary report of effects on ADHD symptoms and parenting. *J Clin Psychiatry*. 2008;69(12):1938-1947.
- https://clinicaltrials.gov/ct2/show/NCT00318981

Reason for exclusion: first phase not randomized; not possible to gather additional information from authors

Coghill2007

- Rhodes SM, Thrower M, Brown A, Esperon J, Coghill DR, Matthews K. Acute neuropsychological effects of the psychostimulant Methylphenidate in drug naive boys with Hyperkinetic Disorder (ADHD*). Society for Neuroscience Abstracts*. 2001;27(2):2341.
- Coghill DR, Rhodes SM, Matthews K. Chronic effects of the psychostimulant drug methylphenidate on neuropsychological functioning in drug-naïve boys with hyperkinetic disorder (ADHD). *J Psychopharmacol*. 2003;17 (3):A74.
- Rhodes SM, Coghill DR, Matthews K. Chronic neuropsychological effects of the psychostimulant drug methylphenidate in drug-naive boys with hyperkinetic disorder (ADHD). *Society for Neuroscience Abstracts.* 2003;Abstract No. 619.617.
- Rhodes SM, Coghill DR, Matthews K. Methylphenidate restores visual memory, but not working memory function in attention deficit-hyperkinetic disorder. *Psychopharmacology (Berl)*. 2004;175(3):319-330
- Rhodes SM, Coghill DR, Matthews K. Acute neuropsychological effects of methylphenidate in stimulant drug-naive boys with ADHD II--broader executive and non-executive domains. *J Child Psychol Psychiatry*. 2006;47(11):1184-1194
- Coghill DR, Rhodes SM, Matthews K. The neuropsychological effects of chronic methylphenidate on drug-naive boys with attention-deficit/hyperactivity disorder. *Biol Psychiatry*. 2007;62(9):954-962.

Reason for exclusion: Cross-over without wash out, no pre cross-over data

Cohen1987

- Cohen DJ, Ort S, Caruso KA, et al. Parotid gland salivary secretion in Tourette's syndrome and attention deficit disorder: a model system for the study of neurochemical regulation. *J Am Acad Child Adolesc Psychiatry.* 1987;26(1):65-68.

Reason for exclusion: Single dose

Cohen1989

- Cohen ML, Kelly PC, Atkinson AW. Parent, teacher, child. A trilateral approach to attention deficit disorder. *Am J Dis Child* (1960). 1989;143(10):1229-1233.
- Kelly PC, Cohen ML, Walker RO, et al. Self-esteem in children medically managed for attention deficit disorder. *Pediatrics*. 1989;83:211-217.

Reason: No information on randomization; not possible to contact the authors

Coleman1979

- Coleman M, Steinberg G, Tippett J, et al. A preliminary study of the effect of pyridoxine administration in a subgroup of hyperkinetic children: a double-blind crossover comparison with methylphenidate. *Biol Psychiatry.* 1979;14(5):741-751.

Reason for exclusion: DSM-II

Collins2006

- Collins SL, Levin FR, Foltin RW, Kleber HD, Evans SM. Response to cocaine, alone and in combination with methylphenidate, in cocaine abusers with ADHD. *Drug Alcohol Depend.* 2006;82(2):158-167.

Reason for exclusion: No RCT

Comly1971

- Comly HH. Cerebral stimulants for children with learning disorders? *J Learn Disabil.* 1971;4(9):484–490.

Reason for exclusion: No RCT

Conners1967

- Conners CK, Eisenberg L, Barcai A. Effect of dextroamphetamineon children. Studies on subjects with learning disabilities andschool behavior problems. *Arch of Gen Psychiatry.* 1967;17(4):478–85.

Reason for exclusion: No DSM/ICD criteria

Conners1969

- Conners CK, Rothschild G, Eisenberg L, Schwartz LS, RobinsonE. Dextroamphetamine sulfate in children with learning disorders. Effects on perception, learning, and achievement. *Arch of Gen Psychiatry.* 1969;21(2):182–90.

Reason for exclusion: no usable data; not possible to contact author

Conners1971

- Conners CK. The effect of stimulant drugs on human figure drawings in children with minimal brain dysfunction. *Psychopharmacologia.*1971;19(4):329-33

Reason for exclusion: No DSM/ICD criteria

Conners1972a

- Conners CK. Symposium: behavior modification by drugs. II. Psychological effects of stimulant drugs in children with minimal brain dysfunction. *Pediatrics.*1972;49(5):702-708.

Reason for exclusion: No DSM/ICD criteria

Conners1972b

- Conners CK, Taylor E, Meo G, Kurtz MA, Fournier M. Magnesium pemoline and dextroamphetamine: a controlled study in children with minimal brain dysfunction. *Psychopharmacologia.* 1972;26(4):321-336

Reason for exclusion: No DSM/ICD criteria

Conners1975

- Conners CK. Controlled trial of methylphenidate in preschool children with minimal brain dysfunction. *Int J Ment Health*. 1975;4: 61-74

Reason for exclusion: Pre-schoolers, No DSM/ICD criteria

Conners1980

- Conners CK, Taylor E. Pemoline, methylphenidate, and placebo in children with minimal brain dysfunction. *Arch Gen Psychiatry.* 1980;37(8):922-930.

Reason for exclusion: No DSM/ICD criteria

Conners1996a

- Conners CK, Levin ED, Sparrow E, et al. Nicotine and attention in adult attention deficit hyperactivity disorder (ADHD). *Psychopharmacol Bull.* 1996;32(1):67-73.

Reason for exclusion: Transdermal formulations

Conners1996b

- Conners CK, Casat CD, Gualtieri CT, et al. Bupropion hydrochloride in attention deficit disorder with hyperactivity. *J Am Acad Child Adolesc Psychiatry*. 1996;35(10):1314-1321.

Reason for exclusion: No usable data; not possible to contact author

Conrad1971

- Conrad W, Dworkin E, Shai A, Tobiessen J. Effects of amphetamine therapy and prescriptive tutoring on the behavior and achievement of lower class hyperactive children. *J Learn Disabil.* 1971;4(9):509-517.

Reason for exclusion: No DSM/ICD criteria as per protocol

Conzelmann2016

- Conzelmann A, Woidich E, Mucha RF, et al. Methylphenidate and emotional-motivational processing in attention-deficit/hyperactivity disorder. *J Neural Transm*. 2016;123(8):971-9.

Reason for exclusion: Single dose

Corkum2008

- Corkum P, Panton R, Ironside S, Macpherson M, Williams T. Acute impact of immediate release methylphenidate administered three times a day on sleep in children with attention-deficit/hyperactivity disorder. *J Pediatr Psychol.* 2008;33(4):368-379
- Ironside S, Davidson F, Corkum P. Circadian motor activity affected by stimulant medication in children with attention-deficit/hyperactivity disorder. *J Sleep Res.* 2010;19(4):546-551.

Reason for exclusion: Cross-over without wash out; no pre cross-over data available

Cotton1988
- Cotton MF, Rothberg AD. Methylphenidate v. placebo - a randomised double-blind crossover study in children with the attention deficit disorder. *S Afr Med J.* 1988;74(6):268-271.

Reason for exclusion: Participants: responders, no DSM/ICD criteria

Cox2000
- Cox DJ, Merkel RL, Kovatchev B, Seward R. Effect of stimulant medication on driving performance of young adults with attention-deficit hyperactivity disorder: a preliminary double-blind placebo controlled trial. *J Nerv Ment Dis.* 2000;188(4):230-234.

Reason for exclusion: Single dose

Cox2004a
- Cox DJ, Humphrey JW, Merkel RL, Penberthy JK, Kovatchev B. Controlled-release methylphenidate improves attention during on-road driving by adolescents with attention-deficit/hyperactivity disorder. *J Am Board Fam Pract.* 2004;17(4):235-239.

Reason for exclusion: Single dose

Cox2004b
- Cox DJ, Penberthy JK, Merkel RL, Kovatchev B. Driving performance among adolescents with attention-deficit/hyperactivity disorder: medication effects. *Eur Neuropsychopharmacol.* 2002:S415.
- Cox DJ, Merkel RL, Penberthy JK, Kovatchev B, Hankin CS. Impact of methylphenidate delivery profiles on driving performance of adolescents with attention-deficit/hyperactivity disorder: a pilot study. *J Am Acad Child Adolesc Psychiatry.* 2004;43(3):269-275.
- Cox DJ, Humphrey JW, Merkel RL, Penberthy JK, Kovatchev B. Impact of OROS (R) methylphenidate on real-life driving performance of adolescents with ADHD. *Pediatr Res*. 2004;55:2A.

Reason for exclusion: Single blind

Cox2006
- Cox DJ, Merkel RL, Moore M, Thorndike F, Muller C, Kovatchev B. Relative benefits of stimulant therapy with OROS methylphenidate versus mixed amphetamine salts extended release in improving the driving performance of adolescent drivers with attention-deficit/hyperactivity disorder. *Pediatrics.* 2006;118(3):e704-710.
- Post hoc analysis in: Wilson HK, Cox DJ, Merkel RL, Moore M, Coghill D. Effect of extended release stimulant-based medications on neuropsychological functioning among adolescents with Attention-Deficit/Hyperactivity Disorder. *Arch Clin Neuropsychol.* 2006;21(8):797-807.
- Cox DJ, Moore M, Burket R, Merkel RL, Mikami AY, Kovatchev B. Rebound effects with long-acting amphetamine or methylphenidate stimulant medication preparations among adolescent male drivers with attention-deficit/hyperactivity disorder. *J Child Adolesc Psychopharmacol.* 2008;18(1):1-10.
- Mikami AY, Cox DJ, Davis MT, Wilson HK, Merkel RL, Burket R. Sex differences in effectiveness of extended-release stimulant medication among adolescents with attention-deficit/hyperactivity disorder. *J Clin Psychol Med Settings.* 2009;16(3):233-242.

Reason for exclusion: Participants included if responsive to stimulants

Cox2008
- Cox DJ, Mikami AY, Cox BS, et al. Effect of long-acting OROS methylphenidate on routine driving in young adults with attention-deficit/hyperactivity disorder. *Arch Pediatr Adolesc Med.* 2008;162(8):793-794.

Reason for exclusion: No RCT

CRIT124EUS09
- https://docslide.com.br/documents/dexmethylphenidate-extended-release-capsules-in-children-with-attention-deficithyperactivity.html

Reason for exclusion: Less than 7 days treatment (refers to Silva RR, Muniz R, Pestreich L, et al. Dexmethylphenidate extended-release capsules in children with attention-deficit/hyperactivity disorder. J Am Acad Child Adolesc Psychiatry. 2008;47(2):199-208., discarded based on the abstract)

CTRI/2017/01/007665

- http://www.ctri.nic.in/Clinicaltrials/pmaindet2.php?trialid=14324

Reasons for exclusion: Atomoxetine vs psychological intervention vs combination

CTRI/2017/02/007888

- http://www.ctri.nic.in/Clinicaltrials/pmaindet2.php?trialid=16335

Reasons for exclusion: No RCT

CTRI/2015/06/005853

- http://www.ctri.nic.in/Clinicaltrials/pmaindet2.php?trialid=3415

Reasons for exclusion: Open label

Cunningham1985

- Cunningham CE, Siegel LS, Offord DR. A developmental dose-response analysis of the effects of methylphenidate on the peer interactions of attention deficit disordered boys. *J Child Psychol Psychiatry.* 1985;26(6):955-971.
- Related to Cunningham CE, Siegel LS, Offord DR. A dose-response analysis of the effects of methylphenidate on the peer interactions and simulated classroom performance of ADD children with and without conduct problems. *J Child Psychol Psychiatry.* 1991;32(3):439-452.

Reason for exclusion: Less than seven days treatment

Cutler2010

- Cutler A, Pestreich L, McCague K, Muniz R. Extended-release dexmethylphenidate improves permp math test performance throughout the laboratory-classroom day in children with adhd. 163rd Annual Meeting of the American Psychiatric Association; 2010 May 22-26; New Orleans, LA2010.

Reason for exclusion: Participants "stabilized" on methylphenidate; authors confirmed this means that participants were "responders"

Cutler2011

- Cutler A, Tenorio E, Wang C, Muniz R. Clonidine extended release tablets for the treatment of ADHD in children and adolescents with inadequate response to stimulants. *Ann Neurol.* 2011;70:S143.

Reason for exclusion: Participants "stabilized" on methylphenidate; authors confirmed this means that participants were "responders"

Daly2012

- Daly B, Kral MC, Brown RT, et al. Ameliorating Attention Problems in Children With Sickle Cell Disease: A Pilot Study of Methylphenidate. *J Dev Behav Pediatr.*2012;33(3):244-251.

Reason for exclusion: Comorbidity with rare inherited condition

Dashti2014 (IRCT201304035393N3)

- Dashti N, Hekmat H, Soltani HR, Rahimdel A, Javaherchian M. Comparison of therapeutic effects of omega-3 and methylphenidate (ritalin) in treating children with attention deficit hyperactivity disorder. *Iran J Psychiatry & Behav Sci.* 2014;8(4):7-11.
- http://www.irct.ir/searchresult.php?id=5393&number=3

Reason for exclusion: From the paper there is no evidence of a formal diagnosis of ADHD; authors contacted but no reply

Davari-Ashtiani2010 (IRCT138901292000N3)

- Davari-Ashtiani R, Shahrbabaki ME, Razjouyan K, Amini H, Mazhabdar H. Buspirone versus methylphenidate in the treatment of attention deficit hyperactivity disorder: a double-blind and randomized trial. *Child Psychiatry Hum Dev.* 2010;41(6):641-648.
- http://www.irct.ir/searchresult.php?id=2000&number=3

Reason for exclusion: Medication of no interest for the present meta-analysis (buspirone) vs. placebo, no other arms

Davidovitch1999

- Davidovitch M, Manning-Courtney P, Hartmann LA, Watson J, Lutkenhoff M, Oppenheimer S. The prevalence of attentional problems and the effect of methylphenidate in children with myelomenigocele. *Pediatr Rehabil.* 1999;3(1):29-35.

Reason for exclusion: No diagnosis of ADHD according to the full criteria (not stated that all DSM criteria were met). Comorbid with a rare inherited condition.

Daviss2001

- Daviss WB, Bentivoglio P, Racusin R, Brown KM, Bostic JQ, Wiley L. Bupropion sustained release in adolescents with comorbid attention-deficit/hyperactivity disorder and depression. *J Am Acad Child Adolesc Psychiatry.* 2001;40(3):307-314.

Reason for exclusion: No RCT

De Bruyckere2016

- De Bruyckere K, Bushe C, Bartel C, et al. Effects of atomoxetine on functional outcomes, and correlation with the core symptoms of Attention-Deficit/Hyperactivity Disorder in adult patients. *ADHD Atten Defic Hyperact Disord.* 2015;7:S49.
- De Bruyckere K, Bushe C, Bartel C, Berggren L, Kan CC, Dittmann RW. Relationships Between Functional Outcomes and Symptomatic Improvement in Atomoxetine-Treated Adult Patients with Attention-Deficit/Hyperactivity Disorder: Post Hoc Analysis of an Integrated Database. *CNS Drugs.* 2016;30(6):541-58.

Reason for exclusion: Pooled analysis of 7 studies (LYAA, LYAO, LYBV (NCT00190931), LYCE (NCT00190736), LYDQ (NCT00190879), LYDZ (NCT00510276), LYEE (NCT00962104) all retrieved in our search

De Jong2009 (B4Z-MC-LYCK (7955); NCT00191906)

- de Jong CG, Van De Voorde S, Roeyers H, et al. Differential effects of atomoxetine on executive functioning and lexical decision in attention-deficit/hyperactivity disorder and reading disorder. *J Child Adolesc Psychopharmacol.* 2009;19(6):699-707.
- de Jong CG, Van De Voorde S, Roeyers H, Raymaekers R, Oosterlaan J, Sergeant JA. How distinctive are ADHD and RD? Results of a double dissociation study. *J Abnorm Child Psychol.* 2009 ;37(7):1007-17.
- https://assets.contentful.com/hadumfdtzsru/6tNU9lDUFak00UYysgcgcK/0daabb317c1833bdbf898689b376c9f9/Atomoxetine-B4Z-MC-LYCK.pdf
- https://www.accessdata.fda.gov/drugsatfda_docs/nda/2002/21-411_Strattera.cfm
- Additional information from first author

Reason for exclusion: No usable data

De Sonneville 1991

- de Sonneville LM, Njiokiktjien C, Hilhorst RC. Methylphenidate-induced changes in ADDH information processors. *J Child Psychol Psychiatry.* 1991;32(2):285-295.

Reason for exclusion: Participants: responders

De Sonneville1994

- de Sonneville LM, Njiokiktjien C, Bos H. Methylphenidate and information processing. Part 1: Differentiation between responders and nonresponders; Part 2: Efficacy in responders. *J Clin Exp Neuropsychol.* 1994;16(6):877-897.

Reason for exclusion: No mention of DSM criteria, no pre cross-over data available (not possible to contact the authors)

Demirci2016

- Demirci E, Erdogan A. Is emotion recognition the only problem in ADHD? effects of pharmacotherapy on face and emotion recognition in children with ADHD. *Atten Defic Hyperact Disord.* 2016 ;8(4):197-204

Reason for exclusion: No double blind

Denhoff1971

- Denhoff E. Effects of Dextroamphetamine on Hyperkinetic Children: A Controlled Double Blind Study. *J Learn Disabil.* 1971(9):491-498.
- Denhoff E, Davids A, Hawkins R. Effects of dextroamphetamine on hyperkinetic children; a controlled double-blind study. *J Learn Disabil.* 1971;4 (9): 27-34

Reason for exclusion: No DSM/ICD criteria

Devitto2009

- DeVito EE, Blackwell AD, Kent L, et al. The effects of methylphenidate on decision making in attention-deficit/hyperactivity disorder. *Biol Psychiatry.* 2008;64(7):636-639.
- DeVito EE, Blackwell AD, Clark L, et al. Methylphenidate improves response inhibition but not reflection-impulsivity in children with attention deficit hyperactivity disorder (ADHD). *Psychopharmacology (Berl).* 2009;202(1-3):531-539.
- DeVito EE, Sahakian BJ. Response to comments on 'Methylphenidate improves response inhibition but notreflection impulsivity in children with attention deficithyperactivity disorder (ADHD)'. *Psychopharmacology (Berl).* 2009 203(1):187.

Reason for exclusion: Single dose

Di Traglia1991

- DiTraglia J. Methylphenidate protocol: feasibility in a pediatric practice. *Clin Pediatr (Phila).* 1991;30(12):656-660.

Reason for exclusion: No DSM/ICD criteria

Diamond1999

- Diamond IR, Tannock R, Schachar RJ. Response to methylphenidate in children with ADHD and comorbid anxiety. *J Am Acad Child Adolesc Psychiatry.* 1999;38(4):402-409.

Reason for exclusion: Co-intervention

Dickson2007a (NCT00191633)

- Dickson RA, Jackiewicz G, Khattak S, Gilchrist W, Szombathy S, Brunner E: Change in ADHD symptoms and functional outcomes in Canadian children during 3 months of atomoxetine treatment. *Presented at the 27th Annual Conference of the Canadian Academy of Child and Adolescent Psychiatry, Montréal, Québec 2007*
- Pooled in: Dickson RA, Maki E, Gibbins C, Gutkin SW, Turgay A, Weiss MD. Time courses of improvement and symptom remission in children treated with atomoxetine for attention-deficit/hyperactivity disorder: analysis of Canadian open-label studies. *Child Adolesc Psychiatry Ment Health.* 2011;5:14. [pooled with NCT00216918 and NCT00191880 (B4Z-CA-LYCS))
- https://clinicaltrials.gov/ct2/show/NCT00191633

Reason for exclusion: Open label

Dickson2007b (NCT00191880)

- Dickson R, Lee B, Turgay A, Chang S, White H, Davis L, Wasdell M, Yoshioka A, Weiss M: Atomoxetine treatment of ADHD: symptomatic, academic, cognitive, and functional outcomes. *Presented at the American Academy of Child and Adolescent Psychiatry, 54th Annual Meeting, Boston, MA 2007*
- Pooled in: Dickson RA, Maki E, Gibbins C, Gutkin SW, Turgay A, Weiss MD. Time courses of improvement and symptom remission in children treated with atomoxetine for attention-deficit/hyperactivity disorder: analysis of Canadian open-label studies. *Child Adolesc Psychiatry Ment Health.* 2011;5:14.
- https://clinicaltrials.gov/ct2/show/NCT00191880

Reason for exclusion: Open label

Dittmann2013 (NCT01106430; SPD489-317;EUCTR2009-011745-94-GB)

- Cardo E, Coghill D, Nagy P, et al. Efficacy of lisdexamfetamine dimesylate and atomoxetine in children and adolescents with ADHD: Head-to-head responder analyses. *Eur Neuropsychopharmacol*. 2013;23:S603-S4.
- Dittmann RW, Cardo E, Nagy P, et al. Efficacy and safety of lisdexamfetamine dimesylate and atomoxetine in the treatment of attention-deficit/hyperactivity disorder: a head-to-head, randomized, double-blind, phase IIIb study. *CNS Drugs.* 2013;27(12):1081-1092
- Dittmann R, Cardo E, Coghill D, et al. A head-to-head, double-blind, randomized, phase 3b trial comparing the efficacy of lisdexamfetamine dimesylate with atomoxetine for the treatment of children and adolescents with attention-deficit/ hyperactivity disorder. *Eur Child Adolesc Psychiatry*. 2013;1):S222-S3.
- Dittmann RW, Cardo E, Coghill DR, et al. Efficacy of Lisdexamfetamine Dimesylate and Atomoxetine in Child and Adolescent Subgroups from a Head-to-Head, Double-Blind, Randomized Trial in Patients with Attention-Deficit/Hyperactivity Disorder. *Eur Psychiatry.* 2014;29.
- Dittmann RW, Cardo E, Nagy P, et al. Treatment response and remission in a double-blind, randomized, head-to-head study of lisdexamfetamine dimesylate and atomoxetine in children and adolescents with attention-deficit hyperactivity disorder. *CNS Drugs.* 2014;28(11):1059-1069.
- Banaschewski T, Rothermel B, Poustka L. Evaluation of a head-to-head study of lisdexamfetamine dimesylate and atomoxetine: evaluation of Dittmann RW, Cardo E, Nagy P, et al. Efficacy and safety of lisdexamfetamine dimesylate and atomoxetine in the treatment of attention-deficit/hyperactivity disorder: a head-to-head, randomised,

double-blind, Phase IIIb study. *CNS Drugs*. 2013;27:1081-1092. doi: 10.1007/s40263-013-0104-8 *Expert Opin Pharmacother.* 2014;15(13):1961-1965.
- Nagy P, Hage A, Coghill DR, et al. Functional outcomes from a head-to-head, randomized, double-blind trial of lisdexamfetamine dimesylate and atomoxetine in children and adolescents with attention-deficit/hyperactivity disorder and an inadequate response to methylphenidate. *Eur Child Adolesc Psychiatry.* 2016;25(2):141-149
- https://clinicaltrials.gov/ct2/show/NCT01106430
- https://www.clinicaltrialsregister.eu/ctr-search/search?query=eudract_number:2009-011745-94

Reason for exclusion: Participants resistant to methylphenidate

Dodson2005
- Dodson WW. Pharmacotherapy of adult ADHD. *J Clin Psychol.* 2005;61(5):589-606.

Reason for exclusion: Review, no empirical data

Donnelly1986
- Donnelly M, Zametkin AJ, Rapoport JL, Ismond DR, Weingartner H, Lane E, Oliver J, Linnoila M, Potter WZ. Treatment of childhood hyperactivity with desipramine: plasma drug concentration, cardiovascular effects, plasma and urinary catecholamine levels, and clinical response. *Clin Pharmacol Ther.* 1986;39, 1, 72-81.

Reason for exclusion: Medication of no interest for the present meta-analysis vs placebo

Donnelly1989
- Donnelly M, Rapoport JL, Ismond DR. Fenfluramine treatment of childhood attention deficit disorder with hyperactivity: a preliminary report. *Psychopharmacol Bull* 1986;22(1):152–4
- Donnelly M, Rapoport JL, Potter WZ, Oliver J, Keysor CS, Murphy DL. Fenfluramine and dextroamphetamine treatment of childhood hyperactivity. Clinical and biochemical findings. *Arch Gen Psychiatry.* 1989;46(3):205-212.

Reason for exclusion: Co-intervention; Cross-over without wash out; no pre-cross over data available.

Donnelly2002
- Donnelly C, Faries D, Swensen A, et al. The effect of atomoxetine on the social and family functioning of children and adolescents with attention-deficit/hyperactivity disorder (ADHD). *Eur Neuropsychopharmacol.* 2002(Suppl 3):S437.

Reason for exclusion: Authors contacted but no further data on the study

Donnelly2009
- Donnelly C, Bangs M, Trzepacz P, et al. Safety and Tolerability of Atomoxetine over 3 to 4 Years in Children with ADHD. *J Am Acad Child Adolesc Psychiatry*. 2009;48:176.

Reason for exclusion: Meta-analysis

Döpfner2004a
- Döpfner M, Schröder S, Schmidt J, Lehmkuhl G. Duration of action of a single dose of methylphenidate Retard compared to twice immediate-release methylphenidate in children and adolescents with ADHD [Wirkdauer einer einmaligen Gabe von Methylphenidat–Retard im Vergleich zu zweimaliger Gabe von schnell freisetzendem Methylphenidat bei Kindern und Jugendlichen mit ADHS]. *Klinikum der Universität zu Köln, Klinik und Poliklinik für Psychiatrie und Psychotherapie des Kindes und Jugendalters 2003.*
- Dopfner M, Gerber WD, Banaschewski T, et al. Comparative efficacy of once-a-day extended-release methylphenidate, two-times-daily immediate-release methylphenidate, and placebo in a laboratory school setting. *Eur Child Adolesc Psychiatry.* 2004;13 Suppl 1:I93-101.
- Lehmkuhl G. Double-blind, non-inferiority trial investigating the duration of action of Medikinet-retard vs. immediate-release methylphenidate vs. placebo across the day in children with attention deficit hyperactivity disorder (ADHD). Integrated final report. Phase III. Universitätsklinikum Essen. Project number Medikinetretard (R) Trial 6520-0073-01 2005.
- Gerber-von Muller G, Petermann U, Petermann F, et al. ADHD summer camp: Development and evaluation of a multimodal intervention program. *Kindheit und Entwicklung.* 2009;18(3):162-172.
- Uebel H, Albrecht B, Kirov R, et al. What can actigraphy add to the concept of labschool design in clinical trials? *Curr Pharm Des.* 2010;16(22):2434-2442
- Gerber W-D, Gerber-von Muller G, Andrasik F, et al. The impact of a multimodal summer camp training on neuropsychological functioning in children and adolescents with ADHD: An exploratory study. *Child Neuropsychol.* 2012;18(3):242-255.

Reason for exclusion: Participants: responders to ADHD drugs; co-interventions

Döpfner2004b

- Dopfner M, Breuer D, Schurmann S, Metternich TW, Rademacher C, Lehmkuhl G. Effectiveness of an adaptive multimodal treatment in children with Attention-Deficit Hyperactivity Disorder - Global outcome. *Eur Child Adolesc Psychiatry, Supplement.* 2004;13(1):I/117-I/129.
- No arms of interest for the present meta-analysis (methylphenidate + behavioural training vs behavioural training)

Reason for exclusion: No arms of interest for the present meta-analysis (methylphenidate + behavioural training vs. behavioural training

Döpfner 2011 (EUCTR2005-003295-38-DE)

- Dopfner M, Ose C, Fischer R, Ammer R, Scherag A. Comparison of the efficacy of two different modified release methylphenidate preparations for children and adolescents with attention-deficit/hyperactivity disorder in a natural setting: comparison of the efficacy of Medikinet((R)) retard and Concerta((R))--a randomized, controlled, double-blind multicenter clinical crossover trial. *J Child Adolesc Psychopharmacol.* 2011;21(5):445-454.
- Doepfner M, Ose C, Fischer R, Ammer R, Scherag A. The CoMeCo-trial: Comparison of the efficacy of two methylphenidate preparations for children and adolescents with ADHD in a natural setting. *Eur Child Adolesc Psychiatr.* 2011;20:S116.
- www.clinicaltrialsregister.eu/ctr-search/search?query=eudract_number:2005-003295-38

Reason for exclusion: Comparison of two formulations of methylphenidate; no placebo arm

Dorrego2002

- Dorrego MF, Canevaro L, Kuzis G, Sabe L, Starkstein SE. A randomized, double-blind, crossover study of methylphenidate and lithium in adults with attention-deficit/hyperactivity disorder: preliminary findings. *J Neuropsychiatry Clin Neurosci.* 2002;14(3):289-295.

Reason for exclusion: Medication of interest (methylphenidate) vs medication of no interest (lithium) for the present meta-analysis, no placebo arm

Dougherty2016

- Dougherty DM, Olvera RL, Acheson A, Hill-Kapturczak N, Ryan SR, Mathias CW. Acute effects of methylphenidate on impulsivity and attentional behavior among adolescents comorbid for ADHD and conduct disorder. *J Adolesc.* 2016;53:222-230.

Reason for exclusion: Less than seven days treatment

Douglas1986

- Douglas VI, Barr RG, O'Neill ME, Britton BG. Short term effects of methylphenidate on the cognitive, learning and academic performance of children with attention deficit disorder in the laboratory and the classroom. *J Child Psychol Psychiatry.*1986;27(2):191-211.
- Douglas VI, Barr RG, Amin K, O'Neill ME, Britton BG. Dosage effects and individual responsivity to methylphenidate in attention deficit disorder. *J Child Psychol Psychiatry.* 1988;29(4):453-475.
- Douglas VI, Barr RG, Desilets J, Sherman E. Do high doses of stimulants impair flexible thinking in attention-deficit hyperactivity disorder? *J Am Acad Child Adolesc Psychiatry.* 1995;34(7):877-885.

Reason for exclusion: Less than seven days treatment

DRKS00011209

- http://www.drks.de/DRKS00011209

Reasons for exclusion: No medication of interest for the present meta-analysis (L-Dopa- amilsupride)

DRKS00006767

- http://www.drks.de/DRKS00006767

Reasons for exclusion: No treatment of interest for the present meta-analysis (neurofeedback)

DRKS00008974

- http://www.drks.de/DRKS00008974

Reasons for exclusion: No treatment of interest for the present meta-analysis (CBT)

DRKS00008975

- http://www.drks.de/DRKS00008975

No design and arms of interest for the present meta-analysis

DRKS00009862

- http://www.drks.de/DRKS00009862

Reasons for exclusion: No treatment of interest for the present meta-analysis

DRKS00010171

- http://www.drks.de/DRKS00010171

Reasons for exclusion: No treatment of interest for the present meta-analysis

DRKS00004879

- http://www.drks.de/DRKS00004879

Reasons for exclusion: Intervention of no interest for the present meta-analysis (neurofeedback)

Drtílková1978

- Drtílková I, Náhunek K, Macháčková V, Podhradská O. Controlled comparison of the effect of dosulepin and diazepam in hyperkinetic children with phenylketonuria. *Act Nerv Super (Praha). 1978*;20(4):247-8

Reason for exclusion: No data reported (summary of findings only); cross-over trial [Antidepressants (Dosulepin) + Diazepam vs. PLB]

Drtílková1990

- Drtilkova I, Misurec J, Nahunek K. The paradox effect of psychostimulants in the treatment of the child hyperkinetic syndrome. *Act Nerv Super (Praha).* 1990;32(4):302-303.

Reason for exclusion: No double blind RCT

Drtílková 1997

- Drtilkova I, Misurec J, Nahunek K. The paradox effect of psychostimulants in the treatment of the child hyperkinetic syndrome. *Act Nerv Super (Praha).* 1990;32:302-3
- Drtilkova I, Misurec J, Balastikova B. Amphetaminil and mesocarb in children with hyperkinetic disorder. Predicting value changes the pharmo-EEG profile of psychostimulatory substances. [Czech]Metylfenidat, amfetaminil a mesocarb u deti s hyperkinetickou poruchou. Prediktivni hodnota zmen farmakologickeho EEG profilu psychostimulacnich latek. *Ceska Slov Psychiatr.*1997;93:44-53.

Reason for exclusion: *Open label*

Duggan2000

- Duggan CM, Mitchell G, Nikles CJ, Glasziou PP, Del Mar CB, Clavarino A. Managing ADHD in general practice. N of 1 trials can help! *Aust Fam Physician.* 2000;29(12):1205-1209.

Reason for exclusion: No RCT

Dukarm2005

- Dukarm CP. Bulimia nervosa and attention deficit hyperactivity disorder: a possible role for stimulant medication. *J Womens Health (Larchmt).* 2005;14(4):345-350.

Reason for exclusion: No RCT

DuPaul1996

- DuPaul GJ, Anastopoulos AD, Kwasnik D, Barkley RA, McMurray MB. Methylphenidate effects on children with attention deficit hyperactivity disorder: Self-report of symptoms, side-effects, and self-esteem. *J Atten Disord.* 1996;1(1):3-15

Reason for exclusion: Cross-over without wash out; no pre-cross over data available

DuPaul2012 (NCT01342445)

- DuPaul GJ, Weyandt LL, Rossi JS, et al. Double-blind, placebo-controlled, crossover study of the efficacy and safety of lisdexamfetamine dimesylate in college students with ADHD. *J Atten Disord.* 2012;16(3):202-220
- https://clinicaltrials.gov/ct2/show/NCT01342445

Reason for exclusion: Cross-over without wash out; no pre-cross over data available

Duric2012

- Duric NS, Assmus J, Gundersen D, Elgen IB. Neurofeedback for the treatment of children and adolescents with ADHD: a randomized and controlled clinical trial using parental reports. *BMC Psychiatry.* 2012;12:107.

Reason for exclusion: No arms of interest for the present meta-analysis (methylphenidate, neurofeedback, methylphenidate +neurofeedback)

Dykman1980

- Dykman RA, Ackerman PT, McCray DS. Effects of methylphenidate on selective and sustained attention in hyperactive, reading-disabled, and presumably attention-disordered boys. *J Nerv Ment Dis.* 1980;168(12):745-752.
- Dykman RA, Holcomb PJ, Ackerman PT, McCray DS. Auditory ERP augmentation-reducion and methylphenidate dosage needs in attention and reading disordered children. *Psychiatry Res.* 1983;9(3):255-269.

Reason for exclusion: no DSM/ICD

Dyme1982

- Dyme IZ, Sahakian BJ, Golinko BE, Rabe EF. Perseveration induced by methylphenidate in children: Preliminary findings. *Prog in Neuropsychophamacol Biol Psychiatry*.1982;6(3):269-273.

Reason for exclusion: Less than seven days treatment; No pre-crossover data; No relevant outcomes (neuropsychological test outcomes only)

Efron1999

- Efron D. Methylphenidate versus dextroamphetamine in ADHD. *J Am Acad Child Adolesc Psychiatry.* 1999;38(5):500.

Reason for exclusion: Letter to the editor, not empirical study

Elbe 2014

- Elbe D, Barr AM, Honer WG, Procyshyn RM. Managing ADHD and disruptive behaviour disorders with combination psychostimulant and antipsychotic treatment. *J Psychiatry Neurosci*. 2014;39:E32-3.

Reason for exclusion: No RCT

Ellis1974

- Ellis MJ, Witt PA, Reynolds R, Sprague RL. Methylphenidate and the activity of hyperactives in the informal setting. *Child Develop.* 1974(1):217-220.

Reason for exclusion: No DSM/ICD criteria

Emilsson2011 (ACTRN12611000533998)

- Emilsson B, Gudjonsson G, Sigurdsson JF, et al. Cognitive behaviour therapy in medication-treated adults with ADHD and persistent Symptoms: A randomized controlled trial. *BMC Psychiatry.* 2011;11(116).
- http://www.anzctr.org.au/ACTRN12611000533998.aspx

Reason for exclusion: Individuals on psychostimulants randomized to non-pharmacological interventions

Epstein2007

- Epstein JN, Casey BJ, Tonev ST, et al. ADHD- and medication-related brain activation effects in concordantly affected parent-child dyads with ADHD. *J Child Psychol Psychiatry.* 2007;48(9):899-913.

Reason for exclusion: Less than seven days treatment

Epstein2011 (NCT01238822)

- Epstein JN, Brinkman WB, Froehlich T, et al. Effects of stimulant medication, incentives, and event rate on reaction time variability in children with ADHD. Neuropsychopharmacology. Apr 2011;36(5):1060-1072
- Froehlich TE, Epstein JN, Nick TG, et al. Pharmacogenetic predictors of methylphenidate dose-response in attention-deficit/hyperactivity disorder. *J Am Acad Child Adolesc Psychiatry.* 2011;50(11):1129-1139 e1122.
- Froehlich TE, Antonini TN, Brinkman WB, et al. Mediators of methylphenidate effects on math performance in children with attention-deficit hyperactivity disorder. *J Dev Behav Pediatr.* 2014;35(2):100-107.
- Post hoc analysis on sleep parameters in: Becker SP, Froehlich TE, Epstein JN. Effects of Methylphenidate on Sleep Functioning in Children with Attention-Deficit/Hyperactivity Disorder. *J Dev Behav Pediatr*. 2016;37(5):395-404.
- *https://clinicaltrials.gov/ct2/show/NCT01238822*

Reason for exclusion: Cross-over without wash out; no pre-cross over data available

Ernst1996

- Ernst M, Liebenauer LL, Jons PH, Tebeka D, Cohen RM, Zametkin AJ. Selegiline in adults with attention deficit hyperactivity disorder: Clinical efficacy and safety. *Psychopharmacol Bull.* 1996;32(3):327-334.
- Ernst M, Liebenauer LL, Tebeka D, et al. Selegiline in ADHD adults: Plasma monoamines and monoamine metabolites. *Neuropsychopharmacology.* 1997;16(4):276-284.

Reason for exclusion: Medicaiton of no interest for the present meta-analysis (selegiline) vs placebo; no other arms

Eslami Shahrbabaki 2012

- Eslami Shahrbabaki M, Sabzevari L, Haghdoost A, Davari-Ashtiani R. Buspiron versus methylphenidate in the treatment of children with ADHD. *Neuropsychiatr Enfance Adolesc. 2012;5:S256*

Reason for exclusion: Despite contacts with authors, no additional information available

EUCTR2005-005701-32-IT

- https://www.clinicaltrialsregister.eu/ctr-search/search?query=eudract_number:2005-005701-32

Reasons for exclusion: Open label

EUCTR2006-005512-27-FR (B4Z-BP-LYBS)

- https://www.clinicaltrialsregister.eu/ctr-search/search?query=eudract_number:2006-005512-27

Reasons for exclusion: Open label

EUCTR2007-007672-41-GB (B4Z-MC-LYDO)

- https://www.clinicaltrialsregister.eu/ctr-search/search?query=eudract_number:2007-007672-41

Reasons for exclusion: Randomized withdrawal

EUCTR2008-000191-24-NL

- https://www.clinicaltrialsregister.eu/ctr-search/search?query=eudract_number:2008-000191-24

Reasons for exclusion: Open label

EUCTR2008-004425-42-NL

- https://www.clinicaltrialsregister.eu/ctr-search/search?query=eudract_number:2008-004425-42

Reasons for exclusion: Contacted authors via http://www.chdr.nl/; no reply

EUCTR2008-000227-25-DE

- https://www.clinicaltrialsregister.eu/ctr-search/search?query=eudract_number:2008-000227-25

Reasons for exclusion: Single blind

EUCTR2008-001291-71-DE

- https://www.clinicaltrialsregister.eu/ctr-search/search?query=eudract_number:2008-001291-71

Reasons for exclusion: Single blind

EUCTR2008-001767-11-GB

- https://www.clinicaltrialsregister.eu/ctr-search/search?query=eudract_number:2008-001767-11

Reasons for exclusion: Open label

EUCTR2008-004827-44-GB

- https://www.clinicaltrialsregister.eu/ctr-search/search?query=eudract_number:2008-004827-44

Reasons for exclusion: Open label

EUCTR2009-011426-33-ES

- https://www.clinicaltrialsregister.eu/ctr-search/search?query=eudract_number:2009-011426-33

Reasons for exclusion: No RCT

EUCTR2009-011887-12-DE

- https://www.clinicaltrialsregister.eu/ctr-search/search?query=eudract_number:2009-011887-12

Reasons for exclusion: Observational

EUCTR2010-019981-94-FI

- https://www.clinicaltrialsregister.eu/ctr-search/search?query=eudract_number:2010-019981-94

Reasons for exclusion: Co-treatment

EUCTR2010-024551-82-GB

- https://www.clinicaltrialsregister.eu/ctr-search/search?query=eudract_number:2010-024551-82

Reasons for exclusion: Open label; genetic syndrome

EUCTR2009-011426-33-ES

- https://www.clinicaltrialsregister.eu/ctr-search/search?query=eudract_number:2009-011426-33

Reasons for exclusion: Open label

EUCTR2009-012261-61-NL

- https://www.clinicaltrialsregister.eu/ctr-search/search?query=eudract_number:2009-011426-33

Reasons for exclusion: No randomised

EUCTR2009-013272-47-NL

- https://www.clinicaltrialsregister.eu/ctr-search/search?query=eudract_number:2009-013272-47

Reasons for exclusion: No randomised

EUCTR2010-019930-28-NL

- https://www.clinicaltrialsregister.eu/ctr-search/search?query=eudract_number:2010-019930-28

Reasons for exclusion: Pre-schoolers

EUCTR2010-020014-28-NL

- https://www.clinicaltrialsregister.eu/ctr-search/search?query=eudract_number:2010-020014-28

Reasons for exclusion: No randomised; No participants with ADHD

EUCTR2007-006538-33-NL

- https://www.clinicaltrialsregister.eu/ctr-search/search?query=eudract_number:2007-006538-33

Reasons for exclusion: No participants with ADHD

EUCTR2009-013334-24-DE

- https://www.clinicaltrialsregister.eu/ctr-search/search?query=eudract_number:2009-013334-24

Reasons for exclusion: No participants with ADHD

EUCTR2010-020951-30-GB

- https://www.clinicaltrialsregister.eu/ctr-search/search?query=eudract_number:2010-020951-30

Reasons for exclusion: Open label

EUCTR2013-003888-59-NL

- https://www.clinicaltrialsregister.eu/ctr-search/search?query=eudract_number:2013-003888-59

Reasons for exclusion: Open label

EUCTR2015-000488-15-DE

- https://www.clinicaltrialsregister.eu/ctr-search/search?query=eudract_number:2015-000488-15

Reasons for exclusion: No RCT

EUCTR2007-001855-20/NL (NTR2109)

- https://www.clinicaltrialsregister.eu/ctr-search/search?query=eudract_number:2007-001855-20
- http://www.trialregister.nl/trialreg/admin/rctview.asp?TC=2109

Reasons for exclusion: incomplete study, no additional data

EUCTR2008-004425-42-NL

- https://www.clinicaltrialsregister.eu/ctr-search/search?query=eudract_number:2008-004425-42

Reasons for exclusion: contacted http://www.chdr.nl/via to enquire about the study; no answer

EUCTR2008-006242-26-DE

- https://www.clinicaltrialsregister.eu/ctr-search/search?query=eudract_number:2008-006242-26

Reasons for exclusion: still ongoing; no contact details

EUCTR2007-004664-46-NL

- https://www.clinicaltrialsregister.eu/ctr-search/search?query=eudract_number:2007-004664-46

Reasons for exclusion: Dr Smith confirmed the study was never finished, no available data

EUCTR2014-001488-11-SE *and* **EUCTR2014-005045-53-SE**

- https://www.clinicaltrialsregister.eu/ctr-search/search?query=eudract_number:2014-001488-11
- https://www.clinicaltrialsregister.eu/ctr-search/search?query=eudract_number:2014-005045-53

Reasons for exclusion: ongoing

EUCTR2010-020601-32-GB

- https://www.clinicaltrialsregister.eu/ctr-search/search?query=eudract_number:2014-001488-11

Reasons for exclusion: participants with ADHD

EUCTR2006-001353-96-DE

- https://www.clinicaltrialsregister.eu/ctr-search/search?query=eudract_number:2006-001353-96

Reasons for exclusion: Open label

EUCTR2007-007552-33-FR

- https://www.clinicaltrialsregister.eu/ctr-search/search?query=eudract_number:2007-007552-33

Reasons for exclusion: Open label

EUCTR2008-003285-26-DE

- https://www.clinicaltrialsregister.eu/ctr-search/search?query=eudract_number:2008-003285-26

Reasons for exclusion: Open label

EUCTR2011-000210-19-DE

- https://www.clinicaltrialsregister.eu/ctr-search/search?query=eudract_number:2011-000210-19

Reasons for exclusion: Open label extension

EUCTR2006-006441-14-DE

- https://www.clinicaltrialsregister.eu/ctr-search/search?query=eudract_number:2006-006441-14

Reasons for exclusion: Not randomised

EUCTR2013-003547-39-NL

- https://www.clinicaltrialsregister.eu/ctr-search/search?query=eudract_number:2013-003547-39

Reasons for exclusion: No participants with ADHD

EUCTR2012-000492-17-NL

- https://www.clinicaltrialsregister.eu/ctr-search/search?query=eudract_number:2012-000492-17

Reasons for exclusion: Subjects selected if responders to previous ADHD medications

EUCTR2005-004037-18-DE

- https://www.clinicaltrialsregister.eu/ctr-search/search?query=eudract_number:2005-004037-18

Reasons for exclusion: Open label

EUCTR2012-000517-37-GB

- https://www.clinicaltrialsregister.eu/ctr-search/search?query=eudract_number:2012-000517-37

Reasons for exclusion: Open label (completed)

EUCTR2014-002002-20-NL

- https://www.clinicaltrialsregister.eu/ctr-search/search?query=eudract_number:2014-002002-20

Reasons for exclusion: Withdrawal design

EUCTR2015-001070-18

- https://www.clinicaltrialsregister.eu/ctr-search/search?query=eudract_number:2015-001070-18

Reasons for exclusion: No RCT

EUCTR2015-001084-39

- https://www.clinicaltrialsregister.eu/ctr-search/search?query=eudract_number:2015-001084-39

Reasons for exclusion: No randomised

EUCTR2015-001216-35

- https://www.clinicaltrialsregister.eu/ctr-search/search?query=eudract_number:2015-001216-35

Reasons for exclusion: No randomised

EUCTR2015-001217-27

- https://www.clinicaltrialsregister.eu/ctr-search/search?query=eudract_number:2015-001217-27

Reasons for exclusion: No randomised

EUCTR2015-001218-92
- https://www.clinicaltrialsregister.eu/ctr-search/search?query=eudract_number:2015-001218-92

Reasons for exclusion: Open label

EUCTR2015-004271-78-GB
- https://www.clinicaltrialsregister.eu/ctr-search/search?query=eudract_number:2015-004271-78

Reasons for exclusion: Ongoing

EUCTR2005-002897-31
- https://www.clinicaltrialsregister.eu/ctr-search/search?query=eudract_number:2005-002897-31

Reasons for exclusion: No randomised

EUCTR2006-002716-94-IS
- https://www.clinicaltrialsregister.eu/ctr-search/search?query=eudract_number:2006-002716-94

Reasons for exclusion: No randomised

EUCTR2009-016667-11-FR
- https://www.clinicaltrialsregister.eu/ctr-search/search?query=eudract_number:2009-016667-11

Reasons for exclusion: No participants with ADHD; No intervention of interest for the present meta-analysis

EUCTR2005-003002-28-DE
- https://www.clinicaltrialsregister.eu/ctr-search/search?query=eudract_number:2005-003002-28

Reasons for exclusion: Subjects were responders ("Patients, whose symptoms are adequately controlled by a stable and well-tolerated dose of a immediate release methylphenidate equivalent of 20mg for one month before screening")

EUCTR2006-004073-10-DE
- https://www.clinicaltrialsregister.eu/ctr-search/search?query=eudract_number:2006-004073-10

Reasons for exclusion: full dataset not available yet

Evans1991
- Evans SW, Pelham WE. Psychostimulant effects on academic and behavioral measures for ADHD junior high school students in a lecture format classroom. *J Abnorm Child Psychol.* 1991;19(5):537-552.

Reason for exclusion: One subject on pemoline; randomization not clear; co-intervention

Fallu2006 (NCT00246207)
- Fallu A, Richard C, Prinzo R, Binder C. Does OROS-methylphenidate improve core symptoms and deficits in executive function? Results of an open-label trial in adults with attention deficit hyperactivity disorder. *Curr Med Res Opin.* 2006;22(12):2557-2566.
- Fallu A, Prinzo R, Binder C. Safety and effectiveness of OROS*Methylphenidate in adults with Attention Deficit Hyperactivity Disorder (ADHD): Results of an open label study. *Int J Neuropsychopharmacol.* 2006; 9(Suppl. 1): 134
- Fallu A, Richard C, Prinzo R, Binder C. OROS-methylphenidate - How safe and how effective is it in ameliorating executive functioning deficits in adults with attention deficit hyperactivity disorder? Results of an open label study. *Biol Psychiatry.* 2006; 59(8, Suppl. S): 203.
- Fallu A, No. OROS*-Methylphenidate and executive functioning in adults with attention deficit hyperactivity disorder. *Conference abstract: 39th International Danube Symposium for Neurological Science and Continuing Education 1st International Congress on ADHD from Childhood to Adult Disease, Würzburg, Deutschland 02/06/2007-05/06/2007*
- https://clinicaltrials.gov/ct2/show/NCT00246207

Reason for exclusion: Open label

Fan2012
- Fan L, Gau SS, Chou T. Neural correlates of atomoxetine improving inhibitory control and spatial processing in adults with attention-deficit/hyperactivity disorder. *Neuropsychiatr Enfance Adolesc. 2012;60(5S):S183*

Reason for exclusion: Not clear if double blind

Feigin1996
- Feigin A, Kurlan R, McDermott MP, et al. A controlled trial of deprenyl in children with Tourette's syndrome and attention deficit hyperactivity disorder. *Neurology.* 1996;46(4):965-968.

Reason for exclusion: Medication of no interest for the present meta-analysis vs placebo, no other arms

Feldman1989
- Feldman H, Crumrine P, Handen BL, Alvin R, Teodori J. Methylphenidate in children with seizures and attention-deficit disorder. *Am J Dis Child (1960).*1989;143(9):1081-1086.

Reason for exclusion: Co-treatment with antiepileptic drugs

Fenichel1995
- Fenichel RR. Combining methylphenidate and clonidine: The role of post-marketing surveillance. *J Child Adolesc Psychopharmacol.* 1995;5(3):155-156.

Reason for exclusion: No RCT

Fiedler1983
- Fiedler NL, Ullman DG. The effects of stimulant drugs on curiosity behaviors of hyperactive boys. *J Abnorm Child Psychol.* 1983;11(2):193-206.

Reason for exclusion: No placebo controlled; Less than seven days treatment

Findling2001
- Findling RL, Short EJ, Manos MJ. Short-term cardiovascular effects of methylphenidate and adderall. *J Am Acad Child Adolesc Psychiatry.* 2001;40(5):525-529.

Reason for exclusion: Clinician blinded to dose but not identity of the medication

Findling2006
- Findling RL, Quinn D, Hatch SJ, Cameron SJ, DeCory HH, McDowell M. Comparison of the clinical efficacy of twice-daily Ritalin (R) and once-daily Equasym (TM) XL with placebo in children with Attention Deficit/Hyperactivity Disorder. *Eur Child Adolesc Psychiatry.* 2006;15(8):450-459.

Reasons for exclusion: Subjects on "stable dose"; contacted first author to clarify if stable = responders but no answer

Findling2007
- Findling RL, Short EJ, McNamara NK, et al. Methylphenidate in the treatment of children and adolescents with bipolar disorder and attention-deficit/hyperactivity disorder. *J Am Acad Child Adolesc Psychiatry.* 2007;46(11):1445-1453.

Reason for exclusion: Co-treatment for bipolar disorder

Findling2009a (NCT00500071; SPD489-310)
- Findling RL, Ginsberg LD, Jain R, Gao J. Effectiveness, safety, and tolerability of lisdexamfetamine dimesylate in children with attention-deficit/hyperactivity disorder: an open-label, dose-optimization study. *J Child Adolesc Psychopharmacol.* 2009;19(6):649-662.
- https://clinicaltrials.gov/ct2/show/NCT00500071

Reason for exclusion: Open label

Findling2009b (NCT00151957; SPD485-303)
- Findling RL, Wigal SB, Bukstein OG, Boellner SW, Abikoff HB, Turnbow JM, Civil R. Long-term tolerability of the methylphenidate transdermal system in pediatric attention-deficit/hyperactivity disorder: a multicenter, prospective, 12-month, open-label, uncontrolled, phase III extension of four clinical trials. *Clin Ther.* 2009 ;31(8):1844-55.
- https://clinicaltrials.gov/ct2/show/NCT00151957

Reason for exclusion: Open label

Findling2010 (NCT00499863; SPD485-40)
- Findling RL, Turnbow J, Burnside J, Melmed R, Civil R, Li Y. A randomized, double-blind, multicenter, parallel-group, placebo-controlled, dose-optimization study of the methylphenidate transdermal system for the treatment of ADHD in adolescents. *CNS Spectr.* 2010;15(7):419-430.
- Extension in: Findling RL, Katic A, Rubin R, Moon E, Civil R, Li Y. A 6-month, open-label, extension study of the tolerability and effectiveness of the methylphenidate transdermal system in adolescents diagnosed with attention-deficit/hyperactivity disorder. *J Child Adolesc Psychopharmacol.* 2010;20(5):365-375.
- Keating GM. Methylphenidate transdermal system in attention-deficit hyperactivity disorder in adolescents: profile report. *Drugs in R&D.* 2012;12(3):171–3.
- https://clinicaltrials.gov/ct2/show/NCT00499863

Reason for exclusion: Transdermal formulation; no oral formulations

Findling2013(NCT00764868; SPD489-306)

- Findling RL, Cutler AJ, Saylor K, et al. A long-term open-label safety and effectiveness trial of lisdexamfetamine dimesylate in adolescents with attention-deficit/hyperactivity disorder. *J Child Adolesc Psychopharmacol.* 2013;23(1):11-21.
- https://clinicaltrials.gov/ct2/show/NCT00764868

Reason for exclusion: Open label

Findling2014

- Findling RL, McBurnett K, White C, Youcha S. Guanfacine extended release adjunctive to a psychostimulant in the treatment of comorbid oppositional symptoms in children and adolescents with attention-deficit/hyperactivity disorder. *J Child Adolesc Psychopharmacol.* 2014;24(5):245-252.

Reason for exclusion: Co-medication; participants with previous suboptimal response to ADHD mediactions

Fine1989

- Fine S, Jewesson B. Active drug placebo trial of methylphenidate - A clinical service for children with an attention deficit disorder. *Can J Psychiatry.* 1989;34(5):447-449.

Reason for exclusion: Definition of ADHD not specified; not possible to contact author; no useful data for the present meta-analysis

Fine1993

- Fine S, Johnston C. Drug and placebo side effects in methylphenidate-placebo trial for attention deficit hyperactivity disorder. *Child Psychiatry Hum Dev*. 1993;24(1):25-30
- Johnston C, Fine S. Methods of evaluating methylphenidate in children with attention deficit hyperactivity disorder: acceptability, satisfaction, and compliance. *J Pediatr Psychol*. 1993;18(6):717-730.

Reason for exclusion: Cross-over without wash out; no pre-cross over data available; No full DSM/ICD criteria

Finnerty1971

- Finnerty RJ, Soltys JJ, Cole JO. The use of D-amphetamine with hyperkinetic children. *Psychopharmacologia.* 1971;21(3):302-308.

Reason for exclusion: No DSM/ICD criteria

Firestone1978

- Firestone P, Davey J, Goodman JT, Peters S. The effects of caffeine and methylphenidate on hyperactive children. *J Am Acad Child Psychiatr.* 1978(3):445-456.

Reason for exclusion: No DSM/ICD criteria

Firestone1981

- Firestone P. Differential Effects of Parent Training and Stimulant Medication with Hyperactives: A Progress Report. *Children's Hospital of Eastern Ontario, Ottawa (Canada). 1979.*
- Firestone P, Kelly MJ, Goodman JT, Davey J. Differential effects of parent training and stimulant medication with hyperactives: a progress report. *J Am Acad Child Psychiatr*. 1981;20(1): 135–47.

Reason for exclusion: No appropriate arms for the present meta-analysis

Firestone1986

- Firestone P, Crowe D, Goodman JT, McGrath P. Vicissitudes of follow-up studies: differential effects of parent training and stimulant medication with hyperactives. *Am J Orthopsychiatry.* 1986;56(2):184-194.

Reason for exclusion: Study arms (parent training +medication; parent training plus placebo; medicatin only) not appropriate for the present meta-analysis

Fischer1991

- Fischer, M, Newby RF Assessment of stimulant response in ADHD children using a refined multimethod clinical protocol. *J Clin Child Psychol.* 1991; 20(3): 232-244

Reason for exclusion: Cross-over without wash out; no pre-cross over data available

Fischer1998

- Fischer M, Newby RF. Use of the restricted academic task in ADHD dose-response relationships. *J Learn Disabil.* 1998;31(6):608-612.

Reason for exclusion: No DSM/ICD criteria

Fisher1978
- Fisher MA. Dextroamphetamine and placebo practice effects on selective attention in hyperactive children. *J Abnorm Child Psychol.* 1978;6(1):25-32.

Reason for exclusion: Less than seven days treatment

Fitzpatrick1992
- Fitzpatrick PS. Effects of Sustained-Release and Standard Preparations of Methylphenidate on Attention Deficit Hyperactivity Disorder: Clinical Outcome, Performance, and Cognitive Event-Related Potentials *[PhD thesis]. New York, USA: University of Rochester, 1990.*
- Fitzpatrick PA, Klorman R, Brumaghim JT, Borgstedt AD. Effects of sustained-release and standard preparations of methylphenidate on attention deficit disorder. *J Am Acad Child Adolesc Psychiatry.* 1992;31(2):226-234.

Reason for exclusion: "Latin square" but no mention of randomisation

Flapper2006
- Flapper BC, Houwen S, Schoemaker MM. Fine motor skills and effects of methylphenidate in children with attention-deficit-hyperactivity disorder and developmental coordination disorder. *Dev Med Child Neurol.* 2006;48(3):165-169.
- Flapper BC, Schoemaker MM. Effects of methylphenidate on quality of life in children with both developmental coordination disorder and ADHD. *Dev Med Child Neurol.* 2008;50(4):294-299.

Reason for exclusion: No mention of randomization; authors confirmed study was not randomized. No outcomes of interest.

Flintoff1982
- Flintoff MM, Barron RW, Swanson JM, Ledlow A, Kinsbourne M. Methylphenidate increases selectivity of visual scanning in children referred for hyperactivity. *J Abnorm Child Psychol.* 1982;10(2):145-161.

Reason for exclusion: DSM-II criteria

Focken1984
- Focken A, et al. Effects of methylphenidate in hyperactive children with minimal cerebral dysfunction: Influence on psychological, physiological and biochemical parameters in a double-blind study. *Z Kinder Jugendpsychiatr Psychother.* 1984;12(3):235-249.

Reason for exclusion: No DSM/ICD criteria

Forness1992a
- Forness SR, Cantwell DP, Swanson JM, Hanna GL, Youpa D. Differential effects of stimulant medication on reading performance of boys with hyperactivity with and without conduct disorder. *J Learn Disabil.* 1991;24(5):304-310.
- Forness SR, Swanson JM, Cantwell DP, Youpa D, Hanna GL. Stimulant medication and reading performance: follow-up on sustained dose in ADHD boys with and without conduct disorders. *J Learn Disabil.* 1992;25(2):115-123.

Reason for exclusion: Subjects selected as being medication responders to a previous study

Forness1992b
- Part of subjects in: Forness SR, Cantwell DP, Swanson JM, Hanna GL, Youpa D. Differential effects of stimulant medication on reading performance of boys with hyperactivity with and without conduct disorder. *J Learn Disabil.* 1991;24(5):304-310. (and in Swanson et al. "in press" at the time this paper was published but not able to identify it; Dr. Swanson let us know that paper was never published)
- Forness SR, Swanson JM, Cantwell D, Guthrie D, Sena R. Response to stimulant medication across six measures of school-related performance in children with ADHD and disruptive behavior. *Behavioral Disorders*. 1992;18(1):42-53

Reason for exclusion: Cross-over without wash out; no pre-cross over data available

Fosco2016
- Fosco WD, White CN, Hawk LW, Jr. Acute Stimulant Treatment and Reinforcement Increase the Speed of Information Accumulation in Children with ADHD. *J Abnorm Child Psychol.* 2017 ;45(5):911-920

Reason for exclusion: Study 1: No RCT. Study 2: Less than seven days treatment

Fox2014 (NCT00446537)

- Fox O, Adi-Japha E, Karni A. The effect of a skipped dose (placebo) of methylphenidate on the learning and retention of a motor skill in adolescents with Attention Deficit Hyperactivity Disorder. *Eur Neuropsychopharmacol.* 2014;24(3):391-396.
- https://clinicaltrials.gov/ct2/show/NCT00446537

Reason for exclusion: Single dose

Francis2001
- Francis S, Fine J, Tannock R. Methylphenidate selectively improves story retelling in children with attention deficit hyperactivity disorder. *J Child Adolesc Psychopharmacol.* 2001;11(3):217-228.

Reason for exclusion: Single dose

Frank1993
- Frank Y. Visual event related potentials after methylphenidate and sodium valproate in children with attention deficit hyperactivity disorder. *Clin Electroencephalogr.* 1993;24(1):19-24.

Reason for exclusion: Single dose

Fredericks2004
- Fredericks EM, Kollins SH. Assessing methylphenidate preference in ADHD patients using a choice procedure. *Psychopharmacology (Berl).* 2004;175(4):391-398.

Reason for exclusion: Less than seven days treatment

Gabriel2003
- Gabriel KH. [EEG diagnosis before beginning and during drug treatment of hyperkinetic children and adolescents]. *Z Kinder Jugendpsychiatr Psychother.* 2003;31(3):231-232; author reply 233-234.

Reason for exclusion: No RCT

Gadow1990a
- Gadow KD, Sverd J. Stimulants for ADHD in child patients with Tourette's syndrome: the issue of relative risk. *J Dev Behav Pediatr*. 1990;11(5):269-271; discussion 272.

Reason for exclusion: Review- commentary, no original data

Gadow1990b
- Gadow KD, Nolan EE, Sverd J, Sprafkin J, Paolicelli L. Methylphenidate in aggressive-hyperactive boys: I. Effects on peer aggression in public school settings. *J Am Acad Child Adolesc Psychiatry*. 1990;29(5):710-718
- Gadow KD, Paolicelli LM, Nolan EE, Schwartz J, Sprafkin J, Sverd J. Methylphenidate in aggressive hyperactive boys: II. Indirect effects of medication treatment on peer behavior. *J Child Adolesc Psychopharmacol*. 1992;2 (1):49–61.

Reason for exclusion: No full diagnostic criteria as per protocol; Cross-over without wash out; no pre-cross over data available

Gadow1991
- Gadow KD, Nolan EE, Paolicelli LM, Sprafkin J. A procedure for assessing the effects of methylphenidate on hyperactive children in public school settings. *J Clin Child Psychol.* 1991;20(3):268-276.

Reason for exclusion: Description of study procedure with a case study

Gadow2001
- Gadow KD, Weiss M. Attention-deficit/hyperactivity disorder in adults: beyond controversy. *Arch Gen Psychiatry.* 2001;58(8):784-785.

Reason for exclusion: Editorial

Gadow2006
- Gadow KD, Sverd J. Attention deficit hyperactivity disorder, chronic tic disorder, and methylphenidate. *Adv Neurol.* 2006;99:197-207.

Reason for exclusion: Review- commentary, no original data

Gadow2007
- Eleven subjects from: "Gadow KD, Sverd J, Sprafkin J, Nolan EE, Grossman S. Long-term methylphenidate therapy in children with comorbid attention-deficit hyperactivity disorder and chronic multiple tic disorder. *Arch Gen Psychiatry*. Apr 1999;56(4):330-336" participated in Sverd J, Gadow KD, Nolan EE, Sprafkin J, Ezor SN. Methylphenidate in hyperactive

boys with comorbid tic disorder: I. Clinic evaluations. *Adv Neurol.* 1992;58:271–81
- Previous study related to: Sverd J, Gadow KD, Nolan EE, Sprafkin J, Ezor SN. Methylphenidate in hyperactive boys with comorbid tic disorder, I: clinic evaluations. In: Chase TN, Friedhoff AJ, Cohen DJ, eds. Tourette Syndrome: Genetics, Neurobiology, and Treatment. New York, NY: Raven Press.
- Eleven subjects from: "Gadow KD, Sverd J, Sprafkin J, Nolan EE, Grossman S. Long-term methylphenidate therapy in children with comorbid attention-deficit hyperactivity disorder and chronic multiple tic disorder. *Arch Gen Psychiatry*. 1999;56(4):330-336" participated in Gadow KD, Nolan EE, Sverd J. Methylphenidate in hyperactive boys with comorbid tic disorder: II. Short-term behavioral effects in school settings. *J Am Acad Child Adolesc Psychiatry*. 1992;31(3):462-471.
- Nolan EE, Gadow KD. Relation between ratings and observations of stimulant drug response in hyperactive children. *J Clin Child Psychol.* 1994;23(1):78-90.
- Gadow KD, Nolan E, Sprafkin J, Sverd J. School observations of children with attention-deficit hyperactivity disorder and comorbid tic disorder: effects of methylphenidate treatment. *J Dev Behav Pediatr.* 1995;16(3):167-176.
- Gadow KD, Sverd J, Sprafkin J, Nolan EE, Ezor SN. Efficacy of methylphenidate for attention-deficit hyperactivity disorder in children with tic disorder. *Arch Gen Psychiatry.* 1995;52(6):444-455 (correction: Gadow KD, Sverd J, Sprafkin J, Nolan EE, et al. "Efficacy of methylphenidate for attention-deficit hyperactivity disorder in children with tic disorder": Correction. *Arch Gen Psychiatry.* 1995;52(10):836)
- Reprint in: Gadow KD, Sverd J, Sprafkin J, Nolan EE, et al. Efficacy of methylphenidate for attention-deficit hyperactivity disorder in children with tic disorder. *Annual Progress in Child Psychiatry & Child Development*. 1996:494-522
- Sprafkin J, Gadow KD. Double-blind versus open evaluations of stimulant drug response in children with attention-deficit hyperactivity disorder. *J Child Adolesc Psychopharmacol.* 1996;6(4):215–28
- Nolan EE, Gadow KD. Children with ADHD and tic disorder and their classmates: behavioral normalization with methylphenidate. *J Am Acad Child Adolesc Psychiatry.* 1997;36(5):597–604.
- Follow-up: in Gadow KD, Sverd J, Sprafkin J, Nolan EE, Grossman S. Long-term methylphenidate therapy in children with comorbid attention-deficit hyperactivity disorder and chronic multiple tic disorder. *Arch Gen Psychiatry*. 1999;56(4):330-336). (NCT00441649)
- Gadow KD, Nolan EE, Sverd J, Sprafkin J, Schwartz J. Anxiety and depression symptoms and response to methylphenidate in children with attention-deficit hyperactivity disorder and tic disorder. *J Clin Psychopharmacol.* 2002;22(3):267-274
- Gadow KD, Sverd J, Nolan EE, Sprafkin J, Schneider J. Immediate-release methylphenidate for ADHD in children with comorbid chronic multiple tic disorder. *J Am Acad Child Adolesc Psychiatry*. 2007;46(7):840-848.
- Gadow KD, Nolan EE, Sverd J, Sprafkin J, Schneider J. Methylphenidate in children with oppositional defiant disorder and both comorbid chronic multiple tic disorder and ADHD. *J Child Neurol.* 2008;23(9):981-990
- Gadow KD, Nolan EE. Methylphenidate and comorbid anxiety disorder in children with both chronic multiple tic disorder and ADHD. *J Atten Disord.* 2011;15(3):246-256.

Note and reason for exclusion: After gathering data on overlap among samples from the author, we decided to retain "Gadow KD, Sverd J, Nolan EE, Sprafkin J, Schneider J. Immediate-release methylphenidate for ADHD in children with comorbid chronic multiple tic disorder. J Am Acad Child Adolesc Psychiatry. Jul 2007;46(7):840-848" for the analyses; however, analyses were not possible due to lack of pre cross-over data (the study was cross-over with no wash out)

Gagliano2005
- Gagliano C, Read S, Thorpe L, Eerdekens M, Van Hove I. Short- and long-term efficacy and safety of risperidone in adults with disruptive behvaior disorders. *Psychopharmacology (Berl).* 2005;179(3):629-36

Reason for exclusion: No participants with ADHD; medication of no interest for the present meta-analysis (risperidone) vs placebo

Gan1982
- Gan J, Cantwell DP. Dosage effects of methylphenidate on paired associate learning: positive/negative placebo responders. *J Am Acad Child Psychiatry.* 1982;21(3):237-242.

Reason for exclusion: Less than seven days treatment

Garfinkel1975a
- Garfinkel BD, Webster CD, Sloman L. Methylphenidate and caffeine in the treatment of children with minimal brain dysfunction. *Am J Psychiatry.* 1975;132(7):723-728.

Reason for exclusion: No DSM/ICD criteria

Garfinkel1975b
- Garfinkel BD, Webster CD, Sloman L. Individual responses to methylphenidate and caffeine in children with minimal brain dysfunction. *Can Med Assoc J.* 1975;113(8):729-732.

Reason for exclusion: Diagnosis of minimal brain dysfunction

Garfinkel1981
- Garfinkel BD, Webster CD, Sloman L. Responses to methylphenidate and varied doses of caffeine in children with attention deficit disorder. *Can J Psychiatry.* 1981;26(6):395-401.

Reason for exclusion: Arms: placebo + methylphenidate; low dose caffeine + methylphenidate; high dose caffeine + methylphenidate

Garfinkel1983
- Garfinkel BD, Wender PH, Sloman L, O'Neill I. Tricyclic antidepressant and methylphenidate treatment of attention deficit disorder in children. *J Am Acad Child Psychiatr.* 1983(4):343-348.

Reason for exclusion: Co-treatment (behavioral therapy and dynamically based psychotherapy)

Garfinkel1986
- Garfinkel BD, Brown WA, Klee SH. Neuroendocrine and cognitive responses to amphetamine in adolescents with a history of attention deficit disorder. *J Am Acad Child Psychiatry.* 1986(4):503-508.

Reason for exclusion: Single dose study

Garg2014 (CTRI/2011/08/001981)
- Garg J, Arun P, Chavan BS. Comparative short term efficacy and tolerability of methylphenidate and atomoxetine in attention deficit hyperactivity disorder. *Indian Pediatr.* 2014;51(7):550-554.
- Subset in: Garg J, Arun P, Chavan BS. Comparative efficacy of methylphenidate and atomoxetine in oppositional defiant disorder comorbid with attention deficit hyperactivity disorder. *Int J Appl Basic Med Res.* 2015;5(2):114-118.
- http://www.ctri.nic.in/Clinicaltrials/pmaindet2.php?trialid=3407

Reason for exclusion: Open label

Garland2004
- Garland M, Kirkpatrick P. Atomoxetine hydrochloride. *Nat Rev Drug Discov.* 2004;3(5):385-386.

Reason for exclusion: Review

Gehricke2006
- Gehricke JG, Whalen CK, Jamner LD, Wigal TL, Steinhoff K. The reinforcing effects of nicotine and stimulant medication in the everyday lives of adult smokers with ADHD: A preliminary examination. *Nicotine Tob Res.* 2006;8(1):37-47.

Reason for exclusion: Less than seven days treatment

Gehricke2011
- Gehricke JG, Hong N, Wigal TL, Chan V, Doan A. ADHD medication reduces cotinine levels and withdrawal in smokers with ADHD. *Pharmacol Biochem Behav.* 2011;98(3):485-491.

Reason for exclusion: No RCT

Geller1981
- Geller B, Guttmacher LB, Bleeg M. Coexistence of childhood onset pervasive developmental disorder and attention deficit disorder with hyperactivity. *Am J Psychiatry.* 1981;138(3):388-389.

Reason for exclusion: Case reports

Gench1992
- Jackson SL, Gench B, Pyfer J, Gorman D. Effects of Ritalin on the postrotatory nystagmus response of hyperactive children with attention deficit disorders. *Clin. Kinesiology.* 1992(2):13-17.

Reason for exclusion: Single day study; participants: responders to ADHD medications

Ghanizadeh2008a
- Ghanizadeh A. Insomnia, night terror, and depression related to clonidine in attention-deficit/hyperactivity disorder. *J Clin Psychopharmacol.* 2008;28(6):725-726.

Reason for exclusion: Case report

Ghanizadeh2008b

- Ghanizadeh A. Methylphenidate-associated enuresis in attention deficit hyperactivity disorder. *J Pediatr Urol.* 2008;4(4):306-307.

Reason for exclusion: Case report

Ghanizadeh2008c

- Ghanizadeh A, Aghakhani K. Photophobia and methylphenidate. *Psychopharmacol Bull.* 2008;41(1):171-173.

Reason for exclusion: Case report

Ghanizadeh2010

- Ghanizadeh A. Visual fields in children with attention-deficit/hyperactivity disorder before and after treatment with stimulants. *Acta Opthalmologica.* 2010;88(2):e56.

Reason for exclusion: Commentary

Ghanizadeh2012

- Ghanizadeh A, Haghighat R. Nortriptyline for treating enuresis in ADHDa randomized double-blind controlled clinical trial. *Pediatr Nephrol.* 2012;27(11):2091-2097.

Reason for exclusion: Study arms not pertinent for the present meta-analysis

Ghanizadeh2013

- Ghanizadeh A, Sayyari Z, Mohammadi MR. Effect of methylphenidate and folic Acid on ADHD symptoms and quality of life and aggression: a randomized double blind placebo controlled clinical trial. *Iran J Psychiatry.* 2013;8(3):108-112.

Reason for exclusion: Study arms not pertinent for the present meta-analysis: methylphenidate+folic acid vs. methylphenidate+placebo

Ghanizadeh2015

- Ghanizadeh A, Haddad B. The effect of dietary education on ADHD, a randomized controlled clinical trial. *Ann Gen Psychiatry.* 2015;14:12.

Reason for exclusion: Arms of no interest for the present meta-aalysis (Methylphenidate+dietary vs. methylphenidate)

Giblin2011 (NCT00807222)

- Giblin JM, Strobel AL. Effect of lisdexamfetamine dimesylate on sleep in children with ADHD. *J Atten Disord.* 2011;15(6):491-498.
- https://clinicaltrials.gov/ct2/show/NCT00807222

Reason for exclusion: "Following completion of the open-label dose-optimization period and successful titration to an optimal dose of LDX, participants were randomized in a 2:1 ratio to either the opti- mized dose of LDX or placeb". Not clear if this means thery were responders; no reply from authors; Shire (manufacturer) does not have access to this study (26.1.17)

Giblin2011

- Giblin JM, Tenorio E, Wang C, Muniz R. Safety and efficacy of chronic administration of Clonidine extended release tablet monotherapy or combination therapy in pediatric patients with ADHD. *Ann Neurol.* 2011;70:S143.

Reason for exclusion: No RCT; Clonidine alone we or in combination with other therapies

Gilbert2006a

- Gilbert DL, Ridel KR, Sallee FR, Zhang J, Lipps TD, Wassermann EM. Comparison of the inhibitory and excitatory effects of ADHD medications methylphenidate and atomoxetine on motor cortex. *Neuropsychopharmacology.* 2006;31(2):442-449.

Reason for exclusion: Single dose study

Gilbert2006b

- Gilbert DL, Wang Z, Sallee FR, et al. Dopamine transporter genotype influences the physiological response to medication in ADHD. *Brain.* 2006;129(Pt 8):2038-2046.

Reason for exclusion: Single dose study

Gillberg1997

- Gillberg C, Melander H, vonKnorring AL, et al. Long-term stimulant treatment of children with attention-deficit hyperactivity disorder symptoms - A randomized, double-blind, placebo-controlled trial. *Arch Gen Psychiatry.* 1997;54(9):857-864.

- Von Knorring AL. Central stimulant treatment in attention deficit-hyperactivity disorder. Long-term effects. *Nord J Psychiatry.* 1998;52(2):102-103.

Reason for exclusion: Phase before randomization where subjects were "optimized" (here, responders since Conners score dropped)

Gilmore2001

- Gilmore A, Milne R. Methylphenidate in children with hyperactivity: review and cost-utility analysis. *Pharmacoepidemiol Drug Saf.* 2001;10(2):85-94.

Reason for exclusion: No RCT

Ginsberg2003a

- Ginsberg DL. Selegiline patch effective for attentional-deficit/hyperactivity disorder in children and adolescents. *Prim psychiatry.* 2003;10(6):19

Reason for exclusion: Review

Ginsberg2003b

- Ginsberg DL. Selegiline Patch Effective for Attention-Deficit/Hyperactivity Disorder in Children and Adolescents. *Prim psychiatry.* 2003;10(6):19.

Reason for exclusion: Not pertinent drug (selegeline) for the present meta-analysis

Gittelman1977

- Gittelman R. Preliminary report on the efficacy of methylphenidate and behavior modification in hyperkinetic children [proceedings]. *Psychopharmacol Bull.* 1977;13(2):53-54.

Reason for exclusion: No DSM/ICD criteria

Gittleman-Klein1975

- Gittleman-Klein R Klein DF. Are behavioral and psychometric changes related in methylphenidate-treated, hyperactive children? *Int J Ment Health.* 1975; 4(1-2):182-198.

Reason for exclusion: No DSM/ICD criteria

Gittelman-Klein1976a

- Gittelman-Klein R, Klein DF, Katz S, Saraf K, Pollack E. Comparative effects of methylphenidate and thioridazine in hyperkinetic children. I. Clinical results. *Arch Gen Psychiatry.* 1976;33(10):1217-1231.
- Halperin JM, Gittelman R, Katz S, Struve FA. Relationship between stimulant effect, electroencephalogram, and clinical neurological findings in hyperactive children. *J Am Acad Child Psychiatry.* 1986;25(6):820-825.

Reason for exclusion: DSM-II criteria

Gittelman-Klein1976b

- Gittelman-Klein R, Klein DF, Abikoff H, Katz S, Gloisten AC, Kates W. Relative efficacy of methylphenidate and behavior modification in hyperkinetic children: an interim report. *J Abnorm Child Psychol.* 1976;4(4):361-379.

Reason for exclusion: No study arms of interest for the present meta-analysis

Gittelman-Klein1980

- Gittelman-Klein R, Abikoff H, Pollack E, Katz, Mattes. Controlled trial of behaviour modification and methylphenidate in hyperactive children. In: Whalen C, Henker B editor(s). *Hyperactive Children: The Social Ecology of Identification and Treatment*. New York: Academic Press, 1980.

Reason for exclusion: No DSM/ICD criteria

Glusker1982

- Glusker P. Interpreting results on optimal doses of methylphenidate. *J Dev Behav Pediatr.* 1982;3:39

Reason for exclusion: Letter to the editor, not empirical study

Godfrey2009

- Godfrey J. Safety of therapeutic methylphenidate in adults: a systematic review of the evidence. *J Psychopharmacol (Oxford, England).* 2009;23(2):194-205.

Reason for exclusion: No participants with ADHD

Goldfield2007

- Goldfield GS, Lorello C, Doucet E. Methylphenidate reduces energy intake and dietary fat intake in adults: a mechanism of reduced reinforcing value of food? *Am J Clin Nutr.* 2007;86(2):308-315.

Reason for exclusion: Less than seven days treatment

Goldfield2011

- Goldfield GS, Lorello C, Cameron J, Chaput JP. Gender differences in the effects of methylphenidate on energy intake in young adults: a preliminary study. *Appl Physiol Nutr Metab.* 2011;36(6):1009-1013.

Reason for exclusion: No participants with ADHD

Golinko1980

- Golinko BE, Rennick PM, Glaros AG. Tolerance to dextroamphetamine sulfate in hyperactive children: assessment using an empirical neuropsychological paradigm--a pilot study. *Prog Neuropsychopharmacol.* 1980(6):601-606.

Reason for exclusion: No RCT

Golinko1981

- Golinko BE, Rennick PM, Lewis RF. Predicting stimulant effectiveness in hyperactive children with a repeatable neuropsychological battery: a preliminary study. *Prog Neuropsychopharmacol.* 1981;5(1):65-68.

Reason for exclusion: No RCT

Golinko1982

- Golinko BE. Side effects of dexedrine in hyperactive children: operationalization and quantification in a short-term trial. *Prog Neuropsychopharmacol Biol Psychiatry.* 1982;6(2):175-183.

Reason for exclusion: No RCT

Golubchik2009

- Golubchik P, Sever J, Weizman A. Influence of methylphenidate treatment on smoking behavior in adolescent girls with attention-deficit/hyperactivity and borderline personality disorders. *Clin Neuropharmacol.* 2009;32(5):239-242.

Reason for exclusion: No RCT

Golubchik2011 (NCT00552266)

- Golubchik P, Sever J, Weizman A, Zalsman G. Methylphenidate treatment in pediatric patients with attention-deficit/hyperactivity disorder and comorbid trichotillomania: a preliminary report. *Clin Neuropharmacol.* 2011;34(3):108-110.
- https://clinicaltrials.gov/ct2/show/NCT00552266

Reason for exclusion: No RCT

Gomatos2002

- Gomatos OG, Antonopoulos MS, Delorme AJ, DePamphilis JL, Garalis DD. Buproprion SR versus methylphenidate in the treatment of adults with ADHD with or without comorbid depression: A cost effective study. *ASHP Midyear Clinical Meeting 2002;37:: 667*

Reason for exclusion: No RCT

Gonzalez-Carpio2016

- Gonzalez-Carpio Hernandez G, Serrano Selva JP. Medication and creativity in Attention Deficit Hyperactivity Disorder (ADHD). *Psicothema.* 2016;28(1):20-25.

Reason for exclusion: Single blind

Gonzalez-Heydrich2010 (NCT00323947)

- Gonzalez-Heydrich JM, Whitney JE, Hsin O, et al. Tolerability of OROS (R) MPH for treatment of ADHD plus epilepsy. *Ann Neurol.* 2006;60(Suppl. 10):S47-S48.
- Gonzalez-Heydrich J. OROS methylphenidate for attention-deficit/hyperactivity disorder plus epilepsy. *P T.* 2006;31(12):725-726.
- Gonzalez-Heydrich J, Whitney J, Waber D, et al. Adaptive phase I study of OROS methylphenidate treatment of attention deficit hyperactivity disorder with epilepsy. *Epilepsy Behav.* 2010;18(3):229-237.
- Gonzalez-Heydrich J, Whitney J, Hsin O, Mrakotsky C,MacMillan C, Torres A, et al. Tolerability of OROS-MPH 18 and 36 mg in paediatric epilepsy plus attention deficit/hyperactivity disorder (ADHD). Epilepsia. *Proceedings of the 26th International Epilepsy Congress; 2005 August 28th -September 1st; Paris, France 2005;46(Suppl s6):179.*
- https://clinicaltrials.gov/ct2/show/NCT00323947

Reason for exclusion: Co-treatment for epilepsy

Gordon1978

- Gordon DA, Forehand R, Picklesimer DK. The effects of dextroamphetamine on hyperactive children using multiple outcomes measures. *J Clin Child Psychol.* 1978;7(2):125-128.

Reason for exclusion: No DSM/ICD criteria; no relevant outcomes; no pre-crossover data

Gorman2006

- Chang HTT. Effects of methylphenidate on performance and private speech of children with attention-deficit/ hyperactivity disorder during the Tower of Hanoi task. *Dissertation Abstracts International: Section B: The Sciences and Engineering.* 2001;62(1-B):540.
- Kopecky H, Chang HT, Klorman R, Thatcher JE, Borgstedt AD. Performance and private speech of children with attention-deficit/hyperactivity disorder while taking the Tower of Hanoi test: effects of depth of search, diagnostic subtype, and methylphenidate. *J Abnorm Child Psychol.* 2005;33(5):625-638.
- Gorman EB, Klorman R, Thatcher JE, Borgstedt AD. Effects of methylphenidate on subtypes of attention-deficit/hyperactivity disorder. *J Am Acad Child Adolesc Psychiatry*. 2006;45(7):808-816.

Reason for exclusion: Cross-over without wash out; no pre-cross over data available

Grade1998

- Grade, C, Redford, B, Chrostowski, J, Toussaint, L, Blackwell, B(1998) Methylphenidate in early poststroke recovery: a double-blind, placebo-controlled study. *Arch Phys Med Rehabil*. 1998 ;79(9):1047-50

Reason for exclusion: No participants with ADHD

Granger1996

- Granger DA, Whalen CK, Henker B, et al. ADHD boys' behavior during structured classroom social activities: Effects of social demands, teacher proximity, and methylphenidate. *J Atten Disord.* 1996;1(1): 16-30.

Reason for exclusion: Co-intervention

Green1973

- Green RP, Scales SM, Rosser PL. Oral medications for minimal brain dysfunction in children. *J Natl Med Assoc.* 1973;65(2):157-160.

Reason for exclusion: No RCT; minimal brain dysfunction

Green2011

- Green T, Weinberger R, Weizman A, Kotler M, Gothelf D. Effect of Methylphenidate on Neurocognitive Functioning in Velocardiofacial Syndrome: A Randomized Placebo-Controlled Trial. *Biol Psychiatry.* 2009;65(8, Suppl. S):147S.
- Green T, Weinberger R, Diamond A, et al. The effect of methylphenidate on prefrontal cognitive functioning, inattention, and hyperactivity in velocardiofacial syndrome. *J Child Adolesc Psychopharmacol.* 2011;21(6):589-595.

Reason for exclusion: Comorbid rare inherited condition

Greenberg1972

- Greenberg LM, Deem MA, McMahon S. Effects of dextroamphetamine, chlorpromazine, and hydroxyzine on behavior and performance in hyperactive children. *Am J Psychiatry.* 1972;129(5):532-539.

Reason for exclusion: No DSM/ICD criteria

Greenberg1975

- Greenberg LM, Yellin AM, Spring C, Metcalf M. Clinical effects of imipramine and methylphenidate in hyperactive children. *Int J Ment Health.* 1975;4(1-2):144-156.
- Greenberg LM, Yellin AM. Blood pressure and pulse changes in hyperactive children treated with imipramine and methylphenidate. *Am J Psychiatry.* 1975;132(12):1325-1326.

Reason for exclusion: No DSM/ICD criteria

Greenberg1987

- Greenberg LM. An objective measure of methylphenidate response: clinical use of the MCA. *Psychopharmacol Bull.* 1987;23(2):279-282.

Reason for exclusion: Not double blind

Greenhill1973

- Greenhill LL, Rieder RO, Wender PH, Buchsbaum M, Zhan TP. Lithium carbonate in the treatment of hyperactive children. *Arch Gen Psychiatry.* 1973;28(5):636-640.

Reason for exclusion: No DSM/ICD criteria

Greenhill1977

- Greenhill LL, Puig-Antich J, Sassin J, Sachar EJ. Hormone and growth responses in hyperkinetic children on stimulant medication [proceedings]. *Psychopharmacol Bull.* 1977;13(2):33-36.

Reason for exclusion: No RCT

Greenhill1987

- Greenhill LL, Cooper T, Solomon M, Fried J, Cornblatt B. Methylphenidate salivary levels in children. *Psychopharmacol Bull.* 1987;23(1):115-119.

Reason for exclusion: Diagnostic criteria not clear and not clear if study reporting the full sample (n=54) has been published; no pre cross-over data; no reply form the author

Greenhill2003

- Greenhill LL, Swanson JM, Steinhoff K, et al. A pharmacokinetic/pharmacodynamic study comparing a single morning dose of adderall to twice-daily dosing in children with ADHD. *J Am Acad Child Adolesc Psychiatry.* 2003;42(10):1234-1241.

Reason for exclusion: Less than seven days treatment

Gross1973

- Gross MD. Imipramine in the treatment of minimal brain dysfunction in children. *Psychosomatics.* 1973;14(5):283-285.

Reason for exclusion: No RCT

Gross1975

- Gross MD. Caffeine in the treatment of children with minimal brain dysfunction or hyperkinetic syndrome. *Psychosomatics.* 1975;16(1):26-27.

Reason for exclusion: No DSM/ICD criteria

Gross1976a

- Gross MD. Growth of hyperkinetic children taking methylphenidate, dextroamphetamine, or imipramine/desipramine. *Pediatrics.* 1976;58(3):423-431.

Reason for exclusion: No RCT; No DSM/ICD criteria

Gross1976b

- Gross MD. A comparison of dextro-amphetamine and racemic-amphetamine in the treatment of the hyperkinetic syndrome or minimal brain dysfunction. *Dis Nerv Syst.* 1976;37(1):14-16.

Reason for exclusion: No DSM/ICD criteria

Gross-Tsur1997

- Gross-Tsur V, Manor O, van der Meere J, Joseph A, Shalev RS. Epilepsy and attention deficit hyperactivity disorder: is methylphenidate safe and effective? *J Pediatr.* 1997;130(4):670-674.

Reason for exclusion: Less than seven days treatment; co-treatment for epilepsy

Gross-Tsur2002

- Gross-Tsur V, Shalev RS, Badihi N, Manor O. Efficacy of methylphenidate in patients with cerebral palsy and attention-deficit hyperactivity disorder (ADHD). *J Child Neurol.* 2002;17(12):863-866.

Reason for exclusion: Cross-over no wash out. Less than seven days treatment

Gualtieri1981

- Gualtieri CT, Kanoy R, Hawk B. Growth hormone and prolactin secretion in adults and hyperactive children: Relation to methylphenidate serum levels. *Psychoneuroendocrinology.* 1981(4):331-339.

Reason for exclusion: Less than seven days treatment

Gualtieri1982

- Gualtieri CT, Wargin W, Kanoy R, et al. Clinical studies of methylphenidate serum levels in children and adults. *J Am Acad Child Psychiatry.* 1982;21(1):19-26.

Reason for exclusion: No RCT

Gualtieri1984

- Gualtieri CT, Hicks RE, Mayo JP, Schroeder SR. The persistence of stimulant effects in chronically treated children: further evidence of an inverse relationship between drug effects and placebo levels of response. *Psychopharmacology (Berl).* 1984;83(1):44-47.
- Hicks RE, Gualtieri CT, Mayo JP, Schroeder SR, Lipton MA. Methylphenidate and homeostasis: drug effects on the cognitive performance of hyperactive children. In: Bloomingdale Lewis M editor(s). *Attention Deficit Disorder: Identification, Course and Treatment Rationale*. New York, USA: SP Medical & Scientific Books, 1985:131–41.

Reason for exclusion: Less than seven days treatment

Gualtieri1985

- Gualtieri, CT, Ondrusek, M G, Finley. Attention deficit disorders in adults. *Clin Neuropharmacol.* 1985(4):343-356.

Reason for exclusion: Less than seven days treatment

Gualtieri1988a

- Gualtieri CT, Evans RW. Stimulant treatment for the neurobe-havioural sequelae of traumatic brain injury. *Brain Inj.* 1988;2(4):273-290

Reason for exclusion: No participants with ADHD

Gualtieri1988b

- Gualtieri CT, Evans RW. Motor performance in hyperactive children treated with imipramine. Perceptual and Motor Skills, 66, 763-769. *Percept Mot Skills.* 1988 ;66(3):763-9

Reason for exclusion: Medication of no interest for the present meta-analysis vs placebo

Gualtieri1991

- Gualtieri CT, Keenan PA, Chandler M. Clinical and neuropsychological effects of desipramine in children with attention deficit hyperactivity disorder. *J Clin Psychopharmacol.* 1991; 11(3):155-159.

Reason for exclusion: Medication of no interest for the present meta-analysis vs placebo

Guerdjikova2016

- Guerdjikova AI, Mori N, Blom TJ, et al. Lisdexamfetamine dimesylate in binge eating disorder: a placebo controlled trial. *Hum Psychopharmacol*. 2016 ;31(5):382-91

Reason for exclusion: Participants with binge eating disorder; No comorbid diagnosis of ADHD according to standardized criteria

Gulley1997

- Gulley V, Northup J. Comprehensive school-based behavioral assessment of the effects of methylphenidate. *J Appl Behav Anal.* 1997;30(4):627-638.

Reason for exclusion: Less than seven days treatment

Gulley2003

- Gulley V, Northup J, Hupp S, Spera S, LeVelle J, Ridgway A. Sequential evaluation of behavioral treatments and methylphenidate dosage for children with attention deficit hyperactivity disorder. *J Appl Behav Anal.* 2003;36(3):375-378.

Reason for exclusion: No RCT

Gunning2010

- Gunning WB. The efficacy of methylphenidate in children with epilepsy and ADHD: the role of dosage, epilepsy type and psychiatric comorbidity. *Epilepsia.* 2004:206.

Reason for exclusion: No RCT

Gunther2010

- Gunther T, Herpertz-Dahlmann B, Konrad K. Sex differences in attentional performance and their modulation by methylphenidate in children with attention-deficit/hyperactivity disorder. *J Child Adolesc Psychopharmacol.* 2010;20(3):179-186.

Reason for exclusion: Less than seven days treatment

H8V-FW-LTBD

- *https://assets.contentful.com/hadumfdtzsru/4vG0ZYQXfO4M4MWwOMuswu/62097a9cd4dde0e6fe5a92246a46a8b7/Atomoxetine-H8V-FW-LTBD.pdf*

Reasons for exclusion: No participants with ADHD, Open label

Hadar2017

- Hadar Y, Hocherman S, lamm O, Tirosh E. Auditory and Visual Executive Functions in Children and Response to Methylphenidate: A Randomized Controlled Trial. *J Att Disorders*, in press, 2017 1:1087054717700978. doi: 10.1177/1087054717700978

Reason for exclusion: No outcomes of interest for the present meta-analysis

Haddock1972

- Haddock ST. Usefulness of methylphenidate. *N Engl J Med.* 1972;286(7):375.

Reason for exclusion: Commentary

Haig1974

- Haig JR, Schroeder CS, Schroeder SR. Effects of methylphenidate on hyperactive children's sleep. *Psychopharmacologia.* 1974;37(4):185-188.

Reason for exclusion: No RCT

Hale2011

- Hale JB, Reddy LA, Semrud-Clikeman M, et al. Executive impairment determines ADHD medication response: implications for academic achievement. *J Learn Disabil*. 2011;44(2):196-212
- Kubas HA, Backenson EM, Wilcox G, Piercy JC, Hale JB. The effects of methylphenidate on cognitive function in children with attention-deficit/hyperactivity disorder. *Postgrad Med*. 2012;124(5):33-48.

Reason for exclusion: Less than seven days treatment; no pre cross-over data available

Hall2017(NCT02209116)

- Hall CL, Valentine AZ, Walker GM, et al. Study of user experience of an objective test (QbTest) to aid ADHD assessment and medication management: a multi-methods approach. *BMC Psychiatry.* 2017;17(1)
- https://clinicaltrials.gov/ct2/show/NCT02209116

Reason for exclusion: RCT not focused on medications

Halliday1976

- Halliday R, Rosenthal JH, Naylor H, Callaway E. Averaged evoked potential predictors of clinical improvement in hyperactive children treated with methylphenidate: an initial study and replication. *Psychophysiology.* 1976;13(5):429-440.

Reason for exclusion: Less than seven days treatment

Halliday1983

- Halliday R, Callaway E, Naylor H. Visual evoked potential changes induced by methylphenidate in hyperactive children: dose/response effects. *Electroencephalogr Clin Neurophysiol.* 1983;55(3):258-267.

Reason for exclusion: Placebo given only on one session

Halliday1984a

- Halliday R, Callaway E, Lynch M. Age, stimulant drug, and practice effects on P3 latency and concurrent reaction time. *Ann N Y Acad Sci.* 1984;425:357-361.

Reason for exclusion: No DSM/ICD criteria; Less than seven days treatment

Halliday1984b

- Halliday R, Callaway E, Rosenthal JH. The visual ERP predicts clinical response to methylphenidate in hyperactive children. *Psychophysiology.* 1984;21(1):114-121.

Reason for exclusion: Placebo given only on one session

Halperin2003

- Halperin JM, Newcorn JH, McKay KE, Siever LJ, Sharma V. Growth hormone response to guanfacine in boys with attention deficit hyperactivity disorder: a preliminary study. *J Child Adolesc Psychopharmacol.* 2003;13(3):283-294.

Reason for exclusion: No RCT

Hamarman2004

- Hamarman S, Fossella J, Ulger C, Brimacombe M, Dermody J. Dopamine receptor 4 (DRD4) 7-repeat allele predicts methylphenidate dose response in children with attention deficit hyperactivity disorder: a pharmacogenetic study. *J Child Adolesc Psychopharmacol.* 2004;14(4):564-574.

Reason for exclusion: No RCT

Hamarman2005

- Hamarman S, Ulger C, Fossella J, Brimacombe M, Dermody J. DAT-1 9R and DRD4 120 alleles do not predict ADHD stimulant response. *158th Annual Meeting of the American Psychiatric Association; 2005 May 21-26; Atlanta, GA2005*

Reason for exclusion: No RCT

Hamedi2014 (IRCT201302201556N51)

- Hamedi M, Mohammdi M, Ghaleiha A, et al. Bupropion in adults with Attention-Deficit/Hyperactivity Disorder: a randomized, double-blind study. *Acta Med Iran.* 2014;52(9):675-680.

Reason for exclusion: No usable data

Handen1996

- Handen BL, Breaux AM, Gosling A, Ploof DL, Feldman H. Efficacy of methylphenidate among mentally retarded children with attention deficit hyperactivity disorder. *Pediatrics*. 1990;86(6):922-930
- Handen BL, Feldman H, Gosling A, Breaux AM, McAuliffe S. Adverse side effects of methylphenidate among mentally retarded children with ADHD. *J Am Acad Child Adolesc Psychiatry*. 1991;30(2):241-245
- Handen BL, Breaux AM, Janosky J, McAuliffe S, Feldman H, Gosling A. Effects and noneffects on methylphenidate in children with mental retardation and ADHD. *J Am Acad Child Adolesc Psychiatry*. 1992;31(3):455-461
- Handen BL, Janosky J, McAuliffe S, Breaux AM, Feldman H. Prediction of response to methylphenidate among children with ADHD and mental retardation. *J Am Acad Child Adolesc Psychiatry*. 1994;33(8):1185-1193
- Handen BL, McAuliffe S, Janosky J, Feldman H, Breaux AM. Methylphenidate in children with mental retardation and ADHD: Effects on independent play and academic functioning. *J Dev Phys Disabil.* 1995;7(2):91-103.
- Handen BL, McAuliffe S, Caro-Martinez L. Stimulant medication effects on learning in children with mental retardation and ADHD. *J Dev Phys Disabil.* 1996;8(4):335-346
- Follow-up in: Handen BL, Janosky J, McAuliffe S. Long-term follow-up of children with mental retardation borderline intellectual functioning and ADHD. *J Abnorm Child Psychol.* 1997;25(4):287-295.

Reason for exclusion: Cross-over without wash out; no pre cross-over data available

Handen1999

- Handen BL, Feldman HM, Lurier A, Murray PJ. Efficacy of methylphenidate among preschool children with developmental disabilities and ADHD. *J Am Acad Child Adolesc Psychiatry.* 1999;38(7):805-812.

Reason for exclusion: Preschoolers

Handen2000

- Handen BL, Johnson CR, Lubetsky M. Efficacy of methylphenidate among children with autism and symptoms of attention-deficit hyperactivity disorder. *J Autism Dev Disord.* 2000;30(3):245-255.

Reason for exclusion: No DSM/ICD criteria

Handen2008

- Handen BL, Sahl R, Hardan AY. Guanfacine in children with autism and/or intellectual disabilities. *J Dev Behav Pediatr.* 2008;29(4):303-308.

Reason for exclusion: Participants resistant to stimulants

Handen2015 (NCT00844753; previously NCT00699205)

- Handen BL, Aman MG, Arnold LE, et al. Atomoxetine, Parent Training, and Their Combination in Children With Autism Spectrum Disorder and Attention-Deficit/Hyperactivity Disorder. *J Am Acad Child Adolesc Psychiatry.* 2015;54(11):905-915.
- Hollway JA, Aman MG, Mendoza-Burcham MI, et al. Caregiver Satisfaction with a Multisite Trial of Atomoxetine and Parent Training for Attention-Deficit/Hyperactivity Disorder and Behavioral Noncompliance in Children with Autism Spectrum Disorder. *J Child Adolesc Psychopharmacol.* Jan 21 2016. *J Child Adolesc Psychopharmacol.* 2016;26(9):807-814
- Smith T, Aman MG, Arnold LE, et al. Atomoxetine and Parent Training for Children With Autism and Attention-Deficit/Hyperactivity Disorder: A 24-Week Extension Study. *J Am Acad Child Adolesc Psychiatry.* 2016;55:868-876.
- https://clinicaltrials.gov/ct2/show/NCT00844753

Reason for exclusion: No DSM/ICD criteria

Hanisch2004

- Hanisch C, Konrad K, Gunther T, Herpertz-Dahlmann B. Age-dependent neuropsychological deficits and effects of methylphenidate in children with attention-deficit/hyperactivity disorder: a comparison of pre- and grade-school children. *J Neural Transm.* 2004;111(7):865-881.

Reason for exclusion: Less than seven days treatment

Hanlon2009

- Hanlon MC, Karayanidis F, Schall U. Intact sensorimotor gating in adult attention deficit hyperactivity disorder. *Int J Neuropsychopharmacol.* 2009;12(5):701-707.

Reason for exclusion: On vs off med for 24 h

Hao2005

- Hao XR, Cui WB. Efficacy of olanzapine versus methylphenidate treatment for childhood hyperkinetic syndrome. [Chinese]. *Chinese Journal of Clinical Rehabilitation.* 2005;9(48):174-175.

Reason for exclusion: One drug of interest vs one drug of no interest for the present meta-analysis

Hart-Santora1992

- Hart-Santora D, Hart LL. Clonidine in attention deficit hyperactivity disorder. *Ann Pharmacother.* 1992;26(1):37-39.

Reason for exclusion: Review

Harvanko2016

- Harvanko A, Martin C, Lile J, Kryscio R, Kelly TH. Individual differences in the reinforcing and subjective effects of d-amphetamine: Dimensions of impulsivity. *Exp Clin Psychopharmacol.* 2016;24:436-446.

Reason for exclusion: No participants with ADHD

Hashemian2011

- Hashemian F, Mohammadian S, Riahi F, Ghaeli P, Ghodsi D. A comparison of the effects of reboxetine and placebo on reaction time in adults with Attention Deficit-Hyperactivity Disorder (ADHD). *Daru.* 2011;19(3):231-235.

Reason for exclusion: No drug of interest for the present meta-analysis vs. placebo

Hawk2003

- Hawk LW, Jr., Yartz AR, Pelham WE, Jr., Lock TM. The effects of methylphenidate on prepulse inhibition during attended and ignored prestimuli among boys with attention-deficit hyperactivity disorder. *Psychopharmacology (Berl).* 2003;165(2):118-127.

Reason for exclusion: Less than seven days treatment

Hazel-Fernandez2006

- Hazel-Fernandez LA. Effects of methylphenidate on the executive function performance of african american children with attention-deficit hyperactivity disorder. *Dissertation Abstracts International: Section B: The Sciences and Engineering.* 2004;64(12-B):6329.
- Hazel-Fernandez LA, Klorman R, Wallace JM, Cook S. Methylphenidate improves aspects of executive function in African American children with ADHD. *J Atten Disord.* 2006;9(4):582-589.

Reason for exclusion: Less than seven days treatment

Hazell2003

- Hazell PL, Stuart JE. A randomized controlled trial of clonidine added to psychostimulant medication for hyperactive and aggressive children. *J Am Acad Child Adolesc Psychiatry.* 2003;42(8):886-894.

Reason for exclusion: Clonidine (or placebo) + methylphenidate

Head2010

- Head TK. Evaluation of medication effects on academic performance, sleep, and core ADHD symptoms in children. *Dissertation Abstracts International: Section B: The Sciences and Engineering.* 2010;71(6-B):3936.

Reason for exclusion: Four children, no mention of randomization

Heber2016

- Heber E, Halperin J, Krone B, Bedard AC, Ivanov I, Newcorn JH. Cognitive and emotional control in youth with attention-deficit/ hyperactivity disorder, and the impact of stimulant and non-stimulant treatment. *J Am Acad Child Adolesc Psychiatry.* 2016;55 (10 Supplement 1):S188.

Reason for exclusion: Abtract only (conference proceeding); contacted Drs Halperin and Newcorn to query about study status; reply: in process of updating clinicaltrila.gov, no further data available

Hechtman1984

- Hechtman L, Weiss G, Perlman T. Young adult outcome of hyperactive children who received long-term stimulant treatment. *J Am Acad Child Psychiatry.* 1984;23(3):261-269.

Reason for exclusion: No RCT

Hechtman2004

- Hechtman L, Abikoff H, Klein RG, et al. Children with ADHD treated with long-term methylphenidate and multimodal psychosocial treatment: impact on parental practices. *J Am Acad Child Adolesc Psychiatry.* 2004;43(7):830-838.

Reason for exclusion: No study design as per protocol

Hechtman2011

- Hechtman L. Treatment of ADHD in patients unresponsive to methylphenidate. *J Psychiatry Neurosci.* 2011;36(3):216.

Reason for exclusion: Case report

Heil2002

- Heil SH, Holmes HW, Bickel WK, et al. Comparison of the subjective, physiological, and psychomotor effects of atomoxetine and methylphenidate in light drug users. *Drug Alcohol Depend.* 2002;67(2):149-156.

Reason for exclusion: No participants with ADHD

Heiligenstein2003

- Heiligenstein J, Michelson D, Wernicke J, et al. Atomoxetine and pregnancy - Reply. *J Am Acad Child Adolesc Psychiatry.* 2003;42(8):884-885.

Reason for exclusion: No RCT

Heiman1983

- Heiman EM. Use of stimulants for alcoholic patients with attention deficit disorder. *Am J Psychiatry.* 1983;140(9):1272.

Reason for exclusion: Case report

Heinrich2013

- Heinrich H, Studer P, Moll GH, Kratz O. Methylphenidate vs atomoxetine: personalized medicine in attention-deficit/hyperactivity disorder. *JAMA Psychiatry.* 2013;70(5):545.

Reason for exclusion: Commentary

Heinzerling2011

- Heinzerling LM, Pichler W, Anliker MD. Acute generalized exanthematous pustulosis induced by methylphenidate: a new adverse effect. *Arch Dermatol.* 2011;147(7):872-873.

Reason for exclusion: Case report

Heiser2004

- Heiser P, Frey J, Smidt J, et al. Objective measurement of hyperactivity, impulsivity, and inattention in children with hyperkinetic disorders before and after treatment with methylphenidate. *Eur Child Adolesc Psychiatry.* 2004;13(2):100-104.

Reason for exclusion: No RCT

Hellwig-Brida2011

- Hellwig-Brida S, Daseking M, Keller F, Petermann F, Goldbeck L. Effects of methylphenidate on intelligence and attention components in boys with attention-deficit/hyperactivity disorder. *J Child Adolesc Psychopharmacol.* 2011;21(3):245-253.

Reason for exclusion: No RCT

Helsel1989

- Helsel WJ, Hersen M, Lubetsky MJ, Fultz SA, Sisson L, Harlovic CH. Stimulant drug treatment of four multihandicapped children using a randomized single-case design. *Journal of the Multihandicapped Person.* 1989;2(2):139-154.

Reason for exclusion: N-of-1 trial

Helseth2015

- Helseth SA, Waschbusch DA, Gnagy EM, et al. Effects of behavioral and pharmacological therapies on peer reinforcement of deviancy in children with ADHD-only, ADHD and conduct problems, and controls. *J Consult Clin Psychol.* 2015;83(2):280-292.

Reason for exclusion: Less than seven days treatment

Henderson2004a

- Henderson TA. Mania induction associated with atomoxetine. *J Clin Psychopharmacol.* 2004;24(5):567-568

Reason for exclusion: Case report

Henderson2004b

- Henderson TA, Hartman K. Aggression, mania, and hypomania induction associated with atomoxetine. Pediatrics. 2004;114(3):895-896.

Reason for exclusion: No RCT

Henker1979

- Henker B, Whalen CK, Collins BE. Double-blind and triple-blind assessments of medication and placebo responses in hyperactive children. *J Abnorm Child Psychol.* 1979;7(1):1-13.

Reason for exclusion: No DSM/ICD criteria

Hensch2010

- Hensch T, Himmerich H, Ulrich H. Stimulants in bipolar disorder: Beyond common beliefs. *CNS Spectr.* 2010;15(7):469-470.

Reason for exclusion: Commentary

Heriot2008

- Heriot SA, Evans IM, Foster TM. Critical influences affecting response to various treatments in young children with ADHD: a case series. *Child Care Health Dev.* 2008;34(1):121-133.

Reason for exclusion: Children aged between 3 and 5.9 years

Hicks1989

- Hicks RE, Mayo JP, Jr., Clayton CJ. Differential psychopharmacology of methylphenidate and the Child Neuropsychol of childhood hyperactivity. *Int J Neurosci.* 1989;45(1-2):7-32.

Reason for exclusion: No mention of randomization; not possible to contact authors (no available email address); not possible to gather pre-cross over data

Hinshaw1984a

- Hinshaw SP, Henker B, Whalen CK. Self-control in hyperactive boys in anger-inducing situations: effects of cognitive-behavioral training and of methylphenidate. *J Abnorm Child Psychol.* 1984;12(1):55-77.

Reason for exclusion: No DSM/ICD criteria; Co-treatment

Hinshaw1984b

- Hinshaw SP, Henker B, Whalen CK. Cognitive-behavioral and pharmacologic interventions for hyperactive boys: comparative and combined effects. *J Consult Clin Psychol.* 1984;52(5):739-749.

Reason for exclusion: Less than seven days treatment; co-intervention

Hinshaw1989a

- Hinshaw SP, Henker B, Whalen CK, Erhardt D, Dunnington RE, Jr. Aggressive, prosocial, and nonsocial behavior in hyperactive boys: dose effects of methylphenidate in naturalistic settings. *J Consult Clin Psychol.* 1989;57(5):636-643.

Reason for exclusion: Co-treatment

Hinshaw1989b

- Hinshaw SP, Buhrmester D, Heller T. Anger control in response to verbal provocation: effects of stimulant medication for boys with ADHD. *J Abnorm Child Psychol.* 1989;17(4):393-407.

Reason for exclusion: Less 7 days; co-treatment

Hinshaw1992

- Hinshaw SP, Heller T, McHale JP. Covert antisocial behavior in boys with attention-deficit hyperactivity disorder: external validation and effects of methylphenidate. *J Consult Clin Psychol.* 1992;60(2):274-281.

Reason for exclusion: Less than seven days treatment (2 sessions)

Hirayama2004

- Hirayama S, Hamazaki T, Terasawa K. Effect of docosahexaenoic acid-containing food administration on symptoms of attention-deficit/hyperactivity disorder - A placebo-controlled double-blind study. *Eur J Clin Nutr.* 2004;58(3):467-473.

Reason for exclusion: Compound of no interest for the present meta-analysis (phosphatidylserine) vs placebo

Hirvikoski 2011

- Hirvikoski T, Waaler E, Alfredsson J, et al. Reduced ADHD symptoms in adults with ADHD after structured skills training group: Results from a randomized controlled trial. *Behav Res Ther.* 2011;49(3):175-185.

Reason for exclusion: No treatment of interest for the present meta-analysis

Hisock 1979

- Hisock M, Kinsbourne M, Caplan B, Swanson JM. Auditory attention in hyperactive children: Effects of stimulant medication on dichotic listening performance. *J Abnorm Psychol.* 1979(1):27-32.

Reason for exclusion: Less than seven days treatment

Hoare2005

- Hoare P, Remschmidt H, Medori R, et al. 12-month efficacy and safety of OROS MPH in children and adolescents with attention-deficit/hyperactivity disorder switched from MPH. *Eur Child Adolesc Psychiatry.* 2005;14(6):305-309.

Reason for exclusion: Open label

Hoekstra2011

- Hoekstra PJ. Is there potential for the treatment of children with ADHD beyond psychostimulants? *Eur Child Adolesc Psychiatry.* 2011;20(9):431-432.

Reason for exclusion: Commentary

Hoeppner1997

- Hoeppner J-AB, Hale J, Bradley A, et al. A clinical protocol for determining methylphenidate dosage levels in ADHD. *J Atten Disord.* 1997;2(1):19-30

Reason for exclusion: Cross-over without wash out; No pre cross-over data available

Hoffman1974

- Hoffman SP, Engelhardt DM, Margolis RA, Polizos P, Waizer J, Rosenfeld R. Response to methylphenidate in low socioeconomic hyperactive children. *Arch Gen Psychiatry.* 1974;30(3):354-359.

Reason for exclusion: No RCT

Hood2005

- Hood J, Baird G, Rankin PM, Isaacs E. Immediate effects of methylphenidate on cognitive attention skills of children with attention-deficit-hyperactivity disorder. *Developmental Medicine & Child Neurology.* 2005;47(6):408-414.

Reason for exclusion: No RCT

Horn1991

- Horn WF, Ialongo NS, Pascoe JM, et al. Additive effects of psychostimulants, parent training, and self-control therapy with ADHD children. *J Am Acad Child Adolesc Psychiatry.* 1991;30(2):233-240.
- Follow-up in: Ialongo NS, Horn WF, Pascoe JM, et al. The effects of a multimodal intervention with attention-deficit hyperactivity disorder children: a 9-month follow-up. *J Am Acad Child Adolesc Psychiatry.* 1993;32(1):182-189.

Reason for exclusion: From Storebo et al (2015): "Email correspondence with study authors: August 2013. Not possible to receive supple-mental information or data through personal email correspondence with study authors. They do not recommend inclusion of the study in this review because of problems with the design and methods used at the time the study was carried out."

Hornig-Rohan2002

- Hornig-Rohan M, Amsterdam JD. Venlafaxine versus stimulant therapy in patients with dual diagnosis ADD and depression. *Prog Neuropsychopharmacol Biol Psychiatry.* 2002;26(3):585-589.

Reason for exclusion: No RCT

Horrigan2001

- Horrigan JP, Barnhill LJ, Kohli RR. Adderall, the atypicals, and weight gain. *J Am Acad Child Adolesc Psychiatry.* 2001;40(6):620.

Reason for exclusion: Case report

Hu2015

- Hu LY, Lin YL, Chang HS, Lu T, Lin WS. Low-dose Methylphenidate Monotherapy for Features of Attention-Deficit/Hyperactivity Disorder Secondary to Hereditary Cerebellar Ataxia. *CNS Neurosci Ther.* 2015;21(8):672-673.

Reason for exclusion: Case report

Huang2002

- Huang MM, Huang GS. Effects of venlafaxine and ritalin in treatment of attention deficit/hyperactivity disorder in children. *Health Psychology Journal.* 2002(1):39-40.

Reason for exclusion: Only abstract available; not possible to contact authors

Huessy1970

- Huessy HR, Wright, AL. The use of imipramine in children's behavior disorders. *Acta Paedopsychiatr.* 1970; 37: 194-199

Reason for exclusion: No RCT

Huestos1975

- Huestos RD, Arnold L, Smeltzer DJ. Caffeine versus methylphenidate and d-amphetamine in minimal brain dysfunctin: A double-blind comparison. *Am J Psychiatry.* 1975;132(8):868-870.

Reason for exclusion: NO DSM/ICD criteria

Hulvershorn2012

- Hulvershorn LA, Hummer T, Wang Y, Loth A, Anand A. The Impact of Methylphenidate on Corticolimbic Functional Connectivity in Children with ADHD and Chronic Irritability. *Biol Psychiatry.* 2012;71:193S.

Reason for exclusion: No RCT

Humphries1978

- Humphries T, Kinsbourne M, Swanson J. Stimulant effects on cooperation and social interaction between hyperactive children and their mothers. *J Child Psychol Psychiatr.* 1978(1):13-22.

Reason for exclusion: NO DSM/ICD criteria

Humphries1979

- Humphries T, Swanson J, Kinsbourne M, Yiu L. Stimulant effects on persistence of motor performance of hyperactive children. *J Pediatr Psychol.* 1979(1):55-66.

Reason for exclusion: No DSM/ICD criteria

Hunt1985

- Hunt RD, Minderaa RB, Cohen DJ. Clonidine benefits children with attention deficit disorder and hyperactivity: report of a double-blind placebo-crossover therapeutic trial. *J Am Acad Child Psychiatry.* 1985;24(5):617-629
- Hunt RD, Minderaa RB, Cohen DJ. The therapeutic effect of clonidine in attention deficit disorder with hyperactivity: a comparison with placebo and methylphenidate. *Psychopharmacol Bull.* 1986;22(1):229-236.

Reason for exclusion: No DSM/ICD criteria as per protocol

Hunt1987

- Hunt RD. Treatment effects of oral and transdermal clonidine in relation to methylphenidate: an open pilot study in ADD-H. *Psychopharmacol Bull.* 1987;23(1):111-114.

Reason for exclusion: Open label

Hurst1978

- Hurst DL. Effect of methylphenidate on academic progress. *J Ped.* 1978;92(1):168.

Reason for exclusion: Commentary

Hurt2011

- Hurt RD, Ebbert JO, Croghan IT, Schroeder DR, Sood A, Hays JT. Methylphenidate for treating tobacco dependence in non-attention deficit hyperactivity disorder smokers: a pilot randomized placebo-controlled trial. *J Negat Results Biomed.* 2011;10:1.

Reason for exclusion: No participants with ADHD

Husarova2014

- Husarova V, Bittsansky M, Ondrejka I, Dobrota D. Prefrontal grey and white matter neurometabolite changes after atomoxetine and methylphenidate in children with attention deficit/hyperactivity disorder: A (1)H magnetic resonance spectroscopy study. *Psychiatry Res.* 2014;222(1-2):75-83.

Reason for exclusion: Not clear if double-blind; authors contacted but no reply

Hwang2013

- Hwang JW, Kim B, Kim Y, et al. Methylphenidate-osmotic-controlled release oral delivery system treatment reduces parenting stress in parents of children and adolescents with attention-deficit/hyperactivity disorder. *Hum Psychopharmacol*. 2013;28(6):600-607.

Reason for exclusion: No RCT

Ialongo1994

- Ialongo NS, Lopez M, Horn WF, Pascoe JM, Greenberg G. Effects of psychostimulant medication on self-perceptions of competence, control, and mood in children with attention deficit hyperactivity disorder. *J Clin Child Psychol.* 1994;23(2):161-173.

Reason for exclusion: The 48 subjects were selected from the larger (randomized) study; this is problematic for the NMA in terms of transitivity assumption

Ibay2003

- Ibay AD, Bascelli LM, Graves RS, Hill J. Clinical inquiries. Does increasing methylphenidate dose aid symptom control in ADHD? *J Fam Pract.* 2003;52(5):400, 403.

Reason for exclusion: Review/commentary

Ibel1992

- Ibel S. Auditory event-related potentials in attention deficit hyperactivity *disorder [Ph.D.]. Ann Arbor, State University of New York at Stony Brook; 1992.*

Reason for exclusion: Single dose

Ickowicz2002

- Ickowicz A. Bupropion-methylphenidate combination and grand mal seizures. *Can J Psychiatry.* 2002;47(8):790-791.

Reason for exclusion: Case report

Idiazabal-Alecha2005

- Idiazabal-Alecha MA, Rodriguez-Vazquez S, Guerrero-Gallo D, Vicent-Sardinero X. [The value of cognitive evoked potentials in assessing the effectiveness of methylphenidate treatment in children with attention deficit hyperactivity disorder]. *Rev Neurol.* 2005;40(Suppl 1): S37-42.

Reason for exclusion: No RCT

Ince2015

- Ince Tasdelen B, Karakaya E, Oztop DB. Effects of Atomoxetine and Osmotic Release Oral System-Methylphenidate on Executive Functions in Patients with Combined Type Attention-Deficit/Hyperactivity Disorder. *J Child Adolesc Psychopharmacol.* 2015;25(6):494-500.
- Ince Tasdelen B, Karakaya E, Oztop D. Effects of atomoxetine and OROS-MPHON executive functions in patients with combined type attention deficit hyperactivity disorder. *Eur Child Adolesc Psychiatry* .2015; (Suppl 1): S247-S249.

Reason for exclusion: Open label

IRCT138808122660N1

- http://www.irct.ir/searchresult.php?id=2660&number=1

Reasons for exclusion: Treatment of no interest for the present meta-analysis (venlafaxine) vs placebo

IRCT201105096424N1

- http://www.irct.ir/searchresult.php?id=6424&number=1

Reasons for exclusion: No participants with ADHD

IRCT201612253979N6

- http://www.irct.ir/searchresult.php?id=3979&number=6

Reasons for exclusion: Abstract only; contacted author to query study status/data no reply

IRCT201701131556N94

- http://www.irct.ir/searchresult.php?id=1556&number=94

Reasons for exclusion: Medication of interest (methylphenidate) vs medication of no interest (buspirone) for the present meta-analysis

IRCT2016053028182N1

- http://www.irct.ir/searchresult.php?id=28182&number=1

Reasons for exclusion: Medication of no interest for the present meta-analysis (memantine) vs placebo

IRCT2016060128182N2

- http://www.irct.ir/searchresult.php?id=28182&number=2

Reasons for exclusion: MPH vs PMH+omega 3 supll

IRCT138803122000N1

- http://www.irct.ir/searchresult.php?id=2000&number=1

Reasons for exclusion: methylphenidate+PUFA vs methylphenidate +Placebo

IRCT138804132000N2

- http://www.irct.ir/searchresult.php?id=2000&number=2

Reasons for exclusion: Single blind

IRCT138810193029N1

- http://www.irct.ir/searchresult.php?id=3029&number=1

Reasons for exclusion: Single blind

IRCT201012205427N1

- http://www.irct.ir/searchresult.php?id=5427&number=1

Reasons for exclusion: No randomised

IRCT201104116168N1

- http://www.irct.ir/searchresult.php?id=6168&number=1

Reasons for exclusion: Medication of interest for the present meta-analysis vs medication of no interest, no placebo

IRCT201104166201N1

- http://www.irct.ir/searchresult.php?id=6201&number=1

Reasons for exclusion: Arms of no interest for the present meta-analysis: Ritalin +placebo vs Zinc+placebo vs omega3 +placebo

IRCT201108067237N1

- http://www.irct.ir/searchresult.php?id=7237&number=1

Reasons for exclusion: Not randomized

IRCT201110127462N

- http://www.irct.ir/searchresult.php?id=7462&number=2

Reasons for exclusion: Medication of interest for the present meta-analysis vs medication of no interest, no placebo

IRCT201203167462N4

- http://www.irct.ir/searchresult.php?id=7462&number=4

Reasons for exclusion: Arms of no interest for the present meta-analysis: methypheniate+placebo vs methylphenidate plus melatonin

IRCT201204188317N1

- http://www.irct.ir/searchresult.php?id=8317&number=1

Reasons for exclusion: Arms of no interest for the present meta-analysis: methyphenidate+propanololo at different dosages

IRCT201205157462N7

- http://www.irct.ir/searchresult.php?id=7462&number=7

Reasons for exclusion: Medication of interest for the present meta-analysis vs medication of no interest, no placebo

IRCT201208058317N2

- http://www.irct.ir/searchresult.php?id=8317&number=2

Reasons for exclusion: Arms of no interest for the present meta-analysis: methylphenidate+memenatidine vs methylphenidate+placebo

IRCT201303036923N2

- http://www.irct.ir/searchresult.php?id=6923&number=2

Reasons for exclusion: Arms of no interest for the present meta-analysis: methylphenidate+piracetam vs methylphenidate+placebo

IRCT201305142531N2

- http://www.irct.ir/searchresult.php?id=2531&number=2

Reasons for exclusion: Single blind; arms of no interest (methylphenidate with or without massage)

IRCT201306189175N5

- http://www.irct.ir/searchresult.php?id=9175&number=5

Reasons for exclusion: Not blinded; medication of interest vs medication of no interest for the present meta-analysis

IRCT2012101510363N2

- http://www.irct.ir/searchresult.php?id=10363&number=2

Reasons for exclusion: Medication of interest for the present meta-analysis vs medication of no interest, no placebo

IRCT2013012712302N1

- http://www.irct.ir/searchresult.php?id=12302&number=1

Reasons for exclusion: Not blinded

IRCT2013090914598N1

- http://www.irct.ir/searchresult.php?id=14598&number=1

Reasons for exclusion: Arms of no intersst for the present meta-analysis: L-carnitine or placebo

IRCT2014062318192N1

- http://www.irct.ir/searchresult.php?id=18192&number=1

Reasons for exclusion: No intervention of interest for the present meta-analysis

IRCT2014112214333N25

- http://www.irct.ir/searchresult.php?id=14333&number=25

Reasons for exclusion: No participants with ADHD

IRCT201203167462N5

- http://www.irct.ir/searchresult.php?id=7462&number=5

Reasons for exclusion: No arms of interest for the present meta-analysis: Intervention group1: Methylphenidate (Ritalin: 1mg/kg) combined with Cyproheptadin (Razi: 12 mg/day- 4mg before each meal) . Intervention 2: Intervention group2: Methylphenidate (Ritalin: 1mg/kg) combined with Folic acid (Alhavi: 5 mg/day)

IRCT201211051743N10

- http://www.irct.ir/searchresult.php?id=1743&number=10

Reasons for exclusion: No arms of interest for the present meta-analysis: methylphenidate with maximum dose of 30 mg per day and placebo for 8 weeks. Intervention 2: methylphenidate with maximum dose of 30 mg per day and risperidone with maximum dose of 1 mg per day for 8 weeks

IRCT201512262639N17

- http://www.irct.ir/searchresult.php?id=2639&number=17

Reasons for exclusion: No arms of interest for the present meta-analysis: Intervention 1: Tablet of 10 mg of Ritalin from Novartis Co, Switzerland in dosage of 2o mg/day divided in two doses and tablet of 50 mg ferrous sulfate from Daroupakhsh Co, Iran in dosage of 2 mg/kg/day single dose for three months. Intervention 2: Tablet of ten mg of Ritalin from Novartis Co, Switzerland in dosage of 2o mg/day divided in two dose for three months

IRCT2014062116465N4

- http://www.irct.ir/searchresult.php?id=16465&number=4

Reasons for exclusion: No arms of inters for the present meta-analysis : Intervention 1: Intervention group: In this group a high protein diet with 35% of total calories from protein will be administered by a dietitian plus methylphenidate (Rytalyn- 1 mg per kg weight of the child). Intervention 2: Control group: In this group no intervention will be done on diet, but children will receive standard treatment with methylphenidate (Rytalyn- 1 mg per kg weight of the child).

IRCT2014062118181N1

- http://www.irct.ir/searchresult.php?id=18181&number=1

Reasons for exclusion: Not blinded; no arms of interest for the present meta-analysis

IRCT2014111519958N1

- http://www.irct.ir/searchresult.php?id=19958&number=1

Reasons for exclusion: No arms of interest for the present meta-analysis: Intervention 1: In control group tablet Ritalin 1mg/kg plus tablet placebo Ginkgo biloba for 4 weeks.. Intervention 2: In intervention group tablet Ritalin 1mg/kg plus tablet Ginkgo biloba 80-120 mg/day(below 30 kg 80mg/day and more than 30 kg 120mg/day) for 4 weeks .

IRCT2015050922165N1

- http://www.irct.ir/searchresult.php?id=22165&number=1

Reasons for exclusion: No arms of interst for the present meta-analysis: Intervention 1: Intervention Group: Sweet almond syrup 5 cc/TDS and an ineffective tablet as placebo for 8 weeks . Intervention 2: Control Group: Ritalin 1mg/kg/day and an ineffective syrup as placebo 5cc/TDS for 8 weeks

IRCT2015092624209N1

- http://www.irct.ir/searchresult.php?id=24209&number=1

Reasons for exclusion: No arms of interest for the present meta-analysis: methylphenidate+supplements vs methylphenidate

IRCT2015092724209N2

- http://www.irct.ir/searchresult.php?id=24209&number=2

Reasons for exclusion: No arms of interest for the present meta-analysis: Methylphenidate plus multi chain polyunsaturated fatty acids vs methylphenidate+placebo

IRCT2016021026505N1

- http://www.irct.ir/searchresult.php?id=26505&number=1

Reasons for exclusion: Not blinded; no arms of interest for the present meta-analysis: Parent training vs Methylphenidate or risperidone

IRCT2016042027506N1

- http://www.irct.ir/searchresult.php?id=27506&number=1

Reasons for exclusion: No arms of interest for the present meta-analysis : Methylphenidate+donezepil vs methylphenidate+placebo

IRCT2016050918927N2

- http://www.irct.ir/searchresult.php?id=18927&number=2

Reasons for exclusion: No arms of interest for the present meta-analysis: Methylphenidate+omega3 vs methylphenidate+placebo

IRCT2016040927304N1

- http://www.irct.ir/searchresult.php?id=27304&number=1

No arms of interest for the present meta-analysis (methylphenidate +folic acid vs. methylphenidate +placebo)

IRCT2016081229310N1

- http://www.irct.ir/searchresult.php?id=29310&number=1

Reasons for exclusion: No participants with ADHD

IRCT201212012269N2

- http://www.irct.ir/searchresult.php?id=2269&number=2

Reasons for exclusion: Intervention 1: Ferrous sulfate (brand name: Ferrous sulfate), 80 mg oral tablet, manufactured by Shahre Daru Co., daily for 3 months. Intervention 2: Placebo, oral tablet, manufactured by Shahre Daru Co., contains: Lactose- Avicel- Polyvinylpyrrolidone (PVP), daily for 3 months

IRCT2015070623099N1

- http://www.irct.ir/searchresult.php?id=23099&number=1

Reasons for exclusion: Intervention 1: Intervention Groups: cyproheptadine, tablets 4 mg, orally, two half a tablet twice a day for 2 months. Intervention 2: In the control group: placebo, tablet, taken orally, once daily for two months

Ishii-Takahashi2015 (JPRN-UMIN000001270)

- Ishii-Takahashi A, Takizawa R, Nishimura Y, et al. Neuroimaging-aided prediction of the effect of methylphenidate in children with attention deficit hyperactivity disorder-a randomized controlled trial. *Biol Psychiatry.* 2014;75 (9), Suppl 1: 231S.
- Ishii-Takahashi A, Takizawa R, Nishimura Y, et al. Neuroimaging-aided prediction of the effect of methylphenidate in children with attention-deficit hyperactivity disorder: A randomized controlled trial. *Neuropsychopharmacology.* 2015;40(12):4676-4685.
- Ishii-Takahashi A, Takizawa R, Nishimura Y, et al. Erratum: Neuroimaging-aided prediction of the effect of methylphenidate in children with attention-deficit hyperactivity disorder: A randomized controlled trial. *Neuropsychopharmacology.* 2015;40(12):2852.
- http://www.umin.ac.jp/ctr/index.htm

Reason for exclusion: Single dose trial

ISRCTN76063113

- http://isrctn.com/ISRCTN76063113

Reasons for exclusion: Diet group vs control group

ISRCTN25691213

- http://isrctn.org/ISRCTN25691213

Reasons for exclusion: Open label

ISRCTN27103516

- http://isrctn.org/ISRCTN27103516

Reasons for exclusion: No participants with ADHD

ISRCTN73911400

- http://isrctn.org/ISRCTN73911400

Reasons for exclusion: Open label, arms of no interest for the present meta-analysis

ISRCTN77828247

- http://isrctn.org/ISRCTN77828247

Reasons for exclusion: No outcomes of interest for the present meta-analysis; written to author to inquire about study status and other possible relevant outcomes; no reply

ISRCTN52376787

- http://isrctn.com/ISRCTN52376787

Reasons for exclusion: Methylphenidate vs. no methylphenidate

ISRCTN11727351

- http://isrctn.com/ISRCTN11727351

Reasons for exclusion: No design/arms of interest for the present meta-analysis

ISRCTN68819261

- http://isrctn.com/ISRCTN68819261

Reasons for exclusion: No pharmacological treatments

ISRCTN76063113

- http://isrctn.com/ISRCTN76063113

Reasons for exclusion: Diet group vs control group

ISRCTN76187185

- http://isrctn.com/ISRCTN76187185

Reasons for exclusion: No treatment of interest for the present meta-analysis; single blind

ISRCTN82524080

- http://isrctn.com/ISRCTN82524080

Reasons for exclusion: No treatment of interest for the present meta-analysis

ISRCTN75690327

- http://isrctn.com/ISRCTN75690327

Reasons for exclusion: No RCT

ISRCTN57997252

- http://isrctn.com/ISRCTN57997252

Reasons for exclusion: No participants with ADHD

ISRCTN20127069

- http://isrctn.com/ISRCTN20127069

Reasons for exclusion: No participants with ADHD

ISRCTN27741572

- http://isrctn.com/ISRCTN27741572

Reasons for exclusion: No interventions of interest for the present meta-analysis

ISRCTN31004502

- http://isrctn.com/ISRCTN31004502

Reasons for exclusion: No participants with ADHD

ISRCTN33930984

- http://isrctn.com/ISRCTN33930984

Reasons for exclusion: No pharmacological interventions

ISRCTN49671147

- http://isrctn.com/ISRCTN49671147

Reasons for exclusion: No pharmacological interventions

ISRCTN50834814

- http://isrctn.com/ISRCTN50834814

Reasons for exclusion: No pharmacological interventions

ISRCTN03732556

- http://isrctn.com/ISRCTN03732556

Reasons for exclusion: No pharmacological treatment (CBT +TAU vs CTB alone)

ISRCTN05214203

- http://isrctn.org/ISRCTN05214203

Reasons for exclusion: Written to author to enquire about study status; no answer

ISRCTN44227400

- http://isrctn.org/ISRCTN44227400

Reasons for exclusion: Open label

ISRCTN16827947

- http://isrctn.com/ISRCTN16827947

Reasons for exclusion: Ongoing

Ivanov2013

- Ivanov I, Liu X, Clerkin S, Schulz K, Fan J, Newcorn J. Methylphenidate and brain activity in a reward/ conflict paradigm. *Neuropsychopharmacology.* 2013;38:S143-S144.

Reason for exclusion: Single dose

Jackson1988

- Jackson SL. The effects of Ritalin on the postrotatory nystagmus response of hyperactive children with attention deficit disorders *[Ph.D.]. Ann Arbor, Texas Woman's University; 1988.*

Reason for exclusion: Single dose

Jacobi-Polishook2009 (NCT00485797)

- Jacobi-Polishook T, Shorer Z, Melzer I. The effect of methylphenidate on postural stability under single and dual task conditions in children with attention deficit hyperactivity disorder - a double blind randomized control trial. *J Neurol Sci.* 2009;280(1-2):15-21.
- https://clinicaltrials.gov/ct2/show/NCT00485797

Reason for exclusion: Authors confirmed it is a single dose study

Jacobson-Kram2008

- Jacobson-Kram D, Mattison D, Shelby M, Slikker W, Tice R, Witt K. Methylphenidate and chromosome damage. *Cancer Lett.* 2008;260(1-2):216-218.

Reason for exclusion: Letter to the editor, no original empirical data

Jaffee2009

- Jaffee WB, Bailey GL, Lohman M, Riggs P, McDonald L, Weiss RD. Methods of recruiting adolescents with psychiatric and substance use disorders for a clinical trial. *Am J Drug Alcohol Abuse.* 2009;35(5):381-384.

Reason for exclusion: No RCT; concomitant behavioral therapy

Jain2007

- Jain U, Hechtman L, Weiss M, et al. Efficacy of a novel biphasic controlled-release methylphenidate formula in adults with attention-deficit/hyperactivity disorder: results of a double-blind, placebo-controlled crossover study. *J Clin Psychiatry.* 2007;68(2):268-277.
- A poster based on this study was presented at the joint 52nd annual meeting of the American Academy of Child and Adolescent Psychiatry/ Canadian Academy of Child and Adolescent Psychiatry; October 18–23, 2005; Toronto, Ontario, Canada.

Reason for exclusion: Cross-over without wash out; no pre cross-over data

Jakala1999

- Jakala P, Riekkinen M, Sirvio J, et al. Guanfacine, but not clonidine, improves planning and working memory performance in humans. *Neuropsychopharmacology.* 1999;20(5):460-470.

Reason for exclusion: No participants with ADHD

James2001

- James RS, Walter JM, Sharp WS, Castellanos FX. Comparative efficacy of 3 amphetamine preparations in children with adhd. *39th Annual Meeting of the American College of* Neuropsychopharmacology. *2000; Dec 10-14; San Juan, Puerto Rico.* 2000.
- James RS, Sharp WS, Bastain TM, et al. Double-blind, placebo-controlled study of single-dose amphetamine formulations in ADHD. *J Am Acad Child Adolesc Psychiatry.* 2001;40(11):1268-1276.

Reason for exclusion: Co-treatment (behavior management techniques used during the programme)

Jans2015

- Jans T, Jacob C, Warnke A, et al. Does intensive multimodal treatment for maternal ADHD improve the efficacy of parent training for children with ADHD? A randomized controlled multicenter trial. *J Child Psychol Psychiatry.* 2015;56(12):1298-1313.
- Jans T, Jacob C, Hennighausen K, et al. Treatment outcome of behavioral parent-child training in childhood ADHD as a function of the treatment of maternal ADHD. *Neuropsychiatr Enfance Adolesc.* 2012;1:S51.

Reason for exclusion: No arms of interest for the present meta-analysis

Janssen2016 (NCT01363544; EUCTR2010-020508-31-NL)

- Janssen TW, Bink M, Gelade K, van Mourik R, Maras A, Oosterlaan J. A randomized controlled trial into the effects of neurofeedback, methylphenidate, and physical activity on EEG power spectra in children with ADHD. *J Child Psychol Psychiatry.* 2016;57(5):633-644.
- Janssen TW, Bink M, Gelade K, van Mourik R, Maras A, Oosterlaan J. A Randomized Controlled Trial Investigating the Effects of Neurofeedback, Methylphenidate, and Physical Activity on Event-Related Potentials in Children with Attention-Deficit/Hyperactivity Disorder. *J Child Adolesc Psychopharmacol.* 2016;26(4):344-353.
- Gelade K, Janssen TWP, Bink M, Van Mourik R, Maras A, Oosterlaan J. Behavioral effects of neurofeedback compared to stimulants and physical activity in attention-deficit/hyperactivity disorder: A randomized controlled trial. *J Clin Psychiatry.* 2016;77:e1270-e1277.
- https://clinicaltrials.gov/ct2/show/NCT01363544
- https://www.clinicaltrialsregister.eu/ctr-search/search?query=eudract_number:2010-020508-31

Reason for exclusion: No arms of interest for the present meta-analysis (neurofeednback, physical activity, MPH; no placebo arm)

Jaselskis1992

- Jaselskis CA, Cook EH, Jr., Fletcher KE, Leventhal BL. Clonidine treatment of hyperactive and impulsive children with autistic disorder. *J Clin Psychopharmacol.* 1992;12(5):322-327.

Reason for exclusion: No diagnostic criteria for ADHD; previously non responders or side effects with MPH

Jensen1999

- Hechtman L. Aims and methodological problems in multimodal treatment studies. *Can J Psychiatry.* 1993;38(6):458-464.
- Richters JE, Arnold LE, Jensen PS, et al. NIMH collaborative multisite multimodal treatment study of children with ADHD: I. Background and rationale. *J Am Acad Child Adolesc Psychiatry.* 1995;34(8):987-1000.
- Greenhill LL, Abikoff HB, Arnold LE, et al. Medication treatment strategies in the MTA Study: relevance to clinicians and researchers. *J Am Acad Child Adolesc Psychiatry.* 1996;35(10):1304-1313.
- Arnold LE, Abikoff HB, Cantwell DP, Conners CK, ElliottGR, Greenhill LL, et al. NIMH Collaborative Multimodal Treatment Study of Children with ADHD (MTA): design, methodology, and protocol evolution. *J Atten Disord.* 1997;2(3):141–58.
- Hinshaw SP, March JS, Abikoff H, Arnold LE, CantwellDP, Conners CK, et al. Comprehensive assessment ofchildhood attention-deficit hyperactivity disorder in thecontext of a multisite, multimodal clinical trial. *J Atten Disord.* 1997;1(4):217–34.
- Arnold LE, Abikoff HB, Cantwell DP, et al. National Institute of Mental Health Collaborative Multimodal Treatment Study of Children with ADHD (the MTA). Design challenges and choices. *Arch Gen Psychiatry.* 1997;54(9):865-870.
- Anonymous. A 14-month randomized clinical trial of treatment strategies for attention-deficit/hyperactivity disorder. The MTA Cooperative Group. Multimodal Treatment Study of Children with ADHD. *Arch Gen Psychiatry.* 1999;56(12):1073-1086.
- Jensen PS, Arnold LE, Richters JE, et al. Moderators and mediators of treatment response for children with attention-deficit/hyperactivity disorder - The multimodal treatment study of children with attention-deficit/hyperactivity disorder. *Arch Gen Psychiatry.* 1999;56(12):1088-1096.
- Swanson J, Lerner M, March J, Gresham FM. Assessment and intervention for attention-deficit/hyperactivity disorder in the schools. Lessons from the MTA study. *Pediatr Clin North Am.* 1999;46(5):993-1009.
- Jensen PS, Arnold LE, Richters JE, Severe JB, Vereen D, Vitiello B, et al. A 14-month randomized clinical trial of treatment strategies for attention-deficit/hyperactivity disorder. *Arch Gen Psychiatry.* 1999;56(12): 1073–86.
- Jensen PS. Fact versus fancy concerning the multimodal treatment study for attention-deficit hyperactivity disorder. *Can J Psychiatry.* 1999;44(10):975-980.
- Hinshaw SP. Moderators and mediators of treatment response for children with attention-deficit/hyperactivity disorder: the multimodal treatment study of children with attention-deficit/hyperactivity disorder. *Arch Gen Psychiatry.* 1999;56(12):1088–96.
- Cunningham CE. In the wake of the MTA: charting a new course for the study and treatment of children with attention-deficit hyperactivity disorder. *Can J Psychiatry.* 1999;44(10):999-1006.
- Boyle MH, Jadad AR. Lessons from large trials: the MTA study as a model for evaluating the treatment of childhood psychiatric disorder. *Can J Psychiatry.* 1999;44(10):991-998.
- Pelham WE, Jr. The NIMH multimodal treatment study for attention-deficit hyperactivity disorder: just say yes to drugs alone? *Can J Psychiatry.* 1999;44(10):981-990.
- Stubbe DE. Attention-deficit/hyperactivity disorder overview. Historical perspective, current controversies, and future directions. *Child Adolesc Psychiatr Clin N Am.* 2000;9(3):469-479, v.

- Pelham WE, Gnagy EM, Greiner AR, et al. Behavioral versus behavioral and pharmacological treatment in ADHD children attending a summer treatment program. *J Abnorm Child Psychol.* 2000;28(6):507-525.
- Barkley RA. Commentary on the multimodal treatment study of children with ADHD. *J Abnorm Child Psychol.* 2000;28(6):595-599.
- Epstein JN, Conners CK, Erhardt D, et al. Familial aggregation of ADHD characteristics. *J Abnorm Child Psychol.* 2000;28(6):585-594.
- Paule MG, Rowland AS, Ferguson SA, et al. Attention deficit/hyperactivity disorder: characteristics, interventions and models. *Neurotoxicol Teratol.* 2000;22(5):631-651.
- March JS, Swanson JM, Arnold LE, et al. Anxiety as a predictor and outcome variable in the multimodal treatment study of children with ADHD (MTA). *J Abnorm Child Psychol.* 2000;28(6):527-541.
- Carey WB. What the multimodal treatment study of children with attention deficit/hyperactivity disorder did and did not say about the use of methylphenidate for attention deficits. *Pediatrics.* 2000;105(4 Pt 1):863-864.
- Pedlow K. Incorporating the management of ADHD into your practice. Can it be done? *Aust Fam Physician.* 2000;29(12):1210-1214.
- Hoza B, Owens JS, Pelham WE, et al. Parent cognitions as predictors of child treatment response in attention-deficit/hyperactivity disorder. *J Abnorm Child Psychol.* 2000;28(6):569-583.
- Hinshaw SP, Owens EB, Wells KC, et al. Family processes and treatment outcome in the MTA: negative/ineffective parenting practices in relation to multimodal treatment. *J Abnorm Child Psychol.* 2000;28(6):555-568.
- Wells KC, Epstein JN, Hinshaw SP, et al. Parenting and family stress treatment outcomes in attention deficit hyperactivity disorder (ADHD): an empirical analysis in the MTA study. *J Abnorm Child Psychol.* 2000;28(6):543-553.
- Wells KC, Pelham WE, Kotkin RA, et al. Psychosocial treatment strategies in the MTA study: rationale, methods, and critical issues in design and implementation. *J Abnorm Child Psychol.* 2000;28(6):483-505.
- Newcorn JH. The multimodal treatment study of children with attention deficit hyperactivity disorder. *Curr Psychiatry Rep.* 2000;2(2):85-89.
- Newcorn JH, Halperin JM, Jensen PS, et al. Symptom profiles in children with ADHD: effects of comorbidity and gender. *J Am Acad Child Adolesc Psychiatry.* 2001;40(2):137-146.
- Horrigan JP. Present and future pharmacotherapeutic options for adult attention deficit/hyperactivity disorder. *Expert Opin Pharmacother.* 2001;2(4):573-586.
- Jensen PS. Introduction--ADHD comorbidity and treatment outcomes in the MTA. *J Am Acad Child Adolesc Psychiatry.* 2001;40(2):134-136.
- Greene RW, Ablon JS. What does the MTA study tell us about effective psychosocial treatment for ADHD? *J Clin Child Psychol.* 2001;30(1):114-121.
- Jensen PS, Hinshaw SP, Kraemer HC, et al. ADHD comorbidity findings from the MTA study: comparing comorbid subgroups. *J Am Acad Child Adolesc Psychiatry.* 2001;40(2):147-158.
- Jensen PS, Hinshaw SP, Swanson JM, et al. Findings from the NIMH Multimodal Treatment Study of ADHD (MTA): implications and applications for primary care providers. *J Dev Behav Pediatr: JDBP.* 2001;22(1):60-73.
- Conners CK, Epstein JN, March JS, et al. Multimodal treatment of ADHD in the MTA: an alternative outcome analysis. *J Am Acad Child Adolesc Psychiatry.* 2001;40(2):159-167.
- Greenhill LL, Swanson JM, Vitiello B, et al. Impairment and deportment responses to different methylphenidate doses in children with ADHD: the MTA titration trial. *J Am Acad Child Adolesc Psychiatry.* 2001;40(2):180-187.
- Harwood TM, Beutler LE. Commentary on Greene and Ablon: What does the MTA study tell us about effective psychosocial treatment for ADHD? *J Clin Child Psychol.* 2001;30(1):141-143.
- Levy F. Implications for Australia of the multimodal treatment study of children with attention-deficit/hyperactivity disorder. *Aust N Z J Psychiatry.* 2001;35(1):45-48.
- Shaywitz BA, Fletcher JM, Shaywitz SE. Attention Deficit Hyperactivity Disorder. *Curr Treat Options Neurol.* 2001;3(3):229-236.
- Wells KC. Comprehensive versus matched psychosocial treatment in the MTA study: conceptual and empirical issues. *J Clin Child Psychol.* 2001;30(1):131-135.
- Anonymous. Clinical practice guideline: treatment of the school-aged child with attention-deficit/hyperactivity disorder. *Pediatrics.* 2001;108(4):1033-1044.
- Abikoff H. Tailored psychosocial treatments for ADHD: the search for a good fit. *J Clin Child Psychol.* 2001;30(1):122-125.
- Swanson JM, Kraemer HC, Hinshaw SP, et al. Clinical relevance of the primary findings of the MTA: success rates based on severity of ADHD and ODD symptoms at the end of treatment. *J Am Acad Child Adolesc Psychiatry.* 2001;40(2):168-179.
- Vitiello B, Severe JB, Greenhill LL, et al. Methylphenidate dosage for children with ADHD over time under controlled conditions: lessons from the MTA. *J Am Acad Child Adolesc Psychiatry.* 2001;40(2):188-196.

- Hoza B. Psychosocial treatment issues in the MTA: a reply to Greene and Ablon. *J Clin Child Psychol.* 2001;30(1):126-130.
- Solanto MV, Abikoff H, Sonuga-Barke E, et al. The ecological validity of delay aversion and response inhibition as measures of impulsivity in AD/HD: a supplement to the NIMH multimodal treatment study of AD/HD. *J Abnorm Child Psychol.* 2001;29(3):215-228.
- Abikoff H, Arnold LE, Newcorn JH, et al. Emergency/Adjunct services and attrition prevention for randomized clinical trials in children: the MTA manual-based solution. *J Am Acad Child Adolesc Psychiatry.* 2002;41(5):498-504.
- Rieppi R, Greenhill LL, Ford RE, et al. Socioeconomic status as a moderator of ADHD treatment outcomes. *J Am Acad Child Adolesc Psychiatry.* 2002;41(3):269-277.
- Jensen P. Longer term effects of stimulant treatments for Attention-Deficit/Hyperactivity Disorder. *J Atten Disord.* 2002;6 Suppl 1:S45-56.
- Johnston C, Murray C, Hinshaw SP, William EP, Jr., Hoza B. Responsiveness in interactions of mothers and sons with ADHD: relations to maternal and child characteristics. *J Abnorm Child Psychol.* 2002;30(1):77-88.
- Hinshaw SP. Intervention research, theoretical mechanisms, and causal processes related to externalizing behavior patterns. *Dev Psychopathol.* 2002;14(4):789-818.
- Dopfner M, Lehmkuhl G. [Evidence based therapy of children and adolescents with attention deficit/hyperactivity hyperkinetic syndrome (ADHS)]. *Prax Kinderpsychol Kinderpsychiatr.* 2002;51(6):419-440.
- Swanson JM, Arnold LE, Vitiello B, et al. Response to commentary on the multimodal treatment study of ADHD (MTA): mining the meaning of the MTA. *J Abnorm Child Psychol.* 2002;30(4):327-332.
- Owens EB, Hinshaw SP, Kraemer HC, et al. Which treatment for whom for ADHD? Moderators of treatment response in the MTA. *J Consult Clin Psychol.* 2003;71(3):540-552.
- Galanter CA, Carlson GA, Jensen PS, et al. Response to methylphenidate in children with attention deficit hyperactivity disorder and manic symptoms in the multimodal treatment study of children with attention deficit hyperactivity disorder titration trial. *J Child Adolesc Psychopharmacol.* 2003;13(2):123-136.
- Arnold LE, Elliot M, Sachs L, et al. Effects of ethnicity on treatment attendance, stimulant response/dose, and 14-month outcome in ADHD. *J Consult Clin Psychol.* 2003;71(4):713-727.
- Waxmonsky J. Assessment and treatment of attention deficit hyperactivity disorder in children with comorbid psychiatric illness. *Curr Opin Pediatr.* 2003;15(5):476-482.
- MTA Cooperative Group. National Institute of Mental Health Multimodal Treatment Study of ADHD follow-up:24-month outcomes of treatment strategies for attention deficit/hyperactivity disorder. *Pediatrics.* 2004;113(4):754–61.
- Anonymous. National Institute of Mental Health Multimodal Treatment Study of ADHD follow-up: changes in effectiveness and growth after the end of treatment. *Pediatrics.* 2004;113(4):762-769.
- Hoza B, Gerdes AC, Hinshaw SP, et al. Self-perceptions of competence in children with ADHD and comparison children. *J Consult Clin Psychol.* 2004;72(3):382-391.
- Jensen PS, Arnold LE, Severe JB, Vitiello B, Hoagwood K.National Institute of Mental Health multimodal treatment study of ADHD follow-up: changes in effectiveness and growth after the end of treatment. *Pediatrics.* 2004;113(4):762–9.
- Eaton Hoagwood K, Jensen PS, Arnold LE, et al. Reliability of the services for children and adolescents-parent interview. *J Am Acad Child Adolesc Psychiatry.* 2004;43(11):1345-1354.
- Leo J. Multiple comparisons in drug efficacy studies: scientific or marketing principles? *Ethical Hum Psychol Psychiatry.* 2004;6(1):3-6.
- Anonymous. National Institute of Mental Health Multimodal Treatment Study of ADHD follow-up: 24-month outcomes of treatment strategies for attention-deficit/hyperactivity disorder. *Pediatrics.* 2004;113(4):754-761.
- Arnold LE, Chuang S, Davies M, et al. Nine months of multicomponent behavioral treatment for ADHD and effectiveness of MTA fading procedures. *J Abnorm Child Psychol.* 2004;32(1):39-51.
- Jensen PS, Eaton Hoagwood K, Roper M, et al. The services for children and adolescents-parent interview: development and performance characteristics. *J Am Acad Child Adolesc Psychiatry.* 2004;43(11):1334-1344.
- Jensen PS, Garcia JA, Glied S, et al. Cost-effectiveness of ADHD treatments: findings from the multimodal treatment study of children with ADHD. *Am J Psychiatry.* 2005;162(9):1628-1636.
- Capp PK, Pearl PL, Conlon C. Methylphenidate HCl: therapy for attention deficit hyperactivity disorder. *Expert Rev Neurother.* 2005;5(3):325-331.
- Hechtman L, Etcovitch J, Platt R, et al. Does multimodal treatment of ADHD decrease other diagnoses? *Clin Neurosci Res.* Dec 2005;5(5-6):273-282.
- Hoza B, Gerdes AC, Mrug S, et al. Peer-assessed outcomes in the multimodal treatment study of children with attention deficit hyperactivity disorder. *J Clin Child Adolesc Psychol.* 2005;34(1):74-86.
- Hansen L, Thomsen PH. [How to treat ADHD/DAMP? Is there a conclusive answer? A critical survey of the MTA trial?]. *Ugeskr Laeger.* 2005;167(48):4555-4559.

- Horst RO, Hendren RL. Integrated pharmacologic treatment of attention-deficit hyperactivity disorder (ADHD). *Essent Psychopharmacol.* 2005;6(5):250-261.
- *Can Child Adolesc Psychiatr Rev*. 2005;14(Supplement 1):2-3.
- Ercan ES, Varan A, Deniz U. Effects of combined treatment on Turkish children diagnosed with attention-deficit/hyperactivity disorder: a preliminary report. *J Child Adolesc Psychopharmacol.* 2005;15(2):203-219.
- Wells KC, Chi TC, Hinshaw SP, et al. Treatment-related changes in objectively measured parenting behaviors in the multimodal treatment study of children with attention-deficit/hyperactivity disorder. *J Consult Clin Psychol.* 2006;74(4):649-657.
- Epstein JN, Conners CK, Hervey AS, et al. Assessing medication effects in the MTA study using neuropsychological outcomes. *J Child Psychol Psychiatry.* 2006;47(5):446-456.
- Hervey AS, Epstein JN, Curry JF, et al. Reaction time distribution analysis of neuropsychological performance in an ADHD sample. *Child neuropsychol.* 2006;12(2):125-140.
- Gerdes AC, Hoza B, Arnold LE, et al. Child and parent predictors of perceptions of parent--child relationship quality. *J Atten Disord.* 2007;11(1):37-48.
- Swanson JM, Hinshaw SP, Arnold LE, et al. Secondary evaluations of MTA 36-month outcomes: propensity score and growth mixture model analyses. *J Am Acad Child Adolesc Psychiatry.* 2007;46(8):1003-1014.
- Swanson JM, Elliott GR, Greenhill LL, et al. Effects of stimulant medication on growth rates across 3 years in the MTA follow-up. *J Am Acad Child Adolesc Psychiatry.* 2007;46(8):1015-1027.
- Jensen PS, Arnold LE, Swanson JM, et al. 3-year follow-up of the NIMH MTA study. *J Am Acad Child Adolesc Psychiatry.* 2007;46(8):989-1002.
- Hinshaw SP. Moderators and mediators of treatment outcome for youth with ADHD: understanding for whom and how interventions work. *J Pediatr Psychol.* 2007;32(6):664-675.
- Manassis K. When attention-deficit/hyperactivity disorder co-occurs with anxiety disorders: effects on treatment. *Expert Rev Neurother.* 2007;7(8):981-988.
- Gerdes AC, Hoza B, Arnold LE, et al. Maternal depressive symptomatology and parenting behavior: exploration of possible mediators. *J Abnorm Child Psychol.* 2007;35(5):705-714.
- Molina BS, Flory K, Hinshaw SP, et al. Delinquent behavior and emerging substance use in the MTA at 36 months: prevalence, course, and treatment effects. *J Am Acad Child Adolesc Psychiatry.* 2007;46(8):1028-1040.
- Stevens J, Kelleher K, Greenhouse J, et al. Empirical evaluation of the generalizability of the sample from the multimodal treatment study for ADHD. *Adm Policy Ment Health.* 2007;34(3):221-232.
- Doig J, McLennan JD, Gibbard WB. Medication effects on symptoms of attention-deficit/hyperactivity disorder in children with fetal alcohol spectrum disorder. *J Child Adolesc Psychopharmacol.* 2008;18(4):365-371.
- Murray DW, Arnold LE, Swanson J, et al. A clinical review of outcomes of the multimodal treatment study of children with attention-deficit/hyperactivity disorder (MTA). *Curr Psychiatry Rep.* 2008;10(5):424-431.
- Swanson J, Arnold LE, Kraemer H, et al. Evidence, interpretation, and qualification from multiple reports of long-term outcomes in the Multimodal Treatment study of Children With ADHD (MTA): part I: executive summary. *J Atten Disord.* 2008;12(1):4-14.
- Swanson J, Arnold LE, Kraemer H, et al. Evidence, interpretation, and qualification from multiple reports of long-term outcomes in the Multimodal Treatment Study of children with ADHD (MTA): Part II: supporting details. *J Atten Disord.* 2008;12(1):15-43.
- Langberg JM, Epstein JN, Altaye M, Molina BS, Arnold LE, Vitiello B. The transition to middle school is associated with changes in the developmental trajectory of ADHD symptomatology in young adolescents with ADHD. *J Clin Child Adolesc Psychol.* 2008;37(3):651-663.
- Karpenko V, Owens JS, Evangelista NM, Dodds C. Clinically significant symptom change in children with attention-deficit/hyperactivity disorder: does it correspond with reliable improvement in functioning? *J Clin Psychol.* 2009;65(1):76-93.
- Molina BS, Hinshaw SP, Swanson JM, et al. The MTA at 8 years: prospective follow-up of children treated for combined-type ADHD in a multisite study. *J Am Acad Child Adolesc Psychiatry.* 2009;48(5):484-500.
- Mrug S, Hoza B, Gerdes AC, et al. Discriminating between children with ADHD and classmates using peer variables. *J Atten Disord.* 2009;12(4):372-380.
- Reeves G, Anthony B. Multimodal treatments versus pharmacotherapy alone in children with psychiatric disorders: implications of access, effectiveness, and contextual treatment. *Paediatr Drugs.* 2009;11(3):165-169.
- Pappadopulos E, Jensen PS, Chait AR, et al. Medication adherence in the MTA: saliva methylphenidate samples versus parent report and mediating effect of concomitant behavioral treatment. *J Am Acad Child Adolesc Psychiatry.* 2009;48(5):501-510.
- Nieweg EH. [Does ADHD medication stop working after 2-3 years? On the surprising, but little-known follow up of the MTA study]. *Tijdschr Psychiatr.* 2010;52(4):245-254.
- Buitelaar JK. [Follow-up of the MTA-study in children with ADHD: what do the data tell us, and what not?]. *Tijdschr Psychiatr.* 2010;52(4):255-257.

- Murray-Close D, Hoza B, Hinshaw SP, et al. Developmental processes in peer problems of children with attention-deficit/hyperactivity disorder in the Multimodal Treatment Study of Children With ADHD: developmental cascades and vicious cycles. *Dev Psychopathol.* 2010;22(4):785-802.
- Jones HA, Epstein JN, Hinshaw SP, et al. Ethnicity as a moderator of treatment effects on parent--child interaction for children with ADHD. *J Atten Disord.* 2010;13(6):592-600.
- Langberg JM, Arnold LE, Flowers AM, Altaye M, Epstein JN, Molina BS. Assessing Homework Problems in Children with ADHD: Validation of a Parent-Report Measure and Evaluation of Homework Performance Patterns. *School Ment Health.* 2010;2(1):3-12.
- Langberg JM, Arnold LE, Flowers AM, et al. Parent-reported homework problems in the MTA study: evidence for sustained improvement with behavioral treatment. *J Clin Child Adolesc Psychol* 2010;39(2):220-233.
- Rabiner DL, Murray DW, Rosen L, Hardy K, Skinner A, Underwood M. Instability in teacher ratings of children's inattentive symptoms: implications for the assessment of ADHD. *J Dev Behav Pediatr : JDBP.* 2010;31(3):175-180.
- Schlander M. The pharmaceutical economics of child psychiatric drug treatment. *Curr Pharm Des.* 2010;16(22):2443-2461.
- Langberg JM, Epstein JN, Simon JO, et al. Parental Agreement on ADHD Symptom-Specific and Broadband Externalizing Ratings of Child Behavior. *J Emot Behav Disord.* 2010;18(1):41-50.
- Mikami AY, Hinshaw SP, Arnold LE, et al. Bulimia nervosa symptoms in the multimodal treatment study of children with ADHD. *Int J Eat Disord.* 2010;43(3):248-259.
- Kleinman RE, Brown RT, Cutter GR, Dupaul GJ, Clydesdale FM. A research model for investigating the effects of artificial food colorings on children with ADHD. *Pediatrics.* 2011;127(6):e1575-1584.
- Loutfi KS, Carvalho AM, Lamounier JA, Nascimento J de A. ADHD and epilepsy: contributions from the use of behavioral rating scales to investigate psychiatric comorbidities. *Epilepsy Behav.* 2011;20(3):484-489.
- Vaughn AJ, Epstein JN, Rausch J, et al. Relation between outcomes on a continuous performance test and ADHD symptoms over time. *J Abnorm Child Psychol.* 2011;39(6):853-864.
- Vitiello B, Elliott GR, Swanson JM, et al. Blood pressure and heart rate over 10 years in the multimodal treatment study of children with ADHD. *Am J Psychiatry.* 2012;169(2):167-177.
- Power TJ, Mautone JA, Soffer SL, et al. A family-school intervention for children with ADHD: results of a randomized clinical trial. *J Consult Clin Psychol.* 2012;80(4):611-623.
- Arruda MA, Bigal ME. Migraine and migraine subtypes in preadolescent children: association with school performance. *Neurology.* 2012;79(18):1881-1888.
- Mrug S, Molina BS, Hoza B, et al. Peer rejection and friendships in children with Attention-Deficit/Hyperactivity Disorder: contributions to long-term outcomes. *J Abnorm Child Psychol.* 2012;40(6):1013-1026
- Schurmann S, Breuer D, Wolff Metternich-Kaizman T, Dopfner M. [Intellectual abilities in adolescents with former ADHD diagnoses - relationships to current ADHD symptoms, comorbid symptoms, and medication history: results of an 8.5-year follow-up of the Cologne Adaptive Multimodal Treatment Study (CAMT)]. *Z Kinder Jugendpsychiatr Psychother.* 2012;40(1):51-60.
- von Polier GG, Vloet TD, Herpertz-Dahlmann B. ADHD and delinquency--a developmental perspective. *Behav Sci Law.* 2012;30(2):121-139.
- Lu B, Marcus S. Evaluating long-term effects of a psychiatric treatment using instrumental variable and matching approaches. *Health Serv Outcomes Res Methodol.* 2012;12(4):288-301.
- Hoza B, McQuade JD, Murray-Close D, et al. Does childhood positive self-perceptual bias mediate adolescent risky behavior in youth from the MTA study? *J Consult Clin Psychol.* 2013;81(5):846-858.
- Molina BS, Hinshaw SP, Eugene Arnold L, et al. Adolescent substance use in the multimodal treatment study of attention-deficit/hyperactivity disorder (ADHD) (MTA) as a function of childhood ADHD, random assignment to childhood treatments, and subsequent medication. *J Am Acad Child Adolesc Psychiatry.* 2013;52(3):250-263.
- Rockhill C, Violette H, Vander Stoep A, Grover S, Myers K. Caregivers' distress: youth with attention-deficit/hyperactivity disorder and comorbid disorders assessed via telemental health. *J Child Adolesc Psychopharmacol.* 2013;23(6):379-385.
- Owens EB, Hinshaw SP. Perinatal problems and psychiatric comorbidity among children with ADHD. *J Clin Child Adolesc Psychol: the official journal for the Society of Clinical Child and Adolescent Psychology, American Psychological Association, Division 53.* 2013;42(6):762-768.
- Bange F, Le Heuzey MF, Acquaviva E, Delorme R, Mouren MC. [Cardiovascular risks and management during Attention Deficit Hyperactivity Disorder treatment with methylphenidate]. *Arch Pediatr.* 2014;21(1):108-112.
- Sotomayor-Zarate R, Jara P, Araos P, et al. Improving amphetamine therapeutic selectivity: N,N-dimethyl-MTA has dopaminergic effects and does not produce aortic contraction. *Toxicology.* 2014;114(5):395-399.
- Perez Algorta G, Kragh CA, Arnold LE, et al. Maternal ADHD Symptoms, Personality, and Parenting Stress: Differences Between Mothers of Children With ADHD and Mothers of Comparison Children. *J Atten Disord.* 2014.
- Greenfield B, Hechtman L, Stehli A, Wigal T. Sexual maturation among youth with ADHD and the impact of stimulant medication. *Adolesc Psychiatry.* 2014;23(9):835-839.

- Duran MH, Guimaraes CA, Montenegro MA, Neri ML, Guerreiro MM. ADHD in idiopathic epilepsy. *Arq Neuropsiquiatr.* 2014;72(1):12-16.
- Wagner DJ, Vallerand IA, McLennan JD. Treatment receipt and outcomes from a clinic employing the attention-deficit/hyperactivity disorder treatment guideline of the children's medication algorithm project. *J Child Adolesc Psychopharmacol.* 2014;24(9):472-480.
- Murray DW, Molina BS, Glew K, et al. Prevalence and Characteristics of School Services for High School Students with Attention-Deficit/Hyperactivity Disorder. *School Ment Health.* 2014;6(4):264-278.
- Elmaadawi AZ, Jensen PS, Arnold LE, et al. Risk for emerging bipolar disorder, variants, and symptoms in children with attention deficit hyperactivity disorder, now grown up. *World J Psychiatry.* 2015;5(4):412-424.
- Fernandez de la Cruz L, Simonoff E, McGough JJ, Halperin JM, Arnold LE, Stringaris A. Treatment of children with attention-deficit/hyperactivity disorder (ADHD) and irritability: results from the multimodal treatment study of children with ADHD (MTA). *J Am Acad Child Adolesc Psychiatry.* 2015;54(1):62-70.e63.
- Minahim D, Rohde LA. Attention deficit hyperactivity disorder and intellectual giftedness: a study of symptom frequency and minor physical anomalies. *Rev Bras Psquiatr (Sao Paulo, Brazil : 1999).* 2015;37(4):289-295.
- Hinshaw SP, Arnold LE. ADHD, Multimodal Treatment, and Longitudinal Outcome: Evidence, Paradox, and Challenge. *Wiley Interdiscip Rev Cogn Sci.* 2015;6(1):39-52.
- Hinshaw SP, Arnold LE. Attention-deficit hyperactivity disorder, multimodal treatment, and longitudinal outcome: evidence, paradox, and challenge. *Wiley Interdiscip Rev Cogn Sci.* 2015;6(1):39-52.
- Howard AL, Molina BS, Swanson JM, et al. Developmental progression to early adult binge drinking and marijuana use from worsening versus stable trajectories of adolescent attention deficit/hyperactivity disorder and delinquency. *Addiction.* 2015;110(5):784-795.
- Wagner DJ, McLennan JD. An Alternative Approach to Scoring the MTA-SNAP-IV to Guide Attention-Deficit/Hyperactivity Disorder Medication Treatment Titration towards Symptom Remission: A Preliminary Consideration. *J Child Adolesc Psychopharmacol.* 2015;25(10):749-753.
- Acosta MT, Swanson J, Stehli A, et al. ADGRL3 (LPHN3) variants are associated with a refined phenotype of ADHD in the MTA study. *NPJ Genom Med.* 2016;4(5):540-547.
- Howard AL, Strickland NJ, Murray DW, et al. Progression of impairment in adolescents with attention-deficit/hyperactivity disorder through the transition out of high school: Contributions of parent involvement and college attendance. *J Abnorm Psychol.* 2016;125(2):233-247.
- Briars L, Todd T. A Review of Pharmacological Management of Attention-Deficit/Hyperactivity Disorder. *J Pediatr Pharmacol Ther.* 2016;21(3):192-206.
- Roy A, Hechtman L, Arnold LE, et al. Childhood Factors Affecting Persistence and Desistence of Attention-Deficit/Hyperactivity Disorder Symptoms in Adulthood: Results From the MTA. *J Am Acad Child Adolesc Psychiatry.* 2016;55(11):937-944.e934.
- Hall CL, Taylor JA, Newell K, Baldwin L, Sayal K, Hollis C. The challenges of implementing ADHD clinical guidelines and research best evidence in routine clinical care settings: Delphi survey and mixed-methods study. *BJPsych open.* 2016;2(1):25-31.
- Hechtman L, Swanson JM, Sibley MH, et al. Functional Adult Outcomes 16 Years After Childhood Diagnosis of Attention-Deficit/Hyperactivity Disorder: MTA Results. *J Am Acad Child Adolesc Psychiatry.* 2016;55(11):945-952.e942.
- McGough JJ. New Insights From the MTA: Who Outgrows Attention-Deficit/Hyperactivity Disorder and What Becomes of Those Who Don't? *J Am Acad Child Adolesc Psychiatry.* 2016;55(11):925-926.
- Bloch MH. Editorial: The continuing contributions of multimodal treatment of attention over nearly two decades to initial attention-deficit hyperactivity disorder pharmacotherapy and long-term clinical course. *J Child Psychol Psychiatry.* 2017;58(6):637-639.
- Bonnefois G, Robaey P, Barriere O, Li J, Nekka F. An Evaluation Approach for the Performance of Dosing Regimens in Attention-Deficit/Hyperactivity Disorder Treatment. *J Child Adolesc Psychopharmacol.* 2017;27(4):320-331.
- Caye A, Sibley MH, Swanson JM, Rohde LA. Late-Onset ADHD: Understanding the Evidence and Building Theoretical Frameworks. *Curr Psychiatry Rep.* 2017;19(12):106.
- DuPaul GJ, Morgan PL, Farkas G, Hillemeier MM, Maczuga S. Eight-Year Latent Class Trajectories of Academic and Social Functioning in Children with Attention-Deficit/Hyperactivity Disorder. *J Abnorm Child Psychol.* 2017.
- Johnson JA, Jakubovski E, Reed MO, Bloch MH. Predictors of Long-Term Risky Driving Behavior in the Multimodal Treatment Study of Children with Attention-Deficit/Hyperactivity Disorder. *J Child Adolesc Psychopharmacol.* 2017;27(8):747-754.
- Kelly C, Castellanos FX, Tomaselli O, et al. Distinct effects of childhood ADHD and cannabis use on brain functional architecture in young adults. *Neuroimage Clin.* 2017;13:188-200.
- Meinzer MC, LeMoine KA, Howard AL, et al. Childhood ADHD and Involvement in Early Pregnancy: Mechanisms of Risk. *J Atten Disord* 2017:1087054717730610.

- Mitchell JT, Weisner TS, Jensen PS, et al. How Substance Users With ADHD Perceive the Relationship Between Substance Use and Emotional Functioning. *J Atten Disord.* 2017:1087054716685842.
- Nichols JQ, Shoulberg EK, Garner AA, et al. Exploration of the Factor Structure of ADHD in Adolescence through Self, Parent, and Teacher Reports of Symptomatology. *J Abnorm Child Psychol.* 2017;45(3):625-641.
- Reed MO, Jakubovski E, Johnson JA, Bloch MH. Predictors of Long-Term School-Based Behavioral Outcomes in the Multimodal Treatment Study of Children with Attention-Deficit/Hyperactivity Disorder. *J Child Adolesc Psychopharmacol.* 2017;27(4):296-309.
- Romanzini LP, Dos Santos AA, Nunes ML. Characteristics of sleep in socially vulnerable adolescents. *Eur J Peadiatr Neurol.* 2017;21(4):627-634.
- Roy A, Hechtman L, Arnold LE, et al. Childhood Predictors of Adult Functional Outcomes in the Multimodal Treatment Study of Attention-Deficit/Hyperactivity Disorder (MTA). *J Am Acad Child Adolesc Psychiatry.* 2017;56(8):687-695.e687.
- Sibley MH, Rohde LA, Swanson JM, et al. Late-Onset ADHD Reconsidered With Comprehensive Repeated Assessments Between Ages 10 and 25. *Am J Psychiatry.* 2017:appiajp201717030298.
- Singh A, Padhy SK. In Reply: Psychotic symptoms in Attention Deficit Hyperactivity Disorder: An analysis of MTA Database. *Asian J Psychiatr.* 2017;30:71-72.
- Swanson JM, Arnold LE, Molina BSG, et al. Young adult outcomes in the follow-up of the multimodal treatment study of attention-deficit/hyperactivity disorder: symptom persistence, source discrepancy, and height suppression. *J Child Psychol Psychiatry.* 2017;58(6):663-678.
- Swanson JM, Wigal T, Jensen PS, et al. The Qualitative Interview Study of Persistent and Nonpersistent Substance Use in the MTA: Sample Characteristics, Frequent Use, and Reasons for Use. *J Atten Disord.* 2017:1087054717714058.
- Vitiello B, Perez Algorta G, Arnold LE, Howard AL, Stehli A, Molina BS. Psychotic Symptoms in Attention-Deficit/Hyperactivity Disorder: An Analysis of the MTA Database. *J Am Acad Child Adolesc Psychiatry.* 2017;56(4):336-343.
- Weisner TS, Murray DW, Jensen PS, et al. Follow-Up of Young Adults With ADHD in the MTA: Design and Methods for Qualitative Interviews. *J Atten Disord.* 2017:1087054717713639.
- Whalen CK. ADHD treatment in the 21st century: pushing the envelope. *J Clin Child Psychol.*2001;30():136-40.

Reason for exclusion: The initial 28-day titration phase is not petinent for the present meta-analysis since this was a cross-over study with daily switching (with less than 24h wash-out period) (this has been published in Greenhill LL, Swanson JM, Vitiello B, et al. Impairment and deportment responses to different methylphenidate doses in children with ADHD: the MTA titration trial. J Am Acad Child Adolesc Psychiatry. 2001;40(2):180-187). The subsequent phase included:
1. Medication management
2. Behavioural treatment
3. Combined treatment (medication management + behavioural treatment)
4. Community care (control group)

Jerome1995

- Jerome L. Neurophysiological effects of stimulants. *J Am Acad Child Adolesc Psychiatry.* 1995;34(2):126-127.
- Jerome L. Central auditory processing disorder and ADHD. *J Am Acad Child Adolesc Psychiatry.* 2000;39(4):399-400.

Reason for exclusion: Single dose

Jerome2001

- Jerome L. Can methylphenidate facilitate sleep in children with attention deficit hyperactivity disorder? *J Child Adolesc Psychopharmacol.* 2001;11(1):109.

Reason for exclusion: Letter to the editor, no empirical data

Jerome2001

- Jerome L, Segal A. Benefit of long-term stimulants on driving in adults with ADHD. *J Ner Ment* Dise. 2001;189(1):63-64.

Reason for exclusion: No RCT (commentary)

Jerome2014

- Jerome L. Effects of methylphenidate on acute Math performance in children with ADHD. *Can J Psychiatry.* 2014;59(5):290.

Reason for exclusion: Commentary

Jesse2016

- Jesse A, Alipio-Jocson V, Inoyama K, et al. Methylphenidate's effects on cognition in patients with epilepsy: A randomized, double-blind, placebo-controlled, crossover, single-dose study. *Neurology. Conference: 68th American Academy of Neurology Annual Meeting, AAN.* 2016;86(16 suppl. 1).

Reason for exclusion: Single dose study

Johnson1994

- Johnson CR, Handen BL, Lubetsky MJ, Sacco KA. Efficacy of methylphenidate and behavioral intervention on classroom behavior in children with ADHD and mental retardation. *Behav Modif.* 1994;18(4):470-487.

Reason for exclusion: Treatment: Less than seven days treatment

Johnston1988

- Pelham WE, Jr., Sturges J, Hoza J, et al. Sustained release and standard methylphenidate effects on cognitive and social behavior in children with attention deficit disorder. *Pediatrics*. 1987;80(4):491-501. (*According to Storebø et al., 2015, linked to Johnston et al. 1998)*
- Pelham WE, Hoza J. Behavioral assessment of psychostimulant effects on ADD children in a summer day treatment program. In: Prinz R editor(s). Advances in Behavioral Assessment of Children and Families. 3,Greenwich, CT: JAI Press, 1987:3–34.
- Johnston C, Pelham WE, Hoza J, Sturges J. Psychostimulant rebound in attention deficit disordered boys. *J Am Acad Child Adolesc Psychiatry.* 1988(6):806-810.

Reason for exclusion: Less than seven days treatment and placebo conditions, no pre-cross over data

Johnson2008

- Johnson KA, Barry E, Bellgrove MA, et al. Dissociation in response to methylphenidate on response variability in a group of medication naive children with ADHD. *Neuropsychologia*. 2008;46(5):1532-1541.

Reason for exclusion: No RCT

Jonkman1997

- Jonkman LM, Kemner C, Verbaten MN, et al. Effects of methylphenidate on event-related potentials and performance of attention-deficit hyperactivity disorder children in auditory and visual selective attention tasks. *Biol Psychiatry*. 1997;41(6):690-702.

Reason for exclusion: Single dose

Jonkman1998

- Jonkman LM, Verbaten MN, de Boer D, et al. Differences in plasma concentrations of the D- and L-threo methylphenidate enantiomers in responding and non-responding children with attention-deficit hyperactivity disorder. *Psychiatry Res.* 1998;78(1-2):115-118.

Reason for exclusion: No RCT

Jonkman1999

- Jonkman LM, Kemner C, Verbaten MN, et al. Perceptual and response interference in children with attention-deficit hyperactivity disorder, and the effects of methylphenidate. *Psychophysiology.* 1999;36(4):419-429.

Reason for exclusion: Single dose

Jonkman2000

- Jonkman LM, Kemner C, Verbaten MN, et al. Attentional capacity, a probe ERP study: differences between children with attention-deficit hyperactivity disorder and normal control children and effects of methylphenidate. *Psychophysiology.* 2000;37(3):334-346.

Reason for exclusion: No RCT

Jonkman2007

- Jonkman LM, van Melis JJM, Kemner C, Markus CR. Methylphenidate improves deficient error evaluation in children with ADHD: an event-related brain potential study. *Biol Psychol.* 2007;76(3):217-229.

Reason for exclusion: No RCT

Joos2013a

- Joos L, Goudriaan AE, Schmaal L, van den Brink W, Sabbe BGC, Dom G. Effect of modafinil on cognitive functions in alcohol dependent patients: A randomized, placebo-controlled trial. *J Psychopharmacol.* 2013;27:998-1006.

Reason for exclusion: No ADHD

Joos2013b

- Joos L, Goudriaan AE, Schmaal L, et al. Effect of modafinil on impulsivity and relapse in alcohol dependent patients: a randomized, placebo-controlled trial. *Eur Neuropsychopharmacol*. 2013;23:948-55.

Reason for exclusion: No participants with ADHD

JPRN-JMA-IIA00113

- https://dbcentre3.jmacct.med.or.jp/jmactr/App/JMACTRE02_04/JMACTRE02_04.aspx?kbn=3&seqno=3202

Reasons for exclusion: No participants with ADHD

JPRN-UMIN000001878

- https://upload.umin.ac.jp/cgi-open-bin/ctr_e/ctr_view.cgi?recptno=R000002264

Reasons for exclusion: No participants with ADHD

JPRN-UMIN000003033

- https://upload.umin.ac.jp/cgi-open-bin/ctr_e/ctr_view.cgi?recptno=R000003676

Reasons for exclusion: No participants with ADHD

JPRN-UMIN000004187

- https://upload.umin.ac.jp/cgi-open-bin/ctr_e/ctr_view.cgi?recptno=R000005015

Reasons for exclusion: No RCT

JPRN-UMIN000005012

- https://upload.umin.ac.jp/cgi-open-bin/ctr_e/ctr_view.cgi?recptno=R000005959

Reasons for exclusion: No participants with ADHD

JPRN-UMIN000008863

- https://upload.umin.ac.jp/cgi-open-bin/ctr_e/ctr_view.cgi?recptno=R000010407

Reasons for exclusion: No participants with ADHD

JPRN-UMIN000009137

- https://upload.umin.ac.jp/cgi-open-bin/ctr_e/ctr_view.cgi?recptno=R000010710

Reasons for exclusion: No participants with ADHD

JPRN-UMIN000012512

- https://upload.umin.ac.jp/cgi-open-bin/ctr_e/ctr_view.cgi?recptno=R000012083

Reasons for exclusion: No participants with ADHD

JPRN-UMIN000016236

- https://upload.umin.ac.jp/cgi-open-bin/ctr_e/ctr_view.cgi?recptno=R000018843

Reasons for exclusion: No participants with ADHD

JPRN-UMIN000021959

- https://upload.umin.ac.jp/cgi-open-bin/ctr_e/ctr_view.cgi?recptno=R000025291

Reasons for exclusion: No participants with ADHD

JPRN-UMIN000002806

- http://www.umin.ac.jp/ctr/index.htm

Reasons for exclusion: Max 120 mg/day (higher than max dose as per our protocol)

JPRN-UMIN000001542

- http://www.umin.ac.jp/ctr/index.htm

Reasons for exclusion: Non randomised

JPRN-UMIN000007108

- http://www.umin.ac.jp/ctr/index.htm

Reasons for exclusion: No participants with ADHD

JPRN-UMIN000010321

- https://upload.umin.ac.jp/cgi-open-bin/ctr_e/ctr_view.cgi?recptno=R000012083

Reasons for exclusion: No participants with ADHD

Jucaite2014

- Jucaite A, Öhd J, Potter AS, et al. A randomized, double- blind, placebo-controlled crossover study of * nicotinic acetylcholine receptor agonist AZD1446 (TC-6683) in adults with attention-deficit/hyperactivity disorder. *Psychopharmacology (Berl)*. 2014; 231 (6): 1251–1265.

Reason for exclusion: Medication of no interest for the present meta-analysis vs placebo, no other arms

Kaelin1996

- Kaelin DL, Cifu DX, Matthies B. Methylphenidate effect on attention deficit in the acutely brain-injured adult. *Archives of Physical Medicine & Rehabilitation.* 1996;77(1):6-9.

Reason for exclusion: No ADHD

Kaga2003

- Kaga M, Miyamoto S. AD/HD (attention deficit and hyperactivity syndrome) and methylphenidate. [Japanese]. *No To Hattatsu.* 2003;35(2):143-146.

Reason for exclusion: No empirical study

Kang2011

- Kang KD, Choi JW, Kang SG, Han DH. Sports therapy for attention, cognitions and sociality. *Int J Sports Med.* 2011;32(12):953-959.

Reason for exclusion: Methylphenidate + behaviorual intervention vs methylphenidate + sport activity

Kaplan1990

- Kaplan SL, Busner J, Kupietz S, Wassermann E, Segal B. Effects of methylphenidate on adolescents with aggressive conduct disorder and ADDH: a preliminary report. *J Am Acad Child Adolesc Psychiatry.* 1990;29(5):719-723.

Reason for exclusion: No appropriate randomization

Kaschnitz1997

- Kaschnitz W, Rumpelsberger G, Schein G, Rossmann P, Scheer P. Evaluation of specific forms of therapy (EPD, Methylphenidate) in attention deficit-hyperactivity syndrom (ADHS). *Monatsschr Kinderheilkd.* 1997: S41.

Reason for exclusion: No mention to randomization; not possible to contact authors

Kash1997

- Kash IJ. Medication for children with attention disorders. *Pediatrics.* 1997;99:922-3.

Reason for exclusion: Commentary

Kasparbauer2016

- Kasparbauer AM, Meyhofer I, Steffens M, et al. Neural effects of methylphenidate and nicotine during smooth pursuit eye movements. *Neuroimage.* 2016;141:52-59.

Reason for exclusion: Single dose (confirmed by authors)

Kawada2013

- Kawada T. Actigraphic evaluation for patients with attention deficit/hyperactivity disorder. *Am J Med Genet B Neuropsychiatr Genet*. 2013;162B:294.

Reason for exclusion: Commentary

Kayser1997

- Kayser KH, Wacker DP, Derby KM, Andelman MS, Golonka Z, Stoner EA. A rapid method for evaluating the necessity for both a behavioral intervention and methylphenidate. *J Appl Behav Anal.* 1997 1997;30(1):177-180.

Reason for exclusion: Case report

Keating2001

- Keating GM, McClellan K, Jarvis B. Methylphenidate (OROS formulation). *CNS Drugs.* 2001;15(6):495-500; discussion 501-493.

Reason for exclusion: Review, no original data

Kelley2006

- Kelley ME, Fisher WW, Lomas JE, Sanders RQ. Some effects of stimulant medication on response allocation: a double-blind analysis. *J Appl Behav Anal.* 2006;39(2):243-247.

Reason for exclusion: Case report

Kelsey2004

- Kelsey D, Sutton V, Sumner C, Schuh K. Efficacy of tomoxetine for children and adolescents with severe hyperactive and inattentive ADHD symptoms. *Pediatr Res.* 2004;55:1A-2A.

Reason for exclusion: Analysis of 3 Lilly ATMX trials (According to: Lilly, all detected with our search)

Kemner1998

- Kemner C, Jonkman LM, Verbaten MN, Engeland H. The effectiveness of methylphenidate on attention processes in ADHD children [abstract*]. 9th Congress of the Association of European Psychiatrists Copenhagen, Denmark 1998:S24-4.*

Reason for exclusion: Likely single dose; not possible to contact authors

Kemner2004

- Kemner C, Jonkman LM, Kenemans JL, Bocker KB, Verbaten MN, Van Engeland H. Sources of auditory selective attention and the effects of methylphenidate in children with attention-deficit/hyperactivity disorder. *Biol Psychiatry*. 2004;55(7):776-778.

Reason for exclusion: No RCT

Kemner2005 (NCT00866996; NCT00866996)

- Kemner JE. Starr HL, Ciccone PE, Lynch1. OROS provides greater ADHD symptom improvement than atomoxetine. Presented at: *157th Annual Meeting of the American Psychiatric Association, New York, May 1-6, 2004*
- Kemner JE, Starr HL, Bowen DL, et al. Greater symptom improvement and response rates with OROS MPH versus atomoxetine in children with ADHD [abstract]. *Int J Neuropsychopharmacology*. 2004;7:S273-S274
- Kemner JE, Starr HL, Ciccone PE, Hooper-Wood CG, Crockett RS. Outcomes of OROS methylphenidate compared with atomoxetine in children with ADHD: a multicenter, randomized prospective study. *Adv Ther.* 2005;22(5):498-512.
- Starr HL, Kemner J. Multicenter, randomized, open-label study of OROS methylphenidate versus atomoxetine: treatment outcomes in African-American children with ADHD. *J Natl Med Assoc.* 2005;97(10 Suppl):11S-16S.
- https://clinicaltrials.gov/ct2/show/NCT00866996

Reason for exclusion: No blind

Kent1995

- Kent JD, Blader JC, Koplewicz HS, Abikoff H, Foley CA. Effects of late-afternoon methylphenidate administration on behavior and sleep in attention-deficit hyperactivity disorder. *Pediatrics.* 1995;96(2 Pt 1):320-325.

Reason for exclusion: Each Less than seven days treatment

Kent1999

- Kent MA, Camfield CS, Camfield PR. Double-blind methylphenidate trials: practical, useful, and highly endorsed by families. *Arch Pediatr Adolesc Med.* 1999;153(12):1292-1296.

Reason for exclusion: *Authors confirmed all participants > 5 y; however, not able to provide us with pre-cross over data*

Kesic2012

- Kesic A, Lakic A, Dronjak D, Stupar D. Effects of OROS methylphenidate (OROS MPH) treatment in children and adolescents with ADHD, mental retardation and epilepsy. *Eur Neuropsychopharmacol.* 2012;22:S420-S1.

Reason for exclusion: No RCT

Khodadust2012 (IRCT201106306923N1)

- Khodadust N, Jalali AH, Ahmadzad-Asl M, Khademolreza N, Shirazi E. Comparison of two brands of methylphenidate (Stimdate vs. Ritalin) in children and adolescents with attention deficit hyperactivity disorder: A double-blind, randomized clinical trial. *Iran J Psychiatry Behav Sci.* 2012(1):26-32.
- http://www.irct.ir/searchresult.php?id=6923&number=1

Reason for exclusion: Comparison of two formulations of the same compound

Kim2011(NCT01012622)

- Kim B-N, Cummins TDR, Kim J-W, et al. Val/Val genotype of brain-derived neurotrophic factor (BDNF) Val66Met polymorphism is associated with a better response to OROS-MPH in Korean ADHD children. *Int J Neuropsychopharmacol.* 2011;14(10):1399-1410.
- https://clinicaltrials.gov/ct2/show/NCT01012622

Reason for exclusion: Note: Open label. Published paper not retrieved in the search but via the link to NCT number.

Kim2013

- Kim SW, Lee JH, Lee SH, Hong HJ, Lee MG, Yook K-H. ABCB1 c.2677G>T variation is associated with adverse reactions of OROS-methylphenidate in children and adolescents with ADHD. *J Clin Psychopharmacol.* 2013;33(4):491-498.

Reason for exclusion: Open label

Kim2015(NCT01912352)

- Kim JW, Sharma V, Ryan ND. Predicting Methylphenidate Response in ADHD Using Machine Learning Approaches. *Int J Neuropsychopharmacol.* 2015;18(11):pyv052.
- Hong SB, Zalesky A, Park S, et al. COMT genotype affects brain white matter pathways in attention-deficit/hyperactivity disorder. *Hum Brain Mapp.* 2015;36(1):367-377.
- https://clinicaltrials.gov/ct2/show/NCT01912352

Reason for exclusion: Open label

Kinsbourne2001

- Kinsbourne M, De Quiros GB, Rufo DT. Adult ADHD - Controlled medication assessment. *Adult Attention Deficit Disorder.* 2001;931:287-296.

Reason for exclusion: Less than seven days treatment

Kinze1986

- Kinze W, Barchmann H, Ettrich KU. On the pharmacotherapy of school children with disturbances of the concentration and with behavioural peculiarities. [German]. Zur Pharmakotherapie Von Schulkindern Mit Konzentrationsstorungen Und Verhaltensauffalligkeiten. *Z Klin Med.* 1986;41(5):381-383.

Reason for exclusion: Expert opinion paper on Haloperidol compared to placebo, concentration training and Aponeuron

Klein1988

- Klein RG, Landa B, Mattes JA, Klein DF. Methylphenidate and growth in hyperactive children. A controlled withdrawal study. *Arch Gen Psychiatry.* 1988;45(12):1127-1130.

Reason for exclusion: No design of interest for the present meta-analysis

Klein1991

- Klein RG. Effects of high methylphenidate doses on the cognitive performance of hyperactive children. *Bratisl Lek Listy.* 1991;92(11):534-539.

Reason for exclusion: No diagnostic criteria as per protocol

Klein1995

- Klein RG. The role of methylphenidate in psychiatry. *Arch Gen Psychiatry.* 1995;52(6):429-433.

Reason for exclusion: Review

Klein1997a

- Klein RG, Abikoff H, Klass E, Ganeles D, Seese LM, Pollack S. Clinical efficacy of methylphenidate in conduct disorder with and without attention deficit hyperactivity disorder. *Arch Gen Psychiatry.* 1997;54(12):1073-1080.

Reason for exclusion: First author not able to provide us with data on subsample with comorbid ADHD

Klein1997b

- Klein RG, Abikoff H. Behavior therapy and methylphenidate in the treatment of children with ADHD. *J Atten Disord.* 1997;2(2):89-114.

Reason for exclusion: No arms of interest for the present meta-analysis (Stimulants, parent and teacher training, stimulants+ parent and teacher training)

Klein2002

- Klein C, Jr Fischer B, Fischer B, Hartnegg K. Effects of methylphenidate on saccadic responses in patients with ADHD. *Exp Brain Res.* 2002;145(1):121-125.

Reason for exclusion: One day with and one day without MPH

Klein2004
- Klein RG, Abikoff H, Hechtman L, Weiss G. Design and rationale of controlled study of long-term methylphenidate and multimodal psychosocial treatment in children with ADHD. *J Am Acad Child Adolesc Psychiatry.* 2004;43(7):792-801.

Reason for exclusion: No arms of interest for the present meta-analysis

Klopper1980
- Klopper JN, Robertson LI, Logue G, Martins U. Methylphenidate for hyperactivity. *S Afr Med J.* 1980;57(24):979-980.

Reason for exclusion: Commentary

Klopper1987
- Klopper JN. Hyperactivity and methylphenidate. *S Afr Med J.* 1987;71(5):331-332.

Reason for exclusion: Commentary

Klorman1979
- Klorman R, Salzman LF, Pass HL. Effects of methylphenidate on hyperactive children's evoked responses during passive and active attention. *Psychophysiology.* 1979(1):23-29.

Reason for exclusion: Single dose

Klorman1982
- Klorman R, Salzman LF, Bauer LO. Dose-response effects of methylphenidate on performance and the late positive complex of cross-situational and borderline hyperactive children's visual EPs. *Psychophysiology.* 1982;19(5):569.

Reason for exclusion: Abstract only; Dr Klorman not able to provide additional data.

Klorman1983
- Klorman R, Salzman LF, Bauer LO, Coons HW, Borgstedt AD, Halpern WI. Effects of two doses of methylphenidate on cross-situational and borderline hyperactive children's evoked potentials. *Electroencephalogr Clin Neurophysiol.* 1983;56(2):169-185.

Reason for exclusion: Less than seven days treatment

Klorman1987
- PhD thesis: Coons HW. Cognitive and clinical effects of methylphenidate treatment on adolescents with a childhood history of attention deficit disorder. Ann Arbor, The University of Rochester, 1986
- Klorman R, Coons HW, Borgstedt AD. Effects of methylphenidate on adolescents with a childhood history of attention deficit disorder: I. Clinical findings. *J Am Acad Child Adolesc Psychiatry.* 1987;26(3):363-367
- Coons HW, Klorman R, Borgstedt AD. Effects of methylphenidate on adolescents with a childhood history of attention deficit disorder: II. Information processing.[Erratum appears in J Am Acad Child Adolesc Psychiatry 1987 Sep;26(5):820]. *J Am Acad Child Adolesc Psychiatry.* 1987;26(3):368-374.
- Klorman R, Coons HW, Brumaghim JT, Borgstedt AD, Fitzpatrick P. Stimulant treatment for adolescents with attention deficit disorder. *Psychopharmacol Bull.* 1988;24(1):88-92.

Reason for exclusion: No pre cross-over data; Dr Klorman not able to provide additional data

Klorman1988
- Klorman R, Brumaghim JT, Salzman LF, et al. Effects of methylphenidate on attention-deficit hyperactivity disorder with and without aggressive/noncompliant features. *J Abnorm Psychol.* 1988;97(4):413-422
- Klorman R, Brumaghim JT, Salzman LF, et al. Comparative effects of methylphenidate on attention-deficit hyperactivity disorder with and without aggressive/noncompliant features. *Psychopharmacol Bull.* 1989;25(1):109-113.
- Klorman R, Brumaghim JT, Salzman LF, et al. Effects of methylphenidate on processing negativities in patients with attention-deficit hyperactivity disorder. *Psychophysiology.* 1990;27(3):328-337

Reason for exclusion: Cross-over without wash out; No pre cross-over data available

Klorman1990
- Klorman R, Brumaghim JT, Fitzpatrick PA, Borgstedt AD. Clinical effects of a controlled trial of methylphenidate on adolescents with attention deficit disorder. *J Am Acad Child Adolesc Psychiatry.* 1990;29(5):702-709.

- Korman R, Brumaghim JT, Fitzpatrick PA, Borgstedt AD. Methylphenidate speeds evaluation processes of attention deficit disorder adolescents during a continuous performance test. *J Abnorm Child Psychol.* 1991;19(3):263-283.
- Klorman R, Brumaghim JT, Fitzpatrick PA, Borgstedt AD. Methylphenidate reduces abnormalities of stimulus classification in adolescents with attention deficit disorder. *J Abnorm Psychol.* 1992;101(1):130-138.

Reason for exclusion: Cross-over without wash out; No pre cross-over data available

Klorman1994

- Klorman R, Brumaghim JT, Fitzpatrick PA, Borgstedt AD, Strauss J. Clinical and cognitive effects of methylphenidate on children with attention deficit disorder as a function of aggression/oppositionality and age. *J Abnorm Psychol.* 1994;103(2):206-221
- Krusch DA, Klorman R, Brumaghim JT, Fitzpatrick PA, Borgstedt AD, Strauss J. Methylphenidate slows reactions of children with attention deficit disorder during and after an error. *J Abnorm Child Psychol.* 1996;24(5):633-650.

Reason for exclusion: Cross-over without wash out; No pre cross-over data available

Kluge2013

- Kluge M, Hegerl U, Sander C, et al. Methylphenidate in mania project (MEMAP): study protocol of an international randomised double-blind placebo-controlled study on the initial treatment of acute mania with methylphenidate. *BMC Psychiatry.* 2013;13.

Reason for exclusion: Study protocol; empirical study not of interest for the present meta-analysis since co-treatment with mood stabilizers

Knight2007

- Knight M. Stimulant-drug therapy for attention-deficit disorder (with or without hyperactivity) and sudden cardiac death. *Pediatrics.* 2007;119(1):154-155.

Reason for exclusion: Commentary

Knopp1973

- Knopp W, Arnold LE, Andras RL, Smeltzer DJ. Predicting amphetamine response in hyperkinetic children by electronic pupillography. *Pharmakopsychiatr Neuropsychopharmakol.* 1973;6(3):158-166.

Reason for exclusion: No RCT

Koblan2015

- Koblan KS, Hopkins SC, Sarma K, et al. Dasotraline for the Treatment of Attention-Deficit/Hyperactivity Disorder: A Randomized, Double-Blind, Placebo-Controlled, Proof-of-Concept Trial in Adults. *Neuropsychopharmacology.* 2015;40(12):2745-2752.

Reason for exclusion: Medication of no interest for the present meta-analysis (dasotraline) vs placebo

Koblan2016

- Koblan KS, Hopkins SC, Sarma K, et al. Assessment of human abuse potential of dasotraline compared to methylphenidate and placebo in recreational stimulant users. *Drug Alcohol Depend.* 2016;159:26-34.

Reason for exclusion: No participants with ADHD (Healthy recreational CNS stimulant users)

Kocher2015

- Kocher J, Adams P. Immediate-release methylphenidate for the treatment of ADHD in adults. *Am Fam Physician.* 2015;91(7):445-446.

Reason for exclusion: Commentary on: Epstein T, Patsopoulos NA, Weiser M. Immediate-release methylphenidate for attention deficit hyperactivity disorder (ADHD) in adults. Cochrane Database Syst Rev. 2014;9:CD005041

Kohn2015 (ANZCTRN12607000535471)

- Protcol: Tsang TW, Kohn MR, Hermens DF, et al. A randomized controlled trial investigation of a non-stimulant in attention deficit hyperactivity disorder (ACTION): rationale and design. *Trials.* 2011;12:77.
- Tsang TW, Kohn MR, Clarke SD, Williams LM. Cognition and emotion in child and adolescent ADHD. *Biol Psychiatry.* 2012;71(8):74S.
- Tsang TW, Kohn MR, Clarke SD, Williams LM. Cognitive and emotion predictors of response to atomoxetine in children and adolescents with attention deficit hyperactivity disorder, with and without comorbid anxiety. *Biol Psychiatry.* 2013;73(9):47S.
- Kohn MR, Griffiths KR, Clarke S, et al. Pharmacological mediation of cognition in children and adolescents presenting with cross-disorder symptoms of adhd and anxiety. *Biol Psychiatry.* 2015;77(9): 119S.
- http://www.anzctr.org.au/ACTRN12607000535471.aspx

Reason for exclusion: Authors contacted to gather full-text; reply: paper under submission, not possible to share data

Kollins1998

- Kollins SH, Shapiro SK, Newland MC, Abramowitz A. Discriminative and participant-rated effects of methylphenidate in children diagnosed with attention deficit hyperactivity disorder (ADHD). *Exp Clin Psychopharmacol.* 1998;6(4):375-389.

Reason for exclusion: Quasi-randomized

Kollins2006 (NCT00018863)

- Greenhill LL. Preschool ADHD treatment study (PATS): science and controversy. Economics of Neuroscience2001;3(5):49–53. [EMBASE: 2001251865]
- Kratochvil CJ, Greenhill LL, March JS, Burke WJ, Vaughan BS. The role of stimulants in the treatment of preschool children with attention-deficit hyperactivity disorder. *CNS drugs.* 2004;18(14):957-966.
- Kollins S, Greenhill L, Swanson J, et al. Rationale, design, and methods of the Preschool ADHD Treatment Study (PATS). *J Am Acad Child Adolesc Psychiatry.* 2006;45(11):1275-1283.
- No authors listed. At a glance... study design sought to balance rigor,subject safety. *The Brown University Child & Adolescent Psychopharmacology Update* 2006;8(12):4–5.
- No authors listed . MPH-related reductions in growth rates. *The Brown University Child & Adolescent Psychopharmacology Update* 2006;8(12):6.
- No authors listed . PATS: safety and tolerability of MPH in ADHD preschoolers. *The Brown University Child & AdolescentPsychopharmacology Update* 2006;8(12):2–4No authors listed . PATS: efficacy of MPH in ADHD preschoolers. T*he Brown University Child & Adolescent Psychopharmacology Update* 2006;8(12):5–6.
- What are the effects of methylphenidate treatment in preschoolers with ADHD? Results from the PreschoolADHD Treatment Study (PATS*). The Brown University Child & Adolescent Psychopharmacology Update* 2006;8 (12):1–2.
- No authors listed. Young ADHD patients may improve with low-dose meds. *Psychiatr Ann.* 2006; 36(12):826.
- Greenhill L, Kollins S, Abikoff H, et al. Efficacy and safety of immediate-release methylphenidate treatment for preschoolers with ADHD. *J Am Acad Child Adolesc Psychiatry.* 2006;45(11):1284-1293.
- Greenhill LL, Kollins S, Abikoff H, McCracken J, RiddleM, Swanson J, et al. Efficacy and safety of immediatereleasemethylphenidate treatment for preschoolers withADHD. *J Am Acad Child Adolesc Psychiatry.* 2006;45(11):1284–93.
- McGough J, McCracken J, Swanson J, Riddle M, Kollins S,Greenhill L, et al. Pharmacogenetics of methylphenidate response in preschoolers with ADHD. *J Am Acad Child Adolesc Psychiatry*. 2006;45(11):1314–22.
- Swanson J, Greenhill L, Wigal T, Kollins S, Stehli A, DaviesM, et al. Stimulant-related reductions of growth rates in the PATS. *J Am Acad Child Adolesc Psychiatry*. 2006;45(11):1304–13.
- Skrobala A, et al. Safety and tolerability of methylphenidatein preschool children with ADHD. *J Am Acad Child Adolesc Psychiatry*. 2006;45(11):1294–303.
- Wigal T, Greenhill L, Chuang S, McGough J, Vitiello B,Skrobala A, et al. Safety and tolerability of methylphenidatein preschool children with ADHD. *J Am Acad Child Adolesc Psychiatry*. 2006;45(11):1294–303.
- Kollins SH, Greenhill L. Evidence base for the use of stimulant medication in preschool children with ADHD. *Infants Young Child*. 2006;19(2):132–41.
- Wigal T, Greenhill L, Chuang S, et al. Safety and tolerability of methylphenidate in preschool children with ADHD. *J Am Acad Child Adolesc Psychiatry*. 2006;45(11):1294-1303.
- Greenhill LL, Kollins S, Abikoff H, McCracken J, RiddleM, Swanson J, et al. Erratum: "Efficacy and safetyof immediate-release MPH treatment for preschoolers with ADHD *J Am Acad Child Adolesc Psychiatry*. 2007;46(1):141. Erratum for: *J Am Acad Child Adolesc Psychiatry.* 2006;45:1284-93.
- Abikoff HB, Vitiello B, Riddle MA, Cunningham C,Greenhill LL, Swanson JM, et al. Methylphenidate effects on functional outcomes in the Preschoolers with Attention-Deficit/Hyperactivity Disorder Treatment Study (PATS). *J Child Adolesc Psychopharmacol.* 2007;17(5):581–92.
- Murray DW, Kollins SH, Hardy KK, et al. Parent versus teacher ratings of attention-deficit/hyperactivity disorder symptoms in the Preschoolers with Attention-Deficit/Hyperactivity Disorder Treatment Study (PATS). *J Child Adolesc Psychopharmacol.* 2007;17(5):605-620.
- Posner K, Melvin GA, Murray DW, et al. Clinical presentation of attention-deficit/hyperactivity disorder in preschool children: the Preschoolers with Attention-Deficit/Hyperactivity Disorder Treatment Study (PATS). *J Child Adolesc Psychopharmacol.* 2007;17(5):547-562.
- Swanson JM, Moyzis RK, McGough JJ, et al. Effects of source of DNA on genotyping success rates and allele percentages in the Preschoolers with Attention-Deficit/Hyperactivity Disorder Treatment Study (PATS). *J Child Adolesc Psychopharmacol.* 2007;17(5):635-646.
- Ghuman JK, Riddle MA, Vitiello B, Greenhill LL, Chuang SZ, Wigal SB, et al. Comorbidity moderates response to methylphenidate in the Preschoolers with Attention-Deficit/Hyperactivity Disorder Treatment Study (PATS). *J Child Adolesc Psychopharmacol.* 2007;17(5):563–80.

- Hardy KK, Kollins SH, Murray DW, et al. Factor structure of parent- and teacher-rated attention-deficit/hyperactivity disorder symptoms in the Preschoolers with Attention-Deficit/Hyperactivity Disorder Treatment Study (PATS). *J Child Adolesc Psychopharmacol.* 2007;17(5):621-634.
- Vitiello B, Abikoff HB, Chuang SZ, Kollins SH,McCrackenJT, Riddle MA, et al. Effectiveness of methylphenidate in the 10-month continuation phase of the Preschoolers withADHD Treatment Study (PATS). *J Child Adolesc Psychopharmacol*. 2007;17(5):593–603.
- Wagner KD. Methylphenidate treatment of ADHD inpreschoolers. Psychiatric Times 2007;24(3):47.
- Reiff MI. Journal article reviews: attention-deficit/hyperactivity disorder. *J Dev Behav Pediatr*. 2007; Vol. 28, issue 1:71–2.
- Riddle MA. New findings from the Preschoolers withAttention-Deficit/Hyperactivity Disorder Treatment Study(PATS). *J Child Adolesc Psychopharmacol.* 2007;17(5):543–6
- Wigal SB, Gupta S, Greenhill L, et al. Pharmacokinetics of methylphenidate in preschoolers with attention-deficit/hyperactivity disorder. *J Child Adolesc Psychopharmacol*. 2007;17(2):153-164.
- No authors listed . PATS shows mixed effect of medication on functional outcomes. The Brown University Child & Adolescent Psychopharmacology Update 2008; Vol. 10, issue 2:6.
- March JS. The preschool ADHD Treatment Study (PATS) as the culmination of twenty years of clinical trials inpediatric psychopharmacology. *J Am Acad Child Adolesc Psychiatry.* 2011;50(5):427–30.
- Riddle MA, Yershova K, Lazzaretto D, Paykina N, YenokyanG, Greenhill L, et al. The Preschool Attention-Deficit/Hyperactivity Disorder Treatment Study (PATS) 6-yearfollow-up. *J Am Acad Child Adolesc Psychiatry*. 2013;52(3):264–78.e2.
- Riddle MA, Yershova K, Lazzaretto D, et al. The Preschool Attention-Deficit/Hyperactivity Disorder Treatment Study (PATS) 6-year follow-up. *J Am Acad Child Adolesc Psychiatry.* 2013;52(3):264-278.e262.
- Vitiello B, Lazzaretto D, Yershova K, et al. Pharmacotherapy of the Preschool ADHD Treatment Study (PATS) Children Growing Up. *J Am Acad Child Adolesc Psychiatry.* 2015;54(7):550-556.
- https://clinicaltrials.gov/ct2/show/NCT00018863

Reason for exclusion: PATS study. Age range not pertinent for the present meta-analysis (Pre-schoolers)

Kollins2009

- Kollins SH, English J, Robinson R, Hallyburton M, Chrisman AK. Reinforcing and subjective effects of methylphenidate in adults with and without attention deficit hyperactivity disorder (ADHD). *Psychopharmacology (Berl).* 2009;204(1):73-83.

Reason for exclusion: Less than seven days treatment

Kollins2011

- Kollins SH, Jain R, Brams M, et al. Clonidine extended-release tablets as add-on therapy to psychostimulants in children and adolescents with ADHD. *Pediatrics.* 2011;127(6):e1406-1413.

Reason for exclusion: Clonidine or placebo as add on to stimulants

Kollins2014

- Kollins SH, English JS, Itchon-Ramos N, et al. A pilot study of lis-dexamfetamine dimesylate (LDX/SPD489) to facilitate smoking cessation in nicotine-dependent adults with ADHD. *J Atten Disord.* 2014;18(2):158-168.

Reason for exclusion: Co-treatment with nicotine patch

Kollins2016

- Kollins SH, Cutler AJ, Khattak S, Weiss MD, Donnelly G, Reiz SJL. A randomized double-blind placebo controlled multicenter study measuring the efficacy and safety of a novel, extended-release formulation of methylphenidate (prc-063) in adolescents with attention-deficit/hyperactivity disorder. *J Am Acad Child Adolesc Psychiatry.* 2016;55 (10 Supplement 1):S217-S218.

Reason for exclusion: Abstract only; contacted authors to retrieve full text but it was not available

Kolko1999

- Kolko DJ, Bukstein OG, Barron J. Methylphenidate and behavior modification in children with ADHD and comorbid ODD or CD: main and incremental effects across settings. *J Am Acad Child Adolesc Psychiatry.* 1999;38(5):578-586.

Reason for exclusion: Less than seven days treatment

Konrad2004

- Konrad K, Gunther T, Hanisch C, Herpertz-Dahlmann B. Differential effects of methylphenidate on attentional functions in children with attention-deficit/hyperactivity disorder. *J Am Acad Child Adolesc Psychiatry.* 2004;43(2):191-198.
- Konrad K, Gunther T, Heinzel-Gutenbrunner M, Herpertz-Dahlmann B. Clinical evaluation of subjective and objective changes in motor activity and attention in children with attention-deficit/hyperactivity disorder in a double-blind methylphenidate trial. *J Child Adolesc Psychopharmacol.* 2005;15(2):180-190. (Overlap of participants between this and the previous reference confirmed by first author)

Reason for exclusion: Less than seven days treatment

Konstenius2010
- Konstenius M, Jayaram-Lindstrom N, Beck O, Franck J. Sustained release methylphenidate for the treatment of ADHD in amphetamine abusers: a pilot study. *Drug Alcohol Depend.* 2010;108(1-2):130-133.
- Konstenius M, Jayaram N, Guterstam J, Franck J. Pharmacological treatment of ADHD with amphetamine dependence. *Acta Neuropsychiatr.* 2013;25(S1): 13-14.

Reason for exclusion: Co-treatment: skills training programme

Konstenius2014 (EUCTR2006-002249-35-SE; ISRCTN77940178)
- Konstenius M, Jayaram N, Guterstam J, Franck J. Pharmacological treatment of ADHD with amphetamine dependence. *Acta Neuropsychiatrica.* 2013; 25(S1): 13-14..
- Konstenius M, Jayaram-Lindstrom N, Guterstam J, Philips B, Beck O, Franck J. Methylphenidate for adhd in adults with substance dependence: A 24-week randomized placebo-controlled trial. *Eur Psychiatry.* 2013;28.
- Konstenius M, Jayaram-Lindstrom N, Guterstam J, Beck O, Philips B, Franck J. Methylphenidate for attention deficit hyperactivity disorder and drug relapse in criminal offenders with substance dependence: a 24-week randomized placebo-controlled trial. *Addiction.* 2014;109(3):440-449.
- https://www.clinicaltrialsregister.eu/ctr-search/search?query=eudract_number:2006-002249-35
- http://isrctn.org/ISRCTN77940178

Reason for exclusion: Dose (180 mg/day) of MPH above licensed one and also above doses recommended in guidelines; Co-treatment (psychotherapy for addiction)

Kooij2001
- Kooij JJ, Middelkoop HA, van Gils K, Buitelaar JK. The effect of stimulants on nocturnal motor activity and sleep quality in adults with ADHD: an open-label case-control study. *J Clin Psychiatry.* 2001;62(12):952-956.

Reason for exclusion: No RCT

Kortekaas-Rijlaarsdam 2017 (NCT02501798)
- Kortekaas-Rijlaarsdam AF, Luman M, Sonuga-Barke E, Bet PM, Oosterlaan J. Short-term effects of methylphenidate on math productivity in children with attention-deficit/hyperactivity disorder are mediated by symptom improvements: Evidence from a placebo-controlled trial. *J Clin Psychopharmacol.* 2017;37:210-219.
- https://clinicaltrials.gov/ct2/show/NCT02501798

Reason for exclusion: First author confirmed that participants were responders to methylphenidate

Kosters2007
- Kosters M, Weinmann S, Becker T. [Methylphenidate in adults with attention-deficit/hyperactivity disorder]. *Nervenarzt.* 2007;78(9):1065-1066; author reply 1066-1068.

Reason for exclusion: Commentary

Kouris1998
- Kouris S. Methylphenidate-induced obsessive-compulsiveness. *J Am Acad Child Adolesc Psychiatry.* 1998;37(2):135.

Reason for exclusion: Case report

Krager1974
- Krager JM, Safer DJ. Type and prevalence of medication used in the treatment of hyperactive children. *N Engl J Med.* 1974;291(21):1118-1120.

Reason for exclusion: Survey

Krakowski1965
- Krakowski, AJ. Amitriptyline in treatment of hyperkinetic children. A double-blind study. *Psychosomatics.* 1965;6(5):355-60

Reason for exclusion: No DSM/ICD criteria

Kramer2001
- Kramer AF, Cepeda NJ, Cepeda ML. Methylphenidate effects on task-switching performance in attention-deficit/hyperactivity disorder. *J Am Acad Child Adolesc Psychiatry.* 2001;40(11):1277-1284.

Reason for exclusion: No RCT

Kratochvil2001
- Kratochvil CJ, Bohac D, Harrington M, Baker N, May D, Burke, WJ. An open-label trial of tomoxetine in pediatric attention deficit hyperactivity disorder. *J Child Adolesc Psychopharmacol*. 2001;11(2):167-70

Reason for exclusion: Open label

Kratochvil2002
- Kratochvil CJ, Heiligenstein JH, Dittmann R, et al. Atomoxetine and methylphenidate treatment in children with ADHD: a prospective, randomized, open-label trial. *J Am Acad Child Adolesc Psychiatry.* 2002;41(7):776-784.

Reason for exclusion: Open label

Kratochvil2005
- Kratochvil CJ, Newcorn JH, Arnold LE, et al. Atomoxetine alone or combined with fluoxetine for treating ADHD with comorbid depressive or anxiety symptoms. *J Am Acad Child Adolesc Psychiatry.* 2005;44(9):915-924.

Reason for exclusion: ATMX+fluoxetine vs. ATMX+placebo

Kratochvil2007
- Kratochvil CJ, Michelson D, Newcorn JH, et al. High-dose atomoxetine treatment of ADHD in youths with limited response to standard doses. *J Am Acad Child Adolesc Psychiatry.* 2007;46(9):1128-1137.

Reason for exclusion: participants responders to previous ADHD medications

Kratochvil2011 (NCT00254462; K23MH066127)
- Kratochvil CJ, Vaughan BS, Stoner JA, et al. A double-blind, placebo-controlled study of atomoxetine in young children with ADHD. *Pediatrics.* 2011;127(4):e862-868.
- https://clinicaltrials.gov/ct2/show/NCT00254462

Reason for exclusion: Co-treatment with behavioral strategies

Kratz2012
- Kratz O, Studer P, Baack J, et al. Differential effects of methylphenidate and atomoxetine on attentional processes in children with ADHD: an event-related potential study using the Attention Network Test. *Prog Neuropsychopharmacol Biol Psychiatry.* 2012;37(1):81-89.

Reason for exclusion: No blind

Kuehn2009
- Kuehn BM. Stimulant use linked to sudden death in children without heart problems. *JAMA.* 2009;302(6):613-614.

Reason for exclusion: Commentary

Kulendran2016
- Kulendran M, Wingfield LR, Sugden C, Darzi A, Vlaev I. Pharmacological manipulation of impulsivity: A randomized controlled trial. *Pers Individ Dif.* 2016;90:321-325.

Reason for exclusion: No participants with ADHD

Kummer2008
- Kummer A, Teixeira A. Methylphenidate in attention deficit hyperactivity disorder and bipolar disorder. *Australas Psychiatry.* 2008;16(6):458-459.

Reason for exclusion: Case reports

Kuperman2001
- Perry PJ, Gaffney GR, Bever Stille K, Holman T, Paulsen J. Bupropion sustained release versus methylphenidate versus placebo in the treatment of adult adhd. *153rd Annual Meeting of the American Psychiatric Association.* 2000
- Kuperman S, Perry PJ, Gaffney GR, et al. Bupropion SR vs. methylphenidate vs. placebo for attention deficit hyperactivity disorder in adults. *Ann Clin Psychiatry.* 2001;13(3):129-134.
- Perry Paul J. Bupropion sustained release versus methylphenidate versus placebo in the treatment of adult adhd. *155th Annual*

Meeting of the American Psychiatric Association. 2002.
Reason for exclusion: No usable data

Kuperman2003
- Kuperman AA, Yaniv I, Stahl B, Tamary H. Methylphenidate as a possible cause of thrombocytopenia. *Ann Pharmacother.* 2003;37(7-8):1146.

Reason for exclusion: Case reports

Kupietz1976
- Kupietz SS, Balka EB. Alterations in the vigilance performance of children receiving amitriptyline and methylphenidate pharmacotherapy. *Psychopharmacology (Berl).* 1976;50(1):29-33.

Reason for exclusion: No DSM/ICD criteria

Kupietz1982
- Kupietz SS, Winsberg BG, Sverd J. Learning ability and methylphenidate (Ritalin) plasma concentration in hyperkinetic children. A preliminary investigation. *J Am Acad Child Psychiatry.* 1982;21(1):27-30.

Reason for exclusion: No RCT

Kupietz1988
- Kupietz SS, Winsberg BG, Richardson E, Maitinsky S, Mendell N. Effects of methylphenidate dosage in hyperactive reading-disabled children: I. Behavior and cognitive performance effects. *J Am Acad Child Adolesc Psychiatry.* 1988(1):70-77.
- Richardson E, Kupietz SS, Winsberg BG, Maitinski S, Mendell N. Effects of methylphenidate dosage in hyperactive reading-disabled children: II. Reading achievement. *J Am Acad Child Adolesc Psychiatry.* 1988(1):78-87.

Reason for exclusion: No usable data

Kupietz1991
- Kupietz SS, Richardson E, Winsberg BG. Stimulants and school performance. *J Am Acad Child Adolesc Psychiatry.* 1991;30(2):335.

Reason for exclusion: Commentary

Kurlan2002
- Kurlan R. Methylphenidate to treat ADHD is not contraindicated in children with tics. *Mov Disord.* 2002;17(1):5-6.

Reason for exclusion: Commentary

Lage2004
- Lage M, Hwang P. Effect of methylphenidate formulation for attention deficit hyperactivity disorder on patterns and outcomes of treatment. *J Child Adolesc Psychopharmacol.* 2004;14(4):575-581.

Reason for exclusion: No RCT

Lajoie2005
- Lajoie G, Anderson V, Anderson P, Tucker AR, Robertson IH, Manly T. Effects of Methylphenidate on Attention Skills in Children with Attention Deficit/Hyperactivity Disorder. *Brain Impair.* 2005;6(1):21-32.

Reason for exclusion: No mention to randomization; Less than seven days treatment

Lanctot2014
- Lanctot KL, Chau SA, Herrmann N, et al. Effect of methylphenidate on attention in apathetic AD patients in a randomized, placebo-controlled trial. *Int Psychogeriatr.* 2014;26(2):239-246.

Reason for exclusion: No participants with ADHD

Langleben2006
- Langleben DD, Monterosso J, Elman I, Ash B, Krikorian G, Austin G. Effect of methylphenidate on Stroop Color-Word task performance in children with attention deficit hyperactivity disorder. *Psychiatry Res.* 2006;141(3):315-320.

Reason for exclusion: No RCT

Larue2008
- Larue RH, Jr., Northup J, Baumeister AA, et al. An evaluation of stimulant medication on the reinforcing effects of play. *J Appl Behav Anal.* 2008;41(1):143-147.

Reason for exclusion: No RCT

Lasser2009

- Lasser R, Weisler R, Young J, et al. Long-term safety and efficacy of lisdexamfetamine dimesylate in adults with attention-deficit/hyperactivity disorder. *Eur Neuropsychopharmacol.* 2009;19:S356.

Reason for exclusion: Open label

Lawrence2005

- Lawrence CA, Barry RJ, Clarke AR, et al. Methylphenidate effects in attention deficit/hyperactivity disorder: electrodermal and ERP measures during a continuous performance task. *Psychopharmacology (Berl).* 2005;183(1):81-91.

Reason for exclusion: No RCT

Leary1986

- Leary PM. Hyperactivity and methylphenidate. *S Afr Med J.* 1986;70(7):383-384.

Reason for exclusion: Editorial

Leddy2009

- Leddy JJ, Waxmonsky JG, Salis RJ, et al. Dopamine-related genotypes and the dose-response effect of methylphenidate on eating in attention-deficit/hyperactivity disorder youths. *J Child Adolesc Psychopharmacol.* 2009;19(2):127-136.

Reason for exclusion: Less than 7 consecutive days treatment for each condition; treatment (behavioral therapy)

Lee2004

- Lee T-SW, Lee TD, Lombroso PJ, King RA. Atomoxetine and tics in ADHD. *J Am Acad Child Adolesc Psychiatry.* 2004;43(9):1068-1069.

Reason for exclusion: Case reports

Lee2005a

- Lee JS, Kim BN, Kang E, et al. Regional cerebral blood flow in children with attention deficit hyperactivity disorder: comparison before and after methylphenidate treatment. *Hum Brain Mapp.* 2005;24(3):157-164.

Reason for exclusion: No RCT

Lee2005b

- Lee, H, Kim, SW, Kim, JM, Shin, IS, Yang, SJ, Yoon, JS. Comparing effects of methylphenidate, sertraline and placebo on neuropsychiatric sequelae in patients with traumatic brain injury. *Hum Psychopharmacol.* 2005; 20: 97–104

Reason for exclusion: No participants with ADHD

Lee2007

- Lee JH, Jung CH, Song CJ, et al. Multi-center study for evaluation of efficacy and safety of methylphenidate-OROS in children with ADHD. *Eur Neuropsychopharmacol.* 2007;17:S571-S2.

Reason for exclusion: Open label

Lee2008

- Lee M-S, Yang J-W, Ko Y-H, et al. Effects of methylphenidate and bupropion on DHEA-S and cortisol plasma levels in attention-deficit hyperactivity disorder. *Child Psychiatry Hum Dev.* 2008;39(2):201-209.

Reason for exclusion: Open label

Lee2013

- Lee SH, Seox WS, Sung HM, et al. Effect of methylphenidate on sleep parameters in children with ADHD. *Psychiatry Investig.* 2013(1):384-390.

Reason for exclusion: Trial comparing 2 different formulations of methylphenidate; no placebo arm

Leitner2007

- Leitner Y, Doniger GM, Barak R, Simon ES, Hausdorff JM. A novel multidomain computerized cognitive assessment for attention-deficit hyperactivity disorder: evidence for widespread and circumscribed cognitive deficits. *J Child Neurol.* 2007;22(3):264-276.

Reason for exclusion: Less than seven days treatment

Leonhard2006

- Leonhard C, Reif A, Beck M, Jacob C, Lesch K-P. Reversible ischaemic neurological deficit associated with short-term methylphenidate medication. *Int J Neuropsychopharmacol.* 2006;9(1):129-130.

Reason for exclusion: Case reports

Lerer1976

- Lerer RJ, Lerer MP. The effects of methylphenidate on the soft neurological signs of hyperactive children. *Pediatrics.* 1976;57(4):521-525.

Reason for exclusion: No DSM/ICD criteria

Lerer1979

- Lerer RJ, Lerer MP, Artner J. The effects of methylphenidate on the handwriting of children with minimal brain dysfunction. *J Pediatr.* 1977;91(1):127-132.
- Lerer RJ, Artner J, Lerer MP. Handwriting deficits in children with minimal brain dysfunction: effects of methylphenidate (Ritalin) and placebo. *J Learn Disabil.* 1979;12(7):450-455.

Reason for exclusion: No DSM/ICD criteria

Lerner2000

- Lerner MA, Modi NB, Gupta S. Optimizing methylphenidate delivery to improve treatment for ADHD: results with OROS (methylphenidate HCI). *Pediatr Res*. 2000:29a.

Reason or exclusion: Healthy adults

Levin1996

- Levin ED, Conners CK, Sparrow E, Hinton SC, Erhardt D, Meck WH, Rose JE, March, J. Nicotine effects on adults with attention-deficit/hyperactivity disorder. *Psychopharmacology (Berl).* 1996 ;123(1):55-63

Reason for exclusion: Active drug trial: 4 h

Levin1998

- Levin FR, Evans SM, McDowell DM, Kleber HD. Methylphenidate treatment for cocaine abusers with adult attention-deficit/hyperactivity disorder: a pilot study. *J Clin Psychiatry.* 1998;59(6):300-305.
- Levin FR, Evans SM, Kleber HD. Methylphenidate Treatment for Cocaine Abusers with Adult Attention-Defict/Hyperactivity Disorder. *NIDA Res Monogr.* 1999:39.

Reason for exclusion: Co-treatment with psychotherapy

Levin2001

- Levin ED, Conners CK, Silva D, Canu W, March J. Effects of chronic nicotine and methylphenidate in adults with attention deficit/hyperactivity disorder. *Exp Clin Psychopharmacol.* 2001;9(1):83-90.

Reason for exclusion: No outcomes and no arms of interest for the present meta-analysis (placebo patch + placebo pill (control), nicotine patch + placebo pill (nicotine), placebo patch + methylphenidate pill (methylphenidate), and nicotine patch + methylphenidate pill (nicotine + methylphenidate)

Levin2006 (NCT00061087)

- Levin FR, Evans SM, Brooks D, Sullivan M, Nunes E, Vosburg S. Treatment of adult ADHD in methadone maintenance patients: Preliminary findings from a double-blind, three-armed, placebo-controlled trial. *Drug Alcohol Depend.* 2002;66:S102.
- Levin FR, Evans SM, Brooks DJ, Kalbag AS, Garawi F, Nunes EV. Treatment of methadone-maintained patients with adult ADHD: double-blind comparison of methylphenidate, bupropion and placebo. *Drug Alcohol Depend.* 2006;81(2):137-148.
- https://clinicaltrials.gov/ct2/show/NCT00061087

Reason for exclusion: *Co-treatment*

Levin2007 (NCT00136734)

- Levin FR, Evans SM, Brooks DJ, Garawi F. Treatment of cocaine dependent treatment seekers with adult ADHD: double-blind comparison of methylphenidate and placebo. *Drug Alcohol Depend.* 2007;87(1):20-29.
- https://clinicaltrials.gov/ct2/show/NCT00136734

Reason for exclusion: Co-treatment with cognitive therapy

Levin2015 (NCT00553319)

- Levin FR, Mariani JJ, Specker S, et al. Extended-Release Mixed Amphetamine Salts vs Placebo for Comorbid Adult

Attention-Deficit/Hyperactivity Disorder and Cocaine Use Disorder: A Randomized Clinical Trial. *JAMA Psychiatry*. 2015;72(6):593-602.

- Levin FR, Mariani JJ, Mahony A, et al. Mixed amphetamine salts-extended release for ADHD adults with cocaine use disorder. *Drug Alcohol Depend.* 2015;146:e175.
- Notzon D, Mariani JJ, Pavlicova M, et al. Mixed-amphetamine salts increase abstinence from marijuana in patients with co-occurring attention-deficit/hyperactivity disorder and cocaine dependence. Drug *Alcohol Depend.* 2015;156:e164.
- Notzon DP, Mariani JJ, Pavlicova M, Glass A, Mahony AL, Brooks DJ, Grabowski J, Levin FR. Mixed-amphetamine salts increase abstinence from marijuana in patients with co-occurring attention-deficit/hyperactivity disorder and cocaine dependence. *Am J Addict*. 2016;25(8):666-672
- https://clinicaltrials.gov/ct2/show/NCT00553319

Reason for exclusion: Co-treatment with cognitive therapy

Levy1988

- Levy F, Hobbes G. The action of stimulant medication in attention deficit disorder with hyperactivity: dopaminergic, noradrenergic, or both? *J Am Acad Child Adolesc Psychiatry.* 1988;27(6):802-805.

Reason for exclusion: Less than seven days treatment

Levy1996

- Levy F, Hobbes G. Does haloperidol block methylphenidate? Motivation or attention? *Psychopharmacology (Berl).* 1996;126(1):70-74.

Reason for exclusion: Less than seven days treatment

Lewis1975

- Lewis JA, Young R. Deanol and methylphenidate in minimal brain dysfunction. *Clin Pharmacol Ther.* 1975;17(5):534-540.

Reason for exclusion: No DSM/ICD criteria

Li1999

- Li X, Chen Z. Clinical comparative observation on duodongning and Ritalin in treating child hyperkinetic syndrome. [Chinese]. *Zhongguo Zhong Xi Yi Jie He Za Zhi.* 1999;19(7):410-411.

Reason for exclusion: Medication of interest vs. med of no interest for the present meta-analysis, without placebo

Li2011

- Li JJ, Li ZW, Wang SZ, et al. Ningdong granule: a complementary and alternative therapy in the treatment of attention deficit/hyperactivity disorder. *Psychopharmacology (Berl).* 2011;216(4):501-509.

Reason for exclusion: Medication of interest vs. med of no interest for the present meta-analysis, without placebo

Li2013

- Li L, Yang L, Zhuo CJ, Wang YF. A randomised controlled trial of combined EEG feedback and methylphenidate therapy for the treatment of ADHD. *Swiss Med Wkly.* 2013;143:w13838.

Reason for exclusion: Methylphenidate+EEG feedback vs. methylphenidate +attention training

Licamele1988

- Licamele WL. Methylphenidate side effects. *J Am Acad Child Adolesc Psychiatry.* 1988;27(4):515-516.

Reason for exclusion: Commentary, no empirical data

Lieberman2000

- Lieberman SC. *The effect of an afternoon dose of methylphenidate on the on-task, accuracy and productivity of the homework completed by children with Attention Deficit Hyperactivity Disorder* [Ph.D.]. Ann Arbor, University of Kansas; 1999.
- Lieberman SG, Christophersen ER. The effect of an afternoon dose of methylphenidate on the accuracy and on-task behavior of the homework completed by children with attention deficit hyperactivity disorder. *Pediatr Res.* 2000(4):29a.
- Lieberman SC. The effect of an afternoon dose of methylphenidate on the on-task, accuracy and productivity of the homework completed by children with attention deficit hyperactivity disorder. *Dissertation Abstracts International: Section B: The Sciences and Engineering.* 2000;60(8-B):4233.

Reason for exclusion: Three studies aimed to assess effects of a third dose of methylphenidate on performing homework in three children

Lijffijt2006

- Lijffijt M, Kenemans JL, ter Wal A, et al. Dose-related effect of methylphenidate on stopping and changing in children with attention-deficit/hyperactivity disorder. *Eur Psychiatry.* 2006;21(8):544-547.

Reason for exclusion: Less than seven days treatment for each condition

Lim2012 (NCT01344044)

- Lim CG, Lee TS, Guan C, Fung DS, Zhao Y, Teng SS, Zhang H, Krishnan KR. A brain-computer interface based attention training program for treating attention deficit hyperactivity disorder. *PLoS One*. 2012;7(10):e46692
- https://clinicaltrials.gov/ct2/show/NCT01344044

Reason for exclusion: No pharmacological interventions

Ling2014

- Ling W, Chang L, Hillhouse M, et al. Sustained-release methylphenidate in a randomized trial of treatment of methamphetamine use disorder. *Addiction.* 2014;109(9):1489-1500.
- Commentary in: Levin FR, Mariani JJ, Bisaga A, Nunes EV. Ling et al.'s 'Sustained-release methylphenidate in a randomized trial of treatment of methamphetamine use disorder'. *Addiction.* 2015;110(5):875-876.
- Ang A, Hillhouse M, Jenkins J, Reed S, Ling W. Methylphenidate for methamphetamine use disorders in participants with and without ADHD. *Drug Alcohol Depend.* 2015;156:e7

Reason for exclusion: No participants with ADHD; Co-treatment with group CBT

Lion-Francois2014

- Lion-Francois L, Gueyffier F, Mercier C, et al. The effect of methylphenidate on neurofibromatosis type 1: a randomised, double-blind, placebo-controlled, crossover trial. *Orphanet J Rare Dis.* 2014;9:142.

Reason for exclusion: ADHD in inherited condition

Lissek2015

- Lissek S, Glaubitz B, Gunturkun O, Tegenthoff M. Noradrenergic stimulation modulates activation of extinction-related brain regions and enhances contextual extinction learning without affecting renewal. *Front Behav Neurosci.* 2015;9.

Reason for exclusion: No participants with ADHD (healthy volunteers)

Litton2005

- Litton P. ADHD, values, and the self. *Am J Bioeth.* 2005;5(3):65-67; discussion W10-62.

Reason for exclusion: Commentary, no empirical data

Liu2007

- Liu J, Zhou Y, Kang C, Xuan X, Wang Y. A Randomized comparative study on impact of methylphenidate with/without parent training in children with comorbid attention deficit hyperactivity disorder and oppositional defiant disorder. *J Neural Transm.* 2007;114(7):XCVII-XCVII.

Reason for exclusion: Methylphenidate vs. Methylphenidate +parent training, no placebo arm

Livingston1992

- Livingston RL, Dykman RA, Ackerman PT. Psychiatric comorbidity and response to two doses of methylphenidate in children with attention deficit disorder. *J Child Adolesc Psychopharmacol.* 1992;2(2):115-122.

Reason for exclusion: Two doses of Methylphenidate, no placebo

Llorente2006

- Llorente AM, Voigt RG, Jensen CL, Berretta MC, Kennard Fraley J, Heird WC. Performance on a visual sustained attention and discrimination task is associated with urinary excretion of norepineprhine metabolite in children with attention-deficit/hyperactivity disorder (AD/HD). *Clin Neuropsychol.* 2006;20(1):133-144.

Reason for exclusion: No RCT

Logemann2013

- Logemann HN, Bocker KB, Deschamps PK, Kemner C, Kenemans JL. The effect of noradrenergic attenuation by clonidine on inhibition in the stop signal task. *Pharmacol Biochem Behav.* 2013;110:104-111.

Reason for exclusion: Healthy participants

Loo2003

- Loo SK, Specter E, Smolen A, Hopfer C, Teale PD, Peite ML. Functional Effects of the DAT1 Polymorphism on EEC Measures in ADHD. *J Am Acad Child Adolesc Psychiatry.* 2003;42(8):986-993.

Reason for exclusion: Single dose

Loo2004
- Loo SK, Teale PD, Reite, ML. EEG correlates of methylphenidate response among children with ADHD: A preliminary report. *Biol Psychiatry*. 1999;45(12):1657-60
- Loo SK, Hopfer C, Teale PD, Reite ML. EEG correlates of methylphenidate response in ADHD: association with cognitive and behavioral measures. *J Clin Neurophysiol.* 2004;21(6):457-464.

Reason for exclusion: Less than seven days treatment

Looby2011
- Looby A, Earleywine M. Expectation to receive methylphenidate enhances subjective arousal but not cognitive performance. *Exp Clin Psychopharmacol.* 2011;19(6):433-444.

Reason for exclusion: Less than seven days treatment

Lopez2003a
- Lopez FA, Chandler MC, Biederman J, Mays DA, Michals MA, Tulloch SJ. Long-term adderall extended release treatment improves quality of life in ADHD children. *156th Annual Meeting of the American* Psychiatric *Association; 2003 May 17-22; San Francisco, CA.* 2003:Nr650.
- McGough JJ, Biederman J, Wigal SB, et al. Long-term tolerability and effectiveness of once-daily mixed amphetamine salts (Adderall XR) in children with ADHD. *J Am Acad Child Adolesc Psychiatry.* 2005;44(6):530-538.

Reason for exclusion: Open-label (confirmed by first author)

Lopez2003b (CRIT124DUS05)
- Lopez F, Silva R, Pestreich L, Muniz R. Comparative efficacy of two once daily methylphenidate formulations (Ritalin LA and Concerta) and placebo in children with attention deficit hyperactivity disorder across the school day. *Paediatr Drugs.* 2003;5(8):545-555. (Erratum in Lopez F, Silva R, Pestreich L, Muniz R. Erratum for: Comparative efficacy of two once daily methylphenidate formulations (Ritalin LA1 and Concerta) and placebo in children with attention deficit hyperactivity disorder across the school day. *Paediatr Drugs.* 2003;5(12):832.
- Lopez FA, Silva RR, Pestreich L, Lee J, Muniz R. Comparative school-day efficacy of Ritalin LA, Concerta, and placebo in children with attention deficit hyperactivity disorder. Ann Neurol. Proceedings of the 32nd Annual Meeting of the Child Neurology Society; 2003. October 1-4; Miami Beach, Florida. New York: John Wiley & Sons, 2003; Vol. 54 (Suppl 7):S143.

Reason for exclusion: Single blind, subjects stabilised on methylphenidate

Lopez2004
- Lopez J, Lopez V, Rojas D, et al. Effect of psychostimulants on distinct attentional parameters in attentional deficit/hyperactivity disorder.[Erratum appears in Biol Res. 2004;37(4):713]. *Biol Res.* 2004;37(3):461-468.

Reason for exclusion: No RCT

Lopez2006
- Lopez FA, Childress A, Brams M, et al. Response to extended-release dexmethylphenidate in ethnically diverse children with ADHD: A 12-hour placebo-controlled laboratory classroom study. *Int J Neuropsychopharmacol.* 2006;9(Suppl. 1):S228-S229.
- Lopez F, Muniz R, McCague K. Treatment of children with ADHD from different ethnic groups with extended release dexmethylphenidate and D,L-methylphenidate: A pooled analysis of two 12-hour placebo-controlled laboratory classroom studies. *J Child Adolesc Psychopharmacol.* 2007;17(6):875-876.

Reason for exclusion: Pooled two RCT in which participants were previously responders to MPH

Lopez2007
- Lopez F, Muniz R, McCague K. Treatment of children with ADHD from different ethnic groups with extended release dexmethylphenidate and D,L-methylphenidate: A pooled analysis of two 12-hour placebo-controlled laboratory classroom studies. *J Child Adolesc Psychopharmacol.* 2007;17(6):875-876.

Reason for exclusion: Abstract presenting pooled analysis of two studies. Participants responders to previous ADHD medications

Lord2000

- Lord J, Paisley S. The clinical effectiveness and cost effectiveness of methylphenidate for hyperactivity in childhood (Provisional abstract). *Database of Abstracts of Reviews of Effects.* 2000(1):64. http://onlinelibrary.wiley.com/o/cochrane/cldare/articles/DARE-12004008144/frame.html.

Reason for exclusion: Provisional abstract of systematic review: no related full text found

Lu2006

- Lu C-K, Kuang T-M, Chou JC-K. Methylphenidate (Ritalin)-associated cataract and glaucoma. *J Chin Med Assoc: JCMA.* 2006;69(12):589-590.

Reason for exclusion: Case reports

Lubar1999

- Lubar JF, White JN, Jr., Swartwood MO, Swartwood JN. Methylphenidate effects on global and complex measures of EEG. *Pediatr Neurol.* 1999;21(3):633-637.

Reason for exclusion: No RCT

Lubow2005

- Lubow RE, Braunstein-Bercovitz H, Blumenthal O, Kaplan O, Toren P. Latent inhibition and asymmetrical visual-spatial attention in children with ADHD. *Child Neuropsychol.* 2005;11(5):445-457.

Reason for exclusion: No RCT

Lufi1997

- Lufi D, Parish-Plass J, Gai E. The effect of methylphenidate on the cognitive and personality functioning of ADHD children. *Isr J Psychiatry Relat Sci.* 1997;34(3):200-209.

Reason for exclusion: Less than seven consecutive days treatment

Lufi2007

- Lufi D, Gai E. The effect of methylphenidate and placebo on eye-hand coordination functioning and handwriting of children with attention deficit hyperactivity disorder. *Neurocase*. 2007;13(5):334-341.

Reason for exclusion: Less than seven consecutive days treatment

Lufi2015

- Lufi D, Bassin-Savion S, Rubel L. The effect of methylphenidate on sustained attention among adolescents with attention-deficit hyperactivity disorder. *Neurocase.* 2015;21(6):802-808.

Reason for exclusion: Single dose

Luman2015

- Luman M, Papanikolau A, Oosterlaan J. The Unique and Combined Effects of Reinforcement and Methylphenidate on Temporal Information Processing in Attention-Deficit/Hyperactivity Disorder. *J Clin Psychopharmacol.* 2015;35(4):414-421.

Reason for exclusion: First author confirmed study is semi-random

Lyon2008

- Lyon G, Coffey B, Castellanos XF, Woods D. Improving TIC-related response inhibition: Comparing the effects of dexmethylphenidate to placebo in children and adolescents with ADHD and chronic TIC disorders. *Int J Neuropsychopharmacol*. 2008;11:292.

Reason for exclusion: Less than seven days treatment

Lyon2011

- Lyon MR, Kapoor MP, Juneja LR. The Effects of L-Theanine (Suntheanine (R)) on Objective Sleep Quality in Boys with Attention Deficit Hyperactivity Disorder (ADHD): a Randomized, Double-blind, Placebo-controlled Clinical Trial. *Altern Med Rev.* 2011;16(4):348-354.

Reason for exclusion: Medication (vs. placebo) not relevant for our meta-analysis

Maayan2003

- Maayan R, Yoran-Hegesh R, Strous R, et al. Three-month treatment course of methylphenidate increases plasma levels of dehydroepiandrosterone (DHEA) and dehydroepiandrosterone-sulfate (DHEA-S) in attention deficit hyperactivity disorder. *Neuropsychobiology.* 2003;48(3):111-115.

Reason for exclusion: No RCT

MacDonald2005

- MacDonald Fredericks E, Kollins SH. A pilot study of methylphenidate preference assessment in children diagnosed with attention-deficit/hyperactivity disorder. *J Child Adolesc Psychopharmacol.* 2005;15(5):729-741.

Reason for exclusion: No RCT; Less than seven days treatment

MacKeith1971

- Mac Keith RC. [Therapy of hyperactive children]. *Ceskoslovenska Pediatrie.* 1971;26(12):591-592.

Reason for exclusion: Not possible to contact author; however, given date of publication, No DSM/ICD criteria

Mackay1973

- Mackay MC, Beck L, Taylor R. Methylphenidate for adolescents with minimal brain dysfunction. *N Y State J Med.* 1973;73(4):550-554.

Reason for exclusion: No DSM/ICD criteria, case reports

Mahon2008

- Mahon AD, Stephens BR, Cole AS. Exercise responses in boys with attention deficit/hyperactivity disorder: effects of stimulant medication. *J Atten Disord.* 2008;12(2):170-176.

Reason for exclusion: No RCT

Mahon2012

- Mahon AD, Woodruff ME, Horn MP, Marjerrison AD, Cole AS. Effect of Stimulant Medication Use by Children With ADHD on Heart Rate and Perceived Exertion. *Adapt Phys Activ Q.* 2012;29(2):151-160.

Reason for exclusion: No RCT

Malek-Ahmadi1999

- Malek-Ahmadi P. Bupropion, periodic limb movement disorder, and ADHD. *J Am Acad Child Adolesc Psychiatry.* 1999;38(6):637-638.

Reason for exclusion: Case report

Malone1988

- Malone MA, Kershner JR, Siegel L. The effects of methylphenidate on levels of processing and laterality in children with attention deficit disorder. *J Abnorm Child Psychol.* 1988;16(4):379-395.

Reason for exclusion: Less than seven days treatment

Malone1993

- Malone MA, Swanson JM. Effects of methylphenidate on impulsive responding in children with attention-deficit hyperactivity disorder. *J Child Neurol.* 1993;8(2):157-163.

Reason for exclusion: Single dose

Malone1994a

- Malone MA, Kershner JR, Swanson JM. Hemispheric processing and methylphenidate effects in attention-deficit hyperactivity disorder. *J Child Neurol.* 1994;9(2):181-189.

Reason for exclusion: Review

Malone1994b

- Malone MA, Couitis J, Kershner JR, Logan WJ. Right-hemisphere dysfunction and methylphenidate effects in children with attention-deficit hyperactivity disorder. *J Child Adolesc Psychopharmacol.* 1994;4(4):245-253.

Reason for exclusion: Single dose

Mangold1975

- Mangold B. [Drug therapy of minimal brain dysfunction syndrome (clinical study using Captagon)]. *Prax Kinderpsychol Kinderpsychiatr.* 1975;24(5):185-190.

Reason for exclusion: No DSM/ICD criteria, no RCT, no medications of interest

Manor2008

- Manor I, Meidad S, Zalsman G, Zemishlany Z, Tyano S, Weizman A. Objective versus subjective assessment of methylphenidate response. *Child Psychiatry Hum Dev.* 2008;39(3):273-282.

Reason for exclusion: No RCT; single dose

Manor2011

- Manor I, Rozen S, Zemishlani Z, Weizman A, Zalsman G. When does it end? Attention-deficit/hyperactivity disorder in the middle aged and older populations. *Clin Neuropharmacol.* 2011;34(4):148-154.

Reason for exclusion: No RCT

Manor2012(NCT01243242)

- Manor I, Ben-Hayun R, Aharon-Peretz J, Salomy D, Weizman A, Daniely Y, Megiddo D, Newcorn JH, Biederman J, Adler LA (2012) A randomized, double-blind, placebo-controlled, multi- center study evaluating the efficacy, safety, and tolerability of extended-release metadoxine in adults with attention-deficit/ hyperactivity disorder. *J Clin Psychiatry.* 2012;73(12):1517-23
- Manor I, Newcorn JH, Faraone SV, Adler LA. Efficacy of Metadoxine Extended Release in Patients With Predominantly Inattentive Subtype Attention-Deficit/Hyperactivity Disorder. *Postgrad Med.* 2013;125(4):181-190.
- https://clinicaltrials.gov/ct2/show/NCT01243242

Reason for exclusion: No medication of interest (metadoxine) for the present meta-analysis vs placebo; no other arms

Manor2014(NCT01685281)

- Manor I, Rubin J, Daniely Y, Adler LA. Attention Benefits After a Single Dose of Metadoxine Extended Release in Adults With Predominantly Inattentive ADHD. *Postgrad Med.* 2014;126(5):7-16.
- https://clinicaltrials.gov/ct2/show/NCT01685281

Reason for exclusion: No medication of interest (metadoxine vs placebo); no other arms

Manos1999

- Manos MJ, Short EJ, Findling RL. Differential effectiveness of methylphenidate and Adderall(R) in school-age youths with attention-deficit/hyperactivity disorder. *J Am Acad Child Adolesc Psychiatry.* 1999;38(7):813-819.
- Manos MJ, Short EJ, Findling RL. Dose response curves across ADHD subtypes: differential effects between adderall and methylphenidate. *Pediatr Res*. 2000(4):30a.
- Findling RL, Short EJ, Manos MJ. Developmental aspects of psychostimulant treatment in children and adolescents with attention-deficit/hyperactivity disorder. *J Am Acad Child Adolesc Psychiatry.* 2001;40(12):1441-1447.
- Faraone SV, Short EJ, Biederman J, Findling RL, Roe C, Manos MJ. Efficacy of Adderall and methylphenidate in attention deficit hyperactivity disorder: a drug-placebo and drug-drug response curve analysis of a naturalistic study. *The Int J Neuropsychopharmacol.* 2002;5(2):121-129.

Reason for exclusion: Design not suitable for a NMA (issue on terms of transitivity)

Manos2015

- Manos MJ, Caserta DA, Short EJ, et al. Evaluation of the Duration of Action and Comparative Effectiveness of Lisdexamfetamine Dimesylate and Behavioral Treatment in Youth With ADHD in a Quasi-Naturalistic Setting. *J Atten Disord.* 2015;19(7):578-590.

Reason for exclusion: Design not pertinent for the present meta-analysis

Manza2016

- Manza P, Hu S, Ide JS, et al. The effects of methylphenidate on cerebral responses to conflict anticipation and unsigned prediction error in a stop-signal task. *J Psychopharmacol.* 2016;30(3):283-293.

Reason for exclusion: No participants with ADHD (healthy volunteers)

Manzi2002

- Manzi S, Law T, Shannon MW. Methylphenidate produces a false-positive urine amphetamine screen. *Pediatr Emerg Care.* 2002;18(5):401.

Reason for exclusion: Letter to the Editor, no RCT

Maoz2014

- Maoz H, Tsviban L, Gvirts HZ, et al. Stimulants improve theory of mind in children with attention deficit/hyperactivity disorder. *J Psychopharmacol.* 2014;28(3):212-219.

Reason for exclusion: No RCT

Marchant2011 (NCT00506285; SLI381-404)

- Marchant BK, Reimherr FW, Robison RJ, Olsen JL, Kondo DG. Methylphenidate transdermal system in ADHD adhd and impact on emotional and oppositional symptoms. *J Atten Disord.* 2011;15(4):295-304.
- Olsen JL, Reimherr FW, Marchant BK, Wender PH, Robison RJ. The effect of personality disorder symptoms on response to treatment with methylphenidate transdermal system in adults with attention-deficit/hyperactivity disorder. *Prim Care Companion CNS Disord.* 2012;14(5).

- Reimherr FW, Marchant BK, Olsen JL, Wender PH, Robison RJ. Oppositional defiant disorder in adults with ADHD. *J Atten Disord.* 2013;17(2):102-113.
- Gift TE, Reimherr FW, Marchant BK, Steans TA, Wender PH. Personality Disorder in Adult Attention-Deficit/Hyperactivity Disorder: Attrition and Change During Long-term Treatment. *J Nerv Ment Dis.* 2016;204(5):355-63.
- https://clinicaltrials.gov/ct2/show/NCT00506285

Reason for exclusion: No oral formulations

Marchant2013

- Marchant BK, Reimherr FW, Robison D, Robison RJ, Wender PH. Psychometric properties of the wender-reimherr adult attention deficit disorder scale. *Psychol Assess.* 2013;25(3):942-950.

Reason for exclusion: No RCT; refers to 5 RCTs, all retrieved in our search

Marchei2013

- Marchei E, Papaseit E, Garcia-Algar O, et al. Sweat testing for the detection of atomoxetine from paediatric patients with attention deficit/ hyperactivity disorder: application to clinical practice. *Drug Test Anal.* 2013;5(3):191-195.

Reason for exclusion: No RCT

Marcus2005

- Marcus SC, Wan GJ, Kemner JE, Olfson M. Continuity of methylphenidate treatment for attention-deficit/hyperactivity disorder.[Erratum appears in *Arch Pediatr Adolesc Med*. 2005;159(9):875]. *Arch Pediatr Adolesc Med.* 2005;159(6):572-578.

Reason for exclusion: No RCT

Martin1967

- Martin DM. Hyperkinetic behavior disorders in children: clinical results with methylphenidate hydrochloride (Ritalin). *West Med Med J West.* 1967;8(1):23-27.

Reason for exclusion: Review-case reports

Martin2002

- Martin CA, Kelly TH, Guenthner G, Lane SD, Bingcang C. Methylphenidate effects on task performance in ADHD adolescents. *Drug Alcohol Depend.* 2002;66(Supplement 1):S112.

Reason for exclusion: Less than seven days treatment

Martin2007

- Martin CA, Guenthner G, Bingcang C, Rayens MK, Kelly TH. Measurement of the subjective effects of methylphenidate in 11- to 15-year-old children with attention-deficit/hyperactivity disorder. *J Child Adolesc Psychopharmacol.* 2007;17(1):63-73.

Reason for exclusion: Less than seven days treatment

Martin2014 (NCT01010750)

- Martin PT, Corcoran M, Zhang P, Katic A. Randomized, double-blind, placebo-controlled, crossover study of the effects of lisdexamfetamine dimesylate and mixed amphetamine salts on cognition throughout the day in adults with attention-deficit/hyperactivity disorder. *Clin Drug Investig.* 2014;34(2):147-157.
- https://clinicaltrials.gov/ct2/show/NCT01010750

Reason for exclusion: "Individuals with a history of successful treatment with an amphetamine-based agent": this is an issue in terms of transitivity property for the NMA, so agreed to exclude this study

Martins2004

- Martins S, Tramontina S, Polanczyk G, Eizirik M, Swanson JM, Rohde LA. Weekend holidays during methylphenidate use in ADHD children: a randomized clinical trial. *J Child Adolesc Psychopharmacol.* 2004;14(2):195-206.

Reason for exclusion: Placebo: Less than seven days treatment

Martsenkovsky 2008

- Martsenkovsky I, Martsenkovska II, Bikshaeva YB. Milnacipran and atomoxetine: treatment of depressive disorder with co-morbid hyperactivity disorder. *Eur Neuropsychopharmacol*. 2008;18:S373-S4.

- Martsenkovsky I, Melakh I, Bikshaeva Y. Milnacipran and atomoxetine efficacy over time in adolescents and adults with depression who have comorbid attention-deficit/hyperactivity disorder. *Int J Neuropsychopharmacol.* 2008;11:199.

Reason for exclusion: Medication of interest (atomoxetine) vs medication of no interest (milnacipram) for the present meta-analysis

Mattes1982
- Mattes JA, Boswell L, Oliver H. Methylphenidate in adults with minimal brain dysfunction. *Psychopharmacol Bull*, 18;41(11):114-115

Reason for exclusion: No RCT

Mattes1984
- Mattes JA, Boswell L, Oliver H. Methylphenidate effects on symptoms of attention deficit disorder in adults. *Arch Gen Psychiatry.* 1984;41(11):1059-1063.

Reason for exclusion: No DSM/ICD criteria

Mattes1985
- Mattes, J. Methylphenidate in mild depression: a double-blind controlled trial. *J Clin Psychiatry.*1985;46(12):525-7

Reason for exclusion: No participants with ADHD

Mattison2010
- Mattison DR. Research on cytogenetic risk of ADHD treatments in children. *J Atten Disord.* 2010;14(3):203-204.

Reason for exclusion: Commentary, no empirical data

Maffla1981
- Maffla, AG. Double-blind assessment of the activity of minaprine (30038-CB) in child psychiatry. *Pharmatherapeutica.* 1981;2(9):601-6

Reason for exclusion: Not an ADHD/hypekinetic sample (predominant diagnosis of sample was depression)

Martsenkovsky2008
- Martsenkovsky I, Melakh I, Bikshaeva Y. Milnacipran and atomoxetine efficacy over time in adolescents and adults with depression who have comorbid attention-deficit/hyperactivity disorder. *Int J Neuropsychopharmacol.* 2008;11(Suppl. 1):199.

Reason for exclusion: Abstract only; not possible to contact the authors

Martsenkovsky2015
- Martsenkovsky I, Inna M. Milnacipran and atomoxetine in the treatment of adolescents with Attention-Deficit/Hyperactivity Disorder. *ADHD Atten Defic Hyperact Disord.* 2015;7:S46.

Reason for exclusion: Abstract only; not possible to contact the authors

Martsenkovsky2015
- Martsenkovsky I, Martsenkovska I, Martsenkovskyi D. Risperidon and atomoxetine in the treatment of several and challending behaviors in children with PDD. *Eur Psychiatry.* 2015;30:195.
- Martsenkovska I. Risperidone and atomoxetine in the treatment of severe and challenging behaviours in children with pervasive developmental disorders. *Eur Neuropsychopharmacol.* 2015;25:S649.

Reason for exclusion: Abstract only; not possible to contact the authors

Masand2005
- Masand PS, Patkar AA, Peindl K, Hooper-Wood C, Ciccone PE, Blazer D. A randomized, double-blind, placebo-controlled, flexible-dose, trial of augmentation with oros methylphenidate in treatment resistant depression. *Neuropsychopharmacology.* 2005;30(Suppl. 1):S180.

Reason for exclusion: No participants with ADHD

Matier1992
- Matier K, Halperin JM, Sharma V, Newcorn JH, Sathaye N. Methylphenidate response in aggressive and nonaggressive ADHD children: distinctions on laboratory measures of symptoms. *J Am Acad Child Adolesc Psychiatry.* 1992;31(2):219-225.

Reason for exclusion: Single dose

Mooney1993

- Mooney, GF, Haas, LJ (1993) Effect of methylphenidate on brain injury-related anger. *Arch Phys Med Rehabil.* 1993;74(2):153-60

Reason for exclusion: No participants with ADHD

Mayes1993

- Mayes SD, Bixler EO. Reliability of global impressions for assessing methylphenidate effects in children with attention-deficit hyperactivity disorder. *Percept Mot Skills.* 1993;77(3 Pt 2):1215-1218.

Reason for exclusion: No randomization, Less than seven days treatment (at least for some participants)

Mayes1994

- Mayes SD, Crites DL, Bixler EO, Humphrey FJ, 2nd, Mattison RE. Methylphenidate and ADHD: influence of age, IQ and neurodevelopmental status. *Dev Med Child Neurol.* 1994;36(12):1099-1107.

Reason for exclusion: No RCT; ABA methylphenidate vs no methylphenidate

Maziade2009 (NCT00216918; B4Z-CA-S013)

- Maziade M, Rouleau N, Lee B, Rogers A, Davis L, Dickson R. Atomoxetine and neuropsychological function in children with attention-deficit/hyperactivity disorder: results of a pilot study. *J Child Adolesc Psychopharmacol.* 2009;19(6):709-718.
- Pooled in: Dickson RA, Maki E, Gibbins C, Gutkin SW, Turgay A, Weiss MD. Time courses of improvement and symptom remission in children treated with atomoxetine for attention-deficit/hyperactivity disorder: analysis of Canadian open-label studies. *Child Adolesc Psychiatry Ment Health.* 2011;5:14.
- https://clinicaltrials.gov/ct2/show/NCT00216918

Reason for exclusion: Open label

McBride1988

- McBride MC. An individual double-blind crossover trial for assessing methylphenidate response in children with attention deficit disorder. *J Pediatr.* 1988;113(1 Pt 1):137-145.

Reason for exclusion: Co-treatments during the study (including behavioral treatment) Cross-over without wash out; no pre-cross over data available

McConnell 1964

- McConnell TR, Jr, Cromwell RL, Bialer I, Son CD. Studies in activity level: VII. Effects of amphetamine drug administration on the activity level of retarded children. *Am J Ment Defic*1964;68(5):647–651

Reason for exclusion: No DSM/ICD criteria

McCracken2003

- McCracken JT, Biederman J, Greenhill LL, et al. Analog classroom assessment of a once-daily mixed amphetamine formulation, SLI381 (Adderall XR), in children with ADHD. *J Am Acad Child Adolesc Psychiatry.* 2003;42(6):673-683.
- Used for a pooled long term analysis is in: McGough JJ, Biederman J, Wigal SB, et al. Long-term tolerability and effectiveness of once-daily mixed amphetamine salts (Adderall XR) in children with ADHD. *J Am Acad Child Adolesc Psychiatry.* 2005;44(6):530-538.
- Findling RL, Biederman J, Wilens TE, et al. Short- and long-term cardiovascular effects of mixed amphetamine salts extended release in children. *J Pediatr.* 2005;147(3):348-354.

Reason for exclusion: Some participants had a history of response to stimulants. Participants entered the randomized phase only if they tolerated well the study drug in an initial open label day

McDonnel2016

- McDonnell M, Wigal S, Childress A, et al. A treatment optimization study of HLD200 in children with attention-deficit/hyperactivity disorder. *Ann Neurol.* 2016;80:S392.

Reason for exclusion: Optimization phase

McDougle2004

- McDougle CJ. Methylphenidate an effective treatment for ADHD? *J Autism Dev Disord.* 2004;34(5):593-594.

Reason for exclusion: Commentary

McElroy2015

- McElroy SL, Hudson JI, Mitchell JE, et al. Efficacy and safety of lisdexamfetamine for treatment of adults with moderate to severe binge-eating disorder: a randomized clinical trial. *JAMA Psychiatry.* 2015;72(3):235-246.

- McElroy SL, Mitchell JE, Wilfley D, et al. Lisdexamfetamine Dimesylate Effects on Binge Eating Behaviour and Obsessive-Compulsive and Impulsive Features in Adults with Binge Eating Disorder. *Eur Eat Disord Rev*. 2016;24(3):223-231.
- McElroy S, Hudson J, Ferreira-Cornwell MC, Radewonuk J, Gasior M. Randomized controlled safety and efficacy trials of lisdexamfetamine dimesylate for adults with moderate to severe binge eating disorder. *CNS spectrums. Conference: 2014 NEI psychopharmacology congress. United states. Conference start: 20141113. Conference end: 20141116.* 2017;20(1):74.

Reason for exclusion: No participants with ADHD

McElroy2016
- Naser N, McElroy S, Hudson J, Ferreira-Cornwell MC, Radewonuk J, Gasior M. Lisdexamfetamine dimesylate for adults with moderate to severe binge eating disorder: Results of two randomized controlled safety and efficacy trials. *Aust N Z J Psychiatry.* 2015;49:116.
- McElroy SL, Hudson J, Ferreira-Cornwell MC, Radewonuk J, Whitaker T, Gasior M. Lisdexamfetamine Dimesylate for Adults with Moderate to Severe Binge Eating Disorder: Results of Two Pivotal Phase 3 Randomized Controlled Trials. *Neuropsychopharmacology.* 2016;41(5):1251-1260.

Reason for exclusion: No participants with ADHD

McGough2003
- McGough JJ, Biederman J, Greenhill LL, et al. Pharmacokinetics of SLI381 (ADDERALL XR), an extended-release formulation of Adderall. *J Am Acad Child Adolesc Psychiatry.* 2003;42(6):684-691.

Reason for exclusion: No outcome of interest available; no pre cross over data; initial selection of patients: "subjects who tolerated the initial study day and exposure to SLI381 were subsequently randomized in a 5-week, double-blind, crossover"

McGough2006a
- McGough J, McCracken J, Swanson J, et al. Pharmacogenetics of methylphenidate response in preschoolers with ADHD. *J Am Acad Child Adolesc Psychiatry.* 2006;45(11):1314-1322.

Reason for exclusion: preschoolers

McGough2006b (NCT00466791)
- McGough JJ, Wigal SB, Abikoff H, Turnbow JM, Posner K, Moon E. A randomized, double-blind, placebo-controlled, laboratory classroom assessment of methylphenidate transdermal system in children with ADHD. *J Atten Disord*.2006;9(3):476-485.
- Wigal S, Turnbow J, Abikoff H, McGough J, Cohen J. Parent rated effects of transdermal methylphenidate in children with ADHD. *Int J Neuropsychopharmacol.* 2008;11(Suppl. 1):232.
- https://clinicaltrials.gov/ct2/show/NCT00466791

Reason for exclusion: No oral formulations

McGough2009
- McGough JJ, McCracken JT, Loo SK, et al. A candidate gene analysis of methylphenidate response in attention-deficit/hyperactivity disorder. *J Am Acad Child Adolesc Psychiatry*. 2009;48(12):1155-1164

Reason for exclusion: Cross-over without wash out; pre-cross over data not available

McInnes2007
- McInnes A, Bedard A-C, Hogg-Johnson S, Tannock R. Preliminary evidence of beneficial effects of methylphenidate on listening comparison in children with attention-deficit/hyperactivity disorder. *J Child Adolesc Psychopharmacol.* 2007;17(1):35-49.

Reason for exclusion: Less than seven days treatment

McIntyre1981
- McIntyre HB, Firemark HM, Cho AK, Bodner L, Gomez M. Computer analyzed EEG in amphetamine-responsive hyperactive children. *Psychiatry Res.* 1981;4(2):189-197.

Reason for exclusion: No DSM/ICD criteria

McLaren2010
- McLaren JL, Cauble S, Barnett RJ. Aripiprazole induced acute dystonia after discontinuation of a stimulant medication. *J Clin Psychopharmacol.* 2010;30(1):77-78.

Reason for exclusion: Case report

McLaughlin1980

- McLaughlin JF, Tso Y. Double-blind trials with stimulants for hyperactivity. *Pediatrics.* 1980;66(3):481-482.

Reason for exclusion: Commentary, no empirical data

McLeod2009

- McLeod M, Laubscher T, Regier L, Jensen B. Taking the stress out of individualizing ADHD drug therapy. *Can Fam Physician.* 2009;55(9):895-898.

Reason for exclusion: Case report

McManis1978

- McManis DL, McCarthy M, Koval R. Effects of a stimulant drug on extraversion level in hyperactive children. *Percept Mot Skills.* 1978;46(1):88-90.

Reason for exclusion: No RCT; no DSM/ICD criteria

McNutt1977

- McNutt BA, Boileau RA, Cohen MN. The effects of long-term stimulant medication on the growth and body composition of hyperactive children [proceedings]. *Psychopharmacol Bull.* 1977;13(2):36-38.

Reason for exclusion: No RCT

Meek2005

- Meek IL, Hunt RD, Vestal BS. Personality Factors Affecting Patients' Preferences Among Medications. *158th Annual Meeting of the American Psychiatric Association; 2005 May 21-26;* Atlanta*, GA.* 2005:Nr39.

Reason for exclusion: Abstract only; no contact for authors

Mehta2000

- Mehta MA, Owen AM, Sahakian BJ, Mavaddat N, Pickard JD, Robbins TW. Methylphenidate enhances working memory by modulating discrete frontal and parietal lobe regions in the human brain. *J Neurosci.* 2000;20(6):RC65.

Reason for exclusion: No RCT

Mehta2004

- Mehta MA, Goodyer IM, Sahakian BJ. Methylphenidate improves working memory and set-shifting in AD/HD: relationships to baseline memory capacity. *J Child Psychol Psychiatry.* 2004;45(2):293-305.

Reason for exclusion: Single dose

Meisel2014

- Meisel V, Servera M, Garcia-Banda G, Cardo E, Moreno I. Reprint of "Neurofeedback and standard pharmacological intervention in ADHD: a randomized controlled trial with six-month follow-up".[Reprint of *Biol Psychol.* 2013;94(1):12-21; PMID: 23665196]. *Biol Psychol.* 2014;95:116-125.

Reason for exclusion: No arms of interest for the present meta-analysis (Methylphenidate vs neurofeedback)

Melamed2004

- Melamed I, Bender BG, Wamboldt, MZ. The benefit of using Ceterizine (Zyrtec) with stimulant in children with comorbid allergy and ADHD. *J Allergy Clin Immunol,2004;113(2):S162.*
- Melamed I, Heffron M. Attention Deficit Disorder and Allergic Rhinitis: Are They Related? *J Immunol Res.* 2016;1596828. (Contacted authors to ask confirmation this reference is related to the previous one but no reply)

Reason for exclusion: Compounds of no interest for the present meta-analysis

Mendez2011

- Mendez L, Singh P, Harrison G, Huang Y-S, Jin X, Cho SC. Academic outcomes in Asian children aged 8-11 years with attention-deficit/hyperactivity disorder treated with atomoxetine hydrochloride. *Int J Psychiatry Clin Pract.* 2011;15(2):145-156.

Reason for exclusion: Open label

Merkel2000

- Merkel RL, Cox DJ, Kovatchev B, et al. The EEG consistency index as a measure of ADHD and responsiveness to medication. *Appl Psychophysiol Biofeedback.* 2000;25(3):133-142.

Reason for exclusion: Single dose

Meyer-Probst1976

- Meyer-Probst B, Vehreschild T. [Influencing the lack of concentration in hyperkinetic school children with Aponeuron]. *Psychiatr Neurol Med Psychol (Leipz).* 1976;28(8):491-499.

Reason for exclusion: No RCT

Michael1981

- Michael RL, Klorman R, Salzman LF. Normalizing effects of methylphenidate on hyperactive children's vigilance performance and evoked potentials. *Psychophysiology*. 1981;18(6):665-77

Reason for exclusion: Single dose

Michelson2003a

- Michelson D, Adler L, Spencer T, Milton D, Jones D. Long-term treatment effects of atomoxetine in adults with attention-deficit/hyperactivity disorder (ADHD). *Eur Neuropsychopharmacol*. 2003;13(Supplement 4):458

Reason for exclusion: Open label phase

Michelson2003b

- Michelson D, Spencer T, Ruff D, Feldman PD. Long-term effects of atomoxetine on growth in children with ADHD. *Eur Neuropsychopharmacol*. 2003;13:S458-S9

Reason for exclusion: Data from all Lilly studies on ATMX; according to Lilly, the present meta-analysis included all their studies on atomoxetine

Michelson2004 (B4Z-MC-LYAF)

- Michelson D, Zhang S, Buitelaar J, et al. Results From a Long- Term Trial of Atomoxetine in the Prevention of Relapse in ADHD. *156th Annual Meeting of the American Psychiatric Association, May 17-22, San Francisco CA.* 2003:Nr639
- Michelson D, Buitelaar JK, Danckaerts M, et al. Relapse prevention in pediatric patients with ADHD treated with atomoxetine: a randomized, double-blind, placebo-controlled study. *J Am Acad Child Adolesc Psychiatry.* 2004;43(7):896-904.
- Analyses in Spanish subsample in: Escobar R, Soutullo C, San Sebastian J, Fernandez E, Julian I, Lahortiga F. Atomoxetine safety and efficacy in children with attention deficit/hyperactivity disorder (ADHD): Initial phase of 10-week treatment in a relapse prevention study with a Spanish sample. [Spanish]. *Actas Esp Psiquiatr.* 2005;33(1):26-32
- Commentary in Zuddas A, Masi G, Millepiedi S, et al. Results of a long-term trial of the use of atomoxetine in the relapse prevention in pediatric patients with ADHD. [Italian]. Risultati di un trial a lungo termine sull'impiego di atomoxetina nella prevenzione delle recidive nell'ADHD. *Psychopathology.* 2005;11(2):251-257.) (link confirmed by Dr Zuddas)
- Hazell P, Zhang S, Wolanczyk T, et al. Comorbid oppositional defiant disorder and the risk of relapse during 9 months of atomoxetine treatment for attention-deficit/hyperactivity disorder. *Eur Child Adolesc Psychiatry.* 2006;15(2):105-110.
- Continuation study: Buitelaar JK, Michelson D, Danckaerts M, Gillberg C, Spencer TJ, Zuddas A, Faries DE, Zhang S, Biederman J. A randomized, double-blind study of continuation treatment for attention-deficit/hyperactivity disorder after 1 year. *Biol Psychiatry*. 2007;1;61(5):694-9. and related:
- Buitelaar J, Michelson D, Danckaerts M, et al. Continued atomoxetine in pediatric patients with attention-deficit/hyperactivity disorder after 1 year of treatment. *Int J Neuropsychopharmacol.*2004;7:S440.
- Additional outcomes, not pertinent for the present meta-analysis, in: Trzepacz PT, Spencer TJ, Zhang S, Bangs ME, Witte MM, Desaiah D. Effect of atomoxetine on Tanner stage sexual development in children and adolescents with attention deficit/hyperactivity disorder: 18-month results from a double-blind, placebo-controlled trial. *Curr Med Res Opin.* 2011;27 (Suppl 2):45-52.
- Thome J, Escobar R, Lipsius S, Upadhyaya H. Predictors of relapse or maintenance of response of Attention-Deficit/Hyperactivity Disorder symptoms after discontinuation of long-term treatment with atomoxetine. ADHD. *Atten Defic Hyperact Disord*. 2015;7:S97.

Reason for exclusion: Subjects responders to open label phase entered the RCT phase

Mikkelsen1981

- Mikkelsen E, Lake CR, Brown GL, Ziegler MG, Ebert MH. The hyperactive child syndrome: peripheral sympathetic nervous system function and the effect of d-amphetamine. *Psychiatry Res.* 1981;4(2):157-169.

Reason for exclusion: Less than seven days treatment

Milich1989

- Milich R, Licht BG, Murphy DA, Pelham WE. Attention-deficit hyperactivity disordered boys' evaluations of and attributions for task performance on medication versus placebo. *J Abnorm Psychol.* 1989;98(3):280-284.

Reason for exclusion: Single dose

Milich1991

- Milich R, Carlson CL, Pelham WE, Jr., Licht BG. Effects of methylphenidate on the persistence of ADHD boys following failure experiences. *J Abnorm Child Psychol.* 1991;19(5):519-536.

Reason for exclusion: Less than seven days treatment

Miller1996

- Miller DC, Kavcic V, Leslie JE. ERP changes induced by methylphenidate in boys with attention-deficit hyperactivity disorder. *J Atten Disord.* 1996;1(2):95-113.

Reason for exclusion: Single dose

Millichap1967a

- Millichap JG, Boldrey EE. Studies in hyperkinetic behavior. II. Laboratory and clinical evaluations of drug treatments. *Neurology.* 1967;17(5):467-471.

Reason for exclusion: No DSM/ICD criteria

Millichap1967b

- Millichap JG, Fowler GW. Treatment of "minimal brain dysfunction" syndromes. Selection of drugs for children with hyperactivity and learning disabilities. *Pediatr Clin North Am.* 1967;14(4):767-777.

Reason for exclusion: No DSM/ICD criteria

Millichap1968a

- Millichap JG. Drugs in management of hyperkinetic and perceptually handicapped children. *JAMA.* 1968;206(7):1527-1530.

Reason for exclusion: Review

Millichap1968b

- Millichap JG, Aymat F, Sturgis LH, Larsen KW, Egan RA. Hyperkinetic behavior and learning disorders. 3. Battery of neuropsychological tests in controlled trial of methylphenidate. *Am J Dis Child (1960).* 1968;116(3):235-244.

Reason for exclusion: No DSM/ICD criteria

Millichap1975

- Millichap JG, Millichap M. Letter: Growth of hyperactive children. *N Engl J Med.* 1975;292(24):1300.

Reason for exclusion: Letter, no empirical data

Millichap1978a

- Millichap JG. Growth of hyperactive children treated with methylphenidate. *J Learn Disabil.* 1978;11(9):567-570.

Reason for exclusion: No RCT

Millichap1978b

- Millichap JG. Growth of hyperkinetic children taking methylphenidate, dextroamphetamine, or imipramine/desipramine. *Pediatrics.* 1978;61(1):146-147.

Reason for exclusion: Letter, no empirical data

Mills1996

- Mills IH. Imipramine and amitriptyline in hyperactive children. *Qjm.* 1996;89(4):321-322.

Reason for exclusion: Letter, non empirical data

Miranda2006

- Miranda A, Jarque S, Rosel J. Treatment of children with ADHD: Psychopedagogical program at school versus psychostimulant medication. *Psicothema.* 2006;18(3):335-341.

Reason for exclusion: Study arms: psychoeducation, methylphenidate, control (no intervention) (confirmed by Dr Miranda)

Modi2000

- Modi NB, Lindemulder B, Gupta SK. Single- and multiple-dose pharmacokinetics of an oral once-a-day osmotic controlled-release OROS (methylphenidate HCl) formulation. *J Clin Pharmacol.* 2000;40(4):379-388.

Reason for exclusion: Open label trials

Mohammadi2004a

- Mohammadi MR, Kashani L, Akhondzadeh S, Izadian ES, Ohadinia S. Efficacy of theophylline compared to methylphenidate for the treatment of attention-deficit hyperactivity disorder in children and adolescents: a pilot double-blind randomized trial. *J Clin Pharm Ther*. 2004;29(2):139-144.
- Ginsberg DL. Theophylline treatment of ADHD. *Prim psychiatry.* 2004;11(10):28

Reason for exclusion: Medication of interest vs. medication of no interest for the present meta-analysis. No placebo arm

Mohammadi2004b

- Mohammadi MR, Ghanizadeh A, Alaghband-Rad J, Tehranidoost M, Mesgarpour B, Soori H. Selegiline in comparison with methylphenidate in attention deficit hyperactivity disorder children and adolescents in a double-blind, randomized clinical trial. *J Child Adolesc Psychopharmacol*. 2004;14(3):418-425.

Reason for exclusion: Medication of interest vs medication of no interest for the present meta-analysis. No placebo arm

Mohammadi2010 (NCT01099059)

- Mohammadi MR, Kazemi MR, Zia E, Rezazadeh SA, Tabrizi M, Akhondzadeh S. Amantadine versus methylphenidate in children and adolescents with attention deficit/hyperactivity disorder: a randomized, double-blind trial. *Hum Psychopharmacol.* 2010;25(7-8):560-565.
- https://clinicaltrials.gov/ct2/show/NCT01099059

Reason for exclusion: Medication of interest vs medication of no interest for the present meta-analysis. No placebo arm

Mohammadi2012a (IRCT 201205157462N7)

- Mohammadi MR, Mostafavi SA, Keshavarz SA, et al. Melatonin effects in methylphenidate treated children with attention deficit hyperactivity disorder: a randomized double blind clinical trial. *Iran J Psychiatry.* 2012;7(2):87-92.
- http://www.irct.ir/searchresult.php?id=7462&number=7

Reason for exclusion: No arms of interest for the present meta-analysis

Mohammadi2012b

- Mohammadi MR, Hafezi P, Galeiha A, Hajiaghaee R, Akhondzadeh S. Buspirone versus methylphenidate in the treatment of children with attention- deficit/ hyperactivity disorder: randomized double-blind study. *Acta Med Iran.* 2012;50(11):723-728.

Reason for exclusion: Medication of interest vs medication of no interest for the present meta-analysis. No placebo arm

Mohammadi2015

- Mohammadi MR, Mohammadzadeh S, Akhondzadeh S. Memantine versus Methylphenidate in Children and Adolescents with Attention Deficit Hyperactivity Disorder: A Double-Blind, Randomized Clinical Trial. *Iran J Psychiatry.* 2015;10(2):106-114.

Reason for exclusion: Medication of interest vs medication of non interest for the present meta-analysis; no placebo arm

Moll2000

- Moll GH, Heinrich H, Trott G, Wirth S, Rothenberger A. Deficient intracortical inhibition in drug-naive children with attention-deficit hyperactivity disorder is enhanced by methylphenidate. *Neurosci Lett.* 2000;284(1-2):121-125.

Reason for exclusion: No RCT

Mollica2004

- Mollica CM, Maruff P, Vance A. Development of a statistical approach to classifying treatment response in individual children with ADHD. *Hum Psychopharmacol.* 2004;19(7):445-456.

Reason for exclusion: No RCT

Monastra2002

- Monastra VJ, Monastra DM, George S. The effects of stimulant therapy, EEG biofeedback, and parenting style on the primary symptoms of attention-deficit/hyperactivity disorder. *Appl Psychophysiol Biofeedback.* 2002;27(4):231-249.

Reason for exclusion: No arms of interest for the present meta-analysis (methylphenidate arm but not placebo arm)

Monden2012

- Monden Y, Dan H, Nagashima M, et al. Right prefrontal activation as a neuro-functional biomarker for monitoring acute effects of methylphenidate in ADHD children: An fNIRS study. *Neuroimage Clin.* 2012;1(1):131-140.
- http://www.umin.ac.jp/ctr/index.htm

Reason for exclusion: Less than seven days treatment

Montagu1975

- Montagu JD, Swarbrick L. Effect of amphetamines in hyperkinetic children: stimulant or sedative? A pilot study. *Dev Med Child Neurol.* 1975(3):293-298.

Reason for exclusion: No DSM/ICD criteria

Monuteaux2007

- Monuteaux MC, Biederman J. A Randomized, double-blind, placebo-controlled clinical trial of bupropion for the prevention of smoking in youth sith ADHD. *158th Annual Meeting of the American Psychiatric Association; 2005 May 21-26; Atlanta, GA.* 2005.
- Monuteaux MC, Spencer TJ, Faraone SV, Wilson AM, Biederman J. A randomized, placebo-controlled clinical trial of bupropion for the prevention of smoking in children and adolescents with attention-deficit/hyperactivity disorder. *The J Clin Psychiatry.* 2007;68(7):1094-1101.

Reason for exclusion: Bupropion assessed for smoking cessation; 50% of subjects on bupropion + stimulants; dose of bupropion used for smoking cessation and exceeds those recommended for ADHD, as per our protocol

Mooney2015

- Mooney ME, Herin DV, Specker S, Babb D, Levin FR, Grabowski J. Pilot study of the effects of lisdexamfetamine on cocaine use: A randomized, double-blind, placebo-controlled trial. *Drug Alcohol Depend.* 2015;153:94-103.

Reason for exclusion: No participants with ADHD

Morash-Conway2016

- Gendron M, Rusak B, Rajda M, Corkum PV. Assessing the impact of methylphenidate on sleep in children with adhd using polysomnography and actigraphy. *Sleep*. 2012;35:A374.
- Morash-Conway J, Gendron M, Corkum P. The role of sleep quality and quantity in moderating the effectiveness of medication in the treatment of children with ADHD. *Atten Defic Hyperact Disord.* 2017;9(1):31-38

Reason for exclusion: No usable outcomes/outcomes for interest

Moreno-Garcia2015

- Moreno-Garcia I, Delgado-Pardo G, de Rey CCV, Meneres-Sancho S, Servera-Barcelo M. Neurofeedback, pharmacological treatment and behavioral therapy in hyperactivity: Multilevel analysis of treatment effects on electroencephalography. *Int J Clin Health Psychol.* 2015;15(3):217-225.

Reason for exclusion: Study arms of no interest for the present meta-analysis

Moshe2012

- Moshe K, Karni A, Tirosh E. Anxiety and methylphenidate in attention deficit hyperactivity disorder: a double-blind placebo-drug trial. *Atten Defic Hyperact Disord*. 2012;4(3):153-158.

Reason for exclusion: Cross-over without wash out; pre-cross over data not available

Mostafavi2012

- Mostafavi SA, Mohammadi MR, Hosseinzadeh P, et al. Dietary intake, growth and development of children with ADHD in a randomized clinical trial of Ritalin and Melatonin co-administration: Through circadian cycle modification or appetite enhancement? *Iran J Psychiatry.* 2012;7(3):114-119.

Reason for exclusion: No arms as per protocol

Mott2004

- Mott TF, Leach L. Is methylphenidate useful for treating adolescents with ADHD? *J Fam Pract.* 2004;53(8):659-661.

Reason for exclusion: Commentary

Moura2007

- Moura MAd. Treatment of comorbid attention deficit hyperactivity disorder and depression in pediatric patient. *Rev Bras Psiquiatr.* 2007;29(2):189-190.

Reason for exclusion: Case report

Muir2010
- Muir VJ, Perry CM. Guanfacine extended-release: in attention deficit hyperactivity disorder. *Drugs.*10 2010;70(13):1693-1702.

Reason for exclusion: Review

Mulder2016
- Mulder R, Hazell P, Rucklidge JJ, Malhi GS. Methylphenidate for attention-deficit/hyperactivity disorder: Too much of a good thing? *Aust N Z J Psychiatry.* 2016;50(2):113-114.

Reason for exclusion: Commentary, no empirical data

Mulhern2004
- Mulhern RK, Khan RB, Kaplan S, et al. Short-term efficacy of methylphenidate: a randomized, double-blind, placebo-controlled trial among survivors of childhood cancer. *J Clin Oncol.* 2004;22(23):4795-4803.

Reason for exclusion: No DSM/ICD criteria

Muller1971
- Muller P. On the effect of methylphenidate in children with the hyperkinetic syndrome. *Prax Kinderpsychol Kinderpsychiatr.* 1971;20(2):71-74.

Reason for exclusion: No DSM/ICD criteria

Muniz2008 (NCT00141050; CRIT124EUS12)
- Muniz R, Brams M, Mao A, McCague K, Pestreich L, Silva R. Efficacy and safety of extended-release dexmethylphenidate compared with d,l-methylphenidate and placebo in the treatment of children with attention-deficit/hyperactivity disorder: a 12-hour laboratory classroom study. *J Child Adolesc Psychopharmacol.* 2008;18(3):248-256.
- https://clinicaltrials.gov/ct2/show/NCT00141050

Reason for exclusion: "Stabilized" participants at baseline; Dr Silva confirmed "stabilized" = "responders"

Murphy1992
- Murphy DA, Pelham WE, Lang AR. Aggression in boys with attention deficit-hyperactivity disorder: methylphenidate effects on naturalistically observed aggression, response to provocation, and social information processing. *J Abnorm Child Psychol.* 1992;20(5):451-466.

Reason for exclusion: Co-intervention; no relevant outcomes of interest

Murray1987
- Murray JB. Psychophysiological effects of methylphenidate (Ritalin). *Psychol Rep.* 1987;61(1):315-336.

Reason for exclusion: Review

Murray2000
- Murray LK, Kollins SH. Effects of methylphenidate on sensitivity to reinforcement in children diagnosed with attention deficit hyperactivity disorder: an application of the matching law. *J Appl Behav Anal.* 2000;33(4):573-591.

Reason for exclusion: No arms of interest for the present meta-analysis

Murray2011 (NCT00799487; EUCTR2015-001042-28; CR015118)
- Murray DW, Childress A, Giblin J, Williamson D, Armstrong R, Starr HL. Effects of OROS methylphenidate on academic, behavioral, and cognitive tasks in children 9 to 12 years of age with attention-deficit/hyperactivity disorder. *Clin Pediatr (Phila).* 2011;50(4):308-320.
- Pooled in: Starr HL, Armstrong R, Damaraju CV, Ascher S. Effects of OROS methylphenidate (MPH) treatment on behavior and performance in children with ADHD with and without comorbid learning disability. *Eur Child Adolesc Psychiatry.* 2011;20:S126.
- Pooled in: Armstrong RB, Damaraju CV, Ascher S, Schwarzman L, O'Neill J, Starr HL. Time course of treatment effect of OROS methylphenidate in children with ADHD. *J Atten Disord.* 2012;16(8):697-705.
- Pooled in: Williamson D, Murray DW, Damaraju CV, Ascher S, Starr HL. Methylphenidate in children with ADHD with or without learning disability. *J Atten Disord.* 2014;18(2):95-104.
- https://clinicaltrials.gov/ct2/show/NCT00799487
- https://www.clinicaltrialsregister.eu/ctr-search/search?query=eudract_number:2015-001042-28

Reason for exclusion: Less than seven days treatment; Participants: responders to previous ADHD medication

Musten1997

- Musten LM. Efficacy of stimulant medication treatment of attention deficit hyperactivity disorder in preschool-aged children[Ph.D.]. *Ann Arbor*. University of Ottawa (Canada); 1996.
- Musten LM, Firestone P, Pisterman S, Bennett S, Mercer J. Effects of methylphenidate on preschool children with ADHD: cognitive and behavioral functions. *J Am Acad Child Adolesc Psychiatry.* 1997;36(10):1407-1415.
- Musten LM. Efficacy of stimulant medication treatment of attention deficit hyperactivity disorder in preschool-aged children. *Dissertation Abstracts International: Section B: The Sciences and Engineering.* 1998;59(3-B):1374.
- Firestone P, Musten LM, Pisterman S, Mercer J, Bennett S. Short-term side effects of stimulant medication are increased in preschool children with attention-deficit/hyperactivity disorder: a double-blind placebo-controlled study. *J Child Adolesc Psychopharmacol.* 1998;8(1):13-25.

Reason for exclusion: Preschoolers (aged 4-6)

Myronuk1996
- Myronuk LD, Weiss M, Cotter L. Combined treatment with moclobemide and methylphenidate for comorbid major depression and adult attention-deficit/hyperactivity disorder. *J Clin Psychopharmacol.* 1996;16(6):468-469.

Reason for exclusion: Case reports

Na2013 (NCT01060150)
- Na K-S, Lee SI, Hong SD, et al. Effect of osmotic-release oral system methylphenidate on learning skills in adolescents with attention-deficit/hyperactivity disorder: an open-label study. *Int Clin Psychopharmacol.* 2013;28(4):184-192.
- https://clinicaltrials.gov/ct2/show/NCT01060150

Reason for exclusion: Non randomized, no double blind

Nagashima2014a
- Nagashima M, Monden Y, Dan I, et al. Neuropharmacological effect of atomoxetine on attention network in children with attention deficit hyperactivity disorder during oddball paradigms as assessed using functional near-infrared spectroscopy. *Neurophotonics.* 2014;1(2):025007.
- Nagashima M, Monden Y, Dan I, et al. Acute neuropharmacological effects of atomoxetine on inhibitory control in ADHD children: a fNIRS study. *Neuroimage Clin.* 2014;6:192-201.

Reason for exclusion: Single dose

Nagashima2014b
- Nagashima M, Monden Y, Dan I, et al. Neuropharmacological effect of methylphenidate on attention network in children with attention deficit hyperactivity disorder during oddball paradigms as assessed using functional near-infrared spectroscopy. *Neurophotonics.* 2014;1(1):015001.

Reason for exclusion: Single dose

Nagel-Hiemke1984
- Nagel-Hiemke M, Berg B, Reinhardt D, Karch D, Pothmann R. The influence of methylphenidate on the sympathoadrenal reactivity in children diagnosed as hyperactive. *Klin Padiatr.* 1984;196(2):78-82.

Reason for exclusion: No DSM/ICD criteria

Nair2009
- Nair V, Mahadevan S. Randomised controlled study-efficacy of clonidine versus carbamazepine in children with ADHD. *J Trop Pediatr.* 2009;55(2):116-121.

Reason for exclusion: Medication of interest vs. medication of no interest for the present meta-analysis

Nass2002
- Nass R, Bressman S. Attention deficit hyperactivity disorder and Tourette syndrome: What's the best treatment? *Neurology.* 2002;58(4):513-514.

Reason for exclusion: Editorial

NCT00585910
- https://clinicaltrials.gov/ct2/show/NCT00585910

Reason for exclusion: Open label

NCT01133847
- https://clinicaltrials.gov/ct2/show/NCT01133847

Reason for exclusion: Single blind

NCT00320528 (*B4Z-IT-LYDS*, EUCTR2005-005701-32-IT)

- https://clinicaltrials.gov/ct2/show/NCT00320528

Reason for exclusion: Open label

NCT02951754

- https://clinicaltrials.gov/ct2/show/NCT02951754

Reason for exclusion: Open label

NCT02999503

- https://clinicaltrials.gov/ct2/show/NCT02999503

Reason for exclusion: Open label

NCT03062839

- https://clinicaltrials.gov/ct2/show/NCT03062839

Reason for exclusion: Medication of no interest for the present meta-analysis (melatonin) vs placebo

NCT00029614

- https://clinicaltrials.gov/ct2/show/NCT00029614

Reason for exclusion: No randomized, open label

NCT00181766

- https://clinicaltrials.gov/ct2/show/NCT00181766

Reason for exclusion: No RCT, no double blind

NCT00181948

- https://clinicaltrials.gov/ct2/show/NCT00181948

Reason for exclusion: No RCT; No double blind; Participants: resistant to stimulants

NCT00191386

- https://clinicaltrials.gov/ct2/show/NCT00191386

Reason for exclusion: No RCT; no double blind

NCT00191659 (B4Z-BP-LYBS)

- https://clinicaltrials.gov/ct2/show/NCT00191659

Reason for exclusion: Open label

NCT00218543

- https://clinicaltrials.gov/ct2/show/NCT00218543

Reason for exclusion: Open label

NCT03088267

- https://clinicaltrials.gov/ct2/show/NCT03088267

Reason for exclusion: Estimated completion date: April 10, 2017

NCT00418262

- https://clinicaltrials.gov/ct2/show/NCT00418262

Reason for exclusion: No RCT;open label

NCT00447278 (B4Z-EW-LYDY)

- https://clinicaltrials.gov/ct2/show/NCT00447278

Reason for exclusion: Open label

NCT00471354 (B4Z-CR-S018)

- https://clinicaltrials.gov/ct2/show/NCT00471354

Reason for exclusion: Open label

NCT00356070

- https://clinicaltrials.gov/ct2/show/NCT00356070

Reason for exclusion: No RCT; open label

NCT00356226

- https://clinicaltrials.gov/ct2/show/NCT00356226

Reason for exclusion: No participants with ADHD

NCT00131573

- https://clinicaltrials.gov/ct2/show/NCT00131573

Reason for exclusion: No participants with ADHD

NCT00181831

- https://clinicaltrials.gov/ct2/show/NCT00181831

Reason for exclusion: No RCT

NCT00200031

- https://clinicaltrials.gov/ct2/show/NCT00200031

Reason for exclusion: No participants with ADHD

NCT00282490

- https://clinicaltrials.gov/ct2/show/NCT00282490

Reason for exclusion: No participants with ADHD

NCT00448175

- https://clinicaltrials.gov/ct2/show/NCT00448175

Reason for exclusion: No participants with ADHD

NCT00534521

- https://clinicaltrials.gov/ct2/show/NCT00534521

Reason for exclusion: No participants with ADHD

NCT00547378

- https://clinicaltrials.gov/ct2/show/NCT00547378

Reason for exclusion: No participants with ADHD

NCT00583219

- https://clinicaltrials.gov/ct2/show/NCT00583219

Reason for exclusion: No participants with ADHD

NCT00600470

- https://clinicaltrials.gov/ct2/show/NCT00600470

Reason for exclusion: Behavioural treatment, single blind

NCT00631280

- https://clinicaltrials.gov/ct2/show/NCT00631280

Reason for exclusion: Behavioural treatment

NCT00706407

- https://clinicaltrials.gov/ct2/show/NCT00706407

Reason for exclusion: No participants with ADHD

NCT00805779

- https://clinicaltrials.gov/ct2/show/NCT00805779

Reason for exclusion: No ADHD

NCT00825708

- https://clinicaltrials.gov/ct2/show/NCT00825708

Reason for exclusion: No pharmacological treatment

NCT00871975

- https://clinicaltrials.gov/ct2/show/NCT00871975

Reason for exclusion: No participants with ADHD

NCT00886483

- https://clinicaltrials.gov/ct2/show/NCT00886483

Reason for exclusion: No pharmacological treatment

NCT00928395

- https://clinicaltrials.gov/ct2/show/NCT00928395

Reason for exclusion: No participants with ADHD

NCT00943904

- https://clinicaltrials.gov/ct2/show/NCT00943904

Reason for exclusion: No participants with ADHD

NCT01023269

- https://clinicaltrials.gov/ct2/show/NCT01023269

Reason for exclusion: No participants with ADHD

NCT01052064

- https://clinicaltrials.gov/ct2/show/NCT01052064

Reason for exclusion: No participants with ADHD

NCT01125722

- https://clinicaltrials.gov/ct2/show/NCT01125722

Reason for exclusion: No participants with ADHD

NCT01194999

- https://clinicaltrials.gov/ct2/show/NCT01194999

Reason for exclusion: No ADHD

NCT01196910

- https://clinicaltrials.gov/ct2/show/NCT01196910

Reason for exclusion: No pharmacological treatment

NCT01214265

- https://clinicaltrials.gov/ct2/show/NCT01214265

Reason for exclusion: No participants with ADHD

NCT01322646

- https://clinicaltrials.gov/ct2/show/NCT01322646

Reason for exclusion: No pharmacological treatment

NCT01369485

- https://clinicaltrials.gov/ct2/show/NCT01369485

Reason for exclusion: No participants with ADHD

NCT01388530

- https://clinicaltrials.gov/ct2/show/NCT01388530

Reason for exclusion: Open label, no pharmacological treatment

NCT01557595

- https://clinicaltrials.gov/ct2/show/NCT01557595

Reason for exclusion: Single group assignment, non-pharmacological

NCT01569061

- https://clinicaltrials.gov/ct2/show/NCT01569061

Reason for exclusion: No participants with ADHD

NCT01574976

- https://clinicaltrials.gov/ct2/show/NCT01574976

Reason for exclusion: Single blind, no pharmacological

NCT01618110

- https://clinicaltrials.gov/ct2/show/NCT01618110

Reason for exclusion: No pharmacological treatment

NCT01711372

- https://clinicaltrials.gov/ct2/show/NCT01711372

Reason for exclusion: No RCT

NCT01723319

- https://clinicaltrials.gov/ct2/show/NCT01723319

Reason for exclusion: No pharmacological treatment

NCT01749800

- https://clinicaltrials.gov/ct2/show/NCT01749800

Reason for exclusion: No participants with ADHD

NCT01781117

- https://clinicaltrials.gov/ct2/show/NCT01781117

Reason for exclusion: No ADHD

NCT00485550 (C1538/3044/AD/US)

- https://clinicaltrials.gov/ct2/show/NCT00485550

Reason for exclusion: Participants: stimulant non responders

NCT00485628 (5286, B4Z-JE-LYBD)

- https://clinicaltrials.gov/ct2/show/NCT00485628

Reason for exclusion: Open label

NCT00485849 (6639, B4Z-UT-S003)

- https://clinicaltrials.gov/ct2/show/NCT00485849

Reason for exclusion: No randomized

NCT00485875 (7953, B4Z-MC-LYCI)

- https://clinicaltrials.gov/ct2/show/NCT00485875

Reason for exclusion: Open label

NCT00530335

- https://clinicaltrials.gov/ct2/show/NCT00530335

Reason for exclusion: Open label (related to Takahashi M, Takita Y, Goto T, et al. An open-label, dose-titration tolerability study of atomoxetine hydrochloride in Japanese adults with attention-deficit/hyperactivity disorder. Psychiatry & Clinical Neurosciences. 2011; 65(1):55-63., discarded based on the title)

NCT00540826

- https://clinicaltrials.gov/ct2/show/NCT00540826

Reason for exclusion: Observational study (Dittmann RW, Banaschewski T, Schacht A, Wehmeier PM. Findings from the observational COMPLY study in children and adolescents with ADHD: core symptoms, ADHD-related difficulties, and patients' emotional expression during psychostimulant or non-stimulant ADHD treatment. Atten Defic Hyperact Disord. 2014 Dec; 6(4):291-302. doi: 10.1007/s12402-014-0136-z. Epub 2014 Apr 6, not found in our original search across databases)

NCT00568685 (11710, B4Z-KL-LYEC)

- https://clinicaltrials.gov/ct2/show/NCT00568685

Reason for exclusion: Open label

NCT00634439

- https://clinicaltrials.gov/ct2/show/NCT00634439

Reason for exclusion: Observational

NCT00636818

- https://clinicaltrials.gov/ct2/show/NCT00636818

Reason for exclusion: Open label

NCT00687609 (12382, B4Z-UT-LYEL)

- https://clinicaltrials.gov/ct2/show/NCT00687609

Reason for exclusion: Open label

NCT00760747 (12305, B4Z-EW-LYFJ)

- https://clinicaltrials.gov/ct2/show/NCT00760747

Reason for exclusion: Open label

NCT00856063

- https://clinicaltrials.gov/ct2/show/NCT00856063

Reason for exclusion: Pre-schoolers (max 70 months)

NCT00953862

- https://clinicaltrials.gov/ct2/show/NCT00953862

Reason for exclusion: No RCT, Open label

NCT00969618 (12397, B4Z-JE-LYEK)

- https://clinicaltrials.gov/ct2/show/NCT00969618

Reason for exclusion: Open label

NCT01057329

- https://clinicaltrials.gov/ct2/show/NCT01057329

Reason for exclusion: Observational

NCT01130467

- https://clinicaltrials.gov/ct2/show/NCT01130467

Reason for exclusion: Observational

NCT01177943

- https://clinicaltrials.gov/ct2/show/NCT01177943

Reason for exclusion: No participants with ADHD

NCT00566371

- https://clinicaltrials.gov/ct2/show/NCT00566371

Reason for exclusion: Dr Owens: data not available due to issues with recruitment

NCT00252278

- https://clinicaltrials.gov/ct2/show/NCT00252278

Reason for exclusion: Dr Owens: data not available due to issues with recruitment

NCT01207622

- https://clinicaltrials.gov/ct2/show/NCT01207622

Reason for exclusion: Withdrawn

NCT01624649

- https://clinicaltrials.gov/ct2/show/NCT01624649

Reason for exclusion: Observational

NCT01802515

- https://clinicaltrials.gov/ct2/show/NCT01802515

Reason for exclusion: No participants with ADHD

NCT00223717

- https://clinicaltrials.gov/ct2/show/NCT00223717

Reason for exclusion: No participants with ADHD

NCT00225251

- https://clinicaltrials.gov/ct2/show/NCT00225251

Reason for exclusion: No participants with ADHD

NCT00252174

- https://clinicaltrials.gov/ct2/show/NCT00252174

Reason for exclusion: No participants with ADHD

NCT00461292

- https://clinicaltrials.gov/ct2/show/NCT00461292

Reason for exclusion: No participants with ADHD

NCT00709371

- https://clinicaltrials.gov/ct2/show/NCT00709371

Reason for exclusion: No participants with ADHD

NCT00321477

- https://clinicaltrials.gov/ct2/show/NCT00321477

Reason for exclusion: No participants with ADHD

NCT00979472

- https://clinicaltrials.gov/ct2/show/NCT00979472

Reason for exclusion: No participants with ADHD

NCT00985387

- https://clinicaltrials.gov/ct2/show/NCT00985387

Reason for exclusion: No participants with ADHD

NCT01012024 (obsolete identifier, gov identifier: NCT01270555) (1999-P-009198)

- https://clinicaltrials.gov/ct2/show/NCT01270555

Reason for exclusion: Open label

NCT01500694 (SPD503-318; EUCTR2011-004668-31-GB)

- https://www.clinicaltrialsregister.eu/ctr-search/search?query=eudract_number:2011-004668-31
- https://clinicaltrials.gov/ct2/show/NCT01500694

Reason for exclusion: Open label (note: Some subjects from SPD503-315, others from SPD503-316)

NCT01985581 (RES 13-001)

- https://clinicaltrials.gov/ct2/show/NCT01985581

Reason for exclusion: Combination guanfacine + methylphenidate only and no Placebo only arm

NCT01146002

- https://clinicaltrials.gov/ct2/show/NCT01146002

Reason for exclusion: Open label

NCT01177306

- https://clinicaltrials.gov/ct2/show/NCT01177306

Reason for exclusion: Open label

NCT00573534

- https://clinicaltrials.gov/ct2/show/NCT00573534

Reason for exclusion: No double blind

NCT00736255 (SPD489-607)

- https://clinicaltrials.gov/ct2/show/NCT00736255

Reason for exclusion: Combined treatment

NCT00746733

- https://clinicaltrials.gov/ct2/show/NCT00746733

Reason for exclusion: No participants with ADHD;No pertinent design (Note: erroneously reported in Pubmed as Wigal T, Brams M, Gasior M, Gao J, Giblin J. Effect size of lisdexamfetamine dimesylate in adults with attention-deficit/hyperactivity disorder. Postgrad Med. Mar 2011; 123(2):169-176.)

NCT00753012

- https://clinicaltrials.gov/ct2/show/NCT00753012

Reason for exclusion: No double blind, no controlled

NCT00922272

- https://clinicaltrials.gov/ct2/show/NCT00922272

Reason for exclusion: No participants with ADHD

NCT01000064

- https://clinicaltrials.gov/ct2/show/NCT01000064

Reason for exclusion: No formal DSM/ICD criteria for ADHD

NCT01017263

- https://clinicaltrials.gov/ct2/show/NCT01017263

Reason for exclusion: No double blind and no controlled

NCT01263548

- https://clinicaltrials.gov/ct2/show/NCT01263548

Reason for exclusion: No RCT, no double blind

NCT01328756 (SPD489-404; obsolete identifier: NCT01413165)

- https://clinicaltrials.gov/ct2/show/NCT01328756

Reason for exclusion: No RCT, no double blind
Note: some subjects participated in another SPD489 study (SPD489-317, SPD489-325, or SPD489 326)

NCT01435759

- https://clinicaltrials.gov/ct2/show/NCT01435759

Reason for exclusion: No participants with ADHD
NCT01730079

- https://www.clinicaltrials.gov/ct2/show/NCT01730079

Reason for exclusion: No RCT, no treatment of interest for the present meta-analysis

NCT01816074

- https://clinicaltrials.gov/ct2/show/NCT01816074

Reason for exclusion: No treatment of interest for the present meta-analysis

NCT00326300

- https://clinicaltrials.gov/ct2/show/NCT00326300

Reason for exclusion: Open-label; Related to Adler LA, Orman C, Starr HL, et al. Long-term safety of OROS methylphenidate in adults with attention-deficit/hyperactivity disorder: an open-label, dose-titration, 1-year study. J Clin Psychopharmacol. Feb 2011; 31(1):108-114. (Excluded just based on the title)

NCT00337285 (NRP104.304)

- https://clinicaltrials.gov/ct2/show/NCT00337285

Reason for exclusion: No RCT; related to Mattingly G, Weisler R, Dirks B, Babcock T, Adeyi B, Scheckner B, Lasser R. Attention deficit hyperactivity disorder subtypes and symptom response in adults treated with lisdexamfetamine dimesylate. Innov Clin Neurosci. 2012 May; 9(5-6):22-30. (discarded based on the title) and Ginsberg L, Katic A, Adeyi B, Dirks B, Babcock T, Lasser R, Scheckner B, Adler LA. Long-term treatment outcomes with lisdexamfetamine dimesylate for adults with attention-deficit/hyperactivity disorder stratified by baseline severity. Curr Med Res Opin. 2011 Jun; 27(6):1097-107. doi:10.1185/03007995.2011.567256. Epub 2011 Mar 28 (not found in the original search across databases)

NCT00396669

- https://clinicaltrials.gov/ct2/show/NCT00396669

Reason for exclusion: No participants with ADHD

NCT00501293
https://clinicaltrials.gov/ct2/show/NCT00501293
Reason for exclusion: Open label

NCT00860925
- https://clinicaltrials.gov/ct2/show/NCT00860925

Reason for exclusion: No participants with ADHD

NCT00861939
- https://clinicaltrials.gov/ct2/show/NCT00861939

Reason for exclusion: No participants with ADHD

NCT01866059
- https://clinicaltrials.gov/ct2/show/NCT01866059

Reason for exclusion: No participants with ADHD

NCT01892813
- https://clinicaltrials.gov/ct2/show/NCT01892813

Reason for exclusion: No participants with ADHD

NCT01046214
- https://clinicaltrials.gov/ct2/show/NCT01046214

Reason for exclusion: No participants with ADHD

NCT01924429
- https://clinicaltrials.gov/ct2/show/NCT01924429

Reason for exclusion: Single blind

NCT00712699
- https://clinicaltrials.gov/ct2/show/NCT00712699

Reason for exclusion: Pre-schoolers (up to 5.5 y)
NCT00776555
- https://clinicaltrials.gov/ct2/show/results/NCT00776555

Reason for exclusion: No participants with ADHD

NCT00152035
- https://clinicaltrials.gov/ct2/show/NCT00152035

Reason for exclusion: No double blind

NCT00329511
- https://clinicaltrials.gov/ct2/show/NCT00329511

Reason for exclusion: No participants with ADHD

NCT00330434
- https://clinicaltrials.gov/ct2/show/NCT00330434

Reason for exclusion: No RCT

NCT00350532
- https://www.clinicaltrials.gov/ct2/show/NCT00350532

Reason for exclusion: No participants with ADHD

NCT00332319
- https://clinicaltrials.gov/ct2/show/NCT00332319

Reason for exclusion: No participants with ADHD

NCT00332644
- https://clinicaltrials.gov/ct2/show/NCT00332644

Reason for exclusion: No participants with ADHD

NCT00343811 (C1538/3048/AD/US)
- https://clinicaltrials.gov/ct2/show/NCT00343811

Reason for exclusion: Participants: responders to modafinil

NCT00776737
- https://clinicaltrials.gov/ct2/show/NCT00776737

Reason for exclusion: No RCT

NCT00879320
- https://clinicaltrials.gov/ct2/show/NCT00879320

Reason for exclusion: No RCT-observational

NCT00883155
- https://clinicaltrials.gov/ct2/show/NCT00883155

Reason for exclusion: No participants with ADHD

NCT00917748
- https://clinicaltrials.gov/ct2/show/NCT00917748

Reason for exclusion: No participants with ADHD

NCT01092780
- https://clinicaltrials.gov/ct2/show/NCT01092780

Reason for exclusion: No participants with ADHD

NCT01148342
- https://clinicaltrials.gov/ct2/show/NCT01148342

Reason for exclusion: No treatment of interest for the present meta-analysis; No participants with ADHD

NCT01165255
- https://clinicaltrials.gov/ct2/show/NCT01165255

Reason for exclusion: No participants with ADHD

NCT01290276
- https://clinicaltrials.gov/ct2/show/NCT01290276

Reason for exclusion: No participants with ADHD; No double blind

NCT01291173
- https://clinicaltrials.gov/ct2/show/NCT01291173

Reason for exclusion: No participants with ADHD

NCT01339286
- https://clinicaltrials.gov/ct2/show/NCT01339286

Reason for exclusion: No double blind RCT

NCT01350986
- https://clinicaltrials.gov/ct2/show/NCT01350986

Reason for exclusion: No treatment of interest for the present meta-analysis

NCT01369459
- https://clinicaltrials.gov/ct2/show/NCT01369459

Reason for exclusion: No double blind and not appropriate arms for the present meta-analysis (combined CBT + Methylphenidate)

NCT01385748
- https://clinicaltrials.gov/ct2/show/NCT01385748

Reason for exclusion: No participants with ADHD

NCT01421342
- https://clinicaltrials.gov/ct2/show/NCT01421342

Reason for exclusion: No participants with ADHD

NCT01439126 (SHN-KAP-401)

- https://clinicaltrials.gov/ct2/show/NCT01439126

Reason for exclusion: Randomized withdrawal design

NCT01458340

- https://clinicaltrials.gov/ct2/show/NCT01458340

Reason for exclusion: Drug of no interest for the present meta-analysis vs. placebo

NCT01483521

- https://clinicaltrials.gov/ct2/show/NCT01483521

Reason for exclusion: No treatment of interest for the present meta-analysis and no double blind

NCT01918436

- https://clinicaltrials.gov/ct2/show/NCT01918436

Reason for exclusion: No treatment of interest for the present meta-analysis

NCT01919073

- https://clinicaltrials.gov/ct2/show/NCT01919073

Reason for exclusion: No double blind and no treatment of interest for the present meta-analysis

NCT00417794

- https://clinicaltrials.gov/ct2/show/NCT00417794

Reason for exclusion: Part of participants aged < 5

NCT00012584

- https://clinicaltrials.gov/ct2/show/NCT00012584

Reason for exclusion: Open label

NCT00409708

- https://clinicaltrials.gov/ct2/show/NCT00409708

Reason for exclusion: Open label; combination behavioural therapy + medication

NCT00414921

- https://clinicaltrials.gov/ct2/show/NCT00414921

Reason for exclusion: Aged 4-6 years old

NCT00418691

- https://clinicaltrials.gov/ct2/show/NCT00418691

Reason for exclusion: No participants with ADHD; Open label

NCT00517504

- https://clinicaltrials.gov/ct2/show/NCT00517504

Reason for exclusion: Aged 36-84 months; single blind titration phase

NCT00664703 (NIMH number 5R43MH081553-02, R43MH081553)

- https://clinicaltrials.gov/ct2/show/NCT00664703

Reason for exclusion: Less than seven days treatment (note: 1 arm is on a drug of no interest for the present meta-analysis; 2 arms are on placebo and methylphenidate, respectively); related to Martin CA, Nuzzo PA, Ranseen JD, Kleven MS, Guenthner G, Williams Y, Walsh SL, Dwoskin LP., Lobeline Effects on Cognitive Performance in Adult ADHD. J Atten Disord. 2013 Aug 21. (Not retrieved in our original search across databases)

NCT00754208

- https://clinicaltrials.gov/ct2/show/NCT00754208

Reason for exclusion: No double blind, no controlled

NCT00773916

- https://clinicaltrials.gov/ct2/show/NCT00773916

Reason for exclusion: No double blind, no controlled

NCT00794040

- https://clinicaltrials.gov/ct2/show/NCT00794040

Reason for exclusion: Citalopram plus methylphenidate vs. placebo plus methylphenidate

NCT00972985

- https://clinicaltrials.gov/ct2/show/NCT00972985

Reason for exclusion: No participants with ADHD; No double blind

NCT00419731

- https://clinicaltrials.gov/ct2/show/NCT00419731

Reason for exclusion: No participants with ADHD

NCT00428480

- https://clinicaltrials.gov/ct2/show/NCT00428480

Reason for exclusion: No participants with ADHD

NCT01040702 (SH-40107)

- https://clinicaltrials.gov/ct2/show/NCT01040702

Reason for exclusion: Single dose

NCT01228604

- https://clinicaltrials.gov/ct2/show/NCT01228604

Reason for exclusion: No RCT

NCT01244269

- https://clinicaltrials.gov/ct2/show/NCT01244269

Reason for exclusion: No participants with ADHD

NCT01377662

- https://clinicaltrials.gov/ct2/show/NCT01377662

Reason for exclusion: No participants with ADHD

NCT01554046 (SHEBA-12-8292-DG-CTIL)

- https://clinicaltrials.gov/ct2/show/NCT01554046

Reason for exclusion: Open label

NCT01599975

- https://clinicaltrials.gov/ct2/show/NCT01599975

Reason for exclusion: No participants with ADHD

NCT01651169

- https://clinicaltrials.gov/ct2/show/NCT01651169

Reason for exclusion: No double blind, single dose

NCT01740206

- https://clinicaltrials.gov/ct2/show/NCT01740206

Reason for exclusion: Open label

NCT01764672

- https://www.clinicaltrials.gov/ct2/show/NCT01764672

Reason for exclusion: Withdrawn

NCT01821170

- https://clinicaltrials.gov/ct2/show/NCT01821170

Reason for exclusion: Open label, medication vs cognitive therapy

NCT01834547

- https://clinicaltrials.gov/ct2/show/NCT01834547

Reason for exclusion: No participants with ADHD

NCT01978431

- https://clinicaltrials.gov/ct2/show/NCT01978431

Reason for exclusion: No participants with ADHD

NCT01993108

- https://clinicaltrials.gov/ct2/show/NCT01993108

Reason for exclusion: Single dose (end of study foreseen in May 2017)

NCT00000304

- https://clinicaltrials.gov/ct2/show/NCT00000304

Reason for exclusion: No participants with ADHD

NCT00000308

- https://clinicaltrials.gov/ct2/show/NCT00000308

Reason for exclusion: No participants with ADHD

NCT00001206

- https://clinicaltrials.gov/ct2/show/NCT00001206

Reason for exclusion: No RCT (no treatment)

NCT00001666

- https://clinicaltrials.gov/ct2/show/NCT00001666

Reason for exclusion: No participants with ADHD

NCT00402857

- https://clinicaltrials.gov/ct2/show/NCT00402857

Reason for exclusion: No treatment of interest for the present meta-analysis

NCT00672347

- https://clinicaltrials.gov/ct2/show/NCT00672347

Reason for exclusion: No participants with ADHD

NCT00863941

- https://clinicaltrials.gov/ct2/show/NCT00863941

Reason for exclusion: No participants with ADHD; No double blind

NCT00864981

- https://clinicaltrials.gov/ct2/show/NCT00864981

Reason for exclusion: No ADHD and no double blind

NCT00865111

- https://clinicaltrials.gov/ct2/show/NCT00865111

Reason for exclusion: No participants with ADHD; No double blind

NCT00865371

- https://clinicaltrials.gov/ct2/show/NCT00865371

Reason for exclusion: No participants with ADHD; No double blind

NCT00865410

- https://clinicaltrials.gov/ct2/show/NCT00865410

Reason for exclusion: No participants with ADHD; No double blind

NCT00865462

- https://clinicaltrials.gov/ct2/show/NCT00865462

Reason for exclusion: No participants with ADHD; No double blind

NCT01500382

- https://clinicaltrials.gov/ct2/show/NCT01500382

Reason for exclusion: No participants with ADHD

NCT01570426

- https://clinicaltrials.gov/ct2/show/NCT01570426

Reason for exclusion: Observational

NCT01592695

- https://clinicaltrials.gov/ct2/show/NCT01592695

Reason for exclusion: No participants with ADHD

NCT01597661

- https://clinicaltrials.gov/ct2/show/NCT01597661

Reason for exclusion: No participants with ADHD

NCT01600885

- https://clinicaltrials.gov/ct2/show/NCT01600885

Reason for exclusion: No ADHD

NCT01601730

- https://clinicaltrials.gov/ct2/show/NCT01601730

Reason for exclusion: No ADHD

NCT01620112

- https://clinicaltrials.gov/ct2/show/NCT01620112

Reason for exclusion: No participants with ADHD; No treatment of interest for the present meta-analysis

NCT01621009

- https://clinicaltrials.gov/ct2/show/NCT01621009

Reason for exclusion: No participants with ADHD

NCT01621022

- https://clinicaltrials.gov/ct2/show/NCT01621022

Reason for exclusion: No participants with ADHD

NCT01667484

- https://clinicaltrials.gov/ct2/show/NCT01667484

Reason for exclusion: No participants with ADHD

NCT01771874

- https://clinicaltrials.gov/ct2/show/NCT01771874

Reason for exclusion: No participants with ADHD

NCT01793610

- https://clinicaltrials.gov/ct2/show/NCT01793610

Reason for exclusion: No participants with ADHD; No treatment of interest for the present meta-analysis

NCT01800097

- https://clinicaltrials.gov/ct2/show/NCT01800097

Reason for exclusion: No participants with ADHD

NCT01986075

- https://clinicaltrials.gov/ct2/show/NCT01986075

Reason for exclusion: No participants with ADHD

NCT02482649

- https://clinicaltrials.gov/ct2/show/NCT02482649

Reason for exclusion: Open label

NCT02623114

- https://clinicaltrials.gov/ct2/show/NCT02623114

Reason for exclusion: No RCT

NCT02874690

- https://clinicaltrials.gov/ct2/show/NCT02874690

Reason for exclusion: Recruitment planned to end in October 2018

NCT01675804

- https://clinicaltrials.gov/ct2/show/NCT01675804

Reason for exclusion: Open label

NCT01678209

- https://clinicaltrials.gov/ct2/show/NCT01678209

Reason for exclusion: Not completed yet (confirmed by author)

NCT01689740

- https://clinicaltrials.gov/ct2/show/NCT01689740

Reason for exclusion: No participants with ADHD; No treatment of interest for the present meta-analysis

NCT01711021

- https://clinicaltrials.gov/ct2/show/NCT01711021

Reason for exclusion: No formulation of interest for the present meta-analysis (transdermal)

NCT01721330

- https://clinicaltrials.gov/ct2/show/NCT01721330

Reason for exclusion: No treatment of interest for the present meta-analysis (naltrexone) vs placebo

NCT01329510

- https://clinicaltrials.gov/ct2/show/NCT01329510

Reason for exclusion: Open label

NCT01798459

- https://clinicaltrials.gov/ct2/show/NCT01798459

Reason for exclusion: Less than seven days treatment

NCT01962181

- https://clinicaltrials.gov/ct2/show/NCT01962181

Reason for exclusion: Non randomised, single blind

NCT02178995

- https://clinicaltrials.gov/ct2/show/NCT02178995

Reason for exclusion: Non randomised

NCT02225106

- https://clinicaltrials.gov/ct2/show/NCT02225106

Reason for exclusion: Non randomised

NCT02625805

- https://clinicaltrials.gov/ct2/show/NCT02625805

Reason for exclusion: Open label

NCT02630017

- https://clinicaltrials.gov/ct2/show/NCT02630017

Reason for exclusion: Non randomised

NCT02675400

- https://clinicaltrials.gov/ct2/show/NCT02675400

Reason for exclusion: Open label

NCT02695355

- https://clinicaltrials.gov/ct2/show/NCT02695355

Reason for exclusion: Open label

NCT02699528

- https://clinicaltrials.gov/ct2/show/NCT02699528

Reason for exclusion: No RCT

NCT02780102

- https://clinicaltrials.gov/ct2/show/NCT02780102

Reason for exclusion: Single blind

NCT02807870

- https://clinicaltrials.gov/ct2/show/NCT02807870

Reason for exclusion: Not all subjects > 5 years old

NCT00262470

- https://clinicaltrials.gov/ct2/show/NCT00262470

Reason for exclusion: No participants with ADHD

NCT01178138

- https://clinicaltrials.gov/ct2/show/NCT01178138

Reason for exclusion: No participants with ADHD

NCT01805401

- https://clinicaltrials.gov/ct2/show/NCT01805401

Reason for exclusion: No participants with ADHD

NCT01808066

- https://clinicaltrials.gov/ct2/show/NCT01808066

Reason for exclusion: Open label, no pharmacological

NCT01848366

- https://clinicaltrials.gov/ct2/show/NCT01848366

Reason for exclusion: No ADHD

NCT01876524

- https://clinicaltrials.gov/ct2/show/NCT01876524

Reason for exclusion: No pharmacological treatment

NCT01883830

- https://clinicaltrials.gov/ct2/show/NCT01883830

Reason for exclusion: No participants with ADHD

NCT01912885

- https://clinicaltrials.gov/ct2/show/NCT01912885

Reason for exclusion: No participants with ADHD

NCT01913912

- https://clinicaltrials.gov/ct2/show/NCT01913912

Reason for exclusion: Placebo taken only once and study never completed (confirmed by Dr Sonuga-Barke)

NCT01940367

- https://clinicaltrials.gov/ct2/show/NCT01940367

Reason for exclusion: No participants with ADHD

NCT01943539

- https://clinicaltrials.gov/ct2/show/NCT01943539

Reason for exclusion: No pharmacological treatment

NCT01960270

- https://clinicaltrials.gov/ct2/show/NCT01960270

Reason for exclusion: No participants with ADHD

NCT01968512

- https://clinicaltrials.gov/ct2/show/NCT01968512

Reason for exclusion: No pharmacological treatment

NCT01972061

- https://clinicaltrials.gov/ct2/show/NCT01972061

Reason for exclusion: No participants with ADHD

NCT02071186

- https://clinicaltrials.gov/ct2/show/NCT02071186

Reason for exclusion: No pharmacological treatment, single blind

NCT02074228

- https://clinicaltrials.gov/ct2/show/NCT02074228

Reason for exclusion: Open label

NCT02094612

- https://clinicaltrials.gov/ct2/show/NCT02094612

Reason for exclusion: Open label

NCT02096952

- https://clinicaltrials.gov/ct2/show/NCT02096952

Reason for exclusion: Open label

NCT02107820

- https://clinicaltrials.gov/ct2/show/NCT02107820

Reason for exclusion: No participants with ADHD

NCT02110680

- https://clinicaltrials.gov/ct2/show/NCT02110680

Reason for exclusion: No participants with ADHD

NCT02112786

- https://clinicaltrials.gov/ct2/show/NCT02112786

Reason for exclusion: No participants with ADHD

NCT02127931

- https://clinicaltrials.gov/ct2/show/NCT02127931

Reason for exclusion: Open label

NCT02141113

- https://clinicaltrials.gov/ct2/show/NCT02141113

Reason for exclusion: Guanfacine as adjunctive medication to psychostimulants. Related to Butterfield ME, Saal J, Young B, Young JL. Supplementary guanfacine hydrochloride as a treatment of attention deficit hyperactivity disorder in adults: A double blind, placebo-controlled study. Psychiatry Res. 2016; 236:136-141. (Excluded based on the abstract)

NCT02251743

- https://clinicaltrials.gov/ct2/show/NCT02251743

Reason for exclusion: Non pharmacological treatment

NCT00202605 (SPD465-203)

- https://clinicaltrials.gov/ct2/show/NCT00202605

Reason for exclusion: Manufacturer not able to provide data on pertinent outcomes

NCT00218322

- https://clinicaltrials.gov/ct2/show/NCT00218322

Reason for exclusion: Co-treatment with CBT

NCT02083783

- https://clinicaltrials.gov/ct2/show/NCT02083783

Reason for exclusion: Written to author to enquire about study status but no answer

NCT00257725

- https://clinicaltrials.gov/ct2/show/NCT00257725

Reason for exclusion: Not randomised

NCT00261872

- https://clinicaltrials.gov/ct2/show/NCT00261872

Reason for exclusion: Co-treatment (behavioural therapy); refers to COMBINE Study Research Group. Testing combined pharmacotherapies and behavioural interventions for alcohol dependence (the COMBINE study): a pilot feasibility study. Alcohol Clin Exp Res. 2003 Jul; 27(7):1123-31.

NCT00428792 (CRIT124DDE04)

- https://clinicaltrials.gov/ct2/show/NCT00428792

Reason for exclusion: Single blind

NCT00536419

- https://clinicaltrials.gov/ct2/show/NCT00536419

Reason for exclusion: Single dose; 4 days treatment

NCT00852059

- https://clinicaltrials.gov/ct2/show/NCT00852059

Reason for exclusion: Open label

NCT00863499

- https://clinicaltrials.gov/ct2/show/NCT00863499

Reason for exclusion: No RCT

NCT00066170

- https://clinicaltrials.gov/ct2/show/NCT00066170

Reason for exclusion: No participants with ADHD

NCT00086411

- https://clinicaltrials.gov/show/NCT00086411

Reason for exclusion: No participants with ADHD

NCT00129285

- https://clinicaltrials.gov/ct2/show/NCT00129285

Reason for exclusion: No participants with ADHD

NCT00132821

- https://clinicaltrials.gov/ct2/show/NCT00132821

Reason for exclusion: No participants with ADHD

NCT00136760

- https://clinicaltrials.gov/ct2/show/NCT00136760

Reason for exclusion: No participants with ADHD

NCT00296647

- https://clinicaltrials.gov/ct2/show/NCT00296647

Reason for exclusion: No participants with ADHD

NCT01075490

- https://clinicaltrials.gov/ct2/show/NCT01075490

Reason for exclusion: No participants with ADHD

NCT01183234

- https://clinicaltrials.gov/ct2/show/NCT01183234

Reason for exclusion: Open label, comparison of two formulations of methylphenidate

NCT01673594

- https://clinicaltrials.gov/ct2/show/NCT01673594

Reason for exclusion: MPH SODA +naltrexone vs methylphenidate +placebo

NCT01951508

- https://clinicaltrials.gov/ct2/show/NCT01951508

Reason for exclusion: No participants with ADHD

NCT02063945

- https://clinicaltrials.gov/ct2/show/NCT02063945

Reason for exclusion: Open label, no controlled

NCT02700685

- https://clinicaltrials.gov/ct2/show/NCT02700685

Reason for exclusion: Estimated completion date: 2019 (Protocol: Verlaet AA, Ceulemans B, Verhelst H, et al. Effect of Pycnogenol on attention-deficit hyperactivity disorder (ADHD): study protocol for a randomised controlled trial. Trials [Electronic Resource]. 2017; 18:145.

NCT02778360

- https://clinicaltrials.gov/ct2/show/NCT02778360

Reason for exclusion: Open label

NCT00829673

- https://clinicaltrials.gov/ct2/show/NCT00829673

Reason for exclusion: No participants with ADHD; No double blind

NCT00829712

- https://clinicaltrials.gov/ct2/show/NCT00829712

Reason for exclusion: No participants with ADHD; No double blind

NCT00299234

- https://clinicaltrials.gov/ct2/show/NCT00299234

Reason for exclusion: No formal diagnosis of ADHD (ADHD symptoms post chemotherapy for leukaemia)

NCT01205204

- https://clinicaltrials.gov/ct2/show/NCT01205204

Reason for exclusion: Withdrawn

NCT01395160

- https://clinicaltrials.gov/ct2/show/NCT01395160

Reason for exclusion: No RCT

NCT01958593

- https://clinicaltrials.gov/ct2/show/NCT01958593

Reason for exclusion: No participants with ADHD

NCT02712996

- https://clinicaltrials.gov/ct2/show/NCT02712996

Reason for exclusion: Attention problems secondary to traumatic brain injury

NCT02717260

- https://clinicaltrials.gov/ct2/show/NCT02717260

Reason for exclusion: No participants with ADHD; No pharmacological treatment

NCT00181714

- https://clinicaltrials.gov/ct2/show/NCT00181714

Reason for exclusion: Open label

NCT00181740

- https://clinicaltrials.gov/ct2/show/NCT00181740

Reason for exclusion: Open label

NCT00181987

- https://clinicaltrials.gov/ct2/show/NCT00181987

Reason for exclusion: Open label

NCT00302406

- https://clinicaltrials.gov/ct2/show/NCT00302406

Reason for exclusion: Single blind, not controlled

NCT00518232 (CR012508CR012508)

- https://clinicaltrials.gov/ct2/show/NCT00518232

Reason for exclusion: Non randomized

NCT00550147

- https://clinicaltrials.gov/ct2/show/NCT00550147

Reason for exclusion: No double blind, co-treatment

NCT00593112

- https://clinicaltrials.gov/ct2/show/NCT00593112

Reason for exclusion: No RCT

NCT00603434

- https://clinicaltrials.gov/ct2/show/NCT00603434

Reason for exclusion: No participants with ADHD; No controlled

NCT00758160

- https://clinicaltrials.gov/ct2/show/NCT00758160

Reason for exclusion: No double blind, no controlled

NCT00778310

- https://clinicaltrials.gov/ct2/show/NCT00778310

Reason for exclusion: Single dose

NCT00783835 (CR013999, 42603ATT4053)

- https://clinicaltrials.gov/ct2/show/NCT00783835

Reason for exclusion: Open label

NCT00842127

- https://clinicaltrials.gov/ct2/show/NCT00842127

Reason for exclusion: Non randomised

NCT00862108

- https://clinicaltrials.gov/ct2/show/NCT00862108

Reason for exclusion: Open label

NCT00889915

- https://clinicaltrials.gov/ct2/show/NCT00889915

Reason for exclusion: No double blind

NCT00901576

- https://clinicaltrials.gov/ct2/show/NCT00901576

Reason for exclusion: No participants with ADHD; Open label

NCT00931398

- https://clinicaltrials.gov/ct2/show/NCT00931398

Reason for exclusion: Withdrawn

NCT01044238

- https://clinicaltrials.gov/ct2/show/NCT01044238

Reason for exclusion: No participants with ADHD

NCT01063153

- https://clinicaltrials.gov/ct2/show/NCT01063153

Reason for exclusion: Non randomized, open label

NCT01109849

- https://clinicaltrials.gov/ct2/show/NCT01109849

Reason for exclusion: Open label

NCT01348607

- https://clinicaltrials.gov/ct2/show/NCT01348607

Reason for exclusion: No participants with ADHD

NCT01393574

- https://clinicaltrials.gov/ct2/show/NCT01393574

Reason for exclusion: Open label

NCT01853280 (2012-P-000379)

- https://clinicaltrials.gov/ct2/show/NCT01853280

Reason for exclusion: Methylphenidate OROS + L-Methylfolate vs. Methylphenidat OROS+ placebo

NCT01858064

- https://clinicaltrials.gov/ct2/show/NCT01858064

Reason for exclusion: Withdrawn

NCT01863459

- https://clinicaltrials.gov/ct2/show/NCT01863459

Reason for exclusion: Estimated completion date: March 2017

NCT02536105

- https://clinicaltrials.gov/ct2/show/NCT02536105

Reason for exclusion: Ongoing (end of study planned in Sept 2017), optimization phase

NCT00514202

- https://clinicaltrials.gov/ct2/show/NCT00514202

Reason for exclusion: Concomitant CBT

NCT00519428

- https://clinicaltrials.gov/ct2/show/NCT00519428

Reason for exclusion: No participants with ADHD

NCT00641329

- https://clinicaltrials.gov/ct2/show/NCT00641329

Reason for exclusion: Methylphenidate+clonidine vs Placebo+clonidine

NCT00650000

- https://clinicaltrials.gov/ct2/show/NCT00650000

Reason for exclusion: No participants with ADHD

NCT00650286
- https://clinicaltrials.gov/ct2/show/NCT00650286

Reason for exclusion: No participants with ADHD

NCT00661063
- https://clinicaltrials.gov/ct2/show/NCT00661063

Reason for exclusion: No participants with ADHD

NCT00919906
- https://clinicaltrials.gov/ct2/show/NCT00919906

Reason for exclusion: Single blind, no treatment of interest

NCT00935493
- https://clinicaltrials.gov/ct2/show/NCT00935493

Reason for exclusion: No participants with ADHD

NCT00936299
- https://clinicaltrials.gov/ct2/show/NCT00936299

Reason for exclusion: Concurrent cognitive behavioural therapy

NCT00937469
- https://clinicaltrials.gov/ct2/show/NCT00937469

Reason for exclusion: No intervention of interest for the present meta-analysis

NCT02048917
- https://clinicaltrials.gov/ct2/show/NCT02048917

Reason for exclusion: No participants with ADHD

NCT02320201
- https://clinicaltrials.gov/ct2/show/NCT02320201

Reason for exclusion: No participants with ADHD

NCT02323633
- https://clinicaltrials.gov/ct2/show/NCT02323633

Reason for exclusion: No pharmacological treatment, single blind

NCT02344784
- https://clinicaltrials.gov/ct2/show/NCT02344784

Reason for exclusion: Observational

NCT02377765
- https://clinicaltrials.gov/ct2/show/NCT02377765

Reason for exclusion: No participants with ADHD

NCT02398578
- https://clinicaltrials.gov/ct2/show/NCT02398578

Reason for exclusion: No participants with ADHD

NCT00928148
- https://clinicaltrials.gov/ct2/show/NCT00928148

Reason for exclusion: Manufacturer could not share data about this study

NCT01330693
- https://clinicaltrials.gov/ct2/show/NCT01330693

Reason for exclusion: Contacted author to enquire about study criteria but no reply

NCT01933880

- https://clinicaltrials.gov/ct2/show/NCT01933880

Reason for exclusion: Open label

NCT02730572
- https://clinicaltrials.gov/ct2/show/NCT02730572

Reason for exclusion: No RCT

NCT02293655
- https://clinicaltrials.gov/ct2/show/NCT02293655

Reason for exclusion: Estimated date completion: 2020

NCT02318017
- https://clinicaltrials.gov/ct2/show/NCT02318017

Reason for exclusion: Single dose

NCT02502799
- https://clinicaltrials.gov/ct2/show/NCT02502799

Reason for exclusion: Open label

NCT02470234
- https://clinicaltrials.gov/ct2/show/NCT02470234

Reason for exclusion: Open label

NCT02255565
- https://clinicaltrials.gov/ct2/show/NCT02255565

Reason for exclusion: single blind

NCT00206986
- https://clinicaltrials.gov/ct2/show/NCT00206986

Reason for exclusion: No participants with ADHD

NCT02704390
- https://clinicaltrials.gov/ct2/show/NCT02704390

Reason for exclusion: Ppen label

NCT00212732
- https://clinicaltrials.gov/ct2/show/NCT00212732

Reason for exclusion: No participants with ADHD

NCT00214981 (C1538D/312/AD/US)
- https://clinicaltrials.gov/ct2/show/NCT00214981

Reason for exclusion: No RCT

NCT00218036
- https://clinicaltrials.gov/ct2/show/NCT00218036

Reason for exclusion: No participants with ADHD

NCT00218062
- https://clinicaltrials.gov/ct2/show/NCT00218062

Reason for exclusion: No participants with ADHD

NCT00218231
- https://clinicaltrials.gov/ct2/show/NCT00218231

Reason for exclusion: No participants with ADHD

NCT00218387
- https://clinicaltrials.gov/ct2/show/NCT00218387

Reason for exclusion: No participants with ADHD

NCT02259517

- https://clinicaltrials.gov/ct2/show/NCT02259517

Reason for exclusion: Open label

NCT02271386

- https://clinicaltrials.gov/ct2/show/NCT02271386

Reason for exclusion: No pharmacological treatment

NCT02271607

- https://clinicaltrials.gov/ct2/show/NCT02271607

Reason for exclusion: No participants with ADHD

NCT02286349

- https://clinicaltrials.gov/ct2/show/NCT02286349

Reason for exclusion: No pharmacological treatment

NCT02477241

- https://clinicaltrials.gov/ct2/show/NCT02477241

Reason for exclusion: No participants with ADHD

NCT02478788

- https://clinicaltrials.gov/ct2/show/NCT02478788

Reason for exclusion: Estimated completion date: 2019

NCT02480829

- https://clinicaltrials.gov/ct2/show/NCT02480829

Reason for exclusion: No participants with ADHD

NCT02897570

- https://clinicaltrials.gov/ct2/show/NCT02897570

Reason for exclusion: Open label; No participants with ADHD

NCT02059642

- https://clinicaltrials.gov/ct2/show/NCT02059642

Reason for exclusion: Drug not pertinent for the meta-analysis (metadoxine) vs placebo; no other arms

NCT00223691

- https://clinicaltrials.gov/ct2/show/NCT00223691

Reason for exclusion: No participants with ADHD

NCT00301639

- https://clinicaltrials.gov/ct2/show/NCT00301639

Reason for exclusion: No participants with ADHD

NCT00302354

- https://clinicaltrials.gov/ct2/show/NCT00302354

Reason for exclusion: No participants with ADHD

NCT00302367

- https://clinicaltrials.gov/ct2/show/NCT00302367

Reason for exclusion: No participants with ADHD

NCT00302393

- https://clinicaltrials.gov/ct2/show/NCT00302393

Reason for exclusion: No participants with ADHD

NCT00364702

- https://clinicaltrials.gov/ct2/show/NCT00364702

Reason for exclusion: Not randomized

NCT00372359

- https://clinicaltrials.gov/ct2/show/NCT00372359

Reason for exclusion: Observational

NCT00393562

- https://clinicaltrials.gov/ct2/show/NCT00393562

Reason for exclusion: No participants with ADHD

NCT00541346

- https://clinicaltrials.gov/ct2/show/NCT00541346

Reason for exclusion: No RCT, drug of no interest for the present meta-analysis (transdermal patch MPH)

NCT00572026

- https://clinicaltrials.gov/ct2/show/NCT00572026

Reason for exclusion: Open label, drug of no interest for the present meta-analysis

NCT00780208

- https://clinicaltrials.gov/ct2/show/NCT00780208

Reason for exclusion: Non randomized, formulation of no interest for the present meta-analysis (transdermal patch)

NCT00802490

- https://clinicaltrials.gov/ct2/show/NCT00802490

Reason for exclusion: Non pharmacological interventions, single blind

NCT02136147

- https://clinicaltrials.gov/ct2/show/NCT02136147

Reason for exclusion: Observational study

NCT02151396

- https://clinicaltrials.gov/ct2/show/NCT02151396

Reason for exclusion: No pharmacological interventions

NCT02430896

- https://clinicaltrials.gov/ct2/show/NCT02430896

Reason for exclusion: No RCT

NCT02640651

- https://clinicaltrials.gov/ct2/show/NCT02640651

Reason for exclusion: No pharmacological interventions

NCT02674633

- https://clinicaltrials.gov/ct2/show/NCT02674633

Reason for exclusion: No pharmacological interventions

NCT02788851

- https://clinicaltrials.gov/ct2/show/NCT02788851

Reason for exclusion: Open label

NCT00439049

- https://clinicaltrials.gov/ct2/show/NCT00439049

Reason for exclusion: No participants with ADHD

NCT02790307

- https://clinicaltrials.gov/ct2/show/NCT02790307

Reason for exclusion: No participants with ADHD; Open label

NCT00152750

- https://clinicaltrials.gov/ct2/show/NCT00152750

Reason for exclusion: Author: final data analysis not available yet

NCT02170298

- https://clinicaltrials.gov/ct2/show/NCT02170298

Reason for exclusion: Study still ongoing, no data (confirmed by author)

NCT00723190

- https://clinicaltrials.gov/ct2/show/NCT00723190

Reason for exclusion: Open label

NCT00580814

- https://clinicaltrials.gov/ct2/show/NCT00580814

Reason for exclusion: No RCT; related to Volkow ND, Wang GJ, Kollins SH, Wigal TL, Newcorn JH, Telang F, Fowler JS, Zhu W, Logan J, Ma Y, Pradhan K, Wong C, Swanson JM. Evaluating dopamine reward pathway in ADHD: clinical implications. JAMA. 2009 Sep 9; 302(10): 1084-91. doi: 10.1001/jama.2009.1308. Erratum in: JAMA. 2009 Oct 7; 302(13): 1420. (Not in our original search across databases; retrieved via NCT number)

NCT00717392

- https://clinicaltrials.gov/ct2/show/NCT00717392

Reason for exclusion: No treatment of interest

NCT00829634

- https://clinicaltrials.gov/ct2/show/NCT00829634

Reason for exclusion: No participants with ADHD

NCT00894166

- https://clinicaltrials.gov/ct2/show/NCT00894166

Reason for exclusion: No participants with ADHD

NCT02179099

- https://clinicaltrials.gov/ct2/show/NCT02179099

Reason for exclusion: No participants with ADHD

NCT02206516

- https://clinicaltrials.gov/ct2/show/NCT02206516

Reason for exclusion: No pharmacological treatment

NCT02452879

- https://clinicaltrials.gov/ct2/show/NCT02452879

Reason for exclusion: No participants with ADHD

NCT02657057

- https://clinicaltrials.gov/ct2/show/NCT02657057

Reason for exclusion: No participants with ADHD

NCT02674269

- https://clinicaltrials.gov/ct2/show/NCT02674269

Reason for exclusion: No participants with ADHD

NCT02635035

- https://clinicaltrials.gov/ct2/show/NCT02635035

Reason for exclusion: E-mail to author to query around study status; reply: study still ongoing

NCT02638168

- https://clinicaltrials.gov/ct2/show/NCT02638168

Reason for exclusion: E-mail to author to query around study status; reply: still ongoing

NCT02493777

- https://clinicaltrials.gov/ct2/show/NCT02493777

Reason for exclusion: E-mail to author to query around study status; reply: still ongoing

NCT02803229

- https://clinicaltrials.gov/ct2/show/NCT02803229

Reason for exclusion: Estimated completion: June 2018

NCT02828644

- https://clinicaltrials.gov/ct2/show/NCT02828644

Reason for exclusion: No drug treatment

NCT02857816

- https://clinicaltrials.gov/ct2/show/NCT02857816

Reason for exclusion: No participants with ADHD

NCT01649232

- https://clinicaltrials.gov/ct2/show/NCT01649232

Reason for exclusion: No double blind, no treatment of interest for the present meta-analysis

NCT01787136

- https://clinicaltrials.gov/ct2/show/NCT01787136

Reason for exclusion: Added on therapy: dextromethorphan added on methylphenidate (MPH) or MPH only

NCT02210728

- https://clinicaltrials.gov/ct2/show/NCT02210728

Reason for exclusion: Open label

NCT02247986

- https://clinicaltrials.gov/ct2/show/NCT02247986

Reason for exclusion: Withdrawn

NCT00531752

- https://clinicaltrials.gov/ct2/show/NCT00531752

Reason for exclusion: Compound of no interest for the present meta-analysis (histamine H3 receptor antagonist) vs placebo

NCT00566449

- https://clinicaltrials.gov/ct2/show/NCT00566449

Reason for exclusion: Compound of no interest for the present meta-analysis (histamine H3 receptor antagonist) vs placebo

NCT01124708

- https://clinicaltrials.gov/ct2/show/NCT01124708

Reason for exclusion: Compound of no interest for the present meta-analysis (branadiclina, nicotininergic recepetor agonist) vs placebo

NCT01472991

- https://clinicaltrials.gov/ct2/show/NCT01472991

Reason for exclusion: Compound of no interest for the present meta-analysis (branadiclina, nicotininergic receptor agonist) vs placebo

NCT00391729

- https://clinicaltrials.gov/ct2/show/NCT00391729

Reason for exclusion: Compound of no interest for the present meta-analysis (nicotininergic receptor agonist) vs placebo

NCT00640419

- https://clinicaltrials.gov/ct2/show/NCT00640419

Reason for exclusion: Compound of no interest for the present meta-analysis (nicotininergic receptor agonist) vs placebo

NCT00640185

- https://clinicaltrials.gov/ct2/show/NCT00640185

Reason for exclusion: Compound of no interest for the present meta-analysis (nicotininergic receptor agonist) vs placebo

NCT02253745

- https://clinicaltrials.gov/ct2/show/NCT02253745

Reason for exclusion: Compound of no interest for the present meta-analysis (adenosine a2 antagonist) vs placebo

NCT02633527

- https://clinicaltrials.gov/ct2/show/NCT02633527

Reason for exclusion: Compound of no interest for the present meta-analysis (viloxazine) vs placebo

NCT00467428

- https://clinicaltrials.gov/ct2/show/NCT00467428

Reason for exclusion: Compound of no interest for the present meta-analysis (serotonin-norepinephrine-dopamine reuptake inhibitor) vs placebo

NCT00419445

- https://clinicaltrials.gov/ct2/show/NCT00419445

Reason for exclusion: Compound of no interest for the present meta-analysis (nicotinic acetylcholine receptor agonist) vs placebo

NCT00683462

- https://clinicaltrials.gov/ct2/show/NCT00683462

Reason for exclusion: Compound of no interest for the present meta-analysis (nicotinic partial agonist) vs placebo

NCT02163915

- https://clinicaltrials.gov/ct2/show/NCT02163915

Reason for exclusion: Compound of no interest for the present meta-analysis (AMPA receptor potentiator) vs placebo

NCT01012375

- https://clinicaltrials.gov/ct2/show/NCT01012375

Reason for exclusion: Compound of no interest for the present meta-analysis (nicotinic acetylcholine receptor agonist) vs placebo

NCT00611533

- https://clinicaltrials.gov/ct2/show/NCT00611533

Reason for exclusion: No participants with ADHD

NCT00169611

- https://clinicaltrials.gov/ct2/show/NCT00169611

Reason for exclusion: No participants with ADHD

NCT00247572

- https://clinicaltrials.gov/ct2/show/NCT00247572

Reason for exclusion: No participants with ADHD, intravenous medication

NCT02477280

- https://clinicaltrials.gov/ct2/show/NCT02477280

Reason for exclusion: 2-day study

NCT02473185

- https://clinicaltrials.gov/ct2/show/NCT02473185

Reason for exclusion: 2-day study

NCT01654250

- https://clinicaltrials.gov/ct2/show/NCT01654250

Reason for exclusion: Open label optimization phase (as detailed in FDA statistical evaluation, https://www.fda.gov/downloads/Drugs/DevelopmentApprovalProcess/DevelopmentResources/UCM483701.pdf)

NCT00794625
- https://www.clinicaltrials.gov/ct2/show/NCT00794625

Reason for exclusion: No arms of interest for the present meta-analysis ("stimulants" or other medications)

NCT01107496
- https://clinicaltrials.gov/ct2/show/NCT01107496

Reason for exclusion: Compound of no interest for the present meta-analysis (selective norepinephrine reuptake inhibitor) vs placebo

NCT01876719
- https://clinicaltrials.gov/ct2/show/NCT01876719

Reason for exclusion: Compound of no interest for the present meta-analysis (adrenergic receptor agonist) vs placebo

NCT02777931
- https://clinicaltrials.gov/ct2/show/NCT02777931

Reason for exclusion: Compound of no interest for the present meta-analysis (metabotropic glutamate receptor modulator) vs placebo

NCT02618434
- https://clinicaltrials.gov/ct2/show/NCT02618434

Reason for exclusion: Compound of no interest for the present meta-analysis (molindone) vs placebo

NCT00610441
- https://clinicaltrials.gov/ct2/show/NCT00610441

Reason for exclusion: Compound of no interest for the present meta-analysis (ampakine) vs placebo

NCT01201187
- https://clinicaltrials.gov/ct2/show/NCT01201187

Reason for exclusion: Compound of no interest for the present meta-analysis (Ginkgo biloba) vs placebo

NCT01415440
- https://clinicaltrials.gov/ct2/show/NCT01415440

Reason for exclusion: Estimated completion date: March 2017; Dr Posner confirmed study is still ongoing

NCT00142961
- https://clinicaltrials.gov/ct2/show/NCT00142961

Reason for exclusion: Contacted author to enquire about study status but no answer

NCT01274221
- https://clinicaltrials.gov/ct2/show/NCT01274221

Reason for exclusion: Withdrawn

NCT02450890
- https://clinicaltrials.gov/ct2/show/NCT02450890

Reason for exclusion: Estimated completion date: January 2017; Manufacturer contacted but not reply

NCT01831622
- https://clinicaltrials.gov/ct2/show/NCT01831622

Reason for exclusion: 2-day study

NCT00458445
- https://clinicaltrials.gov/ct2/show/NCT00458445

Reason for exclusion: Withdrawn

NCT02392169

- https://clinicaltrials.gov/ct2/show/NCT02392169

Reason for exclusion: No RCT

NCT02566824

- https://clinicaltrials.gov/ct2/show/NCT02566824

Reason for exclusion: Single blind, combined treatment

NCT02154321

- https://clinicaltrials.gov/ct2/show/NCT02154321

Reason for exclusion: Observational

NCT02155608

- https://clinicaltrials.gov/ct2/show/NCT02155608

Reason for exclusion: No pharmacological interventions

NCT02578342

- https://clinicaltrials.gov/ct2/show/NCT02578342

Reason for exclusion: Observational

NCT02580890

- https://clinicaltrials.gov/ct2/show/NCT02580890

Reason for exclusion: No pharmacological treatment

NCT02583529

- https://clinicaltrials.gov/ct2/show/NCT02583529

Reason for exclusion: No participants with ADHD

NCT02619721

- https://clinicaltrials.gov/ct2/show/NCT02619721

Reason for exclusion: No participants with ADHD

NCT02620410

- https://clinicaltrials.gov/ct2/show/NCT02620410

Reason for exclusion: No participants with ADHD

NCT02683265

- https://clinicaltrials.gov/ct2/show/NCT02683265

Reason for exclusion: Children aged 4-6 years old

NCT02167048

- https://clinicaltrials.gov/ct2/show/NCT02167048

Reason for exclusion: No arms of interest for the present meta-analysis: low, high dose of psychostimulants and no treatment (no placebo arm)

NCT02139111

- https://clinicaltrials.gov/ct2/show/NCT02139111

Reason for exclusion: No results posted in clincialtrial.gov, manufacturer replied they are not able to provide information on their products

NCT02139124

- https://clinicaltrials.gov/ct2/show/NCT02139124

Reason for exclusion: No results posted in clincialtrial.gov, manufacturer replied they are not able to provide information on their products

NCT02555150

- https://clinicaltrials.gov/ct2/show/NCT02555150

Reason for exclusion: No results posted in clincialtrial.gov, manufacturer replied they are not able to provide information on their products

NCT00914095

- https://clinicaltrials.gov/ct2/show/NCT00914095

Reason for exclusion: No participants with ADHD

NCT00498173

- https://clinicaltrials.gov/ct2/show/NCT00498173

Reason for exclusion: No results available

NCT02048241

- https://clinicaltrials.gov/ct2/show/NCT02048241

Reason for exclusion: No participants with ADHD

NCT01071044

- https://clinicaltrials.gov/ct2/show/NCT01071044

Reason for exclusion: No participants with ADHD

NCT00780650

- https://clinicaltrials.gov/ct2/show/NCT00780650

Reason for exclusion: No participants with ADHD

NCT02704546

- https://clinicaltrials.gov/ct2/show/NCT02704546

Reason for exclusion: Estimated completion April 2017

NCT02604407

- https://clinicaltrials.gov/ct2/show/NCT02604407

Reason for exclusion: Study results submitted; manufacturer not able to provide data

NCT02466425

- https://clinicaltrials.gov/ct2/show/NCT02466425

Reason for exclusion: Paper being drafted; manufacturer not able to provide data (21.2.17)

NCT00716274

- https://clinicaltrials.gov/ct2/show/NCT00716274

Reason for exclusion: Manufacture: no data available yet

NCT00562055

- https://clinicaltrials.gov/ct2/show/NCT00562055

Reason for exclusion: Withdrawn

NCT00485407 (B4Z-MC-LYCL)

- https://clinicaltrials.gov/ct2/show/NCT00485407

Reason for exclusion: Manufcaturer: Dose used is above the licensed doses. Dosing is done mg/kg in children and 2.4mg/kg is above licensed doses; also, no wash out prior to randomization; Increased-dose versus continued same-dose atomoxetine in patients who failed to respond optimally to an initial course of atomoxetine; no placebo arm; no other comparator

NCT01727414

- https://clinicaltrials.gov/ct2/show/NCT01727414

Reason for exclusion: Author let us know that the paper was currently under submission; not possible to share data at that stage

NCT01933217

- https://clinicaltrials.gov/ct2/show/NCT01933217

Reason for exclusion: No formal criteria for ADHD

NCT01940978

- https://clinicaltrials.gov/ct2/show/NCT01940978

Reason for exclusion: No arms of interest for the present meta-analysis: Methylphenidate ER QD +placebo BID vs Methylphenidate ER QD + Cyproheptadine hydrochloride 2.5mg BID +ER QD Cyproheptadine hydrochloride 5.0mg BID, no placebo only arm, no methylphenidate ER arm only

NCT02039908 (MH099030)
- https://clinicaltrials.gov/ct2/show/NCT02039908

Reason for exclusion: Ongoing (end study planned in June 2017), comparison of two doses of the same compound, no placebo

NCT00254033
- https://clinicaltrials.gov/ct2/show/NCT00254033

Reason for exclusion: No ADHD

NCT00890240 (JNJ-31001074)
- http://clinicaltrials.gov/show/NCT00890240

Reasons for exclusion: No pertinent treatment for the present meta-analysis. No other drugs pertinent for the present meta-analysis

NCT00890292 (JNJ-31001074)
- http://clinicaltrials.gov/show/NCT00890292

Reasons for exclusion: Open label; no drug of interest for the present meta-analysis

NCT02489279
- https://clinicaltrials.gov/ct2/show/NCT02489279

Reason for exclusion: Non pharmacological treatment

Neef2005
- Neef NA, Bicard DF, Endo S, Coury DL, Aman MG. Evaluation of pharmacological treatment of impulsivity in children with attention deficit hyperactivity disorder. *J Appl Behav Anal.* 2005;38(2):135-146.

Reason for exclusion: No randomized

Nemzer1986
- Nemzer ED, Arnold LE, Votolato NA, McConnell H. Amino acid supplementation as therapy for attention deficit disorder. *J Am Acad Child Psychiatry.* 1986;25(4):509-513.

  Reason for exclusion: Cross-over without wash out; pre-cross over data not available

Neu2012
- Neu D, De Buisseret FXH, Oswald P, Verbanck P. Dopaminergic crossroads: clinical implications in ADHD and restless leg syndrom. *Eur Neuropsychopharmacol.* 2012;22(Suppl. 2):S419-S420.

Reason for exclusion: No RCT

Newcorn2003
- Newcorn J. ADHD in girls: response to once-daily OROS methylphenidate (MPH). *156th Annual Meeting of the American Psychiatric Association; 2003; San Francisco, CA.* 2003:Nr707.

Reason for exclusion: Only abstract available; First author replied:" I don't think there was a separate paper. Probably these data were culled from the various studies you were able to access"

Newcorn2004
- Newcorn JH. Atomoxetine for comorbid adhd and affective symptoms. *157th Annual Meeting of the American Psychiatric Association*; 2004 May 1-6; New York, NY2004:No. 36.

Reason for exclusion: atomoxetine + placebo vs. atomoxetine + fluoxetine

Newcorn2007
- Newcorn JH. Psychopharmacologic treatment of attention-deficit hyperactivity disorder and disruptive Behavior disorders. *Pediatr Ann.* 2007;37(7):477-+.

Reason for exclusion: Review

Newcorn2008
- Newcorn JH. Nonstimulants and emerging treatments in adults with ADHD. *CNS Spectr.* 2008;13(9):12-16.

Reason for exclusion: Review

Newcorn 2014

- Newcorn J, Duhoux S, Schulz K, et al. Effects of lisdexamfetamine (vyvanse) on reward processing. *Biol Psychiatry.* 2014;75(9 suppl. 1):7s.

Reason for exclusion: First author contacted; reply: results from full dataset not yet available

Newcorn2015

- Newcorn J, Nagy P, Childress A, et al. Randomized, double-blind, active- and placebocontrolled trials of lisdexamfetamine dimesylate in adolescents with Attention-Deficit/Hyperactivity Disorder. *ADHD. Atten Defic Hyperact Disord.* 2015;7:S47.

Reason for exclusion: First author contacted, reply: full paper currently under review, not possible to share data

Newcorn2016a (NCT01081145, EudraCT 2009-018161-12, SPD503-315)

- Newcorn J, Harpin V, Huss M, et al. Long-Term Maintenance of Efficacy of Extended-Release Guanfacine Hydrochloride (GXR) in Children and Adolescents with Attention-Deficit/Hyperactivity Disorder (Adhd): Double-Blind, Placebo-Controlled, Multicentre, Phase 3 Randomized Withdrawal Study. *Eur Psychiatry.* 2014;29
- Pooled in: Huss M, Hervas A, Newcorn JH, et al. Guanfacine extended release for attention-deficit/hyperactivity disorder following inadequate response to prior methylphenidate. *Eur Neuropsychopharmacol.* 2014;24:S727-S728.
- Huss M, Newcorn J, Harpin V, et al. Extended-release guanfacine hydrochloride in children and adolescents with attentiondeficit/hyperactivity disorder: A double-blind, placebocontrolled, multicentre, phase 3 randomized withdrawal study. *Aust N Z J Psychiatry*. 2015;49:111-112.
- Newcorn JH, Harpin V, Huss M, et al. Extended-release guanfacine hydrochloride in 6-17-year olds with ADHD: a randomised-withdrawal maintenance of efficacy study. *J Child Psychol Psychiatry*. 2016;57(6):717-728.
- Used for analysis in : Huss M, Sikirica V, Hervas A, Newcorn JH, Harpin V, Robertson B. Guanfacine extended release for children and adolescents with attention-deficit/hyperactivity disorder: efficacy following prior methylphenidate treatment. *Neuropsychiatr Dis Treat*. 2016;112:1085-1101.
- Used for analysis in: Huss M, Hervas A, Dirks B, Bliss C, Prochazka J, Cutler A. Guanfacine extended release for children and adolescents with attention-deficit/hyperactivity disorder: Efficacy following prior stimulant treatment. *Eur Neuropsychopharmacol.* 2016;26:S737.
- https://clinicaltrials.gov/ct2/show/NCT01081145
- https://www.clinicaltrialsregister.eu/ctr-search/search?query=eudract_number:2009-018161-12

Reason for exclusion: Responders in open label phase to guanfacine extended release randomized to guanfacine extended release or placebo

Newcorn2016b

- Newcorn J. ADHD and disruptive behavior disorders: Neurobiology and response to stimulant and non-stimulant treatment. *Neuropsychopharmacology.* 2016;41:S56.

Reason for exclusion: No empirical paper

Newmark2009

- Newmark SC. Nutritional Intervention in ADHD. *Explore (NY).* 2009;5(3):171-174.

Reason for exclusion: Review

Newsome2009

- Newsome MR, Scheibel RS, Seignourel PJ, et al. Effects of Methylphenidate on Working Memory in Traumatic Brain Injury: A Preliminary fMRI Investigation. *Brain Imaging Behav.* 2009;3(3):298-305.

Reason for exclusion: Only brain injury patients; no comorbid ADHD

Niederhofer2007

- Niederhofer H. St. John's wort may diminish methylphenidate's efficacy in treating patients suffering from attention deficit hyperactivity disorder. *Med Hypotheses.* 2007;68(5):1189.

Reason for exclusion: Case report

Niederhofer2009

- Niederhofer H. Atomoxetine may improve methylphenidates' efficacy in treatment of ADHD? *Psychiatr Danub.* 2009;21(3):330.

Reason for exclusion: Case report

Niederhofer2010

- Niederhofer H. Duloxetine may improve some symptoms of attention-deficit/hyperactivity disorder. *Prim Care Companion J Clin Psychiatry.* 2010;12(2).

Reason for exclusion: Case reports

Nigg1997

- Nigg JT, Swanson JM, Hinshaw SP. Covert visual spatial attention in boys with attention deficit hyperactivity disorder: lateral effects, methylphenidate response and results for parents. *Neuropsychologia.* 1997;35(2):165-176.

Reason for exclusion: No outcome of interest, no pre-cross over data, unclear duration of each condition

Nikishina2008

- Nikishina IS, Chutko LS, Surushina SI, Iakovenko EA, Kropotov ID. [Electroencephalographic study of children with attention deficit hyperactivity disorder before and after treatment with strattera]. *Zh Nevrol Psikhiatr Im S S Korsakova.* 2008;108(12):60-62.

Reason for exclusion: No RCT

Nikles2006

- Nikles CJ, Mitchell GK, Del Mar CB, Clavarino A, McNairn N. An n-of-1 trial service in clinical practice: testing the effectiveness of stimulants for attention-deficit/hyperactivity disorder. *Pediatrics.* 2006;117(6):2040-2046.

Reason for exclusion: N-of-1 trial Duration of each condition: 2 days

Nolan1999

- Nolan EE, Gadow KD, Sprafkin J. Stimulant medication withdrawal during long-term therapy in children with comorbid attention-deficit hyperactivity disorder and chronic multiple tic disorder. *Pediatrics.* 1999;103(4 Pt 1):730-737.

Reason for exclusion: Withdrawal design; Not: 10 subjects participated in a previous study by Gadow et al. (Gadow KD, Sverd J, Sprafkin J, Nolan EE, Ezor SN. Efficacy of methylphenidate for attention-deficit hyperactivity disorder in children with tic disorder. Arch Gen Psychiatry. 1995;52(6):444-455)

Northup1997a

- Northup J, Fusilier I, Swanson V, Roane H, Borrero J. An evaluation of methylphenidate as a potential establishing operation for some common classroom reinforcers. *J Appl Behav Anal.* 1997;30(4):615-625.

Reason for exclusion: No RCT

Northup1997b

- Northup J, Jones K, Broussard C, et al. A preliminary analysis of interactive effects between common classroom contingencies and methylphenidate. *J Appl Behav Anal.* 1997;30(1):121-125.

Reason for exclusion: Single case

Northup1999

- Northup J, Fusilier I, Swanson V, et al. Further analysis of the separate and interactive effects of methylphenidate and common classroom contingencies. *J Appl Behav Anal.* 1999;32(1):35-50.

Reason for exclusion: Four case reports

Novak1995

- Novak GP, Solanto M, Abikoff H. Spatial orienting and focused attention in attention deficit hyperactivity disorder. *Psychophysiology.* 1995;32(6):546-559.

Reason for exclusion: Single case

NTR5679

- http://www.trialregister.nl/trialreg/admin/rctview.asp?TC=5679

Reasons for exclusion: Single dose

NTR996 (ISRCTN32841168)

- http://www.trialregister.nl/trialreg/admin/rctview.asp?TC=996

Reasons for exclusion: No randomised

NTR4206

- http://www.trialregister.nl/trialreg/admin/rctview.asp?TC=4206

Reasons for exclusion: No blinded

NTR4337

- http://www.trialregister.nl/trialreg/admin/rctview.asp?TC=4337

Reasons for exclusion: Ongoing

NTR4877

- http://www.trialregister.nl/trialreg/admin/rctview.asp?TC=4877

Reasons for exclusion: No arms of interest for the present meta-analysis: methylphenidate + alimemazine vs methylphenidate + placebo

NTR1947

- http://www.trialregister.nl/trialreg/admin/rctview.asp?TC=1947

Reasons for exclusion: No blinded

NTR3127

- http://www.trialregister.nl/trialreg/admin/rctview.asp?TC=3127

Reasons for exclusion: No RCT

NTR3201

- http://www.trialregister.nl/trialreg/admin/rctview.asp?TC=3201

Reasons for exclusion: No blind; preschoolers

NTR5252

- http://www.trialregister.nl/trialreg/admin/rctview.asp?TC=5252

Reasons for exclusion: Ongoing; planned to end in October 2017; withdrawal design

NTR447 (ISRCTN25479460)

- http://www.trialregister.nl/trialreg/admin/rctview.asp?TC=447

Reasons for exclusion: No RCT

NTR2848

- http://www.trialregister.nl/trialreg/admin/rctview.asp?TC=2848

Reasons for exclusion: No RCT

NTR2505

- http://www.trialregister.nl/trialreg/admin/rctview.asp?TC=2505

Reasons for exclusion: No RCT (Protocol: Janssen M, Wensing M, van der Gaag RJ, Cornelissen I, van Deurzen P, Buitelaar J. Improving patient care for attention deficit hyperactivity disorder in children by organizational redesign (Tornado program) and enhanced collaboration between psychiatry and general practice: a controlled before and after study. Implement Sci. 2014 Oct 30;9:155.)

NTR3021

- http://www.trialregister.nl/trialreg/admin/rctview.asp?TC=3021

Reasons for exclusion: No pharmacological treatment

NTR3073

- http://www.trialregister.nl/trialreg/admin/rctview.asp?TC=3073

Reasons for exclusion: No participants with ADHD

NTR5223

- http://www.trialregister.nl/trialreg/admin/rctview.asp?TC=5223

Reasons for exclusion: Cognitive training

Nuijten2016

- Nuijten M, Blanken P, Van den Brink W, Goudriaan AE, Hendriks VM. Impulsivity and attentional bias as predictors of modafinil treatment outcome for retention and drug use in crack-cocaine dependent patients: Results of a randomised controlled trial. *J Psychopharmacol*. 2016;30(7):616-26

Reason for exclusion: No participants with ADHD

Null2005

- Null G, Feldman M. The benefits of going beyond conventional therapies for ADHD. *J Orthomol Med.* 2005;20(2):75-88.

Reason for exclusion: Review

O'Driscoll2005

- O'Driscoll GA, Depatie L, Holahan A-LV, et al. Executive functions and methylphenidate response in subtypes of attention-deficit/hyperactivity disorder. *Biol Psychiatry.* 2005;57(11):1452-1460.

Reason for exclusion: Single dose

O'Leary1978

- O'Leary SG, Pelham WE. Behavior therapy and withdrawal of stimulant medication in hyperactive children. *Pediatrics.* 1978;61(2):211-217.

Reason for exclusion: No DSM/ICD criteria; No RCT with placebo arm

O'Malley2000

- O'Malley KD, Koplin B, Dohner VA. Psychostimulant clinical response in fetal alcohol syndrome. *Can J Psychiatry - Revue Canadienne de Psychiatrie.* 2000;45(1):90-91.

Reason for exclusion: Single dose

O'Toole1997

- O'Toole KM. The effects of methylphenidate dose on attention and nonverbal learning in children with attention-deficit hyperactivity disorder [Ph.D.]. *Ann Arbor, Georgia State University; 1994.*
- O'Toole KM. The effects of methylphenidate dose on attention and nonverbal learning in children with attention-deficit hyperactivity disorder. *Dissertation Abstracts International Section A: Humanities and Social Sciences. 1995;55(10-A):3141.*
- O'Toole K, Abramowitz A, Morris R, Dulcan M. Effects of methylphenidate on attention and nonverbal learning in children with attention-deficit hyperactivity disorder. *J Am Acad Child Adolesc Psychiatry.* 1997;36(4):531-538.

Reason for exclusion: Single dose

Oberpichler-Schwenk2006

- Oberpichler-Schwenk H. [Attention deficit disorder with hyperactivity (ADHD). Continuing symptom control with medium term methylphenidate treatment].*Med Monatsschr Pharm.* 2006;29(11):415-416.

Reason for exclusion: Review

Oesterheld1998

- Oesterheld JR, Kofoed L, Tervo R, Fogas B, Wilson A, Fiechtner H. Effectiveness of methylphenidate in Native American children with fetal alcohol syndrome and attention deficit/hyperactivity disorder: a controlled pilot study. *J Child Adolesc Psychopharmacol.* 1998;8(1):39-48.

Reason for exclusion: Duration of each medication condition: 5 days for 3 consecutive weeks.

Oettinger1975

- Oettinger L, Jr. The use of amphetamines in hyperactivity. *Dev Med Child Neurol.* 1975;17(1):117.

Reason for exclusion: Commentary

Ogrim2013

- Ogrim G, Hestad KA, Brunner JF, Kropotov J. Predicting acute side effects of stimulant medication in pediatric attention deficit/hyperactivity disorder: data from quantitative electroencephalography, event-related potentials, and a continuous-performance test. *Neuropsychiatr Dis Treat.* 2013;9:1301-1309.

Reason for exclusion: No RCT

Ogrim2014

- Ogrim G, Kropotov J, Brunner JF, Candrian G, Sandvik L, Hestad KA. Predicting the clinical outcome of stimulant medication in pediatric attention-deficit/hyperactivity disorder: data from quantitative electroencephalography, event-related potentials, and a go/no-go test. *Neuropsychiatr Dis Treat.* 2014;10:231-242.

Reason for exclusion: No RCT

Oh2007

- Oh E, Yoo JH. Adolescent attention deficit/hyperactivity disorder (ADHD) treatment with controlled-release methylphenidate. *Eur Neuropsychopharmacol.* 2007;17(Suppl. 4):S567-S568.

Reason for exclusion: Open trial

Ohlmeier2007a

- Ohlmeier MD, Prox V, Zhang Y, et al. Effects of methylphenidate in ADHD adults on target evaluation processing reflected by event-related potentials. *Neurosci Lett.* 2007;424(3):149-154.

Reason for exclusion: No RCT

Ohlmeier2007b

- Ohlmeier MD. Pharmacotherapy of ADHD in adults with comorbid depression. *Psychiatr Prax.* 2007;34 (Suppl 3):S296-9

Reason for exclusion: Review

Okada2008

- Okada T. [Mechanism for the efficacy of methylphenidate on attention deficit/hyperactivity disorder and related clinical evidence]. *Seishin Shinkeigaku Zasshi - Psychiatria et Neurologia Japonica.* 2008;110(10):932-940.

Reason for exclusion: Review

Olfson2008

- Olfson M, Marcus SC, Zhang HF, Wan GJ. Stimulant dosing in the community treatment of adult attention-deficit/hyperactivity disorder. *J Clin Psychopharmacol.* 2008;28(2):255-257.

Reason for exclusion: No RCT

Orgill1996

- Orgill A, Serfontein S. Behavioural & Cognitive Effects of Stimulant & Non Stimulant Drugs in Childhood Attention Deficit Disorder. *XXth Collegium Internationale Neuro-psychopharmacologicum.* 1996:56.

Reason for exclusion: Single dose.

Ottinger1985

- Ottinger DR, Halpin B, Miller M, Demian L, Hannemann R. Evaluating drug effectiveness in an office setting for children with attention deficit disorders. *Clin Pediatr (Phila).* 1985;24(5):245-251.

Reason for exclusion: Two case reports

Overtoom2003

- Overtoom CC, Verbaten MN, Kemner C, et al. Effects of methylphenidate, desipramine, and L-dopa on attention and inhibition in children with Attention Deficit Hyperactivity Disorder. *Behav Brain Res.* 2003;145(1-2):7-15.

Reason for exclusion: Less than seven days treatment

Overtoom2009

- Overtoom CCE, Bekker EM, Kenemans JL, Verbaten MN, van der Molen MW, Kooij JJS, Buitelaar JK. A dose-response study of methylphenidate and paroxetine on inhibition and attention in adults with Attention Deficit/Hyperactivity Disorder. *J Cog Neurosci*. 2005; 221.
- Overtoom CC, Bekker EM, van der Molen MW, et al. Methylphenidate restores link between stop-signal sensory impact and successful stopping in adults with attention-deficit/hyperactivity disorder. *Biol Psychiatry.* 2009;65(7):614-619.

Reason for exclusion: Less than seven days treatment

Paclt1996

- Paclt I, Florian J, Brunclikova J, Ruzickova I. Effect of Aponeuron in the treatment of children with hyperkinetic syndrome. [Czech]*Ceska Slov Psychiatr.* 1996 ;92 (Suppl 1):41-57

Reason for exclusion: Drug retired from the market

Page1974

- Page JG, Janicki RS, Bernstein JE. Pemoline (Cylert) in the treatment of childhood hyperkinesis. *J learn disabilities.* 1974(8):498-503.

Reason for exclusion: Two arms: placebo and drug of no interest for the present meta-analysis

Palumbo2015

- Palumbo DR, Belden HW, Berry SA. Methylphenidate extended-release oral suspension (MEROS) improves ADHD-rating scale and permanent product measure of performance scores in children with ADHD. *Ann Neurol.* 2015;78:(S19):S166.

Reason for exclusion: Contacted authors on to gather full text; reply: full text not yet publically available

Pan2008

- Pan XX, Ma HW, Wan B, Dai XM. Effectiveness of oral osmotic-methylphenidate in treatment of attention deficit hyperactivity disorder in children. [Chinese]. *Zhongguo Dang Er Ke Za Zhi.* 2008;10(4):471-474.

Reason for exclusion: Comparison of two formulations of the same compound

Park2013

- Park S, Hong SB, Kim JW, et al. White-matter connectivity and methylphenidate-induced changes in attentional performance According to alpha2A-adrenergic receptor gene polymorphisms in Korean children with attention-deficit hyperactivity disorder. *J Neuropsychiatry Clin Neurosci.* 2013;25(3):222-228.

Reason for exclusion: No randomized

Park2014a

- Park S, Kim BN, Kim JW, et al. Neurotrophin 3 genotype and emotional adverse effects of osmotic-release oral system methylphenidate (OROS-MPH) in children with attention-deficit/hyperactivity disorder. *J Psychopharmacol.* 2014;28(3):220-226.

Reason for exclusion: No randomized

Park2014b

- Park S, Kim J-W, Kim B-N, Shin M-S, Yoo H-J, Cho S-C. Catechol-O-methyltransferase Val(158)-Met polymorphism and a response of hyperactive-impulsive symptoms to methylphenidate: A replication study from South Korea. *J Psychopharmacol.* 2014 ;28(7):671-6

Reason for exclusion: No randomized

Park2016

- Park JH, Lee YS, Sohn JH, Han DH. Effectiveness of atomoxetine and methylphenidate for problematic online gaming in adolescents with attention deficit hyperactivity disorder. *Hum Psychopharmacol.* 2016;31(6):427-432

Reason for exclusion: Single blind

Paul-Jordanov2010

- Paul-Jordanov I, Bechtold M, Gawrilow C. Methylphenidate and if-then plans are comparable in modulating the P300 and increasing response inhibition in children with ADHD. *Atten Defic Hyperact Disord.* 2010;2(3):115-126.

Reason for exclusion: No RCT

Pearson1996

- Pearson DA, Santos CW, Roache JD, et al. Effects of methylphenidate on behavioral adjustment in children with mental retardation and ADHD: Preliminary findings from a study in progress. *J Dev Phys Disabil.* 1996;8(4):313-333.

Reason for exclusion: Latin square, no mention of randomization.

Pearson2004

- Pearson DA, Santos CW, Roache JD, et al. Treatment effects of methylphenidate on behavioral adjustment in children with mental retardation and ADHD. *J Am Acad Child Adolesc Psychiatry.* 2003;42(2):209-216.
- Pearson DA, Lane DM, Santos CW, et al. Effects of methylphenidate treatment in children with mental retardation and ADHD: individual variation in medication response. *J Am Acad Child Adolesc Psychiatry.* 2004;43(6):686-698.
- Pearson DA, Santos CW, Casat CD, et al. Treatment effects of methylphenidate on cognitive functioning in children with mental retardation and ADHD. *J Am Acad Child Adolesc Psychiatry.*2004;43(6):677-685.

Reason for exclusion: Latin square, no mention of randomization; Cross-over without wash out; pre-cross over data not available

Pearson2013 (NCT00178503)

- Pearson DA, Santos CW, Aman MG, et al. Effects of extended release methylphenidate treatment on ratings of attention-deficit/hyperactivity disorder (ADHD) and associated behavior in children with autism spectrum disorders and ADHD symptoms. *J Child Adolesc Psychopharmacol.* 2013;23(5):337-351.

- Anonymous. Methylphenidate dosing improved behavior in children with ASD. *The Brown University Child & Adolescent Psychopharmacology Update* 2013;15 (8):4–5.
- https://clinicaltrials.gov/ct2/show/NCT00178503

Cross-over without wash out; pre-cross over data not available

Peeke1984

- Peeke S, Halliday R, Callaway E, Prael R, Reus V. Effects of two doses of methylphenidate on verbal information processing in hyperactive children. *J Clin Psychopharmacol.* 1984;4(2):82-88.

Reason for exclusion: Less than seven days treatment

Peksel2015

- Peksel H, Sobanski E, Leppamaki S, et al. Patterns of response to atomoxetine in the treatment of adult patients with Attention Deficit Hyperactivity Disorder. *Klinik Psikofarmakoloji Bulteni.* 2015;25:S83

Reason for exclusion: Analysis of previous double-blind RCT or open label studies of atomoxetine; according tot Lilly, our dataset includes all studies of atomoxetine

Pelham1980

- Pelham WE, Schnedler RW, Bologna NC, Contreras JA. Behavioral and stimulant treatment of hyperactive children: a therapy study with methylphenidate probes in a within-subject design. *J Appl Behav Anal.* 1980;13(2):221-236.

Reason for exclusion: Cross-over no wash out. DSM-II criteria

Pelham1985

- Pelham WE, Bender ME, Caddell J, Booth S, Moorer SH. Methylphenidate and children with attention deficit disorder. Dose effects on classroom academic and social behavior. *Arch Gen Psychiatry.* 1985;42(10):948-952.

Reason for exclusion: Reason for exclusion: Less than seven days treatment (one week excluded week ends)

Pelham1986

- Pelham WE, Milich R, Walker JL. Effects of continuous and partial reinforcement and methylphenidate on learning in children with Attention Deficit Disorder. *J Abnorm Psychol.* 1986(4):319-325.

Reason for exclusion: Less than seven days treatment; Note: according to:Storebø et al. 2015, linked to Johnston C, Pelham WE, Hoza J, Sturges J. Psychostimulant rebound in attention deficit disordered boys. *J Am Acad Child Adolesc Psychiatry.* 1988;27(6):806–10.

Pelham1988

- Pelham WE, Schnedler RW, Bender ME, et al. The combination of behavior therapy and methylphenidate in the treatment of attention deficit disorders: A therapy outcome study. *Bloomingdale, Lewis M [Ed].* 1988;3:29-48.

Reason for exclusion: No arms of interest for the present meta-analysis (in four arms, CBT; in the fifth group: social skills training only).

Pelham1989

- Pelham WE, Jr., Walker JL, Sturges J, Hoza J. Comparative effects of methylphenidate on ADD girls and ADD boys. *J Am Acad Child Adolesc Psychiatry.* 1989;28(5):773-776.

Reason for exclusion: 5-9 days of data per medication

Pelham1990a

- Pelham WE, Jr., Greenslade KE, Vodde-Hamilton M, et al. Relative efficacy of long-acting stimulants on children with attention deficit-hyperactivity disorder: a comparison of standard methylphenidate, sustained-release methylphenidate, sustained-release dextroamphetamine, and pemoline. *Pediatrics.* 1990;86(2):226-237.

Reason for exclusion: Less than seven days treatment, no outcomes of interest for the present meta-analysis

Pelham1990b

- Pelham WE, Jr., McBurnett K, Harper GW, et al. Methylphenidate and baseball playing in ADHD children: who's on first? *J Consult Clin Psychol.* 1990;58(1):130-133.

Reason for exclusion: Less than seven days treatment; No outcomes of interest for the present meta-analysis

Pelham1991

- Pelham WE, Vodde-Hamilton M, Murphy DA, Greenstein J, Vallano G. The effects of methylphenidate on ADHD adolescents in recreational, peer group, and classroom settings. *J Clin Child Psychol.* 1991;20:293-300.

Reason for exclusion: Duration of medication: from 4 to 11 days

Pelham1991

- Pelham WE, Milich R, Cummings EM, Murphy DA, Schaughency EA, Greiner AR. Effects of background anger, provocation, and methylphenidate on emotional arousal and aggressive responding in attention-deficit hyperactivity disordered boys with and without concurrent aggressiveness. *J Abnorm Child Psychol.* 1991(4):407-426.

Reason for exclusion: Less than seven days treatment

Pelham1992

- Pelham WE, Murphy DA, Vannatta K, et al. Methylphenidate and attributions in boys with attention-deficit hyperactivity disorder. *J Consult Clin Psychol.* 1992;60(2):282-292.
- Pelham WE, Murphy DA, Vannatta K, Milich R, et al. Methylphenidate and attributions in boys with attention-deficit hyperactivity disorder. *Annual Progress in Child Psychiatry & Child Development.* 1993:242-265.

Reason for exclusion: No outcomes of interest for the present meta-analysis; Note: Same procedure as: Pelham WE, Jr., Greenslade KE, Vodde-Hamilton M, et al. Relative efficacy of long-acting stimulants on children with attention deficit-hyperactivity disorder: a comparison of standard methylphenidate, sustained-release methylphenidate, sustained-release dextroamphetamine, and pemoline. Pediatrics. Aug 1990;86(2):226-237 (Less than seven days treatment - 3 to 6 days of treatment each arm).

Pelham1993

- Pelham WE, Jr., Carlson C, Sams SE, Vallano G, Dixon MJ, Hoza B. Separate and combined effects of methylphenidate and behavior modification on boys with attention deficit-hyperactivity disorder in the classroom. *J Consult Clin Psychol.* 1993;61(3):506-515.

Reason for exclusion: Cross-over without wash out; pre-cross over data not available

Pelham1995

- Pelham WE, Swanson JM, Furman MB, Schwindt H. Pemoline effects on children with adhd - a time-response by dose-response analysis on classroom measures. *J Am Acad Child Adolesc Psychiatry.* 1995;34(11):1504-1513.

Reason for exclusion: Medication of no interest for the present meta-analysis vs. placebo

Pelham1997

- Pelham WE, Hoza B, Kipp HL, Gnagy EM, Trane ST. Effects of methylphenidate and expectancy of ADHD children's performance, self-evaluations, persistence, and attributions on a cognitive task. *Exp Clin Psychopharmacol.* 1997;5(1):3-13.

Reason for exclusion: no outcome of interest for the present meta-analysis; likely, less than seven days treatment

Pelham1999

- Pelham WE, Aronoff HR, Midlam JK, et al. A comparison of ritalin and adderall: efficacy and time-course in children with attention-deficit/hyperactivity disorder. *Pediatrics.* 1999;103(4):e43.

Reason for exclusion: Each drug: 5 days treatment; Co–intervention (parent training)

Pelham1999

- Pelham WE, Gnagy EM, Chronis AM, et al. A comparison of morning-only and morning/late afternoon Adderall to morning-only, twice-daily, and three times-daily methylphenidate in children with attention-deficit/hyperactivity disorder. *Pediatrics.* 1999;104(6):1300-1311.
- Chronis AM, Pelham WE, Jr., Gnagy EM, Roberts JE, Aronoff HR. The impact of late-afternoon stimulant dosing for children with ADHD on parent and parent-child domains. *J Clin Child Adolesc Psychol: the official journal for the Society of Clinical Child and Adolescent Psychology, American Psychological Association, Division 53.* 2003;32(1):118-126.

Reason for exclusion: Reason for exclusion: Less than seven days treatment for each condition

Pelham2001a (NCT00269789)

- Pelham WE, Hoffman MT, Lock T. Evaluation of once-a-day OROS methylphenidate HCI (MPH)extended-release tablets versus MPH tid in children with ADHD in natural school settings. *Pediatr Res*. 2000(4):31a.
- Pelham WE, Gnagy EM, Burrows-Maclean L, et al. Once-a-day Concerta methylphenidate versus three-times-daily methylphenidate in laboratory and natural settings. *Pediatrics.* 2001;107(6):E105.
- Williams L. Methylphenidate HCI extended-release tablets for children with ADHD: parent treatment prefence and satisfaction. *Pediatr Res*. 2001:429a.

- Swanson J. Impact of an OROS formulation of mwethylkphenidate on activity levels odf children with ADHD. *Pediatr Res.* 2001:429a.
- Commentary in: Connor. Once a day Concerta methylphenidate was equivalent to 3 times daily methylphenidate in children with ADHD. *Evid Based Ment Health*. 2002, 5, 20.
- Pooled in: Palumbo D, Spencer T, Lynch J, Co-Chien H, Faraone SV. Emergence of tics in children with ADHD: impact of once-daily OROS methylphenidate therapy. *J Child Adolesc Psychopharmacol*. 2004;14(2):185-194.

Reason for exclusion: Co-treatment with behavioral intervention.

Pelham2001b

- Pelham WE, Jr., Waschbusch DA, Hoza B, Pillow DR, Gnagy EM. Effects of methylphenidate and expectancy on performance, self-evaluations, persistence, and attributions on a social task in boys with ADHD. *Exp Clin Psychopharmacol.* 2001;9(4):425-437.

Reason for exclusion: no relevant outcomes for the present meta-analysis; likely, Less than seven days treatment

Pelham2002

- Pelham WE, Hoza B, Pillow DR, et al. Effects of methylphenidate and expectancy on children with ADHD: behavior, academic performance, and attributions in a summer treatment program and regular classroom settings. *J Consult Clin Psychol.* 2002;70(2):320-335.
- King S, Waschbusch DA, Pelham WE, Frankland BW, Corkum PV, Jacques S. Subtypes of Aggression in Children with Attention Deficit Hyperactivity Disorder: Medication Effects and Comparison with Typical Children. *J Clin Child Adolesc Psychol.* 2009;38(5):619-629.
- King S, Waschbusch DA, Pelham WE, Jr., et al. Social information processing in elementary-school aged children with ADHD: medication effects and comparisons with typical children. *J Abnorm Child Psychol.* 2009;37(4):579-589.

Reason for exclusion: Less than seven days treatment, no outcomes of interest for the present meta-analysis

Pelham2005

- Pelham WE, Burrows-Maclean L, Gnagy EM, et al. Transdermal methylphenidate, behavioral, and combined treatment for children with ADHD. *Exp Clin Psychopharmacol.* 2005;13(2):111-126.
- Pelham WE, Jr., Manos MJ, Ezzell CE, et al. A dose-ranging study of a methylphenidate transdermal system in children with ADHD. *J Am Acad Child Adolesc Psychiatry.* 2005;44(6):522-529.

Reason for exclusion: Transdermal formulation, no oral formulations

Pelham2011

- Pelham WE, Waxmonsky JG, Schentag J, et al. Efficacy of a methylphenidate transdermal system versus t.i.d. methylphenidate in a laboratory setting. *J Atten Disord.* 2011;15(1):28-35.

Reason for exclusion: "All participants were receiving a stable dose of IR MPH before enrolment". First author confirmed that "stable" likely means "responders".

Pelham2011

- Pelham WE, Jr., Waschbusch DA, Hoza B, et al. Music and video as distractors for boys with ADHD in the classroom: comparison with controls, individual differences, and medication effects. *J Abnorm Child Psychol.* 2011;39(8):1085-1098.

Reason for exclusion: Co-treatment (behavioral intervention)

Pelham2014 (NCT00050622)

- Fabiano GA, Pelham WE, Gnagy EM, et al. The single and combined effects of multiple intensities of behavior modification and methylphenidate for children with attention deficit hyperactivity disorder in a classroom setting. *School Psychology Review.* 2007;36(2):195-216.
- Pelham WE, Burrows-Maclean L, Gnagy EM, et al. A Dose-Ranging Study of Behavioral and Pharmacological Treatment in Social Settings for Children with ADHD. *J Abnorm Child Psychol*. 2014;42(6):1019-31
- https://clinicaltrials.gov/ct2/show/NCT00050622

Reason for exclusion: Reason for exclusion: Less than seven days treatment; co-treatment with behavioral intervention, except for one arm.

Pelham2016

- Pelham WE, Fabiano GA, Waxmonsky JG, et al. Treatment Sequencing for Childhood ADHD: A Multiple-Randomization Study of Adaptive Medication and Behavioral Interventions. *J Clin Child Adolesc Psychol.* 2016;45(4):396-415.

Reason for exclusion: No design of interest for the present meta-analysis

Pelham2017a

- Pelham WE, Jr., Meichenbaum DL, Smith BH, Sibley MH, Gnagy EM, Bukstein O. Acute Effects of MPH on the Parent-Teen Interactions of Adolescents With ADHD. *J Atten Disord.* 2017;21:158-167.

Reason for exclusion: Single dose. Note: Participants from Evans SW, Pelham WE, Smith BH, et al. Dose-response effects of methylphenidate on ecologically valid measures of academic performance and classroom behavior in adolescents with ADHD. Exp Clin Psychopharmacol. May 2001;9(2):163-175 (excluded) and Smith BH, Pelham WE, Evans S, et al. Dosage effects of methylphenidate on the social behavior of adolescents diagnosed with attention deficit hyperactivity disorder. Exp Clin Psychopharmacol. 1998;6(2):187-204 (excluded)

Pelham2017b

- Pelham WE, Jr., Gnagy EM, Sibley MH, et al. Attributions and Perception of Methylphenidate Effects in Adolescents With ADHD. *J Atten Disord.* 2017; 21(2):129-136.

Reason for exclusion: Less 7 days treatment, no outcomes of interest

Pentikis2002

- Pentikis HS, Simmons RD, Benedict MF, Hatch SJ. Methylphenidate bioavailability in adults when an extended-release multiparticulate formulation is administered sprinkled on food or as an intact capsule. *J Am Acad Child Adolesc Psychiatry.* 2002;41(4):443-449.

Reason for exclusion: Open label

Perez-Alvarez2009

- Perez-Alvarez F, Serra-Amaya C, Timoneda-Gallart CA. Cognitive versus behavioral ADHD phenotype: what is it all about? *Neuropediatrics.* 2009;40(1):32-38.

Reason for exclusion: No arms of interest for the present meta-analysis (medication vs. psychological treatment vs combined)

Perwien2004

- Perwien AR, Faries DE, Kratochvil CJ, Sumner CR, Kelsey DK, Allen AJ. Improvement in health-related quality of life in children with ADHD: an analysis of placebo controlled studies of atomoxetine. *J Dev Behav Pediatr.* 2004;25(4):264-271.

Reason for exclusion: Pooled 3 RCTs (Michelson D, Faries D, Wernicke J, et al. Atomoxetine in the treatment of children and adolescents with attention-deficit/hyperactivity disorder: a randomized, placebo-controlled, dose-response study. Pediatrics. Nov 2001;108(5):E83; Kelsey DK, Sumner CR, Casat CD, et al. Once-daily atomoxetine treatment for children with attention-deficit/hyperactivity disorder, including an assessment of evening and morning behavior: a double-blind, placebo-controlled trial. Pediatrics. Jul 2004;114(1):e1-8; Weiss M, Tannock R, Kratochvil C, et al. A randomized, placebo-controlled study of once-daily atomoxetine in the school setting in children with ADHD. J Am Acad Child Adolesc Psychiatry. Jul 2005;44(7):647-655) all identified in our search.

Pierce2008

- Pierce D, Dixon CM, Wigal SB, McGough JJ. Pharmacokinetics of methylphenidate transdermal system (MTS): results from a laboratory classroom study. *J Child Adolesc Psychopharmacol.* 2008;18(4):355-364.

Reason for exclusion: Transdermal formulation, no oral formulation; prior dose optimization.

Pietrzak2006

- Pietrzak RH, Mollica CM, Maruff P, Snyder PJ. Cognitive effects of immediate-release methylphenidate in children with attention-deficit/hyperactivity disorder (Structured abstract). *Neurosci Biobehav Rev.* 2006(8):1225-1245.

Reason for exclusion: Review

Plenger1996

- Plenger, PM, Dixon, CE, Castillo, RM, Frankowski, RF, Yablon, SA,Levin, HS (1996) Subacute methylphenidate treatment for moder-ate to moderately severe traumatic brain injury: a preliminary double-blind placebo-controlled study. *Arch Phys Med Rehabil.* 77: 536–540

Reason for exclusion: No participants with ADHD

Pliszka1989

- Pliszka SR. Effect of anxiety on cognition, behavior, and stimulant response in ADHD. *J Am Acad Child Adolesc Psychiatry.* 1989;28(6):882-887

- Pliszka SR. Effect of anxiety on cognition, behavior, and stimulant response in ADHD. *Annual Progress in Child Psychiatry and Child Development*. 1990. http://onlinelibrary.wiley.com/o/cochrane/clcentral/articles/147/CN-00212147/frame.html.

Cross-over without wash out: pre-cross over data not available

Pliszka2006

- Pliszka SR, Matthews TL, Braslow KJ, Watson MA. Comparative effects of methylphenidate and mixed salts amphetamine on height and weight in children with attention-deficit/hyperactivity disorder. *J Am Acad Child Adolesc Psychiatry.* 2006;45(5):520-526.

Reason for exclusion: No RCT

Pliszka2007

- Pliszka SR, Liotti M, Bailey BY, Perez R, 3rd, Glahn D, Semrud-Clikeman M. Electrophysiological effects of stimulant treatment on inhibitory control in children with attention-deficit/hyperactivity disorder. *J Child Adolesc Psychopharmacol.* 2007;17(3):356-366.

Cross-over without wash out: pre-cross over data not available

Pliszka2016

- Pliszka SR. A phase 3 registration trial of delayed-release and extended-release methylphenidate (HLD200) in the treatment of early morning functioning impairments in children with attention deficit/ hyperactivity disorder. *J Am Acad Child Adolesc Psychiatry.* 2016;55 (10 Supplement 1):S315.

Reason for exclusion: Presentation of study, not published yet (February 2017)

Poklis1997

- Poklis A. Urinary dextroamphetamine in adult attention deficit/hyperactivity disorder. *J Anal Toxicol.* 1997;21(2):176-177.

Reason for exclusion: No RCT

Pollak2010

- Pollak Y, Shomaly HB, Weiss PL, Rizzo AA, Gross-Tsur V. Methylphenidate effect in children with ADHD can be measured by an ecologically valid continuous performance test embedded in virtual reality. *CNS Spectr.* 2010;15(2):125-130.

Reason for exclusion: Single dose

Pollard1983

- Pollard S. The Effects of Parent Training and Ritalin on the Parent-Child Interactions of Hyperactive Boys. *Child Fam Behav Ther*. 1983(4):51-69.

Reason for exclusion: Three subjects; No design of interest the present meta-analysis (methylphenidate only or methylphenidate + parent training)

Polotskaia2008

- Polotskaia A. *Response of motor and cognitive speed to increasing doses of methylphenidate in children diagnosed with Attention Deficit/Hyperactivity Disorder* [M.Sc.]. Ann Arbor, McGill University (Canada); 2008.

Reason for exclusion: Less than seven days treatment

Poltavski2006

- Poltavski DV, Petros T. Effects of transdermal nicotine on attention in adult non-smokers with and without attentional deficits. *Physiology & Behavior.* 2006;87(3):614-624.

Reason for exclusion: Transdermal formulation, no medication of interest for the present meta-analysis vs. placebo

Porges1981

- Porges SW, Bohrer RE, Keren G, Cheung MN, Franks GJ, Drasgow F. The influence of methylphenidate on spontaneous autonomic activity and behavior in children diagnosed as hyperactive. *Psychophysiology.* 1981;18(1):42-48

Reason for exclusion: No DSM/ICD criteria

Porrino1983

- Porrino LJ, Rapoport JL, Behar D, Ismond DR, Bunney WE, Jr. A naturalistic assessment of the motor activity of hyperactive boys. II. Stimulant drug effects. *Arch Gen Psychiatry.*1983;40(6):688-693.

Reason for exclusion: Not randomized; No DSM/ICD criteria

Potter2004

- Potter AS, Newhouse PA. Acute nicotine administration improves behavioral inhibition in adolescents with attention- deficit/hyperactivity disorder (ADHD). *Society for Neuroscience Abstract Viewer and Itinerary Planner.* 2003;2003:Abstract No. 18.19.
- Potter AS, Newhouse PA. Effects of acute nicotine administration on behavioral inhibition in adolescents with attention-deficit/hyperactivity disorder. *Psychopharmacology (Berl).* 2004;176(2):182-194.

Reason for exclusion: Single dose

Potter2009

- Potter AS, Ryan KK, Newhouse PA. Effects of acute ultra- low dose mecamylamine on cognition in adult attention-deficit/hyperactivity disorder (ADHD). *Hum Psychopharmacol.* 2009;24:309–317.

Reason for exclusion: Medication of no interest for the present meta-analysis vs. placebo, no other arms

Potter2014

- Potter AS, Dunbar G, Mazzulla E, et al. AZD3480, a novel nicotinic receptor agonist, for the treatment of attention-deficit/hyperactivity disorder in adults. *Biol Psychiatry*. 2014;75:207–214.

Reason for exclusion: Medication of no interest for the present meta-analysis vs. placebo, no other arms

Prichep1976

- Prichep LS, Sutton S, Hakerem G. Evoked potentials in hyperkinetic and normal children under certainty and uncertainty: a placebo and methylphenidate study. *Psychophysiology.* 1976;13(5):419-428.

Reason for exclusion: No DSM/ICD criteria. Not randomized

Prince2000

- Prince JB, Wilens TE, Biederman J, et al. A controlled study of nortriptyline in children and adolescents with attention deficit hyperactivity disorder. *J Child Adolesc Psychopharmacol.* 2000;10(3):193-204.

Reason for exclusion: Medication of no interest for the present meta-analysis vs. placebo

Quinn2004

- Quinn D, Wigal S, Swanson J, et al. Comparative pharmacodynamics and plasma concentrations of d-threo-methylphenidate hydrochloride after single doses of d-threo-methylphenidate hydrochloride and d,l-threo-methylphenidate hydrochloride in a double-blind, placebo-controlled, crossover laboratory school study in children with attention-deficit/hyperactivity disorder. *J Am Acad Child Adolesc Psychiatry.* 2004;43(11):1422-1429.

Reason for exclusion: Each arm less than seven consecutive days; subject "responders" to previous ADHD medication.

Quinn2007

- Quinn D, Bode T, Reiz JL, Donnelly GA, Darke AC. Single-dose pharmacokinetics of multilayer-release methylphenidate and immediate-release methylphenidate in children with attention-deficit/hyperactivity disorder. *J Clin Pharmacol.* 2007;47(6):760-766.

Reason for exclusion: Single dose

Quintana1995

- Quintana H, Birmaher B, Stedge D, et al. Use of methylphenidate in the treatment of children with autistic disorder. *J Autism Dev Disord.* 1995;25(3):283-294.
- Quintana H, Birmaher B, Stedge D, Lennon S, et al. Use of methylphenidate in the treatment of children with autistic disorder. *Annual Progress in Child Psychiatry & Child Development.* 1996:295-307.

Reason for exclusion: No ADHD diagnosis

Quintana2005

- Quintana H, Kelsey DK, Cherlin EA, et al. Transition from psychostimulants to atomoxetine in pediatric and adolescent patients with attention-deficit/hyperactivity disorder. *Eur Neuropsychopharmacol.* 2005;15:S632.

Reason for exclusion: No RCT

Rains2004

- Rains A, Scahill L. New long-acting stimulants in children with ADHD. *J Child Adolesc Psychiatr Nurs.* 2004;17(4):177-179.

Reason for exclusion: Review

Rains2006

- Rains A, Scahill L, Hamrin V. Nonstimulant medications for the treatment of ADHD.[Erratum appears in J Child Adolesc Psychiatr Nurs. 2006 May;19(2):96 Note: Hamrin, Vanya [added]]. *J Child Adolesc Psychiatr Nurs.* 2006;19(1):44-47.

Reason for exclusion: Review

Rajesh2006

- Rajesh AS, Bates G, Wright JGC. Atomoxetine-induced electrocardiogram changes. *Arch Dis Child.* 2006;91(12):1023-1024.

Reason for exclusion: Case report

Ramos-Quiroga2007

- Ramos-Quiroga JA, Bosch R, Castells X, Valero S, Nogueira M, Yelmo S, Garcia E, Martinez I, Casas M. A 6 month study of the adherence, effectiveness and safety with methylphenidate adults with ADHD. *Eur Psychiatry.* 2007; 22(S1): 63

Reason for exclusion: No RCT

Ramtvedt2013 (NCT01220440)

- Ramtvedt BE, Roinas E, Aabech HS, Sundet KS. Clinical gains from including both dextroamphetamine and methylphenidate in stimulant trials. *J Child Adolesc Psychopharmacol.* 2013;23(9):597-604.
- Ramtvedt BE, Aabech HS, Sundet K. Minimizing adverse events while maintaining clinical improvement in a pediatric attention-deficit/hyperactivity disorder crossover trial with dextroamphetamine and methylphenidate. *J Child Adolesc Psychopharmacol.* 2014;24(3):130-139.
- Ramtvedt BE, Sandvik L, Sundet K. Correspondence between children's and adults' ratings of stimulant- induced changes in ADHD behaviours in a crossover trial with medication-naive children. *Eur J Dev Psychol.* 2014;11(6):687–700.
- Ramtvedt BE, Sundet K. Relationships between computer- based testing and behavioral ratings in the assessment of attention and activity in a pediatric ADHD stimulant crossover trial. *Clin Neuropsychol.* 2014;28(7): 1146–61.
- https://clinicaltrials.gov/ct2/show/NCT01220440

Cross-over without wash out: pre-cross over data not available

Rapoport1971

- Rapoport J, Abramson A, Alexander D, Lott I. Playroom observations of hyperactive children on medication. *J Am Acad Child Psychiatry.* 1971;10(3):524-534.
- Rapoport JL, Quinn PO, Lamprecht F. Minor physical anomalies and plasma dopamine-beta-hydroxylase activity in hyperactive boys. *Am J Psychiatry.* 1974;131(4):386-390.
- Quinn P, Rapoport JL: Minor physical anomalies and neurologic status in hyperactive boys. *Pediatrics* 1974;53(5):742-7.
- Follow-up in: Quinn PO, Rapoport JL. One-year follow-up of hyperactive boys treated with imipramine or methylphenidate. *Am J Psychiatry* 1975;132(3):241-245.

Reason for exclusion: No DSM/ICD criteria

Rapoport1974

- Rapoport JL, Quinn PO, Bradbard G, Riddle K. Imipramine and methylphenidate treatments of hyperactive boys. *Arch Gen Psychiatry.* 1974;30(6):789-793.
- Rapoport JL, Quinn PO, Lamprecht F. Minor physical anomalies and plasma dopamine-beta-hydroxylase activity in hyperactive boys. *Am J Psychiatry.* 1974;131(4):386-390.
- Quinn P, Rapoport JL: Minor physical anomalies and neurologic status in hyperactive boys. Pediatrics; follow up in : Quinn PO, Rapoport JL. One-year follow-up of hyperactive boys treated with imipramine or methylphenidate. Am J Psychiatry 1975;132(3):241-245.
- Rapoport J, Quinn P, Scribanu N, Murphy DL. Platelet serotonin of hyperactive school age boys. *Br J Psychiatry: J Ment Sci.* 1974;125(0):138-140.

Reason for exclusion: No DSM/ICD criteria

Rapoport1978

- Rapoport JL, Mikkelsen EJ, Ebert MH, Brown GL, Weise VK, Kopin IJ. Urinary catecholamines and amphetamine excretion in hyperactive and normal boys. *J Nerv Ment Dis.* 1978;166(10):731-737.

Reason for exclusion: No DSM/ICD criteria, single dose

Rapoport1980a

- Rapoport JL, Tepsic PN, Grice J, Johnson C, Langer D. Decreased motor activity of hyperactive children on dextroamphetamine during active gym program. *Psychiatry Res.* 1980;2(3):225-229.

Reason for exclusion: Single dose

Rapoport1980b

- Rapoport JL, Buchsbaum MS, Weingartner H, Zahn TP, Ludlow C, Mikkelsen EJ. Dextroamphetamine. Its cognitive and behavioral effects in normal and hyperactive boys and normal men. *Arch Gen Psychiatry.* 1980;37(8):933-943.

Reason for exclusion: No diagnostic criteria as per protocol

Rapoport1982

- Rapoport JL, Nee L, Mitchell S, Polinsky R, Ebert M. Hyperkinetic syndrome and Tourette syndrome. *Adv Neurol.* 1982;35:423-426.

Reason for exclusion: Review/chapter

Rapoport1985a

- Rapoport JL, Zametkin A, Donnelly M, Ismond D. New drug trials in attention deficit disorder. *Psychopharmacol Bull.* 1985;21(2):232-236.

Reason for exclusion: Review

Rappley2005

- Rappley MD. Attention deficit-hyperactivity disorder. *N Engl J Med.* 2005;352(2):165-173.

Reason for exclusion: Review

Rapport1982

- Rapport MD, Murphy H, Bailey JS. Ritalin vs. response cost in the control of hyperactive children: A within-subject comparison. *J Appl Behav Anal.* 1982;15(2):205-216.

Reason for exclusion: No randomised; Two subjects only; less than seven days consecutive treatment

Rapport1985b

- Rapport MD, Stoner G, DuPaul GJ, Birmingham BK, Tucker S. Methylphenidate in hyperactive children: differential effects of dose on academic, learning, and social behavior. *J Abnorm Child Psychol.* 1985;13(2):227-243.
- Rapport MD, DuPaul GJ, Smith NF. Rate-dependency and hyperactivity: methylphenidate effects on operant responding. *Pharmacol Biochem Behav.* 1985;23(1):77-83
- Rapport MD, DuPaul GJ, Stoner G, Birmingham BK, Masse G. Attention deficit disorder with hyperactivity: differential effects of methylphenidate on impulsivity. *Pediatrics.* 1985;76(6):938-943.
- Rapport MD, DuPaul GJ. Hyperactivity and methylphenidate: rate-dependent effects on attention. *Int Clin Psychopharmacol.* 1986;1(1):45-52.
- Rapport MD, DuPaul GJ, Stoner G, Jones TJ. Comparing classroom and clinic measures of attention deficit disorder: differential, idiosyncratic, and dose-response effects of methylphenidate. *J Consult Clin Psychol.* 1986;54(3):334-341.
- Vyse SA, Rapport MD. The effects of methylphenidate on learning in children with ADDH: the stimulus equivalence paradigm. *J Consult Clin Psychol.* 1989;57(3):425-435.

Reason for exclusion: Less than seven consecutive days treatment (6 consecutive days + 1 day for wash out, confirmed by first author).

Rapport1987

- Rapport MD, DuPaul GJ. Methylphenidate: rate-dependent effects on hyperactivity. *Psychopharmacol Bull.* 1986;22(1):223-228.
- Rapport MD, Jones J, DuPaul GJ, et al. Attention deficit disorder and methylphenidate: Group and single-subject analyses of dose effects on attention in clinic and classroom settings. *J Clin Child Psychol.* 1987;16(4):329-338.
- DuPaul GJ, Rapport MD, Vyse SA. ADDH and methylphenidate responders: effects on behavior controlled by complex reinforcement schedules. *Int Clin Psychopharmacol.* 1988;3(4):349-361.
- Kelly KL, Rapport MD, DuPaul GJ. Attention deficit disorder and methylphenidate: a multi-step analysis of dose-response effects on children's cardiovascular functioning. *Int Clin Psychopharmacol.* 1988;3(2):167-81.
- Rapport MD, Stoner G, DuPaul GJ, Kelly KL, Tucker SB, Schoeler T. Attention deficit disorder and methylphenidate: a multilevel analysis of dose-response effects on children’s impulsivity across settings. *J Am Acad Child Adolesc Psychiatry.* 1988;27(1):60–9.

- Rapport MD, DuPaul GJ, Kelly KL. Attention deficit hyperactivity disorder and methylphenidate: the relationship between gross body weight and drug response in children. *Psychopharmacol Bull.* 1989;25(2):285-290.
- Rapport MD, Quinn SO, DuPaul GJ, Quinn EP, Kelly KL. Attention deficit disorder with hyperactivity and methylphenidate: the effects of dose and mastery level on children's learning performance. *J Abnorm Child Psychol.* 1989;17(6):669–89.
- DuPaul GJ, Rapport MD. Does methylphenidate normalize the classroom performance of children with attention deficit disorder? *J Am Acad Child Adolesc Psychiatry.* 1993;32(1):190-198.
- Rapport MD, Denney C, DuPaul GJ, Gardner MJ. Attention deficit disorder and methylphenidate: normalization rates, clinical effectiveness, and response prediction in 76 children. *J Am Acad Child Adolesc Psychiatry.* 1994;33(6):882-893.
- Rapport MD, Loo S, Denney C. The paired associated learning task: Is it an externally valid instrument for assessing methylphenidate response in children with attention deficit disorder? *J Psychopathol Behav Assess.* 1995;17(2):125-144.
- Rapport MD, Denney C. Titrating methylphenidate in children with attention-deficit/hyperactivity disorder: is body mass predictive of clinical response? *J Am Acad Child Adolesc Psychiatry.* 1997;36(4):523-530.
- Denney CB, Rapport MD. Predicting methylphenidate response in children with ADHD: theoretical, empirical, and conceptual models. *J Am Acad Child Adolesc Psychiatry.* 1999;38(4):393-401.
- Rapport MD, Randall R, Moffitt C. Attention-Deficit/Hyperactivity Disorder and methylphenidate: a dose-response analysis and parent-child comparison of somatic complaints. *J Atten Disord.* 2002;6(1):15-24.
- Rapport MD, Kofler MJ, Coiro MM, Raiker JS, Sarver DE, Alderson RM. Unexpected effects of methylphenidate in attention-deficit/hyperactivity disorder reflect decreases in core/secondary symptoms and physical complaints common to all children. *J Child Adolesc Psychopharmacol.* 2008;18(3):237-247.

Reason for exclusion: Less than seven days (6 days) of consecutive treatment.

Rapport1996

- Rapport MD, Loo S, Isaacs P, Goya S, Denney C, Scanlan S. Methylphenidate and attentional training - Comparative effects on behavior and neurocognitive performance in twin girls with attention-deficit/hyperactivity disorder. *Behav Modif.* 1996;20(4):428-450.

Reason for exclusion: No randomised; Two subjects only; less than seven days consecutive treatment

Rashid2007

- Rashid J, Mitelman S. Methylphenidate and somatic hallucinations. *J Am Acad Child Adolesc Psychiatry.* 2007;46(8):945-946.

Reason for exclusion: Case report

Ray2009

- Ray R, Rukstalis M, Jepson C, et al. Effects of atomoxetine on subjective and neurocognitive symptoms of nicotine abstinence. *J Psychopharmacol.* 2009;23(2):168-176.

Reason for exclusion: No participants with ADHD

RBR-8dmcnj

- http://www.ensaiosclinicos.gov.br/rg/RBR-8dmcnj/

Reasons for exclusion: Non pharmacological intervention

RBR-9fqwyw

- http://www.ensaiosclinicos.gov.br/rg/RBR-9fqwyw/

Reasons for exclusion: Non randomized, open label

RBR-39dz5v

- http://www.ensaiosclinicos.gov.br/rg/RBR-39dz5v/

Reasons for exclusion: No participants with ADHD

RBR-64wczh

- http://www.ensaiosclinicos.gov.br/rg/RBR-64wczh/

Reasons for exclusion: No participants with ADHD

Reid1984

- Reid MK, Borkowski JG. Effects of methylphenidate (Ritalin) on information processing in hyperactive children. *J Abnorm Child Psychol.* 1984;12(1):169-185.

Reason for exclusion: Less than seven days treatment

Reimherr1984

- Reimherr FW, Wender PH, Ebert MH, Wood DR. Cerebrospinal fluid homovanillic acid and 5-hydroxy-indoleacetic acid in adults with attention deficit disorder, residual type. *Psychiatry Res.* 1984;11(1):71-78.

Reason for exclusion: Participants started on dietetic intervention before study

Reimherr1986

- Reimherr FW, Wood DR, Wender PH. The use of MK-801, a novel sympathomimetic, in adults with attention deficit disorder, residual type. *Psychopharmacol Bull.* 1986;22(1):237-242.

Reason for exclusion: No double blind RCT

Reinhardt2007

- Reinhardt MC, Benetti L, Victor MM, et al. Is age-at-onset criterion relevant for the response to methylphenidate in attention-deficit/hyperactivity disorder? *J Clin Psychiatry.* 2007;68(7):1109-1116.

Reason for exclusion: No RCT

Remschmidt2005

- Remschmidt H, Hoare P, Ettrich C, et al. Symptom control in children and adolescents with attention-deficit/hyperactivity disorder on switching from immediate-release MPH to OROS MPH Results of a 3-week open-label study. *Eur Child Adolesc Psychiatry.* 2005;14(6):297-304.

Reason for exclusion: Open label

Renaud1997

- Renaud J, Bourassa M, Douglas VI, Pelletier G, Geoffroy G, Robaey P. Methylphenidate and motor organization in children with ADHD [abstract]. *150th Annual Meeting of the* American *Psychiatric Association; 1997 May 17-22; San Diego, CA.* 1997.

Reason for exclusion: Single dose

Rentz2005

- Rentz AM, Matza LS, Secnik K, Swensen A, Revicki DA. Psychometric validation of the child health questionnaire (CHQ) in a sample of children and adolescents with attention-deficit/hyperactivity disorder. *Qual Life Res.* 2005;14(3):719-734.

Reason for exclusion: Secondary data analysis of an open label study

Retz2012 (NCT00730249)

- Retz W, Rosler M, Ose C, et al. Multiscale assessment of treatment efficacy in adults with ADHD: a randomized placebo-controlled, multi-centre study with extended-release methylphenidate. *World J Biol Psychiatry*. 2012;13(1):48-59.
- https://clinicaltrials.gov/ct2/show/NCT00730249

Reason for exclusion: Maximum dose higher than the maximum dose allowed as per our protocol (Ten participants were taking 120 mg/day, confirmed by manufacturer)

Rhodes2006

- Rhodes SM, Thrower M, Brown A, Esperon J, Coghill DR, Matthews K. Acute neuropsychological effects of the psychostimulant Methylphenidate in drug naive boys with Hyperkinetic Disorder (ADHD). *Society for Neuroscience Abstracts*. 2001;27:2341.
- Rhodes SM, Coghill DR, Matthews K. Acute neuropsychological effects of methylphenidate in stimulant drug-naive boys with ADHD II--broader executive and non-executive domains. *J Child Psychol Psychiatry.* 2006;47(11):1184-1194.

Reason for exclusion: Single dose

Riahi2010 (IRCT138905033979N3)

- Riahi F, Tehrani-Doost M, Shahrivar Z, Alaghband-Rad J. Efficacy of reboxetine in adults with attention-deficit/hyperactivity disorder: A randomized, placebo-controlled clinical trial. *Hum Psychopharmacol.* 2010;25(7-8):570-576.
- http://www.irct.ir/searchresult.php?id=3979&number=3

Reason for exclusion: Two arms: placebo and medication of no interest for the present meta-analysis

Riccardelli2004

- Riccardelli RS, Steele MM, Binder C. Effectiveness of Concerta versus usual care methylphenidate immediate release in children with ADHD. 157th Annual Meeting of the American Psychiatric Association; 2004 May 1-6; New York, NY2004.

Reason for exclusion: Open label

Riccio2001

- Riccio CA, Waldrop JJ, Reynolds CR, Lowe P. Effects of stimulants on the continuous performance test (CPT): implications for CPT use and interpretation. *J Neuropsychiatry Clin Neurosci.* 2001;13(3):326-335.

Reason for exclusion: Review

Richmond1978

- Richmond JS, Young JR, Groves JE. Violent dyscontrol responsive to d-amphetamine. *Am J Psychiatry.* 1978;135(3):365-366.

Reason for exclusion: Case report

Ridderinkhof2005

- Ridderinkhof KR, Scheres A, Oosterlaan J, Sergeant JA. Delta plots in the study of individual differences: new tools reveal response inhibition deficits in AD/HD that are eliminated by methylphenidate treatment. *J Abnorm Psychol.* 2005;114(2):197-215.

Reason for exclusion: No RCT

Riddle1995

- Riddle MA, Lynch KA, Scahill L, Devries A, Cohen DJ, Leckman JF. Methylphenidate discontinuation and reinitiation during long-term treatment of children with Tourettes disorder and attention-deficit hyperactivity disorder - a pilot-study. *J Child Adolesc Psychopharmacol.* 1995;5(3):205-214.

Reason for exclusion: No double blind RCT

Rie1976

- Rie HE, Rie ED, Stewart S, Ambuel JP. Effects of methylphenidate on underachieving children. *J Consult Clin Psychol.* 1976;44(2):250-260.

Reason for exclusion: No DSM/ICD criteria

Riggs2004

- Riggs PD, Hall SK, Mikulich-Gilbertson SK, Lohman M, Kayser A. A randomized controlled trial of pemoline for attention-deficit/hyperactivity disorder in substance-abusing adolescents. *J Am Acad Child Adolesc Psychiatry.* 2004;43(4):420-429.

Reason for exclusion: Two arms: placebo and drug of no interest for the present meta-analysis

Riggs2011(NCT00264797)

- Riggs PD, Winhusen T, Davies R, Leimberger J, Mikulich-Gilbertson SK. Los Angeles, CA: American Academy of Addiction Psychiatry Annual Meeting; 2009. A randomized controlled trial of OROS-MPH for ADHD in adolescents with substance use disorders. Los Angeles, CA: American Academy of Addiction Psychiatry Annual Meeting; 2009.
- Riggs PD, Winhusen T, Davies RD, et al. Randomized controlled trial of osmotic-release methylphenidate with cognitive-behavioral therapy in adolescents with attention-deficit/hyperactivity disorder and substance use disorders. *J Am Acad Child Adolesc Psychiatry.* 2011;50(9):903-914.
- Gray KM, Riggs PD, Min SJ, Mikulich-Gilbertson SK, Bandyopadhyay D, Winhusen T. Cigarette and cannabis use trajectories among adolescents in treatment for attention-deficit/hyperactivity disorder and substance use disorders. *Drug Alcohol Depend.* 2011;117(2-3):242-247.
- Pooled in Inhusen TM, Lewis DF, Riggs PD, et al. Subjective effects, misuse, and adverse effects of osmotic-release methylphenidate treatment in adolescent substance abusers with attention-deficit/hyperactivity disorder. *J Child Adolesc Psychopharmacol.* 2011;21(5):455-463.
- Tamm L, Adinoff B, Nakonezny PA, Winhusen T, Riggs P. Attention-deficit/hyperactivity disorder subtypes in adolescents with comorbid substance-use disorder. *Am J Drug Alcohol Abuse.* 2012;38(1):93-100.
- Warden D, Riggs PD, Min SJ, et al. Major depression and treatment response in adolescents with ADHD and substance use disorder. *Drug Alcohol Depend.* 1 2012;120(1-3):214-219.
- Tamm L, Trello-Rishel K, Riggs P, et al. Predictors of treatment response in adolescents with comorbid substance use disorder and attention-deficit/hyperactivity disorder. *J Subst Abuse Treat.* 2013;44(2):224-230.

- McPherson S, Mamey MR, Barbosa-Leiker C, Murphy SM, Roll J. Osmotic-release methylphenidate randomized controlled trial for adolescents with attention-deficit/hyperactivity disorders and substance use disorders: A missing data sensitivity analysis. *Drug Alcohol Depend*. 2015;146:e36.

Reason for exclusion: Add-on CBT for SUD

Ripley2014 (NCT00702364)

- Ripley DL, Morey CE, Gerber D, et al. Atomoxetine for attention deficits following traumatic brain injury: results from a randomized controlled trial. *Brain Inj*. 2014;28(12):1514-1522.
- https://clinicaltrials.gov/ct2/show/NCT00702364

Reason for exclusion: No DSM/ICD criteria

Rivkin2012

- Rivkin A, Alexander RC, Knighton J, et al. A Randomized, Double-Blind, Crossover Comparison of MK-0929 and Placebo in the Treatment of Adults With ADHD. *J Atten Disord.* 2012;16(8):664-674.

Reason for exclusion: Two arms: placebo and drug of no interest for the present meta-analysis

Roca2013

- Roca P, Mulas F, Gandia R, Ortiz-Sanchez P, Abad L. [Executive functioning and evoked potentials P300 pre- and post- treatment in attention deficit hyperactivity disorder]. *Rev Neurol.* 2013;56 (Suppl 1):S107-118.

Reason for exclusion: No RCT

Roesch2013(NCT00919867; SPD503-115)

- Roesch B, Corcoran ME, Fetterolf J, et al. Pharmacokinetics of coadministered guanfacine extended release and lisdexamfetamine dimesylate. *Drugs in R&D.* 2013;13(2):119-128.
- https://clinicaltrials.gov/ct2/show/NCT00919867

Reason for exclusion: No participants with ADHD; no double blind and no controlled

Roman2002

- Roman T, Szobot C, Martins S, Biederman J, Rohde LA, Hutz MH. Dopamine transporter gene and response to methylphenidate in attention-deficit/hyperactivity disorder. *Pharmacogenetics.* 2002;12(6):497-499.

Reason for exclusion: No RCT

Rosch2016

- Rosch KS, Fosco WD, Pelham WE, Jr., Waxmonsky JG, Bubnik MG, Hawk LW, Jr. Reinforcement and Stimulant Medication Ameliorate Deficient Response Inhibition in Children with Attention-Deficit/Hyperactivity Disorder. *J Abnorm Child Psychol.* 2016;44(2):309-321.

Reason for exclusion: No RCT

Ross2006

- Ross RG. Psychotic and manic-like symptoms during stimulant treatment of attention deficit hyperactivity disorder. *Am J Psychiatry.* 2006;163(7):1149-1152.

Reason for exclusion: Case report

Rosse1984

- Rosse RB, Licamele WL. Slow-release methylphenidate: problems when children chew tablets. *J Clin Psychiatry.* 1984;45(12):525.

Reason for exclusion: Case report

Rossel1987

- Rossel E. Sustained attention in hyperkinetic children: A signal detection analysis on the effects of methylphenidate. *Z Kinder Jugendpsychiatr Psychother.* 1987;15(1):6-17.

Reason for exclusion: No DSM/ICD criteria

Rotta1991

- Rotta NT, Guardiola A, Barros HT, Hibig A. Efficacy of imipramine in children with attention deficit hyperactivity disorder. I *Brain Inj*. 1991, 6, 4, 343-346

Reason for exclusion: No randomized, medication of no interest for the present meta-analysis vs. placebo

Rubia2003

- Rubia K, Noorloos J, Smith A, Gunning B, Sergeant J. Motor timing deficits in community and clinical boys with hyperactive behavior: the effect of methylphenidate on motor timing. *J Abnorm Child Psychol.* 2003;31(3):301-313.

Reason for exclusion: No RCT

Rubinsten2008

- Rubinsten O, Bedard A-C, Tannock R. Methylphenidate has differential effects on numerical abilities in ADHD children with and without co-morbid mathematical difficulties. *Open Psychol J.* 2008;1:11–7.

Reason for exclusion: Less than seven days treatment

Rubinstein2006

- Rubinstein S, Malone MA, Roberts W, Logan WJ. Placebo-controlled study examining effects of selegiline in children with attention-deficit/hyperactivity disorder. *J Child Adolesc Psychopharmacol.* 2006;16(4):404-415.

Reason for exclusion: Two arms: placebo and drug of no interest for the present meta-analysis

RUPP2005 (NCT00025779)

- Posey DJ, McDougle CJ, Aman MG, et al. A randomized, double-blind, placebo-controlled, crossover trial of methylphenidate in children with hyperactivity associated with pervasive developmental disorders. *Neuropsychopharmacology.* 2004;29(Suppl. 1):S142-S143.
- RUPP (Research Unit on Pediatric Psychopharmacology). Randomized, controlled, crossover trial of methylphenidate in pervasive developmental disorders with hyperactivity. *Arch Gen Psychiatry*. 2005;62(11):1266-1274
- Posey DJ, Aman MG, McCracken JT, et al. Methylphenidate in pervasive developmental disorders: An analysis of secondary measures. *Neuropsychopharmacology.* 2006;31:S134.
- Posey DJ, Aman MG, McCracken JT, et al. Positive effects of methylphenidate on inattention and hyperactivity in pervasive developmental disorders: an analysis of secondary measures. *Biol Psychiatry.* 2007;61(4):538-544.
- Jahromi LB, Kasari CL, McCracken JT, et al. Positive effects of methylphenidate on social communication and self-regulation in children with pervasive developmental disorders and hyperactivity. *J Autism Dev Disord.* 2009;39(3):395-404.
- Comment in: Sarhangian R, Bearss K, Scahill L. Parent-defined target symptoms in the rupp autism methylphenidate study. *J Investig Med.* 2009;57(3):566.
- McCracken JT, Badashova KK, Posey DJ, et al. Positive effects of methylphenidate on hyperactivity are moderated by monoaminergic gene variants in children with autism spectrum disorders. *Pharmacogenomics J.* 2014;14(3):295-302
- Scahill L, Bearss K, Sarhangian R, et al. Using a Patient-Centered Outcome Measure to Test Methylphenidate Versus Placebo in Children with Autism Spectrum Disorder. *J Child Adolesc Psychopharmacol.* 2016;27:125-131
- *https://clinicaltrials.gov/ct2/show/NCT00025779*

Reason for exclusion: Participants with a primary diagnosis of ASD; no DSM/ICD diagnosis of ADHD

Sadramely2011

- Sadramely MR, Karahmadi M, Azhar M, Koleini N, Farshidfar F. The effect of bupropion in treating attention deficit hyperactivity disorder in 6-17 year old children and adolescents in Isfahan. *Asian J Psychiatr.* 2011;4:S46.

Reason for exclusion: Abstract only, not possible to assess if study meets criteria for the present meta-analysis; no contact for authors

Safer1972

- Safer D, Allen R, Barr E. Depression of growth in hyperactive children on stimulant drugs. *N Engl J Med.* 1972;287(5):217-220.

Reason for exclusion: No RCT

Safer1973a

- Safer DJ, Allen RP. Drug comparison in hyperactive children. *Am J Psychiatry.* 1973;130(8):939-940.

Reason for exclusion: Letter/commentary

Safer1973b

- Safer DJ, Allen RP. Single daily dose methylphenidate in hyperactive children. *Dis Nerv Syst.* 1973;34(6):325-328.

Reason for exclusion: No RCT

Safer1973c

- Safer DJ, Allen RP. Factors influencing the suppressant effects of two stimulant drugs on the growth of hyperactive children. *Pediatrics.* 1973;51(4):660-667.

Reason for exclusion: No RCT

Safer1995

- Safer DJ. Major treatment considerations for attention-deficit hyperactivity disorder. *Curr Probl Pediatr.* 1995;25(4):137-143.

Reason for exclusion: Review

Safren2007

- Safren SA, Duran P, Yovel I, Perlman CA, Sprich S. Medication adherence in psychopharmacologically treated adults with ADHD. *J Atten Disord*. 2007;10(3): 257-260

Reason for exclusion: No RCT

Saguil2012

- Saguil A, Sheridan R. Amphetamines for attention-deficit/hyperactivity disorder in adults. *Am Fam Physician.* 2012;86(5):413-415.

Reason for exclusion: Review

Sainz1966

- Sainz A. Hyperkinetic disease of children: diagnosis and therapy. *Dis Nerv Syst.* 1966;7 Suppl(7):48-50.

Reason for exclusion: No RCT

Sakakihara2013

- Sakakihara Y. More attention to ADHD. *Dev Med Child Neurol.* 2013;55(4):296.

Reason for exclusion: Editorial

Salardini2016 (IRCT201312181556N55)

- Salardini E, Zeinoddini A, Kohi A, et al. Agomelatine as a Treatment for Attention-Deficit/Hyperactivity Disorder in Children and Adolescents: A Double-Blind, Randomized Clinical Trial. *J Child Adolesc Psychopharmacol.* 2016;26(6):513-9

Reason for exclusion: One medication of interest vs. one medication of no interest for the present meta-analysis; no placebo arm

Salehi2010

- Salehi B, Imani R, Mohammadi MR, et al. Ginkgo biloba for attention-deficit/hyperactivity disorder in children and adolescents: a double blind, randomized controlled trial. *Prog Neuropsychopharmacol Biol Psychiatry.* 2010;34(1):76-80.

Reason for exclusion: Methylphenidate vs. drug of non interest for the present meta-analysis; no placebo

Saletu1975

- Saletu B, Saletu M, Simeon J, Viamontes G, Itil TM. Comparative symptomatological and evoked potential studies with d-amphetamine, thioridazine, and placebo in hyperkinetic children. *Biol Psychiatry.* 1975;10(3):253-275.

Reason for exclusion: No DSM/ICD criteria

Sallee2010

- Sallee F, McBurnett K, Wigal T, Lyne A, Youcha S, Rubin J. Twenty-Four-Month Effectiveness of Guanfacine Extended Release in Children and Adolescents Aged 6 to 17 Years with Attention-Deficit/Hyperactivity Disorder. *Biol Psychiatry.* 2010;67:217S-8S.

Reason for exclusion: *Pooled 2 open-label extension trials*

Samuels2006

- Samuels JA, Franco K, Wan F, Sorof JM. Effect of stimulants on 24-h ambulatory blood pressure in children with ADHD: a double-blind, randomized, cross-over trial. *Pediatr Nephrol.* 2006;21(1):92-95.

Reason for exclusion: Each drug condition: 3 days

Sandler2008

- Sandler A, Glesne C, Geller G. Children's and parents' perspectives on open-label use of placebos in the treatment of ADHD. *Child Care Health Dev.* 2008;34(1):111-120.
- Sandler AD, Glesne CE, Bodfish JW. Conditioned placebo dose reduction: a new treatment in attention-deficit hyperactivity disorder? *J Dev Behav Pediatr.* 2010;31(5):369-375.

Reason for exclusion: Author contacted to gather data from the RCT phase; author replied data not available

Sarma1973

- Sarma PS, Falk MA. Drug treatment of hyperactivity in children. *MJ Ill Med J.* 1973;144(2):117-119 passim.

Reason for exclusion: Review

Satterfield1971

- Satterfield JH, Dawson ME. Electrodermal correlates of hyperactivity in children. *Psychophysiology.* 1971;8(2):191-197.

Reason for exclusion: No RCT; placebo: 1 day

Satterfield1972

- Satterfield JH, Cantwell DP, Lesser LI, Podosin RL. Physiological studies of the hyperkinetic child. I. *Am J Psychiatry.* 1972;128(11):1418-1424.

Reason for exclusion: No DSM/ICD criteria

Satterfield1973

- Satterfield JH, Cantwell DP, Saul RE, Lesser LI, Podosin RL. Response to stimulant drug treatment in hyperactive children: prediction from EEG and neurological findings. *J Autism Child Schizophr.* 1973;3(1):36-48.

Reason for exclusion: NO RCT

Satterfield1973

- Satterfield JH, Lesser LI, Saul RE, Cantwell DP. EEG aspects in the diagnosis and treatment of minimal brain dysfunction. *Ann N Y Acad Sci.*1973;205:274-282.

Reason for exclusion: No DSM/ICD (minimal brain dysfunction)

Satterfield1974a

- Satterfield JH, Cantwell DP. Proceedings: CNS function and response to methylphenidate in hyperactive children. *Psychopharmacol Bull.* 1974;10(4):36-37.

Reason for exclusion: Review of studies with no relevant diagnostic criteria

Satterfield1974b

- Satterfield JH, Cantwell DP, Satterfield BT. Pathophysiology of the hyperactive child syndrome. *Arch Gen Psychiatry.* 1974;31(6):839-844.

Reason for exclusion: No empirical study

Satterfield1979

- Satterfield JH, Cantwell DP, Schell A, Blaschke T. Growth of hyperactive children treated with methylphenidate. *Arch Gen Psychiatry.* 1979;36(2):212-217.

Reason for exclusion: No RCT

Satterfield1980

- Satterfield JH, Satterfield BT, Cantwell DP. Multimodality treatment. A two-year evaluation of 61 hyperactive boys. *Arch Gen Psychiatry.* 1980;37(8):915-919.

Reason for exclusion: No RCT

Satterfield1980

- Satterfield JH, Schell AM, Barb SD. Potential risk of prolonged administration of stimulant medication for hyperactive children. *J Dev Behav Pediatr.* 1980;1(3):102-107.

Reason for exclusion: No RCT

Satterfield1987

- Satterfield JH, Satterfield BT, Schell AM. Therapeutic interventions to prevent delinquency in hyperactive boys. *J Am Acad Child Adolesc Psychiatry.* 1987;26(1):56-64.

Reason for exclusion: No RCT

Sawant2004

- Sawant S, Daviss SR. Seizures and prolonged QTc with atomoxetine overdose. *Am J Psychiatry*. 2004;161:757.

Reason for exclusion: Case report

Scahill1994
- Scahill L, Lynch K. The use of methylphenidate in children with attention-deficit hyperactivity disorder. *J Child Adolesc Psychiatr Nurs.* 1994;7(4):44-47.

Reason for exclusion: Review

Scahill1999
- Scahill L, Barloon L, Farkas L. Alpha-2 agonists in the treatment of attention deficit hyperactivity disorder. *J Child Adolesc Psychiatr Nurs.* 1999;12(4):168-173.

Reason for exclusion: Review

Scahill2007
- Scahill L, Pachler M. Treatment of hyperactivity in children with pervasive developmental disorders. *J Child Adolesc Psychiatr Nurs.* 2007;20(1):59-62.

Reason for exclusion: Review

Scahill2015 (NCT01238575)
- Scahill L, McCracken JT, King BH, et al. Extended-Release Guanfacine for Hyperactivity in Children With Autism Spectrum Disorder. *Am J Psychiatry.* 2015;172(12):1197-1206.
- https://clinicaltrials.gov/ct2/show/NCT01238575

Reason for exclusion: No DSM/ICD criteria

Scanlon1970
- Scanlon J. Treatment of hyperkinetic child with dextroamphetamine and ephedrine. *Pediatrics.* 1970;46(6):975-976.

Reason for exclusion: Case report

Schachar1997
- Schachar RJ, Tannock R, Cunningham C, Corkum PV. Behavioral, situational, and temporal effects of treatment of ADHD with methylphenidate. *J Am Acad Child Adolesc Psychiatry.* 1997;36(6):754-763.
- Diamond IR, Tannock R, Schachar RJ. Response to methylphenidate in children with ADHD and comorbid anxiety. *J Am Acad Child Adolesc Psychiatry.* 1999;38(4):402-409.
- Law SF, Schachar RJ. Do typical clinical doses of methylphenidate cause tics in children treated for attention-deficit hyperactivity disorder? *J Am Acad Child Adolesc Psychiatry.* 1999;38(8):944-951.
- Summarized in: Killeen MR. Do typical clinical doses of methylphenidate cause tics in children treated for attention-deficit hyperactivity disorder? *J Child Fam Nurs.* 2000;3(1):46-48.
- Follow up in: Charach A, Ickowicz A, Schachar R. Stimulant treatment over five years: adherence, effectiveness, and adverse effects. *J Am Acad Child Adolesc Psychiatry.* 2004;43(5):559-567.
- Charach A, Figueroa M, Chen S, Ickowicz A, Schachar R. Stimulant treatment over 5 years: effects on growth. *J Am Acad Child Adolesc Psychiatry.* 2006;45(4):415-421.

Reason for exclusion: Co-intervention: parent training

Schachar2008
- Schachar R, Ickowicz A, Crosbie J, et al. Cognitive and behavioral effects of multilayer-release methylphenidate in the treatment of children with attention-deficit/hyperactivity disorder. *J Child Adolesc Psychopharmacol.* 2008;18(1):11-24.

Reason for exclusion: Unclear if participants were responders to methylphenidate prior to the trail. Written to first author who could not address this query; no reply from manufacturer.

Schaeuble2010a
- Schaeuble B, Hofecker M, Buitelaar J, Kooij S, Dejonckheere J, Waechter S. Longterm safety and efficacy outcomes in adults with ADHD treated with prolonged-release methylphenidate. *Eur Neuropsychopharmacol.* 2010;20(Suppl. 3):S249.

Reason for exclusion: Open label

Schaeuble2010b
- Schaeuble B, Alfred A, Lindermueller A, Dichter S, Mattejat F. Improvement in social functioning and decrease in burden of disease in adolescents with ADHD after switching onto OROS MPH, and their care givers. *Eur J Neurol.* 2010;17:469.

Reason for exclusion: Pooled two open label studies

Schain1975

- Schain RJ, Reynard CL. Observations on effects of a central stimulant drug (methylphenidate) in children with hyperactive behavior. *Pediatrics.* 1975;55(5):709-716.

Reason for exclusion: No DSM/ICD criteria

Schaller1997

Schaller JL, Behar D. Selegiline for the delivery of small doses of amphetamine. *J Neuropsychiatry Clin Neurosci.* 1997;9(2):301-302.

Reason for exclusion: Case report

Schaller1999a

- Schaller JL, Behar D. Carbamazepine and methylphenidate in ADHD. *J Am Acad Child Adolesc Psychiatry.* 1999;38(2):112-113.

Reason for exclusion: Case report

Schaller1999b

- Schaller JL, Behar D. Treating comorbid ADHD, major depression, and panic. *J Neuropsychtry Clin Neurosci. 1999;11(4):516.*
  Reason for exclusion: Case report

Schauble2007

- Schauble B, Mattejat F, Hargarter L. Changes in quality of life after transition from immediate release methylphenidate (IR-MPH) to control led-release MPH in patients with ADHD. *Eur Neuropsychopharmacol.* 2007;17:S575.

Reason for exclusion: Interim analysis of an open label study

Scheffer2005

- Scheffer RE, Kowatch RA, Carmody T, Rush AJ. Randomized, placebo-controlled trial of mixed amphetamine salts for symptoms of comorbid ADHD in pediatric bipolar disorder after mood stabilization with divalproex sodium. *Am J Psychiatry.* 2005;162(1):58-64.

Reason for exclusion: Amphetamine added to divalproex sodium

Schell1986

- Schell RM, Pelham WE, Bender ME. The concurrent assessment of behavioral and psychostimulant interventions: A controlled case study. *Behav Assess.* 1986;8(4):373-384.

Reason for exclusion: Case report

Scheres2003

- Scheres A, Oosterlaan J, Swanson J, et al. The effect of methylphenidate on three forms of response inhibition in boys with AD/HD. *J Abnorm Child Psychol.* 2003;31(1):105-120.
- Castellanos FX, Sonuga-Barke EJ, Scheres A, Di Martino A, Hyde C, Walters JR. Varieties of attention-deficit/hyperactivity disorder-related intra-individual variability. *Biol Psychiatry.* 2005;57(11):1416-1423.

Reason for exclusion: Pseudo-randomised

Scheres2006

- Scheres A, Oosterlaan J, Sergeant JA. Speed of inhibition predicts teacher-rated medication response in boys with attention deficit hyperactivity disorder. *Int J Disab, Devel Educ.* 2006;53(1):93-109.

Reason for exclusion: Pseudo-randomised

Schlander2008

- Schlander M, Hjelmgren J. Cost-effectiveness of long-acting methylphenidate for treatment of attention-deficit/hyperactivity disorder (ADHD) in children and adolescents in Finland: an evaluation based upon a randomized clinical trial (RCT). *Value Health.* 2008;11(6):A339-A340.

Reason for exclusion: No double blind

Schleifer1975

- Schleifer M, Weiss G, Cohen N, Elman M, Cvejic H, Kruger E. Hyperactivity in preschoolers and the effect of methylphenidate. *Am J Orthopsychiatry.* 1975;45(1):38-50.

Reason for exclusion: All participants: preschoolers; no DSM/ICD criteria

Schlochtermeier2011

- Schlochtermeier L, Stoy M, Schlagenhauf F, et al. Childhood methylphenidate treatment of ADHD and response to affective stimuli. *Eur Neuropsychopharmacol.* 2011;21(8):646-654.

Reason for exclusion: No RCT

Schlosser2009

- Schlosser RGM, Nenadic I, Wagner G, Zysset S, Koch K, Sauer H. Dopaminergic Modulation of Brain Systems Subserving Decision Making Under Uncertainty: A Study With fMRI and Methylphenidate Challenge. *Synapse.* 2009;63(5):429-442.

Reason for exclusion: Single dose

Schmidt1987

- Schmidt K, Kappraff MS. Diminished effectiveness of methylphenidate on cognitive tasks in attention deficit disorder with hyperactivity. *J Clin Psychopharmacol.* 1987;7(3):204-205.

Reason for exclusion: No RCT

Schmidt1997

- Schmidt MH, Mocks P, Lay B, et al. Does oligoantigenic diet influence hyperactive/conduct-disordered children - A controlled trial. *Eur Child Adolesc Psychiatry.* 1997;6(2):88-95.

Reason for exclusion: No arms of interest (diet vs oligogenic diet) for the present meta-analysis

Schnackenberg1971

- Schnackenberg RC, Bender EP. The effect of methylphenidate hydrochloride on children with minimal brain dysfunction syndrome and subsequent hyperkinetic syndrome. *Psychiatr Forum.* 1971;2(2):32-36.

Reason for exclusion: No DSM/ICD criteria

Schnackenberg1973

- Schnackenberg RC. Caffeine as a substitute for Schedule II stimulants in hyperkinetic children. *Am J Psychiatry.* 1973;130(7):796-798.

Reason for exclusion: No RCT

Schneider2011

- Schneider MKF, Retz W, Gougleris G, Verhoeven WMA, Tulen JHM, Rosler M. Effects of long-acting methylphenidate in adults with attention deficit hyperactivity disorder: a study with paired-pulse transcranial magnetic stimulation. *Neuropsychobiology.* 2011;64(4):195-201.

Reason for exclusion: No RCT

Schnipper2001

- Schnipper, E. Evaluation of participant use and efficacy of an OROS formulation of methylphenidate HCl in Children with ADHD in a community setting. *Pediatr Res*. 2001; 49, 429A

Reason for exclusion: Open label

Schochat2002

- Schochat E, Scheuer CI, de Andrade ER. ABR and auditory P300 findings in children with ADHD. *Arq Neuropsiquiatr.* 2002;60(3B):742-747

Reason for exclusion: No mention of randomization the text; author contacted to clarify but no reply

Schoenberg2014

- Schoenberg PLA, Hepark S, Kan CC, Barendregt HP, Buitelaar JK, Speckens AEM. Effects of mindfulness-based cognitive therapy on neurophysiological correlates of performance monitoring in adult attention-deficit/hyperactivity disorder. *Clin Neurophysiol.* 2014;125(7):1407-1416.

Reason for exclusion: No arms of interest for the present meta-analysis

Schubiner2002

- Downey KK, Sclrubiner H, Schuster CR. Double-blind placebo controlled stimulant trial for cocaine dependent adhd adults. *NIDA Res Monogr*. 2000:116.
- Schubiner H, Saules KK, Arfken CL, et al. Double-blind placebo-controlled trial of methylphenidate in the treatment of adult ADHD patients with comorbid cocaine dependence. *Exp Clin Psychopharmacol.* 2002;10(3):286-294.

Reason for exclusion: Co-intervention (CBT)

Schulz2010

- Schulz E, Fleischhaker C, Hennighausen K, et al. A randomized, rater-blinded, crossover study comparing the clinical efficacy of ritalin la (methylphenidate) treatment in children with attention-deficit hyperactivity disorder under different breakfast conditions over 2 weeks. *Atten Defic Hyperact Disord.* 2010;(3):133-138.

Reason for exclusion: Open label

Schulz2010 (NCT00254878; CRIT124DDE01)

- Schulz E, Fleischhaker C, Hennighausen K, et al. A double-blind, randomized, placebo/active controlled crossover evaluation of the efficacy and safety of Ritalin (R) LA in children with attention-deficit/hyperactivity disorder in a laboratory classroom setting. *J Child Adolesc Psychopharmacol.* 2010;20(5):377-385.
- https://clinicaltrials.gov/ct2/show/NCT00254878

Reason for exclusion: Participants were responders to methylphenidate

Schulz-Juergensen2014

- Schulz-Juergensen S, Thiemann A, Gebhardt J, Baumgarten-Walczak A, Eggert P. Prepulse inhibition of acoustic startle and the influence of methylphenidate in children with ADHD. *J Atten Disord.* 2014;18(2):117-122.

Reason for exclusion: Placebo arm: two days

Schvehla1994

- Schvehla TJ, Mandoki MW, Sumner GS. Clonidine therapy for comorbid attention deficit hyperactivity disorder and conduct disorder: preliminary findings in a children's inpatient unit. *South Med J.* 1994;87(7):692-695.

Reason for exclusion: No RCT

Schwartz1971

- Schwartz ML, Pizzo SV, McKee PA. Minimal brain dysfunction and methylphenidate. *N Engl J Med.* 1971;285(5):293.

Reason for exclusion: Letter to the editor, no empirical data

Schwarz2005

- Schwarz R, Muskalla B. [How safe are ADHD drugs?]. *Kinderkrankenschwester.* 2005;24(10):437.

Reason for exclusion: Commentary

Schwean1993

- Schwean VL, Saklofske DH, Yackulic RA, Quinn D. WISC-III PERFORMANCE OF ADHD CHILDREN. *J Psychoeduc Assess.* 1993:56-70.

Reason for exclusion: Less than seven days treatment

Schweitzer2003

- Schweitzer JB, Lee DO, Hanford RB, et al. A positron emission tomography study of methylphenidate in adults with ADHD: alterations in resting blood flow and predicting treatment response. *Neuropsychopharmacology.* 2003;28(5):967-973.

Reason for exclusion: No RCT

Schweitzer2004

- Schweitzer JB, Lee DO, Hanford RB, et al. Effect of methylphenidate on executive functioning in adults with attention-deficit/hyperactivity disorder: normalization of behavior but not related brain activity. *Biol Psychiatry.* 2004;56(8):597-606.

Reason for exclusion: No RCT

Sebrechts1986

- Sebrechts MM, Shaywitz SE, Shaywitz BA, Jatlow P, Anderson GM, Cohen DJ. Components of attention, methylphenidate dosage, and blood levels in children with attention deficit disorder. *Pediatrics.* 1986;77(2):222-228.

Reason for exclusion: No randomized

Seger1974

- Seger EY, Hallum G. Methylphenidate in children with minimal brain dysfunction: effects on attention span, visual-motor skills, and behavior. *Curr Ther Res Clin Exp.* 1974;16(6):635-641.

Reason for exclusion: No DSM/ICD criteria

Segev2016

- Segev A, Gvirts HZ, Strouse K, et al. A possible effect of methylphenidate on state anxiety: A single dose, placebo controlled, crossover study in a control group. *Psychiatry Res.* 2016;241:232-235.

Reason for exclusion: No ADHD

Seifert2003

- Seifert J, Scheuerpflug P, Zillessen KE, Fallgatter A, Warnke A. Electrophysiological investigation of the effectiveness of methylphenidate in children with and without ADHD. *J Neural Transm.* 2003;110(7):821-829.

Reason for exclusion: No RCT

Seo2010

- Seo WS, Koo BH, Lee KH, Kim KK, Park HK. Changes of sleep parameters after taking methylphenidate in children with adhd. 163rd Annual Meeting of the American Psychiatric Association; 2010 May 22-26; New Orleans, LA2010.

Reason for exclusion: Participants randomized to two formulations of methylphenidate, no placebo arm, no other drugs of interest for the present meta-analysis

Sevak2010

- Sevak RJ, Stoops WW, Rush CR. Behavioral effects of d-amphetamine in humans: influence of subclinical levels of inattention and hyperactivity. *Am J Drug Alcohol Abuse.* 2010;36(4):220-227.

Reason for exclusion: No RCT

Shafrin2014

- Spencer T, Heiligenstein JH, Biederman J, Faries DE, Kratochvil CJ, Conners CK, Potter WZ. Results from 2 proof-of-concept, placebo-controlled studies of atomoxetine in children with attention-deficit/hyperactivity disorder. *J Clin Psychiatry.* 2002;63: 1140–1147
- Michelson D, Faries D, Wernicke J, et al. Atomoxetine in the treatment of children and adolescents with attention-deficit/hyperactivity disorder: a randomized, placebo-controlled, dose-response study. *Pediatrics.* 2001;108(5):E83.
- Spencer T, Biederman J, Coffey B, et al. A double-blind comparison of desipramine and placebo in children and adolescents with chronic tic disorder and comorbid attention-deficit/hyperactivity disorder. *Arch Gen Psychiatry.* 2002;59(7):649-656.
- Michelson D, Allen AJ, Busner J, et al. Once-daily atomoxetine treatment for children and adolescents with attention deficit hyperactivity disorder: a randomized, placebo-controlled study. *Am J Psychiatry.* 2002;159(11):1896-1901.
- Sallee FR, McGough J, Wigal T, Donahue J, Lyne A, Biederman J. Guanfacine extended release in children and adolescents with attention-deficit/hyperactivity disorder: a placebo-controlled trial. *J Am Acad Child Adolesc Psychiatry.* 2009;48(2):155-165.
- Connor DF, Findling RL, Kollins SH, et al. Effects of guanfacine extended release on oppositional symptoms in children aged 612 years with attention-deficit hyperactivity disorder and oppositional symptoms: A randomized, double-blind, placebo-controlled trial. *CNS Drugs.* 2010;24(9):755-768.
- Shafrin J, Sikirica V, Shrestha A, Henkhaus LE, Erder MH, Chandra A. Methodological assessment of matching-adjusted indirect comparisons: Case study application to attention deficit/hyperactivity disorder (ADHD). *Value Health.* 2014;17 (7):A579.

Reason for exclusion: Not original investigation; all trials included in this papers have been included in the present systematic review.

Shafrin2016

- Michelson D, Faries D, Wernicke J, Kelsey D, Kendrick K, Sallee FR, Spencer T. Atomoxetine in the treatment of children and adolescents with attention-deficit/hyperactivity disorder: a randomized, placebo-controlled, dose-response study. *Pediatrics*. 2001; 108: E83
- Michelson D, Allen AJ, Busner J, et al. Once-daily atomoxetine treatment for children and adolescents with attention deficit hyperactivity disorder: a randomized, placebo-controlled study. *Am J Psychiatry.* 2002;159(11):1896-1901.
- Spencer T, Heiligenstein JH, Biederman J, et al. Results from 2 proof-of-concept, placebo-controlled studies of atomoxetine in children with attention-deficit/hyperactivity disorder. *The J Clin Psychiatry.* 2002;63(12):1140-1147.
- Biederman J, Melmed RD, Patel A, et al. A randomized, double-blind, placebo-controlled study of guanfacine extended release in children and adolescents with attention-deficit/hyperactivity disorder. *Pediatrics.* 2008;121(1):e73-84.

- Sallee FR, Kollins SH, Wigal TL. Efficacy of guanfacine extended release in the treatment of combined and inattentive only subtypes of attention-deficit/hyperactivity disorder. *J Child Adolesc Psychopharmacol.* 2012;22(3):206-214.
- Shafrin J, Shrestha A, Chandra A, Erder MH, Sikirica V. Evaluating Matching-Adjusted Indirect Comparisons in Practice: A Case Study of Patients with Attention-Deficit/Hyperactivity Disorder. *Health economics.* 2016.

Reason for exclusion: Pooled the previous studies, all retrieved in our search

Shafritz2004

- Shafritz KM, Marchione KE, Gore JC, Shaywitz SE, Shaywitz BA. The neural correlates of adhd and the effects of methylphenidate on tasks of selective and divided attention: an fMRI study. *Society for Neuroscience Abstract Viewer and Itinerary Planner.* 2002;Abstract No. 804.807.
- Shafritz KM, Marchione KE, Gore JC, Shaywitz SE, Shaywitz BA. The effects of methylphenidate on neural systems of attention in attention deficit hyperactivity disorder. *Am J Psychiatry.* 2004;161(11):1990-1997.

Reason for exclusion: Single dose

Shah2014

- Shah B, Penaloza J, Medina M. Bupropion for the treatment of attention-deficit/hyperactivity disorder in children and adolescents. *J Am Pharm Assoc.* 2014;54 (2):e131-e132.

Reason for exclusion: Refers to three RCTs; abstract only; not possible to contact authors to enquire about the three RCTs

Shahrbabaki2012

- Shahrbabaki ME, Sabzevari L, Haghdoost AA, Davari-Ashtiani R. Buspirone versus methylphenidate in treatment of children with adhd: A randomized double blinded cross-over study of buspirone versus methylphenidate in the treatment of 6-16 year-old children with attention deficit /hyperactivity disorder. *Iran J Psychiatry.* 2012;(1):23.

Reason for exclusion: Drug of interest vs. drug of non interest for the present meta-analysis

Shahrbabaki2013

- Shahrbabaki ME, Sabzevari L, Haghdoost A, Ashtiani RD. A randomized double blind crossover study on the effectiveness of buspirone and methylphenidate in treatment of attention deficit/hyperactivity disorder in children and adolescents. *Iran J Psychiatry and Clinical Psychology.* 2013;18(4):292-297.

Reason for exclusion: Methylphenidate vs buspiron (the latter not of interest for the present meta-analysis). Iranian colleague confirmed the study did not include a placebo arm

Shakibaei2015

- Shakibaei F, Radmanesh M, Salari E, Mahaki B. Ginkgo biloba in the treatment of attention-deficit/hyperactivity disorder in children and adolescents. A randomized, placebo-controlled, trial. *Complement Ther Clin Pract.* 2015;21(2):61-67.

Reason for exclusion: Methylphenidate+G.Bilboa vs. methylphenidate +placebo

Shang2015

- Shang CY, Pan YL, Lin HY, Huang LW, Gau SS. An Open-Label, Randomized Trial of Methylphenidate and Atomoxetine Treatment in Children with Attention-Deficit/Hyperactivity Disorder. *J Child Adolesc Psychopharmacol.* 2015;25(7):566-573.

Reason for exclusion: Open label

Shang2016

- Shang CY, Yan CG, Lin HY, Tseng WY, Castellanos FX, Gau SS. Differential effects of methylphenidate and atomoxetine on intrinsic brain activity in children with attention deficit hyperactivity disorder. *Psychol Med.* 2016 ;46(15):3173-3185

Reason for exclusion: No double blind (confirmed by one of the study authors)

Shanmugan2017

- Shanmugan S, Loughead J, Nanga RPR, et al. Lisdexamfetamine Effects on Executive Activation and Neurochemistry in Menopausal Women with Executive Function Difficulties. *Neuropsychopharmacology.* 2017;42:437-445.

Reason for exclusion: No participants with ADHD

Sharma2014

- Sharma V, Kim J-W, Ryan N. Predicting Side Effects of Methylphenidate in ADHD : A Machine Learning Approach. *Biol Psychiatry.* 2014;75:122S-3S.

Reason for exclusion: Open label

Shaywitz1982a
- Shaywitz SE, Sebrects MM, Jatlow P, et al. Plasma methylphenidate levels predict attention and activity results in a double-blind placebo study. *Pediatr Res*. 1982;16:93A.

Reason for exclusion: No RCT

Shaywitz1982b
- Shaywitz SE, Hunt RD, Jatlow P, et al. Psychopharmacology of attention deficit disorder: pharmacokinetic, neuroendocrine, and behavioral measures following acute and chronic treatment with methylphenidate. *Pediatrics.* 1982;69(6):688-694.

Reason for exclusion: No RCT

Shaywitz1990
- Shaywitz BA, Shaywitz SE, Sebrechts MM, et al. Growth hormone and prolactin response to methylphenidate in children with attention deficit disorder. *Life Sci.* 1990;46(9):625-633.

Reason for exclusion: No RCT; No DSM/ICD diagnostic criteria

Shaywitz2014
- Shaywitz BA, Williams DW, Fox BK, Wietecha LA. Reading outcomes of children and adolescents with attention-deficit/hyperactivity disorder and dyslexia following atomoxetine treatment. *J Child Adolesc Psychopharmacol.* 2014;24(8):419-425.

Reason for exclusion: Open label

Shea1982
- Shea VT. State-dependent learning in children receiving methylphenidate. *Psychopharmacology (Berl).* 1982;78(3):266-270.

Reason for exclusion: Single dose

Shekim1977
- Shekim WO, Dekirmenjian H, Chapel JL. Urinary catecholamine metabolites in hyperkinetic boys treated with d-amphetamine. *Am J Psychiatry.* 1977;134(11):1276-1279.

Reason for exclusion: No RCT; DSM-II criteria

Shekim1979a
- Shekim WO, Dekirmenjian H, Chapel JL, Javaid J, Davis JM. Norepinephrine metabolism and clinical response to dextroamphetamine in hyperactive boys. *J Pediatr.* 1979;95(3):389-394.

Reason for exclusion: No RCT

Shekim1979b
- Shekim WO, Dekirmenjian H, Chapel JL. Urinary MHPG excretion in minimal brain dysfunction and its modification by d-amphetamine. *Am J Psychiatry.* 1979;136(5):667-671.

Reason for exclusion: No RCT; DSM-II criteria

Shekim1982b
- Shekim WO, Davis LG, Bylund DB, Brunngraber E, Fikes L, Lanham J. Platelet MAO in children with attention deficit disorder and hyperactivity: A pilot study. *Am J Psychiatry.* 1982;139(7):936-938.

Reason for exclusion: No RCT

Shekim1982c
- Shekim WO, Dekirmenjian H, Javaid J, Bylund DB, Davis JM. Dopamine-norepinephrine interaction in hyperactive boys treated with d-amphetamine. *J Pediatr.* 1982;100(5):830-834.

Reason for exclusion: No RCT

Shekim1983
- Shekim WO, Javaid J, Davis JM, Bylund DB. Urinary MHPG and HVA excretion in boys with attention deficit disorder and hyperactivity treated with d-amphetamine. *Biol Psychiatry.* 1983;18(6):707-714.

Reason for exclusion: No RCT

Shekim1986

- Shekim WO, Bylund DB, Alexson J, et al. Platelet MAO and measures of attention and impulsivity in boys with attention deficit disorder and hyperactivity. *Psychiatry Res.* 1986;18(2):179-188.

Reason for exclusion: Diet just before the study, no wash out, so not possible to rule out effect of diet on behaviour

Shekim1994

- Shekim WO, Bylund DB, Hodges K, Glaser R, Ray-Prenger C, Oetting G. Platelet alpha-2-adrenergic receptor binding and the effects of d-amphetamine in boys with attention deficit hyperactivity disorder. *Neuropsychobiology.* 1994;29(3):120-124.

Reason for exclusion: Diet just before the study, no wash out, so not possible to rule out effect of diet on behaviour, no pre-cross over data

Sheppard1999

- Sheppard DM, Bradshaw JL, Mattingley JB, Lee P. Effects of stimulant medication on the lateralisation of line bisection judgements of children with attention deficit hyperactivity disorder. [Erratum appears in *J Neurol Neurosurg Psychiatry*. 2000;68(2):256]. *J Neurol Neurosurg Psychiatry.* 1999;66(1):57-63.

Reason for exclusion: No RCT

Shetty1976

- Shetty T, Chase TN. Central monoamines and hyperkinase of childhood. *Neurology.* 1976;26(10):1000-1002.

Reason for exclusion: No DSM/ICD criteria

Shiels2009

- Shiels K, Hawk LW, Jr., Reynolds B, et al. Effects of methylphenidate on discounting of delayed rewards in attention deficit/hyperactivity disorder. *Exp Clin Psychopharmacol.* 2009;17(5):291-301.
- Spencer SV, Hawk LW, Jr., Richards JB, Shiels K, Pelham WE, Jr., Waxmonsky JG. Stimulant treatment reduces lapses in attention among children with ADHD: the effects of methylphenidate on intra-individual response time distributions. *J Abnorm Child Psychol.* 2009;37(6):805-816.

Reason for exclusion: Less than seven days treatment

Short2004

- Short EJ, Manos MJ, Findling RL, Schubel EA. A prospective study of stimulant response in preschool children: insights from ROC analyses. *J Am Acad Child Adolesc Psychiatry.* 2004;43(3):251-259.

Reason for exclusion: Preschoolers

Shouse1978

- Shouse MN, Lubar JF. Physiological basis of hyperkinesis treated with methylphenidate. *Pediatrics.* 1978;62(3):343-351.

Reason for exclusion: No RCT

Shouse1979

- Shouse MN. Operant conditioning of EEG rhythms and ritalin in the treatment of hyperkinesis. *Biofeedback Self Regul.* 1979;4(4):299-312.

Reason for exclusion: No RCT

Shram2012 (NCT01118702)

- Shram MJ, Quinn AM, Chen N, et al. Differences in the In Vitro and In Vivo Pharmacokinetic Profiles of Once-Daily Modified-Release Methylphenidate Formulations in Canada: Examination of Current Bioequivalence Criteria. *Clin Ther.* 2012;34(5):1170-1181.
- https://clinicaltrials.gov/ct2/show/NCT01118702

Reason for exclusion: No participants with ADHD, no double blind

Shytle2002

- Shytle RD, Silver AA, Wilkinson BJ, Sanberg, P.R. A pilot controlled trial of transdermal nicotine in the treatment of attention deficit hyperactivity disorder. *World J Biol Psychiatry*. 2002;3, 150-155.

Reason for exclusion: Transdermal formulation of a medication of no interest for the present meta-analysis vs. placebo

Signorovitch2012

- Signorovitch J, Erder MH, Xie J, et al. Comparative effectiveness research using matching-adjusted indirect comparison: an application to treatment with guanfacine extended release or atomoxetine in children with attention-deficit/hyperactivity disorder and comorbid oppositional defiant disorder. *Pharmacoepidemiol Drug Saf.* 2012;21 (Suppl 2):130-137.

Reason for exclusion: Pooled trials, all retrieved in our search

Silva2004

- Silva RR, Brams M, Childress A, Lopez FA, Pestreich L, Muniz R. Comparison of long-acting methylphenidate formulations in children with ADHD: Pooled analysis of 2 randomized, placebo-controlled studies. *J Child Adolesc Psychopharmacol.* 2004;14(4):514-515.

Reason for exclusion: Two single blind studies

Silva2005a

- Silva R, Muniz R, Pestreich LK, Brams M, Childress A, Lopez FA. Efficacy of two long-acting methylphenidate formulations in children with attention- deficit/hyperactivity disorder in a laboratory classroom setting. *J Child Adolesc Psychopharmacol.* 2005;15(4):637-654.

Reason for exclusion: Single dose

Silva2005b

- Silva RR, Muniz R, Pestreich L, et al. Once-daily dexmethylphenidate: A placebo-controlled crossover study in children with attention-deficit/hyperactivity disorder. *Ann Neurol*. 2005;58:S109-S.

Reason for exclusion: Subjects optimized to previous treatment

Silva2006

- Silva RR, Muniz R, Pestreich L, et al. Efficacy and duration of effect of extended-release dexmethylphenidate versus placebo in schoolchildren with attention-deficit/hyperactivity disorder. *J Child Adolesc Psychopharmacol.* 2006;16(3):239-251.

Reason for exclusion: Less than seven days treatment

Silva2007

- Silva R, Muniz R, McCague K. Efficacy of extended-release dexmethylphenidate compared with D,L-methylphenidate and placebo in boys and girls with ADHD: A combined analysis of two 12-hour laboratory classroom studies. *J Child Adolesc Psychopharmacol.* 2007;17(6):884-884.

Reason for exclusion: Pooled 2 studies in which participants were responders to previous treatment with methylphenidate

Silva2008a (NCT00141063; CRIT124EUS13)

- Silva R, Muniz R, McCague K, Childress A, Brams M, Mao A. Treatment of children with attention-deficit/hyperactivity disorder: results of a randomized, multicenter, double-blind, crossover study of extended-release dexmethylphenidate and D,L-methylphenidate and placebo in a laboratory classroom setting. *Psychopharmacol Bull.* 2008;41(1):19-33.
- https://clinicaltrials.gov/ct2/show/NCT00141063

Reason for exclusion: "Stabilized" participants at baseline. First author confirmed that "stabilized" meant "responders"

Silva2008b

- Silva RR, Muniz R, Pestreich L, et al. Dexmethylphenidate extended-release capsules in children with attention-deficit/hyperactivity disorder. *J Am Acad Child Adolesc Psychiatry*. 2008;47(2):199-208.

Reason for exclusion: "Stabilized" participants at baseline. First author confirmed that "stabilized" meant "responders"

Silverman2014

- Silverman L, Hollway JA, Smith T, et al. A multisite trial of atomoxetine and parent training in children with autism spectrum disorders: Rationale and design challenges. *Res Autism Spectr Disord.* 2014;8(7):899-907.

Reason for exclusion: No empirical data

Simpson1980

- Simpson RL, Reece CA, Kauffman R, Jones F. Stimulant medications and the classroom attention-to-task and deviant social behaviors of twelve hyperactive males. *Learn Disabil Q.* 1980;3(1):19-27.

- Kauffman RE, Smith-Wright D, Reese CA, Simpson R, Jones F. Medication compliance in hyperactive children. *Pediatric pharmacology (New York, N.Y.).* 1981;1(3):231-237.

Reason for exclusion: No DSM/ICD criteria

Simpson2004

- Simpson A, Kratochvil CJ, Newcorn JH, et al. Efficacy of atomoxetine in placebo-controlled studies in children, adolescents, and adults with attention-deficit/hyperactivity disorder. *Int J Neuropsychopharmacol.* 2004;7:S441.

Reason for exclusion: Pooled studies by Lilly; Lilly confirmed our research retrieved all their studies

Singh1979

- Singh V, Ling GM. Amphetamines in the management of children's hyperkinesis. *Bull Narc.* 1979;31(3-4):87-94.

Reason for exclusion: Review

Slama2015

- Slama H, Fery P, Verheulpen D, Vanzeveren N, Van Bogaert P. Cognitive Improvement of Attention and Inhibition in the Late Afternoon in Children With Attention-Deficit Hyperactivity Disorder (ADHD) Treated With Osmotic-Release Oral System Methylphenidate. *J Child Neurol.* 2015;30(8):1000-1009.

Reason for exclusion: Less than seven days treatment

SLCTR/2009/006

- http://slctr.lk/trials/73

Reasons for exclusion: No pharmacological intervention

Sleator1974a

- Sleator EK, Sprague RL. Proceedings: Dose effects of stimulants in hyperkinetic children. *Psychopharmacol Bull.* 1974;10(4):29-33.

Reason for exclusion: No DSM/ICD criteria

Sleator1974b

- Sleator EK, Von Neumann A, Sprague RL. Hyperactive children. A continuous long-term placebo-controlled follow-up. *JAMA.* 1974;229(3):316-317.

Reason for exclusion: No DSM/ICD; Participants: responders to previous ADHD medications; no mention of randomization

Sleator1974c

- Sleator EK, Von Neumann AW. Methylphenidate in the treatment of hyperkinetic children. *Clin Pediatr (Phila).* 1974;13(1):19-24.

Reason for exclusion: No DSM/ICD criteria

Small1971

- Small A, Hibi S, Feinberg I. Effects of dextroamphetamine sulfate on EEG sleep patterns of hyperactive children. *Arch Gen Psychiatry.* 1971;25(4):369-380.

Reason for exclusion: No DSM/ICD, no randomised

Smith1998

- Smith BH. Reliability, validity and unique contributions of self-reports by adolescents being treated for attention-deficit hyperactivity disorder. *Dissertation Abstracts International: Section B: The Sciences and Engineering.* 1997;58(6-B):3328.
- Smith BH, Pelham WE, Gnagy E, Yudell RS. Equivalent effects of stimulant treatment for attention-deficit hyperactivity disorder during childhood and adolescence. *J Am Acad Child Adolesc Psychiatry.* 1998;37(3):314-321.
- Smith BH, Pelham WE, Evans S, et al. Dosage effects of methylphenidate on the social behavior of adolescents diagnosed with attention deficit hyperactivity disorder. *Exp Clin Psychopharmacol.* 1998;6(2):187-204.
- Smith BH, Pelham WE, Jr., Gnagy E, Molina B, Evans S. The reliability, validity, and unique contributions of self-report by adolescents receiving treatment for attention-deficit/hyperactivity disorder. *J Consult Clin Psychol.* 2000;68(3):489-499.
- Evans SW, Pelham WE, Smith BH, et al. Dose-response effects of methylphenidate on ecologically valid measures of academic performance and classroom behavior in adolescents with ADHD. *Exp Clin Psychopharmacol.* 2001;9(2):163-175.

Reason for exclusion: Less than seven days treatment

Smith2004

- Smith R, Larsen D, Derby K, et al. A comparison of teacher checklists used over 15 days and a one-day antecedent analysis to conduct a medication trial. *Psychol Sch.* 2004;41(2):235-240.

Reason for exclusion: N-of-1 trial

Smithee1998

- Smithee JA, Klorman R, Brumaghim JT, Borgstedt AD. Methylphenidate does not modify the impact of response frequency or stimulus sequence on performance and event-related potentials of children with attention deficit hyperactivity disorder. *J Abnorm Child Psychol*. 1998;26(4):233-245

Reason for exclusion: Cross-over without wash out; pre-cross over data not available

Smitherman1990

- Smitherman CH. A drug to ease attention deficit-hyperactivity disorder. *MCN Am J Matern Child Nurs.* 1990;15(6):362-365.

Reason for exclusion: Review/Commentary

Snircova2016

- Snircova E, Marcincakova-Husarova V, Hrtanek I, Kulhan T, Ondrejka I, Nosalova G. Anxiety reduction on atomoxetine and methylphenidate medication in children with ADHD. *Pediatr Int*. 2016;58(6):476-81

Reason for exclusion: Unclear if study was double blind; written to author who confirmed it was not double blind

So2008

- So Y-c. Effectiveness *of methylphenidate and combined treatment (methylphenidate and psychosocial treatment) for Chinese children with attention-deficit/hyperactivity disorder in a community mental health center* [Ph.D.]. Ann Arbor, The Chinese University of Hong Kong (Hong Kong); 2005.
- So CY, Leung PW, Hung SF. Treatment effectiveness of combined medication/behavioural treatment with chinese ADHD children in routine practice. *Behav Res Ther.* 2008;46(9):983-992.

Reason for exclusion: No arms of interest for the present meta-analysis (methylphenidate vs methylphenidate + parent training)

Sobanski2013 (NCT00938743; EUCTR2007-004309-90-DE)

- Sobanski E, Sabljic D, Alm B, et al. A randomized, waiting list-controlled 12-week trial of atomoxetine in adults with ADHD. *Pharmacopsychiatry.* 2012;45(3):100-107.
- Sobanski E, Sabljic D, Alm B, et al. Driving performance in adults with ADHD: results from a randomized, waiting list controlled trial with atomoxetine. *Eur Psychiatry.* 2013;28(6):379-385.
- https://clinicaltrials.gov/ct2/show/NCT00938743
- https://www.clinicaltrialsregister.eu/ctr-search/search?query=eudract_number:2007-004309-90

Reason for exclusion: Atomoxetine vs. waiting list; Open label; Note: Clinicaltrial.gov number erroneously reported in the paper as NCT00619840

Solanto1982

- Solanto MV, Conners CK. A dose-response and time-action analysis of autonomic and behavioral effects of methylphenidate in attention deficit disorder with hyperactivity. *Psychophysiology.* 1982;19(6):658-667.

Reason for exclusion: Less than seven days treatment

Solanto1986

- Solanto MV. Behavioral effects of low-dose methylphenidate in childhood Attention Deficit Disorder: Implications for a mechanism of stimulant drug action. *J Am Acad Child Psychiatry.* 1986(1):96-101.

Reason for exclusion: Single dose

Solanto1989

- Solanto MV, Wender EH. Does methylphenidate constrict cognitive functioning?.[Erratum appears in J Am Acad Child Adolesc Psychiatry 1990 Jan;29(1):156]. *J Am Acad Child Adolesc Psychiatry.* 1989;28(6):897-902.

Reason for exclusion: Less than seven days treatment

Solanto2009 (NCT00824317)

- Subset of: Solanto MV, Gilbert SN, Raj A, Zhu J, Pope-Boyd S, Stepak B, Vail L, Newcorn JH: Neurocognitive functioning in ADHD, Predominantly Inattentive Subtype. *J Abnorm Child Psychol*. 2007;35:729-744.

- Solanto M, Newcorn J, Vail L, Gilbert S, Ivanov I, Lara R. Stimulant drug response in the predominantly inattentive and combined subtypes of attention-deficit/hyperactivity disorder. *J Child Adolesc Psychopharmacol.* 2009;19(6):663-671
- https://clinicaltrials.gov/ct2/show/NCT00824317

Reason for exclusion: No pre cross-over data

Solomons1971

- Solomons G. The role of methylphenidate and dextroamphetamine in hyperactivity in children. *J Iowa Med Soc.* 1971;61(11):658-661.

Reason for exclusion: Review

Song2005

- Song DH, Shin DW, Jon DI, Ha EH. Effects of methylphenidate on quantitative EEG of boys with attention-deficit hyperactivity disorder in continuous performance test. *Yonsei Med J.* 2005;46(1):34-41.

Reason for exclusion: No RCT

Song2014

- Song J, Kim SW, Hong HJ, et al. Association of SNAP-25, SLC6A2, and LPHN3 with OROS methylphenidate treatment response in attention-deficit/hyperactivity disorder. *Clin Neuropharmacol.* 2014;37(5):136-141.

Reason for exclusion: No randomized

Sora2008

- Sora I, Ikari M, Ikeda K. [Drug dependence and methylphenidate]. *Seishin Shinkeigaku Zasshi.* 2008;110(10):941-945.

Reason for exclusion: Commentary

Sostek1980

- Sostek AJ, Buchsbaum MS, Rapoport JL. Effects of amphetamine on vigilance performance in normal and hyperactive children. *J Abnorm Child Psychol.* 1980;8(4):491-500.

Reason for exclusion: No DSM/ICD criteria; Less than seven days treatment

Spear2003

- Spear J, Alderton D. Psychosis associated with prescribed dexamphetamine use. *Aust N Z J Psychiatry.* 2003;37(3):383.

Reason for exclusion: Case reports

Speech1993

- Speech, TJ, Rao, SM, Osmon, DC, Sperry, LT. A double-blind controlled study of methylphenidate treatment in closed head injury. *Brain Inj.* 1993;7: 333-388

Reason for exclusion: No ADHD

Spencer2001

- Spencer T, Biederman J, Heiligenstein J, et al. An open-label, dose-ranging study of atomoxetine in children with attention deficit hyperactivity disorder. *J Child Adolesc Psychopharmacol.* 2001;11(3):251-265.

Reason for exclusion: Open label

Spencer2002

- Spencer TJ, Swanson JM, Markabi S, Weidenman M, Faleck H. Pharmacodynamic and pharmacokinetic profiles of a new modified-release formulation of methylphenidate in children with ADHD. 153rd Annual Meeting of the American Psychiatric Association; 2000 May 13-18; Chicago, ILNr:567.
- Spencer TJ, Markabi S, Weidenman M, Faleck H. Pharmacodynamic and pharmacokinetic profiles of a new modified-release formulation of methylphenidate in children with adhd. 155th Annual Meeting of the American Psychiatric Association 2002

Reason for exclusion: Likely less than seven days but not reply from author

Spencer2002

- Spencer T, Biederman J, Coffey B, et al. A double-blind comparison of desipramine and placebo in children and adolescents with chronic tic disorder and comorbid attention-deficit/hyperactivity disorder. *Arch Gen Psychiatry.* 2002;59(7):649-656.

Reason for exclusion: Medication of no interest for the present meta-analysis vs. placebo

Spencer2003

- Spencer TJ. Preliminary results of a six-month trial of methylphenidate in adults with adhd. *156th Annual Meeting of the American Psychiatric Association, May 17-22, San Francisco CA.* 2003:No. 54B.
- Spencer T, Biederman J, Eric M, Stephen F. Efficacy in a 6-month trial of methylphenidate in adults with attention-deficit/hyperactivity disorder. *Int J Neuropsychopharmacol.* 2004;7(Suppl. 2):S442-S443.

Reason for exclusion: Only abstracts, not clear if linked to any study retrieved in our search; author contacted but not reply

Spencer2004

- Spencer T, Biederman J, Wilens T. Stimulant treatment of adult attention-deficit/hyperactivity disorder. *Psychiatr Clin North Am*. 2004;27(2):361-72

Reason for exclusion: Review, no empirical data

Spencer2006

- Spencer T, Biederman J, Abikoff HB, Pliszka SR, Boellner SW, Lopez FA, Read SC, Tulloch SJ. Safety and efficacy of mixed amphetamine salts extended release in children and adolescents with oppositional defiant disorder (ODD). *157th Annual Meeting of the American Psychiatric Association, New York, 2004*
- Spencer TJ, Abikoff HB, Connor DF, et al. Efficacy and safety of mixed amphetamine salts extended release (adderall XR) in the management of oppositional defiant disorder with or without comorbid attention-deficit/hyperactivity disorder in school-aged children and adolescents: A 4-week, multicenter, randomized, double-blind, parallel-group, placebo-controlled, forced-dose-escalation study. *Clin Ther.* 2006;28(3):402-418.

Reason for exclusion: Randomization not stratified for comorbidity with ADHD, so not correct to consider subgroup of ADHD+ODD

Spencer2007 (SHP465; Study 303)

- Spencer TJ, Anderson CS, Silverberg A, Youcha SH. Triple-bead mixed amphetamine salts (SPD465) improves hyperactivity/impulsivity and inattentiveness in adults with ADHD. *Biol Psychiatry*. 2007;61:171S-2S.
- Spencer TJ, Anderson CS, Silverberg A, Youcha S. Improvement in hyperactivity/impulsivity and inattentiveness associated with adult ADHD after triple-bead mixed amphetamine salts (SPD465) treatment. *Biol Psychiatry.* 2007;61(8, Suppl. S):172S.

Reason for exclusion: Abstract only; manufacturer not able to provide data before publication of the study (26.03.2017)

Spencer2008 (NCT00151996; SPD503-205)

- Spencer T, Greenbaum M, Ginsberg LD, Murphy WR, Farrand K: Open-label coadministration of guanfacine extended re-lease and stimulants in children and adolescents with attention-deficit=hyperactivity disorder. Poster presented at: American Psychiatric Association's 161st Annual Meeting, May 3–8, 2008, Washington, DC.

Reason for exclusion: Open label

Spencer2012 (NCT00302458)

- Spencer TJ, Biederman J, Martin JM, Moorehead TM, Mirto T, Clarke A, Batchelder H, Faraone SV. Importance of pharmacokinetic profile and timing of coadministration of short- and long-acting formulations of methylphenidate on patterns of subjective responses and abuse potential. *Postgrad Med.* 2012;124(1):166-73.
- https://clinicaltrials.gov/ct2/show/NCT00302458

Reason to exclude: No participants with ADHD

Spiga1996

- Spiga R, Pearson DA, Broitman M, Santos CW. Effects of methylphenidate on cooperative responding in children with attention deficit-hyperactivity disorder. *Exp Clin Psychopharmacol.* 1996;4(4):451-458.

Reason for exclusion: Less than seven days treatment

Sprague1970

- Sprague RL, Barnes KR, Werry JS. Methylphenidate and thioridazine: Learning, reaction time, activity, and classroom behavior in disturbed children. *Am J Orthopsychiatry 1970;* 40, 4, 615-628

Reason for exclusion: No DSM/ICD criteria

Sprague1977

- Sprague RL, Sleator EK. Methylphenidate in hyperkinetic children: differences in dose effects on learning and social behavior. *Science.* 1977;198(4323):1274-1276.

Reason for exclusion: No DSM/ICD criteria

Srinivas1992

- Srinivas NR, Hubbard JW, Quinn D, Midha KK. Enantioselective pharmacokinetics and pharmacodynamics of racemic-threo-methylphenidate in children with attention deficit hyperactivity disorder. *Clin Pharmacol Ther.* 1992;52(5):561-568.

Reason for exclusion: Less than seven days treatment

Sripada2011

- Sripada CS, Kessler D, Welsh R, Liberzon I. Fronto-opercular control circuits mediate the effect of methylphenidate on reaction time variability. *Neuropsychopharmacology.* 2011;36:S112-S113.

Reason for exclusion: No participants with ADHD

Sripada2012

- Sripada CS, Kessler DA, Phan KL, Liberzon I. Phase-Specific Engagement of Cognitive Control Circuits Predicts Reaction Time Variability and Discriminates Methylphenidate from Placebo. *Biol Psychiatry.* 2012;71(8, Suppl. S):73S.

Reason for exclusion: No participants with ADHD

Sroufe1973

- Sroufe LA, Stewart MA. Treating problem children with stimulant drugs. *N Engl J Med.* 1973;289(8):407-413.

Reason for exclusion: Review

Stableford1976

- Stableford W, Butz R, Hasazi, Leitenberg H, Peyser J. Sequential withdrawal of stimulant drugs and use of behavior therapy with two hyperactive boys. *Am J Orthopsychiatry.* 1976;46(2):302-312.

Reason for exclusion: Two case reports

Stark2016

- Stark JG, Engelking D, McMahen R, Sikes C. A randomized crossover study to assess the pharmacokinetics of a novel amphetamine extended-release orally disintegrating tablet in healthy adults. *Postgrad med.* 2016;128(7):648-655.

Reason for exclusion: No participants with ADHD

Steeger2016

- Steeger CM, Gondoli DM, Gibson BS, Morrissey RA. Combined cognitive and parent training interventions for adolescents with ADHD and their mothers: A randomized controlled trial. *Child Neuropsychol.* 2016;22(4):394-419.

Reason for exclusion: No arms of interest for the present meta-analysis (behavioural training, cognitive training, behavioural training+cognitive training, placebo)

Steele2004

- Steele M, Riccardelli R, Binder C. The effectiveness of OROS (R) methylphenidate (Concerta (R)) vs. usual treatment with immediate-release methylphenidate (IR MPH) in children aged 6-12 years with attention deficit hyperactivity disorder (ADHD). *Int J Neuropsychopharmacol.* 2004;7(Suppl. 2):S442.

Reason for exclusion: Open label

Steele2005

- Steele MM, Prinzo R, Binder C. Long-term effectiveness and safety of Concerta in children with ADHD: a six-month study. *158th Annual Meeting of the American Psychiatric Association; 2005 May 21-26; Atlanta, GA.* 2005.

Reason for exclusion: Open label

Steele2006 (NCT00304681)

- Prinzo R, Steele MM, Binder C. Effectiveness of concerta versus usual care IR-MPH on comorbid ODD symptoms in children with ADHD. *158th Annual Meeting of the American Psychiatric Association*; 2005 May 21-26; Atlanta, GA2005.
- Steele M, Weiss M, Swanson J, Wang J, Prinzo RS, Binder CE. A randomized, controlled effectiveness trial of OROS-methylphenidate compared to usual care with immediate-release methylphenidate in attention deficit-hyperactivity disorder. *Can J Clin Pharmacol.* 2006;13(1):e50-62.
- Commentary: Shea SE. A comparison of methylphenidate formulations in the treatment of ADHD. *Can J Clin Pharmacol.* 2006;13:e63-4.

- https://clinicaltrials.gov/ct2/show/NCT00304681

Reason for exclusion: Open label

Steele2006

- Steele M. Introduction to remission in ADHD: Raising the bar. *Clin Ther.* 2006;28(11):1879-1881.

Reason for exclusion: Commentary

Stein1996

- Stein MA, Blondis TA, Schnitzler ER, et al. Methylphenidate dosing: twice daily versus three times daily. *Pediatrics.* 1996;98(4 Pt 1):748-756.

Reason for exclusion: Some participants had 3 weeks of treatment, differently from others

Stein2001

- Stein MA. More intensive methylphenidate treatment for children with ADHD. *Pediatr Res.* 2001(4):29a.
- Pelham WE, Gnagy EM, Burrows-Maclean L, et al. Once-a-day Concerta methylphenidate versus three-times-daily methylphenidate in laboratory and natural settings. *Pediatrics.* 2001;107(6):E105.
- Wolraich ML, Greenhill LL, Pelham W, et al. Randomized, controlled trial of oros methylphenidate once a day in children with attention-deficit/hyperactivity disorder. *Pediatrics.* 2001;108(4):883-892.
- Swanson J, Gupta S, Lam A, et al. Development of a new once-a-day formulation of methylphenidate for the treatment of attention-deficit/hyperactivity disorder: proof-of-concept and proof-of-product studies. *Arch Gen Psychiatry.* 2003;60(2):204-211.

Reason for exclusion: Review of 3 trials (All retrieved in our search). (confirmed by Dr Stein)

Stein2003

- Stein MA, Sarampote CS, Waldman ID, et al. A dose-response study of OROS methylphenidate in children with attention-deficit/hyperactivity disorder. *Pediatrics*. 2003;112(5):e404.
- Stein MA, Seymour KE, Black DO, Sarampote CS, Robb A, Conlon C, et al. Effects and side effects of Concerta methylphenidate (MPH) in children with ADHD and comorbid internalizing symptoms. Pediatr Res. 2003 May 3-6; Seattle, Washington. Baltimore: International Pediatr Res Foundation, 2003;53 (4):555A
- Stein MA, Sarampote C, Seymour K. Insomnia and tiredness in ADHD youth: relationship with methylphenidate dose, age, and weight. Pediatr Res. 2004 May 4; San Francisco, CA. Baltimore: International Pediatr Res Foundation, 2004; 55 (4):74A.
- Stein MA, Waldman ID, Sarampote C, Seymour K, Cook EH. Dopamine transporter genotype (DAT1) predicts stimulant response in children with attention deficit hyperactivity disorder. *Pediatr Res.* 2004;55:1A
- Stein MA, Waldman ID, Sarampote CS, et al. Dopamine transporter genotype and methylphenidate dose response in children with ADHD. *Neuropsychopharmacology*. 2005;30(7):1374-1382
- Pooled in:Gruber R, Joober R, Grizenko N, Leventhal BL, Cook EH, Jr., Stein MA. Dopamine transporter genotype and stimulant side effect factors in youth diagnosed with attention-deficit/hyperactivity disorder. *J Child Adolesc Psychopharmacol.* 2009;19(3):233-239

Reason for exclusion: Cross-over without wash out; pre-cross over data not available

Stein2015 (MACRO Study)

- Stein M, Garison M, Hart A, Newcorn J. Sleep problems in ADHD youth before and during treatment with methylphenidate and atomoxetine. *ADHD Atten Defic Hyperact Disord.* 2015;7:S46-S47.
- Stein M, Hildebrandt T, Cook Jr E, Olson E, Waldman I, Newcorn J. Dopamine transporter (DAT1) and dopamine receptor DRD4) genotype and response to methylphenidate and atomoxetine. *ADHD Atten Defic Hyperact Disord.* 2015;7:S60.

Reason for exclusion: Abstract only; contacted author (5.9.16): manuscript submitted to publication, not possible to share data

Steinberg1971

- Steinberg GG, Troshinsky C, Steinberg HR. Dextroamphetamine-responsive behavior disorder in school children. *Am J Psychiatry.* 1971;128(2):174-179.

Reason for exclusion: No DSM/ICD criteria

Steiner2014

- Steiner NJ, Frenette EC, Rene KM, Brennan RT, Perrin EC. In-school neurofeedback training for ADHD: sustained improvements from a randomized control trial. *Pediatrics*. 2014;133(3):483-92.

Reason for exclusion: No arms of interest for the present meta-analysis (neurofeedback, cognitive training, control)

Steingard1993

- Steingard R, Biederman J, Spencer T, Wilens T, Gonzalez A. Comparison of clonidine response in the treatment of attention-deficit hyperactivity disorder with and without comorbid tic disorders. *J Am Acad Child Adolesc Psychiatry.* 1993;32(2):350-353.

Reason for exclusion: No RCT

Steingard1994

- Steingard RJ, Goldberg M, Lee D, DeMaso DR. Adjunctive clonazepam treatment of tic symptoms in children with comorbid tic disorders and ADHD. *J Am Acad Child Adolesc Psychiatry.* 1994;33(3):394-399.

Reason for exclusion: No RCT

Steinhausen1981

- Steinhausen HC, Kreuzer EM. Learning in hyperactive children: are there stimulant-related and state-dependent effects? *Psychopharmacology (Berl).* 1981;74(4):389-390.
- Steinhausen HC, Kreuzer EM, Göebel D, Romahn G. [Learning and attention under the influence of methylphenidate]. - The concentration-disturbed and hyperactive child Das konzentrationsgestoerte und hyperaktive Kind. Ergebnisse aus Klinik und Forschung. 1982:52-62

Reason for exclusion: Single dose.

Stephens1984

- Stephens RS, Pelham WE, Skinner R. State-dependent and main effects of methylphenidate and pemoline on paired-associate learning and spelling in hyperactive children. *J Consult Clin Psychol.* 1984;52(1):104-113.

Reason for exclusion: Less than seven days treatment

Stoner1994

- Stoner G, Carey SP, Ikeda MJ, Shinn MR. The utility of curriculum-based measurement for evaluating the effects of methylphenidate on academic performance. *J Appl Behav Anal.* 1994;27(1):101-113.

Reason for exclusion: Two case studies; Less than seven days treatment

Strand2012

- Strand MT, Hawk LW, Jr., Bubnik M, Shiels K, Pelham WE, Jr., Waxmonsky JG. Improving working memory in children with attention-deficit/hyperactivity disorder: the separate and combined effects of incentives and stimulant medication. *J Abnorm Child Psychol.* 2012;40(7):1193-1207.

Reason for exclusion: Placebo: single dose

Strawn2017

- Strawn JR, Compton SN, Robertson B, Albano AM, Hamdani M, Rynn MA. Extended Release Guanfacine in Pediatric Anxiety Disorders: A Pilot, Randomized, Placebo-Controlled Trial. *J Child Adolesc Psychopharmacol.* 2017;27:29-37.

Reason for exclusion: No participants with ADHD

Stray2009

- Stray LL, Stray T, Iversen S, Ruud A, Ellertsen B. Methylphenidate improves motor functions in children diagnosed with Hyperkinetic Disorder. *Behav Brain Funct: BBF.* 2009;5:21.

Reason for exclusion: Less than seven days treatment

Su2016

- Su Y, Yang L, Stein MA, Cao Q, Wang Y. Methylphenidate Versus Atomoxetine for the Treatment of Attention-Deficit/Hyperactivity Disorder in Chinese Youth: 8-Week Comparative Efficacy and 1-Year Follow-Up. *J Child Adolesc Psychopharmacol.* 2016;26(4):362-371.

Reason for exclusion: Open label

Sudarmadji2016

- Sudarmadji SS, Meliala L, Aziz A. Improvement of cognitive function in attention deficit hyperactivity disorder (ADHD) treatment by methylphenidate (MPH) of elementary school students at Bantul District, Yogyakarta Special Regency. *J Neurol Sci*. 2009:S236, Abstract no: PO16-TU-01.

Reason for exclusion: Abstract only; Not possible to contact author to confirm if study meets inclusion criteria for the present meta-analysis

Sumner2006 (B4Z-US-LYBH)
- Sumner CR, Schuh KJ, Sutton VK, Lipetz R, Kelsey DK. Placebo-controlled study of the effects of atomoxetine on bladder control in children with nocturnal enuresis. *J Child Adolesc Psychopharmacol.* 2006;16(6):699-711.

Reason for exclusion: Not all subjects had ADHD; no outcomes of interest for the present meta-analysis

Sumner2009 (NCT00191048)
- Sumner CR, Gathercole S, Greenbaum M, et al. Atomoxetine for the treatment of attention-deficit/hyperactivity disorder (ADHD) in children with ADHD and dyslexia. *Child & Adolescent Psychiatry & Mental Health [Electronic Resource].* 2009;3:40.
- https://clinicaltrials.gov/ct2/show/NCT00191048

Reason for exclusion: No RCT, no double blind

Sumner2010
- Sumner CR, Haynes VS, Teicher MH, Newcorn JH. Does placebo response differ between objective and subjective measures in children with attention-deficit/hyperactivity disorder? *Postgrad Med.* 2010;122(5):52-61

Reason for exclusion: Cross-over without wash out; pre-cross over data not available

Sund2002
- Sund AM, Zeiner P. Does extended medication with amphetamine or methylphenidate reduce growth in hyperactive children? *Nord J Psychiatry.* 2002;56(1):53-57.

Reason for exclusion: No RCT

Sunohara1997
- Sunohara GA. *Methylphenidate effects on focused and selective attention processing in children with ADHD* [Ph.D.]. Ann Arbor, University of Toronto (Canada); 1997.

Reason for exclusion: No outcomes of interest for the present meta-analysis (no data on drop out). Author confirmed that no data on further outcomes are available

Sunohara1999
- Sunohara GA. *Methylphenidate effects on focused and selective attention processing in children with ADHD* [Ph.D.]. Ann Arbor, University of Toronto (Canada); 1997.
- Sunohara GA. Methylphenidate effects on focused and selective attention processing in children with ADHD. *Dissertation Abstracts International: Section B: The Sciences and Engineering.* 1998;59(6-B):3113.
- Sunohara GA, Malone MA, Rovet J, Humphries T, Roberts W, Taylor MJ. Effect of methylphenidate on attention in children with attention deficit hyperactivity disorder (ADHD): ERP evidence. *Neuropsychopharmacology.* 1999;21(2):218-228.

Reason for exclusion: Less than seven days treatment

Surman2009
- Surman CBH, Adamson JJ, Petty C, et al. Association between attention-deficit/hyperactivity disorder and sleep impairment in adulthood: evidence from a large controlled study. *J Clin Psychiatry.* 2009;70(11):1523-1529.

Reason for exclusion: No RCT

Swanson1976
- Swanson JM, Kinsbourne M. Stimulant-related state-dependent learning in hyperactive children. *Science.* 1976;192(4246):1354-1357.

Reason for exclusion: No DSM/ICD criteria

Swanson1979
- Swanson JM, Barlow A, Kinsbourne M. Task specificity of responses to stimulant drugs in laboratory tests. *Int J Ment Health.* 1979;8:1, 67-82

Reason for exclusion: No diagnostic criteria as per protocol

Swanson1983
- Swanson JM, Sandman CA, Deutsch C, Baren M. Methylphenidate hydrochloride given with or before breakfast: I. Behavioral, cognitive, and electrophysiologic effects. *Pediatrics.* 1983;72(1):49-55.
- Baren M, Swanson JM, Wigal SB. Lack of effect of different breakfast conditions on the pharmacokinetics and efficacy of OROS methylphenidate HCI extended-release tablets in children with ADHD. *Pediatr Res.* 2000(4):23a

Reason for exclusion: Less than seven days treatment

Swanson1991

- Swanson JM, Cantwell D, Lerner M, McBurnett K, Hanna G. Effects of stimulant medication on learning in children with ADHD. *J Learn Disabil.* 1991;24(4):219-230, 255.

Reason for exclusion: Review

Swanson1998

- Swanson JM, Wigal S, Greenhill LL, et al. Analog classroom assessment of Adderall in children with ADHD. *J Am Acad Child Adolesc Psychiatry.* 1998;37(5):519-526.
- Swanson J, Wigal S, Greenhill L, et al. Objective and subjective measures of the pharmacodynamic effects of Adderall in the treatment of children with ADHD in a controlled laboratory classroom setting. *Psychopharmacol Bull.* 1998;34(1):55-60.
- Wigal SB, Swanson JM, Greenhill L, et al. Evaluation of individual subjects in the analog classroom setting: II. Effects of dose of amphetamine (Adderall(R)). *Psychopharmacol Bull.* 1998;34(4):833-838.

Reason for exclusion: History of clinical significant response to methylphenidate

Swanson1999

- Swanson JM, Wigal SB, Udrea D, et al. Evaluation of individual subjects in the analog classroom setting: I. Examples of graphical and statistical procedures for within-subject ranking of responses to different delivery patterns of methylphenidate. *Psychopharmacol Bull.* 1998;34(4):825-832.
- Wigal SB, Gupta S, Guinta D, Swanson JM. Reliability and validity of the SKAMP rating scale in a laboratory school setting. *Psychopharmacol Bull.* 1998;34(1):47-53.
- Swanson J, Gupta S, Guinta D, et al. Acute tolerance to methylphenidate in the treatment of attention deficit hyperactivity disorder in children. *Clin Pharmacol Ther.* 1999;66(3):295-305.

Reason for exclusion: Two studies, both Less than seven days treatment

Swanson2002

- Swanson J, Sadeh A, Lerner MA, Wigal SB. Comparison of the impact of OROS methylphenidate HCI with methylphenidate tid and placebo on the sleep of children with ADHD. *J Dev Behav Pediatr.* 2000;21(5):387–8.
- Swanson JM, Wigal SB, Lemer MA. Comparison of the efficacy and safety of OROS methylphenidate HCI with methylphenidate tid and placebo in children with ADHD. *Pediatr Res.* 2000;47(4):34A
- Wigal S, Swanson JM, Lerner M. Comparison of duration of effect of OROS MPH with MPH tid in ADHD children [abstract]. *2001 Annual Meeting of the American Psychiatric Association*; 2001 May 5-10; New Orleans, LA2001.
- Wigal S, Lerner M, Swanson J. Once-daily methylphenidate formulation: impact on academic productivity and activity levels of children with attention-deficit/hyperactivity disorder. *Eur Neuropsychopharmacol.* 2002:S416
- Swanson JM, Gupta S, Williams L, Agler D, Lerner M, Wigal S. Efficacy of a new pattern of delivery of methylphenidate for the treatment of ADHD: effects on activity level in the classroom and on the playground. *J Am Acad Child Adolesc Psychiatry.* 2002;41(11):1306-1314.

Reason for exclusion: Less 7 days treatment (3 days). Note: This study is also reported in: Swanson J, Gupta S, Lam A, et al. Development of a new once-a-day formulation of methylphenidate for the treatment of attention-deficit/hyperactivity disorder: proof-of-concept and proof-of-product studies. Arch Gen Psychiatry. Feb 2003;60(2):204-211 (part 1). Both studies in this report are not pertinent for the present meta-analysis since they include subjects who responded well to methylphenidate

Swanson2002

- Swanson J, Wigal S, Lerner M. Treatment with a controlled-release formulation of methylphenidate for attention-deficit/hyperactivity disorder: onset and duration of effect. *Eur Neuropsychopharmacol.* 2002(Suppl 3):S414.

Reason for exclusion: Cross-over without wash out; pre-cross over data not available

Swanson2004 (NCT00381758)

- Greenhill LL. Comparison of Classroom Deportment in Six-Twelve Year-Old Children With ADHD After Administration of Two Once-Daily Extended Release Methylphenidate (MPH) Formulations. *156th Annual Meeting of the American Psychiatric Association*; 2003 May 17-22; San Francisco, CA2003:Nr644.
- Swanson JM, Wigal SB, Wigal T, et al. A comparison of once-daily extended-release methylphenidate formulations in children with attention-deficit/hyperactivity disorder in the laboratory school (the Comacs Study). *Pediatrics.* 2004;113(3 Pt 1):e206-216. E
- Sonuga-Barke EJ, Swanson JM, Coghill D, DeCory HH, Hatch SJ. Efficacy of two once-daily methylphenidate formulations compared across dose levels at different times of the day: preliminary indications from a secondary analysis of the COMACS study data. *BMC Psychiatry.* 2004;4:28.

- Sonuge-Barke EJS, Swanson J, Hatch S, Van Lier P, Vandenberghe M.Heterogeneity in ADHD children's response to two long-acting methylphenidate formulations. *J Neural Transm. Abstracts of the 39th International Danube Symposium for Neurological Sciences and Continuing Education and 1st International Congress on ADHD, from Childhood to Adult Disease 2007;114(7): LXXXIX*
- Sonuga-Barke EJ, Coghill D, Markowitz JS, Swanson JM, Vandenberghe M, Hatch SJ. Sex differences in the response of children with ADHD to once-daily formulations of methylphenidate. *J Am Acad Child Adolesc Psychiatry.* 2007;46(6):701-710.
- Sonuga-Barke EJS, Van Lier P, Swanson JM, et al. Heterogeneity in the pharmacodynamics of two long-acting methylphenidate formulations for children with attention deficit/hyperactivity disorder - A growth mixture modelling analysis. *Eur Child Adolesc Psychiatry*. 2008;17(4):245-254.
- Sonuga-Barke EJ, Coghill D, DeBacker M, Swanson J. Measuring methylphenidate response in attention-deficit/hyperactvity disorder: how are laboratory classroom-based measures related to parent ratings? *J Child Adolesc Psychopharmacol.* 2009;19(6):691-698.
- Sonuga-Barke EJ, Coghill D, Wigal T, DeBacker M, Swanson J. Adverse reactions to methylphenidate treatment for attention-deficit/hyperactivity disorder: structure and associations with clinical characteristics and symptom control. *J Child Adolesc Psychopharmacol*. 2009;19(6):683-690.
- https://clinicaltrials.gov/ct2/show/NCT00381758

Reason for exclusion: Cross-over without wash out; pre-cross over data not available

Swartwood1998

- Swartwood MO, Swartwood JN, Lubar JF, Timmermann DL, Zimmerman AW, Muenchen RA. Methylphenidate effects on EEG, behavior, and performance in boys with ADHD. *Pediatr Neurol.* 1998;18(3):244-250.

Reason for exclusion: No RCT

Sykes1971

- Sykes DH, Douglas VI, Weiss G, Minde KK. Attention in hyperactive children and the effect of methylphenidate (ritalin). *J Child Psychol Psychiatry.* 1971;12(2):129-139.

Reason for exclusion: No DSM/ICD criteria

Sykes1972

- Sykes DH, Douglas VI, Morgenstern G. The effect of methylphenidate (ritalin) on sustained attention in hyperactive children. *Psychopharmacologia.* 1972;25(3):262-274.

Reason for exclusion: No DSM/ICD criteria

Syrigou-Papavasiliou1988

- Syrigou-Papavasiliou A, Lycaki H, LeWitt PA, Verma NP, Spivak D, Chayasirisobhon S. Dose-response effects of chronic methylphenidate administration on late event-related potentials in attention deficit disorder. *Clinical EEG (electroencephalography).*1988;19(3):129-133.

Reason for exclusion: After placebo phase, subjects randomly assigned to different dosages of MPH

Szobot2003

- Szobot CM, Ketzer C, Cunha RD, et al. The acute effect of methylphenidate on cerebral blood flow in boys with attention-deficit/hyperactivity disorder. *Eur J Nucl Med Mol Imaging.* 2003;30(3):423-426.

Reason for exclusion: Less than seven days treatment

Szobot2004

- Szobot CM, Ketzer C, Parente MA, Biederman J, Rohde LA. The acute effect of methylphenidate in Brazilian male children and adolescents with ADHD: a randomized clinical trial. *J Atten Disord.* 2004;8(2):37-43.

Reason for exclusion: Less than seven days treatment

Szobot2008

- Szobot CM, Rohde LA, Katz B, et al. A randomized crossover clinical study showing that methylphenidate-SODAS improves attention-deficit/hyperactivity disorder symptoms in adolescents with substance use disorder. *Braz J Med Biol Res.* 2008;41(3):250-257.

Reason for exclusion: Single-blind

Tahir2000

- Tahir E, Yazgan Y, Cirakoglu B, Ozbay F, Waldman I, Asherson PJ. Association and linkage of DRD4 and DRD5 with attention deficit hyperactivity disorder (ADHD) in a sample of Turkish children. *Mol Psychiatry.* 2000;5(4):396-404.

Reason for exclusion: No RCT

Tamm2007

- Tamm L, Carlson CL. Task demands interact with the single and combined effects of medication and contingencies on children with ADHD. *J Atten Disord.* 2007;10(4):372-380.

Reason for exclusion: Single dose study

Tan2005

- Tan M, Appleton R. Attention deficit and hyperactivity disorder, methylphenidate, and epilepsy. *Arch Dis Child.* 2005;90(1):57-59.

Reason for exclusion: Review

Tanaka2013 (B4Z-MC-LYDO)

- Tanaka Y, Upadhyaya H. Assessment of effects of atomoxetine in adult patients with ADHD: Consistency among 3 geographic regions in a response maintenance study. *Eur Neuropsychopharmacol.* 2013;23:S600.
- Thome J, Escobar R, Lipsius S, Upadhyaya H. Predictors of relapse or maintenance of response of Attention-Deficit/Hyperactivity Disorder symptoms after discontinuation of long-term treatment with atomoxetine. *ADHD Atten Defic Hyperact Disord*. 2015;7:S97.

Reason for exclusion: Withdrawal design

Tang2009

- Tang C-S, Chou W-J, Cheng ATA. Atomoxetine hydrochloride-associated transient psychosis in an adolescent with attention-deficit/hyperactivity disorder and mild mental retardation. *J Child Adolesc Psychopharmacol.* 2009;19(3):319-320.

Reason for exclusion: Case report

Tannock1989

- Tannock R, Schachar RJ, Carr RP, Chajczyk D, Logan GD. Effects of methylphenidate on inhibitory control in hyperactive children. *J Abnorm Child Psychol.* 1989;17(5):473-491.
- Tannock R, Schachar RJ, Carr RP, Logan GD. Dose-response effects of methylphenidate on academic performance and overt behavior in hyperactive children. *Pediatrics.* 1989;84(4):648-657.
- Erratum in: Tannock R, Schachar RJ, Carr RP, Chajczyk D, et al. "Effects of methylphenidate on inhibitory control in hyperactive children": Erratum. *J Abnorm Child Psychol.* 1990;18(1):119.

Reason for exclusion: Less 7 days treatment

Tannock1992

- Tannock R, Schachar R. Methylphenidate and cognitive perseveration in hyperactive children. *J Child Psychol Psychiatry.* 1992;33(7):1217-1228.

Reason for exclusion: Less than seven days treatment

Tannock1993

- Tannock R, Schachar R, Logan GD. Does methylphenidate induce overfocusing in hyperactive children? *J Clin Child Psychol.* 1993(1):28-4.

Reason for exclusion: Less 7 days treatment

Tannock1995a

- Tannock R, Ickowicz A, Schachar R. Differential effects of methylphenidate on working memory in ADHD children with and without comorbid anxiety. *J Am Acad Child Adolesc Psychiatry*. 1995;34(7):886-896.
- Subsample in: Tannock R, Fine J, Heintz T, Schachar RJ. A linguistic approach detects stimulant effects in two children with attention-deficit hyperactivity disorder. *J Child Adolesc Psychopharmacol.* 1995(3):177-189.

Reason for exclusion: Less than seven days treatment

Tannock1995b

- Tannock R, Schachar R, Logan G. Methylphenidate and cognitive flexibility: dissociated dose effects in hyperactive children. *J Abnorm Child Psychol.* 1995;23(2):235-266.

Reason for exclusion: Single dose

Tannock2000

- Tannock R, Martinussen R, Frijters J. Naming speed performance and stimulant effects indicate effortful, semantic processing deficits in attention-deficit/hyperactivity disorder. *J Abnorm Child Psychol.* 2000;28(3):237-252.

Reason for exclusion: Single dose study

Tannock2006

- Tannock R, Banaschewski T, Gold D. Color naming deficits and attention-deficit/hyperactivity disorder: a retinal dopaminergic hypothesis. *Behav Brain Funct.* 2006;2:4.

Reason for exclusion: Review

Taragin2013

- Taragin D, Berman S, Zelnik N, Karni A, Tirosh E. Parents' attitudes toward methylphenidate using n-of-1 trial: a pilot study. *Atten Defic Hyperact Disord.* 2013;5(2):105-109.

Reason for exclusion: N-1-of-trial

Taylor1993

- Taylor MJ, Voros JG, Logan WJ, Malone MA. Changes in event-related potentials with stimulant medication in children with attention deficit hyperactivity disorder. *Biol Psychol.* 1993;36(3):139-156.

Reason for exclusion: No available outcome for the present meta-analysis (confirmed by first author)

Taylor1997

- Taylor MJ, Sunohara GA, Khan SC, Malone MA. Parallel and serial attentional processes in ADHD: ERP evidence. *Child Neuropsychol.* 1997;13(4):531-539.

Reason for exclusion: No available outcome for the present meta-analysis (confirmed by first author)

TCTR20150228001

- http://www.clinicaltrials.in.th/index.php?tp=regtrials&menu=trialsearch&smenu=fulltext&task=search&task2=view1&id=1310

Reasons for exclusion: No participants with ADHD

TCTR20160512001

- http://www.clinicaltrials.in.th/index.php?tp=regtrials&menu=trialsearch&smenu=fulltext&task=search&task2=view1&id=1873

Reasons for exclusion: Methylphenidate vs. neurofeedback

Tec1971a

- Tec L. An additional observation on methylphenidate in hyperactive children. *Am J Psychiatry.* 1971;127(10):1424.

Reason for exclusion: Commentary

Tec1971b

- Tec L, Levy HB. Amphetamines in hyperkinetic children. *JAMA.* 1971;216(11):1864-1865.

Reason for exclusion: Commentary

Tehrani-Doost2008

- Tehrani-Doost M, Moallemi S, Shahrivar Z. An open-label trial of reboxetine in children and adolescents with attention-deficit/hyperactivity disorder. *J Child Adolesc Psychopharmacol.* 2008;18(2):179-184.

Reason for exclusion: Open label

Teicher2003

- Teicher MH, Polcari A, Anderson CM, Andersen SL, Lowen SB, Navalta CP. Rate dependency revisited: understanding the effects of methylphenidate in children with attention deficit hyperactivity disorder. *J Child Adolesc Psychopharmacol.* 2003;13(1):41-51.
- Teicher MH, Polcari A, McGreenery CE. Utility of objective measures of activity and attention in the assessment of therapeutic response to stimulants in children with attention-deficit/hyperactivity disorder. *J Child Adolesc Psychopharmacol.* 2008;18(3):265-270

Reason for exclusion: No outcomes of interest available; authors contacted to enquire if the two papers refer to the same study but no reply

Teicher2004

- Teicher MH, Lowen SB, Polcari A, Foley M, McGreenery CE. Novel strategy for the analysis of CPT data provides new insight into the effects of methylphenidate on attentional states in children with ADHD. *J Child Adolesc Psychopharmacol.* 2004;14(2):219-232.

Reason for exclusion: No RCT

Teicher2006
- Teicher MH, Polcari A, Foley M, et al. Methylphenidate blood levels and therapeutic response in children with attention-deficit hyperactivity disorder: I. Effects of different dosing regimens. *J Child Adolesc Psychopharmacol.* 2006;16(4):416-431.

Reason for exclusion: Single day study

Tenenbaum2002
- Tenenbaum S, Paull JC, Sparrow EP, Dodd DK, Green L. An experimental comparison of Pycnogenol and methylphenidate in adults with Attention-Deficit/Hyperactivity Disorder (ADHD). *J Atten Disord.* 2002;6(2):49-60.

Reason for exclusion: No usable data

Tenreiro2001
- Tenreiro KRF. Methylphenidate-Placebo: A Trial for Attention Deficit Disorders. *Int J Pharm Compd.* 2001;5(1):21-22.

Reason for exclusion: No RCT

Tepner2002
- Tepner R, Michelson D, Wernicke J, et al. Placebo controlled trials of atomoxetine for adhd in children, adolescents, and adults. *Int J Neuropsychopharmacol.* 2002(Suppl 1):S162.

Reason for exclusion: No RCT

Tervo2002
- Tervo RC, Azuma S, Fogas B, Fiechtner H. Children with ADHD and motor dysfunction compared with children with ADHD only.[Erratum appears in *Dev Med Child Neurol* 2002;44(9):622 Note: Dosage error in published abstract]. *Dev Med Child Neurol.* 2002;44(6):383-390.

Reason for exclusion: Less than seven days treatment

Tharoor2008
- Tharoor H, Lobos EA, Todd RD, Reiersen AM. Association of dopamine, serotonin, and nicotinic gene polymorphisms with methylphenidate response in ADHD. *Am J Med Genet B Neuropsychiatr Genet.* 2008;147B(4):527-530.

Reason for exclusion: No RCT

Thoenes2011
- Thoenes MM. Heat-related illness risk with methylphenidate use. *J Pediatr Health Care.* 2011;25(2):127-132.

Reason for exclusion: No RCT

Thomas2002
- Thomas S, Upadhyaya H. Adderall and seizures. *J Am Acad Child Adolesc Psychiatry.* 2002;41(4):365.

Reason for exclusion: Case report

Thompson2006
- Thompson AE, Nazir SA, Abbas MJ, Clarke J. Switching from immediate- to sustained- release psychostimulants in routine treatment of children with attention-deficit hyperactivity disorder. *Psychiatr Bull R Coll Psychiatr.* 2006;30(7):247-250.

Reason for exclusion: No RCT

Thomson1998
- Thomson JB, Varley CK. Prediction of stimulant response in children with attention- deficit/hyperactivity disorder. *J Child Adolesc Psychopharmacol.* 1998;8(2):125-132.

Reason for exclusion: No outcomes of interest for the present meta-analysis, not possible to contact authors (no email address)

Thurston1979

- Thurston CM, Sobol MP, Swanson J, Kinsbourne M. Effects of methylphenidate (Ritalin) on selective attention in hyperactive children. *J Abnorm Child Psychol.* 1979;7(4):471-481.

Reason for exclusion: No DSM/ICD criteria

Thurstone2010 (NCT00399763)

- Thurstone C, Riggs PD, Salomonsen-Sautel S, Mikulich-Gilbertson SK. Randomized, controlled trial of atomoxetine for attention-deficit/hyperactivity disorder in adolescents with substance use disorder. *J Am Acad Child Adolesc Psychiatry.* 2010;49(6):573-582.
- Thurstone C, Salomensen-Sautel S, Riggs PD. How adolescents with substance use disorder spend research payments. *Drug Alcohol Depend.* 2010;111(3):262-264.
- https://clinicaltrials.gov/ct2/show/NCT00399763

Reason for exclusion: Co-treatment (CBT)

Tillery2000

- Tillery KL, Katz J, Keller WD. Effects of methylphenidate (Ritalin) on auditory performance in children with attention and auditory processing disorders. *J Speech Lang Hear Res.* 2000;43(4):893-901.

Reason for exclusion: Single dose study

Tilton1998

- Tilton P. Bupropion and guanfacine. *J Am Acad Child Adolesc Psychiatry.* 1998;37(7):682-683.

Reason for exclusion: Commentary

Tirosh1993

- Tirosh E, Elhasid R, Kamah SC, Cohen A. Predictive value of placebo methylphenidate. *Pediatr Neurol.* 1993;9(2):131-133.

Reason for exclusion: No relevant outcomes for the present meta-analysis (in terms drop out, not possible to understand if 1 subject who dropped out was in the methylphenidate or placebo period; auhotr contacted but no reply)

Tirosh1993b

- Tirosh E, Sadeh A, Munvez R, Lavie P. Effects of methylphenidate on sleep in children with attention-deficit hyperactivity disorder: An activity monitor study. *Am J Dis Child.* 1993;147(12):1313-1315.

Reason for exclusion: No mention of randomization, no answer from author (written on 26.11.16 and again on 4.1.17); not possible to gather pre cross over data

Toren1997

- Toren P, Silbergeld A, Eldar S, et al. Lack of effect of methylphenidate on serum growth hormone (GH), GH-binding protein, and insulin-like growth factor I. *Clin Neuropharmacol.* 1997;20(3):264-269.

Reason for exclusion: No RCT

Torrioli2008

- Torrioli MG, Vernacotola S, Peruzzi L, et al. A double-blind, parallel, multicenter comparison of L-acetylcarnitine with placebo on the attention deficit hyperactivity disorder in fragile X syndrome boys. *Am J Med Genet A.* 2008;146(7):803-812.

Reason for exclusion: No drugs relevant for the present meta-analysis

Torrioli2010

- Torrioli MG, Vernacotola S, Setini C, et al. Treatment With Valproic Acid Ameliorates ADHD Symptoms in Fragile X Syndrome Boys. *Am J Med Genet A*. 2010;152A(6):1420-1427.

Reason for exclusion: No RCT; comorbidity with genetic syndrome

Tramontana2014

- Tramontana MG, Cowan RL, Zald D, Prokop JW, Guillamondegui O. Traumatic brain injury-related attention deficits: treatment outcomes with lisdexamfetamine dimesylate (Vyvanse). *Brain Inj.* 2014;28(11):1461-1472.

Reason for exclusion: No DSM/ICD criteria]

Trebaticka2006

- Trebaticka J, Kopasova S, Hradecna Z, et al. Treatment of ADHD with French maritime pine bark extract, Pycnogenol (R). *Eur Child Adolesc Psychiatry.* 2006;15(6):329-335.

- Dvoráková M, Jezová D, Blazícek P, Trebatická J, Skodácek I, Suba J, Iveta W, Rohdewald P, Duracková Z. Urinary catecholamines in children with attention deficit hyperactivity disorder (ADHD): modulation by a polyphenolic extract from pine bark (pycnogenol). *Nutr Neurosci*. 2007;10(3-4):151-7

Reason for exclusion: No drugs of interest for the present meta-analysis

Trommer1991

- Trommer BL, Hoeppner JA, Zecker SG. The go-no go test in attention deficit disorder is sensitive to methylphenidate. *J Child Neurol.* 1991;6 Issue 1:S128-131.

Reason for exclusion: No outcome of interest for the present meta-analysis; not possible to contact author

Trott1992

- Trott GE, Friese HJ, Menzel M, Nissen G. Use of moclobemide in children with attention deficit hyperactivity disorder. *Psychopharmacology (Berl).* 1992;106 (Supp l):S134-136.

Reason for exclusion: No drugs of interest for the present meta-analysis

Tsai2013

- Tsai C-S, Huang Y-S, Wu C-L, et al. Long-term effects of stimulants on neurocognitive performance of Taiwanese children with attention-deficit/hyperactivity disorder. *BMC Psychiatry.* 2013;13:330.

Reason for exclusion: No RCT

Tucha2001

- Tucha O, Lange KW. Effects of methylphenidate on kinematic aspects of handwriting in hyperactive boys. *J Abnorm Child Psychol.* 2001;29(4):351-356.

Reason for exclusion: No RCT

Tucha2004

- Tucha O, Lange KW. Handwriting and attention in children and adults with attention deficit hyperactivity disorder. *Motor control.* 2004;8(4):461-471.

Reason for exclusion: Less than seven days treatment

Tucha2005

- Tucha O, Lange KW. The effect of conscious control on handwriting in children with attention deficit hyperactivity disorder. *J Atten Disord.* 2005;9(1):323-332.

Reason for exclusion: No RCT

Tucha2006

- Tucha O, Prell S, Mecklinger L, et al. Effects of methylphenidate on multiple components of attention in children with attention deficit hyperactivity disorder. *Psychopharmacology (Berl).* 2006;185(3):315-326.

Reason for exclusion: Subjects were likely responder to previous treatment; query to author but no reply

Tucha2006a

- Tucha O, Mecklinger L, Laufkotter R, Klein HE, Walitza S, Lange KW. Methylphenidate-induced improvements of various measures of attention in adults with attention deficit hyperactivity disorder. *J Neural Transm.* 2006;113(10):1575-1592.

Reason for exclusion: Single dose study

Tucha2006b

- Tucha O, Prell S, Mecklinger L, et al. Effects of methylphenidate on multiple components of attention in children with attention deficit hyperactivity disorder. *Psychopharmacology (Berl).* 2006;185(3):315-326.

Reason for exclusion: Only variable usable would be drop out but rates of drop out not reported; written to author but no reply

Tucha2011

- Tucha L, Tucha O, Sontag TA, Stasik D, Laufkotter R, Lange KW. Differential effects of methylphenidate on problem solving in adults with ADHD. *J Atten Disord.* 2011;15(2):161-173.

Reason for exclusion: No RCT

Tucker2009

- Tucker JD, Suter W, Petibone DM, et al. Cytogenetic assessment of methylphenidate treatment in pediatric patients treated for attention deficit hyperactivity disorder. *Mutat Res.* 2009;677(1-2):53-58.
- Zhou Y, Muni R, Tucker JD, Kumar V. Extendedrelease methylphenidate exposure and the frequency of cytogenetic abnormalities in children with attention-deficithyperactivity disorder. *J Child Adolesc Psychopharmacol. Proceedings of the 49th Annual National Institute of Mental Health (NIMH) New Clinical Drug Evaluation Unit (NCDEU) Meeting; 2009 June 29- July 2;Hollywood, Florida 2009;19(6):785.*

Reason for exclusion: Co-intervention (behavioural therapy)

Turner2004

- Turner DC, Clark L, Dowson J, Robbins TW, Sahakian BJ. Modafinil improves cognition and response inhibition in adult attention-deficit/hyperactivity disorder. *Biol Psychiatry.* 2004;55(10):1031-1040.

Reason for exclusion: Single dose

Turner2005

- Turner DC, Blackwell AD, Dowson JH, McLean A, Sahakian BJ. Neurocognitive effects of methylphenidate in adult attention-deficit/hyperactivity disorder. *Psychopharmacology (Berl).* 2005;178(2-3):286-295.

Reason for exclusion: Single dose

Tutee2007

- Tutee O, Tutee L, Waltz S, Stasik D, Laufkotter R, Gerlach M, Klein HE, Lange KW. Differential effects of methylphenidate on problem solving of adults with attention deficit hyperactivity disorder. *J Neural Transm.* 2007; 114: 1004

Reason for exclusion: No RCT

Ullmann1978

Ullman DG, Barkley RA, Brown HW. The behavioral symptoms of hyperkinetic children who successfully responded to stimulant drug treatment. *Am J Orthopsychiatry*. 1978;48(3):425-437.

Reason for exclusion: No DSM/ICD criteria

Ullmann1985

- Ullmann RK, Sleator EK. Attention deficit disorder children with or without hyperactivity. *Clin Pediatr (Phila).* 1985;24(10):547-551.

Reason for exclusion: "Random" not mentioned in the paper; not possible to contact authors to clarify; No pre cross-over data available*

Ullmann1986

- Ullmann RK, Sleator EK. Responders, nonresponders, and placebo responders among children with attention deficit disorder. Importance of a blinded placebo evaluation. *Clin Pediatr (Phila).* 1986;25(12):594-599.

Reason for exclusion: "Random" not mentioned in the paper; not possible to contact authors to clarify; No pre cross-over data available*

Upadhyaya2005

- Upadhyaya HP, Rose K, Wang W, O'Rourke K, Sullivan B, Deas D, Brady KT. Attention-deficit/ hyperactivity disorder, medication treatment, and substance use patterns among adolescents and young adults. *J Child Adolesc Psychopharmacol*. 2005; 15(5): 799-809

Reason for exclusion: No RCT

Upadhyaya2006

- Upadhyaya HP. Methylphenidate and pramipexole drug effects in adolescents and young adults with attention deficit hyperactivity disorder (ADHD) and nicotine dependence. *Neuropsychopharmacology.* 2006; 31(Suppl. 1): 139

Reason for exclusion: No RCT

Upadhyaya2013 (NCT00700427)

- Upadhyaya H, Adler LA, Kutzelnigg A, Williams D, Tanaka Y, Arsenault J. Characteristics of adult patients with adhd in europe compared with non-european adult patients with ADHD participating in a large treatment study with atomoxetine. *Eur Psychiatry.* 2012;27.
- Upadhyaya HP, Camporeale A, Ramos-Quiroga JA, et al. Safety and tolerability of atomoxetine hydrochloride in a placebo-controlled randomized withdrawal study in adults with attention-deficit/hyperactivity disorder. *Neuropsychopharmacology.* 2012;38:S318.

- Upadhyaya H, Ramos-Quiroga JA, Williams D, et al. Maintenance of response after open-label treatment with atomoxetine in adults with attention-deficit/hyperactivity disorder. *Eur Neuropsychopharmacol.* 2012;22:S427-S428.
- Guo Y, Fijal B, Marshall S, et al. Comparison of efficacy and safety between intermediate and extensive/ultra-rapid metabolizers of atomoxetine in adult patients with attention-deficit hyperactivity disorder participating in a large placebo-controlled maintenance of response clinical trial. *Clin Pharmacol Ther.* 2013;93:S29 (2013 Annual Meeting of the American Society for Clin Pharmacol Ther, ASCPT 2013)
- Upadhyaya H, Ramos-Quiroga JA, Adler LA, et al. Maintenance of response after open-label treatment with atomoxetine hydrochloride in international European and non-European adult outpatients with attention-deficit/hyperactivity disorder: A placebo-controlled, randomised withdrawal study. *Eur J Psychiatry.* 2013(3):185-205.
- Camporeale A, Upadhyaya H, Ramos-Quiroga JA, et al. Safety and tolerability of atomoxetine hydrochloride in a long-term, placebo-controlled randomized withdrawal study in European and Non-European adults with attention-deficit/ hyperactivity disorder. *Eur J Psychiatry.* 2013(3):206-224.
- Upadhyaya H, Adler LA, Casas M, et al. Baseline characteristics of European and non-European adult patients with attention deficit hyperactivity disorder participating in a placebo-controlled, randomized treatment study with atomoxetine. *Child Adolesc Psychiatry Ment Health.* 2013;7(1):14.
- Fijal BA, Guo Y, Li SG, et al. CYP2D6 predicted metabolizer status and safety in adult patients with attention-deficit hyperactivity disorder participating in a large placebo-controlled atomoxetine maintenance of response clinical trial. *J Clin Pharmacol.* 2015;55(10):1167-1174.
- Upadhyaya H, Tanaka Y, Williams D, Escobar R, Leppamaki S. Long-term open-label treatment with atomoxetine in European adult outpatients with Attention-Deficit/Hyperactivity Disorder. *ADHD Atten Defic Hyperact Disord.* 2015;7:S52.
- Upadhyaya H, Tanaka Y, Lipsius S, et al. Time-to-onset and -resolution of adverse events before/after atomoxetine discontinuation in adult patients with ADHD. *Postgrad Med.* 2015;127(7):677-685.
- Adler LA, Solanto M, Escobar R, Lipsius S, Upadhyaya H. Executive Functioning Outcomes Over 6 Months of Atomoxetine for Adults With ADHD: Relationship to Maintenance of Response and Relapse Over the Subsequent 6 Months After Treatment. *J Atten Disord.* 2016.

Reason for exclusion: Participants were" responders" from open label phase

Urman1985

- Urman R, Ickowicz A, Fulford P, Tannock R. An exaggerated cardiovascular response to methylphenidate in ADHD children with anxiety. *J Child Adol Psychopharmacol.* 1995(1):29-37.

Reason for exclusion: Less than 7-day treatment

Vaidya1998

- Vaidya CJ, Austin G, Kirkorian G, et al. Selective effects of methylphenidate in attention deficit hyperactivity disorder: a functional magnetic resonance study. *Proc Natl Acad Sci U S A.* 1998;95(24):14494-14499.

Reason for exclusion: No RCT; single dose

Vakula2009

- Vakula IN, Vasianina IS, Gorbunova ZK, Nikiforova EI, Ponomarenko EI. [Effectiveness of strattera in children and adolescents with attention deficit hyperactivity disorder]. *Zh Nevrol Psikhiatr Im S S Korsakova.* 2009;109(8):42-44.

Reason for exclusion: No RCT

Valdizan2004

- Valdizan JR. [The diagnostic evaluation and therapeutic basis of immediate release methylphenidate in attention deficit hyperactivity disorder]. *Rev Neurol.* 2004;38(6):501-506.

Reason for exclusion: No RCT

Valdizan2007

- Valdizan JR, Mercado E, Mercado-Undanivia A. [Clinical variability and characteristics of attention deficit hyperactivity disorder in girls]. *Rev Neurol.* 2007;44(2):S27-30.

Reason for exclusion: No RCT

Vallee2000

- Vallee L. Attention deficit disorder with hyperactivity in children: diagnosis and therapeutic management. [French] *Arch Pediatr.* 2000 ;7(10):1111-6

Reason for exclusion: Review, no empirical data

Van2006

- Van der Feltz-Cornelis CM, Aldenkamp AP. Effectiveness and safety of methylphenidate in adult attention deficit hyperactivity disorder in patients with epilepsy: an open treatment trial. *Epilepsy Behav.* 2006;8(3):659-662.

Reason for exclusion: No double blind

Van2011

- Van de Loo-Neus GHH, Rommelse N, Buitelaar JK. To stop or not to stop? How long should medication treatment of attention-deficit hyperactivity disorder be extended? *Eur Neuropsychopharmacol.* 2011;21(8):584-599.

Reason for exclusion: Review; no empirical data

Van Ameringen2016

- Van Ameringen M, Patterson B, Simpson W, Turna J, Pullia K. Adult ADHD with anxiety disorder and depression comorbidity in a clinical trial cohort. *Neuropsychopharmacology.* 2016;41:S486-S487.

Reason for exclusion: Ongoing study, data not available

Van der Meere1995

- Van der Meere J, Shalev R, Borger N, Gross-Tsur V. Sustained attention, activation and MPH in ADHD: a research note. *J Child Psychol Psychiatry.* 1995;36(4):697-703.

Reason for exclusion: Second author confirmed it is a single dose study

Van der Meere2009

- Van der Meere JJ, Shalev RS, Borger N, Wiersema JR. Methylphenidate, interstimulus interval, and reaction time performance of children with attention deficit/hyperactivity disorder: a pilot study. *Child Neuropsychol.* 2009;15(6):554-566.

Reason for exclusion: Single dose

Van der Oord2007

- Van der Oord S, Prins PJ, Oosterlaan J, Emmelkamp PM. Does brief, clinically based, intensive multimodal behavior therapy enhance the effects of methylphenidate in children with ADHD? *Eur Child Adolesc Psychiatry.* 2007;16(1):48-57.

Reason for exclusion: Pseudo-randomized, open label, no arms of interest for the present meta-analysis

Van der Oord2008a

- Van der Oord S, Prins PJ, Oosterlaan J, Emmelkamp PM. Efficacy of methylphenidate, psychosocial treatments and their combination in school-aged children with ADHD: a meta-analysis. *Clin Psychol Rev.* 2008;28(5):783-800.

Reason for exclusion: Meta-analysis; no additional empirical data

Van der Oord2008

- Van der Oord S, Prins PJ, Oosterlaan J, Emmelkamp PM. Treatment of attention deficit hyperactivity disorder in children. Predictors of treatment outcome. *Eur Child Adolesc Psychiatry.* 2008;17(2):73-81.

Reason for exclusion: Pseudo-randomized

Van der Oord2012

- Van der Oord S, Geurts HM, Prins PJ, Emmelkamp PM, Oosterlaan J. Prepotent response inhibition predicts treatment outcome in attention deficit/hyperactivity disorder. *Child Neuropsychol.* 2012;18(1):50-61.

Reason for exclusion: Pseudo-randomized

Van der Oord2012

- Van der Oord S, Prins PJ, Oosterlaan J, Emmelkamp PM. The adolescent outcome of children with attention deficit hyperactivity disorder treated with methylphenidate or methylphenidate combined with multimodal behaviour therapy: results of a naturalistic follow-up study. *Clin Psychol Psychother.* 2012;19(3):270-278.

Reason for exclusion: No RCT

Van der Schaaf2013

- Van der Schaaf ME, Fallon SJ, Ter Huurne N, Buitelaar J, Cools R. Working memory capacity predicts effects of methylphenidate on reversal learning. *Neuropsychopharmacology.* 2013;38(10):2011-2018.

Reason for exclusion: No participants with ADHD

Van Dyck1997

- Van Dyck, CH, McMahon, TJ, Rosen, MI, O'Malley, SS, O'Connor,PG, Lin, CH, Pearsall, HR, Woods, SW, Kosten, TR. Sustained-release methylphenidate for cognitive impairment inHIV-1-infected drug abusers: a pilot study. *J Neuropsychiatry Clin Neurosci.* 1997*;* 9(1): 29-36

Reason for exclusion: No participants with ADHD

Van Mourik2015
- Van Mourik R, Gelade K, Janssen T, Bink M, Maras A, Oosterlaan J. Train your brain: The effectiveness of neurofeedback compared to medication and physical exercise in ADHD. *Eur Child Adolesc Psychiatry.* 2015;1:S44.

Reason for exclusion: Arms of no interest for the present meta-analysis

Van Reekum1994
- Van Reekum R, Links PS. N of 1 study: Methylphenidate in a patient with borderline personality disorder and attention deficit hyperactivity disorder. *Can J Psychiatry.* 1994;39(3):186-187.

Reason for exclusion: N-of-1 trial

Van Stralen2015
- Van Stralen J, Corsi E. The effect of GXR (guanfacine) as adjunctive treatment with stimulant therapy on executive function and quality of life: A phase IV, single center, randomized, double blind, placebo controlled, crossover evaluation. *ADHD Atten Defic Hyperact Disord.* 2015;7:S98.

Reason for exclusion: Guanfacine extended release as ddd on treatment to stimulants

Van Wyk2012
- Van Wyk GW, Hazell PL, Kohn MR, Granger RE, Walton RJ. How oppositionality, inattention, and hyperactivity affect response to atomoxetine versus methylphenidate: a pooled meta-analysis. *J Atten Disord.* 2012;16(4):314-324.

Reason for exclusion: Meta-analysis; no additional empirical data

Vansickel2007
- Vansickel AR, Stoops WW, Glaser PEA, Rush CR. A pharmacological analysis of stimulant-induced increases in smoking. *Psychopharmacology (Berl).* 2007;193(3):305-313.

Reason for exclusion: No RCT; No participants with ADHD; Single dose

Vansickel2011
- Vansickel AR, Stoops WW, Glaser PE, Poole MM, Rush CR. Methylphenidate increases cigarette smoking in participants with ADHD. *Psychopharmacology (Berl).* 2011;218(2):381-390.

Reason for exclusion: No RCT; Single dose

Varley1982
- Varley CK, Trupin EW. Double-blind administration of methylphenidate to mentally retarded children with attention deficit disorder; a preliminary study. *Am J Ment Defic.* 1982;86(6):560-566.

Cross-over without wash out; pre-cross over data not available

Varley1983
- Varley CK. Effects of methylphenidate in adolescents with attention deficit disorder. *J Am Acad Child Psychiatry.* 1983;22(4):351-354.

Cross-over without wash out; pre-cross over data not available

Varley1983
- Ballinger CT, Varley CK, Nolen PA. Effects of methylphenidate on reading in children with attention deficit disorder. *Am J Psychiatry.* 1984;141(12):1590-1593.
- Varley CK, Trupin EW. Double-blind assessment of stimulant medication for attention deficit disorder: a model for clinical application. *Am J Orthopsychiatry.* 1983;53(3):542-547

Reason for exclusion: Cross-over without wash out; pre-cross over data not available

Varley2001
- Varley CK, Vincent J, Varley P, Calderon R. Emergence of tics in children with attention deficit hyperactivity disorder treated with stimulant medications. *Comprehensive Psychiatry.* 2001;42(3):228-233.

Reason for exclusion: No RCT

Vaughan2008

- Vaughan BS, Wetzel MW, Kratochvil CJ. Beyond the 'typical' patient: treating attention-deficit/hyperactivity disorder in preschoolers and adults. *International Review of Psychiatry.* 2008;20(2):143-149.

Reason for exclusion: Review, no empirical data

Vaughan2009

- Vaughan B, Fegert J, Kratochvil CJ. Update on atomoxetine in the treatment of attention-deficit/hyperactivity disorder. *Expert Opin Pharmacother.* 2009;10(4):669-676.

Reason for exclusion: Review, no empirical data

Vaughan2012

- Vaughan B, Kratochvil CJ. Pharmacotherapy of pediatric attention-deficit/hyperactivity disorder. *Child Adolesc Psychiatr Clin N Am.* 2012;21(4):941-955.

Reason for exclusion: Review, no empirical data

Vaughan2012

- Vaughan BS, March JS, Kratochvil CJ. The evidence-based pharmacological treatment of paediatric ADHD. *Int J Neuropsychopharmacol.* 2012;15(1):27-39.

Reason for exclusion: Review, no empirical data

Velasquez-Tirado2005

- Velasquez-Tirado JD, Pena JA. Current evidence about atomoxetine. A therapeutic alternative for the treatment of attention deficit hyperactivity disorder. [Spanish] *Rev Neurol.* 200516-31;41(8):493-500

Reason for exclusion: Review, no empirical data

Velcea2004

- Velcea G, Winsberg BG. Atomoxetine and nonresponders to stimulants. *Am J Psychiatry.* 2004;161(9):1718-1719.

Reason for exclusion: No RCT

Verbaten1994

- Verbaten MN, Overtoom CC, Koelega HS, et al. Methylphenidate influences on both early and late ERP waves of ADHD children in a continuous performance test. *J Abnorm Child Psychol.* 1994;22(5):561-578.

Reason for exclusion: Single dose

Verbeeck2011

- Verbeeck W, Bekkering Geertruida E, Van den Noortgate W. Bupropion for Attention Deficit Hyperactivity Disorder (ADHD) in adults. *Cochrane Database Syst Rev.* 2011(12).

Reason for exclusion: Protocol of a meta-analysis; no empirical data

Verret2010

- Verret C, Gardiner P, Beliveau L. Fitness level and gross motor performance of children with attention-deficit hyperactivity disorder. *Adapted Physical Activity Quarterly.* 2010;27(4):337-351.
- Verret C. *Condition physique, performance motrice, comportements et fonctions cognitives chez les enfants ayant un trouble du deficit de l'attention avec hyperactivite* [Ph.D.]. Ann Arbor, Universite de Montreal (Canada); 2010.

Reason for exclusion: No RCT

Verster2008 (NCT00223561)

- Verster JC, Bekker EM, de Roos M, Minova A, Eijken EJE, Kooij JJS, Buitelaar JK, Kenemans JL, Verbaten MN, et a, Suppl. Driving ability in adults with attention-deficit hyperactivity disorder significantly improves when treated with methylphenidate. *Eur Neuropsychopharmacol*. 2006; 16: 8-39
- Verster JC, Bekker EM, de Roos M, et al. Methylphenidate significantly improves driving performance of adults with attention-deficit hyperactivity disorder: a randomized crossover trial. *J Psychopharmacol.* May 2008;22(3):230-237.
- Verster JC, Bekker EM, Kooij JJ, et al. Methylphenidate significantly improves declarative memory functioning of adults with ADHD. *Psychopharmacology (Berl).* 2010;212(2):277-281.
- Verster JC, Roth T. Methylphenidate significantly reduces lapses of attention during on-road highway driving in patients with ADHD. *J Clin Psychopharmacol.* 2014;34(5):633-636.
- https://clinicaltrials.gov/ct2/show/NCT00223561

Reason for exclusion: Single dose; patients "optimised", Less than seven days treatment

Vickers2002

- Vickers JN, Rodrigues ST, Brown LN. Gaze pursuit and arm control of adolescent males diagnosed with attention deficit hyperactivity disorder (ADHD) and normal controls: evidence of a dissociation in processing visual information of short and long duration. *J Sports Sci.* 2002;20(3):201-216.

Reason for exclusion: No RCT

Victor2009

- Victor MM, Grevet EH, Salgado CAI, et al. Reasons for pretreatment attrition and dropout from methylphenidate in adults with attention-deficit/hyperactivity disorder: the role of comorbidities. *J Clin Psychopharmacol.* 2009;29(6):614-616.

Reason for exclusion: No RCT

Vincent1990

- Vincent J, Varley CK, Leger P. Effects of methylphenidate on early adolescent growth. *Am J Psychiatry.* 1990;147(4):501-502.

Reason for exclusion: No RCT

Vinson1994

- Vinson DC. Therapy for attention-deficit hyperactivity disorder. *Archives of Family Medicine.* 1994;3(5):445-451.

Reason for exclusion: Review, no additional empirical data

Vitiello2001

- Vitiello B. Methylphenidate in the treatment of children with attention-deficit hyperactivity disorder. *CMAJ.* 2001;165(11):1505-1506.

Reason for exclusion: Commentary, no additional empirical data

Vitiello2008a

- Vitiello B. Improving decision making in the treatment of ADHD. *Am J Psychiatry.* 2008;165(6):666-667.

Reason for exclusion: Commentary, no additional empirical data

Vitiello2008b

- Vitiello B. Understanding the risk of using medications for attention deficit hyperactivity disorder with respect to physical growth and cardiovascular function. *Psychiatr Clin North Am.* 2008;17(2):459-474, xi.

Reason for exclusion: Review, no additional empirical data

Voelker1983

- Voelker S, Lachar D, Gdowski CL. The Personality Inventory for Children and response to methylphenidate: preliminary evidence for predictive utility. *J Pediatr Psychol.* 1983;8(2):161-169.

Reason for exclusion: No RCT

Vogt2011

- Vogt C, Williams T. Early identification of stimulant treatment responders, partial responders and non-responders using objective measures in children and adolescents with hyperkinetic disorder. *Child and Adolescent Mental Health.* 2011;16(3):144-149.

Reason for exclusion: Single dose

Voigt2001

- Voigt RG, Llorente AM, Jensen CL, Fraley JK, Berretta MC, Heird WC. A randomized, double-blind, placebo-controlled trial of docosahexaenoic acid supplementation in children with attention-deficit/hyperactivity disorder. *Journal of Pediatrics.* 2001;139(2):189-196.

Reason for exclusion: No arms of interest for the present meta-analysis (supplementation with fatty acids or placebo). Commentary, no additional empirical data

Volkmar1985

- Volkmar F, Hoder E, Cohen D. Inappropriate use of stimulant medications. *Clin Pediatr.*1985;24:127–30.

Reason for exclusion: Case reports,

Volkow2003

- Volkow ND, Insel TR. What are the long-term effects of methylphenidate treatment? *Biol Psychiatry.* 2003;54(12):1307-1309.

Reason for exclusion: Commentary, no additional empirical data

Volkow2008
- Volkow ND, Fowler JS, Wang GJ, et al. Methylphenidate decreased the amount of glucose needed by the brain to perform a cognitive task. *PLoS One.* 2008;3(4):e2017.

Reason for exclusion: No participants with ADHD; Single dose

Výborová1984a
- Výborová, L, Náhunek K, Mišurec J, Drtílková I, Balaštíková B, Šestáková I. Comparison of amphetaminil and methylphenidate in the treatment of hyperkinetic syndrome in children. *Activitas Nervosa Superior* 1984; 26, 1, 58

Reason for exclusion: Single blind

Výborová1984b
- Výborová L, Náhunek K, Drtílková I, Balaštíková B, Mišurec J. Amphetaminil and methylphenidate in hyperkinetic children: analysis of therapeutic results and EEG changes. *Activitas Nervosa Superior* 1984; 27, 4, 304-306

Reason for exclusion: Single blind trial

Vyse1989
- Vyse SA, Rapport MD. The effects of methylphenidate on learning in children with ADDH: the stimulus equivalence paradigm. *J Consult Clin Psychol.* 1989;57(3):425-435.

Reason for exclusion: Single dose

Wade1976
- Wade MG. Effects of methylphenidate on motor skill acquisition of hyperactive children. *J Learn Disabil.* 1976;9(7):443-447.

Reason for exclusion: Single dose

Wagner2001
- Wagner MW, Markowitz JS, Patrick KS. Methylphenidate ER tablet lodging in esophagus. *J Am Acad Child Adolesc Psychiatry.* 2001;40(11):1244-1245.

Reason for exclusion: Case report

Waldon2016
- Waldon J, Begum E, Gendron M, et al. Concordance of actigraphy with polysomnography in children with and without attention-deficit/hyperactivity disorder. *J Sleep Res.* 2016;25(5):524-533.

Reason for exclusion: Contac with senior author: data other than sleep still being analyzed.

Walitza2007
- Walitza S, Werner B, Romanos M, Warnke A, Gerlach M, Stopper H. Does methylphenidate cause a cytogenetic effect in children with attention deficit hyperactivity disorder? *Environ Health Perspect.* 2007;115(6):936-940.

Reason for exclusion: No RCT

Walitza2009
- Walitza S, Kampf K, Artamonov N, et al. No elevated genomic damage in children and adolescents with attention deficit/hyperactivity disorder after methylphenidate therapy. *Toxicol Lett.* 2009;184(1):38-43.

Reason for exclusion: No RCT

Walitza2010
- Walitza S, Kampf K, Oli RG, Warnke A, Gerlach M, Stopper H. Prospective follow-up studies found no chromosomal mutagenicity of methylphenidate therapy in ADHD affected children. *Toxicol Lett.* 2010;193(1):4-8.

Reason for exclusion: No RCT

Walker1988
- Walker MK, Sprague RL, Sleator EK, Ullmann RK. Effects of methylphenidate hydrochloride on the subjective reporting of mood in children with attention deficit disorder. *Issues in Mental Health Nursing.* 1988(9):373-385.

Reason for exclusion: Single dose

Wallace1994

- Wallace AE, Kofoed LL. Statistical analysis of single case studies in the clinical setting: The example of methylphenidate trials in children with attention-deficit hyperactivity disorder. *J Child Adolesc Psychopharmacol.* 1994;4(3):141-150.

Reason for exclusion: Series of N-of-1 trials. Placebo: 3-6 days.

Wallander1987

- Wallander JL, Schroeder SR, Michelli JA, Gualtieri CT. Classroom social interactions of attention deficit disorder with hyperactivity children as a function of stimulant medication. *J Pediatr Psychol.* 1987;12(1):61-76.

Reason for exclusion: No pre cross-over data

Walsh2003

- Walsh DJ. Upping the Ritalin: fiction. *Neurology.* 2003;60(9):1555-1557.

Reason for exclusion: Commentary

Walsh2013

- Walsh SL, Middleton LS, Wong CJ, et al. Atomoxetine does not alter cocaine use in cocaine dependent individuals: A double blind randomized trial. *Drug Alcohol Depend.* 2013;130(1-3):150-157.

Reason for exclusion: No inclusion of participants with ADHD

Wang1985

- Wang YF. [Urinary 3-methoxy-4-hydroxyphenylglycol sulfate in school children with minimal brain dysfunction syndrome]. *Chung-Hua Shen Ching Ching Shen Ko Tsa Chih [Chinese Journal of Neurology & Psychiatry].* 1985;18(1):45-49.

Reason for exclusion: No DSM/ICD criteria

Wang2011a

- Wang L-J, Huang Y-S, Chiang Y-L, Hsiao C-C, Shang Z-Y, Chen C-K. Clinical symptoms and performance on the Continuous Performance Test in children with attention deficit hyperactivity disorder between subtypes: a natural follow-up study for 6 months. *BMC Psychiatry*. 2011;11:65.

Reason for exclusion: No RCT

Wang2011b

- Wang LJ, Hsiao CC, Huang YS, et al. Association of salivary dehydroepiandrosterone levels and symptoms in patients with attention deficit hyperactivity disorder during six months of treatment with methylphenidate. *Psychoneuroendocrinology.* 2011(8):1209-1216.

Reason for exclusion: No RCT

Wang2013

- Wang G-J, Volkow ND, Wigal T, et al. Long-term stimulant treatment affects brain dopamine transporter level in patients with attention deficit hyperactive disorder. *PLoS ONE [Electronic Resource].* 2013;8(5):e63023.

Reason for exclusion: No RCT

Ward1997

- Ward AS, Kelly TH, Foltin RW, Fischman MW. Effects of d-amphetamine on task performance and social behavior of humans in a residential laboratory. *Exp Clin Psychopharmacol.* 1997;5(2):130-136.

Reason for exclusion: No participants with ADHD

Warikoo2013

- Warikoo N, Faraone SV. Background, clinical features and treatment of attention deficit hyperactivity disorder in children. *Expert Opin Pharmacother.* 2013;14(14):1885-1906.

Reason for exclusion: Systematic review

Warneke1990

- Warneke L. Psychostimulants in psychiatry. *Can J Psychiatry.* 1990;35(1):3-10.

Reason for exclusion: Review

Warshaw2010 (NCT00434213)

- Warshaw EM, Squires L, Li Y, Civil R, Paller AS. Methylphenidate transdermal system: a multisite, open-label study of

dermal reactions in pediatric patients diagnosed with ADHD. *Prim Care Companion J Clin Psychiatry.* 2010;12(6).
- https://clinicaltrials.gov/ct2/show/NCT00434213

Reason for exclusion: No RCT, no double blind

Waschbusch2007

- Waschbusch DA, Craig R, Pelham WE, Jr., King S. Self-handicapping prior to academic-oriented tasks in children with attention deficit/hyperactivity disorder (ADHD): medication effects and comparisons with controls. *J Abnorm Child Psychol.* 2007;35(2):275-286.

Reason for exclusion: Single dose

Watter1973

- Watter N, Dreifuss FE. Modification of hyperkinetic behavior by nortriptyline. *Virginia Medical Monthly.* 1973;100(2):123-126.

Reason for exclusion: No RCT

Waxmonsky2005

- Waxmonsky JG. Nonstimulant therapies for attention-deficit hyperactivity disorder (ADHD) in children and adults. *Essent Psychopharmacol.* 2005;6(5):262-276.

Reason for exclusion: Review

Waxmonsky2008

- Waxmonsky J, Pelham WE, Gnagy E, et al. The efficacy and tolerability of methylphenidate and behavior modification in children with attention-deficit/hyperactivity disorder and severe mood dysregulation. *J Child Adolesc Psychopharmacol.* 2008;18(6):573-588.

Reason for exclusion: Less than seven days treatment

Waxmonsky2010 (NCT00918567; B4Z-US-X053)

- Waxmonsky JG, Waschbusch DA, Pelham WE, Draganac-Cardona L, Rotella B, Ryan L. Effects of atomoxetine with and without behavior therapy on the school and home functioning of children with attention-deficit/hyperactivity disorder. *The J Clin Psychiatry.* 2010;71(11):1535-1551.
- Post hoc analysis in: Waxmonsky JG, Waschbusch DA, Akinnusi O, Pelham WE. A comparison of atomoxetine administered as once versus twice daily dosing on the school and home functioning of children with attention-deficit/hyperactivity disorder. *J Child Adolesc Psychopharmacol.* 2011;21(1):21-32.
- https://clinicaltrials.gov/ct2/show/NCT00918567

Reason for exclusion: Atomoxetine vs Atomoxetine +CBT; open label

Waxmonsky2014 (NCT01127607)

- Babinski DE, Waxmonsky JG, Pelham WE: Treating parents with attention-deficit/hyperactivity disorder: The effects of behavioral parent training and acute stimulant medication treatment on parent–child interactions. *J Abnorm Child Psychol.* 2014;42(7):1129–1140.
- Babinski DE, Waxmonsky JG, Waschbusch DA, Humphery H, Pelham WE, Jr. Parent-Reported Improvements in Family Functioning in a Randomized Controlled Trial of Lisdexamfetamine for Treatment of Parental Attention-Deficit/Hyperactivity Disorder. *J Child Adolesc Psychopharmacol.* 2017;27(3):250-257.
- Waxmonsky JG, Waschbusch DA, Babinski DE, et al. Does pharmacological treatment of ADHD in adults enhance parenting performance? Results of a double-blind randomized trial. *CNS Drugs.* 2014;28(7):665-677.
- https://clinicaltrials.gov/ct2/show/NCT01127607

Reason for exclusion: Optimization phase before randomized phase

Weaver1996

- Weaver A. Attention deficit disorder. *British Journal of Psychiatry.* 1996;169(4):523.

Reason for exclusion: Commentary

Weber1975

- Weber BA, Sulzbacher SI. Use of CNS stimulant medication in averaged electroencephalic audiometry with children with MBD. *J Learn Disabil.* 1975;8(5):300-303.

Reason for exclusion: No DSM/ICD criteria

Weber1977

- Weber A. [Special schooling, education counseling psychotherapy and pharmacotherapy in children with minimal brain damage]. *Therapeutische Umschau.* 1977;34(1):24-28.

Reason for exclusion: No DSM/ICD criteria

Weber1985

- Weber K. Methylphenidate: rate-dependent drug effects in hyperactive boys. *Psychopharmacology (Berl).* 1985;85(2):231-235.
- Weber KA. *Effects of methylphenidate on operant responding in* hyperactive *boys* [Ph.D.]. Ann Arbor, The University of Iowa; 1980.

Reason for exclusion: Single dose

Weber1992

- Weber KS, Frankenberger W, Heilman K. The effects of Ritalin on the academic achievement of children diagnosed with attention-deficit hyperactivity disorder. *Developmental Disabilities Bulletin.* 1992;20(2):49-68.

Reason for exclusion: No RCT

Weber2002

- Weber P, Lutschg J. Methylphenidate treatment. *Pediatr Neurol.* 2002;26(4):261-266.

Reason for exclusion: Review

Weber2003

- Weber P, Bubl R, Lutschg J. Side effects of methylphenidate in children. Prevalence and associated factors. *Monatsschrift Kinderheilkunde.* 2003;151(4):399-404.

Reason for exclusion: No RCT

Weber2007

- Weber P, Lutschg J, Fahnenstich H. Methylphenidate-induced changes in cerebral hemodynamics measured by functional near-infrared spectroscopy. *J Child Neurol.* 2007;22(7):812-817.

Reason for exclusion: No RCT

Weber2008

- Weber W, Vander Stoep A, McCarty RL, Weiss NS, Biederman J, McClellan J. Hypericum perforatum (St John's Wort) for attention-deficit/hyperactivity disorder in children and adolescents - A randomized controlled trial. *Jama.* 2008;299(22):2633-2641.

Reason for exclusion: Compound (Hypericum perforatum) of non interest for the present meta-analysis vs placebo

Weber2009

- Weber J, Siddiqui MA. Lisdexamfetamine dimesylate: in attention-deficit hyperactivity disorder in adults. *CNS Drugs.* 2009;23(5):419-425.

Reason for exclusion: Review

Wehmeier2008

- Wehmeier PM, Schacht A, Dittmann RW, et al. Global impression of perceived difficulties in children and adolescents with attention-deficit/hyperactivity disorder: reliability and validity of a new instrument assessing perceived difficulties from a patient, parent and physician perspective over the day. *Child Adolesc Psychiatry Ment Health* 2008; 2 (1): 10

Reason for exclusion: Open label

Wehmeier2010 (NCT00191737; NCT00191516; B4Z-SB-LYDE)

- Dittmann RW, Wehmeier PM, Schacht A, et al. Atomoxetine treatment and ADHD-related difficulties as assessed by adolescent patients, their parents and physicians. *Child Adolesc Psychiatry Ment Health.* 2009;3(1):21.
- Wehmeier PM, Dittmann RW, Schacht A, et al. Effectiveness of atomoxetine and quality of life in children with attention-deficit/hyperactivity disorder as perceived by patients, parents, and physicians in an open-label study. *J Child Adolesc Psychopharmacol.* 2007;17(6):813-830.
- Wehmeier PM, Schacht A, Dittmann RW, Banaschewski T. Minor differences in ADHD-related difficulties between boys and girls treated with atomoxetine for attention-deficit/hyperactivity disorder. *Atten Defic Hyperact Disord.* 2010;2(2):73-85.
- https://clinicaltrials.gov/ct2/show/NCT00191737
- https://clinicaltrials.gov/ct2/show/NCT00191516

Reason for exclusion: Post hoc analysis of two open label studies

Weingartner1980

- Weingartner H, Rapoport JL, Buchsbaum MS. Cognitive processes in normal and hyperactive children and their response to amphetamine treatment. *J Abnorm Psychol.* 1980(1):25-37.

Reason for exclusion: Single dose; No mention of randomization

Weingartner1982

- Weingartner H, Langer D, Grice J, Rapoport JL. Acquisition and retrieval of information in amphetamine-treated hyperactive children. *Psychiatry Res.* 1982;6(1):21-29.

Reason for exclusion: Less than seven days treatment

Weisler2005

- Weisler RH. Safety, efficacy and extended duration of action of mixed amphetamine salts extended-release capsules for the treatment of ADHD. *Expert Opin Pharmacother.* 2005;6(6):1003-1018.

Reason for exclusion: Review

Weisler2007a

- Weisler RH. Emerging drugs for attention-deficit/hyperactivity disorder. *Expert Opinion on Emerging Drugs.* 2007;12(3):423-434.

Reason for exclusion: Review

Weisler2007b

- Weisler RH. Review of long-acting stimulants in the treatment of attention deficit hyperactivity disorder. *Expert Opin Pharmacother.* 2007;8(6):745-758.

Reason for exclusion: Review

Weiss1968

- Weiss G, Werry J, Minde K, Douglas V, Sykes D. Studies on the hyperactive child. V. The effects of dextroamphetamine and chlorpromazine on behaviour and intellectual functioning. *J Child Psychol Psychiatry.* 1968;9(3):145-156.

Reason for exclusion: No DSM/ICD criteria

Weiss1970

- Weiss G. Treatment of hyperactivity in children. *Curr Psychiatr Ther.* 1970;10:26-29.

Reason for exclusion: Review

Weiss1971

- Weiss G, Minde K, Douglas V, Werry J, Sykes D. Comparison of the effects of chlorpromazine, dextroamphetamine and methylphenidate on the behaviour and intellectual functioning of hyperactive children. *Can Med Assoc J.* Jan 9 1971;104(1):20-25.

Reason for exclusion: Analysis of three studies, all with no DSM/ICD criteria

Weiss1974

- Weiss G, Kruger E, Danielson U, Elman M. Long-term methylphenidate treatment of hyperkinetic children. *Psychopharmacol Bull.* 1974;10(4):34-35.

Reason for exclusion: No RCT

Weiss1975

- Weiss G, Kruger E, Danielson U, Elman M. Effect of long-term treatment of hyperactive children with methylphenidate. *Can Med Assoc J.* 1975;112(2):159-165.

Reason for exclusion: No RCT

Weiss1979

- Weiss G, Hechtman L, Perlman T, Hopkins J, Wener A. Hyperactives as young adults: a controlled prospective ten-year follow-up of 75 children. *Arch Gen Psychiatry.* 1979;36(6):675-681.

Reason for exclusion: No RCT

Weiss1981

- Weiss G. Controversial issues of the pharmacotherapy of the hyperactive child. *Can J Psychiatry.* 1981;26(6):385-392.

Reason for exclusion: Commentary/review

Weiss2003

- Weiss M, Murray C. Assessment and management of attention-deficit hyperactivity disorder in adults. *CMAJ.* 2003;168(6):715-722.

Reason for exclusion: Case report/commentary/review

Weiss2006a

- Weiss M, Hechtman L. A randomized double-blind trial of paroxetine and/or Dextroamphetamine and problem-focused therapy for attention- deficit/hyperactivity disorder in adults. *J Clin Psychiatry* 2006, 67(4):611-619
- Weiss MD, Wasdell M, Gadow KD, Greenfield B, Hechtman L, Gibbins C. Clinical correlates of oppositional defiant disorder and attention-deficit/hyperactivity disorder in adults. *Postgrad Med.* 2011;123(2):177-184.
- Secondary analysis in: Weiss M, Murray C, Wasdell M, Greenfield B, Giles L, Hechtman L. A randomized controlled trial of CBT therapy for adults with ADHD with and without medication. *BMC Psychiatry.* 2012;12:30.

Reason for exclusion: Co-treatment: psychotherapy.

Weiss2006b

- Weiss MD, Wasdell MB, Bomben MM, Rea KJ, Freeman RD. Sleep hygiene and melatonin treatment for children and adolescents with ADHD and initial insomnia. *J Am Acad Child Adolesc Psychiatry.* 2006;45(5):512-519.

Reason for exclusion: Co-treatment: psychotherapy. No treatment of interest for the present meta-analysis (melatonin) vs placebo

Weiss2007

- Weiss M, Hechtman L, Turgay A, et al. Once-daily multilayer-release methylphenidate in a double-blind, crossover comparison to immediate-release methylphenidate in children with attention-deficit/hyperactivity disorder. *J Child Adolesc Psychopharmacol.* 2007;17(5):675-688.

Reason for exclusion: Comparison of two different formulations of methylphenidate. Author confirmed there was no placebo only arm

Weiss2010

- Weiss MD, Gibbins C, Goodman DW, Hodgkins PS, Landgraf JM, Faraone SV. Moderators and mediators of symptoms and quality of life outcomes in an open-label study of adults treated for attention-deficit/hyperactivity disorder. *J Clin Psychiatry.* 2010;71(4):381-390.

Reason for exclusion: No RCT

Weizman1984

- Weizman A, Weitz R, Szekely GA, Tyano S, Belmaker RH. Combination of neuroleptic and stimulant treatment in attention deficit disorder with hyperactivity. *J Am Acad Child Psychiatry.* 1984;23(3):295-298.

Reason for exclusion: Psychostimulants plus other medication

Weizman1987

- Weizman R, Dick J, Gil-Ad I, Weitz R, Tyano S, Laron Z. Effects of acute and chronic methylphenidate administration on beta-endorphin, growth hormone, prolactin and cortisol in children with attention deficit disorder and hyperactivity. *Life Sci.* 1987(23):2247-2252.

Reason for exclusion: No RCT

Weizman1988

- Weizman A, Bernhout E, Weitz R, Tyano S, Rehavi M. Imipramine binding to platelets of children with attention deficit disorder with hyperactivity. *Biol Psychiatry.* 1988;23(5):491-496.

Reason for exclusion: No RCT

Weller1999

- Weller EB, Rowan A, Elia J, Weller RA. Aggressive behavior in patients with attention-deficit/hyperactivity disorder, conduct disorder, and pervasive developmental disorders. *The J Clin Psychiatry.* 1999;60 (Suppl 15):5-11.

Reason for exclusion: Review

Welsh2008

- Welsh JP, Ko C, Hsu WT. Lymphomatoid drug reaction secondary to methylphenidate hydrochloride. *Cutis.* 2008;81(1):61-64.

Reason for exclusion: Case report

Wender1981

- Wender PH, Reimherr FW, Wood DR. Attention deficit disorder (`minimal brain dysfunction') in adults: a replication study of diagnosis and drug treatment. *Arch Gen Psychiatry.* 1981; 38:449-56

Reason for exclusion: Medication of no interest for the present meta-analysis (pemoline) vs placebo; No DSM/ICD criteria

Wender1985a

- Wender PH, Wood DR, Reimherr FW. Pharmacological treatment of attention deficit disorder, residual type (ADD, RT, "minimal brain dysfunction,""hyperactivity") in adults. *Psychopharmacol Bull.* 1985;21(2):222-231.

Reason for exclusion: Commentary

Wender1985b

- Wender PH, Reimherr FW, Wood DR. Stimulant therapy of "adult hyperactivity." *Arch Gen Psychiatry.* 1985;42(8):84

Reason for exclusion: Commentary

Wender1985

- Wender PH, Reimherr FW, Wood D, Ward M. A controlled study of methylphenidate in the treatment of attention deficit disorder, residual type, in adults. *Am J Psychiatry.* 1985;142(5):547-552.

Reason for exclusion: no usable data

Wender1986

- Wender EH. Commentary: Treatment outcome in attention deficit disorder. *J Dev Behav Pediatr..* 1986;7(3):171-172.

Reason for exclusion: Commentary

Wender1986

- Wender PH. Concurrent therapy with d-amphetamine and adrenergic drugs. *Am J Psychiatry.* 1986;143(2):259-260.

Reason for exclusion: Case report

Wender1990

- Wender PH, Reimherr FW. Bupropion treatment of attention-deficit hyperactivity disorder in adults. *Am J Psychiatry.* 1990;147(8):1018-1020.

Reason for exclusion: Open label

Wender1991

- Wender EH, Solanto MV. Effects of sugar on aggressive and inattentive behavior in children with attention-deficit disorder with hyperactivity and normal-children. Pediatrics. 1991;88(5):960-966.

Reason for exclusion: No active treatment of interest for the present meta-analysis (sugar); single dose

Wender1998

- Wender PH. Pharmacotherapy of attention-deficit/hyperactivity disorder in adults. *The J Clin Psychiatry.* 1998;59 (Suppl 7):76-79.

Reason for exclusion: Systematic review

Wender2001

- Wender EH. Managing stimulant medication for attention-deficit/hyperactivity disorder. *Pediatrics in Review.* 2001;22(6):183-190.

Reason for exclusion: Review

Wender2002

- Wender EH. Managing stimulant medication for attention-deficit/hyperactivity disorder: an update. *Pediatrics in Review.* 2002;23(7):234-236.

Reason for exclusion: Review

Wernicke2001

- Wernicke JF, Dunn D, Faries DE, et al. Safety of atomoxetine in placebo-controlled pediatric attention-deficit hyperactivity disorder trials. *Ann Neurol.* 2001;50:S123-S4.

Reason for exclusion: Pooled studies from Lilly on atomoxetine (According to Lilly, the present meta-analysis included all available Lilly studies on atomoxetine)

Wernicke2002

- Wernicke JF, Kratochvil CJ. Safety profile of atomoxetine in the treatment of children and adolescents with ADHD. *J Clin Psychiatry.* 2002;63 (Suppl 12):50-55.

Reason for exclusion: Review

Wernicke2003

- Wernicke JF, Faries D, Girod D, et al. Cardiovascular effects of atomoxetine in children, adolescents, and adults. *Drug safety.* 2003;26(10):729-740.

Reason for exclusion: Systematic review

Wernicke2005

- Wernicke JF, Faries D, Breitung R, Girod D. QT correction methods in children and adolescents. *J Cardiovasc Electrophysiol.* 2005;16(1):76-81.

Reason for exclusion: Systematic review

Wernicke2007

- Wernicke JF, Holdridge KC, Jin L, et al. Seizure risk in patients with attention-deficit-hyperactivity disorder treated with atomoxetine. *Dev Med Child Neurol.* 2007;49(7):498-502.

Reason for exclusion: Systematic review

Werry1964

- Werry JS, Weiss G, Douglas V. Studies on the hyperctive child I: Some preliminary findings. *Can Psyquiatr Assoc J.* 1964;9 (2): 120-130

Reason for exclusion: No RCT

Werry1974

- Werry JS, Sprague RL Methylphenidate in children- Effect of dosage. *Aust N Z J Psychiatry 1974*; 8: 9-19

Reason for exclusion: No DSM/ICD criteria

Werry1975

- Werry JS, Aman MG. Methylphenidate and haloperidol in children. Effects on attention, memory, and activity. *Arch Gen Psychiatry.* 1975;32(6):790-795.

Reason for exclusion: No DSM/ICD criteria

Werry1976

- Werry JS, Aman MG, Lampen E. Haloperidol and methylphenidate in hyperactive children. *Acta Paedopsychiatr.* 1976;42(1):26-40.

Reason for exclusion: Review

Werry1976

- Werry JS. Medication for hyperkinetic children. *Drugs.* 1976;11(2):81-89.

Reason for exclusion: No DSM/ICD criteria

Werry1980

- Werry JS, Aman MG, Diamond E. Imipramine and methylphenidate in hyperactive children. *J Child Psychol Psychiatr.* 1980(1):27-35

Reason for exclusion: No DSM/ICD criteria

Wessner1996

- Wessner B, Vogt HJ, Peters H. Therapy with stimulants of hyperkinetic children. Zur Wirkung der Stimulantienbehandlung bei mental altersgerechten und retardierten Kindern mit hyperkinetischen Storungen. *Sozialpadiatrie und Kinderarztliche Praxis.* 1996;18(8):444-449.

Reason for exclusion: Of the 40 children only 16 underwent a placebo-controlled cross-over trial, the others had no placebo condition. Not all children met ADHD criteria (n = 33 ADHD DSM-III-R), 2 had ADD (DSM-III-R); 14 children had IQ retardation and/or various organic syndromes. Age is range 3:5 to 13:1.

West2002

- West SA, Johnson D, Wigal S, Zeldis J. Withdrawal trial of dex-methylphenidate HCL focalin in children with ADHD [abstract]. 155th Annual Meeting of the American Psychiatric Association; 2002 May 18-23; Philadelphia, PA2002:Nr341.

Reason for exclusion: Withdrawal design

Westover2012

- Westover AN, Halm EA. Do prescription stimulants increase the risk of adverse cardiovascular events?: A systematic review. *BMC Cardiovasc Disord.* 2012;12:41.

Reason for exclusion: Systematic review

Whalen1978

- Whalen CK, Collings BE, Henker B, Alkus SR, Adams D, Stapp J. Behavior observations of hyperactive children and methylphenidate (Ritalin) effects in systematically structured classroom environments: now you see them, now you don't. *J Pediatr Psychol.* 1978(4):177-187.
- Pooled in: Whalen CK, Henker B, Dotemoto S. Teacher response to the methylphenidate (ritalin) versus placebo status of hyperactive boys in the classroom. *Child Dev.* 1981;52(3):1005-1014.

Reason for exclusion: No DSM/ICD criteria

Whalen1979

- Whalen CK, Henker B, Collins BE, Finck D, Dotemoto S. A social ecology of hyperactive boys: medication effects in structured classroom environments. *J Appl Behav Anal.* 1979;12(1):65-81.
- Pooled in: Whalen CK, Henker B, Dotemoto S. Teacher response to the methylphenidate (ritalin) versus placebo status of hyperactive boys in the classroom. *Child Dev.* 1981;52(3):1005-1014.

Reason for exclusion: NO DSM/ICD criteria

Whalen1979

- Whalen CK, Henker B, Collins BE, McAuliffe S, Vaux A. Peer interaction in a structured communication task: comparisons of normal and hyperactive boys and of methylphenidate (Ritalin) and placebo effects. *Child Dev.* 1979;50(2):388-401.

Reason for exclusion: No DSM/ICD criteria

Whalen1980

- Whalen CK, Henker B, Dotemoto S. Methylphenidate and hyperactivity: effects on teacher behaviors. *Science.* 13 1980;208(4449):1280-1282.

Reason for exclusion: No DSM/ICD criteria

Whalen1981

- Whalen CK, Henker B, Finck D. Medication effects in the classroom: three naturalistic indicators. *J Abnorm Child Psychol.* 1981;9(4):419-433.

Reason for exclusion: No DSM/ICD criteria

Whalen1987

- Whalen Carol K. Natural Social Behaviors in Hyperactive Children: Dose Effects of Methylphenidate. *J Consult Clin Psychol.* 1987(2):187-193.

Reason for exclusion: Less than seven days treatment

Whalen1987

- Whalen CK, Henker B, Castro J, Granger D. Peer perceptions of hyperactivity and medication effects. *Child Dev.* 1987;58(3):816-828.

Reason for exclusion: Participants: Responders to previous ADHD medications; No randomized

Whalen1989

- Whalen CK, Henker B, Granger DA. Ratings of medication effects in hyperactive children: Viable or vulnerable? *Behavioral Assessment.* 1989;11(2):179-199.

Reason for exclusion: Two single dose studies. Note: According to NICE 2007, sample of the following study was drawn from the same sample: Buhrmester D, Whalen CK., Henker B, MacDonald V, Hinshaw SP. Prosocial behavior in hyperactive boys: effects of stimulant medication and comparison with normal boys. Journal of Abnormal Child Psychology. 1992; 20(1): 103-121.

Whalen1989
- Whalen CK, Henker B, Buhrmester D, Hinshaw SP, Huber A, Laski K. Does stimulant medication improve the peer status of hyperactive children? *J Consult Clin Psychol.* 1989;57(4):545-549.

Reason for exclusion: Co-intervention (group CBT)

Whalen1990
- Whalen CK, Henker B, Granger DA. Ratings of medication effects in hyperactive children: Viable or vulnerable? *Behavioral Assessment.* 1989;11(2):179-199.
- Whalen CK, Henker B, Hinshaw SP, Granger DA. Externalizing behavior disorders, situational generality, and the type A behavior pattern. *Child Dev.* 1989;60(6):1453-1462.
- Whalen CK, Henker B, Granger DA. Social judgment processes in hyperactive boys: effects of methylphenidate and comparisons with normal peers. *J Abnorm Child Psychol.* Jun 1990;18(3):297-316.

Reason for exclusion: Less than seven days treatment

Whalen1991
- Whalen CK, Henker B. Social impact of stimulant treatment for hyperactive children. *J Learn Disabil.* 1991;24(4):231-241.

Reason for exclusion: Review plus empirical study; Empirical study: single dose

Whalen2010
- Whalen CK, Henker B, Ishikawa SS, Emmerson NA, Swindle R, Johnston JA. Atomoxetine versus stimulants in the community treatment of children with ADHD: an electronic diary study. *J Atten Disord.* 2010;13(4):391-400.

Reason for exclusion: No RCT

White1977
- White JH. The hyperactive child syndrome. *American Family Physician.* 1977;15(4):100-104.

Reason for exclusion: Review

White2000
- White SR, Yadao CM. Characterization of methylphenidate exposures reported to a regional poison control center. *Arch Pediatr Adolesc Med.* 2000;154(12):1199-1203.

Reason for exclusion: No RCT

White2005
- White GB. Splitting the self: the not-so-subtle consequences of medicating boys for ADHD. *Am J Bioeth.* 2005;5(3):57-59.

Reason for exclusion: Commentary

White2006
- White TL, Lott DC, de Wit H. Personality and the subjective effects of acute amphetamine in healthy volunteers. *Neuropsychopharmacology.* 2006;31(5):1064-1074.

Reason for exclusion: No participants with ADHD; Single dose

White2007
- White TL, Lejuez CW, de Wit H. Personality and gender differences in effects of d-amphetamine on risk taking. *Exp Clin Psychopharmacol.* 2007;15(6):599-609.

Reason for exclusion: No participants with ADHD; Single dose

Whitehouse1980
- Whitehouse D, Shah U, Palmer FB. Comparison of sustained-release and standard methylphenidate in the treatment of minimal brain dysfunction. *J Clin Psychiatry.* 1980;41(8):282-285.

Reason for exclusion: No DSM/ICD criteria

Whyte1997

- Whyte J, Hart T, Schuster K, Fleming M, Polansky M, Coslett HB. Effects of methylphenidate on attentional function after traumatic brain injury - A randomized, placebo-controlled trial. *Am J Phys Med Rehabil.* 1997;76(6):440-450.

Reason for exclusion: No participants with ADHD

Whyte2004
- Whyte J, Hart T, Vaccaro M, et al. Effects of methylphenidate on attention deficits after traumatic brain injury: a multidimensional, randomized, controlled trial. *Am J Phys Med Rehabil.* 2004;83(6):401-420.

Reason for exclusion: No participants with ADHD

Wienbruch2005
- Wienbruch C, Paul I, Bauer S, Kivelitz H. The influence of methylphenidate on the power spectrum of ADHD children - an MEG study. *BMC Psychiatry.* 2005;5:29.

Reason for exclusion: Single dose

Wiener1988
- Wiener JM. Medicating children with attention deficit disorder. *Pediatrics.* 1988;82(5):812.

Reason for exclusion: Letter, no empirical data

Wiesegger2007
- Wiesegger G, Kienbacher C, Pellegrini E, et al. Pharmacotherapy of Attention-Deficit/Hyperactivity Disorder (ADHD) and comorbid disorders. [German] Medikamentose behandlung von Aufmerksamkeitsdefizit-Hyperaktivitatssyndrom (ADHS) und komorbiden storungen *Neuropsychiatr.* 2007;21(3):187-206

Reason for exclusion: Review

Wietecha2009 (NCT00191035; B4Z-US-LYCD(7974)
- Secondary analysis in: Taylor K, Williams DW, Schuh KJ, Wietecha L, Greenbaum M. Effects of atomoxetine on self-reported high-risk behaviors and health-related quality of life in adolescents with ADHD. *Curr Med Res Opin.* 2010;26(9):2087-2095
- Wietecha LA, Williams DW, Herbert M, Melmed RD, Greenbaum M, Schuh K. Atomoxetine treatment in adolescents with attention-deficit/hyperactivity disorder. *J Child Adolesc Psychopharmacol.* 2009;19(6):719-730.
- https://clinicaltrials.gov/ct2/show/NCT00191035

Reason for exclusion: Only one medication (atomoxetine) of interest for the present meta-analysis, no placebo arm.

Wietecha2013
- Wietecha LA, Ruff DD, Allen AJ, Greenhill LL, Newcorn JH. Atomoxetine tolerability in pediatric and adult patients receiving different dosing strategies. *J Clin Psychiatry.* 2013;74(12):1217-1223.

Reason for exclusion: Post hoc/systematic review

Wigal1999
- Wigal T, Swanson JM, Regino R, et al. Stimulant medications for the treatment of ADHD: Efficacy and limitations. *Mental Retard and Dev Disabil Res Rev.* 1999;5(3):215-224.

Reason for exclusion: Review

Wigal2002
- Wigal SB. OROS formulation of methylphenidate in treatment of ADHD: duration of effect. *Pediatr Res.* 2002(4):21a; 120.

Reason for exclusion: Only abstract available; author contacted to query about full text paper but no reply

Wigal2003
- Wigal SB, Sanchez DY, DeCory HH, D'Imperio JM, Swanson JM. Selection of the optimal dose ratio for a controlled-delivery formulation of methylphenidate. *Journal of Applied Research.* 2003(1):46-63.

Reason for exclusion: Participnats: Responders to previous ADHD medications

Wigal2007
- Wigal SB, Wigal TL. Special considerations in diagnosing and treating attention-deficit/ hyperactivity disorder. *Prim psychiatry.* 2007;14(6):S1-S14.

Reason for exclusion: Review/commentary

Wigal2007

- Wigal SB, Wilens TE, Wolraich M, Lerner M. Hematologic and blood biochemistry monitoring during methylphenidate treatment in children with attention-deficit/hyperactivity disorder: 2-year, open-label study results. *Pediatrics.* 2007;120(1):e120-128.

Reason for exclusion: No RCT

Wigal2009 (NCT00500149; SPD489-311)

- Pooled in: Jain R, Babcock T, Burtea T, et al. Efficacy and safety of lisdexamfetamine dimesylate in children with attention-deficit/hyperactivity disorder and recent methylphenidate use. *Adv Ther.* 2013;30(5):472-486.
- Vyvanse_Lisdexamfetamine_ApprovalPackage_S036_FDA.pdf (Vyvanse study 3)
- Wigal SB, Kollins SH, Childress AC, Squires L. A 13-hour laboratory school study of lisdexamfetamine dimesylate in school-aged children with attention-deficit/hyperactivity disorder. *Child Adolesc Psychiatry Ment Health.* 2009;3(1):17.
- Post hoc analysis in: Wigal SB, Kollins SH, Childress AC, Adeyi B. Efficacy and tolerability of lisdexamfetamine dimesylate in children with attention-deficit/hyperactivity disorder: sex and age effects and effect size across the day. *Child Adolesc Psychiatry Ment Health.* 2010;4:32.
- https://clinicaltrials.gov/ct2/show/NCT00500149

Reason for exclusion: participants: Responders to previous ADHD medications

Wigal2010 (NCT00697515)

- Brams M, Giblin J, Gasior M, Gao, J, Wigal T. Effects of open-label lisdexamfetamine dimesylate on self-reported quality of life in adults with ADHD. Postgrad Med. 2011;123(3):99-108.
- Wigal T, Brams M, Gasior M, Gao J, Squires L, Giblin J; 316 Study Group. Randomized, double-blind, placebo-controlled, crossover study of the efficacy and safety of lisdexamfetamine dimesylate in adults with attention-deficit/hyperactivity disorder: novel findings using the adult workplace environment design. *Behav Brain Funct.* 2010;6:34.
- Wigal T, Gao J, Gasior M, Giblin J, Valliere S, Brams M. Efficacy and safety of lisdexamfetamine dimesylate in adults with attention-deficit/hyperactivity disorder in the simulated adult workplace environment. *Pharmacotherapy.* 2010;30(10):422e.
- Wigal T, Brams M, Gasior M, Gao J, Giblin J. Effect size of lisdexamfetamine dimesylate in adults with attention-deficit/hyperactivity disorder. *Postgrad Med.* 2011;123(2):169-176.
- https://clinicaltrials.gov/ct2/show/NCT00697515

Reason for exclusion: Participants: responders in open label phase

Wigal2010a

- Wigal SB, Chae S, Patel A, Steinberg-Epstein R. Advances in the treatment of attention-deficit/hyperactivity disorder: a guide for pediatric neurologists. *Sem Pediat Neurol.* 2010;17(4):230-236.

Reason for exclusion: Review

Wigal2010b

Wigal SB, Jun A, Wong AA, Stehli A, Steinberg-Epstein R, Lerner MA. Does prior exposure to stimulants in children with ADHD impact cardiovascular parameters from lisdexamfetamine dimesylate? *Postgrad Med.* 2010(5):27-34.

Reason for exclusion: Single blind

Wigal2011

- Wigal SB, Gupta S, Heverin E, Starr HL. Pharmacokinetics and therapeutic effect of OROS methylphenidate under different breakfast conditions in children with attention-deficit/hyperactivity disorder. *J Child Adolesc Psychopharmacol.* 2011;21(3):255-263.

Reason for exclusion: participants: responders to previous ADHD medications

Wigal2011 (NCT00799409; EUCTR2015-001081-26; Related to the ABC study NCT00799487)

- Pooled in: Armstrong RB, Damaraju CV, Ascher S, Schwarzman L, O'Neill J, Starr HL. Time course of treatment effect of OROS methylphenidate in children with ADHD. *J Atten Disord.* 2012;16(8):697-705.
- Pooled in: Starr HL, Armstrong R, Damaraju CV, Ascher S. Effects of OROS methylphenidate (MPH) treatment on behavior and performance in children with ADHD with and without comorbid learning disability. *Eur Child Adolesc Psychiatry.* June 2011;20:S126.
- Wigal S, Wigal T, Schuck S, et al. Effect of oros methylphenidate treatment on reading performance in children with adhd. 163rd Annual Meeting of the American Psychiatric Association; 2010 May 22-26; New Orleans, LA2010.

- Wigal SB, Wigal T, Schck S, et al. Academic, behavioral, and cognitive effects of OROS(R) methylphenidate on older children with attention-deficit/hyperactivity disorder. *J Child Adolesc Psychopharmacol.* 2011;21(2):121-131.
- Pooled in: Williamson D, Murray DW, Damaraju CV, Ascher S, Starr HL. Methylphenidate in children with ADHD with or without learning disability. *J Atten Disord.* 2014;18(2):95-104.
- https://clinicaltrials.gov/ct2/show/NCT00799409
- https://clinicaltrials.gov/ct2/show/NCT00799487
- https://www.clinicaltrialsregister.eu/ctr-search/search?query=eudract_number:2015-001081-26

Reason for exclusion: Less than seven days treatment; Participants: Responders to previous ADHD medications

Wigal2012 (NCT00733356)
- Wigal SB, Maltas S, Crinella F, et al. Reading performance as a function of treatment with lisdexamfetamine dimesylate in elementary school children diagnosed with ADHD. *J Atten Disord.* 2012;16(1):23-33.
- https://clinicaltrials.gov/ct2/show/NCT00733356

Reason for exclusion: No RCT

Wigal2012
- Wigal SB, Polzonetti CM, Stehli A, Gratton E. Phase synchronization of oxygenation waves in the frontal areas of children with attention-deficit hyperactivity disorder detected by optical diffusion spectroscopy correlates with medication. *Journal of Biomedical Optics.* 2012;17(12):127002.

Reason for exclusion : Review

Wigal2012
- Wigal SB, Truong C, Stehli A. The novel use of objective laboratory school tasks to measure stress responses in children with ADHD. *Postgrad Med.* 2012;124(5):49-57.

Reason for exclusion: No RCT

Wigal2012
- Wigal SB, Wong AA, Jun A, Stehli A, Steinberg-Epstein R, Lerner MA. Adverse events in medication treatment-naive children with attention-deficit/hyperactivity disorder: results from a small, controlled trial of lisdexamfetamine dimesylate. *J Child Adolesc Psychopharmacol.* 2012;22(2):149-156.

Reason for exclusion: No double blind RCT

Wigal2013 (NCT00904670)
- Wigal SB, Childress AC, Belden HW, Berry SA. NWP06, an extended-release oral suspension of methylphenidate, improved attention-deficit/hyperactivity disorder symptoms compared with placebo in a laboratory classroom study. *J Child Adolesc Psychopharmacol.* 2013;23(1):3-10
- Liquid version of methylphenidate shows efficacy in school trial. *The Brown University Child & Adolescent Psychopharmacology Update* 2013;15(3):1–3.
- Robb AS, Findling RL, Childress AC, Berry SA, Belden HW, Wigal SB. Efficacy, Safety, and Tolerability of a Novel Methylphenidate Extended-Release Oral Suspension (MEROS) in ADHD. *J Atten Disord* .2017; 21(4):1180-91.
- https://clinicaltrials.gov/ct2/show/NCT00904670

Reason for exclusion: no pre cross-over data available

Wigal2014 (NCT01269463; AptensioXR(RP-BP-EF001)-(Study 022-004)
- Pooled in: Owens J, Weiss M, Nordbrock E, et al. Effect of Aptensio XR (methylphenidate HCl extended-release) capsules on sleep in children with attention-deficit/hyperactivity disorder. *J Child Adolesc Psychopharmacol.* 2016;26:873-881.
- Wigal SB, Greenhill LL, Nordbrock E, et al. A randomized placebo-controlled double-blind study evaluating the time course of response to methylphenidate hydrochloride extended-release capsules in children with attention-deficit/hyperactivity disorder. *J Child Adolesc Psychopharmacol.* 2014;24(10):562-569.
- Wigal S, Childress A, Greenhill L, et al. Time course of response to methylphenidate extended-release capsules in children with ADHD: a randomized, placebo-controlled, double-blind study. *CNS spectrums. Conference: 2014 NEI psychopharmacology congress. United states. Conference start: 20141113. Conference end: 20141116.* 2017;20(1):68
- https://clinicaltrials.gov/ct2/show/NCT01269463

Reason for exclusion: Subjects entering the double phase were selected based on previous response to ADHD medications

Wigal2016 (NCT02225639)

- Wigal SB, Wigal T, Childress A, Donnelly GA, Reiz JL. The Time Course of Effect of Multilayer-Release Methylphenidate Hydrochloride Capsules: A Randomized, Double-Blind Study of Adults With ADHD in a Simulated Adult Workplace Environment. *J Atten Disord.* Oct 17 2016.
- https://clinicaltrials.gov/ct2/show/NCT02225639

Reason for exclusion: Initial optimization phase

Wiguna2012

- Wiguna T, Guerrero AP, Wibisono S, Sastroasmoro S. Effect of 12-week administration of 20-mg long-acting methylphenidate on Glu/Cr, NAA/Cr, Cho/Cr, and mI/Cr ratios in the prefrontal cortices of school-age children in Indonesia: a study using 1H magnetic resonance spectroscopy (MRS). *Clin Neuropharmacol.* 2012;35(2):81-85.

Reason for exclusion: No randomised

Wilens1992

- Wilens TE, Biederman J. The stimulants. *Psychiatr Clin North Am.* 1992;15(1):191-222.

Reason for exclusion: Review

Wilens1993

- Wilens TE, Biederman J, Geist DE, Steingard R, Spencer T. Nortriptyline in the treatment of ADHD - a chart review of 58 cases. *J Am Acad Child Adolesc Psychiatry.* 1993;32(2):343-349.

Reason for exclusion: No treatment of interest (protriptyline) for the present meta-analysis; no RCT

Wilens1994

- Wilens TE, Biederman J, Spencer T. Clonidine for sleep disturbances associated with attention-deficit hyperactivity disorder. *J Am Acad Child Adolesc Psychiatry.* 1994;33(3):424-426.

Reason for exclusion: Review/commentary

Wilens1995

- Wilens TE, Biederman J, Spencer TJ, Prince J. Pharmacotherapy of adult attention deficit/hyperactivity disorder: a review. *J Clin Psychopharmacol.* 1995;15(4):270-279.

Reason for exclusion: Review

Wilens1996

- Wilens TE, Biederman J, Abrantes AM, Spencer TJ. A naturalistic assessment of protriptyline for attention-deficit hyperactivity disorder. *J Am Acad Child Adolesc Psychiatry.* 1996;35(11):1485-1490.

Reason for exclusion: No treatment of interest (protriptyline) for the present meta-analysis; No RCT

Wilens1996

- Wilens TE, Biederman J, Prince J, et al. Six-week, double-blind, placebo-controlled study of desipramine for adult attention deficit hyperactivity disorder. *Am J Psychiatry.* 1996;153(9):1147-1153.
- Additional data in: Wilens TE, Hammerness PG, Biederman J, et al. Blood pressure changes associated with medication treatment of adults with attention-deficit/hyperactivity disorder. J Clin Psychiatry. 2005;66(2):253-259

Reason for exclusion: No treatment of interest for the present meta-analysis

Wilens1998

- Wilens TE, Biederman J, Spencer TJ. Pharmacotherapy of attention deficit hyperactivity disorder in adults. *CNS Drugs.* 1998;9(5):347-356.

Reason for exclusion: Review

Wilens1999

- Wilens TE, Biederman J, Spencer TJ, et al. Controlled trial of high doses of pemoline for adults with attention-deficit/hyperactivity disorder. *J Clin Psychopharmacol.* 1999;19(3):257-264.
- Additional data in: Wilens TE, Hammerness PG, Biederman J, et al. Blood pressure changes associated with medication treatment of adults with attention-deficit/hyperactivity disorder. *The J Clin Psychiatry.* 2005;66(2):253-259.

Reason for exclusion: No treatment of interest (pemoline) for the present meta-analysis; no other arms of interest

Wilens1999

- Wilens TE, Biederman J, Spencer TJ, et al. A pilot controlled clinical trial of ABT-418, a cholinergic agonist, in the

treatment of adults with attention deficit hyperactivity disorder. *Am J Psychiatry*. 1999; 156(12):1931–1937.
Reason for exclusion: Medication of no interest for the present meta-analysis vs placebo

Wilens1999
- Wilens TE, Spencer TJ, Swanson JM, Connor DF, Cantwell D. Combining methylphenidate and clonidine: a clinically sound medication option. *J Am Acad Child Adolesc Psychiatry.* 1999;38(5):614-619; discussion 619-622.

Reason for exclusion: No RCT

Wilens2000
- Wilens TE, Spencer TJ. The stimulants revisited. *Child Adolesc Psychiatr Clin N Am.* 2000;9(3):573-+.

Reason for exclusion: Review

Wilens2001
- Wilens TE. One-year safety of a once-daily OROS formulation of methylphenidate HCl in children with ADHD: Effects on growth, sleep, appetite, and tics. *Pediatr Res 2001*; 49, 429A

Reason for exclusion: Abstract only contacted author to query about full text paper but not possible to retrieve additional information

Wilens2003 (NCT00269776)
Subjects from previous studies:
- Baren M, Swanson JM, Wigal SB. Lack of effect of different breakfast conditions on the pharmacokinetics and efficacy of OROS methylphenidate HCI extended-release tablets in children with ADHD. *Pediatr Res.* 2000(4):23a.
- Pelham WE, Gnagy EM, Burrows-Maclean L, et al. Once-a-day Concerta methylphenidate versus three-times-daily methylphenidate in laboratory and natural settings. *Pediatrics.* 2001;107(6):E105.
- Swanson J, Gupta S, Lam A, et al. Development of a new once-a-day formulation of methylphenidate for the treatment of attention-deficit/hyperactivity disorder: proof-of-concept and proof-of-product studies. *Arch Gen Psychiatry.* 2003;60(2):204-211.
- Wilens T, Pelham W, Stein M, et al. ADHD treatment with once-daily OROS methylphenidate: interim 12-month results from a long-term open-label study. *J Am Acad Child Adolesc Psychiatry.* 2003;42(4):424-433.
- Wolraich ML, Greenhill LL, Pelham W, et al. Randomized, controlled trial of oros methylphenidate once a day in children with attention-deficit/hyperactivity disorder. *Pediatrics.* 2001;108(4):883-892.
- https://clinicaltrials.gov/ct2/show/NCT00269776

Reason for exclusion: No RCT

Wilens2003
- Wilens TE, Prince JB, Spencer T, et al. An open trial of bupropion for the treatment of adults with attention-deficit/hyperactivity disorder and bipolar disorder. *Biol Psychiatry.* 2003;54(1):9-16.

Reason for exclusion: No RCT

Wilens2003
- Wilens TE. Drug therapy for adults with attention-deficit hyperactivity disorder. *Drugs.* 2003;63(22):2395-2411.

Reason for exclusion: Systematic review

Wilens2004
- Wilens TE. Impact of ADHD and its treatment on substance abuse in adults. *J Clin Psychiatry.* 2004;65 (Suppl 3):38-45.

Reason for exclusion: Review

Wilens2004
- Wilens E, Faraone SV, Biederman J. Attention-deficit/hyperactivity disorder in adults. *Jama.* 2004;292(5):619-623.

Reason for exclusion: Review

Wilens2004
- Wilens TE. Attention-deficit/hyperactivity disorder and the substance use disorders: The nature of the relationship, who is at risk, and treatment issues. *Prim psychiatry.* 2004;11(7):63-70.

Reason for exclusion: Review

Wilens2004

- Wilens TE, Biederman J, Lerner M, Concerta Study G. Effects of once-daily osmotic-release methylphenidate on blood pressure and heart rate in children with attention-deficit/hyperactivity disorder: results from a one-year follow-up study. *J Clin Psychopharmacol.* 2004;24(1):36-41.

Reason for exclusion: No RCT

Wilens2005
- Wilens TE, Waxmonsky J, Scott M, et al. An open trial of adjunctive donepezil in attention-deficit/hyperactivity disorder. *J Child Adolesc Psychopharmacol.* 2005;15(6):947-955.

Reason for exclusion: No RCT

Wilens2005
- Wilens TE, Monuteaux MC, Snyder LE, Moore H, Whitley J, Gignac M. The clinical dilemma of using medications in substance-abusing adolescents and adults with attention-deficit/hyperactivity disorder: What does the literature tell us? *J Child Adolesc Psychopharmacol.* 2005;15(5):787-798.

Reason for exclusion: Systematic review/meta-analysis

Wilens2006
- Wilens TE, Gignac M, Swezey A, Monuteaux MC, Biederman J. Characteristics of adolescents and young adults with ADHD who divert or misuse their prescribed medications. *J Am Acad Child Adolesc Psychiatry.* 2006;45(4):408-414.

Reason for exclusion: No RCT

Wilens2006
- Wilens TE, Kratochvil C, Newcorn JH, Gao H. Do children and adolescents with ADHD responddifferently to atomoxetine? *J Am Acad Child Adolesc Psychiatry.* 2006;45(2):149-157.

Reason for exclusion: Systematic review/meta-analysis

Wilens2006
- Wilens TE, Newcorn JH, Kratochvil CJ, et al. Long-term atomoxetine treatment in adolescents with attention-deficit/hyperactivity disorder. *J Pediatr.* 2006;149(1):112-119.

Reason for exclusion: Systematic review/meta-analysis of Lilly studies. (Lilly confirmed we located all their studies for the present meta-analysis.

Wilens2006
- Wilens TE, Prince JB, Spencer TJ, Biederman J. Stimulants and sudden death: What is a physician to do? *Pediatrics.* 2006;118(3):1215-1219.

Reason for exclusion: Commentary

Wilens2006
- Wilens TE, Verlinden MH, Adler LA, Wozniak PJ, West SA. ABT-089, a neuronal nicotinic receptor partial agonist, for the treatment of attention-deficit/hyperactivity disorder in adults: results of a pilot study. *Biol Psychiatry*; 2006; 59(11): 1065-1070

Reason for exclusion: Medication of no interest for the present meta-analysis vs placebo

Wilens2006
- Wilens TE, Zusman RM, Hammerness PG, et al. An open-label study of the tolerability of mixed amphetamine salts in adults with attention-deficit/hyperactivity disorder and treated primary essential hypertension. *J Clin Psychiatry.* 2006;67(5):696-702.

Reason for exclusion: No RCT

Wilens2006
- Wilens TE. Mechanism of action of agents used in attention-deficit/hyperactivity disorder. *J Clin Psychiatry.* 2006;67 (Suppl 8):32-38.

Reason for exclusion: Review

Wilens2006 (NCT00269815)
- Wilens T, McBurnett K, Stein M, Lerner M, Spencer T, Wolraich M. ADHD treatment with once-daily OROS methylphenidate: final results from a long-term open-label study. *J Am Acad Child Adolesc Psychiatry.* 2005 Oct;44(10):1015-23. Erratum in: J Am Acad Child Adolesc Psychiatry. 2006 May;45(5):632.
- https://clinicaltrials.gov/ct2/show/NCT00269815

Reason for exclusion: Open label

Wilens2006 (NCT00249353)

- Biederman J. P.6.053 Effectiveness and safety of the oncedaily OROS formulation of methylphenidate in adolescents with attention-deficit/hyperactivity disorder. *Eur Neuropsychopharmacol.* 2003;13(Suppl 4):S448.
- Greenhill LL. Safety and efficacy of OROS MPH in adolescents with ADHD. *Proceedings of the 156th Annual Meeting of the American Psychiatric Association; 2003 May 17-22; San Francisco, CA. San Francisco, 2003:16.*
- Analysis of long-term outcomes in: McGough JJ, McBurnett K, Bukstein O, et al. Once-daily OROS methylphenidate is safe and well tolerated in adolescents with attention-deficit/hyperactivity disorder. *J Child Adolesc Psychopharmacol.* 2006;16(3):351-356.
- Newcorn JH, Stein MA, Cooper KM. Dose-Response Characteristics in Adolescents with Attention-Deficit/Hyperactivity Disorder Treated with OROS (R) Methylphenidate in a 4-Week, Open-Label, Dose-Titration Study. *J Child Adolesc Psychopharmacol.* 2010;20(3):187-196.
- Wilens TE. Safety and efficacy of oros methylphenidate in adolescents with ADHD. 157th Annual Meeting of the American Psychiatric Association; 2004 May 1-6; New York, NY2004.
- Wilens TE, McBurnett K, Bukstein O, et al. Multisite controlled study of OROS methylphenidate in the treatment of adolescents with attention-deficit/hyperactivity disorder. *Arch Pediatr Adolesc Med.* 2006;160(1):82-90.
- Oral system methylphenidate for teen ADHD. *The Brown University Child & Adolescent Psychopharmacology Update* 2006;8(3):4–5.
- https://clinicaltrials.gov/ct2/show/NCT00249353

Reason for exclusion: Participants: Responders to previous ADHD medications

Wilens2006a

- Wilens TE, Kratochvil C, Newcorn JH, Gao H. Do children and adolescents with ADHD respond differently to atomoxetine? J *Am Acad Child Adolesc Psychiatry.* Feb 2006;45(2):149-157.

Reason for exclusion: Review

Wilens2007

- Wilens TE, Biederman J, Spencer TJ, Adler LA. ADHD: Prevalence, diagnosis, and issues of comorbidity. *CNS Spectr.* 2007;12(4):3-+.

Reason for exclusion: Review/commentary

Wilens2008

- Wilens TE. Pharmacotherapy of ADHD in adults. *CNS Spectr.* 2008;13(5 Suppl 8):11-13.

Reason for exclusion: Review

Wilens2008

- Wilens TE. Effects of methylphenidate on the catecholaminergic system in attention-deficit/hyperactivity disorder. *J Clin Psychopharmacol.* 2008;28(3 Suppl 2):S46-53.

Reason for exclusion: Review

Wilens2008

- Wilens TE, Adamson J, Monuteaux MC, et al. Effect of prior stimulant treatment for attention-deficit/hyperactivity disorder on subsequent risk for cigarette smoking and alcohol and drug use disorders in adolescents. *Arch Pediatr Adolesc Med.* 2008;162(10):916-921.

Reason for exclusion: No RCT

Wilens2008

- Wilens TE, Adler LA, Adams J, et al. Misuse and diversion of stimulants prescribed for ADHD: a systematic review of the literature. *J Am Acad Child Adolesc Psychiatry.* 2008;47(1):21-31.

Reason for exclusion: Systematic review

Wilens2008

- Wilens TE, Klint T, Adler L, et al. A randomized controlled trial of a novel mixed monoamine reuptake inhibitor in adults with ADHD. *Behav Brain Funct.* 2008;4.

Reason for exclusion: No treatment of interest for the present meta-analysis

Wilens2008a (NCT00151970)

- Frazier TW, Weiss M, Hodgkins P, Manos MJ, Landgraf JM, Gibbins C. Time course and predictors of health-related quality of life improvement and medication satisfaction in children diagnosed with attention-deficit/hyperactivity disorder treated with the methylphenidate transdermal system. *J Child Adolesc Psychopharmacol.* 2010;20(5):355-364.
- López FA, Wilens TE, Wigal SB, Turnbow JM. Effects of variable wear times on transdermalmethylphenidate in attention-deficit/hyperactivity disorder. *Eur Neuropsychopharmacol.* Papers of the 21st ECNP Congress; 2008 August 30 - September 3; Barcelona, Spain. 2008; Vol. 18:S561–2.
- López FA, Landgraf JM, Wilens TE. Quality of life and parent satisfaction with the methylphenidate transdermal system. European Neuropsychopharmacology. *Papers of the 21st ECNP Congress; 2008 August 30 - September 3; Barcelona, Spain. 2008; 4: S562*
- Manos M, Frazier TW, Landgraf JM, Weiss M, Hodgkins P. HRQL and medication satisfaction in children with ADHD treated with the methylphenidate transdermal system. *Curr Med Res Opin.* 2009;25(12):3001-3010.
- Wilens TE, Boellner SW, Lopez FA, et al. Varying the wear time of the methylphenidate transdermal system in children with attention-deficit/hyperactivity disorder. *J Am Acad Child Adolesc Psychiatry.* 2008;47(6):700-708.
- https://clinicaltrials.gov/ct2/show/NCT00151970

Reason for exclusion: Transdermal formulation

Wilens2009

- Wilens TE, Hammerness P, Utzinger L, et al. An open study of adjunct OROS-methylphenidate in children and adolescents who are atomoxetine partial responders: I. Effectiveness. *J Child Adolesc Psychopharmacol.* 2009;19(5):485-492.

Reason for exclusion: No RCT

Wilens2010

- Wilens TE, Prince JB, Waxmonsky J, et al. An Open Trial of Sustained Release Bupropion for Attention-Deficit/Hyperactivity Disorder in Adults with ADHD plus Substance Use Disorders. *Journal of ADHD & related disorders.* 2010;1(3):25-35.

Reason for exclusion: No RCT

Wilens2010 (NCT00586157)

- Wilens T, Hammerness P, Utzinger L, Georgiopoulos A, Doyle R, Brodziak K, et al. Before-school ADHD symptoms and functioning in youth treated with the Methylphenidate Transdermal Patch (MTS). J Child Adolesc Psychopharmacol. Abstracts of the 49th Annual National Institute of Mental Health (NIMH)New Clinical Drug Evaluation Unit (NCDEU) Meeting;2009; June 29 - July 2; Hollywood, Florida. 2009; Vol. 19, 6:785–6.
- Wilens TE, Hammerness P, Martelon M, Brodziak K, Utzinger L, Wong P. A controlled trial of the methylphenidate transdermal system on before-school functioning in children with attention-deficit/hyperactivity disorder. *J Clin Psychiatry.* 2010;71(5):548-556.
- https://clinicaltrials.gov/ct2/show/NCT00586157

Reason for exclusion: Transdermal formulation

Wilens2011

- Wilens TE, Morrison NR. The intersection of attention-deficit/hyperactivity disorder and substance abuse. *Curr Opin Psychiatry.* 2011;24(4):280-285.

Reason for exclusion: Review

Wilens2011

- Wilens TE, Morrison NR, Prince J. An update on the pharmacotherapy of attention-deficit/hyperactivity disorder in adults. *Expert Rev Neurother.* 2011;11(10):1443-1465

Reason for exclusion: Review

Wilens2012

- Wilens TE, Morrison NR. Substance-use disorders in adolescents and adults with ADHD: Focus on treatment. *Neuropsychiatry.* 2012;2(4):301-312.

Reason for exclusion: Review

Wilens2012 (NCT00734578)

- Wilens TE, Bukstein O, Brams M, et al. A controlled trial of extended-release guanfacine and psychostimulants for attention-deficit/hyperactivity disorder. *J Am Acad Child Adolesc Psychiatry.* 2012;51(1):74-85 e72.
- Bukstein O, Turnbow JM, Youcha S, et al. Efficacy and Safety of Morning or Evening Administration of Guanfacine

Extended Release as Adjunctive Therapy to Psychostimulants in Adolescents With ADHD. *Presented at the 164th Annual Meeting of the American Psychiatric Association; 14 - 18 May 2011; Honolulu, Hawaii*
- Cutler AJ, Brams M, Bukstein O, et al. Response/remission with guanfacine extended-release and psychostimulants in children and adolescents with attention-deficit/hyperactivity disorder. *J Am Acad Child Adolesc Psychiatry.* 2014;53(10):1092-1101
- Sikirica V, Haim Erder M, Xie J, et al. Cost effectiveness of guanfacine extended release as an adjunctive therapy to a stimulant compared with stimulant monotherapy for the treatment of attention-deficit hyperactivity disorder in children and adolescents. *Pharmacoeconomics.* 2012;30(8):e1-15.
- https://clinicaltrials.gov/ct2/show/NCT00734578

Reason for exclusion: Participants on psychostimulants plus guanfacine or placebo

Wilens2013
- Wilens TE, McBurnett K, Turnbow J, Rugino T, White C, Youcha S. Morning and Evening Effects of Guanfacine Extended Release Adjunctive to Psychostimulants in Pediatric ADHD: Results From a Phase III Multicenter Trial. *J Atten Disord.* 2013.

Reason for exclusion: Psychostimulant+guanfacine

Wilens2016a
- Wilens TE. Treatment effects on early morning functioning in children with attention deficit/ hyperactivity disorder. *J Am Acad Child Adolesc Psychiatry.* 2016;55 (10 Supp1):S315.

Reason for exclusion: No RCT; Presentation of studies

Wilens2016b
- Wilens TE, McGough JJ. Early morning functioning in attentiondeficit/ hyperactivity disorder: Impact, measurement, and treatment considerations. *J Am Acad Child Adolesc Psychiatry.* 2016;55 10 Supp1):S314.

Reason for exclusion: No RCT; Presentation of studies

Wilkison1995
- Wilkison PC. Elevated reward thresholds or disinhibitory psychopathology in attention deficit hyperactivity disordered boys: A test of two hypotheses [Ph.D.]. Ann Arbor, The University of Utah; 1991.
- Wilkison PC, Kircher JC, McMahon WM, Sloane HN. Effects of methylphenidate on reward strength in boys with attention-deficit hyperactivity disorder. *J Am Acad Child Adolesc Psychiatry.* 1995;34(7):897-901.

Reason for exclusion: Less than seven days treatment

Williams1978
- Williams JI, Cram DM, Tausig FT, Webster E. Relative effects of drugs and diet on hyperactive behaviors: an experimental study. *Pediatrics.* 1978;61(6):811-817.

Reason for exclusion: No DSM/ICD criteria

Williams1998
- Williams SE, Ris MD, Ayyangar R, Schefft BK, Berch D. Recovery in pediatric brain injury: is psychostimulant medication beneficial? *The Journal of head trauma rehabilitation.* 1998;13(3):73-81.

Reason for exclusion: Less than seven days treatment; Reason for exclusion: No DSM/ICD criteria

Williams2001
- Williams L. Methylphenidate HCI extended-release tablets for children with ADHD: parent treatment preference and satisfaction. *Pediatr Res.* 2001(4):429a.

Reason for exclusion: No outcome of interest, only abstract, (asked author additional information but no reply)

Williams2003
- Williams GV, Andrews RD, Ordonez CE, et al. Dependency of methylphenidate effects on cerebral glucose metabolism on the functional circuitry engaged by cognition. *Society for Neuroscience Abstract Viewer and Itinerary Planner.* 2003;2003:Abstract No. 668.662.

Reason for exclusion: No participants with ADHD (healthy subjects)

Williams2008
- Williams LM, Hermens DF, Palmer D, et al. Misinterpreting emotional expressions in attention-deficit/hyperactivity disorder: evidence for a neural marker and stimulant effects. *Biol Psychiatry.* 2008;63(10):917-926.

Reason for exclusion: No RCT

Wilson2013
- Wilson TW, Heinrichs-Graham E, White ML, Knott NL, Wetzel MW. Estimating the Passage of Minutes: Deviant Oscillatory Frontal Activity in Medicated and Unmedicated ADHD. *Child Neuropsychol.* 2013;27(6):654-665.

Reason for exclusion: No RCT

Winsberg1972
- Winsberg BG, Bialer I, Kupietz S, Tobias J. Effects of imipramine and dextroamphetamine on behavior of neuropsychiatrically impaired children. *Am J Psychiatry.* 1972;128(11):1425-1431.

Reason for exclusion: No DSM/ICD criteria

Winsberg1996
- Winsberg B, Klee S, Pollack J. Effectiveness of pemoline among hyperkinetic children who fail to respond to methylphenidate CONFERENCE ABSTRACT. *9th European College of Neuropsychopharmacology Congress. Amsterdam, The Netherlands. 21st-25th September, 1996.* 1996.

Reason for exclusion: No treatment of interest for the present meta-analysis; No RCT

Winsberg1974
- Winsberg BG, Press M, Bialer I, Kupietz S. Dextroamphetamine and methylphenidate in the treatment of hyperactive-aggressive children. *Pediatrics.* 1974;53(2):236-241.

Reason for exclusion: No DSM/ICD criteria

Winsberg1980
- Winsberg BG, Kupietz SS, Yepes LE, Goldstein S. Ineffectiveness of imipramine in children who fail to respond to methylphenidate. *J Autism Dev Disord.* 1980;10(2):129-137.

Reason for exclusion: No treatment of interest for the present meta-analysis (imipramine)

Winsberg1982
- Winsberg BG, Kupietz SS, Sverg J, Hungund BL, Young NL. Methylphenidate oral dose plasma concentrations and behavioral response in children. *Psychopharmacology (Berl).* 1982;76(4):329-332.

Reason for exclusion: No randomised

Winsberg1987
- Winsberg B, Matinsky S, Kupietz S, Richardson E. Is there dose-dependent tolerance associated with chronic methylphenidate therapy in hyperactive children: Oral dose and plasma concentrations. *Psychopharmacol Bull.* 1987;23(1):107-110.

Reason for exclusion: No randomized, no diagnostic criteria

Winsberg1988
- Winsberg BG, Maitinsky S, Richardson E, Kupietz SS. Effects of methylphenidate on achievement in hyperactive children with reading disorders. *Psychopharmacol Bull.* 1988;24(2):238-241.

Reason for exclusion: Not randomized; co-treatment

Winsberg1993
- Winsberg BG, Javitt DC, Silipo GS, Doneshka P. Mismatch negativity in hyperactive children: effects of methylphenidate. *Psychopharmacol Bull.* 1993;29(2):229-233.

Reason for exclusion: Participants: responders; Less than seven days treatment

Winsberg1997
- Winsberg BG, Javitt DC, Silipo GS. Electrophysiological indices of information processing in methylphenidate responders. *Biol Psychiatry.* 1997;42(6):434-445.

Reason for exclusion: Single dose

Witcher2003
- Witcher JW, Long A, Smith B, et al. Atomoxetine pharmacokinetics in children and adolescents with attention deficit hyperactivity disorder. *J Child Adolesc Psychopharmacol.* 2003;13(1):53-63.

Reason for exclusion: No double blind RCT

Witt2008 (NCT00341029)

- Witt KL, Shelby MD, Itchon-Ramos N, et al. Methylphenidate and amphetamine do not induce cytogenetic damage in lymphocytes of children with ADHD. *J Am Acad Child Adolesc Psychiatry.* 2008;47(12):1375-1383.
- https://clinicaltrials.gov/ct2/show/NCT00341029

Reason for exclusion: No double blind

Wodrich1998

- Wodrich DL, Kush JC. The effect of methylphenidate on teachers' behavioral ratings in specific school situations. *Psychology in the Schools.* 1998;35(1):81-88.

Cross-over without wash out; pre-cross over data not available

Wolraich1977

- Wolraich ML. Stimulant drug therapy in hyperactive children: research and clinical implications. *Pediatrics.* 1977;60(4):512-518.

Reason for exclusion: Review

Wolraich1978

- Wolraich M, Drummond T, Salomon MK, O'Brien ML, Sivage C. Effects of methylphenidate alone and in combination with behavior modification procedures on the behavior and academic performance of hyperactive children. *J Abnorm Child Psychol.* 1978;6(1):149-161.

Reason for exclusion: No double blind

Wolraich1989

- Wolraich M. Assessing response to methylphenidate for attention deficit disorder. *J Pediatr.* 1989;114(5):902-903.

Reason for exclusion: Letter; no empirical data

Wolraich1999

- Wolraich ML. The difference between efficacy and effectiveness research in studying attention-deficit/hyperactivity disorder. *Arch Pediatr Adolesc Med.* 1999;153:1220-1.

Reason for exclusion: Commentary

Wolraich2001 (NCT00269802)

- Pooled in: Biederman J. An OROS formulation of methylphenidate in the treatment of ADHD. *2001 Annual Meeting of the American Psychiatric Association* 2001.
- Pooled in: Biederman J. An oros formulation of methylphenidate in the treatment of adhd. *155th Annual Meeting of the American Psychiatric Association* 2002.
- Greenhill LL. Efficacy and safety of once-daily methylphenidate HCl, standard methylphenidate and placebo in children with ADHD. Proceedings of the *153rd Annual Meeting of the American Psychiatric Association; 2000 May 13-18; Chicago, Illinois. Chicago, 2000:NR. 667.*
- Greenhill LL. Evaluation of the efficacy and safety of Concerta (Methylphenidate HCI) extended-release tablets, Ritalin, and placebo in children with ADHD. *Neurology* 2000;54(7):A420–1
- Greenhill LL. Efficacy and safety of once-daily methylpheniadate hcl, standard methylphenidate and placebo in children with adhd. *155th Annual Meeting of the American Psychiatric Association 2002.*
- Swanson J, Greenhill L, Pelham W, Wilens T, Wolraich M, Abikoff H, et al. Initiating Concerta (TM) (OROS methylphenidate HCl) qd in children with attention-deficit hyperactivity disorder. *Journal of Clinical Research* 2000;3: 59–76.
- Related to: Wolraich ML. Evaluation of efficacy and safety of OROS methylphenidate HCI (MPH) extended-release tablets, methylphenidate tid, and placebo in children with ADHD. *Pediatr Res.* 2000(4):36.
- Wolraich ML. Efficacy and safety of OROS(r) methylphenidate HCl (mph) extended-release tablets (CONCERTA(tm)), conventional MPH, and placebo in children with ADHD. *Int J Neuropsychopharmacol*.(Abstracts of the XXII CINP Congress, Brussels, Belgium, July 9-13, 2000)2000:S329.
- Wolraich ML, Greenhill LL, Pelham W, et al. Randomized, controlled trial of oros methylphenidate once a day in children with attention-deficit/hyperactivity disorder. *Pediatrics.* 2001;108(4):883-892
- https://clinicaltrials.gov/ct2/show/NCT00269802

Reason for exclusion: First author confirmed that participants were responders to previous ADHD medications

Wolraich2004

- Wolraich M. Once-daily OROS (R) methylphenidate: Response in girls with ADHD. *Pediatr Res.* 2004;55:2A.

Reason for exclusion: Pooled 4 RCTs (contacted author to enquire about this RCTs but not possible to retrieve any additional information)

Wong2012

- Wong CG, Stevens MC. The effects of stimulant medication on working memory functional connectivity in attention-deficit/hyperactivity disorder. *Biol Psychiatry.* 2012;71(5):458-466.

Reason for exclusion: Less than seven days treatment

Wood1976

- Wood DR, Reimherr FW, Wender PH, Johnson GE. Diagnosis and treatment of minimal brain dysfunction in adults: a preliminary report. *Arch Gen Psychiatry.* 1976;33(12):1453-1460.

Reason for exclusion: No DSM/ICD criteria

Wood1985

- Wood DR, Reimherr FW, Wender, PH. Amino acid precursors for the treatment of attention deficit disorder, residual type. *Psychopharmacol Bull.* 1985; 21(1): 146-149

Reason for exclusion: No relevant medications for the present meta-analysis

Worrall1993

- Worrall A. Evaluating the effects of methylphenidate on the cognitive, behavioural and academic performance of A.D.D. children in the classroom. Southern African Journal of Child and Adolescent Psychiatry 1993; 5 (2): 96-101

Reason for exclusion: Less than seven days treatment; not full diagnostic criteria as per protocol

Wray1975

- Wray SR, Egbe P. Studies of the hyperkinetic syndrome -- Part I. An experimental analysis. *West Indian Medical Journal.* 1975;24(3):160-164.

Reason for exclusion: Animal model

Wu2015

- Wu Z, Hoogman M, Cao Q, et al. Laterality of activation patterns in boys with Attention-Deficit/Hyperactivity Disorder and effects of methylphenidate during verbal working memory task. *ADHD Atten Defic Hyperact Disord.* 2015;7:S36.

Reason for exclusion: Single dose

Wulbert1977

- Wulbert M, Dries R. The relative efficacy of methylphenidate (ritalin) and behavior-modification techniques in the treatment of a hyperactive child. *J Appl Behav Anal.* 1977;10(1):21-31.

Reason for exclusion: Single case

Yang2004

- Yang L, Wang Y-F, Li J, Faraone SV. Association of norepinephrine transporter gene with methylphenidate response. *J Am Acad Child Adolesc Psychiatry.* 2004;43(9):1154-1158.

Reason for exclusion: No RCT

Yang2004

- Yang P, Chung L-C, Chen C-S, Chen C-C. Rapid improvement in academic grades following methylphenidate treatment in attention-deficit hyperactivity disorder. *Psychiatry Clin Neurosci.* 2004;58(1):37-41.

Reason for exclusion: No RCT

Yang2007

- Yang P, Hsu H-Y, Chiou S-S, Chao M-C. Health-related quality of life in methylphenidate-treated children with attention-deficit-hyperactivity disorder: results from a Taiwanese sample. *Aust N Z J Psychiatry.* 2007;41(12):998-1004.

Reason for exclusion: No RCT

Yang2010

- Yang R, Mao S, Li R, Zhao Z. More objective tools should be employed to objectify the therapeutic response. *J Dev Behav Pediatr.* 2010;31(9):733.

Reason for exclusion: Letter/commentary

Yang2011

- Yang R, Mao S, Li R, Zhao Z. Several concerns arise when the results are interpreted. *Hum Psychopharmacol.* 2011;26(1):86-87; author reply 88.

Reason for exclusion: Letter/commentary

Yang2012
- Yang P-C, Lung F-W, Chiou S-S, Yen C-F, Fuh J-L. Quality of life of methylphenidate treatment-responsive adolescents with attention-deficit/hyperactivity disorder. *Kaohsiung J Med Sci.* 2012;28(5):279-284.

Reason for exclusion: No RCT

Yang2012 (NCT01065259; CON-I-07-CN-029-B)
- Yang L, Cao Q, Shuai L, Li H, Chan RC, Wang Y. Comparative study of OROS-MPH and atomoxetine on executive function improvement in ADHD: a randomized controlled trial. *The Int J Neuropsychopharmacol. (CINP).*2011:1-12.
- Yang L, Cao Q, Shuai L, Li H, Chan RCK, Wang Y. Comparative study of OROS-MPH and atomoxetine on executive function improvement in ADHD: A randomized controlled trial. *Int J Neuropsychopharmacol.* 2012(1):15-26.

Reason for exclusion: Single blind

Yang2013
- Yang L, Qian Q, Liu L, Li H, Faraone SV, Wang Y. Adrenergic neurotransmitter system transporter and receptor genes associated with atomoxetine response in attention-deficit hyperactivity disorder children. *J Neural Transm.* 2013;120(7):1127-1133.

Reason for exclusion: No double blind, not controlled

Yang2014
- Yang R, Li R. Could atomoxetine improve sluggish cognitive tempo symptoms? *J Child Adolesc Psychopharmacol.* 2014;24(8):462.

Reason for exclusion: Commentary

Yarmolovsky2016
- Yarmolovsky J, Szwarc T, Schwartz M, Tirosh E, Geva R. Hot executive control and response to a stimulant in a double-blind randomized trial in children with ADHD. *Eur Arch Psychiatry Clin Neurosci.* 2017 Feb;267(1):73-82

Reason for exclusion: Less than seven days treatment

Yellin1978
- Yellin AM, Spring C, Greenberg LM. Effects of imipramine and methylphenidate on behavior of hyperactive children. *Research Communications in Psychology, Psychiatry & Behavior.* 1978;3(1):15-26.

Reason for exclusion: No mention to DSM-ICD criteria; not possible to contact authors

Yellin1981a
- Yellin AM, Greenberg LM. Attention-deficit disorder: monitored data-based assessment and treatment. *Minn Med.* 1981;64(8):487-490.

Reason for exclusion: No RCT

Yellin1981b
- Yellin A, Kendall PC, Greenberg L. Cognitive-behavioral therapy and methylphenidate with hyperactive children: Preliminary comparisons. *Research Communications in Psychology, Psychiatry & Behavior* 1981; 6(3): 213-227.

Reason for exclusion: No RCT

Yellin1982
- Yellin AM, Hopwood JH, Greenberg LM. Adults and adolescents with attention deficit disorder: clinical and behavioral responses to psychostimulants. *J Clin Psychopharmacol.* 1982;2(2):133-136.

Reason for exclusion: No RCT

Yepes1977
- Yepes LE, Balka EB, Winsberg BG, Bialer I. Amitriptyline and methylphenidate treatment of behaviorally disordered children. *J Child Psychol.* 1977(1):39-52.

Reason for exclusion: DSM-II

Yildiz2011

- Yildiz O, Sismanlar SG, Memik NC, Karakaya I, Agaoglu B. Atomoxetine and methylphenidate treatment in children with ADHD: the efficacy, tolerability and effects on executive functions. *Child Psychiatry Hum Dev.* 2011;42(3):257-269.

Reason for exclusion: Open label

Yilmaz2013
- Yilmaz S, Akca OF. Effectiveness of methylphenidate in the treatment of encopresis whether or not attention-deficit/hyperactivity disorder symptoms are present. *J Child Adolesc Psychopharmacol.* 2013;23(9):632-633.

Reason for exclusion: Case report

Yilmaz2014
- Yilmaz S, Bilgic A, Herguner S. Effect of OROS methylphenidate on encopresis in children with attention-deficit/hyperactivity disorder. *J Child Adolesc Psychopharmacol.* 2014;24(3):158-160.

Reason for exclusion: No RCT

Yoo2009
- Yoo HK, Park S, Wang H-R, et al. Effect of methylphenidate on the quality of life in children with epilepsy and attention deficit hyperactivity disorder: and open-label study using an osmotic-controlled release oral delivery system. *Epileptic Disord.* 2009;11(4):301-308.

Reason for exclusion: Open label

Young2006
- Young JL. Treatment of adult ADHD and comorbid disorders. *CNS Spectr.* 2006;11(10 Suppl 11):10-12.

Reason for exclusion: Review

Young2013
- Young JL. Use of lisdexamfetamine dimesylate in treatment of executive functioning deficits and chronic fatigue syndrome: A double blind, placebo-controlled study. *Psychiatry Res.* 2013;207(1-2):127-133.

Reason for exclusion: No ADHD

Zachor2006
- Zachor DA, Roberts AW, Hodgens JB, Isaacs JS, Merrick J. Effects of long-term psychostimulant medication on growth of children with ADHD. *Res Dev Disabil.* 2006;27(2):162-174.

Reason for exclusion: No RCT

Zack2009
- Zack M, Poulos CX. Effects of the atypical stimulant modafinil on a brief gambling episode in pathological gamblers with high vs. low impulsivity. *J Psychopharmacol.* 2009;23(6):660-671.

Reason for exclusion: No participants with ADHD

Zahn1975
- Zahn TP, Abate F, Little BC, Wender PH. Minimal brain dysfunction, stimulant drugs, and autonomic nervous system activity. *Arch Gen Psychiatry.* 1975;32(3):381-387.

Reason for exclusion: No DSM/ICD criteria

Zahn1980
- Zahn TP, Rapoport JL, Thompson CL. Autonomic and behavioral effects of dextroamphetamine and placebo in normal and hyperactive prepubertal boys. *J Abnorm Child Psychol.* 1980;8(2):145-160.

Reason for exclusion: Less than seven days treatment

Zalsman2003
- Zalsman G, Pumeranz O, Peretz G, et al. Attention patterns in children with attention deficit disorder with or without hyperactivity. *Scientificworldjournal.* 2003;3:1093-1107.

Reason for exclusion: No RCT and single dose

Zametkin1984
- Zametkin AJ, Brown GL, Karoum F, et al. Urinary phenethylamine response to d-amphetamine in 12 boys with attention deficit disorder. *Am J Psychiatry.* 1984;141(9):1055-1058.
- Zametkin AJ, Karoum F, Linnoila M, et al. Stimulants, urinary catecholamines, and indoleamines in hyperactivity. A

comparison of methylphenidate and dextroamphetamine. *Arch Gen Psychiatry.* 1985;42(3):251-255 (open label)
Reason for exclusion: Associated diet, no mention of randomisation; contacted author but no reply

Zametkin1985
- Zametkin A, Rapoport JL, Murphy DL, Linnoila M, Ismond D. Treatment of hyperactive children with monoamine oxidase inhibitors. I. Clinical efficacy. *Arch Gen Psychiatry.* Oct 1985;42(10):962-966.
- Zametkin A, Rapoport JL, Murphy DL, et al. Treatment of hyperactive children with monoamine oxidase inhibitors. II. Plasma and urinary monoamine findings after treatment. *Arch Gen Psychiatry.* 1985;42(10):969-973.

Reason for exclusion: Medication of interest vs medication of no interest for the present meta-analysis; placebo used only as wash out (not randomized); Co-intervention: dietetic regimen

Zametekin1986
- Zametkin AJ, Reeves JC, Webster L, Werry JS. Promethazine treatment of children with Attention Deficit Disorder with Hyperactivity--ineffective and unpleasant. *J Am Acad Child Psychiatry.*1986;25(6):854-856.

Reason for exclusion: No double blind RCT

Zametkin1986
- Zametkin AJ, Linnoila M, Karoum F, Sallee R. Pemoline and urinary excretion of catecholamines and indoleamines in children with attention deficit disorder. *Am J Psychiatry.* 1986;143(3):359-362.

Reason for exclusion: No double blind RCT (open trial); no medication of interest for the present meta-analysis (pemoline)

Zametkin1988
- Zametkin AJ, Hamburger SD. The effect of methylphenidate on urinary catecholamine excretion in hyperactivity: a partial replication. *Biol Psychiatry.* 1988;23(4):350-356.

Reason for exclusion: No double blind RCT (open trial)

Zametkin1995
- Zametkin AJ. Attention-deficit disorder. Born to be hyperactive? *JAMA.* 1995;273(23):1871-1874.

Reason for exclusion: Case report/commentary

Zamora2011
- Zamora J, Velasquez A, Troncoso L, Barra P, Guajardo K, Castillo-Duran C. Zinc in the therapy of the attention-deficit/ hyperactivity disorder in children. A preliminar randomized controlled trial. [Spanish] *Arch Latinoam Nutr.* 2011;61(3):242-6

Reason for exclusion: Methylphenidatre+placebo (sucrose) vs methylphenidate +zinc

Zang2005
- Zang Y-F, Jin Z, Weng X-C, et al. Functional MRI in attention-deficit hyperactivity disorder: evidence for hypofrontality. *Brain Dev.* 2005;27(8):544-550.

Reason for exclusion: No RCT; single dose

Zarinara2010
- Zarinara AR, Mohammadi MR, Hazrati N, et al. Venlafaxine versus methylphenidate in pediatric outpatients with attention deficit hyperactivity disorder: a randomized, double-blind comparison trial. *Hum Psychopharmacol.* 2010;25(7-8):530-535.

Reson for exclusion: Medication of interest vs medication of no interest for the present meta-analysis, no placebo arm

Zavadenko2008
- Zavadenko NN, Suvorinova N. [Atomoxetine and piracetam in the treatment of attention deficit hyperactivity disorder in children]. *Zhurnal nevrologii i psikhiatrii imeni S.S. Korsakova / Ministerstvo zdravookhraneniia i meditsinskoi promyshlennosti Rossiiskoi Federatsii, Vserossiiskoe obshchestvo nevrologov [i] Vserossiiskoe obshchestvo psikhiat.* 2008;108(7):43-47.

Reason for exclusion: No RCT; no placebo no treatment of interest for the present meta-analysis (piracetam and atomoxetine)

Zeiner1995
- Zeiner P. Body growth and cardiovascular function after ex- tended treatment (1.75 years) with methylphenidate in boys with attention-deficit hyperactivity disorder. *J Child Adolesc Psychopharm.* 1995;5:129-38

Reason for exclusion: Open label trial. Note: Paper not retrieved by our search but provided in the reference list of Zeiner, P. Do the beneficial effects of extended methylphenidate treatment in boys with attention-deficit hyperactivity disorder dissipate rapidly during placebo treatment? Nordic Journal of Psychiatry 1990, 53(1), 55-60.

Zeiner1999

- Zeiner P, Bryhn, G, Bjercke, C, Truyen, K, Strand, G. Response to methylphenidate in boys with attention-deficit hyperactivity disorder. *Acta Paediatr*. 1999, 88(3), 298-303.

Reason for exclusion: no usable data

- Zeiner P. Do the beneficial effects of extended methylphenidate treatment in boys with attention-deficit hyperactivity disorder dissipate rapidly during placebo treatment? *Nord J Psychiatry* 1999;53(1):55-60.

Reason for exclusion: Part of the population of the previous study and part of the following: Body growth and cardiovascular function after extended treatment (1.75 years) with methylphenidate in boys with attention-deficit hyperactivity disorder. J Child Adolesc Psychopharm 1995;5:129-38 (open label)

- *Another paper retrieved in the bibliography of Zeiner et al. 1999 is Zeiner P, Bryhn G, Bjercke C, Truyen K, Strand G. Prediction of response to methylphenidate in boys with attention-deficit hyperactivity disorder. Acta Paediatr 1999;88:1-6. but not possible to retrieve this paper (not possible to find this reference: wrong reference?)*

Zeiner2011

- Zeiner P, Gjevik E, Weidle B. Response to atomoxetine in boys with high-functioning autism spectrum disorders and attention deficit/hyperactivity disorder. *Acta Paediatrica.* 2011;100(9):1258-1261.

Reason for exclusion: Open label

Zeni2007

- Zeni CP, Guimaraes AP, Polanczyk GV, et al. No significant association between response to methylphenidate and genes of the dopaminergic and serotonergic systems in a sample of Brazilian children with attention-deficit/hyperactivity disorder. *Am J Med Genet. Part B, Neuropsychiatric Genetic.* 2007;144B(3):391-394.

Reason for exclusion: No RCT

Zepf2008

- Zepf FD, Stadler C, Demisch L, Schmitt M, Landgraf M, Poustka F. Serotonergic functioning and trait-impulsivity in attention-deficit/hyperactivity-disordered boys (ADHD): Influence of rapid tryptophan depletion. *Hum Psychopharmacol.* 2008;23(1):43-51.

Reason for exclusion: No treatment of interest for the present meta-analysis (RTD Test Moja-De and TRP-balanced placebo)

Zepf2008

- Zepf FD, Holtmann M, Stadler C, et al. Diminished serotonergic functioning in hostile children with ADHD: tryptophan depletion increases behavioural inhibition. *Pharmacopsychiatry.* 2008;41(2):60-65.

Reason for exclusion: No treatment of interest (tryptophan depletion vs placebo)

Zepf2008

- Zepf FD, Wockel L, Poustka F, Holtmann M. Diminished 5-HT functioning in CBCL pediatric bipolar disorder-profiled ADHD patients versus normal ADHD: Susceptibility to rapid tryptophan depletion influences reaction time performance. *Hum Psychopharmacol.* 2008;23(4):291-299.

Reason for exclusion: No treatment of interest (tryptophan depletion vs placebo)

Zepf2009

- Zepf FD, Holtmann M, Stadler C, Magnus S, Wockel L, Poustka F. Diminished central nervous 5-T neurotransmission and mood self-ratings in children and adolescents with ADHD: No clear effect of rapid tryptophan depletion. *Hum Psychopharmacol.* 2009;24(2):87-94.

Reason for exclusion: No treatment of interest for the present meta-analysis (tryptophan depletion vs placebo)

Zeni2009

- Zeni CP, Tramontina S, Ketzer CR, Pheula GF, Rohde LA. Methylphenidate combined with aripiprazole in children and adolescents with bipolar disorder and attention-deficit/hyperactivity disorder: a randomized crossover trial. *J Child Adolesc Psychopharmacol.* 2009;19(5):553-561.

Reason for exclusion: Aripripazole + methylphenidate vs placebo + methylphenidate

Zepf2010

- Zepf FD, Gaber TJ, Baurmann D, et al. Serotonergic neurotransmission and lapses of attention in children and adolescents with attention deficit hyperactivity disorder: availability of tryptophan influences attentional performance. *Int J Neuropsychopharmacol.* 2010;13(7):933-941.

Reason for exclusion: No treatment of interest for the present meta-analysis (tryptophan depletion vs placebo)

Zhang2005

- Zhang S, Faries DE, Vowles M, Michelson D. ADHD Rating Scale IV: psychometric properties from a multinational study as a clinician-administered instrument. *Int J Methods Psychiatr Res.* 2005;14(4):186-201.

Reason for exclusion: No RCT

Zhang2011

- Zhang L, Jin X, Zhang Y. Effect of methylphenidate on intelligence quotient scores in Chinese children with attention-deficit/hyperactivity disorder. *J Clin Psychopharmacol.* 2011;31(1):51-55.

Reason for exclusion: No randomized, no double blind

Zheng2011

- Zheng Y, Wang Y-F, Qin J, et al. Prospective, naturalistic study of open-label OROS methylphenidate treatment in Chinese school-aged children with attention-deficit/hyperactivity disorder. *Chin Med J.* 2011;124(20):3269-3274.

Reason for exclusion: No double blind RCT (open label)

Zhu2013

- Zhu Y, Gao B, Hua J, et al. Effects of methylphenidate on resting-state brain activity in normal adults: an fMRI study. *Neurosci Bull.* 2013;29(1):16-27.

Reason for exclusion: Single dose study

Ziegler2008b

- Ziegler A. Placebo is more effective than St John's Wort in children with ADHD. [German]. *Dtsch Apoth Ztg.* 148(38), 38-39.

Reason for exclusion: Commentary on Weber W, Vander Stoep A, McCarty RL, Weiss NS, Biederman J, McClellan J. Hypericum perforatum (St John's Wort) for attention-deficit/hyperactivity disorder in children and adolescents - A randomized controlled trial. JAMA. 2008;299(22):2633-2641.

Ziegler2008b

- Ziegler R. Phytopharmaceuticals: Is st. John's wort extract effective for adhd?. [German]. Psychopharmakotherapie, 15(6), 284.

Reason for exclusion: Commentary on Weber W, Vander Stoep A, McCarty RL, Weiss NS, Biederman J, McClellan J. Hypericum perforatum (St John's Wort) for attention-deficit/hyperactivity disorder in children and adolescents - A randomized controlled trial. JAMA. 2008;299(22):2633-2641

Ziegler2009

- Ziegler R. Phytopharmaceuticals: Is st. John's wort extract effective in adhd?. [german] phytopharmaka: Johanniskrautextrakt bei adhs wirksam? *Med Monatsschr Pharm.* 2009; 32(2), 74-75.

Reason for exclusion: Commentary on Weber W, Vander Stoep A, McCarty RL, Weiss NS, Biederman J, McClellan J. Hypericum perforatum (St John's Wort) for attention-deficit/hyperactivity disorder in children and adolescents - A randomized controlled trial. Jama-Journal of the American Medical Association. Jun 2008;299(22):2633-2641.

Zrull1963

- Zrull JP, Westman JC, Arthur B, Bell WA. A comparison of chlordiazepoxide, d-amphetamine, and placebo in the treatment of the hyperkinetic syndrome in children. *Am J Psychiatry.* 1963;120:590-591.

Reason for exclusion: No DSM/ICD criteria

Zrull1964

- Zrull JP, Westman JC, Arthur B, Rice DL. A comparison of diazepam, d-amphetamine and placebo in the treatment of the hyperkinetic syndrome in children. *Am J Psychiatry.*1964;121:388-389.

Reason for exclusion: No DSM/ICD criteria

Appendix 8. Studies/citations retained for the network meta-analysis

1. Abikoff2009

- Abikoff H, Nissley-Tsiopinis J, Gallagher R, et al. Effects of MPH-OROS on the organizational, time management, and planning behaviors of children with ADHD. *J Am Acad Child Adolesc Psychiatry.* 2009;48(2):166-175.
- Anonymous. Concerta for organizational deficits in ADHD. *The Brown University Child and Adolescent Behavior Letter* 2009;25;3:2.

2. Adler2008a, B4Z-MC-LYBV, NCT00190931

- Levine L, Tamura RN, Kelsey DK, Schoepp DD, Allen AJ. Functional outcomes in adults with attention-deficit/hyperactivity disorder following treatment with atomoxetine vs. placebo. *Neuropsychopharmacology.* 2005;30(Suppl. 1):S137.
- Post hoc analysis (with a subsample of subjects) in: Matza LS, Johnston JA, Faries DE, Malley KG, Brod M. Responsiveness of the Adult Attention-Deficit/Hyperactivity Disorder Quality of Life Scale (AAQoL). *Qual Life Res.* 2007;16(9):1511-1520.
- Adler LA, Spencer TJ, Levine LR, et al. Functional outcomes in the treatment of adults with ADHD. *J Atten Disord.* 2008;11(6):720-727.
- Pooled in: Adler L, Wilens T, Zhang S, et al. Retrospective safety analysis of atomoxetine in adult ADHD patients with or without comorbid alcohol abuse and dependence. *Am J Addict.* 2009;18(5):393-401.
- Previous reference related to Adler L, Wilens T, Zhang S, et al. Safety of atomoxetine in ADHD patients with or without comorbid alcohol abuse and dependence. Proceedings of the 70th Annual Scientific Meeting of the College on Problems of Drug Dependence; 2008 June 16-21; San Juan, Puerto Rico, USA2008:2.
- https://clinicaltrials.gov/ct2/show/NCT00190931
- https://assets.contentful.com/hadumfdtzsru/6JMvAj7xM4Q6McgwKiYSwg/062c958c27aa48897f2b098839213bd3/Atomoxetine-B4Z-MC-LYBV.pdf
- https://www.accessdata.fda.gov/drugsatfda_docs/nda/2002/21-411_Strattera.cfm
- Full CSR provided by manufacturer

3. Adler2008b, NRP104.303, NCT00334880

- Adler LA, Goodman DW, Kollins SH, et al. Double-blind, placebo-controlled study of the efficacy and safety of lisdexamfetamine dimesylate in adults with attention-deficit/hyperactivity disorder. *J Clin Psychiatry.* 2008;69(9):1364-1373.
- Goodman D, Adler L, Kollins SH, et al. Efficacy and safety of lisdexamfetamine dimesylate in adults with attention-deficit/hyperactivity disorder. *Int J Neuropsychopharmacol.* 2008;11(Suppl. 1):292-293.
- Post hoc analysis with additional data pertinent for the present meta-analysis: Adler LA, Weisler RH, Goodman DW, Hamdani M, Niebler GE. Short-term effects of lisdexamfetamine demesylate on cardiovascular parameters in 4-week clinical trial in adults with attention-deficits/hyperactivity disorder. *J Clin Psychiatry.* 2009;70(12):1652-1661.
- Open label follow-up in: Weisler R, Young J, Mattingly G, Gao J, Squires L, Adler L. Long-term safety and effectiveness of lisdexamfetamine dimesylate in adults with attention-deficit/ hyperactivity disorder. *CNS Spectr.* 2009;14(10):573-585.
- Sleep outcomes in: Adler LA, Goodman D, Weisler R, Hamdani M, Roth T. Effect of lisdexamfetamine dimesylate on sleep in adults with attention-deficit/hyperactivity disorder. *Behav Brain Funct.* 2009;5:34.
- Pooled in: Goodman D, Faraone SV, Adler LA, Dirks B, Hamdani M, Weisler R. Interpreting ADHD rating scale scores: Linking ADHD rating scale scores and CGI levels in two randomized controlled trials of lisdexamfetamine dimesylate in ADHD. *Prim Psychiatry.* 2010;17(3):44-52.
- Pooled in: Lasser R, Dirks B, Adeyi B, Babcock T. Comparative efficacy and safety of lisdexamfetamine dimesylate and mixed amphetamine salts extended release in adults with attention-deficit/hyperactivity disorder. *Prim Psychiatry.* 2010;17(9):44-54.
- Pooled in: Surman CB, Roth T. Impact of stimulant pharmacotherapy on sleep quality: post hoc analyses of 2 large, double-blind, randomized, placebo-controlled trials. *J Clin Psychiatry.* 2011;72(7):903-908.
- Pooled in: Ginsberg L, Katic A, Adeyi B, et al. Long-term treatment outcomes with lisdexamfetamine dimesylate for adults with attention-deficit/hyperactivity disorder stratified by baseline severity. *Curr Med Res Opin.* 2011;27(6):1097-1107.
- Post hoc analyses in: Kollins SH, Youcha S, Lasser R, Thase ME. Lisdexamfetamine dimesylate for the treatment of attention deficit hyperactivity disorder in adults with a history of depression or history of substance use disorder. *Innov Clin Neurosci.* 2011;8(2):28-32.
- Pooled in: Waxmonsky JG, Waschbusch DA, Glatt SJ, Faraone SV. Prediction of placebo response in 2 clinical

trials of lisdexamfetamine dimeslylate for the treatment of ADHD. *J Clin Psychiatry.* 2011;72(10):1366-1375.

- Analysis in subpopulation in: Babcock T, Dirks B, Adeyi B, Scheckner B. Efficacy of lisdexamfetamine dimesylate in adults with attention-deficit/hyperactivity disorder previously treated with amphetamines: analyses from a randomized, double-blind, multicenter, placebo-controlled titration study. *BMC Pharmacol Toxicol.* 2012;13:18.
- Post hoc analysis in: Faraone SV, Spencer TJ, Kollins SH, Glatt SJ, Goodman D. Dose response effects of lisdexamfetamine dimesylate treatment in adults with ADHD: an exploratory study. *J Atten Disord.* 2012;16(2):118-127.
- Pooled in: Mattingly GW, Weisler RH, Young J, et al. Clinical response and symptomatic remission in short- and long-term trials of lisdexamfetamine dimesylate in adults with attention-deficit/hyperactivity disorder. *BMC Psychiatry.* 2013;13:39.
- Included in: Coghill D, Sorooshian S, Caballero B. Safety outcomes from the clinical development programme for lisdexamfetamine dimesylate: A prodrug stimulant for the treatment of attention-deficit/hyperactivity disorder. *Eur Child Adolesc Psychiatry.* 2013;1):S224
- Pooled in: Adler LA, Dirks B, Adeyi B, Squires LA. Efficacy Outcomes in Age and Sex Subgroups from Two Clinical Trials of Lisdexamfetamine Dimesylate in the Treatment of Adults with Attention-Deficit/Hyperactivity Disorder. *Eur Psychiatry.* 2014;29, S1:1
- Pooled in: Weisler RH, Adler LA, Kollins SH, et al. Analysis of individual items on the attention deficit/hyperactivity disorder symptom rating scale in children and adults: The effects of age and sex in pivotal trials of lisdexamfetamine dimesylate. *Neuropsychiatr Dis Treat.* 2014;10:1-12.
- https://clinicaltrials.gov/ct2/show/NCT00334880
- https://www.shireaustralia.com.au/-/media/shire/shireglobal/shireaustralia/pdffiles/product%20information/vyvanse-pi.pdf
- Additional data/information provided by Shire from full CSR

4. Adler2009a, B4Z-US-LYDQ, NCT00190879

- Adler LA, Liebowitz M, Kronenberger W, et al. Atomoxetine treatment in adults with attention-deficit/hyperactivity disorder and comorbid social anxiety disorder. *Depress Anxiety.* 2009;26(3):212-221.
- https://clinicaltrials.gov/ct2/show/NCT00190879
- https://assets.contentful.com/hadumfdtzsru/5zxD0FZNfiQ6SSqmoqwwge/8b34012d75dbb9544834e1b4b57a03ab/Atomoxetine-B4Z-US-LYDQ.pdf
- https://www.accessdata.fda.gov/drugsatfda_docs/nda/2002/21-411_Strattera.cfm
- Additional data/information provided by Lilly

5. Alder2009b, B4Z-US-LYCU, NCT00190736

- Adler LA, Spencer T, Brown TE, et al. Once-daily atomoxetine for adult attention-deficit/hyperactivity disorder: a 6-month, double-blind trial. *J Clin Psychopharmacol.* 2009;29(1):44-50.
- Spencer TJ, Adler LA, Meihua Q, et al. Validation of the adult ADHD investigator symptom rating scale (AISRS). *J Atten Disord.* 2010;14(1):57-68.
- Summary and critical analysis in: Lall AS, Averbuch R. Once-daily atomoxetine in the treatment of attention-deficit/hyperactivity disorder in adults. *Curr Psychiatry Rep.* 2010;12(5):363-365.
- Additional analysis in: Brown TE, Holdnack J, Saylor K, et al. Effect of atomoxetine on executive function impairments in adults with ADHD. *J Atten Disord.* 2011;15(2):130-138.
- Pooled in: Wietecha LA, Clemow DB, Buchanan AS, Young JL, Sarkis EH, Findling RL. Atomoxetine Increased Effect over Time in Adults with Attention-Deficit/Hyperactivity Disorder Treated for up to 6 Months: Pooled Analysis of Two Double-Blind, Placebo-Controlled, Randomized Trials. *CNS Neurosci Ther.* 2016;22(7):546-557.
- https://clinicaltrials.gov/ct2/show/NCT00190736
- https://assets.contentful.com/hadumfdtzsru/4NjwYiwKeckyyuA0IIcSK8/45a6c712ee38a7f228914fa31b6cf6df/Atomoxetine-B4Z-US-LYCU.pdf
- https://www.accessdata.fda.gov/drugsatfda_docs/nda/2002/21-411_Strattera.cfm
- Full CSR provided by Lilly

6. Adler2009c, CR011560, NCT00326391

- Adler LA, Zimmerman B, Starr HL, et al. Efficacy and safety of OROS methylphenidate in adults with attention-deficit/hyperactivity disorder: a randomized, placebo-controlled, double-blind, parallel group, dose-escalation study. *J Clin Psychopharmacol.* 2009;29(3):239-247.
- Part of subjects participated in: Adler LA, Orman C, Starr HL, et al. Long-term safety of OROS methylphenidate in adults with attention-deficit/hyperactivity disorder: an open-label, dose-titration, 1-year study. *J Clin Psychopharmacol.* 2011;31(1):108-114. (NCT00326300).
- https://clinicaltrials.gov/ct2/show/NCT00326391

- https://www.accessdata.fda.gov/drugsatfda_docs/nda/2000/21-121_Concerta.cfm
- Additional data/information provided by Janssen

7. Adler2013, SPD489-403, NCT01101022

- Adler LA, Dirks B, Deas PF, et al. Lisdexamfetamine dimesylate in adults with attention-deficit/ hyperactivity disorder who report clinically significant impairment in executive function: results from a randomized, double-blind, placebo-controlled study. *J Clin Psychiatry.* 2013;74(7):694-702.
- Adler LA, Dirks B, Deas P, et al. Self-Reported quality of life in adults with attention-deficit/hyperactivity disorder and executive function impairment treated with lisdexamfetamine dimesylate: a randomized, double-blind, multicenter, placebo-controlled, parallel-group study. *BMC Psychiatry.* 2013;13:253
- Weisler R, Ginsberg L, Dirks B, Deas P, Adeyi B, Adler LA. Treatment With Lisdexamfetamine Dimesylate Improves Self- and Informant-Rated Executive Function Behaviors and Clinician- and Informant-Rated ADHD Symptoms in Adults: Data From a Randomized, Double-Blind, Placebo-Controlled Study. *J Atten Disord.* 2014, in press, 10.1177/1087054713518242
- Post hoc analyses: Adler L, Wu J, Madhoo M, Caballero B. Post hoc analyses of the efficacy of lisdexamfetamine dimesylate in adults previously treated with attention deficit/hyperactivity disorder medication. *Eur Psychiatry.* 2015;30, S1:566.
- https://clinicaltrials.gov/ct2/show/NCT01101022
- http://www.shiretrials.com/-/media/files/clinical%20trials/clinicaltrialsen/clinical%20study%20reports/shire-spd489-403-clinical-study-report-redact.pdf
- Additional data/information provided by Shire

8. Allen2005, B4Z-MC-LYAS

- Lewis D, Linder S, Kurlan R, et al. Atomoxetine for the treatment of attention deficit hyperactivity disorder and comorbid tics in children. *Ann Neurol.* 2003;54(Suppl. 7):S106.
- Bangs ME, Allen AJ, Kurlan R, et al. Atomoxetine treatment in children with attention-deficit/hyperactivity disorder and comorbid TIC disorders. *Int J Neuropsychopharmacol*. 2004;7:S441.
- Coffey BJ, Kelsey DK, Feldman PD, et al. Atomoxetine treatment in children with ADHD and comorbid tic disorders. *157th Annual Meeting of the American Psychiatric Association; 2004 May 1-6; New York, NY 2004.*
- Allen AJ, Kurlan RM, Gilbert DL, et al. Atomoxetine treatment in children and adolescents with ADHD and comorbid tic disorders. *Neurology.* 2005;65(12):1941-1949.
- Post hoc subgroup analysis in: Spencer TJ, Sallee FR, Gilbert DL, et al. Atomoxetine treatment of ADHD in children with comorbid Tourette syndrome. *J Atten Disord.* 2008;11(4):470-481.
- Pooled in: Newcorn JH, Sutton VK, Zhang S, et al. Characteristics of placebo responders in pediatric clinical trials of attention-deficit/hyperactivity disorder. *J Am Acad Child Adolesc Psychiatry.* 2009;48(12):1165-1172.
- Commentary in: Anonymous. Atomoxetine may improve tic severity in children with ADHD, Tourette syndrome. *Brown university child & adolescent psychopharmacology update.* 2017;10(3):1, 6-7
- https://assets.contentful.com/hadumfdtzsru/5dD90Njz9uwIc2k00aEASe/ecc2afee105479b66fcc818a322d997d/Atomoxetine-B4Z-MC-LYAS.pdf
- Full CSR provided by Lilly

9. Amiri2008

- Amiri S, Mohammadi MR, Mohammadi M, Nouroozinejad GH, Kahbazi M, Akhondzadeh S. Modafinil as a treatment for Attention-Deficit/Hyperactivity Disorder in children and adolescents: a double blind, randomized clinical trial. *Prog Neuropsychopharmacol Biol Psychiatry.* 2008;32(1):145-149.

10. Arnold2006

- Arnold LE, Aman MG, Cook AM, et al. Atomoxetine for hyperactivity in autism spectrum disorders: placebo-controlled crossover pilot trial. *J Am Acad Child Adolesc Psychiatry.* 2006;45(10):1196-1205.
- Additional data/information provided by the authors

11. Arnold2014, C1538/2027/AD/US, NCT00315276

- Arnold VK, Feifel D, Earl CQ, Yang R, Adler LA. A 9-week, randomized, double-blind, placebo-controlled, parallel-group, dose-finding study to evaluate the efficacy and safety of modafinil as treatment for adults with ADHD. *J Atten Disord.* 2014;18(2):133-144.
- https://clinicaltrials.gov/ct2/show/NCT00315276 (additional ID: C1538/2027/AD/US)

12. Bain2013, NCT00429091

- Bain E, Robieson W, Garimella T, Abi-Saab W, Apostol G, Saltarelli MD. A randomized, double-blind, placebo-controlled Phase 2 study of alpha4beta2 agonist ABT-894 in adults with ADHD. *Biochem Pharmacol.* 2011;82 (8):1043.
- Bain EE, Robieson W, Pritchett Y, et al. A randomized, double-blind, placebo-controlled phase 2 study of 42 agonist ABT-894 in adults with ADHD. *Neuropsychopharmacology*. 2013;38(3):405-413
- https://clinicaltrials.gov/ct2/show/NCT00429091

13. Bangs2007, B4Z-MC-LYAX
- Bangs ME, Emslie GJ, Spencer TJ, et al. A study of atomoxetine in adolescents with ADHD and comorbid depression. *45th Annual NCDEU (New Clinical Drug Evaluation Unit) Meeting; 2005 June 6 - 9; Boca Raton, FL. 2005*:184.
- Bangs ME, Emslie GJ, Spencer TJ, et al. Efficacy and safety of atomoxetine in adolescents with attention-deficit/hyperactivity disorder and major depression. *J Child Adolesc Psychopharmacol.* 2007;17(4):407-420.
- Pooled in: Newcorn JH, Sutton VK, Zhang S, et al. Characteristics of placebo responders in pediatric clinical trials of attention-deficit/hyperactivity disorder. *J Am Acad Child Adolesc Psychiatry.* 2009;48(12):1165-1172.
- http://art45-paediatric-studies-docs.ema.europa.eu/GROUP%20A/Atomoxetine/atomoxetine%20B4Z-MC-LYAX%20-%20clinical%20study%20summary.pdf https://www.accessdata.fda.gov/drugsatfda_docs/nda/2002/21-411_Strattera.cfm
- Full CSR provided by Lilly

14. Bangs2008 , B4Z-MC-LYBX, NCT00191698
- Bangs ME, Hazell P, Danckaerts M, et al. Atomoxetine for the treatment of attention-deficit/hyperactivity disorder and oppositional defiant disorder. [Erratum appears in *Pediatrics*. 2008;122(1): 227]. *Pediatrics.* 2008;121(2):e314-320.
- Post hoc analysis in: Hazell P, Becker K, Nikkanen EA, et al. Relationship between atomoxetine plasma concentration, treatment response and tolerability in attention-deficit/hyperactivity disorder and comorbid oppositional defiant disorder. *Atten Defic Hyperact Disord.* 2009;1(2):201-210.
- https://clinicaltrials.gov/ct2/show/NCT00191698
- https://assets.contentful.com/hadumfdtzsru/1S1dKYi8huoyqCecagkagQ/eff47adc1fb45190a286081c7b731169/Atomoxetine-B4Z-MC-LYBX.pdf
- https://www.accessdata.fda.gov/drugsatfda_docs/nda/2002/21-411_Strattera.cfm
- Full CSR provided by Lilly

15. Bedard2015, NCT00183391
- Bedard AC, Stein MA, Halperin JM, Krone B, Rajwan E, Newcorn JH. Differential impact of methylphenidate and atomoxetine on sustained attention in youth with attention-deficit/hyperactivity disorder. *J Child Psychol Psychiatry.* 2015;56(1):40-48.
- Newcorn J. ADHD and disruptive behavior disorders: Neurobiology and response to stimulant and non-stimulant treatment. *Neuropsychopharmacology.* 2016;41:S56.
- https://www.clinicaltrials.gov/ct2/show/NCT00183391
- http://www.shirecanada.com/-/media/shire/shireglobal/shirecanada/pdffiles/product%20information/adderall-xr-pm-en.pdf
- https://www.accessdata.fda.gov/drugsatfda_docs/nda/2001/21303_Adderall.cfm

16. Biederman2002, SLI 381-301
- Lopez FA, Biederman J, Chandler M, Smith W, Boellner SW, Arnold LE. Randomized, controlled trial of a once-daily amphetamine formulation, SLI381, in children with attention-deficit hyperactivity disorder. *Ann Neurol.* 2001;50(3):S96-S96.
- Biederman J, Lopez FA, Boellner SW, Chandler MC. A randomized, double-blind, placebo-controlled, parallel-group study of SLI381 (Adderall XR) in children with attention-deficit/hyperactivity disorder. *Pediatrics.* 2002;110(2 Pt 1):258-266.
- Findling RL, McCracken JT, Frazer N, Tulloch SJ. Time course of vital signs after once-daily adderall extended release in ADHD children. *156th Annual Meeting of the American Psychiatric Association; 2003 May 17-22; San Francisco, CA 2003:Nr649*
- Findling RL, Biederman J, Wilens TE, et al. Short- and long-term cardiovascular effects of mixed amphetamine salts extended release in children. *J Pediatr.* 2005;147(3):348-354.
- Pooled in: McGough JJ, Biederman J, Wigal SB, et al. Long-term tolerability and effectiveness of once-daily mixed amphetamine salts (Adderall XR) in children with ADHD. *J Am Acad Child Adolesc Psychiatry.* 2005;44(6):530-538.

- Additional information provided by Shire

17. Biederman2005, Study 311 Cephalon

- Biederman J, Swanson JM, Lopez FA. Modafinil improves adhd symptoms in children in a randomized, double-blind, placebo-controlled study. *156th Annual Meeting of the American Psychiatric Association, May 17-22, San Francisco CA. 2003*:No. 36.
- Biederman J, Swanson JM, Wigal SB, et al. Efficacy and safety of modafinil film-coated tablets in children and adolescents with attention-deficit/hyperactivity disorder: results of a randomized, double-blind, placebo-controlled, flexible-dose study. *Pediatrics.* 2005;116(6):e777-784.
- Swanson JM, Boellner S, Rugino T, Sangal RB, Wigal SB. Pediatric formulation of modafinil effective in children and adolescents with adhd. *158th Annual Meeting of the American Psychiatric Association; 2005 May 21-26; Atlanta, GA.2005*:No. 47.
- Pooled in: Wigal SB, Biederman J, Swanson JM, Yang R, Greenhill LL. Efficacy and safety of modafinil film-coated tablets in children and adolescents with or without prior stimulant treatment for attention-deficit/hyperactivity disorder: pooled analysis of 3 randomized, double-blind, placebo-controlled studies. *Prim Care Companion J Clin Psychiatry.* 2006;8(6):352-360.
- Barry RJ, Clarke AR. Modafinil improves symptoms of ADHD compared with placebo in young people. *Evid Based Ment Health.* 2006;9(3):68.
- Pooled in: Biederman J, Pliszka SR. Modafinil improves symptoms of attention-deficit/hyperactivity disorder across subtypes in children and adolescents. *J Pediatr.* 2008;152(3):394-399.
- https://www.accessdata.fda.gov/drugsatfda_docs/nda/98/020717A_Provigil.cfm
- Modafinil (CEP-1538) Tablets, Supplemental NDA 20-717/S-019, ADHD Indication
  Briefing Document for Psychopharmacologic Drugs Advisory Committee Meeting (Document provided by Dr Eric Konofal)

18. Biederman2006a, (subsample of NCT00181571)

- Biederman J, Spencer T, Surman C, et al. A double-blind placebo-controlled randomized study of OROS methylphenidate in the treatment of adults with attention deficit hyperactivity disorder (ADHD): An interim analysis. *Biol Psychiatry.* 2005;57(8):106S-106S
- Biederman J, Mick E, Surman C, et al. A randomized, double-blind, placebo-controlled study of OROS methylphenidate in the treatment of adults with attention-deficit/hyperactivity disorder. *Eur Neuropsychopharmacol.*2005;15(Suppl. 3):S631
- Biederman J, Mick E, Surman C, et al. A randomized, placebo-controlled trial of OROS methylphenidate in adults with attention-deficit/hyperactivity disorder. *Biol Psychiatry.* 2006;59(9):829-835.
- Post hoc analysis in: Mick E, Biederman J, Spencer T, Faraone SV, Sklar P. Absence of association with DAT1 polymorphism and response to methylphenidate in a sample of adults with ADHD. *Am J Med Genet B Neuropsychiatr Genet.* 2006;141B(8):890-894
- Pooled in: Biederman J, Mick EO, Surman C, et al. Comparative acute efficacy and tolerability of OROS and immediate release formulations of methylphenidate in the treatment of adults with attention-deficit/hyperactivity disorder. *BMC Psychiatry.* 2007;7:49.
- Post hoc analysis in: Mick E, Faraone SV, Spencer T, Zhang HF, Biederman J. Assessing the validity of the Quality of Life Enjoyment and Satisfaction Questionnaire Short Form in adults with ADHD. *J Atten Disord.* 2008;11(4):504-509
- Related to previous: Biederman J, Spencer TJ, Mick E, Suppl. Comparative acute efficacy and tolerability of OROS and immediate release formulations of methylphenidate in the treatment of adults with ADHD. Eur Neuropsychopharmacol. 2006; 16: 8-21
- Related to previous: Biederman J. Comparative and acute efficacy and tolerability of OROS and immediate release formulations of methylphenidate in the treatment of adults with attention deficit hyperactivity disorder. *Biol Psychiatry*. 2006; 59(8, Suppl. S): 115.
- Part of subjects from this study participated in: Bush G, Spencer TJ, Holmes J, et al. Functional magnetic resonance imaging of methylphenidate and placebo in attention-deficit/hyperactivity disorder during the multi-source interference task. *Arch Gen Psychiatry.* 2008;65(1):102-114

Note: Biederman2006a is a subsample of Biederman2010 (NCT00181571) (see list of excluded studies)

19. Biederman2006b

- Biederman J, Swanson JM, Lopez FA. Modafinil improves adhd symptoms in children in a randomized, double-blind, placebo-controlled study. *156th Annual Meeting of the American Psychiatric Association, May 17-22, San Francisco CA. 2003*:No. 36.
- Swanson JM, Greenhill LL, Biederman J. Modafinil in children with ADHD: a randomized, placebo-controlled

study. *156th Annual Meeting of the American Psychiatric Association; 2003 May 17-22; San Francisco, CA. 2003*:No. 44.
- Biederman J, Swanson JM, Boellner SW, Lopez E. Modafinil as therapy for ADHD in children: A 4-week, double-blind, placebo-controlled study. *Eur Neuropsychopharmacol.* 2004;14, S3, S364.
- Swanson JM, Biederman J, Boelliner SW, Lopez FA. Modafinil as therapy for ADHD in children: A 4-week, doubleblind, placebo-controlled study. *J Psychopharmacol.* 2004;18(3, Suppl. S):A53.
- Biederman J, Swanson JM, Wigal SB, Boellner SW, Earl CQ, Lopez FA. A comparison of once-daily and divided doses of modafinil in children with attention-deficit/hyperactivity disorder: a randomized, double-blind, and placebo-controlled study. *J Clin Psychiatry.* 2006;67(5):727-735.

20. Biederman2007, NRP104-301, NCT00248092
- Biederman J, Krishnan S, Zhang Y, McGough JJ, Findling RL. Efficacy and tolerability of lisdexamfetamine dimesylate (NRP-104) in children with attention-deficit/hyperactivity disorder: a phase III, multicenter, randomized, double-blind, forced-dose, parallel-group study. *Clin Ther.* 2007;29(3):450-463.
- Lopez FA, Ginsberg LD, Arnold V. Effect of lisdexamfetamine dimesylate on parent-rated measures in children aged 6 to 12 years with attention-deficit/hyperactivity disorder: a secondary analysis. *Postgrad Med.* 2008;120(3):89-102.
- Findling RL, Adeyi B, Chen G, et al. Clinical response and symptomatic remission in children treated with lisdexamfetamine dimesylate for attention-deficit/hyperactivity disorder. *CNS Spectr.* 2010(9):559-568.
- Pooled for a post hoc analysis in: Goodman D, Faraone SV, Adler LA, Dirks B, Hamdani M, Weisler R. Interpreting ADHD rating scale scores: Linking ADHD rating scale scores and CGI levels in two randomized controlled trials of lisdexamfetamine dimesylate in ADHD. *Primary Psychiatry.* 2010;17(3):44-52.
- Pooled for a post hoc analysis in: Waxmonsky JG, Waschbusch DA, Glatt SJ, Faraone SV. Prediction of placebo response in 2 clinical trials of lisdexamfetamine dimeslylate for the treatment of ADHD. *J Clin Psychiatry.* 2011;72(10):1366-1375.
- Post hoc analysis in: Jain R, Babcock T, Burtea T, et al. Efficacy of lisdexamfetamine dimesylate in children with attention-deficit/hyperactivity disorder previously treated with methylphenidate: a post hoc analysis. *Child Adolesc Psychiatry Ment Health.* 2011;5(1):35. (NCT00556296)
- Previous reference related to: Conference proceeding: Jain R, Babcock T, Burtea T, et al. Efficacy of lisdexamfetamine dimesylate in children with attention-deficit/hyperactivity disorder and suboptimal response to methylphenidate. *163rd Annual Meeting of the American Psychiatric Association; 2010 May 22-26; New Orleans, LA* 2010.
- Jain R, Duncan D, Babcock T, et al. Lisdexamfetamine dimesylate (LDX) in children with ADHD after suboptimal response to methylphenidate. *Eur Child Adolesc Psychiatry.* 2011;20:S126-S127.
- Pooled for a post hoc analysis in: McGough JJ, Greenbaum M, Adeyi B, et al. Sex subgroup analysis of treatment response to lisdexamfetamine dimesylate in children aged 6 to 12 years with attention-deficit/hyperactivity disorder. *J Clin Psychopharmacol.* 2012;32(1):138-140.
- Included in: Coghill D, Sorooshian S, Caballero B. Safety outcomes from the clinical development programme for lisdexamfetamine dimesylate: A prodrug stimulant for the treatment of attention-deficit/hyperactivity disorder. *Eur Child Adolesc Psychiatry*. 2013;1):S224.
- Post hoc analysis in: Childress AC, Arnold V, Adeyi B, et al. The effects of lisdexamfetamine dimesylate on emotional lability in children 6 to 12 years of age with ADHD in a double-blind placebo-controlled trial. *J Atten Disord.* 2014;18(2):123-132.
- Used in a post hoc analysis in: Weisler RH, Adler LA, Kollins SH, et al. Analysis of individual items on the attention deficit/hyperactivity disorder symptom rating scale in children and adults: The effects of age and sex in pivotal trials of lisdexamfetamine dimesylate. *Neuropsychiatr Dis Treat.* 2014;10:1-12.
- Lopez FA, Childress A, Adeyi B, et al. ADHD Symptom Rebound and Emotional Lability With Lisdexamfetamine Dimesylate in Children Aged 6 to 12 Years. *J Atten Disord.* 2017;21(1):52-61.
- https://clinicaltrials.gov/ct2/show/NCT00248092
- https://www.accessdata.fda.gov/drugsatfda_docs/nda/2007/021977s000TOC.cfm
- Additional data provided by Shire

21. Biederman2008, SPD503-301, NCT00152009
- Biederman J, Melmed RD, Patel A, et al. A randomized, double-blind, placebo-controlled study of guanfacine extended release in children and adolescents with attention-deficit/hyperactivity disorder. *Pediatrics.* 2008;121(1):e73-84.
- Open label continuation phase: Biederman J, Melmed RD, Patel A, McBurnett K, Donahue J, Lyne A. Long-term, open-label extension study of guanfacine extended release in children and adolescents with ADHD. *CNS Spectr.* 2008;13(12):1047-1055.

- Pooled in: Faraone SV, Glatt SJ. Effects of extended-release guanfacine on ADHD symptoms and sedation-related adverse events in children with ADHD. *J Atten Disord.* 2010;13(5):532-538.
- Pooled in: Sallee FR, Kollins SH, Wigal TL. Efficacy of guanfacine extended release in the treatment of combined and inattentive only subtypes of attention-deficit/hyperactivity disorder. *J Child Adolesc Psychopharmacol.* 2012;22(3):206-214.
- Pooled in: Huss M, McBurnett K, Cutler AJ, et al. Separating efficacy and sedative effects of guanfacine extended release in children and adolescents with ADHD from four randomized, controlled, phase 3 clinical trials. *Eur Psychiatry.* 2016;33:S76-S77.
- https://clinicaltrials.gov/ct2/show/NCT00152009
- https://www.accessdata.fda.gov/drugsatfda_docs/nda/2009/022037_intuniv_toc.cfm
- http://www.shirecanada.com/-/media/shire/shireglobal/shirecanada/pdffiles/product%20information/intuniv-xr-pm-en.pdf
- Additional data provided by Shire

22. Biederman2012, 2008P000971, NCT00801229

- Biederman J, Fried R, Hammerness P, et al. The effects of lisdexamfetamine dimesylate on the driving performance of young adults with ADHD: a randomized, double-blind, placebo-controlled study using a validated driving simulator paradigm. *J Psychiatr Res.* 2012;46(4):484-491.
- Biederman J, Fried R, Hammerness P, et al. The effects of lisdexamfetamine dimesylate on driving behaviors in young adults with ADHD assessed with the Manchester driving behavior questionnaire. *J Adolesc Health.* 2012;51(6):601-607.
- Biederman J, Fried R. The effects of lisdexamfetamine dimesylate on the driving performance of young adults with ADHD. *Eur Neuropsychopharmacol.* 2012;22:S436.
- https://clinicaltrials.gov/ct2/show/NCT00801229 (other ID: 2008P000971)

23. Biehl2016

- Biehl SC, Merz CJ, Dresler T, et al. Increase or Decrease of fMRI Activity in Adult Attention Deficit/ Hyperactivity Disorder: Does It Depend on Task Difficulty? *Int J Neuropsychopharmacol.* 2016; doi: 10.1093/ijnp/pyw049
- https://clinicaltrials.gov/ct2/show/NCT01351272
- https://www.clinicaltrialsregister.eu/ctr-search/search?query=eudract_number:2008-006242-26 (EU 2008-006242-26)
- Additional information provided by the first author

24. Block2009, B4Z-US-LYCC, NCT00486122

- Sutton V, Kelsey D, Lewis D, Schuh K, Sumner C, Quintana H. Morning-dosed Versus evening-dosed atomoxetine for treating ADHD in children. *158th Annual Meeting of the American Psychiatric Association; 2005 May 21-26; Atlanta, GA.* 2005.
- Kelsey DK, Sutton V, Lewis D, Schuh K, Sumner C, Quintana H. Morning-versus evening-dosed atomoxetine: effects on core ADHD symptoms. *158th Annual Meeting of the American Psychiatric Association; 2005 May 21-26; Atlanta, GA, 2005*
- Related: Kratochvil CJ, Faries D, Vaughan B, et al. Emotional expression during attention-deficit/hyperactivity disorders treatment: initial assessment of treatment effects. *J Child Adolesc Psychopharmacol.* 2007;17(1):51-62.
- Block SL, Kelsey D, Coury D, et al. Once-daily atomoxetine for treating pediatric attention-deficit/hyperactivity disorder: comparison of morning and evening dosing. *Clin Pediatr (Phila).* 2009;48(7):723-733.
- Pooled in: Newcorn JH, Sutton VK, Zhang S, et al. Characteristics of placebo responders in pediatric clinical trials of attention-deficit/hyperactivity disorder. *J Am Acad Child Adolesc Psychiatry.* 2009;48(12):1165-1172.
- Pooled in: Block SL, Williams D, Donnelly CL, Dunn DW, Saylor KE, Ruberg SJ. Post hoc analysis: early changes in ADHD-RS items predict longer term response to atomoxetine in pediatric patients. *Clin Pediatr (Phila).* 2010;49(8):768-776.
- https://clinicaltrials.gov/ct2/show/NCT00486122
- https://assets.contentful.com/hadumfdtzsru/33eUP3LTUcsAgCAawSQ4Yy/25d05bd80b6f4f5ba59a7c332464835b/Atomoxetine-B4Z-US-LYCC.pdf
- https://www.accessdata.fda.gov/drugsatfda_docs/nda/2002/21-411_Strattera.cfm
- Full CSR provided by Lilly

25. Bron2014

- Bron TI, Bijlenga D, Marije Boonstra A, et al. OROS-methylphenidate efficacy on specific executive functioning deficits in adults with ADHD: A randomized, placebo-controlled cross-over study. *Eur Neuropsychopharmacol.* 2014;24(4):519-28

- http://isrctn.org/ISRCTN52392534 (ID: ISRCTN52392534)
- First author provided additional information

26. Buitelaar1996

- Additional analyses in: Buitelaar JK, Van der Gaag RJ, Swaab-Barneveld H, Kuiper M. Prediction of clinical response to methylphenidate in children with attention-deficit hyperactivity disorder. *J Am Acad Child Adolesc Psychiatry.* 1995;34(8):1025-1032.
- Buitelaar JK, van der Gaag RJ, Swaab-Barneveld H, Kuiper M. Pindolol and methylphenidate in children with attention-deficit hyperactivity disorder. Clinical efficacy and side-effects. *J Child Psychol Psychiatry.* 1996;37(5):587-595.
- Buitelaar JK, Swaab Barneveld H, Gaag RJ. Prediction of Clinical Response to Methylphenidate in Children with ADHD. *Xth World Congress of Psychiatry.1996*

27. Casas2013, EudraCT #: 2007-002111-82

- Ramos-Quiroga JA, Kooij S, Trott GE, et al. Predictors of treatment outcome with long-acting methylphenidate in attention deficit hyperactivity disorder in adults. *Eur Neuropsychopharmacol*. 2009;19:S352-S3.
- Pooled in: Buitelaar JK, Sobanski E, Stieglitz RD, Dejonckheere J, Waechter S, Schauble B. Predictors of placebo response in adults with attention-deficit/hyperactivity disorder: data from 2 randomized trials of osmotic-release oral system methylphenidate. *J Clin Psychiatry.* 2012;73(8):1097-1102.
- Casas M, Rosler M, Sandra Kooij JJ, et al. Efficacy and safety of prolonged-release OROS methylphenidate in adults with attention deficit/hyperactivity disorder: a 13-week, randomized, double-blind, placebo-controlled, fixed-dose study. *World J Biol Psychiatry.* 2013;14(4):268-281.
- Post hoc analysis in: Kooij JJ, Rosler M, Philipsen A, et al. Predictors and impact of non-adherence in adults with attention-deficit/hyperactivity disorder receiving OROS methylphenidate: results from a randomized, placebo-controlled trial. *BMC Psychiatry.* 2013;13:36.
- https://clinicaltrials.gov/ct2/show/NCT00714688
- https://www.clinicaltrialsregister.eu/ctr-search/search?query=eudract_number:2007-002111-82
- https://yoda.yale.edu/sites/default/files/nct00714688.pdf
- Additional information from study authors

28. Casat1989

- Casat CD, Pleasants DZ, Fleet JV. A double-blind trial of bupropion in children with attention-deficit disorder. *Psychopharmacol Bull.* 1987;23(1):120-122.
- Casat CD, Pleasants DZ, Schroeder DH, Parler DW. Bupropion in children with attention deficit disorder. *Psychopharmacol Bull.* 1989;25(2):198-201.

29. Childress2009, CRIT124E2305, NCT00301236

- Childress A, Muniz R, Miller J, Arnold V, HarperL, Gerstner O, et al. Fixed-dose titration study of dexmethylphenidate extended release in children withADHD: effects on teacher-rated scales. Ann Neurol2007;62(Suppl 11):S125.
- Greenbaum M, Muniz R, Brams M, Boellner S,Gerstner O, Borello M, et al. Cardiovascular safety of dexmethylphenidate extended release in children with ADHD. Ann Neurol 2007;62(Suppl 11):S146.
- Lopez F, Muniz R, Joyce JM, Franklin ER, Gerstner O, Borello M, et al. Fixed-dose titration study of dexmethylphenidate extended release in children with ADHD: effects on parent-rated scales. *Ann Neurol* 2007;62(Suppl 11):S122.
- Childress AC, Spencer T, Lopez F, et al. Efficacy and safety of dexmethylphenidate extended-release capsules administered once daily to children with attention-deficit/hyperactivity disorder. *J Child Adolesc Psychopharmacol.* 2009;19(4):351-361.
- Anonymous. Dose related safety and efficacy of dexmethylphenidate ER. The Brown University Child & Adolescent. Psychopharmacology Update 2009;11,11:7–8.
- https://clinicaltrials.gov/ct2/show/NCT00301236 (additional ID: CRIT124E2305)
- https://www.novctrd.com/CtrdWeb/displaypdf.nov?trialresultid=2418

30. Coghill2013, SPD489-325

- Coghill DR, Tobias B, Lecendreux ML, et al. Efficacy and safety of lisdexamfetamine dimesylate in children and adolescents with attention- deficit/hyperactivity disorder: a Phase 3, randomized, double-blind, multicenter, parallel-group, placebo-and-active-controlled, dose- optimization study in Europe. Proceedings of the American Academy of Child and Adolescent Psychiatry and Canadian Academy of Child and Adolescent Psychiatry Joint Annual Meeting; October 18–23, 2011; Toronto, Canada

- Gasior M, Coghill D, Soutullo C, Lyne A, Johnson M. Efficacy and safety of lisdexamfetamine dimesylate in children and adolescents with ADHD: A phase 3, randomized, double-blind, multicenter, parallel-group, placebo- and active-controlled, dose-optimized study in Europe. *Acta Neuropsychiatrica.* 2012;24:24.
- Coghill DR, Banaschewski T, Lecendreux M, et al. The first European study of lisdexamfetamine dimesylate in children and adolescents with ADHD: Overview. *Neuropsychiatr Enfance Adolesc*. 2012;1):S76.
- Coghill D, Banaschewski T, Lecendreux M, et al. Maintenance of efficacy of lisdexamfetamine dimesylate in children and adolescents with ADHD: Randomised withdrawal design. *Eur Neuropsychopharmacol.* 2012;22:S431-S432. (Withdrawal extension phase NCT00784654, SPD489-326 (EUCTR2008-000720-10-DE)
- Hodgkins P, Coghill DR, Soutullo CA, Bloomfield R, Gasio RM, JohnsonM. Poster 14: Effect of lisdexamfetamine dimesylate on functional impairment in children and adolescents with attention-deficit/hyperactivity disorder. Acta Neuropsychiatrica. Abstracts of the Scandinavian College of Neuropsychopharmacology (SCNP) 53rd Annual Meeting; 2012 April 25-27; Copenhagen, Denmark 2012;24(Suppl s1):27–8.
- Soutullo CA, Banaschewski T, Lecendreux ML, Bloomfield R, Hodgkins P, Coghill DR. Effect of lisdexamfetamine dimesylate on functional impairment in children and adolescents with attention-deficit/hyperactivity disorder. *Eur Psychiatry.* 2012;27.
- Soutullo C, Banaschewski T, Lecendreux M, et al. Effect of lisdexamfetamine dimesylate on functional impairment in children and adolescents with ADHD. *Neuropsychiatr Enfance Adolesc.* 2012;1):S76.
- Zuddas A, Banaschewski T, Lecendreux M, et al. Clinical efficacy of lisdexamfetamine dimesylate in children and adolescents with ADHD: A post-hoc analysis. *Eur Neuropsychopharmacol.* 2012;22:S431.
- Zuddas A, Banaschewski T, Lecendreux M, et al. Duration of response of lisdexamfetamine dimesylate in children and adolescents with ADHD. *Neuropsychiatr Enfance Adolesc.* 2012;1):S76.
- Zuddas A, Coghill DR, Banaschewski T, et al. Weight-related safety outcomes of lisdexamfetamine dimesylate in children and adolescents with ADHD: Post-hoc analysis from a phase 3, randomized, double-blind, multicentre, parallel-group, placebo-and active-controlled, dose-optimized study in Europe. *Neuropsychiatr Enfance Adolesc.* 2012;1):S263.
- Lecendreux ML, Dittmann RW, Gasior M, et al. Psychiatric-related safety outcomes of lisdexamfetamine dimesylate in children and adolescents with attention-deficit/hyperactivity disorder: A phase 3, randomized, double-blind, multi centre, parallel-group, placebo-and active-controlled, dose-optimized study in Europe. *Neuropsychiatr Enfance Adolesc.* 2012;1):S263.
- Banaschewski T, Johnson M, Zuddas A, et al. Health-related quality of life outcomes in children and adolescents with ADHD treated with lisdexamfetamine dimesylate. *Neuropsychiatr Enfance Adolesc.* 2012;1):S77.
- Setyawan J, Banaschewski T, Hodgkins P, et al. Health utility scores in children and adolescents with attention-deficit/hyperactivity disorder: Response to stimulant treatment. *Value Health.* 2012;15 (7):A284.
- Coghill D, Banaschewski T, Lecendreux M, Soutullo C, Johnson M, Zuddas A, et al. Post hoc comparison of the efficacy of lisdexamfetamine dimesylate and osmotic-release oral system methylphenidate in children and adolescents with ADHD. *Eur Psychiatry*. Abstracts of the 21th European Congress of Psychiatry 2013;28(Suppl 1)
- Coghill D, Banaschewski T, Lecendreux M, et al. Impact of previous ADHD medication on the efficacy of lisdexamfetamine dimesylate in the treatment of ADHD: Post hoc analyses. *Eur Neuropsychopharmacol.* 2013;23:S599-S600.
- Coghill D, Banaschewski T, Lecendreux M, et al. European, randomized, phase 3 study of lisdexamfetamine dimesylate in children and adolescents with attention-deficit/hyperactivity disorder. *Eur Neuropsychopharmacol.* 2013;23(10):1208-1218.
- Banaschewski T, Soutullo C, Lecendreux M, et al. Health-related quality of life and functional outcomes from a randomized, controlled study of lisdexamfetamine dimesylate in children and adolescents with attention deficit hyperactivity disorder. *CNS Drugs.* 2013;27(10):829-840.
- Hodgkins P, Setyawan J, Banaschewski T, et al. Health utility scores in children and adolescents with attention-deficit/hyperactivity disorder: Response to stimulant treatment. *Eur Child Adolesc Psychiatry.* 2013;1):S127.
- Lecendreux M, Banaschewski T, Soutullo C, et al. Efficacy of lisdexamfetamine dimesylate in children and adolescents with attention-deficit/hyperactivity disorder: Effect of age, sex and baseline disease severity. *Eur Psychiatry.* 2013;28.
- Coghill D, Banaschewski T, Lecendreux M, et al. The first European studies of lisdexamfetamine dimesylate in children and adolescents with attention-deficit/hyperactivity disorder. *Eur Child Adolesc Psychiatry*. 2013;1):S125.
- Coghill D, Banaschewski T, Zuddas A, et al. Health-related quality of life outcomes in a long-term study of lisdexamfetamine dimesylate in children and adolescents with ADHD. *Eur Neuropsychopharmacol.* 2013;23:S598-S9. (extension phase: NCT00784654, SPD489-326 (EUCTR2008-000720-10-DE)
- Banaschewski T, Soutullo C, Lecendreux M, et al. The child health and illness profile as a measure of health-related quality of life in stimulant-treated children and adolescents with ADHD. *Eur Child Adolesc Psychiatry.* 2013;1):S125-S126. (refers to both SPD489-325 and SPD489-326)
- Lecendreux M, Zuddas A, Banaschewski T, et al. Weight-related safety outcomes of lisdexamfetamine dimesylate

in children and adolescents with attention-deficit/hyperactivity disorder. *Eur Child Adolesc Psychiatry.* 2013;1:S223.
- Sorooshian S, Banaschewski T, Lecendreux M, et al. Post hoc comparison of the efficacy of lisdexamfetamine dimesylate and osmotic-release oral system methylphenidate in children and adolescents with attention deficit/hyperactivity disorder. *Aust N Z J Psychiatry.* 2013;47:89-90.
- Sorooshian S, Markarian M, Ermer J, Civil R, Gasior M, Coghill D. Pharmacokinetic and clinical profiles of the longacting prodrug stimulant lisdexamfetamine dimesylate: Results from two studies in children and adolescents with attention deficit/hyperactivity disorder. *Aust N Z J Psychiatry.* 2013;47:89.
- Soutullo C, Banaschewski T, Lecendreux M, et al. A post hoc comparison of the effects of lisdexamfetamine dimesylate and osmotic-release oral system methylphenidate on symptoms of attention-deficit hyperactivity disorder in children and adolescents. *CNS Drugs.* 2013;27(9):743-751.
- Soutullo C, Banaschewki T, Lecendreux M, et al. Assessing ADHD stimulant treatment efficacy using the weiss functional impairment rating scale: Strengths and weaknesses. *Eur Child Adolesc Psychiatry.* 2013;1):S126.
- Coghill DR, Banaschewski T, Lecendreux M, et al. Efficacy of lisdexamfetamine dimesylate throughout the day in children and adolescents with attention-deficit/hyperactivity disorder: results from a randomized, controlled trial. *Eur Child Adolesc Psychiatry.* 2014;23(2):61-68.
- Fridman M, Erder MH. Interpreting the Results of a Retrospective Comparison of Test and Reference Treatments in a Randomized Clinical Trial Setting. *Clin Drug Investig.* 2014;35(2):133-140.
- Coghill DR, Banaschewski T, Lecendreux M, et al. Post hoc analyses of the impact of previous medication on the efficacy of lisdexamfetamine dimesylate in the treatment of attention-deficit/hyperactivity disorder in a randomized, controlled trial. *Neuropsychiatr Dis Treat.* 2014;10:2039-2047.
- Doddamani L, Hodgkins P, Adeyi B, Squires LA, Civil R, Coghill DR. Functional impairment in children and adolescents with attention deficit hyperactivity disorder: Results from short-and long-term studies of lisdexamfetamine dimesylate. *Aust N Z J Psychiatry.* May 2014;48:115.
- Banaschewski T, Johnson M, Lecendreux M, et al. Health-related quality of life and functional outcomes from a randomized-withdrawal study of long-term lisdexamfetamine dimesylate treatment in children and adolescents with attention-deficit/hyperactivity disorder. *CNS Drugs.* 2014;28(12):1191-1203. (withdrawal extension phase: NCT00784654, SPD489-326 (EUCTR2008-000720-10-DE)
- Coghill DR, Banaschewski T, Lecendreux M, Johnson M, Zuddas A, Anderson CS, Civil R, Dauphin M, Higgins N, Lyne A, Gasior M, Squires LA. Maintenance of efficacy of lisdexamfetamine dimesylate in children and adolescents with attention-deficit/hyperactivity disorder: randomized-withdrawal study design. *J Am Acad Child Adolesc Psychiatry*. 2014;53(6):647-657.e1 (Withdrawal extension: NCT00784654, SPD489-326 (EUCTR2008-000720-10-DE)
- Doddamani L, Sikirica V, Caballero B, Adeyi B, Naser N, Coghill D. Post HOC comparison of lisdexamfetamine dimesylate and osmotic-release oral system methylphenidate: Symptoms, health-related quality of life and functional impairment in children and adolescents with attention deficit/hyperactivity disorder. *Aust N Z J Psychiatry.* 2015;49:108.
- Included in: Coghill DR, Nagy P, Frick G, Adeyi B, Newcorn J. Relative efficacy of lisdexamfetamine dimesylate and osmotic controlled-release methylphenidate in attention-deficit/ hyperactivity disorder patients. *Eur Neuropsychopharmacol*. 2015;25:S647-S8
- Grebla R, Setyawan J, Yang H, et al. Development of a risk score to guide individualized treatment selection in attention-deficit/hyperactivity disorder. *Eur Child Adolesc Psychiatry.* 2015;1):S244.
- Setyawan J, Yang H, Cheng D, et al. Developing a Risk Score to Guide Individualized Treatment Selection in Attention Deficit/Hyperactivity Disorder. *Value Health.* 2015;18(6):824-831.
- Zuddas A, Banaschewski T, Nagy P, et al. Long-term safety and efficacy of lisdexamfetamine dimesylate in children and adolescents with attention-deficit/hyperactivity disorder. *Eur Neuropsychopharmacol.* 2015;25:S648. (Extension phase: NCT00784654, SPD489-326 (EUCTR2008-000720-10-DE)
- https://clinicaltrials.gov/ct2/show/NCT00763971
- Additional Information provided by Shire from full CSR

31. Connor2000
- Connor DF, Barkley RA, Davis HT. A pilot study of methylphenidate, clonidine, or the combination in ADHD comorbid with aggressive oppositional defiant or conduct disorder. *Clin Pediatr (Phila).* 2000;39(1):15-25.

32. Connor2010 , SPD503-307, NCT00367835
- Sallee F, Spencer T, Connor D, Lopez F, Lyne A, Tremblay G. Effects of Guanfacine Extended Release on Secondary Measures in Children With Attention-Deficit/Hyperactivity Disorder and Oppositional Symptoms. *Biol Psychiatry.* 2009;65(8, Suppl. S):149S.
- Connor DF, Findling RL, Kollins SH, et al. Effects of guanfacine extended release on oppositional symptoms in

children aged 612 years with attention-deficit hyperactivity disorder and oppositional symptoms: A randomized, double-blind, placebo-controlled trial. *CNS Drugs.* 2010;24(9):755-768.
- Connor D, Kollins S, Findling R, et al. Effects of Guanfacine Extended Release in Children Aged 6 to 12 Years with Oppositional Symptoms and a Diagnosis of Attention-Deficit/Hyperactivity Disorder. *Biol Psychiatry.* 2010;67:217S.
- Connor DF, Sallee FR, Lyne A, Stat C, Youcha S. Changes in parental stress with guanfacine extended release in children with attention-deficit/ hyperactivity disorder and oppositional symptoms. *163rd Annual Meeting of the American Psychiatric Association; 2010 May 22-26; New Orleans, LA 2010.*
- https://clinicaltrials.gov/ct2/show/NCT00367835 (other ID:SPD503-307**)**
- Additional data provided by Shire

33. Cook1993
- Cook JR. The Effects of Methylphenidate on Resource Allocation in the Mental Processing of ADD Children [PhD thesis]. University of North Dakota, 1989.
- Cook JR, Mausbach T, Burd L, et al. A preliminary study of the relationship between central auditory processing disorder and attention deficit disorder. *J Psychiatry Neurosci.* 1993;18(3):130-137.
- Additional information provided by: Dr. Storebø (from the following Cochrane meta-analysis: Storebø et al. *Cochrane Database Syst Rev*. 2015)

34. CRIT124DUS02
- https://www.novctrd.com/CtrdWeb/displaypdf.nov?trialresultid=1624
- Additional information from Novartis

35. Dell'Agnello2009
- Dell'Agnello G, Maschietto D, Bravaccio C, et al. Atomoxetine hydrochloride in the treatment of children and adolescents with attention-deficit/hyperactivity disorder and comorbid oppositional defiant disorder: A placebo-controlled Italian study. *Eur Neuropsychopharmacol.* 2009;19(11):822-834.
- Dell'Agnello G, Maschietto D, Pascotto A, et al. Efficacy of atomoxetine in pediatric patients with attentiondeficit/ hyperactivity disorder and comorbid oppositional defiant disorder. *World Psychiatry*. 2009:252.
- Pooled in: Montoya A, Quail D, Anand E, Cardo E, Alda JA, Escobar R. Prognostic factors of improvement in health-related quality of life in atomoxetine-treated children and adolescents with attention-deficit/hyperactivity disorder, based on a pooled analysis. *Atten Defic Hyperact Disord.* 2014;6(1):25-34.
- Previous reference related to: Montoya A, Quail D, Anand E, Cardo E, Alda JA, Escobar R. Prognostic factors of improvement in health related quality of life in children and adolescents with attention deficit/hyperactivity disorder, after atomoxetine treatment. *Eur Psychiatr.* 2012;27).
- https://clinicaltrials.gov/ct2/show/NCT00192023

36. Dittmann2011
- Dittmann R.W, Schacht A, Helsberg K, Schneider-Fresenius C, Lehmann M, Lehmkuhl G, Wehmeier PM. Randomisierte, plazebokontrollierte Doppelblindstudie zur Wir- ksamkeit und Vertra¨glichkeit von Atomoxetin (ATX) bei Kindern und Jugendlichen mit ADHS und oppositionellem Trotzverhalten bzw. Sto¨rung des Sozialverhaltens. In Frank Schneider und Michael Gro¨zinger (Eds.), Psychiatric disorders through lifespan. abstract book of the DGPPN congress 2009, 25–28 November 2009 (p. 230). Germany: ICC Berlin. doi:10.3287/dgppn.2009.1
- Dittmann RW, Schacht A, Helsberg K, et al. Atomoxetine versus placebo in children and adolescents with attention-deficit/hyperactivity disorder and comorbid oppositional defiant disorder: a double-blind, randomized, multicenter trial in Germany. *J Child Adolesc Psychopharmacol.* 2011;21(2):97-110.
- Wehmeier PM, Schacht A, Dittmann RW, et al. Effect of atomoxetine on quality of life and family burden: results from a randomized, placebo-controlled, double-blind study in children and adolescents with ADHD and comorbid oppositional defiant or conduct disorder. *Qual Life Res.* 2011;20(5):691-702.
- https://www.clinicaltrialsregister.eu/ctr-search/search?query=eudract_number:2006-001471-37 (additional ID:B4Z-SB-LYDW**)**
- https://clinicaltrials.gov/ct2/show/NCT00406354
- Full CSR provided by Lilly

37. Döpfner2003
- Lehmkuhl G. Placebo-controlled, double-blind multicenter trial on the efficacy of sustained-release methylphenidate in children suffering from attention deficit hyperactivity disorder (ADHD), Phase III. Integrated Final Report.

Institut für medizinische Informatik, *Biometrie und Epidemiologie, Universitätsklinikum Essen*, Trial no 6520-9979-02, 1-9 2002

- Dopfner M, Banaschewski T, Schmidt J, et al. Langzeitwirksames Methylphenidat bei Kinderen mit Aufmerksamkeitsdefizit-Hyperaktivitatsstorungen-eine multizentrische Studie [Long-acting methylphenidate preparation in children with ADHD-a multicenter study]. Nervenheilkunde. 2003(2):85-92.
- Sinzig JK, Döpfner M, Plück J, Banaschewski T, Stephani U, Lehmkuhl G, et al. Does a morning dose of methylphenidate retard reduce hyperkinetic symptoms in the afternoon? [Lassen sich hyperkinetische auffälligkeiten am nachmittag durch eine morgengabe von methylphenidat retard vermindern?]. *Z Kinder Jugendpsychiatr Psychother*. 2004;32 (4):225–33.
- Sinzig J, Döpfner M, Lehmkuhl G, Uebel H, Schmeck K, Poustka F, et al. Long-acting methylphenidate has an effect on aggressive behavior in children with attention-deficit/ hyperactivity disorder. *J Child Adolesc Psychopharmacol.* 2007;17(4):421–32.

38. Durell2013, B4Z-US-LYDZ, NCT00510276

- This study was presented in poster form at the Society of Biological Psychiatry 65th Annual Convention & Scientific Program, May 2010
- Altin M, Alev L, Durell TM, et al. Atomoxetine for the treatment of ADHD in young adults with an assessment of associated functional outcomes. *Klinik Psikofarmakoloji Bulteni.* 2011;21:S135-S136.
- Durell TM, Adler LA, Williams DW, et al. Atomoxetine treatment of attention-deficit/hyperactivity disorder in young adults with assessment of functional outcomes: a randomized, double-blind, placebo-controlled clinical trial. *J Clin Psychopharmacol.* 2013;33(1):45-54.
- Adler LA, Clemow DB, Williams DW, Durell TM. Atomoxetine effects on executive function as measured by the BRIEF--a in young adults with ADHD: a randomized, double-blind, placebo-controlled study. *PLoS ONE [Electronic Resource].* 2014;9(8):e104175.
- Addendum neurophysiological study (not pertinent for the present meta-analysis): Leuchter AF, McGough JJ, Korb AS, et al. Neurophysiologic predictors of response to atomoxetine in young adults with attention deficit hyperactivity disorder: a pilot project. *J Psychiatr Res.* 2014;54:11-18.
- https://clinicaltrials.gov/ct2/show/NCT00510276 (additional ID: B4Z-US-LYDZ)
- Lilly provided full CSR

39. Efron1997

- Efron D, Jarman F, Barker M. Methylphenidate versus dexamphetamine in children with attention deficit hyperactivity disorder: A double-blind, crossover trial. *Pediatrics.* 1997;100(6):E6.
- Efron D, Jarman F, Barker M. Side effects of methylphenidate and dexamphetamine in children with attention deficit hyperactivity disorder: a double-blind, crossover trial. *Pediatrics.* 1997;100(4):662-666.
- Efron D, Jarman FC, Barker MJ. Child and parent perceptions of stimulant medication treatment in attention deficit hyperactivity disorder. *J Paediatr Child Health.* 1998;34(3):288-292.
- Efron DJ. Methylphenidate vs dexamphetamine in children with attention deficit hyperactivity disorder: a double-blind cross-over trial. *J Paediatr Child Health.* 1997:A21.
- Follow-up in: Efron D, Jarman FC, Barker MJ. Medium-term outcomes are comparable with short-term outcomes in children with attention deficit hyperactivity disorder treated with stimulant medication. *J Paediatr Child Health.* 2000;36(5):457-461.
- Additional information from first author

40. Findling2008 , NCT00444574

- Swanson JM. Transdermal methylphenidate more effective than placebo for treating ADHD. *Evidence-based mental health.* 2008;11(4):118.
- Findling RL et al. A randomized, double-blind, placebo-controlled, parallel-group study of methylphenidate transdermal system in pediatric patients with attention-deficit/hyperactivity disorder. *J Clin Psychiatry.* 2008;69(1):149-59.
- Erratum in: Findling RL, Bukstein OG, Melmed RD, Lopez FA, Sallee FR, Arnold LE, et al. “A randomized, double-blind, placebo-controlled, parallel-group study of methylphenidate transdermal system in pediatric patients with attention deficit/hyperactivity disorder”: correction. *J Clin Psychiatry.* 2008;69(2):329.
- Faraone SV, Glatt SJ, Bukstein OG, Lopez FA, Arnold LE, Findling RL. Effects of once-daily oral and transdermal methylphenidate on sleep behavior of children with ADHD. *J Atten Disord.* 2009;12(4):308-315.
- Extension study: Findling RL, Katic A, Rubin R, Moon E, Civil R, Li Y. A 6-month, open-label, extension study of the tolerability and effectiveness of the methylphenidate transdermal system in adolescents diagnosed with attention-deficit/hyperactivity disorder. *J Child Adolesc Psychopharmacol.* 2010;20(5):365-375. (NCT00501293)
- https://clinicaltrials.gov/ct2/show/NCT00444574

41. Findling2011, SPD 489-305, NCT00735371

- Childress A, Cutler A, Gasior M, et al. Double-Blind, Placebo-Controlled Efficacy and Safety Study of Lisdexamfetamine Dimesylate in Adolescents with Attention Deficit Hyperactivity Disorder (ADHD). *J Child Adolesc Psychopharmacol.* 2010;20(6):533-533.
- Gasior M, Findling R, Childress A, Cutler A, Hamdani M, Ferreira-Cornwell MC. Double-blind, placebo-controlled efficacy and safety study of lisdexamfetamine dimesylate in adolescents with attention-deficit/ hyperactivity disorder. Neuropsychopharmacology. 2010;35:S103-S104.
- Findling RL, Childress AC, Cutler AJ, et al. Efficacy and safety of lisdexamfetamine dimesylate in adolescents with attention-deficit/hyperactivity disorder. *J Am Acad Child Adolesc Psychiatry.* 2011;50(4):395-405.
- Findling R, Childress A, Cutler A, et al. Long-term safety of lisdexamfetamine dimesylate (LDX) in adolescents with attention-deficit/hyperactivity disorder. *Eur Child Adolesc Psychiatry.* 2011;20:S117-S118.
- Included in: Coghill D, Sorooshian S, Caballero B. Safety outcomes from the clinical development programme for lisdexamfetamine dimesylate: A prodrug stimulant for the treatment of attention-deficit/hyperactivity disorder. *Eur Child Adolesc Psychiatry*. 2013;1):S224.
- Open phase in: Findling RL, Cutler AJ, Saylor K, et al. A long-term open-label safety and effectiveness trial of lisdexamfetamine dimesylate in adolescents with attention-deficit/hyperactivity disorder. *J Child Adolesc Psychopharmacol.* 2013;23(1):11-21.
- Post hoc analysis of long term phase: Childress AC, Cutler AJ, Saylor K, et al. Participant-perceived quality of life in a long-term, open-label trial of lisdexamfetamine dimesylate in adolescents with attention-deficit/hyperactivity disorder. *J Child Adolesc Psychopharmacol.* 2014;24(4):210-217.
- https://clinicaltrials.gov/ct2/show/NCT00735371 (additional ID: SPD489-305)
- https://www.accessdata.fda.gov/drugsatfda_docs/nda/2007/021977s000TOC.cfm
- Additional Information provided by Shire from full CSR

42. Frick2017, SPD465-303, NCT00152022

- Spencer TJ, Adler LA, Weisler RH, Youcha SH, Anderson CS, Silverberg A. Efficacy and safety of a novel enhanced extended-release amphetamine formulation (triple-bead mixed amphetamine salts, SPD465) in adults with ADHD. Presented at: 160th Annual Meeting of the American Psychiatric Association; May 19–24, 2007; San Diego, CA.
- Pooled in: Brown TE, Landgraf JM. Improvements in executive function correlate with enhanced performance and functioning and health-related quality of life: evidence from 2 large, double-blind, randomized, placebo-controlled trials in ADHD. *Postgrad Med.* 2010;122(5):42-51.
- Frick G, Yan B, Adler LA. Triple-bead mixed amphetamine salts (SHP465) in adults with ADHD: results of a phase 3, doubel-blind, randomized, forced-dose trial. *J Att Dis*. 2017 Apr 1:1087054717696771. doi: 10.1177/108705471769677
- https://clinicaltrials.gov/ct2/show/NCT00152022 (additional ID: SPD463-303)

43. Gau2007 , B4Z-TW-S010, NCT00485459

- Gau SS, Huang YS, Soong WT, et al. A randomized, double-blind, placebo-controlled clinical trial on once-daily atomoxetine in Taiwanese children and adolescents with attention-deficit/hyperactivity disorder. *J Child Adolesc Psychopharmacol.* 2007;17(4):447-460.
- https://clinicaltrials.gov/ct2/show/NCT00485459
- https://assets.contentful.com/hadumfdtzsru/5VQbWaMDAswCWsK2OCki2c/bf817d7446e489369c57231d112c4c80/Atomoxetine-B4Z-TW-S010.pdf
- https://www.accessdata.fda.gov/drugsatfda_docs/nda/2002/21-411_Strattera.cfm
- Full protocol provided by Lilly

44. Geller2007, B4Z-US-LYBP

- Geller D, Donnelly C, Lopez F, et al. Atomoxetine treatment for pediatric patients with attention-deficit/hyperactivity disorder with comorbid anxiety disorder. *J Am Acad Child Adolesc Psychiatry.* 2007;46(9):1119-1127.
- Pooled in: Newcorn JH, Sutton VK, Zhang S, et al. Characteristics of placebo responders in pediatric clinical trials of attention-deficit/hyperactivity disorder. *J Am Acad Child Adolesc Psychiatry.* 2009;48(12):1165-1172.
- https://assets.contentful.com/hadumfdtzsru/6DvfHWkAakuaUkQgM6UGwS/b3c20317fc84b267cfb138cc326e8c21/Atomoxetine-B4Z-US-LYBP_a_.pdf
- https://www.accessdata.fda.gov/drugsatfda_docs/nda/2002/21-411_Strattera.cfm
- Full CSR provided by Lilly

45. Ginsberg2012, EUCTR2006-002553-80-SE

- Ginsberg Y, Lindefors N. Methylphenidate treatment of adult male prison inmates with attention-deficit hyperactivity disorder: randomised double-blind placebo-controlled trial with open-label extension. *Br J Psychiatry.* 2012;200(1):68-73.
- Long term non controlled phase in: Ginsberg Y, Hirvikoski T, Grann M, Lindefors N. Long-term functional outcome in adult prison inmates with ADHD receiving OROS-methylphenidate. *Eur Arch Psychiatry Clin Neurosci.* Dec 2012;262(8):705-724.
- Grann M, Ginsberg Y, Hirvikoski T, Lindefors N. Methylphenidate treatment of adult prison inmates with ADHD: A randomised double-blind placebo-controlled trial with open-label extension. *Acta Neuropsychiatr*. 2013;1):13.
- Long term non controlled phase in: Ginsberg Y, Hirvikoski T, Grann M, Lindefors N. Osmotic-release oral system methylphenidate (OROS-MPH) treatment of adult prison inmates with ADHD: A randomised controlled trial with open-label extension. *Eur Psychiatry.* 2013;28.
- Ginsberg Y. Pharmacological treatment of offenders with ADHD. *ADHD Attention Deficit and Hyperactivity Disorders.* 2015;7:S4.
- Follow-up in: Ginsberg Y, Langstrom N, Larsson H, Lindefors N. Long-term treatment outcome in adult male prisoners with attention- deficit/hyperactivity disorder: Three-year naturalistic follow-up of a 52-week methylphenidate trial. *J Clin Psychopharmacol.* 2015;35(5):535-543.
- https://clinicaltrials.gov/ct2/show/NCT00482313
- https://www.clinicaltrialsregister.eu/ctr-search/search?query=eudract_number:2006-002553-80
- Additional information from study author

46. Goodman2016, NCT00937040

- Goodman DW, Starr HL, Ma YW, Rostain AL, Ascher S, Armstrong RB. Randomized, 6-Week, Placebo-Controlled Study of Treatment for Adult Attention-Deficit/Hyperactivity Disorder: Individualized Dosing of Osmotic-Release Oral System (OROS) Methylphenidate With a Goal of Symptom Remission. *J Clin Psychiatry.* 2017;78(1):105-114
- https://clinicaltrials.gov/ct2/show/NCT00937040
- Additional information form Janssen

47. Goto2017, B4Z-JE-LYEE, NCT00962104

- Hirata Y, Goto T, Takita Y, et al. Efficacy and safety of atomoxetine in Asian adults with ADHD: A multinational 10-week randomized, double-blind placebo-controlled Asian study. *Int J Neuropsychopharmacol.* 2012;15:221-222.
- Goto T, Hirata Y, Takita Y, et al. Efficacy and Safety of Atomoxetine Hydrochloride in Asian Adults With ADHD. *J Atten Disord. 2017;212): 100-109.*(Former reference: Goto T, Hirata Y, Takita Y, et al. Efficacy and Safety of Atomoxetine Hydrochloride in Asian Adults With ADHD: A Multinational 10-Week Randomized Double-Blind Placebo-Controlled Asian Study. *J Atten Disord.* 2013.)
- Post hoc analysis in Korean sample in: Lee SI, Song DH, Shin DW, et al. Efficacy and safety of atomoxetine hydrochloride in Korean adults with attention-deficit hyperactivity disorder. *Asia Pac Psychiat.* 2014;6(4): 386-396.
- Follow-up, open label study in: Hirata Y, Goto T, Takita Y, et al. Long-term safety and tolerability of atomoxetine in Japanese adults with attention deficit hyperactivity disorder. *Asia Pac Psychiatry*. 2014;6(3):292-301
- Conference proceeding related to previous reference: Hirata Y, Goto T, Takita Y, et al. Long-term safety and efficacy of atomoxetine in adult ADHD Japanese patients. *Eur Psychiatry*. 2013;28.
- https://clinicaltrials.gov/ct2/show/NCT00962104 (other ID:B4Z-JE-LYEE)
- Full CSR provided by Lilly

48. Greenhill2002

- Greenhill LL, Findling RL, Swanson JM. A double-blind, placebo-controlled study of modified-release methylphenidate in children with attention-deficit/hyperactivity disorder. *Pediatrics.* 2002;109(3):E39.

49. Greenhill2006a, Study 309 Cephalon

- Greenhill, L.L., Biederman, J., Boellner, S.W., Rugino, T.A., Sangal, R.B., Swanson, J.M., 2005. Modafinil film coated tablets significantly improve symptoms on ADHD Rating Scale-IV School and Home and overall clinical condition in children and adolescents with attention-deficit/hyperactivity disorder. J. Child Adolesc. Psychopharmacol. 15 (6), 849e850. NCDEU 45th Annual Meeting Boca Raton, Florida, USA Session I-88
- Greenhill LL, Wigal SB, Kratochvil C, Boellner S. ADHD symptoms and behavior improved with modafinil pediatric formulation. *158th Annual Meeting of the American Psychiatric Association; 2005 May 21-26; Atlanta, GA 2005.*
- Greenhill LL, Biederman J, Boellner SW, et al. A randomized, double-blind, placebo-controlled study of modafinil film-coated tablets in children and adolescents with attention-deficit/hyperactivity disorder. *J Am Acad Child*

Adolesc Psychiatry. 2006;45(5):503-511.
- Pooled in: Wigal SB, Biederman J, Swanson JM, Yang R, Greenhill LL. Efficacy and safety of modafinil film-coated tablets in children and adolescents with or without prior stimulant treatment for attention-deficit/hyperactivity disorder: pooled analysis of 3 randomized, double-blind, placebo-controlled studies. *Prim Care Companion J Clin Psychiatry.* 2006;8(6):352-360.
- Pooled in: Biederman J, Pliszka SR. Modafinil improves symptoms of attention-deficit/hyperactivity disorder across subtypes in children and adolescents. *J Pediatr.* 2008;152(3):394-399.
- https://www.accessdata.fda.gov/drugsatfda_docs/nda/98/020717A_Provigil.cfm
- Modafinil (CEP-1538) Tablets, Supplemental NDA 20-717/S-019, ADHD Indication Briefing Document for Psychopharmacologic Drugs Advisory Committee Meeting (Document provided by Dr Eric Konofal)

50. Greenhill2006b , CRIT124E2301
- Greenhill LL, Muniz R, Ball RR, Levine A, Pestreich L, Jiang H. Efficacy and safety of dexmethylphenidate extended-release capsules in children with attention-deficit/hyperactivity disorder. *J Am Acad Child Adolesc Psychiatry.* 2006;45(7):817-823.
- https://www.novctrd.com/CtrdWeb/displaypdf.nov?trialresultid=1622 (ID: CRIT124E2301)
- https://www.accessdata.fda.gov/drugsatfda_docs/nda/2005/021802s000TOC.cfm

51. Grizenko2012
- Ben AL, Grizenko N, Mbekou V, Lageix P, Baron C, Schwartz G, et al. Dopamine transporter gene and behavioral response to methylphenidate (MPH) in children with attention deficit hyperactivity disorder (ADHD). *Am J Med Genet A*. Abstracts of the Xth World Congress of Psychiatric Genetics; 2002; Brussels, Belgium. 2002; Vol. 114 (7):724–5.
- Grizenko N, Lachance M, Collard V, et al. Sensitivity of tests to assess improvement in ADHD symptomatology. *Can J Psychiatry.* 2004;13(2):36-39.
- Schwartz G, Amor LB, Grizenko N, Lageix P, Baron C, Boivin DB, et al. Actigraphic monitoring during sleep of children with ADHD on methylphenidate and placebo. *J Am Acad Child Adolesc Psychiatry.* 2004;43(10):1276–82.
- Gruber R, Grizenko N, Schwartz G, Benamor L, Terstepanian M, Joober R. The associations between sleep and attention in children with attention deficit hyperactivity disorder while on placebo and while on methylpheniclate. *Sleep (Rochester).* 2005;28(Suppl. S):A93-A94.
- Overlap with subjects from: Grizenko N, Bhat M, Schwartz G, Ter-Stepanian M, Joober R. Efficacy of methylphenidate in children with attention-deficit hyperactivity disorder and learning disabilities: a randomized crossover trial. *J Psychiatry Neurosci.* 2006;31(1):46-51.
- Grizenko N, Kovacina B, Amor LB, Schwartz G, Ter-Stepanian M, Joober R. Relationship between response to methylphenidate treatment in children with ADHD and psychopathology in their families. *J Am Acad Child Adolesc Psychiatry.* 2006;45(1):47-53.
- Gruber R, Grizenko N, Schwartz G, Ben Amor L, Gauthier J, De Guzman R, et al. Sleep and COMT polymorphismin ADHD children: preliminary actigraphic data. *J Am Acad Child Adolesc Psychiatry.* 2006;45(8):982–9.
- Gruber R, Grizenko N, Schwartz G, Bellingham J, Guzman R, Joober R. Performance on the continuous performance test in children with ADHD is associated with sleep efficiency. *Sleep.* 2007;30(8):1003-1009.
- Joober R, Grizenko N, Sengupta S, et al. Dopamine transporter 3'-UTR VNTR genotype and ADHD: a pharmaco-behavioural genetic study with methylphenidate. Neuropsychopharmacology. 2007;32(6):1370-1376.
- Harvey WJ, Reid G, Grizenko N, Mbekou V, Ter-Stepanian M, Joober R. Fundamental movement skills and children with attention-deficit hyperactivity disorder: peer comparisons and stimulant effects. *J Abnorm Child Psychol.* 2007;35(5):871-882.
- Thesis related to the previous reference: Harvey WJ. Fundamental movement skills and associated physical activity experiences of children with ADHD. *Dissertation Abstracts International Section A: Humanities and Social Sciences.* 2007;68(3-A):927.
- Grizenko N, Shayan YR, Polotskaia A, Ter-Stepanian M, Joober R. Relation of maternal stress during pregnancy to symptom severity and response to treatment in children with ADHD. *J Psychiatry Neurosci.* 2008;33(1):10-16.
- Sengupta S, Grizenko N, Schmitz N, Schwartz G, Bellingham J, Polotskaia A, et al. COMT Val108/158Met polymorphism and the modulation of task-oriented behavior in children with ADHD. Neuropsychopharmacology. 2008;33(13):3069–77.
- Data from this cohort pooled in: Gruber R, Joober R, Grizenko N, Leventhal BL, Cook EH, Jr., Stein MA. Dopamine transporter genotype and stimulant side effect factors in youth diagnosed with attention-deficit/hyperactivity disorder. *J Child Adolesc Psychopharmacol.* 2009;19(3):233-239.
- Grizenko N, Paci M, Joober R. Is the inattentive subtype of ADHD different from the combined/hyperactive

subtype? *J Atten Disord.* 2010;13(6):649-657.
- Ter-Stepanian M, Grizenko N, Zappitelli M, Joober R. Clinical response to methylphenidate in children diagnosed with attention-deficit hyperactivity disorder and comorbid psychiatric disorders. *Can J Psychiatry.* 2010;55(5):305-312.
- Thakur GA, Grizenko N, Sengupta SM, Schmitz N, Joober R. The 5-HTTLPR polymorphism of the serotonin transporter gene and short term behavioral response to methylphenidate in children with ADHD. *BMC Psychiatry.* 2010;10:50.
- Lee J, Grizenko N, Bhat V, Sengupta S, Polotskaia A, Joober R. Relation between therapeutic response and side effects induced by methylphenidate as observed by parents and teachers of children with ADHD. *BMC Psychiatry.* 2011;11:70.
- Grizenko N, Zhang DDQ, Polotskaia A, Joober R. Effcacy of methylphenidate in ADHD children across the normal and the gifted intellectual spectrum. *J Can Acad Child Adolesc Psychiatry.* 2012;21(4):282-288.
- Thakur GA, Sengupta SM, Grizenko N, Choudhry Z, Joober R. Comprehensive phenotype/genotype analyses of the norepinephrine transporter gene (SLC6A2) in ADHD: relation to maternal smoking during pregnancy. *PLoS One.* 2012;7(11):e49616.
- Labbe A, Liu A, Atherton J, et al. Refining psychiatric phenotypes for response to treatment: contribution of LPHN3 in ADHD. *Am J Med Genet B Neuropsychiatr Genet.* 2012;159B(7):776-785.
- Grizenko N, Cai E, Jolicoeur C, Ter-Stepanian M, Joober R. Effects of methylphenidate on acute math performance in children with attention-deficit hyperactivity disorder. *Can J Psychiatry.* 2013;58(11):632-639.
- Grizenko N, Rodrigues Pereira RM, Joober R. Sensitivity of scales to evaluate change in symptomatology with psychostimulants in different ADHD subtypes. *J Can Acad Child Adolesc Psychiatry.* 2013;22(2):153-158.
- Fortier ME, Sengupta SM, Grizenko N, Choudhry Z, Thakur G, Joober R. Genetic evidence for the association of the hypothalamic-pituitary-adrenal (HPA) axis with ADHD and methylphenidate treatment response. *Neuromolecular medicine.* 2013;15(1):122-132.
- https://clinicaltrials.gov/ct2/show/NCT00483106

Note: Dr. Grizenko provided us with pre cross-over data for the largest sample of the cohort

52. Harfterkamp2012, NCT00380692
- Harfterkamp M, van de Loo-Neus G, Minderaa RB, et al. A randomized double-blind study of atomoxetine versus placebo for attention-deficit/hyperactivity disorder symptoms in children with autism spectrum disorder. *J Am Acad Child Adolesc Psychiatry.* 2012;51(7):733-741.
- van der Meer JM, Harfterkamp M, van de Loo-Neus G, et al. A randomized, double-blind comparison of atomoxetine and placebo on response inhibition and interference control in children and adolescents with autism spectrum disorder and comorbid attention-deficit/hyperactivity disorder symptoms. *J Clin Psychopharmacol.* 2013;33(6):824-827.
- Follow-up in: Harfterkamp M, Buitelaar JK, Minderaa RB, van de Loo-Neus G, van der Gaag RJ, Hoekstra PJ. Long-term treatment with atomoxetine for attention-deficit/hyperactivity disorder symptoms in children and adolescents with autism spectrum disorder: an open-label extension study. *J Child Adolesc Psychopharmacol.* 2013;23(3):194-199.
- Harfterkamp M, Van Der Meer J. A Randomized double-blind study of atomoxetine vs. placebo followed by an open label extension period of treatment with atomoxetine for ADHD symptoms in children with ASD. *Eur Child Adolesc Psychiatry.* 2013;1):S216-S217.
- Harfterkamp M, Buitelaar JK, Minderaa RB, van de Loo-Neus G, van der Gaag RJ, Hoekstra PJ. Atomoxetine in autism spectrum disorder: no effects on social functioning; some beneficial effects on stereotyped behaviors, inappropriate speech, and fear of change. *J Child Adolesc Psychopharmacol.* 2014;24(9):481-485.
- Harfterkamp M, van der Meer D, van der Loo-Neus G, Buitelaar JK, Minderaa RB, Hoekstra PJ. No evidence for predictors of response to atomoxetine treatment of attention-deficit/hyperactivity disorder symptoms in children and adolescents with autism spectrum disorder. *J Child Adolesc Psychopharmacol.* 2015;25(4):372-375.
- https://clinicaltrials.gov/ct2/show/NCT00380692

53. Herring2012, NCT00475735
- Herring WJ, Adler LA, Baranak CC, Liu K, Snavely D, Michelson D. Effects of the histamine inverse agonist MK-0249 in adult attention deficit disorder: A randomized, controlled, crossover study. *Biol Psychiatry.* 2010;1):217S.
- Herring WJ, Wilens TE, Adler LA, et al. Randomized controlled study of the histamine H3 inverse agonist MK-0249 in adult attention-deficit/hyperactivity disorder. *J Clin Psychiatry.* 2012;73(7):e891-898.
- https://clinicaltrials.gov/ct2/show/NCT00475735

54. Hervas2014 , SPD503-316, NCT01244490, EudraCT: 2010- 018579-12
- Hervas A, Huss M, Johnson M, et al. Efficacy and safety of extended-release guanfacine hydrochloride in children

and adolescents with attention-deficit/hyperactivity disorder: a randomized, controlled, phase III trial. *Eur Neuropsychopharmacol.* 2014;24(12):1861-1872.

- Hervas A, Huss M, Johnson M, et al. Guanfacine Xr (Gxr) for Children and Adolescents with Attention-Deficit/Hyperactivity Disorder (Adhd): Phase 3, Blind, Multicenter, Placebo- and Active-Reference Study. *Eur Psychiatry.* 2014;29.
- Pooled in: Huss M, Hervas A, Newcorn JH, et al. Guanfacine extended release for attention-deficit/hyperactivity disorder following inadequate response to prior methylphenidate. *Eur Neuropsychopharmacol.* 2014;24:S727-S728.
- Huss M, Hervas A, Johnson M, et al. Efficacy and Safety of Extended-Release Guanfacine Hydrochloride in Children and Adolescents with Attention-Deficit/Hyperactivity Disorder: A Randomized, Double-Blind, Multicentre, Placebo- and Active-Reference Phase 3 Study. *Aust N Z J Psychiatry.* 2015;49:111-111.
- Hervas A, Johnson M, McNicholas F, et al. Clinical global impressions-improvement scores by visit in a European, randomized, double-blind, placebo and active-controlled clinical trial of guanfacine extended release in children and adolescents with attention-deficit/hyperactivity disorder. *Eur Child Adolesc Psychiatry.* 2015;1):S138.
- Huss M, Johnson M, McNicholas F, et al. Attention-deficit/hyperactivity disorder rating scale IV subscale analysis by visit in a European, phase 3, randomized, double-blind clinical trial of guanfacine extended release in children and adolescents with attention-deficit/hyperactivity deficit. *Eur Child Adolesc Psychiatry.* 2015;1):S133-S134.
- Pooled in: Huss M, McBurnett K, Cutler A, Hervas A, Adeyi B, Dirks B. Clinical efficacy of guanfacine extended release among children with primarily inattentive subtype of Attention-Deficit/Hyperactivity Disorder: Results from four phase 3 studies. *ADHD Attention Deficit and Hyperactivity Disorders.* 2015;7:S44.
- Pooled in: Huss M, McBurnett K, Cutler AJ, et al. Separating efficacy and sedative effects of guanfacine extended release in children and adolescents with ADHD from four randomized, controlled, phase 3 clinical trials. *Eur Psychiatry.* 2016;33:S76-S77.
- Used for analysis in: Huss M, Sikirica V, Hervas A, Newcorn JH, Harpin V, Robertson B. Guanfacine extended release for children and adolescents with attention-deficit/hyperactivity disorder: efficacy following prior methylphenidate treatment. *Neuropsychiatric Disease & Treatment.* 2016;112:1085-1101.
- Secondary analysis in: Huss M, Hervas A, Dirks B, Bliss C, Prochazka J, Cutler A. Guanfacine extended release for children and adolescents with attention-deficit/hyperactivity disorder: Efficacy following prior stimulant treatment. *Eur Neuropsychopharmacol.* 2016;26:S737.
- Extension (EUCTR2011-004668-31-GB)**:** Huss M, Dirks B, Gu J, Ramos-Quiroga JA. Long-term growth-related safety outcomes of guanfacine extended release in children and adolescents with ADHD. *Eur Neuropsychopharmacol. Conference: 29th european college of* neuropsychopharmacology *congress, ECNP 2016. Austria. Conference start: 20160917. Conference end: 20160920.* 2017;26:S735-s736.
- https://clinicaltrials.gov/ct2/show/NCT01244490
- http://www.shiretrials.com/-/media/files/clinical%20trials/clinicaltrialsen/clinical%20study%20reports/shire-spd503-316-clinical-study-report-redact.pdf
- https://www.clinicaltrialsregister.eu/ctr-search/search?query=eudract_number:2010-018579-12
- Additional data provided by Shire

55. Huss2014 , CRIT124D2302, EUCTR2010-021533-31-DE, NCT01259492

- Huss M, Ginsberg Y, Philipsen A, et al. 40-Week, randomized, double-blind, placebo-controlled, multicenter, efficacy and safety study of methylphenidate hydrochloride modified release (MPH-LA) in adults with attention deficit hyperactivity disorder (ADHD). *Eur Psychiatry.* 2013;28.
- Huss M, Ginsberg Y, Tvedten T, et al. Methylphenidate hydrochloride modified release improved inattention and hyperactivity/impulsivity scores in adult ADHD patients. *Eur Neuropsychopharmacol.* 2013;23:S604-S605.
- Open label extension (NCT01338818) in: Ginsberg Y, Arngrim T, Philipsen A, et al. Long-term (1 year) safety and efficacy of methylphenidate modified-release long-acting formulation (MPH-LA) in adults with attention-deficit hyperactivity disorder: a 26-week, flexible-dose, open-label extension to a 40-week, double-blind, randomised, placebo-controlled core study. *CNS Drugs.* 2014;28(10):951-962.
- Post hoc analysis in: Huss M, Ginsberg Y, Arngrim T, et al. Open-label dose optimization of methylphenidate modified release long acting (MPH-LA): a post hoc analysis of real-life titration from a 40-week randomized trial. *Clin Drug Investig.* 2014;34(9):639-649.
- Huss M, Ginsberg Y, Tvedten T, et al. Methylphenidate hydrochloride modified-release in adults with attention deficit hyperactivity disorder: a randomized double-blind placebo-controlled trial. *Adv Ther.* 2014;31(1):44-65.
- Additional analyses in: Huss M, Ginsberg Y, Arngrim T, et al. Added benefits of long-term treatment of methylphenidate modified-release long-acting formulation in adults with ADHD. *Eur Neuropsychopharmacol.* 2014;24:S210.
- Huss M, Ginsberg Y, Arngrim T, et al. Efficacy and safety of methylphenidate modified release long-acting after reinstatement of therapy in placebo treated patients. *Eur Neuropsychopharmacol.* 2014;24:S209-S210.
- Huss M, Ginsberg Y, Arngrim T, et al. Adult ADHD treated with methylphenidate modified-release (MPH-LA)

maintained functional improvement over a period of 1 year. *ADHD Attention Deficit and Hyperactivity Disorders.* 2015;7:S50.

- Huss M, Ginsberg Y, Arngrim T, et al. Long-term safety of methylphenidate modified-release in adult ADHD upon continuous exposure up to 66 weeks. *ADHD Attention Deficit and Hyperactivity Disorders.* 2015;7:S50-S51.
- https://clinicaltrials.gov/ct2/show/NCT01259492
- https://www.clinicaltrialsregister.eu/ctr-search/search?query=eudract_number:2010-021533-31
- https://www.novctrd.com/CtrdWeb/displaypdf.nov?trialresultid=9544(study ID: CRIT124D2302)
- Additional information provided by Medice

56. Jafarinia2012

- Jafarinia M, Mohammadi MR, Modabbernia A, et al. Bupropion versus methylphenidate in the treatment of children with attention-deficit/hyperactivity disorder: randomized double-blind study. *Hum Psychopharmacol.* 2012;27(4):411-418.(ID: IRCT201012021556N18)

57. Jain2011, NCT00556959

- Jain R, Segal S, Kollins SH, Khayrallah M. Clonidine extended-release tablets for pediatric patients with attention-deficit/hyperactivity disorder. *J Am Acad Child Adolesc Psychiatry.* 2011;50(2):171-179.
- Summary in: Nguyen M, White KA, Bussing R. Clonidine extended release in the treatment of attention-deficit/ hyperactivity disorder. *Curr Psychiatry Rep*. 2011;13(5):313-315.
- https://clinicaltrials.gov/ct2/show/NCT00556959
- https://www.fda.gov/downloads/Drugs/DevelopmentApprovalProcess/DevelopmentResources/UCM224106.pdf
- https://www.fda.gov/downloads/Drugs/DevelopmentApprovalProcess/DevelopmentResources/UCM265919.pdf
- https://www.fda.gov/downloads/Drugs/DevelopmentApprovalProcess/DevelopmentResources/UCM224108.pdf&title=22331%20Clonidine%20Statistical%20PREA

58. Kahbazi2009

- Akhondzadeh S, Amiri S, Mohammadi M-R. A clinical trial of modafinil treatment in children and adolescents with attention deficit hyperactivity disorder: a double-blind and randomized trial. *Br J Clin Pharmacol.* 2008;65(6):981.
- Kahbazi M, Ghoreishi A, Rahiminejad F, Mohammadi MR, Kamalipour A, Akhondzadeh S. A randomized, double-blind and placebo-controlled trial of modafinil in children and adolescents with attention deficit and hyperactivity disorder. *Psychiatry Res.* 2009;168(3):234-237.

59. 60. Kay2009a,b

- Kay GG, Michaels MA, Pakull B. Simulated driving changes in young adults with ADHD receiving mixed amphetamine salts extended release and atomoxetine. *J Atten Disord.* 2009;12(4):316-329. (These data were presented in part at the 158th Annual Meeting of the American Psychiatric Association in Atlanta, GA, on May 24, 2005
- https://clinicaltrials.gov/ct2/show/NCT00557960 (additional ID: SLI381-316)

Note: cross-over with no wash out; only usable data: drop outs in pre cross-over phase

61. Kelsey2004, B4Z-US-LYBG

- Kelsey DK, Sumner CR, Sutton V, et al. Once-Daily Atomoxetine in Childhood ADHD: Continuous Symptom Relief. *156th Annual Meeting of the American Psychiatric Association, May 17-22, San Francisco CA2003:Nr716.*
- Kelsey DK, Sumner CR, Casat CD, et al. Once-daily atomoxetine treatment for children with attention-deficit/hyperactivity disorder, including an assessment of evening and morning behavior: a double-blind, placebo-controlled trial. *Pediatrics.* 2004;114(1):e1-8.
- Pooled in: Perwien AR, Faries DE, Kratochvil CJ, Sumner CR, Kelsey DK, Allen AJ. Improvement in health-related quality of life in children with ADHD: an analysis of placebo controlled studies of atomoxetine. *J Dev Behav Pediatr.* 2004;25(4):264-271.
- Pooled in: Biederman J, Spencer TJ, Newcorn JH, et al. Effect of comorbid symptoms of oppositional defiant disorder on responses to atomoxetine in children with ADHD: a meta-analysis of controlled clinical trial data. *Psychopharmacology (Berl)*. 2007;190:31-41
- Previous reference related to: Biederman J, Spencer T, Newcorn J, Gao H, Milton D, Feldman P. Does the Presence of Comorbid ODD Affect Responses to Atomoxetine? 158th Annual Meeting of the American Psychiatric Association; 2005 May 21-26; Atlanta, GA2005.
- Pooled in: Newcorn JH, Sutton VK, Weiss MD, Sumner CR. Clinical responses to atomoxetine in attention-deficit/hyperactivity disorder: the Integrated Data Exploratory Analysis (IDEA) study. *J Am Acad Child Adolesc Psychiatry.* 2009;48(5):511-518.
- Pooled in: Newcorn JH, Sutton VK, Zhang S, et al. Characteristics of placebo responders in pediatric clinical trials

of attention-deficit/hyperactivity disorder. *J Am Acad Child Adolesc Psychiatry.* 2009;48(12):1165-1172.
- Pooled in: Adler LA, Wilens T, Zhang S, et al. Atomoxetine treatment outcomes in adolescents and young adults with attention-deficit/hyperactivity disorder: results from a post hoc, pooled analysis. *Clin Ther.* 2012;34(2):363-373.
- https://assets.contentful.com/hadumfdtzsru/6Paceu2aB2yO0YO4qI8gEI/946961353d42e70064bf5e491ee77748/Atomoxetine-B4Z-US-LYBG.pdf
- https://www.accessdata.fda.gov/drugsatfda_docs/nda/2002/21-411_Strattera.cfm
- Full CSR provided by Lilly

62. Kollins2011, SPD503-206, NCT00150592
- Kollins SH, Wigal T, Vince B, et al. Guanfacine extended release in ADHD: Safety and sleep effects. *Proceedings of the Meeting of the American Academy of Child and Adolescent 2007; 23-28, Boston, MA.*
- Turnbow J, Kollins S, Lopez F, Lyne A, Youcha S, Rubin J. Response to Guanfacine Extended Release in Children and Adolescents with Attention-Deficit/Hyperactivity Disorder (ADHD). *Biol Psychiatry.* 2010;67(9, Suppl. S):218S.
- Kollins SH, Lopez FA, Vince BD, et al. Psychomotor functioning and alertness with guanfacine extended release in subjects with attention-deficit/hyperactivity disorder. *J Child Adolesc Psychopharmacol.* 2011;21(2):111-120.
- https://clinicaltrials.gov/ct2/show/results/NCT00150592 (additional ID:SPD503-206)
- Additional data provided by Shire

63. Kooij2004
- Kooij JJ, Burger H, Boonstra AM, Van der Linden PD, Kalma LE, Buitelaar JK. Efficacy and safety of methylphenidate in 45 adults with attention-deficit/hyperactivity disorder. A randomized placebo-controlled double-blind cross-over trial. *Psychol Med.* 2004;34(6):973-982.
- Boonstra AM, Kooij JJ, Oosterlaan J, Sergeant JA, Buitelaar JK. Does methylphenidate improve inhibition and other cognitive abilities in adults with childhood-onset ADHD? *J Clin Exp Neuropsychol.* 2005;27(3):278-298.
- Boonstra AM, Kooij JJ, Oosterlaan J, Sergeant JA, Buitelaar JK, Van Someren EJ. Hyperactive night and day? Actigraphy studies in adult ADHD: a baseline comparison and the effect of methylphenidate. *Sleep.* 2007;30(4):433-442.
- Kooij JS, Boonstra AM, Vermeulen SH, et al. Response to methylphenidate in adults with ADHD is associated with a polymorphism in SLC6A3 (DAT1). *Am J Med Genet B Neuropsychiatr Genet.* 2008;147B(2):201-208.
- Additional data provided by study author

64. Kurlan2002
- Kurlan R, Tourette Syndrome Study Group. Treatment of attention-deficit-hyperactivity disorder in children with Tourette's syndrome (TACT Trial). Ann Neurol.Abstracts from the American Neurological Association's 125th Annual Meeting; 2000 October 15-18; Boston, Massachusetts 2000;48(6):953.
- Kurlan R, Goetz CG, McDermott MP, et al. Treatment of ADHD in children with tics - A randomized controlled trial. *Neurology.* 2002;58(4):527-536.
- Kurlan R, Goldberg J. Clonidine and methylphenidate were effective for attention deficit hyperactivity disorder in children with comorbid tics. *Evidence-Based Medicine.* 2002;7(5):157.
- Deputy SR. Treatment of ADHD in children with tics: a randomized controlled trial. *Clin Pediatr (Phila).* 2002;41(9):736.
- Anon. (2002). Treatment of ADHD in children with tics. Hopital Extra, *Am J Nurs*. 102, 9, 24D.
- Goldberg J. Clonidine and methylphenidate were effective for attention deficit hyperactivity disorder in children with comorbid tics. *Evid Based Ment Health.* 2002, 5, 122
- Goldberg, J. (2002). Clonidine and methylphenidate were effective for attention deficit hyperactivity disorder in children with comorbid tics. *ACP J Club.* 137, 70.
- Additional data provided by study author

65. Lin2014, NCT00922636
- Lin DY, Kratochvil CJ, Xu W, et al. A randomized trial of edivoxetine in pediatric patients with attention-deficit/hyperactivity disorder. *J Child Adolesc Psychopharmacol.* 2014;24(4):190-200.
- https://clinicaltrials.gov/ct2/show/NCT00922636

66. Lin2016, NCT00917371
- Lin HY, Gau SS. Atomoxetine Treatment Strengthens an Anti-Correlated Relationship between Functional Brain Networks in Medication-Naive Adults with Attention-Deficit Hyperactivity Disorder: A Randomized Double-Blind Placebo-Controlled Clinical Trial. *Int J Neuropsychopharmacol.* 2015;19(3).

- https://clinicaltrials.gov/ct2/show/NCT00917371

67. Martenyi2010, B4Z-MW-LYCZ, NCT00386581

- Martenyi F, Zavadenko N, Jarkova N. Atomoxetine in the treatment of psychostimulant-naive children and adolescents with ADHD: a 6-week, randomized, placebo-controlled trial in Russia. *Eur Neuropsychopharmacol.* 2006;16(Suppl. 4):S527-S528.
- Martenyi F, Zavadenko NN, Jarkova NB, et al. Atomoxetine in children and adolescents with attention-deficit/hyperactivity disorder: a 6-week, randomized, placebo-controlled, double-blind trial in Russia. *Eur Child Adolesc Psychiatry.* 2010;19(1):57-66.
- https://clinicaltrials.gov/ct2/show/NCT00386581
- https://assets.contentful.com/hadumfdtzsru/6LOREfRmGAiCwAKSsokise/35fe9f24fed03897e6d6756c85e13670/Atomoxetine-B4Z-MW-LYCZ.pdf
- Full CSR provided by Lilly

68. McCracken2016

- Nurmi EL, Mallya A, Mallya KS, et al. Pharmacogenetics of growth effects complicating ADHDtreatment. Neuropsychopharmacology. 2013;38:S492-S493.
- Nurmi EL, Mallya KS, McGough J, et al. Pharmacogenetic moderators of methylphenidate and guanfacine response in children and adolescents with adhd. Neuropsychopharmacology. 2012;38:S219.
- McCracken JT, McGough JJ, Loo SK, et al. Combined Stimulant and Guanfacine Administration in Attention-Deficit/Hyperactivity Disorder: A Controlled, Comparative Study. *J Am Acad Child Adolesc Psychiatry.* 2016;55(8):657-666.
- Sayer GR, McGough JJ, Levitt J, et al. Acute and Long-Term Cardiovascular Effects of Stimulant, Guanfacine, and Combination Therapy for Attention-Deficit/Hyperactivity Disorder. *Journal of child and adolescent psychopharmacology.* 2016;26(10):882-888.
- Bilder RM, Loo S, McGough JJ, et al. Cognitive effects of stimulant, guanfacine, and combined treatment in child and adolescent attention-deficit/hyperactivity disorder. *Biol Psychiatry.* 2016;1):158S.
- Loo SK, Bilder RM, Cho AL, et al. Effects of d-Methylphenidate, Guanfacine, and Their Combination on Electroencephalogram Resting State Spectral Power in Attention-Deficit/Hyperactivity Disorder. *J Am Acad Child Adolesc Psychiatry.* 2016;55(8):674-682.
- https://clinicaltrials.gov/ct2/show/NCT00429273
- Additional information from study author

69. McRae-Clark2010, R21DA018221, NCT00360269

- McRae-Clark AL, Carter RE, Killeen TK, Carpenter MJ, White KG, Brady KT. A placebo-controlled trial of atomoxetine in marijuana-dependent individuals with attention deficit hyperactivity disorder. *Am J Addict.* 2010;19(6):481-489. (ID: R21DA018221).
- https://clinicaltrials.gov/ct2/show/NCT00360269
- Additional data from study author

70. Medori2008 , LAMDA-I EUCTR2004-000730-37, NCT00246220

- Medori R, Kooij S, Ramos-Quiroga JA, Buitelaar J, Lee E, Casas M. Efficacy and safety of OROS methylphenidate in adults with ADHD the long-acting methylphenidate in adult ADHD (LAMDA) trial. *J Neural Transm (Vienna).* 2007; 114(7).
- Medori R, Kooij S, Buitelaar J, et al. Double-blind study of the efficacy and safety of prolonged-release methylphenidate in adults with ADHD - the LAMDA trial. *Eur Neuropsychopharmacol.* 2007;17(Suppl. 4):S589.
- Sandner F. First results of the European LAMDA study on ADHD: Long-acting methylphenidate is effective and well tolerated in adults. [German]Retardiertes methylphenidat ist auch bei erwachsenen effektiv und vertraglich. *Journal fur Pharmakologie und Therapie.* 2007;16(6):181-182.
- Gerve M, Philipsen A, Roesler M, Sobanski E, Schauble B, Medori R, Trott GE. Effectiveness and compatibility of OROS (R)-methylphenidate for adult with ADHS - The long-acting methylphenidate in adult ADHD (LAMDA) trial. *Nervenarzt* 2007; 78(S2): 208-209; (46): 1-4.
- Roesler M, Trott GE, Philipsen A, Gerwe M, Lee E, Medori R, Schauble B, No. Efficacy and safety of OROS((R)) methylpheniclate in adults with ADHD: the long-acting methylphenidate in adult ADHD (LAMDA) trial. *Pharmacopsychiatry*. 2007; 40: 205.
- No authors listed. Erste Ergebnisse der europäischen LAMDA-Studie zu ADHS: Retardiertes Methylphenidat ist auch bei Erwachsenen effektiv und verträglich. *Journal für Pharmakologie und Therapie*. 2007; 16(6): 181.

- Kooij S, Medori R, Buitelaar J, Ramos-Quiroga JA, Lee E, Casas M. Open-label extension trial of the safety and tolerability of OROSO methylphenidate in adults with ADHD – the long-acting methylphenidate in adult ADHD (lamda) trial. *J Neural Transm (Vienna).* 2007; 114(7).
- Kooij J, Medori R, Buitelaar J, Ramos-Quiroga J, Lee E, Casas M. Open-label extension trial of the safety and tolerability of OROS methylphenidate in adults with adhd - the long-acting methylphenidate in adult ADHD (LAMDA) trial. *Program and abstracts of the American Psychiatric Association 2007 Annual Meeting; May 19-24, 2007; San Diego, California. Poster NR 647.*
- Medori R, Ramos-Quiroga JA, Casas M, et al. A randomized, placebo-controlled trial of three fixed dosages of prolonged-release OROS methylphenidate in adults with attention-deficit/hyperactivity disorder. *Biol Psychiatry.* 2008;63(10):981-989.
- Van Oene JC, Imhof L, Nyerod HJ, et al. Efficacy outcomes during longterm prolonged-release methylphenidate treatment of adults with ADHD. *Eur Neuropsychopharmacol.* 2009;19:S353-S4.
- Schaeuble B, Orman C, Palumbo JM. Efficacy of prolonged-release methylphenidate in a randomized controlled trial in adults with ADHD: Secondary endpoints. *Eur Neuropsychopharmacol.* 2009;19:S679-S680 (link confirmed by Dr Buitelaar)
- Schaeuble B, Hofecker M, Buitelaar JK, et al. Long-term treatment outcomes in adults with ADHD treated with OROS MPH. *Eur J Neurol.* 2010;17(Suppl. 3, Sp. Iss. SI):470.
- Post hoc analysis: Buitelaar JK, Kooij JJS, Ramos-Quiroga JA, et al. Predictors of treatment outcome in adults with ADHD treated with OROS methylphenidate. *Prog Neuropsychopharmacol Biol Psychiatry.* 2011(2):554-560.
- Open label extension in : Buitelaar JK, Casas M, Philipsen A, et al. Functional improvement and correlations with symptomatic improvement in adults with attention deficit hyperactivity disorder receiving long-acting methylphenidate. *Psychol Med.* 2012;42(1):195-204.
- Open label extension in : Buitelaar JK, Trott GE, Hofecker M, et al. Long-term efficacy and safety outcomes with OROS-MPH in adults with ADHD. *Int J Neuropsychopharmacol.* 2012(1):1-13. (NCT00307684)
- Pooled analysis in: Buitelaar JK, Sobanski E, Stieglitz RD, Dejonckheere J, Waechter S, Schauble B. Predictors of placebo response in adults with attention-deficit/hyperactivity disorder: data from 2 randomized trials of osmotic-release oral system methylphenidate. *J Clin Psychiatry.* 2012;73(8):1097-1102.
- Rosler M, Ginsberg Y, Arngrim T, et al. Correlation of symptomatic improvements with functional improvements and patient-reported outcomes in adults with attention-deficit/hyperactivity disorder treated with OROS methylphenidate. *World J Biol Psychiatry.* 2013;14(4):282-290.
- https://clinicaltrials.gov/ct2/show/NCT00246220
- Additional data from Janssen

71. Michelson2001, B4Z-MC-LYAC

- Michelson D, Faries D, Wernicke J, et al. Atomoxetine in the treatment of children and adolescents with attention-deficit/hyperactivity disorder: a randomized, placebo-controlled, dose-response study. *Pediatrics.* Nov 2001;108(5):E83.
- Pooled in: Dunn DW, Wernicke JF, Faries D, et al. Efficacy of atomoxetine in placebo-controlled pediatric attention-deficit hyperactivity disorder trials. *Ann Neurol.* 2001;50:S96-S.
- Pooled in: Allen AJ, Michelson D, Harder DR, Trapp NJ, Kelsey DK. Efficacy of atomoxetine in children and adolescents with ADHD. 155th Annual Meeting of the American Psychiatric Association; 2002 May 18-23; Philadelphia, PA2002:Nr348.
- Donnelly C, Faries D, Swensen A, et al. The effect of atomoxetine on the social and family functioning of children and adolescents with attention-deficit/hyperactivity disorder (ADHD). *Eur Neuropsychopharmacol.* 2002:S437.
- Allen AJ, Milton DR, Michelson D, Kelsey DK. Efficacy of atomoxetine treatment for adolescents with ADHD. 156th Annual Meeting of the American Psychiatric Association; 2003 May 17-22; San Francisco, CA2003:Nr643.
- Matza LS, Rentz AM, Secnik K, et al. The link between health-related quality of life and clinical symptoms among children with attention-deficit hyperactivity disorder. *J Dev Behav Pediatr.* 2004;25(3):166-174.
- Pooled in: Perwien AR, Faries DE, Kratochvil CJ, Sumner CR, Kelsey DK, Allen AJ. Improvement in health-related quality of life in children with ADHD: an analysis of placebo controlled studies of atomoxetine. *J Dev Behav Pediatr.* 2004;25(4):264-271.
- Newcorn JH, Spencer TJ, Biederman J, Milton DR, Michelson D. Atomoxetine treatment in children and adolescents with attention-deficit/hyperactivity disorder and comorbid oppositional defiant disorder. *J Am Acad Child Adolesc Psychiatry*. 2005;44(3):240-248.
- Pooled in: Michelson D, Read HA, Ruff DD, Witcher J, Zhang S, McCracken J. CYP2D60 and Clinical Response to Atomoxetine in Children and Adolescents with ADHD. *J Am Acad Child Adolesc Psychiatry*. 2007 2012-12-14 2007;46(2):242-251.

- Pooled in: Newcorn JH, Sutton VK, Weiss MD, Sumner CR. Clinical responses to atomoxetine in attention-deficit/hyperactivity disorder: the Integrated Data Exploratory Analysis (IDEA) study. *J Am Acad Child Adolesc Psychiatry*. 2009;48(5):511-518.
- Pooled in: Newcorn JH, Sutton VK, Zhang S, et al. Characteristics of placebo responders in pediatric clinical trials of attention-deficit/hyperactivity disorder. *J Am Acad Child Adolesc Psychiatry*. 2009;48(12):1165-1172.
- Pooled in: Block SL, Williams D, Donnelly CL, Dunn DW, Saylor KE, Ruberg SJ. Post hoc analysis: early changes in ADHD-RS items predict longer term response to atomoxetine in pediatric patients. *Clin Pediatr (Phila).* 2010;49(8):768-776.
- Pooled in: Adler LA, Wilens T, Zhang S, et al. Atomoxetine treatment outcomes in adolescents and young adults with attention-deficit/hyperactivity disorder: results from a post hoc, pooled analysis. *Clin Ther*. 2012;34(2):363-373.
- https://assets.contentful.com/hadumfdtzsru/E5UgeEzzbwqGagscMqAIC/4ec70be50f8aff4b6daac82c2e67e98c/Atomoxetine-B4Z-MC-LYAC.pdf
- https://www.accessdata.fda.gov/drugsatfda_docs/nda/2002/21-411_Strattera.cfm
- Full CSR provided by Lilly

72. Michelson2002, B4Z-MC-LYAT

- Michelson D, Allen AJ, Busner J, et al. Once-daily atomoxetine treatment for children and adolescents with attention deficit hyperactivity disorder: a randomized, placebo-controlled study. *Am J Psychiatry*. 2002;159(11):1896-1901.
- Pooled in: Allen AJ, Michelson D, Harder DR, Trapp NJ, Kelsey DK. Efficacy of atomoxetine in children and adolescents with ADHD. 155th Annual Meeting of the American Psychiatric Association; 2002 May 18-23; Philadelphia, PA2002:Nr348.
- Michelson D. Once-daily administration of atomoxetine: a new treatment for adhd. *155th Annual Meeting of the American Psychiatric Association.* 2002.
- Pooled in: Allen AJ, Milton DR, Michelson D, Kelsey DK. Efficacy of atomoxetine treatment for adolescents with ADHD. 156th Annual Meeting of the American Psychiatric Association; 2003 May 17-22; San Francisco, CA2003:Nr643
- Bukstein OG Once-daily atomoxetine may reduce attention deficit hyperactivity disorder symptoms in children and adolescents. *Evid Based Ment Health*. 2003; 6, 42. (Commentary)
- Pooled in: Biederman J, Gao H, Rogers AK, Spencer TJ. Comparison of parent and teacher reports of attention-deficit/hyperactivity disorder symptoms from two placebo-controlled studies of atomoxetine in children. *Biol Psychiatry*. 2006;60(10):1106-1110.
- Pooled in: Biederman J, Spencer TJ, Newcorn JH, et al. Effect of comorbid symptoms of oppositional defiant disorder on responses to atomoxetine in children with ADHD: a meta-analysis of controlled clinical trial data. *Psychopharmacology* (Berl). 2007;190:31-41.
- Previous reference related to: Biederman J, Spencer T, Newcorn J, Gao H, Milton D, Feldman P. Does the Presence of Comorbid ODD Affect Responses to Atomoxetine? 158th Annual Meeting of the American Psychiatric Association; 2005 May 21-26; Atlanta, GA2005.
- Pooled in: Michelson D, Read HA, Ruff DD, Witcher J, Zhang S, McCracken J. CYP2D60 and Clinical Response to Atomoxetine in Children and Adolescents with ADHD. *J Am Acad Child Adolesc Psychiatry.* 2007;46(2):242-251. (B4Z-MC-LYAI(4331); NCT00190684)
- Pooled in: Newcorn JH, Sutton VK, Weiss MD, Sumner CR. Clinical responses to atomoxetine in attention-deficit/hyperactivity disorder: the Integrated Data Exploratory Analysis (IDEA) study. *J Am Acad Child Adolesc Psychiatry.* 2009;48(5):511-518.
- Pooled in: Newcorn JH, Sutton VK, Zhang S, et al. Characteristics of placebo responders in pediatric clinical trials of attention-deficit/hyperactivity disorder. *J Am Acad Child Adolesc Psychiatry.* 2009;48(12):1165-1172.
- Pooled in: Block SL, Williams D, Donnelly CL, Dunn DW, Saylor KE, Ruberg SJ. Post hoc analysis: early changes in ADHD-RS items predict longer term response to atomoxetine in pediatric patients. *Clin Pediatr (Phila).* 2010;49(8):768-776.
- Pooled in: Adler LA, Wilens T, Zhang S, et al. Atomoxetine treatment outcomes in adolescents and young adults with attention-deficit/hyperactivity disorder: results from a post hoc, pooled analysis. *Clin Ther.* 2012;34(2):363-373.
- https://assets.contentful.com/hadumfdtzsru/6iueDKScs8sa0SO0y8IOCA/8cdf3a662993055780f76a30fd07fd30/Atomoxetine-B4Z-MC-LYAT.pdf
- https://www.accessdata.fda.gov/drugsatfda_docs/nda/2002/21-411_Strattera.cfm
- Full CSR provided by Lilly

73. 74. Michelson2003a,b

- Michelson D, Adler L, Spencer T, et al. Atomoxetine in adults with ADHD: two randomized, placebo-controlled studies. *Biol Psychiatry.* 2003;53(2):112-120.
- Spencer TJ. Efficacy and safety of atomoxetine in adults with ADHD. *156th Annual Meeting of the American Psychiatric Association, May 17-22, San Francisco CA.* 2003:No. 32.
- Biederman J, Faraone S, Spencer T, Michelson D, Jones D, Adler L. Effects of atomoxetine treatment on executive functioning in adults with attention-deficit/hyperactivity disorder (ADHD). *Eur Neuropsychopharmacol.* 2003;13(Supplement 4):S458.
- Michelson D, Marchant B. Emotional Dysregulation in ADHD and Response to Atomoxetine. *156th Annual Meeting of the American Psychiatric Association, May 17-22, San Francisco CA.* 2003:Nr640
- Included in: Wernicke JF, Adler L, Spencer T, et al. Changes in symptoms and adverse events after discontinuation of atomoxetine in children and adults with attention deficit/hyperactivity disorder: a prospective, placebo-controlled assessment. *J Clin Psychopharmacol.* 2004;24(1):30-35.
- Faraone SV, Biederman J, Spencer T, et al. Efficacy of atomoxetine in adult attention-deficit/hyperactivity disorder: a drug-placebo response curve analysis. *Behav Brain Funct.* 2005;1:16.
- Faraone SV, Biederman J, Spencer T, et al. Atomoxetine and stroop task performance in adult attention-deficit/hyperactivity disorder. *J Child Adolesc Psychopharmacol.* 2005;15(4):664-670.
- Reimherr FW, Faraone SV, Marchant BK, et al. Gender differences in adults with ADHD, pretreatment and following treatment with atomoxetine under double-blind conditions. *Eur Neuropsychopharmacol.* 2005;15(Suppl. 3):S604,S601.
- Reimherr FW, Marchant BK, Strong RE, et al. Emotional dysregulation in adult ADHD and response to atomoxetine. *Biol Psychiatry.* 2005;58(2):125-131.
- Previous reference related to: Reimherr FRE, Hedges DW, West SA, Adler LA, Michelson D, Marchant B. Emotional Dysregulation in ADHD and Response to Atomoxetine. *156th Annual Meeting of the American Psychiatric Association, May 17-22, San Francisco CA.* 2003:Nr640
- Post hoc analysis in: Spencer TJ, Faraone SV, Michelson D, et al. Atomoxetine and adult attention-deficit/hyperactivity disorder: the effects of comorbidity. *J Clin Psychiatry.* 2006;67(3):415-420.
- Robison RJ, Reimherr FW, Marchant BK, Faraone SV, Adler LA, West SA. Gender differences in 2 clinical trials of adults with attention-deficit/hyperactivity disorder: A retrospective data analysis. *J Clin Psychiatry.* 2008;69(2):213-221.
- Pooled in: Adler LA, Faraone SV, Spencer TJ, et al. The reliability and validity of self- and investigator ratings of ADHD in adults. *J Atten Disord.* 2008;11(6):711-719.
- Pooled in: Reimherr FW, Olsen J, Marchant BK, et al. ADHD Symptoms Associated with Comorbid Alcohol Dependence Or Abuse: Comparison of Subject Attributes within ADHD Clinical Trials. *Biol Psychiatry.* 2009;65:116S.
- Pooled in: Adler L, Wilens T, Zhang S, et al. Retrospective safety analysis of atomoxetine in adult ADHD patients with or without comorbid alcohol abuse and dependence. *Am J Addict.* 2009;18(5):393-401.
- Post hoc analysis in: Durell T, Adler L, Wilens T, Paczkowski M, Schuh K. Atomoxetine treatment for ADHD: younger adults compared with older adults. *J Atten Disord.* 2010;13(4):401-406.
- Follow-up in: Marchant BK, Reimherr FW, Halls C, et al. Long-term open-label response to atomoxetine in adult ADHD: influence of sex, emotional dysregulation, and double-blind response to atomoxetine. *Atten Defic Hyperact Disord.* 2011;3(3):237-244.
- Pooled in: Adler LA, Wilens T, Zhang S, et al. Atomoxetine treatment outcomes in adolescents and young adults with attention-deficit/hyperactivity disorder: results from a post hoc, pooled analysis. *Clin Ther.* 2012;34(2):363-373.
- Dittmann RW, Adler L, Michelson D, Wernicke J. Efficacy and safety of atomoxetine in adults with attention deficit/hyperactivity disorder. *Pharmacopsychiatry.* 2003;36:221.
- https://assets.contentful.com/hadumfdtzsru/5xQa80jOzm4qQE4GUYO8YQ/68a9810927a09ee745f4819bd9098dde/Atomoxetine-B4Z-MC-LYAA.pdf
  https://assets.contentful.com/hadumfdtzsru/6fLop0oa0oYGcu8M0mWWwA/17754d66b6dbcecf14f2e93aa21afb6b/Atomoxetine-B4Z-MC-LYAO.pdf
- https://www.accessdata.fda.gov/drugsatfda_docs/nda/2002/21-411_Strattera.cfm
- Full CSR for both studies provided by Lilly

75. Moharari2012 , IRCT201012295500N1

- Moharari F, Soltanifar A, Mokhber N, Pasandideh M, Samadi R, Soltanifar A. A double-blind, randomized comparison of efficacy and side effects of bupropion versus methylphenidate in children with ADHD. *Quarterly Journal of Sabzevar University of Medical Sciences*, 2012; 19,3.
- Moharreri F, Mokhber N, Samadi R, Soltanifar A. Double-blind randomized comparison of efficacy and side effects of bupropion versus methylphenidate for children with ADHD. *Eur Psychiatry*. 2013;28.

- http://www.irct.ir/searchresult.php?keyword=&id=5500&number=1&prt=1558&total=10&m=1 (IRCT201012295500N1)

76. Montoya2009, B4Z-XM-LYDM, NCT00191945
- Montoya A, Hervas A, Cardo E, et al. Evaluation of atomoxetine for first-line treatment of newly diagnosed, treatment-naive children and adolescents with attention deficit/hyperactivity disorder. *Curr Med Res Opin.* 2009;25(11):2745-2754.
- Subsample analysed in: Montoya A, Escobar R, Garcia-Polavieja MJ, et al. Changes of urine dihydroxyphenylglycol to norepinephrine ratio in children with attention-deficit hyperactivity disorder (ADHD) treated with atomoxetine. *J Child Neurol*. 2011;26(1):31-36.
- Post hoc analysis in: Escobar R, Montoya A, Polavieja P, et al. Evaluation of patients' and parents' quality of life in a randomized placebo-controlled atomoxetine study in attention-deficit/hyperactivity disorder. *J Child Adolesc Psychopharmacol*. 2009;19(3):253-263.
- Study from previous reference pooled in: Montoya A, Quail D, Anand E, Cardo E, Alda JA, Escobar R. Prognostic factors of improvement in health-related quality of life in atomoxetine-treated children and adolescents with attention-deficit/hyperactivity disorder, based on a pooled analysis. *Atten Defic Hyperact Disord*. 2014;6(1):25-34).
- Previous reference related to Montoya A, Quail D, Anand E, Cardo E, Alda JA, Escobar R. Prognostic factors of improvement in health related quality of life in children and adolescents with attention deficit/hyperactivity disorder, after atomoxetine treatment. *Eur Psychiatry*. 2012;27.
- https://clinicaltrials.gov/ct2/show/NCT00191945 (other ID: B4Z-XM-LYDM)
- https://www.clinicaltrialsregister.eu/ctr-search/search?query=eudract_number:2004-004088-31
- Additional information from study author

77. NCT01069523
- https://clinicaltrials.gov/ct2/show/NCT01069523
- Additional information provided by Dr. Pliszka

78. Newcorn2008, B4Z-MC-LYBI
- Michelson D. A double-blind placebo-controlled comparison of atomoxetine, OROS methylphenidate and placebo. Presented at: American Academy of Child and Adolescent Psychiatry Meeting, Washington, DC, October19-23,2004.
- From Gibson AP, Bettinger TL, Patel NC, Crismon ML. Atomoxetine versus stimulants for treatment of attention deficit/hyperactivity disorder. *Ann Pharmacother.* 2006;40(6):1134-1142.
- Newcorn JH, Michelson D, Kratochvil CJ, Allen AJ, Ruff DD, Moore RJ. Low-dose atomoxetine for maintenance treatment of attention-deficit/hyperactivity disorder. *Pediatrics.* 2006;118(6):e1701-1706.
- Study from previous reference pooled in: Adler LA, Wilens T, Zhang S, et al. Atomoxetine treatment outcomes in adolescents and young adults with attention-deficit/hyperactivity disorder: results from a post hoc, pooled analysis. *Clin Ther*. 2012;34(2):363-373.
- Newcorn JH, Kratochvil CJ, Allen AJ, et al. Atomoxetine and osmotically released methylphenidate for the treatment of attention deficit hyperactivity disorder: acute comparison and differential response. *Am J Psychiatry.* 2008;165(6):721-730.
- Newcorn JH, Kratochvil CJ, Allen AJ, et al. Osmotically released methylphenidate compared to atomoxetine for ADHD. The Brown University Child & Adolescent Psychopharmacology Update 2008; 10: 8:1.
- Pooled in: Newcorn JH, Sutton VK, Zhang S, et al. Characteristics of placebo responders in pediatric clinical trials of attention-deficit/hyperactivity disorder. *J Am Acad Child Adolesc Psychiatry.* 2009;48(12):1165-1172.
- Toplak ME. Osmotically released methylphenidate is more effective than atomoxetine in children and adolescents with ADHD. *Evid Based Ment Health.* 2009;12(1):19.
- https://assets.contentful.com/hadumfdtzsru/1kdmkwXhF0Qi4GoGcqwOEK/73114fca375740ef3010383870b7762e/Atomoxetine-B4Z-MC-LYBI.pdf
- Full CSR provided by Lilly

79. Newcorn2013, SPD503-314, NCT00997984
- Newcorn JH, Stein MA, Childress AC, et al. Randomized, double-blind trial of guanfacine extended release in children with attention-deficit/hyperactivity disorder: morning or evening administration. *J Am Acad Child Adolesc Psychiatry*. 2013;52(9):921-930.
- Young J, Rugino T, Dammerman R, Lyne A, Newcorn JH. Efficacy of guanfacine extended release assessed during the morning, afternoon, and evening using a modified Conners' Parent Rating Scale-revised: Short Form. *J Child Adolesc Psychopharmacol*. 2014;24(8):435-441.

- Stein MA, Sikirica V, Weiss MD, Robertson B, Lyne A, Newcorn JH. Does Guanfacine Extended Release Impact Functional Impairment in Children with Attention-Deficit/Hyperactivity Disorder? Results from a Randomized Controlled Trial. *CNS Drugs*. 2015;29(11):953-962.
- https://clinicaltrials.gov/ct2/show/NCT00997984 (other ID: SPD503-314)
- Additional data provided by Shire

80. Palumbo2008, NCT00031395

- Palumbo DR, Sallee FR, Pelham WE, Bukstein OG, Daviss WB, McDermott MP. Clonidine for attention-deficit/hyperactivity disorder treatment study: I. Efficacy and tolerability outcomes. *J Am Acad Child Adolesc Psychiatry*. 2008;47:179-187.
- Daviss, WB, Patel NC, Robb AS et al. Clonidine for attention-deficit/hyperactivity disorder: II. ECG changes and adverse events analysis. *J Am Acad Child Adolesc Psychiatry*. 2008;47(2):189-98.
- Cannon M, Pelham WH, Sallee FR, Palumbo DR, Bukstein O, Daviss WB. Effects of clonidine and methylphenidate on family quality of life in attention-deficit/hyperactivity disorder. *J Child Adolesc Psychopharmacol*. 2009;19(5):511-517.
- https://clinicaltrials.gov/ct2/show/NCT00031395

81. Paterson1999

- Paterson R, Douglas C, Hallmayer J, Hagan M, Krupenia Z. A randomised, double-blind, placebo-controlled trial of dexamphetamine in adults with attention deficit hyperactivity disorder. *The Aust N Z J Psychiatry*. 1999;33(4):494-502.
- Additional information provided by first author

82. Philipsen2015, EUCTR2006-000222-31-DE, ISRCTN54096201

- Philipsen A, Graf E, Tebartz van Elst L, et al. Evaluation of the efficacy and effectiveness of a structured disorder tailored psychotherapy in ADHD in adults: study protocol of a randomized controlled multicentre trial. *Atten Defic Hyperact Disord.* 2010;2(4):203-212.
- Schlander M, Philipsen A, Schwarz O. The cost effectiveness of clinically proven treatment strategies for attention-deficit/hyperactivity disorder (ADHD) in adult patients. *Value Health*. 2011;14 (7):A403.
- Philipsen A, Colla M, Jacob C, et al. Efficacy of psychotherapy in the treatment of adult ADHD-a randomized controlled multicentre trial. *Neuropsychiatr Enfance Adolesc.* 2012;1):S50-S51.
- Philipsen A, Graf E, Jans T, et al. A randomized controlled multicenter trial on the multimodal treatment of adult attention-deficit hyperactivity disorder: enrollment and characteristics of the study sample. *Atten Defic Hyperact Disord.* 2014;6(1):35-47.
- Philipsen A, Jans T, Matthies S, et al. Multimodal treatment of adult ADHD: A randomized controlled multicentre trial (COMPAS). *Atten Defic Hyperact Disord* . 2015;7:S16.
- Philipsen A, Jans T, Graf E, et al. Effects of Group Psychotherapy, Individual Counseling, Methylphenidate, and Placebo in the Treatment of Adult Attention-Deficit/Hyperactivity Disorder: A Randomized Clinical Trial. *JAMA Psychiatry*. 2015;72(12):1199-1210. [Erratum appears in *JAMA Psychiatry*. 2016;73(1):90; PMID: 26747290].
- van Elst LT, Maier S, Kloppel S, et al. The effect of methylphenidate intake on brain structure in adults with ADHD in a placebo-controlled randomized trial. *J Psychiatry Neurosci*. 2016;41(6):422-430.
- https://www.clinicaltrialsregister.eu/ctr-search/search?query=eudract_number:2006-000222-31
- http://isrctn.org/ISRCTN54096201
- Additional information provided by study author

83. Pliszka2000

- Pliszka SR, Wynne SK, Olvera RL, Browne RG. Comparing adderall methylphenidate in ADHD. *152nd Annual Meeting of the American Psychiatric Association; 1999 May 15-20; Washington, DC.* 1999:Nr595.
- Pliszka SR, Browne RG, Olvera RL, Wynne SK. A double-blind, placebo-controlled study of Adderall and methylphenidate in the treatment of attention-deficit/hyperactivity disorder. *J Am Acad Child Adolesc Psychiatry*. 2000;39(5):619-626.
- Faraone SV, Pliszka SR, Olvera RL, Skolnik R, Biederman J. Efficacy of Adderall and methylphenidate in attention deficit hyperactivity disorder: a reanalysis using drug-placebo and drug-drug response curve methodology. *J Child Adolesc Psychopharmacol*. 2001;11(2):171-180.
- Faraone SV, Pliszka SR, Olvera RL, Biederman J. Adderall and methylphenidate in ADHD. *Annual Meeting of the American Psychiatric Association, 2001.*
- Additional information from study author

84. Reimherr2005

- Reimherr Frederick W. Six-week, double-blind, placebo-controlled trial of bupropion sustained release in the treatment of adults with adhd. *155th Annual Meeting of the American Psychiatric Association.* 2002. http://onlinelibrary.wiley.com/o/cochrane/clcentral/articles/109/CN-00430109/frame.html.
- Reimherr FW, Hedges DW, Strong RE, Marchant BK, Williams ED. Bupropion SR in adults with ADHD: a short-term, placebo-controlled trial. *Neuropsychiatr Dis Treat.* 2005;1(3):245-251.
- Additional information from study author

85. Reimherr2007
- Reimherr FW, Williams ED, Strong RE, Mestas R, Soni P, Marchant BK. A double-blind, placebo-controlled, crossover study of osmotic release oral system methylphenidate in adults with ADHD with assessment of oppositional and emotional dimensions of the disorder. *J Clin Psychiatry.* 2007;68(1):93-101.
- Related to previous: Reimherr FW, Marchant BK, Olsen JL, Kondo D, Strong RE, Robison RJ. Serotonin Receptor Genes are Associated with Treatment Response and Symptom Severity in Adults with ADHD. *Biol Psychiatry.* 2010;67(9, Suppl. S):220S.
- Gale P, Reimherr FW, Marchant BK, et al. The incidence of personality disorder in adults with ADHD, and the effects on response to treatment with OROS methylphenidate (OROS MPH). *Biol Psychiatry*. 2008;63:245S.
- Post hoc analysis in: Marchant BK, Reimherr FW, Williams ED, Strong RE, Halls C, Soni P. Assessment of personality disorder in adult ADHD using data from a clinical trial of OROS methylphenidate (OROS MPH). *Biol Psychiatry.* 2008;63(7, Suppl. S):246S.
- Previous reference related to: Williams E, Reimherr FW, Marchant BK, Strong RE, Halls C, Soni P. Personality Disorder Assessment in Adult ADHD Utilizing Subjects Enrolled in a Clinical Trial of OROS (R) Methylphenidate (OROS (R) MPH). *J Child Adolesc Psychopharmacol.* 2008;18(6):635-636.
- Reimherr FW, Marchant BK, Olsen JL, Kondo D, Strong RE, Robison RJ. Serotonin Receptor Genes are Associated with Treatment Response and Symptom Severity in Adults with ADHD. *Biol Psychiatry.* 2010;67:220S.
- Follow-up in: Marchant BK, Reimherr FW, Halls C, Williams ED, Strong RE. OROS methylphenidate in the treatment of adults with ADHD: a 6-month, open-label, follow-up study. *Ann Clin Psychiatry.* 2010;22(3):196-204.
- Post hoc analysis in: Williams ED, Reimherr FW, Marchant BK, et al. Personality disorders in ADHD Part 1: Assessment of personality disorder in adult ADHD using data from a clinical trial of OROS methylphenidate. *Ann Clin Psychiatry.* 2010;22(3):84-93.
- Robison RJ, Reimherr FW, Gale PD, et al. Personality disorders in ADHD Part 2: The effect of symptoms of personality disorder on response to treatment with OROS methylphenidate in adults with ADHD. *Ann Clin Psychiatry.* 2010;22(2):94-102.
- Reimherr FW, Marchant BK, Williams ED, Strong RE, Halls C, Soni P. Personality disorders in ADHD Part 3: Personality disorder, social adjustment, and their relation to dimensions of adult ADHD. *Ann Clin Psychiatry.* 2010;22(2):103-112.
- Olsen JL, Reimherr FW, Marchant BK, Strong RE, Robison RJ. BDNF and TPH2 are Associated with Personality Disorder in Adults with Attention-Deficit Hyperactivity Disorder. *Biol Psychiatry.* 2010;67(9, Suppl. S):245S.
- Reimherr FW, Marchant BK, Gift TE, Steans TA, Wender PH. Types of adult attention-deficit hyperactivity disorder (ADHD): baseline characteristics, initial response, and long-term response to treatment with methylphenidate. *Atten Defic Hyperact Disord.* 2015;7(2):115-128.
- https://clinicaltrials.gov/ct2/show/NCT02215538
- Additional information from study author

86. Rosler2009
- Rosler M, Fischer R, Ammer R, Ose C, Retz W. A randomised, placebo-controlled, 24-week, study of low-dose extended-release methylphenidate in adults with attention-deficit/hyperactivity disorder. *Eur Arch Psychiatry Clin Neurosci.* 2009;259(2):120-129.
- Post hoc analysis in: Rosler M, Retz W, Fischer R, et al. Twenty-four-week treatment with extended release methylphenidate improves emotional symptoms in adult ADHD. *World J Biol Psychiatry.* 2010;11(5):709-718.
- Post hoc analysis in: Sobanski E, Retz W, Fischer R, et al. Treatment adherence and persistence in adult ADHD: results from a twenty-four week controlled clinical trial with extended release methylphenidate. *Eur Psychiatry: the Journal of the Association of European Psychiatrists.* 2014;29(5):324-330.
- https://clinicaltrials.gov/ct2/show/NCT00619840
- Additional data provided by Medice

87. Rugino2003
- Rugino TA, Samsock TC, Adkins L. Modafinil in children with ADHD: a double-blind, placebo-controlled study. *155th Annual Meeting of the American Psychiatric Association; 2002 May 18-23; Philadelphia, PA.* 2002:No. 77.

- Rugino TA, Samsock TC. Modafinil in children with attention-deficit hyperactivity disorder. *Pediatr Neurol.* 2003;29(2):136-142.

88. Rugino2014, NCT01156051
- Rugino TA. Effect on Primary Sleep Disorders When Children With ADHD Are Administered Guanfacine Extended Release. *J Atten Disord.* 2014. DOI: 10.1177/1087054714554932.
- https://clinicaltrials.gov/ct2/show/NCT01156051

89. Sallee2009, SPD503-304, NCT00150618
- Biederman J, Lyne A, Sallee FR, et al. Efficacy and Safety of Guanfacine Extended Release in Attention-Deficit/ Hyperactivity Disorder. *Presented at the 54th Annual Meeting of the American Academy of Child & Adolescent Psychiatry; 23 - 28 October 2007 Boston, Massachusetts*
- Sallee FR, McGough J, Wigal T, Donahue J, Lyne A, Biederman J. Guanfacine extended release in children and adolescents with attention-deficit/hyperactivity disorder: a placebo-controlled trial. *J Am Acad Child Adolesc Psychiatry.* 2009;48(2):155-165.
- Pooled in: Sallee FR, Lyne A, Wigal T, McGough JJ. Long-term safety and efficacy of guanfacine extended release in children and adolescents with attention-deficit/hyperactivity disorder. *J Child Adolesc Psychopharmacol.* 2009;19(3):215-226. (open label extension plus another open label study)
- Related to previous reference: Sallee F, McGough JJ, Wigall T, Farrand K, Lyne A, Biederman J. Long-term safety and efficacy of guanfacine extended release in children and adolescents with Attention-Deficit/Hyperactivity disorder. *Biol Psychiatry.* 2008;63:247S.
- Pooled in: Faraone SV, Glatt SJ. Effects of extended-release guanfacine on ADHD symptoms and sedation-related adverse events in children with ADHD. *J Atten Disord.* 2010;13(5):532-538.
- Pooled in: Sallee FR, Kollins SH, Wigal TL. Efficacy of guanfacine extended release in the treatment of combined and inattentive only subtypes of attention-deficit/hyperactivity disorder. *J Child Adolesc Psychopharmacol.* 2012;22(3):206-214.
- https://www.clinicaltrials.gov/ct2/show/NCT00150618 (other ID: SPD503-304)
- https://www.accessdata.fda.gov/drugsatfda_docs/nda/2009/022037_intuniv_toc.cfm
- http://www.shirecanada.com/-/media/shire/shireglobal/shirecanada/pdffiles/product%20information/intuniv-xr-pm-en.pdf
- Additional data provided by Shire

90. Sangal2006, B4Z-US-LYAV
- Sangal RB, Owens J, AlleRn AJ, Sutton V, Schuh K, Kelsey D. Effects of atomoxetine and methylphenidate on sleep in children with ADHD. *Sleep.* 2006;29(12):1573-1585.
- Sangal R, Owens, Allen AI, et aI. Effects of atomoxetine and methylphenidate on sleep in children with ADHD. Presented at: Associated Professional Sleep Societies Meeting, Philadelphia, June5-10, 2004
- Additional data/information provided by Lilly (ID: B4Z-US-LYAV (5002))

91. Scahill2001, NCT00004376
- Scahill L, Chappell P, Kim YS, et al. Guanfacine in the treatment of children with tic disorders and attention deficit hyperactivity disorder: a placebo-controlled study. *39th Annual Meeting of the American College of Neuropsychopharmacology 2000; Dec 10-14; San Juan, Puerto Rico 2000.*
- Scahill L, Chappell PB, Kim YS, et al. A placebo-controlled study of guanfacine in the treatment of children with tic disorders and attention deficit hyperactivity disorder. *Am J Psychiatry.* 2001;158(7):1067-1074.
- https://clinicaltrials.gov/ct2/show/NCT00004376

92. Schrantee2016, NTR3103, EUCTR2010-023654-37-NL
- Bottelier MA, Schouw M, Klomp A, et al. The effects of Psychotropic drugs On Developing brain (ePOD) study: methods and design. *BMC Psychiatry.* 2014;14(1):48. (Protocol).
- Ferguson B, Schrantee AGM, De Ruiter MB, Bottelier MA, Reneman L. Opposite effects of acute methylphenidate administration in children versus adult ADHD patients during emotional processing. *Eur Neuropsychopharmacol.* 2014;24:S728-S729.
- Schrantee A, Tamminga HGH, Bouziane C, et al. Age-dependent effects of methylphenidate on the human dopaminergic system in young vs adult patients with attention-deficit/hyperactivity disorder: A randomized clinical trial. *JAMA Psychiatry.* 2016;73:955-962.
- Schrantee A, Tamminga HGH, Bouziane C, et al. A randomized clinical trial on the age-dependent effects of methylphenidate on the human dopaminergic system. *Biol Psychiatry.* 2016;(1):157S.
- Note: Full study protocol in supplemental material of Schrantee et al. *JAMA Psychiatry* 2016

- http://www.trialregister.nl/trialreg/admin/rctview.asp?TC=3103 (additional ID: NL34509.000.10)
- https://www.clinicaltrialsregister.eu/ctr-search/search?query=eudract_number:2010-023654-37
- Additional information provided by first author

93. Schulz2012
- Schulz KP, Fan J, Bedard AC, et al. Common and unique therapeutic mechanisms of stimulant and nonstimulant treatments for attention-deficit/hyperactivity disorder. *Arch Gen Psychiatry.* 2012;69(9):952-961.

94. Simonoff2013 , ISRCTN68384912
- Simonoff E, Taylor E, Baird G, et al. Randomized controlled double-blind trial of optimal dose methylphenidate in children and adolescents with severe attention deficit hyperactivity disorder and intellectual disability. *J Child Psychol Psychiatry.* 2013;54(5):527-535.
- *Commentary*: Lipkin PH. Methylphenidate reduces ADHD symptoms in children with severe ADHD and intellectual disability. *Evid Based Ment Health.* 2013;16(4):104.
- http://isrctn.org/ISRCTN68384912
- Additional information from study author

95. Singer1995
- Singer HS, Brown J, Quaskey S, Rosenberg LA, Mellits ED, Denckla MB. The treatment of attention-deficit hyperactivity disorder in Tourette's syndrome: a double-blind placebo-controlled study with clonidine and desipramine. *Pediatrics*. 1995;95(1):74-81.
- Singer HS, Brown J, Quaskey S, Mellits ED, Denckla MB, Rosenberg LA. The treatment of attention-deficit hyperactivity disorder in Tourette syndrome - a double-blind placebo-controlled study with clonidine and desipramine. *Ann Neurol.* 1991;30:485-.

96.SPD489-405, NCT01552915
- https://clinicaltrials.gov/ct2/show/NCT01552915
- Additional data/information provided by Shire

97. SPD489-406, NCT01552902
- https://clinicaltrials.gov/ct2/show/NCT01552902
- Additional data/information provided by Shire

98. Spencer1995
- Spencer T, Wilens T, Biederman J, Faraone SV, Ablon JS, Lapey K. A double-blind, crossover comparison of methylphenidate and placebo in adults with childhood-onset attention-deficit hyperactivity disorder. *Arch Gen Psychiatry.* 1995;52(6):434-443.
- Wilens TE, Hammerness PG, Biederman J, et al. Blood pressure changes associated with medication treatment of adults with attention-deficit/hyperactivity disorder. *J Clin Psychiatry.* 2005;66(2):253-259.

99. Spencer1998
- Spencer T, Biederman J, Wilens T, Prince J, Hacth M, Jones J, Harding M, Faraone SV, Seidman L. Effectiveness and tolerability of tomoxetine in adults with attention deficit hyperactivity disorder. *Am J Psychiatry*. 1998;155 (5):693-695.

100. Spencer2001
- Spencer TJ, Biederman J, Bostic JQ, Prince JB, Gerard K. Efficacy and tolerability of a mixed amphetamine salts compound in adults with adhd. 153rd *Annual Meeting of the American Psychiatric Association 2000.*
- Spencer T, Biederman J, Wilens T, et al. Efficacy of a mixed amphetamine salts compound in adults with attention-deficit/hyperactivity disorder. *Arch Gen Psychiatry.* 2001;58(8):775-782.
- Commentary in: Newcorn JH. Amphetamine salt compound treatment for adults with attention deficit hyperactivity disorder. *Curr Psychiatry. Rep.* 2002;4(2):85-86.
- Spencer TJ. Efficacy and tolerability of a mixed amphetamine salts compound in adults with adhd. *155th Annual Meeting of the American Psychiatric Association.* 2002.
- Faraone SV, Biederman J, Spencer TJ, et al. Dose-Response Efficacy of Mixed Amphetamine Salts Extended Release in Adults With ADHD. *157th Annual Meeting of the American Psychiatric Association; 2004 May 1-6; New York, NY 2004:Nr440.*
- Post hoc analysis in: Wilens TE, Hammerness PG, Biederman J, et al. Blood pressure changes associated with medication treatment of adults with attention-deficit/hyperactivity disorder. *J Clin Psychiatry.* 2005;66(2):253-259.

101. 102. Spencer2002 , B4Z-MC-HFBD, B4Z-MC-HFBK

- Pooled in: Dunn DW, Wernicke JF, Faries D, et al. Efficacy of atomoxetine in placebo-controlled pediatric attention-deficit hyperactivity disorder trials. *Ann Neurol*. 2001;50:S96-S.
- Stein MA, Lewis J, Conlon C, et al. A comparison of tomoxetine hydrochloride and placebo in pediatric patients with Attention-Deficit/Hyperactivity Disorder. *Pediatr Res.* 2001;49(4 Part 2):451A.
- Spencer T, Heiligenstein JH, Biederman J, et al. Results from 2 proof-of-concept, placebo-controlled studies of atomoxetine in children with attention-deficit/hyperactivity disorder. *J Clin Psychiatry.* 2002;63(12):1140-1147.
- Data in girls only in: Biederman J, Heiligenstein JH, Faries DE, et al. Efficacy of atomoxetine versus placebo in school-age girls with attention-deficit/hyperactivity disorder. *Pediatrics.* 2002;110(6):e75.
- Subset analysis in: Kaplan S, Heiligenstein J, West S, et al. Efficacy and safety of atomoxetine in childhood attention-deficit/hyperactivity disorder with comorbid oppositional defiant disorder. *J Atten Disord.* 2004;8(2):45-52.
- Included in: Wernicke JF, Adler L, Spencer T, et al. Changes in symptoms and adverse events after discontinuation of atomoxetine in children and adults with attention deficit/hyperactivity disorder: a prospective, placebo-controlled assessment. *J Clin Psychopharmacol.* 2004;24(1):30-35.
- Pooled in: Michelson D, Read HA, Ruff DD, Witcher J, Zhang S, McCracken J. CYP2D60 and Clinical Response to Atomoxetine in Children and Adolescents with ADHD. *J Am Acad Child Adolesc Psychiatry.* 2007;46(2):242-251.
- Pooled in: Newcorn JH, Sutton VK, Weiss MD, Sumner CR. Clinical responses to atomoxetine in attention-deficit/hyperactivity disorder: the Integrated Data Exploratory Analysis (IDEA) study. *J Am Acad Child Adolesc Psychiatry.* 2009;48(5):511-518.
- Pooled in: Newcorn JH, Sutton VK, Zhang S, et al. Characteristics of placebo responders in pediatric clinical trials of attention-deficit/hyperactivity disorder. *J Am Acad Child Adolesc Psychiatry.* 2009;48(12):1165-1172.
- Pooled in: Block SL, Williams D, Donnelly CL, Dunn DW, Saylor KE, Ruberg SJ. Post hoc analysis: early changes in ADHD-RS items predict longer term response to atomoxetine in pediatric patients. *Clin Pediatr (Phila).* 2010;49(8):768-776.
- Pooled in: Adler LA, Wilens T, Zhang S, et al. Atomoxetine treatment outcomes in adolescents and young adults with attention-deficit/hyperactivity disorder: results from a post hoc, pooled analysis. *Clin Ther.* 2012;34(2):363-373.
- https://assets.contentful.com/hadumfdtzsru/mPlwKp0FRQawsC8CU4KmW/5abf313ad3718e1aebc62b09682712fc/Atomoxetine-B4Z-MC-HFBD.pdf ; https://assets.contentful.com/hadumfdtzsru/665NUmKWsgCEmMAoKaEGgy/ce8a8ad38895ab7a3bd7ce5f0db7423b/Atomoxetine-B4Z-MC-HFBK.pdf
- https://www.accessdata.fda.gov/drugsatfda_docs/nda/2002/21-411_Strattera.cfm
- Full CSR provided by Lilly for both studies.

103. Spencer2005

- Spencer TJ. Preliminary results of a six-month trial of methylphenidate in adults with adhd. 156th Annual Meeting of the American Psychiatric Association, May 17-22, San Francisco CA2003:No. 54B.
- Spencer T, Biederman J, Eric M, Stephen F. Efficacy in a 6-month trial of methylphenidate in adults with attention-deficit/hyperactivity disorder. *Int J Neuropsychopharmacol.* 2004;7:S442-S3.
- Spencer T, Biederman J, Wilens T, et al. A large, double-blind, randomized clinical trial of methylphenidate in the treatment of adults with attention-deficit/hyperactivity disorder. *Biol Psychiatry.* 2005;57(5):456-463.
- Pooled in: Mick E, Biederman J, Spencer T, Faraone SV, Sklar P. Absence of association with DAT1 polymorphism and response to methylphenidate in a sample of adults with ADHD. *Am J Med Genet B Neuropsychiatr Genet.* 2006;141B(8):890-894.
- Pooled in: Biederman J, Mick EO, Surman C, et al. Comparative acute efficacy and tolerability of OROS and immediate release formulations of methylphenidate in the treatment of adults with attention-deficit/hyperactivity disorder. *BMC Psychiatry.* 2007;7:49.
- Pooled in: Mick E, Faraone SV, Spencer T, Zhang HF, Biederman J. Assessing the validity of the Quality of Life Enjoyment and Satisfaction Questionnaire Short Form in adults with ADHD. *J Atten Disord*. Jan 2008;11(4):504-509
- Surman CBH, Monuteaux MC, Petty CR, et al. Representativeness of participants in a clinical trial for attention-deficit/hyperactivity disorder? Comparison with adults from a large observational study. *J Clin Psychiatry.* 2010;71(12):1612-1616.

104. Spencer2006, SLI381-314, NCT00507065

- Wilens TE, Spencer TJ, Biederman J, Weisler RH, Read SC, Partiot A. Mixed amphetamine salts XR: cardiovascular safety in adolescents with ADHD. *158th Annual Meeting of the American Psychiatric Association; 2005 May 21-26; Atlanta, GA2005*

- Wilens TE, Spencer TJ, Biederman J. Short- and long-term cardiovascular effects of mixed amphetamine salts extended-release in adolescents with ADHD. *CNS Spectr.* 2005;10(10 Suppl 15):22-30.
- Spencer TJ, Wilens TE, Biederman J, Weisler RH, Read SC, Pratt R. Efficacy and safety of mixed amphetamine salts extended release (Adderall XR) in the management of attention-deficit/hyperactivity disorder in adolescent patients: a 4-week, randomized, double-blind, placebo-controlled, parallel-group study. *Clin Ther.* 2006;28(2):266-279.
- https://clinicaltrials.gov/ct2/show/NCT00507065 (additional ID: SLI381-314**)**
- Additional data provided by Shire

105. Spencer2007, CRIT124E2302
- Spencer TJ, Kim S, Jiang H. Efficacy of Dexmethylphenidate Extended Release Capsules in Adults With ADHD. *157th Annual Meeting of the American Psychiatric Association; 2004 May 1-6; New York, NY.* 2004:Nr455.
- Adler LA, Spencer T, Wang J, Pestreich L, Muniz R. Efficacy and safety of once-daily extended-release dexmethylphenidate 30 and 40 mg in adults with ADHD: A double-blind, fixed-dose, placebo-controlled, randomized study. *Int J Neuropsychopharmacol.* 2006;9(Suppl. 1):S229.
- Spencer TJ, Adler LA, McGough JJ, Muniz R, Jiang H, Pestreich L. Efficacy and safety of dexmethylphenidate extended-release capsules in adults with attention-deficit/hyperactivity disorder. *Biol Psychiatry.* 2007;61(12):1380-1387.
- Follow-up in: Adler LA, Spencer T, McGough JJ, Jiang H, Muniz R. Long-term effectiveness and safety of dexmethylphenidate extended-release capsules in adult ADHD. *J Atten Disord.* 2009;12(5):449-459.
- https://www.novctrd.com/CtrdWeb/displaypdf.nov?trialresultid=1623 (other iD: CRIT124E2302)
- https://www.accessdata.fda.gov/drugsatfda_docs/nda/2005/021802s000TOC.cfm

106. Spencer2008, SPD465-301, NCT00150579
- Spencer TJ, Adler LA, Weisler RH, Youcha SH. Triple-bead mixed amphetamine salts (SPD465), a novel, enhanced extended-release amphetamine formulation for the treatment of adults with ADHD: a randomized, double-blind, multicenter, placebo-controlled study. *J Clin Psychiatry.* 2008;69(9):1437-1448.
- Spencer TJ, Landgraf JM, Adler LA, Weisler RH, Anderson CS, Youcha SH. Attention-deficit/hyperactivity disorder-specific quality of life with triple-bead mixed amphetamine salts (SPD465) in adults: results of a randomized, double-blind, placebo-controlled study. *J Clin Psychiatry.* 2008;69(11):1766-1775.
- Pooled in: Brown TE, Landgraf JM. Improvements in executive function correlate with enhanced performance and functioning and health-related quality of life: evidence from 2 large, double-blind, randomized, placebo-controlled trials in ADHD. *Postgrad Med.* 2010;122(5):42-51.
- Secondary analysis in: Surman CB, Roth T. Impact of stimulant pharmacotherapy on sleep quality: post hoc analyses of 2 large, double-blind, randomized, placebo-controlled trials. *J Clin Psychiatry.* 2011;72(7):903-908.
- https://clinicaltrials.gov/ct2/show/NCT00150579 (other ID: SPD465-301)
- Additional data provided by Shire

107. Stein2011, NCT00393042
- Stein MA, Waldman ID, Sarampote C, Seymour K, Cook EH. Dopamine transporter genotype (DAT1) predicts stimulant response in children with attention deficit hyperactivity disorder. *Pediatr Res*. 2004;55:1A.
- Wiebe S, Gruber R, Charney E, Aryal S, Waldman I, Newcorn J, et al. Sleep and emotional reactivity to extended release dexmethylphenidate versus mixed amphetamine salts: a double-blind, placebo controlled study. European Child & Adolescent Psychiatry. Proceedings of the Eunethydis 1st International ADHD Conference: From Data to Best Clinical Practice; 2010 May 26-28; Amsterdam, Netherlands 2010;19(Suppl 1):S82
- Stein MA, Waldman ID, Charney E, et al. Dose effects and comparative effectiveness of extended release dexmethylphenidate and mixed amphetamine salts. *J Child Adolesc Psychopharmacol.* 2011;21(6):581-588.
- Santisteban JA, Stein MA, Bergmame L, Gruber R. Effect of extended-release dexmethylphenidate and mixed amphetamine salts on sleep: a double-blind, randomized, crossover study in youth with attention-deficit hyperactivity disorder. *CNS Drugs.* 2014;28(9):825-833. (NCT00393042)
- Santisteban JA, Stein MA, Gruber R. Effects of dosage on sleep duration during stimulant treatment of ADHD in youth. *Paediatr Child Health (Canada).* 2014;19 (6):e54-e55.
- Stein MA, Waldman I, Newcorn J, Bishop J, Kittles R, Cook EH, Jr. Dopamine transporter genotype and stimulant dose-response in youth with attention-deficit/hyperactivity disorder. *J Child Adolesc Psychopharmacol.* 2014;24(5):238-244.
- Conference proceeding related to previous reference: Newcorn J, Stein M. Dopamine transporter genotype and dose-response following treatment with psychostimulants: Implications for optimization of acute and longer-term treatment. *Atten Defic Hyperact Disord.* 2015;7:S67.
- https://clinicaltrials.gov/ct2/show/NCT00393042

- Additional information/data provided by the first author

108. Sutherland2012, NCT00174226

- Sutherland SM, Adler LA, Chen C, Smith MD, Feltner DE. An 8-week, randomized controlled trial of atomoxetine, atomoxetine plus buspirone, or placebo in adults with ADHD. *J Clin Psychiatry.* 2012;73(4):445-450.
- https://clinicaltrials.gov/ct2/show/NCT00174226

109. Svanborg2009, B4Z-SO-LY15, EUCTR2004-003941-42-SE, NCT00191542

- Svanborg P, Thernlund G, Gustafsson PA, Hagglof B, Poole L, Kadesjo B. Efficacy and safety of atomoxetine as add-on to psychoeducation in the treatment of attention deficit/hyperactivity disorder: A randomized, double-blind, placebo-controlled study in stimulant-naive Swedish children and adolescents. *Eur Child Adolesc Psychiatry.* 2009;18(4):240-249.
- Svanborg P, Thernlund G, Gustafsson PA, Hagglof B, Schacht A, Kadesjo B. Atomoxetine improves patient and family coping in attention deficit/hyperactivity disorder: a randomized, double-blind, placebo-controlled study in Swedish children and adolescents. *Eur Child Adolesc Psychiatry.* 2009;18(12):725-735.
- Myren KJ, Thernlund G, Nylen A, Schacht A, Svanborg P. Atomoxetine's effect on societal costs in Sweden. *J Atten Disord.* 2010;13(6):618-628.
- Pooled in: Montoya A, Quail D, Anand E, Cardo E, Alda JA, Escobar R. Prognostic factors of improvement in health-related quality of life in atomoxetine-treated children and adolescents with attention-deficit/hyperactivity disorder, based on a pooled analysis. *Atten Defic Hyperact Disord.* 2014;6(1):25-34.
- Previous reference Related to: Montoya A, Quail D, Anand E, Cardo E, Alda JA, Escobar R. Prognostic factors of improvement in health related quality of life in children and adolescents with attention deficit/hyperactivity disorder, after atomoxetine treatment. *Eur Psychiatry.* 2012;27
- https://www.clinicaltrialsregister.eu/ctr-search/search?query=eudract_number:2004-003941-42
- https://clinicaltrials.gov/ct2/show/NCT00191542 (other ID: B4Z-SO-LY15. 6671**)**

110. Swanson2006

- Biederman J, Wilens TE, Lopez FA. Modafinil pediatric formulation has early and sustained effect in ADHD. *158th Annual Meeting of the American Psychiatric Association; 2005 May 21-26; Atlanta, GA2005.*
- Swanson JM, Greenhill LL, Lopez FA, et al. Modafinil film-coated tablets in children and adolescents with attention-deficit/hyperactivity disorder: results of a randomized, double-blind, placebo-controlled, fixed-dose study followed by abrupt discontinuation. *J Clin Psychiatry.* 2006;67(1):137-147.
- Pooled in: Wigal SB, Biederman J, Swanson JM, Yang R, Greenhill LL. Efficacy and safety of modafinil film-coated tablets in children and adolescents with or without prior stimulant treatment for attention-deficit/hyperactivity disorder: pooled analysis of 3 randomized, double-blind, placebo-controlled studies. *Prim Care Companion J Clin Psychiatry.* 2006;8(6):352-360.
- Pooled in: Biederman J, Pliszka SR. Modafinil improves symptoms of attention-deficit/hyperactivity disorder across subtypes in children and adolescents. *J Pediatr.* 2008;152(3):394-399.
- https://www.accessdata.fda.gov/drugsatfda_docs/nda/98/020717A_Provigil.cfm
- Modafinil (CEP-1538) Tablets, Supplemental NDA 20-717/S-019, ADHD Indication
Briefing Document for Psychopharmacologic Drugs Advisory Committee Meeting (Document provided by Dr Eric Konofal)

111. Takahashi2009, B4Z-JE-LYBC, NCT00191295

- Takahashi M, Takita Y, Yamazaki K, et al. A randomized, double-blind, placebo-controlled study of atomoxetine in Japanese children and adolescents with attention-deficit/hyperactivity disorder. *J Child Adolesc Psychopharmacol.* 2009;19(4):341-350.
- https://assets.contentful.com/hadumfdtzsru/4Twp3uVNc4a6eIMiGmAWKI/93fcf81d15873e185bd5eeba6f32aeb5/Atomoxetine-B4Z-JE-LYBC.pdf
- https://www.accessdata.fda.gov/drugsatfda_docs/nda/2002/21-411_Strattera.cfm
- https://clinicaltrials.gov/ct2/show/NCT00191295

112. Takahashi2014, NCT01323192

- Takahashi N, Koh T, Tominaga Y, Saito Y, Kashimoto Y, Matsumura T. A randomized, double-blind, placebo-controlled, parallel-group study to evaluate the efficacy and safety of osmotic-controlled release oral delivery system methylphenidate HCl in adults with attention-deficit/hyperactivity disorder in Japan. *World J Biol Psychiatry.* 2014.
- https://clinicaltrials.gov/ct2/show/NCT01323192
- https://yoda.yale.edu/sites/default/files/nct01323192.pdf

113. Taylor1987

- Taylor E, Schachar R, Thorley G, Wieselberg HM, Everitt B, Rutter M. Which boys respond to stimulant medication? A controlled trial of methylphenidate in boys with disruptive behaviour. *Psychol Med.* 1987;17(1):121-143.
- Schachar R, Taylor E, Wieselberg M, Thorley G, Rutter M. Changes in family function and relationships in children who respond to methylphenidate. *J Am Acad Child Adolesc Psychiatry.* 1987;26(5):728-732.
- Additional information from study author

114. Taylor2000

- Taylor FB, Russo J. Efficacy of modafinil compared to dextroamphetamine for the treatment of attention deficit hyperactivity disorder in adults. *J Child Adolesc Psychopharmacol.* 2000;10(4):311-320.
- Taylor IF. Comparing modafinil to dextroamphetamine in the treatment of adult adhd. *153rd Annual Meeting of the American Psychiatric Association.* 2000.
- Taylor FB. Comparing modafinil to dextroamphetamine in the treatment of adult adhd. *155th Annual Meeting of the American Psychiatric Association.* 2002.

115. Taylor2001

- Taylor FB, Russo J. Comparing guanfacine and dextroamphetamine for the treatment of adult attention-deficit/hyperactivity disorder. *J Clin Psychopharmacol.* 2001;21(2):223-228.

Taylor IF. Comparing guanfacine and dextroamphetamine for adult adhd: efficacy and implications. *153rd Annual Meeting of the American Psychiatric Association.* 2000.

- Taylor FB. Comparing guanfacine and dextroamphetamine for adult adhd: efficacy and implications. 155th Annual Meeting of the American Psychiatric Association 2002

116. van der Meere1999

- van der Meere J, Gunning B, Stemerdink N. The effect of methylphenidate and clonidine on response inhibition and state regulation in children with ADHD. *J Child Psychol Psychiatry.* 1999;40(2):291-298.
- Gunning WB. A Controlled Trial of Clonidine in Hyperkinetic Children. Erasmus Universiteit Rotterdam, 1992. http://repub.eur.nl/res/pub/8339/920311_Gunning,%20Willem%20Boudewijn.pdf

117. Wang2007, NCT00486083, B4Z-MC-LYBR (6934)

- Levine L, Wang Y, Zheng Y, Du Y, Cho SC, Gao H, Marquez-Caraveo ME, Rogers AK, Williams DW. Atomoxetine vs. methylphenidate in ADHD treatment: an international pediatric trials. *Eur Neuropsychopharamacol* 2005, 15, Suppl 3, S602.
- Wang Y, Zheng Y, Du Y, et al. Atomoxetine versus methylphenidate in paediatric outpatients with attention deficit hyperactivity disorder: a randomized, double-blind comparison trial. *The Aust N Z J Psychiatry.* 2007;41(3):222-230.
- https://clinicaltrials.gov/ct2/show/NCT00486083
- https://assets.contentful.com/hadumfdtzsru/4RGQpHv0BOWMMcCcAyii2s/a91d17111b5aa1326bef55e28cf7cc06/Atomoxetine-B4Z-MC-LYBR.pdf
- https://www.accessdata.fda.gov/drugsatfda_docs/nda/2002/21-411_Strattera.cfm
- Additional information from Lilly

118. Wehmeier2012, B4Z-SB-LYDV, NCT00546910

- Wehmeier PM, Schacht A, Wolff C, Otto WR, Dittmann RW, Banaschewski T. Neuropsychological outcomes across the day in children with attention-deficit/hyperactivity disorder treated with atomoxetine: results from a placebo-controlled study using a computer-based continuous performance test combined with an infra-red motion-tracking device. *J Child Adolesc Psychopharmacol.* 2011;21(5):433-444.
- Wehmeier PM, Schacht A, Ulberstad F, et al. Does atomoxetine improve executive function, inhibitory control, and hyperactivity? Results from a placebo-controlled trial using quantitative measurement technology. *J Clin Psychopharmacol.* 2012;32(5):653-660.
- Post hoc analysis in: Wehmeier PM, Kipp L, Banaschewski T, Dittmann RW, Schacht A. Does Comorbid Disruptive Behavior Modify the Effects of Atomoxetine on ADHD Symptoms as Measured by a Continuous Performance Test and a Motion Tracking Device? *J Atten Disord.* 2015;19(7):591-602
- Post hoc analysis in: Wehmeier PM, Dittmann RW, Banaschewski T, Schacht A. Does stimulant pretreatment modify atomoxetine effects on core symptoms of ADHD in children assessed by quantitative measurement technology? *J Atten Disord.* 2014;18(2):105-116.
- https://clinicaltrials.gov/ct2/show/NCT00546910 (additional ID: B4Z-SB-LYDV(11148)**;**

- Additional information provided by Lilly

119. Weisler2006, SLI381-303

- Weisler RH, Biederman J, Chrisman AK, Timothy TP, Frazer N, Tulloch SJ. Long-Term Safety and Efficacy of Once-Daily Adderall Extended Release in Adults With ADHD. 156th Annual Meeting of the American Psychiatric Association, May 17-22, San Francisco CA2003:Nr647.
- Weisler RH, Chrisman AK, Wilens TE. Adderall xr dosed once daily in adult patients with adhd. 156th *Annual Meeting of the American Psychiatric Association, May 17-22, San Francisco CA. 2003:No. 33.* http://onlinelibrary.wiley.com/o/cochrane/clcentral/articles/838/CN-00592838/frame.html.
- Long-term follow-up in: Biederman J, Spencer TJ, Wilens TE, Weisler RH, Read SC, Tulloch SJ. Long-term safety and effectiveness of mixed amphetamine salts extended release in adults with ADHD. *CNS Spectr.* 2005;10(12 Suppl 20):16-25.
- Weisler RH, Biederman J, Spencer TJ, Wilens TE. Long-term cardiovascular effects of mixed amphetamine salts extended release in adults with ADHD. *CNS Spectr.* 2005;10(12 Suppl 20):35-43.
- Weisler RH, Biederman J, Spencer TJ, et al. Mixed amphetamine salts extended-release in the treatment of adult ADHD: a randomized, controlled trial. *CNS Spectr.* 2006;11(8):625-639.
- Pooled in: Lasser R, Dirks B, Adeyi B, Babcock T. Comparative efficacy and safety of lisdexamfetamine dimesylate and mixed amphetamine salts extended release in adults with attention-deficit/hyperactivity disorder. *Prim psychiatry.* 2010;17(9):44-54.
- Additional data provided by Shire (ID: SLI381-303)

120. Weisler2012, NCT00880217

- Weisler RH, Pandina GJ, Daly EJ, Cooper K, Gassmann-Mayer C, Investigato ATTS. Randomized Clinical Study of a Histamine H-3 Receptor Antagonist for the Treatment of with Attention-Deficit Hyperactivity Disorder. *CNS Drugs*. 2012;26(5):421-434.
- https://clinicaltrials.gov/ct2/show/NCT00880217 (additional ID: 31001074-ATT2001)
- Additional information from manufacturer

121. Weiss2005, B4Z-MC-LYAW

- Weiss M, Tannock R, Kratochvil C, et al. Placebo-controlled study of once-daily atomoxetine in the school setting. *Eur Neuropsychopharmacol.* 2003;13(Supplement 4):S456-S457.
- Pooled in: Perwien AR, Faries DE, Kratochvil CJ, Sumner CR, Kelsey DK, Allen AJ. Improvement in health-related quality of life in children with ADHD: an analysis of placebo controlled studies of atomoxetine. *J Dev Behav Pediatr.* 2004;25(4):264-271.
- Weiss M, Tannock R, Kratochvil C, et al. A randomized, placebo-controlled study of once-daily atomoxetine in the school setting in children with ADHD. *J Am Acad Child Adolesc Psychiatry.* 2005;44(7):647-655.
- Bohnstedt BN, Kronenberger WG, Dunn DW, et al. Investigator ratings of ADHD symptoms during a randomized, placebo-controlled trial of atomoxetine: a comparison of parents and teachers as informants. *J Atten Disord.* 2005;8(4):153-159.
- Brown RT, Perwien A, Faries DE, Kratochvil CJ, Vaughan BS. Atomoxetine in the management of children with ADHD: effects on quality of life and school functioning. *Clin Pediatr (Phila).* 2006;45(9):819-827.
- Commentary: Barton J. Atomoxetine improves teacher rated symptoms in children with ADHD more than placebo. *Evid Based Ment Health.* 2006;9(1):7.
- Pooled in: Biederman J, Gao H, Rogers AK, Spencer TJ. Comparison of parent and teacher reports of attention-deficit/hyperactivity disorder symptoms from two placebo-controlled studies of atomoxetine in children. *Biol Psychiatry.* 2006;60(10):1106-1110.
- Pooled in: Biederman J, Spencer TJ, Newcorn JH, et al. Effect of comorbid symptoms of oppositional defiant disorder on responses to atomoxetine in children with ADHD: a meta-analysis of controlled clinical trial data. *Psychopharmacology (Berl).* 2007;190:31-41
- Related to previous reference: Biederman J, Spencer T, Newcorn J, Gao H, Milton D, Feldman P. Does the Presence of Comorbid ODD Affect Responses to Atomoxetine? 158th Annual Meeting of the American Psychiatric Association; 2005 May 21-26; Atlanta, GA2005.
- https://assets.contentful.com/hadumfdtzsru/1PT69wZkT2UosACGuAImuw/f80b9521e50d1cb2dce4fcd31c96d845/Atomoxetine-B4Z-MC-LYAW_a_.pdf
- https://assets.contentful.com/hadumfdtzsru/28G1mHvyIkSqww8w4AueWU/e99d85ebd3c15c789c20791f3d52410f/Atomoxetine-B4Z-MC-LYAW_b_.pdf
- https://www.accessdata.fda.gov/drugsatfda_docs/nda/2002/21-411_Strattera.cfm
- Full CSR provided by Lilly

122. Wender2011

- Wender PH, Reimherr FW, Marchant B, Czajkowski L, Sanford ME. A placebo-controlled, long-term trial of methylphenidate in the treatment of adults with ADHD. *2001 Annual Meeting of the American Psychiatric Association.* 2001.
- Wender Paul H. A placebo-controlled, long-term trial of methylphenidate in the treatment of adults with adhd. *155th Annual Meeting of the American Psychiatric Association 2002.*
- Wender PH, Szajkowski L, Marchant B, Reimherr FW, Sanford E, Eden J. A Long-Term Study of Methylphenidate in the Treatment of ADHD in Adults. *156th Annual Meeting of the American Psychiatric Association, May 17-22, San Francisco CA2003:Nr708.*
- Wender PH, Reimherr FW, Marchant B, Sanford E, Czajkowski L. A Long-Term Trial of Methylphenidate in the Treatment of ADHD in Adults. 156th Annual Meeting of the American Psychiatric Association, May 17-22, San Francisco CA. 2003:Nr709.
- Wender PH, Reimherr FW, Marchant BK, Sanford ME, Czajkowski LA, Tomb DA. A one year trial of methylphenidate in the treatment of ADHD. *J Atten Disord.* 2011;15(1):36-45.
- https://clinicaltrials.gov/ct2/show/NCT00693212
- Additional data from study author

123. Wietecha2013, NCT00607919

- Shaywitz SE, Shaywitz BA, Wietecha LA, et al. Effects of atomoxetine on reading abilities in children with dyslexia and children with attention deficit/hyperactivity disorder and comorbid dyslexia. *Ann Neurol.* 2012;72:S204.
- Wietecha L, Williams D, Shaywitz S, et al. Atomoxetine improved attention in children and adolescents with attention-deficit/hyperactivity disorder and dyslexia in a 16 week, acute, randomized, double-blind trial. *J Child Adolesc Psychopharmacol.* 2013;23(9):605-613.
- Post-hoc analysis in: McBurnett K, Clemow D, Williams D, Villodas M, Wietecha L, Barkley R. Atomoxetine-Related Change in Sluggish Cognitive Tempo Is Partially Independent of Change in Attention-Deficit/Hyperactivity Disorder Inattentive Symptoms. *J Child Adolesc Psychopharmacol.* 2016;27:38-42.
- Shaywitz S, Shaywitz B, Wietecha L, et al. Effect of Atomoxetine Treatment on Reading and Phonological Skills in Children with Dyslexia or Attention-Deficit/Hyperactivity Disorder and Comorbid Dyslexia in a Randomized, Placebo-Controlled Trial. *J Child Adolesc Psychopharmacol.* 2016;27(1):19-28.
- https://clinicaltrials.gov/ct2/show/NCT00607919
- Additional information from Lilly

124. Wigal2004

- Connors K, Casant C, Elia J, et al. Randomized Trial of d-MPH Focalin and d,1-MPH in Children with ADHD [abstract]. 155th Annual Meeting of the American Psychiatric Association; 2002 May 18-23; Philadelphia, PA2002
- Wigal S, Swanson JM, Feifel D, et al. A double-blind, placebo-controlled trial of dexmethylphenidate hydrochloride and d,l-threo-methylphenidate hydrochloride in children with attention-deficit/hyperactivity disorder. *J Am Acad Child Adolesc Psychiatry.* 2004;43(11):1406-1414.
- Post hoc analysis in: Weiss M, Wasdell M, Patin J. A post hoc analysis of d-threo-methylphenidate hydrochloride (focalin) versus d,l-threo-methylphenidate hydrochloride (ritalin). *J Am Acad Child Adolesc Psychiatry.* 2004;43(11):1415-1421.
- https://www.accessdata.fda.gov/drugsatfda_docs/nda/2001/21-278_Focalin_medr_P2.pdf

125. Wigal 2005, SLI381-404, NCT00506727

- Wigal S, McGough JJ, Posner K, Kollins SH, Michaels A, Tulloch SJ. Analog classroom study of amphetamine extended release and atomoxetine in youth with ADHD. *157th Annual Meeting of the American Psychiatric Association; 2004 May 1-6; New York, NY.* 2004.
- Wigal SB, McGough JJ, McCracken JT, et al. A laboratory school comparison of mixed amphetamine salts extended release (Adderall XR) and atomoxetine (Strattera) in school-aged children with attention deficit/hyperactivity disorder. *J Atten Disord.* 2005;9(1):275-289.
- Data on girls only in: Biederman J, Wigal SB, Spencer TJ, McGough JJ, Mays DA. A post hoc subgroup analysis of an 18-day randomized controlled trial comparing the tolerability and efficacy of mixed amphetamine salts extended release and atomoxetine in school-age girls with attention-deficit/hyperactivity disorder. Clin Ther. Feb 2006;28(2):280-293.
- Post hoc analysis in: Faraone SV, Wigal SB, Hodgkins P. Forecasting three-month outcomes in a laboratory school comparison of mixed amphetamine salts extended release (Adderall XR) and atomoxetine (Strattera) in school-aged children with ADHD. *J Atten Disord.* 2007;11(1):74-82.
- https://clinicaltrials.gov/ct2/show/NCT00506727 (other ID: SLI381-404**)**
- Additional information from manufacturer

126. Wigal2015, NCT01239030

- Wigal SB, Nordbrock E, Adjei AL, Childress A, Kupper RJ, Greenhill L. Efficacy of Methylphenidate Hydrochloride Extended-Release Capsules (Aptensio XR™) in Children and Adolescents with Attention-Deficit/Hyperactivity Disorder: A Phase III, Randomized, Double-Blind Study. *CNS Drugs.* 2015;29(4):331-40. doi: 10.1007/s40263-015-0241-3.
- Wigal SB, Adjei AL, Childress A, Chang WW, Kupper RJ. A study of methylphenidate extended-release capsules in a randomized, double-blind, placebo-controlled protocol in children and adolescents with ADHD. *CNS spectrums. Conference: 2014 NEI psychopharmacology congress. United states. Conference start: 20141113. Conference end: 20141116.* 2017;20(1):67-68.
- Pooled in: Owens J, Weiss M, Nordbrock E, et al. Effect of Aptensio XR (methylphenidate HCl extended-release) capsules on sleep in children with attention-deficit/hyperactivity disorder. *J Child Adolesc Psychopharmacol.* 2016;26:873-881
- https://clinicaltrials.gov/ct2/show/NCT01239030 (other ID: RP-BP-EF002)

127. Wilens2001

- Wilens TE, Spencer TJ, Biederman J, et al. A controlled clinical trial of bupropion for attention deficit hyperactivity disorder in adults. *Am J Psychiatry.* 2001;158(2):282-288.
- Commentary in: Ferre, J.C. & Nutt, D. (2001). Bupropion improved symptoms in adults with attention deficit hyperactivity disorder. Evidence Based Mental Health, 158, 282-288
- Commentary in: Newcorn JH. Bupropion for adults with attention deficit hyperactivity disorder. *Curr Psychiatry Rep.* 2002;4(2):86-87.
- Post hoc analysis in: Wilens TE, Hammerness PG, Biederman J, et al. Blood pressure changes associated with medication treatment of adults with attention-deficit/hyperactivity disorder. *J Clin Psychiatry.* 2005;66(2):253-259.

128. Wilens2005, NCT00048360

- Wilens TE, Hudziak JJ, Connor DF, et al. A Controlled Trial of Extended-Release Bupropion in Adult ADHD. *157th Annual Meeting of the American Psychiatric Association; 2004 May 1-6; New York, NY2004:Nr576.*
- Wilens TE, Haight BR, Horrigan JP, et al. Bupropion XL in adults with attention-deficit/hyperactivity disorder: a randomized, placebo-controlled study. *Biol Psychiatry.* 2005;57(7):793-801.
- https://clinicaltrials.gov/ct2/show/NCT00048360 (other ID: AK130934)

129. Wilens2008, B4Z-MC-LYBY, NCT00190957

- Wilens TE, Adler LA, Weiss MD, Ramsey JL, Moore RF, Renard D, Trzepacz PT, Schuh LM, Dittmann RW, Levine LR, No. Atomoxetine treatment of adults with ADHD and comorbid alcohol abuse disorder. *Pharmacopsychiatry*. 2007; 40: 150
- Wilens TE, Adler LA, Weiss MD, et al. Atomoxetine treatment of adults with ADHD and comorbid alcohol abuse. *Proceedings of the 69th Annual Scientific Meeting of the College on Problems of Drug Dependence; 2007 June 16-21; Quebec City, Canada. 2007.*
- Wilens TE, Adler LA, Weiss MD, et al. Atomoxetine treatment of adults with ADHD and comorbid alcohol use disorders. *Drug Alcohol Depend.* 2008;96(1-2):145-154.
- Pooled in: Adler L, Wilens T, Zhang S, et al. Retrospective safety analysis of atomoxetine in adult ADHD patients with or without comorbid alcohol abuse and dependence. *Am J Addict.* 2009;18(5):393-401
- Pooled in: Reimherr FW, Olsen J, Marchant BK, et al. ADHD Symptoms Associated with Comorbid Alcohol Dependence Or Abuse: Comparison of Subject Attributes within ADHD Clinical Trials. *Biol Psychiatry*. 2009;65:116S.
- Post hoc analysis in: Wilens TE, Adler LA, Tanaka Y, et al. Correlates of alcohol use in adults with ADHD and comorbid alcohol use disorders: exploratory analysis of a placebo-controlled trial of atomoxetine. *Curr Med Res Opin.* 2011;27(12):2309-2320.
- https://clinicaltrials.gov/ct2/show/NCT00190957
- https://assets.contentful.com/hadumfdtzsru/1PXcFgjDCAW6EUOYMg4I80/da1e7c4d91490b68c9fd23385bdc3920/Atomoxetine-B4Z-MC-LYBY.pdf
- Full CSR provided by Lilly

130. Wilens2011, NCT00528697

- Wilens TE, Gault LM, Childress A, et al. Safety and efficacy of ABT-089 in pediatric attention-deficit/hyperactivity disorder: results from two randomized placebo-controlled clinical trials. *J Am Acad Child Adolesc Psychiatry.* 2011;50(1):73-84 e71
- https://clinicaltrials.gov/ct2/show/NCT00528697

131. Wilens2015, SPD503-312, EUCTR2011-002221-21, NCT01081132

- Wilens TE, Robertson B, Sikirica V, et al. A Randomized, Placebo-Controlled Trial of Guanfacine Extended Release in Adolescents with Attention-Deficit/Hyperactivity Disorder. *J Am Acad Child Adolesc Psychiatry.* 2015;54(11):916-925.e912.
- Wilens TE, Harper L, Young JL, et al. Clinical response and symptomatic remission in adolescents with Attention-Deficit/Hyperactivity Disorder receiving guanfacine extended release in a phase 3 study. *ADHD Attention Deficit and Hyperactivity Disorders.* 2015;7:S98.
- Cutler A, Harper L, Young J, Adeyi B, Dirks B, Wilens T. Guanfacine extended release: Daytime sleepiness outcomes from a phase 3 clinical study in adolescents with attention-deficit/hyperactivity disorder. *Eur Neuropsychopharmacol.* 2015;25:S648-S649.
- Pooled in: Huss M, McBurnett K, Cutler A, Hervas A, Adeyi B, Dirks B. Clinical efficacy of guanfacine extended release among children with primarily inattentive subtype of Attention-Deficit/Hyperactivity Disorder: Results from four phase 3 studies. *Atten Defic Hyperact Disord.* 2015;7:S44.
- Pooled in: Huss M, McBurnett K, Cutler AJ, et al. Separating efficacy and sedative effects of guanfacine extended release in children and adolescents with ADHD from four randomized, controlled, phase 3 clinical trials. *Eur Psychiatry.* 2016;33:S76-S77.
- Secondary analysis in: Huss M, Hervas A, Dirks B, Bliss C, Prochazka J, Cutler A. Guanfacine extended release for children and adolescents with attention-deficit/hyperactivity disorder: Efficacy following prior stimulant treatment. *Eur Neuropsychopharmacol.* 2016;26:S737.
- https://clinicaltrials.gov/ct2/show/NCT01081132 (other ID: SPD503-312)
- https://www.clinicaltrialsregister.eu/ctr-search/search?query=eudract_number:2011-002221-21
- Additional data provided by Shire

132. Winhusen2010, NCT00253747

- Winhusen TM, Somoza EC, Brigham GS, et al. Impact of attention-deficit/hyperactivity disorder (ADHD) treatment on smoking cessation intervention in ADHD smokers: a randomized, double-blind, placebo-controlled trial. *J Clin Psychiatry.* 2010;71(12):1680-1688.
- Covey LS, Hu MC, Winhusen T, Weissman J, Berlin I, Nunes EV. OROS-methylphenidate or placebo for adult smokers with attention deficit hyperactivity disorder: racial/ethnic differences. *Drug Alcohol Depend.* 2010;110(1-2):156-159.
- Covey LS, Hu MC, Weissman J, Croghan I, Adler L, Winhusen T. Divergence by ADHD subtype in smoking cessation response to OROS-methylphenidate. *Nicotine Tob Res.* 2011;13(10):1003-1008.
- Pooled in: Winhusen TM, Lewis DF, Riggs PD, et al. Subjective effects, misuse, and adverse effects of osmotic-release methylphenidate treatment in adolescent substance abusers with attention-deficit/hyperactivity disorder. *J Child Adolesc Psychopharmacol.* 2011;21(5):455-463.
- Berlin I, Hu MC, Covey LS, Winhusen T. Attention-deficit/hyperactivity disorder (ADHD) symptoms, craving to smoke, and tobacco withdrawal symptoms in adult smokers with ADHD. *Drug Alcohol Depend.* 2012;124(3):268-273.
- Westover AN, Nakonezny PA, Winhusen T, Adinoff B, Vongpatanasin W. Risk of methylphenidate-induced prehypertension in normotensive adult smokers with attention deficit hyperactivity disorder. *J Clin Hypertens (Greenwich, Conn.).* 2013;15(2):124-132.
- Post hoc analysis in: Nunes EV, Covey LS, Brigham G, et al. Treating nicotine dependence by targeting attention-deficit/ hyperactivity disorder (ADHD) with OROS methylphenidate: the role of baseline ADHD severity and treatment response. *J Clin Psychiatry.* 2013;74(10):983-990.
- Post hoc analysis in: Heffner JL, Lewis DF, Winhusen TM. Osmotic release oral system methylphenidate prevents weight gain during a smoking-cessation attempt in adults with ADHD. *Nicotine Tob Res: official journal of the Society for Research on Nicotine and Tobacco.* 2013;15(2):583-587.
- Post hoc analysis in: Heffner JL, Lewis DF, Winhusen TM. Preliminary evidence that adherence to counseling mediates the effects of pretreatment self-efficacy and motivation on outcome of a cessation attempt in smokers with ADHD. *Nicotine Tob Res: official journal of the Society for Research on Nicotine and Tobacco.* 2013;15(2):393-400.
- Secondary analysis in: Luo, S.X., Covey, L., Hu, M.C., Levin, F.R., & Nunes, E.V. (2013). Predictive modeling and nonlinear treatment effects in a multicenter, randomized controlled trial of methylphenidate in smoke cessation intervention. *Am J Addict.* 22 (3), 305.
- Secondary analysis in: Luo SX, Covey LS, Hu MC, Levin FR, Nunes EV, Winhusen TM. Toward personalized smoking-cessation treatment: Using a predictive modeling approach to guide decisions regarding stimulant medication treatment of attention-deficit/hyperactivity disorder (ADHD) in smokers. *Am J Addict.* 2015;24(4):348-356.

- Luo SX, Wall MM, Covey LS, et al. Modeling potential mechanisms of differential treatment effects in osmotic-release methylphenidate for smoking cessation. *Drug Alcohol Depend.* 2015;146:e188.
- https://clinicaltrials.gov/ct2/show/NCT00253747
- Additional information from study authors

133. Young2011, B4Z-US-LYCW, NCT00190775

- Young JL, Sarkis E, Qiao M, Wietecha L. Once-daily treatment with atomoxetine in adults with attention-deficit/hyperactivity disorder: a 24-week, randomized, double-blind, placebo-controlled trial. *Clin Neuropharmacol.* 2011;34(2):51-60.
- Pooled in: Wietecha L, Young J, Ruff D, Dunn D, Findling RL, Saylor K. Atomoxetine once daily for 24 weeks in adults with attention-deficit/hyperactivity disorder (ADHD): impact of treatment on family functioning. *Clin Neuropharmacol.* 2012;35(3):125-133.
- Wietecha LA, Clemow DB, Buchanan AS, Young JL, Sarkis EH, Findling RL. Atomoxetine Increased Effect over Time in Adults with Attention-Deficit/Hyperactivity Disorder Treated for up to 6 Months: Pooled Analysis of Two Double-Blind, Placebo-Controlled, Randomized Trials. *CNS Neurosci Ther.* 2016;22(7):546-557.
- https://clinicaltrials.gov/ct2/show/NCT00190775 (other ID: B4Z-US-LYCW(9043)
- Full CSR provided by Lilly

Table S1. Scales/subscales for children/adolescents considered for possible inclusion

Note: If ADHD core symptoms were measured only in subscales, rather than in the total scale, data from the subscale were requested to study authors when not reported in the study report; if not available, the study was discarded from the analysis of efficacy outcomes.

Questionnaire/scale	Abbreviation	Sub-scales	Scales/subscales on ADHD core symptoms considered for the present meta-analysis
ADHD Rating Scale (including parent and teacher version)	ADHD-RS	Inattention; Impulsivity/hyperactivity; Total	Total; if not available, inattention and/or impulsivity/hyperactivity
ADHD Symptoms Rating Scale	ADHD-SRS	Inattention; Impulsivity/hyperactivity; Total	Total; if not available, inattention and/or impulsivity/hyperactivity
Swanson, Nolan, and Pelham-IV (teaching and parent rating scales), 90 items	SNAP-IV	Inattention; Hyperactivity/impulsivity; Oppositional Defiant Disorder; Inattention/overactivity; Aggression/defiance; Conners Index	Inattention/overactivity; if not available, inattention and/or impulsivity/hyperactivity
Swanson, Nolan, and Pelham-IV, 26 items	SNAP-IV, 26-item	Inattention; Hyperactivity/impulsivity; Oppositional Defiant Disorder (ODD); Total	Inattention and/or impulsivity/hyperactivity
Swanson, Nolan, and Pelham-IV, 18 items	SNAP-IV, 18-item	Inattention; Hyperactivity/impulsivity; Combined	Combined; if not available: Inattention and/or impulsivity/hyperactivity
Conners' Parent Rating Scale-Revised, long version and Conners' Teacher Rating Scale-Revised, long version	CPRS-R:L and CTRS-R:L	Oppositional; Cognitive Problems/Inattention; Hyperactivity; Anxious-Shy; Perfectionism; Social Problems; Psychosomatic (for parent version); ADHD Index; Conners' Global Index (CGI) restless-impulsive; Conners' Global Index (CGI), emotional lability; Conners' Global Index (CGI), total; DSM-IV inattentive, DSM-IV Hyperactive Impulsive, DSM IV total	ADHD index, DSM- IV total; if not available, cognitive problems/inattention and/or hyperactivity or DSM-IV inattentive, DSM-IV and/or Hyperactive Impulsive
Conners' Parent Rating Scale-Revised, short version and Conners' Teacher Rating Scale-Revised, short version	CPRS-R:S and CTRS-R:S	Oppositional Cognitive Problems Hyperactive-Impulsive ADHD Index	ADHD index; if not available: cognitive problems and/or Hyperactive-Impulsive
Conners'- Wells' Adolescent Self Report Scale, long version	CASS-L	Family Problems Conduct Problems Anger Control Problems Emotional Problems	ADHD index; if not available: cognitive problems and/or Hyperactive-Impulsive

		Cognitive Problems<br>Hyperactive-Impulsive<br>DSM-IV Symptoms<br>ADHD Index	
Conners'- Wells' Adolescent Self Report Scale, short version	CASS-S	Conduct<br>Cognitive<br>Hyperactivity<br>ADHD index	ADHD index; if not available: cognitive and/or Hyperactivity
Conners 3- Parent, parent and teachers	Conners 3-P and Conners 3-T	Inattention; Hyperactivity-Impulsivity; Learning problems; executive function; aggression; peer relations	Inattention and/or impulsivity-hyperactivity
IOWA Conners Parent Rating Scale and IOWA Conners Teacher Rating Scale; adolescent form also available	IOWA CPRS and IOWA CTRS	Inattentive/overactive<br>Oppositional-defiant; Total	Inattentive/overactive
Swanson, Kotkin, Atkins, M-Flynn, Pelham Scale	SKAMP	Inattention, deportment (behavior); Total (combined)	Total; if not available, inattention and/or deportment
Vanderbilt ADHD teacher report	VADTRS	Inattention; Hyperactivity/ impulsivity; ADHD combined; Oppositional Defiant/Conduct; Anxiety Depression; Academic performance; Classroom behavioral performance.	ADHD combined; if not available, inattention and/or Hyperactivity/ impulsivity
Vanderbilt ADHD parent report	VADPRS	Inattention; Hyperactivity/ impulsivity; ADHD combined; Oppositional Defiant/Conduct; Anxiety Depression	ADHD combined; if not available, inattention and/or Hyperactivity/ impulsivity
Strengths and Weaknesses of ADHD Symptoms and Normal Behavior Scale (SWAN), teacher and parent version	SWAN	Inattention, Hyperactivity-Impulsivity, ADHD, oppositional defiant disorder (ODD)	ADHD; if not available, inattention and/or Hyperactivity-impulsivity
Attention Deficit Disorder Evaluation Scale-Second Edition, parent (home) and teacher version, (short version)	ADDES-1 (S)	Inattention;<br>Hyperactivity/impulsivity<br>Total	Total; if not available, inattention and/or Hyperactivity-impulsivity
ACTeRS-second edition, parent and teacher version	ACTeRS-second edition	Attention, Hyperactivity, Social Skills, Oppositional Behavior; Early childhood problems (only in parent version)	Inattention and/or Hyperactivity
ACTeRS-second edition adolescent self-report	ACTeRS-second edition	Inattention, Hyperactivity/Impulsivity, Social adjustment	Inattention and/or Hyperactivity

Plus any other scale including ADHD core symptoms

Table S2. Scales/subscales for adults considered for possible inclusion

Note: If ADHD core symptoms were measured only in subscales, rather than in the total scale, data from the subscale were requested to study authors when not reported in the study report; if not available, the study was discarded from the analysis of efficacy outcomes.

Questionnaire/scale	Abbreviation	Sub-scales	Scales/subscales on ADHD core symptoms considered for the present meta-analysis
Adult ADHD Self-Report Scale	ASRS	Inattention; Impulsivity/hyperactivity; Total	Total; if not available, inattention and/or impulsivity/hyperactivity
Adult ADHD Investigator Rating Scale	AIRS	Inattention; Impulsivity/hyperactivity; Total	Total; if not available, inattention and/or impulsivity/hyperactivity
Conners' Adult ADHD Rating Scale, self and observer, long version	CAARS-S:L and CAARS-O:L	Factor-Derived Subscales<br>Inattention/Memory Problems<br>Hyperactivity/Restlessness<br>Impulsivity/Emotional Lability<br>Problems with Self-Concept<br>DSM-IV™ ADHD Subscales<br>DSM-IV Inattentive Symptoms<br>DSM-IV Hyperactive-Impulsive Symptoms<br>DSM-IV Total ADHD Symptoms<br>ADHD index<br>Inconsistency index	In order of preference: DSM-IV Total ADHD Symptoms, ADHD index; if not available, Inattentive Symptoms DSM-IV and/or Hyperactive-Impulsive Symptoms
Conners' Adult ADHD Rating Scale, self and observer, short version	CAARS-S:S and CAARS-O:S	Factor-Derived Subscales<br>Inattention/Memory Problems<br>Hyperactivity/Restlessness<br>Impulsivity/Emotional Lability<br>Problems with Self-Concept<br>DSM-IV™ ADHD Subscales<br>DSM-IV Inattentive Symptoms<br>DSM-IV Hyperactive-Impulsive Symptoms<br>DSM-IV Total ADHD Symptoms<br>ADHD index<br>Inconsistency index	DSM-IV Total ADHD Symptoms, ADHD index; if not available, DSM-IV Inattentive Symptoms DSM-IV and/or Hyperactive-Impulsive Symptoms
ADHD raring scale with adult prompt	ADHD-RS with adult prompts	Inattention; Impulsivity/hyperactivity; Total	Total; if not available, inattention and/or impulsivity/hyperactivity
Wender-Reimherr Adult Attention Deficit Disorder Scale	WRAADS	attention difficulties<br>hyperactivity/restlessness<br>temper<br>affective lability<br>emotional over-reactivity<br>disorganization<br>impulsivity	Attention difficulties and/or hyperactivity/restlessness and/or impulsivity

Barkley Adult ADHD Rating Scale	BAARS-IV	Current inattention, current hyperactive-impulsive, current total ADHD, current sluggish cognitive tempo, child inattention, child hyperactive-impulsive, child total ADHD	Current total ADHD; if not available, current inattention and/or current impulsivity/hyperactivity

Plus any other scale including ADHD core symptoms

Table S3. Maximum FDA licensed doses or maximum doses recommended in guidelines/formularies for children/adolescents

Drug	FDA licensed maximum daily dose as reported in: http://www.accessdata.fda.gov/	Maximum daily doses as suggested in guidelines/formularies
Methylphenidate hydrochloride immediate release	60 mg	AACAP: > 50 Kg: 100 mg<br>CADDRA: 60 mg<br>BNF: 90 mg; 2.1 mg/Kg<br>Australian guidelines: 60 mg
Methylphenidate hydrochloride intermediate acting	60 mg	AACAP: > 50 Kg: 100 mg<br>CADDRA: children: 60 mg; adolescents: 80 mg<br>BNF: 90 mg<br>Australian guidelines: 60 mg
Methylphenidate hydrochloride long acting (OROS)	54 mg (children 6-12 y)<br>72 mg (adolescents 13-17 y)<br>(do not exceed 2 mg/Kg/day)	AACAP: 108 mg<br>CADDRA: children: 72 mg; adolescents: 90 mg<br>BNF: 108 mg<br>Australian guidelines: 54 mg (children and adolescents)
Methylphenidate hydrochloride oral solution	60 mg	
Methylphenidate hydrochloride chewable tablets	60 mg	
d,l-threo Methylphenidate ER	60 mg	AACAP: > 50 Kg: 100 mg
Dexmethylphenidate (d-threo-methylphenidate) immediate release	20 mg	AACAP: 50 mg
Dexmethylphenidate (d-threo-methylphenidate) ER	30 mg	AACAP: 50 mg
Dextro-amphetamine immediate release	40 mg	CADDRA: 20 mg (children); 30 mg (adolescents)<br>BNF: 40 mg<br>Australian guidelines: 40 mg
Dextro-amphetamine SR Spansule	40 mg	AACAP: > 50 Kg: 60 mg<br>CADDRA: 30 mg
Mixed Amphetamine Salts	40 mg	AACAP: > 50 Kg: 60 mg
Mixed Amphetamine Salts XR	30 mg (children)<br>20 mg (adolescents)	AACAP: > 50 Kg: 60 mg<br>CADDRA: 30 mg (children); 50 mg (adolescents)
Methamphetamine	25 mg	
Lisdexamfetamine	70 mg	CADDRA: 60 mg (children); 70 mg (adolescents)<br>BNF: 70 mg
Atomoxetine	Children and adolescents up to	

Drug	FDA licensed maximum daily dose<br>as reported in:<br>http://www.accessdata.fda.gov/	Maximum daily doses as suggested in guidelines/formularies
	70 kg: 1.4 mg/Kg, up to 100 mg; Children and adolescents over 70 kg: 100 mg	AACAP: lesser of 1.8 mg/kg or 100 mg<br>CADDRA; lesser of 1.4 mg/kg or 60 mg (children) or 100 mg (adolescents)<br>BNF: 120 mg<br>Australian guidelines: children: 1.4 mg/Kg or 100 mg; adolescents: 100 mg
Clonidine immediate release	NOT FDA LICENSED	
Clonidine extended release	0.4 mg	
Guanfacine immediate release	NOT FDA LICENSED	
Guanfacine extended release	4 mg	CADDRA: 4 mg<br>BNF: 7 mg (adolescents)
Bupropion IR	NOT FDA LICENSED	
Bupropion SR	NOT FDA LICENSED	
Bupropion XL	NOT FDA LICENSED	
Modafinil	NOT FDA LICENSED	

Most commonly used guidelines/formularies referred to in the table (in alphabetical order):
AACAP: Practice parameter of the American Academy of Child and Adolescent Psychiatry [45]
Australian formulary [46]
BNF: British National Formulary [47]
CADDRA: Guidelines of the Canadian ADHD Resource Alliance[48]

Table S4. Maximum FDA licensed doses or maximum doses recommended in guidelines/formularies for adults

Drug	FDA max daily dose as reported in: http://www.accessdata.fda.gov/	Maximum daily doses as suggested in guidelines/formularies
Methylphenidate hydrochloride immediate release	60 mg	BNF: 100 mg<br>CADDRA: 100 mg
Methylphenidate hydrochloride intermediate acting	60 mg	80 mg<br>Communication form Novartis: “This is the max. daily dose in all European countries where the adult indication is registered (Austria, Denmark, Finland, Germany, Hungary, Iceland, Ireland, Norway, Portugal & Sweden). Please refer to the Irish SmPC (see section 4.2, Adults)”<br>BNF: 100 mg<br>CADDRA: 100 mg<br>Australian guidelines: 80 mg
Methylphenidate hydrochloride long acting	72 mg	BNF: 108 mg<br>CADDRA: 108 mg
Methylphenidate hydrochloride oral solution	60 mg	
Methylphenidate hydrochloride chewable tablets	60 mg	
d,l-threo Methylphenidate slow release	60 mg	
Dexmethylphenidate (d-threo-methylphenidate) immediate release	NOT FDA LICENSED	
Dexmethylphenidate (d-threo-methylphenidate) ER	40 mg	
Dextro-amphetamine immediate release	NOT FDA LICENSED	BNF: 60 mg<br>CADDRA: 50 mg
Dextro-amphetamine ER	NOT FDA LICENSED	CADDRA: 50 mg
Mixed Amphetamine Salts	40 mg	
Mixed Amphetamine Salts ER	20 mg	CADDRA: 50 mg
Lisdexamfetamine	70 mg	CADDRA: 70 mg
Atomoxetine	100 mg	BNF: 120 mg<br>CADDRA: lesser of 1.4 mg/Kg or 100 mg<br>Australian guidelines: 100 mg
Clonidine immediate release	NOT FDA LICENSED	
Clonidine extended release	NOT FDA LICENSED	
Guanfacine immediate release	NOT FDA LICENSED	
Guanfacine extended release	NOT FDA LICENSED	
Bupropion IR	NOT FDA LICENSED	
Bupropion SR	NOT FDA LICENSED	
Bupropion XL	NOT FDA LICENSED	
Modafinil	NOT FDA LICENSED	

Most commonly used guidelines/formularies referred to in the table (in alphabetical order):
Australian formulary [46]
BNF: British National Formulary [47]
CADDRA: Guidelines of the Canadian ADHD Resource Alliance[48]

Table S5. Washout periods

Drug	Washout
Methylphenidate	1 day
Amphetamine derivatives	3-5 days
Lisdexamfetamine dimesylate	2-3 days
Atomoxetine	1 day
Clonidine	3 days
Guanfacine	3-4 days
Bupropion	2-4 days
Modafinil	3-4 days

These washout periods were established according to the UK National Institute from Clinical Care and Excellent (NICE) committee for the Guidelines on ADHD

Table S6. Starting doses in children/adolescents

Drug	Min. daily dose
Methylphenidate hydrochloride immediate release	10 mg
Methylphenidate hydrochloride intermediate acting	20 mg
Methylphenidate hydrochloride long acting	18 mg
Methylphenidate hydrochloride oral solution	10 mg
Methylphenidate hydrochloride chewable tablets	10 mg
d,l-threo Methylphenidate slow release	20 mg
Dexmethylphenidate (d-threo-methylphenidate) immediate release	5 mg
Dexmethylphenidate (d-threo-methylphenidate) XR	5 mg (children)
Dextro-amphetamine immediate release	2.5 mg (children 3-5 y)<br>5 mg (children ≥ 6 y)
Dextro-amphetamine ER	10 mg (children 6-12 y and adolescents 13-17 y)
Mixed Amphetamine Salts	2.5 mg (children 3-5 y)<br>5 mg (children ≥ 6 y)
Mixed Amphetamine Salts XR	10 mg (children ≥ 6 y)<br>10 mg, increased to 20 mg (adolescents 13-17 y)
Lisdexamfetamine	30 mg (individuals ≥ 6 y)
Atomoxetine	0.5 mg/Kg (children and adolescents ≤ 70 Kg)<br>40 mg (children and adolescents > 70 Kg and adults)
Clonidine immediate release	Children/adolescents < 45 Kg: 0.05 mg<br>Children/adolescents > 45 Kg: 0.1 mg
Clonidine extended release	0.1 mg
Guanfacine immediate release	0.5 mg
Guanfacine extended release	1 mg
Bupropion IR	N/S
Buproprion SR	N/S
Bupropion XL	N/S
Modafinil	200 mg

Table S7. Starting doses in adults

Drug	Min. daily dose
Methylphenidate hydrochloride immediate release	10 mg
Methylphenidate hydrochloride intermediate acting	20 mg
Methylphenidate hydrochloride long acting	18-36 mg
Methylphenidate hydrochloride oral solution	10 mg
Methylphenidate hydrochloride chewable tablets	10 mg
d,l-threo Methylphenidate slow release	20 mg
Dexmethylphenidate (d-threo-methylphenidate) immediate release	5 mg
Dexmethylphenidate (d-threo-methylphenidate) XR	10 mg (adult)
Dextro-amphetamine immediate release	N/S
Dextro-amphetamine ER	20 mg
Mixed Amphetamine Salts	N/S
Mixed Amphetamine Salts XR	20 mg
Lisdexamfetamine	30 mg
Atomoxetine	N/S
Clonidine immediate release	N/S
Clonidine extended release	0.1 mg
Guanfacine immediate release	0.5 mg
Guanfacine extended release	1 mg
Bupropion IR	100 mg bid
Buproprion SR	150 mg qam
Bupropion XL	150 mg qam
Modafinil	200 mg

Table S8. Inclusion/exclusion criteria for each study included in the network meta-analysis

Study name	Inclusion criteria	Exclusion criteria
**Abikoff2007**	Meeting DSM-IV criteria for ADHD (Combined or Inattentive type), based on the Diagnostic Interview Schedule for Children IV (DISC-IV)-Parent version and corroborated via clinical interview; meeting dimensional criteria for ADHD symptom severity on the Conners Teacher Rating Scale-Revised, long-form, defined as a score at least 1.5 SD above age and sex norms on the DSM-IV Hyperactive/Impulsive scale (for children diagnosed as Combined type) or on the DSM-IV Inattentive scale (for children diagnosed as Inattentive type); impaired OTMP functioning, defined by a mean Total score at least 1 SD below the norm on the COSS-T or COSS-P; and a score of at least 80 on the Wechsler Abbreviated Scale of Intelligence.	Diagnosis of autism, major depression, substance abuse, obsessive-compulsive disorder, post- traumatic stress disorder, panic disorder, tic disorders, significant suicidality, or a lifetime history of psychosis or mania. Any exclusionary diagnoses noted on the DISC-IV-Parent version had to be confirmed by the clinical interview. Youngsters were also excluded if they had a learning disability according to a school individualized educational plan or were taking other CNS medications.
**Adler2008a**<br>**B4Z-MC-LYBV**<br>**NCT00190931**	Aged 18 to 50 years old, meet criteria for current ADHD and a historical childhood diagnosis of ADHD according to the *Diagnostic and Statistical Manual of Mental Disorders*, a severity of illness of at least 4 (moderate) on the Clinician Global Impressions Severity Scale and be employed for at least 20 hours per week for 6 months prior to study entry.	Diagnosis of current major depression, an anxiety disorder (including generalized anxiety disorder, panic disorder, or social phobia), any current alcohol or sub- stance abuse, or any lifetime history of bipolar illness or psychotic disorder. They were also excluded if they had any medical illness that would contraindicate the use of atomoxetine, current or past hypertension, and any history of organic brain disease or seizures other than febrile. Participants were free of all psychotropic medications for at least 1 week prior to randomization.
**Adler2008b**<br>**NRP104.303**<br>**NCT00334880**	Primary diagnosis of ADHD by *Diagnostic and Statistical Manual of Mental Disorders*, Fourth Edition, Text Revision (DSM- IV-TR) criteria. ADHD diagnosis was based on a comprehensive psychiatric interview that included the Adult ADHD Clinical Diagnostic Scale. All subjects were required to meet at least 6 of the 9 DSM-IV-TR subtype criteria and to have moderate to severe ADHD as rated by a clinician at baseline (ADHD-RS scores ≥ 28). Other inclusion criteria included 12-lead electrocardiogram (ECG) with QT/QTc-F interval < 450 ms for men and < 470 ms for women, resting heart rate 40 to 100 bpm, PR interval < 200 ms, and QRS interval < 110 ms.	comorbid psychiatric diagnosis with significant symptoms that, in the judgment of the investigator, might preclude treatment with lisdexamfetamine; history of seizures; taking medications that affect the central nervous system or blood pressure (excluding current ADHD medications, which were washed out); known cardiac structural abnormality or any other condition that might affect cardiac performance; clinically significant ECG or laboratory abnormality at screening or baseline; history of hypertension, or a resting sitting systolic blood pressure (SBP) > 139 mm Hg or diastolic blood pressure (DBP) > 89 mm Hg; pregnancy or lactation; and positive urine drug results at screening or baseline (except for subject's current stimulant therapy). Women of child- bearing potential had to comply with contraceptive restrictions (negative pregnancy test, double-barrier or hormonal contraceptives, or abstinence from sexual activity).

Study name	Inclusion criteria	Exclusion criteria
**Adler2009a B4Z-US-LYDQ NCT00190879**	DSM-IV-TR diagnoses for both ADHD and social anxiety disorder, were enrolled. The diagnostic criteria for ADHD were assessed with the Conners' Adult ADHD Diagnostic Interview for DSM-IV and for social anxiety disorder by the Structured Clinical Interview for DSM-IV- TR Axis I Disorders-Research Version. Additionally, patients had an LSAS Total score of at least 50 at Visit 1, no more than a 30% decrease in LSAS Total score at Visit 2, and a Clinical Global Impression-Overall-Severity (CGI-O-S) score of 4 or greater at Visits 1 and 2. Concomitant Axis I diagnoses (current or lifetime)-specific phobias, Generalized Anxiety Disorder (GAD), and dysthymia were allowed. Current diagnosis of major depressive disorder was allowed only if diagnosed more than 6 months before Visit 1.	Current or lifetime diagnosis of obsessive–compulsive disorder, bipolar affective disorder, psychosis, factitious disorder, or somatoform disorders, and/or current diagnosis of panic disorder, posttraumatic stress disorder, or an eating disorder within the year preceding Visit 1. Current diagnosis of alcohol, drugs of abuse, or prescription medication abuse meeting DSM-IV-TR criteria were also excluded.
**Adler2009b B4Z-US-LYCU NCT00190736**	Adults, aged 18 to 54 years, who met DSM-IV, Text Revision (DSM-IV-TR) criteria for adult ADHD as assessed by the Adult ADHD Clinician Diagnostic Scale version 1.2, had a Clinical Global Impressions ADHD Severity of Illness (CGI- ADHD-S) score of 4 (moderate symptoms) or higher, had AISRS Symptom Checklist scores that did not change by more than 25% between visits 1 and 2, and had impairment due to ADHD symptoms in the home setting as indicated in the diagnostic interview were eligible to participate.	Diagnostic criteria for current major depression, a current anxiety disorder, any history of bipolar disorder, or any history of a psychotic disorder. Failure to respond to an adequate trial of treatment with ADHD stimulant medication, bupropion, or other nonstimulant medications (based upon the clinician's judgment) was also exclusionary. Patients were recruited during routine office visits for ADHD, by referral, and by advertisement.

Study name	Inclusion criteria	Exclusion criteria
**Adler2009c CR011560NCT00 326391**	Adults between 18 to 65 years of age (inclusive) with ADHD and weighed a minimum of 100 lb (45.4 kg). At subject screening, the diagnosis of ADHD inattentive, hyperactive/impulsive, or combined type as defined by the Diagnostic and Statistical Manual of Mental Disorders, Fourth Edition (DSM-IV) criteria was established through clinical evaluation by the investigator. The subject must have described a chronic course of ADHD symptoms from childhood to adulthood, have had an AISRS score of 24 or greater, and have had a global assessment of functioning score of between 41 and 60 (inclusive), indicating moderate or serious symptoms (according to DSM-IV criteria). Previous formal diagnosis of and/or treatment of ADHD were not required. Diagnosis of ADHD was confirmed by using the Adult ADHD Clinical Diagnostic Scale version 1.2 at baseline.	The Hamilton Anxiety Rating Scale (HAM-A) and the Hamilton Depression Rating Scale (HAM-D) were administered to assess possible symptoms of anxiety and depression, and subjects with symptoms of marked anxiety, tension, agitation, or a HAM-A score of 21 or greater or with symptoms of moderate severity of depression ratings using a HAM-D score of 17 or higher were excluded. The patients who met the DSM- IV criteria for depressive or anxiety disorders were excluded from the study, even if their HAM scores did not reach these cutoffs. Known nonresponders to methylphenidate were also excluded, as were subjects with a history of allergy to methylphenidate; any coexisting medical condition or taking any medication that was likely to interfere with the safe administration of methylphenidate; known or suspected structural cardiac abnormality as assessed by history, physical examination, or electrocardiogram (ECG); diagnosis or family history of Tourette syndrome or motor or verbal tics; or history of seizure disorder, uncontrolled hyperthyroidism, or hypothyroidism. Patients with comorbid psychiatric diagnosis per DSM-IV criteria of bipolar disorder, cyclothymic disorder, schizophrenia, pervasive developmental disorder, severe obsessive-compulsive disorders, or any other diagnosis that in the judgment of the investigator could have deemed the subject to be inappropriate for the study were excluded. Subjects with a history of drug or alcohol abuse within the past 6 months or with suicidal ideation or behavior during the past year were also excluded, as were subjects with a current or history of an eating disorder for the last 3 years. Patients taking antipsychotic medication, bupropion, modafinil, clonidine or other alpha-2 adrenergic receptor agonists, tricyclic antidepressants, theophylline, coumarin anticoagulants, anticonvulsants, monoamine oxidase inhibitors, guanethidine, or a serotonin norepinephrine reuptake inhibitor (eg, venlafaxine and duloxetine) were excluded from the study.Patients taking a selective serotonin reuptake inhibitor (eg, fluoxetine, paroxetine, sertraline, citalopram, or escitalopram) who were not stable on their medication for at least 30 days

Study name	Inclusion criteria	Exclusion criteria
**Adler2013<br>SPD489-403<br>NCT01101022**	Adults aged 18–55 years who met full *DSM-IV-TR* criteria for a primary diagnosis of ADHD were eligible. Participants were required to be in a close domicile relationship (eg, spouse or significant other) for ≥ 6 months prior to screening to ensure the availability of an informant who was willing to report on the participant's behavior and symptoms. Additional inclusion criteria included a baseline BRIEF-A Global Executive Composite (GEC) T-score ≥ 65, indicating clinically significant executive function impairment at baseline, and a baseline total score ≥ 28 on the ADHD-RS-IV with adult prompts.	Adults with comorbid psychiatric conditions that were controlled with a prohibited medication or were uncontrolled and associated with significant symptoms, including severe Axis I or II disorders, were excluded from the study. Other key exclusion criteria included cardiovascular disease, which may increase vulnerability to the sympathomimetic effects of a psychostimulant; a history of moderate to severe hypertension; ADHD that was well controlled on current ADHD therapy; and a history of failure to respond to an adequate course of amphetamine therapy.
**Allen2005<br>B4Z-MC-LYAS**	All study subjects met the Diagnostic and Statistical Manual of Mental Disorders (DSM-IV) criteria for ADHD and had concurrent Tourette syndrome or chronic motor tic disorder, as diagnosed by clinical interview and examination by the investigator and confirmed by the Schedule for Affective Disorders and Schizophrenia for School-age Children–Present and Lifetime Version (K-SADS-PL). Subjects' scores on the Attention-Deficit/Hyperactivity Disorder Rating Scale-IV-Parent Version: Investigator Administered and Scored (ADHDRS-IV-Parent:Inv) had to be at least 1.5 standard deviations above the age and sex norm for diagnostic subtype (predominantly inattentive or predominantly hyperactive–impulsive), or for the total score for the combined subtype (if DSM-IV criteria were met for the combined subtype), using published norms for the ADHDRS-IV-Parent:Inv at Visits 1 (enrollment) and 2 (randomization). Subjects' Yale Global Tic Severity Scale (YGTSS) total scores had to be at least 5 at both Visits 1 and 2.	Exclusion criteria included a Children's Yale–Brown Obsessive–Compulsive Scale (C-YBOCS) total score 15 or diagnosis of obsessive-compulsive disorder severe enough, in the investigator's opinion, to require pharmacotherapy; a Children's Depression Rating Scale–Revised (CDRS-R) total score 40 or diagnosis of depression severe enough to require pharmacotherapy; a history of bipolar disorder or psychosis; seizure disorder; or current use of any psychotropic medication other than study drug.
**Amiri2008**	Participants between the ages of 6–15 who clearly met the DSM-IV-TR diagnostic criteria for ADHD. Additional inclusion criteria included total and/or subscale scores on Attention-Deficit/Hyperactivity Disorder Rating Scale IV (ADHD-RS-IV) School Version at least 1.5 standard deviations above norms for patient's age and gender.	Children were excluded if they had a history or current diagnosis of pervasive developmental disorders, schizophrenia or other psychiatric disorders (DSM-IV axis I); any current psychiatric comorbidity that required pharmacotherapy; any evidence of suicide risk and mental retardation (I.Q.< 70 based on clinical judgment). In addition, patients were excluded if they had a clinically significant chronic medical condition, including organic brain disorder, seizures and, current abuse or dependence on drugs within 6 months. Additional exclusion criteria were hypertension, hypotension and habitual consumption of more than 250 mg/day of caffeine.

Study name	Inclusion criteria	Exclusion criteria
**Arnold2006**	Children/adolescents ages 5 to 15 years with mental age > 18 months who had an ASD and symptoms of ADHD. They met the first four of five DSM-IV criteria for ADHD: symptom count, impairment, chronicity, and pervasiveness across settings (the fifth criterion would technically rule out ADHD by the presence of PDD) and had to have a parent-rated symptom mean Q1.5 on either the nine inattentive or the nine hyperactive-impulsive ADHD symptoms, rated 0 to 3.	Exclusion criteria included cardiovascular disease, glaucoma, unstable seizure disorder, other significant physical illness, psychosis, severe mood disorder, substance abuse, or pregnancy.
**Arnold2014 C1538/2027/AD/ US NCT00315276**	Patients were included in the study if they met the full *Diagnostic and Statistical Manual of Mental Disorders* criteria for ADHD (combined type, predominantly inattentive subtype, or predominantly hyperactive-impulsive subtype), for which symptoms were present before the age of 7 years and persisted for at least the prior 6 months, according to a psychiatric/clinical evaluation using the Adult ADHD Clinical Diagnostic Scale (ACDS). Eligible patients were also required to have a Hamilton Anxiety Scale (HAM-A) and Hamilton Depression Scale score <15, and an AISRS total score of >24 at the screening and baseline visits, with a difference in the AISRS total score from screening to baseline <25%. In addition, a CGI Severity of Illness rating for ADHD of ≥4 at the baseline visit was required for study entry. Women of childbearing potential were required to use a medically accepted method of contraception during the study and for 30 days following their participation.	Exclusion criteria included a history or current diagnosis of schizophrenia, bipolar disorder, or other psychotic disorders; suicidal ideation, history of suicide attempt, or a clinical assessment of suicide risk; any acute psychiatric comorbidity (including but not limited to depression or other mood or anxiety disorder) that required pharmacotherapy, as determined by the Structured Clinical Interview for *DSM-IV-TR* (SCID) module assessment; a clinically significant sleep disorder; being intellectually challenged, as determined by the investigator; being satisfied with his or her current ADHD medication and having no unacceptable side effects; previous use of modafinil; use of other prescription medications for ADHD with psychoactive properties as of the baseline visit; drug or alcohol dependence within the prior 6 months; use of any antidepressant within 2 weeks before baseline; being pregnant or lactating; and presence of any clinically significant uncontrolled medical conditions.
**Bain2013 NCT00429091**	Adult male and female patients (aged 18–60 years) met the DSM-IV-TR criteria for ADHD, confirmed by the Adult ADHD Clinical Diagnostic Scale V 1.2 at Screening. Eligible individuals also demonstrated scores X2 (pretty much, often) on at least 6 of 9 items on the Inattentive score or the Hyperactive/Impulsive score of the Conners' Adult Rating Scale–Investigator Rated Scale (CAARS:Inv), a total CAARS:Inv score of X20, and a Clinical Global Impression-ADHD Severity (CGI-ADHD-S) score of moderate or more impairment (X4) at Screening and Baseline.	Any history of lifetime psychotic disorder, bipolar disorder, obsessive-compulsive disorder, or mental retardation; current generalized anxiety disorder, post-traumatic stress disorder, sleep disorder requiring treatment, or a current major depressive episode; any unstable medical condition; any condition that could affect cognitive performance; or if they were a pregnant or lactating female. Excluded psychotropic medication included anxiolytics, antipsychotics, anti-depressants, mood stabilizers, nicotine replacement therapies, or varenicline. The use of atomoxetine was prohibited within 3 months before screening, and subjects receiving psychostimulants required a 7-day washout before randomization. Because of the potential of past or present nicotine use to influence the response to a nicotinic receptor agonist, participants were queried about their tobacco use. Individual

Study name	Inclusion criteria	Exclusion criteria
		study subjects were designated as nontobacco user, current tobacco user, or ex-tobacco user based on each subject's self-identification as such. Tobacco use was defined as the use of cigarettes, pipes, cigars, or chewing tobacco. Current tobacco users were allowed to continue use during the study.
**Bangs2007 B4Z-MC-LYAX**	Adolescents aged 12–18 years who met the criteria for both ADHD and MDD per the *Diagnostic and Statistical Manual of Mental Disorders*, 4th edition (DSM–IV). For inclusion, patients were required to have a score on the ADHD Rating Scale-IV, Parent version, Investigator-administered and -scored (AD-HDRS-IV-Parent:Inv) at least 1.5 standard deviations (SD) above age and sex norms and a Children's Depression Rating Scale–Revised (CDRS-R) total score of 40 at every visit prior to randomization (visit 4).	Patients beginning structured psychotherapy for ADHD and/or depression less than 1 month before trial entry were excluded. Directly affiliated with the conduct of this study, or are immediate family of someone directly affiliated with the conduct of this study. Have received treatment within the last 30 days with a drug that has not received regulatory approval for any indication at the time of study entry. Patients who weigh less than 33 kg or greater than 90 kg at study entry. Patients who have a documented history of Bipolar I or II disorder, any history of psychosis or pervasive development disorder. Patients with a history of any seizure disorder (other than febrile seizures) or patients who have taken (or are currently taking) anticonvulsants for seizure control are not eligible to participate. Patients at serious suicidal risk as assessed by the investigator. Patients with a history of severe allergies to more than 1 class of medications or multiple adverse drug reactions. Patients taking any psychotropic medication on a regular basis, including health-food supplements that the investigator feels have central nervous system activity (for example, St. John's Wort, melatonin), must have a washout equal to a minimum of 5 half-lives of that medication prior to Visit 2. Patients with a history of alcohol or drug abuse on repeated basis within the past 3 months are excluded. Patients who screen positive at study entry for drugs of abuse not prescribed by a physician are excluded from the study, unless, as described above, the screen is positive for marijuana or another cannabinoid. Patients with significant prior or current medical conditions (for example surgically corrected congenital heart defects). Patients who have any medical condition that would increase sympathetic nervous system activity markedly. Use of monoamine oxidase inhibitors (MAOIs) during the 2 weeks (14 days) prior to Visit 2. Current or past history of hypertension. Patients who have participated in a prior clinical study of atomoxetine or used an investigational drug within the previous 30 days. Patients who, in the opinion of the investigator, are unsuitable in any other way to participate in this study. Patients who at any time during the study

Study name	Inclusion criteria	Exclusion criteria
		begin a structured psychotherapy aimed at ADHD and/or depression symptoms are excluded. Psychotherapy initiated at least 1 month prior to study participation can continue during the study.
**Bangs2008 B4Z-MC-LYBX NCT00191698**	Patients were aged 6 to 12 years and met *Diagnostic and Statistical Manual of Mental Disorders, Fourth Edition* (DSM- IV), diagnostic criteria for ADHD (any subtype) and co- morbid ODD as determined by an investigator's clinical assessment; a structured interview (Kiddie Schedule for Affective Disorders and Schizophrenia for School Aged Children-Present and Lifetime Version); Swanson, Nolan, and Pelham Rating Scale-Revised (SNAP-IV) ADHD subscale score above age and gender norms; Clinical Global Impressions-Severity Scale score 4 at visits 1 and 2; and SNAP-IV ODD subscale score of 15 at both visits 1 and 2. If other comorbid conditions were present, either ADHD or ODD was the primary diagnosis.	Patients who had a history of bipolar I or II disorder, psychosis, or pervasive developmental disorder were excluded. Patients also were excluded if they had a current diagnosis of major depressive disorder, posttraumatic stress disorder, a Children's Depression Rating Scale-Revised total raw score 40 at visit 1, or if they were determined to be at serious suicidal risk. Patients with a history of any seizure disorder (other than febrile seizures), a history of alcohol or drug abuse within the past 3 months, current cardiovascular disease or other conditions that could be aggravated by an increased heart rate or increased blood pressure, a medical condition that would markedly increase sympathetic nervous system activity, or severe gastrointestinal narrowing were excluded. Finally, patients who, in the investigator's judgment, were likely to need psychotropic medications apart from the drug under study or who at any time during the study were likely to begin structured psychotherapy were excluded.
**Bedard2015 NCT00183391**	Participants, ages 6–17 years; all youth had a DSM-IV diagnosis of ADHD, any subtype. Other comorbidity was permitted provided ADHD was the primary disorder and the comorbid condition did not require medication treatment. Participants may have been previously treated with ATX or MPH, but must not have been nonresponders to an adequate trial and must not have experienced disabling adverse effects with either medication. Most participants were medication naıve (65%).	Exclusionary criteria were: WISC-IV full-scale IQ below 75, non-English speaking parent or child, neurological dysfunction, systemic medical illness, uncorrected sensory impairments, and history of psychosis or bipolar disorder.
**Biederman2002 SLI381-301**	Children aged 6 to 12 years who satisfied *Diagnostic and Statistical Manual of Mental Disorders, Fourth Edition* criteria for a primary diagnosis of hyperactive-impulsive or combined subtypes of ADHD were recruited for the study. Participants were required to be in a school setting in which the same teacher was able to make assessments of both morning and afternoon behavior. The teacher had to be able to spend sufficient time with a participant to make valid assessments in both the morning and afternoon. Children were either known to be responsive to stimulants or naıve to stimulant treatment.	Participants incapable of understanding or following the instructions given in the study, known non-responders to stimulant medication, and those with a comorbid psychiatric diagnosis (psychosis, bipolar illness, pervasive) were excluded. Participants with a history of seizure (exclusive of febrile seizure), tic disorder, or a family history of Tourette's disorder, those with a documented allergy or intolerance to Adderall, and participants taking clonidine, anticonvulsant drugs, pemoline (within 30 days), medications that affect blood pressure or heart rate, steroids, or other medications that have central nervous system effects or affect performance (such as sedating antihistamines and decongestant sympathomimetics, either oral or topical) also were

Study name	Inclusion criteria	Exclusion criteria
		excluded. Other exclusion criteria included a concurrent chronic or acute illness or condition that might con- found results or increase risk to the participant, history of suspected substance abuse disorder, or living with someone with a current diagnosed substance abuse disorder.
**Biederman2005 Study311Cephalon**	Patients were 6 to 17 years of age and had a diagnosis of ADHD on the basis of criteria in the *Diagnostic and Statistical Manual of Mental Disorders, Fourth Edition* (DSM-IV) for ADHD at screening, as manifested by a psychiatric/clinical evaluation and the Diagnostic Interview Schedule for Children, Fourth Edition, with a Clinical Global Impression Severity of Illness (CGI-S) rating of 4 or higher ("moderately ill" or worse). In addition, patients were attending full-time school (ie, they were not being home-schooled); had a teacher-/investigator-rated Attention-Deficit/ Hyperactivity Disorder Rating Scale-IV (ADHD-RS-IV) School Version total and/or subscale score at least 1.5 SDs above normal values for age and gender, were between the 5th and 95th percentile for weight and height on the basis of National Center for Health Statistics guidelines, had an IQ of at least 80 as estimated by the Wechsler Intelligence Scale for Children–Third Edition, and had a score of at least 80 on the Wechsler Individual Achievement Test–Second Edition–Abbreviated.	Patients were excluded when they had a history or current diagnosis of pervasive developmental disorder, schizophrenia, or other psychotic disorders (DSM-IV Axis I); evidence of suicide risk; current psychiatric comorbidity that required pharmacotherapy; or other active clinically significant disease. To avoid potential ethical concerns, patients whose ADHD was well controlled and who were satisfied with current ADHD therapy (with low levels of side effects) were also excluded, as were those who had failed to respond to 2 or more adequate courses (dose and duration) of stimulant therapy for ADHD. Other exclusion criteria included a clinically significant drug sensitivity to stimulants, a history of alcohol or substance abuse as defined by DSM-IV criteria, consumption of 250 mg/day caffeine, absolute neutrophil count 1 10/L, hypertension (systolic blood pressure [SBP] of 122 mm Hg or diastolic blood pressure [DBP] of 78 mm Hg for patients aged 6 –9 years; SBP of 126 mm Hg or DBP of 82 mm Hg for patients aged 10 –12 years; SBP of 136 mm Hg or DBP of 86 mm Hg for patients aged 13–17 years), hypotension (sitting SBP 50 mm Hg for patients younger than 12 years or 80 mm Hg for patients 12 years and older), and resting heart rate outside the range of 60 to 115 beats per minute. Concomitant use of prescription or nonprescription agents with psychotropic properties, including ADHD treatments and dietary supplements, was prohibited within 1 week of the baseline visit (within 2 weeks for monoamine oxidase inhibitors and selective serotonin reuptake inhibitors) and during the study.
**Biederman2006a (subsampleofNCT00181571)**	Subjects were outpatient adults with ADHD aged 19–60 years. To be included, subjects had to satisfy full diagnostic criteria for DSM-IV ADHD on the basis of clinical assessment and confirmation by structured diagnostic interview. Participants with anxiety disorders and depression who were receiving a stable medication regimen for at least 3 months and who had a disorder-specific Clinical Global Impression Scale (CGI)-Severity score of 3 or less (mildly ill) were not excluded. Thus, subjects receiving stable doses of non–monoamine oxidase inhibitor antidepressants	Potential subjects were excluded if they had clinically significant chronic medical conditions, abnormal baseline laboratory values, intelligence quotient less than 80, delirium, dementia, or amnesic disorders, other clinically unstable psychiatric conditions (i.e., bipolar disorder, psychosis, suicidality), drug or alcohol abuse or dependence within the 6 months preceding the study, or a previous adequate trial of MPH. We also excluded pregnant or nursing women.

Study name	Inclusion criteria	Exclusion criteria
	or benzodiazepines for more than 3 months were eligible for this study.	
**Biederman2006b**	Children aged 6 to 13 years whose height and weight corresponded to greater than the fifth percentile in standardized growth charts and who were attending full-day kindergarten, elementary school, or middle school were eligible. Participants met complete criteria of the Diagnostic and Statistical Manual of Mental Disorders, Fourth Edition (DSM-IV), for ADHD (combined type, predominantly inattentive type, or predominantly hyperactive-impulsive type) at screening, as determined by a psychiatric/clinical evaluation and confirmed by the Diagnostic Interview Schedule for Children, Fourth Edition. Eligibility was restricted to those children who were stimulant-naive (i.e., who had not received stimulant medication in the past) or who had manifested an unsatisfactory response to stimulant therapy. At screening, an intelligence quotient (IQ) of at least 80, as estimated on the Wechsler Intelligence Scale for Children, Third Edition, and a score of 80 or higher on the screener version (for learning disabilities) of the Wechsler Individual Achievement Test were used to rule out low IQ or learning disabilities as contributing causes of symptoms and were required for inclusion. At the baseline visit, children were required to have a clinician-rated Clinical Global Impressions of Severity (CGI-S) score of 4 or more, reflecting their overall clinical condition (moderately ill or worse). For each child, availability of a parent and a weekday teacher who were willing to participate in the study was required.	Main exclusion criteria included active, clinically significant gastrointestinal, cardiovascular, hepatic, renal, hematologic, neoplastic, endocrine, neurologic, immunodeficiency, pulmonary, or other major clinically significant disorder or disease; any current psychiatric comorbidity, including but not limited to depression or other mood disorder, anxiety disorder, or pervasive mental disorder that required pharmacotherapy; use of any prescription (e.g., clonidine, guanfacine) or nonprescription medication with psychoactive properties (e.g., over-the-counter medications or dietary supplements containing ephedrine, pseudoephedrine, caffeine, or phenylpropanolamine) within 1 week of the start of the washout period; and a history or evidence of substance abuse.
**Biederman2007 NRP104-301NCT00248092**	The intention was to enrol children who were not adequately treated with their current medication for ADHD or had not previously been treated for ADHD. DSM-IV-TR criteria for ADHD. ADHD-RS score > 28. Academic function at age appropriate level. Normal blood pressure and ECG. Absence of medical conditions or treatment that could confound the results, inability to swallow capsules.	Exclusion criteria included comorbid psychiatric diagnosis (eg, psychosis, bipolar disorder), history of seizures or current diagnosis or family history of Tourette's disorder, obesity based on the investigator's opinion, weight <25 kg (55 lb), positive screening for illicit drug use, and/or current health conditions or use of medications that might confound the results of the study or increase risk to the patient. Female patients of childbearing potential were required to have a negative result on urine pregnancy testing and were given specific instructions on avoiding pregnancy throughout the period of study drug exposure.

Study name	Inclusion criteria	Exclusion criteria
**Biederman2008 SPD503-301 NCT00152009**	Patients who were aged 6 to 17 years inclusive and met *Diagnostic and Statistical Manual of Mental Disorders, Fourth Edition, Text Revision*, criteria for a primary diagnosis of ADHD combined subtype, predominantly inattentive subtype, or predominantly hyperactive-impulsive subtype were eligible to participate in the study. Patients were also required to function intellectually at age-appropriate levels; have electrocardiogram (ECG) results within the reference range; and have blood pressure (BP) measurements within the 95th percentile for their age, gender, and height.	Patients were excluded from the study when they had a current, uncontrolled, comorbid psychiatric diagnosis (except oppositional defiant disorder) with significant symptoms, such as any severe comorbid Axis II disorder or severe Axis I disorder, or when other symptomatic manifestations would, in the opinion of the examining physician, contraindicate GXR treatment or confound efficacy or safety assessments. Patients who weighed 55 lb or were morbidly overweight or obese, pregnant, lactating, or hypertensive were also excluded. In addition, patients were not enrolled when they had any of the following: a QTc interval of 440 milliseconds; a history of seizure during the past 2 years (exclusive of febrile seizures); a tic disorder; family history of Tourette's disorder; a positive urine drug screen; any abnormal thyroid function that was not adequately treated; or any cardiac condition or family history of cardiac condition that, in the opinion of the physician investigator, would require exclusion. Patients who had taken an investigational drug within 28 days, were taking medications that affect BP or heart rate, or were taking other medications that have central nervous system effects or affect performance were also not eligible to participate.
**Biederman2012 2008P000971 NCT00801229**	Subjects were both male and female outpatients, 18e26 years of age, who met full DSM-IV criteria for ADHD based on a clinical evaluation supplemented by structured diagnostic interview. Subjects had an onset of symptoms in childhood, a persistence of impairing symptoms into adulthood, and did not have pharmacological treatment for ADHD in the past month. By physician assessment, no subject was considered either stimulant refractory or stimulant intolerant.	Potential subjects were excluded if they had any other clinically significant psychiatric or medical conditions, including clinically significant laboratory or ECG values, hypertension, pre-existing structural cardiac abnormalities, or a known hypersensitivity to LDX or any amphetamine compounds. Also excluded individuals who used psychotropics or any medication in the past month with clinically significant central nervous system effects. Individuals with an IQ < 80, or a history of substance dependence or abuse within six months preceding the study were also excluded, as were pregnant or nursing females and individuals who had never held a valid driver's license.
**Biehl2016**	Diagnoses were made by an experienced psychiatrist according to DSM-IV-TR (2000). Patients had to be medication- naïve or without medication for at least 3 months prior to testing with no obvious comorbid disorders to be approached for participation.	Comorbid axis I disorders.
**Block2009 B4Z-US-LYCC NCT00486122**	Children, 6 to 12 years old, who met *Diagnostic and Statistical Manual of Mental Disorders*, 4th ed., Revised, (*DSM-IV-TR*) criteria for ADHD. All patients were required to meet a symptom severity threshold with scores at least 1.5 standard deviations	Exclusion criteria included serious medical illness, a history of psychosis or bipolar disorder, weight <20 kg or >65 kg at visit 1, uncontrolled hypertension, previous nonresponse to an adequate trial of atomoxetine, intolerable side effects while receiving atomoxetine,

Study name	Inclusion criteria	Exclusion criteria
	above age and gender norms on the Attention-Deficit/Hyperactivity Disorder Rating Scale- IV–Parent Version: Investigator Administered and Scored (ADHD RS). The Total score could meet the threshold or the Inattention or Hyperactivity/ Impulsivity subscores as appropriate for the patient's ADHD subtype.	alcohol or drug abuse within the past 3 months, and ongoing use of psychoactive medications other than the study drug. Patients were recruited during routine office visits for ADHD, by referral, and by advertisement.
**Bron2014**	Drug-naive patients between 18 and 55 years of age who were diagnosed with the combined subtype of ADHD.	Exclusion criteria were: severe comorbid psychiatric disorders at time of the screening interview (using the Structured Clinical Interview for DSM disorders; SCID), treatment with stimulants, antipsychotics, clonidine, benzodiazepines, or beta-blockers within one month prior to study participation or any medication that could influence the CPT performance (i.e. TCA or SSRI), any cognitive disorder like dementia or amnesic disorder, mental retardation, or being pregnant or nursing.
**Buitelaar1996**	ADHD as per DSM-III-R; CBCL and CTRS hyperactivity factors scores in the clinical range; attention deficits on neuropsychological test; no previous treatment with psychotropic medications; clinical indication for treatment with stimulants.	Exclusion criteria: a diagnosis of tic disorder or pervasive developmental disorder, a family history of tic disorder, and the usual contra-indications for treatment with beta-blockers such as cardiac diseases, in particular conduction abnormalities and bradycardia, hypotension, obstructive pulmonary diseases, and insulin-dependent diabetes.
**Casas2013 EudraCT#:2007-002111-82**	Eligible subjects were adults (18–65 years) with ADHD according to the criteria described in the Diagnostic and Statistical Manual for Mental Disorders 4th Edition Text Revision (DSM-IV-TR) (American Psychiatric Association 2000), confirmed using Conners' Adult ADHD Diagnostic Interview Part II for DSM-IV. To be eligible, subjects had to score 24 on the 18 DSM-IV items measured by the investigator-rated Conners Adult ADHD Rating Scale – Screening Version (CAARS-O:SV). Women of child-bearing potential had to use appropriate contraception during the study.	Key exclusion criteria included known non- response to MPH; any clinically unstable psychiatric condition; family history of schizophrenia or affective psychosis; autism, Asperger's syndrome, eating disorder, motor tics or history (including family history) of Tourette's syndrome; substance use disorder (not including caffeine or nicotine dependence), hyperthyroidism, myocardial infarction or stroke 6 months before screening; history of seizures, glaucoma or uncontrolled hypertension; and angina pectoris or cardiac arrhythmias. Women who were pregnant or breastfeeding were also excluded.
**Casat1989**	6-12 y, ADHD as per DSM-III. Good physical health, normal laboratory test, ECG, EEG.	IQ < 70; body weight< 20 kg.

Study name	Inclusion criteria	Exclusion criteria
**Childress2009 CRIT124E2305 NCT00301236**	The study population consisted of children of either gender (aged 6–12 years) diagnosed with ADHD of any subtype as per Diagnostic and Statistical Manual of Mental Disorders, 4 edition, Text Revision (DSM-IV-TR) criteria (predominantly inattentive, pre- dominantly hyperactive=impulsive or combined). It was essential that patients attending school had the same teacher (English or math) for the entire duration of the study, who was willing and able to spend sufficient time with the patient to make valid weekly assessments reflecting the child's symptoms over the past week. Patients were eligible for screening only if they were either drug naıve or not treated with any MPH-related medication in the month prior to the study. Patients receiving psychological or behavioral therapies before the screening visit were considered eligible to participate, provided that the therapy had been ongoing for at least 3 months with the same therapist. Patients had to have an academic competence appropriate to their age and the following subscale total scores on the Conners' ADHD=DSM-IV Scales for teacher (CADS-T): For boys, baseline scores on the CADS-T subscale total were required to be 27 for those 6–8 years old, 24 for those 9–11 years old, or 19 for those 12 years old. For girls, the respective baseline cutoff scores on the CADS-T for the same age groups were 16, 13, or 12.	The exclusion criteria included home-schooled children, any medical condition that interfered with study assessments or that was not stable for at least 3 months before screening, clinically significant abnormalities detected during screening, family history of long-QT syndrome, current diagnosis or a history of cardiac abnormalities, seizures, psychiatric disorders such as schizophrenia, schizoaffective disorder, severe obsessive-compulsive disorder, conduct disorder, autism, chronic tic disorder, Tourette disorder, and any mood or anxiety disorder. Antidepressants, antipsychotics, herbal preparations with psychotropic effects, amphetamine-based medications, benzodiazepines, barbiturates, sedatives or hypnotics, monoamine oxidase inhibitors, and atomoxetine had to be stopped 1–4 weeks prior to randomization according to their half-lives. All concomitant medications that could interfere with the absorption, metabolism, and distribution of the study drug were excluded from start of screening until the end of all evaluations. Over-the-counter analgesics, short-term antibiotic treatment for minor infections, and any medication needed to treat adverse events (AEs) were allowed. Additionally patients who were judged by the investigator as likely to be noncompliant with study procedures, including those with a suspected history of substance abuse, or those living with a person diagnosed with a substance abuse disorder or whose parent or guardian was unable or unwilling to complete the Conners' ADHD=DSM-IV Scales for parents (CADS-P) were also excluded from the investigation.
**Coghill2013 SPD489-325**	Male and female children (6–12 years old) and adolescents (13–17 years old) who satisfied the Diagnostic and Statistical Manual of Mental Disorders, Fourth Edition, Text Revision (DSM-IV-TR) criteria for a primary diagnosis of ADHD. Patients had ADHD of at least moderate severity, as defined by a baseline ADHD Rating Scale version IV (ADHD-RS-IV) total score of 28 or higher. Additional inclusion criteria included: age-appropriate intellectual functioning; blood pressure measurements within the 95th percentile for age, sex, and height; and ability to swallow a capsule. Girls of childbearing potential had to have a negative urine pregnancy test at baseline and to comply with any contraceptive requirements of the protocol.	Key exclusion criteria included: failure to respond to previous OROS-MPH therapy; presence of a comorbid psychiatric diagnosis with significant symptoms (based on Kiddie-Schedule for Affective Disorders and Schizophrenia for school age children – Present and Lifetime – diagnostic interview); conduct disorder (excluding oppositional defiant disorder); pregnancy or lactation; weight below 22.7kg; body mass index (BMI, kg/m) greater than the 97th percentile for age and sex; positive urine drug test (with the exception of the patient's current ADHD therapy); clinically significant electrocardiogram or laboratory abnormalities; suspected substance abuse or dependence disorder (excluding nicotine) within the previous 6 months; history of seizures; tics or Tourette's disorder; known structural cardiac abnormality; or any other condition that might increase vulnerability to the sympathomimetic effects of a stimulant

Study name	Inclusion criteria	Exclusion criteria
		drug. Patients whose current ADHD medication provided effective control of symptoms with acceptable tolerability were also excluded.
**Connor2000**	ADHD as per DSM-III-R plus aggressive ODD or CD; score > 1.5 SD at the inattentive subscale of the CBCL and > 93rd centile on the CAPS. 11/24 history of treatment with MPH; 48 h wash out. Normal findings on general physical examination.	Medical history contraindicating use of MPH or clonidine.
**Connor2010 SPD503-307NCT0036783 5**	Male and female subjects aged 6–12 years with a Diagnostic and Statistical Manual of Mental Disorders, Fourth Edition, Text Revision a baseline score ≥24 on the ADHD Rating Scale IV (ADHD-RS-IV) and a baseline score ≥ 14 (males) or ≥12 (females) on the oppositional subscale of the Conners' Parent Rating Scale- Revised: Long Form (CPRS-R:L).	Subjects were excluded for any current co-morbid psychiatric diagnosis (except oppositional defiant disorder [ODD], dysthymia or simple phobias), weight <55 lb (<25 kg), pre-existing cardiovascular complications, or current use of medications that affect the CNS, blood pressure or heart rate (except for ADHD therapies, which were discontinued during the washout period).
**Cook1993**	Subjects were included if they were male, between the ages of six and ten, and if their full-scale IQ (FIQ) scores on the Wechsler Intelligence Scale for Children-Revised were 85 or above. DSM-III criteria for ADD.	Seizures, cerebral palsy, learning disability, speech or learning problems, vision or peripheral learning problems, thought disorder, abnormal auditory brainstem evoked potentials, previous drugs for ADD.
**CRIT124DUS02**	Female 12-17 y, ADHD confirmed by DISC4; age appropriate academic functioning.	Medical condition interfering with study participation, pregnancy, difficulty swallowing capsules, sensitivity to study drug or drugs same class, use of any investigational medication in the past 30 days.
**Dell'Agnello2009**	The study group included patients of both sexes aged 6–15 years, with ADHD and ODD diagnosed according to the DSM-IV criteria To be eligible in the study, patients were required to have a score of at least 1.5 SD above the age norm for the ADHD subscale of the SNAP-IV, a CGI-S ≥4 at both screening and baseline, a SNAP-IV ODD subscale score of at least 15, and a normal intelligence, i.e. a score of ≥70 on an Intelligence Quotient (IQ) test.	Patients with any of the following conditions were excluded from study participation: body weight b20 kg; history of bipolar I or II disorder, or history of psychosis or pervasive development disorder; history of any seizure disorder (other than febrile seizures) or past/concomitant intake of anticonvulsants for seizure control; serious risk of suicide; history of severe drug allergies; current or past (within 3 months) alcohol or drug abuse; clinically significant cardiovascular disease (including hypertension) or other conditions that could be worsened by an increased heart rate or increased blood pressure; cant laboratory or ECG abnormalities; medical conditions likely to increase sympathetic nervous system activity or regular intake of sympathomimetic drugs; narrow-angle glaucoma; uncontrolled thyroid dysfunction; likelihood of start of structured psychotherapy at any time during the study; pregnant or breastfeeding females, or females at risk of pregnancy.

Study name	Inclusion criteria	Exclusion criteria
**Dittmann2011**	Patients were aged 6 to 17 years and had to meet DSM-IV, Text Revision (DSM-IV-TR) (American Psychiatric Association 2000) criteria for ADHD (any subtype), and DSM-IV-TR criteria A–C of ODD. The presence of DSM-IV-TR diagnostic criteria for CD was not exclusionary.	Patients who had a history of bipolar I or II disorder, psychosis, pervasive developmental disorder, or seizure disorder (other than febrile seizures) were excluded. Patients were excluded if they were at serious suicidal risk, as determined by the investigator, or if they were likely to require psychotropic medications other than study drug or a structured psychotherapy. Psychotherapy initiated before study participation was acceptable.
**Dopfner2003**	- Written consent of the parents and the patient<br>- Diagnosis of an ADHD according to DSM IV (DCL-HKS)<br>- Ambulatory-treated patients aged 6 to 16 years<br>- Substantial hyperkinetic symptoms in the judgment of the class teacher (FBB-HKS> 1.0)<br>- IQ > 85 (CFT)<br>- Body weight ≥ 20 kg	- Severe depression or anxiety disorder (DCL-DES, DCL-ANG)<br>- Tic- / Tourette disorder or the familial occurrence of a tic disorder<br>- Pervasive Developmental Disorder - Psychosis<br>- History of seizure or vulnerability to seizure in the EEG<br>- Previous treatment with MPH or other psychostimulants three weeks before the start of the study<br>- Contraindication to the treatment according
**Durell2013B4Z-US-LYDZNCT00510276**	Adults, aged 18 to 30 years, met DSM-IV, Text Revision (DSM-IV-TR) criteria for ADHD as determined by a clinical interview and assessed by the Adult ADHD Clinician Diagnostic Scale version 1.2. All participants also must have had a Clinical Global Impression-ADHD-Severity (CGI-S) score of 4 (moderate symptoms) or greater to be eligible for study participation. Participants with concomitant current or lifetime diagnoses of specific phobias, generalized anxiety disorder, or social anxiety disorder were allowed in the trial, as were participants with a history of dysthymia within 2 years of study screening.	Potential participants were excluded from the trial if they had current major depression, panic disorder, posttraumatic stress disorder, an eating disorder, or substance abuse or dependence, as well as current or lifetime obsessive-compulsive disorder, bipolar disorder, or psychosis. In addition, any participant who had more than a 25% reduction in their ADHD symptoms as measured by the Conners' Adult ADHD Rating Scale: Investigator-Rated: Screening Version (CAARS-Inv:SV) Total ADHD Symptoms scores between visits 1 and 2 (screening period) was excluded from the study.
**Efron1997**	Criteria for enrollment in the trial were 1) age between 5 and 15 years; 2) satisfy Diagnostic and Statistical Manual of Mental Disorders, 4th ed (DSM–IV) criteria for ADHD; 3) *T* score of at least 1.5 SD units above the mean on the attention problems scale of the Child Behavior Checklist (CBCL) or Teacher Report Form (TRF).	History of intellectual disability, gross neurologic abnormality, or Tourette's syndrome.
**Findling2008 NCT00444574**	Children aged 6 to 12 years, inclusive, who were diagnosed with ADHD according to *Diagnostic and Statistical Manual of Mental Disorders,* Fourth Edition, Text Revision (DSM-IV-TR) criteria (predominantly hyperactive/ impulsive, inattentive, or combined type) were eligible for study inclusion. Participants were either naive to stimulant treatment or known to be responsive to stimulants. At screening, participants were required to have a	Children were excluded from enrollment if they had any comorbid psychiatric diagnosis (with the exception of oppositional defiant disorder), a history of seizures during the last 2 years, a tic disorder, or any concurrent illness or skin disorder that might compromise safety or study assessments. Participants could not have taken clonidine, atomoxetine, antidepressants, antihypertensives, investigational medications, hepatic or cytochrome P450 enzyme altering agents,

Study name	Inclusion criteria	Exclusion criteria
	Kaufman Brief Intelligence Test (KBIT) IQ score of ≥ 80, a total score of ≥ 26 on the ADHD Rating Scale–Version IV (ADHD-RS-IV; maximum possible score of 54) while unmedicated, and normal laboratory parameters and vital signs, including electrocardiogram (ECG) results.	medications with central nervous system effects, sedatives, antipsychotics, or anxiolytics within the 30 days prior to study entry.
**Findling2011 SPD489-305 NCT00735371**	The study enrolled adolescent participants (13 through 17 years at the time of consent and baseline) who met *DSM-IV-TR* diagnostic criteria for ADHD. ADHD diagnosis was confirmed using the Kiddie-SADS— Present and Lifetime Diagnostic Interview (K-SADS- PL). Participants were required to have moderate to severe ADHD symptoms at baseline (score of 28 on the ADHD Rating Scale IV: Clinician Version [ADHD- RS-IV] assessment). Other inclusion criteria included age-appropriate intellectual function and blood pressure (BP) measurements 95th percentile for age, gender, and height.	Participants with conduct disorder or a comorbid psychiatric diagnosis (oppositional defiant disorder was not exclusionary) requiring medication were excluded. Those participants with a concurrent chronic/acute medical condition that might confound efficacy/safety assessments or pose a safety risk, a history of seizures, tic disorder or family history of Tourette disorder, family history of sudden cardiac death or arrhythmia, abnormal thyroid function (a stable dose of thyroid medication for at least 3 months was permitted), glaucoma, or those considered a suicide risk were excluded. Body mass index could not be 5th or 97th percentile for age and gender. Participants who tested positive on urine drug screen (except current stimulant therapy), or had a recent history of suspected substance abuse (excluding nicotine) were not enrolled. Pregnant/lactating females were not included. Participants with clinically significant electro- cardiogram (ECG) findings, who required medications with central nervous system effects, with failure to respond to and/or intolerance of amphetamine therapy, and/or who were well controlled on current ADHD medication with acceptable safety and efficacy were disqualified. Participants could continue participation in behavioral therapy during the study as long as they had been receiving the therapy for at least 1 month at the time of the baseline visit and the therapy did not change during the study. Anyone who previously participated in an LDX trial could not participate.
**Frick2017 SPD465-303 NCT00152022**	Adults (men or nonpregnant, nonlactating women aged 18-55 years) meeting *Diagnostic and Statistical Manual of Mental Disorders* criteria for a primary ADHD diagnosis established using the Adult ADHD Clinical Diagnostic Scale Version 1.2 and having baseline ADHD-RS-IV total scores ≥32 were eligible. All eligible participants had satisfactory medical assessments, with no clinically significant or relevant abnormalities.	Key exclusion criteria included current comorbid psychiatric disorders (defined by the Structured Clinical Interview for the *DSM-IV-TR* [SCID] Axis I Disorders and controlled with prohibited medications or uncontrolled and associated with significant symptoms); any conditions/ symptoms that could confound clinical assessments at screening; chronic or acute illnesses or unstable medical conditions that could confound safety assessments, lead to increased risk, or make it difficult to comply with the proto- col; a history of seizures

Study name	Inclusion criteria	Exclusion criteria
		(excluding infantile febrile seizures), any tic disorder, or a current diagnosis and/or family history of Tourette disorder; known cardiac abnormalities or conditions affecting cardiac performance, a history of hypertension, or sitting SBP >139 mmHg or DBP >89 mmHg; a clinically significant electrocardiogram (ECG) or laboratory abnormality at baseline or screening; the use of a psychoactive prescription medication or over-the-counter medication requiring more than a 28-day washout period (excluding hormonal contraceptives); participation in a clinical study within 30 days of screening; a drug dependence or substance abuse disorder (excluding SCID-defined nicotine dependence) within 6 months before screening or a positive urine drug result at screening or baseline (excluding current psychostimulant medications); and a documented allergy, intolerance, or history of nonresponse to MPH or amphetamine.
**Gau2007 B4Z-TW-S010 NCT00485459**	Children and adolescents, aged 6–16 years old, were eligible to participate if they met the *Diagnostic and Statistical Manual of Mental Disorders*, 4th edition (DSM-IV). The inclusion criteria were: (1) a total score on the ADHD Rating Scale-IV–Parent version: Investigator-Administered and scored (AD- HDRS-IV) of at least 25 for boys or 22 for girls, or greater than 12 for their diagnostic subtype at both visit 1 and visit 2; (2) a Clinical Global Impressions–ADHD–Severity (CGI-ADHD-S) score 4 at both visit 1 and visit 2; (3) normal intelligence as judged by investigators; and (4) no ADHD treatment medication, or completion of the washout procedures before entering this study. Subjects could have been stimulant naïve or previously treated with stimulants.	Subjects were excluded if they weighed less than 20 kg or more than 60 kg; had a serious medical illness, such as a cardiovascular dis-ease; had a history of bipolar I or II disorder, psychosis, or pervasive developmental disorder; had anxiety disorder based on the DSM- IV criteria at study entry; had a history of any seizure disorder or prior electroencephalogram (EEG) abnormalities related to epilepsy, or had taken (or were taking) anticonvulsants for seizure control; had a history of alcohol or drug abuse within the past 3 months; or if they might have to use psychoactive medications other than the study drug during the study period. “For ethical consideration, we did not persuade patients to participate in this study, especially when they were under stable treatment with stimulants, nor did we intend to recruit subjects who were poor responders to or had intolerable adverse events of methylphenidate.”

Study name	Inclusion criteria	Exclusion criteria
**Geller2007 B4Z-US-LYBP**	Participants had to met the DSM-IV (American Psychiatric Association, 1994) criteria for ADHD and for at least one of the following anxiety disorders: separation anxiety disorder, generalized anxiety disorder, or social phobia. At visits 2 and 3, patients must have had a total or subscale score on the Attention-Deficit/Hyperactivity Disorder Rating Scale-IV- Parent Version: Investigator Administered and Scored (ADHDRS- IV-PI) of at least 1.5 SDs above age and sex norms for ADHD subtype, and a total score on the Pediatric Anxiety Rating Scale (PARS; Research Unit on Pediatric Psychopharmacology (RUPP) of at least 15 (maximum score 25). ADHD diagnoses were confirmed clinically, and anxiety and ADHD diagnoses were confirmed using the Kiddie Schedule for Affective Disorders and Schizophrenia for School-Age Children-Present and Lifetime version administered to parent and child.	Patients were excluded if they had significant abnormalities in baseline laboratory or electrocardiogram (ECG) results; met diagnostic criteria for current posttraumatic stress disorder, panic disorder, specific phobias, or obsessive-compulsive disorder; scored Q15 on the Children`s Yale-Brown Obsessive-Compulsive Scale; or had a history of hypertension or bipolar, psychotic, pervasive developmental, or seizure disorders. Patients in the following categories were excluded: pregnant and lactating females, users of monoamine oxidase inhibitors within 2 weeks of visit 2, recent substance abusers, and individuals at serious suicidal risk or with medical or personal conditions likely to affect the trial or health outcomes.
**Ginsberg2012 EUCTR2006-002553-80-SE**	Eligible participants were adult male prison inmates, aged 21–61 years, with ADHD according to DSM-IV criteria. To enter the trial, participants had to have confirmed ADHD in accordance with DSM-IV and to agree not to behave violently during the study. Participants with comorbid disorders such as autism-spectrum disorder, anxiety and depression could take part if they were considered to be stable at baseline. Previous drug- elicited episodes of psychosis were not a cause for exclusion, other than chronic psychoses. Concurrent medication not interfering with methylphenidate was permitted for treating comorbid disorders, as long as doses were stable for at least 1 month at baseline. Hepatitis C without liver insufficiency did not preclude inclusion.	Medications interfering with methylphenidate had to be tapered off before the baseline visit took place. Participants were excluded if they were known to be non-responsive or intolerant to methylphenidate, or intolerant to lactose. In addition, participants were excluded if they showed evidence of substance misuse up to 3 months before baseline, assessed in urine samples. Intellectual disability, epilepsy, glaucoma, uncontrolled hypertension, angina pectoris, cardiac arrhythmias, cardiac abnormality or a family history of serious cardiac illnesses were exclusion criteria
**Goodman2016 NCT00937040**	Eligible participants were adults aged 18 to 65 years with a diagnosis of ADHD, as defined by the *Diagnostic and Statistical Manual of Mental Disorders*, Fourth Edition, and as evaluated at baseline with the adult ADHD Clinical Diagnostic Scale (ACDS), version 1.2, and the Mini- International Neuropsychiatric Interview. Prospective subjects had an adult ADHD Investigator Symptom Rating Scale(AISRS) score >24 at the screening/baseline visit. Those with mild depression according to the Hamilton Depression Rating Scale (HDRS; HDRS score <18) or mild anxiety according to the Hamilton Anxiety Rating Scale (HARS; HARS score <21) were eligible for study participation.	Subjects excluded were those with a history of diagnosis of substance or alcohol dependence or admission/ hospitalization for rehabilitation for dependence, moderate or severe anxiety (HARS score ≥21), moderate or severe depression (HDRS score ≥ 18), and a history of stimulants or atomoxetine use within 5 years or other ADHD medications within 30 days and those for whom, in the investigator's opinion, methylphenidate posed an unacceptable risk through a potential drug interaction or a concurrent medical, neurologic, or psychiatric illness.

Study name	Inclusion criteria	Exclusion criteria
**Goto2017 B4Z-JE-LYEE NCT00962104**	Patients were adults ≥18 years of age who met the *Diagnostic and Statistical Manual of Mental Disorders* criteria for current ADHD and had a historical diagnosis of ADHD during childhood, as assessed by the Conners' Adult ADHD Diagnostic Interview. Patients were required to meet the following additional criteria: scored ≥2 on at least 6 items of either the inattentive or hyperactive/impulsive subscale scores at Visits 1 and 2 on the Conners' Adult ADHD Rating Scale–Investigator Rated: Screening Version (CAARS-Inv: SV); and a CGI-ADHD-S score ≥4 at Visits 1 and 2.	Major exclusion criteria included a history of bipolar disorder or schizophrenia, depressive disorder with a score ≥12 on the 17-item Hamilton Depression Rating Scale, or any current anxiety disorder.
**Greenhill2002**	Children recruited were 6 to 16 years of age and had a primary diagnosis of ADHD, combined subtype or the predominately hyperactive-impulsive subtype as defined in *DSM-IV*. Children had to be in a first-grade or higher school setting in which a single teacher could assess their behavior in the morning and afternoon on specified days. Blood pressure, heart rate, and oral temperature had to be within normal range.	Exclusion criteria included a comorbid psychiatric diagnosis; history of seizure or tic disorder or a family history of Tourette's syndrome; IQ below 80; inability to follow or understand study instructions; female who had undergone menarche; use of am- phetamines, pemoline, or an investigational drug within 30 days of study entry; concomitant use of clonidine, anticonvulsant drugs, or medications known to affect blood pressure, heart rate, or central nervous system function; hyperthyroidism or glaucoma; or any concurrent chronic or acute illness (eg, allergic rhinitis, severe cold) or disability that could confound the study results. Also excluded were children who had failed a previous trial of stimulants for ADHD, had required a third daily dose in the afternoon or evening, had a documented allergy or intolerance to MPH, or were living with anyone who currently had substance abuse disorder (excluding dependency).
**Greenhill2006a Study309Cephal on**	6 to 17 years of age, inclusive; the National Institute of Mental Health Diagnostic Interview Schedule for Children, Fourth Edition (DISC-IV) was used to establish the patients' diagnosis of ADHD using the full DSM-IV diagnostic criteria; Clinical Global Impression of Severity of Illness (CGI-S) rating of 4 or higher (moderately ill or worse); weight and height between the 5th and 95th percentile based on the National Center for Health Statistics; intelligence quotient of at least 80; absence of learning disabilities, with a score of at least 80 on the Wechsler Individual Achievement Test, Second Edition, Abbreviated; attending a full-time school (not home school), with a teacher and parent or legal guardian willing to participate; and total and/or factor scores on the teacher-/ investigator-rated Attention-Deficit/Hyperactivity	Patients were excluded if they had a history or current diagnosis of pervasive developmental disorder, schizophrenia, or other psychotic disorders (DSM-IV axis I); any current psychiatric comorbidity that required pharmacotherapy; any evidence of suicide risk; or ADHD symptoms well controlled on current therapy with tolerable side effects. Patients who had failed to respond to two or more adequate courses (dose and duration) of stimulant therapy for ADHD were also excluded. Additional exclusion criteria were absolute neutrophil count (ANC) below 1 10/L; hypertension (defined as systolic blood pressure [SBP] Q122 mmHg or diastolic blood pressure [DBP] Q78 mmHg for children 6 to 9 years old; Q126 mmHg or Q82 mmHg, respectively, for ages 10 to 12; and Q136 mmHg or Q86 mmHg, respectively, for ages 13 to 17); hypotension (defined as sitting SBP G50 mmHg for children G12

Study name	Inclusion criteria	Exclusion criteria
	Disorder Rating Scale-IV (ADHD-RS-IV) School Version at least 1.5 standard deviations (SD) above the norm for the patient's age and gender.	years of age, G80 mmHg for children Q12 years of age); resting heart rate outside the range of 60 to 115 beats per minute; a history of alcohol or substance abuse as defined by DSM-IV criteria; and consumption of 9250 mg/day of caffeine. Concomitant use of prescription or non-prescription agents with psychotropic properties, including ADHD treatments and dietary supplements, was prohibited within 1 week of the baseline visit and during the study. Monoamine oxidase inhibitors and selective serotonin reuptake inhibitors were prohibited within 2 weeks of baseline testing and throughout the study.
**Greenhill2006b CRIT124E2301**	Eligible participants were males and females 6 to 17 years of age who met DSM-IV criteria for ADHD of any type, as established by a psychiatric examination and a semistructured diagnostic interview (the ADHD module of the Schedule for Affective Disorders and Schizophrenia for School-Age Children-Present and Lifetime Version). For boys, baseline scores on the Conners ADHD/DSM- IV Scale-Teacher version (CADS-T) DSM-IV total subscale were required to be Q27 for those 6 to 8 years old, Q24 for those 9 to 11 years old, Q19 for those 12 to 14 years old, and Q14 for those 15 to 17 years old. For girls, the respective baseline cutoff scores on the CADS-T were Q16, Q13, Q12, and Q6. Patients had to be functioning at age-appropriate levels academically, and female patients who had reached menarche were required to have a negative pregnancy test and to be using adequate and reliable contraception throughout the study.	Excluded were those patients with clinically significant abnormalities in vital signs, physical examinations, or laboratory tests; those with a history of seizures or use of anticonvulsant medication, comorbid psychiatric conditions (obtained by clinical interview); those with any medical condition that could interfere with study participation or assessments or that may pose a danger with administration of methylphenidate; those taking psychotropic medications; and those who initiated psychotherapy within the past 3 months. Patients with a positive urine drug screen or with a history of poor response or intolerance to methylphenidate were also excluded, as were those who were pregnant or nursing or were taking any other investigational drug within 30 days of study entry.
**Grizenko2012**	ADHD according to the criteria of the Diagnostic and Statistical Manual of Mental Disorders, fourth edition (DSM-IV)	Exclusion criteria included an IQ of less than 70, a history of Tourette's syndrome, pervasive developmental disorder, psychosis, and previous intolerance or allergic reaction to MPH.

Study name	Inclusion criteria	Exclusion criteria
**Harfterkamp2012 NCT00380692**	Children and adolescents between 6 and 17 years with a clinical diagnosis of ASD and concomitant ADHD symptoms. Children had to have a confirmed diagnosis of ASD and to have concomitant ADHD symptoms according to our study criteria, plus an intelligence quotient (IQ) of at least 60 on a Wechsler Intelligence Scale. Study criteria of ADHD symptoms were in accordance with *DSM-IV-TR* criteria A through D for any subtype of ADHD, corroborated by scores of at least 1.5 SD above the age norm for children's diagnostic subtype using published norms for the parent- based ADHD-RS. Apart from psychosis and bipolar disorder, all other forms of comorbidity were allowed for entering the study. Also, prior experience with ADHD medication was not an exclusion criterion.	Exclusion criteria included a weight of less than 20 kg, presence of psychosis, bipolar disorder, or sub- stance abuse, a serious medical illness, history of seizures, ongoing use of psychoactive medications other than the study drug, and intended start of a structured psychotherapy or inpatient treatment.
**Herring2012 NCT00475735**	Patients meeting *DSM-IV* criteria for ADHD of either inattentive or combined subtype and having a chronic course of behavior disorder (initiated by age 7 years), as assessed via structured interview using the Adult ADHD Clinician Diagnostic Scale, version 1.2, were enrolled. The main inclusion criteria were age of 18–55 years, a total symptom severity score on the Conners Adult ADHD Rating Scales-Observer Screening Version of ≥ 24, and a score of ≥ 4 (moderately ill) on the Clinical (CAARS-O:SV)Global Impressions-Severity of Illness scale (CGI-S).	The main exclusion criteria were history of other psychiatric disorders (including sleep disorders and substance abuse) or neurologic disorders and history of poor or no response to a prior course of methylphenidate or other stimulant for ADHD.
**Hervas2014 SPD503-316 NCT01244490 EudraCT:2010-018579-12**	Male and female children/adolescents (6–17 years old) with a diagnosis of ADHD of at least moderate severity, as defined by a baseline ADHD-RS-IV with a total score of 32 or higher and a minimum Clinical Global Impression-Severity (CGI-S) score of 4, were enrolled in the study. Those with age-appropriate intellectual functioning; blood pressure measurements within the 95th percentile for age, sex and height; and the ability to swallow tablets or capsules were included. Girls of childbearing potential had to have a negative urine pregnancy test at screening and baseline and to comply with any protocol contraceptive requirements. In addition, participants and their parent/legal guardian had to be willing, able and likely to fully comply with the study procedures and restrictions defined in the protocol.	Exclusion criteria included: clinically significant illness, including a clinically significant abnormal screening visit; current, comorbid psychiatric diagnosis (except oppositional defiant disorder [ODD]); history/presence of cardiac abnormalities, cardiovascular or cerebrovascular disease, serious heart rhythm abnormalities, syncope, tachycardia, cardiac conduction problems, exercise-related cardiac events or clinically significant bradycardia; orthostatic hypotension and/or a known history of hypertension; seizures; and glaucoma. In addition, those with a family history of sudden cardiac death, ventricular arrhythmia or QT prolongation, a patient history of alcohol or substance abuse and those patients with serious tic disorder, including Tourette's syndrome, were excluded. In addition, enrollment was managed to ensure that approximately 25% of those enrolled were adolescents and at least 25% were female. Furthermore, at least 70% of those enrolled were to come from European centers and the

Study name	Inclusion criteria	Exclusion criteria
		remaining 30% from USA/ Canada.
**Huss2014 CRIT124D2302 EUCTR2010-021533-31-DE NCT01259492**	Adult patients (18–60 years) with diagnosis of ADHD, all types, with a confirmed childhood onset according to DSM-IV diagnostic criteria and a DSM-IV ADHD RS total score of >30 at screening and baseline were included in the study.	Exclusion criteria were: pre-existing cardiovascular or cerebrovascular diseases, or any other co-morbid psychiatric disorder requiring medical intervention/therapy or that might interfere with the study conduct at the time of enrollment; patients demonstrating a >30% improvement in DSM-IV ADHD RS total score at baseline relative to that at screening were also excluded from this study. Any psychological or behavioral therapies for the treatment of ADHD were discontinued at least 1 month prior to the screening visit. Patients who initiated these therapies within 3 months prior to screening visit for reasons other than ADHD were excluded from the trial. Additionally, patients with either hypersensitivity or history of poor response or intolerance to stimulants as per the investigator's judgment were excluded from this study. Other exclusion criteria included pregnancy, seizures, recent alcohol or drug abuse and patients with body mass index < 18.5 kg/m or >35 kg/m.
**Jafarinia2012**	Patients were children and adolescents aged 6 to 17years who met the Diagnostic and Statistic Manual (DSM)-IV-TR diagnostic criteria for ADHD. To be included, the patients should have total and/or subscale scores on Attention-Deficit/Hyperactivity Disorder Rating Scale-IV (ADHD-RS-IV) School Version of at least 1.5 standard deviations (SDs) above norms for patient's age and gender.	Exclusion criteria were psychiatric comorbidities (excluding oppositional defiant disorder), high risk of suicide, mental retardation (IQ 70), clinically important chronic medical condition (such as epilepsy and organic brain disorders), drug abuse or dependence in the last 6 months, hypertension or hypotension, history of allergy to bupropion or methylphenidate, abnormal electrocardiogram, and psychotropic medication use in the last 14 days.
**Jain2011 NCT00556959**	Patients 6 to 17 years of age with a diagnosis of ADHD of the hyperactive or combined inattentive/hyperactive subtype according to criteria set forth in the *Diagnostic and Statistical*	Female patients of childbearing age who were pregnant or lactating or who refused to use birth control were excluded from the study. Patients were also excluded if they had a clinically significant illness or

Study name	Inclusion criteria	Exclusion criteria
	Manual of Mental Disorders, fourth edition, and each patient's clinical research physician and a minimum score of 26 on the ADHD Rating Scale–IV (ADHD-RS-IV) were eligible to participate in the study. Patients were required to be in good health, be able to swallow tablets, be mentally competent, and have a body mass index of at least the fifth percentile for the patients' age group. Patients with a concomitant diagnosis of tics or oppositional defiant disorder were eligible for study inclusion.	abnormality that would increase the safety risk of clonidine or if they had a clinically significant abnormality on electrocardiographic readings that were interpreted by a single entity. Patients with a concomitant diagnosis or history of a psychiatric disorder that required psychotropic medication and patients with a severe concomitant Axis I or II disorder that could interfere with assessment of clonidine safety and efficacy were also excluded. In addition, patients with a history of conduct disorders, syncopal episodes, or seizures (except for febrile seizure before 2 years of age) were not enrolled. Patients with known drug abuse, a history of drug abuse, or a history of clonidine intolerance, including dermatologic reaction to transdermal clonidine, were excluded. Patients were also not enrolled if they had used any investigational drug within 30 days of the study initiation or had a positive drug test result for any medications other than those used for the treatment of ADHD.
**Kahbazi2009**	Between the ages of 6 and 15 who clearly met the DSM-IV-TR diagnostic criteria for ADHD. Additional inclusion criteria included total and/or subscale scores on the Attention-Deficit/Hyperactivity Disorder Rating Scale-IV (ADHD-RS- IV) School Version at least 1.5 standard deviations above norms for patient's age and gender. To participate, parents and children had to be willing to comply with all requirements of the study.	Children were excluded if they had a history or current diagnosis of pervasive developmental disorders, schizophrenia or other psychiatric disorders (DSM-IV axis I); any current psychiatric comorbidity that required pharmacotherapy; any evidence of suicide risk and mental retardation (I.Q. b70 based on clinical judgment). In addition, patients were excluded if they had a clinically significant chronic medical condition, including organic brain disorder, seizures, and current abuse or dependence on drugs within the preceding 6 months. Additional exclusion criteria were hypertension, hypotension and habitual consumption of more than 250 mg/day of caffeine.
**Kay2009a,b**	Eligible subjects were men or women, aged 19 to 25 years, who satisfied *Diagnostic and Statistical Manual of Mental Disorders–Fourth Edition, Text Revision* (*DSM-IV-TR*; American Psychiatric Association, 2000) criteria for a primary diagnosis of ADHD. Women of childbearing potential were included only if they had a negative serum beta human chorionic gonadotropin pregnancy test and abstained from sexual activity that could result in pregnancy or used acceptable contraceptives from time of informed consent throughout the study duration. Additional inclusion criteria included a valid driver's license and 💿 3 years of driving experience, abstinence from illegal drug use during the study, and willingness and ability to comply with all study requirements defined in the protocol. A score □□24 (severity	Women who were pregnant or lactating were excluded from study participation. Additional exclusion criteria included a recent history (past 6 months) of drug dependence or substance abuse (excluding nicotine); a positive urine drug screen; alcohol use 24 hours before any test day; any cardiac condition that, in the opinion of the investigator, would require exclusion; a current comorbid psychiatric diagnosis (controlled or uncontrolled) with significant symptoms that, in the opinion of the investigator, would confound efficacy or safety assessments; documented allergic or adverse reactions to MAS XR or atomoxetine; documented history of failure to respond clinically to amphetamines or atomoxetine; history of at least one seizure within the past 2 years, a tic disorder, or family history of Tourette's syndrome; inadequately treated thyroid dysfunction; history of

Study name	Inclusion criteria	Exclusion criteria
	worse than mild to moderate range) on the ADHD Rating Scale - with adult prompts based on the ADHD-RS- Version IV—was required. Subjects were eligible for inclusion in the study if they demonstrated no greater than average performance on at least one of two standardized measures of executive function: a score >50th percentile on either the Stroop Color and Word Test or the Halstead–Reitan Category Test.	glaucoma; any concurrent chronic or acute illness (including severe allergic rhinitis or severe cold) that might interfere with assessments; and use of any medication that is contraindicated with MAS XR or atomoxetine or that might have confounded results of the safety assessment. In addition, subjects who were naïve to pharmacologic treatment for ADHD were excluded.
**Kelsey2004 B4Z-US-LYBG**	Children 6 to 12 years of age who met *Diagnostic and Statistical Manual of Mental Disorders* (4th ed.) criteria for ADHD. All patients were required to meet a symptom severity threshold, with a symptom severity score at least 1.5 SDs above age and gender normative values, as assessed with the Attention-Deficit/Hyperactivity Disorder Rating Scale-IV-Parent Version: Investigator-Administered and Scored (ADHD RS), for the total score or either of the inattentive or hyperactive/ impulsive subscales.	Important exclusion criteria included serious medical illness, a history of psychosis or bipolar disorder, alcohol or drug abuse within the past 3 months, and ongoing use of psychoactive medications other than the study drug.
**Kollins2011 SPD503-206 NCT00150592**	Male and female subjects aged 6 to 17 years meeting Diagnostic and Statistical Manual of Mental Disorders, Fourth Edition, Text Revision (DSM-IV-TR) (American Psychiatric Association 2000) criteria for a diagnosis of ADHD, a baseline score 24 on the ADHD Rating Scale IV (ADHD-RS-IV) and a baseline score 4 on the Clinical Global Impressions-Severity (CGI-S) scale, were enrolled.	Reasons for exclusion included any current comorbid psychiatric diagnosis (except oppositional defiant dis- order), weight <25 kg (55 lb), cardiac conditions that might have increased the safety risk to the subject, or a Pediatric Daytime Sleepiness Scale (PDSS) score ≥ 22 at screening and/or baseline.
**Kooij2004**	ADHD as per DSM-IV. Subjects with co-morbid psychiatric disorders were included, unless these disorders required to be treated first or when treatment with methylphenidate was contra-indicated.	Subjects with clinically significant medical conditions, abnormal baseline laboratory values, a history of tic dis- orders, mental retardation (IQ<75), organic brain disorders, clinically unstable psychiatric conditions (i.e. suicidal behaviours, psychosis, mania, physical aggression, currently ongoing substance abuse), current use of psychotropics, prior use of methylphenidate or amphetamines, as well as pregnant or nursing women.

Study name	Inclusion criteria	Exclusion criteria
**Kurlan2002**	Subjects were aged 7–14 years, in school, and of any race or ethnic background. Each subject met *Diagnostic and Statistical Manual of Mental Disorders, 4th ed.* (DSM-IV) criteria for ADHD of any subtype. severity of ADHD symptoms above specified cutoff scores (boys: grade 2–3 10, grade 4 and above 9; girls: grade 2–3 7, grade 4 and above 6) on the Iowa Conners teacher rating scale. The investigator's rating of global functioning on the Child-Global Assessment Scale (C-GAS) had to be 70 (indicating difficulty in at least one area, such as school). Each subject also met DSM-IV criteria for Tourette disorder, chronic motor tic disorder or chronic vocal tic disorder.	Subjects were excluded if there was evidence of a secondary tic disorder (e.g., tardive tics, neuroacanthocytosis, Huntington disease), major depression, pervasive develop- mental disorder, autism, psychosis, mental retardation, anorexia nervosa, bulimia, a serious cardiovascular (e.g., significant hypotension, congenital heart disease) or other medical disorder that would preclude the safe use of MPH or CLON, impaired renal function (a routine urinalysis was performed), or pregnancy (a urine pregnancy test was performed for all adolescent girls. The following cardiac features were considered exclusions for enrollment: pro- longed Q-Tc interval (440 milliseconds), high-grade ventricular ectopy, AV block beyond first degree, bundle branch block, intraventricular conduction block (100 milliseconds), pacemaker rhythm or heart rate less than 60 on the electrocardiogram (ECG), cardiomyopathy, complex heart disease, aortic or pulmonary stenosis, family history of long QT syndrome, cardiomyopathy or premature (age 45 years) sudden death, history of syncope, and blood pressure less than 2 standard deviations from the age- and gender-adjusted mean. Subjects could not receive any other medications for the treatment of ADHD, tics, or other associated behavioral symptoms. Any such treatment had to be discontinued at least 6 weeks (2 weeks for MPH) before enrollment. Non- pharmacologic (e.g., behavioral) interventions were al- lowed, but remained unchanged throughout the course of the study. Prior use of MPH or CLON, whether judged to be beneficial or not, was permitted. Subjects who reported a worsening of tics during prior treatment with a stimulant were not excluded.
**Lin2014**<br>**NCT00922636**	The study included female and male patients ≥ 6 years and < 17 years and 9 months of age at the time of informed consent. Study participants had to meet Diagnostic and Statistical Manual of Mental Disorders, 4th ed., Text Revision (DSM-IV-TR) criteria for ADHD, based on a clinician interview, and confirmed using the Kiddie Schedule for Affective Disorders and Schizophrenia for School Aged Children- Present and Lifetime version (K-SADS-PL) (at screening), have an ADHD-RS-IV-Parent:Inv total score ≥1.5 standard deviations above the age and gender norms (at screening and week 0), and have a CGI-ADHD-S score ≥4 (at screening and week 0).	The following were primary exclusion criteria: Body weight < 18 kg or >75kg; history of bipolar I or II disorder, or psychosis. seizure disorder or pervasive developmental disorder; presence of motor tics or a diagnosis of Tourette's syndrome; marked anxiety, tension, or agitation sufficient to contraindicate treatment with OROS MPH; history of electroencephalographic abnormalities; clinically significant abnormal electrocardiogram; serious or un- stable medical illness; any medical condition that would increase sympathetic nervous system activity markedly (e.g., catecholamine- secreting neural tumor); requiring the daily use of medications with sympathomimetic activity (e.g., albuterol, pseudoephedrine); any medical condition that would be exacerbated by an increase in norepinephrine tone; or current or past history of clinically significant hypertension.

Study name	Inclusion criteria	Exclusion criteria
**Lin2016 NCT00917371**	The inclusion criteria were (1) that subjects had typical ADHD symptoms before 7 years old which meet the DSM-IV-TR ADHD at childhood and currently based on Gau's clinic diagnosis and the ADHD supplement of the K-SADS for adults; (2) that their Clinical Global Impressions-ADHD-Severity (CGI-ADHD-S) score>4 and psychotropic medication-naïve for the past year; (3) that their IQ greater than 80; and (4) that they consent to this study and they can keep appointments for clinic visits and all tests (from https://clinicaltrials.gov/ct2/show/NCT00917371)	(1) Comorbid with DSM-IV-TR diagnosis of pervasive developmental disorder, schizophrenia, schizoaffective disorder, delusional disorder, other psychotic disorder, organic psychosis, schizotypal personality disorder, bipolar affective disorder, and mental retardation; (2) In the major depressive episode, comorbid with severe anxiety disorders or during substance intoxication or withdrawal at the time of evaluation; (3) With neurodegenerative disorder, epilepsy, involuntary movement disorder, congenital metabolic disorder, brain tumor, history of severe head trauma, and history of craniotomy; (4) A history of alcohol or drug abuse within the past 3 months; (5) The need of psychotropic medications apart from MPH or atomoxetine, including Chinese medicine or health-food supplements that have central nervous system activity; and (6) With visual or hearing impairments, or motor disability which may influence the process of neuropsychological assessment.
**Martenyi2010 B4Z-MW-LYCZ NCT00386581**	Outpatient children and adolescents, 6–16 years of age, with a DSM-IV diagnosis of ADHD, confirmed by the Russian version of the Kiddie Schedule for Affective Disorders and Schizophrenia for School-aged Children-Present and Lifetime Version (K-SADS-PL. At both visits, 1 and 2 (screening and randomization), had a minimum score of 25 for boys and 22 for girls, or [12 for their diagnostic sub- type on the Attention-Deficit/Hyperactivity Disorder Rating Scale-IV-Parent Version: Investigator-Administered and Scored as well as a score of C4 on the Clinical Global Impressions-ADHD-Severity (CGI-ADHD-S) scale; had not taken any medication for the treatment of ADHD or completed washout procedures; had no significant abnormalities in laboratory results and baseline ECG; and were able to communicate suitably with the investigator and study coordinator.	Patients were excluded if they weighed <20 kg or >60 kg at study entry; experienced no clinical benefit after an adequate trial with methylphenidate or amphetamine(all patients were psychostimulant naıve, but it was not required by the protocol); had been treated, within the previous 30 days, with a drug (not including study drug) that had not received a regulatory approval for any indication at the time of study entry; had a history of bipolar I or II disorder, psychosis, or pervasive developmental disorder; met DSM-IV criteria for an anxiety disorder (as assessed by the investigator and confirmed by the K-SADS-PL); had a history of any seizure disorder (other than febrile seizures) or prior electroencephalogram abnormalities related to epilepsy; had taken (or were taking) anticonvulsants for seizure control; were at serious suicidal risk or had a serious medical illness; or were pregnant or breast-feeding. Sexually active female patients had to use a medically acceptable method of contraception. Female patients of child-bearing potential, who were abstinent, were allowed to enter the study, provided they agreed that if they became sexually active, they would use a medically acceptable method of contraception.
**McCracken2016**	Male or female individuals 7 to 14 years of age; DSM-IV ADHD (any subtype) diagnosed by semi- structured diagnostic interview	Autistic disorder, chronic tic disorder, psychosis, bipolar disorder, or structural heart defects; current major depression or panic disorder;

Study name	Inclusion criteria	Exclusion criteria
	(Kiddie-Schedule for Affective Disorders and Schizophrenia LPL [K-SADS-PL]) and clinical interview; and Clinical Global Impression—Severity (CGI-S) score ≥ 4 for ADHD.	systolic or diastolic blood pressure >95th or <5th percentile for age and body mass index (BMI); medical condition contraindicating stimulants or a agonists; and need for chronic use of other central nervous system (CNS) medications.
**McRae-Clark2010 R21DA018221 NCT00360269**	Subjects had to be between 18 and 65 years of age and meet DSM-IV criteria for marijuana dependence. Participants also had to meet DSM-IV criteria for ADHD with the exception of the criterion that the age of onset of symptoms had to be prior to 7 years of age. This adjustment was made based on the DSM-IV field trial which found that the use of the 7 years of age criterion diminished the reliability of clinical diagnosis. Participants were therefore included if symptoms of ADHD were present prior to the age of 12.	dependence on any other substance (with the exception of caffeine or nicotine); history of psychotic disorder; current major depression or eating disorder; current treatment with a psychoactive medication; major medical illnesses; cognitive impairment; and pregnancy, nursing, or inadequate birth control.
**Medori2008 LAMDA-IEUCTR2004-000730-37 NCT00246220**	Adult men and women with a diagnosis of ADHD according to the criteria of the Diagnostic and Statistical Manual of Mental Diseases, Fourth Edition (DSM-IV) and confirmed by the Conners' Adult ADHD Diagnostic Interview for DSM-IV (CAADID). Other requirements for inclusion were age 18 to 65 years; chronic course of ADHD symptomatology from childhood to adulthood with some symptoms present before age 7 years, as determined by investigators following the CAADID interview; and CAARS total score of ≥ 24 at screening.	History of poor response or intolerance to methylphenidate; any current clinically unstable psychiatric condition (e.g., acute mood disorder, bipolar disorder, acute obsessive-compulsive disorder), as determined by the investigator; or they substance use disorder (abuse/dependence) according to DSM-IV criteria within the last 6 months. Other exclusion criteria included family history of schizophrenia or affective psychosis; serious illnesses (e.g., hepatic or renal insufficiency or significant cardiac, gastrointestinal, psychiatric, or metabolic disturbances); hyperthyroidism, myocardial infarction, or stroke within 6 months of screening; and history of seizures, glaucoma, or uncontrolled hypertension.
**Michelson2001 B4Z-MC-LYAC**	Children and adolescents who were 8 to 18 years of age were eligible to participate if they met the *Diagnostic and Statistical Manual of Mental Disorders, Fourth Edition* (*DSM-IV*) criteria for ADHD by clinical assessment, confirmed by a structured interview (the behavioral module of the Kiddie Schedule for Affective Disorders and Schizophrenia for School Aged Children–Present and Lifetime Versions (KSADS-PL). Patients also had to have a symptom severity score at least 1.5 standard deviations (SD) above age and gender norms on the Attention-Deficit/Hyperactivity Disorder Rating Scale-IV–Parent Version: Investigator Administered and Scored (ADHD RS) for the total score or either of the inattentive or the hyperactive/ impulsive subscales	Important exclusion criteria included IQ 80 as assessed by the Wechsler Intelligence Scale for Children–3rd Edition, serious medical illness, comorbid psychosis or bipolar disorder, history of a seizure disorder, or ongoing use of psychoactive medications other than the study drug.

Study name	Inclusion criteria	Exclusion criteria
**Michelson2002 B4Z-MC-LYAT**	Children and adolescents, 6–16 years of age, who met DSM-IV criteria for ADHD, as assessed by clinical interview and confirmed by the Schedule for Affective Disorders and Schizophrenia for School-Age Children—Present and Lifetime Version (K-SADS-PL) (7), were eligible to participate. All patients were required to meet a symptom severity threshold: a score at least 1.5 standard deviations above age and gender norms as assessed by the investigator- administered and -scored parent version of the ADHD Rating.	Important exclusion criteria included serious medical illness, a history of psychosis or bipolar disorder, alcohol or drug abuse within the past 3 months, and on- going use of psychoactive medications other than the study drug.
**Michelson2003a, b**	Adults who met DSM-IV criteria for ADHD as assessed by clinical interview and confirmed by the Conners' Adult ADHD Diagnostic Interview for DSM-IV were recruited from clinics and by advertisement. Patients were required to have at least moderate symptom severity, and the diagnosis had to be corroborated by a second reporter for either current symptoms (by a significant other) or childhood symptoms (by a parent or older sibling).	Patients who met diagnostic criteria for current major depression or anxiety disorder or for current or past bipolar or psychotic disorders were excluded, as were patients with serious medical illness and patients who met DSM-IV criteria for alcohol dependence. A history of episodic recreational drug use did not exclude patients, but patients actively using drugs of abuse at the time of study entry were excluded.
**Moharari2012 IRCT2010122955 00N1**	At least 20 in ADHD-RS; Parents' consent; IQ more than 70.	Medical or neurologic disorders such as epilepsy; history of taking MPH or bupropion. If the parents change their mind at any step of the research to take their child out of research; encountering serious side effects or neurologic symptoms such as epilepsy.
**Montoya2009 B4Z-XM-LYDM NCT00191945**	Newly diagnosed (time since diagnosis 3 months), treatment-naıve cases of ADHD defined according to the criteria of the revised fourth edition of the Diagnostic and Statistical Manual of Mental Disorders (DSM-IV-TR). The Kiddie Schedule for Affective Disorders and Schizophrenia-Present and Lifetime version (K-SADS-PL) was used at screening stage to confirm the diagnosis. Other inclusion criteria were: age between 6 and 15 years, and an ADHDRS-IV-Parent:Inv total score 1.5 standard deviations above the age norm for their diagnostic subtype.	History of bipolar disorder, psychosis, pervasive developmental disorder or seizure disorder, glaucoma or hypertension, intelligence quotient (IQ) below 70 at investigator's judgment, any pervasive developmental disorder, alcohol or drug abuse within the past 3 months, planned start of structured psychotherapy at any time during the study, and taking any regular psychoactive or sympathomimetic medication.
**NCT01069523**	Children aged 6-12 years; meet criteria for Attention Deficit Hyperactivity Disorder	Do not meet criteria for Major Depression, Bipolar, Autism<br>Talking any psychotropic medication for a condition other than ADHD<br>History of epilepsy, severe head injury or loss of consciousness<br>History of Intolerance to Guanfacine.
**Newcorn2008 B4Z-MC-LYBI**	The patients were children and adolescents, ages 6 to 16 years, who met the DSM-IV criteria for ADHD, any subtype, as deter-mined by clinical history and confirmed by a semistructured	Patients who had seizures, bipolar disorder, a psychotic illness, or a pervasive developmental disorder or who were taking concomitant psychoactive medications were excluded from the study. Because

Study name	Inclusion criteria	Exclusion criteria
	interview, the Schedule for Affective Disorders and Schizophrenia for School Aged Children—Present and Lifetime Version (K-SADS-PL) . Symptom severity at entry was required to be at least 1.5 standard deviations above the U.S. age and gender norms as assessed by the ADHD Rating Scale-IV—Parent Version: Investigator-Administered and –Scored. Concurrent psychiatric diagnoses (other than anxiety or tic disorders), including major depressive disorder, were permitted as long as ADHD was the primary diagnosis and therefore an appropriate target of treatment.	anxiety and tic disorders are relative contraindications for use of osmotically released methylphenidate (according to the product label), patients with these conditions were also excluded. Subjects could either have been treated previously with stimulants or be treatment naive. However, for ethical reasons subjects were excluded if they had been treated previously with an adequate trial of methylphenidate or amphetamine and either did not experience at least some improvement in ADHD signs and symptoms (non responders) or had intolerable adverse events. An adequate trial was defined as lasting at least 4 weeks and reaching a total daily dose of at least 1.0 mg/kg per day or 60 mg/day (whichever was lower) of immediate-release methylphenidate, 36 mg/day of osmotically released methylphenidate, or 0.5 mg/kg per day of *d*-amphetamine or mixed amphetamine salts.
**Newcorn2013 SPD503-314 NCT00997984**	Outpatient children aged 6 to 12 years with a primary diagnosis of ADHD with combined subtype or hyperactive/impulsive subtype, as defined by the DSM-IV-TR, based on psychiatric evaluation using the Kiddie-Schedule for Affective Disorders and Schizophrenia–Present and Lifetime version (K-SADS-PL). Children were required to have a baseline ADHD-RS-IV total score ≥28 and a Clinical Global Impressions–Severity of Illness Scale score ≥4.	Any current controlled or uncontrolled comorbid psychiatric diagnosis (except oppositional defiant disorder), including any severe comorbid Axis II disorders or Axis I disorders (e.g., posttraumatic stress disorder, bipolar illness, psychosis, pervasive developmental disorder, obsessive compulsive disorder, substance abuse disorder, or other symptomatic manifestations) that could confound efficacy or safety assessments, or for which GXR treatment might be contraindicated; at risk for suicide currently or in the past; history or presence of cardiac abnormalities or a primary sleep disorder; body weight <55 lbs or body mass index >95th percentile; and use of another investigational product within 30 days of baseline.
**Palumbo2008 NCT00031395**	Children ages 7 to 12 years of any race and ethnic background and in school were enrolled. Each subject met DSM-IV criteria for ADHD of any subtype. Severity of ADHD symptoms above specified cutoff scores (boys: grades 2-3 = 10; grade 4 and above = 9; girls: grades 2-3 = 7, grade 4 and above = 6) on the Iowa Conners Teacher Rating Scale. A designated parent in daily contact with the subject also had to indicate the presence of sufficient ADHD symptoms at home on the Iowa Conners Parent Rating Scale. The investigator's rating of global functioning on the Child Global Assessment Scale (CGAS) had to be ≤70 with difficulty evident in at least two areas, such as school and home.	Evidence of a tic disorder, major depression, pervasive developmental disorder, autism, psychosis, mental retardation, anorexia nervosa, bulimia, a serious cardiovascular (e.g., significant hypotension, congenital heart disease) or other medical disorder that would preclude the safe use of methylphenidate or clonidine, impaired renal function (a routine urinalysis was performed), or pregnancy (a urine pregnancy test was performed for all adolescent girls). Family history of long QT syndrome, cardiomyopathy, or premature (age ≤45 years) sudden death were also exclusions. Subjects could not receive any other medications for the treatment of ADHD or other associated psychiatric symptoms. Previous use of methylphenidate or clonidine was permitted. However, any such treatment had to be discontinued at least 6 weeks (2 weeks for methylphenidate) before enrollment.

Study name	Inclusion criteria	Exclusion criteria
**Paterson1999**	Patients were asked to fill out a DSM-IV ADHD symptom checklist. Patients were eligible for inclusion in the trial if they reported the presence of at least four inattentive and/or five hyperactive symptoms during the previous 6 months	Patients were excluded from the study on the grounds of either having an insufficient ADHD score, or comorbidity for other major psychiatric disorders, including a history of current substance abuse. Patients were also screened for organic disorders that would contraindicate the use of dexamphetamine. Finally, all patients eligible for the trial had a sample of urine tested to screen for illicit substance abuse.
**Philipsen2015 EUCTR2006-000222-31-DE ISRCTN5409620 1**	Male and female • Subjects must speak German fluently • Aged 18–60 years inclusive • Diagnosis of ADHD according to the DSM-IV criteria • A score of greater than 30 on the short version of the Wender Utah Rating Scale • Chronic course of ADHD symptoms from childhood to adulthood • Subjects provided written informed consent in accordance with international guidelines and local legislation • Unobtrusive physical examination (including blood pressure/heart rate) without serious or uncontrolled findings • Lab results without clinically relevant findings (e.g., blood count, renal retention data, tests of liver function, thyroid parameters). EKG and EEG without pathologically relevant results • The screening has been fully completed. Laboratory results are not more than 6 weeks old and (if applicable) pregnancy test is not more than 2 weeks before the time of randomization. • It is possible to conduct the baseline assessment within 7 days of randomization and to begin therapy within 14 days	• IQ <85 according to a score of <17 on the Multiple-Choice Vocabulary Intelligence Test (MWT-B, German version)<br>• Schizophrenia, bipolar affective disorder, borderline personality disorder, antisocial personality disorder, suicidality or self-harm, autism, motor tics, Tourette Syndrome<br>• Substance abuse or dependence in the previous 6 months before the screening. Episodic consumption is not an exclusion criterion. A positive drug test during screening<br>• Neurological disorders, seizures, pathological EEG results (lateral differences, lesion, epileptiform potentials), glaucoma, diabetes mellitus, fasting blood glucose level >110 mg/dl, hyperlipidemia, uncontrolled arterial hypertension (according to the guidelines of the German Hypertension Society), angina pectoris, known arterial occlusive disease or another manifestation of vascular disease, known tachycardic arrythmias<br>• History of stroke<br>• Known enlarged prostate<br>• Current eating disorder (bulimia nervosa, anorexia nervosa, Body Mass Index <19)<br>• Participation in a clinical trial within 3 months before the beginning of the study or concurrent participation in another clinical trial<br>• Medication with stimulants or ADHD-specific psychotherapy within the previous 6 months before the beginning of the study<br>• Known hypersensitivity to methylphenidate, other sympathomimetic drugs, or any other excipients<br>• Unwillingness or inability to comply with the requirements of the study protocol<br>• Patient is unable to understand the nature, significance, and scope of the study<br>• Current or planned pregnancy, without the use of defined methods

Study name	Inclusion criteria	Exclusion criteria
		of contraception; lactation; positive pregnancy test during screening<br>• Use of another psychopharmacological medication in addition to randomized treatment before the start of treatment or during study participation (definition of nonapproved medication and the required timing of weaning before treatment)<br>• Regular participation in other outpatient psychotherapy during study participation
**Pliszka2000**	ADHD as per DISC, grades 1-5. Children with comorbid conditions such as oppositional defiant disorder, conduct disorder, or mild anxiety disorders could participate.	No other medical illness, no current psychotropic treatment. Major depression, manic episode, tic disorder, psychosis. The child also had to be a least 1.5 SD above the mean for his/her age and sex on the IOWA CTRS Inattention/Overactivity (IIO) factor. The score on the parent Conners Global Index had to be similarly elevated. The KBIT composite IQ could not be lower than 75. in the study
**Reimherr2005**	Outpatient subjects aged at least 18 years were required to meet not only DSM-IV but also the more restrictive Utah Criteria for ADHD in adults and to have a minimum score of 15 on the Wender-Reimherr Adult Attention Deficit Disorder Scale (WRAADDS). Subjects were required to have a spouse or close family member who was willing to attend visits with the patients. Additionally, subjects had to have at least moderate impairment in one area of social adjustment as measured by the Weissman Social Adjustment Scale (WSAS). Patients with a history of a single episode of major depression associated with a significant life stress were allowed in the study.	Any history of stimulant drug abuse or other recent substance abuse would exclude a patient from clinical trials involving stimulants. The Utah Criteria also exclude patients with the following characteristics or disorders:1. bipolar and depressive mood disorders 2. signs and symptoms of schizophrenic spectrum disorders 3. borderline personality disorder 4. antisocial personality disorder. In addition to the exclusion factors in the Utah Criteria, eating disorders, seizure disorders, history of significant head injury, and situational stresses that were severe enough to confuse interpretation of outcome measures were exclusionary factors. Women who were pregnant or breast feeding, subjects under custody of the criminal justice system, subjects with a history of treatment with bupropion, and subjects at risk for suicide were excluded. Finally, individuals with other axis I disorders were excluded. To avoid confounding the antidepressant effects of bupropion with its putative properties in ADHD, we were particularly concerned about excluding patients with significant depressive symptoms. Consequently, patients scoring over 15 on the Hamilton Depression Scale (HAM- D), having a score of 8 or more on the sum of HAM-D items #1, #2, #3, and #7, or meeting DSM-IV criteria for current major depression or dysthymia were excluded.
**Reimherr2007**	Current diagnosis of adult ADHD using DSM-IV-TR criteria for current ADHD based on the Conners Adult ADHD Diagnostic Interview for DSM-IV with at least moderate ADHD symptoms and	Current diagnosis of major depressive disorder, generalized anxiety disorder, panic disorder, obsessive-compulsive disorder, posttraumatic stress dis- order, bipolar disorder, schizophrenia, or other psychotic

Study name	Inclusion criteria	Exclusion criteria
	the Utah Criteria for ADHD in adults. Subjects were between 18 and 65 years of age. Female subjects were eligible to enter and participate in this study if they were of non–childbearing potential or agreed to use an approved form of contraception.	disorder. Subjects with a seizure disorder were also excluded. Subjects with hyperthyroidism or hypothyroid- ism were excluded. Finally, subjects with significant medical conditions likely to become unstable during the trial or likely to be destabilized by treatment with methylphenidate (e.g., cardiovascular disease) were excluded.
**Rosler2009**	Subjects were outpatients with ADHD aged >18 years. For study inclusion the subject had to fulfil the DSM-IV criteria for ADHD. The diagnosis was established by psychiatric expert assessment including a German version of the ADHD Rating Scale-IV (ADHD RS-IV, ADHD-DC).	Individuals with low intelligence (IQ < 85), schizophrenia, bipolar disorder, acute depressive episode, acute anxiety disorders and other unstable psychiatric conditions were excluded, as were subjects with any serious medical illness. Also subjects with evidence of drug or alcohol dependence during the preceding 6 months, pregnant or nursing women, persons who had participated in a previous drug trial in the last 30 days and individuals treated with any psychopharmacological drug in addition to study medication were not included.
**Rugino2003**	ADHD as per DSM-IV; reliable transportation to and from the development center; (2) regular school attendance; (3) an average Conners Teacher Rating Scale ADHD index *t* score of 70 or higher; (4) an average percentile score for the ADHD Rating Scale IV of 70 or higher; and (5) a verbal intelligence quotient of 80 or higher.	Acute medical or uncontrolled psychiatric illness; (2) allergy to modafinil or any of the components of the tablet; (3) mitral valve prolapse, left ventricular hypertrophy, cardiac ischemia, clinically significant cardiac arrhythmia, or history of syncope; (4) use of the following medications within 30 days before the study: psychoactive medications other than stimulants prescribed to manage ADHD, antiepileptics, or medications metabolized primarily through the hepatic cytochrome P450 system; (5) more than three migraine headaches within 3 months before the study; (6) female with potential of becoming pregnant during the study; (7) uncontrolled seizure disorder; (8) sleep disorder with insomnia; and (9) history of manic episodes or psychosis.
**Rugino2014 NCT01156051**	Sequential children 6 to 12 years of age with ADHD, who had a self- or parent-reported concern with sleep duration or quality despite adequate sleep hygiene practices and caffeine restriction. To be included in the study, the ADHDRS had to confirm the diagnosis of ADHD with >6 inattentive symptoms scoring ≥2 (often) and/or >6 hyperactivity– impulsivity symptoms scoring ≥2 (often), and the ADHD CGI-S score had to confirm at least mild severity (≥3).	Children were excluded from the study if the body mass index was less than fifth percentile for age or if the body weight was >176 pounds (80 kg). Clinically significant psychiatric pathology, such as autism, autism spectrum disorder, major depression, bipolar disorder, or anxiety, was also exclusionary. Children with clinically significant medical conditions such as hepatic, neurologic, hemodynamic, cardiac, or renal dysfunction (including clinically significant electrocardiographic findings), or with clinically significant laboratory findings were excluded. Medications and supplements with sedative, hemodynamic, or neuropsychiatric properties, or that have known drug–drug interactions with guanfacine had to be discontinued or weaned before baseline assessments. If the child had been administered medications

Study name	Inclusion criteria	Exclusion criteria
		for ADHD at the time of the screening visit, wean or discontinuation was completed so that the child was free of these medications for at least 1 week prior to completion of Although permitted by the protocol, none of the enrolled participants had been administered atomoxetine within a month prior to screening. Although obstructive sleep apnea and periodic limb movement disorders were to be exclusionary, no participant failed screening due to either of these disorders.
**Sallee2009 SPD503-304NCT0015061 8**	Male and female subjects ages 6 to 17 years with a DSM-IV-TR diagnosis of ADHD and a minimum baseline score of 24 on the ADHD Rating Scale-IV (ADHD-RS-IV) were enrolled.	Subjects were excluded for any current severe Axis I or Axis II disorders or any other current uncontrolled comorbid psychiatric diagnosis (excluding oppositional defiant disorder), weight of less than 55 lb (25 kg), morbid obesity (body mass index Q35), current use of medications that affect blood pressure (BP) or heart rate (except for ADHD therapies, which were discontinued during the washout period), hypertension or orthostatic hypotension, abnormal electrocardiogram or vital signs, previous treatment of ADHD with GXR, or intolerance of guanfacine.
**Sangal2006 B4Z-US-LYAV**	Patients were 6 to 14 years old at study entry. They were diagnosed with ADHD using the Diagnostic and Statistical Manual of Mental Disorders, Fourth Edition (DSM-IVTM) criteria as well as severity criteria. In addition, patients had an ADHD Rating Scale-IV- Parent Version: Investigator-Administered and Scored (ADHD RS) score at least 1.0 standard deviation above normative values for age and sex for either the inattentive or hyperactive/impulsive subscore, or for the combined score. All patients scored at least 80 on the Wechsler Intelligence Scale for Children 3rd edition.	Important exclusion criteria included serious medical illness, a history of symptoms suggestive of a primary sleep disorder and abnormal□laboratory values or electrocardiogram (ECG) readings.□
**Scahill2011 NCT00004376**	Both boys and girls were eligible for the study. Entry criteria included age between 7 and 15 years, a DSM-IV diagnosis of ADHD (any type), a DSM-IV tic disorder (any type), and a score of □1.5 standard deviation units for age and gender on the 10-item Conners hyperactivity index (33) rated by the teacher or a parent. To be eligible, children had to be enrolled in the same school for at least a month before entry, with no planned change in school placement for at least 10 weeks after entry.	Exclusion criteria included evidence of current major depression, generalized anxiety disorder, separation anxiety disorder, or psychotic symptoms (based on all available information); WISC- R IQ < 70; and a prior adequate trial of guanfacine (dose of □1.5 mg/day for at least 2 weeks). Subjects had to be free of all psychotropic medication for at least 2 weeks and free of any significant medical problem. Children with moderate or more severe tic symptoms (or significant obsessive-compulsive symptoms were also excluded because of their likely need for pharmacological treatment targeting these symptoms.
**Schrantee2016 NTR3103 EUCTR2010-**	ADHD according to the *DSM-IV;* Age range is 10-12 or 23-40 years of age at the time of study entry; stimulants naïve. □	Co-morbid Axis I psychiatric disorders requiring treatment with medication at study entry, and a history of major neurological or medical illness (including epilepsy, traumatic brain injury and chronic

Study name	Inclusion criteria	Exclusion criteria
**023654-37-NL**		severe tics or Tourette syndrome).□-IQ < 80 (subtest Wechsler Intelligence Scale for children-Revised (WISC-R; Wechsler 1981) or National Adult Reading Test - Current or previous treatment with medications that influence the DA system (for adults before 23 years of age) such as: neuroleptics, antipsychotics, D2/D3 agonists (pramipexole and ropinirole)□-Current or previous dependency of drugs that influence the DA system (for adults before 23 years of age), such as: MDMA, amphetamine, methamphetamine, cocaine, heroin and LSD. -Contraindications to MPH treatment: cardiovascular diseases such as hypertension, arrhythmia, hyperthyroidism, glaucoma, suicidality, psychosis, Tourette disorder. -Prenatal use of MPH by mother of the patients.□-Contraindications to MRI (metal implants, pacemakers, claustrophobia, etc.)
**Schulz2012**	Participants all met the *DSM-IV* criteria for ADHD, any subtype, on the Kiddie Schedule for Affective Disorders and Schizophrenia for School-Age Children–Present and Lifetime Version and were rated at least 1.5 SD above age and sex norms on the ADHD Rating Scale-IV-Parent Version (ADHD-RS-IV).	Poor response or tolerability to an adequate trial of either methylphenidate or atomoxetine; a substance abuse history or a positive urine screening test result; participation in a treatment study in the past 30 days; a past or present primary diagnosis of mood, anxiety, or psychotic disorder; head injury; and any medical condition that could affect brain function.
**Simonoff2013 ISRCTN68384912**	7–15 years of age; a diagnosis of ICD-10 hyperkinetic disorder; and a full-scale IQ of 30–69. Living in a stable situation and regular school attendance.	Current stimulant use; use of neuroleptic medication in the last 6 months; history of a sensitivity reaction to stimulant medication; a diagnosis of a dementing disorder; epilepsy with daily seizures; presence of a psychotic, bipolar, severe obsessive-compulsive disorder or severe Tourette syndrome; or a household resident with a current substance abuse disorder.
**Singer1995**	DSM-III criteria for ADHD and Tourette's syndrome.	Other concurrent medications.
**SPD489-405 NCT01552915**	• Subject must be 13-17 years of age, inclusive, at the time of consent.<br>• Subject must weigh more than 79.5lb.<br>• The parent/LAR must be available at approximately 7:00AM (±2 hours) to dispense the dose of investigational product for the study duration.<br>• Subject, who is a female, must have a negative serum beta human chorionic gonadotropin (β-HCG) pregnancy test and a negative urine pregnancy test and agree to comply with any applicable contraceptive requirements of the protocol.	• Subject has a current, controlled (with medications prohibited in this study) or uncontrolled, comorbid psychiatric diagnosis with significant symptoms such as any significant comorbid Axis II disorder or significant Axis I disorder (such as post traumatic stress disorder, psychosis, bipolar illness, pervasive developmental disorder, severe obsessive compulsive disorder, depressive or anxiety disorder.<br>• Diagnosis of conduct disorder. Oppositional defiant disorder is not exclusionary.<br>• Subject is considered a suicide risk, has previously made a suicide attempt, or is currently demonstrating active suicidal

Study name	Inclusion criteria	Exclusion criteria
	• Subject has an ADHD-RS-IV total score ≥28.<br>• Subject is able to swallow a capsule.<br>• Subject does not have hypertension and has a resting sitting blood pressure less than or equal to 135/85mmHg.	ideation. Subjects with intermittent passive suicidal ideation are not necessarily excluded.<br>• Subject is underweight or overweight.<br>• Subject has a concurrent chronic or acute illness (such as severe allergic rhinitis or an infectious process requiring antibiotics), disability, or other condition. Mild, stable asthma is not exclusionary.<br>• Subject has a history of seizures (other than infantile febrile seizures), a chronic or current tic disorder, or a current diagnosis and/or a known family history of Tourette's Disorder.<br>• Subject has a known history of symptomatic cardiovascular disease, advanced arteriosclerosis, structural cardiac abnormality, cardiomyopathy, serious heart rhythm abnormalities, coronary artery disease, or other serious cardiac problems that may place him/her at increased vulnerability to the sympathomimetic effects of a stimulant medication.<br>• Subject has a known family history of sudden cardiac death or ventricular arrhythmia.<br>• Subject has any clinically significant ECG or clinically significant laboratory abnormality.<br>• Subject has current abnormal thyroid function, defined as abnormal thyroid stimulating hormone (TSH) and thyroxine (T4). Treatment with a stable dose of thyroid medication for at least 3 months is permitted.<br>• Subject has a documented allergy, hypersensitivity, or intolerance to amphetamine or to any excipients in the investigational product.<br>• Subject has a documented allergy, hypersensitivity, or intolerance to MPH or to any excipients in the reference product.<br>• Subject has failed to fully respond to an adequate course(s) (dose and duration) of MPH or amphetamine therapy.<br>• Subject has a history of suspected substance abuse or dependence disorder (excluding nicotine). Subjects with a lifetime history of amphetamine, cocaine, or other stimulant abuse and/or dependence will be excluded.

Study name	Inclusion criteria	Exclusion criteria
		• Subject has a positive urine drug result.<br>• Subject has previously participated in this study or another clinical study involving SPD489/NRP104.<br>• Subject has glaucoma.<br>• Subject is required to take or anticipates the need to take medications that have CNS effects or affect performance, such as sedating antihistamines and decongestant sympathomimetics, or are monoamine oxidase inhibitors. Stable use of bronchodilator inhalers is not exclusionary.<br>• Subject is female and is pregnant or lactating.<br>• Subject is well controlled on his/her current ADHD medication.<br>Subject has a pre-existing severe gastrointestinal tract narrowing.
**SPD489-406 NCT01552902**	Subject must be 13-17 years of age, inclusive, at the time of consent.<br>Subject must weigh more than 79.5lb.<br>The parent/LAR must be available at approximately 7:00AM (±2 hours) to dispense the dose of investigational product for the study duration.<br>Subject, who is a female, must have a negative serum beta human chorionic gonadotropin (β-HCG) pregnancy test and a negative urine pregnancy test and agree to comply with any applicable contraceptive requirements of the protocol.<br>Subject has an ADHD-RS-IV total score ≥28.<br>Subject is able to swallow a capsule.<br>Subject does not have hypertension and has a resting sitting blood pressure less than or equal to 135/85mmHg.	• Subject has a current, controlled (with medications prohibited in this study) or uncontrolled, comorbid psychiatric diagnosis with significant symptoms such as any significant comorbid Axis II disorder or significant Axis I disorder (such as post traumatic stress disorder, psychosis, bipolar illness, pervasive developmental disorder, severe obsessive compulsive disorder, depressive or anxiety disorder.<br>• Diagnosis of conduct disorder. Oppositional defiant disorder is not exclusionary.<br>• Subject is considered a suicide risk, has previously made a suicide attempt, or is currently demonstrating active suicidal ideation. Subjects with intermittent passive suicidal ideation are not necessarily excluded.<br>• Subject is underweight or overweight.<br>• Subject has a concurrent chronic or acute illness (such as severe allergic rhinitis or an infectious process requiring antibiotics), disability, or other condition. Mild, stable asthma is not exclusionary.<br>• Subject has a history of seizures (other than infantile febrile seizures), a chronic or current tic disorder, or a current diagnosis and/or a known family history of Tourette's Disorder.<br>• Subject has a known history of symptomatic cardiovascular disease, advanced arteriosclerosis, structural cardiac abnormality, cardiomyopathy, serious heart rhythm abnormalities, coronary artery disease, or other serious

Study name	Inclusion criteria	Exclusion criteria
		cardiac problems that may place him/her at increased vulnerability to the sympathomimetic effects of a stimulant medication.<br>• Subject has a known family history of sudden cardiac death or ventricular arrhythmia.<br>• Subject has any clinically significant ECG or clinically significant laboratory abnormality.<br>• Subject has current abnormal thyroid function, defined as abnormal thyroid stimulating hormone (TSH) and thyroxine (T4). Treatment with a stable dose of thyroid medication for at least 3 months is permitted.<br>• Subject has a documented allergy, hypersensitivity, or intolerance to amphetamine or to any excipients in the investigational product.<br>• Subject has a documented allergy, hypersensitivity, or intolerance to MPH or to any excipients in the reference product.<br>• Subject has failed to fully respond to an adequate course(s) (dose and duration) of MPH or amphetamine therapy.<br>• Subject has a history of suspected substance abuse or dependence disorder (excluding nicotine). Subjects with a lifetime history of amphetamine, cocaine, or other stimulant abuse and/or dependence will be excluded.<br>• Subject has a positive urine drug result.<br>• Subject has previously participated in this study or another clinical study involving SPD489/NRP104.<br>• Subject has glaucoma.<br>• Subject is required to take or anticipates the need to take medications that have CNS effects or affect performance, such as sedating antihistamines and decongestant sympathomimetics, or are monoamine oxidase inhibitors. Stable use of bronchodilator inhalers is not exclusionary.<br>• Subject is female and is pregnant or lactating.<br>• Subject is well controlled on his/her current ADHD medication.<br>Subject has a pre-existing severe gastrointestinal tract narrowing.

Study name	Inclusion criteria	Exclusion criteria
**Spencer1995**	Between 18-60 y; DSM-III-R diagnosis of ADHD. Other comorbid disorders were not exclusionary, unless they were associated with a contraindication to methylphenidate.	Medical conditions, abnormal laboratory values, tic disorders, IQ < 75, organic brain disorders, unstable psychiatric conditions, substance or alcohol abuse or dependence within 6 months prior to the stud; pregnant women.
**Spencer1998**	Adults with ADHD between 19 and 60 years of age.	Clinically significant chronic medical conditions, abnormal baseline laboratory values, mental retardation (IQ less than 75), organic brain disorders, clinically unstable active psychiatric conditions, drug or alcohol abuse within the last 6 months, current use of psychotropics, and, for women, pregnancy or nursing.
**Spencer2001**	19-60 years. DSM-IV diagnosis of ADHD.	Clinically significant chronic medical conditions, abnormal baseline laboratory values, mental retardation (IQ less than 80), organic brain disorders, clinically unstable active psychiatric conditions, drug or alcohol abuse within the last 6 months, current use of psychotropics, and, for women, pregnancy or nursing. Previous adequate trial of Aderall.
**Spencer2002a,b**<br>**B4Z-MC-HFBD**<br>**B4Z-MC-HFBK**	7-13 years; normal intelligence; DSM-IV diagnosis of ADHD; ADHD-RS scores at least 1.5 SD above cut-off; comorbid depression or anxiety disorders: not exclusionary; stimulant naïve (not clear if this was a required inclusion criterion).	Poor metabolizers of CYP2D6; less than 25 Kg; Bipolar I or II; psychosis; organic brain disease; seizure; current psychotropic medication; history of alcohol or drug abuse within 3 months; significant current or prior medical conditions.
**Spencer2005**	Subjects had to satisfy full diagnostic criteria for DSM-IV ADHD based on clinical assessment and confirmed by structured diagnostic interview.	Clinically significant chronic medical conditions; abnormal base- line laboratory values; IQ 80; delirium, dementia, or amnestic disorders; other clinically unstable psychiatric conditions (i.e., bipolar disorder, psychosis, suicidality); drug or alcohol abuse or dependence within the 6 months preceding the study; previous adequate trial of stimulant (0.5 mg/kg/day of MPH or equivalent); or current use of other psychotropics. We also excluded pregnant or nursing women.
**Spencer2006**<br>**SLI381-314**<br>**NCT00507065**	Adolescents aged 13 to 17 years, weighing -<75kg (-<165 lb), who satisfied *DSM-IV-TR 1* criteria for primary diagnosis of ADHD combined subtype (predominantly inattentive subtype or hyperactive-impulsive subtype), were eligible for the study. Key inclusion criteria were an intelligence quotient score > 80,normal blood pressure (girls--systolic blood pressure, 128- 132 mm Hg; diastolic blood pressure, 84-86 mm Hg; boys--systolic blood pressure, 130-140 mm Hg; diastolic blood pressure, 84-89 mm Hg),2s electrocardiographic (ECG) findings within the normal range, and a willingness and ability to comply with protocol requirements in conjunction with a parent or caregiver.	comorbid illness that could interfere with study participation or impact the efficacy and tolerability of MAS XR; a history of non- response to stimulant medication; a documented allergy or intolerance to MAS, MAS XR, or amphetamines; and medication use (not including ADHD medication) that could affect blood pressure or heart rate. Other exclusion criteria included a current co- morbid psychiatric diagnosis except oppositional defiant disorder, hypertension, history of seizure disorder within the last 2 years, tic disorder, Tourette's syndrome, abnormal thyroid function, cardiac disorder, and significant laboratory abnormalities. In addition, patients with a history of drug abuse or who were current abusers of drugs or other substances or who had a

Study name	Inclusion criteria	Exclusion criteria
	Adolescents who were known to be nonresponsive to stimulants (defined as no clinical improvement after trials of 2 stimulant medications, taken for at least 3 weeks each) or naive to stimulant treatment were eligible for enrollment.<br>Note: “Adolescents who were known to be nonresponsive to stimulants” contrasts with exclusionary criterion; author contacted but no reply.	parent or guardian who abused drugs were excluded.
**Spencer2007 CRIT124E2302**	Participants eligible for inclusion were aged 18 to 60 years, diagnosed with DSM-IV ADHD (any subtype) with childhood onset of symptoms. They had to have a DSM-IV ADHD Rating Scale (ADHD-RS) total score of at least 24 at screening and baseline. In addition, they were required to display functional impairment, defined as a Global Assessment of Functioning (GAF) score of 60 or less.	Patients with a history of alcohol or substance abuse within the last 6 months were excluded, as were patients with any psychiatric or medical comorbidity that may have interfered with study participation or assessments or for which MPH treatment may have posed a risk. Patients were also excluded if the investigator judged that they had a history of poor response or intolerance to stimulants (e.g., MPH, d-MPH, amphetamine salts, or dextroamphetamine salts). No patient had previously used d-MPH-ER. Women were excluded if they were pregnant, nursing, or not using acceptable methods of contraception.
**Spencer2008 SPD465-301 NCT00150579**	Men or non pregnant/non lactating women (women of childbearing age agreed to use acceptable methods of contraception throughout the study) between the ages of 18 and 55 years, inclusive; meet the DSM-IV-TR criteria for a primary diagnosis of ADHD; have a satisfactory medical assessment with no clinically significant or relevant abnormalities; have a baseline ADHD Rating Scale-IV (ADHD- RS-IV) score ≥ 24; and provide informed consent.	Subjects were excluded if they had a body mass index < 18.5 kg/m^2; morbid obesity; comorbid psychiatric diagnosis with, in the opinion of the investigator, significant symptoms; seizure history, tic disorder, or diagnosis or family history of Tourette’s syndrome; current chronic or acute illness or an unstable medical condition; mental retardation; known cardiac structural abnormality or any other cardiac condition that could affect cardiac performance; clinically significant electrocardiogram (ECG) or laboratory abnormalities at screening; used psychotropic medications that require more than a 28-day washout period; a history of controlled or uncontrolled hypertension or a resting, sitting systolic blood pressure > 139 mm Hg or diastolic blood pressure > 89 mm Hg at screening; allergy, intolerance, or nonresponse to methylphenidate or amphetamines; drug dependence or substance use disorder (excluding nicotine) within 6 months before screening; a positive urine drug test result at screening or baseline; participation in another investigational trial within 30 days of screening; or pregnancy or lactation. The concomitant use of psychoactive medications that, in the opinion of the investigator, could interfere with the efficacy, safety, or tolerability of triple-bead MAS was not al- lowed during the study.

Study name	Inclusion criteria	Exclusion criteria
**Stein2011 NCT00393042**	DSM-IV diagnosis of ADHD (mistake in the text)	Youth with mental retardation, autism, severe mood disorders, Tourette's disorder, seizure disorders, or other medical disorders that were contraindications of stimulant treatment or that mimic ADHD (e.g., thyroid disorder) were excluded.
**Sutherland2012 NCT00174226**	Adults aged 18 to 60 years who met the *Diagnostic and Statistical Manual of Mental Disorders*, Fourth Edition, Text Revision *DSM-IV-TR*) criteria for ADHD, via the Adult ADHD Clinician Diagnostic Scale version 1.2, and scored ≥24 on the adult ADHD Investigator Symptom Rating Scale (AISRS).	Lifetime or current history of psychosis, bipolar disorder, mental retardation or learning disability; had current anxiety or depressive disorders; had substance abuse or dependence within 3 months of screening or positive urine screen for drugs of abuse at screening; used atomoxetine, buspirone, or a monoamine oxidase inhibitor within 2 weeks prior to screening; had seizure disorder, urinary retention, narrow-angle glaucoma, or cardiac conduction defects; had any current general medical conditions considered clinically significant as judged by the investigator; or were poor metabolizers of cytochrome P450 2D6 (CYP2D6). Use of substances with psychoactive properties and potent CYP3A4 or CYP2D6 inducers or inhibitors was prohibited.
**Svanborg2009 B4Z-SO-LY15 EUCTR2004-003941-42-SE NCT00191542**	Male and female patients 7–15 years of age were included if they met the criteria for ADHD of the Diagnostic and Statistical Manual of Mental Disorders, Fourth Edition (DSM- IV) and had a severity threshold of 1.5 standard deviations above the US age and gender norms for their diagnostic subtype on the ADHD rating scale- parent version: Investigator Administered and Scored. Eligible patients had to be stimulant-naıve.	General impairment of intelligence, as clinically assessed by the investigator, serious medical illness, a history of psychosis or bipolar disorder, alcohol or drug abuse within the previous 3 months, or ongoing use of psychoactive medication other than the study drug. Patients who required immediate pharmacotherapy or structured psychotherapy were also excluded.
**Swanson2006**	6-17 years; DSM-IV diagnosis of ADHD: CGI-S> 4; ADHD-RS total score at least > 1.5 SD; IQ> 80; full time school.	PDD or psychosis, suicide risk or other conditions requiring immediate treatment; those satisfied with current ADHD treatment and those who fail to respond to 2 or more adequate courses of stimulant therapy, with trials on a range of doses and immediate and controlled-release formulations.
**Takahashi2009 B4Z-JE-LYBC NCT00191295**	Japanese children and adolescents who were at least 6 years old but younger than 18 years of age were eligible to participate if: (1) they met the DSM-IV criteria for ADHD by clinical assessment (American Psychiatric Association 1994) and (2) their diagnosis was confirmed in structured interviews with investigators using the behavior module for ADHD of the Kiddie Schedule for Affective Disorders and Schizophrenia for School- Aged Children–Present and Lifetime Versions (K- SADS-PL). Also, patients had to have a Clinical Global Impressions–ADHD-Severity (CGI-ADHD-S) assessment score 3 and a symptom severity score at least 1.5	Important exclusion criteria included patients who took any antipsychotic medication within 26 weeks of study visit 1, had a history of bipolar disorder or psychosis, or were determined by the investigator to be at suicidal risk.

Study name	Inclusion criteria	Exclusion criteria
	standard deviations (SD) above Japanese pediatric age and gender norms on the Attention-Deficit=Hyperactivity Disorder Rating Scale-IV–Parent Version:Investigator Administered and Scored=Translated and Validated in Japanese (ADHD RS-IV- J:I). Patients were also required to be of normal intelligence (IQ 80).	
**Takahashi2014 NCT01323192**	Between 18 and 64 years of age, who met the DSM-IV Text Revision (DSM-IV-TR) criteria for ADHD both at present and in childhood (onset of symptoms before the age of 7 years according to DSM-IV-TR criteria) based on Conners' Adult ADHD Diagnostic Interview for DSM-IV (CAADID) Japanese version at screening. The CAADID assesses patients based on the 18 symptoms criteria for ADHD contained in the DSM-IV. Also required to have a DSM-IV Total ADHD Symptoms subscale score of 24 at baseline on the investigator-rated Conners' Adult ADHD Rating Scale-Observer: Screening Version.	Patients were excluded from the study if they were a non-responder to MPH and/or had a history of hypersensitivity or intolerance to MPH or had been treated with MPH or any other medications for ADHD within 4 weeks before the screening visit. Other exclusion criteria included diagnosis of bipolar I dis- order, schizophrenia, schizoaffective disorder, severe obsessive-compulsive disorder, pervasive developmental disorder (e.g., autistic disorder or Asperger's disorder) or suicidality. Patients with confirmed cancer or other serious illnesses (e.g., hepatic or renal insufficiency or significant cardiac, gastrointestinal, psychiatric, or metabolic disturbances) were also excluded.
**Taylor1987**	IQ> 65, lived at home primary school, naïve to stimulant. The diagnoses were various: 7(18%) were diagnosed as 'hyperkinetic syndrome' in the ICD-9 scheme; 26(68%) as 'conduct disorder'; 2(5%) as 'relationship problems'; and for 3 children the presence of misery or anxiety led to a diagnosis of disturbance of emotions specific to childhood' in spite of the presence of other problems. When the definitions of DSM-III were applied, 24(63%) were included as 'attention deficit disorder with hyperactivity'. (note: only data on drop outs from this study were used since data were available on completers rather than ITT).	Autistic features, neurologic signs.
**Taylor2000**	DSM-IV diagnosis of ADHD; scoring above 93rd percentile of DSM-IV ADHD Behavior Checklist	Narcolepsy and conditions associated with altered cognitive abilities including schizophrenia, Tourette's disorder, and diagnosable neurologic conditions. Medical conditions likely to effect mood and cognition, such as metabolic disorders, mental retardation, untreated endocrine disorders, and pregnancy precluded entry into the study. Subjects using any cannabis, cocaine, heroin, or non prescription amphetamines within 6 months of beginning drug trial were excluded. Subjects taking tricyclic antidepressants, venlafaxine, or bupropion within 3 months of starting the study or prescription stimulants within 2 weeks prior to the beginning of the study were excluded.

Study name	Inclusion criteria	Exclusion criteria
**Taylor2001**	DSM-IV criteria of ADHD. Scoring above the 93rd percentile of the ADHD Behavior Checklist for Adults.	Exclusion criteria consisted of conditions already associated with frontostriatal pathology, including organic brain disorders, schizophrenia, and Tourette disorder. Besides a basic neurologic examination, tests that screen for subtle neurologic soft signs were used to identify and exclude subjects with psychopathology possibly caused by neurologic insult. Medical conditions likely to effect mood or cognition, such as metabolic disorders, central nervous system conditions, mental retardation, untreated endocrine disorders, and pregnancy precluded entry into the study. Subjects using substances such as cannabis, amphetamines, cocaine, and heroin within 6 months of beginning drug trials were excluded. Subjects taking tricyclics, venlafaxine, or bupropion within 3 months, or stimulants within 2 weeks.
**VanDerMeere1999**	DSM-III-R diagnosis of ADHD.	To avoid carry-over effects, none of the children had used stimulant drugs or clonidine or psychoactive medications of any kind during the 6 months prior to entering the study. Additional psychoactive drugs were not allowed during the trial.
**Wang2007 NCT00486083 B4Z-MC-LYBR(6934)**	Eligible participants included outpatient children and adolescents, 6 16 years of age, weighing between 20 and 60 kg, who met the Diagnostic and Statistical Manual of Mental Disorders, 4th edition (DSM-IV) criteria for ADHD as assessed by clinical interview and confirmed by structured diagnostic interview using the Kiddie Schedule for Affective Disorders and Schizophrenia for School-Aged Children-Present and Lifetime Version (K-SADS-PL). All patients were required to meet the following symptom severity thresholds: a score of ≥> 25 for boys or ≥> 22 for girls, or /12 for a specific subtype, on the Attention Deficit Hyperactivity Disorder Rating Scale-IV-Parent Version: Investigator-Administered and -Scored (ADHDRS-IV- Parent:Inv), as well as a Clinical Global Impressions-Attention Deficit Hyperactivity Disorder-Severity (CGI-ADHD-S [28]) score of ≥/4.	Exclusion criteria included any history of bipolar, psychotic or pervasive developmental disorders; suicidal risk; or ongoing use of psychoactive medications other than the study drug. Patients with motor tics, a diagnosis or family history of Tourette's syndrome or those who met DSM-IV criteria for anxiety disorder as assessed by the investigator and confirmed by the K-SADS-PL were also excluded from participating.
**Wehmeier2012 B4Z-SB-LYDV NCT00546910**	Eligible were girls and boys aged 6 to 12 years with a diagnosis of ADHD according to Diagnostic and Statistical Manual of Mental Disorders, Fourth Edition, Text Revision, criteria. Psychotherapy initiated before the study was acceptable.	Exclusion criteria comprised previous treatment with ATX, treatment with psychotropic medication other than the study drug, clinically relevant overweight and underweight, a history of bipolar disorder, psychosis, pervasive developmental disorder, seizure disorder (other than febrile seizures), serious suicidal risk, and other relevant acute or unstable medical condition.

Study name	Inclusion criteria	Exclusion criteria
**Weisler2006 SLI381-303**	Subjects were outpatients >18 years of age who were referred by clinics and had a primary diagnosis of ADHD established by psychiatric evaluation using *DSM-IV-TR* criteria. Subjects were in good physical health, with normal vital signs and 12-lead electrocardiogram (ECG) measurements.	Subjects incapable of following study instructions or having an intelligence quotient <80 (Kaufman Brief Intelligence Test) were excluded from the study. Several comorbid psychiatric diagnoses were excluded: psychosis, bipolar ill- ness, pervasive developmental disorder, severe obsessive-compulsive disorder, and severe depressive (17-item Hamilton Rating Scale for Depression score >19) and anxiety disorders. (14-item Hamilton Rating Scale for Anxiety score >17). Subjects were excluded for a positive drug screen or substance abuse history (or living with someone with a substance abuse disorder); glaucoma; hyperthyroidism; seizure, tic disorder, or Tourette syndrome; and pregnancy or lactation. Also excluded were subjects who were taking within 30 days of the screening visit any anticonvulsant drugs, clonidine, guanfacine, systemic steroids, medications that affect blood pressure (BP) or the heart or have central nervous system effects, pemoline, or investigational drugs.
**Weisler2012 NCT00880217**	Men and women (aged 18–55 years) who met the following inclusion criteria: (a) an established DSM-IV-TR diagnosis of ADHD as confirmed by the Conners Adult ADHD Diagnostic Interview for DSM-IV (CAADID); (b) a Clinical Global Impression-Severity (CGI-S) score of ≥4 at screening and baseline; and (c) a Conners Adult ADHD Rating Scale Self-Report: Screening Version (CAARS-S:SV) DSM-IV ADHD Total Symptoms subscale score depending on age and gender (18–39 years: ≥26 men and ≥32 women; ≥40 years: ≥29 men and ≥27 women) to ensure adequate symptom severity at baseline.	any current Axis I psychiatric condition including major depressive disorder, bipolar disorder, schizophrenia, generalized anxiety disorder, obsessive-compulsive disorder, post-traumatic stress disorder, borderline personality disorder, or eating disorder; taken any mood stabilizer, antipsychotic, antidepressant or anxiolytic within 3 months prior to screening; history of a previous suicide attempt, participants currently experiencing acute suicidal ideation or behaviour; history of alcohol or substance use disorder within 6 months prior to screening (nicotine and caffeine dependence were not exclusionary) or positive result for urine drug screen at screening or baseline; known or suspected mental retardation; and demonstrated history of non-response to treatment with a psychostimulant medication or to treatment with atomoxetine or methylphenidate.
**Weiss2005 4Z-MC-LYAW**	Children aged 8 to 12 years with ADHD (any subtype) as defined by DSM-IV were eligible to participate. Symptom severity had to be at least 1.0 SD above age and sex norms on the Attention-Deficit/Hyperactivity Disorder Rating Scale-IV-Teacher Version: Investigator administered and scored (ADHDRS-IV-Teacher: Inv). Patients were also required to have a mean Conners Parent Rating Scale (CPRS-R:S) ADHD Index score at least 1.5 SDs above age and sex norms. Children with concurrent learning disorders were included.	Important exclusion criteria included unavailability of a primary teacher willing to keep telephone appointments and to provide ratings and reports as part of the study, evidence of a significant intellectual deficit, serious medical illness, or use of other psychotropic medication.

Study name	Inclusion criteria	Exclusion criteria
**Wender2011**	Utah Criteria, which corresponds to *DSM-IV* ADHD, Combined Type; 21-55 years.	Other Axis I and Axis II diagnoses were excluded.
**Wietecha2013 NCT00607919**	Subjects with ADHD + D and ADHD-only met Diagnostic and Statistical Manual of Mental Disorders, 4th ed., Text Revision diagnostic criteria for ADHD. At visits 2 and 3, subjects with ADHD + D and ADHD-only also had an ADHD Rating Scale-IV-Parent-Version:Investigator-Administered and Scored (ADHDRS-IV-Parent:Inv) Total score ≥1.5 standard deviations above age and gender norms.	Excluded were subjects with a documented history of bi-polar I or bipolar II disorder, psychosis, autism, Asperger's syndrome, or pervasive developmental disorder, and subjects who were currently taking anticonvulsants for seizure control.
**Wigal2004**	ADHD was diagnosed using the *DSM-IV* criteria for the three subtypes (predominantly inattentive, predominantly hyperactive/impulsive, or combined) and was confirmed by the National Institute of Mental Health's Diagnostic Interview Schedule for Children (DISC-IV) administered to parents. Patients were eligible to participate in the study if they were enrolled in elementary school, were within 30% of normal body weight, and anticipated being available for the entire length of the study. Female subjects were required to be premenarche.	Patients were excluded from the study if they had a history or evidence of cardiovascular, renal, respiratory (other than asthma/allergy), endocrine, or immune-system disease; a history of substance abuse; hypersensitivity to *d,l*-MPH or other stimulants; or treatment with any investigational drug within 30 days of screening. Exclusion criteria also included any other significant central nervous system disorders, such as mental retardation; Tourette's or chronic tic disorder; psychosis; pervasive developmental disorder; eating disorders; obsessive-compulsive disorder, impulse control disorder, or sleep disorders requiring medication; major depressive disorder; or generalized anxiety disorder. Patients treated with the following medications were excluded from the study: antidepressants (tricyclic antidepressants, serotonin reuptake inhibitors, and monoamine oxidase inhibitors), sedatives/hypnotics (e.g., barbiturates, benzodiazepine), neuroleptics/antipsychotics, mood stabilizers; anticonvulsants, beta-blockers; α_2-agonists, thyroid medications, and chronic oral steroids.
**Wigal2005 SLI381-404 NCT00506727**	(a) male or female aged 6 to 12 years; (b) diagnosis of *Diagnostic and Statistical Manual of Mental Disorders* (4th ed., Text Revision *DSM-IV-TR*; American Psychiatric Association, 2000) ADHD combined subtype or predominantly hyperactive/impulsive subtype; (c) weight between 40 lb (18.18 kg) and 120 lb (54.54 kg) at enrollment; and (d) capable of understanding and following classroom instruction and generally functioning academically at age-appropriate levels.	*DSM-IV-TR* diagnosis of ADHD, predominantly (b) current controlled or uncontrolled comorbid psychiatric diagnosis (except oppositional defiant disorder) with significant symptoms such as pervasive developmental disorder, post-traumatic stress disorder, psychosis, bipolar illness, severe obsessive-compulsive disorder, severe depression, or severe anxiety disorder; (c) documented history of aggressive behavior serious enough to preclude participation in regular classroom activities, or a *DSM-IV-TR* diagnosis of conduct disorder; (d) documented allergies, adverse reactions, or intolerance of stimulants, including MAS XR, atomoxetine, or tricyclic antidepressants, or a history of failure to respond clinically to adequate doses of these medications; (e) history of suspected substance abuse

Study name	Inclusion criteria	Exclusion criteria
		or drug abuse (excluding nicotine) or living with someone with such history or suspicion; (f) taking any prohibited medication including antidepressants, antipsychotics, neuroleptics, anxiolytics, and anticonvulsants; and (g) history of seizure during the past 2 years, a tic disorder, or a family history of Tourette's disorder.
**Wigal2015 NCT01239030**	Children and adolescents (male and female) aged 6–18 years at time of consent with an ADHD diagnosis of all subtypes (except Not Otherwise Specified) as defined in the Diagnostic and Statistical Manual of Mental Disorders, 4th Edition, Text Revision (DSM-IV-TR). Recorded baseline ADHD-RS-IV total or subscale scores had to be >90th percentile relative to the general population of children by age and sex at screening or baseline. Patients had to require pharmacological treatment for ADHD.	Exclusion criteria included an Estimated Full Scale intellectual level <80 using the four-subtest form of the Wechsler Abbreviated Scale of Intelligence (WASI), and a current primary psychiatric diagnosis of severe anxiety disorder, conduct disorder, psychotic disorder, pervasive developmental disorder, eating disorder, obsessive-compulsive disorder, major depressive disorder, bipolar disorder, substance use disorder, chronic tic disorder, or a personal or family history of Tourette's syndrome as defined by the DSM- IV-TR criteria and supported by the K-SADS-PL. Patients with a chronic medical illness (seizure, cardiac disorders, untreated thyroid disease, glaucoma), using monoamine oxidase inhibitors or psychotropic medication within 14days of screening or another experimental drug or device within 30 days of screening, who had a clinically significant electrocardiogram (ECG) or clinical laboratory abnormality at screening and/or baseline, or who were pregnant or lactating were also excluded from the study.
**Wilens2001**	DSM-IV ADHD diagnosis; 20-59 years.	Any clinically significant chronic medical conditions, a history of cardiac arrhythmias or seizures, mental retardation (IQ <75), organic brain disorders, clinically unstable psychiatric conditions, bipolar dis- order, drug or alcohol abuse or dependence within the 6 months preceding the study, or current use of psychotropics.
**Wilens2005 NCT00048360**	Men and women aged 18 to 60 years were eligible for the study if they met criteria for a current diagnosis of ADHD (all types) as defined by the Diagnostic and Statistical Manual of Mental Disorders, 4th Edition (DSM-IV). Subjects were required to have met full DSM-IV criteria for a diagnosis of ADHD by age 7 (as determined by the Schedule for Affective Disorders and Schizophrenia for School-Age Children, Epidemiologic Version 5), with a chronic course of ADHD from childhood to adulthood. In addition, subjects were required to have a moderate to severe level of impairment due to symptoms of ADHD at the randomization visit, with a minimum score of 4 (moderately ill) out	Subjects with a current diagnosis of major depressive disorder; a current or lifetime diagnosis of bipolar or psychotic disorders; a current primary diagnosis of panic disorder, obsessive-compulsive disorder, posttraumatic stress r acute stress disorder; or who met criteria for alcohol or substance abuse within the last year were excluded. Subjects were also excluded if they were found during medical examination or interview to have an unstable medical disorder or a predisposition to seizures. In addition, subjects were queried during screening regarding previous pharmacotherapy for ADHD; those with a reported history of inadequate response to bupropion (for the treatment of ADHD) or inadequate responses to two or more adequate

Study name	Inclusion criteria	Exclusion criteria
	of 7 on the Clinical Global Impression-Severity of Illness (CGI-S) scale as well as a 25 out of 54 on the investigator-rated ADHD Rating Scale (ADHD-RS). In addition, subjects were required to be in good general health based on physical and laboratory examinations and medical history. Premenopausal women, except those who had undergone surgical sterilization, were required to use a reliable form of contraception having a 1% failure rate throughout the study period. A history of past successful treatment with bupropion or psychostimulants was not exclusionary.	trials of psychostimulants were not eligible. The use of psychoactive drugs, including benzodiazepines and psychostimulants, alpha-adrenergic antihypertensives, or beta-adrenergic antagonists, within 1 week of randomization was prohibited. The use of any potentially psychoactive herbal or nutritional supplements, 5-hydroxytryptophan or 5-hydroxy-1-tryptophan, anticonvulsants, antidepressants (excluding fluoxetine), or lithium within 2 weeks of randomization or fluoxetine within 4 weeks of randomization also was prohibited. Subjects with positive blood tests for alcohol or urine tests for substances of abuse at screening were not eligible for the study.
**Wilens2008 B4Z-MC-LYBY NCT00190957**	Subject ≥18 years of age meeting DSM-IV-TR criteria for ADHD (any subtype), determined by clinical interview and confirmed by the Adult ADHD Clinician Diagnostic Scale. ADHD symptom severity was ≥20 on the ADHD Investigator Symptom Rating Scale (AISRS). Subjects also met Diagnostic and Statistical Manual of Mental Disorders, Fourth Edition, Text Revision criteria for alcohol use disorders (abuse or dependence). Other substance use histories did not preclude participation provided the primary substance which the patient abused or had dependence (as judged by the investigator) was alcohol and subjects were not actively abusing other substances at study entry. This study focused on very recently abstinent adults at high relapse risk to heavy alcohol use; hence, all subjects were alcohol-free for at least 4 days before randomization but not longer than 30 days. The minimum four abstinent days had to be consecutive and overlap with the week before randomization. Psychotherapy, pharmacological, or other interventions for substance abuse (other than 12-step participation) were not permitted.	Exclusion criteria included diagnosis of current bipolar disorder, major depressive disorder, or psychosis as determined by Structured Clinical Interview for DSM-IV-TR Axis I Disorders or Hamilton Depression Rating Scale (HAM-D-17) or Hamilton Anxiety Scale (HAM-A) scores >18 at the evaluation visit. Subjects with significant cognitive impairment, judged by the investigator, were excluded. No other psychopharmacological treatments were permitted during the study, other than limited, intermittent hypnotic use.
**Wilens2011 NCT00528697**	Male and female children aged 6 through 12 years, with a DSM-IV diagnosis of any ADHD subtype, confirmed by the Kiddie Schedule for Affective Disorders and Schizophrenia for School-Age Children– Present and Lifetime Version (K-SADS-PL), and a rating of 4 or higher on the Clinical Global Impression– ADHD-Severity Scale (CGI-ADHD-S).	Exclusion criteria included the following: current or past diagnosis of bipolar I, II, or not otherwise specified (NOS) disorder; psychotic disorder; autism, Asperger's syndrome, or pervasive developmental disorder; tics or Tourette's syndrome; seizure disorder; traumatic brain injury; current diagnosis of obsessive- compulsive disorder, eating disorder, anxiety disorder, or depressive disorder requiring treatment of any kind; psychotropic medications within 14 days or five half-lives (7 days for stimulants), whichever was longer, before the Day 1 ADHD Rating Scale-IV: Home Version (ADHD-RS-IV [HV]) assessment; in Study 1, atomoxetine within 3 months of randomization or not a

Study name	Inclusion criteria	Exclusion criteria
		suitable candidate to receive atomoxetine. Failure to respond to two or more adequate trials of U.S. Food and Drug Administration–approved ADHD medication was also exclusionary.
**Wilens2015 SPD503-312 EUCTR2011-002221-21 NCT01081132**	Adolescent outpatients aged 13 to 17 years with a diagnosis of ADHD (any subtype). Consistent with DSM-IV-TR criteria, a primary ADHD diagnosis was confirmed by clinical evaluation using the behavior module of the Kiddie Schedule for Affective Disorders and Schizophrenia–Present and Lifetime version (K-SADS-PL) at screening (visit 1). Participants were also required to have a minimum ADHD-RS-IV total score of 32 and a minimum CGI-S score of 4 at baseline (visit 2). Supine and standing blood pressure measurements within the 95th percentile for age, sex, and height were also required.	Any current controlled or uncontrolled comorbid psychiatric diagnosis (except oppositional defiant disorder), including severe comorbid Axis II disorders or severe Axis I disorders, such as anxiety disorder, posttraumatic stress disorder, depression, bipolar illness, psychosis, pervasive developmental disorder, obsessive-compulsive disorder, substance abuse disorder within 6 months, or other symptomatic manifestations or lifetime history of bipolar or unipolar illness (e.g., active suicidality), psychosis, or conduct disorder that, in the opinion of the investigator, contraindicated treatment with GXR or could confound efficacy or safety assessments. Other exclusion criteria included history/presence of structural cardiac abnormalities, serious heart rhythm abnormalities, syncope, cardiac conduction problems, exercise-related cardiac events, orthostatic hypotension, history of controlled or uncontrolled hypertension, or clinically significant bradycardia. Participants who used any medications that affect blood pressure or heart rate, have central nervous system effects, or affect cognitive performance (such as sedating antihistamines) were also excluded.
**Winhusen2010 NCT00253747**	Eligible participants were interested in quitting smoking and were between 18 and 55 years of age and in good physical health as determined by a medical history, electrocardiogram, and vital sign. The vital signs criterion cut-off was 135/85 mm Hg for blood pressure and 90 bpm for heart rate for the first 143 participants randomly assigned into the trial. The criterion was made more restrictive for participants aged 40 years or older, with cut-off values being 130/80 mm Hg and/or a heart rate > 88 bpm for the remainder of the trial. *Diagnostic and Statistical Manual of Mental Disorders*, Fourth Edition (*DSM-IV*) criteria for ADHD as assessed by the Adult Clinical Diagnostic Scale, version 1.2; to have a *DSM-IV* ADHD Rating Scale (ADHD-RS) total score > 22; to smoke at least 10 cigarettes per day; to have a carbon mon-oxide (CO) level ≥ 8 ppm; and to have smoked cigarettes for at least 3 months.	Candidates were excluded if they were a significant suicidal/homicidal risk; had used tobacco products other than cigarettes in the past week; had a positive urine screen for an illicit drug; or met *DSM-IV* criteria for current abuse or dependence for any psychoactive substance other than nicotine, current major depression, any current anxiety disorder except specific phobias, antisocial personality disorder, or a lifetime diagnosis of bipolar disorder or psychosis. Other exclusion criteria included a history of narrow angle glaucoma or seizure disorder, tics, or a family history of Tourette syndrome. Individuals were also excluded if they had been treated for ADHD with psychomotor stimulants or had used smoking cessation counseling programs or medications within the last 30 days, if they were currently taking a medication that could adversely interact with OROS-MPH, if they had a known allergy to OROS-MPH, or if they had been non-responders to a reasonable course of MPH treatment. Women were ineligible if they were pregnant or breastfeeding or unwilling to use an adequate method of birth control.

Study name	Inclusion criteria	Exclusion criteria
**Young2011 B4Z-US-LYCW NCT00190775**	Adults 18 years of age or older were required to meet DSM-IV-TR criteria for adult ADHD and have a historical diagnosis of ADHD during childhood, both of which were assessed by the Conners' Adult ADHD Diagnostic Interview for DSM-IV. Additionally, patients were required to have a Clinical Global Impressions - ADHD-Severity (CGI-ADHD-S) score of 4 (moderate symptoms) or greater and meet family unit criteria (reciprocal relationship with a person of the opposite sex and living in the same defined household with at least 1 child between ages 6 and 17 years old)	Patients were excluded if diagnostic criteria were met for any history of bipolar or psychotic disorder, current major depression, anxiety disorder, or DSM-IV-TR criteria for substance abuse. Patients who were currently taking or had previously taken atomoxetine or were taking any psychotropic medication on a regular basis were excluded.

Table S9. Participants medication status at baseline, for each study included in the network meta-analysis

Study name	Including some participants resistant to ADHD medications	Including some participants responders to ADHD medications	Excluding participants resistant to ADHD medications	Excluding participants responders to ADHD medications	Excluding participants naïve to ADHD medications	% of participants with prior ADHD treatment	Including only participants naïve to ADHD medications
**Abikoff2007**	-	-	-	-	-	0% (stimulants)	"No history of stimulant treatment"
**Adler2008a B4Z-MC-LYBV NCT00190931**	-	-	-	-	-	ATMX: 26.2%; PBO: 20.4% (stimulants)	-
**Adler2008b NRP104.303 NCT00334880**	-	-	-	-	-	NS	-
**Adler2009a B4Z-US-LYDQ NCT00190879**	-	-	-	-	-	ATMX: 22.32%; PBO: 24.77% (stimulants)	-
**Adler2009b B4Z-US-LYCU NCT00190736**	-	-	"Failure to respond to an adequate trial of treatment with ADHD stimulant medication, bupropion, or other nonstimulant medications (based upon the clinician's judgment) was also exclusionary"	-	-	ATMX: 22.58%; PBO: 27.89 (stimulants)	-
**Adler2009c CR011560NCT00326391**	-	-	"Known nonresponders to	-	-	"35.4% [of participants] had previously taken	-

Study name	Including some participants resistant to ADHD medications	Including some participants responders to ADHD medications	Excluding participants resistant to ADHD medications	Excluding participants responders to ADHD medications	Excluding participants naïve to ADHD medications	% of participants with prior ADHD treatment	Including only participants naïve to ADHD medications
			methylphenidate were also excluded”			medications for ADHD”	
**Adler2013 SPD489-403 NCT01101022**	-	-	“Exclusionary: History of failure to respond to an adequate course of amphetamine therapy. “	“Exclusionary: ADHD that was well controlled on current ADHD therapy”	-	NS	-
**Allen2005 B4Z-MC-LYAS**	-	-	-	-	-	ATMX: 72.4%; PBO: 63.9% (stimulants)	-
**Amiri2008**	-	-	-	-	-	NS	-
**Arnold2006**	-	-	-	-	-	NS	-
**Arnold2014 C1538/2027/AD/US NCT00315276**	-	-	-	“Being satisfied with his or her current ADHD medication”	-	Modafinil: 36%; PBO: 39% (ADHD medications within past 5 years)	-
**Bain2013 NCT00429091**	-	-	-	-	-	All: stimulants: 49%; atomoxetine: 12%	-
**Bangs2007 B4Z-MC-LYAX**	-	-	-	-	-	ATMX: 79.2%; PBO: 82.9% (stimulants)	-
**Bangs2008 B4Z-MC-LYBX NCT00191698**	-	-	-	-	-	ATMX: 67.7%; PBO: 74.3% (stimulants)	-

Study name	Including some participants resistant to ADHD medications	Including some participants responders to ADHD medications	Excluding participants resistant to ADHD medications	Excluding participants responders to ADHD medications	Excluding participants naïve to ADHD medications	% of participants with prior ADHD treatment	Including only participants naïve to ADHD medications
**Bedard2015 NCT00183391**	-	-	"Participants may have been previously treated with ATX or MPH, but must not have been nonresponders to an adequate trial and must not have experienced disabling adverse effects with either medication. Most participants were medication naıve (65%)".	-	-	35% "previously medicated"	-
**Biederman2002 SLI381-301**	-	"Children were either known to be responsive to stimulants or naıve to stimulant treatment."	known non-responders to stimulant medication were excluded	-	-	SLI381, 30 mg: 66.7%; SLI381, 20 mg: 65.2%; SLI381, 10 mg: 60.9%; PBO: 55.2% (stimulants) SLI381, 30 mg: 0%; SLI381, 20 mg: 3.6%; SLI381, 10 mg: 0.8%; PBO: 2.0%	-
**Biederman2005 Study311Cephalon**		-	"Those who had failed to respond to 2 or more adequate	"To avoid potential ethical concerns,	-	Modafinil: 34% PBO: 33% (methylphenidate) Modafinil: 27%	-

Study name	Including some participants resistant to ADHD medications	Including some participants responders to ADHD medications	Excluding participants resistant to ADHD medications	Excluding participants responders to ADHD medications	Excluding participants naïve to ADHD medications	% of participants with prior ADHD treatment	Including only participants naïve to ADHD medications
			courses (dose and duration) of stimulant therapy for ADHD were excluded”	patients whose ADHD was well controlled and who were satisfied with current ADHD therapy (with low levels of side effects) were also excluded”		PBO: 23% (dexamphetamine) Modafinil: 15% PBO: 13% (atomoxetine) Modafinil: 6% PBO: 2% (other)	
**Biederman2006a (subsampleofNCT00181571)**	-	-	-	Participants with a previous adequate trial of MPH were excluded	-	NS	-
**Biederman2006b**	Eligibility was restricted to those children who were stimulant-naive (i.e., who had not received stimulant medication in the past) or who had manifested an unsatisfactory response	-	-	-	-	31% “had taken stimulants for ADHD within 30 days of screening, with MPH being the most commonly used medication.”	-

Study name	Including some participants resistant to ADHD medications	Including some participants responders to ADHD medications	Excluding participants resistant to ADHD medications	Excluding participants responders to ADHD medications	Excluding participants naïve to ADHD medications	% of participants with prior ADHD treatment	Including only participants naïve to ADHD medications
	to stimulant therapy						
**Biederman2007 NRP104-301NCT00248092**	The intention of this study was to enroll children who were not adequately treated with their current medication for ADHD or had not previously been treated for ADHD.	-	-	-	-	LDX 30 mg: 9.9%<br>LDX 50 mg: 9.5%<br>LDX 70 mg: 2.7%<br>PBO: 8.3%<br>(amphetamines)<br>LDX 30 mg: 19.7%<br>LDX 50 mg: 17.6%<br>LDX 70 mg: 11.0%<br>PBO: 16.7%<br>(MPH)<br>LDX 30 mg: 4.2%<br>LDX 50 mg: 4.1%<br>LDX 70 mg: 6.8%<br>PBO: 2.8%<br>(stimulants)<br>LDX 30 mg: 2.8%<br>LDX 50 mg: 0%<br>LDX 70 mg: 2.7%<br>PBO: 1.4%<br>(atomoxetine)<br>LDX 30 mg: 1.4%<br>LDX 50 mg: 2.7%<br>LDX 70 mg: 4.1%<br>PBO: 5.6%<br>(stimulant/atomoxetine)<br>LDX 30 mg: 2.8%<br>LDX 50 mg: 1.4%<br>LDX 70 mg: 2.7%<br>PBO: 1.4%	-

Study name	Including some participants resistant to ADHD medications	Including some participants responders to ADHD medications	Excluding participants resistant to ADHD medications	Excluding participants responders to ADHD medications	Excluding participants naïve to ADHD medications	% of participants with prior ADHD treatment	Including only participants naïve to ADHD medications
						(other)	
**Biederman2008 SPD503-301 NCT00152009**	-	-	-	-	-	NS	-
**Biederman2012 2008P000971 NCT00801229**	-	-	-	-	-	"Out of seventy-five subjects enrolled in this study - and of 61 completers - 30 subjects had a history of prior ADHD medication treatment; 29 of whom had received trials of stimulant class medications. Of these 29 subjects, the majority (83%) had prior treatment histories of both methylphenidate and amphetamine formulations; 69% within the past 6 months. "	-
**Biehl2016**	-	-	-	-	-	"Nineteen of these patients (66%) were medication naïve, while 7 patients (24%) had had previous treatment attempts	-

Study name	Including some participants resistant to ADHD medications	Including some participants responders to ADHD medications	Excluding participants resistant to ADHD medications	Excluding participants responders to ADHD medications	Excluding participants naïve to ADHD medications	% of participants with prior ADHD treatment	Including only participants naïve to ADHD medications
						with MPH and 1 patient had had a previous treatment attempt with atomoxetine. "	
**Block2009 B4Z-US-LYCC NCT00486122**			Exclusion criterion: previous non response to study medication			ATMX/PBO: 71.2% PBO/ATMX: 67.8% PBO/PBO: 62.6% (stimulants)	
**Bron2014**	-	-	-	-	-	0%	Drug-naive patients
**Buitelaar1996**	-	-	-	-	-	0%	No previous treatment with psychotropic medications
**Casas2013 EudraCT#:2007-002111-82**	-	-	Key exclusion criteria included known non-response to MPH	-	-	NS	-
**Casat1989**	-	4/30 participants previously responders to MPH	-	-	-	All: 13.3% (MPH)	-
**Childress2009 CRIT124E2305 NCT00301236**	-	-	-	-	-	d-MPH XR: 27.1%; PBO: 35.4% (medications for ADHD)	-

Study name	Including some participants resistant to ADHD medications	Including some participants responders to ADHD medications	Excluding participants resistant to ADHD medications	Excluding participants responders to ADHD medications	Excluding participants naïve to ADHD medications	% of participants with prior ADHD treatment	Including only participants naïve to ADHD medications
**Coghill2013 SPD489-325**	-	-	Key exclusion criteria included: failure to respond to previous OROS-MPH therapy	Patients whose current ADHD medication provided effective control of symptoms with acceptable tolerability were also excluded	-	LDX: 57.7%<br>OROS-MPH: 54.1%<br>PBO: 52.7%	-
**Connor2000**	-	-	-	-	-	All: 45.8% (methylphenidate)	-
**Connor2010 SPD503-307NCT00367835**	-	-	-	-	-	NS	-
**Cook1993**	-	-	-	-	-	0%	Previous drugs for ADD: exclusionary criterion
**CRIT124DUS02**	-	-	-	-	-	NS	-
**Dell'Agnello2009**	-	-	-	-	-	ATX: 20.0%<br>PBO: 12.5%<br>(previous drug therapy)	-
**Dittmann2011**	-	-	-	-	-	ATX:<br>PBO:<br>(stimulants)	-

Study name	Including some participants resistant to ADHD medications	Including some participants responders to ADHD medications	Excluding participants resistant to ADHD medications	Excluding participants responders to ADHD medications	Excluding participants naïve to ADHD medications	% of participants with prior ADHD treatment	Including only participants naïve to ADHD medications
**Dopfner2003**	-	-	-	-	-	All: 71.6% (methylphenidate)	-
**Durell2013B4Z-US-LYDZNCT00510276**	-	-	-	-	-	ATX: 37.7% PBO: 35.1% (stimulants)	-
**Efron1997**	-	-	-	-	-	NS	-
**Findling2008 NCT00444574**	-	Participants were either naive to stimulant treatment or known to be responsive to stimulants.	-	-	-	OROS-MPH: 13% PBO: 12% (medications to treat ADHD)	-
**Findling2011 SPD489-305 NCT00735371**	-	-	Participants with failure to respond to and/or intolerance of amphetamine therapy… were disqualified	Participants who were well controlled on current ADHD medication with acceptable safety and efficacy were disqualified	-	NS	-
**Frick2017 SPD465-303 NCT00152022**	-	-	History of nonresponse to MPH or amphetamine was exclusionary	-	-	NS	-

Study name	Including some participants resistant to ADHD medications	Including some participants responders to ADHD medications	Excluding participants resistant to ADHD medications	Excluding participants responders to ADHD medications	Excluding participants naïve to ADHD medications	% of participants with prior ADHD treatment	Including only participants naïve to ADHD medications
**Gau2007 B4Z-TW-S010 NCT00485459**	-	-	"For ethical consideration, … subjects who were poor responders to or had intolerable adverse events of methylphenidate."	"For ethical consideration, we did not persuade patients to participate in this study, especially when they were under stable treatment with stimulants"	-	ATMX: 56.9%; PBO: 58.8% (psychostimulants)	-
**Geller2007 B4Z-US-LYBP**	-	-	-	-	-	ATMX: 60.92%; PBO: 64.04% (psychostimulants)	-
**Ginsberg2012 EUCTR2006-002553-80-SE**	-	-	Excluded those subjects known to be non-responsive or intolerant to methylphenidate	-	-	All: 16.6% (methylphenidate)	-
**Goodman2016 NCT00937040**	-	-	-	-	-	NS (history of stimulants or atomoxetine use within 5 years or other ADHD medications within 30 days was exclusionary)	-
**Goto2017 B4Z-JE-LYEE NCT00962104**	-	-	-	-	-	ATMX:22.3% PBO: 21.5%	-

Study name	Including some participants resistant to ADHD medications	Including some participants responders to ADHD medications	Excluding participants resistant to ADHD medications	Excluding participants responders to ADHD medications	Excluding participants naïve to ADHD medications	% of participants with prior ADHD treatment	Including only participants naïve to ADHD medications
**Greenhill2002**	-	-	Excluded were children who had failed a previous trial of stimulants for ADHD	-	-	MPH: 64%<br>PBO: 64%<br>(previous treatment for ADHD)	-
**Greenhill2006a Study309Cephalon**	-	-	Patients who had failed to respond to two or more adequate courses (dose and duration) of stimulant therapy for ADHD were also excluded	Patients with ADHD symptoms well controlled on current therapy with tolerable side effect were excluded	-	Modafinil: 34%<br>PBO: 43%<br>(methylphenidate)<br>Modafinil: 29%<br>PBO: 39%<br>(amphetamine salts)<br>Modafinil: 11%<br>PBO: 18%<br>(atomoxetine)	-
**Greenhill2006b CRIT124E2301**	-	-	Patients with a history of poor response or intolerance to methylphenidate were also excluded	-	-	d-MPH-ER: 37.7%<br>PBO: 40%<br>(medications for ADHD)	-
**Grizenko2012**	-	-	-	-	-	“38.9% of children in our sample had been on some medication in the past” (relative to Grizenko et al., 2012 publication)	-

Study name	Including some participants resistant to ADHD medications	Including some participants responders to ADHD medications	Excluding participants resistant to ADHD medications	Excluding participants responders to ADHD medications	Excluding participants naïve to ADHD medications	% of participants with prior ADHD treatment	Including only participants naïve to ADHD medications
**Harfterkamp2012 NCT00380692**	-	-	-	-	-	ATMX: 62.5% PBO: 63.3% (psychopharmacologic al treatment for ADHD)	-
**Herring2012 NCT00475735**	-	-	Exclusion criterion: poor or no response to a prior course of methylphenidate or other stimulant for ADHD	-	-	OROS-MPH: 4.5% PBO: 1.9% (stimulants)	-
**Hervas2014 SPD503-316 NCT01244490 EudraCT:2010-018579-12**	-	-	-	-	-	The use of at least one prior stimulant medication was reported by approximately 50% of all patients (GXR: 54 [47.4%]; ATX: 57 [50.9%]; placebo: 56 [50.5%]), and the use of non-stimulant, non-antipsychotic, psychotropic medication was reported by 20.8% of patients (GXR: 30 [26.3%]; ATX: 22 [19.6%]; placebo: 18 [16.2%]).	-

Study name	Including some participants resistant to ADHD medications	Including some participants responders to ADHD medications	Excluding participants resistant to ADHD medications	Excluding participants responders to ADHD medications	Excluding participants naïve to ADHD medications	% of participants with prior ADHD treatment	Including only participants naïve to ADHD medications
**Huss2014 CRIT124D2302 EUCTR2010-021533-31-DE NCT01259492**	-	-	Additionally, patients with either hypersensitivity or history of poor response or intolerance to stimulants as per the investigator's judgment were excluded from this study.	-	-	All: 13.3% (stimulants) (MPH: 9.1%, mixed amphetamine salts: 2.5%, lisdexamfetamine dismesylate:1.1%)	-
**Jafarinia2012**	-	-	-	-	-	0%	All drug-naïve
**Jain2011 NCT00556959**	-	-	-	-	-	NS	-
**Kahbazi2009**	-	-	-	-	-	NS	-
**Kay2009a,b**	-	-	Documented history of failure to respond clinically to amphetamines or atomoxetine was exclusionary	-	-	100%	All non-naïve
**Kelsey2004 B4Z-US-LYBG**	-	-	-	-	-	ATMX: 53.4 % PBO: 48.4% (stimulants)	-
**Kollins2011 SPD503-206 NCT00150592**	-	-	-	-	-	GXR: PBO: (stimulants use in the past 12 months)	-

Study name	Including some participants resistant to ADHD medications	Including some participants responders to ADHD medications	Excluding participants resistant to ADHD medications	Excluding participants responders to ADHD medications	Excluding participants naïve to ADHD medications	% of participants with prior ADHD treatment	Including only participants naïve to ADHD medications
**Kooij2004**	-	-	-	-	-	NS	-
**Kurlan2002**	-	-	-	-	-	MPH: 62%<br>Clonidine: 62%<br>PBO: 63%<br>(stimulants)<br>MPH: 27%<br>Clonidine: 32%<br>PBO: 56%<br>(clonidine)	-
**Lin2014<br>NCT00922636**	-	-	-	-	-	OROS-MPH: 0%<br>PBO: 43.6%<br>(psychostimulants)	-
**Lin2016<br>NCT00917371**	-	-	-	-	-	0%	Medication-naïve adults with ADHD
**Martenyi2010<br>B4Z-MW-LYCZ<br>NCT00386581**	-	-	-	-	-	0%	All medication-naïve
**McCracken2016**	-	-	-	-	-	NS	-
**McRae-Clark2010<br>R21DA018221<br>NCT00360269**	-	-	-	-	-	NS	-
**Medori2008<br>LAMDA-IEUCTR2004-000730-37<br>NCT00246220**	-	-	Exclusion criteria: History of poor response or intolerance to methylphenidate	-	-	NS	-
**Michelson2001<br>B4Z-MC-LYAC**	-	-	-	-	-	70% previous treatment with stimulants	-

Study name	Including some participants resistant to ADHD medications	Including some participants responders to ADHD medications	Excluding participants resistant to ADHD medications	Excluding participants responders to ADHD medications	Excluding participants naïve to ADHD medications	% of participants with prior ADHD treatment	Including only participants naïve to ADHD medications
**Michelson2002 B4Z-MC-LYAT**	-	-	-	-	-	ATMX: 56.5% PBO: 54.1% (stimulants)	-
**Michelson2003a,b**	-	-	-	-	-	Study 1: ATMX: 44% PBO: 48.9% (stimulants) Study 2: ATMX: 50.4% PBO: 43.3% (stimulants)	-
**Moharari2012 IRCT201012295500N1**	-	-	-	-	-	0% (methylphenidate or bupropion)	No history of methylphenidat e or bupropion
**Montoya2009 B4Z-XM-LYDM NCT00191945**	-	-	-	-	-	0%	Treatment naïve
**NCT01069523**	-	-	-	-	-	NS	-
**Newcorn2008 B4Z-MC-LYBI**	-	-	Subjects could either have been treated previously with stimulants or be treatment naive. However, for ethical reasons subjects were excluded if they had been treated previously with an adequate	-	-	All: 74.8% (stimulants)	-

Study name	Including some participants resistant to ADHD medications	Including some participants responders to ADHD medications	Excluding participants resistant to ADHD medications	Excluding participants responders to ADHD medications	Excluding participants naïve to ADHD medications	% of participants with prior ADHD treatment	Including only participants naïve to ADHD medications
			trial of methylphenidate or amphetamine and either did not experience at least some improvement in ADHD signs and symptoms (nonresponders) or had intolerable adverse events				
**Newcorn2013 SPD503-314 NCT00997984**	-	-	-	-	-	NS	-
**Palumbo2008 NCT00031395**	-	-	-	-	-	MPH: 41.4 % Clonidine: 58.1% PBO: 40% (stimulants) MPH: 7.1% Clonidine: 6.5% PBO: 3.3% (clonidine)	-
**Paterson1999**	-	-	-	-	-	NS	-
**Philipsen2015 EUCTR2006-000222-31-DE ISRCTN54096201**	-	-	-	-	-	MPH+clinical management: 15.9% PBO+clinical management: 23.34% (methylphenidate, amphetamine, other psychostimulants)	-

Study name	Including some participants resistant to ADHD medications	Including some participants responders to ADHD medications	Excluding participants resistant to ADHD medications	Excluding participants responders to ADHD medications	Excluding participants naïve to ADHD medications	% of participants with prior ADHD treatment	Including only participants naïve to ADHD medications
**Pliszka2000**	-	-	-	-	-	Adderall: 20%<br>MPH: 25%<br>PBO: 6%<br>(stimuants)	-
**Reimherr2005**	-	-	-	-	-	NS	-
**Reimherr2007**	-	-	-	-	-	NS	-
**Rosler2009**	-	-	-	-	-	NS	-
**Rugino2003**	-	-	-	-	-	NS	-
**Rugino2014 NCT01156051**	-	-	-	-	-	GXR: 63.6%<br>PBO: 31.2%<br>(stimulants)	-
**Sallee2009 SPD503-304NCT00150618**	-	-	-	-	-	NS	-
**Sangal2006 B4Z-US-LYAV**	-	-	-	-	-	NS	-
**Scahill2011 NCT00004376**	-	-	-	-	-	NS	-
**Schrantee2016 NTR3103 EUCTR2010-023654-37-NL**	-	-	-	-	-	0% (stimulants)	Stimulants naïve
**Schulz2012**	-	-	Poor response or tolerability to an adequate trial of either methylphenidate or atomoxetine	-	-	MPH: 44%<br>ATMX: 28%<br>(stimulants)	-
**Simonoff2013 ISRCTN68384912**	-	-	-	-	-	NS	-

Study name	Including some participants resistant to ADHD medications	Including some participants responders to ADHD medications	Excluding participants resistant to ADHD medications	Excluding participants responders to ADHD medications	Excluding participants naïve to ADHD medications	% of participants with prior ADHD treatment	Including only participants naïve to ADHD medications
**Singer1995**	-	-	-	-	-	NS	-
**SPD489-405 NCT01552915**	-	-	Subject has failed to fully respond to an adequate course(s) (dose and duration) of MPH or amphetamine therapy.	-	-	NS	-
**SPD489-406 NCT01552902**	-	-	Subject has failed to fully respond to an adequate course(s) (dose and duration) of MPH or amphetamine therapy.	-	-	NS	-
**Spencer1995**	-	-	-	-	-	NS	-
**Spencer1998**	-	-	-	-	-	NS	-
**Spencer2001**	-	-	-	"Previous adequate trial of Aderall" (not clear what adequate means; author contacted but no reply)	-	All: 29.6% (stimulants)	-

Study name	Including some participants resistant to ADHD medications	Including some participants responders to ADHD medications	Excluding participants resistant to ADHD medications	Excluding participants responders to ADHD medications	Excluding participants naïve to ADHD medications	% of participants with prior ADHD treatment	Including only participants naïve to ADHD medications
**Spencer2002a,b B4Z-MC-HFBD B4Z-MC-HFBK**	-	-	-	-	-	0%	Stimulant-naïve
**Spencer2005**				Previous adequate trial of stimulant (not clear what adequate means; author contacted but no reply)		MPH: 10%<br>PBO: 7%<br>(anti ADHD medication)	
**Spencer2006 SLI381-314 NCT00507065**	-	-	A history of non-response to stimulant medication was exclusionary (although text also states: Adolescents who were known to be nonresponsive to stimulants (defined as no clinical improvement after trials of 2 stimulant medications, taken for at least 3 weeks each) or naive to stimulant	-	-	Prior treatment within previous 30 days: all: 21.2%<br>MAS XR 10 mg/day: 11.1%<br>MAS XR 20 mg/day: 24.5%<br>MAS XR 30 mg/day: 27.6%<br>MAS XR 40 mg/day: 27.9%<br>PBO: 13.5%<br>(any ADHD treatment)	-

Study name	Including some participants resistant to ADHD medications	Including some participants responders to ADHD medications	Excluding participants resistant to ADHD medications	Excluding participants responders to ADHD medications	Excluding participants naïve to ADHD medications	% of participants with prior ADHD treatment	Including only participants naïve to ADHD medications
			treatment were eligible for enrollment. (Author contacted but no reply)				
**Spencer2007 CRIT124E2302**	-	-	Patients were also excluded if the investigator judged that they had a history of poor response or intolerance to stimulants (e.g., MPH, d-MPH, amphetamine salts, or dextroamphetamine salts). No patient had previously used d-MPH-ER.	-	-	D-MPH-ER 20 mg/day:31.6% D-MPH-ER 30 mg/day: 16.7% D-MPH-ER 40 mg/day:27.8% PBO: 35.8% (MPH/d-MPH:) D-MPH-ER 20 mg/day: 14.0% D-MPH-ER 30 mg/day: 7.4% D-MPH-ER 40 mg/day: 18.5% PBO: 13.2% (MPH stimulants) D-MPH-ER 20 mg/day: 5.3% D-MPH-ER 30 mg/day: 14.8%9.3% D-MPH-ER 40 mg/day: PBO: 17.0% (nonstimulants)	-
**Spencer2008 SPD465-301 NCT00150579**	-	-	Nonresponse to methylphenidate or	-	-	Triple-bead MAS: 24.1% PBO: 24.4%	-

Study name	Including some participants resistant to ADHD medications	Including some participants responders to ADHD medications	Excluding participants resistant to ADHD medications	Excluding participants responders to ADHD medications	Excluding participants naïve to ADHD medications	% of participants with prior ADHD treatment	Including only participants naïve to ADHD medications
			amphetamines was exclusionary			(any ADHD medication)<br>Triple-bead MAS: 5.1%<br>PBO: 1.5%<br>(MAS immediate)<br>Triple-bead MAS:12.4%<br>PBO: 17.8%<br>(MAS extended release)<br>Triple-bead MAS: 0%<br>PBO: 2.2%<br>(atomoxetine)<br>Triple-bead MAS: 1.5%<br>PBO: 2.2%<br>(bupropion)<br>Triple-bead MAS: 0.7%<br>PBO: 0.7%<br>MAS (dextroamphetamine)<br>Triple-bead MAS: 5.8%<br>PBO: 2.2.%<br>MAS (methylphenidate)	
**Stein2011 NCT00393042**	-	-	-	-	-	Previously treated with MPH :43%<br>Previously treated with AMPH□: 5%	-

Study name	Including some participants resistant to ADHD medications	Including some participants responders to ADHD medications	Excluding participants resistant to ADHD medications	Excluding participants responders to ADHD medications	Excluding participants naïve to ADHD medications	% of participants with prior ADHD treatment	Including only participants naïve to ADHD medications
						Previously treated with MPH and AMPH: 22%	
**Sutherland2012 NCT00174226**	-	-	-	-	-	NS	-
**Svanborg2009 B4Z-SO-LY15 EUCTR2004-003941-42-SE NCT00191542**	-	-	-	-	-	0%	Stimulant naïve
**Swanson2006**	-	-	Those who fail to respond to 2 or more adequate courses of stimulant therapy, with trials on a range of doses and immediate and controlled-release formulations were excluded	Those satisfied with current ADHD treatment were excluded	-	Modafinil: 34% PBO: 41% (MPH) Modafinil: 32% PBO: 28% (AMPH salts) Modafinil: 18% PBO: 20% (ATMX) Modafinil: 5% PBO: 9% (other) Modafinil: 53% PBO: 59% (total)	
**Takahashi2009 B4Z-JE-LYBC NCT00191295**	-	-	-	-	-	ATMX 0.5 mg/Kg/day: 54.8% ATMX 1.2 mg/Kg/day: 55% ATMX 1.8 mg/Kg/day:54.1% PBO: 51.6% (stimulants)	-

Study name	Including some participants resistant to ADHD medications	Including some participants responders to ADHD medications	Excluding participants resistant to ADHD medications	Excluding participants responders to ADHD medications	Excluding participants naïve to ADHD medications	% of participants with prior ADHD treatment	Including only participants naïve to ADHD medications
**Takahashi2014 NCT01323192**	-	-	Patients were excluded from the study if they were a non-responder to MPH	-	-	NS	-
**Taylor1987**	-	-	-	-	-	0% (stimulants)	Stimulants naive
**Taylor2000**	-	-	-	-	-	NS	-
**Taylor2001**	-	-	-	-	-	NS	-
**VanDerMeere1999**	-	-	-	-	-	0%	Stimulant naïve (according to Storebo et al., 2015)
**Wang2007 NCT00486083 B4Z-MC-LYBR(6934)**	-	-	-	-	-	ATMX: 23.2% MPH: 25.3% (stimulants)	-
**Wehmeier2012 B4Z-SB-LYDV NCT00546910**	-	-	-	-	-	ATMX: 20.6% PBO: 29% (stimulants)	-
**Weisler2006 SLI381-303**	-	-	-	-	-	All: about 25% (stimulants)	-
**Weisler2012 NCT00880217**	-	-	History of non-response to treatment with a psychostimulant medication or to treatment with atomoxetine or methylphenidate was exclusionary	-	-	OROS-MPH: 7% ATMX: 10 % PBO: 14% (prior psychotropic treatment in the previous 3 months)	-

Study name	Including some participants resistant to ADHD medications	Including some participants responders to ADHD medications	Excluding participants resistant to ADHD medications	Excluding participants responders to ADHD medications	Excluding participants naïve to ADHD medications	% of participants with prior ADHD treatment	Including only participants naïve to ADHD medications
**Weiss2005 4Z-MC-LYAW**	-	-	-	-	-	ATMX: 61.4%<br>PBO: 55.8%<br>(stimulants)	-
**Wender2011**	-	-	-	-	-	NS	-
**Wietecha2013 NCT00607919**	-	-	-	-	-	NS	-
**Wigal2004**	-	-	-	-	-	d-MPH: 25%<br>d,l-MPH: 30.4%<br>PBO: 28.6%<br>(stimulants within 30 days before screening)	-
**Wigal2005 SLI381-404 NCT00506727**			failure to respond clinically to adequate doses of stimulants was exclusionary			NS	
**Wigal2015 NCT01239030**	-	-	-	-	-	NS	-
**Wilens2001**	-	-	-	-	-	NS	-
**Wilens2005 NCT00048360**	-	-	Inadequate response to bupropion (for the treatment of ADHD) or inadequate responses to two or more adequate trials	-	-	Bupropion XL: 47%<br>PBO: 39%<br>(previous treatment for ADHD)	-

Study name	Including some participants resistant to ADHD medications	Including some participants responders to ADHD medications	Excluding participants resistant to ADHD medications	Excluding participants responders to ADHD medications	Excluding participants naïve to ADHD medications	% of participants with prior ADHD treatment	Including only participants naïve to ADHD medications
			of psychostimulants was exclusionary				
**Wilens2008 B4Z-MC-LYBY NCT00190957**	-	-	-	-	-	NS	-
**Wilens2011 NCT00528697**	-	-	Failure to respond to two or more adequate trials of U.S. Food and Drug Administration–approved ADHD medication was also exclusionary.	-	-	ATMX:45% PBO: 43% (previous treatment for diagnosed ADHD)	-
**Wilens2015 SPD503-312 EUCTR2011-002221-21 NCT01081132**	-	-	-	-	-	At least 1 prior stimulant medication was used by 77.4% of participants in the placebo group and 70.1% in the GXR group. The most frequently used prior stimulant medications were MPH hydrochloride (48.4%), mixed amphetamine salts (34.6%),	-

Study name	Including some participants resistant to ADHD medications	Including some participants responders to ADHD medications	Excluding participants resistant to ADHD medications	Excluding participants responders to ADHD medications	Excluding participants naïve to ADHD medications	% of participants with prior ADHD treatment	Including only participants naïve to ADHD medications
						lisdexamfetamine mesylate (27.9%), dexmethylphenidate hydrochloride (14.4%), and methylphenidate (10.3%).	
**Winhusen2010 NCT00253747**	-	-	Participants were excluded if they had been non-responders to a reasonable course of MPH treatment	-	-	NS	-
**Young2011 B4Z-US-LYCW NCT00190775**	-	-	-	-	-	ATMX: 17.6% PBO: 15% (stimulants)	-

Table S10. Characteristics of the trials included in the network meta-analysis. ***Only study arms relevant for the present network meta-analysis are reported. Additional study details, such as effect estimates, are reported in the dataset of the meta-analysis, freely available upon publication.***

Study name Country	Design	Duration (weeks)	Criteria	Drug	Dose (min-max)	N	Age (M, SD)	Males (%)	Comorbid. (%)	Spons.	Source of information/data
Abikoff2009 USA	Cross-over w/ washout	4	DSM-IV	MPH-ER	18-54 mg/d	19	10.1 (1.6)	78.9	ODD: 26. AD: 10.6 DD: 5.3 CD: 5.3	Yes	Journal article
				PBO	-						
Adler2008a B4Z-MC-LYBV NCT00190931 USA	Parallel	24	DSM-IV-TR	ATMX	40-100 mg/d	271	37.1 (8.3)	56.1	None	Yes	Journal articles ClinicalTrials.gov FDA Short CSR Full CSR
				PBO	-	139	36.0 (8.4)	63.3			
Adler2008b NRP104.303 NCT00334880 USA	Parallel	4	DSM-IV-TR	LDX	30 mg/d	119	35.3 (10.1)	56	None	Yes	Journal articles ClinicalTrials.gov FDA Short CSR Additional information/data from manufacturer
				LDX	50 mg/d	117	34.2 (10)	56			
				LDX	70 mg/d	122	35.8 (10.5)	52			
				PBO	-	62	35.2 (10.9)	52			
Adler2009a B4Z-US-LYDQ NCT00190879 USA	Parallel	14	DSM-IV-TR	ATMX	40-100 mg/d	224	37.9 (11.8)	54.5	SAD: 100	Yes	Journal article ClinicalTrials.gov FDA Short CSR Additional information/data from manufacturer
				PBO	-	218	38.1 (11.4)	52.8	SAD: 100		
Adler2009b B4Z-US-LYCU NCT00190736 USA	Parallel	24	DSM-IV-TR	ATMX	25-100 mg/d	250	37.7 (10.4)	49.6	None	Yes	Journal article ClinicalTrials.gov FDA Short CSR Full CSR
				PBO	-	251	37.4 (9.8)	51.8			

Study name Country	Design	Duration (weeks)	Criteria	Drug	Dose (min-max)	N	Age (M, SD)	Males (%)	Comorbid. (%)	Spons.	Source of information/data
Adler2009c CR011560 NCT00326391 USA	Parallel	7	DSM-IV	MPH-ER	36-108 mg/d	113	39.9 (12.3)	57.3	None	Yes	Journal article ClinicalTrials.gov FDA Additional information/data from manufacturer
				PBO	-	116	38.2 (11.4)	55.2			
Adler2013 SPD489-403 NCT01101022 USA	Parallel	10	DSM-IV-TR	LDX	30-70 mg/d	80	34.2 (10.6)	50.6	None	Yes	Journal article ClinicalTrials.gov Short CSR Additional information/data from manufacturer
				PBO	-	81	34.9 (11.02)	53.8			
Allen2005 B4Z-MC-LYAS USA	Parallel	18	DSM-IV	ATMX	0.5-1.5 mg/d	76	10.9 (2.5)	92.1	TD: 100 ODD: 22.4 GAD: 2.6 OCD: 2.6	Yes	Journal article Short CSR Full CSR
				PBO	-	72	11.5 (2.4)	84.7	TD: 100 ODD: 20.8 GAD: 4.2 OCD: 2.8 DD: 1.4		
Amiri2008 Iran	Parallel	6	DSM-IV-TR	MODA	200-300 mg/d	30	9.2 (2.5)	76.6	None	No	Journal article
				MPH IR	20-30 mg/d	30	9.0 (2.3)	90			
Arnold2006 USA	Cross-over w/ washout	6	DSM-IV	ATMX	1.2-1.4 mg/kg/day	16	9.3 (2.9)	75	ASD: 100	Yes	Journal article Additional information/data provided by the authors
				PBO	-						
Arnold2014 C1538/2027/AD/US NCT00315276 USA	Parallel	9	DSM-IV-TR	MODA	255-255 mg/d	73	41.1 (10.5)	70	None	Yes	Journal article ClinicalTrials.gov
				MODA	340-340 mg/d	73	37.6 (11.5)	58			
				MODA	425-425 mg/d	74	39.6 (12.1)	55			

Study name Country	Design	Duration (weeks)	Criteria	Drug	Dose (min-max)	N	Age (M, SD)	Males (%)	Comorbid. (%)	Spons.	Source of information/data
				MODA	510-510 mg/d	44	39.6 (12.6)	66			
				PBO	-	74	38.6 (11.2)	53			
Bain2013 NCT00429091 USA	Cross-over w/ wash-out	4	DSM-IV-TR	ATMX	40 mg/day	53	NS	NS	MD: 21% AD: 3% CD: < 1% (lifetime)	Yes	Journal articles ClinicalTrials.gov
				PBO	-						
Bangs2007 B4Z-MC-LYAX USA	Parallel	9	DSM-IV	ATMX	1.2-1.8 mg/d	72	14.6 (1.8)	72.2	MDD: 100	Yes	Journal articles FDA Short CSR Full CSR
				PBO	-	70	14.2 (1.5)	74.3	MDD: 100		
Bangs2008 B4Z-MC-LYBX NCT00191698 Europe and Australia	Parallel	8	DSM-IV	ATMX	1.2mg/d	156	9.5 (1.9)	91.7	ODD: 100	Yes	Journal articles ClinicalTrials.gov FDA Short CSR Full CSR
				PBO	-	70	9.7 (1.9)	97.1	ODD: 100		
Bedard2015 NCT00183391 USA	Cross-over w washout	6	DSM-IV	MPH-ER	18-72 mg/d	143	NS	NS	-	No	Journal articles ClinicalTrials.gov FDA Short CSR
				ATMX	0.5-1.8 mg/d						
Biederman SLI 381-301 2002 USA	Parallel	3	DSM-IV	MAS-ER	10mg/d	129	8.5 (1.6)	78.1	Total: 32	Yes	Journal articles Additional information/data from manufacturer
				MAS-ER	20mg/d	121	8.4 (1.7)	80.4	Total: 27.7		
				MAS-ER	30mg/d	124	8.8 (1.8)	80.0	Total: 30.8		
				PBO	-	210	8.6 (1.7)	72.9	Total: 30.0		
Biederman Study 311 Cephalon 2005 USA	Parallel	9	DSM-IV	MODA	170-425 mg/day	164	10.4 (6-17)	69	None of clinical relevance	Yes	Journal articles FDA Investigator brochure
				PBO	-	84	10.1 (6-17)	74			
Biederman2006	Parallel	3	DSM-IV-	MPH-	36 mg/d-	72	32.7	57	SUD: 61	Yes	Journal articles

Study name Country	Design	Duration (weeks)	Criteria	Drug	Dose (min-max)	N	Age (M, SD)	Males (%)	Comorbid. (%)	Spons.	Source of information/data
a (subsample of NCT00181571) USA			TR	ER	1.3 mg/Kg/d		(18.5)		AD: 22 CD: 9 ASPD:8 MD:6 BD:4 (lifetime)		
				PBO	-	77	37.6 (8.4)	47	SUD: 71 AD: 20 CD: 20 ASPD: 11 MD: 4 BD: 4 (lifetime)		
Biederman2006 b USA	Parallel	4	DSM-IV	MODA	300 mg/d	50	8.8 (2)	66	None (current)	Yes	Journal articles
				MODA	300 mg/d	49	8.8 (2.1)	80			
				MODA	300 mg/d	48	9.2 (2.1)	79			
				MODA	400 mg/d	50	10.5 (1.6)	74			
				PBO	-	51	8.9 (2)	75			
Biederman2007 NRP104-301 NCT00248092 USA	Parallel	4	DSM-IV-TR	LDX	30mg/d	71	9 (1.9)	74	None	Yes	Journal articles ClinicalTrials.gov FDA Additional information/data from manufacturer
				LDX	50mg/d	74	8.9 (1.8)	62			
				LDX	70mg/d	73	8.7 (1.8)	71			
				PBO	-	72	9.4 (1.7)	69			
Biederman2008 SPD503-301 NCT00152009 USA	Parallel	8	DSM-IV-TR	GXR	2mg/day	87	10.6 (2.4)	77	None	Yes	Journal articles ClinicalTrials.gov FDA Product monograph
				GXR	3mg/day	86	10.8 (2.8)	80.2			

Study name Country	Design	Duration (weeks)	Criteria	Drug	Dose (min-max)	N	Age (M, SD)	Males (%)	Comorbid. (%)	Spons.	Source of information/data
				GXR	4mg/day	86	10.1 (2.9)	66.3			Additional information/data from manufacturer
				PBO	-	86	10.6 (2.7)	74.4			
Biederman2012 2008P000971 NCT00801229 2012 USA	Parallel	6	DSM-IV	LDX	30-70 mg/d	35	18-26 (range)	62	None	Yes	Journal articles ClinicalTrials.gov
				PBO	-	34	18-26 (range)				
Biehl2016 Germany	Parallel	6	DSM-IV-TR	MPH	10-60 mg/d	19	36.7 (10)	67	None	No	Journal articles ClinicalTrials.gov Additional information from the authors
				PBO	-	16	35.5 (10.1)	57	None		
Block2009 B4Z-US-LYCC NCT00486122 USA	Parallel	6	DSM-IV-TR	ATMX am	0.47-1.81 mg/kg/d	102	8.8 (1.7)	67.7	ODD: 34.3	Yes	Journal articles ClinicalTrials.gov FDA Short CSR Full CSR
				ATMX pm	0.51-1.81 mg/kg/d	93	9.1 (1.6)	76.3	ODD: 25.8		
				PBO	-	93	8.9 (1.7)	74.2	ODD: 34.4		
Bron2014 Netherlands	Cross-over w/ wash out	2	DSM-IV	MPH-ER	36-72 mg/d	14	30.5 (7.4)	77.3	MDD: 50% SUD: 40.7% AD: 13.6% ED: 4.5%	No	Journal articles Additional information/data from study authors
				PBO	-	13					
Buitelaar1996 Netherlands	Cross-over w/ washout	4	DSM-III-R	MPH	10 mg/bid	10	7-13 (range)	93.7	None	No	Journal article
				PBO	-	11					
Casas2013 EudraCT: 2007-002111-82 Spain	Parallel	13	DSM-IV-TR	MPH-ER	54 mg/d	90	35.8 (11.7)	48.9	None (clinically unstable psychiatric condition)	Yes	Journal articles ClinicalTrials.gov Short CSR Additional information/data from study authors
				MPH-ER	72 mg/d	92	35.8 (10.1)	54.3			
				PBO	-	97	35.5 (8.8)	53.6			
Casat1989 USA	Parallel	4	DSM-III	BUP	6mg/kg/day	20	6-12 (range)	-	-	Yes	Journal article

Study name<br>Country	Design	Duration (weeks)	Criteria	Drug	Dose (min-max)	N	Age (M, SD)	Males (%)	Comorbid. (%)	Spons.	Source of information/data
				PBO	-	10	6-12 (range)	-			
Childress2009<br>CRIT124E2305<br>NCT00301236<br>USA	Parallel	5	DSM-IV-TR	d-threo MPH-ER	10 mg/d	66	8.3 (1.7)	63.6	None	Yes	Journal articles<br>ClinicalTrials.gov<br>Short CSR
				d-threo MPH-ER	20 mg/d	62	8.7 (1.9)	61.3			
				d-threo MPH-ER	30 mg/d	60	9.0 (1.9)	66.7			
				PBO	-	65	8.9 (1.8)	66.2			
Coghill2013<br>SPD489-325<br>EU and USA	Parallel	7	DSM-IV-TR	LDX	30-70 mg/d	113	10.9 (2.9)	78.4	Any: 17.1<br>ODD: 7.2	Yes	Journal articles<br>ClinicalTrials.gov<br>Additional information from manufacturer
				MPH-ER	18-54 mg/d	112	10.9 (2.6)	81.1	Any: 26.1<br>ODD: 9		
				PBO	-	111	11 (2.8)	82.7	Any: 18.2<br>ODD: 7.3		
Connor2000<br>USA	Parallel	12	DSM-III-R	CLON	0.3 mg/d	8	9.3 (1.7)	100	All: ODD or CD	No	Journal article
				MPH	40 mg/d	8	8.9 (2.6)	100			
Connor2010<br>SPD503-307<br>NCT00367835<br>USA	Parallel	9	DSM-IV	GXR	1-4 mg/d	138	9.4 (1.73)	64	-	Yes	Journal articles<br>ClinicalTrials.gov<br>Additional information from manufacturer
				PBO	-	79	9.3 (2.04)	76.9			
Cook1993<br>USA	Cross-over w/ washout	3	DSM-III	MPH	0.20-0.38 mg/Kg/d	15	104 months (11.2)	100	-	No	Journal article
				PBO	-						
CRIT124<br>DUS02<br>USA	Cross-over w/ washout	4	NS	MPH MR	20-60 mg/d	109	13.8 (1.6)	0	NS	Yes	Short CSR<br>Additional information from manufacturer
				PBO	-						
Dell'Agnello 2009<br>Italy	Parallel	8	DSM-IV	ATMX	0.85-1.33 mg/Kg/d	107	9.7 (2.2)	93.3	GAD: 9.5<br>Dys:8.6<br>PHO: 7.6	Yes	Journal articles<br>ClinicalTrials.gov

Study name Country	Design	Duration (weeks)	Criteria	Drug	Dose (min-max)	N	Age (M, SD)	Males (%)	Comorbid. (%)	Spons.	Source of information/data
									SAD: 4.8<br>OCD: 1.9<br>PD: 1.9<br>MDD: 1.9<br>Adj D: 1.0<br>SPD:1.0<br>Other DD: 0.0		
				PBO	-	32	10 (2.4)	90.6	GAD: 15.6<br>Dys: 0.0<br>PHO: 6.3<br>SAD: 0.0<br>OCD: 3.1<br>PD: 3.1<br>MDD: 0.0<br>Adj D: 1.0<br>SPD: 3.1<br>Other DD: 0.0		
Dittman2011 Germany	Parallel	9	DSM-IV-TR	ATMX fast	0.5-1.2 mg/kg	61	10.8 (3.4)	86.9	ODD: 73.3<br>CD: 23.3<br>Disr Beh: 1.7<br>Adj Dis: 1.7	Yes	Journal articles<br>ClinicalTrials.gov<br>Full CSR
				ATMX slow	0.5-1.2 mg/kg	60	11.1 (2.9)	85	ODD: 73.8%<br>CD: 26.2<br>Disr Beh: 0.0<br>Adj Dis: 0.0		
				PBO	-	59	11.1 (2.8)	81.4	ODD: 76.3%<br>CD: 23.7<br>Disr Beh: 0.0<br>Adj Dis: 0.0		
Dopfner2003 Germany	Parallel	4	DSM-IV	MPH-MR	20-60 mg/d	43	9.8 (2.4)	86	NS	Yes	Journal articles<br>Additional information/data from author
				PBO	-	42	9.8 (2.1)	90			
Durell2013 B4Z-US-LYDZ	Parallel	12	DSM-IV-TR	ATMX	40-100 mg/d	220	24.7 (3.4)	58.2	-	Yes	Journal articles<br>ClinicalTrials.gov

Study name Country	Design	Duration (weeks)	Criteria	Drug	Dose (min-max)	N	Age (M, SD)	Males (%)	Comorbid. (%)	Spons.	Source of information/data
NCT00510276 USA				PBO	-	225	24.7 (3.5)	56.4			Full CSR
Efron1997 Australia	Cross-over w/ wash out	2	DSM-IV	MPH	0.3 mg/Kg/ dose	125	8.73 (2.3)	91.2	NS	No	Journal articles Additional information/data from study author
				Dextro - AMPH	0.15 mg/Kg/ dose						
Findling2008 NCT00444574 USA	Parallel	7	DSM-IV	MPH ER	18-54 mg/d	94	8.8 (1.94)	66	None (except for ODD)	Yes	Journal articles ClinicalTrials.gov
				PBO	-	88	11.1 (2.8)	81.4	None (except for ODD)		
Findling2011 SPD 489-305 NCT00735371 USA	Parallel	2	DSM-IV-TR	LDX	30 mg/d	78	14.6 (1.4)	75.6	None (except for ODD or disorders not requiring treatment)	Yes	Journal articles ClinicalTrials.gov FDA Additional information from manufacturer
				LDX	50 mg/d	79	14.7 (1.3)	79.7			
				LDX	70 mg/d	78	14.4 (1.3)	56.4			
				PBO	-	79	14.5 (1.25)	68.4			
Frick2017 SPD465-303 NCT00152022 USA	Parallel	6	DSM-IV-TR	Triple bead MAS	25 mg/d	104	38 (9.9)	51.9	-	Yes	Journal article ClinicalTrials.gov
				Triple bead MAS	50 mg/d	101	37.2 (9.5)	65.3			
				Triple bead MAS	75 mg/d	102	37.9 (9.7)	53.9			
				PBO	-	104	35.6 (9.8)	55.8			
Gau2007 B4Z-TW-S010 NCT00485459 Taiwan	Parallel	2	DSM-IV-TR	ATMX	0.8-1.2 mg/ Kg/d	72	9.1(2)	90.3	ODD: 19.4 CD: 9.7	Yes	Journal article ClinicalTrials.gov FDA Short CSR

Study name Country	Design	Duration (weeks)	Criteria	Drug	Dose (min-max)	N	Age (M, SD)	Males (%)	Comorbid. (%)	Spons.	Source of information/data
				PBO	-	34	9.5 (2.4)	85.3	ODD: 8.8 CD: 5.9		Full protocol provided by manufacturer
Geller2007 B4Z-US-LYBP USA	Parallel	12	DSM-IV	ATMX	0.8-1.2	87	12.2 (2.8)	62.1	NS	Yes	Journal article FDA Short CSR Full CSR
				PBO	-	89	11.8 (2.5)	67.4	NS		
Ginsberg2012 EUCTR2006-002553-80-SE Sweden	Parallel	5	DSM-IV	MPH-ER	72 mg/d	15	33.5 (NS)	100	SUD: 100	No	Journal article ClinicalTrials.gov Additional information from study author
				PBO	-	15	35.3 (NS)	100	SUD: 100		
Goodman2016 NCT00937040 USA	Parallel	6	DSM-IV	MPH-ER	16-72 mg/d	178	36.8 (11.9)	50.6%	DD: 14.9% AD: 39.1%	Yes	Journal article ClinicalTrials.gov Additional information from manufacturer
				PBO	-	179	34.7 (11.6)	54.9%	DD: 14.3% AD: 34.9%		
Goto2017 B4Z-JE-LYEE NCT00962104 Japan	Parallel	10	DSM-IV	ATMX	80-120 mg/d	195	32.8 (8.1)	47	0	Yes	Journal article ClinicalTrials.gov Full CSR
				PBO	-	196	31.7 (7.8)	49	0		
Greenhill2002 USA	Parallel	3	DSM-IV	MPH MR	20-60 mg/d	158	9 (2)	83	NS	Yes	Journal article
				PBO	-	163	9 (1.8)	81			
Greenhill2006a Study 309 Cephalon USA	Parallel	9	DSM-IV	MODA	170-424 mg/d	133	9.9 (NS)	73	NS	Yes	Journal article FDA Product investigator brochure
				PBO	-	67	9.9 (NS)	73			
Greenhill2006b CRIT124E2301 USA	Parallel	7	DSM-IV	d-threo MPH-ER	5-30 mg/d	53	9.6 (2.8)	58.5	NS	Yes	Journal article FDA Short CSR
				PBO	-	50	10.4 (2.7)	70			
Grizenko2012 Canada	Cross-over w/o washout	2	DSM-IV	MPH	0.5 mg/Kg/d	237	NS	NS	NS	No	Journal article Additional information from study author
				PBO	-	258					
Harfterkamp	Parallel	8	DSM-IV	ATMX	1.2	48	9.9	87.5	ASD: 98	Yes	Journal article

Study name Country	Design	Duration (weeks)	Criteria	Drug	Dose (min-max)	N	Age (M, SD)	Males (%)	Comorbid. (%)	Spons.	Source of information/data
2012 NCT00380692 Netherlands					mg/Kg/d		(2.7)				ClinicalTrials.gov
				PBO	-	49	10 (2.9)	83.7	ASD 98		
Herring2012 NCT00475735 USA	Cross-over w/ washout	4	DSM-IV	MPH-ER	54-72 mg/d	23	NS	NS	None	Yes	Journal article ClinicalTrials.gov
				PBO	-	28	NS	NS	None		
Hervas2014 SPD503-316 NCT01244490 EudraCT: 2010-018579-12 Europe USA Canada	Parallel	13	DSM-IV-TR	GUA ER	1-7 mg/d	115	10.9 (2.8)	66.7	ODD: 14.9 Oppositional Symptoms: 53.1 Other: 0.9	Yes	Journal article ClinicalTrials.gov Short CSR Additional information from manufacturer
				ATMX	0.5-1.4 mg/kg/day	112	10.5 (2.8)	77.7	ODD: 8.9 Oppositional Symptoms: 61.8 Other: 0.9		
				PBO	-	111	9.2 (2.8)	77.5	ODD: 12.6 % Oppositional Symptoms: 54.1 Other: 0.9		
Huss2014 CRIT124D2302 EUCTR2010-021533-31-DE NCT01259492 EU Colombia Singapore South Africa USA	Parallel	9	DSM-IV	MPH MR	40-40 mg/d	181	35.1 (11.4)	51.9	NS	Yes	Journal article ClinicalTrials.gov Short CSR
				MPH MR	60-60 mg/d	182	34.8 (10.8)	57.7			
				MPH MR	80-80 mg/d	181	34.9 (11.1)	52.5			
				PBO	-	181	36.8 (12.2)	55.8			
Jafarinia2012 Iran	Parallel	7	DSM-IV-TR	BUP	50-150 mg/d	22	9.4 (2.6)	65	NS	No	Journal article
				MPH IR	10-30 mg/d	22	9.7 (1.9)	70			

Study name Country	Design	Duration (weeks)	Criteria	Drug	Dose (min-max)	N	Age (M, SD)	Males (%)	Comorbid. (%)	Spons.	Source of information/data
Jain2011 NCT00556959 USA	Parallel	6	DSM-IV	CLON ER	0.4	80	9,.4 (2.9)	70.5	NS	Yes	Journal article ClinicalTrials.gov Additional information from author
				CLON ER	0.2	78	9.6 (2.9)	78.4			
				PBO	-	78	9.4 (2.9)	68.4			
Kahbazi2009 Iran	Parallel	6	DSM-IV-TR	MODA	200/300 mg/d	23	9.6 (2.1)	78	NS	No	Journal article
				PBO	-	23	8.5 (2)	73			
Kay2009a USA	Cross-over w/o washout	6	DSM-IV-TR	MAS-XR	20-50 mg/d	9	22.3 (2.1)	89.5	NS	Yes	Journal article ClinicalTrials.gov
				PBO	-	10					
Kay2009b USA	Cross-over w/o washout	6	DSM-IV-TR	ATMX	40-80 mg/d	8	22.4 (1.8)	87.5	NS	Yes	Journal article ClinicalTrials.gov
				PBO	-	8					
Kelsey2004 B4Z-US-LYBG USA	Parallel	8	DSM-IV	ATMX	1.2 mg/kg/day to 120 mg/d	133	9.5 (1.8)	70.7	ODD: 37.6% CD: 5.35	Yes	Journal article FDA Short CSR Full CSR
				PBO	-	64	9.4 (1.8)	70.3			
Kollins2011 SPD503-206 NCT00150592 USA	Parallel	6	DSM-IV-TR	GUA ER	1-3mg/d	121	12.6 (2.8)	102	NS	Yes	Journal article ClinicalTrials.gov Additional data provided by manufacturer
				PBO	-	57	12.8 (2.8)	93	NS		
Kooij2004 Netherlands	Cross-over w/ washout	3	DSM-IV	MPH IR	0.54–1.04 mg/kg	45	39.1 (NS)	53.3	DD: 3 BD: 1 AD: (Any) 63 SUD: 51 ED: 9 PD: 33	No	Journal article Additional data provided by study author

Study name Country	Design	Duration (weeks)	Criteria	Drug	Dose (min-max)	N	Age (M, SD)	Males (%)	Comorbid. (%)	Spons.	Source of information/data
				PBO	-				DD: 33<br>BD: 13<br>AD: (Any) 63<br>SUD: 51<br>ED: 9<br>PD: 33		
Kurlan2002<br>USA	Parallel	16	DSM-IV	CLON IR	0.6 mg/d	34	9.7 (1.8)	85	OCD: 15%<br>ODD: 48<br>CD: 9<br>GAD: 12<br>MDD: 9	No	Journal article<br>Additional data provided by study author
				MPH IR	60 mg/d	37	10.7 (2.0)	92	OCD: 11%<br>ODD: 33<br>CD: 8<br>GAD: 6<br>MDD: 3		
				PBO	-	32	9.7 (1.8)	91	OCD: 22%<br>ODD: 41<br>CD: 3<br>GAD: 3<br>MDD: 3		
Lin2014<br>NCT00922636<br>Taiwan<br>North America<br>Mexico<br>Puerto Rico	Parallel	8	DSM-IV-TR	MPH-ER	18-54 mg/kg	36	9.9 (NS)	75%	ODD: 27,8<br>CD: 0<br>MDD: 2.8<br>GAD: 0	Yes	Journal article<br>ClinicalTrials.gov
				PBO	-	78	11.4 (NS)	67.9%	ODD: 20.5<br>CD: 2.6<br>MDD: 0<br>GAD: 1.3		
Lin2016<br>NCT00917371<br>Taiwan	Parallel	8	DSM-IV-TR	ATMX	0.5-1.2 mg/kg	12	27.8 (8.2)	50%	-	No	Journal article<br>ClinicalTrials.gov
				PBO	-	12	32.5 (9.8)	41.67%	-		
Martenyi2010<br>B4Z-MW-LYCZ<br>NCT00386581	Parallel	6	DSM-IV	ATMX	0.8-1.8 mg/kg/day	72	9.9 (2.9)	87.5	NS	Yes	Journal article<br>ClinicalTrials.gov<br>Short CSR
				PBO	-	33	9.6	81.8	NS		

Study name Country	Design	Duration (weeks)	Criteria	Drug	Dose (min-max)	N	Age (M, SD)	Males (%)	Comorbid. (%)	Spons.	Source of information/data
Russia							(2.7)				Full CSR
McCracken2016 USA	Parallel	8	DSM-IV	GUA IR	1-3mg/d	71	10.1 (2.1)	66.2	NS	No	Journal article ClinicalTrials.gov Additional information from study author
				PBO	-	70	10.1 (2.0)	66.7	NS		
				GUA IR	1-3mg/d	71	9.9 (2.2)	72.9	NS		
McRae-Clark2010 R21DA018221 NCT00360269 USA	Parallel	12	DSM-IV	ATMX	25-100 mg/d	39	29.4 (10.0)	84.21	SUD: (marijuana) 100	No	Journal article ClinicalTrials.gov Additional data from study author
				PBO	-	39	30.4 (13)	68.42	SUD: (marijuana) 100		
Medori2008 LAMDA-I EUCTR2004-000730-37 NCT00246220 Europe	Parallel	5	DSM-IV	MPH ER	36-72 mg/d	102	33.6 (NS)	53.9	SUD: (all, historical plus current) 15.7	Yes	Journal article ClinicalTrials.gov Additional data from manufacturer
				MPH ER	36-36 mg/d	102	33.8 (NS)	45.1	SUD: (all, historical plus current) 15.7		
				MPH ER	18-18 mg/d	101	34.2 (NS)	57.4	SUD: (all, historical plus current) 12.95		
				PBO	-	96	34.5 (NS)	61.5	SUD: (all, historical plus current) 12.5		
Michelson2001 B4Z-MC-LYAC USA	Parallel	8	DSM-IV	ATMX	0.5-0.5 mg/kg/day	44	11.3 (2.5)	70.45	ODD: 47.7% DD: 0 AD: 0	Yes	Journal article FDA Short CSR Full CSR
				ATMX	1.2-1.2 mg/kg/day	84	11.5 (2.4)	71.42	ODD: 29.8% DD: 0 AD: 0		
				PBO	-	84	10.9 (2.1)	71.42	ODD: 36.9% DD: 0 AD: 1.2		
Michelson2002 B4Z-MC-LYAT	Parallel	6	DSM-IV	ATMX	1 – 1.5 mg/Kg/d	85	10.1 (2.3)	70.6	ODD: 21.2 DD: 1.2	Yes	Journal articles FDA

Study name Country	Design	Duration (weeks)	Criteria	Drug	Dose (min-max)	N	Age (M, SD)	Males (%)	Comorbid. (%)	Spons.	Source of information/data
USA									AD: 0<br>Specific phobia: 2.4		Short CSR<br>Full CSR
				PBO	-	85	10.5 (2.5)	70.6	ODD: 18.8<br>DD: 2.4<br>AD: 1.2<br>Specific phobia: 3.5		
Michelson2003a USA	Parallel	10	DSM-IV	ATMX	60-120mg/d	141	40.2 (11.7)	64.5	-	Yes	Journal article<br>FDA<br>Short CSR<br>Full CSR
				PBO	-	139	40.3 (11.6)	62.6	-		
Michelson2003b USA	Parallel	10	DSM-IV	ATMX	60-120 mg/d	129	43.0 (10.3)	64.3	-	Yes	Journal article<br>FDA<br>Short CSR<br>Full CSR
				PBO	-	127	41.2 (11.2)	68.5	-		
Moharari2012 IRCT201012295 500N1 Iran	Parallel	8	DSM-IV	MPH	0.5 mg/d	20	8.45 (1.7)	NS	NS	No	Journal article
				BUP	5 mg/Kg/d	20	9.5 (2.0)	NS			
Montoya2009 B4Z-XM-LYDM NCT00191945 Spain	Parallel	6	DSM-IV	ATMX	1.2-1.4 mg/kg/day	100	10.3 (2.5)	79	ODD: 28.3<br>TIC: 16.2<br>AD: 13.1<br>Affective disorder: 3	Yes	Journal article<br>ClinicalTrials.gov<br>Additional information from study author
				PBO	-	51	10.3 (2.4)	80.4	ODD: 20<br>TIC: 18<br>AD: 12<br>Affective disorder: 4		
NCT01069523 USA	Parallel	4	DSM-IV	GUA-ER	4mg/d	16	7.8 (1.2)	0.46	NS	Yes	ClinicalTrials.gov<br>Additional information from study author
				PBO	-	13	8.8 (1.8)	0.62			
Newcorn2008 B4Z-MC-LYBI	Parallel	6	DSM-IV	ATMX	0.8-1.2 (at week 6)	222	10.3 (2.2)	78	ODD: 39 %<br>DD: 0	Yes	Journal article<br>Short CSR

Study name Country	Design	Duration (weeks)	Criteria	Drug	Dose (min-max)	N	Age (M, SD)	Males (%)	Comorbid. (%)	Spons.	Source of information/data
USA					mg/d				AD: 0		Full CSR
				MPH ER	18-54 mg/d	220	10.2 (2.5)	72	ODD: 36 %<br>DD: 0<br>AD: 0		
				PBO	-	74	10.1 (2.7)	74	ODD: 35 %<br>DD: 0<br>AD: 0		
Newcorn2013<br>SPD503-314<br>NCT00997984<br>USA<br>Canada	Parallel	8	DSM-IV-TR	GUA ER	1-4 mg/d	113	9.1 (1.8)	67.3	NS	Yes	Journal article<br>ClinicalTrials.gov<br>Additional information from manufacturer
				GUA ER	1-4 mg mg/d	114	9.3 (1.8)	68.4	NS		
				PBO	-	113	8.9 (1.8)	75.9	NS		
Palumbo2008<br>NCT00031395<br>USA	Parallel	16	DSM-IV	MPH	5-60 mg TDS	29	9.4 (1.6)	82.8	ODD: 44.8 %<br>CD: 3.5	No	Journal article<br>ClinicalTrials.gov
				CLON	0.05-0.6 mg TDS	31	9.4 (1.2)	87.1	ODD: 43.3 %<br>CD: 16.7		
				PBO	-	30	9 (1.5)	76.7	ODD: 50 %<br>CD: 10		
Paterson1999<br>Australia	Parallel	6	DSM-IV	Dextro - AMPH	5-30 mg/d	24	NS	NS	-	No	Journal article<br>Additional information from study author
				PBO	-	21	NS	NS	-		
Philipsen2015<br>EUCTR2006-000222-31-DE<br>ISRCTN540962 01<br>Germany	Parallel	13	DSM-IV	MPH MR	10-60 mg/d	110	35 (10)	50.5%	Affective Disorder (current): 21.5%<br>AD: (current): 18.7<br>PD: (Cluster A): 0<br>PD: (Cluster B): 6.5	No	Journal article (including protocol)<br>Additional information from study author

Study name Country	Design	Duration (weeks)	Criteria	Drug	Dose (min-max)	N	Age (M, SD)	Males (%)	Comorbid. (%)	Spons.	Source of information/data
									PD (Cluster C): 9.3 SUD: 20.6		
				PBO	-	107	35 (10)	43.7%	Affective Disorder (current): 35 AD: (current): 20.4 PD: (Cluster A): 3.9 PD: (Cluster B): 3.9 PD: (Cluster C): 12.6 SUD: 10.7		
Pliszka2000 USA	Parallel	3	DSM-IV	MPH IR	5-50 mg/d	20	8.1 (1)	84.5	ODD: 70 CD: 5 AD: 20	Yes	Journal article Additional information from study author
				MAS	5-30 mg/d	20	8.6 (1.5)	84.5	ODD: 60 CD: 15 AD: 5		
				PBO	-	18	7.8 (1.7)	Missing	ODD: 55 CD: 11 AD: 5		
Reimherr2005 USA	Parallel	6	DSM-IV	BUP ER	100-400 mg/d	35	34.3 (14.8)	71.4	-	Yes	Journal article Additional information from study author
				PBO	-	24	34.6 (11.2)	75.0			
Reimherr2007 USA	Cross-over w/o washout	4	DSM-IV-TR	MPH ER	18-90 mg/d	23	30.6 (10.8)	NS	-	Yes	Journal article ClinicalTrials.gov Additional information from study author
				PBO	-	24			-		

Study name Country	Design	Duration (weeks)	Criteria	Drug	Dose (min-max)	N	Age (M, SD)	Males (%)	Comorbid. (%)	Spons.	Source of information/data
Rosler2009 Germany	Parallel	24	DSM-IV	MPH MR	10-60 mg/day	241	35.2 (10.1)	50	BD: 2<br>DD: 12<br>SUD (Alcohol): 2<br>SUD (drug): 5<br>AD (Panic Disorder): 2<br>AD (phobia): 20<br>AD (GAD): 1<br>AD (OCD): 7<br>AD (PTSD): 2	Yes	Journal article ClinicalTrials.gov Additional information from manufacturer
				PBO	-	118	33.8 (10.6)	50	BD: 1<br>DD: 6<br>SUD (Alcohol): 3<br>SUD (drug): 4<br>AD (Panic Disorder): 1<br>AD (phobia): 9<br>AD (GAD): 0.4<br>AD (OCD): 2<br>AD (PTSD): 1		
Rugino2003 USA	Parallel	6	DSM-IV	MODA	200-300 mg/d	13	7.6 (1.9)	63.6	Specific phobia: 36.6<br>ODD/CD: 27.27<br>Enuresis: 18.18	No	Journal article
				PBO	-	11	8.2 (2.3)	63.6	Separation Anxiety: 27.27<br>ODD/CD: 27.17<br>Enuresis: 9.09		
Rugino2014	Parallel	5	DSM-IV	GUA	1-4	12	9.2	81.8	NS	Yes	Journal article

Study name Country	Design	Duration (weeks)	Criteria	Drug	Dose (min-max)	N	Age (M, SD)	Males (%)	Comorbid. (%)	Spons.	Source of information/data
NCT01156051 USA				ER	mg/d		(1.7)				ClinicalTrials.gov
				PBO	-	17	8.8 (1.9)	50			
Sallee2009 SPD503-304 NCT00150618 USA	Parallel	4	DSM-IV-TR	GUA ER	1 mg/d	62	9.3 (2.2)	66.1	NS	Yes	Journal article ClinicalTrials.gov FDA Product monograph Additional data from manufacturer
				GUA ER	2 mg/d	65	10.6 (2.8)	70.7			
				GUA ER	3 mg/d	65	11.1 (2.9)	73.8			
				GUA ER	4 mg/d	66	10.5 (2.5)	81.5			
				PBO	-	66	10.8 (2.9)	68.1			
Sangal2006 B4Z-US-LYAV USA	Cross-over w/ washout	7	DSM-IV	ATMX	15-100 mg/d	85	10.1 (2.0)	73.5	ODD: 48.2 CD: 3.5 Agoraphobia: 1.2	Yes	Journal article Additional data from manufacturer
				MPH	15-60 mg/d				ODD: 48.2 CD: 3.5 Agoraphobia: 1.2		
Schahill2001 NCT00004376 USA	Parallel	8	DSM-IV	GUA	4 mg/d	17	10.4 (2.0)	NS	NS	No	Journal article ClinicalTrials.gov
				PBO	-	17					
Schrantee2016 NTR3103 EUCTR2010-023654-37-NL Netherlands	Parallel	16	DSM-IV	MPH	0.5-20 mg/d	25	11.4 (0.8)	100	NS	No	Journal article Additional data from study author
				PBO	-	25	11.3 (0.9)	100			
				MPH	0.5-40 mg/d	25	28.6 (4.6)	100			
				PBO	-	24	29 (4.9)	100			
Schulz2012 USA	Parallel	6-8	DSM-IV	MPH-ER	18-72 mg/d	21	11 (2.4)	83	ODD: 44	Yes	Journal article
				ATMX	0.8-1.8	21	11.4	83	ODD: 39		

Study name Country	Design	Duration (weeks)	Criteria	Drug	Dose (min-max)	N	Age (M, SD)	Males (%)	Comorbid. (%)	Spons.	Source of information/data
					mg/Kg/d		(3.0)				
Simonoff2013 ISRCTN68384912 UK	Parallel	16	ICD-10	MPH	0.5-1.5 mg/kg/day	61	10.8 (2.4)	74	Intellectual disability: 100	No	Journal article Additional data from study author
				PBO	-	61	11.5 (2.3)	66			
Singer1995 USA	Cross-over w/ wash out	6	DSM-III-R	CLON IR	0.2 mg/d	37	NS	NS	NS	No	Journal article
				PBO	-						
SPD489-405 NCT01552915 USA	Parallel	8	DSM-IV-TR	MPH-ER	18-72 mg/d	184	14.7 (1.4)	66.3	NS	Yes	ClinicalTrials.gov Additional data from manufacturer
				LDX	30-70 mg/d	184	14.7 (1.3)	66.3			
				PBO	-	91	14.8 (1.4)	67.0			
SPD489-406 NCT01552902 USA Canada Europe	Parallel	6	DSM-IV-TR	MPH-ER	18-72 mg/d	219	14.7 (1.4)	68.4	NS	Yes	ClinicalTrials.gov Additional data from manufacturer
				LDX	30-70 mg/d	218	14.6 (1.4)	61.9			
				PBO	-	110	14.7 (1.4)	69.0			
Spencer1995 USA	Cross-over w/ washout	3	DSM-III-R	MPH	up to 1 mg/kg	25	40 (2.1)	43	DD: 52 AD: 39 SUD (drugs): 9 SUD (alcohol): 17 PD (Antisocial): 4 CD: 13	No	Journal article
				PBO	-				DD: 52 AD: 39 SUD (drugs): 9 SUD (alcohol): 17 PD (Antisocial): 4 CD: 13		

Study name Country	Design	Duration (weeks)	Criteria	Drug	Dose (min-max)	N	Age (M, SD)	Males (%)	Comorbid. (%)	Spons.	Source of information/data
Spencer1998 USA	Cross-over w/ washout	3	DSM-III-R	ATMX	40-80 mg/kg	22	34.0 (9.0)	47.62%	-	Yes	Journal article
				PBO	-				-		
Spencer2001 USA	Cross-over w/ washout	3	DSM-IV	MAS	Up to 40 mg/d	30	38.8 (9.3)	56	DD: 44 AD (at least two): 19 SUD (drugs): 15 SUD (alcohol): 26 PD (Antisocial): 22 CD: 22	Yes	Journal article
				PBO	-				DD: 44 AD (at least two): 19 SUD (drugs): 15 SUD (alcohol): 26 PD (Antisocial): 22% CD: 22%		
Spencer 2002a B4Z-MC-HFBD USA	Parallel	9	DSM-IV	ATMX	90 mg/day	64	9.8 (1.6)	79.7	NS	Yes	Journal article FDA Short CSR Full CSR
				MPH	60 mg/kg/day	18	9.7 (1.4)	88.9			
				PBO	-	62	9.9 (1.4)	80.6			
Spencer 2002b B4Z-MC-HFBK USA	Parallel	9	DSM-IV	ATMX	90 mg/day	65	9.1 (1.6)	72.3	NS	Yes	Journal article FDA Short CSR Full CSR
				MPH	60 mg/kg/day	20	9.6 (1.6)	95.0			

Study name Country	Design	Duration (weeks)	Criteria	Drug	Dose (min-max)	N	Age (M, SD)	Males (%)	Comorbid. (%)	Spons.	Source of information/data
				PBO	-	62	10.0 (1.6)	85.5			
Spencer2005 USA	Parallel	6	DSM-IV	MPH	1.3 mg/kg/day	104	35.6 (9.7)	59.6	MDD: 9 AD: (at least 2): 3 ODD: 4 (current)	No	Journal article
				PBO	-	42	40.3 (10)	54.8	MDD: 7 ODD: 2 (current)		
Spencer2006 SLI381-314 NCT00507065 USA	Parallel	4	DSM-IV-TR	MAS ER	10 mg/d	56	14.4 (1.2)	61.1	NS	Yes	Journal article ClinicalTrials.gov Additional data from manufacturer
				MAS ER	20 mg/d	56	14.2 (1.2)	69.8			
				MAS ER	30 mg/d	58	14.2 (1.2)	65.5			
				MAS ER	40 mg/d	63	14(1.2)	63.9			
				PBO	-	54	14.5 (1.3)	67.3			
Spencer2007 CRIT124E2302 USA	Parallel	5	DSM-IV	d-threo MPH ER	20-40 mg/d	168	38.8 (NS)	59.5	NS	Yes	Journal article FDA Short CSR
				PBO	-	53	38.1 (NS)	50.94	NS		
Spencer2008 SPD465-301, NCT00150579 USA	Parallel	7	DSM-IV-TR	MAS	12.5-75 mg/d	137	36.1 (10.1)	50.4	NS	Yes	Journal article ClinicalTrials.gov Additional data from manufacturer
				PBO	-	137	37 (10.3)	49.6	NS		
Stein2011 NCT00393042 USA	Cross-over w/o washout	8	DSM-IV	d-threo MPH ER	10 mg/d	20	11.8 (2.2)	73	NS	Yes	Journal article ClinicalTrials.gov Additional data from study author
				MAS ER	10 mg/d	20	11.8 (2.2)	73	NS		
				PBO	-	24	11.8 (2.2)	73	NS		

Study name Country	Design	Duration (weeks)	Criteria	Drug	Dose (min-max)	N	Age (M, SD)	Males (%)	Comorbid. (%)	Spons.	Source of information/data
Sutherland2012 NCT00174226 USA	Parallel	7	DSM-IV-TR	ATMX	40-100 mg/d	97	NS	NS	NS	Yes	Journal article ClinicalTrials.gov
				PBO	-	47	NS	NS	NS		
Svanborg2009 B4Z-SO-LY15 EUCTR2004-003941-42-SE NCT00191542 Sweden	Parallel	10	DSM-IV	ATMX	0.5-1.2 mg/kg/day	49	11.6 (2.3)	39	ODD: 22.4 DD: 4.1 Tics: 12.2	Yes	Journal article ClinicalTrials.gov
				PBO	-	50	11.3 (2.1)	41	ODD: 18 DD: 6 Tics: 16		
Swanson2006 USA	Parallel	7	DSM-IV-TR	MODA	340-425 mg/d	126	10.1 (NS)	74	NS	Yes	Journal article FDA Investigator brochure
				PBO	-	64	9.7 (NS)	77	NS		
Takahashi2009 B4Z-JE-LYBC NCT00191295 Japan	Parallel	8	DSM-IV	ATMX	0.5 mg/kg/day	62	10.2 (2.6)	83.9	NS	Yes	Journal article ClinicalTrials.gov FDA Short CSR
				ATMX	1.2 mg/kg/day	60	10.6 (2.74)	86.7	NS		
				ATMX	1.8 mg/kg/day	61	10.5 (2.7)	86.9	NS		
				PBO	-	62	10.7 (2.0)	83.9	NS		
Takahashi2014 NCT01323192 Japan	Parallel	8	DSM-IV-TR	MPH ER	18-72 mg/d	143	33.4 (8.8)	49.7	NS	Yes	Journal article ClinicalTrials.gov Short CSR
				PBO	-	141	34.1 (9.0)	48.2	NS		
Taylor1987 UK	Cross-over w/ washout	3	DSM-III	MPH	30 mg/d	24	NS	NS	NS	Yes	Journal article Additional information from study author
				PBO	-				NS		
Taylor2000 USA	Cross-over w/ washout	2	DSM-IV	MODA	50-400 mg/d	22	40.8 (12.5)	59	DD: 46	No	Journal article
				Dextro - AMPH	5-40 mg/d						

Study name Country	Design	Duration (weeks)	Criteria	Drug	Dose (min-max)	N	Age (M, SD)	Males (%)	Comorbid. (%)	Spons.	Source of information/data
				IR							
				PBO	-						
Taylor2001 USA	Cross-over w/ washout	2	DSM-IV	GUA IR	0.25-2 mg/d	17	41.2 (11.4)	41	NS	No	Journal article
				Dextro - AMPH	2.5-20 mg/d				NS		
				PBO	-				NS		
Van der Meere1999 Netherlands	Parallel	7	DSM-III-R	MPH	0.6 mg/Kg/d	24	NS	NS	NS	Yes	Journal article Thesis
				CLON	4.0 microg/Kg/d	24	NS				
				PBO	-	24	NS				
Wang2007 NCT00486083, B4Z-MC-LYBR (6934) China, Korea, Mexico	Parallel	8	DSM-IV	ATMX	0.8-1.8 mg/kg/day	164	9.4 (2,0)	82.9	ODD: 24.4	Yes	Journal article FDA ClinicalTrials.gov Short CSR Additional information from manufacturer
				MPH	0.2-0.6 mg/kg/twice day	166	9.9 (2.3)	80.7	ODD: 17.5		
Wehmeier 2012 B4Z-SB-LYDV NCT00546910 Germany	Parallel	8	DSM-IV-TR	ATMX	1.2 mg/kg/day	64	9.1 (1.9)	74.6	ODD: 31.7 CD: 14.3 Tic: 1.6	Yes	Journal article ClinicalTrials.gov Additional information from manufacturer
				PBO	-	64	8.9 (1.6)	80.6	ODD: 30.6 CD: 19.4 MOOD: 1.6		
Weisler2006 SLI381-303 USA	Parallel	4	DSM-IV-TR	MAS ER	40 mg/d	64	38.9 (NS)	48	-	Yes	Journal article Additional data from manufacturer
				MAS ER	20 mg/d	66	38.8 (NS)	64	-		
				PBO	-	64	39.3 (NS)	68	-		
Weisler2012 NCT00880217 USA	Parallel	6	DSM-IV-TR	ATMX	80 mg/d	74	34.6 (10.4)	53.4	None	Yes	Journal article ClinicalTrials.gov
				MPH	54 mg/d	68	33.2	66.2			

Study name Country	Design	Duration (weeks)	Criteria	Drug	Dose (min-max)	N	Age (M, SD)	Males (%)	Comorbid. (%)	Spons.	Source of information/data
				ER			(9.7)				Additional information from manufacturer
				PBO	-	74	33.4 (10.3)	58.9			
Weiss2005 B4Z-MC-LYAW USA, Canada, Puerto Rico	Parallel	7	DSM-IV	ATMX	1.2-1.8 mg/kg/day	101	9.9 (1.4)	82.2	ODD: 32.7 AD: 3.0 LD: 29.3 MSD: 4.9 Comm.: 4.9	Yes	Journal article FDA Short CSR Full CSR
				PBO	-	52	9.9 (1.3)	76.9	ODD: 34.6 AD: 1.9 LD: 31.0 MSD: 9.5 Comm.: 14.3		
Wender2011 USA	Cross-over w/o washout	2	Equivalent to DSM-IV, comb. type	MPH	10-90 mg/d	59	NS	NS	None	No	Journal article ClinicalTrials.gov Additional data from study author
				PBO	-	57					
Wietecha2013 NCT00607919 USA	Parallel	16	DSM-IV	ATMX	1.0-1.4 mg/kg/day	64	12.2 (1.7)	60.9	NS	Yes	Journal article ClinicalTrials.gov Additional data from manufacturer
				PBO	-	60	12.3 (1.9)	66.7			
Wigal2004 USA	Parallel	4	DSM-IV	d-threo MPH	2.5-10 mg bid	44	10.0 (2.5)	93.2	NS	Yes	Journal article FDA
				d,l-threoMPH IR	5-20 mg bid	46	9.8 (2.8)	87.0			
				PBO	-	42	9.6 (2.7)	83.3			
Wigal2005 SLI381-404 NCT00506727 USA	Parallel	3	DSM-IV-TR	MAS ER	10-30 mg/day	107	8.8 (1.8)	74.5	NS	Yes	Journal article ClinicalTrials.gov Additional information from manufacturer
				ATMX	1.2-1.4 mg/kg/day	108	8.6 (1.8)	69.3			
Wigal2015 NCT01239030 USA	Parallel	1	DSM-IV-TR	MPH ER	10 mg/d	49	10.5 (2.9)	61.2	NS	Yes	Journal article ClinicalTrials.gov

Study name Country	Design	Duration (weeks)	Criteria	Drug	Dose (min-max)	N	Age (M, SD)	Males (%)	Comorbid. (%)	Spons.	Source of information/data
				MPH ER	15 mg/d	44	10.2 (3.1)	68.2	NS		
				MPH ER	20 mg/d	45	11.1 (3.5)	68.9	NS		
				MPH ER	40 mg/d	45	11.2 (2.5)	73.3	NS		
				PBO	-	47	10.9 (3.1)	63.8	NS		
Wilens2001 USA	Parallel	6	DSM-IV	BUP ER	100-200 mg bid	21	37 (11.8)	57	DD: 32 AD: 5 Smoking: 14	Yes	Journal article
				PBO	-	19	39.6 (10.4)	53	DD: 6 AD: 11 Smoking: 5		
Wilens2005 NCT00048360 USA	Parallel	8	DSM-IV	BUP ER	300-450 mg/d	81	39.1 (10.3)	60	Smoking: 14	Yes	Journal article ClinicalTrials.gov
				PBO	-	81	41.4 (10)	59	Smoking: 15		
Wilens2008 B4Z-MC-LYBY NCT00190957 USA and Canada	Parallel	12	DSM-IV-TR	ATMX	80-100 mg/d	72	34.3 (10.2)	84.7	SUD (Alcohol abuse): 45.8 SUD (Alcohol dependence): 54.2	Yes	Journal article ClinicalTrials.gov Short CSR Full CSR
				PBO	-	75	34.8 (9.9)	85.3	SUD (Alcohol abuse): 42.7 SUD (Alcohol dependence): 57.3		
Wilens2011 NCT00528697 USA	Parallel	8	DSM-IV	ATMX	0.5-1.2 mg/Kg/d	50	8.7 (2.0)	69	NS	Yes	Journal article ClinicalTrials.gov
				PBO	-	47	8.6 (1.8)	61			
Wilens2015 SPD503-312	Parallel	13	DSM-IV-TR	GUA-ER	1-7 mg/Kg/d	157	14.5 (1.4)	64.7	ODD: 12.7	Yes	Journal article ClinicalTrials.gov

Study name Country	Design	Duration (weeks)	Criteria	Drug	Dose (min-max)	N	Age (M, SD)	Males (%)	Comorbid. (%)	Spons.	Source of information/data
EUCTR2011-002221-21 NCT01081132 USA				PBO	-	157	14.6 (1.4)		ODD: 10.3		Additional data from manufacturer
Winhusen2010 NCT00253747 USA	Parallel	11	DSM-IV	MPH ER	18-72 mg/d	127	38.1 (10.4)	60.6	DD: 32.3 AD: 34.6 SUD: 63 Smoking: 100	No	Journal article ClinicalTrials.gov Additional information from study author
				PBO	-	128	37.5 (9.6)	52.3	DD: 35.9 AD: 32.8 SUD: 58.6 Smoking:100		
Young2011 B4Z-US-LYCW NCT00190775 USA	Parallel	24	DSM-IV-TR	ATMX	60-100 mg/d	268	41.2 (6.9)	51.1	NS	Yes	Journal article ClinicalTrials.gov Full CSR
				PBO	-	234	41.4 (7.5)	43.6			

Abbreviation for Medications: AMPH: Amphetamines; BUP: Bupropion; CLON: Clonidine; GUA: Guanfacine; GXR: Guanfacine Extended Release LDX: Lisdexamfetamine; MAS: Mixed Amphetamine Salts; MODA: Modafinil; MPH: Methylphenidate (ER: Extended release: SR: sustained release); PBO: Placebo.
Abbreviation for Comorbidity: AD: Aggression/Defiance; A/D: Abuse/Dependence; Adj Dis: Adjustment Disorder with mixed disturbance of emotions and conduct; ASD: Autism Spectrum Disorder; ASPD: Antisocial Personality Disorder; BD: Bipolar Disorder; CD: Conduct Disorder; Comm: Communications Disorder; DD: Depression Disorder; Disr Beh: Disruptive Behavior Disorder; GAD: Generalized Anxiety Disorder; LD: Learning disorder; MD: Major Depression; MOOD: Mood disorder; MSD: Motor Skills Disorder; OCD: Obsessive–Compulsive Disorder; ODD: Oppositional Defiant Disorder; PD: Personality disorder; PHO: Phobia; SAD: Separation anxiety disorder; SPD: Seasonal pattern disorders; SUD: substance use disorder; TD: Tic Disorders.

Tables S11. Rating of individual items of the Risk of Bias tool for each study

Note: CSR: Clinical Study Report.

Abikoff 2007

ITEM	RATING	SUPPORT
**Sequence generation**	UNCLEAR	"Children received MPH-OROS and placebo in a random order, double-blind" (Journal article, pag. 168), but no details on randomization sequence generation.
**Allocation concealment**	UNCLEAR	No information.
**Blinding participants/parents**	UNCLEAR	"Double-blind" (Journal article, pag. 168), but no detail on how blinding was preserved.
**Blinding therapist**	UNCLEAR	"Double-blind" (Journal article, pag. 168), but no detail on how blinding was preserved.
**Blinding assessor**	UNCLEAR	"Double-blind" (Journal article, pag. 168), but no detail on how blinding was preserved.
**Incomplete data outcome**	LOW	"Posttreatment scores for MPH-OROS and placebo were obtained from parents on all 19 study children. Because one child's treatment was delayed and ran beyond the end of the school year, teacher data were analyzed for 18 youngsters." (Journal article, pag. 170).
**Selective reporting**	UNCLEAR	No protocol/CSR/CT available.
**Notes**		First author contacted but not able to provide additional information.

Adler 2008a, B4Z-MC-LYBV, NCT00190931

ITEM	RATING	SUPPORT
**Sequence generation**	LOW	Information from full CSR, pag. 103 (available upon request from the manufacturer).
**Allocation concealment**	LOW	Information from full CSR, pag. 103 (available upon request from the manufacturer).
**Blinding participants/parents**	UNCLEAR	Additional information from full CSR, pag. 26 and 103 (available upon request from the manufacturer), but not details on how blinding was preserved.
**Blinding therapist**	UNCLEAR	Additional information from full CSR, pag. 26 and 103 (available upon request from the manufacturer), but not details on how blinding was preserved.
**Blinding assessor**	UNCLEAR	Additional information from full CSR, pag. 26 and 103 (available upon request from the manufacturer), but not details on how blinding was preserved.
**Incomplete data outcome**	UNCLEAR	Information from full CSR, pag. 103 (available upon request from the manufacturer).<br>Variable number of subjects included in the ITT analysis: of the 410 patients randomized, between 294

		(71.7%) and 385 (93.9%) included in ITT analysis for primary outcome (Journal article, Figure 3). Balanced drop outs for efficacy: "Patients did not differ between groups in their reason for discontinuation (Table 2), with the exception of adverse events, for which the percentage was greater for the atomoxetine group (14.0%) compared to the placebo group (2.2%, *p*= .001)." (Journal article, pag. 723).
**Selective reporting**	LOW	Outcomes relevant for the present meta-analysis mentioned in the full CSR were reported in the Journal article or in the publically available short CSR.
**Notes**		Manufacturer provided full CSR.

<u>Adler 2008b, NRP104.303, NCT00334880</u>

ITEM	RATING	SUPPORT
**Sequence generation**	LOW	Information from full CSR, Section 9.4.3 Method of Assigning Subjects to Treatment Groups, pag. 32 (available upon request from the manufacturer).
**Allocation concealment**	LOW	Information from full CSR, Section 9.4.3 Method of Assigning Subjects to Treatment Groups, pag. 32 (available upon request from the manufacturer).
**Blinding participants/parents**	LOW	"Investigator and the patient were blinded to treatment. To maintain blinding, all investigational products were supplied as capsules identical in size, weight, and shape." (Journal article, pag. 1366). Information from full CSR, Blinding, pag. 33 (available upon request from the manufacturer).
**Blinding therapist**	LOW	"Investigator and the patient were blinded to treatment. To maintain blinding, all investigational products were supplied as capsules identical in size, weight, and shape." (Journal article, pag. 1366). Information from full CSR, Blinding, pag. 33 (available upon request from the manufacturer).
**Blinding assessor**	LOW	"Investigator and the patient were blinded to treatment. To maintain blinding, all investigational products were supplied as capsules identical in size, weight, and shape." (Journal article, pag. 1366). Information from full CSR, Blinding, pag. 33 (available upon request from the manufacturer).
**Incomplete data outcome**	LOW	420 randomized, 414 analyzed in ITT (Journal article, pag. 1367). Drop out rate < 20% ("Of the 420 enrolled subjects, 71 (17%) terminated before study completion") (Journal article, pag. 1367). Balanced reasons for drop outs for lack of efficacy (LDX 30 mg/day:1/119; LDX 50 mg/day: 2/117; LDX 70 mg/day:1/122)) (Journal article, Table 1). LOCF.
**Selective reporting**	LOW	Manufacturer confirmed that "All outcomes were reported in published papers".
**Notes**		Manufacturer provided additional information.

<u>Adler 2009a, B4Z-US-LYDQ, NCT00190879</u>

ITEM	RATING	SUPPORT
**Sequence**	LOW	"Eligible patients were randomized to a treatment group at Visit 2 (Week 0) via a computer algorithm that

**generation**		blindly assigned patients to either study drug or PBO at a 1:1 ratio at the site level. The treatment assignments were kept blinded until after the database was locked." (Journal article, Pag. 213).
**Allocation concealment**	LOW	"Investigative sites dispensed the blinded study drug, via telephone interactive voice response system, at the end of Visit 2 and instructed patients to begin dosing the next morning." (Journal article, Pag. 213).
**Blinding participants/parents**	UNCLEAR	"To maintain the double- blind, patients and site personnel were informed that a PBO treatment period would occur at some point during the study but were blinded to the timing and duration." (Journal article, Pag. 214) but no details on how blinding was preserved.
**Blinding therapist**	UNCLEAR	"To maintain the double- blind, patients and site personnel were informed that a PBO treatment period would occur at some point during the study but were blinded to the timing and duration. "(Journal article, Pag. 214) but no details on how blinding was preserved.
**Blinding assessor**	UNCLEAR	"To maintain the double- blind, patients and site personnel were informed that a PBO treatment period would occur at some point during the study but were blinded to the timing and duration. "(Journal article, Pag. 214) but no details on how blinding was preserved.
**Incomplete data outcome**	UNCLEAR	A sizable portion of subjects who dropped out was not included in the ITT analysis: Randomised n=442; ITT analysis n=329 (74.4%). (Journal article, pag. 215)<br>Quite high, but balanced, drop out: "Of randomized patients, 56.7% (127/224) randomized to ATX and 62.8% (137/218) randomized to PBO completed the study." (Journal article, pag. 215)<br>Quite balanced reasons for drop out in placebo lead in phase; balanced reasons for drop out for lack of efficacy (active drug: 9/224; PBO:8/218) (Journal article, figure 1)
**Selective reporting**	LOW	Outcomes relevant for the present meta-analysis mentioned in the full CSR were reported in the Journal article.
**Notes**		CSR available.

Adler 2009b, B4Z-US-LYCU, NCT00190736

ITEM	RATING	SUPPORT
**Sequence generation**	LOW	"A computer algorithm generated randomization numbers to blindly assign patients to study drug or placebo in a 1:1 fashion at the site level."(Journal article, pag. 45).
**Allocation concealment**	LOW	"A computer algorithm generated randomization numbers to blindly assign patients to study drug or placebo in a 1:1 fashion at the site level. These randomization numbers were made available to the investigative site via a telephone Interactive Voice Response System, and the treatment assignements were not unblinded until after the database was locked." (Journal article, pag. 45).
**Blinding participants/parents**	LOW	Information from full CSR, pag. 144 (available upon request from the manufacturer).
**Blinding therapist**	LOW	Information from full CSR, pag. 144 (available upon request from the manufacturer).
**Blinding assessor**	LOW	Information from full CSR, pag. 144 (available upon request from the manufacturer).

**Incomplete data outcome**	LOW	Information from full CSR, pag. 73 (available upon request from the manufacturer).
**Selective reporting**	LOW	Outcomes relevant for the present meta-analysis mentioned in the full CSR were reported in the Journal article.
**Notes**		Manufacturer provided full CSR.

Adler 2009c, CR011560, NCT00326391

ITEM	RATING	SUPPORT
**Sequence generation**	LOW	“Subjects were randomized using a computer-generated randomization schedule” (Journal article, pag. 240)
**Allocation concealment**	LOW	“To randomize subjects, a qualified study staff used an interactive voice recognition system and entered the subject’s date of birth, sex, and responses to selected eligibility questions. The system first verified that each subject randomized was unique and then, following the randomization schedule, identified the unique kit number of the dosing package that the study staff was to dispense to the subject at the baseline visit. (Journal article, pag. 240)
**Blinding participants/parents**	LOW	“Each investigator received an allotment of double-blind medication before the study started, and each subject received overencapsulated tablets that appeared identical to the treatment of all other subjects at the beginning of the study” (Journal article, pag. 240)
**Blinding therapist**	LOW	Additonal information provided by the manufacturer, available upon request
**Blinding assessor**	LOW	Additonal information provided by the manufacturer, available upon request
**Incomplete data outcome**	LOW	“Overall, 348 subjects were assessed for eligibility, and 229 subjects were randomized to treatment (113 subjects randomized to OROS methylphenidate and 116 subjects randomized to placebo). Three subjects randomized to OROS methylphenidate did not meet inclusion/exclusion criteria and were withdrawn from the study before they received study medication. Therefore, 226 subjects were included in the ITT population (110 in the OROS methylphenidate group and 116 in the placebo group).“ (Journal article, pag. 242).<br>“The primary efficacy analysis was performed using the intention-to-treat (ITT) population, which included all subjects who were randomized during the study and who were dispensed study medication. The analyses used a LOCF approach” (Journal article, pag. 241).<br>Moderate to high drop out rate after randomization, quite unbalanced between arms (“In the OROS methylphenidate group, 37.2% (42/113) of the subjects withdrew. In the placebo group, 22.4% (26/116) of the subjects withdrew” (Journal article, pag. 242). Reasons for withdrawal quite balanced: “The reasons for withdrawal among subjects who withdrew were adverse events (OROS methylphenidate, 16/42 subjects; placebo, 6/26 subjects), subjects’ request (OROS methylphenidate, 8/42 subjects; placebo, 5/26 subjects), non adherence (OROS methylphenidate, 5/42 subjects; placebo, 5/26 subjects), and other

		reasons (OROS methylphenidate, 2/42 subjects; placebo, 6/26 subjects)" (Journal article, pag. 242).
**Selective reporting**	LOW	Outcomes relevant for the present meta-analysis mentioned in the CSR were reported in the Journal article.
**Notes**		CSR available. Additional information provided by the manufacturer.

Adler 2013, SPD489-403, NCT01101022

ITEM	RATING	SUPPORT
**Sequence generation**	LOW	"To protect the study blind, the Interactive Voice/Web Response System (Oracle/Phase Forward; Waltham, Massachusetts) was used to randomize participants and for treatment allocation." (Journal article, pag. 696)
**Allocation concealment**	LOW	"To protect the study blind, the Interactive Voice/Web Response System (Oracle/Phase Forward; Waltham, Massachusetts) was used to randomize participants and for treatment allocation" (Journal article, pag. 696)
**Blinding participants/parents**	LOW	"Medication, lisdexamfetamine dimesylate or matching placebo capsules" (Journal article, pag. 696)
**Blinding therapist**	UNCLEAR	Double-blind but not clear who else, other than the participants, was blinded.
**Blinding assessor**	UNCLEAR	Double-blind but not clear who else, other than the participants, was blinded.
**Incomplete data outcome**	UNCLEAR	Low attrition between randomized and ITT. "Of the 161 adults enrolled, 80 were randomized to receive lisdexamfetamine dimesylate and 81 to receive placebo. The safety population included 79 participants from the lisdexamfetamine dimesylate group and 80 from the placebo group; the full analysis set included 79 participants in the lisdexamfetamine dimesylate group and 75 in the placebo group." (Journal article,

		pag. 697).<br>However, moderate to-high-dropout rate post randomization (21.5% in the active medication arm and 33.8 % in the placebo arm) and unbalanced reasons for drop out for lack of efficacy (Journal article, figure 1) so that despite LOCF, bias may still occur.
**Selective reporting**	LOW	Information form manufacturer: some outcomes were not reported in the Journal article. However, none of these is relevant for the present meta-analysis.
**Notes**		Manufacturer provided additional information from the full CSR.

Allen 2005, B4Z-MC-LYAS

ITEM	RATING	SUPPORT
**Sequence generation**	LOW	"Randomization was carried out at Visit 2 by a computerized Interactive Voice Response System." (Journal article, pag. 1942)
**Allocation concealment**	LOW	"Randomization was carried out at Visit 2 by a computerized Interactive Voice Response System." (Journal article, pag. 1942)
**Blinding participants/parents**	LOW	Information from full CSR, pag. 50-1, available upon request from the manufacturer.
**Blinding therapist**	LOW	Information from full CSR, pag. 51, available upon request from the manufacturer.
**Blinding assessor**	LOW	Information from full CSR, pag. 51, available upon request from the manufacturer.
**Incomplete data outcome**	LOW	Low attrition between randomized and analyzed (148 randomized, 145 analyzed); Balanced reasons for drop out for lack of efficacy in the two arms (n= 38/76 in active drug arm, n= 45/72 in placebo arm; Journal article, figure 1); LOCF.
**Selective reporting**	HIGH	Outcomes relevant for the present meta-analysis mentioned in the full CSR were reported in the Journal article, except for CTRS-R:S, (secondary outcome of the study) reported only in the full CSR, not publically available.
**Notes**		Manufacturer provided the full CSR.

Amiri, 2008

ITEM	RATING	SUPPORT
**Sequence generation**	LOW	"Patients were randomized to receive modafinil film-coated tablets or methylphenidate in a 1:1 ratio using a computer-generated code" (Journal article, pag. 146).
**Allocation concealment**	LOW	"The assignments were kept in sealed, opaque envelopes until the point of analysis of data. The randomization and allocation process was done by the pharmacist at the Roozbeh Hospital." (Journal

		article, pag. 146).
**Blinding participants/parents**	LOW	"Throughout the study the person who administrated the medications, the rater and the patients along with their parents were blind to group assignments." (Journal article, pag. 147).<br>"Both tablets were encapsulated and were identical." (Journal article, pag. 146).
**Blinding therapist**	LOW	"Throughout the study the person who administrated the medications, the rater and the patients along with their parents were blind to group assignments." (Journal article, pag. 147).<br>"Both tablets were encapsulated and were identical." (Journal article, pag. 146).
**Blinding assessor**	LOW	"Throughout the study the person who administrated the medications, the rater and the patients along with their parents were blind to group assignments." (Journal article, pag. 147).<br>"Both tablets were encapsulated and were identical." (Journal article, pag. 146).
**Incomplete data outcome**	LOW	Low and balanced drop out rate and reasons for drop out: "Two patients dropped out from the modafinil group and three from the methylphenidate group due to lost to follow up (lack of collaboration of parents), leaving 55 patients who completed the trial" (Journal article, pag. 147).
**Selective reporting**	UNCLEAR	No protocol/CSR/CT available.
**Notes**		Author contacted but no reply.

Arnold 2006

ITEM	RATING	SUPPORT
**Sequence generation**	UNCLEAR	"Don't recall for sure, but it was probably something like this: the unblinded dispenser generated randomization blocks of 4 for the first 8-12 participants and blocks of 2 after that by flipping a coin." (E-mail from first author).
**Allocation concealment**	LOW	"Only the unblinded medication dispenser knew the randomized sequence and did not meet the participant or parent until after the final assessment." (E-mail from first author).
**Blinding participants/parents**	LOW	"Matched placebo and ATX in six sizes from 2.5 to 40 mg were supplied by the manufacturer." (E-mail from first author). First author confirmed participants were blinded.
**Blinding therapist**	LOW	"Only the unblinded medication dispenser knew the randomized sequence and did not meet the participant or parent until after the final assessment." (communication from first author). Therefore, although unblinded, the dispenser did not bias results.
**Blinding assessor**	LOW	"Matched placebo and ATX in six sizes from 2.5 to 40 mg were supplied by the manufacturer." (E-mail from first author). First author confirmed assessors were blinded.
**Incomplete data outcome**	LOW	Low drop out rate (< 20%). Balanced drop out. "Sixteen subjects (Table 1) were randomized. Three terminated early, one each after the third, fourth, and fifth weeks of the second condition (one on ATX, two on placebo). The intent-to-treat analysis included all 16" (Journal article, pag. 1198-99)
**Selective reporting**	UNCLEAR	No protocol/CSR/CT available.
**Notes**		First author provided additional information.

Arnold 2014, C1538/2027/AD/US, NCT00315276

ITEM	RATING	SUPPORT
**Sequence generation**	UNCLEAR	No description of sequence generation.
**Allocation concealment**	UNCLEAR	No details on allocation concealment.
**Blinding participants/parents**	LOW	"Patients and investigators were blinded to treatment assignement during the study until the database was locked for analysis and the treatment assignments unblinded…..matching placebo" (Journal article, Pag. 135).
**Blinding therapist**	LOW	"Patients and investigators were blinded to treatment assignement during the study until the database was locked for analysis and the treatment assignments unblinded…..matching placebo" (Journal article, Pag. 135).
**Blinding assessor**	LOW	"Patients and investigators were blinded to treatment assignement during the study until the database was locked for analysis and the treatment assignments unblinded…..matching placebo" (Journal article, Pag. 135).
**Incomplete data outcome**	LOW	"The intent-to-treat (ITT) population included all patients who received the study drug and had at least one postbaseline AISRS assessment. The safety analysis set included all patients who received at least 1 dose of the study drug. Efficacy analyses were performed using the ITT population" (Journal article, pag.135)<br>"Of the 456 patients screened, 338 met entry criteria and were randomized (Figure 1). Of these, 330 received at least one dose of the study drug and were included in the safety analysis; 8 did not receive the study drug. Of the 330 patients evaluated for tolerability, 96% had at least one postbaseline AISRS assessment and were thus evaluable for efficacy" (Journal article, pag.136)<br>Balanced drop out and reasons for drop out for lack of efficacy (n=1/73, 3/73, 4/74, 2/44 and 3/74 in the five study arms, respectively; Journal article, fig. 1)
**Selective reporting**	UNCLEAR	No protocol/CSR/CT available.
**Notes**		Corresponding author contacted but not able to provide additional information; not possible to contact drug company.

Bain 2013, NCT00429091

ITEM	RATING	SUPPORT
**Sequence generation**	UNCLEAR	No details on sequence generation.
**Allocation concealment**	UNCLEAR	No details on allocation concealment.

**Blinding participants/parents**	UNCLEAR	Double-blind but no details on who was blinded and how blinding was preserved.
**Blinding therapist**	UNCLEAR	Double-blind but no details on who was blinded and how blinding was preserved.
**Blinding assessor**	UNCLEAR	Double-blind but no details on who was blinded and how blinding was preserved.
**Incomplete data outcome**	HIGH	Completers-only analyzed for primary efficacy outcome (not ITT).<br>“A total of 238 patients were assessed for safety end points, 236 patients were included in the intent-to-treat data set, and 196 were included in the completers data set, which was the prespecified, primary data set for efficacy” (Abstract, pag. 405).
**Selective reporting**	LOW	Outcomes listed in CT were reported in the journal article
**Notes**		Corresponding author contacted but no reply.

Bangs 2007, B4Z-MC-LYAX

ITEM	RATING	SUPPORT
**Sequence generation**	LOW	Information from full CSR, pag. 156 (available upon request from manufacturer).
**Allocation concealment**	LOW	Information from full CSR, pag. 174 (available upon request from manufacturer).
**Blinding participants/parents**	LOW	Information from full CSR, pag. 4 and 155 (available upon request from manufacturer).
**Blinding therapist**	LOW	Information from full CSR, pag. 4 and 155 (available upon request from manufacturer).
**Blinding assessor**	LOW	Information from full CSR, pag. 4 and 155 (available upon request from manufacturer).
**Incomplete data outcome**	LOW	Information from full CSR, pag. 26 (available upon request from manufacturer).
**Selective reporting**	LOW	Outcomes pertinent to the present meta-analysis identified in the full CSR reported in the Journal article.
**Notes**		Manufacturer provided full CSR.

Bangs 2008, B4Z-MC-LYBX, NCT00191698

ITEM	RATING	SUPPORT
**Sequence generation**	LOW	Information from full CSR, pag. 1343 (available upon request from manufacturer).
**Allocation concealment**	LOW	Information from full CSR, pag. 1463 (available upon request from manufacturer).
**Blinding**	LOW	Information from full CSR, pag. 4 (available upon request from manufacturer).

participants/parents		
**Blinding therapist**	LOW	Information from full CSR, pag. 4 and 42 (available upon request from manufacturer).
**Blinding assessor**	LOW	Information from full CSR, pag. 4 and 42 (available upon request from manufacturer).
**Incomplete data outcome**	UNCLEAR	Withdrawal for protocol violation different across arms (5.1% in ATX and 0 in PBO arm, respectively) (Journal article, fig. 1); also, double rate of withdrawals in ATX arm (15%) vs. PCB (7%).
**Selective reporting**	LOW	Outcome measures listed in full CSR reported in journal article.
**Notes**		Manufacturer provided full CSR.

Bedard 2015, NCT0018339

ITEM	RATING	SUPPORT
**Sequence generation**	UNCLEAR	No description of sequence generation.
**Allocation concealment**	UNCLEAR	No details on allocation concealment.
**Blinding participants/parents**	LOW	“Matching placebo” (Journal article, pag. 42)
**Blinding therapist**	UNCLEAR	Not specified.
**Blinding assessor**	LOW	Blinded raters (Journal article, pag. 42)
**Incomplete data outcome**	LOW	8 drop outs per arm, with quite balanced reasons for withdrawal (Journal article, fig. 2)
**Selective reporting**	UNCLEAR	No protocol/CSR/CT.
**Notes**		Corresponding author not able to provide additional details.

Biederman 2002, SLI 381-301

ITEM	RATING	SUPPORT
**Sequence generation**	UNCLEAR	Additional information from full CSR, pag. 21 (pag. 29 of PDF), available upon request from the manufacturer, but method of generation of randomization not specified.
**Allocation concealment**	LOW	Additional information from full CSR, pag. 21-22 (pag. 29-30 of PDF), available upon request from the manufacturer.
**Blinding participants/parents**	LOW	Additional information from full CSR, pag. 21-22 (pag. 29-30 of PDF), available upon request from the manufacturer.
**Blinding therapist**	UNCLEAR	No information.
**Blinding assessor**	UNCLEAR	No information.

**Incomplete data outcome**	LOW	563/584 (96.4% of randomized participants) in ITT analysis (Journal article, Table 1)<br>Moderate to high discontinuation rate (23% in total sample); reasons for withdrawal for efficacy quite unbalanced across arms but low (< 5%) (Journal article, Table 1)
**Selective reporting**	UNCLEAR	All outcomes of relevance in full CSR reported in published journal article.
**Notes**		First author unable to provide additional details. Additional information provided by manufacturer from full CSR.

Biederman 2005, Study 311 Cephalon

ITEM	RATING	SUPPORT
**Sequence generation**	LOW	“The randomization code was generated by Cephalon, Inc (West Chester, PA) and implemented by a central agency (Phoenix Data Systems, Valley Forge, PA)” (Journal article, pag. e778).
**Allocation concealment**	LOW	“The randomization code was generated by Cephalon, Inc (West Chester, PA) and implemented by a central agency (Phoenix Data Systems, Valley Forge, PA)” (Journal article, pag. e778).
**Blinding participants/parents**	LOW	“film– coated tablets or matching placebo” (Journal article, pag. e778).
**Blinding therapist**	UNCLEAR	Not specified who else (other than participants) was blinded.
**Blinding assessor**	UNCLEAR	Not specified who else (other than participants) was blinded.
**Incomplete data outcome**	HIGH	Withdrawals due to lack of efficacy were double (n=37; 44%) in PBO arms compared to ATX (n=34; 21%) – this presents high RoB since withdrawal is unbalanced and related to efficacy outcome.
**Selective reporting**	UNCLEAR	No protocol/full CSR/CT available.
**Notes**		First author unable to provide additional details. Not possible to contact manufacturer.

Biederman 2006a (subsample of NCT00181571)

ITEM	RATING	SUPPORT
**Sequence generation**	UNCLEAR	No details.
**Allocation concealment**	UNCLEAR	No details.
**Blinding participants/parents**	LOW	“MPH and placebo were delivered in identical-appearing tablets” (Journal article, pag. 830)
**Blinding therapist**	UNCLEAR	Not specified
**Blinding assessor**	LOW	“Raters and subjects were blind to treatment assignment.”; “MPH and placebo were delivered in identical-

		appearing tablets" (Journal article, pag. 830)
**Incomplete data outcome**	LOW	Reasons for drop out quite balanced between arms for efficacy outcomes "Of 343 children screened, 248 were randomized.. Twenty-two (11%) randomized to modafinil discontinued the study for the following reasons: adverse event (N = 9), insufficient efficacy (N = 2), loss to follow-up (N = 4), noncompliance (N = 2), consent withdrawn (N = 2), protocol violation (N = 1), and other (N = 2). Three (6%) randomized to placebo discontinued: 2 because of insufficient efficacy and 1 because of noncompliance." (Journal article, pag. 730). LOCF
**Selective reporting**	UNCLEAR	No protocol/CSR/CT available.
**Notes**		First author not able to provide additional information.

Biederman 2006b

ITEM	RATING	SUPPORT
**Sequence generation**	UNCLEAR	No information.
**Allocation concealment**	UNCLEAR	No information.
**Blinding participants/parents**	LOW	Matching placebo (Abstract of Journal article)
**Blinding therapist**	UNCLEAR	No information.
**Blinding assessor**	UNCLEAR	No information.
**Incomplete data outcome**	LOW	Balanced drop out, including for lack of efficacy (Journal article, Table 1.) ITT: 196/198 for children receiving 300 mg/day.
**Selective reporting**	UNCLEAR	No protocol/CSR/CT available.
**Notes**		First author unable to provide additional information. Not possible to contact manufacturer.

Biederman 2007, NRP104-301, NCT00248092

ITEM	RATING	SUPPORT
**Sequence generation**	LOW	"Computer-generated randomization schedule." (Journal article, Pag. 452).
**Allocation concealment**	LOW	Information from full CSR, pag. 32-33 (available upon request from manufacturer).
**Blinding participants/parents**	LOW	"Both the investigator and the patient (and his/her parent/guardian) were blinded to treatment. To maintain blinding, all investigational products were supplied as white capsules identical in size, weight, and shape." (Journal article, Pag. 452).
**Blinding therapist**	LOW	"Both the investigator and the patient (and his/her parent/guardian) were blinded to treatment. To maintain blinding, all investigational products were supplied as white capsules identical in size, weight, and shape." (Journal article, Pag. 452).
**Blinding assessor**	LOW	"Both the investigator and the patient (and his/her parent/guardian) were blinded to treatment. To maintain blinding, all investigational products were supplied as white capsules identical in size, weight, and shape." (Journal article, Pag. 452).
**Incomplete data outcome**	UNCLEAR	98% of subjects included in ITT: "290 (201 boys, 89 girls; mean [SD] age, 9 [1.8] years) received the randomized and blinded treatment, 285 were included in the ITT population" (Journal article, pag. 454)<br>Quite unbalanced reasons for withdrawal across arms for outcomes of interest (efficacy). (LDX 30 mg/day:1/71; LDX 50 mg/day:0/74; LDX 70 mg/day 1/73; PBO: 12/72)<br>Method used for imputation not clear. LOCF may not be appropriate when withdrawals are unbalanced across arms
**Selective reporting**	LOW	All outcomes reported in published papers (Information provided by manufacturer).
**Notes**		First author unable to provide additional details. Manufacturer provided additional information from full CSR.

Biederman 2008, SPD503-301, NCT00152009

ITEM	RATING	SUPPORT
**Sequence generation**	LOW	Information provided by manufacturer, from protocol SPD503-301 (1.7.2002) section 5.5 Randomization and Code Breaks, pag. 23.
**Allocation concealment**	LOW	Information provided by manufacturer, from protocol 503-301 (07 March 2006) section 5.4.5.2 Reasons for breaking the blind, pag. 44).
**Blinding participants/parents**	LOW	"Matching GXR and placebo tablets were provided to patients in the form of weekly prepackaged individual study drug kits, identical in appearance, according to the randomization schedule." (Journal article, pag. e74).<br>"Information provided by manufacturer, from protocol SPD503-301 (1.7.2002) section 5.5 Randomization

		and Code Breaks, pag. 23).
**Blinding therapist**	LOW	"Matching GXR and placebo tablets were provided to patients in the form of weekly prepackaged individual study drug kits, identical in appearance, according to the randomization schedule." (Journal article, pag. e74).<br>Information provided by manufacturer, from protocol SPD503-301 (1.7.2002) section 5.5 Randomization and Code Breaks, pag. 23)
**Blinding assessor**	LOW	"Matching GXR and placebo tablets were provided to patients in the form of weekly prepackaged individual study drug kits, identical in appearance, according to the randomization schedule." (Journal article, pag. e74).<br>Information provided by manufacturer, from protocol SPD503-301 (1.7.2002) section 5.5 Randomization and Code Breaks, pag. 23)
**Incomplete data outcome**	UNCLEAR	ITT population (97.6%). High (~ 35%) and quite unbalanced number of drop out across arms. Balanced reasons for drop out for lack of efficacy (PBO: 15/86; GXR 2 mg/day: 8/87; GXR 3 mg/day: 6/86; GXR 4 mg/day: 7.86). (Journal article, fig. 1, pag. e76)
**Selective reporting**	LOW	Some outcomes were not published (information provided by manufacturer; however, these are not relevant for the present meta-analysis.
**Notes**		First author unable to provide additional details. Manufacturer provided additional information from protocol.

Biederman 2012, 2008P000971, NCT00801229

ITEM	RATING	SUPPORT
**Sequence generation**	UNCLEAR	No information.
**Allocation concealment**	UNCLEAR	No information.
**Blinding participants/parents**	UNCLEAR	"Physician raters and subjects were blind to treatment assignment" (Journal article, pag. 486) but no description of how blinding was preserved.
**Blinding therapist**	UNCLEAR	No details.
**Blinding assessor**	UNCLEAR	"Physician raters and subjects were blind to treatment assignment" (Journal article, pag. 486) was preserved.
**Incomplete data outcome**	LOW	Balanced drop outs (Journal article, figure 1).
**Selective reporting**	UNCLEAR	No protocol/CSR available. Outcomes in CT do not appear to cover all outcomes of the study.
**Notes**		First author unable to provide additional details. manufacturer not able to provide data since investigator initiated grant.

Biehl 2016

ITEM	RATING	SUPPORT
**Sequence generation**	LOW	“The random allocation sequences were generated by Medice Arzneimittel Pütter GmbH & Co. KG (Iserlohn, Germany), and a medical laboratory assistant/study nurse assigned participants to the intervention.. Randomization lists were generated using Rancode 3.6 (Isi Medien GmbH, München, Germany” (Journal article, pag. 3).
**Allocation concealment**	LOW	“The random allocation sequences were generated by Medice Arzneimittel Pütter GmbH & Co. KG (Iserlohn, Germany), and a medical laboratory assistant/study nurse assigned participants to the intervention” (Journal article, pag. 3).
**Blinding participants/parents**	LOW	Placebo and active medication identical in shape and aspect (Information provided by the first author). Parent and participants were blinded. (Information provided by the first author).
**Blinding therapist**	LOW	Placebo and active medication identical in shape and aspect (Information provided by the first author). Therapist were blinded. (Information provided by the first author).
**Blinding assessor**	LOW	Placebo and active medication identical in shape and aspect (Information provided by the first author). Assessors were blinded. (Information provided by the first author).
**Incomplete data outcome**	LOW	Only 1 drop out per arm (Journal article, figure 1).
**Selective reporting**	LOW	First author confirmed that all planned outcomes are reported in the Journal article.
**Notes**		First author provided additional information.

Block 2009, B4Z-US-LYCC, NCT00486122

ITEM	RATING	SUPPORT
**Sequence generation**	LOW	“A computer algorithm generated randomization numbers to blindly assign study drug to each of the 3 arms at the site level. This information was available to the investigative site via a telephone Interactive Voice Response System (IVRS), and the treatment assignments were not unblinded until after the database was locked.” (Journal article, Pag. 724).
**Allocation concealment**	LOW	“A computer algorithm generated randomization numbers to blindly assign study drug to each of the 3 arms at the site level. This information was available to the investigative site via a telephone Interactive Voice Response System (IVRS), and the treatment assignments were not unblinded until after the database was locked.” (Journal article, Pag. 724).
**Blinding participants/parents**	LOW	Information from full CSR, pag. 53 (available upon request from manufacturer).
**Blinding therapist**	LOW	Information from full CSR, pag. 53 (available upon request from manufacturer).

**Blinding assessor**	LOW	Information from full CSR, pag. 53 (available upon request from manufacturer).
**Incomplete data outcome**	LOW	Balanced number and reasons of drop outs (Journal article, figure 1). Information from full CSR, pag. 162 (available upon request from manufacturer).
**Selective reporting**	LOW	No protocol available. Primary and secondary outcomes listed in the full CSR reported in the Journal article, except for the Brown ADD scale (not an outcome of the present meta-analysis).
**Notes**		Manufacturer provided full CSR.

Bron 2014

ITEM	RATING	SUPPORT
**Sequence generation**	LOW	Sequence generated by a computer (Information provided by the first author)
**Allocation concealment**	LOW	"After the study was completed, the pharmacy revealed the allocation of participants to the randomization order." (information provided by the first author)
**Blinding participants/parents**	LOW	"An independent pharmaceutical company manufactured the visually identical over-encapsulated tablets containing either OROS-mph or placebo" (Journal article, pag. 520)
**Blinding therapist**	LOW	"Both patient and investigators were blinded" (information provided by the first author).
**Blinding assessor**	LOW	"Both patient and investigators were blinded" (information provided by the first author).
**Incomplete data outcome**	HIGH	Very small sample (<15 in each arm) with unbalanced drop-out due to AEs with no imputation (ITT not used).
**Selective reporting**	LOW	No protocol; first author confirmed that all planned outcomes were reported.
**Notes**		First author provided additional information.

Buitelaar 1996

ITEM	RATING	SUPPORT
**Sequence generation**	UNCLEAR	Not described.
**Allocation concealment**	UNCLEAR	Not described.
**Blinding participants/parents**	LOW	"Methylphenidate, pindolol, and placebo were administered at breakfast and at noon in identical-looking tablets manufactured by the local pharmacy" (Journal article, pag. 589)
**Blinding therapist**	UNCLEAR	No information.
**Blinding assessor**	UNCLEAR	No information.

**Incomplete data outcome**	LOW	Low attrition, ITT "There were two children with poor compliance under methylphenidate treatment; this became apparent after the blind code had been broken. Due to side-effects they were reluctant to take the tablets for several days. Since an intention to treat analysis was planned, data for these subjects was nonetheless included in the data analysis." (Journal article, pag. 590-1)
**Selective reporting**	UNCLEAR	No protocol/CSR/CT available.
**Notes**		First author unable to provide additional information.

Casas, 2013, EudraCT #: 2007-002111-82

ITEM	RATING	SUPPORT
**Sequence generation**	LOW	"Randomization was based on a computer-generated scheme prepared by the sponsor, balanced by using permuted blocks of treatments and stratified by study centre. Based on this scheme, study drug was packaged for each subject." (Journal article, pag. 269)
**Allocation concealment**	LOW	"Medication kit numbers were pre-printed on drug labels and assigned as subjects were randomly assigned to treatment. Treatment codes were obtained from a central interactive voice response system giving a medication kit number for the drug to which the subject had been assigned." (Journal article, pag. 269)
**Blinding participants/parents**	LOW	Information from full CSR (available upon request from manufacturer).
**Blinding therapist**	LOW	Information from full CSR (available upon request from manufacturer).
**Blinding assessor**	LOW	Information from full CSR (available upon request from manufacturer).
**Incomplete data outcome**	UNCLEAR	Unclear how may participants provided follow-up data at each time point.
**Selective reporting**	LOW	No protocol available. Outcomes listed in the CSR synopsis reported in the journal article.
**Notes**		Authors contacted but no reply. Additional information provided by manufacturer.

Casat, 1989

ITEM	RATING	SUPPORT
**Sequence generation**	UNCLEAR	Not described.
**Allocation concealment**	UNCLEAR	Not described.
**Blinding participants/parents**	UNCLEAR	Not reported how blinding was preserved.

**Blinding therapist**	UNCLEAR	Not reported how blinding was preserved.
**Blinding assessor**	UNCLEAR	Not reported how blinding was preserved.
**Incomplete data outcome**	LOW	Low attrition, balanced (1 drop out per arm).
**Selective reporting**	UNCLEAR	No protocol/CSR/CT available.
**Notes**		Not possible to contact authors.

Childress, 2009 CRIT124E2305, NCT00301236

ITEM	RATING	SUPPORT
**Sequence generation**	LOW	"Randomization was performed using a validated system that automated the assignment of treatment" (Journal article, pag. 353)
**Allocation concealment**	LOW	"Randomization was performed using a validated system that automated the assignment of treatment" (Journal article, pag. 353)
**Blinding participants/parents**	LOW	"Matching placebo" (Journal article, pag. 353)
**Blinding therapist**	UNCLEAR	Not reported
**Blinding assessor**	UNCLEAR	Not reported.
**Incomplete data outcome**	LOW	"Among 253 randomized patients, the ITT population consisted of 240 patients (94.9%) and the safety population consisted of 245 patients (96.8%)" (Journal article, pag. 354)<br>Balanced drop out across study arms (Journal article, figure 2).
**Selective reporting**	UNCLEAR	No protocol/CSR/CT available.
**Notes**		Author contacted but not reply.

Coghill 2013, SPD489-325

ITEM	RATING	SUPPORT
**Sequence generation**	LOW	Computer generated. (Information provided by first author).
**Allocation concealment**	LOW	"An interactive voice/web response system was used to allocate a unique randomization number to each patient." (Journal article, pag. 1210).
**Blinding participants/parents**	LOW	Study drugs were over-encapsulated and appeared identical. (Journal article, pag. 1210).
**Blinding therapist**	LOW	"Study drugs were over-encapsulated and appeared identical" (Journal article, pag. 1210). First author confirmed therapist was blinded.

**Blinding assessor**	LOW	"Study drugs were over-encapsulated and appeared identical" (Journal article, pag. 1210). First author confirmed therapist was blinded.
**Incomplete data outcome**	HIGH	Unbalanced drop out for lack of efficacy. ITT analyses may not be adequate when there are unbalanced reasons for drop out for lack of efficacy.
**Selective reporting**	LOW	First author confirmed that all measures listed in the protocol are reported in the journal article.
**Notes**		First author provided additional information.

Connor 2000

ITEM	RATING	SUPPORT
**Sequence generation**	HIGH	3 subjects refused to be randomized to MPH alone due to experience with lack of efficacy - these were randomized to clonidine or the combination arm (Journal article, pag. 17)
**Allocation concealment**	UNCLEAR	No information.
**Blinding participants/parents**	LOW	"All medication capsules and placebo capsules were prepared by the UMMS (University of Massachusetts Medical School) Pharmacy in identical capsules to disguise taste and smell." (Journal article, pag. 17).
**Blinding therapist**	LOW	"Teachers, school nurses, parents, children and research assistants completing dependent measures were blinded to the child's treatment group for the study duration" (Journal article, pag. 17).<br>"All medication capsules and placebo capsules were prepared by the UMMS (University of Massachusetts Medical School) Pharmacy in identical capsules to disguise taste and smell." (Journal article, pag. 17).
**Blinding assessor**	LOW	"Teachers, school nurses, parents, children and research assistants completing dependent measures were blinded to the child's treatment group for the study duration" (Journal article, pag. 17).<br>"All medication capsules and placebo capsules were prepared by the UMMS (University of Massachusetts Medical School) Pharmacy in identical capsules to disguise taste and smell." (Journal article, pag. 17).
**Incomplete data outcome**	UNCLEAR	LOCF but not specified if there were drop outs and reasons for drop out in each arm, so not possible to assess to which extent drop outs were balanced.
**Selective reporting**	UNCLEAR	No study protocol/CSR/CT available; Cochrane review (Storebo et al., 2014): "Reply from study author on our request for the protocol: protocol described in the study".
**Notes**		First author informed that additional data are not available anymore.

Connor 2010, SPD503-307, NCT00367835

ITEM	RATING	SUPPORT
**Sequence generation**	LOW	"The randomization schedule was prepared by a third party using a computerized random-number generator and implemented by an automated telephone system." (Journal article, Pag. 757).

**Allocation concealment**	LOW	The randomization schedule was prepared by a third party using a computerized random-number generator and implemented by an automated telephone system. (Journal article, Pag. 757).
**Blinding participants/parents**	LOW	Subjects and study personnel were blinded to the treatment (Journal article, pag. 757).<br>Information from full CSR, 4.6.1, Description of blinding, pag. 35 (available upon request from manufacturer)
**Blinding therapist**	LOW	Subjects and study personnel were blinded to the treatment (Journal article, pag. 757).<br>Information provided by manufacturer, from full CSR, 4.6.1 Description of blinding, pag. 35 (available upon request from manufacturer)
**Blinding assessor**	LOW	Subjects and study personnel were blinded to the treatment<br>Information provided by manufacturer, from full CSR, 4.6.1, pag. 35 (available upon request from manufacturer)
**Incomplete data outcome**	LOW	Low attrition. "A total of 217 subjects were enrolled; 138 were randomized to receive guanfacine XR and 79 to receive placebo (figure 1). The safety population and full analysis set included 214 subjects (three subjects did not receive a dose of medication)." (Journal article, pag. 758)
**Selective reporting**	LOW	Information provided by the manufacturer: some outcomes were not reported in the journal article (however, available from Clinicaltrials.gov.)
**Notes**		Manufacturer provided additional information.

Cook 1993

ITEM	RATING	SUPPORT
**Sequence generation**	LOW	"Table of random numbers known only to the pharmacist." (Journal article, pag. 132).
**Allocation concealment**	LOW	"All patients who met the inclusion criteria and who had given their consent to participate in the study were sent to a separate building, where they were assigned to groups by a table of random numbers known only to the pharmacist." (Journal article, pag. 132).
**Blinding participants/parents**	UNCLEAR	"The physician, audiologist, teachers and parents involved in behavior rating and the subjects themselves were blind to the treatment assignment." (Journal article, pag. 132) but not clear how blinding was preserved.
**Blinding therapist**	UNCLEAR	"The physician, audiologist, teachers and parents involved in behavior rating and the subjects themselves were blind to the treatment assignment." (Journal article, pag. 132) but not clear how blinding was preserved
**Blinding assessor**	UNCLEAR	"The physician, audiologist, teachers and parents involved in behavior rating and the subjects themselves were blind to the treatment assignment." (Journal article, pag. 132) but not clear how blinding was preserved
**Incomplete data outcome**	UNCLEAR	Drop out not reported.

**Selective reporting**	UNCLEAR	Protocol/CSR/CT not available.
**Notes**		No possible to contact authors.

CRIT124DUS02

ITEM	RATING	SUPPORT
**Sequence generation**	UNCLEAR	Not specified
**Allocation concealment**	UNCLEAR	Not specified
**Blinding participants/parents**	LOW	PBO and active drug identical (summary of CSR)
**Blinding therapist**	UNCLEAR	Not specified
**Blinding assessor**	UNCLEAR	Not specified
**Incomplete data outcome**	UNCLEAR	Drop out for each arm not specified
**Selective reporting**	UNCLEAR	Only document publically available: summary of study report. So not possible to assess if all planned outcomes were reported.
**Notes**		No additional information from drug company.

Dell' Agnello 2009

ITEM	RATING	SUPPORT
**Sequence generation**	UNCLEAR	No information.
**Allocation concealment**	UNCLEAR	No information.
**Blinding participants/parents**	UNCLEAR	Not clear how blinding was preserved.
**Blinding therapist**	UNCLEAR	Not clear how blinding was preserved.
**Blinding assessor**	UNCLEAR	Not clear how blinding was preserved.
**Incomplete data outcome**	LOW	Low drop-out rate (Journal article, figure 2); LOCF.
**Selective reporting**	UNCLEAR	No protocol/CSR/CT available.
**Notes**		Authors and masnufacturer not able to provide additional information.

Dittmann 2011

ITEM	RATING	SUPPORT
**Sequence generation**	LOW	“Randomization was based on a computer-generated random sequence using an interactive voice response system” (Journal article, pag. 100).
**Allocation concealment**	LOW	“Randomization was based on a computer-generated random sequence using an inter- active voice response system” (Journal article, pag. 100).
**Blinding participants/parents**	LOW	Information from full CSR, pag. 21 and 27 (available upon request from manufacturer).
**Blinding therapist**	LOW	Information from full CSR, pag. 21 and 27 (available upon request from manufacturer).
**Blinding assessor**	LOW	Information from full CSR, pag. 21 and 27 (available upon request from manufacturer).
**Incomplete data outcome**	UNCLEAR	LOCF but quite unbalanced drop out for lack of efficacy (Journal article, fig. 1): ATX fast: 7/60; ATX slow: 4/61; PBO: 17/59
**Selective reporting**	LOW	Primary and secondary outcomes listed in full CSR all reported in the journal article.
**Notes**		Manufacturer provided full CSR.

Dopfner 2003

ITEM	RATING	SUPPORT
**Sequence generation**	LOW	Central randomization. (Information provided the authors).
**Allocation concealment**	LOW	Central randomization. (Information provided the authors).
**Blinding participants/parents**	LOW	First author confirmed that active medication and placebo were identical.
**Blinding therapist**	LOW	First author confirmed that active medication and placebo were identical and that study personnel was blinded.
**Blinding assessor**	LOW	First author confirmed that active medication and placebo were identical and that study personnel was blinded.
**Incomplete data outcome**	LOW	3 dropouts in the active medication arm and 3 in the PBO arm
**Selective reporting**	LOW	First author confirmed that all planned outcomes are reported in the Journal article.
**Notes**		First author provided additional information.

Durell 2013, B4Z-US-LYDZ, NCT00510276

ITEM	RATING	SUPPORT
**Sequence generation**	LOW	"Assignment to treatment groups was determined by a computer-generated random sequence using an interactive voice response system, which assigned packages of double- blind study drug to each participant". (Journal article, pag. 46).
**Allocation concealment**	LOW	"Assignment to treatment groups was determined by a computer-generated random sequence using an interactive voice response system, which assigned packages of double- blind study drug to each participant". (Journal article, pag. 46).
**Blinding participants/parents**	LOW	Information from full CSR, pag. 13, 18, and 20 (available upon request from manufacturer).
**Blinding therapist**	LOW	Information from full CSR, pag. 18 and 20 (available upon request from manufacturer).
**Blinding assessor**	LOW	Information from full CSR, pag. 18 and 20 (available upon request from manufacturer).
**Incomplete data outcome**	HIGH	Overall, 115 (52.3%) of the 220 participants randomized to atomoxetine and 130 (57.8%) of the 225 participants randomized to placebo completed the study, and the difference in completion rate between treatment groups was not statistically significant (P = 0.25). The most common reasons for early discontinuation were lost to follow-up (n = 97), participant decision (n = 54), and adverse events (n = 27). Adverse event as reason for discontinuation was statistically significantly more common in the atomoxetine group (n = 21, 9.5%) compared with the placebo group (n = 6, 2.7%; P = 0.003). Other reasons for discontinuation were not statistically significant.<br>LOCF on 192/220 patients assigned to ATX and 199/225 patients assigned to PBO. LOCF analysis is significant departure from protocol ITT analysis plan which includes all randomised participants for the primary efficacy outcome. (Journal article, pag. 48).<br>Note: 5 patients completed the study in violation of the study protocol.
**Selective reporting**	LOW	Relevant outcomes for the present meta-analysis listed in the full CSR are reported in the Journal article
**Notes**		Manufacturer provided full CSR.

Efron 1997

ITEM	RATING	SUPPORT
**Sequence generation**	LOW	Table of random numbers. (Information provided by the first author).
**Allocation concealment**	LOW	Research assistant kept blinding. (Information provided by the first author).
**Blinding participants/parents**	LOW	"Both drugs were presented in identical form, as a crushed powder in opaque gelatin capsules. The investigators, families, subjects, and teachers were blind to the randomization order throughout the study period." (Journal article, pag. 663).

**Blinding therapist**	LOW	"Both drugs were presented in identical form, as a crushed powder in opaque gelatin capsules. The investigators, families, subjects, and teachers were blind to the randomization order throughout the study period." (Journal article, pag. 663).
**Blinding assessor**	LOW	"Both drugs were presented in identical form, as a crushed powder in opaque gelatin capsules. The investigators, families, subjects, and teachers were blind to the randomization order throughout the study period." (Journal article, pag. 663).
**Incomplete data outcome**	LOW	Balanced drop out:<br>1 was a non starter – allocation D – M<br>1 dropped out after poor response to Dex at phase 1 (headaches, irritability, rage) allocation D - M<br>2 dropped out at baseline, no info – allocation D – M<br>3 dropped out at baseline, no info – allocation M - D<br>(Information provided by the first author)
**Selective reporting**	LOW	No protocol available. Primary and secondary outcome measures all reported. (Information provided by the study author).
**Notes**		First author provided additional information.

Findling 2008, NCT00444574

ITEM	RATING	SUPPORT
**Sequence generation**	LOW	"Computer-generated random-numbers" (Journal article, pag. 151)
**Allocation concealment**	UNCLEAR	Not specified.
**Blinding participants/parents**	LOW	"The OROS methylphenidate/placebo tablets were encapsulated to blind the identity of the capsule's content (Journal article, pag. 151)
**Blinding therapist**	UNCLEAR	Unclear who was blinded, other than the participants.
**Blinding assessor**	UNCLEAR	Unclear who was blinded, other than the participants.
**Incomplete data outcome**	UNCLEAR	Withdrawn "continued in long-term (Fig 2): not clear; contacted author to clarify but no reply from author
**Selective reporting**	UNCLEAR	No protocol/CSR/CT available.
**Notes**		Author contacted but no reply. No additional information from manufacturer.

Findling 2011, SPD 489-305, NCT00735371

ITEM	RATING	SUPPORT
**Sequence**	LOW	"An Interactive Voice Response System/Interactive Web Response System Screening was used for

generation		randomization/treatment assignments". (Journal article, pag. 396)
Allocation concealment	LOW	"An Interactive Voice Response System/Interactive Web Response System Screening was used for randomization/treatment assignments". (Journal article, pag. 396)
Blinding participants/parents	LOW	Information from full CSR, pag. 22 (available upon request from manufacturer)
Blinding therapist	LOW	Information from full CSR (pag. 22), available from manufacturer upon request; "All doses appeared identical to maintain the integrity of the blind". (Journal article, pag. 396).
Blinding assessor	LOW	Information from full CSR (pag. 22), available from manufacturer upon request; "All doses appeared identical to maintain the integrity of the blind". (Journal article, pag. 396).
Incomplete data outcome	UNCLEAR	Quite unbalanced drop out for lack of efficacy (6/235 in LDX arm and 4/79 in PBO arm) but relatively small drop-out (Journal article, figure 1). LOCF
Selective reporting	LOW	Only outcomes planned in the protocol and not reported in the Journal article were height and BMI (information provided by Shire), not relevant for the present meta-analysis.
Notes		Manufacturer provided additional data.

Frick 2017, SPD465-303, NCT00152022

ITEM	RATING	SUPPORT
Sequence generation	LOW	Information from protocol, pag. 25-27
Allocation concealment	LOW	Information from protocol, pag. 25-27 (available upon request from manufacturer)
Blinding participants/parents	UNCLEAR	"The blind was inadvertently broken for four participants during the study (three participants had urine drug screens required by their employers or housing development; one participant had a urine sample erroneously processed as a baseline urine drug screen sample)." (Journal article, pag. 3) with only 4 blinds broken out of 400 randomised – impact if likely to be very small<br>Not specified how blinding was achieved.
Blinding therapist	UNCLEAR	Not specified.
Blinding assessor	UNCLEAR	Not specified.
Incomplete data outcome	LOW	Randomized: n=411; ITT: n= 405; Balanced drop-out (Journal article, figure 1)
Selective reporting	LOW	Shire confirmed that the only outcome not reported in the Journal article is the AIM-A (not an outcome for the present meta-analysis)
Notes		Manufacturer provided additional information.

Gau 2007, B4Z-TW-S010, NCT00485459

ITEM	RATING	SUPPORT
**Sequence generation**	LOW	"Assignment to treatment groups was determined by a computer-generated random sequence using an interactive voice response system." (Journal article, pag. 449).
**Allocation concealment**	LOW	"Assignment to treatment groups was determined by a computer-generated random sequence using an interactive voice response system." (Journal article, pag. 449).
**Blinding participants/parents**	LOW	Information from protocol, pag. 19 (available upon request from manufacturer)
**Blinding therapist**	LOW	Information from protocol, pag. 19 and 23 (available upon request from manufacturer)
**Blinding assessor**	LOW	Information from protocol, pag. 19 and 23 (available upon request from manufacturer)
**Incomplete data outcome**	UNCLEAR	Randomized: ATX arm: n=72; PBO arm: n=34; ITT: ATX: n=69; PBO: n= 29) (Journal article, Table 2). Among 106 patients randomly assigned to a treatment group, 98 (92.5%) completed the study. Three (4.2%) and five (14.7%) subjects in the atomoxetine and placebo groups, respectively, withdrew from this study. (Fisher's exact test, p " 0.108). In the atomoxetine group, 1 patient discontinued the study due to adverse events (decreased appetite, nausea, dizziness, and abdominal pain), one for lack of efficacy, and one due to the patient's personal conflict. In the placebo group, all 5 patients who had been withdrawn from the study were because of lack of efficacy. LOCF. But bias may occur despite LOCF when there is an excess of early withdrawals due to lack of efficacy in placebo arm.
**Selective reporting**	LOW	Outcomes planned in protocol (available upon request from manufacturer) reported in the Journal article.
**Notes**		Author contacted but no reply. Manufacturer provided full protocol.

Geller 2007, B4Z-US-LYBP

ITEM	RATING	SUPPORT
**Sequence generation**	LOW	Information from full CSR, pag. 43 (available upon request from manufacturer)
**Allocation concealment**	LOW	Information from full CSR, pag. 43 (available upon request from manufacturer)
**Blinding participants/parents**	LOW	Information from full CSR, pag. 43 (available upon request from manufacturer)
**Blinding therapist**	LOW	Information from full CSR, pag. 43 (available upon request from manufacturer)
**Blinding assessor**	LOW	Information from full CSR, pag. 43 (available upon request from manufacturer)
**Incomplete data outcome**	LOW	Balanced drop out (Journal article, figure 1). LOCF. Additional LOCF analysis with all randomized participants
**Selective reporting**	LOW	Some secondary outcomes in full CSR, pag. 3 (available upon request from manufacturer) not reported in

		the journal article, but these outcomes are not relevant for the present meta-analysis.
**Notes**		Manufacturer provided full CSR.

Ginsberg 2012, EUCTR2006-002553-80-SE

ITEM	RATING	SUPPORT
**Sequence generation**	LOW	"..random number table.." (Journal article, pag. 69).
**Allocation concealment**	LOW	"The random number table was stored in the pharmacy department and was concealed from study staff and participants until completion of the study." (Journal article, pag. 69).
**Blinding participants/parents**	LOW	"The placebo and methylphenidate capsules and packaging were identical in appearance and were coded with a unique randomisation number." (Journal article, pag. 69).<br>"Both study staff and participants were masked to assignment during the RCT". (Journal article, pag. 69).
**Blinding therapist**	LOW	"The placebo and methylphenidate capsules and packaging were identical in appearance and were coded with a unique randomisation number." (Journal article, pag. 69).<br>"Both study staff and participants were masked to assignment during the RCT". (Journal article, pag. 69).
**Blinding assessor**	LOW	"The placebo and methylphenidate capsules and packaging were identical in appearance and were coded with a unique randomisation number." (Journal article, pag. 69).<br>"Both study staff and participants were masked to assignment during the RCT". (Journal article, pag. 69).
**Incomplete data outcome**	LOW	LOCF including all randomized patients (Journal article, pag. 70). No drop outs (Journal article, Figure 1).
**Selective reporting**	LOW	All outcomes have been published in Journal articles (Information provided by the first author).
**Notes**		First author provided additional information

Goodman 2016, NCT00937040

ITEM	RATING	SUPPORT
**Sequence generation**	LOW	"Subjects were randomly assigned by an interactive voice response system". (Pag. e2).
**Allocation concealment**	LOW	"Subjects were randomly assigned by an interactive voice response system". (Pag. e2).
**Blinding participants/parents**	LOW	Information form full CSR (available upon request from manufacturer)
**Blinding therapist**	LOW	Information form full CSR (available upon request from manufacturer)
**Blinding assessor**	LOW	Information form full CSR (available upon request from manufacturer)

**Incomplete data outcome**	LOW	Balanced drop out. ITT on 95% of subjects randomized (Journal article, figure 1).
**Selective reporting**	LOW	All of the secondary measures listed in the protocol are mentioned in the paper but not all results are listed. For those measures where results were not discussed specifically in the paper, they were reported on the clinicaltrials.gov website at the following hyperlink: https://clinicaltrials.gov/ct2/show/results/NCT00937040 (Information provided by manufacturer).
**Notes**		manufacturer provided additional information.

Goto 2017, B4Z-JE-LYEE, NCT00962104

ITEM	RATING	SUPPORT
**Sequence generation**	LOW	Information from full CSR, pag. 42 (available upon request from manufacturer)
**Allocation concealment**	LOW	Information from full CSR, pag. 42 (available upon request from manufacturer)
**Blinding participants/parents**	LOW	Information from full CSR, pag. 44 (available upon request from manufacturer)
**Blinding therapist**	LOW	Information from full CSR, pag. 44 (available upon request from manufacturer)
**Blinding assessor**	LOW	Information from full CSR, pag. 44 (available upon request from manufacturer)
**Incomplete data outcome**	LOW	Balanced drop outs. “Although significantly more patients randomized to placebo completed the study (171/196: 87.2%, *p* = .042) compared with those assigned to atomoxetine (155/195: 79.5%), none of the reasons for discontinuation were reported with significantly greater frequency in either treatment group” (Journal article, pag. 3). MMRM analysis.
**Selective reporting**	LOW	CGI not reported in the journal article but available from Clinicaltrials.gov
**Notes**		Manufacturer provided full CSR.

Greenhill 2002

ITEM	RATING	SUPPORT
**Sequence generation**	UNCLEAR	Not clear how sequence was generated.
**Allocation concealment**	UNCLEAR	Not clear how concealment was preserved.
**Blinding**	LOW	“Identically appearing MPH MR and placebo capsules “ (Journal article, pag. 2).

**participants/parents**		
**Blinding therapist**	UNCLEAR	No information.
**Blinding assessor**	UNCLEAR	No information.
**Incomplete data outcome**	LOW	N=321 randomized; n=314 included in ITT efficacy population. "Twenty-eight children (17%) who received placebo withdrew from the 3-week trial, whereas only 17 children (11%) who were assigned to MPH MR withdrew. Reasons for withdrawal were similar for both treatment groups." (Journal article, pag. 3)
**Selective reporting**	UNCLEAR	No protocol/CSR/CT available.
**Notes**		First author contacted, no reply. Not possible to gather any further information from manufacturer

Greenhill 2006a, Study 309 Cephalon

ITEM	RATING	SUPPORT
**Sequence generation**	UNCLEAR	Not clear how sequence was generated
**Allocation concealment**	LOW	Central randomization (Journal article, pag. 505).
**Blinding participants/parents**	LOW	Matching placebo (Journal article, pag. 505).
**Blinding therapist**	UNCLEAR	Not detailed.
**Blinding assessor**	UNCLEAR	Not detailed.
**Incomplete data outcome**	HIGH	ITT on the majority of randomized patients (randomized: modafinil 133, placebo 67; safety analysis: modafinil 131, placebo 67; efficacy analysis: modafinil 128, placebo 66). Discontinued for lack of efficacy: n=15/133 in active drug arm. N=19/67 in PBO arm (unbalanced dropout)
**Selective reporting**	UNCLEAR	No protocol/CSR/CT available.
**Notes**		Author contacted but no reply; not possible to contact manufacturer

Greenhill 2006b, CRIT124E2301

ITEM	RATING	SUPPORT
**Sequence generation**	UNCLEAR	Not reported
**Allocation concealment**	UNCLEAR	Not reported
**Blinding**	UNCLEAR	Double blind, but not described how blinding was assured

participants/parents		
Blinding therapist	UNCLEAR	Double blind, but not described how blinding was assured
Blinding assessor	UNCLEAR	Double blind, but not described how blinding was assured
Incomplete data outcome	LOW	N=103 randomized; ITT for efficacy (primary outcome: n= 97. Balanced drop-outs: “Of the 53 patients randomized to d-MPH-ER, 48 completed the study. The remaining patients discontinued because of unsatisfactory therapeutic effect (n = 2), being lost to follow-up (n = 2), and administrative problems (n = 1). Of the 50 patients randomized to placebo, 37
Selective reporting	UNCLEAR	No protocol/CSR/CT available.
Notes		Author contacted but no reply. Manufacturer unable to provide requested information.

Grizenko 2012

ITEM	RATING	SUPPORT
Sequence generation	LOW	Computer generated. (Information provided by the authors).
Allocation concealment	LOW	Sealed envelopes [Journal article (Gruber et al., 2007), pag. 155)
Blinding participants/parents	LOW	Active drug and placebo identical [Journal article (Grizenko et al., 2013), pag. 155]
Blinding therapist	LOW	Active drug and placebo identical; therapists were blinded. (Information provided by the authors)
Blinding assessor	LOW	Active drug and placebo identical; assessors were blinded. (Information provided by the authors)
Incomplete data outcome	LOW	No drop out
Selective reporting	LOW	Planned outcomes reported in the journal articles. (Information provided by the authors)
Notes		Authors provided additional information.

Harfterkamp 2012, NCT00380692

ITEM	RATING	SUPPORT
Sequence generation	LOW	“Independent pharmacists dispensed either placebo or atomoxetine capsules according to a computer-generated randomization list” (Journal article, pag. 735)
Allocation concealment	LOW	“All study personnel and participants were blinded to treatment assignment for the duration of the study. The code was not revealed to the researchers until data collection for the study had been fully completed. The study statisticians were also blinded until completion of the analyses. Thus, maximum allocation concealment has been achieved”. (Journal article, pag. 735-6).

**Blinding participants/parents**	LOW	"Atomoxetine and placebo were available as capsules and were identical in appearance. To preserve the blinding, all doses were given in two capsules, which had to be taken together in the morning" (Pag. 735) "All study personnel and participants were blinded to treatment assignment for the duration of the study. The code was not revealed to the researchers until data collection for the study had been fully completed. The study statisticians were also blinded until completion of the analyses. Thus, maximum allocation concealment has been achieved". (Journal article, pag. 735-6).
**Blinding therapist**	LOW	"Atomoxetine and placebo were available as capsules and were identical in appearance. To preserve the blinding, all doses were given in two capsules, which had to be taken together in the morning" (Pag. 735) "All study personnel and participants were blinded to treatment assignment for the duration of the study. The code was not revealed to the researchers until data collection for the study had been fully completed. The study statisticians were also blinded until completion of the analyses. Thus, maximum allocation concealment has been achieved". (Journal article, pag. 735-6).
**Blinding assessor**	LOW	Atomoxetine and placebo were available as capsules and were identical in appearance. To preserve the blinding, all doses were given in two capsules, which had to be taken together in the morning" (Pag. 735) "All study personnel and participants were blinded to treatment assignment for the duration of the study. The code was not revealed to the researchers until data collection for the study had been fully completed. The study statisticians were also blinded until completion of the analyses. Thus, maximum allocation concealment has been achieved". (Journal article, pag. 735-6).
**Incomplete data outcome**	LOW	Balanced drop out (Journal article. Figure 1); LOCF
**Selective reporting**	LOW	No protocol/CSR available. Outcomes of interest for the present meta-analysis reported in Clinicaltrials.gov were all reported in the Journal article.
**Notes**		

<u>Herring 2012, NCT00475735</u>

ITEM	RATING	SUPPORT
**Sequence generation**	LOW	"Randomization (stratified by site) was achieved using a computer-generated allocation schedule prepared by a blinded statistician at Merck." (Journal article, pag. e892).
**Allocation concealment**	LOW	"Randomization (stratified by site) was achieved using a computer-generated allocation schedule prepared by a blinded statistician at Merck. Blinded drug supplies were provided in numbered containers." (Journal article, pag. e892).
**Blinding participants/parents**	LOW	"All study personnel, including investigators, study site personnel, patients, and Merck staff, remained blinded to treatment allocation throughout the study." (Journal article, pag. e892).
**Blinding therapist**	LOW	"All study personnel, including investigators, study site personnel, patients, and Merck staff, remained blinded to treatment allocation throughout the study." (Journal article, pag. e892).
**Blinding assessor**	LOW	"All study personnel, including investigators, study site personnel, patients, and Merck staff, remained

		blinded to treatment allocation throughout the study." (Journal article, pag. e892).
**Incomplete data outcome**	LOW	"All study personnel, including investigators, study site personnel, patients, and Merck staff, remained blinded to treatment allocation throughout the study." (Journal article, pag. e892).
**Selective reporting**	LOW	No protocol available. Outcomes listed in Clinicaltrials.gov and of interest for the present meta-analysis reported in the Journal article.
**Notes**		

Hervas 2014, SPD503-316, NCT01244490, EudraCT: 2010- 018579-12

ITEM	RATING	SUPPORT
**Sequence generation**	LOW	"Randomization occurred at baseline (day 0) and eligible participants were randomized, using a 1:1:1 ratio, to GXR, ATX or placebo (automatically, randomly assigned by the interactive voice response system)." (Journal article, pag. 1863)
**Allocation concealment**	LOW	"Randomization occurred at baseline (day 0) and eligible participants were randomized, using a 1:1:1 ratio, to GXR, ATX or placebo (automatically, randomly assigned by the interactive voice response system)." (Journal article, pag. 1863)
**Blinding participants/parents**	LOW	"Matching placebo tablet" (Journal article, pag. 1863) Information form full CSR, pag. 15 (available upon request from manufacturer)
**Blinding therapist**	UNCLEAR	Not specified.
**Blinding assessor**	UNCLEAR	Not specified.
**Incomplete data outcome**	UNCLEAR	One patient was randomized to GXR but did not receive any treatment and was excluded from the FAS and the safety population. Quite unbalanced drop outs for lack of efficacy (GXR: n=5/115; ATX: n=5/112; PBO: n=14/111). (Journal article, fig. 2).
**Selective reporting**	LOW	No protocol available; Manufcaturer confirmed that all the outcomes of interest for the present meta-analysis are reported in the Journal article.
**Notes**		Manufacturer provided additional information.

Huss 2014, CRIT124D2302, EUCTR2010-021533-31-DE, NCT01259492

ITEM	RATING	SUPPORT
**Sequence generation**	LOW	"An unbiased, confidential patient randomization list was produced by the IVRS/ IWRS provider using a validated system.." (Journal article, pag. 48).
**Allocation concealment**	LOW	"A unique, confidential randomization number was assigned to each patient and IVRS/ IWRS allocated medication accordingly, as assigned, throughout the respective treatment periods." (Journal article, pag. 48).
**Blinding**	LOW	"All sites and personnel for clinical, medical, statistical, data management and monitoring were blinded,

**participants/parents**		and randomization data were kept strictly confidential until the time of unblinding after the conclusion of the study. The identity of the treatments has been concealed by the use of study drugs that are all identical in packaging, labeling, schedule of administration, appearance, taste, and odor, in line with Consort guidelines." (Journal article, pag. 48).
**Blinding therapist**	LOW	"All sites and personnel for clinical, medical, statistical, data management and monitoring were blinded, and randomization data were kept strictly confidential until the time of unblinding after the conclusion of the study. The identity of the treatments has been concealed by the use of study drugs that are all identical in packaging, labeling, schedule of administration, appearance, taste, and odor, in line with Consort guidelines." (Journal article, Pag. 48)
**Blinding assessor**	LOW	"All sites and personnel for clinical, medical, statistical, data management and monitoring were blinded, and randomization data were kept strictly confidential until the time of unblinding after the conclusion of the study. The identity of the treatments has been concealed by the use of study drugs that are all identical in packaging, labeling, schedule of administration, appearance, taste, and odor, in line with Consort guidelines." (Journal article, Pag. 48)
**Incomplete data outcome**	LOW	FAS on the large majority of randomized patients (> 96%). Drop put for lack of efficacy < 10% in each arm (MPH-LA 40 mg/day: n=2/181; MPH-LA 60 mg/day: n=2/182; MPH-LA 80 mg/day: n=4/181; PBO: 11/181)
**Selective reporting**	LOW	No protocol available. Outcomes listed in Clinicaltrials.gov of interest for the present meta-analysis all reported in the Journal article.
**Notes**		

Jafarinia 2012

ITEM	RATING	SUPPORT
**Sequence generation**	LOW	"..computerized random number generator". (Journal article, pag. 413)
**Allocation concealment**	LOW	"Allocation was concealed from the rater and the participants with the use of sequentially numbered, opaque, and sealed envelopes. Random allocation and clinical rating of the patients was carried out by different individuals. The patient and his or her parents, the clinician referring physician, the physician who prescribed the medication and rated the patients, and the statistician were blind to allocation." (Journal article, pag. 413)
**Blinding participants/parents**	UNCLEAR	Not clear how blinding was preserved
**Blinding therapist**	UNCLEAR	Not clear how blinding was preserved
**Blinding assessor**	UNCLEAR	Not clear how blinding was preserved
**Incomplete data outcome**	LOW	Balanced drop out (Journal article, fig. 1) only 1/22 participants discontinued per arm.

**Selective reporting**	UNCLEAR	No protocol/CSR/CT available.
**Notes**		Authors contacted but no reply.

Jain 2011, NCT00556959

ITEM	RATING	SUPPORT
**Sequence generation**	LOW	Randomization via a computer-generated sequence (Information provided by first author).
**Allocation concealment**	UNCLEAR	“if I recall correctly, we had sent each site a randomization allotment of envelopes with computer-generated randomization schedule - the sites then used this list to assign subjects in sequential order - thereby assuring true, concealed, randomization” (Information provided by first author based on retrospective recall).
**Blinding participants/parents**	LOW	Placebo and active medication were identical. (Information provided by first author).
**Blinding therapist**	LOW	Placebo and active medication were identical; therapist was blinded. (Information provided by first author).
**Blinding assessor**	LOW	Placebo and active medication were identical; assessor was blinded. (Information provided by first author).
**Incomplete data outcome**	LOW	ITT on > 94% of randomized subjects.<br>“Three subjects in the placebo group and two subjects in the CLON 0.2 mg/day group did not meet all entry criteria and were granted an exception by the sponsor for study enrollment.” (FDA report, pag. 23).
**Selective reporting**	LOW	All outcomes listed in the FDA report and of interest for the present meta-analysis were reported in the journal article.
**Notes**		First author provided additional information.

Kahbazi 2009

ITEM	RATING	SUPPORT
**Sequence generation**	LOW	“computer-generated code” (Journal article, pag. 235)
**Allocation concealment**	LOW	“The assignments were kept in sealed, opaque envelopes until the point of analysis of data.” (Journal article, pag. 235)
**Blinding participants/parents**	LOW	“Both tablets were encapsulated and were identical” (Journal article, pag. 235)
**Blinding therapist**	LOW	“Throughout the study, the person who administrated the medications, rater and patients were blind to assignments” (Journal article, pag. 235) “Both tablets were encapsulated and were identical” (Journal article, pag. 235)

**Blinding assessor**	LOW	"Throughout the study, the person who administrated the medications, rater and patients were blind to assignments" (Journal article, pag. 235). "Both tablets were encapsulated and were identical" (Journal article, pag. 235)
**Incomplete data outcome**	LOW	Balanced and low drop out: One patient dropped out from the modafinil group and two from the placebo group and were lost to follow-up (Journal article, pag. 235)
**Selective reporting**	UNCLEAR	No protocol/CSR/CT available.
**Notes**		Corresponding author contacted but no reply.

Kay 2009a

ITEM	RATING	SUPPORT
**Sequence generation**	UNCLEAR	Not specified.
**Allocation concealment**	UNCLEAR	Not specified.
**Blinding participants/parents**	LOW	"Study drugs (e.g., MAS XR and atomoxetine) and identical matching placebos were administered orally.." (Journal article, pag. 319).
**Blinding therapist**	UNCLEAR	Not specified.
**Blinding assessor**	UNCLEAR	Not specified.
**Incomplete data outcome**	LOW	"Treatment compliance was similar between cohorts. The mean treatment compliance in Cohort 1 was 97.9% among subjects receiving MAS XR and 96.1% among those receiving placebo. …The mean treatment compliance in Cohort 2 was 97.0% among subjects receiving atomoxetine and 97.3% among those receiving placebo." (Journal article, pag. 322). Balanced and low drop put (Journal article, fig.3)
**Selective reporting**	UNCLEAR	No protocol/CSR/CT available
**Notes**		Written to first author and manufacturer, but not possible to retrieve additional information for this study.

Kay 2009b

ITEM	RATING	SUPPORT
**Sequence generation**	UNCLEAR	Not specified.
**Allocation concealment**	UNCLEAR	Not specified.
**Blinding participants/parents**	LOW	"Study drugs (e.g., MAS XR and atomoxetine) and identical matching placebos were administered orally.." (Journal article, pag. 319).

**Blinding therapist**	UNCLEAR	Not specified.
**Blinding assessor**	UNCLEAR	Not specified.
**Incomplete data outcome**	LOW	"Treatment compliance was similar between cohorts. The mean treatment compliance in Cohort 1 was 97.9% among subjects receiving MAS XR and 96.1% among those receiving placebo. …The mean treatment compliance in Cohort 2 was 97.0% among subjects receiving atomoxetine and 97.3% among those receiving placebo." (Journal article, pag. 322). Balanced and low drop put (Journal article, fig.3)
**Selective reporting**	UNCLEAR	No protocol/CSR/CT available
**Notes**		Written to first author and manufacturer, but not possible to retrieve additional information for this study.

Kelsey 2004, B4Z-US-LYBG

ITEM	RATING	SUPPORT
**Sequence generation**	LOW	Information from full CSR, pag. 43 (available upon request from manufacturer)
**Allocation concealment**	LOW	Information from full CSR, pag. 43 (available upon request from manufacturer)
**Blinding participants/parents**	LOW	Information from full CSR, pag. 43 (available upon request from manufacturer)
**Blinding therapist**	LOW	Information from full CSR, pag. 43 (available upon request from manufacturer)
**Blinding assessor**	LOW	Information from full CSR, pag. 43 (available upon request from manufacturer)
**Incomplete data outcome**	UNCLEAR	Quite unbalanced drop out (between 10 and 20%) for lack of efficacy (2/133 in ATX and 8/64 in PBO). LOCF on 126/133 ATX assigned participants and 60/64 PBO assigned participants
**Selective reporting**	LOW	Outcomes of interest for the present meta-analysis and listed in full CSR (available upon request from manufacturer) are reported in the Journal article.
**Notes**		Manufacturer provided full CSR.

Kollins 2011, SPD503-206, NCT00150592

ITEM	RATING	SUPPORT
**Sequence generation**	LOW	Information from full CSR, 5.4.2 Method of assigning subjects to treatment groups, pag. 50-51 (available upon request from manufacturer)
**Allocation concealment**	LOW	Information from full CSR, 5.4.2 Method of assigning subjects to treatment groups, pag. 50-51 (available upon request from manufacturer)
**Blinding participants/parents**	LOW	"Subject, investigators, sponsor, and study site staff were blinded" (Journal article, pag. 112); matching placebo tablets (Journal article, figure 1).
**Blinding therapist**	LOW	"Subject, investigators, sponsor, and study site staff were blinded" (Journal article, pag. 112); matching

		placebo tablets (Journal article, figure 1).
**Blinding assessor**	LOW	"Subject, investigators, sponsor, and study site staff were blinded" (journal article, pag. 112); matching placebo tablets (Journal article, figure 1).
**Incomplete data outcome**	LOW	Randomized, n= 182; FAS/safety population: n= 178; low attrition (Journal article, figure 2)
**Selective reporting**	LOW	No protocol available. Manufacturer confirmed that the only planned outcome not reported in the Journal article, was the CGI-S, not of interest for the present meta-analysis.
**Notes**		Manufacturer provided additional information.

Kooij 2004

ITEM	RATING	SUPPORT
**Sequence generation**	LOW	"The order of treatment (methylphenidate–placebo or placebo–methylphenidate) was randomized by the pharmacist using a computer-generated list". (Journal article, pag. 976)
**Allocation concealment**	LOW	"Allocation was preserved until end of study; Allocation codes were at the pharmacy, not in our possession". (Information provided by the first author).
**Blinding participants/parents**	LOW	"Weekly supplies of methylphenidate or placebo were dispensed by the pharmacy in identically appearing tablets of 10 mg. Medication was prescribed under double-blind conditions in four or five times a day dosing". (Journal article, pag. 976)
**Blinding therapist**	LOW	First author confirmed therapist was blinded.
**Blinding assessor**	LOW	First author confirmed assessor was blinded.
**Incomplete data outcome**	LOW	No drop out.
**Selective reporting**	LOW	All planned outcomes are reported in the journal article (Information provided by the first author).
**Notes**		First author provided additional information.

Kurlan 2002

ITEM	RATING	SUPPORT
**Sequence generation**	LOW	"…computer-generated randomization plan". (Journal article, pag. 529).
**Allocation concealment**	LOW	"Each site was supplied with sealed envelopes that contained their subjects' treatment assignments in the event that such information was needed for emergent medical care, but in no instance did unblinding on this basis occur." (Journal article, pag. 529).
**Blinding**	LOW	"Only the programmer in the Biostatistics Center who generated the plan and the pharmacist in the

**participants/parents**		Pharmacy Center who packaged and labeled the drug were aware of the treatment assignments. Treatment assignments were not revealed to the subjects or investigators until the entire study was completed and data were analyzed.” (Journal article, pag. 529). “ matching placebo tablets”. (Journal article, pag. 529).
**Blinding therapist**	LOW	“Only the programmer in the Biostatistics Center who generated the plan and the pharmacist in the Pharmacy Center who packaged and labeled the drug were aware of the treatment assignments. Treatment assignments were not revealed to the subjects or investigators until the entire study was completed and data were analyzed.” (Journal article, pag. 529). “ matching placebo tablets” (Journal article, pag. 529).
**Blinding assessor**	LOW	“Only the programmer in the Biostatistics Center who generated the plan and the pharmacist in the Pharmacy Center who packaged and labeled the drug were aware of the treatment assignments. Treatment assignments were not revealed to the subjects or investigators until the entire study was completed and data were analyzed.” (Journal article, pag. 529). “ matching placebo tablets” (Journal article, pag. 529).
**Incomplete data outcome**	LOW	“The primary statistical analyses were performed according to the intention to treat principle and were based on all randomized subjects, as randomized. For the analyses of the outcome variables for efficacy, if a subject was missing a response at a particular visit, the last available observation for that subject was carried forward and imputed for that visit. “ (Journal article, pag. 530). Balanced and low drop out (Journal article, figure 1, pag. 530).
**Selective reporting**	LOW	First author confirmed that all planned outcomes are reported in the Journal article.
**Notes**		First author provided additional information.

Lin 2014, NCT00922636

ITEM	RATING	SUPPORT
**Sequence generation**	LOW	“An interactive voice-response system was used for randomization and to determine which study drug to dispense”. (Journal article, pag. 192).
**Allocation concealment**	LOW	“An interactive voice-response system was used for randomization and to determine which study drug to dispense”. (Journal article, pag. 192).
**Blinding participants/parents**	UNCLEAR	No details on how blinding was assured.
**Blinding therapist**	UNCLEAR	No details on how blinding was assured.
**Blinding assessor**	UNCLEAR	No details on how blinding was assured.
**Incomplete data outcome**	LOW	“The ITT database included data from all randomized patients”. (Journal article, pag. 193). Balanced drop out (for lack of efficacy: PBO: 6/63; OROS MPH: 1/26), Journal article, fig. 2)
**Selective reporting**	LOW	No protocol available. Outcomes of interest for the present meta-analysis and listed in Clinicaltrials.gov are reported in the Journal article.

Notes		Manufacturer contacted but could not share data.

Lin 2016, NCT00917371

ITEM	RATING	SUPPORT
**Sequence generation**	LOW	"..computer-generated random sequencing..". (Journal article, pag. 3).
**Allocation concealment**	UNCLEAR	Not specified.
**Blinding participants/parents**	UNCLEAR	Not reported how blinding was assured.
**Blinding therapist**	UNCLEAR	Not reported how blinding was assured.
**Blinding assessor**	UNCLEAR	Not reported how blinding was assured.
**Incomplete data outcome**	LOW	No drop outs (Journal article, figure 1).
**Selective reporting**	UNCLEAR	No protocol/CSR/Clinicaltrials.gov available. (Results not published in Clinicaltrials.gov available)
**Notes**		Corresponding author contacted but no reply.

Martenyi 2010, B4Z-MW-LYCZ, NCT00386581

ITEM	RATING	SUPPORT
**Sequence generation**	LOW	"Assignment to treatment groups was determined by a computer-generated random sequence using an interactive voice response system". (Journal article, pag. 59).
**Allocation concealment**	LOW	"Assignment to treatment groups was determined by a computer-generated random sequence using an interactive voice response system" (Journal article, pag. 59).
**Blinding participants/parents**	LOW	"Patients assigned to the placebo arm received identically matched placebo treatment." (Journal article, pag. 59). "..neither study personnel (rater, staff physician or nurse) nor patients was knowledgeable about the administration of active or placebo treatment." (Journal article, pag. 59).
**Blinding therapist**	LOW	"Patients assigned to the placebo arm received identically matched placebo treatment." (Journal article, pag. 59). "..neither study personnel (rater, staff physician or nurse) nor patients was knowledgeable about the administration of active or placebo treatment." (Journal article, pag. 59).
**Blinding assessor**	LOW	"Patients assigned to the placebo arm received identically matched placebo treatment." (Journal article, pag. 59)."..neither study personnel (rater, staff physician or nurse) nor patients was knowledgeable about the administration of active or placebo treatment." (Journal article, pag. 59).

**Incomplete data outcome**	LOW	LOCF on all randomized participants. Balanced drop outs, none for lack of efficacy (ATX: 5/72; PBO: 1/33, Journal article, fig. 1).
**Selective reporting**	LOW	Outcomes of interest for the present meta-analysis and listed in the full CSR (available upon request from manufacturer) are reported in the Journal article
**Notes**		Manufacturer provided full CSR.

McCracken 2016

ITEM	RATING	SUPPORT
**Sequence generation**	LOW	"Randomization was generated by a computer program, created by our Data Management Center". (Information provided by the first author)
**Allocation concealment**	UNCLEAR	No information.
**Blinding participants/parents**	UNCLEAR	Unclear how blinding was preserved.
**Blinding therapist**	UNCLEAR	Unclear how blinding was preserved.
**Blinding assessor**	UNCLEAR	Unclear how blinding was preserved.
**Incomplete data outcome**	LOW	Balanced drop outs (Journal article, fig. 1)
**Selective reporting**	UNCLEAR	No protocol/CSR/CT available.
**Notes**		First author provided additional information.

McRae-Clark 2010, R21DA018221, NCT00360269

ITEM	RATING	SUPPORT
**Sequence generation**	LOW	"Randomization was done using a simple randomization technique (random number generator) in a 1:1 (Active vs. Placebo) assignment pattern. Randomization was done by the dispensing pharmacy and the blind was kept until study completion." (Information provided by the first author)
**Allocation concealment**	LOW	"As above, and participants did not have access to the randomization sequence" (Information provided by the first author).
**Blinding participants/parents**	LOW	"Matching placebo" (Journal article, abstract)
**Blinding therapist**	LOW	First author confirmed they were blinded.
**Blinding assessor**	LOW	First author confirmed they were blinded.

**Incomplete data outcome**	HIGH	Balanced drop out but quite a large gap between randomized and ITT (78 randomized, ITT: 38).
**Selective reporting**	LOW	"All the ADHD outcomes we collected were reported" (Information provided by the first author).
**Notes**		First author provided additional information.

Medori 2008, LAMDA-I EUCTR2004-000730-37, NCT00246220

ITEM	RATING	SUPPORT
**Sequence generation**	LOW	"Randomization was based on a computer-generated randomization" (Journal article, pag. 982)
**Allocation concealment**	LOW	"Randomization was balanced by using permuted blocks of treatments, stratified by study center, and implemented via an interactive voice response system". (Journal article, pag. 982).
**Blinding participants/parents**	LOW	"Matching placebo" (in related paper: Rosler M, Ginsberg Y, Arngrim T, et al. Correlation of symptomatic improvements with functional improvements and patient-reported outcomes in adults with attention-deficit/hyperactivity disorder treated with OROS methylphenidate. The world journal of biological psychiatry; 2013;14(4):282-290, pag. 283)
**Blinding therapist**	LOW	"The over-encapsulated OROS methylphenidate and placebo were identical in appearance. The subjects, those delivering the medication and the assessors were all blinded." (information provided by manufacturer)
**Blinding assessor**	LOW	"The over-encapsulated OROS methylphenidate and placebo were identical in appearance. The subjects, those delivering the medication and the assessors were all blinded." (information provided by manufacturer)
**Incomplete data outcome**	LOW	"A total of 448 patients were screened and 402 patients were randomized into placebo or one of the three PR methylphenidate groups… The statistical analysis of efficacy included 394 patients, and safety assessment included 401 patients who received at least one dose of trial medication. Overall, 365 (91%) of randomized patients completed the 5-week double-blind study period", (Journal article, pag. 983). "The most common reason for study discontinuation was an adverse event in subjects receiving OROS MPH (*n* % 12; 3.9%) and lack of efficacy in subjects receiving placebo (*n* % 3; 3.1%)." (In related paper: Rosler M, Ginsberg Y, Arngrim T, et al. Correlation of symptomatic improvements with functional improvements and patient-reported outcomes in adults with attention-deficit/hyperactivity disorder treated with OROS methylphenidate. The world journal of biological psychiatry; 2013;14(4):282-290, pag. 285)
**Selective reporting**	LOW	All protocol study outcomes are reported in the journal article. (Information provided by manufacturer)
**Notes**		Manufacturer provided additional information

Michelson 2001, B4Z-MC-LYAC

ITEM	RATING	SUPPORT
**Sequence generation**	LOW	"Patients were randomized using computer-generated codes via an interactive voice response system" (Journal article, pag. 2).
**Allocation concealment**	LOW	"Patients were randomized using computer-generated codes via an interactive voice response system" (Journal article, pag. 2).
**Blinding participants/parents**	LOW	"The study drug for all treatment groups was identical in appearance." (Journal article, pag. 2)
**Blinding therapist**	LOW	Information from full CSR, pag. 96-97 (available upon request form manufacturer)
**Blinding assessor**	LOW	Information from full CSR, pag. 96-97 (available upon request form manufacturer) "The study drug for all treatment groups was identical in appearance." (Journal article, pag. 2)
**Incomplete data outcome**	LOW	"Analyses of efficacy measures included all randomized patients with both a baseline and a postbaseline measurement. Analyses of safety measures were restricted to randomized patients who took at least 1 dose of the study drug (either atomoxetine or placebo; 294 of 297 [98.9%] randomized patients)" (Journal article, pag. 2). Analysis of primary efficacy outcome: placebo: n=83(out of 84); ATX 0.5 mg/day: n=43 (out of 44); ATX 1.2 mg/day: n=82 (out of 84); ATX 1.8 mg/day: n=82 (out of 85).<br>Drop out less 20% and balanced (Journal article, figure 1)
**Selective reporting**	LOW	No protocol available. Outcomes listed in CSR (available upon request form manufacturer) are reported in the Journal article.
**Notes**		Manufacturer provided full CSR.

Michelson 2002, B4Z-MC-LYAT

ITEM	RATING	SUPPORT
**Sequence generation**	LOW	Information from full CSR, pag. 44 (available upon request form manufacturer)
**Allocation concealment**	LOW	Information from full CSR, pag. 44 (available upon request form manufacturer)
**Blinding participants/parents**	LOW	Information from full CSR, pag. 44 (available upon request form manufacturer)
**Blinding therapist**	LOW	Information from full CSR, pag. 44 (available upon request form manufacturer)
**Blinding assessor**	LOW	Information from full CSR, pag. 44 (available upon request form manufacturer)
**Incomplete data outcome**	LOW	"Patient data were analyzed on an intent-to-treat basis." (Journal article, pag. 1987); Information from full CSR, pag. 59 (available upon request form manufacturer)
**Selective reporting**	LOW	The only secondary outcome listed in the full CSR but not reported in the journal article is the SSRS-P scale, not relevant for the present meta-analysis.

**Notes**		Manufacturer provided full CSR.

Michelson 2003a

ITEM	RATING	SUPPORT
**Sequence generation**	LOW	"Patients were randomized according to computer-generated treatment codes obtained from an interactive voice-response system." (Journal article, pag. 113)
**Allocation concealment**	LOW	"Patients were randomized according to computer-generated treatment codes obtained from an interactive voice-response system." (Journal article, pag. 113)
**Blinding participants/parents**	LOW	"Patients and primary efficacy raters were blinded to both timing and magnitude of dosage increases"; "Study drug materials for both treatment groups were identical in appearance." (Journal article, pag. 113)
**Blinding therapist**	UNCLEAR	Additional information form full CSR (available upon request from manufacturer) but not clear if principal study investigators were the ones who delivered the treatment.
**Blinding assessor**	LOW	Information from full CSR, pag. 68 (available upon request from manufacturer)<br>"Efficacy raters for the primary outcome measure were blind to all details of the study design, including severity criteria for entry, dose titration, and timing of the initiation of therapy, and were not allowed to evaluate or ask about adverse events." "Study drug materials for both treatment groups were identical in appearance." (Journal article, pag. 113).
**Incomplete data outcome**	LOW	Balanced drop outs for lack of efficacy, both in study 1 and 2 (Journal article, fig 1 and 3). Analyzed 133/141 (ATX arm); 134/139 (PBO arm) (study 1); 124/129(ATX arm); 124/127 (PB) arm (study 2)
**Selective reporting**	UNCLEAR	Outcomes of interest for the present meta-analysis, and listed in the full CSR, are reported in the Journal article, with the exception of weight (one of our outcomes). Manufacturer replied that weight was not analyzed at week 4 (endpoint considered in our meta-analysis).
**Notes**		Manufacturer provided full CSR.

Michelson 2003b

ITEM	RATING	SUPPORT
**Sequence generation**	LOW	"Patients were randomized according to computer-generated treatment codes obtained from an interactive voice-response system." (Journal article, pag. 113)
**Allocation concealment**	LOW	"Patients were randomized according to computer-generated treatment codes obtained from an interactive voice-response system." (Journal article, pag. 113)
**Blinding participants/parents**	LOW	"Patients and primary efficacy raters were blinded to both timing and magnitude of dosage increases"; "Study drug materials for both treatment groups were identical in appearance." (Journal article, pag. 113)
**Blinding therapist**	UNCLEAR	Additional information form full CSR (available upon request from manufacturer) but not clear if principal study investigators were the ones who delivered the treatment.

**Blinding assessor**	LOW	Information from full CSR, pag. 68 (available upon request from manufacturer) "Efficacy raters for the primary outcome measure were blind to all details of the study design, including severity criteria for entry, dose titration, and timing of the initiation of therapy, and were not allowed to evaluate or ask about adverse events." "Study drug materials for both treatment groups were identical in appearance." (Journal article, pag. 113).
**Incomplete data outcome**	LOW	Balanced drop outs for lack of efficacy, both in study 1 and 2 (Journal article, fig 1 and 3). Analyzed 133/141 (ATX arm); 134/139 (PBO arm) (study 1); 124/129(ATX arm); 124/127 (PB) arm (study 2)
**Selective reporting**	UNCLEAR	Outcomes of interest for the present meta-analysis, and listed in the full CSR, are reported in the Journal article, with the exception of weight (one of our outcomes). Manufacturer replied that weight was not analyzed at week 4 (endpoint considered in our meta-analysis).
**Notes**		Manufacturer provided full CSR.

Moharari 2012, IRCT201012295500N1

ITEM	RATING	SUPPORT
**Sequence generation**	LOW	Table of random digits.
**Allocation concealment**	UNCLEAR	Not described.
**Blinding participants/parents**	UNCLEAR	Not described how blinding was preserved; only information available: "Assessor, patient, and family were unaware of drugs because the interventions were delivered within two packages named one and two." (Information provided by the translator)
**Blinding therapist**	UNCLEAR	Not described how blinding was preserved; only information available: "Assessor, patient, and family were unaware of drugs because the interventions were delivered within two packages named one and two." (Information provided by the translator)
**Blinding assessor**	UNCLEAR	Not described how blind was preserved; only information available: "Assessor, patient, and family were unaware of drugs because the interventions were delivered within two packages named one and two." (Information provided by the translator)
**Incomplete data outcome**	LOW	2/20 drop out in active medication arm, 0/20 in placebo (Information provided by the translator)
**Selective reporting**	LOW	Based on the registered protocol in http://www.irct.ir/searchresult.php?id=5500&number=1, C-GAS has been measured in three time points (before, 4 weeks and 8 weeks) but not reported [Only p value is available]), but this is not an outcome for our meta-analysis
**Notes**		One of our co-authors (FS) translated the paper from Farsi.

Montoya 2009, B4Z-XM-LYDM, NCT00191945

ITEM	RATING	SUPPORT
**Sequence generation**	LOW	"Allocation of who was to receive which treatment was performed by an ongoing centralized computer-generated random sequence". (Journal article, pag. 2747).
**Allocation concealment**	LOW	"Allocation of who was to receive which treatment was performed by an ongoing centralized computer-generated random sequence. The investigators accessed an automatic system and obtained in return a randomization number to identify the treatment to be used." (Journal article, pag. 2747)."The best way of ensuring allocation concealment was to use a centralized service, IVRS" (Information provided by first author).
**Blinding participants/parents**	LOW	"Patients, parents of participants, investigators (who administered the drugs) and raters were blinded"; "placebo and atomoxetine were identical in appearance" (Information provided by first author)
**Blinding therapist**	LOW	"Patients, parents of participants, investigators (who administered the drugs) and raters were blinded";"placebo and atomoxetine were identical in appearance" (Information provided by first author)
**Blinding assessor**	LOW	"Patients, parents of participants, investigators (who administered the drugs) and raters were blinded" "; "placebo and atomoxetine were identical in appearance" (Information provided by first author)
**Incomplete data outcome**	LOW	"The analyses were conducted with intention-to-treat principle; i.e., data from all randomized patients who took at least one dose of randomized treatment were used and patients were analyzed according to their initially assigned treatment group regardless of subsequent switching" (Journal article, pag. 2747). Balanced drop outs, not for reasons related to outcomes. Randomized: ATX: 100, PBO: 51; analyzed: ATX: 99; PBO: 50
**Selective reporting**	LOW	"Complementary measurement of the broader efficacy of atomoxetine based on health related quality of life was also obtained with both patient and parent-completed versions of the Child Health and Illness Profile and results were reported separately". (Information provided by first author) (not an outcome for the present meta-analysis)
**Notes**		First author provided additional information

NCT01069523

ITEM	RATING	SUPPORT
**Sequence generation**	LOW	Random number generator in Excel spreadsheet (Information provided by Dr. Pliszka, PI)
**Allocation concealment**	LOW	Only one staff was familiar with assignment (Information provided by Dr. Pliszka, PI)
**Blinding participants/parents**	LOW	Parent, child and assessing clinician were all blind. Staff member who randomized selected unmarked bottle from bin and gave to parent. Matching placebo (https://clinicaltrials.gov/ct2/show/NCT01069523)
**Blinding therapist**	UNCLEAR	Unclear

**Blinding assessor**	LOW	Parent, child and assessing clinician were all blind (Information confirmed by Dr. Pliszka, PI)
**Incomplete data outcome**	LOW	7/16 and 4/13 dropouts, in active and placebo arms, respectively
**Selective reporting**	LOW	Information for this study available from Clinicaltrials.gov; outcomes reported as planned in Clinicaltrials.gov
**Notes**		Additional information provided by Dr. Pliszka, PI

Newcorn 2008, B4Z-MC-LYBI

ITEM	RATING	SUPPORT
**Sequence generation**	LOW	Information from full CSR, pag. 198 (Available upon request from manufacturer)
**Allocation concealment**	LOW	Information from full CSR, pag. 33 (Available upon request from manufacturer)
**Blinding participants/parents**	LOW	"Identically appearing capsules" (Journal article, pag. 723)
**Blinding therapist**	LOW	Information from full CSR, pag. 198 (Available upon request from manufacturer)
**Blinding assessor**	LOW	Information from full CSR, pag. 198 (Available upon request from manufacturer)
**Incomplete data outcome**	LOW	ITT analysis on all randomized participants (Journal article, table 1). Not fully balanced in terms of drop out for lack of efficacy but very low drop out due to this reason (Journal article, pag. 727, figure 1): ATX: 0/186; MPH: 4/180; PBO: 4/74
**Selective reporting**	LOW	Outcomes listed in the full CSR (available upon request from manufacturer)are reported in the Journal article.
**Notes**		Manufacturer provided full CSR

Newcorn 2013, SPD503-314, NCT00997984

ITEM	RATING	SUPPORT
**Sequence generation**	UNCLEAR	Information provided by Shire from full CSR, Allocation of Subjects to Treatment, pag. 33/34 (available upon request from manufacturer) but not clear how the sequence was generated
**Allocation concealment**	LOW	"Eligible subjects were randomized 1:1:1 via an interactive web response system" (in related journal article, Stein MA, Sikirica V, Weiss MD, Robertson B, Lyne A, Newcorn JH. Does Guanfacine Extended Release Impact Functional Impairment in Children with Attention-Deficit/Hyperactivity Disorder? Results from a Randomized Controlled Trial. *CNS Drugs.* 2015;29(11):953-962., pag. 955)
**Blinding**	HIGH	"Because there was a high rate of somnolence observed in the GXR groups, there is a possibility that the

**participants/parents**		blind could have been broken, thereby potentially affecting the observed effect size." (Journal article, pag. 929)
**Blinding therapist**	HIGH	Because there was a high rate of somnolence observed in the GXR groups, there is a possibility that the blind could have been broken, thereby potentially affecting the observed effect size." (Journal article, pag. 929)
**Blinding assessor**	HIGH	Because there was a high rate of somnolence observed in the GXR groups, there is a possibility that the blind could have been broken, thereby potentially affecting the observed effect size." (Journal article, pag. 929)
**Incomplete data outcome**	HIGH	Unbalanced drop out for lack of efficacy (journal article, fig 1, pag 924), so although LOCF was implemented, still high risk of bias.
**Selective reporting**	LOW	Primary and secondary outcomes listed in the full CSR (available upon request from manufacturer) and of interest for the present meta-analysis all reported in the Journal articles. (CGI-I. reported in Stein MA, Sikirica V, Weiss MD, Robertson B, Lyne A, Newcorn JH. Does Guanfacine Extended Release Impact Functional Impairment in Children with Attention-Deficit/Hyperactivity Disorder? Results from a Randomized Controlled Trial. *CNS Drugs.* 2015;29(11):953-962, pag. 959).
**Notes**		Manufacturer provided additional information.

<u>Palumbo 2008, NCT00031395</u>

ITEM	RATING	SUPPORT
**Sequence generation**	LOW	"Computer-generated randomization plan". (Journal article, pag. 181).
**Allocation concealment**	LOW	Sealed envelopes (Journal article, pag. 182).
**Blinding participants/parents**	UNCLEAR	Matching placebo (abstract and pag. 182), although "Unblinding did occur for two subjects due to a severe allergic reaction that resulted in an emergency department visit, which was later determined not to be related to study medication"
**Blinding therapist**	UNCLEAR	Matching placebo (abstract and pag. 182), although "Unblinding did occur for two subjects due to a severe allergic reaction that resulted in an emergency department visit, which was later determined not to be related to study medication"
**Blinding assessor**	UNCLEAR	Matching placebo (abstract and pag. 182), although "Unblinding did occur for two subjects due to a severe allergic reaction that resulted in an emergency department visit, which was later determined not to be related to study medication"
**Incomplete data outcome**	HIGH	Unbalanced withdrawals for reasons related to the outcomes, with >25% withdrawal due to lack of efficacy in PCO arm (Journal article, fig. 1, pag. 183)
**Selective reporting**	LOW	All outcomes in Clinicaltrials.gov reported in the journal article.

**Notes**		Authors contacted, no reply.

Paterson 1999

ITEM	RATING	SUPPORT
**Sequence generation**	UNCLEAR	First author not able to provide the information.
**Allocation concealment**	UNCLEAR	First author not able to provide the information.
**Blinding participants/parents**	LOW	Blinding not broken (information provided by the first author).
**Blinding therapist**	LOW	Blinding not broken (information provided by the first author).
**Blinding assessor**	LOW	Blinding not broken (information provided by the first author).
**Incomplete data outcome**	LOW	1 drop out, included in the ITT analysis (Journal article, pag. 497)
**Selective reporting**	LOW	All outcomes of the protocol reported in the journal article. (Information provided by the first author)
**Notes**		First author provided additional information.

Philipsen 2015, EUCTR2006-000222-31-DE, ISRCTN54096201

ITEM	RATING	SUPPORT
**Sequence generation**	LOW	"The random sequence was computer generated". (Information provided by the authors).
**Allocation concealment**	LOW	"Centrally assigned" (Journal article, pag. 1200)
**Blinding participants/parents**	LOW	"Blinding is restricted to medical treatment (methylphenidate, placebo) and observer ratings of symptoms of ADHD (CAARS-O-L) and clinical global impression. Raters are not informed on the treatment allocation and are not involved in the trial except of interviewing the subjects. "(Protocol, published in Philipsen et al., ADHD Atten Def Hyp Disord (2010) 2:203–212, pag. 209). "Everybody was blind, and the blinding was preserved having identical capsules for medication and placebo". (Information provided by the authors)
**Blinding therapist**	LOW	"Blinding is restricted to medical treatment (methylphenidate, placebo) and observer ratings of symptoms of ADHD (CAARS-O-L) and clinical global impression. Raters are not informed on the treatment allocation and are not involved in the trial except of interviewing the subjects. "(protocol, published in Philipsen et al., ADHD Atten Def Hyp Disord (2010) 2:203–212, pag. 209). "Everybody was blind, and the blinding was preserved having identical capsules for medication and placebo" (information provided by the authors)

**Blinding assessor**	LOW	"Blinding is restricted to medical treatment (methylphenidate, placebo) and observer ratings of symptoms of ADHD (CAARS-O-L) and clinical global impression. Raters are not informed on the treatment allocation and are not involved in the trial except of interviewing the subjects. "(Protocol, published in Philipsen et al., ADHD Atten Def Hyp Disord (2010) 2:203–212, pag. 209). "Everybody was blind, and the blinding was preserved having identical capsules for medication and placebo" (Information provided by the authors)
**Incomplete data outcome**	UNCLEAR	For this study, we considered only two arms those with MPH/PBO plus CM (see figure 1) since the other arms do not meet our protocol criteria: for these, drop out for "patient wish" (not clear if this is for lack of efficacy) is 16/107 in PBO and 7/110 in MPH (Journal article, fig. 1, pag. 1201)
**Selective reporting**	LOW	Secondary outcomes (Symptom-checklist–SCL-90-R, Quality of Life Enjoyment and Satisfaction Questionnaire-Short Form (Q-LES-QSF), SF-36 V2.0, EQ-5D) reported in the protocol (published in Philipsen et al., ADHD Atten Def Hyp Disord (2010) 2:203–212, pag. 308, Table 3) but not in the journal article, but these are not of interest for the present meta-analysis.
**Notes**		Authors provided additional information.

<u>Pliszka 2000</u>

ITEM	RATING	SUPPORT
**Sequence generation**	LOW	" Random number list" (Information provided by the first author)
**Allocation concealment**	LOW	"Allocation concealment was password protected, unknown to participants and personnel; inly PI had access to list" (Information provided by the first author).
**Blinding participants/parents**	LOW	They were blinded and blinding was not broken. (Information provided by the first author).
**Blinding therapist**	LOW	Psychiatrist was blinded and blinding was not broken. (Information provided by the first author).
**Blinding assessor**	LOW	Assessor was blinded and blinding was not broken. (Information provided by the first author).
**Incomplete data outcome**	LOW	Balanced and low drop out: 2/20 drop outs in the placebo arm, 2/20 in the Adderall arms, and 1/18 in the MPH arm. (Journal article, pag. 621)
**Selective reporting**	LOW	All outcomes in the protocol were reported. (Information provided by the first author).
**Notes**		First author provided additional data.

<u>Reimherr, 2005</u>

ITEM	RATING	SUPPORT
**Sequence generation**	LOW	Generated by a computer. (Information provided by the author).

**Allocation concealment**	LOW	"Allocation concealment was preserved by the pharmacy putting med packets together independent of research staff. There was no staff overlap between research staff and the University's pharmacy." (Information provided by the author).
**Blinding participants/parents**	LOW	Active medication and PBO were identical in aspect. (Information provided by the author).
**Blinding therapist**	LOW	Active medication and PBO were identical in aspect. Therapist was blinded. (Information provided by the author).
**Blinding assessor**	LOW	Active medication and PBO were identical in aspect. Assessor was blinded. (Information provided by the author).
**Incomplete data outcome**	UNCLEAR	"Of the 59 patients who entered the study, 47 provided outcome data during the double-blind period. Patients dropping out either did not complete the single-blind evaluation or did not furnish outcome data during the double- blind period following randomization. There were no significant pre-treatment differences between these patients and those continuing in the study." (Journal article, pag. 248). Not clear if withdrawals were balanced between arms.
**Selective reporting**	LOW	Author confirmed that all planned outcomes were reported in the journal article.
**Notes**		Author provided additional information.

Reimherr, 2007

ITEM	RATING	SUPPORT
**Sequence generation**	LOW	Generated by a computer. (Information provided by the author).
**Allocation concealment**	LOW	"Allocation concealment was preserved by the pharmacy putting med packets together independent of research staff. There was no staff overlap between research staff and the University's pharmacy." (Information provided by the author).
**Blinding participants/parents**	LOW	Active medication and PBO were identical in aspect. (Information provided by the author).
**Blinding therapist**	LOW	Active medication and PBO were identical in aspect. Therapist was blinded. (Information provided by the author).
**Blinding assessor**	LOW	Active medication and PBO were identical in aspect. Assessor was blinded. (Information provided by the author).
**Incomplete data outcome**	LOW	"One patient dropped out during each treatment arm without contributing usable efficacy data." (Journal article, pag. 96)
**Selective reporting**	LOW	Author confirmed that all planned outcomes were reported in the journal article.
**Notes**		Author provided additional information.

Rosler, 2009

ITEM	RATING	SUPPORT
**Sequence generation**	LOW	Information provided by manufacturer, available upon request
**Allocation concealment**	UNCLEAR	No information.
**Blinding participants/parents**	LOW	Information provided by manufacturer, available upon request
**Blinding therapist**	LOW	Information provided by manufacturer, available upon request
**Blinding assessor**	UNCLEAR	No information.
**Incomplete data outcome**	HIGH	"The drop-out rate was lower in the MPH ER group compared to the placebo group (24 vs. 43%; Fisher's Exact Test, P < 0.001)" (Journal article, pag. 122). Unbalanced withdrawals for lack of efficacy (10% in active drug vs. 25% in placebo). Therefore, although LOCF was used, still high risk.
**Selective reporting**	LOW	Information provided by manufacturer: All protocol outcomes pertinent for the present meta-analysis reported in the Journal article.
**Notes**		Manufacturer provided additional information

Rugino, 2003

ITEM	RATING	SUPPORT
**Sequence generation**	LOW	"Computer-generated randomized list" (Journal article, pag. 137)
**Allocation concealment**	LOW	"A nurse not otherwise involved with the investigation assigned each patient into either the control or the treatment group according to a computer-generated randomized list. Without communicating which group the patient was assigned to, this nurse provided the caretaker with enough capsules to administer medication for approximately 2 weeks, with resupply at the reassessment visits." (Journal article, pag. 137).
**Blinding participants/parents**	UNCLEAR	How blinding was assured is not specified.
**Blinding therapist**	UNCLEAR	How blinding was assured is not specified.
**Blinding assessor**	UNCLEAR	How blinding was assured is not specified.
**Incomplete data outcome**	LOW	"One patient (5-year-old male with ADHD combined type and comorbid cerebral palsy, later deter- mined to have been assigned to the modafinil group) manifested repeated emesis that necessitated withdrawal from the study before the first postmedication evaluation. One patient (6-year-old female with ADHD combined type, later determined to have been assigned to the modafinil group) became unavailable before the first postmedication evaluation visit because of untoward social circumstances (house fire).

		Although these two children were excluded from data analysis, their TOVA ADHD *z* scores were similar to the completers (-4.10 and –3.63), suggesting that exclusion would not significantly alter the results of the data analysis." (Journal article, pag. 138).
**Selective reporting**	UNCLEAR	No protocol/CSR/Clinicaltrials.gov available
**Notes**		Author contacted, no reply; not possible to contact manufacturer.

Rugino, 2014, NCT01156051

ITEM	RATING	SUPPORT
**Sequence generation**	UNCLEAR	"As a result of early termination of the study, the randomization did not yield equal numbers of children in each group. Nonetheless, the placebo group and treatment group were reasonably well matched for baseline data (including for overnight polysomnographic parameters)." No description of sequence generation (Journal article, pag. 6)
**Allocation concealment**	UNCLEAR	No information.
**Blinding participants/parents**	UNCLEAR	No information.
**Blinding therapist**	UNCLEAR	No information.
**Blinding assessor**	UNCLEAR	No information.
**Incomplete data outcome**	UNCLEAR	1 drop out in each arm (journal article, pag.3) but study discontinued early
**Selective reporting**	LOW	No protocol/CSR available; outcomes listed in Clinicaltrails.gov reported in the journal article
**Notes**		Author contacted, no reply. Manufacturer not able to provide additional data/information.

Sallee 2009, SPD503-304, NCT00150618

ITEM	RATING	SUPPORT
**Sequence generation**	UNCLEAR	Information from CSR, section 5.4.2 Method of assigning subjects to treatment groups, pag. 44/45 (available upon request from manufacturer) but not clear how sequence was generated
**Allocation concealment**	LOW	Information from CSR, section 5.4.2 Method of assigning subjects to treatment groups, pag. 44/45 (available upon request from manufacturer)
**Blinding participants/parents**	LOW	"The true identity of each pill depended on the randomization regimen and remained unknown to clinicians and the subjects" (Journal article pag. 156). Information from CSR, section 5.4.2 Method of assigning subjects to treatment groups, pag. 44/45 (available upon request from manufacturer)
**Blinding therapist**	LOW	"The true identity of each pill depended on the randomization regimen and remained unknown to

		clinicians and the subjects" (Journal article pag. 156). Information from CSR, section 5.4.2 Method of assigning subjects to treatment groups, pag. 44/45 (available upon request from manufacturer)
**Blinding assessor**	LOW	"The true identity of each pill depended on the randomization regimen and remained unknown to clinicians and the subjects" (Journal article pag. 156). Information from CSR, section 5.4.2 Method of assigning subjects to treatment groups, pag. 44/45 (available upon request from manufacturer)
**Incomplete data outcome**	LOW	LOCF (PBO: 63/66; GXR 1 mg/day: 57/62; GXR 2 mg/day: 4/65; GXR 3 mg/day: 63/65; GXR 4 mg/day: 60/66); Quite balanced drop out for lack of efficacy (PBO: 6/66; GXR 1 mg/day: 1/62; GXR 2 mg/day: 4/65; GXR 3 mg/day: 7/65; GXR 4 mg/day: 4/66) (Journal article, fig.2, pag. 158)
**Selective reporting**	LOW	No protocol/CSR available; outcomes listed in Clinicaltrials.gov reported in the journal article (except for CHQ-PF50, not related to the outcomes of the present meta-analysis)
**Notes**		Manufacturer provided additional information.

Sangal 2006, B4Z-US-LYAV

ITEM	RATING	SUPPORT
**Sequence generation**	UNCLEAR	No clear information.
**Allocation concealment**	UNCLEAR	No clear information.
**Blinding participants/parents**	UNCLEAR	Not clear how blinding was preserved.
**Blinding therapist**	UNCLEAR	Not clear how blinding was preserved.
**Blinding assessor**	UNCLEAR	Not clear how blinding was preserved.
**Incomplete data outcome**	HIGH	85 subjects were randomised, but only 50 were analyzed (journal article, pag. 1578). No imputation methods were used.
**Selective reporting**	UNCLEAR	No protocol/CSR/Clinicaltrials available.
**Notes**		manufacturer provided additional information.

Scahill 2011, NCT00004376

ITEM	RATING	SUPPORT
**Sequence generation**	UNCLEAR	Not clear how sequence was generated.

**Allocation concealment**	UNCLEAR	Not clear how allocation concealment was preserved.
**Blinding participants/parents**	UNCLEAR	Not clear how blinding was assured.
**Blinding therapist**	UNCLEAR	Not clear how blinding was assured.
**Blinding assessor**	UNCLEAR	Not clear how blinding was assured.
**Incomplete data outcome**	UNCLEAR	Drop outs not clearly stated.
**Selective reporting**	UNCLEAR	No protocol/CSR/CT available.
**Notes**		Author contacted, not able to provide additional information.

Schrantee 2016, NTR3103, EUCTR2010-023654-37-NL

ITEM	RATING	SUPPORT
**Sequence generation**	LOW	"Central computer using a specialized computer program developed by the Clinical Research Unit" (Protocol, pag. 28)
**Allocation concealment**	LOW	"Allocation will be concealed for all parties" (Protocol, pag. 28)<br>"pharmacy controlled using sequentially numbered containers" (Information provided by first author).
**Blinding participants/parents**	LOW	"The placebo tablet matches the MPH tablet with respect to appearance, size, shape and presence of scoring line" (Protocol, pag. 25)
**Blinding therapist**	LOW	"The placebo tablet matches the MPH tablet with respect to appearance, size, shape and presence of scoring line" (Protocol, pag. 25). Therapist was blinded (information provided by the first author)
**Blinding assessor**	LOW	"The placebo tablet matches the MPH tablet with respect to appearance, size, shape and presence of scoring line" (Protocol, pag. 25). Assessor was blinded (information provided by the first author)
**Incomplete data outcome**	LOW	Low number of drop outs, balanced, none for lack of efficacy (Journal article, fig. 1)
**Selective reporting**	LOW	Results on additional outcomes on ADHD severity are currently being published elsewhere (Information provided by first author).
**Notes**		First author provided additional information. Full study protocol in supplemental material of Schrantee et al. *JAMA Psychiatry* 2016

Schulz 2012

ITEM	RATING	SUPPORT
**Sequence generation**	UNCLEAR	No information.
**Allocation concealment**	UNCLEAR	No information.
**Blinding participants/parents**	UNCLEAR	No information on how blinding was preserved.
**Blinding therapist**	UNCLEAR	No information on how blinding was preserved.
**Blinding assessor**	UNCLEAR	No information on how blinding was preserved.
**Incomplete data outcome**	LOW	Low and balanced withdrawals (2/21 discontinuation in each arm for lack of efficacy, Journal article figure 1, pag. 954)
**Selective reporting**	UNCLEAR	No protocol/CSR/Clinicaltrials.gov available.
**Notes**		Not possible to gather additional information from authors.

Simonoff 2013, ISRCTN68384912

ITEM	RATING	SUPPORT
**Sequence generation**	LOW	Computer generated. (Confirmed by the first author).
**Allocation concealment**	LOW	"Allocation was undertaken independently of the trial team" (Journal article, pag. 528)<br>"Once participants agreed to take part and all baseline data were collected, their participant number was sent to the Clinical Trials Unit who undertook randomization and sent the allocation directly to the centralized dispensing pharmacy, bypassing the research team. The dispensing pharmacy (St Thomas Hospital) sent medication directly to patients" (Information provided by the first author).
**Blinding participants/parents**	LOW	"..matching placebo in identical 'doses'" (Journal article, pag. 528)<br>"blinded were participants, researchers and all members of the trial team who had contact or potential contact with the participants (e.g., also the senior investigators). Only the pharmacy and the CTU had access the to the randomization allocation". (Information provided by the first author)
**Blinding therapist**	LOW	"…matching placebo in identical doses'" (Journal article, pag. 528)<br>"blinded were participants, researchers and all members of the trial team who had contact or potential contact with the participants (e.g., also the senior investigators). Only the pharmacy and the CTU had access the to the randomization allocation". (Information provided by the first author)
**Blinding assessor**	LOW	"..matching placebo in identical 'doses'" (Journal article, pag. 528)<br>"blinded were participants, researchers and all members of the trial team who had contact or potential

		contact with the participants (e.g., also the senior investigators). Only the pharmacy and the CTU had access the to the randomization allocation". (Information provided by the first author)
**Incomplete data outcome**	LOW	Data from all participants analyzed with multiple imputation by chained equations. (Journal article, pag. 529)
**Selective reporting**	LOW	"All measures agreed in the SAP were reported". (Information provided by the first author).
**Notes**		First author provided additional information.

Singer 1995

ITEM	RATING	SUPPORT
**Sequence generation**	UNCLEAR	Not specified.
**Allocation concealment**	UNCLEAR	Not specified.
**Blinding participants/parents**	LOW	"Uniform appearing capsules in numbered containers..all health care providers, raters, patients and parents were blinded" (Journal article, pag. 75).
**Blinding therapist**	LOW	"Uniform appearing capsules in numbered containers..all health care providers, raters, patients and parents were blinded" (Journal article, pag. 75).
**Blinding assessor**	LOW	"Uniform appearing capsules in numbered containers..all health care providers, raters, patients and parents were blinded" (Journal article, pag. 75).
**Incomplete data outcome**	LOW	Low drop out. "Of the 37 patients who entered the study, three were withdrawn either during or immediately after the first treatment phase One was removed for failure to comply with medication administration, and two at the request of the parents: on because the parents believed that the first "medication" was so successful and the other because it caused so many side effects that they refused to allow their child to continue in this blinded study. When the code was broken, in both instances the individual was receiving placebo." (Journal article, pag. 76)
**Selective reporting**	UNCLEAR	No protocol/CSR/Clinicaltrials.gov available
**Notes**		Not possible to contact authors.

SPD489-405, NCT01552915

ITEM	RATING	SUPPORT
**Sequence generation**	UNCLEAR	No information

**Allocation concealment**	UNCLEAR	No information
**Blinding participants/parents**	UNCLEAR	No information
**Blinding therapist**	UNCLEAR	No information
**Blinding assessor**	UNCLEAR	No information
**Incomplete data outcome**	LOW	Drop outs for lack of efficacy < 10% in each arm (https://clinicaltrials.gov/ct2/show/NCT01552915; additonal data provided by manufacturer, available upon request from manufacturer)
**Selective reporting**	LOW	Information for this study available from Clinicaltrials.gov; outcomes reported as planned in Clinicaltrials.gov.
**Notes**		Manufacturer provided additional data

SPD489-406, NCT01552902

ITEM	RATING	SUPPORT
**Sequence generation**	UNCLEAR	No information
**Allocation concealment**	UNCLEAR	No information
**Blinding participants/parents**	UNCLEAR	No information
**Blinding therapist**	UNCLEAR	No information
**Blinding assessor**	UNCLEAR	No information
**Incomplete data outcome**	LOW	A total of 547 participants were treated and the reasons for 2 'randomized but not treated' participants included withdrawal by 1 participant in the Methylphenidate group and 1 participant with a protocol violation in the Lisdexamfetamine group (https://clinicaltrials.gov/ct2/show/NCT01552902, additonal information provided by manufacturer, available upon request from manufacturer)
**Selective reporting**	LOW	Information for this study available from Clincialtrials.gov; outcomes reported as planned in Clinicaltrials.gov.
**Notes**		Additional data for this study provided by manufacturer

Spencer 1995

ITEM	RATING	SUPPORT
**Sequence generation**	UNCLEAR	No information.

**Allocation concealment**	UNCLEAR	No information.
**Blinding participants/parents**	LOW	Placebo and active dug “identically appearing”. (Journal article, pag. 436).
**Blinding therapist**	UNCLEAR	No information.
**Blinding assessor**	UNCLEAR	No information.
**Incomplete data outcome**	UNCLEAR	2/25 subjects dropped out, both in the MPH arm, not included in the analyses.
**Selective reporting**	UNCLEAR	No protocol/CSR/Clinicaltrials.gov available.
**Notes**		First author contacted, no reply.

Spencer 1998

ITEM	RATING	SUPPORT
**Sequence generation**	UNCLEAR	No information.
**Allocation concealment**	UNCLEAR	No information.
**Blinding participants/parents**	UNCLEAR	No information.
**Blinding therapist**	UNCLEAR	No information.
**Blinding assessor**	UNCLEAR	No information.
**Incomplete data outcome**	LOW	“Of the 22 patients enrolled in the study, one was dropped because of emergent anxiety and irritability during the second week of tomoxetine treatment. Thus, the final study group consisted of 11 women and 10 men” (Journal article, pag. 694).
**Selective reporting**	UNCLEAR	No protocol/CSR/Clinicaltrials.gov available.
**Notes**		First author contacted, no reply.

Spencer 2001

ITEM	RATING	SUPPORT
**Sequence generation**	UNCLEAR	“The order of treatment (Adderall, placebo, or placebo, Adderall) was randomized by the research pharmacy”. (Journal article, pag. 776), but not clear how sequence was generated.
**Allocation concealment**	UNCLEAR	No information.

**Blinding participants/parents**	LOW	Active medication and placebo: identically-appearing. (Journal article, pag. 776).
**Blinding therapist**	LOW	“Study physicians prescribed medication under double-blind condition”. (Journal article, pag. 776). Active medication and placebo: identically-appearing. (Journal article, pag. 776).
**Blinding assessor**	LOW	“Raters were blind to treatment assignment”. (Journal article, pag. 776). Active medication and placebo: identically-appearing. (Journal article, pag. 776).
**Incomplete data outcome**	UNCLEAR	3/30 participants in the placebo arm dropped out; reasons not clear.
**Selective reporting**	UNCLEAR	No protocol/CSR/CT available.
**Notes**		First author contacted, no reply.

Spencer 2002a, B4Z-MC-HFBD

ITEM	RATING	SUPPORT
**Sequence generation**	LOW	Information from full CSR, pag. 76 (available upon request from manufacturer)
**Allocation concealment**	LOW	Information from full CSR, pag. 76 (available upon request from manufacturer)
**Blinding participants/parents**	LOW	Information from full CSR, pag. 2650 (available upon request from manufacturer)
**Blinding therapist**	LOW	Information from full CSR, pag. 76 and 2650 (available upon request from manufacturer)
**Blinding assessor**	LOW	Information from full CSR, pag. 76 and 2650 (available upon request from manufacturer)
**Incomplete data outcome**	UNCLEAR	Unbalanced drop out for lack of efficacy (The most common reason for early study discontinuation in both studies combined was lack of efficacy (atomoxetine 7.8%, placebo 13.7%). Imputation methods not adequate when unbalanced drop out. (Journal article, fig.1, and pag. 1144)
**Selective reporting**	LOW	CGI-I (one of the outcomes for the present meta-analysis) not reported in the Journal article but reported in the shorter CSR (publically available)
**Notes**		Manufacturer provided full CSR

Spencer 2002b, B4Z-MC-HFBK

ITEM	RATING	SUPPORT
**Sequence generation**	LOW	Information from full CSR, pag. 76 (available upon request from manufacturer)
**Allocation**	LOW	Information from full CSR, pag. 76 (available upon request from manufacturer)

**concealment**		
**Blinding participants/parents**	LOW	Information from full CSR, pag. 2614 (available upon request from manufacturer)
**Blinding therapist**	LOW	Information from full CSR, pag. 76 and 2614 (available upon request from manufacturer)
**Blinding assessor**	LOW	Information from full CSR, pag. 76 and 2614 (available upon request from manufacturer)
**Incomplete data outcome**	UNCLEAR	Unbalanced drop out for lack of efficacy (The most common reason for early study discontinuation in both studies combined was lack of efficacy (atomoxetine 7.8%, placebo 13.7%). Imputation methods not adequate when unbalanced drop out. (Journal article, fig.1, and pag. 1144)
**Selective reporting**	LOW	CGI-I (one of the outcomes for the present meta-analysis) not reported in the Journal article but reported in the shorter CSR (publically available)
**Notes**		Manufacturer provided full CSR

<u>Spencer 2005</u>

ITEM	RATING	SUPPORT
**Sequence generation**	UNCLEAR	No information.
**Allocation concealment**	UNCLEAR	No information.
**Blinding participants/parents**	LOW	"Weekly supplies of MPH or placebo were dispensed by the pharmacy in identically appearing 5- and10-mg capsules". (Journal article, pag. 457)
**Blinding therapist**	UNCLEAR	"Study physicians prescribed medication under double-blind conditions"; "Weekly supplies of MPH or placebo were dispensed by the pharmacy in identically appearing 5- and10-mg capsules". (Journal article, pag. 457).
**Blinding assessor**	UNCLEAR	"Raters were blind to treatment assignment"; "Weekly supplies of MPH or placebo were dispensed by the pharmacy in identically appearing 5- and10-mg capsules". (Journal article, pag. 457).
**Incomplete data outcome**	HIGH	Unbalanced reasons for drop out: "Of the 146 subjects enrolled in the study, 136 (93%) completed at least 2 weeks of treatment. Of those, 110 (81%) completed the full 6 weeks. The dropout rate did not differ between medication and placebo … (Table 2). Of reasons for dropout, only "no effect" was statistically significant (placebo > MPH)." Journal article, pag 459. Imputation methods not adequate when unbalanced drop out.
**Selective reporting**	UNCLEAR	No protocol/CSR/CT available.
**Notes**		First author contacted, no reply.

Spencer 2006, SLI381-314, NCT00507065

ITEM	RATING	SUPPORT
**Sequence generation**	UNCLEAR	Information from CSR, section 5.4.2 Method of assigning subjects to treatment groups, pag. 32 (available upon request from manufacturer), but not clear how sequence was generated.
**Allocation concealment**	LOW	Information from CSR, 5.4.1. Description of batch information, pag. 32/33 (available upon request from manufacturer)
**Blinding participants/parents**	LOW	Information from CSR section 5.4.5.1 Description of blinding (available upon request from manufacturer)
**Blinding therapist**	LOW	Information from CSR section 5.4.5.1 Description of blinding (available upon request from manufacturer)
**Blinding assessor**	LOW	Information from CSR section 5.4.5.1 Description of blinding (available upon request from manufacturer)
**Incomplete data outcome**	LOW	ITT on 54/56 in MAS XR 10 mg/day, 53/56 in MAS XR 20 mg/day, 58/58 MAS XR 30 mg/day, 61/64 MAS XR 40 mg/day, 52/54 in PBO arms, respectively. Balanced reasons for drop outs; no drop out specifically stated for lack of efficacy. (Journal article, fig. 2)
**Selective reporting**	LOW	The only planned outcome not reported in the Journal article was the CGI-S, not relevant for the present meta-analysis. (Information provided by manufacturer).
**Notes**		Manufacturer provided additional information.

Spencer 2007, CRIT124E2302

ITEM	RATING	SUPPORT
**Sequence generation**	UNCLEAR	No information.
**Allocation concealment**	UNCLEAR	No information.
**Blinding participants/parents**	UNCLEAR	No information.
**Blinding therapist**	UNCLEAR	No information.
**Blinding assessor**	UNCLEAR	No information.
**Incomplete data outcome**	LOW	Balanced and low drop out. ITT: 218; randomized : 221. (Journal article, fig. 1)
**Selective reporting**	UNCLEAR	No protocol/CSR/CT available.
**Notes**		First author contacted, no reply. No additional information from manufacturer.

Spencer 2008, SPD465-301, NCT00150579

ITEM	RATING	SUPPORT
**Sequence generation**	LOW	Information from protocol, Version 2.0, 18 July 2005, sections 5.1.1.1 Randomization and blinding 5.1.1.2 Allocation of subjects to treatment 5.1.1.3 Labeling, packaging, storage and handling, pag. 25/26/27 (available upon request from manufacturer)
**Allocation concealment**	LOW	Information from protocol, Version 2.0, 18 July 2005, sections 5.1.1.1 Randomization and blinding 5.1.1.2 Allocation of subjects to treatment 5.1.1.3 Labeling, packaging, storage and handling, pag. 25/26/27 (available upon request from manufacturer)
**Blinding participants/parents**	LOW	"Matching placebo" (Journal article, pag. 1439).
**Blinding therapist**	UNCLEAR	No information.
**Blinding assessor**	UNCLEAR	No information.
**Incomplete data outcome**	HIGH	ITT in 95.7% of randomized participants, but unbalanced drop out for lack of efficacy (PBO: 30/137; Triple-bead MAS: 10/137) (Journal article, Table 1).
**Selective reporting**	LOW	The only outcome listed in the protocol and not reported was the AIM-A (secondary outcomes), not relevant for the present meta-analysis. (Information provided by manufacturer)
**Notes**		First author contacted, no reply. Manufacturer provided additional information.

Stein 2011, NCT00393042

ITEM	RATING	SUPPORT
**Sequence generation**	LOW	"Sequence was computer generated by a research pharmacist". (Information provided by the first author).
**Allocation concealment**	LOW	"The research pharmacist developed a randomization schedule for order of study drug and randomization of the placebo weeks, and prepared weekly blister packs for each subject containing capsules of study drug, which were indistinguishable from each other." (Information provided by the first author).
**Blinding participants/parents**	LOW	"The research pharmacist developed a randomization schedule for order of study drug and randomization of the placebo weeks, and prepared weekly blister packs for each subject containing capsules of study drug, which were indistinguishable from each other." (Journal article, pag. 582).
**Blinding therapist**	LOW	"The research pharmacist developed a randomization schedule for order of study drug and randomization of the placebo weeks, and prepared weekly blister packs for each subject containing capsules of study drug, which were indistinguishable from each other." (Journal article, pag. 582). "Therapist was blinded". (Information provided by the first author).
**Blinding assessor**	LOW	"The research pharmacist developed a randomization schedule for order of study drug and randomization of the placebo weeks, and prepared weekly blister packs for each subject containing capsules of study drug, which were indistinguishable from each other. "(Journal article, pag. 582). "Assessor was blinded".

		(Information provided by the first author).
**Incomplete data outcome**	UNCLEAR	Not clear in which arm/phase the 9 drop outs occurred.
**Selective reporting**	LOW	All outcome from the protocol reported in journal article, except sleep outcomes (not relevant for our meta-analysis, reported in another journal article. (Information provided by the first author).
**Notes**		First author provided additional information.

Sutherland 2012, NCT00174226

ITEM	RATING	SUPPORT
**Sequence generation**	UNCLEAR	No information.
**Allocation concealment**	UNCLEAR	No information.
**Blinding participants/parents**	UNCLEAR	No information.
**Blinding therapist**	UNCLEAR	No information.
**Blinding assessor**	UNCLEAR	No information.
**Incomplete data outcome**	UNCLEAR	"A total of 241 adults were randomized to treatment… The intent-to-treat population was based on 241 participants" (Journal article, pag. 447). Method used: MMRM. But high drop-out rate (33%).
**Selective reporting**	LOW	No protocol/CSR available; all outcomes listed in clinialtrial.gov reported in the journal article (which includes additional outcomes)
**Notes**		Corresponding author and manufacturer not able to provide additional information.

Svanborg 2009, B4Z-SO-LY15, EUCTR2004-003941-42-SE, NCT00191542

ITEM	RATING	SUPPORT
**Sequence generation**	UNCLEAR	"Randomization using an interactive voice system, stratified by site" (Journal article, pag. 242) but not clear how sequence was generated.
**Allocation concealment**	LOW	"Randomization using an interactive voice system, stratified by site" (Journal article, pag. 242)
**Blinding participants/parents**	LOW	"..identical placebo capsules" (Journal article, pag. 242).
**Blinding therapist**	UNCLEAR	No information.
**Blinding assessor**	UNCLEAR	Not information.

**Incomplete data outcome**	LOW	No drop outs (Journal article, figure 1).
**Selective reporting**	LOW	Outcomes of interest for the present meta-analysis listed in the full CSR reported in the journal article.
**Notes**		Manufacturer provided full CSR.

Swanson 2006

ITEM	RATING	SUPPORT
**Sequence generation**	UNCLEAR	No information.
**Allocation concealment**	UNCLEAR	No information.
**Blinding participants/parents**	LOW	“Matching placebo” (Journal article, pag. 139).
**Blinding therapist**	UNCLEAR	No information.
**Blinding assessor**	UNCLEAR	No information.
**Incomplete data outcome**	HIGH	ITT on 63/64 participants assigned to PBO and 120/126 participants assigned to Modafinil. Unbalanced drop out for lack of efficacy (PBO: 17/64; Modafinil: 17/126). Imputation methods not adequate if unbalanced drop out.
**Selective reporting**	UNCLEAR	No protocol/CSR/CT available.
**Notes**		Author contacted, no reply; not possible to contact manufacturer.

Takahashi 2009, B4Z-JE-LYBC, NCT00191295

ITEM	RATING	SUPPORT
**Sequence generation**	LOW	“Patients were randomized using computer-generated codes” (Journal article, pag. 343).
**Allocation concealment**	LOW	“Patients were randomized using computer-generated codes” (Journal article, pag. 343).
**Blinding participants/parents**	LOW	“Both investigators and patients were blinded to the dose by using capsules that were identical in appearance for all treatment groups” (Journal article, pag. 343)
**Blinding therapist**	LOW	“Both investigators and patients were blinded to the dose by using capsules that were identical in appearance for all treatment groups” (Journal article, pag. 343)
**Blinding assessor**	LOW	“Both investigators and patients were blinded to the dose by using capsules that were identical in

		appearance for all treatment groups" (Journal article, pag. 343)
**Incomplete data outcome**	LOW	LOCF; low drop out (1/62in PBO; 2/62 in ATMX 0.5 mg/day; 3/60 in ATMX 1.2 mg/day; 5/61 in ATMX 1.8 mg/day) (Journal article, fig. 1)
**Selective reporting**	LOW	Outcomes listed in the short CSR reported in the journal article.
**Notes**		

Takahashi 2014, NCT01323192

ITEM	RATING	SUPPORT
**Sequence generation**	LOW	"..computer-generated randomization schedule" (Journal article, pag. 489)
**Allocation concealment**	LOW	"the study drug container had a multipart label containing the protocol number, medication kit number, code number and other information on each part. It was impossible to identify the study drug by using the label information" (Journal article, pag. 491)
**Blinding participants/parents**	LOW	"matching placebo" (Journal article, pag. 490)
**Blinding therapist**	UNCLEAR	No information
**Blinding assessor**	UNCLEAR	No information
**Incomplete data outcome**	LOW	Balanced drop out. LOCF on 140/141 participants assigned to PBO and 143/143 assigned to active medication.
**Selective reporting**	LOW	Outcomes listed in CT reported in the Journal article.
**Notes**		First author contacted, no reply. Manufacturer does not have access to the CSR of this trial.

Taylor 1987

ITEM	RATING	SUPPORT
**Sequence generation**	LOW	Generated via computer (Information provided by first author)
**Allocation concealment**	LOW	"allocation to order conditions was carried out by pharmacy staff who knew only the name and identifying number of each case.. Tablets were dispensed to a study member, who did not know what they contained, and were handed by him to parents. It is therefore unlikely that any extraneous cues could have broken the blindness of the initial allocation" (Journal article, pag. 124)
**Blinding participants/parents**	LOW	"allocation to order conditions was carried out by pharmacy staff who knew only the name and identifying number of each case.. Tablets were dispensed to a study member, who did not know what they contained, and were handed by him to parents. It is therefore unlikely that any extraneous cues could have broken

		the blindness of the initial allocation" (Journal article, pag. 124)
**Blinding therapist**	LOW	"Tablets were dispensed to a trial member, who did not know what they contained and handed them to parents" (Journal article, pag. 124)
**Blinding assessor**	UNCLEAR	"However, shortage of personnel made it necessary on several occasions for the psychiatrist interviewing to be the same person who had supervised medication. His judgement, although blind, could have been contaminated by knowledge of how the child had changed in other settings." (Journal article, pag. 125). – outcome assessor should not be same person supervising medication assignment.
**Incomplete data outcome**	LOW	39 entered the trial, and 38 completed it (Journal article, pag. 128).
**Selective reporting**	LOW	"I am confident that there were no planned outcomes that were not reported. But unfortunately I have not been able to find a copy of the original approved protocol to give full documentary assurance of this" (Information provided by first author)
**Notes**		First author provided additional information

<u>Taylor 2000</u>

ITEM	RATING	SUPPORT
**Sequence generation**	UNCLEAR	No information.
**Allocation concealment**	UNCLEAR	No information.
**Blinding participants/parents**	LOW	"..drug or placebo in unmarked capsules". (Journal article, pag. 313).
**Blinding therapist**	UNCLEAR	No information.
**Blinding assessor**	LOW	Throughout, a pharmacy prepared, distributed and tracked all the drugs, separately from raters and subjects in order to maintain double-blind conditions." (Journal article, pag. 313)
**Incomplete data outcome**	UNCLEAR	Drop outs not specified.
**Selective reporting**	UNCLEAR	No protocol/CSR/CT available.
**Notes**		Contacted author but not valid e-mail; no other e-mail addresses found

Taylor 2001

ITEM	RATING	SUPPORT
**Sequence generation**	UNCLEAR	Not specified
**Allocation concealment**	UNCLEAR	Not specified
**Blinding participants/parents**	LOW	"drug or placebo in unmarked capsules" (Journal article, pag. 224)
**Blinding therapist**	UNCLEAR	Not specified
**Blinding assessor**	UNCLEAR	Not specified
**Incomplete data outcome**	UNCLEAR	Drop outs not specified
**Selective reporting**	UNCLEAR	No protocol/CSR/CT available.
**Notes**		Contacted author but not valid e-mail; no other e-mail addresses found.

Van Der Meere, 1999

ITEM	RATING	SUPPORT
**Sequence generation**	UNCLEAR	Storebo et al. Cochrane meta-analysis (2015): randomization list by pharmacist, but not clear how randomization sequence was generated.
**Allocation concealment**	UNCLEAR	To ensure blinding, pharmacists applied randomisation blocks at random at a length of 2 or 4 participants (Storebo et al. Cochrane meta-analysis (2015), but not clear how sequence was concealed
**Blinding participants/parents**	UNCLEAR	No information on how blinding was preserved.
**Blinding therapist**	UNCLEAR	No information on how blinding was preserved.
**Blinding assessor**	UNCLEAR	No information on how blinding was preserved.
**Incomplete data outcome**	UNCLEAR	Storebo et al. Cochrane meta-analysis (2015): ITT but not clear which method was used and if drop outs were balanced across arms
**Selective reporting**	UNCLEAR	No protocol/CSR/CT
**Notes**		Author unable to provide additional data; additional information from Storebo et al. Cochrane meta-analysis (2015), who gathered the text of the related thesis and sent us part of it.

Wang 2007, NCT00486083, B4Z-MC-LYBR (6934)

ITEM	RATING	SUPPORT
**Sequence generation**	LOW	Information provided by manufacturer (available upon request from manufacturer).
**Allocation concealment**	LOW	Information provided by manufacturer (available upon request from manufacturer).
**Blinding participants/parents**	LOW	Information provided by manufacturer (available upon request from manufacturer).
**Blinding therapist**	UNCLEAR	No information.
**Blinding assessor**	UNCLEAR	No information.
**Incomplete data outcome**	UNCLEAR	"The overall study completion rate was high (87.9% [290/330]), and the methylphenidate group had a significantly higher completion rate compared with the atomoxetine group (91.6% [152/166] vs. 84.1% [138/164]; p 0.044). Significantly more atomoxetine- treated patients discontinued because of TEAEs compared with methylphenidate-treated patients (p 0.011)." (Journal article, Pag. 225) but no data on the drop out for lack of efficacy. LOCF.
**Selective reporting**	LOW	Outcomes listed in short CSR reported in the journal article.
**Notes**		E-mail address for corresponding author not valid; no other email addresses found. Manufacturer provided additional information.

Wehmeier 2012, B4Z-SB-LYDV, NCT00546910

ITEM	RATING	SUPPORT
**Sequence generation**	LOW	"computer-generated randomization scheme" (Journal article, pag. 655)
**Allocation concealment**	LOW	"centralized telephone-based system" (Journal article, pag. 655)
**Blinding participants/parents**	LOW	"capsules looking identically to study drug" (Journal article, pag. 654)
**Blinding therapist**	LOW	"capsules looking identically to study drug" (Journal article, pag. 654). "Investigators, patients, and study team were blinded until the database for the entire study was locked." (Journal article, pag. 655)
**Blinding assessor**	LOW	"capsules looking identically to study drug" (Journal article, pag. 654). "Investigators, patients, and study team were blinded until the database for the entire study was locked." (Journal article, pag. 655)
**Incomplete data outcome**	UNCLEAR	"Of 135 patients initially assessed, 128 patients were randomly assigned to treatment. A total of 125 patients received at least 1 dose of study drug (ATX, n = 63; placebo, n = 62; FAS). Of these patients, 105 patients (84.0%) completed the study. The most common reasons for early discontinuation were lack of efficacy (9.6%), followed by AEs (4.0%), patient decision (1.6%), and physician decision (0.8%). The

		retention rate was slightly lower in the placebo group (82.3%) than in the ATX group (85.7%). There were 4 patients with protocol violations leading to the exclusion from the per protocol analysis (ATX, n = 1; placebo, n = 3)." (Journal article, pag. 255) but data not presented on reason for drop out in each arm.
**Selective reporting**	LOW	All outcomes listed in the protocol and Clinicaltrials.gov reported in the journal article
**Notes**		Manufacturer provided study protocol.

Weisler 2006, SLI381-303

ITEM	RATING	SUPPORT
**Sequence generation**	LOW	Information from CSR, section 9.4.6 Blinding pag. 21/22 (available upon request from the manufacturer)
**Allocation concealment**	LOW	Information from CSR, section 9.4.3 Method of Assigning Subjects to Treatment Groups, pag. 21(available upon request from the manufacturer)
**Blinding participants/parents**	LOW	Information from CSR section 9.4.6 Blinding, pag. 21/22 (available upon request from the manufacturer)
**Blinding therapist**	LOW	Information from CSR section 9.4.6 Blinding, pag. 21/22 (available upon request from the manufacturer)
**Blinding assessor**	LOW	Information from CSR section 9.4.6 Blinding, pag. 21/22 (available upon request from the manufacturer)
**Incomplete data outcome**	HIGH	Unbalanced drop out for lack of efficacy: PBO: 14/64; MAS XR 20 mg/day: 5/66; MAS XR 40 mg/day: 6/64; MAS XR 60 mg/day: 4/61 (Journal article, pag. 632)
**Selective reporting**	LOW	Outcomes listed in protocol but not reported in the Journal article: HAM-A, HAM-D, SAS-SR, Q-LES-Q, Smoking history/habits, all of no interest for the present meta-analysis (Information provided by manufacturer
**Notes**		Manufacturer provided additional data.

Weisler 2012, NCT00880217

ITEM	RATING	SUPPORT
**Sequence generation**	LOW	Computer-generated (Journal article, pag. 424)
**Allocation concealment**	LOW	"interactive voice response system" (Journal article, pag. 424)
**Blinding participants/parents**	LOW	"The blinding of the study medication was achieved by over-encapsulation, with all study drugs provided as opaque hard-gelatin capsules that were identical in shape, size, and appearance." (Journal article, pag. 424).
**Blinding therapist**	LOW	Information provided by manufacturer (available upon request from manufacturer)

**Blinding assessor**	LOW	Information provided by manufacturer (available upon request from manufacturer)
**Incomplete data outcome**	LOW	ITT (MMR). Drop out for lack of efficacy balanced across arms (Journal article, fig. 1)
**Selective reporting**	LOW	All outcomes listed in clinicaltrials.gov reported in the journal article.
**Notes**		Manufacturer provided additional information.

Weiss 2005, B4Z-MC-LYAW

ITEM	RATING	SUPPORT
**Sequence generation**	LOW	Information from full CSR, pag. 41 (available upon request from manufacturer)
**Allocation concealment**	LOW	"Assignment to treatment groups was accomplished at each study site by an interactive voice response system via telephone (i.e., centralized randomization system)". (Journal article, Pag. 649).
**Blinding participants/parents**	LOW	"Patients assigned to placebo were given study medication identical in appearance to atomoxetine." (Journal article, pag. 649).<br>Information from full CSR, pag. 41 (available upon request from manufacturer)
**Blinding therapist**	LOW	"Patients assigned to placebo were given study medication identical in appearance to atomoxetine." (Journal article, pag. 649).<br>Information from full CSR, pag. 41 (available upon request from manufacturer)
**Blinding assessor**	LOW	"Patients assigned to placebo were given study medication identical in appearance to atomoxetine." (Journal article, pag. 649).<br>Information from full CSR, pag. 41 (available upon request from manufacturer)
**Incomplete data outcome**	LOW	LOCF (100/101 in ATMX arm and 51/52 in PBO arm); balanced drop out for lack of efficacy: ATMX: 5/101; PBO: 2/52 (Journal article, fig.1)
**Selective reporting**	LOW	All outcomes listed in the full CSR (available upon request from manufacturer) reported in the journal article.
**Notes**		Manufacturer provided full CSR.

Wender 2011

ITEM	RATING	SUPPORT
**Sequence generation**	LOW	"Randomization was determined by a random number-table and implemented by the clinic's study facilitator, while staff involved in treatment/evaluation remained blinded to assignment." (Journal article, pag. 38)
**Allocation**	LOW	"Randomization was determined by a random number-table and implemented by the clinic's study

concealment		facilitator, while staff involved in treatment/evaluation remained blinded to assignment." (Journal article, pag. 38)
Blinding participants/parents	UNCLEAR	Not clear how blinding was preserved.
Blinding therapist	UNCLEAR	Not clear how blinding was preserved.
Blinding assessor	UNCLEAR	Not clear how blinding was preserved.
Incomplete data outcome	UNCLEAR	"Participants then entered the double-blind crossover trial; 11 dropped out before completion and 105 completed the double-blind trial". Not clear in which arm/phase the drop out occurred.
Selective reporting	UNCLEAR	No protocol/CSR/CT available.
Notes		Authors not able to provide additional information.

Wietecha 2013, NCT00607919

ITEM	RATING	SUPPORT
Sequence generation	LOW	"computer-generated, random sequence" (Journal article, pag. 606)
Allocation concealment	LOW	"interactive voice response system" (Journal article, pag. 606)
Blinding participants/parents	UNCLEAR	Additional information provided by manufacturer but unclear how blinding was preserved
Blinding therapist	UNCLEAR	Additional information provided by manufacturer but unclear how blinding was preserved
Blinding assessor	UNCLEAR	Additional information provided by manufacturer but unclear how blinding was preserved
Incomplete data outcome	LOW	MMRM. (115/120 for active medication arm; 86/89 PBO). Total drop outs: 16/89 (18%) in PBO and 34/120 (28%) in active medication. Drop outs for lack of efficacy: PBO: 2/89 (2%); 1/120 (0.8%) (active medication). (Journal article, pag. 609)
Selective reporting	LOW	Outcomes listed in Clinicaltrials.gov of interest for the present meta-analysis reported in the Journal article.
Notes		Manufacturer provided additional information.

Wigal 2004

ITEM	RATING	SUPPORT
Sequence generation	UNCLEAR	Not specified
Allocation concealment	UNCLEAR	Not specified

**Blinding participants/parents**	LOW	"Drug (*d*- MPH and *d,l*-MPH) and placebo were identical in appearance." (journal article, pag. 1408)
**Blinding therapist**	UNCLEAR	Not specified
**Blinding assessor**	UNCLEAR	Not specified
**Incomplete data outcome**	UNCLEAR	"Of the 132 patients who entered the double-blind phase, 44 patients were randomized to *d*-MPH, 46 to *d,l*-MPH, and 42 to placebo. Thirteen patients (10%) discontinued the study prematurely, and 119 patients (90%) completed the 4-week, double-blind treatment phase. Discontinuations in each group occurred primarily during the final 2 weeks of the study. The most common reasons for study discontinuation were protocol violations such as lack of compliance, missing appointments, parent uncooperative in filling out ratings and giving medications, or Teacher SNAP never being performed (two patients each in the *d*-MPH and placebo groups and one patient in the *d,l*-MPH group); AEs (two patients each in the *d,l*-MPH and placebo groups); and lost to follow-up (three patients in the *d,l*-MPH group). Two patients in the placebo group reported therapeutic failure as the reason for discontinuation, with one of these patients also reporting AEs." (Journal article, pag. 140). "Analyses of all efficacy parameters were performed on the intent-to-treat sample, which included those patients who received medication, had a baseline efficacy evaluation, and had at least one postbaseline efficacy evaluation." (Journal article, pag. 1408)
**Selective reporting**	UNCLEAR	No protocol/CSR/Clinicaltrials.gov available.
**Notes**		Not possible to retrieve information from authors; no additional useful information in the Focalin medical review (Focalin medical review study 97-M-02). Not possible to gather additonal information from manufacturer.

Wigal 2005, SLI381-404, NCT00506727

ITEM	RATING	SUPPORT
**Sequence generation**	UNCLEAR	Additional information from CSR, Section 5.4.4.1 Description of randomization pag. 13/14 (available upon request from manufacturer), but not clear how sequence was generated.
**Allocation concealment**	LOW	Information from CSR, Section 5.4.4.1 Description of randomization pag. 13/14. (available upon request from manufacturer)
**Blinding participants/parents**	LOW	"All study drug was provided in numbered medication cards as a single-capsule formulation and was identical in appearance and weight" (Journal article, pag. 278)
**Blinding therapist**	UNCLEAR	No information.
**Blinding assessor**	UNCLEAR	No information.
**Incomplete data outcome**	LOW	"The proportion of subjects who did not complete the study and reasons for study discontinuation were similar between treatment groups, including 6.5% (7/107) subjects in the MAS XR group who withdrew because of adverse events compared with 3.7% (4/108) of the atomoxetine subjects" (Journal article, pag. 280). Balanced drop out (Journal article, Table 2). "The primary efficacy analysis was conducted using the intent-to-treat sample and a two-way ANCOVA model" (Journal article, pag. 279). ITT in 102/107

		participants in the MAS XR arm and 101/108 in the ATMX arm.
**Selective reporting**	UNCLEAR	No information.
**Notes**		Manufacturer provided additional information.

Wigal 2015, NCT01239030

ITEM	RATING	SUPPORT
**Sequence generation**	LOW	"computer-generated randomization" (Journal article, pag. 333).
**Allocation concealment**	UNCLEAR	Not clear how concealment of allocation was assured.
**Blinding participants/parents**	UNCLEAR	Unclear how blinding was preserved.
**Blinding therapist**	UNCLEAR	Unclear how blinding was preserved.
**Blinding assessor**	UNCLEAR	Unclear how blinding was preserved.
**Incomplete data outcome**	LOW	"The safety/intent-to-treat (ITT) population included all patients who took at least one dose of study medication. The efficacy population included all patients who completed the double-blind phase" (Journal article, pag. 334).<br>Not specified in which arm the drop outs were; however, low drop out (Journal article, fig. 1)
**Selective reporting**	LOW	Outcomes listed in Clinicaltrials.gov reported in the Journal article.
**Notes**		Written to first author, who contacted manufacturer, but we have not received the requested information by the manufacturer.

Wilens 2001

ITEM	RATING	SUPPORT
**Sequence generation**	UNCLEAR	Not specified.
**Allocation concealment**	UNCLEAR	Not specified.
**Blinding participants/parents**	LOW	"Weekly supplies of bupropion or placebo were dispensed by the pharmacy in identically appearing 100-mg capsules" (Journal article, pag. 283)
**Blinding therapist**	UNCLEAR	Not specified.
**Blinding assessor**	UNCLEAR	Not specified.

**Incomplete data outcome**	LOW	"Thirty-eight subjects completed the protocol; two subjects dropped out because of noncompliance (both receiving bupropion)." (Journal article, pag. 284)
**Selective reporting**	UNCLEAR	No protocol/CSR/Clinicaltrials.gov available.
**Notes**		First author suggested to contact manufacturer. No reply from manufacturer.

Wilens 2005, NCT00048360

ITEM	RATING	SUPPORT
**Sequence generation**	UNCLEAR	No information.
**Allocation concealment**	UNCLEAR	No information.
**Blinding participants/parents**	LOW	"..placebo tablets were identical in appearance.." (Journal article, pag. 794)
**Blinding therapist**	UNCLEAR	No information.
**Blinding assessor**	UNCLEAR	No information.
**Incomplete data outcome**	UNCLEAR	LOCF; "During the course of the study, 16 (20%) of the bupropion XL group and 13 (16%) of the placebo group withdrew prematurely for the following reasons: 8 withdrew consent (5 bupropion XL, 3 placebo); 4 for AEs (all bupropion XL group); 8 were lost to follow-up (3 bupropion XL, 5 placebo); 2 for protocol violations (1 from each group); and 7 for other reasons (3 for lack of efficacy [1 bupropion XL, 2 placebo], 2 for noncompliance [1 bupropion XL, 1 placebo], 1 for taking an excluded medication [placebo], and 1 for appropriate rater not available [bupropion XL]). The remaining 133 (82%) subjects (65 bupropion XL, 68 placebo) completed the 8 weeks of treatment." (Journal article, pag. 795). LOCF
**Selective reporting**	LOW	Outcomes listed in Clinicaltrials.gov reported in the journal article.
**Notes**		Author contacted, suggested to contact manufacturer. Attempt to contact manufacturer not successful.

Wilens 2008, B4Z-MC-LYBY, NCT00190957

ITEM	RATING	SUPPORT
**Sequence generation**	LOW	Information from full CSR, pag. 123 (available upon request from manufacturer)
**Allocation concealment**	LOW	Information from full CSR, pag. 123 (available upon request from manufacturer)
**Blinding participants/parents**	LOW	Information from full CSR, pag. 35 (available upon request from manufacturer)
**Blinding therapist**	LOW	Information from full CSR, pag. 35 and 124 (available upon request from manufacturer)

**Blinding assessor**	LOW	Information from full CSR, pag. 35 and 124 (available upon request from manufacturer)
**Incomplete data outcome**	UNCLEAR	Drop out: ATMX: 35/72; PBO: 25/75; drop out for lack of efficacy ATMX: 2/75; PBO: 0/72. LOCF. (Journal article, fig.1, pag. 148)
**Selective reporting**	UNCLEAR	One of the secondary outcomes (WRAADDS), a possible alternative outcome for the present meta-analysis, listed in Clinicaltrials.gov not reported in the journal article
**Notes**		Manufacturer provided full CSR.

<u>Wilens 2011, NCT00528697</u>

ITEM	RATING	SUPPORT
**Sequence generation**	UNCLEAR	No information.
**Allocation concealment**	LOW	“a central interactive voice response system (IVRS) was used” (Journal article, pag. 75).
**Blinding participants/parents**	LOW	“study drug and placebo were identical in appearance.” (Journal article, pag. 75).
**Blinding therapist**	LOW	“The sponsor, investigative sites, and subject were blinded to each subject’s treatment” (Journal article, pag. 75); “study drug and placebo were identical in appearance.” (Journal article, pag. 75).
**Blinding assessor**	LOW	“The sponsor, investigative sites, and subject were blinded to each subject’s treatment” (Journal article, pag. 75).; “study drug and placebo were identical in appearance.” (Journal article, pag. 75).
**Incomplete data outcome**	LOW	Balanced drop out ,including drop out for lack efficacy, in study 1 (PBO and ATMX arms, the arms considered for this meta-analysis. ITT on 45/46 (PBO) and 49/50 (ATMX) (Journal article, fig. 1)
**Selective reporting**	UNCLEAR	Only missing outcome in the Journal article (among those of potential interest for the present meta-analysis): teachers rating (secondary outcome of the study according to CT) but not clear if these refer to ADHD core symptoms (and hence of interest for our meta-analysis).
**Notes**		First author contacted to contact manufacturer; attempts to contact manufacturer not successful.

<u>Wilens 2015, SPD503-312, EUCTR2011-002221-21, NCT01081132</u>

ITEM	RATING	SUPPORT
**Sequence generation**	UNCLEAR	Unclear how sequence was generated
**Allocation concealment**	LOW	Randomization via “automatic interactive response technology” (Journal article, pag. 917)
**Blinding**	UNCLEAR	Not clear how blinding was preserved

**participants/parents**		
**Blinding therapist**	UNCLEAR	Not clear how blinding was preserved
**Blinding assessor**	UNCLEAR	Not clear how blinding was preserved
**Incomplete data outcome**	UNCLEAR	Drop out for lack of efficacy: PBO: 25/157; GXR: 9/157 (Journal article, fig. 1). Imputation methods not appropriate when unbalanced drop outs for efficacy outcomes
**Selective reporting**	LOW	Outcomes related to the present meta-analysis listed in Clinicaltrials.gov reported in the journal article
**Notes**		Not possible to gather additonal information from study author and manufacturer

Winhusen 2010, NCT00253747

ITEM	RATING	SUPPORT
**Sequence generation**	LOW	Generated via computer. (Journal article, pag. 1682).
**Allocation concealment**	LOW	Sequence centrally generated. (Journal article, pag. 1682).
**Blinding participants/parents**	LOW	Matching placebo. (Journal article, pag. 1681).
**Blinding therapist**	LOW	Therapist was blinded. (Information provided by the first author). Matching placebo. (Journal article, pag. 1681).
**Blinding assessor**	LOW	Assessor was blinded. (Information provided by the first author). Matching placebo. (Journal article, pag. 1681).
**Incomplete data outcome**	LOW	Balanced drop out between the 2 study arms; no drop outs specifically stated for lack of efficacy (Journal article, fig. 2).
**Selective reporting**	LOW	Outcomes listed in Clinicaltrials.gov reported in the journal article
**Notes**		First author provided additional information.

Young 2011, B4Z-US-LYCW, NCT00190775

ITEM	RATING	SUPPORT
**Sequence generation**	LOW	Computer generated (Journal article, pag. 52).
**Allocation concealment**	LOW	"Treatment assignment and drug dispensation, managed by a telephone voice-response/interactive Web-based system, remained blinded until after database lock." (Journal article, pag. 52).
**Blinding participants/parents**	LOW	Information from full CSR, pag. 58 (available upon request from manufacturer)

**Blinding therapist**	LOW	Information from full CSR, pag. 58 (available upon request from manufacturer)
**Blinding assessor**	LOW	Information from full CSR, pag. 58 (available upon request from manufacturer)
**Incomplete data outcome**	UNCLEAR	LOCF; balanced drop out for "lack of efficacy" (ATX: 28/268; PBO: 32/234) but high drop-out (however, it was a 24-week study): PBO: 100/234; 149/268 ATX (Journal article, fig.2, pag.54)
**Selective reporting**	LOW	Outcomes of interest for the present meta-analysis listed in the full CSR (available upon request from manufacturer) reported in the journal article.
**Notes**		Manufacturer provided full CSR.

Tables S12. Results of the pairwise meta-analyses for each of the primary and secondary outcomes closest to 12 weeks in the Main dose analysis, and related heterogeneity

In each table in this section, the bottom left triangle refers to results in children/adolescents and the top right triangle refers to results in adults. Comparisons should be read from left to right and from top to bottom, in a diagonal. For each outcome, the estimate is located at the intersection of the top left treatment and the bottom right treatment in the diagonal for the given pairwise comparison. Significant results are in bold and underlined.

Results of the Main dose analyses, Lisdexamfetamine and other Amphetamines lumped

Efficacy – Mean overall change in ADHD core symptoms, Teachers' Ratings

**Atomoxetine**	-	-	-	-	-
-	**Bupropion**	-	-	-	-
-	-	**Guanfacine**	-	-	-
-	0.50 (-0.40; 1.40)	-	**Methylphenidate**	-	-
-	-	-	-0.08 (-0.59; 0.43)	**Modafinil**	-
**<u>-0.29 (-0.59; -0.00)</u>**	-	-0.63 (-1.33; 0.06)	**<u>-0.83 (-1.05; -0.60)</u>**	**<u>-0.80 (-1.29; -0.32)</u>**	**Placebo**

Mean change in symptoms is reported as a standardized mean difference (SMD) along with 95% confidence intervals. An SMD below 0 favours the medication on the top left vs. the medication on the bottom right in the diagonal. The bottom left triangle refers to results in children/adolescents and the top right triangle to results in adults.

Efficacy – Mean overall change in ADHD core symptoms, Clinician's Ratings

**Amphetamines**	-	-	-	-	-	-	**<u>-0.79 (-0.91; -0.67)</u>**

**-0.32 (-0.60; -0.05)**	**Atomoxetine**	-	-	-	0.03 (-0.30; 0.36)	-	**-0.46 (-0.61; -0.30)**
-	-	**Bupropion**	-	-	-	-	**-0.47 (-0.75; -0.19)**
-	-	-	**Clonidine**	-	-	-	-
-	-	-	-	**Guanfacine**	-	-	-
**-0.32 (-0.47; -0.16)**	**0.25 (0.08; 0.41)**	-0.18 (-0.80; 0.44)	-	-	**Methylphenidate**	-	**-0.49 (-0.62; -0.36)**
-	-	-	-	-	0.13 (-0.38; 0.63)	**Modafinil**	0.16 (-0.10; 0.42)
**-1.04 (-1.32; -0.76)**	**-0.56 (-0.66; -0.45)**	-	**-0.71 (-0.99; -0.42)**	**-0.67 (-0.84; -0.50)**	**-0.83 (-0.98; -0.68)**	**-0.62 (-0.91; -0.33)**	**Placebo**

Mean change in symptoms is reported as a standardized mean difference (SMD) along with 95% confidence intervals. An SMD below 0 favours the medication on the top left vs. the medication on the bottom right in the diagonal. The bottom left triangle refers to results in children/adolescents and the top right triangle to results in adults.

Efficacy – ADHD core symptoms, Parents' Ratings

**Amphetamines**	-	-	-	-	-	-
-	**Atomoxetine**	-	-	-	-	-
-	-	**Bupropion**	-	-	-	-
-	-	-	**Guanfacine**	-	-	-
-	**0.29 (0.11; 0.48)**	**1.07 (0.40; 1.74)**	-	**Methylphenidate**	-	-
-	-	-	-	-	**Modafinil**	-

**-1.07 (-1.36; -0.79)**	**-0.62 (-0.73; -0.50)**	-	-0.23 (-0.90; 0.45)	**-0.83 (-0.95; -0.70)**	**-0.46 (-0.65; -0.27)**	**Placebo**

Mean change in symptoms is reported as a standardized mean difference (SMD) along with 95% confidence intervals. An SMD below 0 favours the medication on the top left vs. the medication on the bottom right in the diagonal. The bottom left triangle refers to results in children/adolescents and the top right triangle to results in adults.

Efficacy – ADHD core symptoms, Self Ratings

**Amphetamines**	-	-	0.18 (-0.49, 0.85)	-	0.15 (-0.44, 0.75)	**-0.54 (-0.79, -0.28)**
-	**Atomoxetine**	-	-	0.09 (-0.24, 0.42)	-	**-0.38 (-0.48, -0.27)**
-	-	**Bupropion**	-	-	-	-0.30 (-0.61, 0.01)
-	-	-	**Guanfacine**	-	-	**-0.77 (-1.46, -0.08)**
-	-	-	-	**Methylphenidate**	-	**-0.42 (-0.54, -0.30)**
-	-	-	-	-	**Modafinil**	-0.43 (-1.38, 0.51)
-	-	-	-	-	-	**Placebo**

Mean change in symptoms is reported as a standardized mean difference (SMD) along with 95% confidence intervals. An SMD below 0 favours the medication on the top left vs. the medication on the bottom right in the diagonal. The bottom left triangle refers to results in children/adolescents and the top right triangle to results in adults.

Tolerability

**Amphetamines**	-	-	-	-	-	-	**3.26 (1.64; 6.48)**
1.81 (0.52; 6.25)	**Atomoxetine**	-	-	-	1.25 (0.41; 3.82)	-	**2.33 (1.09; 5.01)**

-	-	**Bupropion**	-	-	-	-	2.58 (0.34; 19.57)
-	-	-	**Clonidine**	-	-	-	-
-	-	-	-	**Guanfacine**	-	-	-
**1.78 (1.01; 3.12)**	0.90 (0.38; 2.16)	1.00 (0.06; 16.51)	0.29 (0.01; 8.37)	-	**Methylphenidate**	-	**2.51 (1.51; 4.19)**
-	-	-	-	-	1.00 (0.02; 52.00)	**Modafinil**	**4.01 (1.67; 9.66)**
**2.01 (1.14; 3.54)**	1.55 (0.81; 2.97)	1.62 (0.06; 43.25)	**11.16 (1.47; 84.77)**	**3.22 (1.13; 9.22)**	1.32 (0.78; 2.23)	1.29 (0.56; 2.94)	**Placebo**

Discontinuation due to adverse events is reported for each comparison as odds ratio (OR), along with 95% confidence intervals. An OR below 1 favours the medication on the top left vs. the medication on the bottom right in the diagonal. The bottom left triangle refers to results in children/adolescents and the top right triangle to results in adults.

Functioning - Clinical Global Impression (CGI)

**Amphetamines**	-	-	-	-	-	-	**4.85 (3.38; 6.96)**
**5.26 (2.86; 10)**	**Atomoxetine**	-	-	-	-	-	1.95 (0.96; 3.97)
-	-	**Bupropion**	-	-	-	-	**3.87 (1.20; 12.45)**
-	-	-	**Clonidine**	-	-	-	-
-	-	-	-	**Guanfacine**	-	-	-
**1.74 (1.19; 2.55)**	-	-	0.38 (0.13; 1.10)	-	**Methylphenidate**	-	**3.17 (2.10; 4.78)**
-	-	-	-	-	-	**Modafinil**	0.89 (0.51; 1.54)

**7.09 (4.56; 11.03)**	**2.62 (1.51; 4.55)**	-	**3.55 (1.28; 9.84)**	**3.58 (2.59; 4.95)**	**6.06 (4.08; 8.98)**	**3.26 (2.31; 4.60)**	**Placebo**

Improvement in clinical global functioning is reported for each comparison as odds ratio (OR), along with 95% confidence intervals. An OR below 1 favours the medication on the bottom right vs. the medication on the top left in the diagonal. The bottom left triangle refers to results in children/adolescents and the top right triangle to results in adults.

Weight (Kgs)

**Amphetamines**	-	-	-	-	-	-	**-0.60 (-1.16; -0.03)**
-	**Atomoxetine**	-	-	-	**0.52 (0.18; 0.85)**	-	-0.32 (-0.70; 0.05)
-	-	**Bupropion**	-	-	-	-	**-0.78 (-1.10; -0.46)**
-	-	-	**Clonidine**	-	-	-	-
-	-	-	-	**Guanfacine**	-	-	-
**-0.25 (-0.50; -0.01)**	0.04 (-0.26; 0.35)	-	**0.72 (0.20; 1.24)**	-	**Methylphenidate**	-	**-0.77 (-1.15; -0.39)**
-	-	-	-	-	-	**Modafinil**	-
**-0.60 (-1.13; -0.07)**	**-0.88 (-1.22; -0.55)**	-	0.25 (-0.25; 0.76)	0.10 (-0.02; 0.22)	**-0.84 (-1.26; -0.42)**	**-0.92 (-1.09; -0.74)**	**Placebo**

Mean change in body weight is reported as a standardized mean difference (SMD) along with 95% confidence intervals. An SMD below 0 favours the medication on the bottom right vs. the medication on the top left in the diagonal. The bottom left triangle refers to results in children/adolescents and the top right triangle to results in adults.

Systolic Blood Pressure

**Amphetamines**	-	-	-	-	-	-	0.10 (-0.03; 0.24)
-	**Atomoxetine**	-	-	-	-	-	0.11 (-0.00; 0.21)
-	-	**Bupropion**	-	-	-	-	0.27 (-0.46; 1.01)
-	-	-	**Clonidine**	-	-	-	-
-	-	-	-	**Guanfacine**	-	-	-
0.06 (-0.12; 0.25)	0.04 (-0.20; 0.28)	-	0.02 (-0.48; 0.53)	-	**Methylphenidate**	-	**0.18 (0.03; 0.33)**
-	-	-	-	-	-	**Modafinil**	-
0.09 (-0.03; 0.20)	**0.11 (0.01; 0.21)**	-	0.13 (-0.37; 0.63)	**-0.24 (-0.40; -0.08)**	**0.15 (0.05; 0.25)**	0.06 (-0.09; 0.21)	**Placebo**

Mean change in systolic blood pressure is reported as a standardized mean difference (SMD) along with 95% confidence intervals. An SMD above 0 indicates an increase in blood pressure values and favours the medication on the bottom right vs. the medication on the top left in the diagonal. The bottom left triangle refers to results in children/adolescents and the top right triangle to results in adults.

Diastolic Blood Pressure

**Amphetamines**	-	-	-	-	-	-	0.03 (-0.09; 0.15)
-	**Atomoxetine**	-	-	-	-	-	**0.19 (0.08; 0.30)**
-	-	**Bupropion**	-	-	-	-	0.20 (-0.54; 0.93)

-	-	-	**Clonidine**	-	-	-	-
-	-	-	-	**Guanfacine**	-	-	-
-0.01 (-0.14; 0.11)	0.10 (-0.07; 0.26)	-	0.11 (-0.39; 0.62)	-	**Methylphenidate**	-	**0.20 (0.08; 0.32)**
-	-	-	-	-	-	**Modafinil**	-
**0.22 (0.08; 0.36)**	**0.26 (0.15; 0.38)**	-	0.01 (-0.49; 0.51)	**-0.18 (-0.36; -0.00)**	**0.27 (0.17; 0.38)**	-0.03 (-0.18; 0.11)	**Placebo**

Mean change in diastolic blood pressure is reported as a standardized mean difference (SMD) along with 95% confidence intervals. An SMD above 0 indicates an increase in blood pressure values and favours the medication on the bottom right vs. the medication on the top left in the diagonal. The bottom left triangle refers to results in children/adolescents and the top right triangle to results in adults.

Acceptability

**Amphetamines**	-	-	-	-	-	-	**0.68 (0.50; 0.93)**
1.33 (0.57; 3.03)	**Atomoxetine**	-	-	-	1.57 (0.71; 3.46)	-	1.28 (0.82; 2.01)
-	-	**Bupropion**	-	-	-	-	1.12 (0.58; 2.19)
-	-	-	**Clonidine**	-	-	-	-
-	-	-	-	**Guanfacine**	-	-	-
1.04 (0.77; 1.41)	0.83 (0.49; 1.40)	1.50 (0.33; 6.75)	2.33 (0.17; 32.58)	-	**Methylphenidate**	-	1.09 (0.84; 1.41)
-	-	-	-	-	1.56 (0.24; 10)	**Modafinil**	**1.91 (1.10; 3.30)**
0.78 (0.49; 1.24)	0.91 (0.69; 1.20)	0.47 (0.03; 8.46)	0.58 (0.34; 1.00)	0.78 (0.60; 1.01)	**0.66 (0.47; 0.92)**	0.72 (0.44; 1.18)	**Placebo**

Discontinuation due to any reason is reported for each comparison as odds ratio (OR), along with 95% confidence intervals. An OR below 1 favours the medication on the bottom right vs. the medication on the top left in the diagonal. The bottom left triangle refers to results in children/adolescents and the top right triangle to results in adults.

HETEROGENEITY

Abbreviation for Medications: AMPH: Amphetamines; BUP: Bupropion; CLON: Clonidine; GUA: Guanfacine; GXR: Guanfacine Extended Release LDX: Lisdexamfetamine; MODA: Modafinil; MPH: Methylphenidate; PBO: Placebo

Main dose analyses, Lisdexamfetamine and other Amphetamines lumped

Efficacy – Mean overall change in ADHD core symptoms, Children/adolescents, Teachers' Ratings

Comparison	No. of studies	P-value	I^2(%)	τ^2
ATMX vs PBO	3	0.648	0.00	0.0000
MPH vs PBO	5	0.199	33.40	0.0218
MODA vs PBO	4	0.000	85.90	0.2027

Efficacy – Mean overall change in ADHD core symptoms, Children/adolescents, Clinician's Ratings

Comparison	No. of studies	P-value	I^2(%)	τ^2
ATMX vs PBO	21	0.008	47.90	0.0274
MODA vs PBO	5	0.006	72.20	0.0741
GUA vs PBO	7	0.012	63.30	0.0334
MPH vs PBO	9	0.066	45.40	0.0229
AMPH vs PBO	6	0.000	82.90	0.1036
AMPH vs MPH	3	0.242	29.50	0.0057

ATMX vs MPH	3	0.374	0.00	0.0000

Efficacy – Mean overall change in ADHD core symptoms, Adults, Clinician's Ratings

Comparison	No. of studies	P-value	I^2(%)	τ^2
AMPH vs PBO	5	0.647	0.00	0.0000
MPH vs PBO	11	0.011	56.30	0.0259
ATMX vs PBO	11	0.000	76.50	0.0500

Efficacy – ADHD core symptoms, Children, Parents' Ratings

Comparison	No. of studies	P-value	I^2(%)	τ^2
MPH vs PBO	9	0.475	0.00	0.0000
ATMX vs PBO	9	0.84	0.00	0.0000
MODA vs PBO	4	0.169	40.40	0.0156

Efficacy – ADHD core symptoms, Adults, Self-Ratings

Comparison	No. of studies	P-value	I^2(%)	τ^2
AMPH vs PBO	4	0.798	0.00	0.0000
ATMX vs PBO	5	0.915	0.00	0.0000
MPH vs PBO	8	0.082	44.50	0.0132

Tolerability, Children/adolescents

Comparison	No. of studies	P-value	I^2(%)	τ^2
ATMX vs PBO	13	0.9780	0.00	0.0000
MPH vs PBO	22	0.9220	0.00	0.0000
AMPH vs PBO	9	0.5030	0.00	0.0000
MODA vs PBO	6	0.4340	0.00	0.0000
GUA vs PBO	7	0.1120	41.90	0.7755
AMPH vs MPH	6	0.4650	0.00	0.0000
ATMX vs MPH	4	0.9500	0.00	0.0000

Tolerability, Adults

Comparison	No. of studies	P-value	I^2(%)	τ^2
AMPH vs PBO	6	0.8240	0.00	0.0000
MPH vs PBO	12	0.8850	0.00	0.0000
ATMX vs PBO	6	0.1990	31.50	0.2698
BUP vs PBO	3	0.4850	0.00	0.0000

Functioning - Clinical Global Impression (CGI), Children/adolescents

Comparison	No. of studies	P-value	I^2(%)	τ^2
MPH vs PBO	11	0.005	60.10	0.2395
ATMX vs PBO	4	0.145	44.30	0.1359
AMPH vs PBO	8	0.000	74.20	0.2801
MODA vs PBO	4	0.459	0.00	0.0000
BUP vs PBO	6	0.193	32.30	0.0515
AMPH vs MPH	4	0.227	30.80	0.0465

Functioning - Clinical Global Impression (CGI), Adults

Comparison	No. of studies	P-value	I^2(%)	τ^2
AMPH vs PBO	6	0.140	39.90	0.0778
MPH vs PBO	6	0.021	62.50	0.1488

Weight, Children/adolescents

Comparison	No. of studies	P-value	I^2	τ^2
ATMX vs PBO	13	0.0000	91.00	0.3468
MPH vs PBO	12	0.0000	94.30	0.5114
MODA vs PBO	3	0.4670	0.00	0.0000
GUA vs PBO	5	0.4680	0.00	0.0000
AMPH vs PBO	6	0.0000	95.50	0.4230
AMPH vs MPH	3	0.0290	71.80	0.0336
ATMX vs MPH	3	0.1570	45.90	0.0354

Weight, Adults

Comparison	No. of studies	P-value	I^2(%)	τ^2
AMPH vs PBO	6	0.0000	95.00	0.4688
MPH vs PBO	5	0.0000	82.30	0.1486
ATMX vs PBO	4	0.0000	89.50	0.1288

Systolic Blood Pressure, Children/adolescents

Comparison	No. of studies	P-value	I^2(%)	τ^2
ATMX vs PBO	12	0.542	0.00	0.0000
MPH vs PBO	11	0.741	0.00	0.0000
AMPH vs PBO	7	0.163	34.70	0.0085
MODA vs PBO	4	0.825	0.00	0.0000
GUA vs PBO	6	0.128	41.60	0.0160
AMPH vs MPH	3	0.131	50.70	0.0134
ATMX vs MPH	3	0.269	23.90	0.0131

Systolic Blood Pressure, Adults

Comparison	No. of studies	P-value	I^2(%)	τ^2
AMPH vs PBO	6	0.305	16.80	0.0046
MPH vs PBO	6	0.174	35.00	0.0125
ATMX vs PBO	4	0.589	0.00	0.0000

Diastolic Blood Pressure, Children/adolescents

Comparison	No. of studies	P-value	I^2(%)	τ^2
ATMX vs PBO	12	0.2450	20.20	0.0077
MPH vs PBO	11	0.3900	5.60	0.0018
AMPH vs PBO	7	0.0380	54.90	0.0194
MODA vs PBO	4	0.7300	0.00	0.0000
GUA vs PBO	6	0.0580	53.10	0.0255

AMPH vs MPH	3	0.4030	0.00	0.0000
ATMX vs MPH	3	0.9290	0.00	0.0000

Diastolic Blood Pressure, Adults

Comparison	No. of studies	P-value	I^2(%)	τ^2
AMPH vs PBO	6	0.7880	0.00	0.0000
MPH vs PBO	6	0.5500	0.00	0.0000
ATMX vs PBO	4	0.6580	0.00	0.0000

Acceptability, Children/adolescents

Comparison	No. of studies	P-value	I^2(%)	τ^2
ATMX vs PBO	14	0.4330	1.50	0.0042
MPH vs PBO	21	0.0140	45.10	0.2160
AMPH vs PBO	9	0.0010	68.80	0.3093
MODA vs PBO	6	0.1230	42.30	0.1406
GUA vs PBO	8	0.4090	2.70	0.0043
AMPH vs MPH	6	0.8140	0.00	0.0000
ATMX vs MPH	4	0.2790	21.90	0.0680

Acceptability, Adults

Comparison	No. of studies	P-value	I^2(%)	τ^2
AMPH vs PBO	6	0.7230	0.00	0.0000
MPH vs PBO	11	0.9120	0.00	0.0000
ATMX vs PBO	6	0.0610	52.70	0.1501
BUP vs PBO	3	0.4000	0.00	0.0000

Tables S13. Results of the network meta-analyses for each of the primary outcomes closest to 12 weeks in the Main dose analysis

In each table in this section, the bottom left triangle refers to results in children/adolescents and the top right triangle refers to results in adults. Comparisons should be read from left to right and from top to bottom, in a diagonal. For each outcome, the estimate is located at the intersection of the top left treatment and the bottom right treatment in the diagonal for the given pairwise comparison. Significant results are in bold and underlined.

Main dose analyses, Lisdexamfetamine and other Amphetamines lumped

Efficacy – ADHD core symptoms, Teachers' Ratings

**Atomoxetine**	-	-	-	-	-
-0.00 (-0.90,0.90)	**Bupropion**	-	-	-	-
0.31 (-0.79,1.42)	0.31 (-0.92,1.55)	**Guanfacine**	-	-	-
0.50 (-0.11,1.10)	0.50 (-0.17,1.17)	0.18 (-0.86,1.22)	**Methylphenidate**	-	-
0.44 (-0.19,1.07)	0.44 (-0.38,1.26)	0.12 (-0.93,1.18)	-0.06 (-0.53,0.42)	**Modafinil**	-
-0.32 (-0.82,0.18)	-0.32 (-1.07,0.43)	-0.63 (-1.62,0.35)	**-0.82 (-1.16,-0.48)**	**-0.76 (-1.15,-0.37)**	**Placebo**

Mean change in symptoms is reported as a standardized mean difference (SMD) along with 95% confidence intervals. An SMD below 0 favours the medication on the top left vs. the medication on the bottom right in the diagonal. The bottom left triangle refers to results in children/adolescents and the top right triangle to results in adults.

Efficacy – ADHD core symptoms, Clinicians' Ratings

**Amphetamines**	**-0.34<br>(-0.58, -0.10)**	-0.33<br>(-0.77, 0.11)	-	-	**-0.29<br>(-0.54, -0.05)**	**-0.94 (-1.43, -<br>0.46)**	**-0.79<br>(-0.99, -0.58)**
**-0.46<br>(-0.65, -0.27)**	**Atomoxetine**	0.01<br>(-0.41, 0.42)	-	-	0.04<br>(-0.14, 0.23)	**-0.61<br>(-1.06, -0.15)**	**-0.45<br>(-0.58, -0.32)**

-0.06 (-0.81, 0.68)	0.40 (-0.34, 1.14)	**Bupropion**	-	-	0.04 (-0.38, 0.45)	**-0.62 (-1.20, -0.03)**	**-0.46 (-0.85, -0.07)**
-0.31 (-0.81, 0.18)	0.15 (-0.33, 0.63)	-0.25 (-1.12, 0.62)	**Clonidine**	-	-	-	-
**-0.35 (-0.59, -0.10)**	0.11 (-0.09, 0.32)	-0.28 (-1.04, 0.47)	-0.03 (-0.53, 0.46)	**Guanfacine**	-	-	-
**-0.24 (-0.44, -0.05)**	**0.22 (0.05, 0.39)**	-0.18 (-0.90, 0.54)	0.07 (-0.42, 0.56)	0.11 (-0.13, 0.34)	**Methylphenidate**	**-0.65 (-1.11, -0.19)**	**-0.49 (-0.64, -0.35)**
**-0.39 (-0.67, -0.12)**	0.07 (-0.17, 0.31)	-0.33 (-1.10, 0.43)	-0.08 (-0.59, 0.43)	-0.05 (-0.32, 0.23)	-0.15 (-0.41, 0.10)	**Modafinil**	0.16 (-0.28, 0.59)
**-1.02 (-1.19, -0.85)**	**-0.56 (-0.66, -0.45)**	**-0.96 (-1.69, -0.22)**	**-0.71 (-1.17, -0.24)**	**-0.67 (-0.85, -0.50)**	**-0.78 (-0.93, -0.62)**	**-0.62 (-0.84, -0.41)**	**Placebo**

Mean change in symptoms is reported as a standardized mean difference (SMD) along with 95% confidence intervals. An SMD below 0 favours the medication on the top left vs. the medication on the bottom right in the diagonal. The bottom left triangle refers to results in children/adolescents and the top right triangle to results in adults.

Tolerability

**Amphetamines**	1.40 (0.54,3.66)	1.28 (0.14,11.40)	-	-	1.36 (0.54,3.43)	0.81 (0.23,2.93)	**3.26 (1.54,6.92)**
1.54 (0.79,3.01)	**Atomoxetine**	0.91 (0.11,7.77)	-	-	0.97 (0.47,2.02)	0.58 (0.18,1.93)	**2.33 (1.28,4.25)**
1.53 (0.17,13.88)	0.99 (0.11,9.15)	**Bupropion**	-	-	1.07 (0.13,8.92)	0.64 (0.06,6.37)	2.55 (0.33,19.93)
0.51 (0.08,3.27)	0.33 (0.05,2.14)	0.33 (0.02,5.51)	**Clonidine**	-	-	-	-
0.87 (0.35,2.16)	0.57 (0.22,1.47)	0.57 (0.06,5.77)	1.71 (0.24,12.22)	**Guanfacine**	-	-	-
1.60 (0.94,2.73)	1.04 (0.55,1.94)	1.05 (0.12,9.14)	3.14 (0.51,19.33)	1.83 (0.74,4.57)	**Methylphenidate**	0.60 (0.19,1.92)	**2.39 (1.40,4.08)**
1.72 (0.64,4.59)	1.11 (0.40,3.09)	1.12 (0.11,11.62)	3.36 (0.46,24.64)	1.97 (0.63,6.16)	1.07 (0.41,2.83)	**Modafinil**	**4.01 (1.42,11.33)**
**2.30 (1.36,3.89)**	1.49 (0.84,2.64)	1.51 (0.17,13.27)	4.52 (0.75,27.03)	**2.64 (1.20,5.81)**	1.44 (0.90,2.31)	1.34 (0.57,3.18)	**Placebo**

Discontinuation due to adverse events is reported for each comparison as odds ratio (OR), along with 95% confidence intervals. An OR above 1 favours the medication on the bottom right vs. the medication on the top left in the diagonal. The bottom left triangle refers to results in children/adolescents and the top right triangle to results in adults.

Tables S14. NMA heterogeneity, Main dose analysis, primary outcomes

In children/adolescents, the common heterogeneity SD for efficacy (teachers' and clinicians' ratings), and tolerability was 0.355, 0.188, and 0.268, respectively. In adults, the common heterogeneity SD for efficacy rated by clinicians and tolerability was 0.178 and 0.282, respectively.

Analyses in children/adolescents

Main dose analyses, Lisdexamfetamine and other Amphetamines lumped

Model assumption	SD Heterogeneity
**Efficacy, teachers' ratings**	
Consistency	0.355
Inconsistency	0.397
**Efficacy, clinicians' ratings**	
Consistency	0.188
Inconsistency	0.198
**Tolerability**	
Consistency	0.268
Inconsistency	0.348

Analyses in adults

Main dose analyses, Lisdexamfetamine and other Amphetamines lumped

Model assumption	SD Heterogeneity
**Efficacy, clinicians' ratings**	
Consistency	0.178
Inconsistency	0.195
**Tolerability**	
Consistency	0.282
Inconsistency	0.340

Tables S15. Results of the network meta-analyses for each of the secondary outcomes closest to 12 weeks in the Main dose analysis

Main dose analyses, Lisdexamfetamine and other Amphetamines lumped

Efficacy – ADHD core symptoms, Parents' Ratings

**Amphetamines**	-	-	-	-	-	-
**-0.47 (-0.77;-0.17)**	**Atomoxetine**	-	-	-	-	-
**-1.31 (-2.05;-0.58)**	**-0.84 (-1.52;-0.16)**	**Bupropion**	-	-	-	-
**-0.85 (-1.58;-0.12)**	-0.37 (-1.06;0.31)	0.46 (-0.49;1.42)	**Guanfacine**	-	-	-
-0.24 (-0.54;0.07)	**0.23 (0.10;0.37)**	**1.07 (0.40;1.74)**	0.61 (-0.07;1.29)	**Methylphenidate**	-	-
**-0.61 (-0.93;-0.29)**	-0.14 (-0.32;0.04)	0.70 (0.00;1.39)	0.23 (-0.46;0.92)	**-0.38 (-0.56;-0.19)**	**Modafinil**	-
**-1.07 (-1.36;-0.79)**	**-0.60 (-0.71;-0.50)**	0.24 (-0.44;0.92)	-0.23 (-0.90;0.45)	**-0.84 (-0.95;-0.72)**	**-0.46 (-0.61;-0.31)**	**Placebo**

Mean change in symptoms is reported as a standardized mean difference (SMD) along with 95% confidence intervals. An SMD below 0 favours the medication on the top left vs. the medication on the bottom right in the diagonal. The bottom left triangle refers to results in children/adolescents and the top right triangle to results in adults.

Efficacy – ADHD core symptoms, Self-Ratings

**Amphetamines**	-0.08 (-0.35,0.19)	-0.15 (-0.56,0.25)	0.24 (-0.36,0.85)	-0.04 (-0.31,0.22)	-0.31 (-0.63,0.01)	**-0.45 (-0.70,-0.20)**
-	**Atomoxetine**	-0.07 (-0.40,0.26)	0.33 (-0.29,0.94)	0.04 (-0.09,0.17)	-0.23 (-0.48,0.03)	**-0.37 (-0.47,-0.27)**
-	-	**Bupropion**	0.40 (-0.28,1.08)	0.11 (-0.21,0.44)	-0.15 (-0.55,0.24)	-0.30 (-0.61,0.01)
-	-	-	**Guanfacine**	-0.29 (-0.90,0.32)	-0.55 (-1.20,0.09)	**-0.70 (-1.30,-0.09)**

-	-	-	-	**Methylphenidate**	**-0.27 (-0.52,-0.02)**	**-0.41 (-0.50,-0.33)**
-	-	-	-	-	**Modafinil**	-0.15 (-0.38,0.09)
-	-	-	-	-	-	**Placebo**

Mean change in symptoms is reported as a standardized mean difference (SMD) along with 95% confidence intervals. An SMD below 0 favours the medication on the top left vs. the medication on the bottom right in the diagonal. The bottom left triangle refers to results in children/adolescents and the top right triangle to results in adults.

Functioning - Clinical Global Impression (CGI)

**Amphetamines**	2.39 (0.93;6.18)	1.42 (0.55,3.64)	-	-	1.58 (0.91,2.75)	**5.46 (2.17;13.77)**	**4.86 (3.30;7.17)**
**3.39 (1.95;5.88)**	**Atomoxetine**	0.60 (0.18,2.00)	-	-	0.66 (0.26;1.69)	2.28 (0.68;7.64)	2.03 (0.85;4.84)
-	-	**Bupropion**	-	-	1.11 (0.44,2.81)	**3.85 (1.16,12.84)**	**3.43 (1.45,8.14)**
2.77 (0.87;8.83)	0.82 (0.24;2.78)	-	**Clonidine**	-	-	-	-
**2.13 (1.24;3.66)**	0.63 (0.32;1.22)	-	0.77 (0.23;2.56)	**Guanfacine**	-	-	-
1.38 (0.92;2.07)	**0.41 (0.23;0.74)**	-	0.50 (0.16;1.54)	0.65 (0.38;1.12)	**Methylphenidate**	**3.46 (1.36;8.81)**	**3.08 (2.04;4.65)**
**2.40 (1.29;4.46)**	0.71 (0.34;1.47)	-	0.86 (0.25;2.95)	1.13 (0.57;2.22)	1.73 (0.93;3.23)	**Modafinil**	0.89 (0.38;2.04)
**7.71 (5.52;10.77)**	**2.28 (1.38;3.76)**	-	2.78 (0.91;8.53)	**3.63 (2.36;5.57)**	**5.57 (3.99;7.79)**	**3.22 (1.91;5.43)**	**Placebo**

Improvement in clinical global functioning is reported for each comparison as odds ratio (OR), along with 95% confidence intervals. An OR above 1 favours the medication on the top left vs. the medication on the bottom right in the diagonal. The bottom left triangle refers to results in children/adolescents and the top right triangle to results in adults.

Weight (Kgs)

**Amphetamines**	-0.31 (-0.96;0.34)	0.18 (-0.93;1.29)	-	-	0.14 (-0.49;0.77)	-	**-0.60 (-1.03;-0.18)**
0.13 (-0.40;0.67)	**Atomoxetine**	0.49 (-0.65;1.63)	-	-	0.45 (-0.18;1.07)	-	-0.29 (-0.78;0.20)

-	-	**Bupropion**	-	-	-0.04 (-1.17;1.09)	-	-0.78 (-1.81;0.25)
-0.81 (-1.95;0.33)	-0.95 (-2.05;0.16)	-	**Clonidine**	-	-	-	-
**-0.80 (-1.48;-0.13)**	**-0.94 (-1.54;-0.33)**	-	0.01 (-1.17;1.19)	**Guanfacine**	-	-	-
0.06 (-0.43;0.55)	-0.07 (-0.49;0.35)	-	0.87 (-0.19;1.94)	**0.86 (0.26;1.47)**	**Methylphenidate**	-	**-0.74 (-1.20;-0.28)**
0.22 (-0.58;1.02)	0.09 (-0.65;0.82)	-	1.03 (-0.22;2.29)	**1.02 (0.19;1.86)**	0.16 (-0.58;0.90)	**Modafinil**	-
**-0.71 (-1.15;-0.27)**	**-0.84 (-1.16;-0.52)**	-	0.10 (-0.96;1.17)	0.09 (-0.42;0.60)	**-0.77 (-1.09;-0.45)**	**-0.93 (-1.59;-0.26)**	**Placebo**

Mean change in body weight is reported as a standardized mean difference (SMD) along with 95% confidence intervals. An SMD below 0 favours the medication on the bottom right vs. the medication on the top left in the diagonal. The bottom left triangle refers to results in children/adolescents and the top right triangle to results in adults.

Systolic Blood Pressure

**Amphetamines**	-0.00 (-0.18,0.17)	-0.17 (-0.92,0.59)	-0.07 (-0.25,0.11)	-	0.11 (-0.02,0.23)
-0.03 (-0.16; 0.10)	**Atomoxetine**	-0.16 (-0.91,0.59)	-0.07 (-0.24,0.11)	-	0.11 (-0.01,0.23)
-	-	**Bupropion**	0.10 (-0.66,0.85)	-	0.27 (-0.47,1.01)
0.00 (-0.11; 0.11)	0.03 (-0.10; 0.15)	-	**Methylphenidate**	-	**0.17 (0.05,0.30)**
0.03 (-0.14; 0.21)	0.06 (-0.12; 0.24)	-	0.03 (-0.15; 0.21)	**Modafinil**	-
**0.09 (0.00; 0.18)**	**0.12 (0.02; 0.22)**	-	0.09 (-0.01; 0.19)	0.06 (-0.09; 0.21)	**Placebo**

Mean change in systolic blood pressure is reported as a standardized mean difference (SMD) along with 95% confidence intervals. An SMD above 0 indicates an increase in blood pressure values and favours the medication on the bottom right vs. the medication on the top left in the diagonal. The bottom left triangle refers to results in children/adolescents and the top right triangle to results in adults.

Diastolic Blood Pressure

**Amphetamines**	**-0.16 (-0.32,-0.00)**	-0.17 (-0.91,0.58)	**-0.17 (-0.34,-0.00)**	-	0.03 (-0.09,0.15)
-0.06 (-0.19,0.07)	**Atomoxetine**	-0.00 (-0.75,0.74)	-0.01 (-0.17,0.15)	-	**0.19 (0.08,0.30)**
-	-	**Bupropion**	-0.00 (-0.75,0.74)	-	0.20 (-0.54,0.93)
-0.02 (-0.13,0.08)	0.04 (-0.08,0.16)	-	**Methylphenidate**	-	**0.20 (0.08,0.32)**
**0.24 (0.07,0.42)**	**0.31 (0.13,0.48)**	-	**0.27 (0.09,0.45)**	**Modafinil**	-
**0.21 (0.12,0.31)**	**0.28 (0.18,0.37)**	-	**0.24 (0.14,0.33)**	-0.03 (-0.18,0.12)	**Placebo**

Mean change in diastolic blood pressure is reported as a standardized mean difference (SMD) along with 95% confidence intervals. An SMD above 0 indicates an increase in blood pressure values and favours the medication on the bottom right vs. the medication on the top left in the diagonal. The bottom left triangle refers to results in children/adolescents and the [illegible]

Acceptability

**Amphetamines**	0.53 (0.35,0.83)	0.61 (0.29,1.31)	-	-	0.65 (0.42,0.99)	0.36 (0.18,0.70)	**0.68 (0.49,0.95)**
0.91 (0.60,1.40)	**Atomoxetine**	1.14 (0.55,2.39)	-	-	1.21 (0.83,1.76)	0.67 (0.35,1.29)	1.28 (0.97,1.70)
0.87 (0.20,3.75)	0.96 (0.22,4.11)	**Bupropion**	-	-	1.06 (0.51,2.21)	0.59 (0.24,1.45)	1.12 (0.57,2.22)
1.31 (0.53,3.20)	1.43 (0.59,3.49)	1.50 (0.29,7.83)	**Clonidine**	-	-	-	-
0.96 (0.57,1.62)	1.05 (0.62,1.77)	1.10 (0.25,4.86)	0.73 (0.29,1.85)	**Guanfacine**	-	-	-
1.13 (0.78,1.62)	1.23 (0.84,1.80)	1.29 (0.31,5.32)	0.86 (0.36,2.05)	1.18 (0.72,1.93)	**Methylphenidate**	0.55 (0.29,1.05)	1.06 (0.81,1.38)
1.11 (0.63,1.98)	1.22 (0.69,2.17)	1.27 (0.28,5.76)	0.85 (0.33,2.23)	1.16 (0.62,2.18)	0.99 (0.57,1.70)	**Modafinil**	**1.91 (1.06,3.42)**
0.78 (0.56,1.09)	0.85 (0.61,1.18)	0.89 (0.21,3.74)	0.60 (0.26,1.37)	0.81 (0.54,1.23)	**0.69 (0.52,0.91)**	0.70 (0.43,1.13)	**Placebo**

Discontinuation due to any reason is reported for each comparison as odds ratio (OR), along with 95% confidence intervals. An OR below 1 favours the medication on the top left vs. the medication on the bottom right in the diagonal. The bottom left triangle refers to results in children/adolescents and the top right triangle to results in adults.

Tables S16. Sensitivity analyses, primary outcomes for the Main dose analysis, outcomes closest to 12 weeks. ***In each table, the bottom left triangle refers to results in children/adolescents and the top right triangle refers to results in adults***

Excluding trials lasting < 2 weeks

Efficacy – ADHD core symptoms, Teachers' Ratings

The analysis is not reported since results are the same as those for the Main dose analysis, total number of studies

Efficacy – ADHD core symptoms, Clinicians' Ratings

**Amphetamines**	-	-	-	-	-	-	-
**-0.47 (-0.66,-0.27)**	**Atomoxetine**	-	-	-	-	-	-
-0.05 (-0.80,0.70)	0.41 (-0.33,1.16)	**Bupropion**	-	-	-	-	-
-0.32 (-0.82,0.18)	0.15 (-0.33,0.63)	-0.27 (-1.15,0.61)	**Clonidine**	-	-	-	-
**-0.35 (-0.60,-0.11)**	0.11 (-0.09,0.32)	-0.30 (-1.07,0.46)	-0.03 (-0.54,0.47)	**Guanfacine**	-	-	-
**-0.23 (-0.43,-0.03)**	**0.24 (0.06,0.42)**	-0.18 (-0.90,0.55)	0.09 (-0.41,0.59)	0.12 (-0.12,0.36)	**Methylphenidate**	-	-
**-0.40 (-0.67,-0.12)**	0.07 (-0.17,0.31)	-0.34 (-1.12,0.43)	-0.08 (-0.60,0.44)	-0.04 (-0.32,0.24)	-0.17 (-0.43,0.09)	**Modafinil**	-
**-1.02 (-1.20,-0.85)**	**-0.56 (-0.67,-0.45)**	**-0.97 (-1.72,-0.23)**	**-0.71 (-1.18,-0.24)**	**-0.67 (-0.85,-0.50)**	**-0.79 (-0.96,-0.63)**	**-0.63 (-0.85,-0.41)**	**Placebo**

Mean change in symptoms is reported as a standardized mean difference (SMD) along with 95% confidence intervals. An SMD below 0 favours the medication on the top left vs. the medication on the bottom right in the diagonal. *Note: The analysis on adults is not reported since results are the same as those for the Main dose analysis, total number of studies in adults.*

Tolerability

**Amphetamines**	-	-	-	-	-	-	-
1.53 (0.78,2.99)	**Atomoxetine**	-	-	-	-	-	-
1.50	0.99	**Bupropion**	-	-	-	-	-

(0.16,13.75)	(0.11,9.17)						
0.50 (0.08,3.26)	0.33 (0.05,2.16)	0.34 (0.02,5.56)	**Clonidine**	-	-	-	-
0.85 (0.34,2.14)	0.56 (0.21,1.47)	0.57 (0.06,5.75)	1.69 (0.24,12.19)	**Guanfacine**	-	-	-
1.58 (0.91,2.73)	1.04 (0.55,1.95)	1.05 (0.12,9.18)	3.13 (0.51,19.43)	1.85 (0.74,4.65)	**Methylphenidate**	-	-
1.68 (0.62,4.55)	1.10 (0.40,3.09)	1.12 (0.11,11.61)	3.34 (0.45,24.64)	1.97 (0.63,6.21)	1.07 (0.40,2.84)	**Modafinil**	-
**2.27 (1.33,3.88)**	1.49 (0.84,2.64)	1.51 (0.17,13.30)	4.50 (0.75,27.08)	**2.66 (1.20,5.87)**	1.44 (0.89,2.33)	1.35 (0.57,3.21)	**Placebo**

Discontinuation due to adverse events is reported for each comparison as odds ratio (OR), along with 95% confidence intervals. An OR above 1 favours the medication on the bottom right vs. the medication on the top left in the diagonal. Note: results of the analysis on adults is not reported since results are the same as those at 12 weeks.

Excluding trials lasting < 3 weeks

Efficacy – ADHD core symptoms, Teachers' Ratings

The analysis is not reported since results are the same as those for the Main dose analysis at 12 weeks.

Efficacy – ADHD core symptoms, Clinician's Ratings

**Amphetamines**	**-0.34 (-0.57,-0.11)**	-0.33 (-0.75,0.10)	**-0.33 (-0.56,-0.09)**	**-0.94 (-1.40,-0.49)**	**-0.79 (-0.98,-0.60)**
-	**Atomoxetine**	0.01 (-0.38,0.41)	0.01 (-0.16,0.19)	**-0.61 (-1.04,-0.17)**	**-0.45 (-0.57,-0.32)**
-	-	**Bupropion**	0.00 (-0.40,0.40)	**-0.62 (-1.18,-0.06)**	**-0.46 (-0.84,-0.08)**
-	-	-	**Methylphenidate**	**-0.62 (-1.05,-0.18)**	**-0.46 (-0.60,-0.32)**
-	-	-	-	**Modafinil**	0.16 (-0.26,0.57)
-	-	-	-	-	**Placebo**

Mean change in symptoms is reported as a standardized mean difference (SMD) along with 95% confidence intervals. An SMD below 0 favours the medication on the top left vs. the medication on the bottom right in the diagonal. Note: results of the analysis on children is not reported since results are the same as those at 12 weeks.

Tolerability

**Amphetamines**	1.47 (0.55,3.92)	1.34 (0.15,12.05)	-	-	1.45 (0.56,3.75)	0.85 (0.23,3.14)	**3.41 (1.58,7.38)**
1.51 (0.77,2.98)	**Atomoxetine**	0.91 (0.11,7.79)	-	-	0.99 (0.47,2.08)	0.58 (0.17,1.96)	**2.32 (1.27,4.26)**
1.49 (0.16,13.61)	0.98 (0.11,9.15)	**Bupropion**	-	-	1.09 (0.13,9.16)	0.64 (0.06,6.42)	2.55 (0.33,19.97)
0.50 (0.08,3.25)	0.33 (0.05,2.17)	0.34 (0.02,5.61)	**Clonidine**	-	-	-	-
0.85 (0.34,2.14)	0.56 (0.21,1.47)	0.57 (0.06,5.79)	1.69 (0.23,12.22)	**Guanfacine**	-	-	-
1.55 (0.89,2.71)	1.03 (0.54,1.94)	1.04 (0.12,9.14)	3.10 (0.50,19.31)	1.83 (0.73,4.63)	**Methylphenidate**	0.58 (0.18,1.92)	**2.35 (1.35,4.06)**
1.67 (0.62,4.53)	1.10 (0.39,3.10)	1.12 (0.11,11.70)	3.34 (0.45,24.73)	1.97 (0.62,6.24)	1.08 (0.40,2.88)	**Modafinil**	**4.01 (1.40,11.51)**
**2.26 (1.32,3.87)**	1.49 (0.84,2.65)	1.52 (0.17,13.42)	4.51 (0.75,27.22)	**2.67 (1.20,5.91)**	1.45 (0.89,2.37)	1.35 (0.57,3.23)	**Placebo**

Discontinuation due to adverse events is reported for each comparison as odds ratio (OR), along with 95% confidence intervals. An OR below 1 favours the medication on the top left vs. the medication on the bottom right in the diagonal. The bottom left triangle refers to results in children/adolescents and the top right triangle to results in adults.

Excluding trials with overall high or unclear risk of bias

Efficacy – ADHD core symptoms, 12 weeks, Teachers' Ratings

No NMA as only 1 study per comparison

Efficacy – ADHD core symptoms, Clinician's Ratings

**Amphetamines**	**-0.33 (-0.63,-0.02)**	-	**-0.37 (-0.67,-0.08)**	**-0.81 (-1.09,-0.54)**
-	**Atomoxetine**	-	-0.05 (-0.20,0.11)	**-0.48 (-0.61,-0.36)**
-	0.25 (-0.08,0.57)	**Guanfacine**	-	**-**
-	0.23 (-0.07,0.53)	-0.02 (-0.44,0.41)	**Methylphenidate**	**-0.44 (-0.54,-0.34)**
-	**-0.47 (-0.61,-0.33)**	**-0.72 (-1.01,-0.43)**	**-0.70 (-1.01,-0.39)**	**Placebo**

Mean change in symptoms is reported as a standardized mean difference (SMD) along with 95% confidence intervals. An SMD below 0

favours the medication on the top left vs. the medication on the bottom right in the diagonal. The bottom left triangle refers to results in children/adolescents and the top right triangle to results in adults.

Tolerability

**Amphetamines**	1.05 (0.10,10.62)	-	1.40 (0.17,11.65)	3.80 (0.50,28.78)
2.34 (0.33,16.47)	**Atomoxetine**	-	1.34 (0.47,3.79)	**3.63 (1.18,11.20)**
0.83 (0.07,9.41)	0.36 (0.06,2.28)	**Guanfacine**	-	-
2.27 (0.42,12.15)	0.97 (0.32,2.98)	2.73 (0.41,18.12)	**Methylphenidate**	**2.72 (1.45,5.07)**
3.62 (0.54,24.35)	1.55 (0.52,4.63)	4.36 (0.97,19.60)	1.59 (0.51,5.03)	**Placebo**

Discontinuation due to adverse events is reported for each comparison as odds ratio (OR), along with 95% confidence intervals. An OR below 1 favours the medication on the top left vs. the medication on the bottom right in the diagonal. The bottom left triangle refers to results in children/adolescents and the top right triangle to results in adults.

Excluding trials with overall high risk of bias

Efficacy – ADHD core symptoms, Teachers' Ratings

**Atomoxetine**	-	-	-	-	-
0.03 (-1.00,1.05)	**Bupropion**	-	-	-	-
0.31 (-0.92,1.54)	0.28 (-1.09,1.66)	**Guanfacine**	-	-	-
0.52 (-0.17,1.22)	0.50 (-0.25,1.25)	0.21 (-0.95,1.37)	**Methylphenidate**	-	-
0.66 (-0.17,1.49)	0.64 (-0.35,1.62)	0.35 (-0.89,1.60)	0.14 (-0.51,0.78)	**Modafinil**	-
-0.32 (-0.90,0.25)	-0.35 (-1.20,0.50)	-0.63 (-1.72,0.45)	**-0.85 (-1.25,-0.45)**	**-0.99 (-1.59,-0.38)**	**Placebo**

Mean change in symptoms is reported as a standardized mean difference (SMD) along with 95% confidence intervals. An SMD below 0 favours the medication on the top left vs. the medication on the bottom right in the diagonal. The bottom left triangle refers to results in children/adolescents and the top right triangle to results in adults.

Efficacy – ADHD core symptoms, Clinician's Ratings

**Amphetamines**	**-0.49 (-0.69,-0.29)**	**-0.40 (-0.74,-0.05)**	-	-	**-0.40 (-0.60,-0.20)**	**-1.02 (-1.36,-0.68)**	**-0.86 (-1.04,-0.69)**
**-0.39 (-0.58,-0.19)**	**Atomoxetine**	0.09 (-0.22,0.41)	-	-	0.09 (-0.04,0.22)	**-0.53 (-0.84,-0.22)**	**-0.37 (-0.46,-0.28)**
0.00 (-0.74,0.75)	0.39 (-0.34,1.13)	**Bupropion**	-	-	-0.00 (-0.32,0.31)	**-0.62 (-1.04,-0.20)**	**-0.47 (-0.77,-0.17)**
-0.23 (-0.72,0.25)	0.15 (-0.31,0.62)	-0.24 (-1.10,0.62)	**Clonidine**	-	-	-	-
**-0.27 (-0.53,-0.02)**	0.12 (-0.10,0.33)	-0.28 (-1.03,0.48)	-0.04 (-0.53,0.45)	**Guanfacine**	-	-	-
-0.17 (-0.38,0.04)	**0.22 (0.04,0.39)**	-0.18 (-0.89,0.54)	0.06 (-0.42,0.54)	0.10 (-0.14,0.34)	**Methylphenidate**	**-0.62 (-0.93,-0.31)**	**-0.47 (-0.56,-0.37)**
-0.09 (-0.47,0.30)	0.30 (-0.06,0.66)	-0.09 (-0.89,0.71)	0.15 (-0.42,0.72)	0.19 (-0.21,0.58)	0.09 (-0.27,0.44)	**Modafinil**	0.16 (-0.14,0.45)
**-0.94 (-1.12,-0.76)**	**-0.55 (-0.66,-0.45)**	**-0.94 (-1.68,-0.21)**	**-0.71 (-1.16,-0.25)**	**-0.67 (-0.85,-0.48)**	**-0.77 (-0.93,-0.61)**	**-0.85 (-1.20,-0.51)**	**Placebo**

Mean change in symptoms is reported as a standardized mean difference (SMD) along with 95% confidence intervals. An SMD below 0 favours the medication on the top left vs. the medication on the bottom right in the diagonal. The bottom left triangle refers to results in children/adolescents and the top right triangle to results in adults.

Tolerability

**Amphetamines**	1.33 (0.33,5.39)	0.99 (0.09,10.49)	-	-	1.12 (0.31,4.09)	0.63 (0.13,3.03)	2.51 (0.78,8.06)
1.59 (0.81,3.13)	**Atomoxetine**	0.74 (0.08,6.71)	-	-	0.85 (0.37,1.95)	0.47 (0.13,1.75)	1.89 (0.88,4.07)
1.55 (0.17,14.16)	0.98 (0.11,9.02)	**Bupropion**	-	-	1.14 (0.13,9.62)	0.64 (0.06,6.45)	2.55 (0.33,19.99)
0.22 (0.03,1.86)	0.14 (0.02,1.18)	0.14 (0.01,2.81)	**Clonidine**	-	-	-	-
1.10 (0.42,2.86)	0.69 (0.26,1.86)	0.71 (0.07,7.21)	5.09 (0.54,47.80)	**Guanfacine**	-	-	-
1.60 (0.91,2.81)	1.01 (0.54,1.89)	1.03 (0.12,8.94)	7.41 (0.87,62.83)	1.46 (0.56,3.81)	**Methylphenidate**	0.56 (0.17,1.85)	**2.24 (1.29,3.89)**
0.95 (0.16,5.76)	0.60	0.61 (0.04,9.67)	4.38 (0.29,65.58)	0.86 (0.13,5.88)	0.59 (0.10,3.48)	**Modafinil**	**4.01**

	(0.10,3.65)						**(1.39,11.59)**
**2.41 (1.38,4.20)**	1.52 (0.86,2.68)	1.55 (0.18,13.57)	**11.16 (1.39,89.39)**	2.19 (0.96,5.03)	1.51 (0.92,2.45)	2.55 (0.45,14.37)	**Placebo**

Discontinuation due to adverse events is reported for each comparison as odds ratio (OR), along with 95% confidence intervals. An OR above 1 favours the medication on the bottom right vs. the medication on the top left in the diagonal. The bottom left triangle refers to results in children/adolescents and the top right triangle to results in adults.

Excluding trials where all participants have psychiatric/neurological comorbidities

Efficacy – ADHD core symptoms, Teachers' Ratings

**Bupropion**	-	-	-
0.50 (-0.24,1.24)	**Methylphenidate**	-	-
0.47 (-0.45,1.38)	-0.03 (-0.57,0.50)	**Modafinil**	-
-0.32 (-1.16,0.52)	**-0.82 (-1.21,-0.43)**	**-0.78 (-1.22,-0.35)**	**Placebo**

Mean change in symptoms is reported as a standardized mean difference (SMD) along with 95% confidence intervals. An SMD below 0 favours the medication on the top left vs. the medication on the bottom right in the diagonal.

Efficacy – ADHD core symptoms, Clinician's Ratings

**Amphetamines**	**-0.34 (-0.58,-0.09)**	-0.33 (-0.77,0.11)	-	-	**-0.33 (-0.57,-0.08)**	**-0.94 (-1.42,-0.47)**	**-0.79 (-0.99,-0.59)**
**-0.47 (-0.68,-0.26)**	**Atomoxetine**	0.01 (-0.41,0.43)	-	-	0.01 (-0.18,0.21)	**-0.61 (-1.06,-0.15)**	**-0.45 (-0.59,-0.31)**
-0.06 (-0.84,0.71)	0.41 (-0.36,1.18)	**Bupropion**	-	-	0.00 (-0.41,0.42)	**-0.62 (-1.20,-0.03)**	**-0.46 (-0.85,-0.07)**
-0.31 (-0.84,0.22)	0.16 (-0.36,0.67)	-0.25 (-1.16,0.66)	**Clonidine**	-	-	-	-
**-0.38 (-0.65,-0.11)**	0.09 (-0.15,0.33)	-0.32 (-1.11,0.47)	-0.07 (-0.61,0.47)	**Guanfacine**	-	-	-
**-0.24 (-0.45,-0.03)**	**0.23 (0.04,0.42)**	-0.18 (-0.92,0.57)	0.07 (-0.45,0.60)	0.14 (-0.12,0.40)	**Methylphenidate**	**-0.62 (-1.08,-0.16)**	**-0.46 (-0.60,-0.32)**
**-0.38 (-0.67,-0.09)**	0.09 (-0.18,0.35)	-0.32 (-1.11,0.47)	-0.07 (-0.62,0.48)	-0.00 (-0.31,0.30)	-0.14 (-0.41,0.13)	**Modafinil**	0.16 (-0.28,0.59)

**-1.02 (-1.20,-0.84)**	**-0.55 (-0.68,-0.42)**	**-0.96 (-1.72,-0.19)**	**-0.71 (-1.20,-0.21)**	**-0.64 (-0.84,-0.44)**	**-0.78 (-0.94,-0.61)**	**-0.63 (-0.86,-0.40)**	**Placebo**

Mean change in symptoms is reported as a standardized mean difference (SMD) along with 95% confidence intervals. An SMD below 0 favours the medication on the top left vs. the medication on the bottom right in the diagonal. The bottom left triangle refers to results in children/adolescents and the top right triangle to results in adults.

Tolerability

**Amphetamines**	1.47 (0.53,4.06)	1.28 (0.14,11.57)	-	-	1.35 (0.52,3.47)	0.81 (0.22,3.05)	**3.26 (1.52,7.02)**
1.76 (0.89,3.49)	**Atomoxetine**	0.87 (0.10,7.62)	-	-	0.92 (0.42,2.01)	0.55 (0.16,1.97)	**2.22 (1.14,4.33)**
1.49 (0.17,13.36)	0.84 (0.09,7.79)	**Bupropion**	-	-	1.05 (0.12,8.91)	0.64 (0.06,6.51)	2.55 (0.32,20.02)
0.19 (0.02,1.60)	0.11 (0.01,0.93)	0.13 (0.01,2.55)	**Clonidine**	-	-	-	-
0.99 (0.39,2.48)	0.56 (0.20,1.53)	0.66 (0.07,6.70)	5.17 (0.56,47.69)	**Guanfacine**	-	-	-
1.62 (0.96,2.74)	0.92 (0.49,1.74)	1.09 (0.13,9.42)	**8.52 (1.02,70.78)**	1.65 (0.64,4.22)	**Methylphenidate**	0.60 (0.18,2.02)	**2.42 (1.39,4.19)**
1.61 (0.62,4.22)	0.92 (0.33,2.57)	1.09 (0.11,11.06)	8.48 (0.91,78.96)	1.64 (0.52,5.17)	1.00 (0.38,2.59)	**Modafinil**	**4.01 (1.37,11.78)**
**2.13 (1.27,3.57)**	1.21 (0.66,2.22)	1.43 (0.16,12.44)	**11.16 (1.41,88.03)**	2.16 (0.95,4.89)	1.31 (0.82,2.09)	1.32 (0.57,3.06)	**Placebo**

Discontinuation due to adverse events is reported for each comparison as odds ratio (OR), along with 95% confidence intervals. An OR above 1 favours the medication on the bottom right vs. the medication on the top left in the diagonal. The bottom left triangle refers to results in children/adolescents and the top right triangle to results in adults.

Excluding cross-over trials

Efficacy – ADHD core symptoms, Teachers' Ratings

The analysis is not reported since results are the same as those for the Main dose analysis, total number of studies

Efficacy – ADHD core symptoms, Clinician's Ratings

**Amphetamines**	**-0.40 (-0.56,-0.25)**	**-0.32 (-0.64,-0.00)**	**-0.34 (-0.51,-0.18)**	**-0.95 (-1.26,-0.64)**	**-0.79 (-0.92,-0.66)**

-	**Atomoxetine**	0.08 (-0.22,0.38)	0.06 (-0.06,0.18)	**-0.54 (-0.84,-0.25)**	**-0.39 (-0.47,-0.31)**
-	-	**Bupropion**	-0.02 (-0.33,0.28)	**-0.63 (-1.03,-0.22)**	**-0.47 (-0.76,-0.18)**
-	-	-	**Methylphenidate**	**-0.60 (-0.90,-0.31)**	**-0.44 (-0.54,-0.35)**
-	-	-	-	**Modafinil**	0.16 (-0.12,0.44)
-	-	-	-	-	**Placebo**

Mean change in symptoms is reported as a standardized mean difference (SMD) along with 95% confidence intervals. An SMD below 0 favours the medication on the top left vs. the medication on the bottom right in the diagonal. *The analysis on children/adolescents is not reported since results are the same as those for the Main dose analysis, total number of studies*

Tolerability

**Amphetamines**	1.48 (0.54,4.07)	1.34 (0.15,12.28)	-	-	1.43 (0.53,3.82)	0.85 (0.22,3.30)	**3.42 (1.55,7.53)**
1.52 (0.75,3.08)	**Atomoxetine**	0.91 (0.10,7.90)	-	-	0.97 (0.44,2.11)	0.58 (0.16,2.05)	**2.31 (1.22,4.37)**
1.49 (0.16,13.80)	0.98 (0.10,9.27)	**Bupropion**	-	-	1.06 (0.12,9.12)	0.63 (0.06,6.58)	2.54 (0.32,20.08)
0.51 (0.08,3.36)	0.34 (0.05,2.24)	0.34 (0.02,5.77)	**Clonidine**	-	-	-	-
0.85 (0.33,2.19)	0.56 (0.21,1.50)	0.57 (0.06,5.86)	1.67 (0.23,12.25)	**Guanfacine**	-	-	-
1.54 (0.87,2.74)	1.01 (0.51,2.01)	1.03 (0.12,9.10)	3.02 (0.48,19.13)	1.81 (0.70,4.68)	**Methylphenidate**	0.60 (0.17,2.07)	**2.39 (1.33,4.30)**
1.69 (0.61,4.65)	1.11 (0.39,3.17)	1.13 (0.11,11.90)	3.30 (0.44,24.92)	1.98 (0.62,6.36)	1.09 (0.40,3.00)	**Modafinil**	**4.01 (1.34,12.03)**
**2.31 (1.33,4.01)**	1.51 (0.83,2.75)	1.54 (0.17,13.76)	4.51 (0.74,27.62)	**2.71 (1.21,6.06)**	1.50 (0.89,2.52)	1.37 (0.56,3.31)	**Placebo**

Discontinuation due to adverse events is reported for each comparison as odds ratio (OR), along with 95% confidence intervals. An OR below 1 favours the medication on the top left vs. the medication on the bottom right in the diagonal. The bottom left triangle refers to results in children/adolescents and the top right triangle to results in adults.

Excluding trials for which imputation of missing data was required

Efficacy – ADHD core symptoms, Teachers' Ratings

**Atomoxetine**	-	-	-	-	-
0.09 (-0.83,1.02)	**Bupropion**	-	-	-	-
0.31 (-0.80,1.43)	0.22 (-1.04,1.48)	**Guanfacine**	-	-	-
0.59 (-0.04,1.22)	0.50 (-0.18,1.17)	0.28 (-0.78,1.34)	**Methylphenidate**	-	-
0.58 (-0.10,1.26)	0.49 (-0.37,1.35)	0.27 (-0.82,1.36)	-0.01 (-0.53,0.52)	**Modafinil**	-
-0.32 (-0.83,0.19)	-0.42 (-1.19,0.36)	-0.63 (-1.63,0.36)	**-0.91 (-1.29,-0.54)**	**-0.90 (-1.36,-0.45)**	**Placebo**

Mean change in symptoms is reported as a standardized mean difference (SMD) along with 95% confidence intervals. An SMD below 0 favours the medication on the top left vs. the medication on the bottom right in the diagonal.

Efficacy – ADHD core symptoms, Clinician's Ratings

**Amphetamines**	**-0.43 (-0.58,-0.27)**	-0.39 (-1.04,0.25)	-	-	**-0.33 (-0.49,-0.17)**	-	**-0.79 (-0.92,-0.66)**
**-0.46 (-0.65,-0.26)**	**Atomoxetine**	0.03 (-0.60,0.67)	-	-	0.10 (-0.02,0.22)	-	**-0.36 (-0.45,-0.28)**
-0.07 (-0.82,0.69)	0.39 (-0.36,1.14)	**Bupropion**	-	-	0.07 (-0.57,0.71)	-	-0.40 (-1.03,0.24)
-0.31 (-0.82,0.20)	0.14 (-0.34,0.63)	-0.24 (-1.13,0.64)	**Clonidine**	-	-	-	-
**-0.29 (-0.56,-0.03)**	0.16 (-0.06,0.39)	-0.23 (-1.00,0.55)	0.02 (-0.50,0.53)	**Guanfacine**	-	-	-
**-0.25 (-0.45,-0.04)**	**0.21 (0.03,0.40)**	-0.18 (-0.91,0.55)	0.07 (-0.44,0.57)	0.05 (-0.21,0.30)	**Methylphenidate**	-	**-0.46 (-0.55,-0.37)**
**-0.35 (-0.65,-0.06)**	0.10 (-0.17,0.37)	-0.29 (-1.07,0.50)	-0.04 (-0.58,0.50)	-0.06 (-0.38,0.26)	-0.11 (-0.39,0.17)	**Modafinil**	
**-1.02 (-1.19,-0.84)**	**-0.56 (-0.67,-0.45)**	**-0.95 (-1.70,-0.20)**	**-0.71 (-1.18,-0.23)**	**-0.72 (-0.92,-0.53)**	**-0.77 (-0.94,-0.61)**	**-0.66 (-0.91,-0.42)**	**Placebo**

Mean change in symptoms is reported as a standardized mean difference (SMD) along with 95% confidence intervals. An SMD below 0 favours the medication on the top left vs. the medication on the bottom right in the diagonal. The bottom left triangle refers to results in children/adolescents and the top right triangle to results in adults.

Tolerability

No NMA as no studies available.

Excluding trials where all participants have IQ< 70

This analysis was not performed since only one study included all participants with IQ < 70.

Studies sponsored by pharmaceutical manufacturers

(Note: only 4 studies in children and adolescents and 2 in adults were not sponsored, so the subgroup analysis on non sponsored studies was not performed)

Efficacy – ADHD core symptoms, Clinician's Ratings

**Amphetamines**	**-0.34 (-0.56,-0.13)**	-0.33 (-0.73,0.08)	-	-	**-0.36 (-0.58,-0.14)**	**-0.95 (-1.37,-0.52)**	**-0.79 (-0.97,-0.61)**
**-0.47 (-0.64,-0.29)**	**Atomoxetine**	0.02 (-0.36,0.40)	-	-	-0.02 (-0.18,0.15)	**-0.60 (-1.00,-0.20)**	**-0.44 (-0.56,-0.33)**
-	-	**Bupropion**	-	-	-0.03 (-0.42,0.35)	**-0.62 (-1.15,-0.09)**	**-0.46 (-0.82,-0.10)**
-0.32 (-0.77,0.13)	0.15 (-0.28,0.58)	-	**Clonidine**	-	-	-	-
**-0.41 (-0.64,-0.18)**	0.06 (-0.14,0.25)	-	-0.09 (-0.55,0.36)	**Guanfacine**	-	-	-
**-0.23 (-0.41,-0.06)**	**0.23 (0.07,0.40)**	-	0.08 (-0.36,0.53)	0.18 (-0.04,0.40)	**Methylphenidate**	**-0.59 (-0.99,-0.18)**	**-0.43 (-0.56,-0.30)**
**-0.55 (-0.81,-0.28)**	-0.08 (-0.32,0.16)	-	-0.23 (-0.70,0.25)	-0.13 (-0.41,0.14)	**-0.31 (-0.57,-0.05)**	**Modafinil**	0.16 (-0.23,0.54)
**-1.02 (-1.18,-0.87)**	**-0.56 (-0.65,-0.46)**	-	**-0.71 (-1.13,-0.28)**	**-0.61 (-0.78,-0.44)**	**-0.79 (-0.93,-0.65)**	**-0.48 (-0.69,-0.26)**	**Placebo**

Mean change in symptoms is reported as a standardized mean difference (SMD) along with 95% confidence intervals. An SMD below 0 favours the medication on the top left vs. the medication on the bottom right in the diagonal. The bottom left triangle refers to results in children/adolescents and the top right triangle to results in adults.

Efficacy – ADHD core symptoms, Teachers' Ratings

**Atomoxetine**	-	-	-
**0.54 (0.20,0.89)**	**Methylphenidate**	-	-
0.20 (-0.15,0.54)	**-0.35 (-0.60,-0.10)**	**Modafinil**	-
**-0.29 (-0.59,-0.00)**	**-0.84 (-1.02,-0.66)**	**-0.49 (-0.67,-0.31)**	**Placebo**

Mean change in symptoms is reported as a standardized mean difference (SMD) along with 95% confidence intervals. An SMD below 0 favours the medication on the top left vs. the medication on the bottom right in the diagonal. The bottom left triangle refers to results in children/adolescents and the top right triangle to results in adults.

Tolerability

**Amphetamines**	1.36 (0.50,3.67)	1.28 (0.14,11.59)	-	-	1.31 (0.50,3.43)	0.81 (0.22,3.07)	**3.26 (1.51,7.04)**
1.54 (0.79,3.01)	**Atomoxetine**	0.94 (0.11,8.16)	-	-	0.96 (0.45,2.08)	0.60 (0.17,2.09)	**2.40 (1.28,4.50)**
1.53 (0.17,13.88)	0.99 (0.11,9.15)	**Bupropion**	-	-	1.02 (0.12,8.72)	0.63 (0.06,6.52)	2.55 (0.32,20.04)
0.51 (0.08,3.27)	0.33 (0.05,2.14)	0.33 (0.02,5.51)	**Clonidine**	-	-	-	-
0.87 (0.35,2.16)	0.57 (0.22,1.47)	0.57 (0.06,5.77)	1.71 (0.24,12.22)	**Guanfacine**	-	-	-
1.60 (0.94,2.73)	1.04 (0.55,1.94)	1.05 (0.12,9.14)	3.14 (0.51,19.33)	1.83 (0.74,4.57)	**Methylphenidate**	0.62 (0.18,2.12)	**2.49 (1.40,4.44)**
1.72 (0.64,4.59)	1.11 (0.40,3.09)	1.12 (0.11,11.62)	3.36 (0.46,24.64)	1.97 (0.63,6.16)	1.07 (0.41,2.83)	**Modafinil**	**4.01 (1.36,11.83)**
**2.30 (1.36,3.89)**	1.49 (0.84,2.64)	1.51 (0.17,13.27)	4.52 (0.75,27.03)	**2.64 (1.20,5.81)**	1.44 (0.90,2.31)	1.34 (0.57,3.18)	**Placebo**

Discontinuation due to adverse events is reported for each comparison as odds ratio (OR), along with 95% confidence intervals. An OR below 1 favours the medication on the top left vs. the medication on the bottom right in the diagonal. The bottom left triangle refers to results in children/adolescents and the top right triangle to results in adults.

Excluding studies with unfair dose comparisons

Efficacy – ADHD core symptoms, Teachers' Ratings

**Atomoxetine**	-	-	-	-	-
0.44 (-0.62,1.51)	**Bupropion**	-	-	-	-
0.32 (-0.74,1.37)	-0.13 (-1.47,1.21)	**Guanfacine**	-	-	-
0.50 (-0.08,1.07)	0.05 (-0.84,0.94)	0.18 (-0.81,1.18)	**Methylphenidate**	-	-
0.43 (-0.17,1.03)	-0.02 (-1.02,0.98)	0.11 (-0.90,1.13)	-0.07 (-0.52,0.38)	**Modafinil**	-
-0.32 (-0.80,0.16)	-0.76 (-1.71,0.18)	-0.63 (-1.58,0.31)	**-0.82 (-1.14,-0.49)**	**-0.75 (-1.12,-0.38)**	**Placebo**

Mean change in symptoms is reported as a standardized mean difference (SMD) along with 95% confidence intervals. An SMD below 0 favours the medication on the top left vs. the medication on the bottom right in the diagonal.

Efficacy – ADHD core symptoms, Clinicians' Ratings

**Amphetamines**	-	-	-	-	-	-	-
**-0.39 (-0.57,-0.20)**	**Atomoxetine**	-	-	-	-	-	-
-0.01 (-0.73,0.72)	0.38 (-0.34,1.10)	**Bupropion**	-	-	-	-	-
-0.23 (-0.69,0.23)	0.16 (-0.28,0.60)	-0.22 (-1.05,0.61)	**Clonidine**	-	-	-	-
**-0.27 (-0.50,-0.03)**	0.12 (-0.07,0.31)	-0.26 (-0.99,0.48)	-0.04 (-0.49,0.42)	**Guanfacine**	-	-	-
-0.19 (-0.38,0.01)	**0.20 (0.03,0.37)**	-0.18 (-0.88,0.52)	0.04 (-0.41,0.49)	0.08 (-0.14,0.30)	**Methylphenidate**	-	-
**-0.33 (-0.58,-0.07)**	0.06 (-0.16,0.28)	-0.32 (-1.05,0.42)	-0.10 (-0.57,0.38)	-0.06 (-0.32,0.20)	-0.14 (-0.38,0.10)	**Modafinil**	-

**-0.93 (-1.10,-0.77)**	**-0.55 (-0.65,-0.45)**	**-0.93 (-1.64,-0.21)**	**-0.71 (-1.13,-0.28)**	**-0.67 (-0.83,-0.51)**	**-0.75 (-0.90,-0.60)**	**-0.61 (-0.81,-0.41)**	**Placebo**

Mean change in symptoms is reported as a standardized mean difference (SMD) along with 95% confidence intervals. An SMD below 0 favours the medication on the top left vs. the medication on the bottom right in the diagonal. *Note: the results of the analysis on adults is not reported since no studies were excluded and results are the same as those for the Main dose analysis.*

Tolerability

**Amphetamines**	-	-	-	-	-	-	-
1.62 (0.81,3.25)	**Atomoxetine**	-	-	-	-	-	-
1.57 (0.11,21.54)	0.97 (0.07,13.41)	**Bupropion**	-	-	-	-	-
0.55 (0.08,3.58)	0.34 (0.05,2.22)	0.35 (0.02,7.98)	**Clonidine**	-	-	-	-
0.94 (0.36,2.40)	0.58 (0.22,1.53)	0.60 (0.04,8.81)	1.71 (0.24,12.44)	**Guanfacine**	-	-	-
1.60 (0.90,2.85)	0.99 (0.52,1.87)	1.02 (0.08,13.47)	2.93 (0.47,18.33)	1.71 (0.67,4.35)	**Methylphenidate**	-	-
1.84 (0.67,5.09)	1.14 (0.40,3.21)	1.17 (0.08,17.73)	3.37 (0.45,25.15)	1.97 (0.62,6.27)	1.15 (0.43,3.10)	**Modafinil**	-
**2.51 (1.42,4.43)**	1.55 (0.87,2.76)	1.60 (0.12,20.93)	4.59 (0.76,27.86)	**2.68 (1.21,5.96)**	1.57 (0.96,2.57)	1.36 (0.57,3.27)	**Placebo**

Discontinuation due to adverse events is reported for each comparison as odds ratio (OR), along with 95% confidence intervals. An OR below 1 favours the medication on the top left vs. the medication on the bottom right in the diagonal. *Note: The analysis on adults is not reported since no studies were excluded and results are the same as those for the Main dose analysis.*

Excluding studies recruiting participants resistant to ADHD medications

N/A for lack of information/data (see Appendix Table 7 in this supplement)

Excluding studies recruiting only non treatment naïve participants

N/A for lack of information/data (see Appendix Table 7 in this supplement)

Tables S17. Results of the post-hoc analyses for each of the primary and secondary outcomes closest to 12 weeks.

PAIRWISE META-ANALYSES

Results of the FDA dose analyses, Lisdexamfetamine and other Amphetamines lumped

Efficacy – ADHD core symptoms, Teachers' Ratings

**Atomoxetine**	-	-
-	**Methylphenidate**	-
**-0.29 (-0.59; -0.00)**	**-0.83 (-1.05; -0.60)**	**Placebo**

Mean change in symptoms is reported as a standardized mean difference (SMD) along with 95% confidence intervals. An SMD below 0 favours the medication on the top left vs. the medication on the bottom right in the diagonal.

Efficacy – ADHD core symptoms, Clinician's Ratings

**Amphetamines**	-	-	-	-	**-0.80 (-0.98; -0.62)**
**-0.32 (-0.60; -0.05)**	**Atomoxetine**	-	-	0.03 (-0.30; 0.36)	**-0.46 (-0.61; -0.30)**
-	-	**Clonidine**	-	-	-
-	-	-	**Guanfacine**	-	-
**-0.32 (-0.47; -0.16)**	**0.25 (0.08; 0.41)**	-	-	**Methylphenidate**	**-0.49 (-0.62; -0.36)**
**-1.04 (-1.32; -0.76)**	**-0.56 (-0.66; -0.45)**	**-0.71 (-0.99; -0.42)**	**-0.61 (-0.75; -0.46)**	**-0.83 (-0.98; -0.68)**	**Placebo**

Mean change in symptoms is reported as a standardized mean difference (SMD) along with 95% confidence intervals. An SMD below 0 favours the medication on the top left vs. the medication on the bottom right in the diagonal. The bottom left triangle refers to results in children/adolescents and the top right triangle to results in adults.

Efficacy – ADHD core symptoms, Parents' Ratings

**Amphetamines**	-	-	-
-	**Atomoxetine**	-	-
-	**0.29 (0.11; 0.48)**	**Methylphenidate**	-
**-1.07 (-1.36; -0.79)**	**-0.62 (-0.73; -0.51)**	**-0.83 (-0.96; -0.70)**	**Placebo**

Mean change in symptoms is reported as a standardized mean difference (SMD) along with 95% confidence intervals. An SMD below 0 favours the medication on the top left vs. the medication on the bottom right in the diagonal.

Efficacy – ADHD core symptoms, Self Ratings

**Amphetamines**	-	-	**-0.45 (-0.82, -0.09)**
-	**Atomoxetine**	0.09 (-0.24, 0.42)	**-0.38 (-0.48, -0.27)**
-	-	**Methylphenidate**	**-0.42 (-0.54, -0.30)**
-	-	-	**Placebo**

Mean change in symptoms is reported as a standardized mean difference (SMD) along with 95% confidence intervals. An SMD below 0 favours the medication on the top left vs. the medication on the bottom right in the diagonal.

Tolerability

**Amphetamines**	-	-	-	-	**3.44 (1.29; 9.18)**
1.82 (0.52; 6.25)	**Atomoxetine**	-	-	1.25 (0.41; 3.82)	**2.33 (1.09; 5.01)**
-	-	**Clonidine**	-	-	-
-	-	-	**Guanfacine**	-	-
**1.78 (1.01; 3.12)**	0.90 (0.38; 2.16)	-	-	**Methylphenidate**	**2.51 (1.51; 4.19)**
**2.01 (1.14; 3.54)**	1.55 (0.81; 2.97)	**11.16 (1.47; 84.77)**	**3.68 (1.20; 11.29)**	1.32 (0.78; 2.23)	**Placebo**

Discontinuation due to adverse events is reported for each comparison as odds ratio (OR), along with 95% confidence intervals. An OR below 1 favours the medication on the top left vs. the medication on the bottom right in the diagonal. The bottom left triangle refers to results in children/adolescents and the top right triangle to results in adults.

Functioning - Clinical Global Impression (CGI)

**Amphetamines**	-	-	-	**4.08 (2.47; 6.73)**
**5.26 (2.86; 10)**	**Atomoxetine**	-	-	1.95 (0.96; 3.97)
-	-	**Guanfacine**	-	-
**1.74 (1.19; 2.55)**	-	-	**Methylphenidate**	**3.17 (2.10; 4.78)**
**7.09 (4.56; 11.03)**	**2.62 (1.51; 4.55)**	**3.50 (2.63; 4.65)**	**6.06 (4.08; 8.98)**	**Placebo**

Improvement in clinical global functioning is reported for each comparison as odds ratio (OR), along with 95% confidence intervals. An OR below 1 favours the medication on the bottom right vs. the medication on the top left in the diagonal. The bottom left triangle refers to results in children/adolescents and the top right triangle to results in adults.

Weight (Kgs)

**Amphetamines**	-	-	-	-0.10 (-0.28; 0.08)
-	**Atomoxetine**	-	**0.52 (0.18; 0.85)**	-0.32 (-0.70; 0.05)
-	-	**Guanfacine**	-	-
**-0.25 (-0.50; -0.01)**	0.04 (-0.26; 0.35)	-	**Methylphenidate**	**-0.77 (-1.15; -0.39)**
**-0.60 (-1.13; -0.07)**	**-0.88 (-1.22; -0.55)**	0.10 (-0.02; 0.22)	**-0.83 (-1.26; -0.41)**	**Placebo**

Mean change in body weight is reported as a standardized mean difference (SMD) along with 95% confidence intervals. An SMD below 0 favours the medication on the bottom right vs. the medication on the top left in the diagonal. The bottom left triangle refers to results in children/adolescents and the top right triangle to results in adults.

Systolic Blood Pressure

**Amphetamines**	-	-	-	0.10 (-0.20; 0.41)
-	**Atomoxetine**	-	-	0.11 (-0.00; 0.21)
-	-	**Guanfacine**	-	-
0.06 (-0.12; 0.25)	0.04 (-0.20; 0.28)	-	**Methylphenidate**	**0.18 (0.03; 0.33)**
0.09 (-0.03; 0.20)	**0.11 (0.01; 0.21)**	**-0.25 (-0.42; -0.08)**	**0.15 (0.05; 0.25)**	**Placebo**

Mean change in systolic blood pressure is reported as a standardized mean difference (SMD) along with 95% confidence intervals. An SMD above 0 indicates an increase in blood pressure values and favours the medication on the bottom right vs. the medication on the top left in the diagonal. The bottom left triangle refers to results in children/adolescents and the top right triangle to results in adults.

Diastolic Blood Pressure

**Amphetamines**	-	-	-	-0.05 (-0.23; 0.13)
-	**Atomoxetine**	-	-	**0.19 (0.08; 0.30)**
-	-	**Guanfacine**	-	-
-0.01 (-0.14; 0.11)	0.10 (-0.07; 0.26)	-	**Methylphenidate**	**0.20 (0.08; 0.32)**
**0.22 (0.08; 0.36)**	**0.26 (0.15; 0.38)**	**-0.22 (-0.39; -0.06)**	**0.27 (0.17; 0.38)**	**Placebo**

Mean change in diastolic blood pressure is reported as a standardized mean difference (SMD) along with 95% confidence intervals. An SMD above 0 indicates an increase in blood pressure values and favours the medication on the bottom right vs. the medication on the top left in the diagonal. The bottom left triangle refers to results in children/adolescents and the top right triangle to results in adults.

Acceptability

**Amphetamines**	-	-	-	-	0.76 (0.51; 1.14)
1.33 (0.57; 3.03)	**Atomoxetine**	-	-	1.57 (0.71; 3.46)	1.28 (0.82; 2.01)
-	-	**Clonidine**	-	-	-
-	-	-	**Guanfacine**	-	-
1.04 (0.77; 1.41)	0.83 (0.49; 1.40)	-	-	**Methylphenidate**	1.09 (0.84; 1.41)
0.78 (0.49; 1.24)	0.91 (0.69; 1.20)	0.61 (0.35; 1.06)	0.77 (0.58; 1.01)	**0.66 (0.47; 0.92)**	**Placebo**

Discontinuation due to any reason is reported for each comparison as odds ratio (OR), along with 95% confidence intervals. An OR below 1 favours the medication on the bottom right vs. the medication on the top left in the diagonal. The bottom left triangle refers to results in children/adolescents and the top right triangle to results in adults.

Results of the Inclusive dose analyses, Lisdexamfetamine and other Amphetamines lumped

Efficacy – ADHD core symptoms, Teachers' Ratings

**Atomoxetine**	-	-	-	-	-
-	**Bupropion**	-	-	-	-
-	-	**Guanfacine**	-	-	-
-	0.50 (-0.40; 1.40)	-	**Methylphenidate**	-	-
-	-	-	-0.08 (-0.59; 0.43)	**Modafinil**	-
**-0.39 (-0.59; -0.19)**	-	-0.64 (-1.33; 0.06)	**-0.75 (-0.98; -0.51)**	**-0.80 (-1.29; -0.32)**	**Placebo**

Mean change in symptoms is reported as a standardized mean difference (SMD) along with 95% confidence intervals. An SMD below 0 favours the medication on the top left vs. the medication on the bottom right in the diagonal. The bottom left triangle refers to results in children/adolescents and the top right triangle to results in adults.

Efficacy – ADHD core symptoms, Clinician's Ratings

**Amphetamines**	-	-	-	-	-	-	**-0.79 (-0.91; -0.67)**
**-0.32 (-0.60; -0.05)**	**Atomoxetine**	-	-	-	0.03 (-0.30; 0.36)	-	**-0.50 (-0.62; -0.37)**
-	-	**Bupropion**	-	-	-	-	**-0.47 (-0.75; -0.19)**
-	**0.45 (0.18; 0.71)**	-	**Guanfacine**	-	-	-	-
-	-	-	-	**Clonidine**	-	-	-

**-0.32 (-0.47; -0.16)**	**0.22 (0.09; 0.34)**	-0.18 (-0.80; 0.44)	-	-	**Methylphenidate**	-	**-0.49 (-0.61; -0.36)**
-	-	-	-	-	0.13 (-0.38; 0.63)	**Modafinil**	0.16 (-0.10; 0.42)
**-1.05 (-1.32; -0.77)**	**-0.56 (-0.66; -0.47)**	-	**-0.69 (-0.82; -0.56)**	**-0.71 (-0.99; -0.42)**	**-0.83 (-0.98; -0.68)**	**-0.62 (-0.91; -0.33)**	**Placebo**

Mean change in symptoms is reported as a standardized mean difference (SMD) along with 95% confidence intervals. An SMD below 0 favours the medication on the top left vs. the medication on the bottom right in the diagonal. The bottom left triangle refers to results in children/adolescents and the top right triangle to results in adults.

Efficacy – ADHD core symptoms, Parents' Ratings

**Amphetamines**	-	-	-	-	-	-
-	**Atomoxetine**	-	-	-	-	-
-	-	**Bupropion**	-	-	-	-
-	-	-	**Guanfacine**	-	-	-
-	0.18 (-0.00; 0.36)	**1.07 (0.40; 1.74)**	-	**Methylphenidate**	-	-
-	-	-	-	-	**Modafinil**	-
**-1.07 (-1.36; -0.79)**	**-0.62 (-0.71; -0.52)**	-	-0.23 (-0.90; 0.45)	**-0.77 (-0.92; -0.61)**	**-0.46 (-0.65; -0.27)**	**Placebo**

Mean change in symptoms is reported as a standardized mean difference (SMD) along with 95% confidence intervals. An SMD below 0 favours the medication on the top left vs. the medication on the bottom right in the diagonal. The bottom left triangle refers to results in children/adolescents and the top right triangle to results in adults.

Efficacy – ADHD core symptoms, Self Ratings

**Amphetamines**	-	-	0.18 (-0.49, 0.85)	-	0.15 (-0.44, 0.75)	**-0.54 (-0.78, -0.30)**
-	**Atomoxetine**	-	-	0.09 (-0.24, 0.42)	-	**-0.38 (-0.47, -0.29)**
-	-	**Bupropion**	-	-	-	-0.30 (-0.61, 0.01)
-	-	-	**Guanfacine**	-	-	**-0.77 (-1.46, -0.08)**
-	-	-	-	**Methylphenidate**	-	**-0.38 (-0.52, -0.25)**
-	-	-	-	-	**Modafinil**	-0.43 (-1.38, 0.51)
-	-	-	-	-	-	**Placebo**

Mean change in symptoms is reported as a standardized mean difference (SMD) along with 95% confidence intervals. An SMD below 0 favours the medication on the top left vs. the medication on the bottom right in the diagonal.

Tolerability

**Amphetamines**	-	-	-	-	-	-	**3.21 (1.62; 6.37)**
1.82 (0.52; 6.25)	**Atomoxetine**	-	-	-	1.25 (0.41; 3.82)	-	**2.43 (1.57; 3.76)**
-	-	**Bupropion**	-	-	-	-	2.58 (0.34; 19.57)
-	-	-	**Clonidine**	-	-	-	-
-	-	-	-	**Guanfacine**	-	-	-

**1.78 (1.01; 3.12)**	1.50 (0.81; 2.78)	1.00 (0.06; 16.51)	0.29 (0.01; 8.37)	-	**Methylphenidate**	-	**2.65 (1.69; 4.13)**
-	-	-	-	-	1.00 (0.02; 50)	**Modafinil**	**4.01 (1.67; 9.66)**
**2.01 (1.14; 3.54)**	1.60 (0.95; 2.69)	1.62 (0.06; 43.25)	**11.16 (1.47; 84.77)**	**3.22 (1.13; 9.22)**	1.32 (0.78; 2.23)	1.29 (0.56; 2.94)	**Placebo**

Discontinuation due to adverse events is reported for each comparison as odds ratio (OR), along with 95% confidence intervals. An OR below 1 favours the medication on the top left vs. the medication on the bottom right in the diagonal. The bottom left triangle refers to results in children/adolescents and the top right triangle to results in adults.

Functioning - Clinical Global Impression (CGI)

**Amphetamines**	-	-	-	-	-	-	**4.87 (3.46; 6.86)**
**5.26 (2.86; 10)**	**Atomoxetine**	-	-	-	-	-	1.95 (0.96; 3.97)
-	-	**Bupropion**	-	-	-	-	**3.87 (1.20; 12.45)**
-	-	-	**Clonidine**	-	-	-	-
-	0.64 (0.37; 1.09)	-	-	**Guanfacine**	-	-	-
**1.74 (1.19; 2.55)**	-	-	0.38 (0.13; 1.10)	-	**Methylphenidate**	-	**3.02 (2.13; 4.28)**
-	-	-	-	-	-	**Modafinil**	0.89 (0.51; 1.54)
**7.21 (4.69; 11.08)**	**2.89 (1.79; 4.67)**	-	**3.55 (1.28; 9.84)**	**3.15 (2.42; 4.10)**	**6.19 (4.27; 8.97)**	**3.26 (2.31; 4.60)**	**Placebo**

Improvement in clinical global functioning is reported for each comparison as odds ratio (OR), along with 95% confidence intervals. An OR below 1 favours the medication on the bottom right vs. the medication on the top left in the diagonal. The bottom left triangle refers to results in children/adolescents and the top right triangle to results in adults.

Weight (Kgs)

**Amphetamines**	-	-	-	-	-	-	**-0.60 (-1.16; -0.03)**
-	**Atomoxetine**	-	-	-	**0.52 (0.18; 0.85)**	-	**-0.54 (-0.86; -0.22)**
-	-	**Bupropion**	-	-	-	-	**-0.78 (-1.10; -0.46)**
-	-	-	**Clonidine**	-	-	-	-
-	-0.25 (-0.51; 0.01)	-	-	**Guanfacine**	-	-	-
**-0.25 (-0.50; -0.01)**	-0.03 (-0.26; 0.21)	-	**0.72 (0.20; 1.24)**	-	**Methylphenidate**	-	**-0.87 (-1.13; -0.61)**
-	-	-	-	-	-	**Modafinil**	-
**-0.60 (-1.13; -0.08)**	**-0.92 (-1.17; -0.68)**	-	0.25 (-0.25; 0.76)	0.09 (-0.01; 0.18)	**-0.78 (-1.18; -0.38)**	**-0.92 (-1.09; -0.74)**	**Placebo**

Mean change in body weight is reported as a standardized mean difference (SMD) along with 95% confidence intervals. An SMD below 0 favours the medication on the bottom right vs. the medication on the top left in the diagonal. The bottom left triangle refers to results in children/adolescents and the top right triangle to results in adults.

Systolic Blood Pressure

**Amphetamines**	-	-	-	-	-	-	0.12 (-0.03; 0.26)
-	**Atomoxetine**	-	-	-	-	-	**0.19 (0.10; 0.29)**
-	-	**Bupropion**	-	-	-	-	0.27 (-0.46; 1.01)
-	-	-	**Clonidine**	-	-	-	-

-	**0.39 (0.13; 0.66)**	-	-	**Guanfacine**	-	-	-
0.06 (-0.12; 0.25)	0.00 (-0.13; 0.14)	-	0.02 (-0.48; 0.53)	-	**Methylphenidate**	-	**0.17 (0.05; 0.30)**
-	-	-	-	-	-	**Modafinil**	-
0.09 (-0.01; 0.20)	**0.12 (0.02; 0.21)**	-	0.13 (-0.37; 0.63)	**-0.26 (-0.38; -0.15)**	**0.15 (0.06; 0.25)**	0.06 (-0.09; 0.21)	**Placebo**

Mean change in systolic blood pressure is reported as a standardized mean difference (SMD) along with 95% confidence intervals. An SMD above 0 indicates an increase in blood pressure values and favours the medication on the bottom right vs. the medication on the top left in the diagonal. The bottom left triangle refers to results in children/adolescents and the top right triangle to results in adults.

Diastolic Blood Pressure

**Amphetamines**	-	-	-	-	-	-	0.04 (-0.08; 0.15)
-	**Atomoxetine**	-	-	-	-	-	**0.25 (0.15; 0.35)**
-	-	**Bupropion**	-	-	-	-	0.20 (-0.54; 0.93)
-	-	-	**Clonidine**	-	-	-	-
-	**0.59 (0.32; 0.85)**	-	-	**Guanfacine**	-	-	-
-0.01 (-0.14; 0.11)	0.09 (-0.03; 0.22)	-	0.11 (-0.39; 0.62)	-	**Methylphenidate**	-	**0.20 (0.09; 0.31)**
-	-	-	-	-	-	**Modafinil**	-
**0.23 (0.09; 0.37)**	**0.22 (0.14; 0.31)**	-	0.01 (-0.49; 0.51)	**-0.22 (-0.35; -0.09)**	**0.28 (0.18; 0.38)**	-0.03 (-0.18; 0.11)	**Placebo**

Mean change in diastolic blood pressure is reported as a standardized mean difference (SMD) along with 95% confidence intervals. An SMD above 0 indicates an increase in blood pressure values and favours the medication on the bottom right vs. the medication on the top left in the diagonal. The bottom left triangle refers to results in children/adolescents and the top right triangle to results in adults.

Acceptability

**Amphetamines**	-	-	-	-	-	-	**0.67 (0.49; 0.90)**
1.33 (0.57; 3.03)	**Atomoxetine**	-	-	-	1.57 (0.71; 3.46)	-	**1.39 (1.08; 1.80)**
-	-	**Bupropion**	-	-	-	-	1.12 (0.58; 2.19)
-	-	-	**Clonidine**	-	-	-	-
-	-	-	-	**Guanfacine**	-	-	-
1.04 (0.77; 1.41)	1.05 (0.67; 1.64)	1.50 (0.33; 6.75)	2.33 (0.17; 32.58)	-	**Methylphenidate**	-	1.25 (0.99; 1.57)
-	-	-	-	-	1.56 (0.24; 10)	**Modafinil**	**1.91 (1.10; 3.30)**
0.78 (0.49; 1.25)	0.94 (0.77; 1.16)	0.47 (0.03; 8.46)	0.58 (0.34; 1.00)	0.78 (0.60; 1.01)	**0.66 (0.47; 0.92)**	0.72 (0.44; 1.18)	**Placebo**

Discontinuation due to any reason is reported for each comparison as odds ratio (OR), along with 95% confidence intervals. An OR below 1 favours the medication on the bottom right vs. the medication on the top left in the diagonal. The bottom left triangle refers to results in children/adolescents and the top right triangle to results in adults.

Results of the Main dose analyses, separating Lisdexamfetamine from other Amphetamines

Efficacy – ADHD core symptoms, Teachers' Ratings

N/A (no comparisons including amphetamines)

Efficacy – ADHD core symptoms, Clinician's Ratings

**Amphetamines**		-	-	-	-		-	**-0.76 (-0.91; -0.60)**
**-0.32 (-0.60; -0.05)**	**Atomoxetine**	-	-	-		0.03 (-0.30; 0.36)	-	**-0.46 (-0.61; -0.30)**
		**Bupropion**					-	**-0.47 (-0.75; -0.19)**
		-	**Guanfacine**				-	
		-	-	**Clonidine**			-	
	-	-	-	-	**Lisdexamfetamine**		-	**-0.86 (-1.07; -0.65)**
-	**0.25 (0.08; 0.41)**	-0.18 (-0.80; 0.44)	-	-	**-0.32 (-0.47; -0.16)**	**Methylphenidate**		**-0.49 (-0.62; -0.36)**
						0.13 (-0.38; 0.63)	**Modafinil**	0.16 (-0.10; 0.42)
**-0.74 (-1.08; -0.40)**	**-0.56 (-0.66, -0.45)**	-	**-0.67 (-0.84; -0.50)**	**-0.71 (-0.99; -0.42)**	**-1.10 (-1.42; -0.78)**	**-0.83 (-0.98; -0.68)**	**-0.62 (-0.91; -0.33)**	**Placebo**

Mean change in symptoms is reported as a standardized mean difference (SMD) along with 95% confidence intervals. An SMD below 0 favours the medication on the top left vs. the medication on the bottom right in the diagonal. The bottom left triangle refers to results in children/adolescents and the top right triangle to results in adults.

Efficacy – ADHD core symptoms, Parents’ Ratings

**Atomoxetine**	-	-	-	-	-	-
-	**Bupropion**	-	-	-	-	-
-	-	**Guanfacine**	-	-	-	-
-	-	-	**Lisdexamfetamine**	-	-	-
**0.29 (0.11; 0.48)**	**1.07 (0.40; 1.74)**	-	-	**Methylphenidate**	-	-
-	-	-	-	-	**Modafinil**	-
**-0.62 (-0.73; -0.50)**	-	-0.23 (-0.90; 0.45)	**-1.07 (-1.36; -0.79)**	**-0.83 (-0.95; -0.70)**	**-0.46 (-0.65; -0.27)**	**Placebo**

Mean change in symptoms is reported as a standardized mean difference (SMD) along with 95% confidence intervals. An SMD below 0 favours the medication on the top left vs. the medication on the bottom right in the diagonal. The bottom left triangle refers to results in children/adolescents and the top right triangle to results in adults.

Efficacy – ADHD core symptoms, Self Ratings

No studies on Lisdexamfetamine were found, thus effects were identical to those in the analysis of Lisdexamfetamine and Other Amphetamines lumped.

Tolerability

**Amphetamines**	-	-	-	-	-	-	-	**3.56 (1.52; 8.37)**
1.82 (0.52; 6.41)	**Atomoxetine**	-	-	-	-	1.25 (0.41; 3.82)	-	**2.33 (1.09; 5.01)**
-	-	**Bupropion**	-	-	-	-	-	2.58 (0.34; 19.57)

-	-	-	**Clonidine**	-	-	-	-	-
-	-	-	-	**Guanfacine**	-	-	-	-
-	-	-	-	-	**Lisdexamfeta mine**	-	-	2.77 (0.87; 8.81)
2.28 (0.48; 10.80)	0.91 (0.38; 2.16)	1.00 (0.06; 16.51)	0.29 (0.01; 8.37)	-	2.12 (0.77; 5.82)	**Methylphenida te**	-	**2.51 (1.51; 4.19)**
-	-	-	-	-	-	1.00 (0.19; 52.36)	**Modafinil**	**4.01 (1.67; 9.66)**
1.21 (0.49; 2.99)	1.55 (0.81; 2.97)	1.62 (0.06; 43.25)	**11.16 (1.47; 84.77)**	**3.22 (1.13; 9.22)**	**2.78 (1.35; 5.76)**	1.32 (0.78; 2.23)	1.29 (0.56; 2.94)	**Placebo**

Discontinuation due to adverse events is reported for each comparison as odds ratio (OR), along with 95% confidence intervals. An OR below 1 favours the medication on the top left vs. the medication on the bottom right in the diagonal. The bottom left triangle refers to results in children/adolescents and the top right triangle to results in adults.

Functioning - Clinical Global Impression (CGI)

**Amphetamines**	-	-	-	-	-	-	-	**4.91 (2.88; 8.36)**
**5.26 (2.86; 10)**	**Atomoxetine**	-	-	-	-	-	-	1.95 (0.96; 3.97)
-	-	**Bupropion**	-	-	-	-	-	**3.87 (1.20; 12.45)**
-	-	-	**Clonidine**	-	-	-	-	-
-	-	-	-	**Guanfacine**	-	-	-	-
-	-	-	-	-	**Lisdexamfetamine**	-	-	**4.82 (2.58; 9.01)**
4.85 (0.86; 27.22)	-	-	0.38 (0.13; 1.10)	-	**1.65 (1.14; 2.39)**	**Methylphenidate**	-	**3.17 (2.10; 4.78)**

-	-	-	-	-	-	-	**Modafinil**	0.89 (0.51; 1.54)
**6.19 (3.34; 11.46)**	**2.62 (1.51; 4.55)**	-	**3.55 (1.28; 9.84)**	**3.58 (2.59; 4.95)**	**7.52 (3.95; 14.30)**	**6.06 (4.08; 8.98)**	**3.26 (2.31; 4.60)**	**Placebo**

Improvement in clinical global functioning is reported for each comparison as odds ratio (OR), along with 95% confidence intervals. An OR below 1 favours the medication on the bottom right vs. the medication on the top left in the diagonal. The bottom left triangle refers to results in children/adolescents and the top right triangle to results in adults.

Weight (Kgs)

**Amphetamines**	-	-	-	-	-	-	-	**-0.87 (-1.47; -0.27)**
-	**Atomoxetine**	-	-	-	-	**0.52 (0.18; 0.85)**	-	-0.32 (-0.70; 0.05)
-	-	**Bupropion**	-	-	-	-	-	**-0.78 (-1.10; -0.46)**
-	-	-	**Clonidine**	-	-	-	-	-
-	-	-	-	**Guanfacine**	-	-	-	-
-	-	-	-	-	**Lisdexamfetamine**	-	-	-0.07 (-0.28; 0.13)
-	0.04 (-0.26; 0.35)	-	**0.72 (0.20; 1.24)**	-	**-0.25 (-0.50; -0.01)**	**Methylphenidate**	-	**-0.77 (-1.15; -0.39)**
-	-	-	-	-	-	-	**Modafinil**	-
**-0.47 (-0.81; -0.14)**	**-0.88 (-1.22; -0.55)**	-	0.25 (-0.25; 0.76)	0.10 (-0.02; 0.22)	-0.62 (-1.25; 0.01)	**-0.84 (-1.26; -0.42)**	**-0.92 (-1.09; -0.74)**	**Placebo**

Mean change in body weight is reported as a standardized mean difference (SMD) along with 95% confidence intervals. An SMD below 0 favours the medication on the bottom right vs. the medication on the top left in the diagonal. The bottom left triangle refers to results in children/adolescents and the top right triangle to results in adults.

Systolic Blood Pressure

**Amphetamines**	-	-	-	-	-	-	-	**0.16 (0.01; 0.30)**
-	**Atomoxetine**	-	-	-	-	-	-	0.11 (-0.00; 0.21)
-	-	**Bupropion**	-	-	-	-	-	0.27 (-0.46; 1.01)
-	-	-	**Clonidine**	-	-	-	-	-
-	-	-	-	**Guanfacine**	-	-	-	-
-	-	-	-	-	**Lisdexamfetamine**	-	-	-0.01 (-0.35; 0.33)
-	0.04 (-0.20; 0.28)	-	0.02 (-0.48; 0.53)	-	0.06 (-0.12; 0.25)	**Methylphenidate**	-	**0.18 (0.03; 0.33)**
-	-	-	-	-	-	-	**Modafinil**	-
-0.04 (-0.27; 0.19)	**0.11 (0.01; 0.21)**	-	0.13 (-0.37; 0.63)	**-0.24 (-0.40; -0.08)**	**0.14 (0.01; 0.27)**	**0.15 (0.05; 0.25)**	0.06 (-0.09; 0.21)	**Placebo**

Mean change in systolic blood pressure is reported as a standardized mean difference (SMD) along with 95% confidence intervals. An SMD above 0 indicates an increase in blood pressure values and favours the medication on the bottom right vs. the medication on the top left in the diagonal. The bottom left triangle refers to results in children/adolescents and the top right triangle to results in adults.

Diastolic Blood Pressure

**Amphetamines**	-	-	-	-	-	-	-	0.08 (-0.07; 0.22)
-	**Atomoxetine**	-	-	-	-	-	-	**0.19 (0.08; 0.30)**
-	-	**Bupropion**	-	-	-	-	-	0.20 (-0.54; 0.93)

-	-	-	**Clonidine**	-	-	-	-	-
-	-	-	-	**Guanfacine**	-	-	-	-
-	-	-	-	-	**Lisdexamfetamine**	-	-	-0.07 (-0.27; 0.14)
-	0.10 (-0.07; 0.26)	-	0.11 (-0.39; 0.62)	-	-0.01 (-0.14; 0.11)	**Methylphenidate**	**-**	**0.20 (0.08; 0.32)**
							**Modafinil**	-
**0.17 (0.02; 0.32)**	**0.26 (0.15; 0.38)**	-	0.01 (-0.49; 0.51)	**-0.18 (-0.36; -0.00)**	**0.24 (0.04; 0.45)**	**0.27 (0.17; 0.38)**	-0.03 (-0.18; 0.11)	**Placebo**

Mean change in systolic blood pressure is reported as a standardized mean difference (SMD) along with 95% confidence intervals. An SMD above 0 indicates an increase in blood pressure values and favours the medication on the bottom right vs. the medication on the top left in the diagonal. The bottom left triangle refers to results in children/adolescents and the top right triangle to results in adults.

Acceptability

**Amphetamines**	-	-	-	-	-	-	-	**0.62 (0.41; 0.93)**
1.33 (0.57; 3.03)	**Atomoxetine**	-	-	-	-	1.57 (0.71; 3.46)	-	1.28 (0.82; 2.01)
-	-	**Bupropion**	-	-	-	-	-	1.12 (0.58; 2.19)
-	-	-	**Clonidine**	-	-	-	-	-
-	-	-	-	**Guanfacine**	-	-	-	-
-	-	-	-	-	**Lisdexamfetamine**	-	-	0.77 (0.48; 1.25)
1.60 (0.49; 5.18)	0.83 (0.49; 1.40)	1.50 (0.33; 6.75)	2.33 (0.17; 32.58)	-	1.01 (0.74; 1.39)	**Methylphenidate**	-	1.09 (0.84; 1.41)

-	-	-	-	-	-	1.56 (0.24; 10)	**Modafinil**	**<u>1.91 (1.10; 3.30)</u>**
0.92 (0.41; 2.06)	0.91 (0.69; 1.21)	0.47 (0.03; 8.46)	0.58 (0.34; 1.00)	0.78 (0.60; 1.01)	0.72 (0.38; 1.38)	**<u>0.66 (0.47; 0.92)</u>**	0.72 (0.44; 1.18)	**Placebo**

Discontinuation due to any reason is reported for each comparison as odds ratio (OR), along with 95% confidence intervals. An OR below 1 favours the medication on the bottom right vs. the medication on the top left in the diagonal. The bottom left triangle refers to results in children/adolescents and the top right triangle to results in adults.

Results of the FDA dose analyses, separating Lisdexamfetamine from other Amphetamines

Efficacy – ADHD core symptoms, Teachers' Ratings

N/A (no comparisons including amphetamines)

Efficacy – ADHD core symptoms, Clinician's Ratings

**Amphetamines**	-	-	-	-	-	**-0.63 (-1.00; -0.27)**
**-0.32 (-0.60; -0.05)**	**Atomoxetine**	-	-	-	0.03 (-0.30; 0.36)	**-0.46 (-0.61; -0.30)**
-	-	**Clonidine**	-	-	-	-
-	-	-	**Guanfacine**	-	-	-
-	-	-	-	**Lisdexamfetamine**	-	**-0.86 (-1.07; -0.64)**
-	**0.25 (0.08; 0.41)**	-	-	**-0.32 (-0.47; -0.16)**	**Methylphenidate**	**-0.49 (-0.62; -0.36)**
**-0.74 (-1.08; -0.40)**	**-0.56 (-0.66; -0.45)**	**-0.71 (-0.99; -0.42)**	**-0.61 (-0.75; -0.46)**	**-1.10 (-1.42; -0.78)**	**-0.83 (-0.98; -0.68)**	**Placebo**

Mean change in symptoms is reported as a standardized mean difference (SMD) along with 95% confidence intervals. An SMD below 0 favours the medication on the top left vs. the medication on the bottom right in the diagonal. The bottom left triangle refers to results in children/adolescents and the top right triangle to results in adults.

Efficacy – ADHD core symptoms, Parents' Ratings

**Atomoxetine**	-	-	
-	**Lisdexamfetamine**	-	

**0.29 (0.11; 0.48)**	-	**Methylphenidate**	-
**-0.62 (-0.73; -0.51)**	**-1.07 (-1.36; -0.79)**	**-0.83 (-0.95; -0.70)**	**Placebo**

Mean change in symptoms is reported as a standardized mean difference (SMD) along with 95% confidence intervals. An SMD below 0 favours the medication on the top left vs. the medication on the bottom right in the diagonal.

Efficacy – ADHD core symptoms, Self Ratings

No studies on Lisdexamfetamine were found, thus effects were identical to those in the analysis of Lisdexamfetamine and Other Amphetamines lumped.

Tolerability

**Amphetamines**	-	-	-	-	-	5.96 (0.91; 38.84)
1.81 (0.51; 6.41)	**Atomoxetine**	-	-	-	1.25 (0.41; 3.82)	**2.33 (1.09; 5.01)**
-	-	**Clonidine**	-	-	-	-
-	-	-	**Guanfacine**	-	-	-
-	-	-	-	**Lisdexamfetamine**	-	2.77 (0.87; 8.81)
2.28 (0.48; 10.80)	0.91 (0.38; 2.16)	-	-	2.12 (0.77; 5.82)	**Methylphenidate**	**2.51 (1.51; 4.19)**
1.21 (0.49; 2.99)	1.55 (0.81; 2.97)	**11.16 (1.47; 84.77)**	**3.68 (1.20; 11.29)**	**2.78 (1.35; 5.76)**	1.32 (0.78; 2.23)	**Placebo**

Discontinuation due to adverse events is reported for each comparison as odds ratio (OR), along with 95% confidence intervals. An OR below 1 favours the medication on the top left vs. the medication on the bottom right in the diagonal. The bottom left triangle refers to results in children/adolescents and the top right triangle to results in adults.

Functioning - Clinical Global Impression (CGI)

**Amphetamines**	-	-	-	-	**2.75 (1.29; 5.84)**
**5.26 (2.86; 10)**	**Atomoxetine**	-	-	-	1.95 (0.96; 3.97)
-	-	**Guanfacine**	-	-	-
-	-	-	**Lisdexamfetamine**	-	**4.82 (2.58; 9.01)**
4.85 (0.86; 27.22)	-	-	**1.65 (1.14; 2.39)**	**Methylphenidate**	**3.17 (2.10; 4.78)**
**6.19 (3.34; 11.46)**	**2.62 (1.51; 4.55)**	**3.50 (2.63; 4.65)**	**7.52 (3.95; 14.30)**	**6.06 (4.08; 8.98)**	**Placebo**

Improvement in clinical global functioning is reported for each comparison as odds ratio (OR), along with 95% confidence intervals. An OR below 1 favours the medication on the bottom right vs. the medication on the top left in the diagonal. The bottom left triangle refers to results in children/adolescents and the top right triangle to results in adults.

Weight (Kgs)

**Amphetamines**	-	-	-	-	-0.18 (-0.53; 0.18)
-	**Atomoxetine**	-	-	**0.52 (0.18; 0.85)**	-0.32 (-0.70; 0.05)
-	-	**Guanfacine**	-	-	-
-	-	-	**Lisdexamfetamine**	-	-0.07 (-0.28; 0.13)
-	0.04 (-0.26; 0.35)	-	**-0.25 (-0.50; -0.01)**	**Methylphenidate**	**-0.77 (-1.15; -0.39)**
**-0.47 (-0.81; -0.14)**	**-0.88 (-1.22; -0.55)**	0.10 (-0.02; 0.22)	-0.62 (-1.25; 0.01)	**-0.83 (-1.26; -0.41)**	**Placebo**

Mean change in body weight is reported as a standardized mean difference (SMD) along with 95% confidence intervals. An SMD below 0 favours the medication on the bottom right vs. the medication on the top left in the diagonal. The bottom left triangle refers to results in children/adolescents and the top right triangle to results in adults.

Systolic Blood Pressure

**Amphetamines**	-	-	-	-	**0.37 (0.01; 0.72)**
-	**Atomoxetine**	-	-	-	0.11 (-0.00; 0.21)
-	-	**Guanfacine**	-	-	-
-	-	-	**Lisdexamfetamine**	-	-0.01 (-0.35; 0.33)
-	0.04 (-0.20; 0.28)	-	0.06 (-0.12; 0.25)	**Methylphenidate**	**0.18 (0.03; 0.33)**
-0.04 (-0.27; 0.19)	**0.11 (0.01; 0.21)**	**-0.25 (-0.42; -0.08)**	**0.14 (0.01; 0.27)**	**0.15 (0.05; 0.25)**	**Placebo**

Mean change in systolic blood pressure is reported as a standardized mean difference (SMD) along with 95% confidence intervals. An SMD above 0 indicates an increase in blood pressure values and favours the medication on the bottom right vs. the medication on the top left in the diagonal. The bottom left triangle refers to results in children/adolescents and the top right triangle to results in adults.

Diastolic Blood Pressure

**Amphetamines**	-	-	-	-	0.01 (-0.34; 0.36)
-	**Atomoxetine**	-	-	-	**0.19 (0.08; 0.30)**
-	-	**Guanfacine**	-	-	-
-	-	-	**Lisdexamfetamine**	-	-0.07 (-0.27; 0.14)

-	0.10 (-0.07; 0.26)	-	-0.01 (-0.14; 0.11)	**Methylphenidate**	**0.20 (0.08; 0.32)**
**0.17 (0.02; 0.32)**	**0.26 (0.15; 0.38)**	**-0.22 (-0.39; -0.06)**	**0.24 (0.04; 0.45)**	**0.27 (0.17; 0.38)**	**Placebo**

Mean change in diastolic blood pressure is reported as a standardized mean difference (SMD) along with 95% confidence intervals. An SMD above 0 indicates an increase in blood pressure values and favours the medication on the bottom right vs. the medication on the top left in the diagonal. The bottom left triangle refers to results in children/adolescents and the top right triangle to results in adults.

Acceptability

**Amphetamines**	-	-	-	-	-	0.74 (0.36; 1.52)
1.33 (0.57; 3.03)	**Atomoxetine**	-	-	-	1.57 (0.71; 3.46)	1.28 (0.82; 2.01)
-	-	**Clonidine**	-	-	-	-
-	-	-	**Guanfacine**	-	-	-
-	-	-	-	**Lisdexamfetamine**	-	0.77 (0.48; 1.25)
1.60 (0.49; 5.18)	0.83 (0.50; 1.40)	-	-	1.01 (0.74; 1.39)	**Methylphenidate**	1.09 (0.84; 1.41)
0.92 (0.41; 2.06)	0.91 (0.69; 1.21)	0.61 (0.35; 1.06)	0.77 (0.58; 1.01)	0.72 (0.38; 1.38)	**0.66 (0.47; 0.92)**	**Placebo**

Discontinuation due to any reason is reported for each comparison as odds ratio (OR), along with 95% confidence intervals. An OR below 1 favours the medication on the bottom right vs. the medication on the top left in the diagonal. The bottom left triangle refers to results in children/adolescents and the top right triangle to results in adults.

Results of the Inclusive dose analyses, separating Lisdexamfetamine from other Amphetamines

Efficacy – ADHD core symptoms, Teachers' Ratings

N/A (no comparisons including amphetamines)

Efficacy – ADHD core symptoms, Clinician's Ratings

**Amphetamines**	-	-	-	-	-	-	-	**-0.75 (-0.90; -0.61)**
**-0.32 (-0.60; -0.05)**	**Atomoxetine**	-	-	-	-	0.03 (-0.30; 0.36)	-	**-0.50 (-0.62; -0.37)**
-	-	**Bupropion**	-	-	-	-	-	**-0.47 (-0.75; -0.19)**
-	-	-	**Clonidine**	-	-	-	-	-
-	**0.45 (0.18; 0.71)**	-	-	**Guanfacine**	-	-	-	-
-	-	-	-	-	**Lisdexamfetamine**	-	-	**-0.86 (-1.07; -0.65)**
-	**0.22 (0.09; 0.34)**	-0.18 (-0.80; 0.44)	-	-	-0.32 (-0.47; -0.16)	**Methylphenidate**	-	**-0.49 (-0.61; -0.36)**
-	-	-	-	-	-	0.13 (-0.38; 0.63)	**Modafinil**	0.16 (-0.10; 0.42)
**-0.80 (-1.11; -0.49)**	**-0.56 (-0.66; -0.47)**	-	**-0.71 (-0.99; -0.42)**	**-0.69 (-0.82; -0.56)**	**-1.10 (-1.42; -0.78)**	**-0.83 (-0.98; -0.68)**	**-0.62 (-0.91; -0.33)**	**Placebo**

Mean change in symptoms is reported as a standardized mean difference (SMD) along with 95% confidence intervals. An SMD below 0 favours the medication on the top left vs. the medication on the bottom right in the diagonal. The bottom left triangle refers to results in children/adolescents and the top right triangle to results in adults.

Efficacy – ADHD core symptoms, Parents' Ratings

**Atomoxetine**	-	-	-	-	-	-
-	**Bupropion**	-	-	-	-	-
-	-	**Guanfacine**	-	-	-	-
-	-	-	**Lisdexamfetamine**	-	-	-
0.18 (-0.00; 0.36)	**1.07 (0.40; 1.74)**	-	-	**Methylphenidate**	-	-
-	-	-	-	-	**Modafinil**	-
**-0.62 (-0.71; -0.52)**	-	-0.23 (-0.90; 0.45)	**-1.07 (-1.36; -0.79)**	**-0.77 (-0.92; -0.61)**	**-0.46 (-0.65; -0.27)**	**Placebo**

Mean change in symptoms is reported as a standardized mean difference (SMD) along with 95% confidence intervals. An SMD below 0 favours the medication on the top left vs. the medication on the bottom right in the diagonal. The bottom left triangle refers to results in children/adolescents and the top right triangle to results in adults.

Efficacy – ADHD core symptoms, Self-Ratings

No studies on Lisdexamfetamine were found, thus effects were identical to those in the analysis of Lisdexamfetamine and Other Amphetamines lumped.

Tolerability

**Amphetamines**	-	-	-	-	-	-	-	**3.48 (1.48; 8.14)**
1.81 (0.51; 6.25)	**Atomoxetine**	-	-	-	-	1.25 (0.41; 3.82)	-	**2.43 (1.57; 3.76)**

-	-	Bupropion	-	-	-	-	-	2.58 (0.34; 19.57)
-	-	-	Guanfacine	-	-	-	-	-
-	-	-	-	Clonidine	-	-	-	-
-	-	-	-	-	Lisdexamfetamine	-	-	2.77 (0.87; 8.81)
2.28 (0.48; 10.80)	1.50 (0.81; 2.78)	1.00 (0.06; 16.51)	-	0.29 (0.01; 8.37)	2.12 (0.77; 5.82)	Methylphenidate	-	**2.65 (1.69; 4.13)**
-	-	-	-	-	-	1.00 (0.02; 50)	Modafinil	**4.01 (1.67; 9.66)**
1.22 (0.50; 3.01)	1.60 (0.95; 2.69)	1.62 (0.06; 43.25)	**3.22 (1.13; 9.22)**	**11.16 (1.47; 84.77)**	**2.78 (1.34; 5.76)**	1.32 (0.78; 2.23)	1.29 (0.56; 2.94)	Placebo

Discontinuation due to adverse events is reported for each comparison as odds ratio (OR), along with 95% confidence intervals. An OR below 1 favours the medication on the top left vs. the medication on the bottom right in the diagonal. The bottom left triangle refers to results in children/adolescents and the top right triangle to results in adults.

Functioning - Clinical Global Impression (CGI)

Amphetamines	-	-	-	-	-	-	-	**4.97 (3.04; 8.13)**
**5.26 (2.86; 10)**	Atomoxetine	-	-	-	-	-	-	1.95 (0.96; 3.97)
-	-	Bupropion	-	-	-	-	-	**3.87 (1.20; 12.45)**
-	-	-	Clonidine	-	-	-	-	-
-	0.64 (-0.37;1.09)	-	-	Guanfacine	-	-	-	-
-		-	-	-	Lisdexamfetamine	-	-	**4.82 (2.58; 9.01)**

4.85 (0.86; 27.22)		-	0.38 (0.13; 1.10)	-	**1.65 (1.14; 2.39)**	**Methylphenidate**	-	**3.02 (2.13; 4.28)**
-	-	-	-	-	-	-	**Modafinil**	0.89 (0.51; 1.54)
**6.37 (3.91; 10.38)**	**2.89 (1.79; 4.67)**	-	**3.55 (1.28; 9.84)**	**3.15 (2.42; 4.10)**	**7.52 (3.95; 14.30)**	**6.19 (4.27; 8.97)**	**3.26 (2.31; 4.60)**	**Placebo**

Improvement in clinical global functioning is reported for each comparison as odds ratio (OR), along with 95% confidence intervals. An OR below 1 favours the medication on the bottom right vs. the medication on the top left in the diagonal. The bottom left triangle refers to results in children/adolescents and the top right triangle to results in adults.

Weight (Kgs)

**Amphetamines**	-	-	-	-	-	-	-	**-0.87 (-1.49; -0.24)**
-	**Atomoxetine**	-	-	-	-	**0.52 (0.18; 0.85)**	-	**-0.54 (-0.86; -0.22)**
-	-	**Bupropion**	-	-	-	-	-	**-0.78 (-1.10; -0.46)**
-	-	-	**Clonidine**	-	-	-	-	-
-	-0.25 (-0.51; 0.01)	-		**Guanfacine**	-	-	-	-
-	-	-	-	-	**Lisdexamfetamine**	-	-	-0.07 (-0.28; 0.13)
-	-0.03 (-0.26; 0.21)	-	**0.72 (0.20; 1.24)**	-	**-0.25 (-0.50; -0.01)**	**Methylphenidate**	-	**-0.87 (-1.13; -0.61)**
-	-	-	-	-	-	-	**Modafinil**	**-**
**-0.49 (-0.79; -0.18)**	**-0.91 (-1.15; -0.66)**	-	0.25 (-0.25; 0.76)	0.09 (-0.01; 0.18)	-0.62 (-1.25; 0.01)	**-0.78 (-1.18; -0.38)**	**-0.92 (-1.09; -0.74)**	**Placebo**

Mean change in body weight is reported as a standardized mean difference (SMD) along with 95% confidence intervals. An SMD below 0 favours the medication on the bottom right vs. the medication on the top left in the diagonal. The bottom left triangle refers to results in children/adolescents and the top right triangle to results in adults.

Systolic Blood Pressure

**Amphetamines**	-	-	-	-	-	-	-	**0.17 (0.03; 0.31)**
-	**Atomoxetine**	-	-	-	-	-	-	**0.19 (0.10; 0.29)**
-	-	**Bupropion**	-	-	-	-	-	0.27 (-0.46; 1.01)
-	-	-	**Clonidine**	-	-	-	-	-
-	**0.39 (0.13; 0.66)**	-		**Guanfacine**	-	-	-	-
-	-		-	-	**Lisdexamfetamine**	-	-	-0.01 (-0.35; 0.33)
-	0.00 (-0.13; 0.14)	-	0.02 (-0.48; 0.53)	-	0.06 (-0.12; 0.25)	**Methylphenidate**	-	**0.17 (0.05; 0.30)**
-	-	-	-	-	-	-	**Modafinil**	-
0.01 (-0.14; 0.15)	**0.12 (0.02; 0.21)**	-	0.13 (-0.37; 0.63)	**-0.26 (-0.38; -0.15)**	**0.14 (0.01; 0.27)**	**0.15 (0.06; 0.25)**	0.06 (-0.09; 0.21)	**Placebo**

Mean change in systolic blood pressure is reported as a standardized mean difference (SMD) along with 95% confidence intervals. An SMD above 0 indicates an increase in blood pressure values and favours the medication on the bottom right vs. the medication on the top left in the diagonal. The bottom left triangle refers to results in children/adolescents and the top right triangle to results in adults.

Diastolic Blood Pressure

**Amphetamines**	-	-	-	-	-	-	-	0.09 (-0.05; 0.23)
-	**Atomoxetine**	-	-	-	-	-	-	**0.25 (0.15; 0.35)**
-	-	**Bupropion**	-	-	-	-	-	0.20 (-0.54; 0.93)

-	-	-	**Clonidine**	-	-	-	-	-
-	**0.59 (0.32; 0.85)**	-	0.59 (0.32; 0.85)	**Guanfacine**	-	-	-	-
-	-	-	-	-	**Lisdexamfetamine**	-	-	-0.12 (-0.34; 0.10)
-	0.09 (-0.03; 0.22)	-	0.11 (-0.39; 0.62)	-	-0.01 (-0.14; 0.11)	**Methylphenidate**	-	**0.20 (0.09; 0.31)**
-	-	-	-	-	-	-	**Modafinil**	-
**0.18 (0.03; 0.33)**	**0.22 (0.13; 0.30)**	-	0.01 (-0.49; 0.51)	**-0.22 (-0.35; -0.09)**	**0.24 (0.04; 0.45)**	**0.28 (0.18; 0.38)**	-0.03 (-0.18; 0.11)	**Placebo**

Mean change in diastolic blood pressure is reported as a standardized mean difference (SMD) along with 95% confidence intervals. An SMD above 0 indicates an increase in blood pressure values and favours the medication on the bottom right vs. the medication on the top left in the diagonal. The bottom left triangle refers to results in children/adolescents and the top right triangle to results in adults.

Acceptability

**Amphetamines**	-	-	-	-	-	-	-	**0.60 (0.41; 0.89)**
1.33 (0.57; 3.03)	**Atomoxetine**	-	-	-	-	1.57 (0.71; 3.46)	-	**1.39 (1.08; 1.80)**
-	-	**Bupropion**	-	-	-	-	-	1.12 (0.58; 2.19)
-	-	-	**Clonidine**	-	-	-	-	-
-	-	-	-	**Guanfacine**	-	-	-	-
-	-	-	-	-	**Lisdexamfetamine**	-	-	0.77 (0.48; 1.25)
1.60 (0.49; 5.18)	1.05 (0.67; 1.64)	1.50 (0.33; 6.75)	2.33 (0.17; 32.58)	-	1.01 (0.74; 1.39)	**Methylphenidate**	-	1.25 (0.99; 1.57)
-	-	-	-	-	-	1.56 (0.24; 10)	**Modafinil**	**1.91 (1.10; 3.30)**

0.93 (0.41; 2.11)	0.94 (0.77; 1.16)	0.47 (0.03; 8.46)	0.58 (0.34; 1.00)	0.78 (0.60; 1.01)	0.72 (0.38; 1.38)	**0.66 (0.47; 0.92)**	0.72 (0.44; 1.18)	**Placebo**

Discontinuation due to any reason is reported for each comparison as odds ratio (OR), along with 95% confidence intervals. An OR below 1 favours the medication on the bottom right vs. the medication on the top left in the diagonal. The bottom left triangle refers to results in children/adolescents and the top right triangle to results in adults.

HETEROGENEITY

Abbreviation for Medications: AMPH: Amphetamines; BUP: Bupropion; CLON: Clonidine; GUA: Guanfacine; GXR: Guanfacine Extended Release LDX: Lisdexamfetamine; MODA: Modafinil; MPH: Methylphenidate; PBO: Placebo

FDA dose analyses, Lisdexamfetamine and other Amphetamines lumped

Efficacy – Mean overall change in ADHD core symptoms, Children/adolescents, Teachers' Ratings

Comparison	No. of studies	P-value	I^2(%)	τ^2
ATMX vs PBO	3	0.648	0.00	0.0000
MPH vs PBO	5	0.199	33.40	0.0218

Efficacy – Mean overall change in ADHD core symptoms, Children/adolescents, Clinician's Ratings

Comparison	No. of studies	P-value	I^2(%)	τ^2
ATMX vs PBO	21	0.008	47.90	0.0274
GUA vs PBO	6	0.123	42.30	0.0137
MPH vs PBO	9	0.066	45.40	0.0229
AMPH vs PBO	6	0.000	82.90	0.1036
AMPH vs MPH	3	0.242	29.50	0.0057
ATMX vs MPH	3	0.374	0.00	0.0000

Efficacy – Mean overall change in ADHD core symptoms, Adults, Clinician's Ratings

Comparison	No. of studies	P-value	I^2(%)	$τ^2$
AMPH vs PBO	3	0.514	0.00	0.0000
MPH vs PBO	11	0.011	56.30	0.0259
ATMX vs PBO	11	0.000	76.50	0.0500

Efficacy – ADHD core symptoms, Children, Parents' Ratings

Comparison	No. of studies	P-value	I^2(%)	$τ^2$
MPH vs PBO	9	0.475	0.00	0.0000
ATMX vs PBO	9	0.840	0.00	0.0000

Efficacy – ADHD core symptoms, Adults, Self Ratings

Comparison	No. of studies	P-value	I^2(%)	$τ^2$
ATMX vs PBO	5	0.915	0.00	0.0000
MPH vs PBO	8	0.082	44.50	0.0132

Tolerability, Children/adolescents

Comparison	No. of studies	P-value	I^2(%)	$τ^2$
ATMX vs PBO	13	0.9780	0.00	0.0000
MPH vs PBO	22	0.9220	0.00	0.0000
AMPH vs PBO	9	0.5030	0.00	0.0000

GUA vs PBO	6	0.0850	48.30	0.8858
AMPH vs MPH	6	0.4650	0.00	0.0000
ATMX vs MPH	4	0.9500	0.00	0.0000

Tolerability, Adults

Comparison	No. of studies	P-value	I^2(%)	$τ^2$
AMPH vs PBO	5	0.7070	0.00	0.0000
MPH vs PBO	12	0.8850	0.00	0.0000
ATMX vs PBO	6	0.1990	31.50	0.2698

Functioning - Clinical Global Impression (CGI), Children/adolescents

Comparison	No. of studies	P-value	I^2(%)	$τ^2$
MPH vs PBO	11	0.005	60.10	0.2395
ATMX vs PBO	4	0.145	44.30	0.1359
AMPH vs PBO	8	0.000	74.20	0.2801
GUA vs PBO	5	0.302	17.60	0.0187
AMPH vs MPH	4	0.227	30.80	0.0465

Functioning - Clinical Global Impression (CGI), Adults

Comparison	No. of studies	P-value	I^2(%)	$τ^2$
AMPH vs PBO	3	0.197	38.40	0.0758
MPH vs PBO	6	0.021	62.50	0.1488

Weight, Children/adolescents

Comparison	No. of studies	P-value	I^2(%)	$τ^2$
ATMX vs PBO	13	0.0000	91.00	0.3468
MPH vs PBO	12	0.0000	94.30	0.5248
GUA vs PBO	5	0.4680	0.00	0.0000
AMPH vs PBO	6	0.0000	95.50	0.4230
AMPH vs MPH	3	0.0290	71.80	0.0336
ATMX vs MPH	3	0.1570	45.90	0.0354

Weight, Adults

Comparison	No. of studies	P-value	I^2(%)	$τ^2$
AMPH vs PBO	3	0.8590	0.00	0.0000
MPH vs PBO	5	0.0000	82.30	0.1486
ATMX vs PBO	4	0.0000	89.50	0.1288

Systolic Blood Pressure, Children/adolescents

Comparison	No. of studies	P-value	I^2(%)	$τ^2$
ATMX vs PBO	12	0.542	0.00	0.0000
MPH vs PBO	11	0.742	0.00	0.0000
AMPH vs PBO	7	0.163	34.70	0.0085
GUA vs PBO	5	0.093	49.70	0.0186
AMPH vs MPH	3	0.131	50.70	0.0134
ATMX vs MPH	3	0.269	23.90	0.0131

Systolic Blood Pressure, Adults

Comparison	No. of studies	P-value	I^2(%)	τ^2
AMPH vs PBO	3	0.054	65.70	0.0480
MPH vs PBO	6	0.174	35.00	0.0125
ATMX vs PBO	4	0.589	0.00	0.0000

Diastolic Blood Pressure, Children/adolescents

Comparison	No. of studies	P-value	I^2(%)	τ^2
ATMX vs PBO	12	0.2450	20.20	0.0077
MPH vs PBO	11	0.3840	6.30	0.0021
AMPH vs PBO	7	0.0380	54.90	0.0194
CLON vs PBO	5	0.1210	45.10	0.0155
AMPH vs MPH	3	0.4030	0.00	0.0000
ATMX vs MPH	3	0.9290	0.00	0.0000

Diastolic Blood Pressure, Adults

Comparison	No. of studies	P-value	I^2(%)	τ^2
AMPH vs PBO	3	0.5900	0.00	0.0000
MPH vs PBO	6	0.5500	0.00	0.0000
ATMX vs PBO	4	0.6580	0.00	0.0000

Acceptability, Children/adolescents

Comparison	No. of studies	P-value	I^2(%)	τ^2
ATMX vs PBO	14	0.4330	1.50	0.0042
MPH vs PBO	21	0.0140	45.10	0.2160
AMPH vs PBO	9	0.0010	68.80	0.3093
GUA vs PBO	7	0.3710	7.50	0.0108
AMPH vs MPH	6	0.8140	0.00	0.0000
ATMX vs MPH	4	0.2790	21.90	0.0680

Acceptability, Adults

Comparison	No. of studies	P-value	I^2(%)	τ^2
AMPH vs PBO	5	0.7290	0.00	0.0000
MPH vs PBO	11	0.9120	0.00	0.0000
ATMX vs PBO	6	0.0610	52.70	0.1501

Inclusive dose analyses, Lisdexamfetamine and other Amphetamines lumped

Efficacy – Mean overall change in ADHD core symptoms, Children/adolescents, Teachers' Ratings

Comparison	No. of studies	P-value	I^2(%)	τ^2
ATMX vs PBO	5	0.765	0.00	0.0000
MPH vs PBO	6	0.068	51.30	0.0433
MODA vs PBO	4	0.000	85.90	0.2027

Efficacy – Mean overall change in ADHD core symptoms, Children/adolescents, Clinician's Ratings

Comparison	No. of studies	P-value	I^2(%)	τ^2
ATMX vs MPH	5	0.655	0.00	0.0000
ATMX vs PBO	24	0.005	47.60	0.0261
MODA vs PBO	5	0.006	72.20	0.0741
GUA vs PBO	8	0.095	42.40	0.0142
MPH vs PBO	9	0.066	45.40	0.0229
AMPH vs PBO	6	0.000	82.50	0.0981
AMPH vs MPH	3	0.242	29.50	0.0057

Efficacy – Mean overall change in ADHD core symptoms, Adults, Clinician's Ratings

Comparison	No. of studies	P-value	I^2(%)	τ^2
AMPH vs PBO	5	0.651	0.00	0.0000
MPH vs PBO	13	0.005	57.90	0.0278
ATMX vs PBO	11	0.001	65.40	0.0291

Efficacy – ADHD core symptoms, Children, Parents' Ratings

Comparison	No. of studies	P-value	I^2(%)	τ^2
ATMX vs MPH	4	0.246	27.60	0.0094
MPH vs PBO	10	0.118	36.30	0.0215
ATMX vs PBO	13	0.962	0.00	0.0000
MODA vs PBO	4	0.169	40.40	0.0156

Efficacy – ADHD core symptoms, Adults, Self Ratings

Comparison	No. of studies	P-value	I^2(%)	τ^2
AMPH vs PBO	4	0.823	0.00	0.0000
ATMX vs PBO	8	0.971	0.00	0.0000
MPH vs PBO	10	0.016	55.50	0.0228

Tolerability, Children/adolescents

Comparison	No. of studies	P-value	I^2(%)	τ^2
ATMX vs MPH	7	0.5200	0.00	0.0000
ATMX vs PBO	21	0.9980	0.00	0.0000
MPH vs PBO	22	0.9220	0.00	0.0000
AMPH vs PBO	9	0.5020	0.00	0.0000
MODA vs PBO	6	0.4340	0.00	0.0000
GUA vs PBO	7	0.1120	41.90	0.7755
AMPH vs MPH	6	0.4650	0.00	0.0000

Tolerability, Adults

Comparison	No. of studies	P-value	I^2(%)	τ^2
AMPH vs PBO	6	0.8670	0.00	0.0000
MPH vs PBO	15	0.9120	0.00	0.0000
ATMX vs PBO	10	0.4550	0.00	0.0000
BUP vs PBO	3	0.4850	0.00	0.0000

Functioning - Clinical Global Impression (CGI), Children/adolescents

Comparison	No. of studies	P-value	I^2(%)	$τ^2$
MPH vs PBO	12	0.008	57.00	0.2203
ATMX vs PBO	5	0.131	43.70	0.1266
AMPH vs PBO	8	0.000	73.20	0.2618
MODAsPBO	4	0.459	0.00	0.0000
GUA vs PBO	8	0.154	34.30	0.0478
AMPH vs MPH	4	0.227	30.80	0.0465

Functioning - Clinical Global Impression (CGI), Adults

Comparison	No. of studies	P-value	I^2(%)	$τ^2$
AMPH vs PBO	6	0.167	36.00	0.0636
MPH vs PBO	7	0.065	49.40	0.0966

Weight, Children/adolescents

Comparison	No. of studies	P-value	I^2(%)	$τ^2$
ATMX vs MPH	5	0.0230	64.70	0.0415
ATMX vs PBO	21	0.0000	90.10	0.2913
MPH vs PBO	13	0.0000	94.00	0.4948
MODA vs PBO	3	0.4670	0.00	0.0000
GUA vs PBO	7	0.6950	0.00	0.0000
AMPH vs PBO	6	0.0000	95.50	0.4117
AMPH vs MPH	3	0.0290	71.80	0.0336

Weight, Adults

Comparison	No. of studies	P-value	I^2(%)	τ^2
AMPH vs PBO	6	0.0000	95.20	0.4610
MPH vs PBO	8	0.0000	73.20	0.0937
ATMX vs PBO	7	0.0000	92.10	0.1680

Systolic Blood Pressure, Children/adolescents

Comparison	No. of studies	P-value	I^2(%)	τ^2
ATMX vs MPH	5	0.3400	11.60	0.0030
ATMX vs PBO	18	0.1060	30.70	0.0128
MPH vs PBO	12	0.8110	0.00	0.0000
AMPH vs PBO	7	0.2600	22.20	0.0044
MODA vs PBO	4	0.8250	0.00	0.0000
GUA vs PBO	8	0.2260	25.40	0.0068
AMPH vs MPH	3	0.1310	50.70	0.0134

Systolic Blood Pressure, Adults

Comparison	No. of studies	P-value	I^2(%)	τ^2
AMPH vs PBO	6	0.1930	32.40	0.0104
MPH vs PBO	8	0.3180	14.30	0.0046
ATMX vs PBO	6	0.4020	2.20	0.0003

Diastolic Blood Pressure, Children/adolescents

Comparison	No. of studies	P-value	I^2(%)	τ^2
ATMX vs MPH	5	0.4340	0.00	0.0000
ATMX vs PBO	19	0.1890	21.80	0.0080
MPH vs PBO	12	0.4700	0.00	0.0000
AMPH vs PBO	7	0.0390	54.70	0.0190
MODA vs PBO	4	0.7300	0.00	0.0000
GUA vs PBO	8	0.1110	40.10	0.0134
AMPH vs MPH	3	0.4030	0.00	0.0000

Diastolic Blood Pressure, Adults

Comparison	No. of studies	P-value	I^2(%)	τ^2
AMPH vs PBO	6	0.7710	0.00	0.0000
MPH vs PBO	8	0.7730	0.00	0.0000
ATMX vs PBO	5	0.3360	12.20	0.0017

Acceptability, Children/adolescents

Comparison	No. of studies	P-value	I^2(%)	τ^2
ATMX vs MPH	7	0.1910	31.00	0.1046
ATMX vs PBO	22	0.4260	2.50	0.0062
MPH vs PBO	21	0.0140	45.10	0.2160
AMPH vs PBO	9	0.0010	69.10	0.3119
MODA vs PBO	6	0.1230	42.30	0.1406
GUA vs PBO	8	0.4090	2.70	0.0043
AMPH vs MPH	6	0.8140	0.00	0.0000

Acceptability, Adults

Comparison	No. of studies	P-value	I^2(%)	τ^2
AMPH vs PBO	6	0.7430	0.00	0.0000
MPH vs PBO	13	0.6870	0.00	0.0000
ATMX vs PBO	10	0.1920	27.40	0.0433
BUP vs PBO	3	0.4000	0.00	0.0000

Main dose analyses, Lisdexamfetamine separated from other Amphetamines

Efficacy – Mean overall change in ADHD core symptoms, Children/adolescents, Teachers' Ratings

Comparison	No. of studies	P-value	I^2(%)	τ^2
ATMX vs PBO	3	0.648	0.00	0.0000
MPH vs PBO	5	0.199	33.40	0.0218
MODA vs PBO	4	0.000	85.90	0.2027

Efficacy – Mean overall change in ADHD core symptoms, Children/adolescents, Clinician's Ratings

Comparison	No. of studies	P-value	I^2(%)	τ^2
ATMX vs PBO	21	0.008	47.90	0.0274
MODA vs PBO	5	0.006	72.20	0.0741
GUA vs PBO	7	0.012	63.30	0.0334
MPH vs PBO	9	0.066	45.40	0.0229
LDX vs PBO	5	0.000	84.70	0.1116
LDX vs MPH	3	0.242	29.50	0.0057

ATMX vs MPH	3	0.374	0.00	0.0000

Efficacy – Mean overall change in ADHD core symptoms, Adults, Clinician's Ratings

Comparison	No. of studies	P-value	I^2(%)	$τ^2$
AMPH vs PBO	3	0.437	0.00	0.0000
MPH vs PBO	11	0.011	56.30	0.0259
ATMX vs PBO	11	0.000	76.50	0.0500

Efficacy – ADHD core symptoms, Children, Parents' Ratings

Comparison	No. of studies	P-value	I^2(%)	$τ^2$
MPH vs PBO	9	0.475	0.00	0.0000
ATMX vs PBO	9	0.840	0.00	0.0000
MODA vs PBO	4	0.169	40.40	0.0156

Efficacy – ADHD core symptoms, Adults, Self Ratings

N/A

Tolerability, Children/adolescents

Comparison	No. of studies	P-value	I^2(%)	$τ^2$
ATMX vs PBO	13	0.9780	0.00	0.0000
MPH vs PBO	22	0.9220	0.00	0.0000
AMPH vs PBO	4	0.5830	0.00	0.0000
MODA vs PBO	6	0.4340	0.00	0.0000

GUA vs PBO	7	0.1120	41.90	0.7755
LDX vs PBO	5	0.4970	0.00	0.0000
LDX vs MPH	3	0.1120	54.20	0.4315
AMPH vs MPH	3	0.9390	0.00	0.0000
ATMX vs MPH	4	0.9500	0.00	0.0000

Tolerability, Adults

Comparison	No. of studies	P-value	I^2(%)	τ^2
AMPH vs PBO	3	0.4960	0.00	0.0000
MPH vs PBO	12	0.8850	0.00	0.0000
ATMX vs PBO	6	0.1990	31.50	0.2698
BUP vs PBO	3	0.4850	0.00	0.0000
LDX vs PBO	3	0.7200	0.00	0.0000

Functioning - Clinical Global Impression (CGI), Children/adolescents

Comparison	No. of studies	P-value	I^2(%)	τ^2
MPH vs PBO	11	0.005	60.10	0.2395
ATMX vs PBO	4	0.145	44.30	0.1359
AMPH vs PBO	3	0.151	47.10	0.1383
MODA vs PBO	4	0.459	0.00	0.0000
GUA vs PBO	6	0.193	32.30	0.0515
LDX vs PBO	5	0.000	82.80	0.4428
LDX vs MPH	3	0.237	30.60	0.0326

Functioning - Clinical Global Impression (CGI), Adults

Comparison	No. of studies	P-value	I^2(%)	$τ^2$
AMPH vs PBO	4	0.092	53.50	0.1478
MPH vs PBO	6	0.021	62.50	0.1488

Weight, Children/adolescents

Comparison	No. of studies	P-value	I^2(%)	$τ^2$
ATMX vs PBO	13	0.0000	91.00	0.3468
MPH vs PBO	12	0.0000	94.30	0.5114
MODA vs PBO	3	0.4670	0.00	0.0000
GUA vs PBO	5	0.4680	0.00	0.0000
LDX vs PBO	5	0.0000	96.40	0.4984
LDX vs MPH	3	0.0290	71.80	0.0336
ATMX vs MPH	3	0.1570	45.90	0.0354

Weight, Adults

Comparison	No. of studies	P-value	I^2(%)	$τ^2$
AMPH vs PBO	4	0.0000	92.60	0.3390
MPH vs PBO	5	0.0000	82.30	0.1486
ATMX vs PBO	4	0.0000	89.50	0.1288

Systolic Blood Pressure, Children/adolescents

Comparison	No. of studies	P-value	I^2(%)	$τ^2$

ATMX vs PBO	12	0.5420	0.00	0.0000
MPH vs PBO	11	0.7410	0.00	0.0000
MODA vs PBO	4	0.8250	0.00	0.0000
GUA vs PBO	6	0.1280	41.60	0.0160
LDX vs PBO	5	0.2950	18.80	0.0041
LDX vs MPH	3	0.1310	50.70	0.0134
ATMX vs MPH	3	0.2690	23.90	0.0131

Systolic Blood Pressure, Adults

Comparison	No. of studies	P-value	I^2(%)	τ^2
AMPH vs PBO	4	0.6120	0.00	0.0000
MPH vs PBO	6	0.1740	35.00	0.0125
ATMX vs PBO	4	0.5890	0.00	0.0000

Diastolic Blood Pressure, Children/adolescents

Comparison	No. of studies	P-value	I^2(%)	τ^2
ATMX vs PBO	12	0.2450	20.20	0.0077
MPH vs PBO	11	0.3900	5.60	0.0018
MODA vs PBO	4	0.7300	0.00	0.0000
GUA vs PBO	6	0.0580	53.10	0.0255
LDX vs PBO	5	0.0130	68.40	0.0379
LDX vs MPH	3	0.4030	0.00	0.0000
ATMX vs MPH	3	0.9290	0.00	0.0000

Diastolic Blood Pressure, Adults

Comparison	No. of studies	P-value	I^2(%)	τ^2
AMPH vs PBO	4	0.9770	0.00	0.0000
MPH vs PBO	6	0.5500	0.00	0.0000
ATMX vs PBO	4	0.6580	0.00	0.0000

Acceptability, Children/adolescents

Comparison	No. of studies	P-value	I^2(%)	τ^2
ATMX vs PBO	14	0.4330	1.50	0.0042
MPH vs PBO	21	0.0140	45.10	0.2160
AMPH vs PBO	4	0.1700	40.30	0.2757
MODA vs PBO	6	0.1230	42.30	0.1406
GUA vs PBO	8	0.4090	2.70	0.0043
LDX vs PBO	5	0.0000	80.60	0.4422
LDX vs MPH	3	0.6210	0.00	0.0000
AMPH vs MPH	3	0.6870	0.00	0.0000
ATMX vs MPH	4	0.2790	21.90	0.0680

Acceptability, Adults

Comparison	No. of studies	P-value	I^2(%)	τ^2
AMPH vs PBO	3	0.7400	0.00	0.0000
MPH vs PBO	11	0.9120	0.00	0.0000
ATMX vs PBO	6	0.0610	52.70	0.1501

BUP vs PBO	3	0.4000	0.00	0.0000
LDX vs PBO	3	0.4140	0.00	0.0000

FDA dose analyses, Lisdexamfetamine separated from other Amphetamines

Efficacy – Mean overall change in ADHD core symptoms, Children/adolescents, Teachers' Ratings

Comparison	No. of studies	P-value	I^2(%)	τ^2
ATMX vs PBO	3	0.648	0.00	0.0000
MPH vs PBO	5	0.199	33.40	0.0218

Efficacy – Mean overall change in ADHD core symptoms, Children/adolescents, Clinician's Ratings

Comparison	No. of studies	P-value	I^2(%)	τ^2
ATMX vs PBO	21	0.008	47.90	0.0274
GUA vs PBO	6	0.123	42.30	0.0137
MPH vs PBO	9	0.066	45.40	0.0229
LDX vs PBO	5	0.000	84.70	0.1116
LDX vs MPH	3	0.242	29.50	0.0057
ATMX vs MPH	3	0.374	0.00	0.0000

Efficacy – Mean overall change in ADHD core symptoms, Adults, Clinician's Ratings

Comparison	No. of studies	P-value	I^2(%)	τ^2
MPH vs PBO	11	0.011	56.30	0.0259
ATMX vs PBO	11	0.000	76.50	0.0500
LDX vs PBO	2	0.616	0.00	0.0000

Efficacy – ADHD core symptoms, Children, Parents' Ratings

Comparison	No. of studies	P-value	I^2(%)	τ^2
MPH vs PBO	9	0.475	0.00	0.0000
ATMX vs PBO	9	0.840	0.00	0.0000
ATMX vs MPH	2	0.444	0.00	0.0000

Efficacy – ADHD core symptoms, Adults, Self Ratings

N/A (no comparison with lisdexamfetamine)

Tolerability, Children/adolescents

Comparison	No. of studies	P-value	I^2(%)	τ^2
ATMX vs PBO	13	0.9780	0.00	0.0000
MPH vs PBO	22	0.9220	0.00	0.0000
AMPH vs PBO	4	0.5830	0.00	0.0000
GUA vs PBO	6	0.0850	48.30	0.8858
LDX vs PBO	5	0.4970	0.00	0.0000
LDX vs MPH	3	0.1120	54.20	0.4315
AMPH vs MPH	3	0.9390	0.00	0.0000
ATMX vs MPH	4	0.9500	0.00	0.0000

Tolerability, Adults

Comparison	No. of studies	P-value	I^2(%)	τ^2

MPH vs PBO	12	0.8850	0.00	0.0000
ATMX vs PBO	6	0.1990	31.50	0.2698
LDX vs PBO	3	0.7200	0.00	0.0000

Functioning - Clinical Global Impression (CGI), Children/adolescents

Comparison	No. of studies	P-value	I^2(%)	τ^2
MPH vs PBO	11	0.0050	60.10	0.2395
ATMX vs PBO	4	0.1450	44.30	0.1359
AMPH vs PBO	3	0.1510	47.10	0.1383
GUA vs PBO	5	0.3020	17.60	0.0187
LDX vs PBO	5	0.0000	82.80	0.4428
LDX vs MPH	3	0.2370	30.60	0.0326

Functioning - Clinical Global Impression (CGI), Adults

Comparison	No. of studies	P-value	I^2(%)	τ^2
MPH vs PBO	6	0.0210	62.50	0.1488

Weight, Children/adolescents

Comparison	No. of studies	P-value	I^2(%)	τ^2
ATMX vs PBO	13	0.0000	91.00	0.3468
MPH vs PBO	12	0.0000	94.30	0.5248
GUA vs PBO	5	0.4680	0.00	0.0000

LDX vs PBO	5	0.0000	96.40	0.4984
LDX vs MPH	3	0.0290	71.80	0.0336
ATMX vs MPH	3	0.1570	45.90	0.0354

Weight, Adults

Comparison	No. of studies	P-value	I^2(%)	$τ^2$
MPH vs PBO	5	0.0000	82.30	0.1486
ATMX vs PBO	4	0.0000	89.50	0.1288

Systolic Blood Pressure, Children/adolescents

Comparison	No. of studies	P-value	I^2(%)	$τ^2$
ATMX vs PBO	12	0.5420	0.00	0.0000
MPH vs PBO	11	0.7420	0.00	0.0000
GUA vs PBO	5	0.0930	49.70	0.0186
LDX vs PBO	5	0.2950	18.80	0.0041

Systolic Blood Pressure, Adults

Comparison	No. of studies	P-value	I^2(%)	$τ^2$
MPH vs PBO	6	0.1740	35.00	0.0125
ATMX vs PBO	4	0.5890	0.00	0.0000

Diastolic Blood Pressure, Children/adolescents

Comparison	No. of studies	P-value	I^2(%)	τ^2
ATMX vs PBO	12	0.2450	20.20	0.0077
MPH vs PBO	11	0.3840	6.30	0.0021
GUA vs PBO	5	0.1210	45.10	0.0155
LDX vs PBO	5	0.0130	68.40	0.0379
LDX vs MPH	3	0.4030	0.00	0.0000
ATMX vs MPH	3	0.9290	0.00	0.0000

Diastolic Blood Pressure, Adults

Comparison	No. of studies	P-value	I^2(%)	τ^2
MPH vs PBO	6	0.5500	0.00	0.0000
ATMX vs PBO	4	0.6580	0.00	0.0000

Acceptability, Children/adolescents

Comparison	No. of studies	P-value	I^2(%)	τ^2
ATMX vs PBO	14	0.4330	1.50	0.0042
MPH vs PBO	21	0.0140	45.10	0.2160
AMPH vs PBO	4	0.1700	40.30	0.2757
GUA vs PBO	7	0.3710	7.50	0.0108
LDX vs PBO	5	0.0000	80.60	0.4422
LDX vs MPH	3	0.6210	0.00	0.0000

AMPH vs MPH	3	0.6870	0.00	0.0000
ATMX vs MPH	4	0.2790	21.90	0.0680

Acceptability, Adults

Comparison	No. of studies	P-value	I^2(%)	$τ^2$
MPH vs PBO	11	0.9120	0.00	0.0000
ATMX vs PBO	6	0.0610	52.70	0.1501
LDX vs PBO	3	0.4140	0.00	0.0000

Inclusive dose analyses, Lisdexamfetamine separated from other Amphetamines

Efficacy – Mean overall change in ADHD core symptoms, Children/adolescents, Teachers' Ratings

Comparison	No. of studies	P-value	I^2(%)	$τ^2$
ATMX vs PBO	5	0.765	0.00	0.0000
MPH vs PBO	6	0.068	51.30	0.0433
MODA vs PBO	4	0.000	85.90	0.2027

Efficacy – Mean overall change in ADHD core symptoms, Children/adolescents, Clinician's Ratings

Comparison	No. of studies	P-value	I^2(%)	$τ^2$
ATMX vs MPH	5	0.655	0.00	0.0000

ATMX vs PBO	24	0.005	47.60	0.0261
MODA vs PBO	5	0.006	72.20	0.0741
GUA vs PBO	8	0.095	42.40	0.0142
MPH vs PBO	9	0.066	45.40	0.0229
LDX vs PBO	5	0.000	84.70	0.1116
LDX vs MPH	3	0.242	29.50	0.0057

Efficacy – Mean overall change in ADHD core symptoms, Adults, Clinician's Ratings

Comparison	No. of studies	P-value	I^2(%)	τ^2
AMPH vs PBO	3	0.448	0.00	0.0000
MPH vs PBO	13	0.005	57.90	0.0278
ATMX vs PBO	11	0.001	65.40	0.0291

Efficacy – ADHD core symptoms, Children, Parents' Ratings

Comparison	No. of studies	P-value	I^2(%)	τ^2
ATMX vs MPH	4	0.246	27.60	0.0094
MPH vs PBO	10	0.118	36.30	0.0215
ATMX vs PBO	13	0.962	0.00	0.0000
MODA vs PBO	4	0.169	40.40	0.0156

Efficacy – ADHD core symptoms, Adults, Self Ratings

N/A (no comparison with lisdexamfetamine)

Tolerability, Children/adolescents

Comparison	No. of studies	P-value	I^2(%)	τ^2
ATMX vs MPH	7	0.5200	0.00	0.0000
ATMX vs PBO	21	0.9980	0.00	0.0000
MPH vs PBO	22	0.9220	0.00	0.0000
AMPH vs PBO	4	0.5700	0.00	0.0000
MODA vs PBO	6	0.4340	0.00	0.0000
GUA vs PBO	7	0.1120	41.90	0.7755
LDX vs PBO	5	0.4970	0.00	0.0000
LDX vs MPH	3	0.1120	54.20	0.4315
AMPH vs MPH	3	0.9390	0.00	0.0000

Tolerability, Adults

Comparison	No. of studies	P-value	I^2(%)	τ^2
AMPH vs PBO	3	0.5720	0.00	0.0000
MPH vs PBO	15	0.9120	0.00	0.0000
ATMX vs PBO	10	0.4550	0.00	0.0000
BUP vs PBO	3	0.4850	0.00	0.0000
LDX vs PBO	3	0.7200	0.00	0.0000

Functioning - Clinical Global Impression (CGI), Children/adolescents

Comparison	No. of studies	P-value	I^2(%)	τ^2
MPH vs PBO	12	0.0080	57.00	0.2203
ATMX vs PBO	5	0.1310	43.70	0.1266

AMPH vs PBO	3	0.2380	30.30	0.0611
MODA vs PBO	4	0.4590	0.00	0.0000
GUA vs PBO	8	0.1540	34.30	0.0478
LDX vs PBO	5	0.0000	82.80	0.4428
LDX vs MPH	3	0.2370	30.60	0.0326

Functioning - Clinical Global Impression (CGI), Adults

Comparison	No. of studies	P-value	I^2(%)	τ^2
AMPH vs PBO	4	0.1140	49.50	0.1180
MPH vs PBO	7	0.0650	49.40	0.0966

Weight, Children/adolescents

Comparison	No. of studies	P-value	I^2(%)	τ^2
ATMX vs MPH	5	0.0230	64.70	0.0415
ATMX vs PBO	21	0.0000	89.70	0.2871
MPH vs PBO	13	0.0000	94.00	0.4948
MODA vs PBO	3	0.4670	0.00	0.0000
GUA vs PBO	7	0.6950	0.00	0.0000
LDX vs PBO	5	0.0000	96.40	0.4984
LDX vs MPH	3	0.0290	71.80	0.0336

Weight, Adults

Comparison	No. of studies	P-value	I^2(%)	τ^2
AMPH vs PBO	4	0.0000	93.70	0.3760

MPH vs PBO	8	0.0000	73.20	0.0937
ATMX vs PBO	7	0.0000	92.10	0.1680

Systolic Blood Pressure, Children/adolescents

Comparison	No. of studies	P-value	I^2(%)	τ^2
ATMX vs MPH	5	0.3400	11.60	0.0030
ATMX vs PBO	18	0.1060	30.70	0.0128
MPH vs PBO	12	0.8110	0.00	0.0000
MODA vs PBO	4	0.8250	0.00	0.0000
GUA vs PBO	8	0.2260	25.40	0.0068
LDX vs PBO	5	0.2950	18.80	0.0041
LDX vs MPH	3	0.1310	50.70	0.0134

Systolic Blood Pressure, Adults

Comparison	No. of studies	P-value	I^2(%)	τ^2
AMPH vs PBO	4	0.4200	0.00	0.0000
MPH vs PBO	8	0.3180	14.30	0.0046
ATMX vs PBO	6	0.4020	2.20	0.0003

Diastolic Blood Pressure, Children/adolescents

Comparison	No. of studies	P-value	I^2(%)	τ^2
ATMX vs MPH	5	0.4340	0.00	0.0000
ATMX vs PBO	19	0.2460	17.00	0.0060
MPH vs PBO	12	0.4700	0.00	0.0000

MODA vs PBO	4	0.7300	0.00	0.0000
GUA vs PBO	8	0.1110	40.10	0.0134
LDX vs PBO	5	0.0130	68.40	0.0379
LDX vs MPH	3	0.4030	0.00	0.0000

Diastolic Blood Pressure, Adults

Comparison	No. of studies	P-value	I^2(%)	τ^2
AMPH+ vs PBO	4	0.9980	0.00	0.0000
MPH vs PBO	8	0.7730	0.00	0.0000
ATMX vs PBO	5	0.3360	12.20	0.0017

Acceptability, Children/adolescents

Comparison	No. of studies	P-value	I^2(%)	τ^2
ATMX vs MPH	7	0.1910	31.00	0.1046
ATMX vs PBO	22	0.4260	2.50	0.0062
MPH vs PBO	21	0.0140	45.10	0.2160
AMPH vs PBO	4	0.1510	43.40	0.2914
MODA vs PBO	6	0.1230	42.30	0.1406
GUA vs PBO	8	0.4090	2.70	0.0043
LDX vs PBO	5	0.0000	80.60	0.4422
LDX vs MPH	3	0.6210	0.00	0.0000
AMPH vs MPH	3	0.6870	0.00	0.0000

Acceptability, Adults

Comparison	No. of studies	P-value	I^2(%)	τ^2
AMPH vs PBO	3	0.8550	0.00	0.0000
MPH vs PBO	13	0.6870	0.00	0.0000
ATMX vs PBO	10	0.1920	27.40	0.0433
BUP vs PBO	3	0.4000	0.00	0.0000
LDX vs PBO	3	0.4140	0.00	0.0000

NETWORK META-ANALYSES

In each table, the bottom left triangle refers to results in children/adolescents and the top right triangle refers to results in adults. **The estimates must be read from left to right, both in below and above triangle.**

FDA dose analyses, Lisdexamfetamine and other Amphetamines lumped

Efficacy – ADHD core symptoms, Teachers' Ratings

**Atomoxetine**	-	-
**0.54 (0.19;0.90)**	**Methylphenidate**	-
-0.30 (-0.59;0.00)	**-0.84 (-1.03;-0.65)**	**Placebo**

Mean change in symptoms is reported as a standardized mean difference (SMD) along with 95% confidence intervals.
An SMD below 0 favours the medication on the top left vs. the medication on the bottom right in the diagonal.

Efficacy – ADHD core symptoms, Clinicians' Ratings

**Amphetamines**	**-0.34 (-0.67;-0.01)**	-	-	-0.29 (-0.63;0.04)	**-0.79 (-1.09;-0.50)**

**-0.47 (-0.65;-0.29)**	**Atomoxetine**	-	-	0.05 (-0.15;0.25)	**-0.45 (-0.60;-0.31)**
-0.32 (-0.79;0.15)	0.15 (-0.30;0.60)	**Clonidine**	-	-	-
**-0.41 (-0.65;-0.17)**	0.05 (-0.15;0.26)	-0.09 (-0.57;0.38)	**Guanfacine**	-	-
**-0.23 (-0.42;-0.05)**	**0.24 (0.07;0.40)**	0.09 (-0.38;0.55)	0.18 (-0.05;0.41)	**Methylphenidate**	**-0.50 (-0.65;-0.35)**
**-1.02 (-1.18;-0.86)**	**-0.56 (-0.66;-0.45)**	**-0.71 (-1.15;-0.26)**	**-0.61 (-0.79;-0.43)**	**-0.79 (-0.94;-0.64)**	**Placebo**

Mean change in symptoms is reported as a standardized mean difference (SMD) along with 95% confidence intervals. An SMD below 0 favours the medication on the top left vs. the medication on the bottom right in the diagonal. The bottom left triangle refers to results in children/adolescents and the top right triangle to results in adults.

Efficacy – ADHD core symptoms, Parents' Ratings

**Amphetamines**	-	-	-
**-0.47 (-0.77;-0.17)**	**Atomoxetine**	-	-
-0.24 (-0.54;0.07)	**0.23 (0.10;0.37)**	**Methylphenidate**	-
**-1.07 (-1.36;-0.79)**	**-0.60 (-0.71;-0.50)**	**-0.84 (-0.95;-0.72)**	**Placebo**

Mean change in symptoms is reported as a standardized mean difference (SMD) along with 95% confidence intervals. An SMD below 0 favours the medication on the top left vs. the medication on the bottom right in the diagonal.

Efficacy – ADHD core symptoms, Self-Ratings

**Amphetamines**	-0.08 (-0.46,0.30)	-0.04 (-0.42,0.33)	**-0.45 (-0.82,-0.09)**
-	**Atomoxetine**	0.04 (-0.09,0.17)	**-0.37 (-0.47,-0.27)**
-	-	**Methylphenidate**	**-0.41 (-0.50,-0.33)**
-	-	-	**Placebo**

Mean change in symptoms is reported as a standardized mean difference (SMD) along with 95% confidence intervals. An SMD below 0 favours the medication on the top left vs. the medication on the bottom right in the diagonal.

Tolerability

**Amphetamines**	1.46 (0.43;4.88)	-	-	1.42 (0.44;4.58)	**3.40 (1.21;9.59)**
1.54 (0.79,3.02)	**Atomoxetine**	-	-	0.97 (0.46;2.07)	**2.34 (1.25;4.36)**
0.20 (0.02,1.78)	0.13 (0.01,1.17)	**Clonidine**	-	-	-
0.80 (0.31,2.03)	0.52 (0.19,1.38)	3.93 (0.41,37.60)	**Guanfacine**	-	-
1.65 (0.96,2.85)	1.07 (0.57,2.02)	8.13 (0.94,70.48)	2.07 (0.80,5.33)	**Methylphenidate**	**2.40 (1.39;4.16)**
**2.27 (1.33,3.86)**	1.47 (0.83,2.61)	**11.16 (1.36,91.58)**	**2.84 (1.25,6.44)**	1.37 (0.85,2.23)	**Placebo**

Discontinuation due to adverse events is reported for each comparison as odds ratio (OR), along with 95% confidence intervals. An OR above 1 favours the medication on the bottom right vs. the medication on the top left in the diagonal. The bottom left triangle refers to results in children/adolescents and the top right triangle to results in adults.

Functioning - Clinical Global Impression (CGI)

**Amphetamines**	2.00 (0.70;5.71)	-	1.31 (0.64;2.67)	**4.08 (2.34;7.12)**
**3.38 (1.91;5.98)**	**Atomoxetine**	-	0.66 (0.25;1.73)	2.04 (0.84;4.97)
**2.24 (1.27;3.96)**	0.66 (0.33;1.32)	**Guanfacine**	-	-
1.39 (0.91;2.11)	**0.41 (0.22;0.75)**	0.62 (0.35;1.09)	**Methylphenidate**	**3.11 (2.00;4.86)**
**7.75 (5.48;10.97)**	**2.29 (1.36;3.85)**	**3.45 (2.20;5.42)**	**5.59 (3.96;7.90)**	**Placebo**

Improvement in clinical global functioning is reported for each comparison as odds ratio (OR), along with 95% confidence intervals. An OR above 1 favours the medication on the top left vs. the medication on the bottom right in the diagonal. The bottom left triangle refers to results in children/adolescents and the top right triangle to results in adults.

Weight (Kgs)

**Amphetamines**	0.18 (-0.35;0.70)	-	**0.65 (0.12;1.17)**	-0.11 (-0.52;0.30)
0.14 (-0.42;0.69)	**Atomoxetine**	-	**0.47 (0.04;0.91)**	-0.28 (-0.62;0.05)
**-0.80 (-1.50;-0.10)**	**-0.93 (-1.56;-0.31)**	**Guanfacine**	-	-
0.06 (-0.45;0.56)	-0.08 (-0.51;0.36)	**0.86 (0.23;1.48)**	**Methylphenidate**	**-0.76 (-1.08;-0.43)**
**-0.70 (-1.16;-0.25)**	**-0.84 (-1.17;-0.51)**	0.09 (-0.43;0.62)	**-0.76 (-1.10;-0.43)**	**Placebo**

Mean change in body weight is reported as a standardized mean difference (SMD) along with 95% confidence intervals. An SMD below 0 favours the medication on the bottom right vs. the medication on the top left in the diagonal. The bottom left triangle refers to results in children/adolescents and the top right triangle to results in adults.

Systolic Blood Pressure

**Amphetamines**	-0.01 (-0.26,0.24)	-0.08 (-0.33,0.17)	0.10 (-0.11,0.30)
-0.02 (-0.16,0.11)	**Atomoxetine**	-0.07 (-0.28,0.13)	0.11 (-0.04,0.25)
0.00 (-0.11,0.12)	0.02 (-0.11,0.15)	**Methylphenidate**	**0.18 (0.03,0.32)**
0.09 (-0.00,0.19)	**0.12 (0.02,0.22)**	0.09 (-0.01,0.19)	**Placebo**

Mean change in systolic blood pressure is reported as a standardized mean difference (SMD) along with 95% confidence intervals. An SMD above 0 indicates an increase in blood pressure values and favours the medication on the bottom right vs. the medication on the top left in the diagonal. The bottom left triangle refers to results in children/adolescents and the top right triangle to results in adults.

Diastolic Blood Pressure

**Amphetamines**	**-0.24 (-0.45,-0.03)**	**-0.25 (-0.46,-0.03)**	-0.05 (-0.23,0.13)

-0.06 (-0.20,0.08)	**Atomoxetine**	-0.01 (-0.17,0.15)	**0.19 (0.08,0.30)**
-0.03 (-0.14,0.09)	0.03 (-0.09,0.16)	**Methylphenidate**	**0.20 (0.08,0.32)**
**0.21 (0.12,0.31)**	**0.27 (0.17,0.37)**	**0.24 (0.14,0.34)**	**Placebo**

Mean change in diastolic blood pressure is reported as a standardized mean difference (SMD) along with 95% confidence intervals. An SMD above 0 indicates an increase in blood pressure values and favours the medication on the bottom right vs. the medication on the top left in the diagonal. The bottom left triangle refers to results in children/adolescents and the top right triangle to results in adults.

Acceptability

**Amphetamines**	0.60 (0.36,1.00)	-	-	0.72 (0.44,1.20)	0.76 (0.50,1.17)
0.92 (0.59,1.41)	**Atomoxetine**	-	-	1.21 (0.82,1.79)	1.28 (0.95,1.72)
1.29 (0.48,3.45)	1.41 (0.53,3.76)	**Clonidine**	-	-	-
0.99 (0.58,1.69)	1.08 (0.63,1.84)	0.77 (0.28,2.12)	**Guanfacine**	-	-
1.12 (0.77,1.63)	1.22 (0.83,1.80)	0.87 (0.33,2.29)	1.13 (0.68,1.88)	**Methylphenidate**	1.06 (0.80,1.39)
0.78 (0.56,1.10)	0.86 (0.61,1.19)	0.61 (0.24,1.53)	0.79 (0.52,1.21)	**0.70 (0.53,0.93)**	**Placebo**

Discontinuation due to any reason is reported for each comparison as odds ratio (OR), along with 95% confidence intervals. An OR below 1 favours the medication on the top left vs. the medication on the bottom right in the diagonal. The bottom left triangle refers to results in children/adolescents and the top right triangle to results in adults.

Inclusive dose analyses, Lisdexamfetamine and other Amphetamines lumped

Efficacy – ADHD core symptoms, Teachers' Ratings

**Atomoxetine**	-	-	-	-	-
-0.14 (-0.88;0.61)	**Bupropion**	-	-	-	-
0.24 (-0.71;1.20)	0.38 (-0.74;1.50)	**Guanfacine**	-	-	-
0.36 (-0.07;0.79)	0.49 (-0.11;1.10)	0.12 (-0.82;1.05)	**Methylphenidate**	-	-
0.33 (-0.15;0.80)	0.46 (-0.27;1.19)	0.09 (-0.87;1.05)	-0.03 (-0.43;0.37)	**Modafinil**	-
**-0.39 (-0.72;-0.06)**	-0.26 (-0.92;0.41)	-0.63 (-1.53;0.26)	**-0.75 (-1.02;-0.48)**	**-0.72 (-1.06;-0.38)**	**Placebo**

Mean change in symptoms is reported as a standardized mean difference (SMD) along with 95% confidence intervals. An SMD below 0 favours the medication on the top left vs. the medication on the bottom right in the diagonal.

Efficacy – ADHD core symptoms, Clinicians' Ratings

**Amphetamines**	**-0.30 (-0.50,-0.11)**	-0.32 (-0.70,0.05)	-	-	**-0.31 (-0.51,-0.11)**	**-0.95 (-1.34,-0.55)**	**-0.79 (-0.95,-0.62)**
**-0.46 (-0.63,-0.29)**	**Atomoxetine**	-0.02 (-0.38,0.33)	-	-	-0.01 (-0.15,0.14)	**-0.64 (-1.02,-0.27)**	**-0.49 (-0.59,-0.38)**
-0.07 (-0.80,0.65)	0.38 (-0.33,1.10)	**Bupropion**	-	-	0.01 (-0.34,0.37)	**-0.62 (-1.11,-0.13)**	**-0.46 (-0.80,-0.12)**
-0.31 (-0.78,0.15)	0.14 (-0.30,0.59)	-0.24 (-1.08,0.60)	**Clonidine**	-	-	-	-
**-0.30 (-0.52,-0.09)**	0.16 (-0.02,0.33)	-0.23 (-0.96,0.50)	0.01 (-0.45,0.47)	**Guanfacine**	-	-	-
**-0.25 (-0.43,-0.08)**	0.21 (0.06,0.35)	-0.18 (-0.88,0.53)	0.06 (-0.39,0.52)	0.05 (-0.15,0.25)	**Methylphenidate**	**-0.63 (-1.01,-0.26)**	**-0.48 (-0.58,-0.37)**
**-0.41 (-0.66,-0.15)**	0.05 (-0.17,0.27)	-0.33 (-1.07,0.41)	-0.09 (-0.57,0.39)	-0.10 (-0.36,0.15)	-0.15 (-0.39,0.08)	**Modafinil**	0.16 (-0.20,0.51)
**-1.02 (-1.18,-0.86)**	**-0.56 (-0.65,-0.47)**	**-0.95 (-1.66,-0.23)**	**-0.71 (-1.14,-0.27)**	**-0.72 (-0.87,-0.57)**	**-0.77 (-0.90,-0.63)**	**-0.61 (-0.82,-0.41)**	**Placebo**

Mean change in symptoms is reported as a standardized mean difference (SMD) along with 95% confidence intervals. An SMD below 0 favours the medication on the top left vs. the medication on the bottom right in the diagonal. The bottom left triangle refers to results in children/adolescents and the top right triangle to results in adults.

Efficacy – ADHD core symptoms, Parents' Ratings

**Amphetamines**	-	-	-	-	-	-
**-0.46 (-0.78;-0.15)**	**Atomoxetine**	-	-	-	-	-
**-1.39 (-2.13;-0.64)**	**-0.93 (-1.61;-0.24)**	**Bupropion**	-	-	-	-
**-0.85 (-1.59;-0.10)**	-0.39 (-1.08;0.30)	0.54 (-0.43;1.51)	**Guanfacine**	-	-	-
-0.31 (-0.63;0.01)	**0.15 (0.03;0.26)**	**1.07 (0.40;1.75)**	0.53 (-0.16;1.23)	**Methylphenidate**	-	-
**-0.61 (-0.95;-0.27)**	-0.15 (-0.34;0.03)	**0.77 (0.07;1.47)**	0.23 (-0.47;0.93)	**-0.30 (-0.49;-0.11)**	**Modafinil**	-
**-1.07 (-1.37;-0.77)**	**-0.61 (-0.71;-0.52)**	0.31 (-0.37;1.00)	-0.23 (-0.91;0.46)	**-0.76 (-0.87;-0.65)**	**-0.46 (-0.62;-0.30)**	**Placebo**

Mean change in symptoms is reported as a standardized mean difference (SMD) along with 95% confidence intervals. An SMD below 0 favours the medication on the top left vs. the medication on the bottom right in the diagonal.

Efficacy – ADHD core symptoms, Self-Ratings

**Amphetamines**	-0.09 (-0.34,0.16)	-0.17 (-0.56,0.22)	0.24 (-0.37,0.84)	-0.08 (-0.32,0.17)	**-0.32 (-0.64,-0.01)**	**-0.47 (-0.70,-0.24)**
-	**Atomoxetine**	-0.08 (-0.40,0.25)	0.33 (-0.28,0.94)	0.02 (-0.10,0.13)	-0.23 (-0.48,0.02)	**-0.38 (-0.47,-0.29)**
-	-	**Bupropion**	0.41 (-0.27,1.09)	0.09 (-0.23,0.42)	-0.15 (-0.54,0.24)	-0.30 (-0.61,0.01)
-	-	-	**Guanfacine**	-0.31 (-0.92,0.29)	-0.56 (-1.20,0.08)	**-0.71 (-1.31,-0.10)**
-	-	-	-	**Methylphenidate**	-0.25 (-0.49,0.00)	**-0.39 (-0.47,-0.31)**
-	-	-	-	-	**Modafinil**	-0.15 (-0.38,0.09)
-	-	-	-	-	-	**Placebo**

Mean change in symptoms is reported as a standardized mean difference (SMD) along with 95% confidence intervals. An SMD below 0 favours the medication on the top left vs. the medication on the bottom right in the diagonal.

Tolerability

**Amphetamines**	1.29 (0.56;2.97)	1.25 (0.14;10.79)	-	-	1.27 (0.55;2.92)	0.80 (0.25;2.59)	**3.21 (1.58;6.52)**
1.33 (0.72;2.47)	**Atomoxetine**	0.97 (0.12;7.76)	-	-	0.98 (0.55;1.76)	0.62 (0.22;1.74)	**2.48 (1.61;3.84)**
1.59 (0.17;14.62)	1.20 (0.13;10.87)	**Bupropion**	-	-	1.02 (0.13;8.16)	0.64 (0.07;6.03)	2.57 (0.34;19.69)
0.52 (0.08;3.41)	0.39 (0.06;2.50)	0.33 (0.02;5.52)	**Clonidine**	-	-	-	-
0.85 (0.34;2.14)	0.64 (0.26;1.60)	0.53 (0.05;5.41)	1.62 (0.22;11.72)	**Guanfacine**	-	-	-
**1.73 (1.02;2.95)**	1.30 (0.78;2.17)	1.09 (0.12;9.54)	3.30 (0.53;20.65)	2.04 (0.83;5.01)	**Methylphenidate**	0.63 (0.22;1.78)	**2.53 (1.62;3.94)**
1.69 (0.62;4.60)	1.27 (0.47;3.40)	1.06 (0.10;11.08)	3.23 (0.43;24.05)	1.99 (0.62;6.36)	0.98 (0.37;2.57)	**Modafinil**	**4.01 (1.57;10.23)**
**2.29 (1.35;3.89)**	**1.72 (1.09;2.72)**	1.44 (0.16;12.73)	4.37 (0.72;26.58)	**2.69 (1.22;5.95)**	1.32 (0.84;2.09)	1.36 (0.56;3.25)	**Placebo**

Discontinuation due to adverse events is reported for each comparison as odds ratio (OR), along with 95% confidence intervals. An OR above 1 favours the medication on the bottom right vs. the medication on the top left in the diagonal. The bottom left triangle refers to results in children/adolescents and the top right triangle to results in adults.

Functioning - Clinical Global Impression (CGI)

**Amphetamines**	**2.44 (1.02;5.79)**	1.47 (0.63,3.43)	-	-	**1.66 (1.04;2.65)**	**5.47 (2.48;12.08)**	**4.87 (3.48;6.81)**
**3.12 (1.91;5.10)**	**Atomoxetine**	0.60 (0.38;2.04)	-	-	0.68 (0.29;1.61)	2.25 (0.77;6.59)	2.00 (0.90;4.46)
-	-	**Bupropion**	-	-	1.13 (0.49,2.59)	3.72 (1.29,10.76)	**3.31 (1.51,7.24)**
2.81 (0.91;8.70)	0.90 (0.28;2.94)	-	**Clonidine**	-	-	-	-
**2.40 (1.52;3.80)**	0.77 (0.46;1.28)	-	0.85 (0.27;2.70)	**Guanfacine**	-	-	-
1.38 (0.94;2.03)	**0.44 (0.26;0.75)**	-	0.49 (0.16;1.47)	**0.57 (0.36;0.91)**	**Methylphenidate**	**3.29 (1.49;7.29)**	**2.93 (2.08;4.12)**

**2.46 (1.35;4.47)**	0.79 (0.41;1.54)	-	0.88 (0.26;2.94	1.02 (0.56;1.88)	1.78 (0.98;3.22)	**Modafinil**	0.89 (0.43;1.82)
**7.92 (5.76;10.89)**	**2.54 (1.65;3.89)**	-	2.82 (0.94;8.41)	**3.30 (2.35;4.62)**	**5.73 (4.18;7.85)**	**3.22 (1.94;5.34)**	**Placebo**

Improvement in clinical global functioning is reported for each comparison as odds ratio (OR), along with 95% confidence intervals. An OR above 1 favours the medication on the top left vs. the medication on the bottom right in the diagonal. The bottom left triangle refers to results in children/adolescents and the top right triangle to results in adults.

Weight (Kgs)

**Amphetamines**	-0.09 (-0.59;0.42)	0.18 (-0.82;1.18)	-	-	0.26 (-0.25;0.76)	-	**-0.60 (-0.98;-0.22)**
0.18 (-0.28;0.63)	**Atomoxetine**	0.27 (-0.72;1.25)	-	-	0.34 (-0.11;0.80)	-	**-0.52 (-0.85;-0.18)**
-	-	**Bupropion**	-	-	0.08 (-0.90;1.06)	-	-0.78 (-1.71;0.14)
-0.82 (-1.88;0.24)	-1.00 (-2.01;0.01)	-	**Clonidine**	-	-	-	-
**-0.72 (-1.28;-0.16)**	**-0.90 (-1.33;-0.46)**	-	0.10 (-0.96;1.17)	**Guanfacine**	-	-	-
0.03 (-0.41;0.47)	-0.15 (-0.46;0.17)	-	0.85 (-0.14;1.85)	**0.75 (0.28;1.22)**	**Methylphenidate**	-	**-0.86 (-1.20;-0.53)**
0.23 (-0.50;0.96)	0.05 (-0.60;0.70)	-	1.05 (-0.12;2.22)	**0.95 (0.22;1.67)**	0.20 (-0.47;0.87)	**Modafinil**	-
**-0.70 (-1.10;-0.29)**	**-0.88 (-1.10;-0.65)**	-	0.12 (-0.87;1.12)	0.02 (-0.37;0.41)	**-0.73 (-1.00;-0.46)**	**-0.93 (-1.54;-0.31)**	**Placebo**

Mean change in body weight is reported as a standardized mean difference (SMD) along with 95% confidence intervals. An SMD below 0 favours the medication on the bottom right vs. the medication on the top left in the diagonal. The bottom left triangle refers to results in children/adolescents and the top right triangle to results in adults.

Systolic Blood Pressure

**Amphetamines**	-0.08 (-0.25,0.10)	-0.15 (-0.91,0.61)	-0.06 (-0.24,0.13)	-	0.12 (-0.01,0.25)
-0.01 (-0.14,0.11)	**Atomoxetine**	-0.08 (-0.83,0.68)	0.02 (-0.15,0.19)	-	**0.19 (0.08,0.31)**

-	-	**Bupropion**	0.10 (-0.66,0.86)	-	0.27 (-0.48,1.02)
-0.01 (-0.13,0.10)	0.00 (-0.10,0.10)	-	**Methylphenidate**	-	**0.17 (0.05,0.30)**
0.05 (-0.14,0.23)	0.06 (-0.12,0.24)	-	0.06 (-0.12,0.24)	**Modafinil**	-
**0.11 (0.01,0.20)**	**0.12 (0.04,0.20)**	**-**	**0.12 (0.03,0.21)**	0.06 (-0.10,0.22)	**Placebo**

Mean change in systolic blood pressure is reported as a standardized mean difference (SMD) along with 95% confidence intervals. An SMD above 0 indicates an increase in blood pressure values and favours the medication on the bottom right vs. the medication on the top left in the diagonal. The bottom left triangle refers to results in children/adolescents and the top right triangle to results in adults.

Diastolic Blood Pressure

**Amphetamines**	**-0.21 (-0.36,-0.06)**	-0.16 (-0.90,0.59)	**-0.16 (-0.32,0.00)**	-	0.04 (-0.08,0.15)
-0.03 (-0.15,0.09)	**Atomoxetine**	0.06 (-0.68,0.80)	0.05 (-0.10,0.20)	-	**0.25 (0.16,0.35)**
-	-	**Bupropion**	-0.00 (-0.75,0.74)	-	0.20 (-0.54,0.93)
-0.01 (-0.12,0.10)	0.02 (-0.08,0.12)	-	**Methylphenidate**	-	**0.20 (0.09,0.31)**
**0.24 (0.06,0.43)**	**0.28 (0.10,0.45)**	**-**	**0.25 (0.07,0.43)**	**Modafinil**	-
**0.21 (0.11,0.31)**	**0.24 (0.17,0.32)**	**-**	**0.22 (0.13,0.31)**	-0.03 (-0.19,0.13)	**Placebo**

Mean change in diastolic blood pressure is reported as a standardized mean difference (SMD) along with 95% confidence intervals. An SMD above 0 indicates an increase in blood pressure values and favours the medication on the bottom right vs. the medication on the top left in the diagonal. The bottom left triangle refers to results in children/adolescents and the top right triangle to results in adults.

Acceptability

**Amphetamines**	**0.48 (0.32,0.72)**	0.60 (0.28,1.30)	-	-	**0.55 (0.36,0.84)**	**0.35 (0.17,0.71)**	**0.67 (0.48,0.94)**
0.85 (0.59,1.24)	**Atomoxetine**	1.25 (0.60,2.61)	-	-	1.15 (0.83,1.61)	0.74 (0.38,1.42)	**1.40 (1.12,1.76)**

0.88 (0.21,3.72)	1.03 (0.25,4.31)	**Bupropion**	-	-	0.92 (0.44,1.93)	0.59 (0.23,1.49)	1.12 (0.56,2.25)
1.30 (0.55,3.07)	1.53 (0.67,3.51)	1.48 (0.29,7.52)	**Clonidine**	-	-	-	-
0.96 (0.58,1.59)	1.13 (0.71,1.78)	1.09 (0.25,4.76)	0.74 (0.30,1.80)	**Guanfacine**	-	-	-
1.13 (0.80,1.60)	1.33 (0.98,1.80)	1.29 (0.32,5.25)	0.87 (0.38,2.00)	1.18 (0.74,1.88)	**Methylphenidate**	0.64 (0.33,1.24)	1.22 (0.94,1.57)
1.12 (0.65,1.95)	1.31 (0.78,2.20)	1.27 (0.29,5.64)	0.86 (0.34,2.16)	1.17 (0.64,2.13)	0.99 (0.59,1.66)	**Modafinil**	**1.91 (1.03,3.53)**
0.78 (0.57,1.07)	0.91 (0.72,1.16)	0.88 (0.21,3.65)	0.60 (0.27,1.32)	0.81 (0.55,1.20)	**0.69 (0.53,0.89)**	0.69 (0.44,1.10)	**Placebo**

Discontinuation due to any reason is reported for each comparison as odds ratio (OR), along with 95% confidence intervals. An OR below 1 favours the medication on the top left vs. the medication on the bottom right in the diagonal. The bottom left triangle refers to results in children/adolescents and the top right triangle to results in adults.

Main dose analyses, separating Lisdexamfetamine from other Amphetamines

Efficacy – ADHD core symptoms, Teachers' Ratings

No NMA since no studies available

Efficacy – ADHD core symptoms, Clinicians' Ratings

**Amphetamines**	-0.29 (-0.58;0.01)	-0.28 (-0.76;0.20)	-	-	0.12 (-0.30;0.55)	-0.24 (-0.54;0.06)	**-0.90 (-1.42;-0.38)**	**-0.74 (-1.00;-0.48)**
-0.26 (-0.60;0.08)	**Atomoxetine**	0.01 (-0.42;0.43)	-	-	**0.41 (0.05;0.77)**	0.04 (-0.15;0.23)	**-0.61 (-1.08;-0.14)**	**-0.45 (-0.59;-0.32)**
0.16 (-0.65;0.97)	0.42 (-0.32;1.16)	**Bupropion**	-	-	0.40 (-0.12;0.93)	0.04 (-0.39;0.46)	**-0.62 (-1.22;-0.02)**	**-0.46 (-0.86;-0.06)**
-0.10 (-0.68;0.47)	0.16 (-0.31;0.63)	-0.26 (-1.13;0.60)	**Clonidine**	-	-	-	-	-
-0.14 (-0.52;0.24)	0.12 (-0.08;0.33)	-0.30 (-1.05;0.46)	-0.03 (-0.53;0.46)	**Guanfacine**	-	-	-	-

0.27 (-0.12;0.66)	**0.54 (0.32;0.75)**	0.11 (-0.63;0.86)	0.38 (-0.12;0.88)	**0.41 (0.15;0.67)**	**Lisdexamfetamine**	-0.37 (-0.73;-0.00)	**-1.02 (-1.58;-0.46)**	**-0.86 (-1.20;-0.53)**
-0.02 (-0.39;0.35)	**0.24 (0.07;0.42)**	-0.18 (-0.90;0.54)	0.09 (-0.40;0.57)	0.12 (-0.11;0.35)	**-0.29 (-0.50;-0.09)**	**Methylphenidate**	**-0.65 (-1.13;-0.18)**	**-0.50 (-0.64;-0.35)**
-0.18 (-0.59;0.22)	0.08 (-0.16;0.32)	-0.34 (-1.11;0.42)	-0.08 (-0.59;0.43)	-0.05 (-0.32;0.23)	**-0.46 (-0.74;-0.18)**	-0.17 (-0.42;0.09)	**Modafinil**	0.16 (-0.29;0.61)
**-0.81 (-1.15;-0.47)**	**-0.55 (-0.65;-0.44)**	**-0.97 (-1.70;-0.23)**	**-0.71 (-1.17;-0.25)**	**-0.67 (-0.84;-0.50)**	**-1.08 (-1.28;-0.89)**	**-0.79 (-0.94;-0.64)**	**-0.62 (-0.84;-0.41)**	**Placebo**

Mean change in symptoms is reported as a standardized mean difference (SMD) along with 95% confidence intervals. An SMD below 0 favours the medication on the top left vs. the medication on the bottom right in the diagonal. The bottom left triangle refers to results in children/adolescents and the top right triangle to results in adults.

Efficacy – ADHD core symptoms, Parents' Ratings

**Atomoxetine**	-	-	-	-	-	-
**-0.84 (-1.52;-0.16)**	**Bupropion**	--	-	-	-	-
-0.37 (-1.06;0.31)	0.46 (-0.49;1.42)	**Guanfacine**	-	-	-	-
**0.47 (0.17;0.77)**	**1.31 (0.58;2.05)**	**0.85 (0.12;1.58)**	**Lisdexamfetamine**	-	-	-
**0.23 (0.10;0.37)**	**1.07 (0.40;1.74)**	0.61 (-0.07;1.29)	-0.24 (-0.54;0.07)	**Methylphenidate**	-	-
-0.14 (-0.32;0.04)	0.70 (0.00;1.39)	0.23 (-0.46;0.92)	**-0.61 (-0.93;-0.29)**	**-0.38 (-0.56;-0.19)**	**Modafinil**	-
**-0.60 (-0.71;-0.50)**	0.24 (-0.44;0.92)	-0.23 (-0.90;0.45)	**-1.07 (-1.36;-0.79)**	**-0.84 (-0.95;-0.72)**	**-0.46 (-0.61;-0.31)**	**Placebo**

Mean change in symptoms is reported as a standardized mean difference (SMD) along with 95% confidence intervals. An SMD below 0 favours the medication on the top left vs. the medication on the bottom right in the diagonal. The bottom left triangle refers to results in children/adolescents and the top right triangle to results in adults.

Efficacy – ADHD core symptoms, Self-Ratings

No studies on Lisdexamfetamine were included, thus effects were identical to those in the analysis of Lisdexamfetamine and Other Amphetamines lumped.

Tolerability

**Amphetamines**	1.57 (0.49;5.03)	1.44 (0.15;14.22)	-	-	1.34 (0.28;6.48)	1.53 (0.49;4.71)	0.91 (0.21;3.97)	**3.66 (1.36;9.87)**
1.26 (0.54;2.93)	**Atomoxetine**	0.92 (0.11;7.92)	-	-	0.85 (0.22;3.37)	0.97 (0.46;2.06)	0.58 (0.17;2.03)	**2.34 (1.26;4.34)**
1.19 (0.12;11.89)	0.95 (0.10;8.82)	**Bupropion**	-	-	0.93 (0.08;10.24)	1.06 (0.13;8.97)	0.63 (0.06;6.53)	2.55 (0.32;20.04)
0.40 (0.06;2.87)	0.32 (0.05;2.09)	0.34 (0.02;5.60)	**Clonidine**	-	-	-	-	-
0.69 (0.23;2.04)	0.55 (0.21;1.43)	0.58 (0.06;5.85)	1.71 (0.24;12.29)	**Guanfacine**	-	-	-	-
0.68 (0.26;1.77)	0.54 (0.24;1.21)	0.57 (0.06;5.34)	1.68 (0.25;11.26)	0.99 (0.37;2.65)	**Lisdexamfetamine**	1.14 (0.30;4.35)	0.68 (0.13;3.50)	2.74 (0.80;9.30)
1.24 (0.53;2.89)	0.98 (0.51;1.87)	1.04 (0.12;9.06)	3.07 (0.50;18.99)	1.80 (0.71;4.53)	1.82 (0.97;3.43)	**Methylphenidate**	0.60 (0.18;2.01)	**2.40 (1.39;4.14)**
1.36 (0.43;4.28)	1.08 (0.38;3.01)	1.14 (0.11;11.80)	3.36 (0.46;24.81)	1.97 (0.62;6.20)	1.99 (0.69;5.75)	1.10 (0.41;2.92)	**Modafinil**	**4.01 (1.36;11.85)**
1.83 (0.84;4.02)	1.45 (0.82;2.58)	1.53 (0.17;13.52)	4.54 (0.75;27.31)	**2.66 (1.20;5.87)**	**2.69 (1.40;5.16)**	1.48 (0.92;2.38)	1.35 (0.57;3.22)	**Placebo**

Discontinuation due to adverse events is reported for each comparison as odds ratio (OR), along with 95% confidence intervals. An OR above 1 favours the medication on the bottom right vs. the medication on the top left in the diagonal. The bottom left triangle refers to results in children/adolescents and the top right triangle to results in adults.

Functioning - Clinical Global Impression (CGI)

**Amphetamines**	2.38 (0.82;6.90)	1.39 (0.48;4.04)	-	-	1.01 (0.42;2.45)	1.55 (0.78;3.08)	**5.52 (1.88;16.17)**	**4.91 (2.87;8.38)**
**3.41 (1.77;6.58)**	**Atomoxetine**	0.58 (0.16;2.14)	-	-	0.42 (0.13;1.35)	0.65 (0.24;1.79)	2.31 (0.62;8.58)	2.06 (0.82;5.17)
-	-	**Bupropion**	-	-	0.73 (0.23;2.32)	1.11 (0.41;3.02)	**3.96 (1.06;14.74)**	**3.52 (1.39;8.90)**
2.82 (0.79;10.02)	0.83 (0.24;2.89)	-	**Clonidine**	-	-	-	-	-
**2.15 (1.05;4.41)**	0.63 (0.32;1.25)	-	0.77 (0.23;2.59)	**Guanfacine**	-	-	-	-

1.02 (0.50;2.06)	**0.30 (0.15;0.58)**	-	0.36 (0.11;1.20)	**0.47 (0.26;0.87)**	**Lisdexamfetamine**	1.53 (0.67;3.51)	**5.45 (1.69;17.57)**	**4.85 (2.40;9.83)**
1.40 (0.73;2.70)	**0.41 (0.22;0.77)**	-	0.50 (0.16;1.56)	0.65 (0.37;1.14)	1.38 (0.87;2.18)	**Methylphenidate**	**3.56 (1.26;10.02)**	**3.17 (2.03;4.95)**
**2.43 (1.11;5.31)**	0.71 (0.34;1.51)	-	0.86 (0.25;3.04)	1.13 (0.56;2.26)	**2.39 (1.21;4.73)**	1.73 (0.92;3.28)	**Modafinil**	0.89 (0.35;2.26)
**7.82 (4.43;13.81)**	**2.29 (1.35;3.88)**	-	2.78 (0.89;8.67)	**3.63 (2.34;5.64)**	**7.69 (5.03;11.75)**	**5.58 (3.95;7.88)**	**3.22 (1.88;5.49)**	**Placebo**

Improvement in clinical global functioning is reported for each comparison as odds ratio (OR), along with 95% confidence intervals. An OR above 1 favours the medication on the top left vs. the medication on the bottom right in the diagonal. The bottom left triangle refers to results in children/adolescents and the top right triangle to results in adults.

Weight (Kgs)

**Amphetamines**	-0.60 (-1.23;0.04)	-0.11 (-1.13;0.91)	-	-	**-0.81 (-1.60;-0.02)**	-0.14 (-0.76;0.48)	-	**-0.89 (-1.35;-0.42)**
0.37 (-0.85;1.58)	**Atomoxetine**	0.49 (-0.52;1.50)	-	-	-0.21 (-0.98;0.56)	0.45 (-0.10;1.01)	-	-0.29 (-0.72;0.14)
-	-	**Bupropion**	-	-	-0.70 (-1.81;0.40)	-0.04 (-1.03;0.96)	-	-0.78 (-1.69;0.13)
-0.58 (-2.17;1.02)	-0.94 (-2.06;0.18)	-	**Clonidine**	-	-	-	-	-
-0.57 (-1.85;0.71)	**-0.94 (-1.55;-0.33)**	-	0.01 (-1.19;1.21)	**Guanfacine**	-	-	-	-
0.27 (-1.00;1.54)	-0.10 (-0.67;0.48)	-	0.85 (-0.32;2.02)	**0.84 (0.13;1.55)**	**Lisdexamfetamine**	0.67 (-0.09;1.42)	-	-0.08 (-0.71;0.56)
0.30 (-0.92;1.52)	-0.07 (-0.49;0.36)	-	0.88 (-0.20;1.96)	**0.87 (0.26;1.48)**	0.03 (-0.49;0.55)	**Methylphenidate**	-	**-0.74 (-1.16;-0.33)**
0.45 (-0.90;1.80)	0.09 (-0.66;0.83)	-	1.03 (-0.24;2.30)	**1.02 (0.17;1.87)**	0.18 (-0.65;1.01)	0.15 (-0.60;0.90)	**Modafinil**	-
-0.47 (-1.65;0.70)	**-0.84 (-1.16;-0.52)**	-	0.10 (-0.98;1.18)	0.09 (-0.42;0.61)	**-0.75 (-1.23;-0.26)**	**-0.78 (-1.11;-0.45)**	**-0.93 (-1.60;-0.26)**	**Placebo**

Mean change in body weight is reported as a standardized mean difference (SMD) along with 95% confidence intervals. An SMD below 0 favours the medication on the bottom right vs. the medication on the top left in the diagonal. The bottom left triangle refers to results in children/adolescents and the top right triangle to results in adults.

Systolic Blood Pressure

**Amphetamines**	0.05 (-0.15,0.25)	-0.11 (-0.87,0.65)	0.16 (-0.11,0.43)	-0.02 (-0.22,0.19)	-	**0.16 (0.00,0.31)**
-0.14 (-0.33,0.05)	**Atomoxetine**	-0.16 (-0.92,0.59)	0.11 (-0.14,0.36)	-0.07 (-0.24,0.11)	-	0.11 (-0.01,0.23)
-	-	**Bupropion**	0.27 (-0.51,1.04)	0.10 (-0.66,0.85)	-	0.27 (-0.47,1.01)
-0.16 (-0.36,0.04)	-0.02 (-0.16,0.12)	-	**Lisdexamfetamine**	-0.17 (-0.43,0.08)	-	0.00 (-0.22,0.22)
-0.12 (-0.32,0.07)	0.02 (-0.11,0.14)	-	0.04 (-0.08,0.15)	**Methylphenidate**	-	**0.17 (0.05,0.30)**
-0.08 (-0.30,0.15)	0.06 (-0.12,0.24)	-	0.08 (-0.10,0.27)	0.05 (-0.13,0.23)	**Modafinil**	-
-0.02 (-0.18,0.15)	**0.12 (0.03,0.22)**	-	**0.14 (0.03,0.25)**	**0.11 (0.01,0.20)**	0.06 (-0.09,0.21)	**Placebo**

Mean change in systolic blood pressure is reported as a standardized mean difference (SMD) along with 95% confidence intervals. An SMD above 0 indicates an increase in blood pressure values and favours the medication on the bottom right vs. the medication on the top left in the diagonal. The bottom left triangle refers to results in children/adolescents and the top right triangle to results in adults.

Diastolic Blood Pressure

**Amphetamines**	-0.12 (-0.29,0.06)	-0.12 (-0.87,0.63)	0.15 (-0.10,0.39)	-0.12 (-0.31,0.07)	-	0.08 (-0.07,0.22)
-0.11 (-0.30,0.09)	**Atomoxetine**	-0.00 (-0.75,0.74)	0.26 (0.03,0.49)	-0.01 (-0.17,0.15)	-	**0.19 (0.08,0.30)**
-	-	**Bupropion**	0.27 (-0.50,1.03)	-0.00 (-0.75,0.74)	-	0.20 (-0.54,0.93)
-0.06 (-0.27,0.14)	0.04 (-0.10,0.19)	-	**Lisdexamfetamine**	**-0.27 (-0.50,-0.03)**	-	-0.07 (-0.27,0.14)
-0.08 (-0.27,0.12)	0.03 (-0.09,0.15)	-	-0.01 (-0.13,0.11)	**Methylphenidate**	-	**0.20 (0.08,0.32)**
0.20 (-0.03,0.43)	**0.31 (0.12,0.49)**	-	**0.26 (0.07,0.46)**	**0.28 (0.09,0.46)**	**Modafinil**	-
0.17 (-0.00,0.34)	**0.28 (0.18,0.37)**	-	**0.23 (0.12,0.35)**	**0.25 (0.15,0.34)**	-0.03 (-0.18,0.12)	**Placebo**

Mean change in diastolic blood pressure is reported as a standardized mean difference (SMD) along with 95% confidence intervals. An SMD above 0 indicates an increase in blood pressure values and favours the medication on the bottom right vs. the medication on the top left in the diagonal. The bottom left triangle refers to results in children/adolescents and the top right triangle to results in adults.

Acceptability

**Amphetamines**	**0.49 (0.28,0.83)**	0.56 (0.24,1.27)	-	-	0.80 (0.41,1.58)	0.59 (0.35,1.00)	**0.33 (0.15,0.70)**	**0.62 (0.40,0.98)**
1.06 (0.58,1.93)	**Atomoxetine**	1.14 (0.54,2.43)	-	-	1.65 (0.92,2.96)	1.21 (0.82,1.78)	0.67 (0.34,1.32)	1.28 (0.96,1.72)
1.03 (0.22,4.77)	0.97 (0.22,4.21)	**Bupropion**	-	-	1.44 (0.61,3.40)	1.06 (0.50,2.24)	0.59 (0.23,1.48)	1.12 (0.56,2.24)
1.54 (0.56,4.25)	1.45 (0.59,3.61)	1.49 (0.28,7.91)	**Clonidine**	-	-	-	-	-
1.12 (0.56,2.25)	1.06 (0.62,1.80)	1.09 (0.24,4.87)	0.73 (0.28,1.88)	**Guanfacine**	-	-	-	-
1.27 (0.64,2.51)	1.20 (0.72,2.00)	1.24 (0.28,5.42)	0.83 (0.32,2.11)	1.13 (0.63,2.03)	**Lisdexamfetamine**	0.74 (0.41,1.31)	**0.41 (0.19,0.90)**	0.78 (0.47,1.29)
1.33 (0.73,2.41)	1.25 (0.85,1.85)	1.29 (0.31,5.36)	0.86 (0.36,2.09)	1.18 (0.72,1.96)	1.04 (0.68,1.61)	**Methylphenidate**	0.55 (0.28,1.08)	1.06 (0.80,1.39)
1.30 (0.62,2.72)	1.23 (0.69,2.21)	1.27 (0.28,5.77)	0.85 (0.32,2.25)	1.16 (0.61,2.21)	1.03 (0.55,1.93)	0.98 (0.57,1.71)	**Modafinil**	1.91 (1.04,3.49)
0.92 (0.52,1.61)	0.86 (0.62,1.21)	0.89 (0.21,3.75)	0.60 (0.26,1.39)	0.82 (0.54,1.24)	0.72 (0.48,1.08)	**0.69 (0.52,0.91)**	0.70 (0.43,1.14)	**Placebo**

Discontinuation due to any reason is reported for each comparison as odds ratio (OR), along with 95% confidence intervals. An OR below 1 favours the medication on the top left vs. the medication on the bottom right in the diagonal. The bottom left triangle refers to results in children/adolescents and the top right triangle to results in adults.

FDA dose analyses, separating Lisdexamfetamine from other Amphetamines

Efficacy – ADHD core symptoms, Teachers' Ratings

No NMA since no available studies.

Efficacy – ADHD core symptoms, Clinicians' Ratings

**Amphetamines**	-0.18 (-0.74;0.38)	-	-	0.23 (-0.42;0.88)	-0.13 (-0.70;0.43)	**-0.63 (-1.18;-0.09)**
-0.26 (-0.59;0.06)	**Atomoxetine**	-	-	**0.41 (0.02;0.80)**	0.05 (-0.16;0.25)	**-0.45 (-0.60;-0.31)**
-0.10 (-0.65;0.44)	0.16 (-0.29;0.61)	**Clonidine**	-	-	-	-
-0.20 (-0.57;0.17)	0.06 (-0.14;0.27)	-0.09 (-0.56;0.37)	**Guanfacine**	-	-	-
0.28 (-0.09;0.65)	**0.54 (0.34;0.75)**	0.38 (-0.09;0.85)	**0.48 (0.23;0.73)**	**Lisdexamfetamine**	-0.36 (-0.75;0.03)	**-0.86 (-1.22;-0.50)**
-0.00 (-0.36;0.35)	**0.26 (0.09;0.43)**	0.10 (-0.36;0.56)	0.20 (-0.03;0.42)	**-0.28 (-0.48;-0.09)**	**Methylphenidate**	**-0.50 (-0.66;-0.35)**
**-0.81 (-1.13;-0.49)**	**-0.55 (-0.65;-0.44)**	**-0.71 (-1.14;-0.27)**	**-0.61 (-0.79;-0.44)**	**-1.09 (-1.27;-0.91)**	**-0.81 (-0.96;-0.66)**	**Placebo**

Mean change in symptoms is reported as a standardized mean difference (SMD) along with 95% confidence intervals. An SMD below 0 favours the medication on the top left vs. the medication on the bottom right in the diagonal. The bottom left triangle refers to results in children/adolescents and the top right triangle to results in adults.

Efficacy – ADHD core symptoms, Parents' Ratings

**Atomoxetine**	-	-	-
**0.47 (0.17;0.77)**	**Lisdexamfetamine**	-	-
**0.23 (0.10;0.37)**	-0.24 (-0.54;0.07)	**Methylphenidate**	-
**-0.60 (-0.71;-0.50)**	**-1.07 (-1.36;-0.79)**	**-0.84 (-0.95;-0.72)**	**Placebo**

Mean change in symptoms is reported as a standardized mean difference (SMD) along with 95% confidence intervals. An SMD below 0 favours the medication on the top left vs. the medication on the bottom right in the diagonal.

Efficacy – ADHD core symptoms, Self-Ratings

No studies on Lisdexamfetamine were found, thus effects were identical to those in the analysis of Lisdexamfetamine and Other Amphetamies lumped.

Tolerability

**Amphetamines**	2.50 (0.33;19.06)	-	-	2.14 (0.22;21.10)	2.43 (0.33;18.14)	5.84 (0.85;40.32)
1.27 (0.54;2.98)	**Atomoxetine**	-	-	0.86 (0.21;3.41)	0.97 (0.45;2.08)	**2.34 (1.25;4.38)**
0.16 (0.02;1.56)	0.13 (0.01;1.15)	**Clonidine**	-	-	-	-
0.64 (0.21;1.92)	0.50 (0.19;1.35)	3.90 (0.40;37.66)	**Guanfacine**	-	-	-
0.69 (0.26;1.82)	0.54 (0.24;1.23)	4.22 (0.46;38.70)	1.08 (0.39;2.99)	**Lisdexamfetamine**	1.14 (0.29;4.38)	2.73 (0.80;9.36)
1.29 (0.55;3.06)	1.02 (0.53;1.96)	7.92 (0.90;69.41)	2.03 (0.78;5.32)	1.88 (0.98;3.58)	**Methylphenidate**	**2.41 (1.39;4.17)**
1.82 (0.82;4.03)	1.43 (0.80;2.56)	**11.16 (1.35;92.43)**	**2.86 (1.26;6.52)**	**2.64 (1.36;5.12)**	1.41 (0.86;2.30)	**Placebo**

Discontinuation due to adverse events is reported for each comparison as odds ratio (OR), along with 95% confidence intervals. An OR above 1 favours the medication on the bottom right vs. the medication on the top left in the diagonal. The bottom left triangle refers to results in children/adolescents and the top right triangle to results in adults.

Functioning - Clinical Global Impression (CGI)

**Amphetamines**	1.34 (0.33;5.39)	-	0.57 (0.16;2.00)	0.87 (0.28;2.75)	2.75 (0.96;7.86)
**3.41 (1.72;6.75)**	**Atomoxetine**	-	0.42 (0.14;1.33)	0.65 (0.24;1.77)	2.05 (0.82;5.12)
**2.29 (1.08;4.84)**	0.67 (0.33;1.37)	**Guanfacine**	-	-	-
1.02 (0.49;2.13)	**0.30 (0.15;0.60)**	**0.45 (0.24;0.85)**	**Lisdexamfetamine**	1.54 (0.68;3.50)	**4.85 (2.42;9.70)**
1.41 (0.72;2.78)	**0.41 (0.22;0.79)**	0.62 (0.34;1.11)	1.38 (0.85;2.23)	**Methylphenidate**	**3.15 (1.98;5.02)**
**7.89 (4.37;14.25)**	**2.31 (1.34;3.99)**	**3.45 (2.17;5.48)**	**7.71 (4.96;11.99)**	**5.59 (3.91;7.99)**	**Placebo**

Improvement in clinical global functioning is reported for each comparison as odds ratio (OR), along with 95% confidence intervals. An OR above 1 favours the medication on the top left vs. the medication on the bottom right in the diagonal. The bottom left triangle refers to results in children/adolescents and the top right triangle to results in adults.

Weight (Kgs)

**Amphetamines**	0.11 (-0.74;0.96)	-	-0.10 (-1.03;0.83)	0.58 (-0.27;1.42)	-0.18 (-0.95;0.59)
0.37 (-0.89;1.63)	**Atomoxetine**	-	-0.21 (-0.84;0.43)	**0.47 (0.01;0.93)**	-0.29 (-0.64;0.07)
-0.57 (-1.90;0.76)	**-0.94 (-1.57;-0.30)**	**Guanfacine**	-	-	-
0.27 (-1.04;1.58)	-0.10 (-0.69;0.50)	**0.84 (0.10;1.57)**	**Lisdexamfetamine**	**0.68 (0.05;1.31)**	-0.08 (-0.60;0.45)
0.29 (-0.97;1.56)	-0.07 (-0.51;0.37)	**0.86 (0.23;1.50)**	0.02 (-0.51;0.56)	**Methylphenidate**	**-0.75 (-1.10;-0.41)**
-0.47 (-1.69;0.74)	**-0.84 (-1.17;-0.51)**	0.09 (-0.44;0.63)	**-0.74 (-1.25;-0.24)**	**-0.77 (-1.11;-0.43)**	**Placebo**

Mean change in body weight is reported as a standardized mean difference (SMD) along with 95% confidence intervals. An SMD below 0 favours the medication on the bottom right vs. the medication on the top left in the diagonal. The bottom left triangle refers to results in children/adolescents and the top right triangle to results in adults.

Systolic Blood Pressure

**Amphetamines**	0.26 (-0.15,0.67)	0.37 (-0.08,0.82)	0.19 (-0.22,0.60)	0.37 (-0.02,0.76)
-0.14 (-0.34,0.06)	**Atomoxetine**	0.11 (-0.16,0.38)	-0.07 (-0.26,0.12)	0.11 (-0.03,0.24)
-0.16 (-0.38,0.05)	-0.02 (-0.17,0.12)	**Lisdexamfetamine**	-0.18 (-0.45,0.09)	-0.00 (-0.23,0.23)
-0.13 (-0.34,0.08)	0.01 (-0.12,0.14)	0.03 (-0.09,0.16)	**Methylphenidate**	**0.18 (0.04,0.32)**
-0.02 (-0.20,0.16)	**0.12 (0.02,0.22)**	**0.14 (0.03,0.26)**	**0.11 (0.01,0.21)**	**Placebo**

Mean change in systolic blood pressure is reported as a standardized mean difference (SMD) along with 95% confidence intervals. An SMD above 0 indicates an increase in blood pressure values and favours the medication on the bottom right vs. the medication on the top left in the diagonal. The bottom left triangle refers to results in children/adolescents and the top right triangle to results in adults.

Diastolic Blood Pressure

**Amphetamines**	-0.18 (-0.55,0.19)	0.08 (-0.33,0.49)	-0.19 (-0.56,0.18)	0.01 (-0.34,0.36)
-0.10 (-0.32,0.11)	**Atomoxetine**	**0.26 (0.03,0.49)**	-0.01 (-0.17,0.15)	**0.19 (0.08,0.30)**
-0.06 (-0.29,0.16)	0.04 (-0.11,0.19)	**Lisdexamfetamine**	**-0.27 (-0.50,-0.03)**	-0.07 (-0.27,0.14)
-0.07 (-0.29,0.14)	0.03 (-0.10,0.16)	-0.01 (-0.14,0.12)	**Methylphenidate**	**0.20 (0.08,0.32)**
0.17 (-0.02,0.36)	**0.27 (0.17,0.38)**	**0.23 (0.11,0.35)**	**0.25 (0.14,0.35)**	**Placebo**

Mean change in diastolic blood pressure is reported as a standardized mean difference (SMD) along with 95% confidence intervals. An SMD above 0 indicates an increase in blood pressure values and favours the medication on the bottom right vs. the medication on the top left in the diagonal. The bottom left triangle refers to results in children/adolescents and the top right triangle to results in adults.

Acceptability

**Amphetamines**	0.57 (0.24,1.34)	-	-	0.94 (0.36,2.44)	0.69 (0.30,1.61)	0.73 (0.33,1.62)
1.06 (0.58,1.95)	**Atomoxetine**	-	-	1.65 (0.89,3.03)	1.21 (0.81,1.83)	1.28 (0.94,1.75)
1.52 (0.50,4.57)	1.43 (0.52,3.90)	**Clonidine**	-	-	-	-
1.16 (0.57,2.36)	1.09 (0.63,1.89)	0.77 (0.27,2.17)	**Guanfacine**	-	-	-
1.28 (0.64,2.54)	1.20 (0.71,2.02)	0.84 (0.30,2.36)	1.10 (0.60,2.00)	**Lisdexamfetamine**	0.74 (0.40,1.34)	0.78 (0.46,1.32)
1.33 (0.72,2.43)	1.25 (0.84,1.86)	0.87 (0.33,2.35)	1.14 (0.68,1.92)	1.04 (0.67,1.61)	**Methylphenidate**	1.06 (0.79,1.42)
0.92 (0.52,1.63)	0.87 (0.62,1.22)	0.61 (0.24,1.57)	0.79 (0.52,1.22)	0.72 (0.48,1.10)	**0.70 (0.52,0.93)**	**Placebo**

Discontinuation due to any reason is reported for each comparison as odds ratio (OR), along with 95% confidence intervals. An OR below 1 favours the medication on the top left vs. the medication on the bottom right in the diagonal. The bottom left triangle refers to results in children/adolescents and the top right triangle to results in adults.

Inclusive dose analyses, separating Lisdexamfetamine from other Amphetamines

Efficacy – ADHD core symptoms, Teachers' Ratings

No NMA since no studies available.

Efficacy – ADHD core symptoms, Clinicians' Ratings

**Amphetamines**	**-0.26 (-0.50;-0.02)**	-0.28 (-0.69;0.12)	-	-	0.12 (-0.24;0.47)	**-0.27 (-0.51;-0.03)**	**-0.90 (-1.33;-0.48)**	**-0.75 (-0.96;-0.53)**
-0.28 (-0.60;0.03)	**Atomoxetine**	-0.02 (-0.39;0.34)	-	-	**0.37 (0.07;0.67)**	-0.01 (-0.16;0.14)	**-0.65 (-1.03;-0.26)**	**-0.49 (-0.60;-0.38)**
0.11 (-0.66;0.89)	0.40 (-0.32;1.12)	**Bupropion**	-	-	0.40 (-0.05;0.84)	0.02 (-0.35;0.38)	**-0.62 (-1.12;-0.12)**	**-0.46 (-0.81;-0.12)**
-0.14 (-0.67;0.40)	0.15 (-0.29;0.59)	-0.25 (-1.08;0.58)	**Clonidine**	-	-	-	-	-
-0.12 (-0.47;0.22)	0.16 (-0.01;0.33)	-0.24 (-0.97;0.49)	0.01 (-0.44;0.47)	**Guanfacine**	-	-	-	-
0.24 (-0.12;0.59)	**0.52 (0.33;0.71)**	0.12 (-0.61;0.85)	0.37 (-0.10;0.84)	**0.36 (0.13;0.59)**	**Lisdexamfetamine**	**-0.38 (-0.68;-0.08)**	**-1.02 (-1.48;-0.56)**	**-0.86 (-1.14;-0.58)**
-0.06 (-0.40;0.27)	**0.22 (0.08;0.37)**	-0.18 (-0.88;0.52)	0.07 (-0.38;0.52)	0.06 (-0.14;0.26)	**-0.30 (-0.49;-0.11)**	**Methylphenidate**	**-0.64 (-1.02;-0.25)**	**-0.48 (-0.59;-0.37)**
-0.23 (-0.60;0.15)	0.06 (-0.16;0.28)	-0.34 (-1.08;0.40)	-0.09 (-0.57;0.39)	-0.10 (-0.35;0.15)	**-0.46 (-0.73;-0.20)**	-0.16 (-0.40;0.07)	**Modafinil**	0.16 (-0.21;0.52)
**-0.84 (-1.15;-0.53)**	**-0.56 (-0.65;-0.47)**	**-0.96 (-1.67;-0.24)**	**-0.71 (-1.14;-0.27)**	**-0.72 (-0.87;-0.57)**	**-1.08 (-1.25;-0.90)**	**-0.78 (-0.91;-0.64)**	**-0.61 (-0.82;-0.41)**	**Placebo**

Mean change in symptoms is reported as a standardized mean difference (SMD) along with 95% confidence intervals. An SMD below 0 favours the medication on the top left vs. the medication on the bottom right in the diagonal. The bottom left triangle refers to results in children/adolescents and the top right triangle to results in adults.

Efficacy – ADHD core symptoms, Parents' Ratings

**Atomoxetine**	-	-	-	-	-	-
**-0.93 (-1.61;-0.24)**	**Bupropion**	-	-	-	-	-

-0.39 (-1.08;0.30)	0.54 (-0.43;1.51)	**Guanfacine**	-	-	-	-
**0.46 (0.15;0.78)**	**1.39 (0.64;2.13)**	**0.85 (0.10;1.59)**	**Lisdexamfetamine**	-	-	-
**0.15 (0.03;0.26)**	**1.07 (0.40;1.75)**	0.53 (-0.16;1.23)	-0.31 (-0.63;0.01)	**Methylphenidate**	-	-
-0.15 (-0.34;0.03)	**0.77 (0.07;1.47)**	0.23 (-0.47;0.93)	**-0.61 (-0.95;-0.27)**	**-0.30 (-0.49;-0.11)**	**Modafinil**	-
**-0.61 (-0.71;-0.52)**	0.31 (-0.37;1.00)	-0.23 (-0.91;0.46)	**-1.07 (-1.37;-0.77)**	**-0.76 (-0.87;-0.65)**	**-0.46 (-0.62;-0.30)**	**Placebo**

Mean change in symptoms is reported as a standardized mean difference (SMD) along with 95% confidence intervals. An SMD below 0 favours the medication on the top left vs. the medication on the bottom right in the diagonal.

Efficacy – ADHD core symptoms, Self-Ratings

No studies on Lisdexamfetamine were found, thus effects were identical to those in the analysis of Lisdexamfetamine and Other Amphetamies lumped.

Tolerability

**Amphetamines**	1.41 (0.51;3.88)	1.37 (0.15;12.80)	-	-	1.27 (0.29;5.68)	1.39 (0.50;3.83)	0.88 (0.23;3.30)	**3.51 (1.41;8.72)**
1.13 (0.49;2.60)	**Atomoxetine**	0.97 (0.12;7.84)	-	-	0.90 (0.25;3.20)	0.98 (0.54;1.78)	0.62 (0.21;1.80)	**2.49 (1.59;3.88)**
1.32 (0.13;13.27)	1.17 (0.13;10.69)	**Bupropion**	-	-	0.93 (0.09;9.87)	1.01 (0.13;8.20)	0.64 (0.07;6.12)	2.56 (0.33;19.76)
0.44 (0.06;3.17)	0.39 (0.06;2.50)	0.33 (0.02;5.63)	**Clonidine**	-	-	-	-	-
0.71 (0.23;2.14)	0.62 (0.25;1.58)	0.53 (0.05;5.46)	1.61 (0.22;11.74)	**Guanfacine**	-	-	-	-
0.74 (0.28;1.96)	0.65 (0.30;1.40)	0.56 (0.06;5.30)	1.68 (0.24;11.46)	1.04 (0.38;2.87)	**Lisdexamfetamine**	1.09 (0.31;3.86)	0.69 (0.15;3.17)	2.75 (0.84;9.00)
1.43 (0.62;3.29)	1.26 (0.74;2.13)	1.08 (0.12;9.50)	3.24 (0.51;20.44)	2.02 (0.81;5.01)	**1.93 (1.02;3.67)**	**Methylphenidate**	0.63 (0.22;1.83)	**2.53 (1.61;3.97)**
1.41 (0.44;4.54)	1.25 (0.46;3.37)	1.07 (0.10;11.20)	3.20 (0.42;24.16)	1.99 (0.62;6.44)	1.91 (0.65;5.64)	0.99 (0.37;2.63)	**Modafinil**	**4.01 (1.53;10.54)**
1.93 (0.87;4.24)	**1.70 (1.07;2.71)**	1.46 (0.16;12.93)	4.37 (0.71;26.84)	**2.72 (1.22;6.05)**	**2.61 (1.34;5.08)**	1.35 (0.85;2.14)	1.37 (0.56;3.30)	**Placebo**

Discontinuation due to adverse events is reported for each comparison as odds ratio (OR), along with 95% confidence intervals. An OR above 1 favours the medication on the bottom right vs. the medication on the top left in the diagonal. The bottom left triangle refers to results in children/adolescents and the top right triangle to results in adults.

Functioning - Clinical Global Impression (CGI)

**Amphetamines**	2.44 (0.94;6.36)	1.46 (0.57;3.75)	-	-	1.03 (0.48;2.20)	1.65 (0.93;2.93)	**5.55 (2.22;13.85)**	**4.94 (3.13;7.79)**
**3.24 (1.77;5.93)**	**Atomoxetine**	0.60 (0.18;1.93)	-	-	0.42 (0.15;1.19)	0.67 (0.27;1.67)	2.27 (0.71;7.22)	2.02 (0.87;4.69)
-	-	**Bupropion**	-	-	0.70 (0.25;1.97)	1.13 (0.47;2.74)	**3.80 (1.20;12.02)**	**3.38 (1.47;7.79)**
2.97 (0.87;10.13)	0.91 (0.28;3.02)	-	**Clonidine**	-	-	-	-	-
**2.52 (1.35;4.72)**	0.78 (0.46;1.32)	-	0.85 (0.27;2.72)	**Guanfacine**	-	-	-	-
1.08 (0.55;2.10)	**0.33 (0.18;0.60)**	-	0.36 (0.11;1.17)	**0.43 (0.25;0.73)**	**Lisdexamfetamine**	1.60 (0.79;3.26)	**5.41 (1.98;14.74)**	**4.81 (2.60;8.89)**
1.46 (0.79;2.70)	**0.45 (0.26;0.78)**	-	0.49 (0.16;1.50)	**0.58 (0.36;0.93)**	1.35 (0.88;2.10)	**Methylphenidate**	**3.37 (1.40;8.08)**	**3.00 (2.07;4.34)**
**2.59 (1.23;5.44)**	0.80 (0.40;1.58)	-	0.87 (0.26;2.96)	1.03 (0.55;1.91)	**2.40 (1.25;4.62)**	1.77 (0.96;3.26)	**Modafinil**	0.89 (0.40;1.97)
**8.34 (4.88;14.23)**	**2.57 (1.65;4.01)**	-	2.81 (0.93;8.51)	**3.30 (2.34;4.67)**	**7.73 (5.16;11.59)**	**5.71 (4.13;7.89)**	**3.22 (1.92;5.39)**	**Placebo**

Improvement in clinical global functioning is reported for each comparison as odds ratio (OR), along with 95% confidence intervals. An OR above 1 favours the medication on the top left vs. the medication on the bottom right in the diagonal. The bottom left triangle refers to results in children/adolescents and the top right triangle to results in adults.

Weight (Kgs)

**Amphetamines**	-0.37 (-0.89;0.15)	-0.11 (-1.04;0.83)	-	-	**-0.81 (-1.53;-0.09)**	-0.02 (-0.54;0.50)	-	**-0.89 (-1.31;-0.46)**
0.37 (-0.72;1.46)	**Atomoxetine**	0.27 (-0.62;1.15)	-	-	-0.44 (-1.09;0.22)	0.35 (-0.06;0.76)	-	**-0.51 (-0.82;-0.21)**
-	-	**Bupropion**	-	-	-0.70 (-1.72;0.31)	0.08 (-0.80;0.97)	-	-0.78 (-1.61;0.05)

-0.61 (-2.07;0.85)	-0.99 (-2.00;0.03)	-	**Clonidine**	-	-	-	-	-
-0.51 (-1.65;0.62)	**-0.88 (-1.32;-0.44)**	-	0.10 (-0.97;1.17)	**Guanfacine**	-	-	-	-
0.24 (-0.91;1.40)	-0.13 (-0.62;0.35)	-	0.86 (-0.22;1.93)	**0.75 (0.17;1.34)**	**Lisdexamfetamine**	**0.79 (0.13;1.44)**	-	-0.08 (-0.66;0.50)
0.24 (-0.86;1.34)	-0.13 (-0.45;0.19)	-	0.86 (-0.14;1.85)	**0.75 (0.28;1.23)**	0.00 (-0.47;0.47)	**Methylphenidate**	-	**-0.86 (-1.17;-0.56)**
0.44 (-0.79;1.67)	0.07 (-0.59;0.72)	-	1.05 (-0.12;2.22)	**0.95 (0.22;1.68)**	0.20 (-0.56;0.95)	0.20 (-0.48;0.87)	**Modafinil**	-
-0.49 (-1.55;0.58)	**-0.86 (-1.09;-0.64)**	-	0.12 (-0.87;1.12)	0.02 (-0.37;0.41)	**-0.73 (-1.17;-0.29)**	**-0.73 (-1.01;-0.46)**	**-0.93 (-1.54;-0.31)**	**Placebo**

Mean change in body weight is reported as a standardized mean difference (SMD) along with 95% confidence intervals. An SMD below 0 favours the medication on the bottom right vs. the medication on the top left in the diagonal. The bottom left triangle refers to results in children/adolescents and the top right triangle to results in adults.

Systolic Blood Pressure

**Amphetamines**	-0.02 (-0.22,0.18)	-0.09 (-0.86,0.67)	0.18 (-0.10,0.46)	0.00 (-0.20,0.21)	-	**0.18 (0.02,0.34)**
-0.13 (-0.32,0.07)	**Atomoxetine**	-0.08 (-0.83,0.68)	0.19 (-0.06,0.45)	0.02 (-0.15,0.19)	-	**0.19 (0.08,0.31)**
-	-	**Bupropion**	0.27 (-0.51,1.05)	0.10 (-0.66,0.86)	-	0.27 (-0.48,1.02)
-0.15 (-0.37,0.06)	-0.03 (-0.16,0.11)	-	**Lisdexamfetamine**	-0.18 (-0.44,0.09)	-	-0.00 (-0.23,0.23)
-0.13 (-0.33,0.07)	-0.01 (-0.11,0.10)	-	0.02 (-0.10,0.15)	**Methylphenidate**	-	**0.17 (0.05,0.30)**
-0.06 (-0.30,0.18)	0.06 (-0.11,0.24)	-	0.09 (-0.10,0.29)	0.07 (-0.11,0.25)	**Modafinil**	-
-0.00 (-0.18,0.18)	**0.12 (0.04,0.20)**	-	**0.15 (0.04,0.27)**	**0.13 (0.04,0.22)**	0.06 (-0.10,0.22)	**Placebo**

Mean change in systolic blood pressure is reported as a standardized mean difference (SMD) along with 95% confidence intervals. An SMD above 0 indicates an increase in blood pressure values and favours the medication on the bottom right vs. the medication on the top left in the diagonal. The bottom left triangle refers to results in children/adolescents and the top right triangle to results in adults.

Diastolic Blood Pressure

**Amphetamines**	-0.16 (-0.33,0.01)	-0.11 (-0.85,0.64)	0.21 (-0.05,0.47)	-0.11 (-0.29,0.07)	-	0.09 (-0.05,0.23)
-0.06 (-0.25,0.13)	**Atomoxetine**	0.06 (-0.68,0.80)	**0.37 (0.13,0.61)**	0.05 (-0.10,0.20)	-	**0.25 (0.16,0.35)**
-	-	**Bupropion**	0.31 (-0.45,1.08)	-0.00 (-0.75,0.74)	-	0.20 (-0.54,0.93)
-0.04 (-0.25,0.17)	0.02 (-0.12,0.15)	-	**Lisdexamfetamine**	**-0.32 (-0.56,-0.07)**	-	-0.12 (-0.34,0.10)
-0.04 (-0.24,0.15)	0.02 (-0.08,0.12)	-	-0.00 (-0.12,0.12)	**Methylphenidate**	-	**0.20 (0.09,0.31)**
0.21 (-0.02,0.45)	**0.27 (0.10,0.45)**	-	**0.26 (0.06,0.45)**	**0.26 (0.07,0.44)**	**Modafinil**	**-**
**0.18 (0.01,0.35)**	**0.24 (0.16,0.32)**	-	**0.22 (0.11,0.34)**	**0.23 (0.13,0.32)**	-0.03 (-0.19,0.13)	**Placebo**

Mean change in diastolic blood pressure is reported as a standardized mean difference (SMD) along with 95% confidence intervals. An SMD above 0 indicates an increase in blood pressure values and favours the medication on the bottom right vs. the medication on the top left in the diagonal. The bottom left triangle refers to results in children/adolescents and the top right triangle to results in adults.

Acceptability

**Amphetamines**	**0.43 (0.26,0.71)**	0.54 (0.23,1.24)	-	-	0.77 (0.39,1.53)	**0.50 (0.30,0.83)**	**0.32 (0.15,0.69)**	**0.60 (0.38,0.94)**
1.00 (0.58,1.74)	**Atomoxetine**	1.25 (0.60,2.63)	-	-	**1.80 (1.02,3.17)**	1.15 (0.82,1.62)	0.74 (0.38,1.44)	1.40 (1.11,1.77)
1.05 (0.23,4.74)	1.04 (0.25,4.38)	**Bupropion**	-	-	1.44 (0.60,3.44)	0.92 (0.43,1.95)	0.59 (0.23,1.51)	1.12 (0.55,2.26)
1.54 (0.59,4.07)	1.54 (0.66,3.58)	1.48 (0.29,7.57)	**Clonidine**	-	-	-	-	-
1.14 (0.59,2.20)	1.13 (0.71,1.81)	1.09 (0.25,4.76)	0.74 (0.30,1.81)	**Guanfacine**	-	-	-	-
1.29 (0.67,2.46)	1.28 (0.82,2.00)	1.23 (0.29,5.31)	0.83 (0.34,2.04)	1.13 (0.65,1.97)	**Lisdexamfetamine**	0.64 (0.36,1.14)	**0.41 (0.18,0.92)**	0.78 (0.46,1.30)
1.35 (0.77,2.37)	1.35 (0.99,1.83)	1.29 (0.31,5.28)	0.87 (0.38,2.03)	1.19 (0.74,1.91)	1.05 (0.70,1.57)	**Methylphenidate**	0.64 (0.32,1.26)	1.22 (0.94,1.58)
1.32 (0.66,2.67)	1.32 (0.78,2.23)	1.27 (0.28,5.64)	0.86 (0.34,2.18)	1.17 (0.63,2.15)	1.03 (0.56,1.88)	0.98 (0.58,1.66)	**Modafinil**	**1.91 (1.01,3.58)**
0.92 (0.54,1.57)	0.92	0.88	0.60	0.81	0.71 (0.49,1.05)	**0.68 (0.53,0.88)**	0.70	**Placebo**

	(0.72,1.17)	(0.21,3.65)	(0.27,1.34)	(0.54,1.21)			(0.44,1.11)	

Discontinuation due to any reason is reported for each comparison as odds ratio (OR), along with 95% confidence intervals. An OR below 1 favours the medication on the top left vs. the medication on the bottom right in the diagonal. The bottom left triangle refers to results in children/adolescents and the top right triangle to results in adults.

Tables S18. NMA heterogeneity, *post hoc* analyses, primary outcomes

Analyses in children/adolescents

FDA dose analyses, Lisdexamfetamine and other Amphetamines lumped

Model assumption	SD Heterogeneity
**Efficacy, teachers' ratings**	
Consistency	0.036
Inconsistency	-
**Efficacy, clinicians' ratings**	
Consistency	0.172
Inconsistency	0.181
**Tolerability**	
Consistency	0.288
Inconsistency	0.411

Inclusive dose analyses, Lisdexamfetamine and other Amphetamines lumped

Model assumption	SD Heterogeneity
**Efficacy, teachers' ratings**	
Consistency	0.292
Inconsistency	0.323
**Efficacy, clinicians' ratings**	
Consistency	0.167
Inconsistency	0.177
**Tolerability**	
Consistency	0.314
Inconsistency	0.335

Main dose analyses, splitting Lisdexamfetamine from other Amphetamines

Model assumption	SD Heterogeneity
**Efficacy, teachers' ratings**	
Consistency	0.355
Inconsistency	0.397
**Efficacy, clinicians' ratings**	
Consistency	0.185
Inconsistency	0.201
**Tolerability**	

Consistency	0.283
Inconsistency	0.336

FDA dose analyses, splitting Lisdexamfetamine from other Amphetamines

Model assumption	SD Heterogeneity
**Efficacy, teachers' ratings**	
Consistency	0.036
Inconsistency	-
**Efficacy, clinicians' ratings**	
Consistency	0.168
Inconsistency	0.184
**Tolerability**	
Consistency	0.305
Inconsistency	0.365

Inclusive dose analyses, splitting Lisdexamfetamine from other Amphetamines

Model assumption	SD Heterogeneity
**Efficacy, teachers' ratings**	
Consistency	0.292
Inconsistency	0.323
**Efficacy, clinicians' ratings**	
Consistency	0.166
Inconsistency	0.181
**Tolerability**	
Consistency	0.336
Inconsistency	0.286

Analyses in adults

FDA dose analyses, Lisdexamfetamine and other Amphetamines lumped

Model assumption	SD Heterogeneity
**Efficacy, clinicians' ratings**	
Consistency	0.202
Inconsistency	0.224
**Tolerability**	
Consistency	0.335
Inconsistency	0.396

Inclusive dose analyses, Lisdexamfetamine and other Amphetamines lumped

Model assumption	SD Heterogeneity
**Efficacy, clinicians' ratings**	
Consistency	0.123
Inconsistency	0.140
**Tolerability**	
Consistency	0.164
Inconsistency	0.221

Main dose analyses, splitting Lisdexamfetamine from other Amphetamines

Model assumption	SD Heterogeneity
**Efficacy, clinicians' ratings**	
Consistency	0.185
Inconsistency	0.204
**Tolerability**	
Consistency	0.323
Inconsistency	0.382

FDA dose analyses, splitting Lisdexamfetamine from other Amphetamines

Model assumption	SD Heterogeneity
**Efficacy, clinicians' ratings**	
Consistency	0.207
Inconsistency	0.231
**Tolerability**	
Consistency	0.343
Inconsistency	0.406

Inclusive dose analyses, splitting Lisdexamfetamine from other Amphetamines

Model assumption	SD Heterogeneity
**Efficacy, clinicians' ratings**	
Consistency	0.130
Inconsistency	0.148
**Tolerability**	
Consistency	0.204
Inconsistency	0.258

Tables S19. Evaluation of incoherence *(loop-specific approach, node-splitting approach, design-by-treatment interaction model)*

Efficacy – ADHD core symptoms, Children/Adolescents, Teachers' Ratings

- *Loop-specific approach*

Loop	IF	seIF	z_value	p_value	CI_95	Loop_Heterog_tau2
Placebo-Methylphenidate-Modafinil	0.019	0.668	0.028	0.977	(0.00,1.33)	0.111

- *Node-splitting approach*

Side	Direct Coef.	Std. Err.	Indirect Coef.	Std. Err.	Difference Coef.	Std. Err.	P>|z|	tau
1 2	-.8105185	.2026987	-.8627785	.5284844	.05226	.5667433	0.927	.3971073
1 3	.	.	.	.	.	.	.	.
1 4	.	.	.	.	.	.	.	.
1 6	-.7835421	.2343138	-.7311989	.5152743	-.0523432	.5667697	0.926	.3971123
2 5 *	.4964558	.3411957	2.129303	141.4427	-1.632847	141.4427	0.991	.3553797
2 6	.0793002	.4737315	.02697	.3111377	.0523302	.5667699	0.926	.3971121

* Warning: all the evidence about these contrasts comes from the trials which directly compare them.

Legend: 1 Placebo, 2 Methylphenidate, 3 Atomoxetine, 4 Guanfacine, 5 Bupropion, 6 Modafinil

- *Design-by-treatment test*

chi2(1) = 0.01
Prob > chi2 = 0.9264

Efficacy – ADHD core symptoms, Adults, Clinician's Ratings

Loop-specific approach

Loop	IF	seIF	z_value	p_value	CI_95	Loop_Heterog_tau2
Placebo-Methylphenidate-Atomoxetine	0.022	0.363	0.059	0.953	(0.00,0.73)	0.042

- *Node-splitting approach*

Side	Direct Coef.	Std. Err.	Indirect Coef.	Std. Err.	Difference Coef.	Std. Err.	P>|z|	tau
1 2 *	-.4986312	.0751955	-.4401977	.457818	-.0584335	.4634245	0.900	.1870832
1 3	.	.	.	.	.	.	.	.
1 4 *	-.452266	.0694555	-.4454633	.4654242	-.0068028	.4702312	0.988	.18701
1 5	.	.	.	.	.	.	.	.
1 6	.	.	.	.	.	.	.	.
2 4	.0297282	.2519279	.0477428	.1058669	-.0180146	.273246	0.947	.1872611

* Warning: all the evidence about these contrasts comes from the trials which directly compare them.

Legend: 1 Placebo, 2 Methylphenidate, 3 Amphetamines, 4 Atomoxetine, 5 Bupropion, 6 Modafinil

- *Design-by-treatment test*

chi2(2) = 0.02
Prob > chi2 = 0.9901

Efficacy – ADHD core symptoms, Children/Adolescents, Clinician's Ratings

- *Loop-specific approach*

Loop	IF	seIF	z_value	p_value	CI_95	Loop_Heterog_tau2
Placebo-Methylphenidate-Modafinil	0.369	0.413	0.894	0.372	(0.00,1.18)	0.040
Methylphenidate-Amphetamines-Atomoxetine	0.253	0.199	1.270	0.204	(0.00,0.64)	0.004
Placebo-Amphetamines-Atomoxetine	0.154	0.287	0.537	0.591	(0.00,0.72)	0.047
Placebo-Methylphenidate-Amphetamines	0.123	0.218	0.566	0.572	(0.00,0.55)	0.047
Placebo-Methylphenidate-Atomoxetine	0.025	0.174	0.141	0.888	(0.00,0.36)	0.027

- *Node-splitting approach*

Side	Direct Coef.	Std. Err.	Indirect Coef.	Std. Err.	Difference Coef.	Std. Err.	P>|z|	tau
1 2	-.8295893	.0836465	-.5282192	.179629	-.3013701	.1974921	0.127	.1804612
1 3	-1.038195	.0986903	-.9464678	.1930033	-.0917274	.2181349	0.674	.191632
1 4	-.5576194	.0572769	-.5468452	.2125858	-.0107742	.2210794	0.961	.1929196
1 5	.	.	.	.	.	.	.	.
1 6	.	.	.	.	.	.	.	.
1 8	-.5876246	.1166406	-.9199598	.3291484	.3323353	.3489125	0.341	.1874675
2 3	-.3244045	.1287961	-.1193831	.1558655	-.2050214	.2021323	0.310	.1893332
2 4	.2826914	.1565925	.1917766	.1084127	.0909147	.1889527	0.630	.1933981
2 7 *	-.1778574	.3686075	1.376655	200.3568	-1.554512	200.3566	0.994	.1882096
2 8	-.1254802	.3193009	.2068594	.1406654	-.3323395	.3489123	0.341	.1874675
3 4	.3233745	.2375735	.4900884	.1067739	-.1667139	.2604647	0.522	.1909779

* Warning: all the evidence about these contrasts comes from the trials which directly compare them.

Legend: 1 Placebo, 2 Methylphenidate, 3 Amphetamines, 4 Atomoxetine, 5 Clonidine, 6 Guanfacine, 7 Bupropion, 8 Modafinil

- *Design-by-treatment test*

chi2(6) = 4.06
Prob > chi2 = 0.6686

Tolerability –Children/Adolescents

- *Loop-specific approach*

Loop	ROR	z_value	p_value	CI_95	Loop_Heterog_tau2
Placebo-Methylphenidate-Clonidine	28.676	1.666	0.096	(1.00,1487.57)	0.000
Placebo-Amphetamines+Lisdexamfetamine-Atomoxetine	1.408	0.440	0.660	(1.00,6.47)	0.000
Placebo-Methylphenidate-Amphetamines+Lisdexamfetamine	1.290	0.491	0.623	(1.00,3.57)	0.000
Placebo-Methylphenidate-Atomoxetine	1.279	0.391	0.696	(1.00,4.40)	0.000
Placebo-Methylphenidate-Bupropion	1.221	0.090	0.928	(1.00,94.81)	0.000
Methylphenidate-Amphetamines+Lisdexamfetamine-Atomoxetine	1.080	0.093	0.926	(1.00,5.52)	0.000
Placebo-Methylphenidate-Modafinil	1.028	0.013	0.989	(1.00,60.26)	0.000

- *Node-splitting approach*

Side	Direct Coef.	Std. Err.	Indirect Coef.	Std. Err.	Difference Coef.	Std. Err.	P>|z|	tau
1 2	.3153371	.2697621	.6012647	.6114629	-.2859276	.6781192	0.673	.3007663
1 3	.7003784	.3153353	1.270469	.5889428	-.5700902	.6906398	0.409	.2719143
1 4	.4583675	.3422141	.2452206	.5845088	.213147	.6857612	0.756	.2863719
1 5	2.412284	1.064711	-.9063042	1.743234	3.318588	2.042664	0.104	.2517474
1 6	.	.	.	.	.	.	.	.
1 7	.4795731	1.699572	.3630057	1.463882	.1165674	2.243099	0.959	.2745161
1 8	.2932662	.4518586	.3653081	2.049265	-.0720418	2.098118	0.973	.2741918
2 3	.6212773	.3436846	.185739	.4788595	.4355383	.5841547	0.456	.296087
2 4	-.0951121	.4699699	.1596047	.450327	-.2547168	.6567766	0.698	.2967523
2 5	-1.223487	1.726503	2.094704	1.091524	-3.318191	2.042567	0.104	.2517473
2 7	.0000862	1.443634	.1164426	1.716626	-.1163564	2.242835	0.959	.2745158
2 8	.0000669	2.034783	-.0720476	.5112177	.0721146	2.098012	0.973	.274192
3 4	-.5988174	.7079649	-.3920548	.3959039	-.2067626	.8111436	0.799	.2979027

- *Design-by-treatment test*

chi2(1) = 0.01
Prob > chi2 = 0.9598

Tolerability –Adults

- *Loop-specific approach*

Loop	ROR	z_value	p_value	CI_95	Loop_Heterog_tau2
Placebo-Methylphenidate-Atomoxetine	1.349	0.430	0.667	(1.00,5.27)	0.000

- *Node-splitting approach*

Side	Direct Coef.	Std. Err.	Indirect Coef.	Std. Err.	Difference Coef.	Std. Err.	P>|z|	tau
1 2 *	.93435	.2872124	-.1368242	1.395574	1.071174	1.448894	0.460	.3133719
1 3	.	.	.	.	.	.	.	.
1 4 *	.8283768	.3373995	1.09148	1.388985	-.2631028	1.468684	0.858	.328752
1 5	.	.	.	.	.	.	.	.
1 6	.	.	.	.	.	.	.	.
2 4	.241321	.6542756	-.168163	.4737381	.4094841	.8084844	0.613	.3251721

* Warning: all the evidence about these contrasts comes from the trials which directly compare them.

Legend: 1 Placebo, 2 Methylphenidate, 3 Amphetamines, 4 Atomoxetine, 5 Bupropion, 6 Modafinil

- *Design-by-treatment test*

  chi2(2) = 0.66

Tables S20. Ranking according to SUCRA and Mean rank for the primary and secondary outcomes, Main dose analysis, closest to 12 weeks

Efficacy on ADHD core symptoms, teachers' rating, children/adolescents

Treatment	SUCRA	Mean Rank
Placebo	8.0	5.6
Methylphenidate	81.9	1.9
Atomoxetine	36.5	4.2
Guanfacine	61.7	2.9
Bupropion	36.5	4.2
Modafinil	75.4	2.2

Efficacy on ADHD core symptoms, clinicians' rating, children/adolescents

Treatment	SUCRA	Mean Rank
Placebo	0.1	8.0
Methylphenidate	66.2	3.4
Amphetamines	92.2	1.5
Atomoxetine	26.4	6.2
Clonidine	52.8	4.3
Guanfacine	47.8	4.7
Bupropion	74.4	2.8
Modafinil	40.1	5.2

Efficacy on ADHD core symptoms, parents' rating, children/adolescents

Treatment	SUCRA	Mean Rank
Placebo	16.9	6.0
Methylphenidate	83.6	2.0
Amphetamines	98.8	1.1
Atomoxetine	63.1	3.2
Guanfacine	33.5	5.0
Bupropion	7.8	6.5
Modafinil	46.4	4.2

Efficacy on ADHD core symptoms, clinicians' rating, adults

Treatment	SUCRA	Mean Rank
Placebo	15.4	5.2
Methylphenidate	65.2	2.7
Amphetamines	98.2	1.1
Atomoxetine	56.2	3.2
Bupropion	59.6	3.0
Modafinil	5.5	5.7

Efficacy on ADHD core symptoms, self-ratings, adults

Treatment	SUCRA	Mean Rank
Placebo	2.6	6.8
Methylphenidate	67.1	3.0
Amphetamines	71.7	2.7
Atomoxetine	55.6	3.7
Guanfacine	88.0	1.7
Bupropion	44.1	4.4
Modafinil	20.9	5.7

Tolerability, children

Treatment	SUCRA	Mean Rank
Placebo	88.4	1.8
Methylphenidate	62.0	3.7
Amphetamines	28.8	6.0
Atomoxetine	58.6	3.9
Clonidine	16.3	6.9
Guanfacine	24.9	6.3
Bupropion	55.6	4.1
Modafinil	65.4	3.4

Tolerability, adults

Treatment	SUCRA	Mean Rank
Placebo	96.0	1.2
Methylphenidate	50.6	3.5
Amphetamines	31.5	4.4
Atomoxetine	52.2	3.4
Bupropion	47.4	3.6
Modafinil	22.3	4.9

CGI, children

Treatment	SUCRA	Mean Rank
Placebo	0.6	7.0
Methylphenidate	80.5	2.2
Amphetamines	98.2	1.1
Atomoxetine	27.0	5.4
Clonidine	42.0	4.5
Guanfacine	54.7	3.7
Modafinil	46.9	4.2

CGI, adults

Treatment	SUCRA	Mean Rank

**Placebo**	13.1	5.3
**Methylphenidate**	65.4	2.7
**Amphetamines**	93.6	1.3
**Atomoxetine**	45.8	3.7
**Bupropion**	71.9	2.4
**Modafinil**	10.2	5.5

Weight, children

Treatment	SUCRA	Mean Rank
**Placebo**	20.6	5.8
**Methylphenidate**	70.6	2.8
**Amphetamines**	65.2	3.1
**Atomoxetine**	78.0	2.3
**Clonidine**	19.0	5.9
**Guanfacine**	14.9	6.1
**Modafinil**	81.8	2.1

Weight, adults

Treatment	SUCRA	Mean Rank
**Placebo**	4.9	4.8
**Methylphenidate**	76.8	1.9
**Amphetamines**	62.9	2.5
**Atomoxetine**	33.7	3.7
**Bupropion**	71.7	2.1

Diastolic blood pressure, children

Treatment	SUCRA	Mean Rank
**Placebo**	16.2	4.4
**Methylphenidate**	73.9	2.0
**Amphetamines**	62.3	2.5
**Atomoxetine**	88.6	1.5
**Modafinil**	8.9	4.6

Diastolic blood pressure, adults

Treatment	SUCRA	Mean Rank
**Placebo**	15.2	4.4
**Methylphenidate**	75.1	2.0
**Amphetamines**	26.5	3.9
**Atomoxetine**	73.6	2.1
**Bupropion**	59.5	2.6

Systolic blood pressure, children

Treatment	SUCRA	Mean Rank
**Placebo**	7.1	4.7
**Methylphenidate**	60.3	2.6
**Amphetamines**	62.3	2.5
**Atomoxetine**	76.4	1.9
**Modafinil**	44.0	3.2

Systolic blood pressure, adults

Treatment	SUCRA	Mean Rank
**Placebo**	8.3	4.7
**Methylphenidate**	73.7	2.1
**Amphetamines**	49.7	3.0
**Atomoxetine**	50.8	3.0
**Bupropion**	67.5	2.3

Acceptability, children/adolescents

Treatment	SUCRA	Mean Rank
**Placebo**	14.8	7.0
**Methylphenidate**	69.3	3.1
**Amphetamines**	51.7	4.4
**Atomoxetine**	39.0	5.3
**Clonidine**	72.2	2.9
**Guanfacine**	46.3	4.8
**Bupropion**	42.8	5.0
**Modafinil**	64.1	3.5

Acceptability, adults

Treatment	SUCRA	Mean Rank
**Placebo**	64.6	2.8
**Methylphenidate**	55.0	3.3
**Amphetamines**	97.0	1.1
**Atomoxetine**	28.8	4.6
**Bupropion**	49.0	3.6
**Modafinil**	5.5	5.7

Tables S21. Results of the pairwise meta-analyses for each of the primary and secondary outcomes in the Main, FDA and Inclusive dose analysis (outcomes closest to 26 and 52 weeks)

In each table in this section, the bottom left triangle refers to results in children/adolescents and the top right triangle refers to results in adults.
Comparisons should be read from left to right and from top to bottom, in a diagonal. For each outcome, the estimate is located at the intersection of the top left treatment and the bottom right treatment in the diagonal for the given pairwise comparison. Significant results are in bold and underlined. Results were reported only for the comparisons with available studies with outcomes closest to the 26 or 52 weeks.

Results of the Main dose analyses, Lisdexamfetamine and other Amphetamines lumped

Efficacy – ADHD core symptoms, Clinician's Ratings (closest to 26 weeks), adults

**Atomoxetine**	-	**<u>-0.33 (-0.65,-0.01)</u>**
-	**Methylphenidate**	**<u>-0.35 (-0.57,-0.13)</u>**
-	-	**Placebo**

Mean change in symptoms is reported as a standardized mean difference (SMD) along with 95% confidence intervals. An SMD below 0 favours the medication on the top left vs. the medication on the bottom right in the diagonal.

Efficacy – ADHD core symptoms, Self-Ratings (closest to 26 weeks), adults

**Atomoxetine**	-	**<u>-0.35 (-0.49,-0.21)</u>**
-	**Methylphenidate**	**<u>-0.28 (-0.51,-0.06)</u>**
-	-	**Placebo**

Mean change in symptoms is reported as a standardized mean difference (SMD) along with 95% confidence intervals. An SMD below 0 favours the medication on the top left vs. the medication on the bottom right in the diagonal.

Functioning - Clinical Global Impression (CGI; closest to 26 weeks), adults

**Methylphenidate**	<u>2.07 (1.32; 3.25)</u>
-	**Placebo**

Improvement in clinical global functioning is reported for each comparison as odds ratio (OR), along with 95% confidence intervals. An OR below 1 favours the medication on the bottom right vs. the medication on the top left in the diagonal.

Systolic Blood Pressure (closest to 26 weeks), adults

**Atomoxetine**	-	0.15 (0.04; 0.25)
-	**Methylphenidate**	0.07 (-0.15; 0.29)
-	-	**Placebo**
Mean change in systolic blood pressure is reported as a standardized mean difference (SMD) along with 95% confidence intervals. An SMD above 0 indicates an increase in blood pressure values and favours the medication on the bottom right vs. the medication on the top left in the diagonal.		

Diastolic Blood Pressure (closest to 26 weeks), adults

**Atomoxetine**	-	0.15 (0.04; 0.25)
-	**Methylphenidate**	0.00 (-0.22; 0.22)
-	-	**Placebo**
Mean change in diastolic blood pressure is reported as a standardized mean difference (SMD) along with 95% confidence intervals. An SMD above 0 indicates an increase in blood pressure values and favours the medication on the bottom right vs. the medication on the top left in the diagonal.		

Weight (Kgs; closest to 26 weeks), adutls

**Atomoxetine**	-	-0.12 (-0.32; 0.07)
-	**Methylphenidate**	0.00 (-0.22; 0.22)
-	-	**Placebo**
Mean change in body weight is reported as a standardized mean difference (SMD) along with 95% confidence intervals. An SMD below 0 favours the medication on the bottom right vs. the medication on the top left in the diagonal.		

Inclusive dose analyses, Lisdexamfetamine and other Amphetamines lumped

Efficacy – ADHD core symptoms, Clinician's Ratings (closest to 26 weeks), adults

**Atomoxetine**	-	**-0.33 (-0.65,-0.01)**
-	**Methylphenidate**	**-0.34 (-0.52,-0.15)**

-	-	**Placebo**

Mean change in symptoms is reported as a standardized mean difference (SMD) along with 95% confidence intervals. An SMD below 0 favours the medication on the top left vs. the medication on the bottom right in the diagonal.

Efficacy – ADHD core symptoms, Clinician's Ratings (closest to 52 weeks), adults

**Methylphenidate**	-0.27 (-0.65,0.11)
-	**Placebo**

Improvement in clinical global functioning is reported for each comparison as odds ratio (OR), along with 95% confidence intervals. An OR below 1 favours the medication on the bottom right vs. the medication on the top left in the diagonal.

Tables S22. Summary of the GRADE ratings

ADHD core symptoms reduction in children/adolescents, teachers' ratings

	N. of studies	Study limitation	Indirectness	Inconsistency		Imprecision	Publication bias		
**Mixed estimates**				**Heterogeneity**	**Incoherence**			**GRADE**	Reasons
Atomoxetine vs Placebo	3	Downgrade because >50% contribution from moderate RoB comparisons	Downgrade because >50% contribution from comparisons with partially indirectness	no concerns	no concerns	Downgrade because confidence interval cross one of the limits of the clinically important effects zone (-0.2; 0.2)	undetected	VERY LOW	SL, indir, IMPR
Bupropion vs Methylphenidate	2	Downgrade because >50% contribution from moderate RoB comparisons	no concerns	no concerns	no concerns	Downgrade because confidence interval cross one of the limits of the clinically	undetected	LOW	SL, IMPR

						important effects zone (-0.2; 0.2)			
Guanfacine vs Placebo	1	Downgrade because >50% contribution from moderate RoB comparisons	Downgrade because >50% contribution from comparisons with partially indirectness	no concerns	no concerns	Downgrade because confidence interval cross one of the limits of the clinically important effects zone (-0.2; 0.2)	undetected	VERY LOW	SL, indir, IMPR
Methylphenidate vs Modafinil	1	Downgrade because >50% contribution from moderate RoB comparisons	no concerns	no concerns	no concerns	Downgrade because confidence interval cross both limits of the clinically important effects zone (-0.2; 0.2)	undetected	VERY LOW	SL, IMPRX 2
Methylphenidate vs Placebo	5	Downgrade because >50% contribution from moderate RoB comparisons	no concerns	Downgrade because orediction interval extends into clinically important effects (-0.2; 0.2) or	no concerns	no concerns	undetected	LOW	SL, INCON (HET)

				unimportant effects					
Modafinil vs Placebo	4	Downgrade because >50% contribution from high RoB comparisons	no concerns	Downgrade because orediction interval extends into clinically important effects (-0.2; 0.2) or unimportant effects	no concerns	no concerns	undetected	VERY LOW	SLX2, INCON (HET)
**Indirect estimates**									
Atomoxetine vs Bupropion	-	Downgrade because >50% contribution from moderate RoB comparisons	no concerns	no concerns	no concerns	Downgrade because confidence interval cross both limits of the clinically important effects zone (-0.2; 0.2)	undetected	VERY LOW	SL, IMPRX 2
Atomoxetine vs Guanfacine	-	Downgrade because >50% contribution from moderate	Downgrade because >50% contribution from compariso	no concerns	no concerns	Downgrade because confidence interval cross both limits of	undetected	VERY LOW	SL, INDIR, IMPRx 2

		RoB comparisons	ns with partially indirectness			the clinically important effects zone (-0.2; 0.2)			
Atomoxetine vs Methylphenidate	-	Downgrade because >50% contribution from moderate RoB comparisons	no concerns	no concerns	no concerns	Downgrade because confidence interval cross one of the limits of the clinically important effects zone (-0.2; 0.2)	undetected	LOW	SL, IMPR
Atomoxetine vs Modafinil	-	Downgrade because >50% contribution from moderate RoB comparisons	no concerns	no concerns	no concerns	Downgrade because confidence interval cross one of the limits of the clinically important effects zone (-0.2; 0.2)	undetected	LOW	SL, IMPR
Bupropion vs Guanfacine	-	Downgrade because >50% contribution from moderate RoB	no concerns	no concerns	no concerns	Downgrade because confidence interval cross both limits of the	undetected	VERY LOW	SL, IMPRX 2

		compariso ns				clinically important effects zone (- 0.2; 0.2)			
Bupropion vs Modafinil	-	Downgrad e because >50% contributio n from moderate RoB compariso ns	no concerns	no concerns	no concerns	Downgrad e because confidenc e interval cross one of the limits of the clinically important effects zone (- 0.2; 0.2)	undetected	LOW	SL, IMPR
Bupropion vs Placebo	-	Downgrad e because >50% contributio n from moderate RoB compariso ns	no concerns	no concerns	no concerns	Downgrad e because confidenc e interval cross both limits of the clinically important effects zone (- 0.2; 0.2)	undetected	VERY LOW	SL, IMPRX 2
Guanfacine vs Methylphenid ate	-	Downgrad e because >50% contributio n from moderate RoB compariso ns	no concerns	no concerns	no concerns	Downgrad e because confidenc e interval cross both limits of the clinically important	undetected	VERY LOW	SL, IMPRX 2

						effects zone (-0.2; 0.2)			
Guanfacine vs Modafinil	-	Downgrade because >50% contribution from moderate RoB comparisons	no concerns	no concerns	no concerns	Downgrade because confidence interval cross both limits of the clinically important effects zone (-0.2; 0.2)	undetected	VERY LOW	SL, IMPRX 2

ADHD core symptoms reduction in children/adolescents, clinicians' ratings

Mixed estimates	N. of studies	Study limitation	Indirectness	Inconsistency: Heterogeneity	Inconsistency: Incoherence	Imprecision	Publication bias	GRADE	Reasons
Amphetamines vs Atomoxetine	1	Downgrade because >50% contribution from moderate RoB comparisons	no concerns	Downgrade because prediction interval extends into clinically important effects (-0.2; 0.2) or unimportant effects	no concerns	no concerns	Undetected	LOW	SL, INCON (HET)
Amphetamines vs Methylphenidate	3	Downgrade because >50% contribution from moderate RoB comparisons	no concerns	no concerns	no concerns	Downgrade because confidence interval cross one of the limits of the clinically important effects zone (-0.2; 0.2)	Undetected	LOW	SL, IMPR

Amphetamines vs Placebo	6	Downgrade because >50% contribution from moderate RoB comparisons	no concerns	no concerns	no concerns	no concerns	Undetected	MODERATE	**SL**
Atomoxetine vs Methylphenidate	3	Downgrade because >50% contribution from moderate RoB comparisons	no concerns	no concerns	no concerns	Downgrade because confidence interval cross one of the limits of the clinically important effects zone (-0.2; 0.2)	Undetected	LOW	SL, IMPR
Atomoxetine vs Placebo	21	Downgrade because >50% contribution from moderate RoB comparisons	no concerns	Downgrade because prediction interval extends into clinically important effects (-0.2; 0.2) or unimportant effects	no concerns	no concerns	Undetected	LOW	SL, INCON (HET)
Bupropion vs Methylphenidate	1	Downgrade because >50% contribution from moderate RoB	no concerns	no concerns	no concerns	Downgrade because confidence interval cross both limits of the clinically	Undetected	VERY LOW	SL, IMPRx2

		comparison s				important effects zone (-0.2; 0.2)			
Clonidine vs Placebo	1	Downgrade because >50% contribution from moderate RoB comparison s	no concerns	no concerns	no concerns	no concerns	Undetecte d	MODERAT E	**SL**
Guanfacine vs Placebo	7	Downgrade because >50% contribution from moderate RoB comparison s	no concerns	no concerns	no concerns	no concerns	Undetecte d	MODERAT E	**SL**
Methylphenidat e vs Modafinil	1	Downgrade because >50% contribution from moderate RoB comparison s	no concerns	no concerns	no concerns	Downgrade because confidence interval cross one of the limits of the clinically important effects zone (-0.2; 0.2)	Undetecte d	LOW	SL, IMPR

Methylphenidate vs Placebo	9	Downgrade because >50% contribution from moderate RoB comparisons	no concerns	no concerns	no concerns	no concerns	Undetected	MODERATE	**SL**
Modafinil vs Placebo	5	Downgrade because >50% contribution from high RoB comparisons	no concerns	no concerns	no concerns	no concerns	Undetected	LOW	SLx2
**Indirect estimates**									
Amphetamines vs Bupropion	-	Downgrade because >50% contribution from moderate RoB comparisons	no concerns	no concerns	no concerns	Downgrade because confidence interval cross both limits of the clinically important effects zone (-0.2; 0.2)	Undetected	VERY LOW	SL, IMPRx2

Amphetamines vs Clonidine	-	Downgrade because >50% contribution from moderate RoB comparisons	no concerns	no concerns	no concerns	Downgrade because confidence interval cross one of the limits of the clinically important effects zone (-0.2; 0.2)	Undetected	LOW	SL, IMPR
Amphetamines vs Guanfacine	-	Downgrade because >50% contribution from moderate RoB comparisons	no concerns	no concerns	no concerns	Downgrade because confidence interval cross one of the limits of the clinically important effects zone (-0.2; 0.2)	Undetected	LOW	SL, IMPR
Amphetamines vs Modafinil	-	Downgrade because >50% contribution from moderate RoB comparisons	no concerns	no concerns	no concerns	Downgrade because confidence interval cross one of the limits of the clinically important effects zone (-0.2; 0.2)	Undetected	LOW	SL, IMPR

Atomoxetine vs Bupropion	-	Downgrade because >50% contribution from moderate RoB comparison s	no concerns	no concerns	no concerns	Downgrade because confidence interval cross one of the limits of the clinically important effects zone (-0.2; 0.2)	Undetecte d	LOW	SL, IMPR
Atomoxetine vs Clonidine	-	Downgrade because >50% contribution from moderate RoB comparison s	no concerns	no concerns	no concerns	Downgrade because confidence interval cross one of the limits of the clinically important effects zone (-0.2; 0.2)	Undetecte d	LOW	SL, IMPR
Atomoxetine vs Guanfacine	-	Downgrade because >50% contribution from moderate RoB comparison s	no concerns	no concerns	no concerns	Downgrade because confidence interval cross one of the limits of the clinically important effects zone (-0.2; 0.2)	Undetecte d	LOW	SL, IMPR

Atomoxetine vs Modafinil	-	Downgrade because >50% contribution from moderate RoB comparisons	no concerns	no concerns	no concerns	Downgrade because confidence interval cross one of the limits of the clinically important effects zone (-0.2; 0.2)	Undetected	LOW	SL, IMPR
Bupropion vs Clonidine	-	Downgrade because >50% contribution from moderate RoB comparisons	no concerns	no concerns	no concerns	Downgrade because confidence interval cross both limits of the clinically important effects zone (-0.2; 0.2)	Undetected	VERY LOW	SL, IMPRx2
Bupropion vs Guanfacine	-	Downgrade because >50% contribution from moderate RoB comparisons	no concerns	no concerns	no concerns	Downgrade because confidence interval cross both limits of the clinically important effects zone (-0.2; 0.2)	Undetected	VERY LOW	SL, IMPRx2

Bupropion vs Modafinil	-	Downgrade because >50% contribution from moderate RoB comparisons	no concerns	no concerns	no concerns	Downgrade because confidence interval cross both limits of the clinically important effects zone (-0.2; 0.2)	Undetected	VERY LOW	SL, IMPRx2
Bupropion vs Placebo	-	Downgrade because >50% contribution from moderate RoB comparisons	no concerns	no concerns	no concerns	no concerns	Undetected	MODERATE	**SL**
Clonidine vs Guanfacine	-	Downgrade because >50% contribution from moderate RoB comparisons	no concerns	no concerns	no concerns	Downgrade because confidence interval cross both limits of the clinically important effects zone (-0.2; 0.2)	Undetected	VERY LOW	SL, IMPRx2
Clonidine vs Methylphenidate	-	Downgrade because >50% contribution from moderate RoB comparison	no concerns	no concerns	no concerns	Downgrade because confidence interval cross both limits of the clinically important	Undetected	VERY LOW	SL, IMPRx2

		s				effects zone (-0.2; 0.2)			
Clonidine vs Modafinil	-	Downgrade because >50% contribution from moderate RoB comparisons	no concerns	no concerns	no concerns	Downgrade because confidence interval cross both limits of the clinically important effects zone (-0.2; 0.2)	Undetected	VERY LOW	SL, IMPRx2
Guanfacine vs Methylphenidate	-	Downgrade because >50% contribution from moderate RoB comparisons	no concerns	no concerns	no concerns	Downgrade because confidence interval cross one of the limits of the clinically important effects zone (-0.2; 0.2)	Undetected	LOW	SL, IMPR
Guanfacine vs Modafinil	-	Downgrade because >50% contribution from moderate RoB comparisons	no concerns	no concerns	no concerns	Downgrade because confidence interval cross one of the limits of the clinically important	Undetected	LOW	SL, IMPR

						effects zone (-0.2; 0.2)			

ADHD core symptoms reduction in adults, clinicians' ratings

				**Inconsistency**					
**Mixed estimates**	**N. of studies**	**Study limitation**	**Indirectness**	**Heterogeneity**	**Incoherence**	**Imprecision**	**Publication bias**	GRADE	Reasons
Amphetamines vs Placebo	5	Downgrade because >50% contribution from moderate RoB comparisons	no concerns	no concerns	no concerns	no concerns	no concerns	MODERATE	SL

Atomoxetine vs Methylphenidate	1	no concerns	no concerns	no concerns	no concerns	Downgrade because confidence interval cross one of the limits of the clinically important effects zone (-0.2; 0.2)	no concerns	MODERATE	IMP
Atomoxetine vs Placebo	11	Downgrade because >50% contribution from moderate RoB comparisons	no concerns	Downgrade because prediction interval extends into clinically important effects (-0.2; 0.2) or unimportant effects	no concerns	no concerns	no concerns	LOW	SL, INCONS
Bupropion vs Placebo	2	Downgrade because >50% contribution from moderate RoB comparisons	no concerns	no concerns	no concerns	Downgrade because confidence interval cross one of the limits of the clinically important effects zone (-0.2; 0.2)	no concerns	LOW	SL, IMPR

Methylphenidate vs Placebo	11	no concerns	no concerns	Downgrade because prediction interval extends into clinically important effects (-0.2; 0.2) or unimportant effects	no concerns	no concerns	no concerns	MODERATE	INCON
Modafinil vs Placebo	1	Downgrade because >50% contribution from moderate RoB comparisons	no concerns	no concerns	no concerns	Downgrade because confidence interval cross one of the limits of the clinically important effects zone (-0.2; 0.2)	no concerns	LOW	SL, IMPR
**Indirect estimates**									

Amphetamines vs Atomoxetine	-	Downgrade because >50% contribution from moderate RoB comparisons	no concerns	no concerns	no concerns	Downgrade because confidence interval cross one of the limits of the clinically important effects zone (-0.2; 0.2)	no concerns	LOW	SL, IMPR
Amphetamines vs Bupropion	-	Downgrade because >50% contribution from moderate RoB comparisons	no concerns	no concerns	no concerns	Downgrade because confidence interval cross one of the limits of the clinically important effects zone (-0.2; 0.2)	no concerns	LOW	SL, IMPR
Amphetamines vs Methylphenidate	-	Downgrade because >50% contribution from moderate RoB comparisons	no concerns	no concerns	no concerns	Downgrade because confidence interval cross one of the limits of the clinically important effects zone (-0.2; 0.2)	no concerns	LOW	SL, IMPR

Amphetamines vs Modafinil	-	Downgrade because >50% contribution from moderate RoB comparisons	no concerns	no concerns	no concerns	no concerns	no concerns	MODERATE	SL
Atomoxetine vs Bupropion	-	Downgrade because >50% contribution from moderate RoB comparisons	no concerns	no concerns	no concerns	Downgrade because confidence interval cross one of the limits of the clinically important effects zone (-0.2; 0.2)	no concerns	LOW	SL, IMPR
Atomoxetine vs Modafinil	-	Downgrade because >50% contribution from moderate RoB comparisons	no concerns	no concerns	no concerns	Downgrade because confidence interval cross one of the limits of the clinically important effects zone (-0.2; 0.2)	no concerns	LOW	SL, IMPR

Bupropion vs Methylphenidate	-	Downgrade because >50% contribution from moderate RoB comparisons	no concerns	no concerns	no concerns	Downgrade because confidence interval cross one of the limits of the clinically important effects zone (-0.2; 0.2)	no concerns	LOW	SL, IMPR
Bupropion vs Modafinil	-	Downgrade because >50% contribution from moderate RoB comparisons	no concerns	no concerns	no concerns	Downgrade because confidence interval cross one of the limits of the clinically important effects zone (-0.2; 0.2)	no concerns	LOW	SL, IMPR
Methylphenidate vs Modafinil	-	Downgrade because >50% contribution from moderate RoB comparisons	no concerns	no concerns	no concerns	Downgrade because confidence interval cross one of the limits of the clinically important effects zone (-0.2; 0.2)	no concerns	LOW	SL, IMPR

Tolerability in children/adolescents

				Inconsistency					
**Mixed estimates**	**N. of studies**	**Study limitation**	**Indirectness**	**heterogeneity**	**incoherence**	**Imprecision**	**Publication bias**	**GRADE**	
Amphetamines vs Atomoxetine	1	Downgrade because >50% contribution from moderate RoB comparisons	no concerns	no concerns	no concerns	Downgrade because confidence interval cross one of the limits of the clinically important effects zone (0.75; 1.25)	undetected	LOW	SL, IMPR
Amphetamines vs Methylphenidate	6	Downgrade because >50% contribution from moderate RoB comparisons	no concerns	no concerns	no concerns	Downgrade because confidence interval cross one of the limits of the clinically important effects zone (0.75; 1.25)	undetected	LOW	SL, IMPR

Amphetamines vs Placebo	9	Downgrade because >50% contribution from moderate RoB comparison s	no concerns	no concerns	no concerns	no concerns	undetected	MODERATE	SL
Atomoxetine vs Methylphenidat e	4	Downgrade because >50% contribution from moderate RoB comparison s	no concerns	no concerns	no concerns	Downgrade because confidence interval cross both limits of the clinically important effects zone (0.75; 1.25)	undetected	VERY LOW	SL, IMPRX2
Atomoxetine vs Placebo	13	Downgrade because >50% contribution from moderate RoB comparison s	no concerns	no concerns	no concerns	Downgrade because confidence interval cross one of the limits of the clinically important effects zone (0.75; 1.25)	undetected	LOW	SL, IMPR
Bupropion vs Methylphenidat e	2	Downgrade because >50% contribution from moderate RoB	no concerns	no concerns	no concerns	Downgrade because confidence interval cross both limits of the clinically	undetected	VERY LOW	SL, IMPRX2

		comparisons				important effects zone (0.75; 1.25)			
Bupropion vs Placebo	1	Downgrade because >50% contribution from moderate RoB comparisons	no concerns	no concerns	no concerns	Downgrade because confidence interval cross both limits of the clinically important effects zone (0.75; 1.25)	undetected	VERY LOW	SL, IMPRX2
Clonidine vs Methylphenidate	1	Downgrade because >50% contribution from moderate RoB comparisons	no concerns	no concerns	Downgrade for side-splitting p=0.104	Downgrade because confidence interval cross both limits of the clinically important effects zone (0.75; 1.25)	undetected	VERY LOW	SL, INCONS (INCOH), IMPR
Clonidine vs Placebo	1	Downgrade because >50% contribution from moderate RoB comparisons	no concerns	no concerns	Downgrade for side-splitting p=0.104	Downgrade because confidence interval cross both limits of the clinically important effects zone (0.75; 1.25)	undetected	VERY LOW	SL, INCONS (INCOH), IMPR

Guanfacine vs Placebo	7	Downgrade because >50% contribution from moderate RoB comparisons	no concerns	no concerns	no concerns	Downgrade because confidence interval cross one of the limits of the clinically important effects zone (0.75; 1.25)	undetected	LOW	SL, IMPR
Methylphenidate vs Modafinil	1	Downgrade because >50% contribution from moderate RoB comparisons	no concerns	no concerns	no concerns	Downgrade because confidence interval cross both limits of the clinically important effects zone (0.75; 1.25)	undetected	VERY LOW	SL, IMPRX2
Methylphenidate vs Placebo	22	Downgrade because >50% contribution from moderate RoB comparisons	no concerns	no concerns	no concerns	Downgrade because confidence interval cross one of the limits of the clinically important effects zone (0.75; 1.25)	undetected	LOW	SL, IMPR

Modafinil vs Placebo	6	Downgrade because >50% contribution from high RoB comparisons	no concerns	no concerns	no concerns	Downgrade because confidence interval cross both limits of the clinically important effects zone (0.75; 1.25)	undetected	VERY LOW	SLX2, IMPRX2
**Indirect estimates**									
Amphetamines vs Bupropion		Downgrade because >50% contribution from moderate RoB comparisons	no concerns	no concerns	no concerns	Downgrade because confidence interval cross both limits of the clinically important effects zone (0.75; 1.25)	undetected	VERY LOW	SL, IMPRX2
Amphetamines vs Clonidine		Downgrade because >50% contribution from moderate RoB comparisons	no concerns	no concerns	no concerns	Downgrade because confidence interval cross both limits of the clinically important effects zone (0.75; 1.25)	undetected	VERY LOW	SL, IMPRX2

Amphetamines vs Guanfacine		Downgrade because >50% contribution from moderate RoB comparisons	no concerns	no concerns	no concerns	Downgrade because confidence interval cross both limits of the clinically important effects zone (0.75; 1.25)	undetected	VERY LOW	SL, IMPRX2
Amphetamines vs Modafinil		Downgrade because >50% contribution from moderate RoB comparisons	no concerns	no concerns	no concerns	Downgrade because confidence interval cross both limits of the clinically important effects zone (0.75; 1.25)	undetected	VERY LOW	SL, IMPRX2
Atomoxetine vs Bupropion		Downgrade because >50% contribution from moderate RoB comparisons	no concerns	no concerns	no concerns	Downgrade because confidence interval cross both limits of the clinically important effects zone (0.75; 1.25)	undetected	VERY LOW	SL, IMPRX2
Atomoxetine vs Clonidine		Downgrade because >50% contribution from moderate	no concerns	no concerns	no concerns	Downgrade because confidence interval cross both limits of the	undetected	VERY LOW	SL, IMPRX2

		RoB comparisons				clinically important effects zone (0.75; 1.25)			
Atomoxetine vs Guanfacine		Downgrade because >50% contribution from moderate RoB comparisons	no concerns	no concerns	no concerns	Downgrade because confidence interval cross both limits of the clinically important effects zone (0.75; 1.25)	undetected	VERY LOW	SL, IMPRX2
Atomoxetine vs Modafinil		Downgrade because >50% contribution from moderate RoB comparisons	no concerns	no concerns	no concerns	Downgrade because confidence interval cross both limits of the clinically important effects zone (0.75; 1.25)	undetected	VERY LOW	SL, IMPRX2
Bupropion vs Clonidine		Downgrade because >50% contribution from moderate RoB comparisons	no concerns	no concerns	no concerns	Downgrade because confidence interval cross both limits of the clinically important effects zone (0.75; 1.25)	undetected	VERY LOW	SL, IMPRX2

Bupropion vs Guanfacine		Downgrade because >50% contribution from moderate RoB comparisons	no concerns	no concerns	no concerns	Downgrade because confidence interval cross both limits of the clinically important effects zone (0.75; 1.25)	undetected	VERY LOW	SL, IMPRX2
Bupropion vs Modafinil		Downgrade because >50% contribution from moderate RoB comparisons	no concerns	no concerns	no concerns	Downgrade because confidence interval cross both limits of the clinically important effects zone (0.75; 1.25)	undetected	VERY LOW	SL, IMPRX2
Clonidine vs Guanfacine		Downgrade because >50% contribution from moderate RoB comparisons	no concerns	no concerns	no concerns	Downgrade because confidence interval cross both limits of the clinically important effects zone (0.75; 1.25)	undetected	VERY LOW	SL, IMPRX2
Clonidine vs Modafinil		Downgrade because >50% contribution from moderate	no concerns	no concerns	no concerns	Downgrade because confidence interval cross both limits of the	undetected	VERY LOW	SL, IMPRX2

		RoB comparison s				clinically important effects zone (0.75; 1.25)			
Guanfacine vs Methylphenidat e		Downgrade because >50% contribution from moderate RoB comparison s	no concerns	no concerns	no concerns	Downgrade because confidence interval cross both limits of the clinically important effects zone (0.75; 1.25)	undetected	VERY LOW	SL, IMPRX2
Guanfacine vs Modafinil		Downgrade because >50% contribution from moderate RoB comparison s	no concerns	no concerns	no concerns	Downgrade because confidence interval cross both limits of the clinically important effects zone (0.75; 1.25)	undetected	VERY LOW	SL, IMPRX2

Tolerability in adults

**Mixed estimates**	**N. of studies**	**Study limitation**	**Indirectness**	**Inconsistency**		**Imprecision**	**Publication bias**	**GRADE**	
				**Heterogeneity**	**Incoherence**				
Amphetamines vs Placebo	6	Downgrade because >50% contribution from moderate RoB comparisons	no concerns	no concerns	no concerns	no concerns	undetected	MODERATE	SL
Atomoxetine vs Methylphenidate	1	no concerns	no concerns	no concerns	no concerns	Downgrade because confidence interval cross both limits of the clinically important effects zone (0.75; 1.25)	undetected	LOW	IMPRx2
Atomoxetine vs Placebo	6	Downgrade because >50% contribution from moderate RoB comparisons	no concerns	Downgrade because orediction interval extends into clinically important effects (0.75; 1.25) or unimportant effects	no concerns	no concerns	undetected	LOW	SL, INCONS (HETER)

Bupropion vs Placebo	3	Downgrade because >50% contribution from moderate RoB comparisons	no concerns	no concerns	no concerns	Downgrade because confidence interval cross both limits of the clinically important effects zone (0.75; 1.25)	undetected	VERY LOW	SL, IMPRx2
Methylphenidate vs Placebo	12	no concerns	no concerns	no concerns	no concerns	no concerns	undetected	HIGH	
Modafinil vs Placebo	1	Downgrade because >50% contribution from moderate RoB comparisons	no concerns	no concerns	no concerns	no concerns	undetected	MODERATE	SL
**Indirect estimates**									
Amphetamines vs Atomoxetine		Downgrade because >50% contribution from moderate RoB comparisons	no concerns	no concerns	no concerns	Downgrade because confidence interval cross both limits of the clinically important effects zone (0.75; 1.25)	undetected	VERY LOW	SL, IMPRx2

Amphetamines vs Bupropion		Downgrade because >50% contribution from moderate RoB comparisons	no concerns	no concerns	no concerns	Downgrade because confidence interval cross both limits of the clinically important effects zone (0.75; 1.25)	undetected	VERY LOW	SL, IMPRx2
Amphetamines vs Methylphenidate		Downgrade because >50% contribution from moderate RoB comparisons	no concerns	no concerns	no concerns	Downgrade because confidence interval cross both limits of the clinically important effects zone (0.75; 1.25)	undetected	VERY LOW	SL, IMPRx2
Amphetamines vs Modafinil		Downgrade because >50% contribution from moderate RoB comparisons	no concerns	no concerns	no concerns	Downgrade because confidence interval cross both limits of the clinically important effects zone (0.75; 1.25)	undetected	VERY LOW	SL, IMPRx2

Atomoxetine vs Bupropion		Downgrade because >50% contribution from moderate RoB comparisons	no concerns	no concerns	no concerns	Downgrade because confidence interval cross both limits of the clinically important effects zone (0.75; 1.25)	undetected	VERY LOW	SL, IMPRx2
Atomoxetine vs Modafinil		Downgrade because >50% contribution from moderate RoB comparisons	no concerns	no concerns	no concerns	Downgrade because confidence interval cross both limits of the clinically important effects zone (0.75; 1.25)	undetected	VERY LOW	SL, IMPRx2
Bupropion vs Methylphenidate		Downgrade because >50% contribution from moderate RoB comparisons	no concerns	no concerns	no concerns	Downgrade because confidence interval cross both limits of the clinically important effects zone (0.75; 1.25)	undetected	VERY LOW	SL, IMPRx2

Bupropion vs Modafinil		Downgrade because >50% contribution from moderate RoB comparisons	no concerns	no concerns	no concerns	Downgrade because confidence interval cross both limits of the clinically important effects zone (0.75; 1.25)	undetected	VERY LOW	SL, IMPRx2
Methylphenidate vs Modafinil		Downgrade because >50% contribution from moderate RoB comparisons	no concerns	no concerns	no concerns	Downgrade because confidence interval cross both limits of the clinically important effects zone (0.75; 1.25)	undetected	VERY LOW	SL, IMPRx2

Figures S1. Network plots for the secondary outcomes, Main dose analysis, outcomes closest to 12 weeks

Each drug is represented with a circle and randomized comparisons between drugs are shown by lines between the circles

Efficacy on ADHD core symptoms, parents' rating, children/adolescents

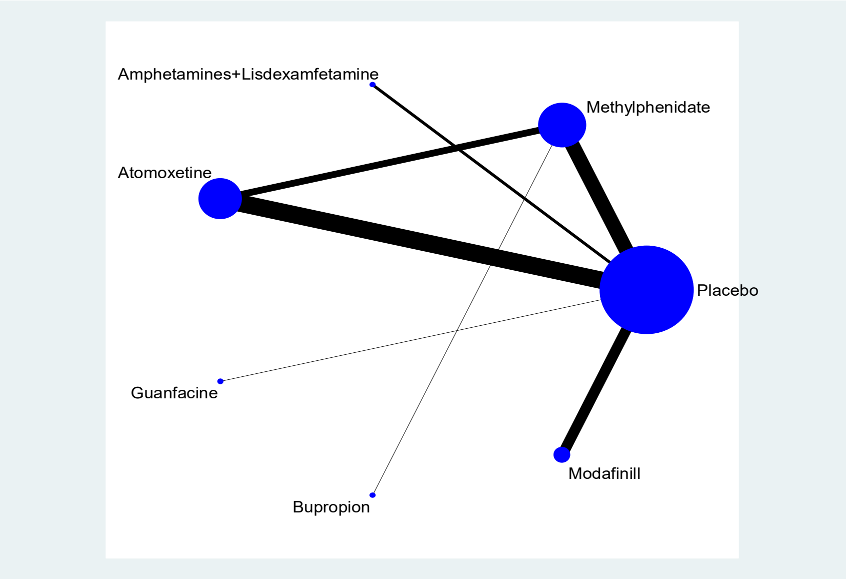

Efficacy on ADHD core symptoms, self-ratings, adults

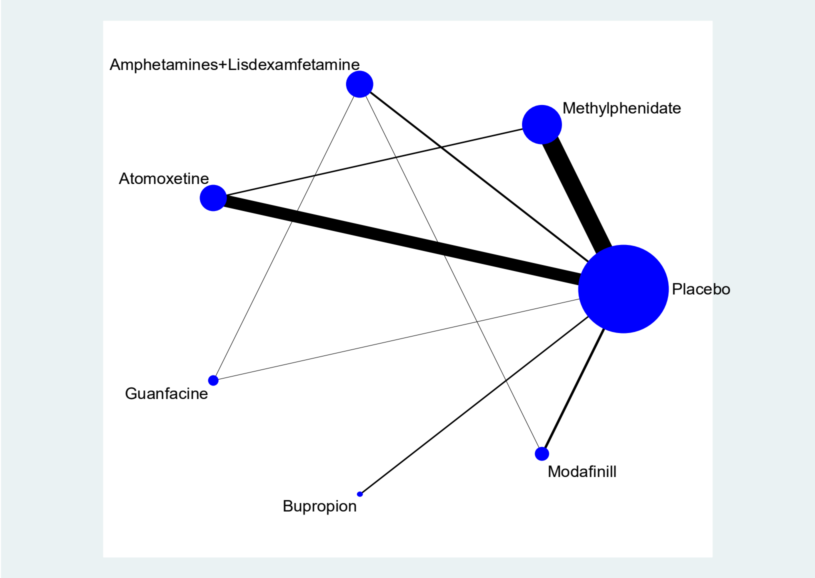

CGI, children

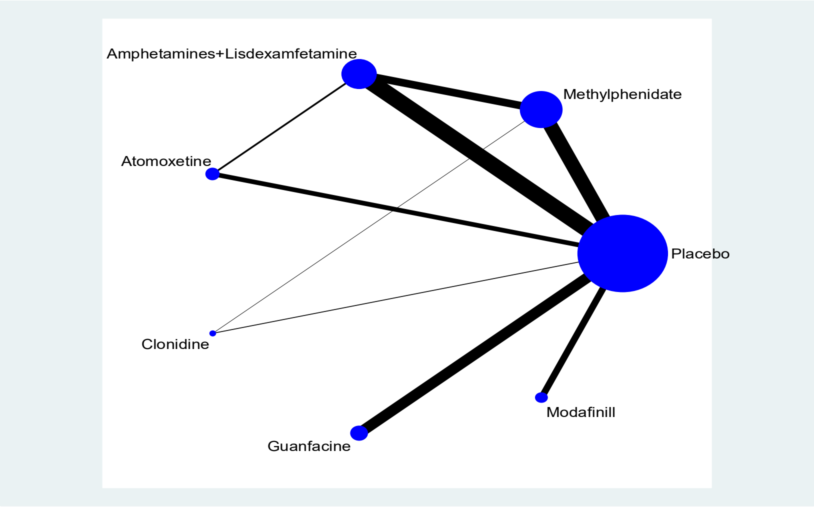

CGI, adults

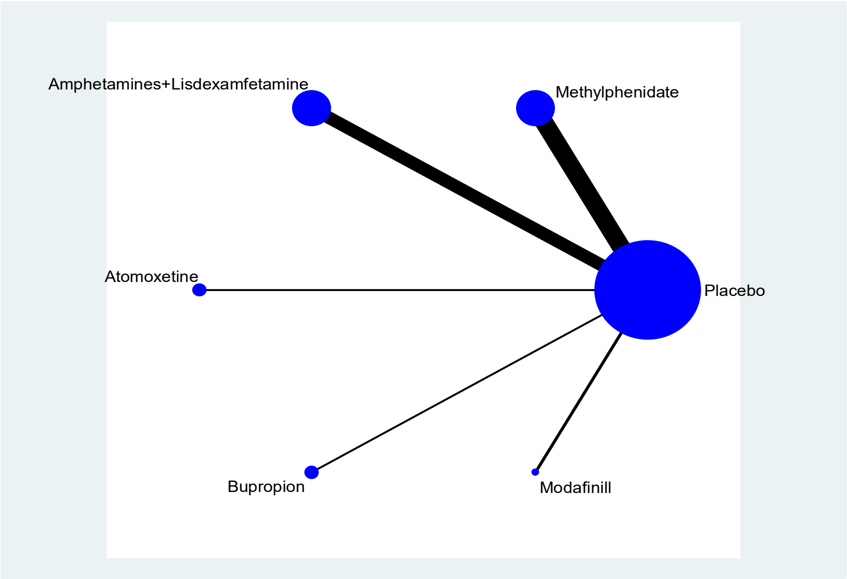

Weight, children

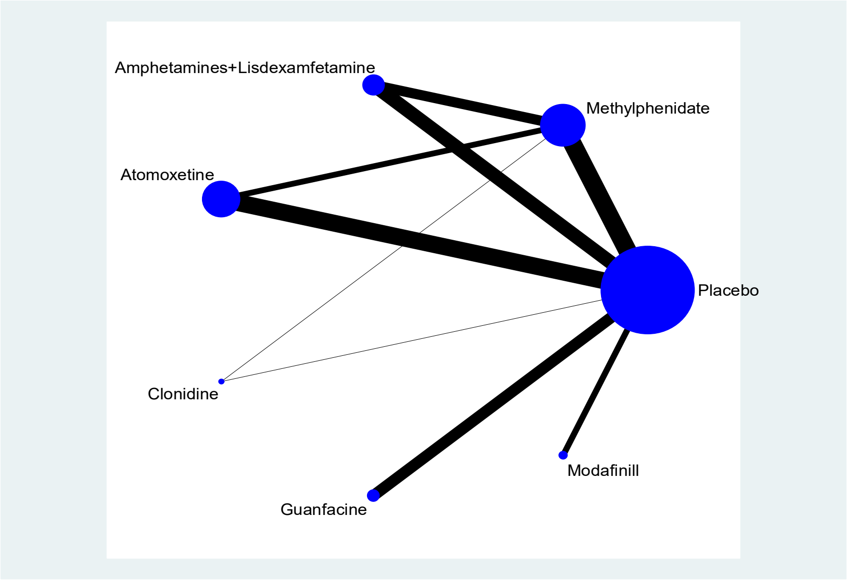

Weight, adults

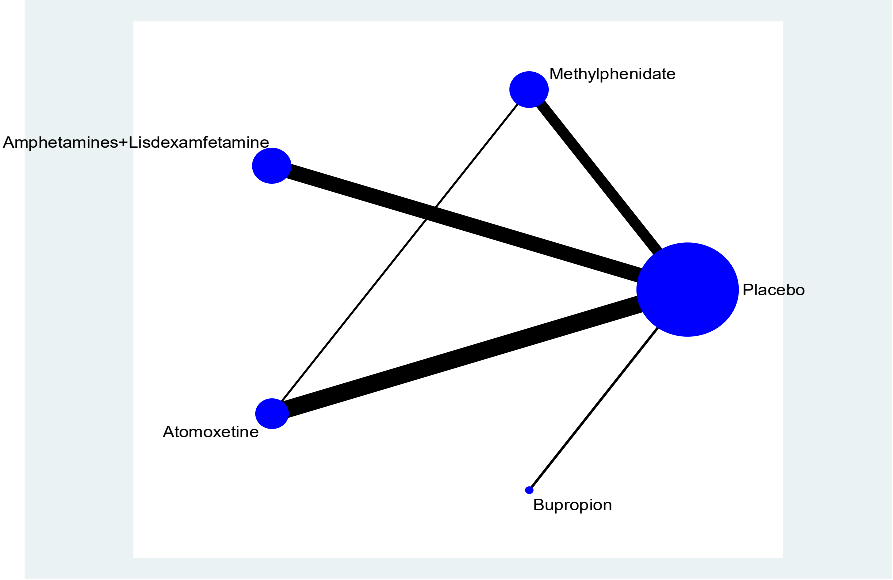

Diastolic blood pressure, children

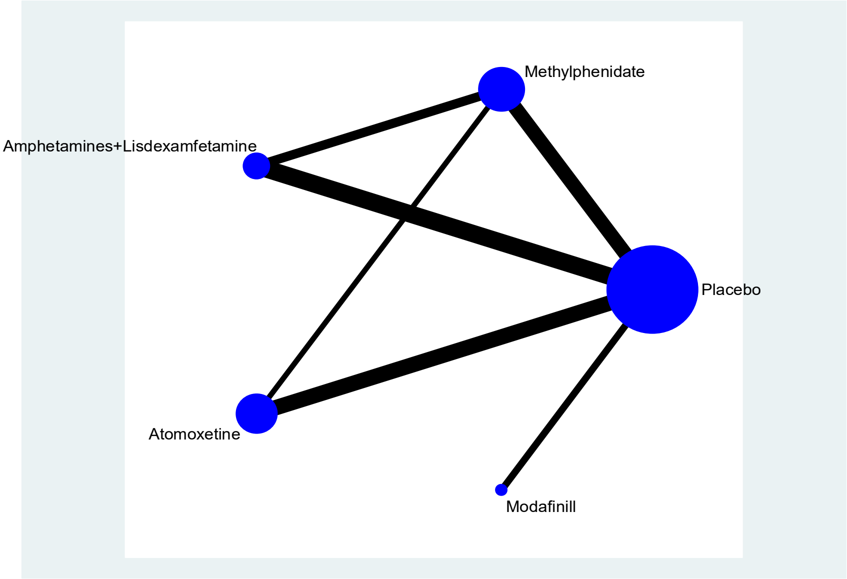

Diastolic blood pressure, adults

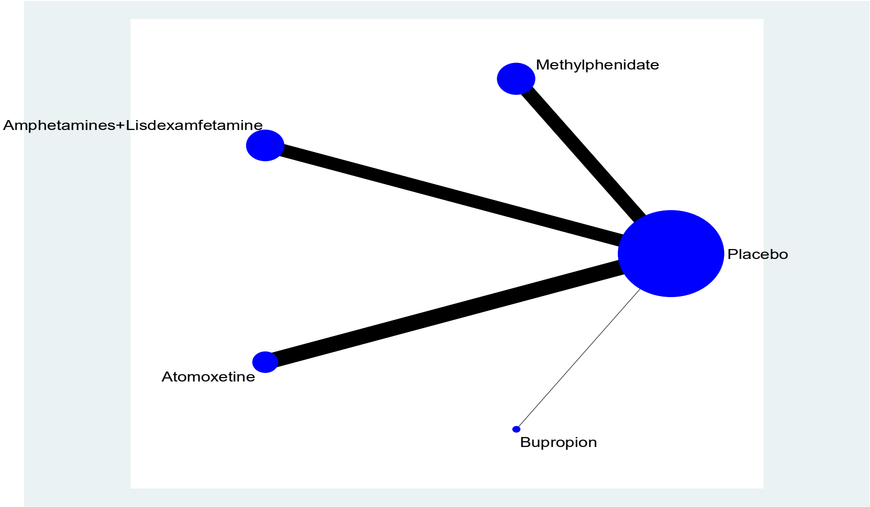

Systolic blood pressure, children

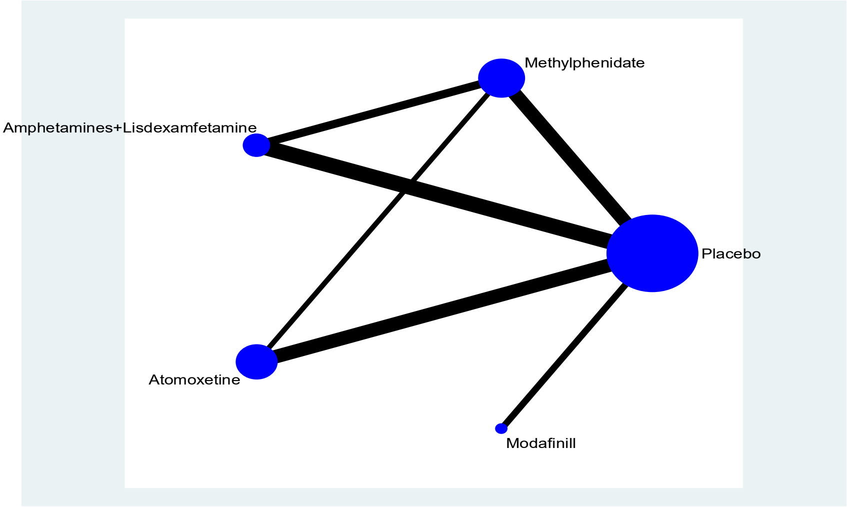

Systolic blood pressure, adults

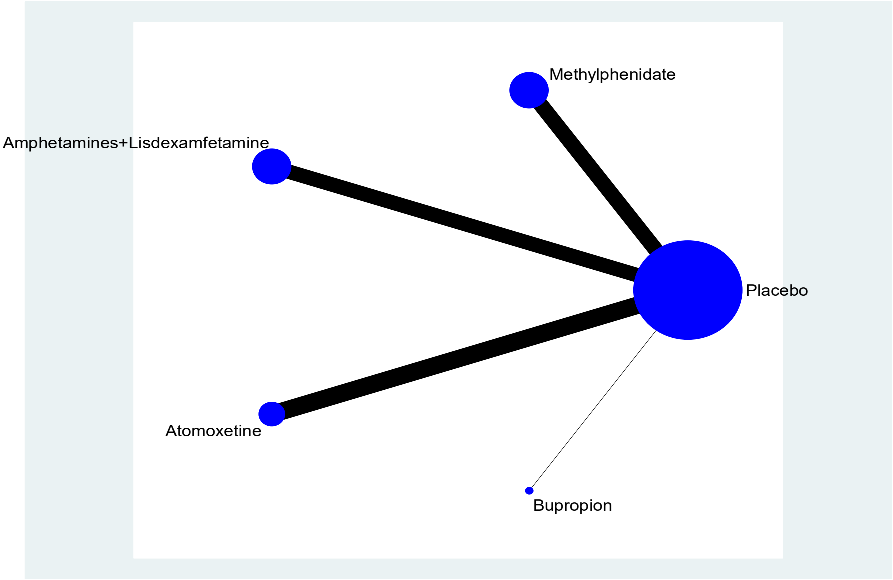

Acceptability, children

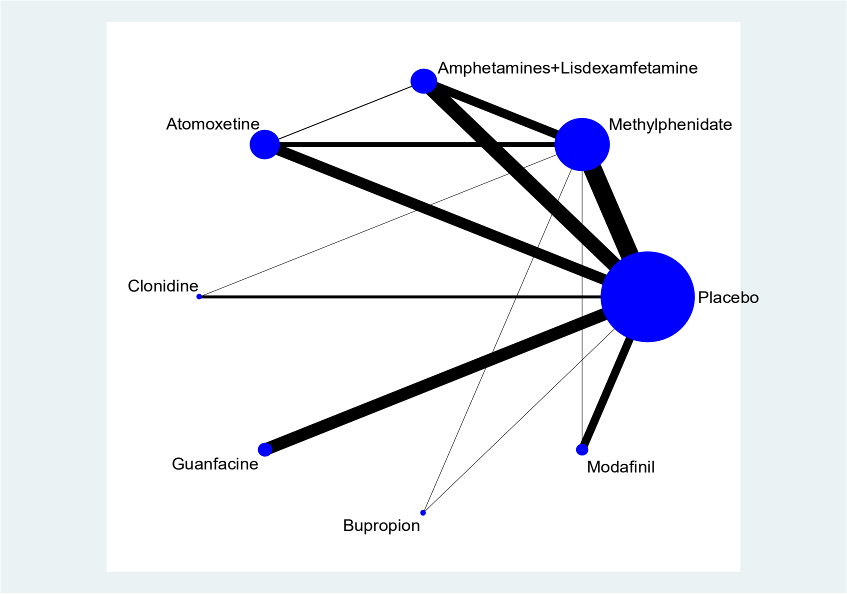

Acceptability, adults

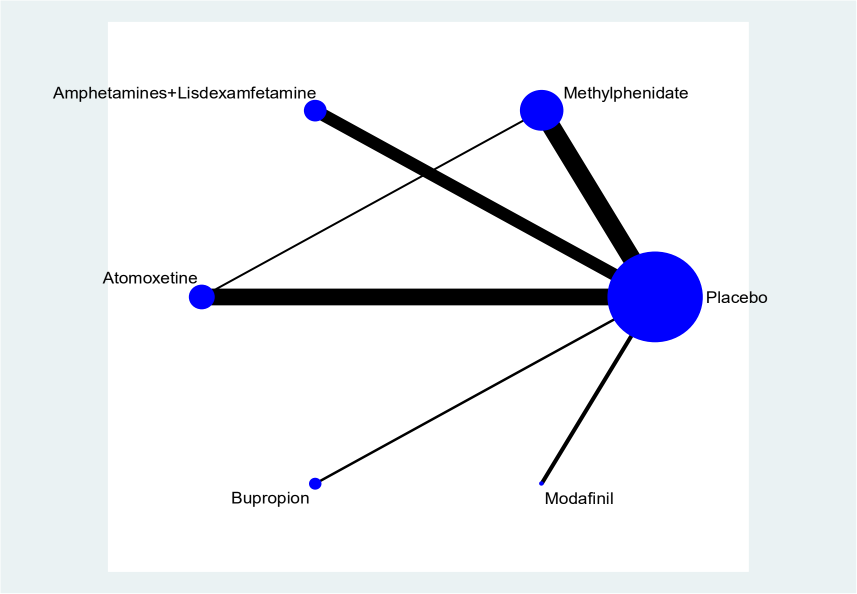

Figures S2. Comparison-adjusted funnel plots *(all drugs vs. placebo)*

Efficacy – ADHD core symptoms, Children/Adolescents, Teachers' Ratings

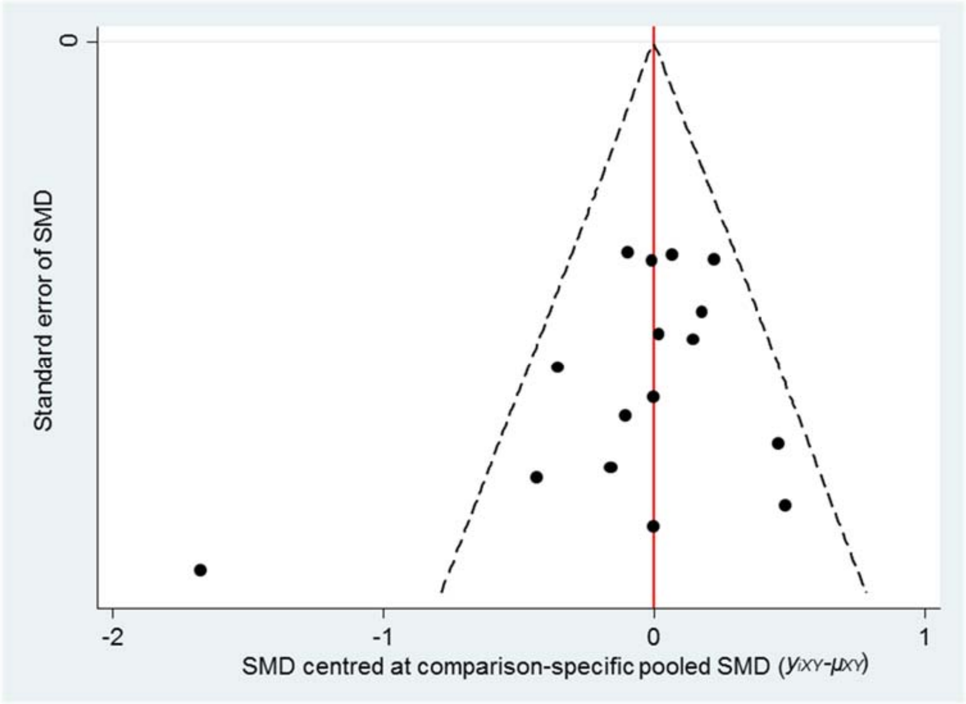

Efficacy – ADHD core symptoms, Adults, Clinicians' Ratings

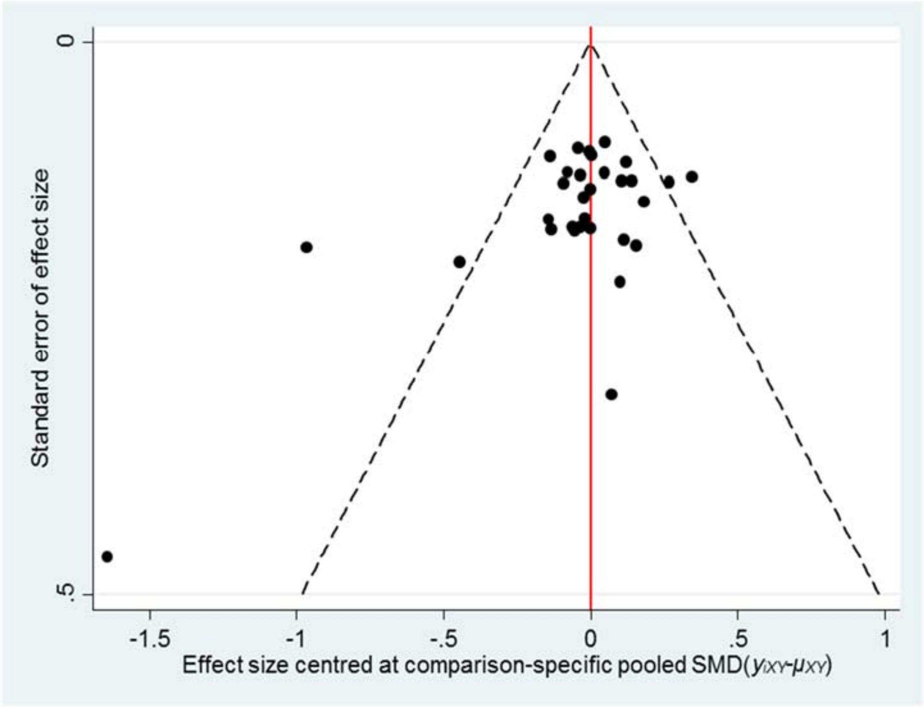

Efficacy – ADHD core symptoms, Children/Adolescents, Clinicians' Ratings

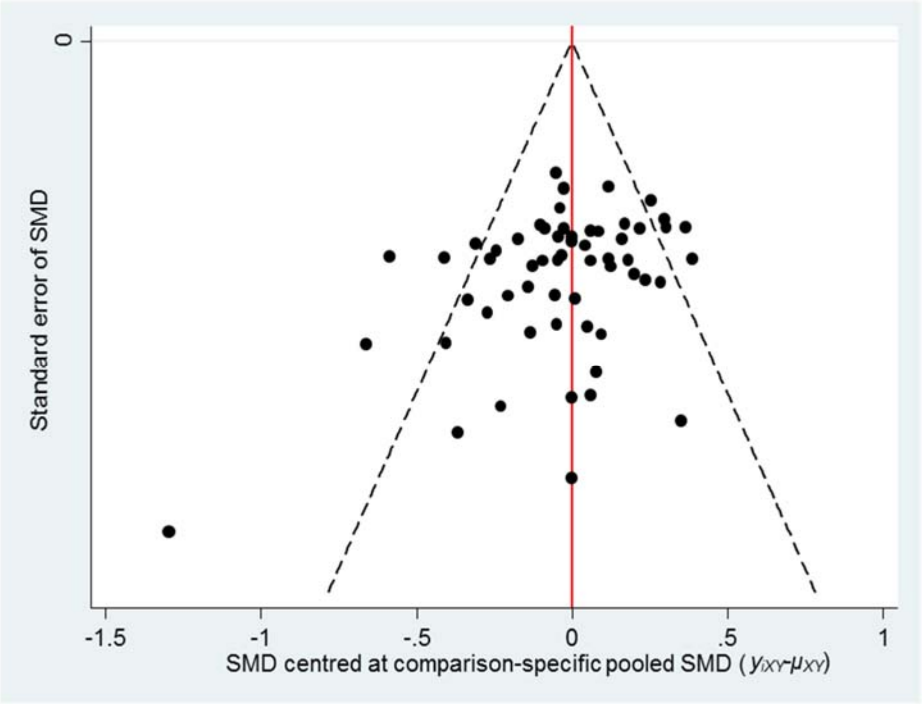

Tolerability –Children/Adolescents

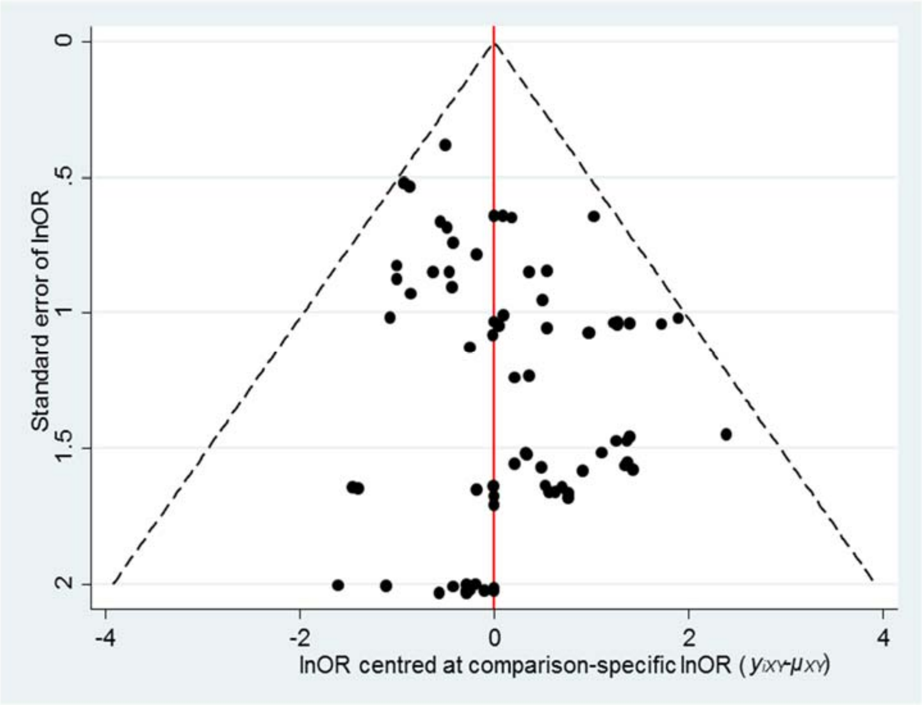

Tolerability –Adults

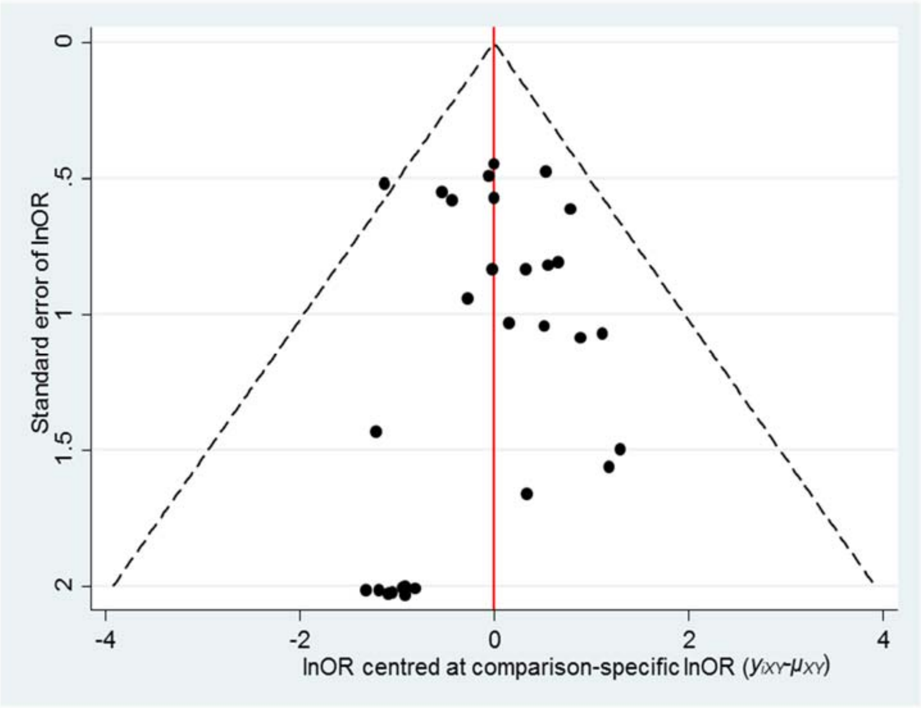

Figures S3. Bar plots showing the contribution of the risk of bias from each direct comparison to the network estimates

Efficacy – ADHD core symptoms, Children/Adolescents, Teachers' Ratings

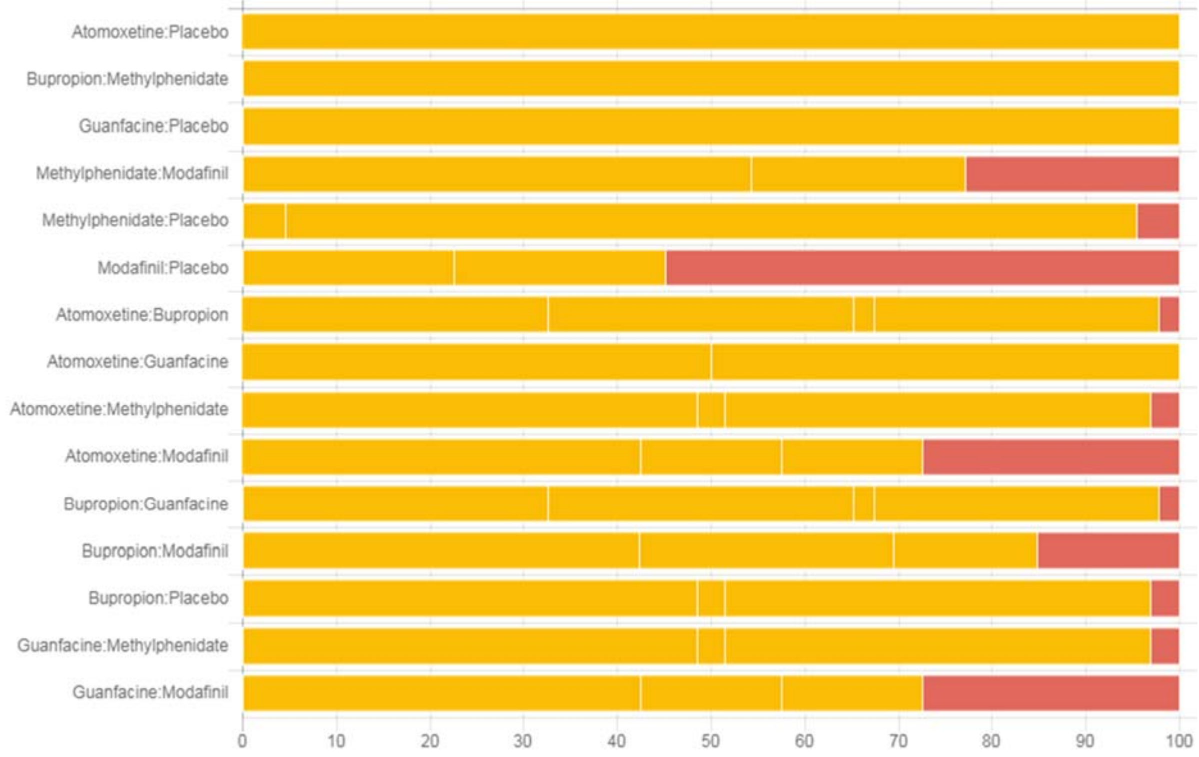

Efficacy – ADHD core symptoms, Adults, Clinicians' Ratings

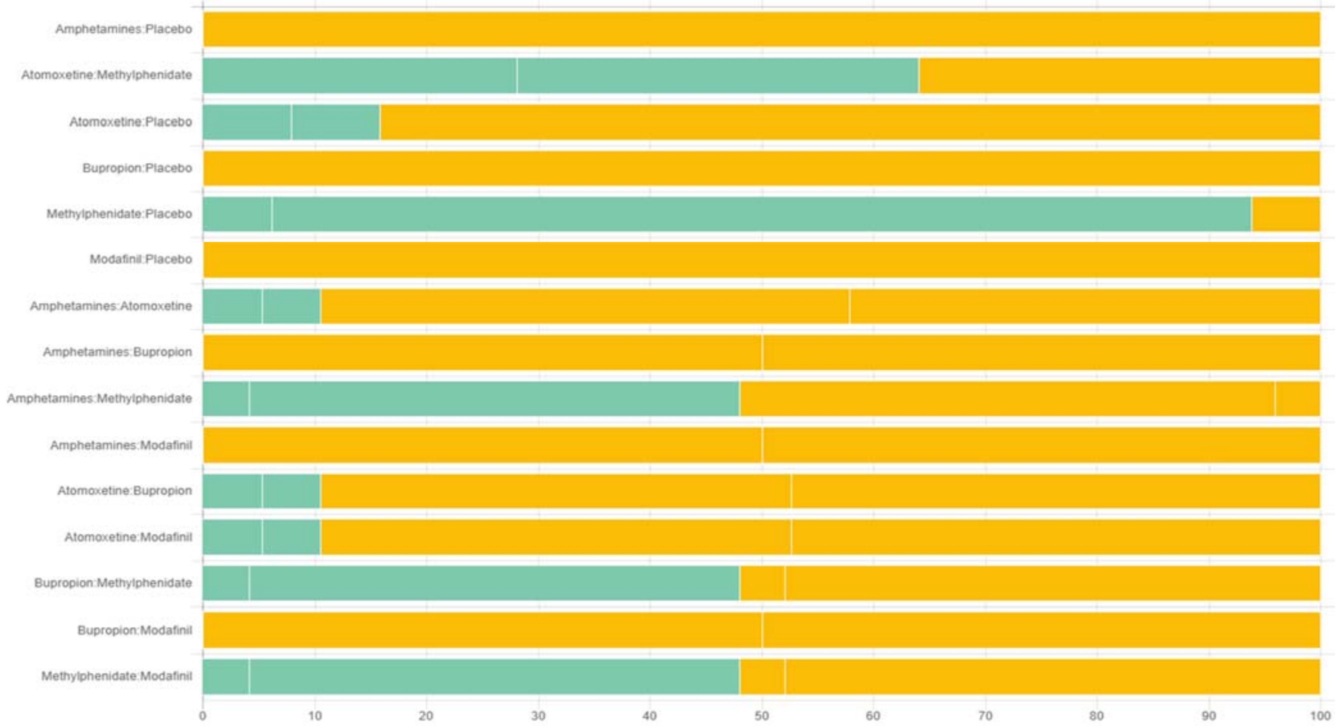

Efficacy – ADHD core symptoms, Children, Clinicians' Ratings

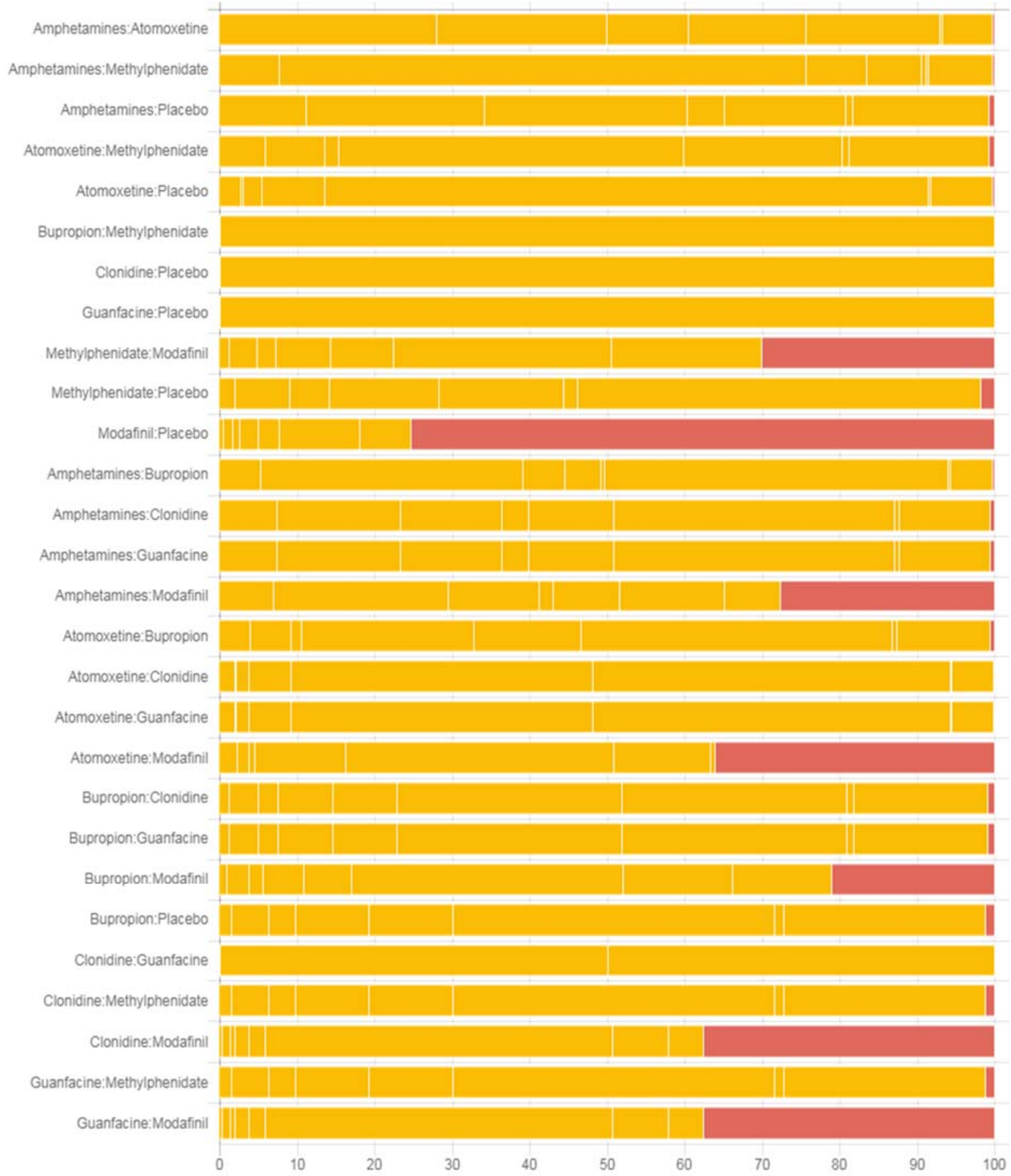

Tolerability, Children/Adolescents

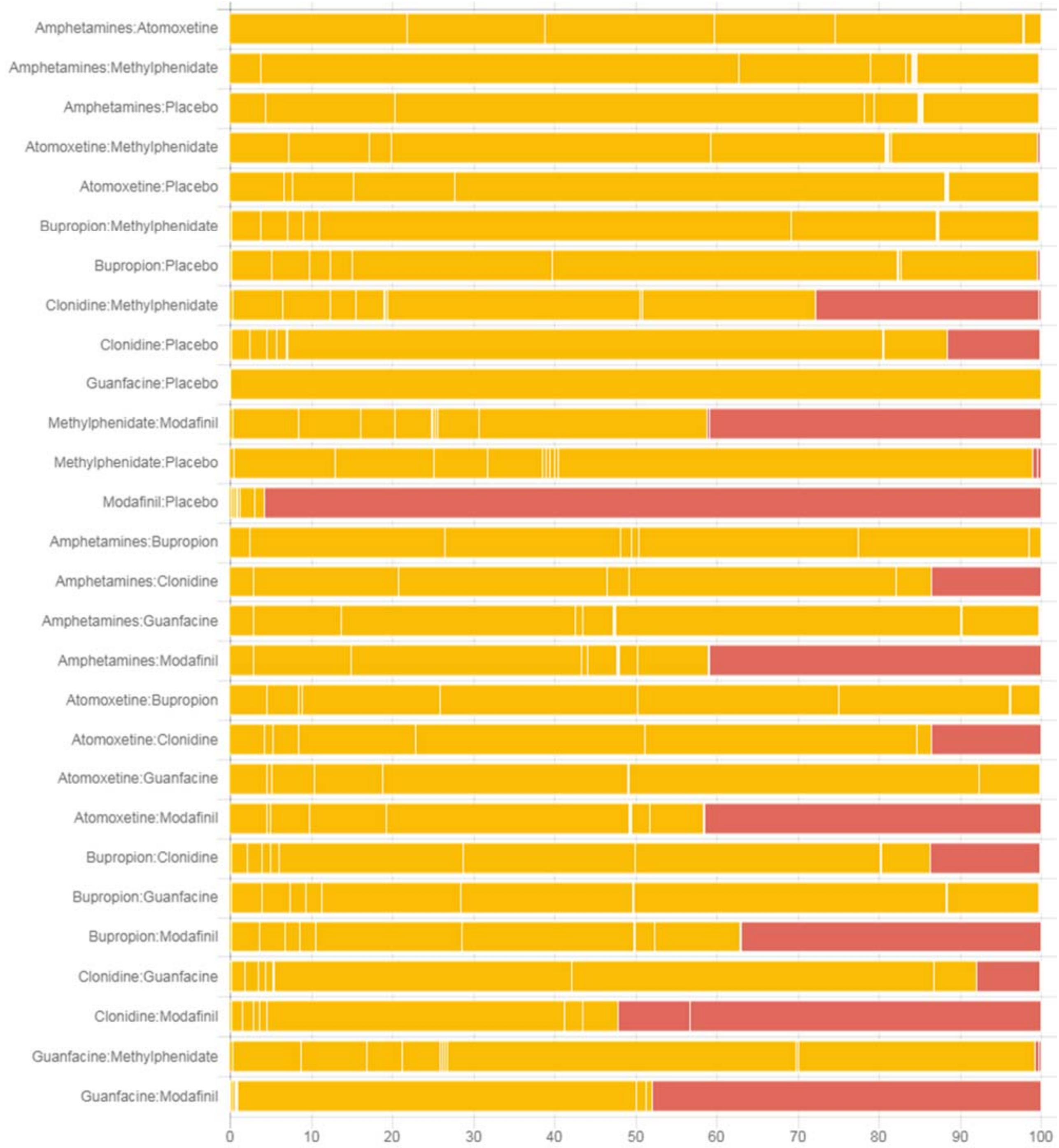

Tolerability, Adults

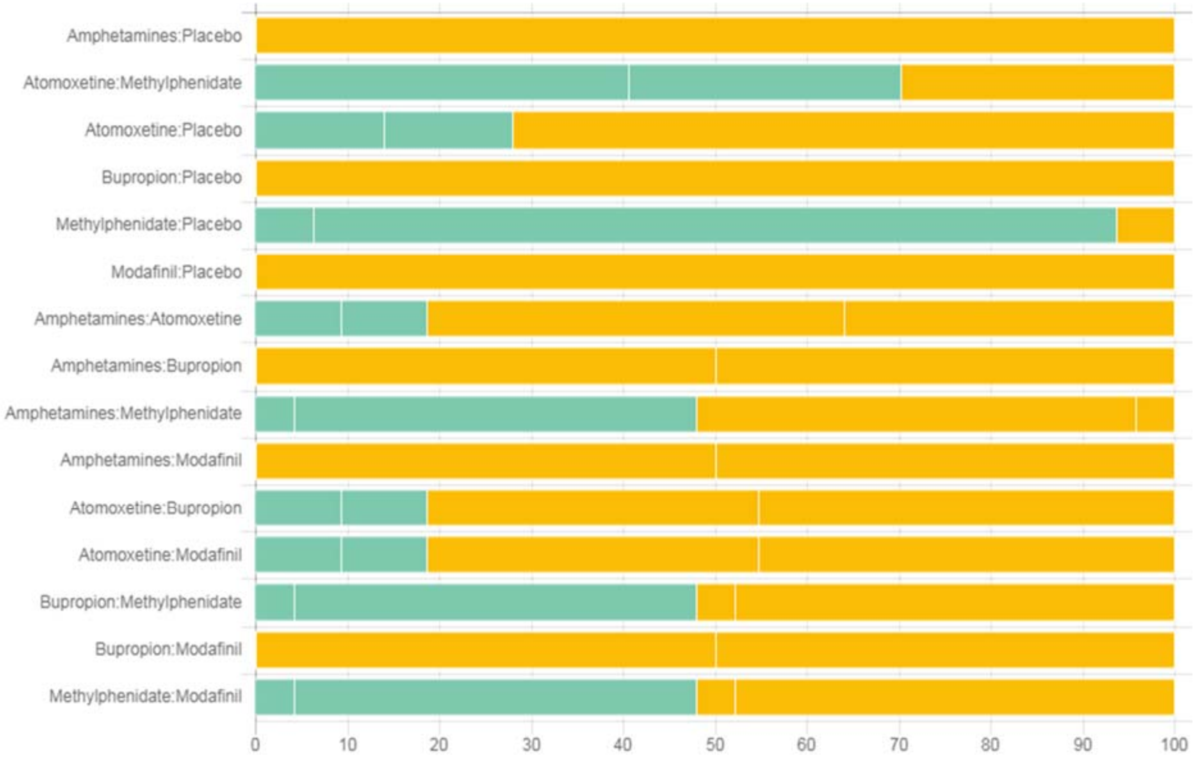

Figures S4. Plots presenting the confidence and predictive intervals for each network estimate

Efficacy – ADHD core symptoms, Children/Adolescents, Teachers' Ratings

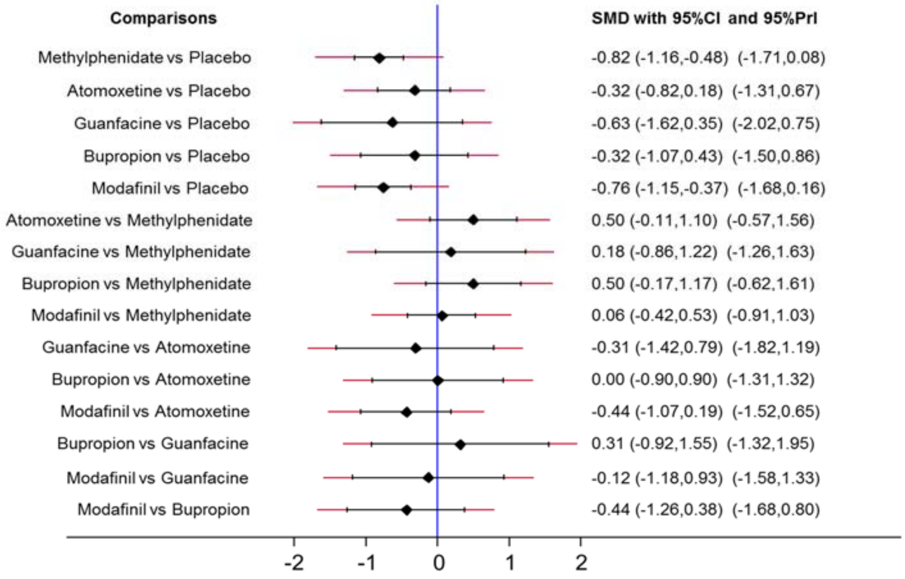

Efficacy – ADHD core symptoms, Adults, Clinicians' Ratings

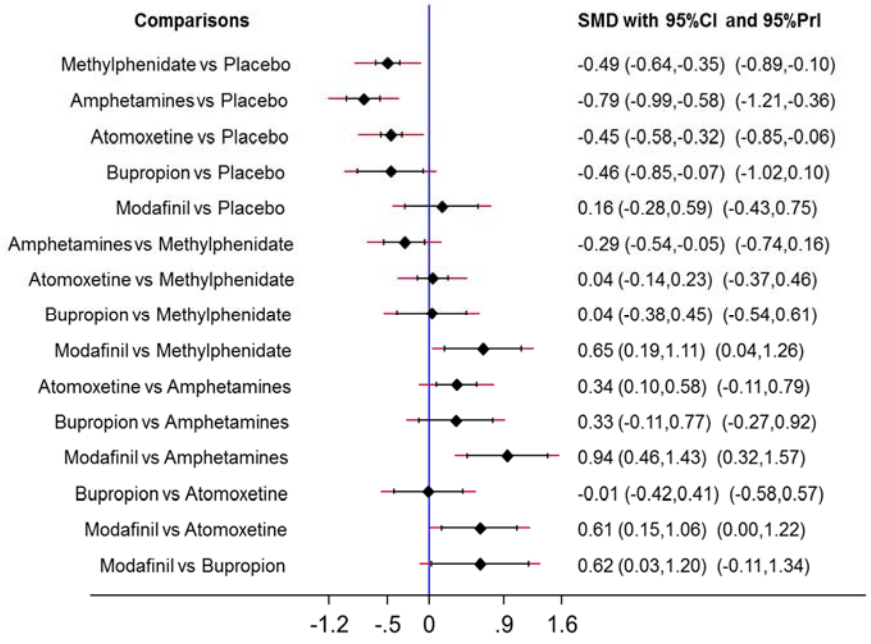

Efficacy – ADHD core symptoms, Children/Adolescents, Clinicians' Ratings

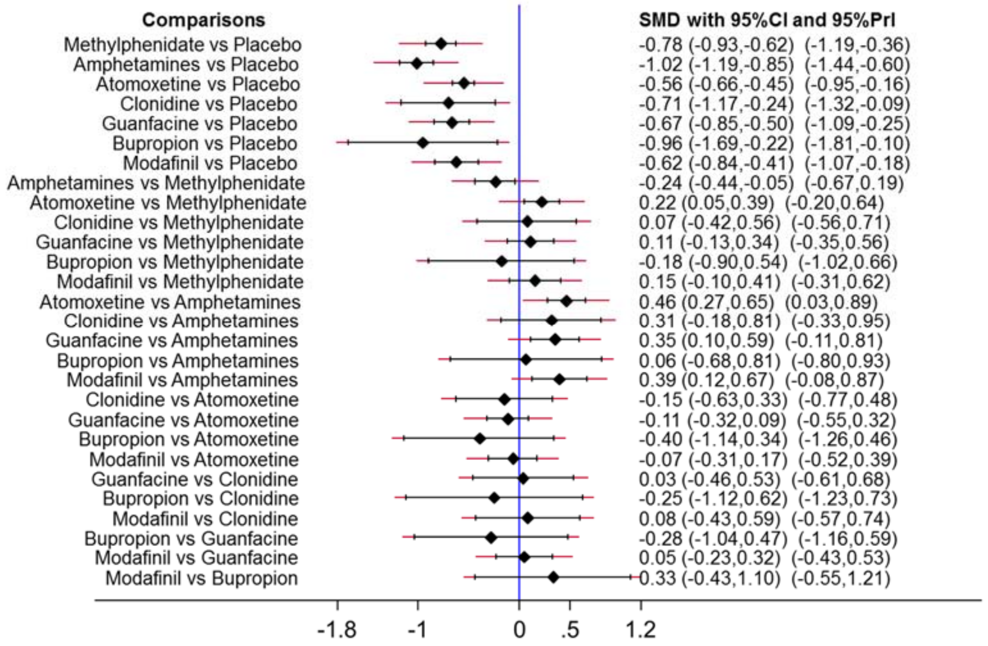

Tolerability –Children/Adolescents

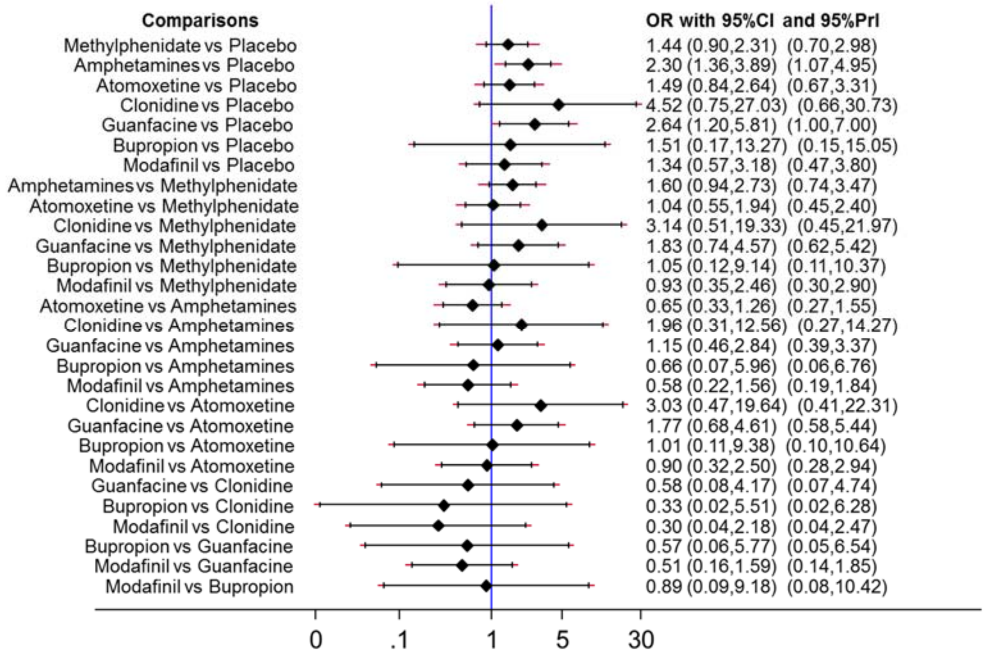

Tolerability –Adults

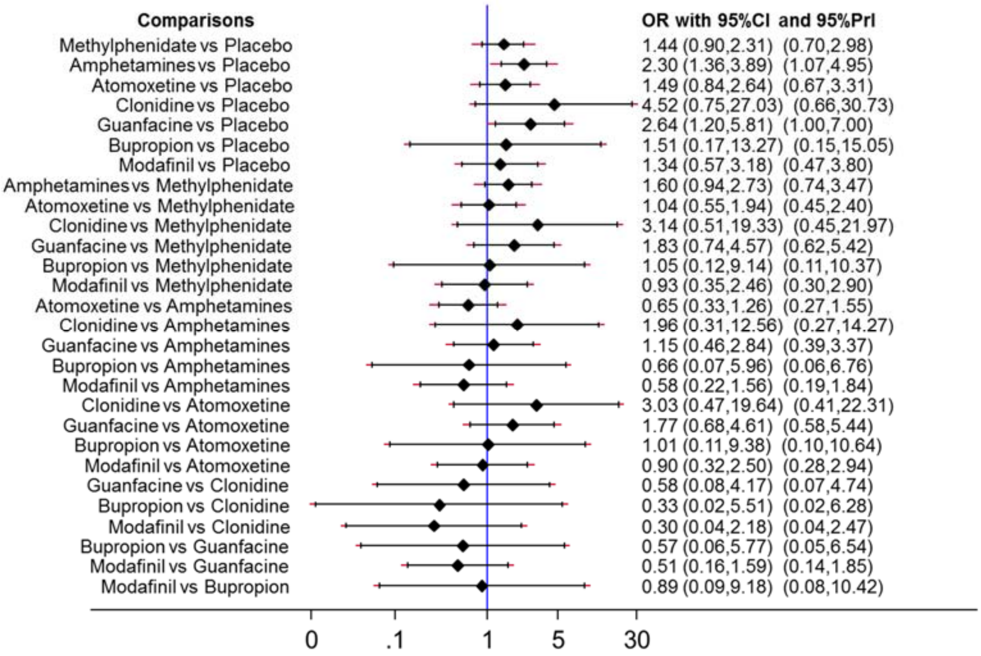

Figures S5. Bar plots showing the contribution of indirectness from each direct comparison to the network estimates

Efficacy – ADHD core symptoms, Children/Adolescents, Teachers' Ratings

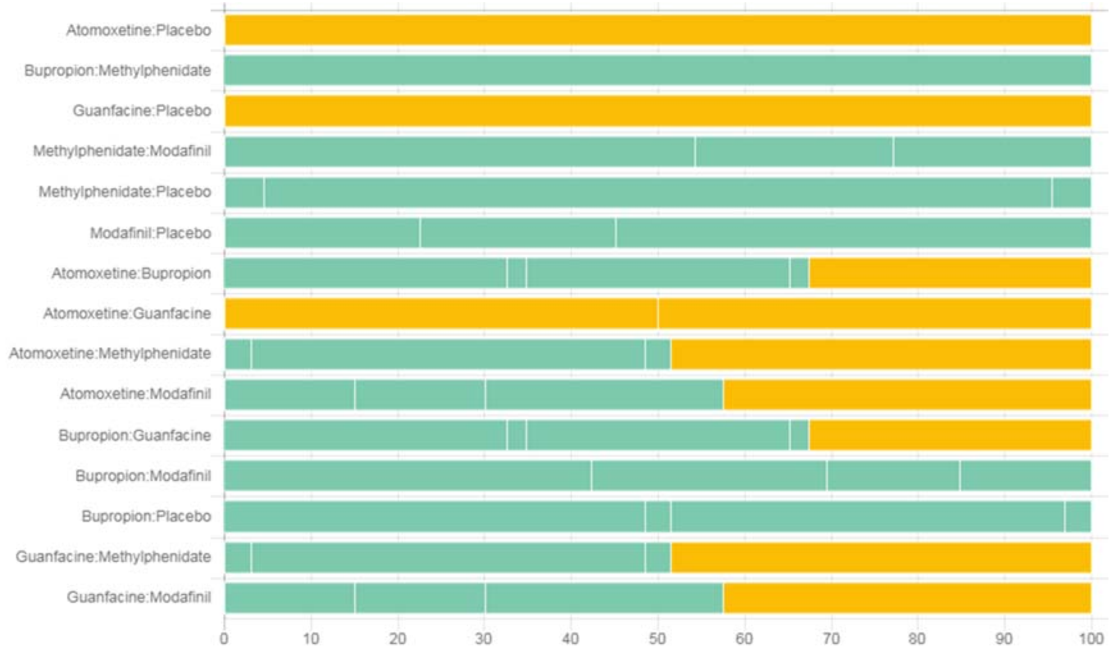

Efficacy – ADHD core symptoms, Adults, Clinicians' Ratings

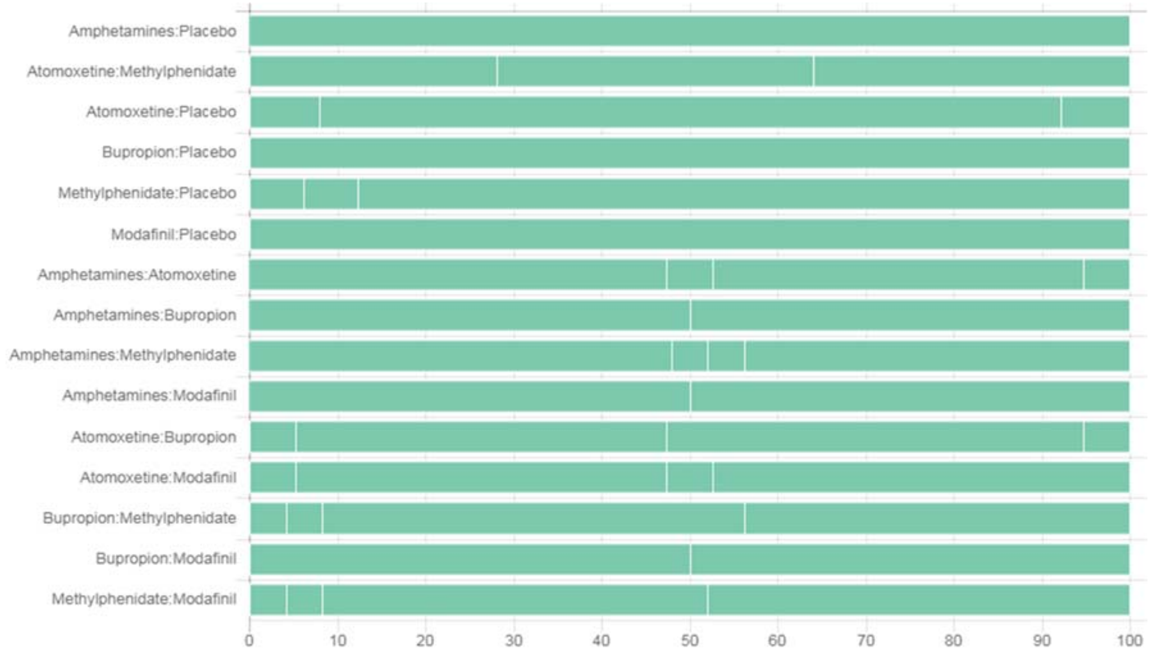

Efficacy – ADHD core symptoms, Children/Adolescents, Clinicians' Ratings

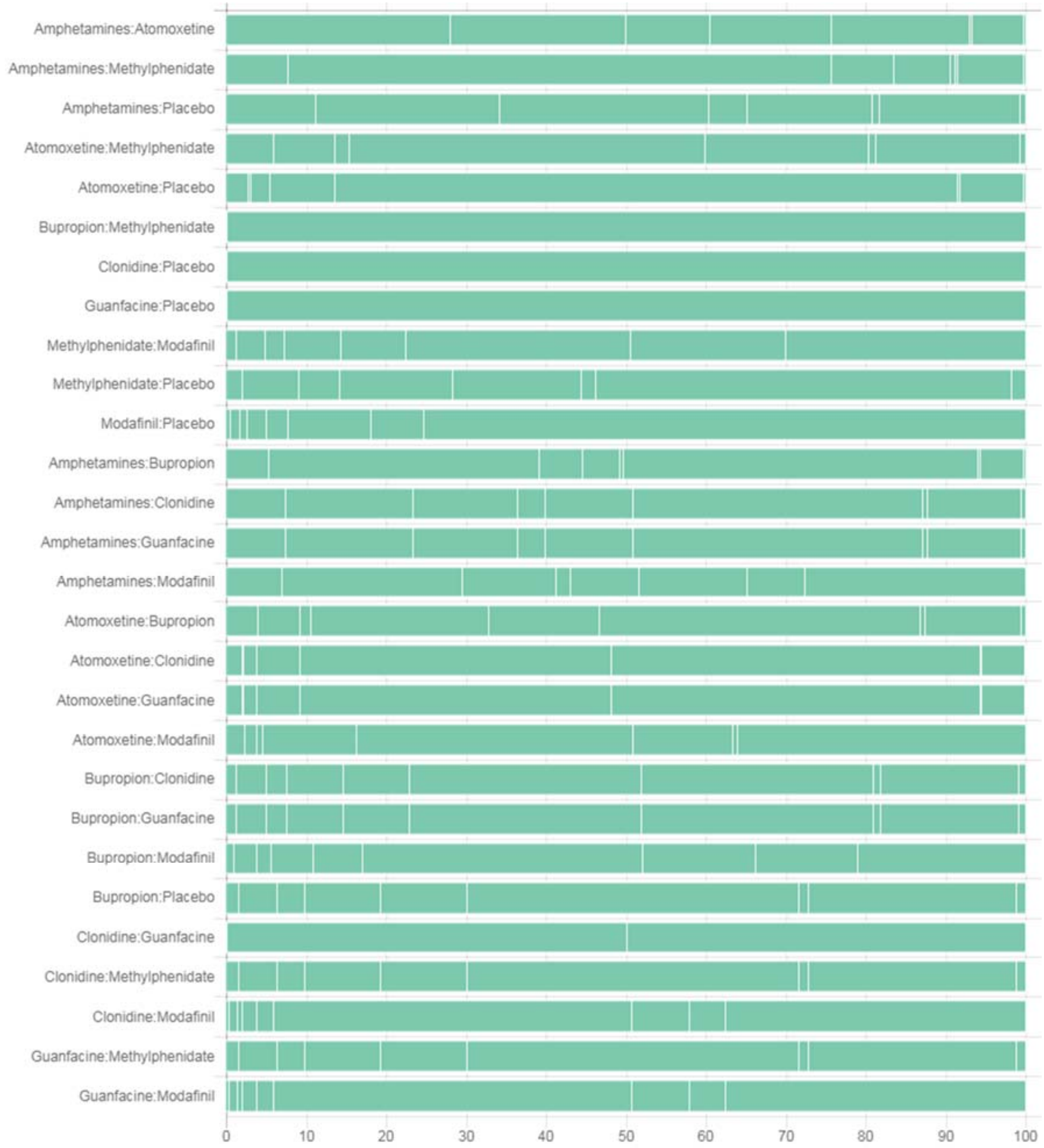

Tolerability –Children/Adolescents

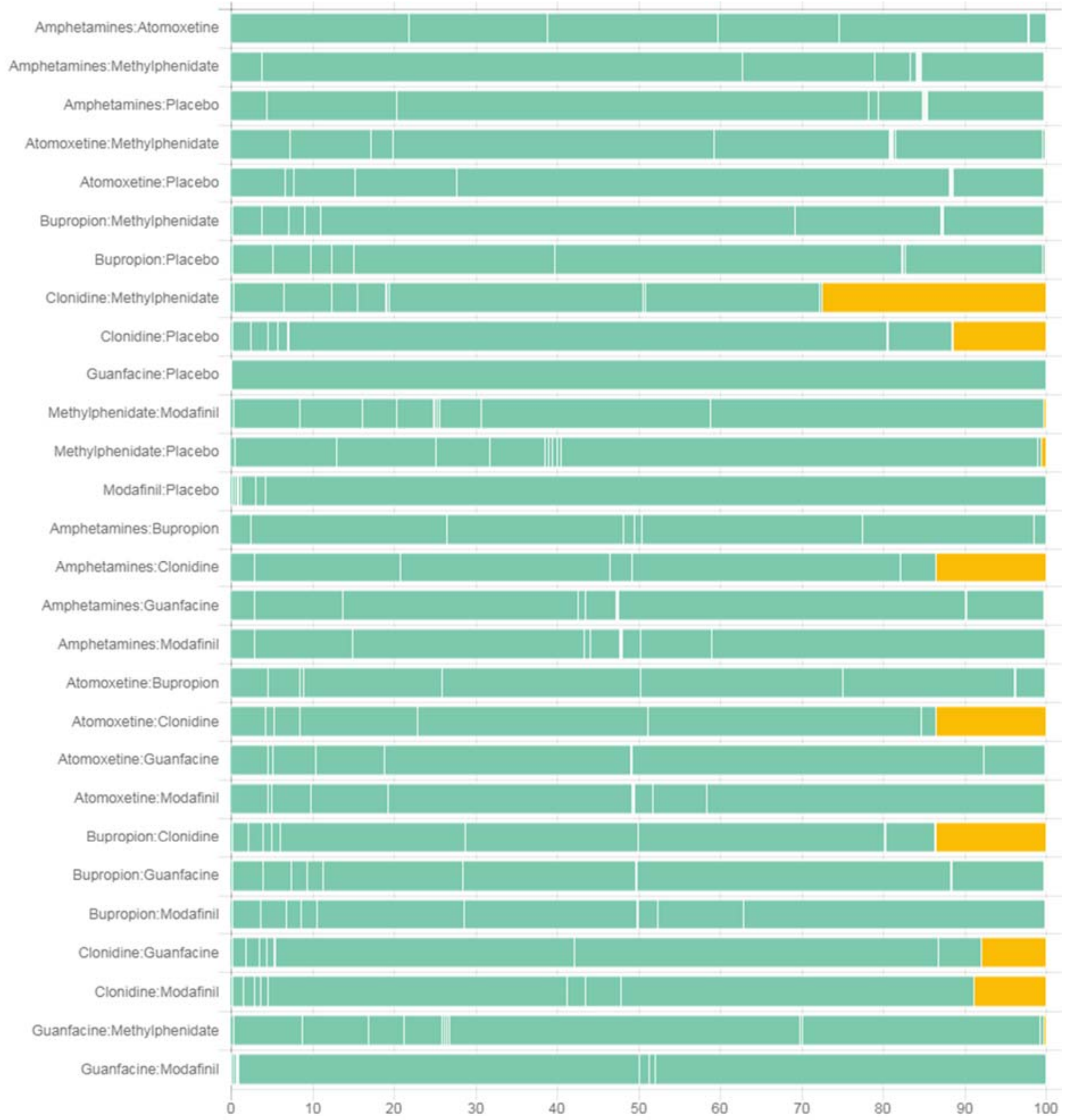

Tolerability –Adults

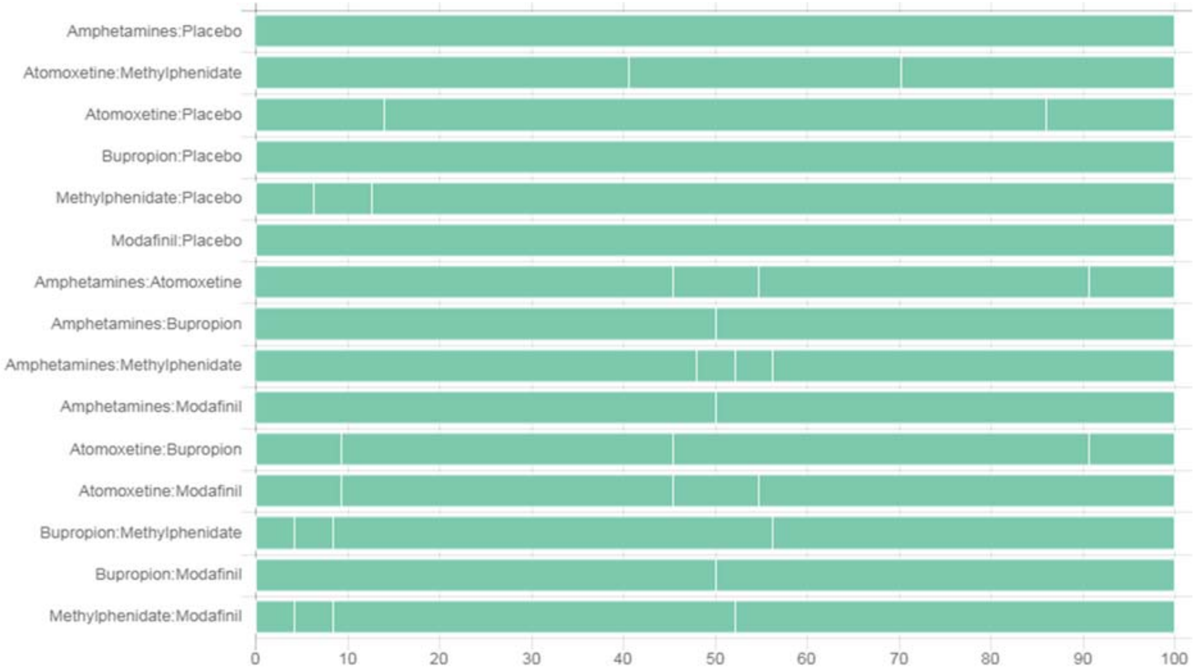

Supplemental references

1. Lefebvre C, Manheimer E, Glanville J. Chapter 6: Searching for studies. In: Higgins JPT, Green S, editors. Cochrane Handbook for Systematic Reviews of Interventions. Version 5.1.0 [updated March 2011] ed: The Cochrane Collaboration; 2011.

2. Higgins JPT, Lasserson T, Chandler J, Tovey D, Churchill R. Methodological Expectations of Cochrane Intervention Reviews. London: Cochrane; 2016.

3. Moher D, Liberati A, Tetzlaff J, Altman DG. Preferred reporting items for systematic reviews and meta-analyses: the PRISMA statement. PLoS Med 2009;6(7):e1000097.
4. McGowan J, Sampson M, Salzwedel DM, Cogo E, Foerster V, Lefebvre C. PRESS Peer Review of Electronic Search Strategies: 2015 Guideline Statement. J Clin Epidemiol 2016;75:40-46.

5. Chalmers I, Glasziou P. Avoidable waste in the production and reporting of research evidence. Lancet 2009;374(9683):86-89.

6. Agarwal A, Johnston BC, Vernooij RW et al. Authors seldom report the most patient-important outcomes and absolute effect measures in systematic review abstracts. J Clin Epidemiol 2017;81:3-12.

7. Chan AW, Hrobjartsson A, Haahr MT, Gotzsche PC, Altman DG. Empirical evidence for selective reporting of outcomes in randomized trials: comparison of protocols to published articles. JAMA 2004;291(20):2457-2465.

8. Glasziou P, Altman DG, Bossuyt P et al. Reducing waste from incomplete or unusable reports of biomedical research. Lancet 2014;383(9913):267-276.

9. Mantziari S, Demartines N. Poor outcome reporting in medical research; building practice on spoilt grounds. Ann Transl Med 2017;5(Suppl 1):S15.

10. Mavridis D, Giannatsi M, Cipriani A, Salanti G. A primer on network meta-analysis with emphasis on mental health. Evid Based Ment Health 2015;18(2):40-46.

11. Storebo OJ, Ramstad E, Krogh HB et al. Methylphenidate for children and adolescents with attention deficit hyperactivity disorder (ADHD). Cochrane Database Syst Rev 2015;11:CD009885.

12. Catala-Lopez F, Hutton B, Nunez-Beltran A et al. The pharmacological and non-pharmacological treatment of attention deficit hyperactivity disorder in children and adolescents: protocol for a systematic review and network meta-analysis of randomized controlled trials. Syst Rev 2015;4:19.

13. Curtin F, Elbourne D, Altman DG. Meta-analysis combining parallel and cross-over clinical trials. III: The issue of carry-over. Stat Med 2002;21(15):2161-2173.

14. Elbourne DR, Altman DG, Higgins JP, Curtin F, Worthington HV, Vail A. Meta-analyses involving cross-over trials: methodological issues. Int J Epidemiol 2002;31(1):140-149.

15. Higgins JPT, Green S (editors).*Cochrane Handbook for Systematic Reviews of Interventions*Version 5.1.0 [updated March 2011]. The Cochrane Collaboration, 2011. Available fromwww.cochrane-handbook.org.

16. Ukoumunne OC, Gulliford MC, Chinn S, Sterne JA, Burney PG. Methods for evaluating area-wide and organisation-based interventions in health and health care: a systematic review. Health Technol Assess 1999;3(5):iii-92.

17. da Costa BR, Nuesch E, Rutjes AW et al. Combining follow-up and change data is valid in meta-analyses of continuous outcomes: a meta-epidemiological study. J Clin Epidemiol 2013;66(8):847-855.

18. Cipriani A, Zhou X, Del GC et al. Comparative efficacy and tolerability of antidepressants for major depressive disorder in children and adolescents: a network meta-analysis. Lancet 2016;388(10047):881-890.

19. Cipriani A, Furukawa TA, Salanti G, Chaimani A, Atkinson LZ, Ogawa Y, Leucht S, Ruhe HG, Turner EH, Higgins JPT, Egger M, Takeshima N, Hayasaka Y, Imai H, Shinohara K, TajikaA, Ioannidis JPA, Geddes JR. Comparative efficacy and acceptability of 21 antidepressant drugs for the acute treatment of adults with major depressive disorder: a systematic review and network meta-analysis. Lancet 2018;391(10128):1357-1366.

20. Chaimani A, Higgins JP, Mavridis D, Spyridonos P, Salanti G. Graphical tools for network meta-analysis in STATA. PLoS One 2013;8(10):e76654.

21. White IR. Multivariate random-effects meta-regression: updates to mvmeta. Stata Journal 2011;11:255-70.

22. White IR, Barrett JK, Jackson D, Higgins JP. Consistency and inconsistency in network meta-analysis: model estimation using multivariate meta-regression. Res Synth Methods 2012;3(2):111-125.

23. Altman DG, Bland JM. Detecting skewness from summary information. BMJ 1996;313(7066):1200.

24. Furukawa TA, Barbui C, Cipriani A, Brambilla P, Watanabe N. Imputing missing standard deviations in meta-analyses can provide accurate results. J Clin Epidemiol 2006;59(1):7-10.

25. Salanti G. Indirect and mixed-treatment comparison, network, or multiple-treatments meta-analysis: many names, many benefits, many concerns for the next generation evidence synthesis tool. Res Synth Methods 2012;3(2):80-97.

26. Jansen JP, Naci H. Is network meta-analysis as valid as standard pairwise meta-analysis? It all depends on the distribution of effect modifiers. BMC Med 2013;11:159.

27. Higgins JP, Thompson SG, Deeks JJ, Altman DG. Measuring inconsistency in meta-analyses. BMJ 2003;327(7414):557-560.

28. Jackson D, Barrett JK, Rice S, White IR, Higgins JP. A design-by-treatment interaction model for network meta-analysis with random inconsistency effects. Stat Med 2014;33(21):3639-3654.

29. Turner RM, Davey J, Clarke MJ, Thompson SG, Higgins JP. Predicting the extent of heterogeneity in meta-analysis, using empirical data from the Cochrane Database of Systematic Reviews. Int J Epidemiol 2012;41(3):818-827.

30. Rhodes KM, Turner RM, Higgins JP. Predictive distributions were developed for the extent of heterogeneity in meta-analyses of continuous outcome data. J Clin Epidemiol 2015;68(1):52-60.

31. Veroniki AA, Vasiliadis HS, Higgins JP, Salanti G. Evaluation of inconsistency in networks of interventions. Int J Epidemiol 2013;42(1):332-345.

32. Dias S, Welton NJ, Caldwell DM, Ades AE. Checking consistency in mixed treatment comparison meta-analysis. Stat Med 2010;29(7-8):932-944.

33. Salanti G, Marinho V, Higgins JP. A case study of multiple-treatments meta-analysis demonstrates that covariates should be considered. J Clin Epidemiol 2009;62(8):857-864.

34. Higgins JP, Jackson D, Barrett JK, Lu G, Ades AE, White IR. Consistency and inconsistency in network meta-analysis: concepts and models for multi-arm studies. Res Synth Methods 2012;3(2):98-110.

35. Mayes TL, Tao R, Rintelmann JW et al. Do children and adolescents have differential response rates in placebo-controlled trials of fluoxetine? CNS Spectr 2007;12(2):147-154.

36. Cipriani A, Furukawa TA, Salanti G et al. Comparative efficacy and acceptability of 12 new-generation antidepressants: a multiple-treatments meta-analysis. Lancet 2009;373(9665):746-758.

37. CINeMA:*Confidence in Network Meta-Analysis*[Software]. Institute of Social and Preventive Medicine, University of Bern, 2017. Available from: cinema.ispm.ch.

38. Chaimani A, Salanti G. Using network meta-analysis to evaluate the existence of small-study effects in a network of interventions. Res Synth Methods 2012;3(2):161-176.

39. Cortese S, Adamo N, Mohr-Jensen C et al. Comparative efficacy and tolerability of pharmacological interventions for attention-deficit/hyperactivity disorder in children, adolescents and adults: protocol for a systematic review and network meta-analysis. BMJ Open 2017;7(1):e013967.

40. Martel MM, Schimmack U, Nikolas M, Nigg JT. Integration of symptom ratings from multiple informants in ADHD diagnosis: a psychometric model with clinical utility. Psychol Assess 2015;27(3):1060-1071.

41. Silva RR, Muniz R, Pestreich L et al. Dexmethylphenidate extended-release capsules in children with attention-deficit/hyperactivity disorder. J Am Acad Child Adolesc Psychiatry 2008;47(2):199-208.

42. Barkley RA, DuPaul GJ, McMurray MB. Attention deficit disorder with and without hyperactivity: clinical response to three dose levels of methylphenidate. Pediatrics 1991;87(4):519-531.

43. Blum NJ, Jawad AF, Clarke AT, Power TJ. Effect of osmotic-release oral system methylphenidate on different domains of attention and executive functioning in children with attention-deficit-hyperactivity disorder. Dev Med Child Neurol 2011;53(9):843-849.

44. King S, Griffin S, Hodges Z, et al. A systematic review and economic model of the effectiveness and cost-effectiveness of methylphenidate, dexamfetamine and atomoxetine for the treatment of attention deficit hyperactivity disorder in children and adolescents. Health technology assessment (Winchester, England); 2006;10(23):iii-iv, xiii-146.

45. Pliszka S. Practice parameter for the assessment and treatment of children and adolescents with attention-deficit/hyperactivity disorder. J Am Acad Child Adolesc Psychiatry 2007;46(7):894-921.

46. https://www.nps.org.au/. Last access February 10th, 2018

47. https://www.bnf.org/products/bnf-online/. Last access February 10th, 2018

48. https://www.caddra.ca/practice-guidelines/. Last access February 10th, 2018

Additional acknowledgments

We thank these study authors for replying to our requests, providing additional information, or unpublished data:
Joseph Biederman (Harvard University); Guido Biele (Norwegian Institute of Public Health); Hendrik J Butter (University of Ottawa); Ann Childress (Las Vegas Psychiatric and Behavioral Health Services); Stephen Collins (McMaster University); Angela Dean (The University of Queensland); Blanca Domingo Arnaiz (Landstingent Vastmanland); Ben Handen (University of Pittsburgh School of Medicine); Rakesh Jain (Texas Tech University School of Medicine); Rachel Klein (New York University); Rafael Klorman (University of Rochester); Roger Kurlan (The Center for Neurological and Neurodevelopmental Health); Dubi Lufi (Max Stern Yezreel Valley College); James T McCracken; Eric Mick (UMass Medical School) (UCLA); Jaap Osterlaan (University of Amsterdam); Donna Palumbo (formerly at the University of Rochester, where the study included in the meta-analysis was undertaken); Paul Perry (University of Iowa); William Pelham (Florida International University); Alexandra Philipsen (University of Freiburg); Liesbeth Reneman (University of Amsterdam); Sophia Shonka (University of Washington); Raul Silva (Child Mind Institute, NY); Eva Snircova (Comenius University in Bratislava); Mary Solanto (New York University); Atefeh Soltanifar (Mashhad University of Medical Sciences); James Swanson (UC Irvine); Margot Taylor (Hospital for Sick Children); Jaap van der Meere (University of Groningen); Timothy Wilens (Harvard University); Theresa Winhusen (University of Cincinnati College of Medicine); David Wodrich (University of Arizona); Mark Wolraich (Oklahoma University Child Study Center).

We thank employees of drug manufacturers for additional information: Chris Bushe and Andrea Gadd (Lilly).

For help formatting the supplemental materials, we thank: Megane Atkinson, Ellen Hawkes, Rosie Heape; Jade Mendelez, and Jess Taylor (University of Southampton, Southampton, UK).
